# Supplementary figures and images for: Comparative analysis of MAPK and MKK gene families reveals differential evolutionary patterns in Brachypodium distachyon inbred lines
Source: PeerJ. 2021 Apr 6;9:e11238. doi: 10.7717/peerj.11238 (PMC8034371; doi:10.7717/peerj.11238)

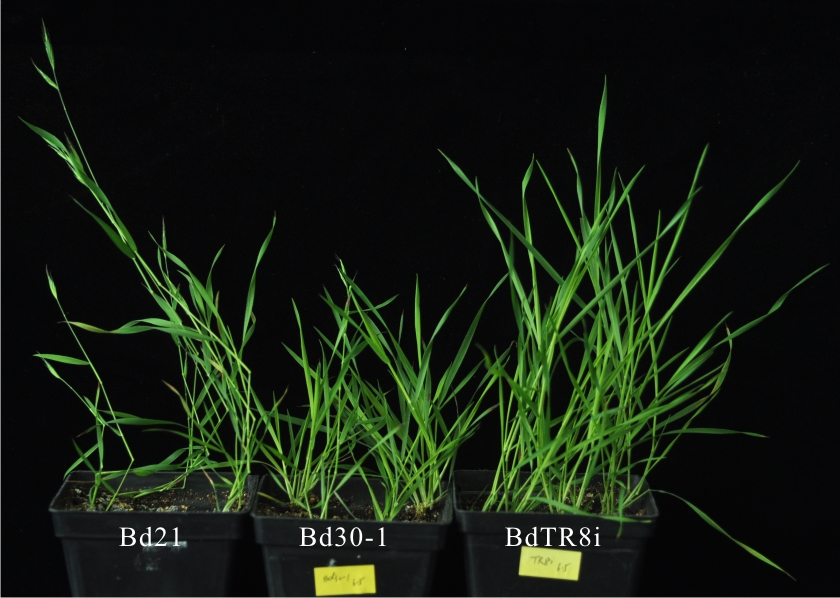

Supplement: Supplemental Information 1 [file peerj-09-11238-s001.jpg]

MPK3：


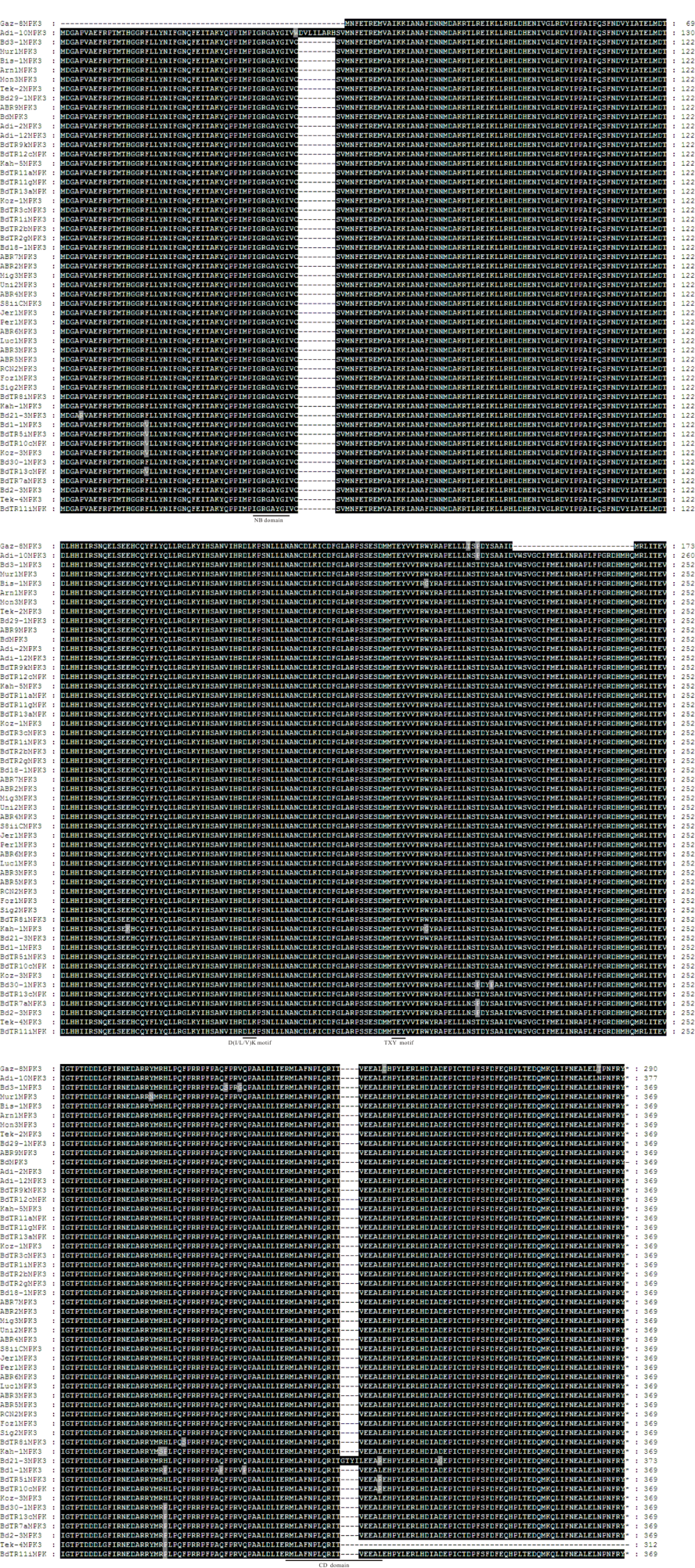


MPK4：


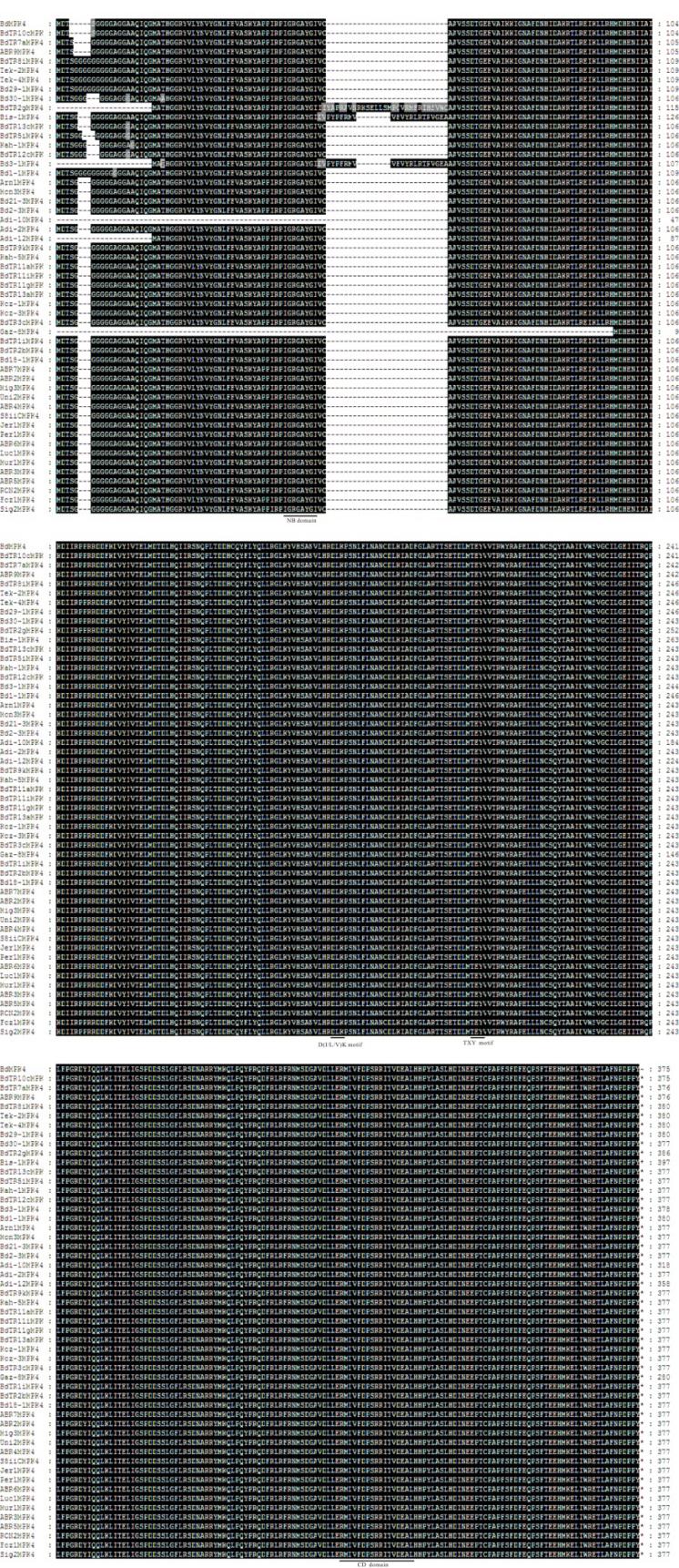


MPK6：


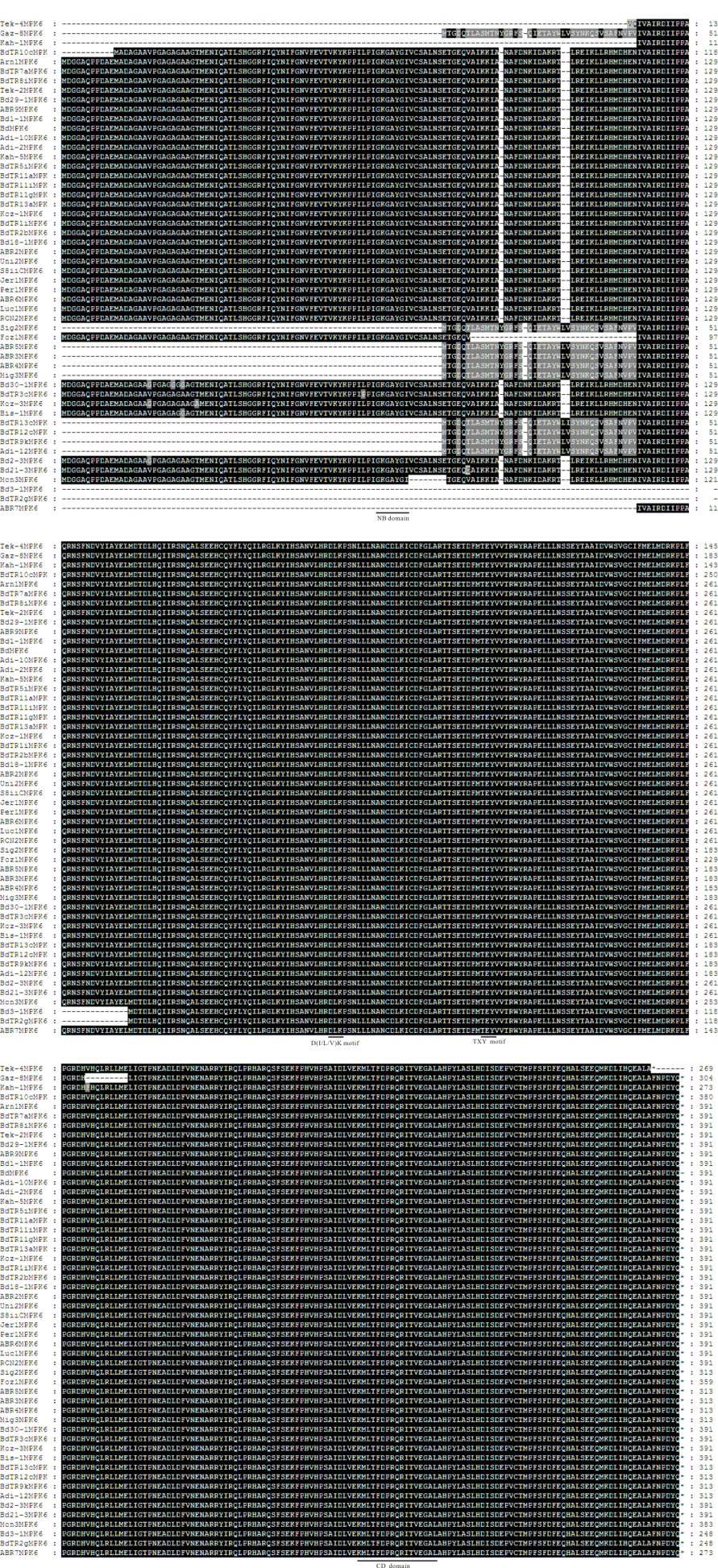


MPK7-1：


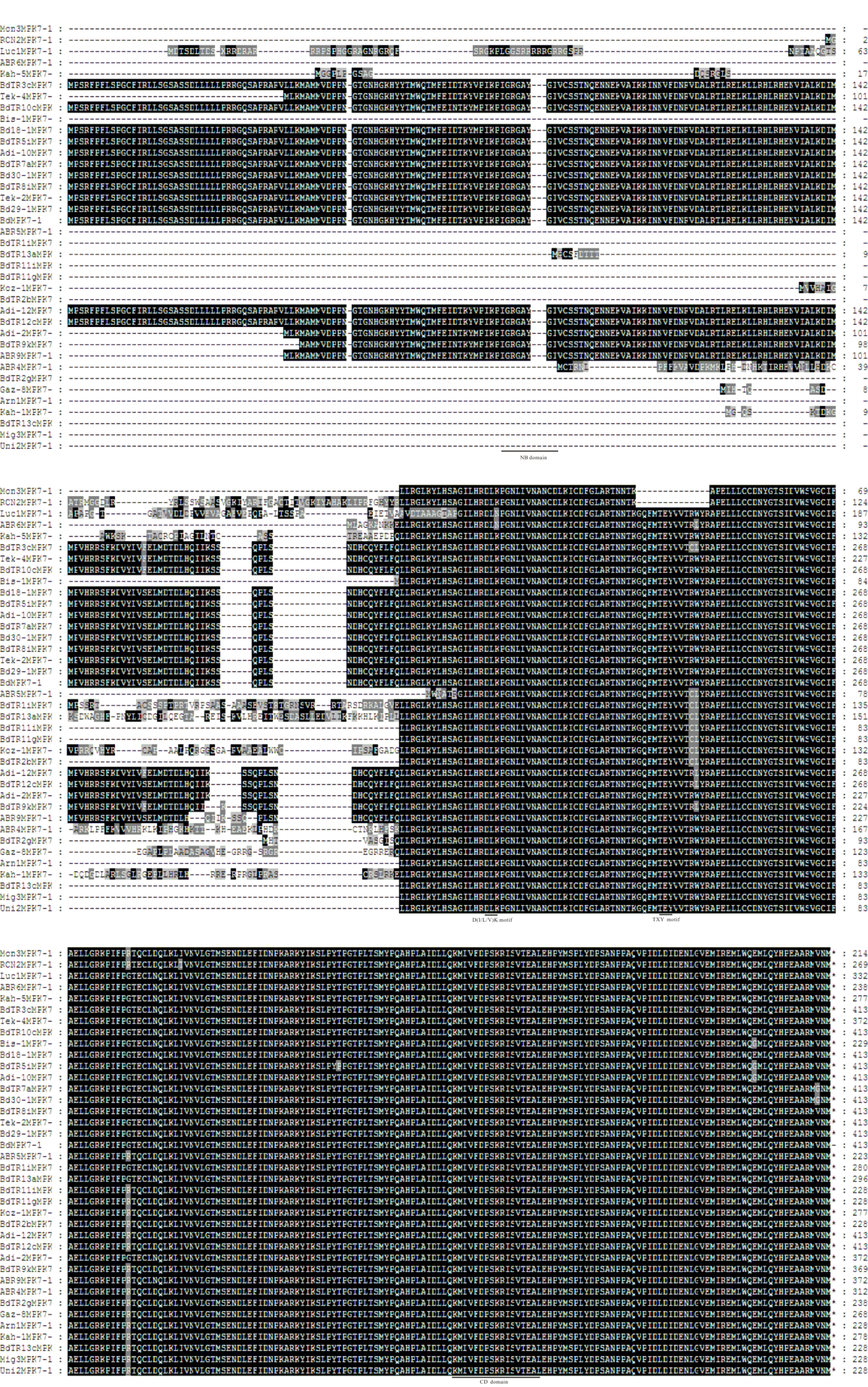


MPK7-2：


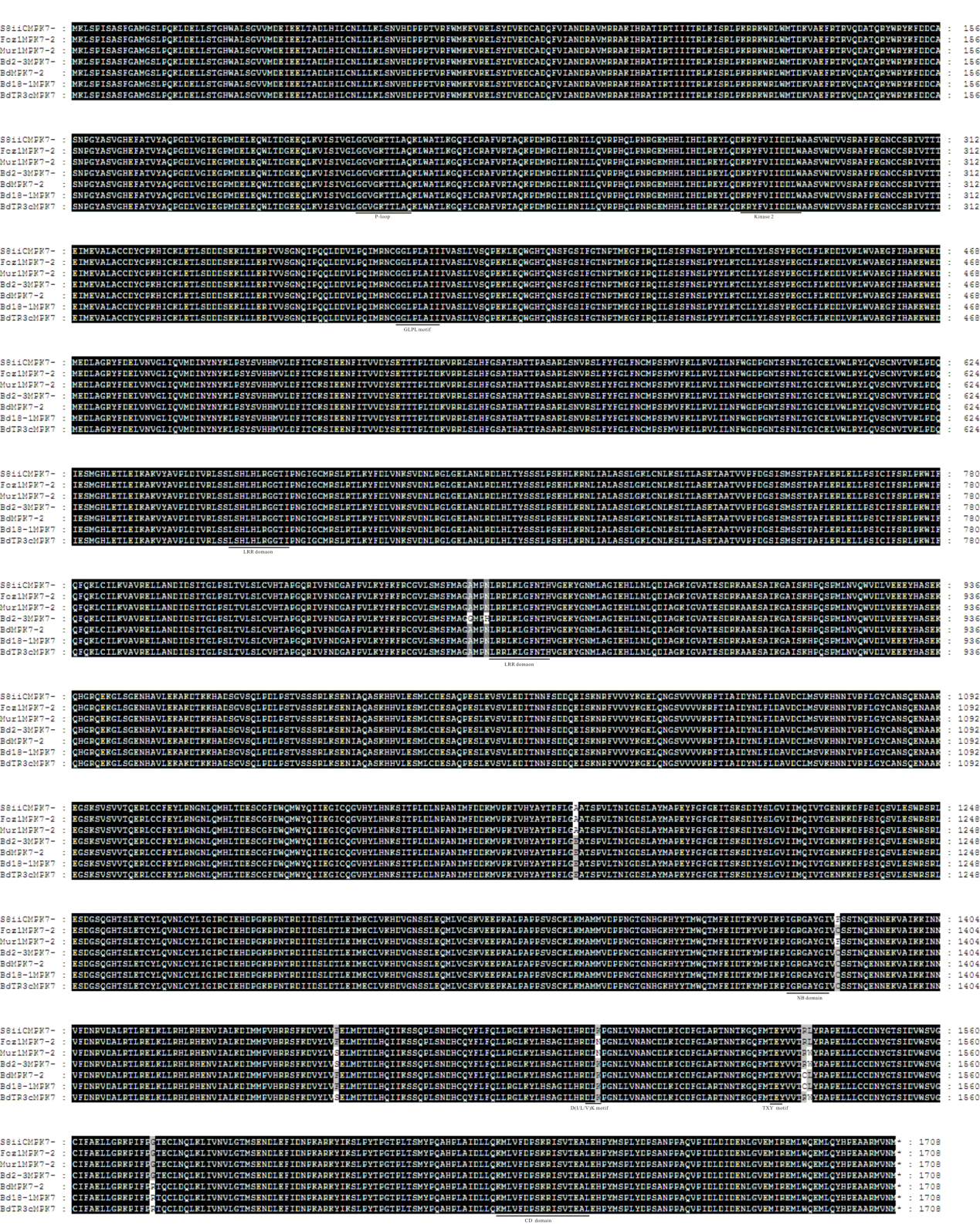


MPK11：


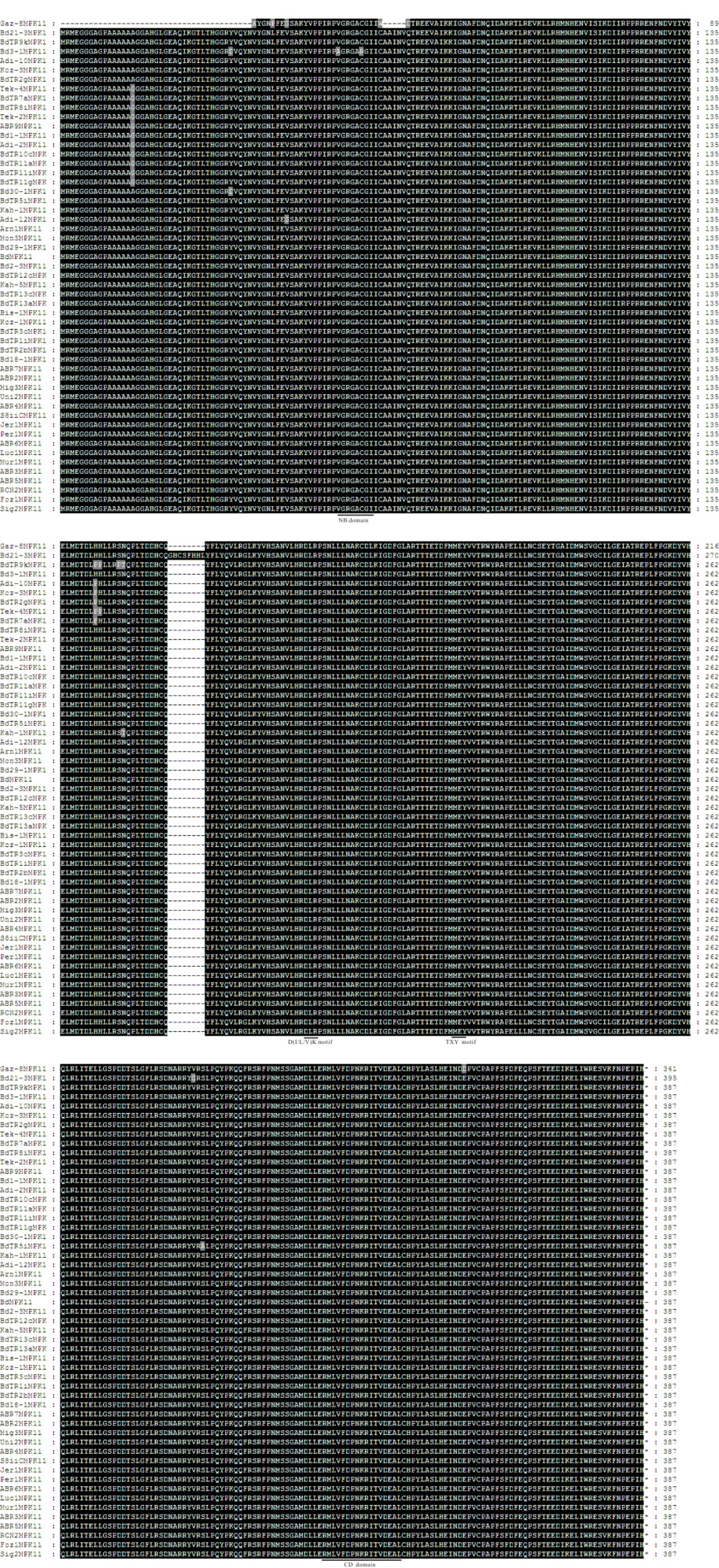


MPK14：


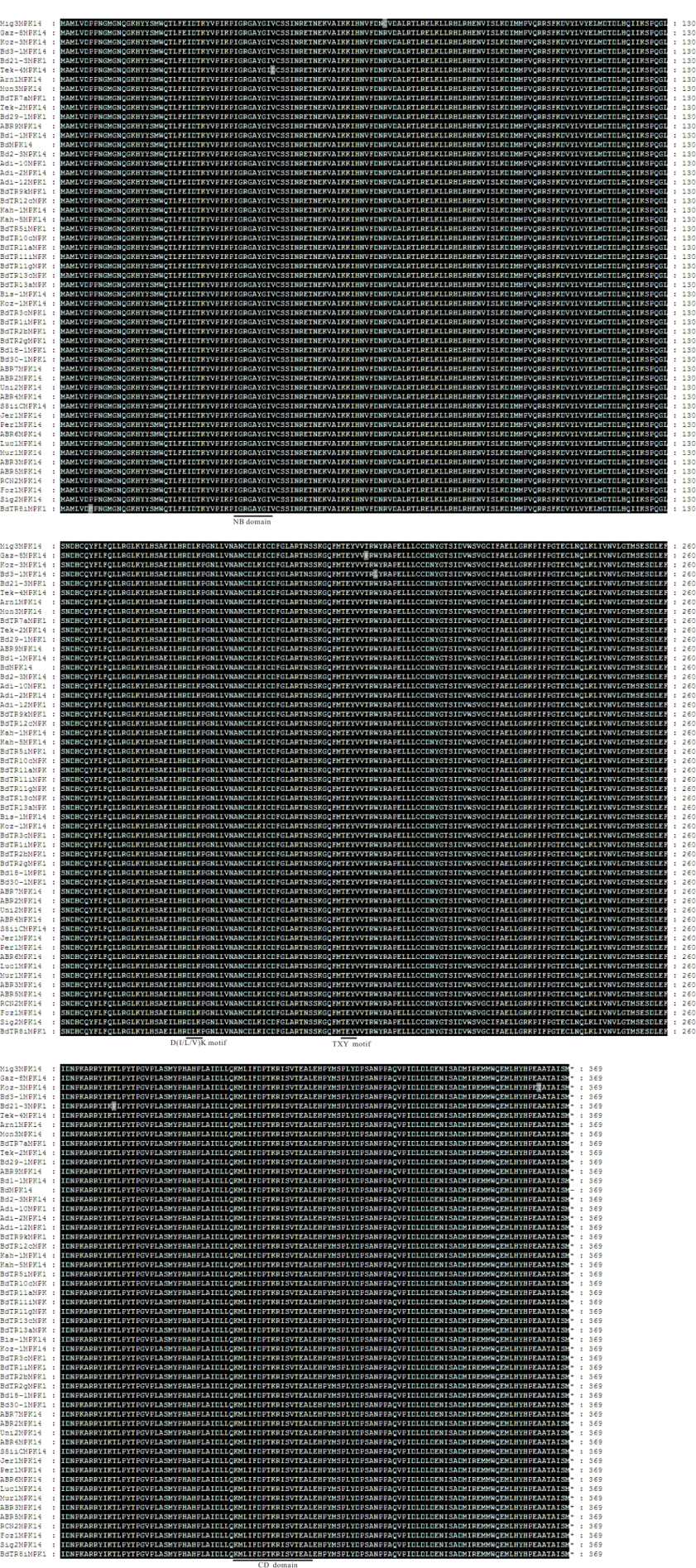


MPK16：


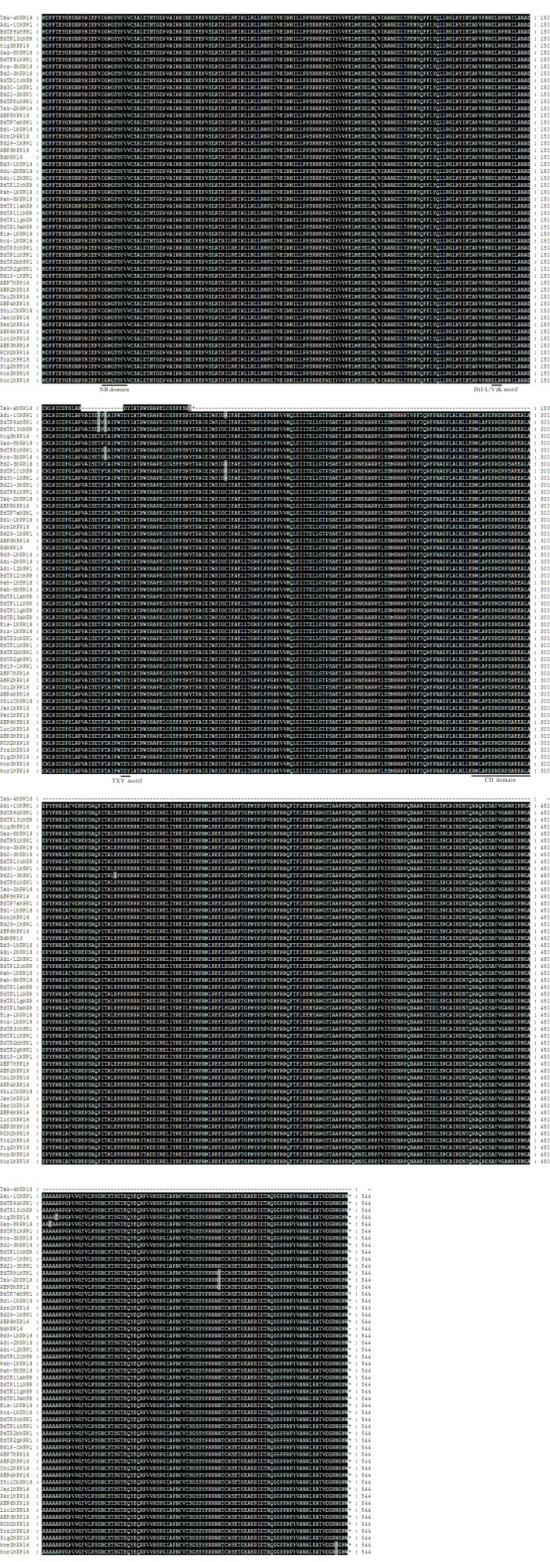


MPK17：


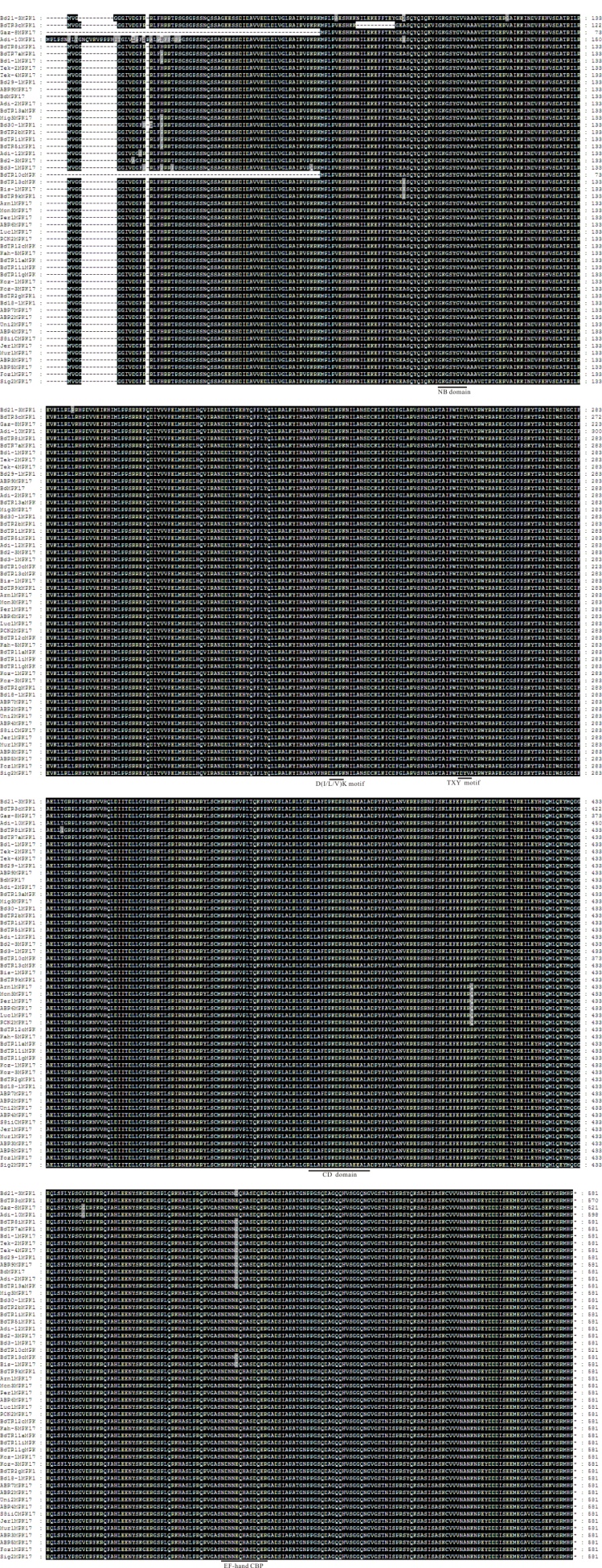


MPK20-1：


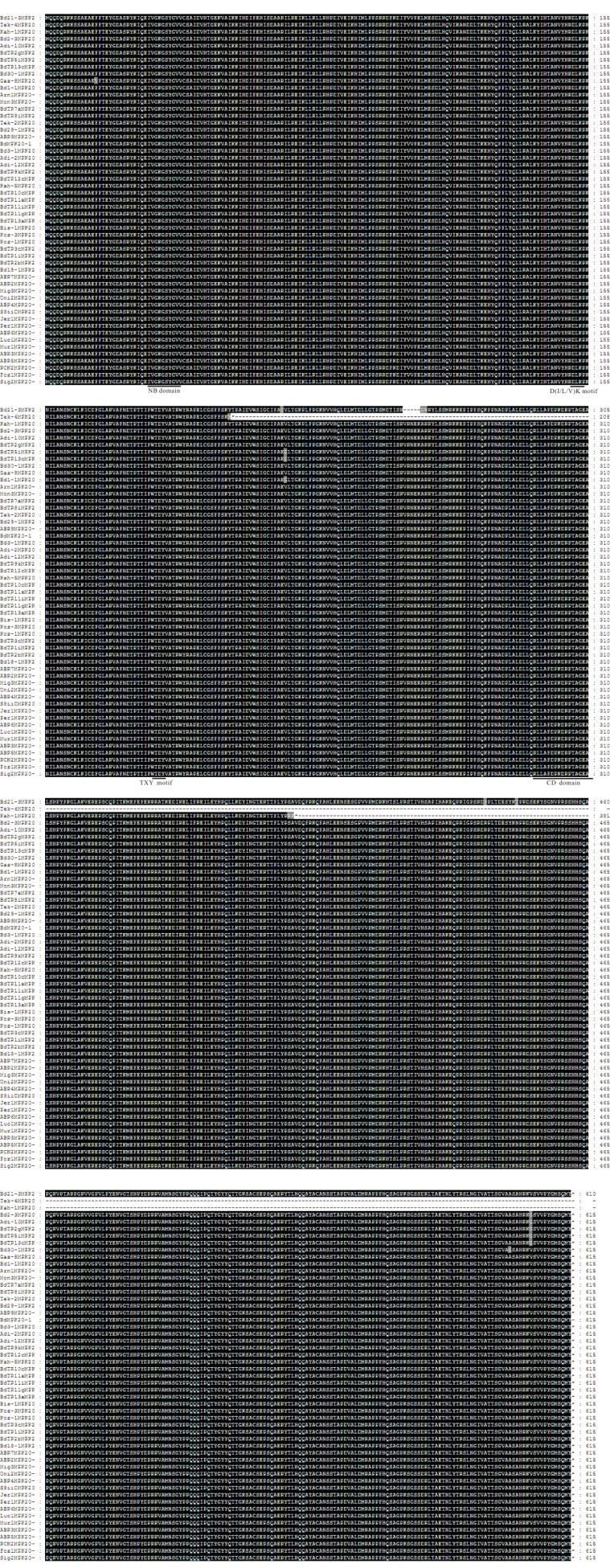


MPK20-2：


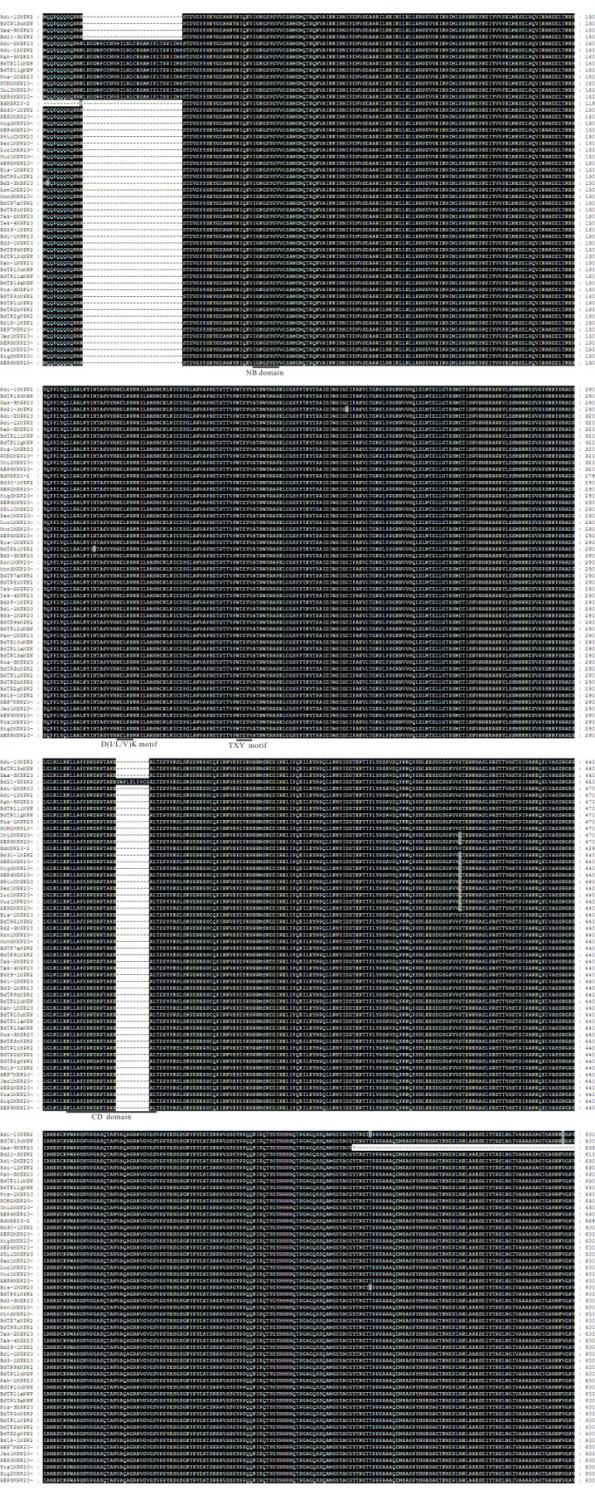


MPK20-3：


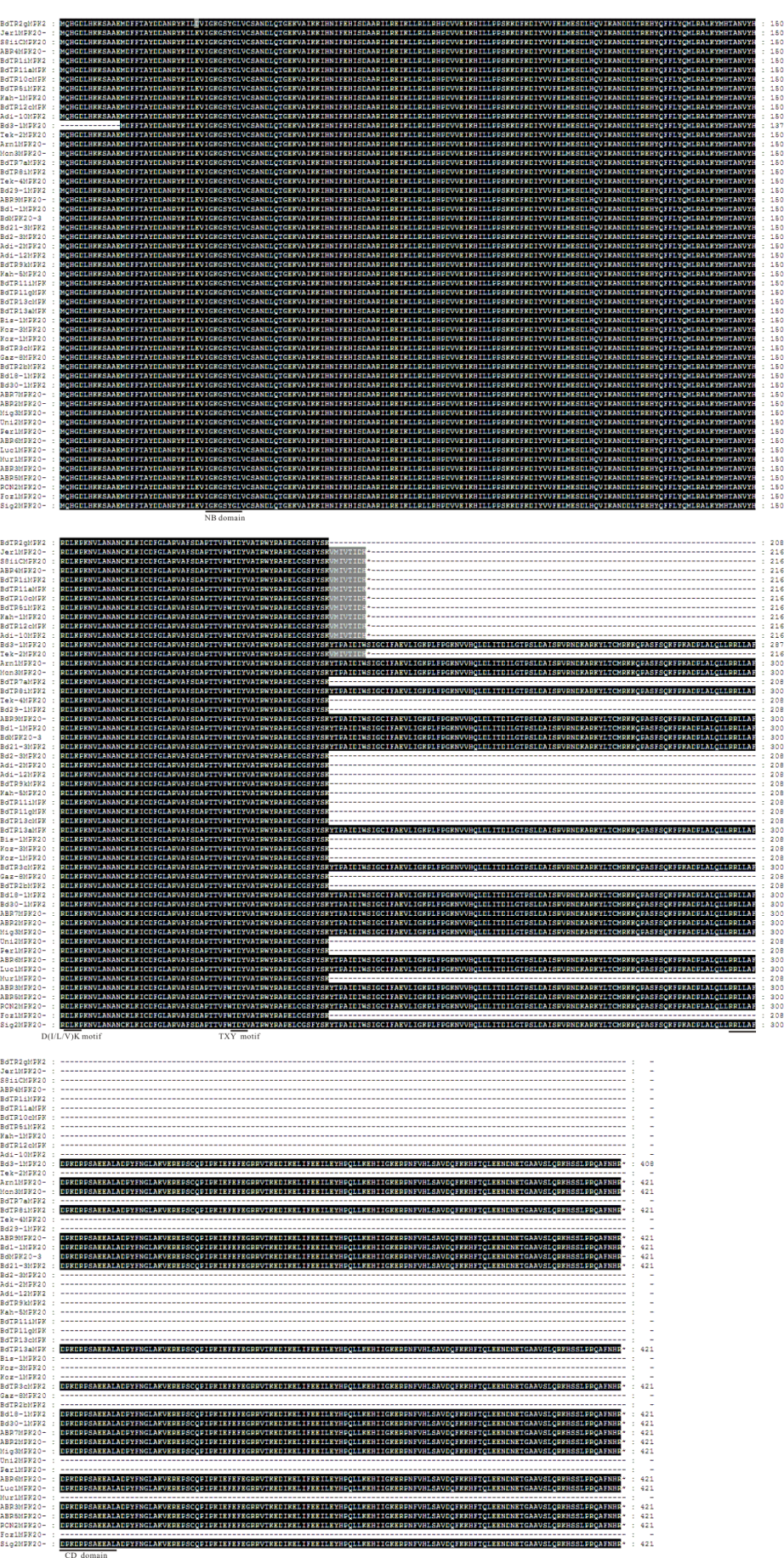


MPK20-4:


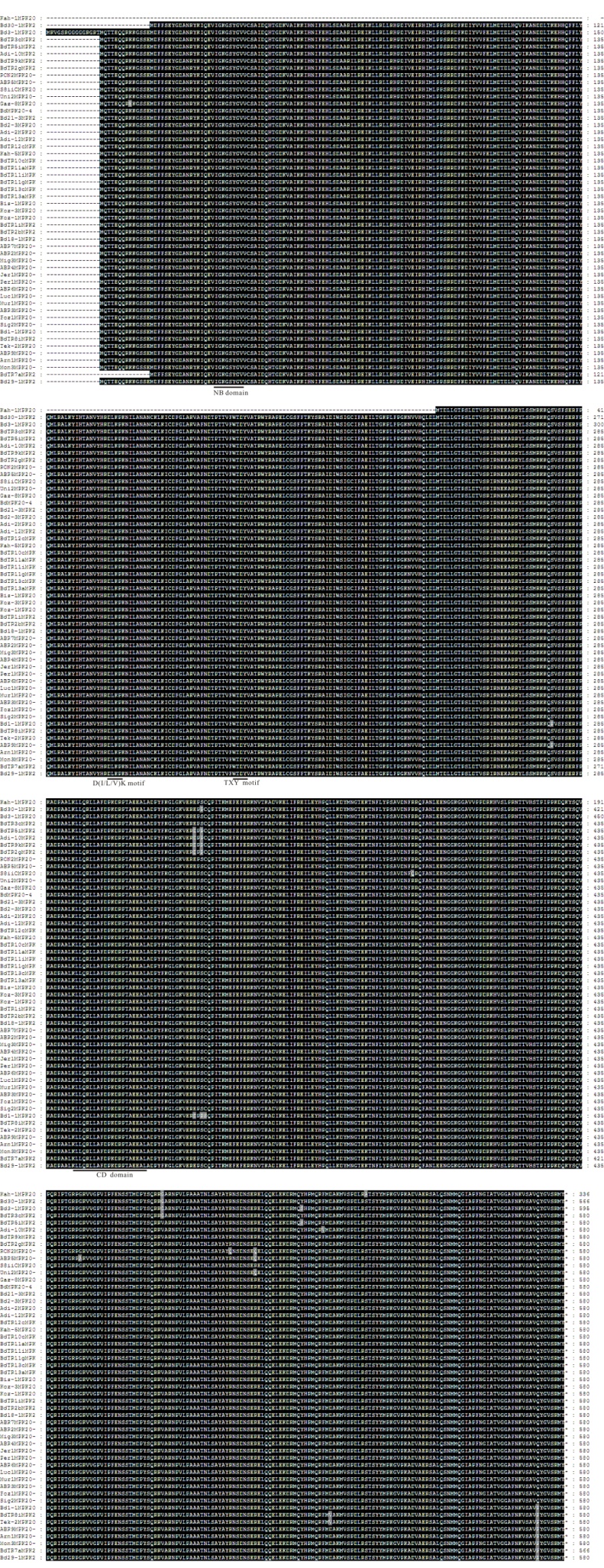


MPK20-5:


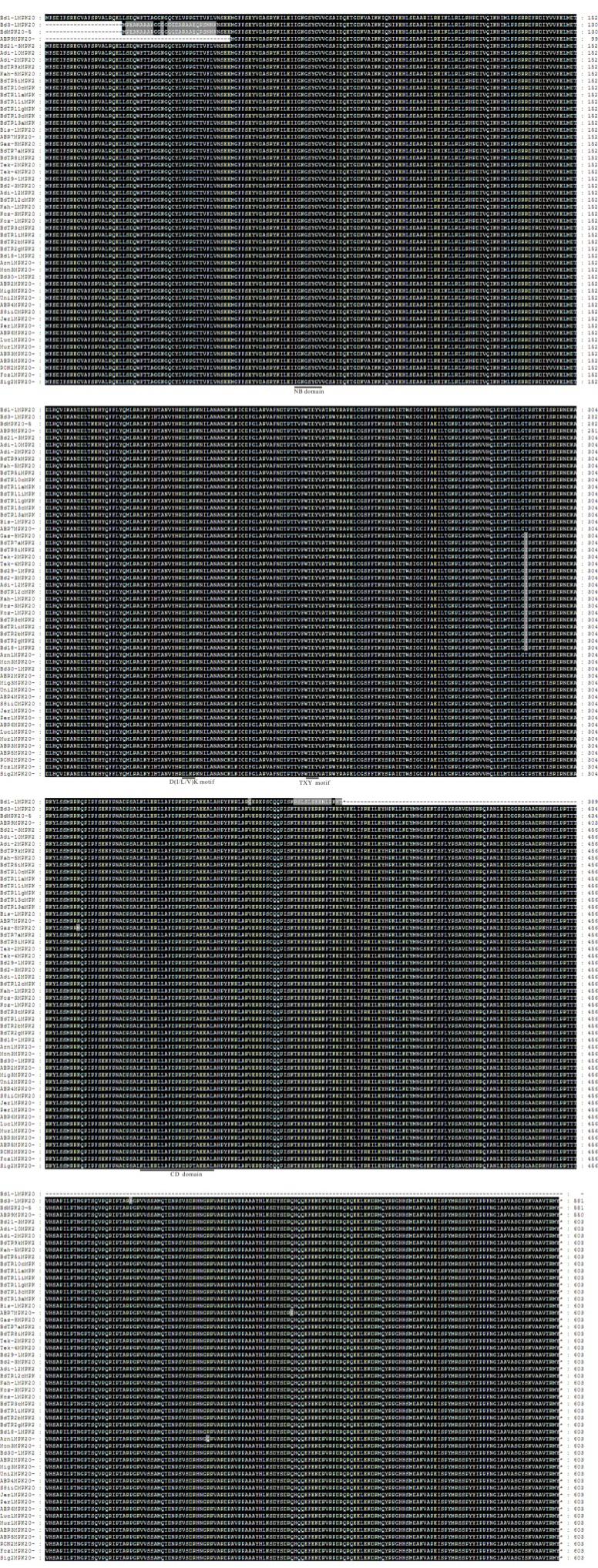


MPK21-1:


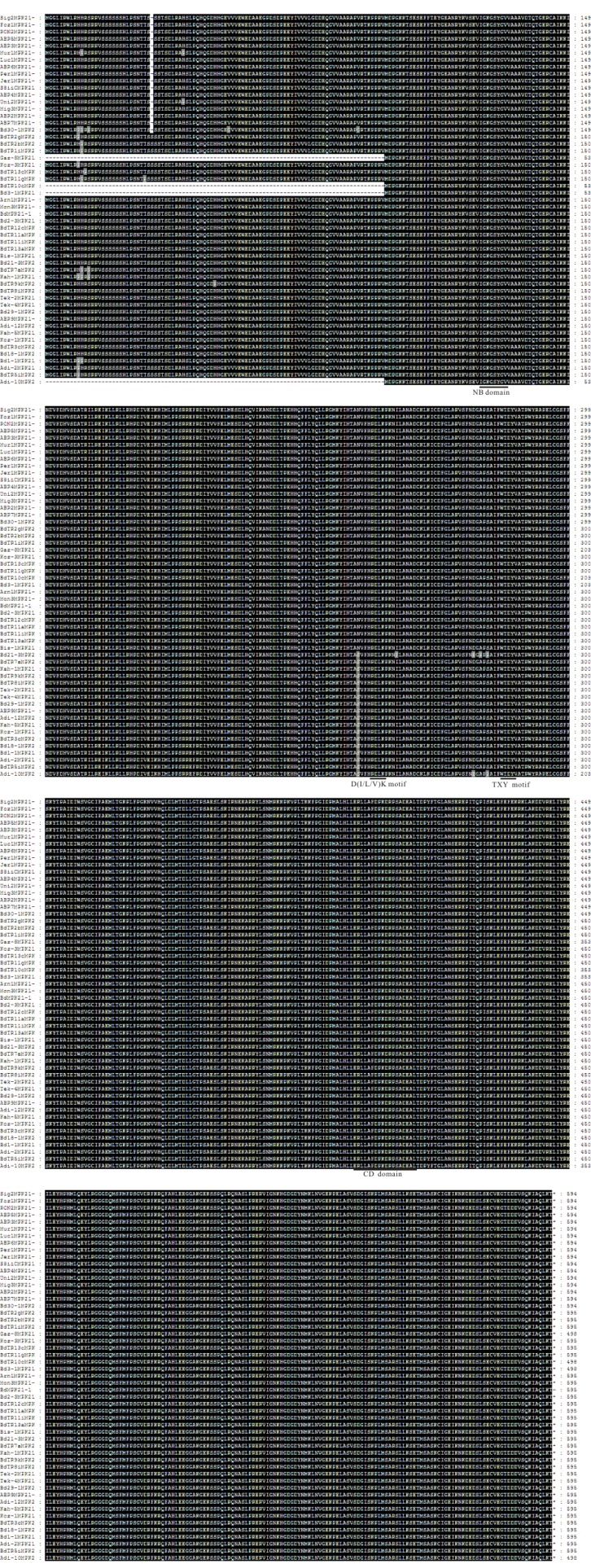


MPK21-2:


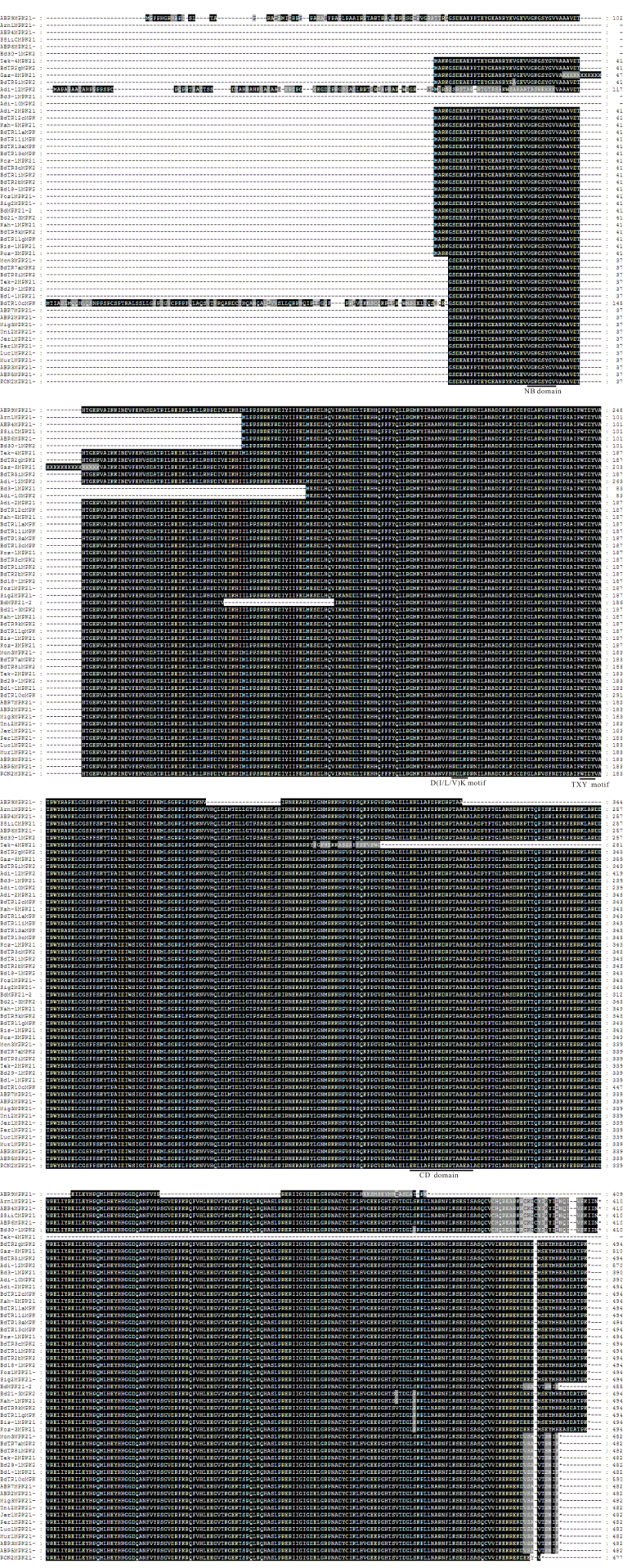

Supplement: Supplemental Information 8 [file peerj-09-11238-s008.docx]

MKK1：


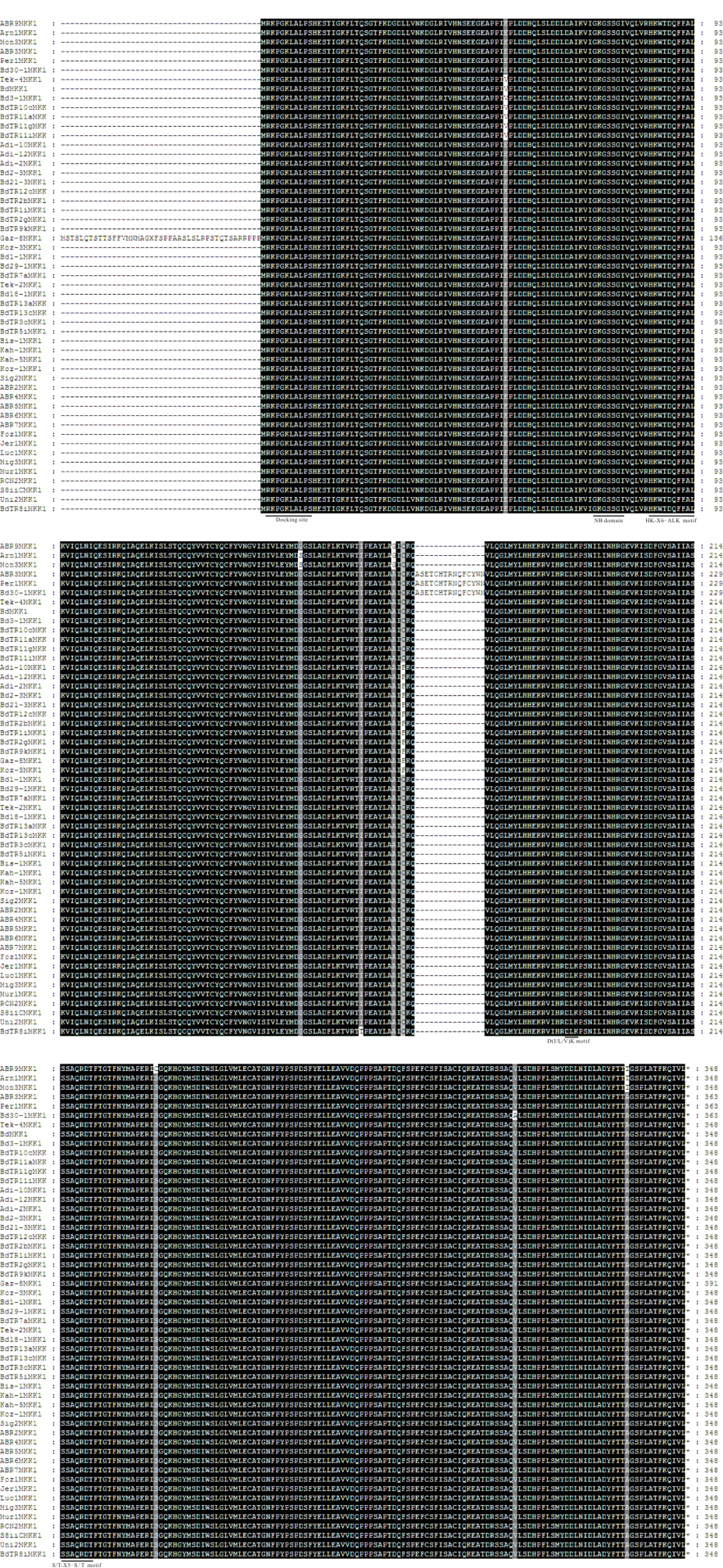


MKK3-1：


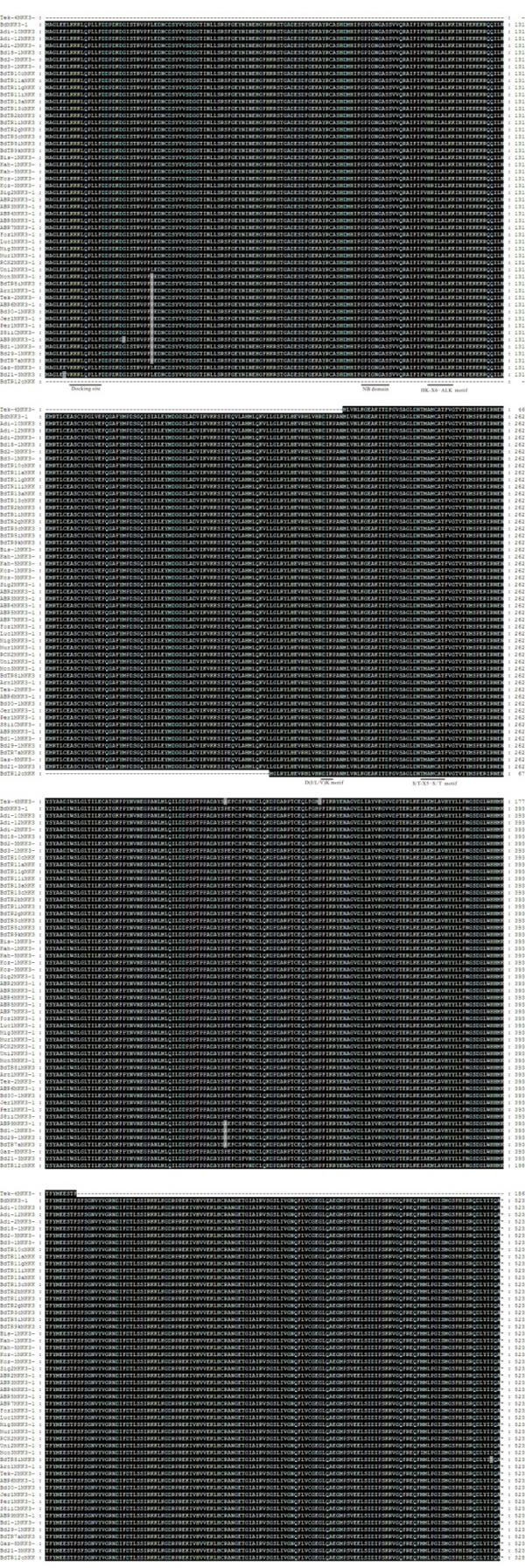


MKK3-2：


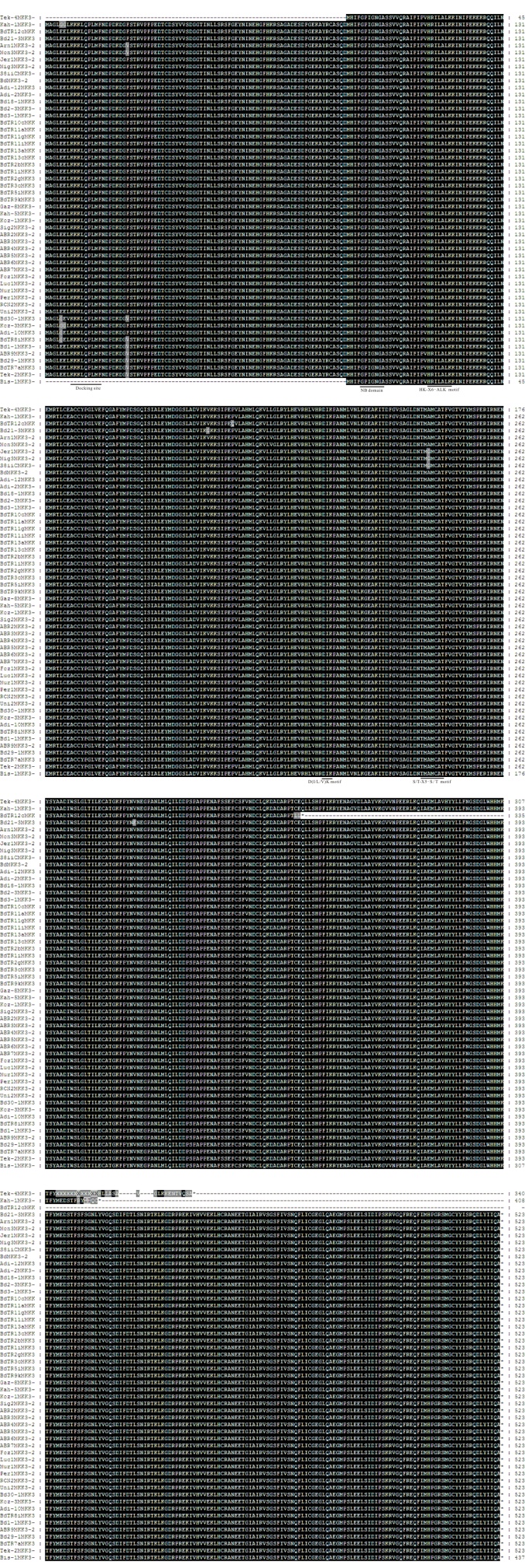


MKK3-3：


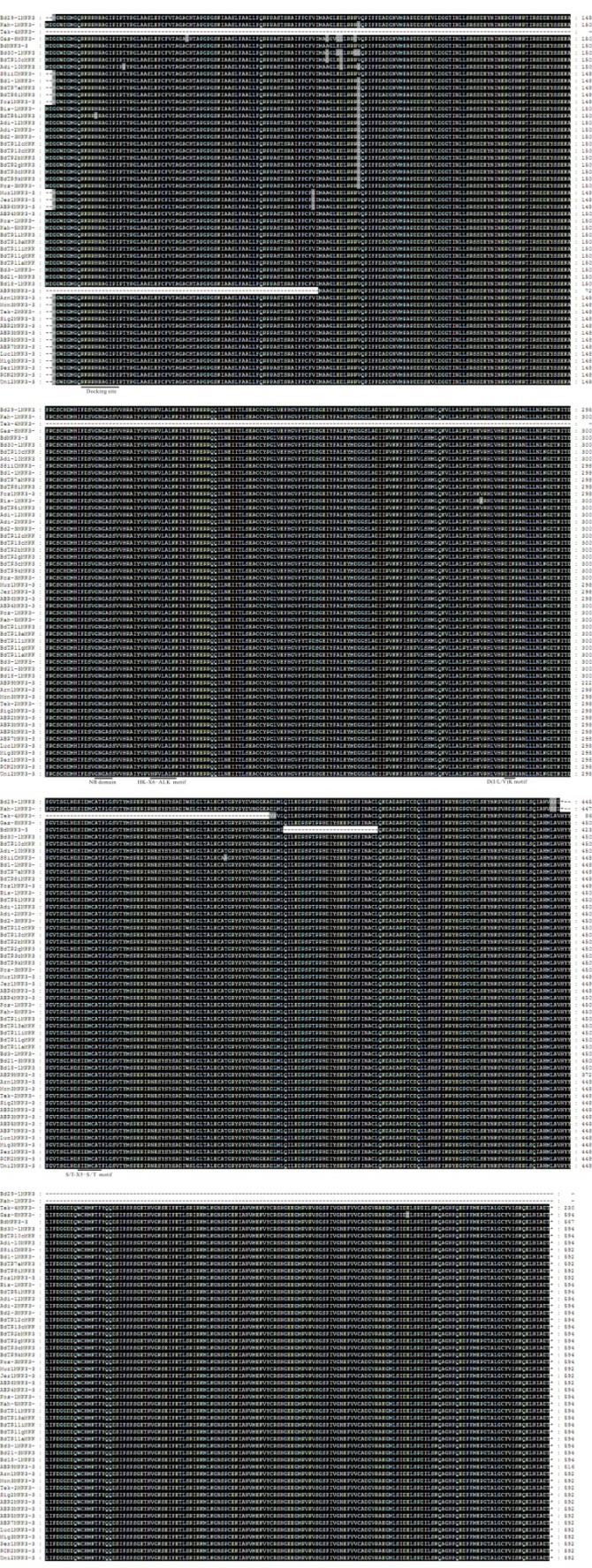


MKK4：


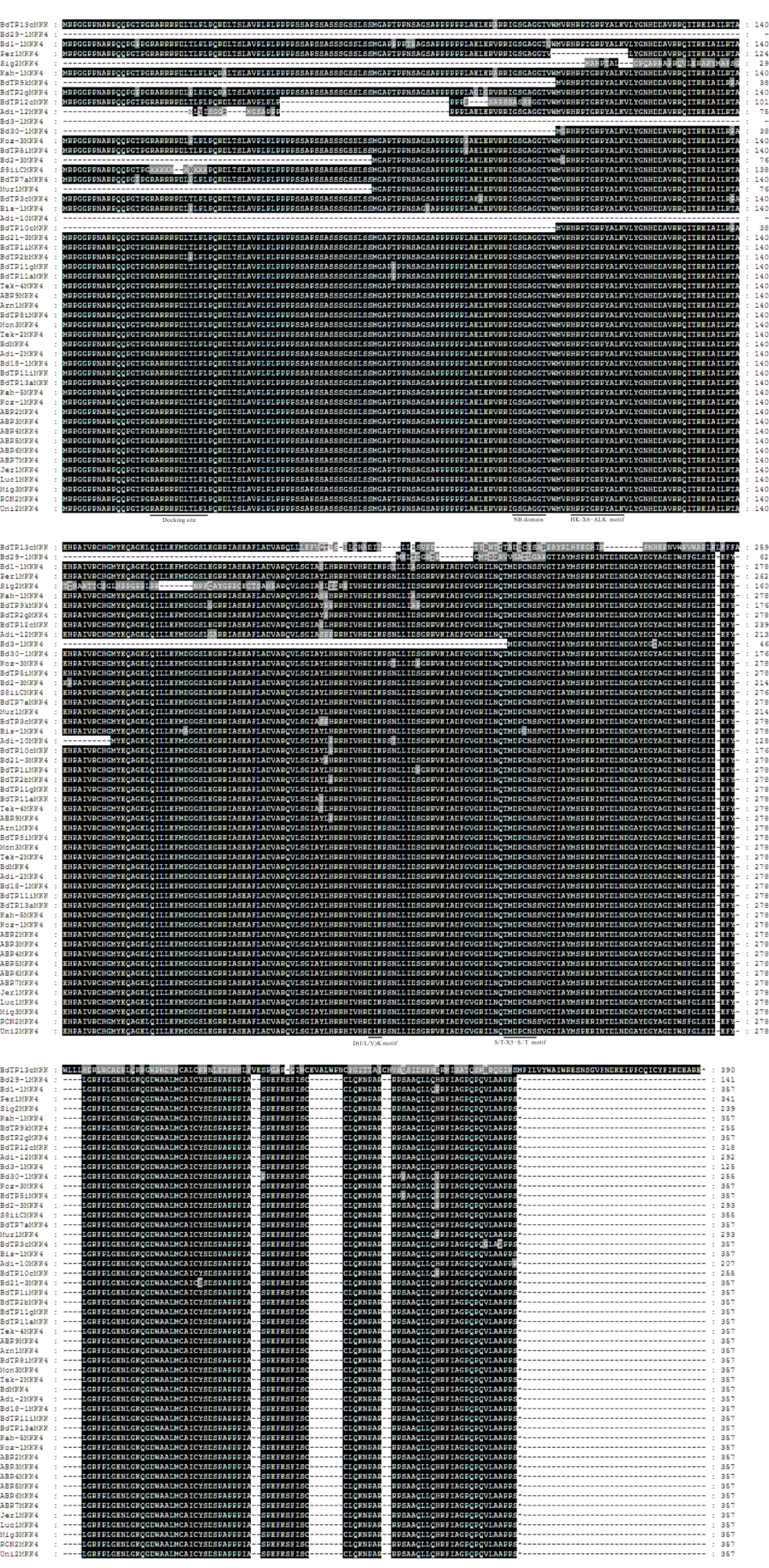


MKK5：


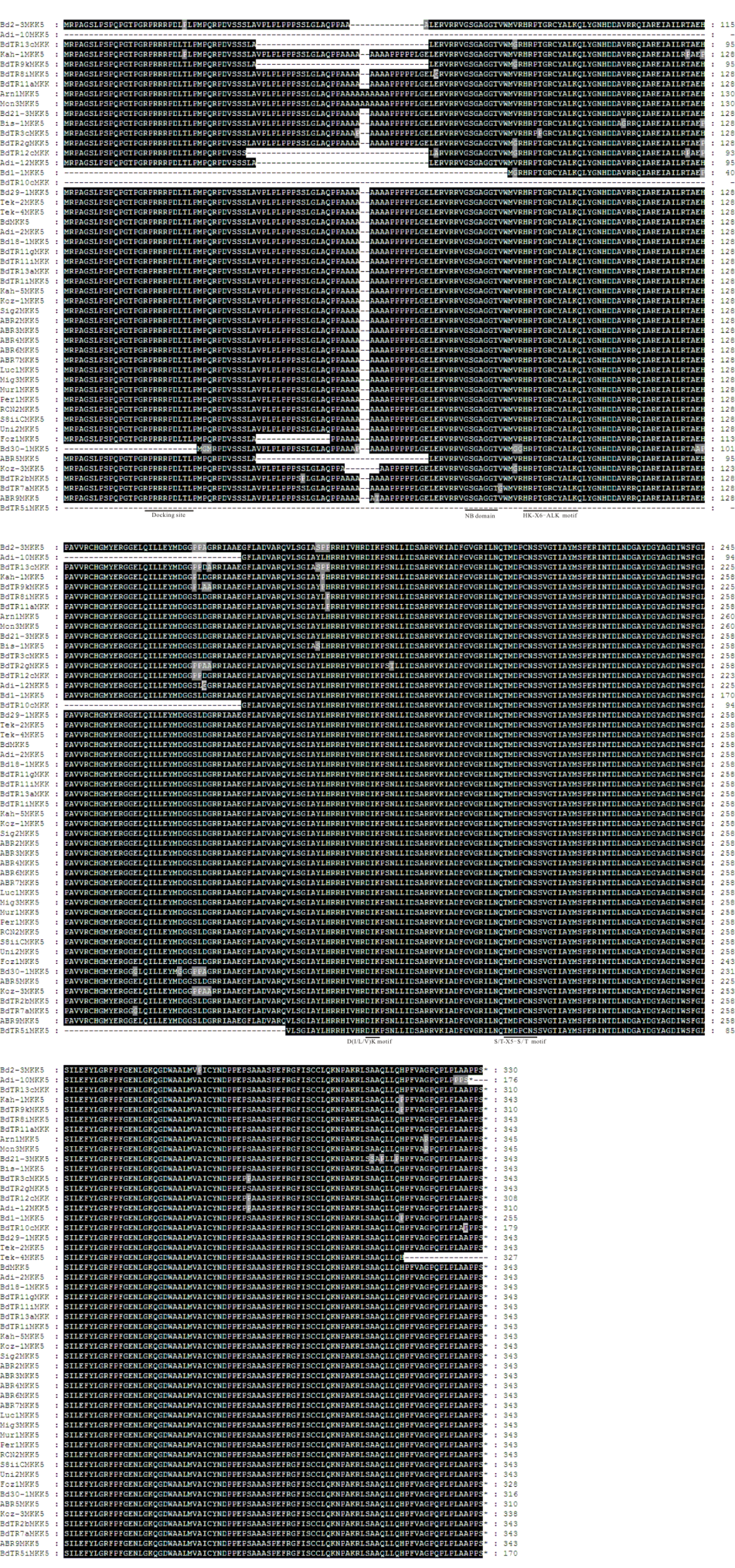


MKK6：


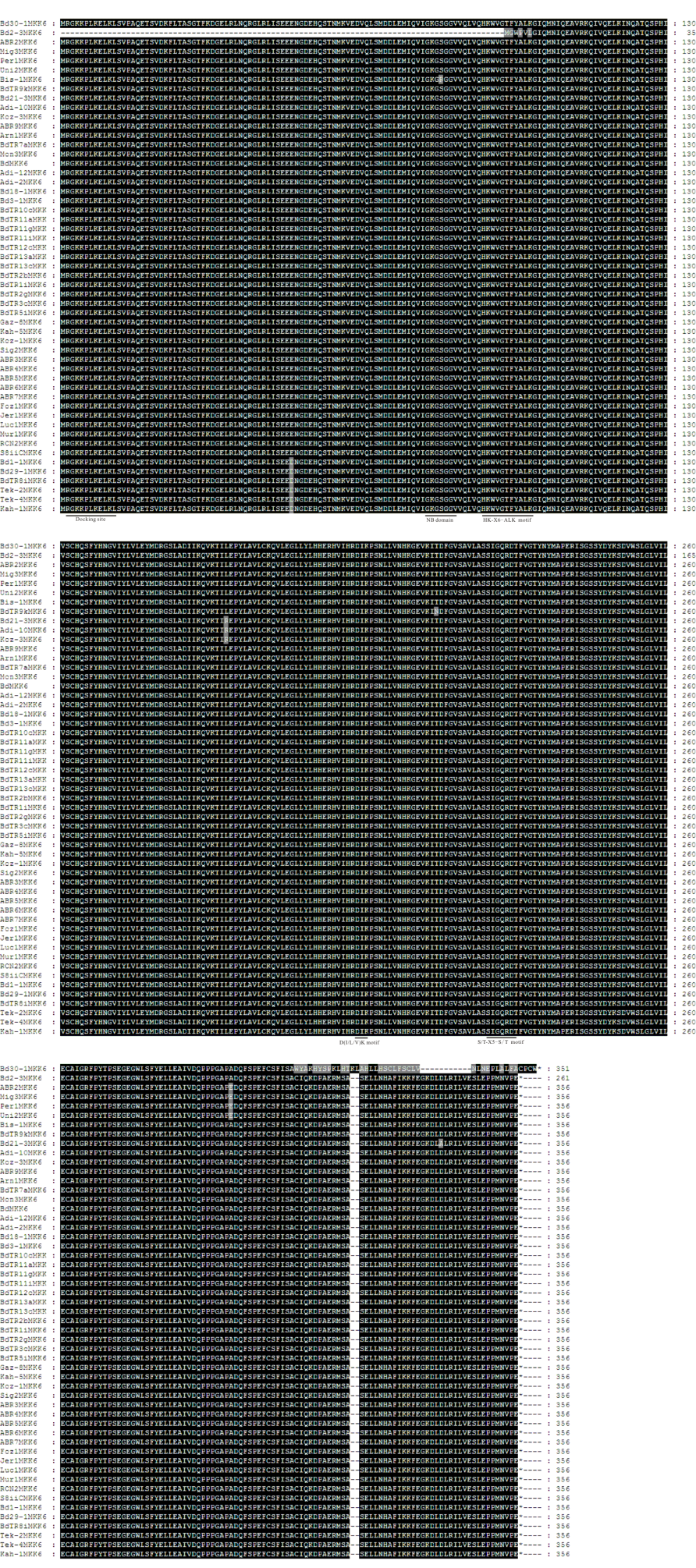


MKK10-1：


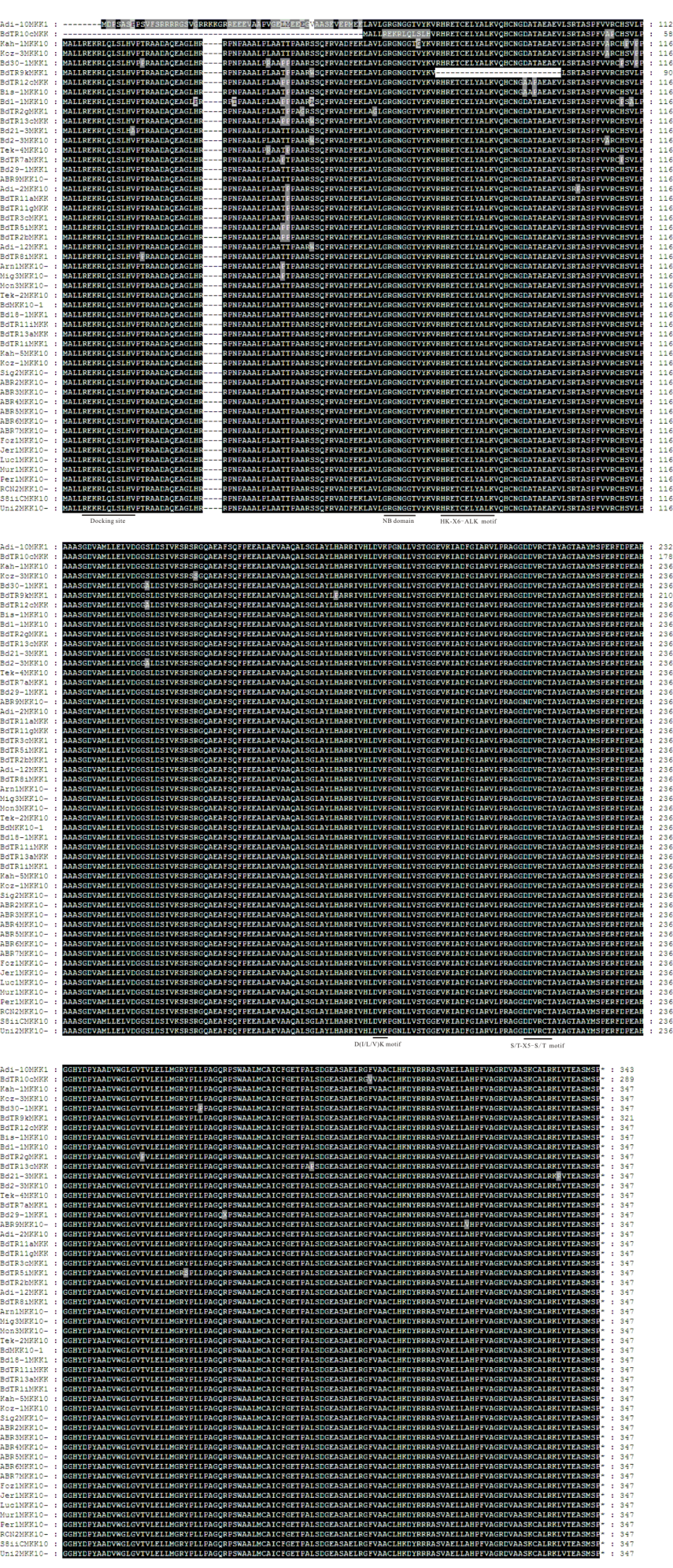


MKK10-2：


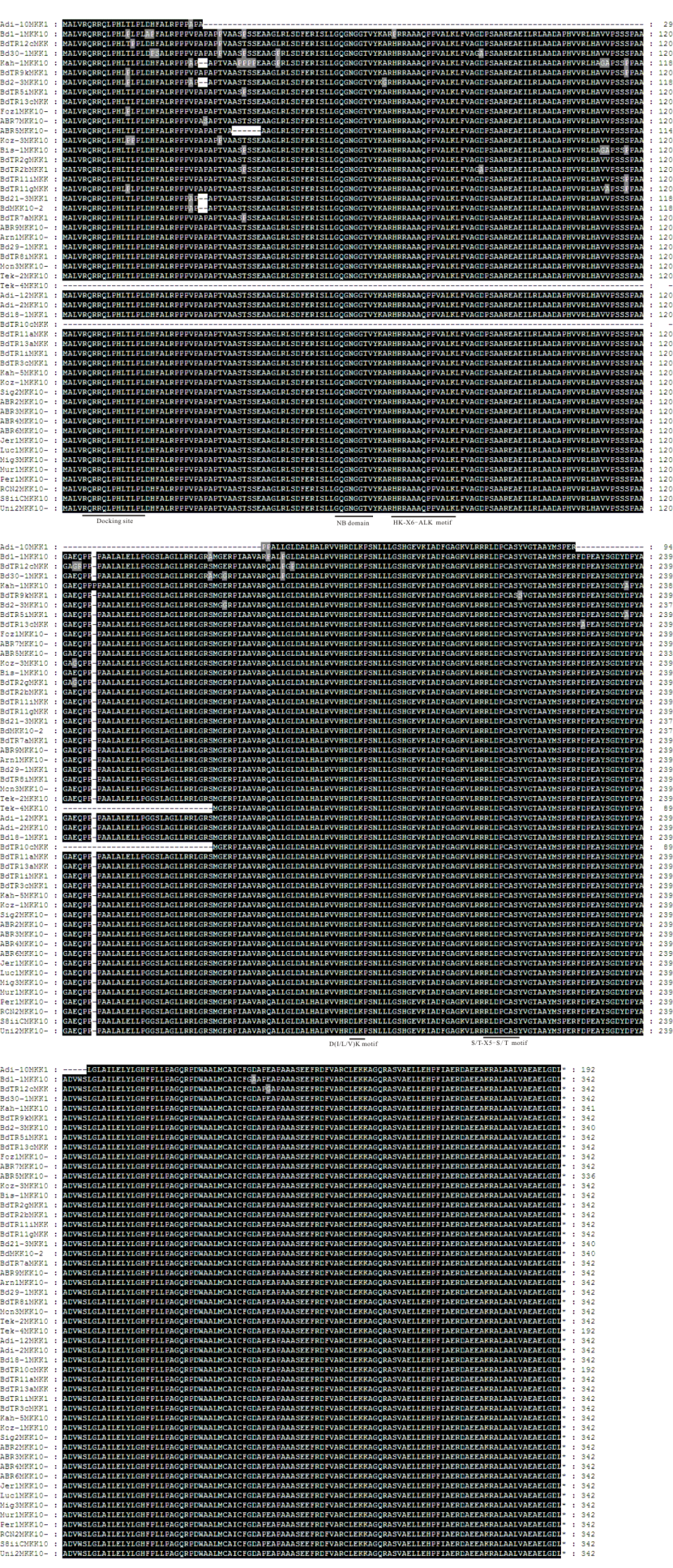


MKK10-3：


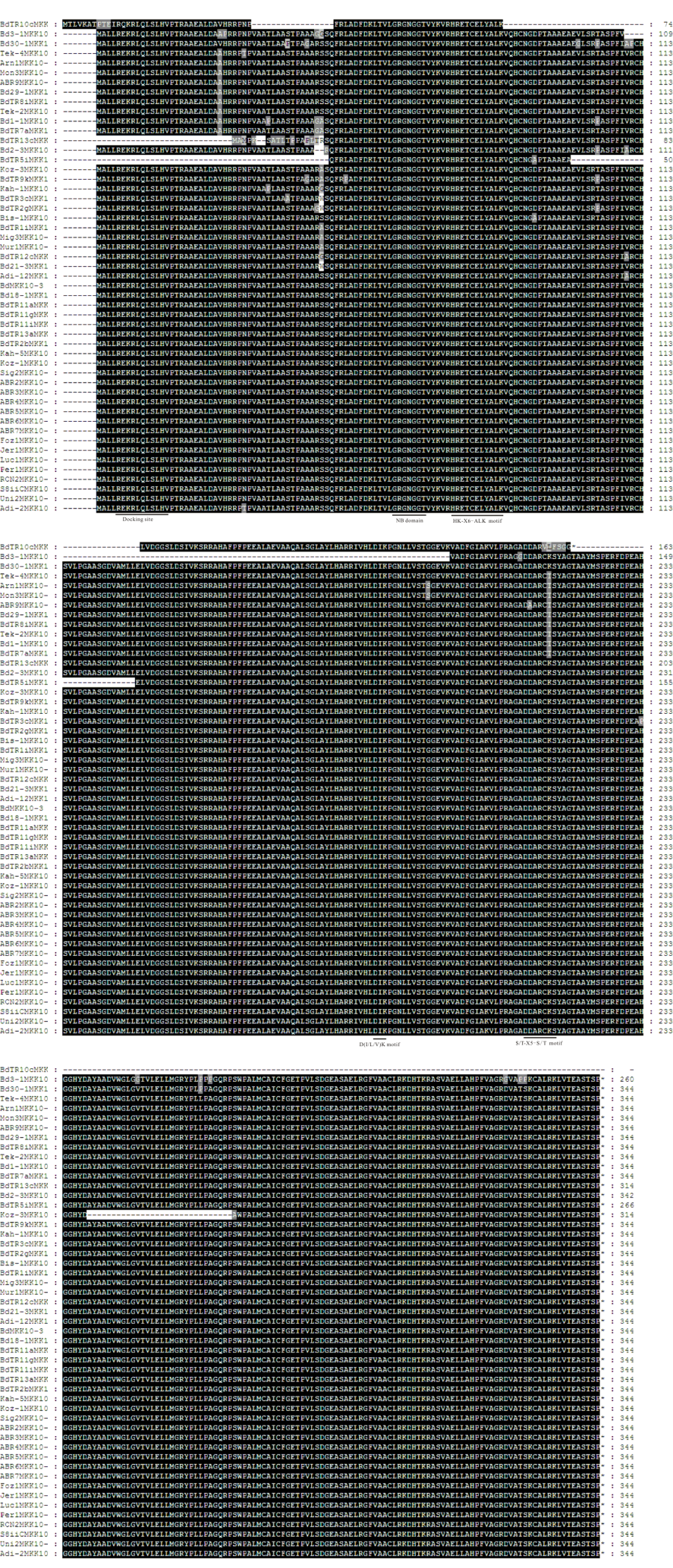


MKK10-4：


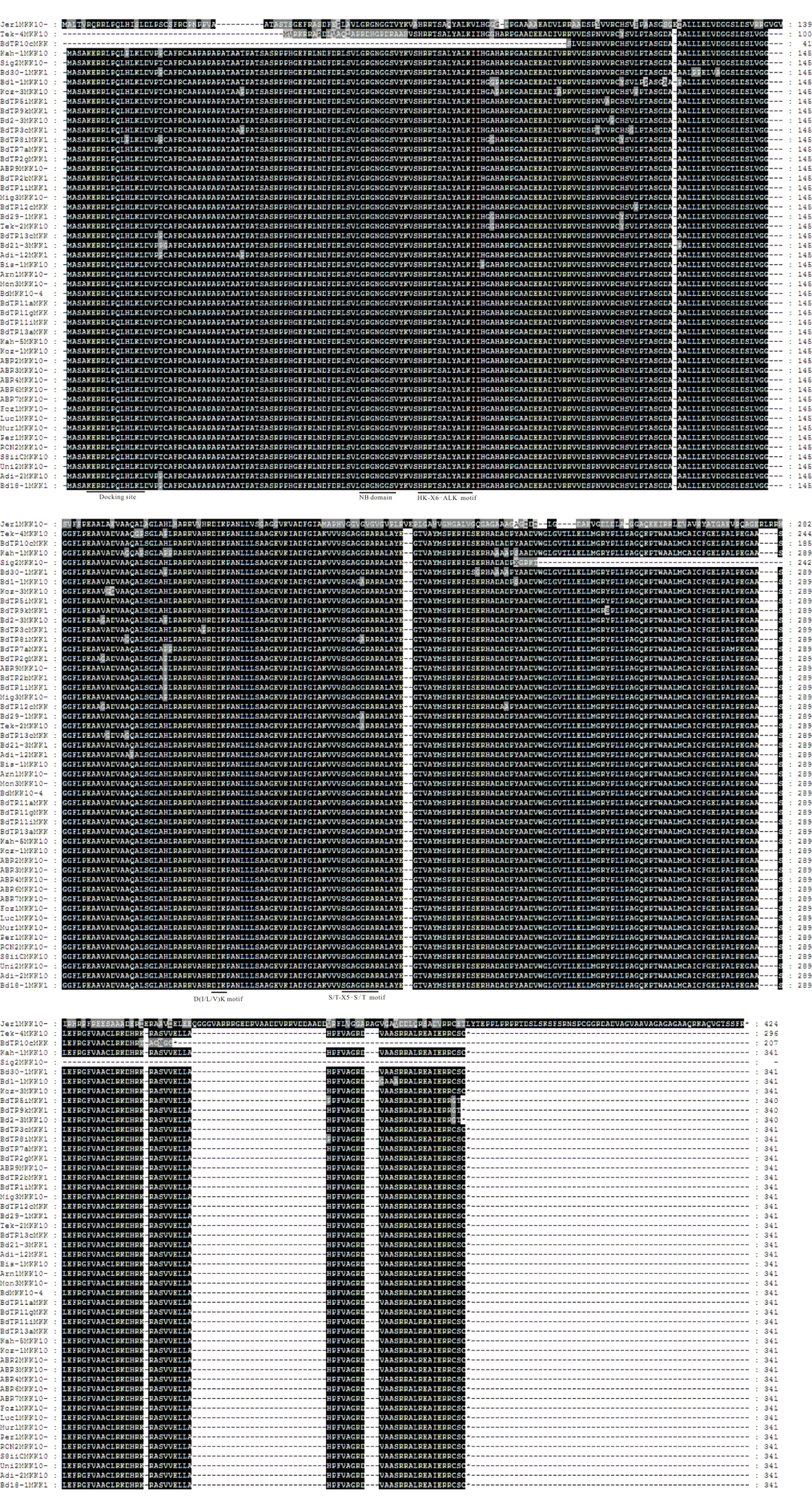


MKK10-5：


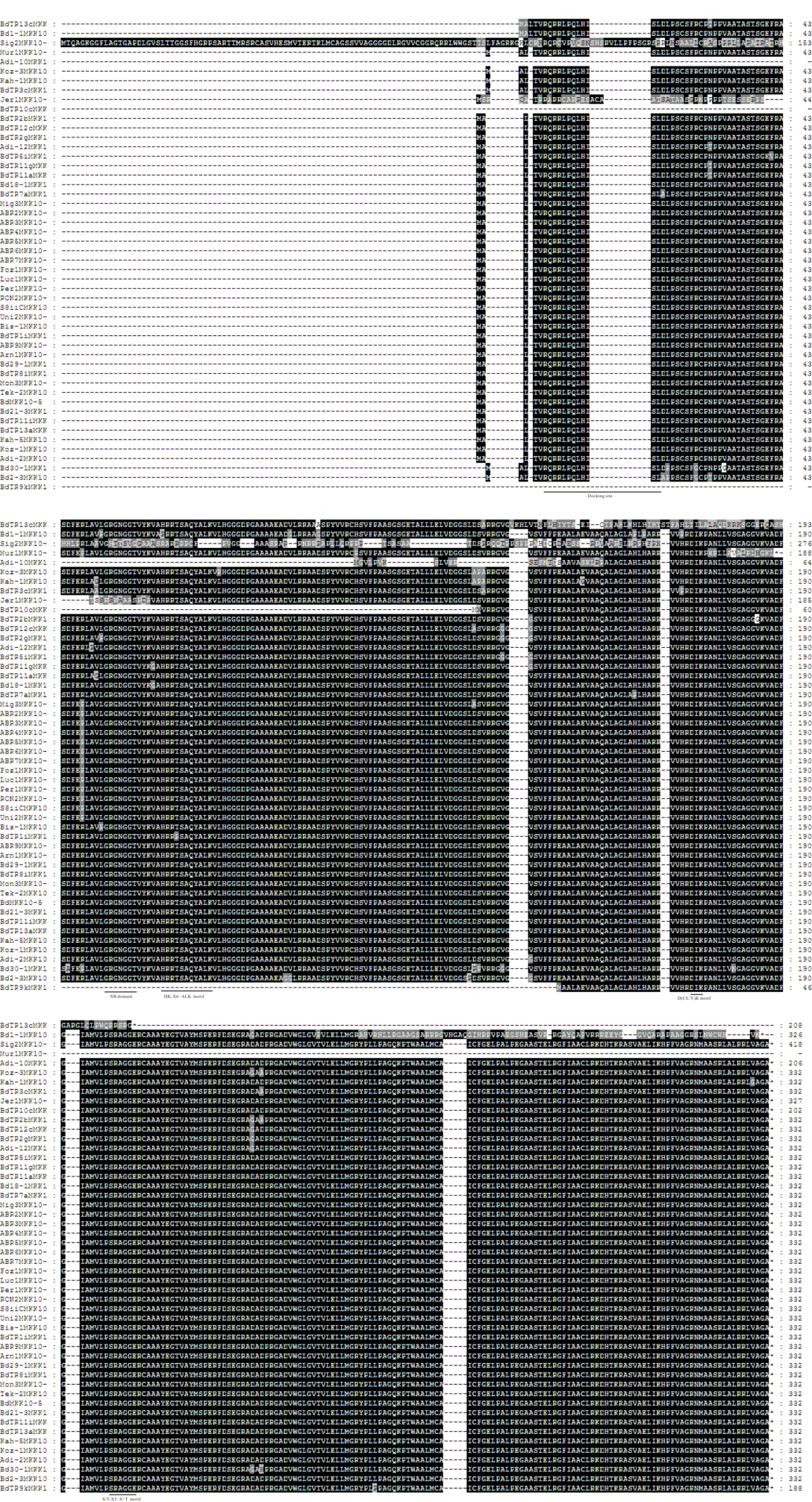

Supplement: Supplemental Information 10 [file peerj-09-11238-s010.docx]
