# Supplementary material for: Comparative analysis of MAPK and MKK gene families reveals differential evolutionary patterns in Brachypodium distachyon inbred lines
Source: PeerJ. 2021 Apr 6;9:e11238. doi: 10.7717/peerj.11238 (PMC8034371; doi:10.7717/peerj.11238)

Fig. S4. The exon/intron structures of Brachypodium inbred lines *MPK* genes.

MPK3：


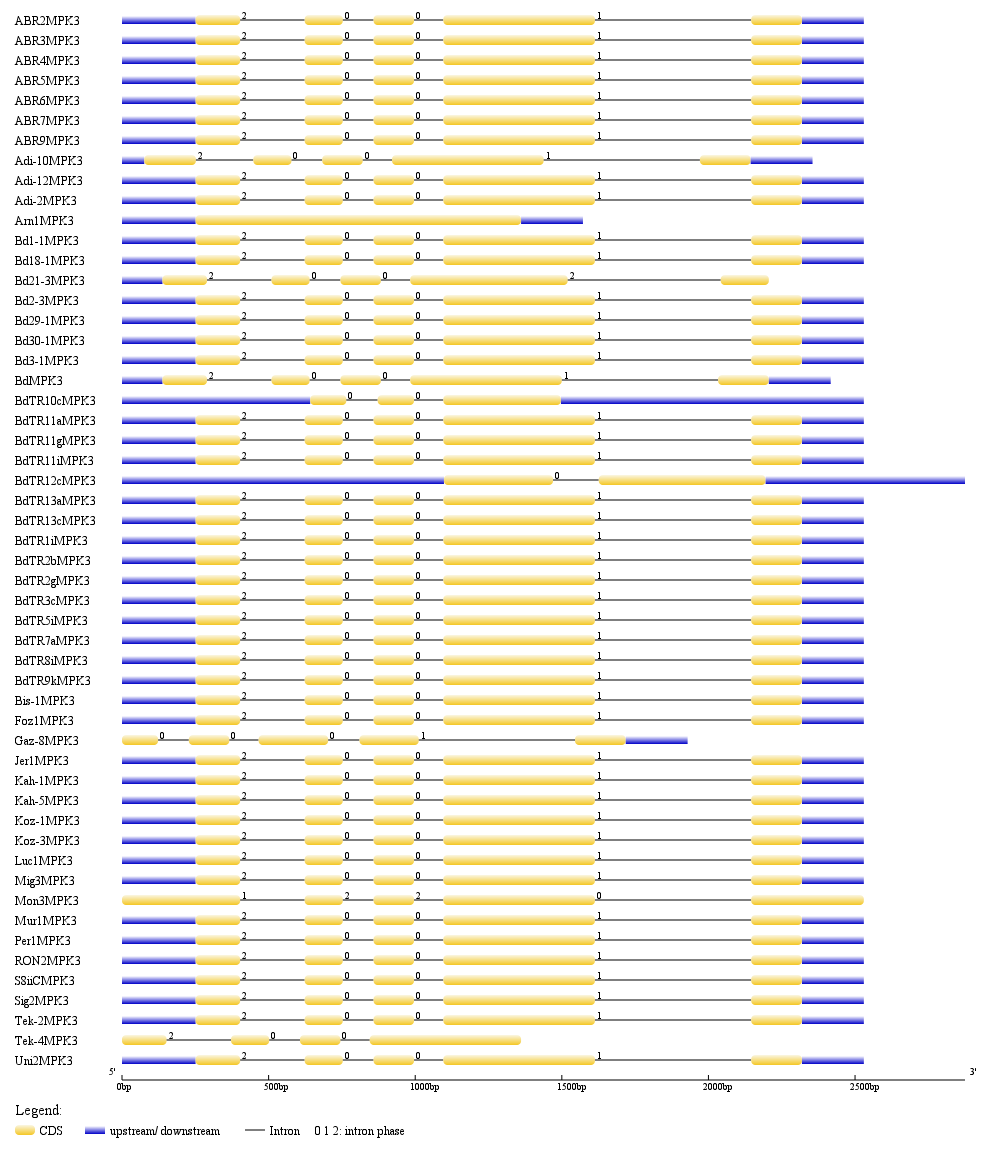


MPK4：


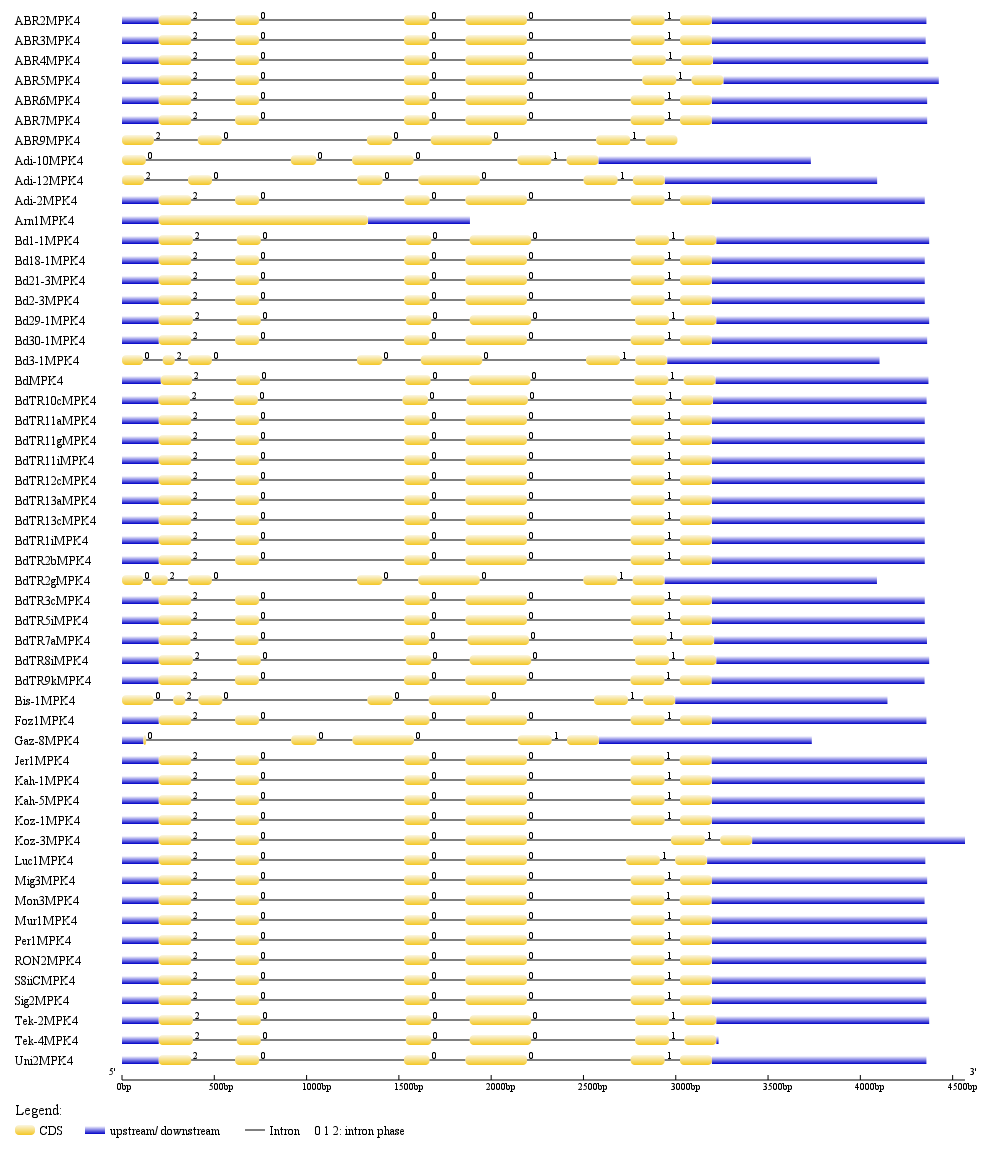


MPK6：


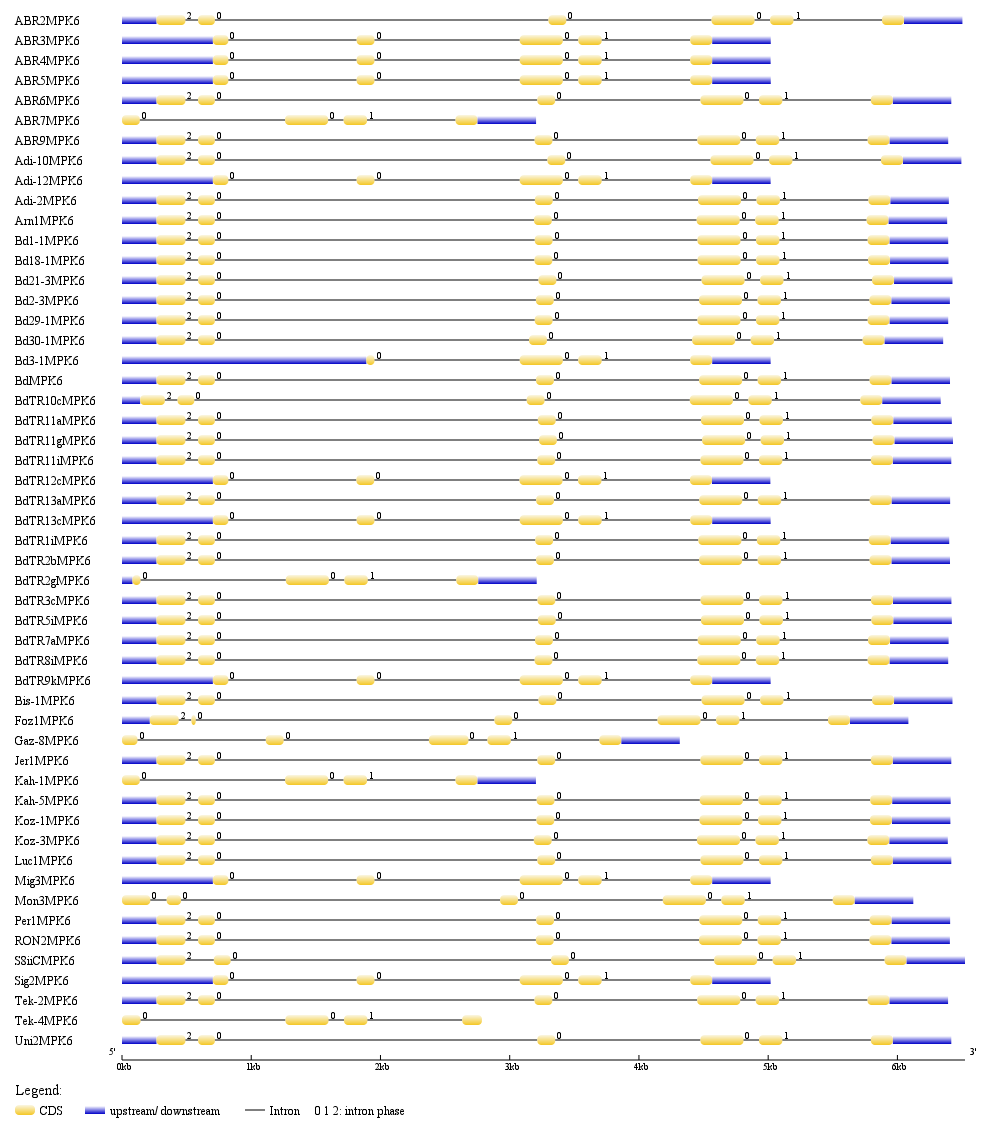


MPK7-1：


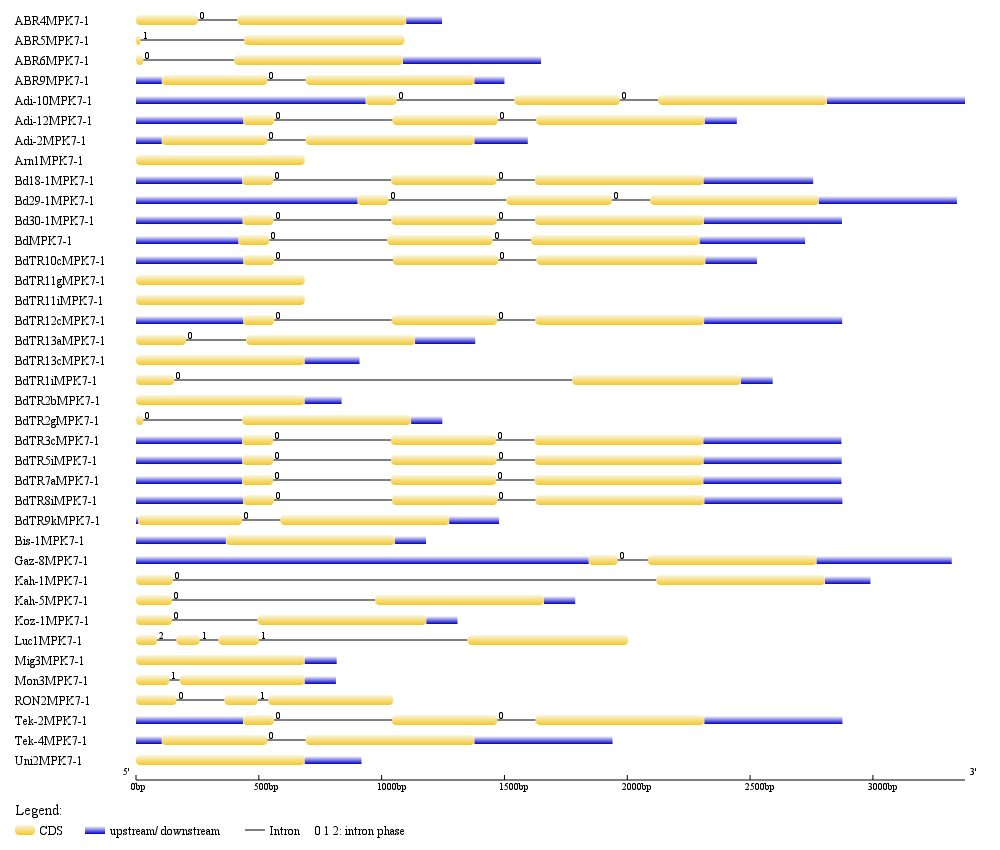


MPK7-2：


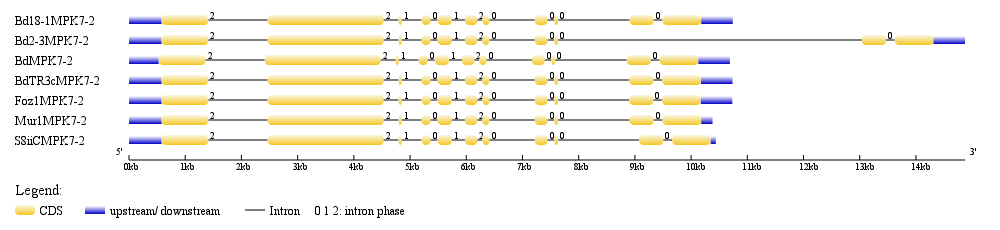


MPK11：


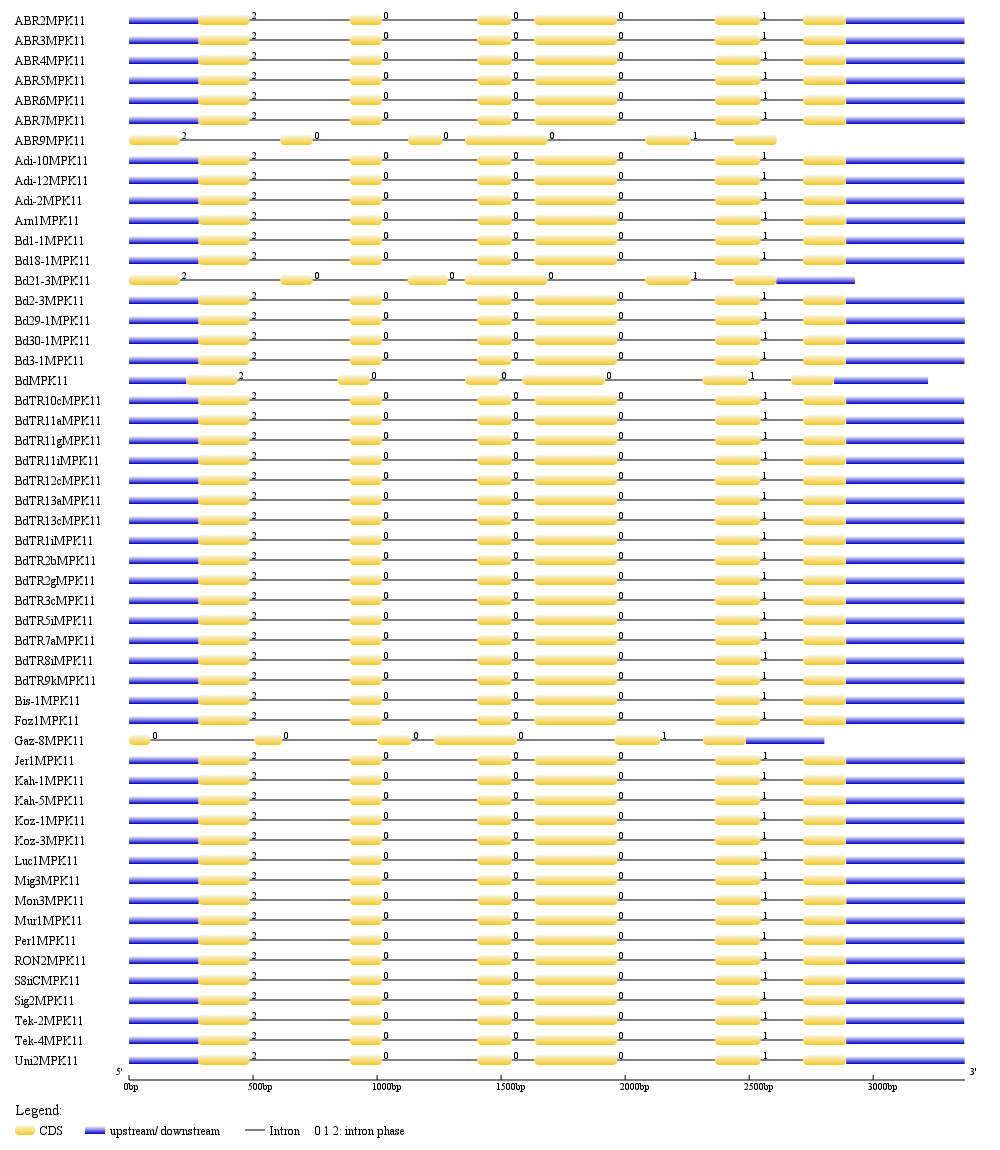


MPK14：


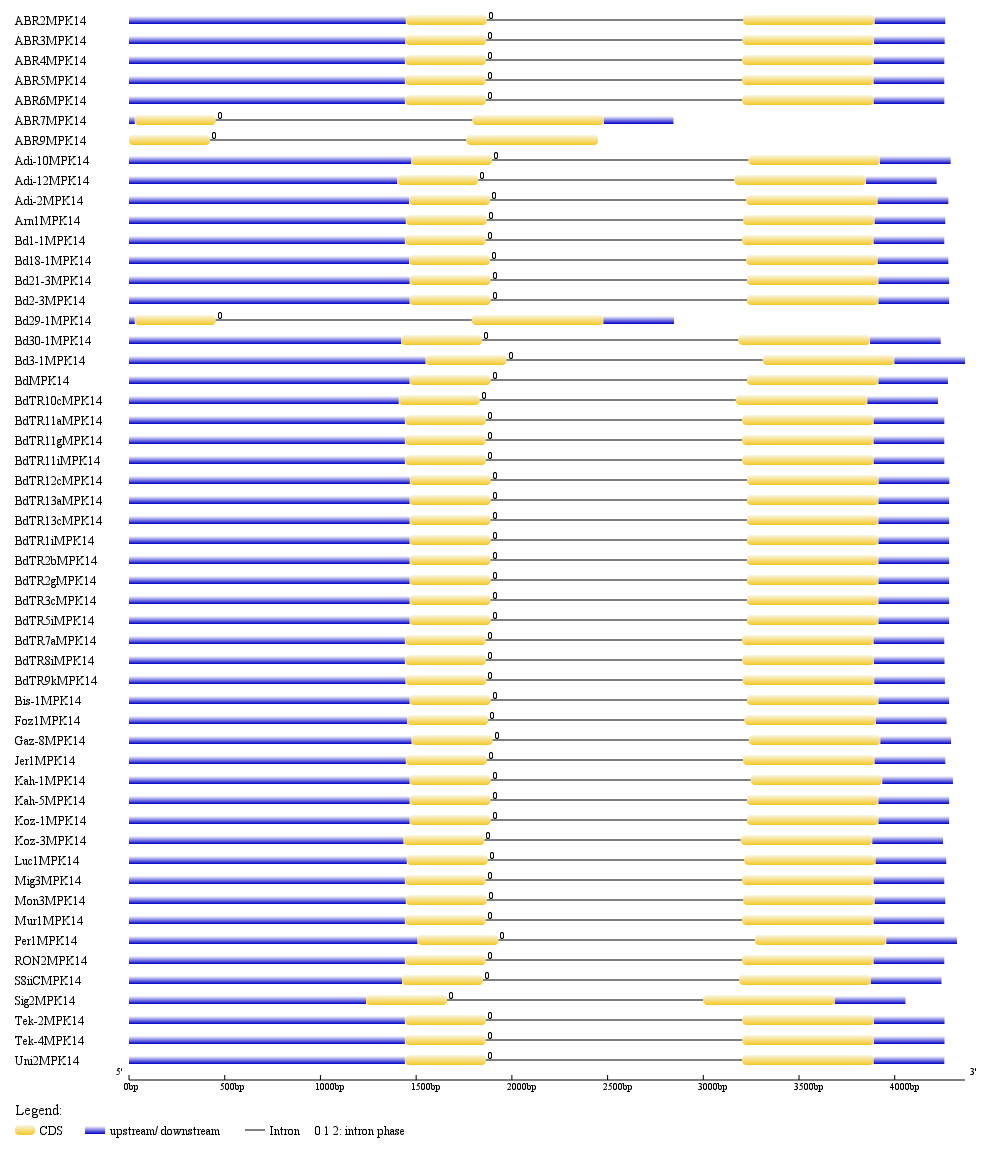


MPK16：


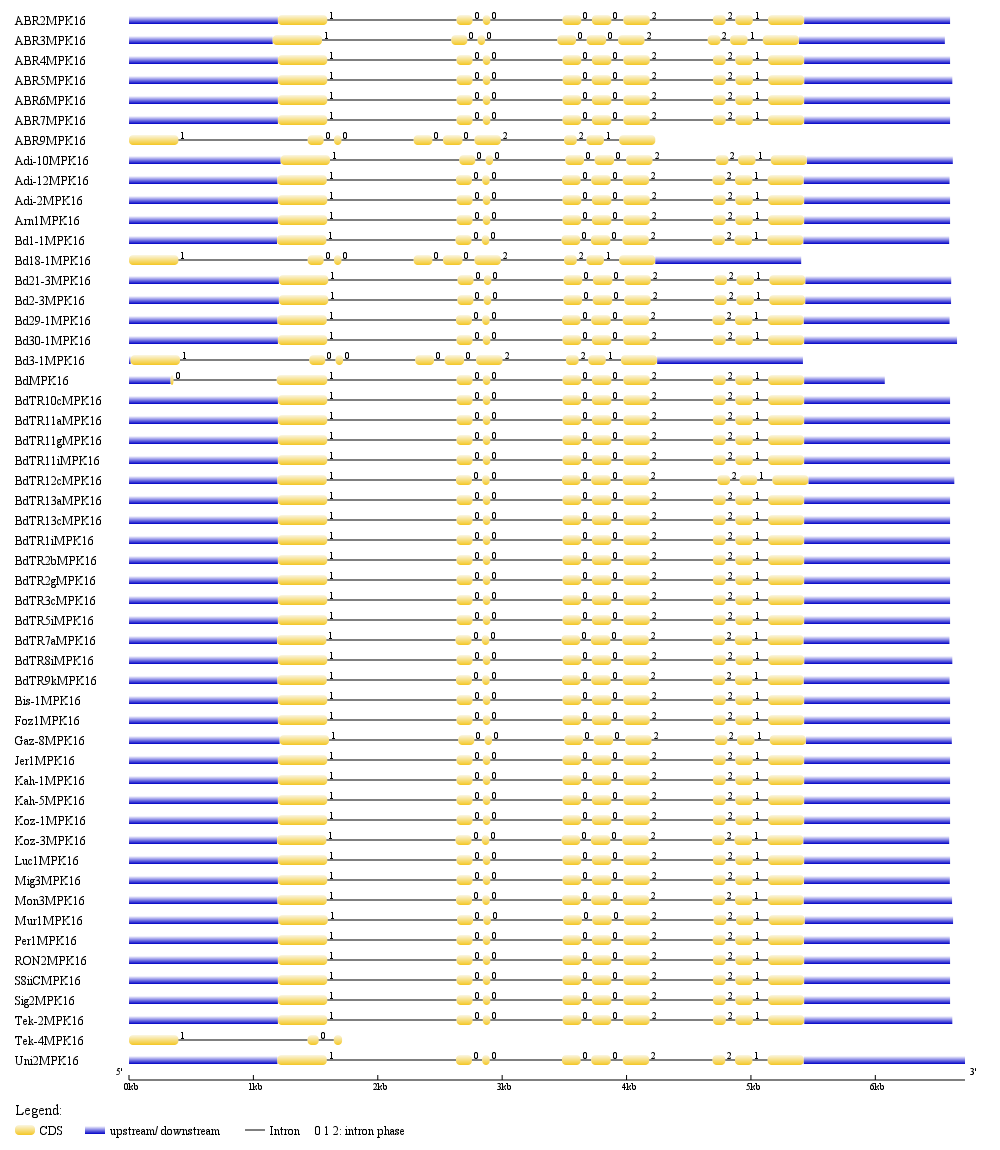


MPK17：


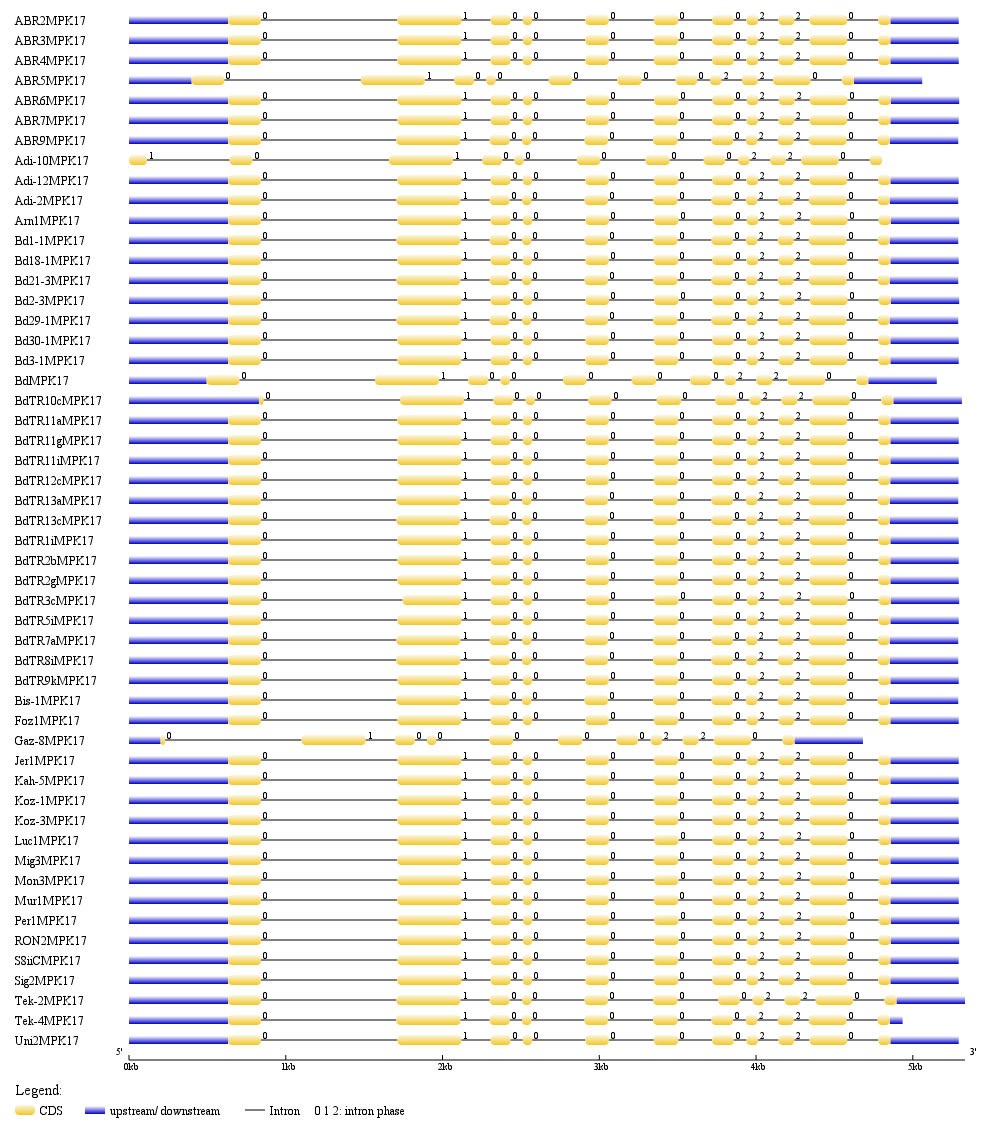


MPK20-1：


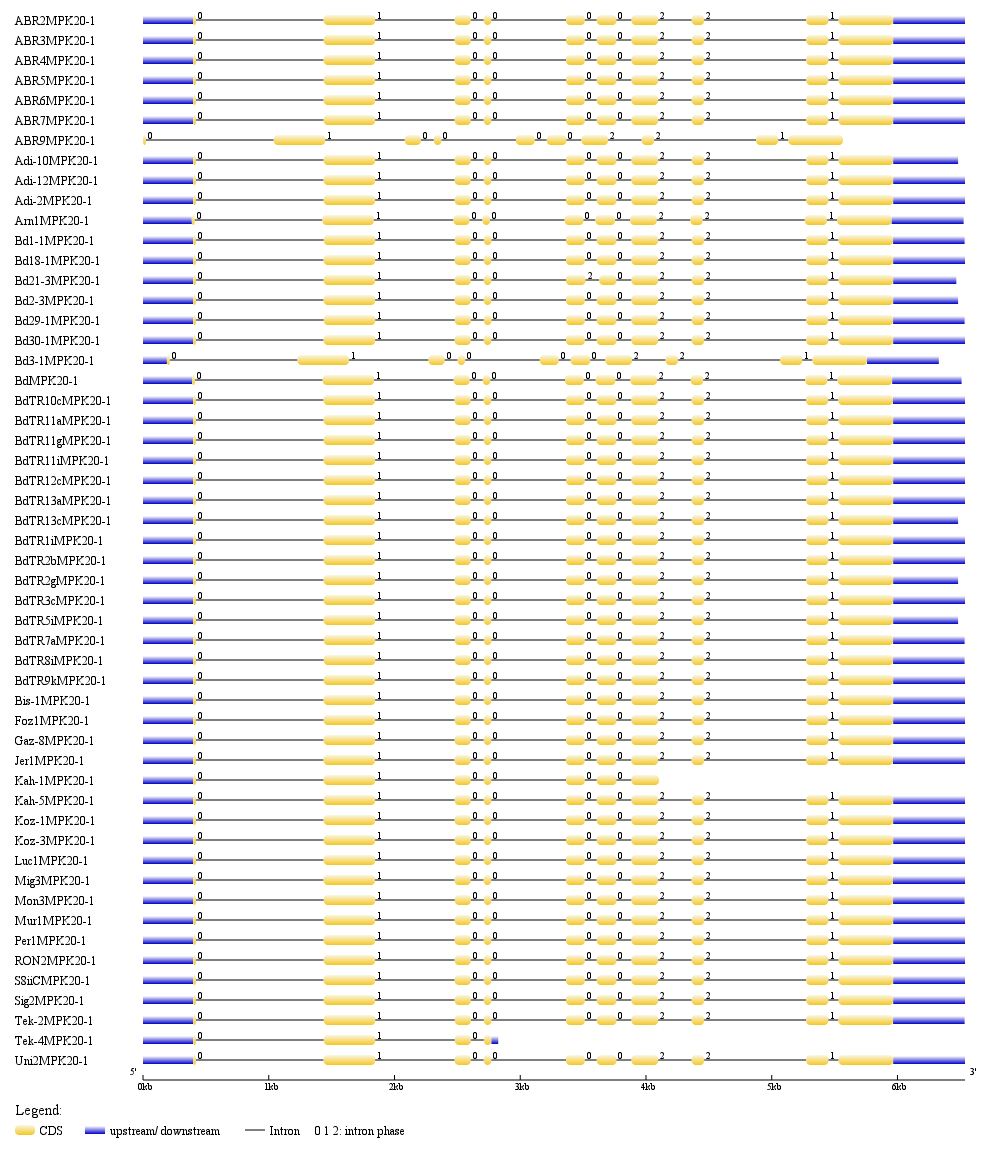


MPK20-2：


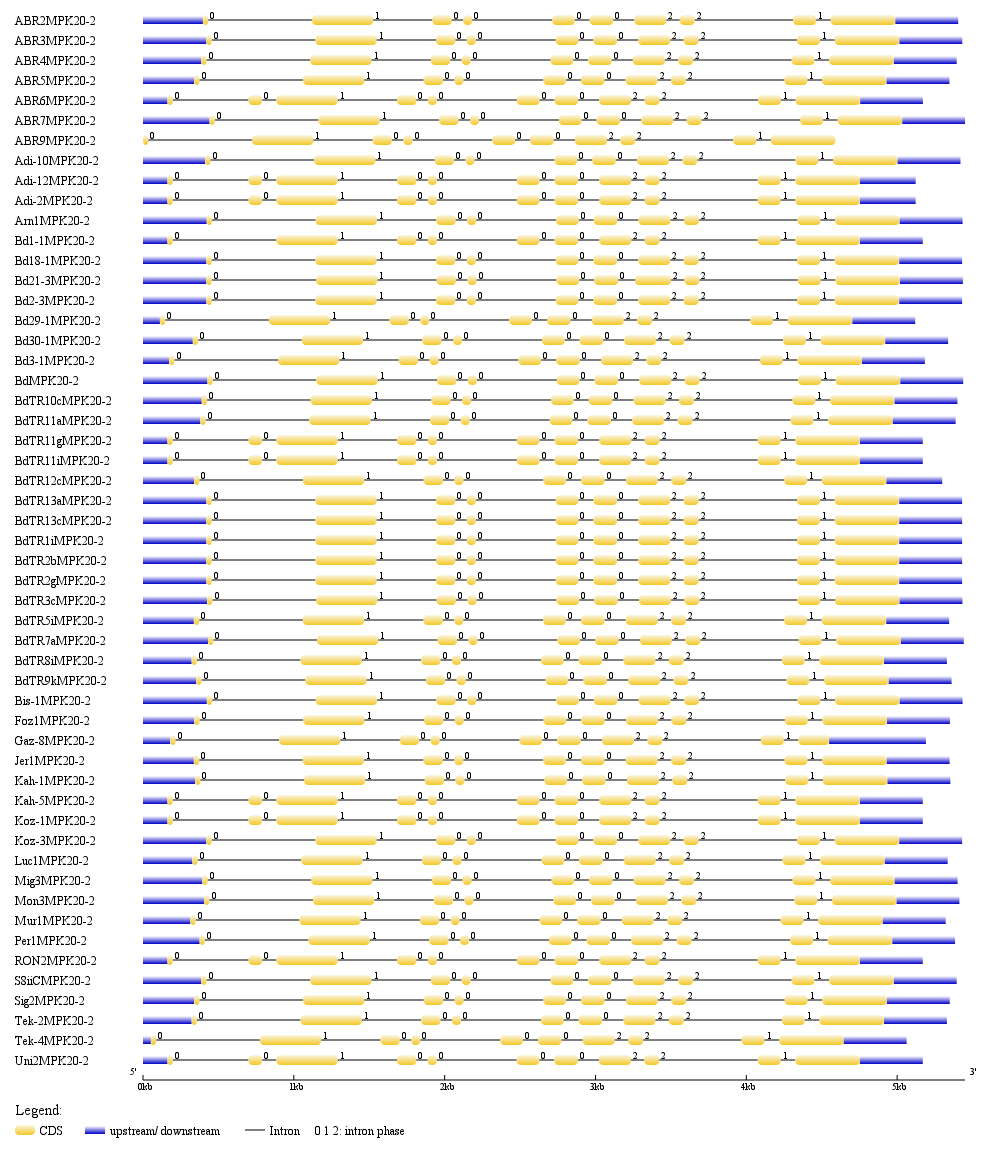


MPK20-3：


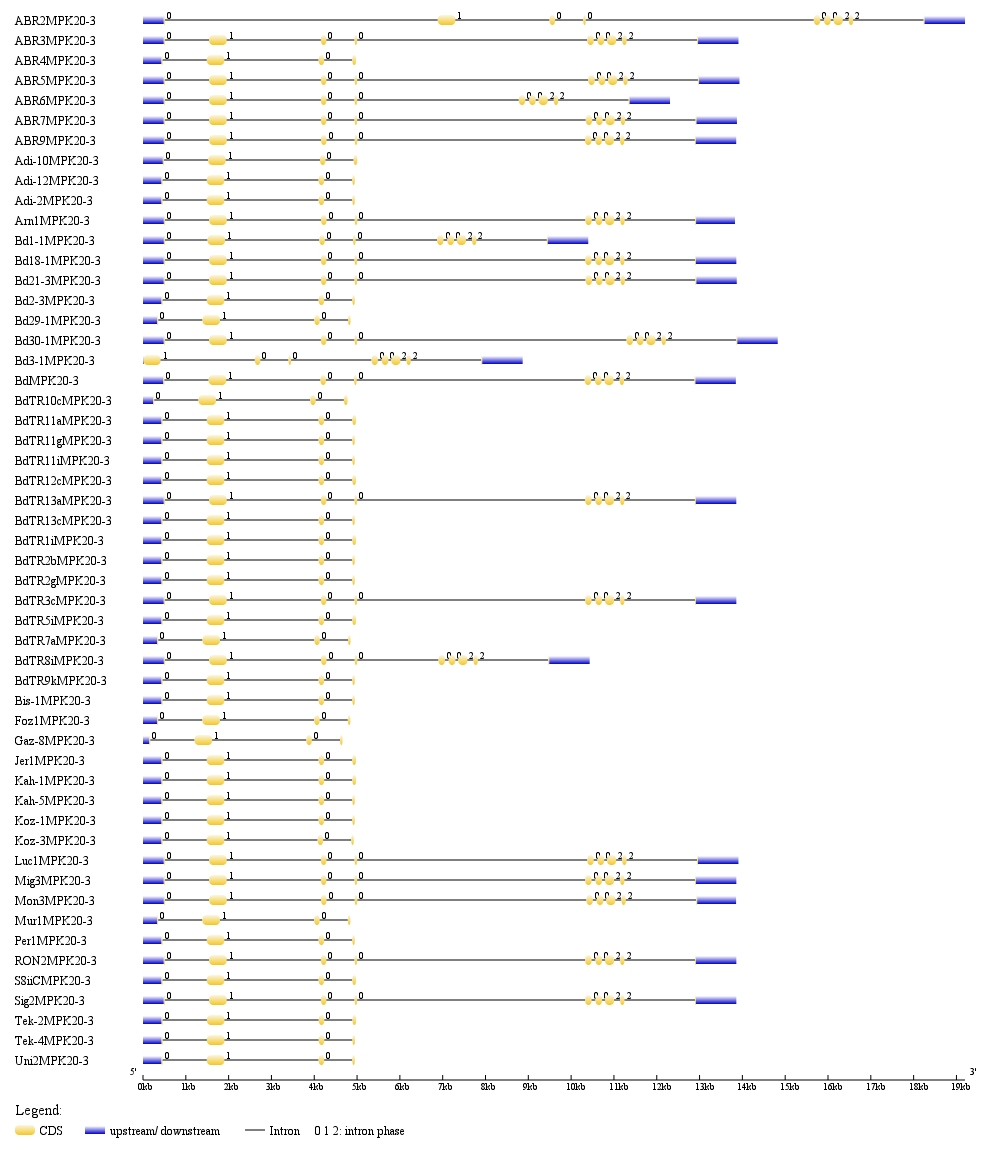


MPK20-4：


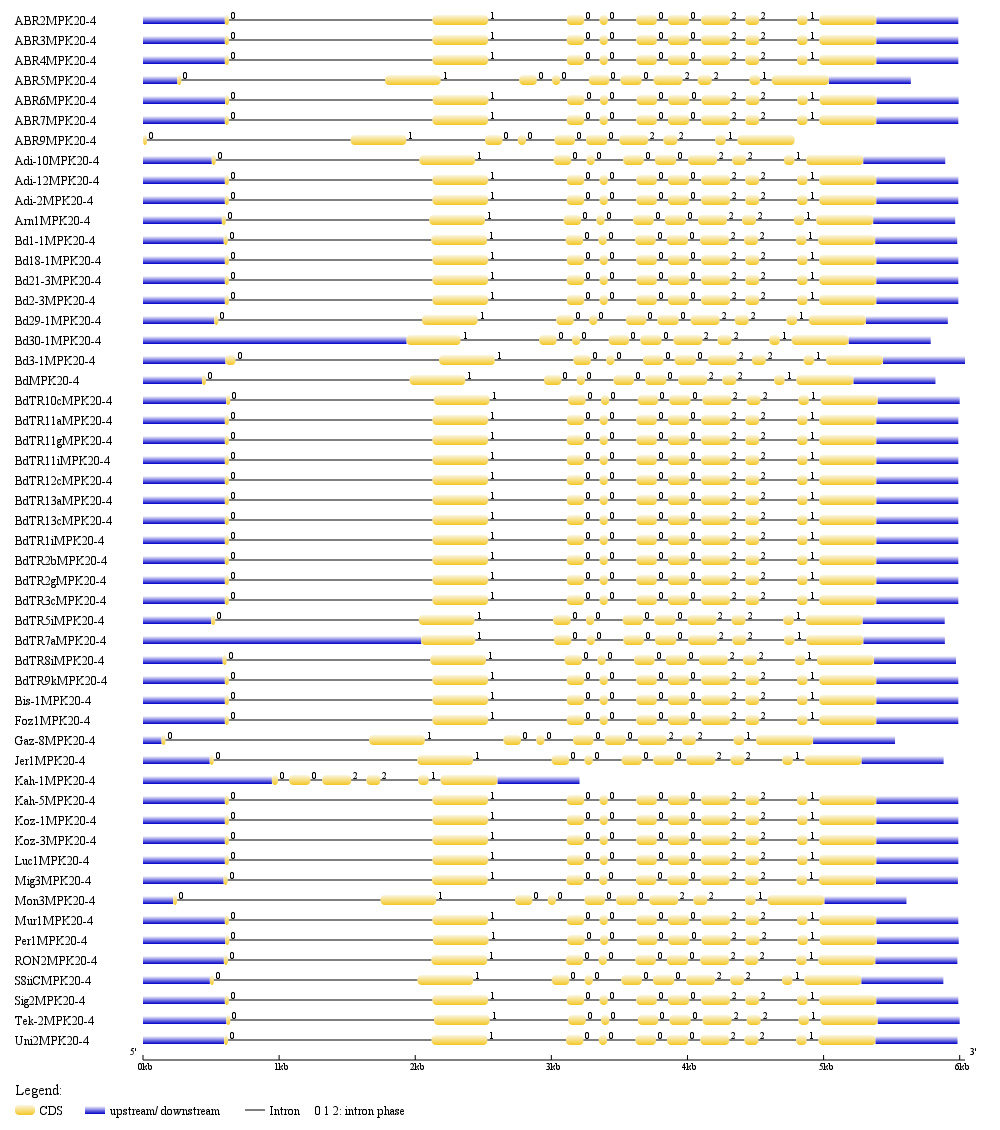


MPK20-5：


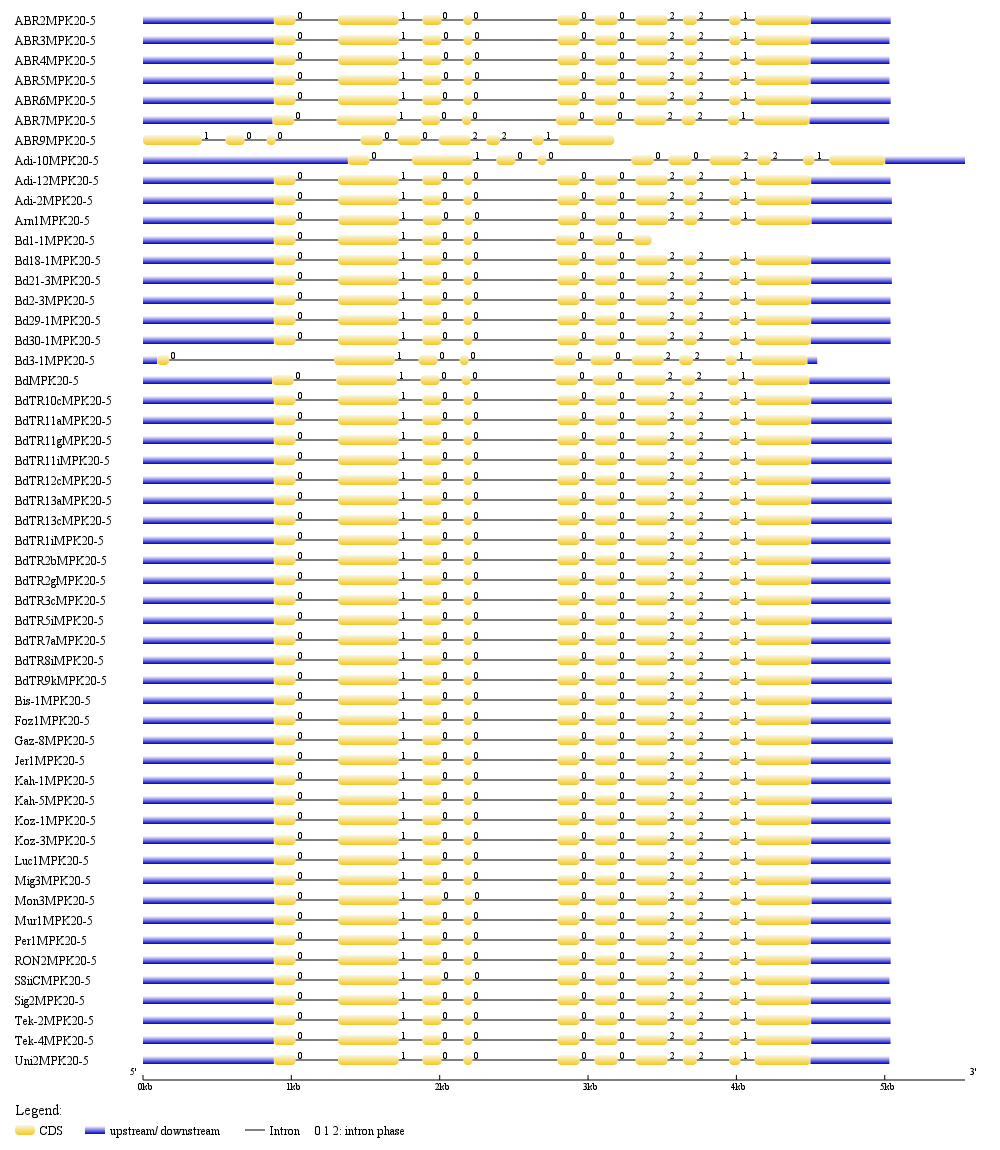


MPK21-1：


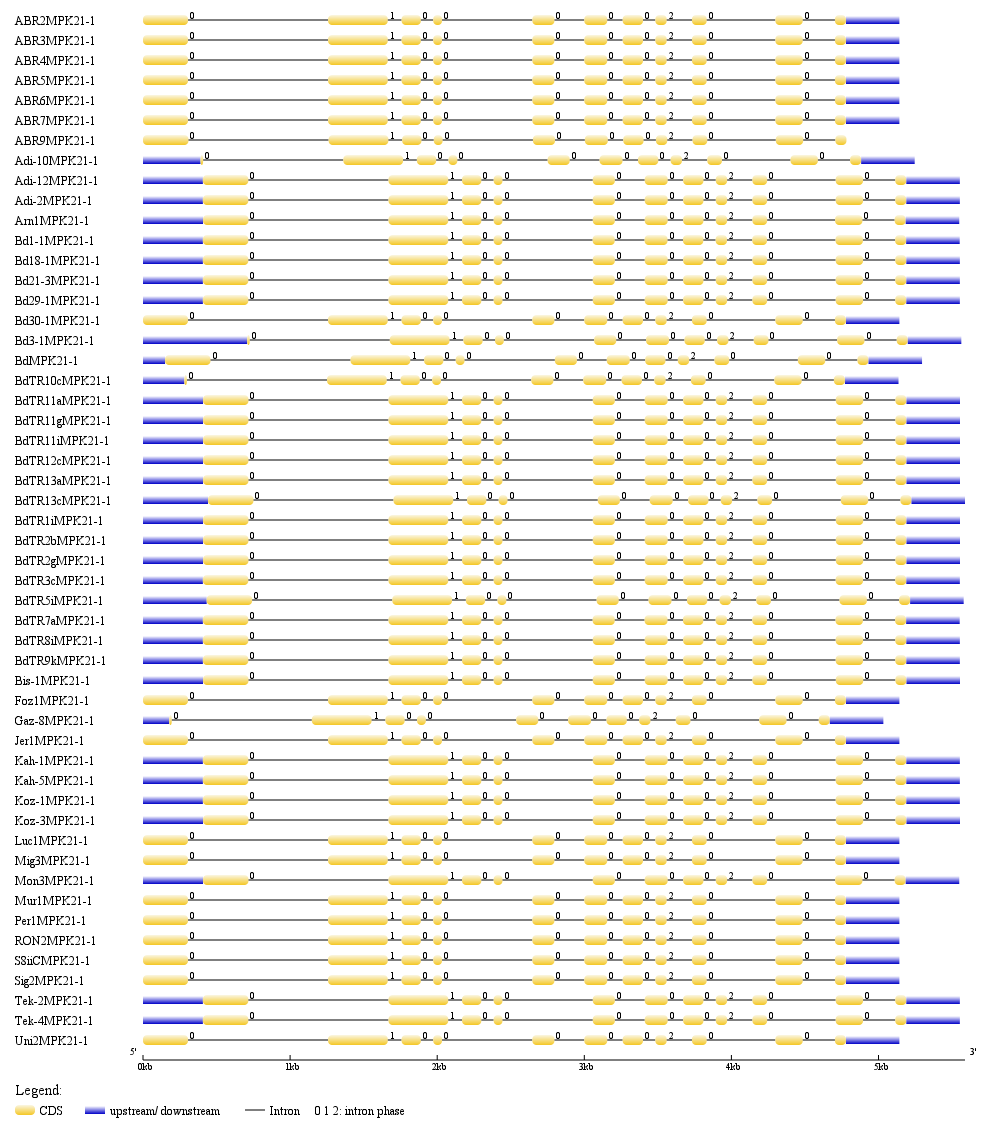

Supplement: Supplemental Information 6 [file peerj-09-11238-s006.docx]
