# Supplementary material for: Comparative analysis of MAPK and MKK gene families reveals differential evolutionary patterns in Brachypodium distachyon inbred lines
Source: PeerJ. 2021 Apr 6;9:e11238. doi: 10.7717/peerj.11238 (PMC8034371; doi:10.7717/peerj.11238)

Fig. S5. The exon/intron structures of Brachypodium inbred lines *MKK* genes.

MKK1：


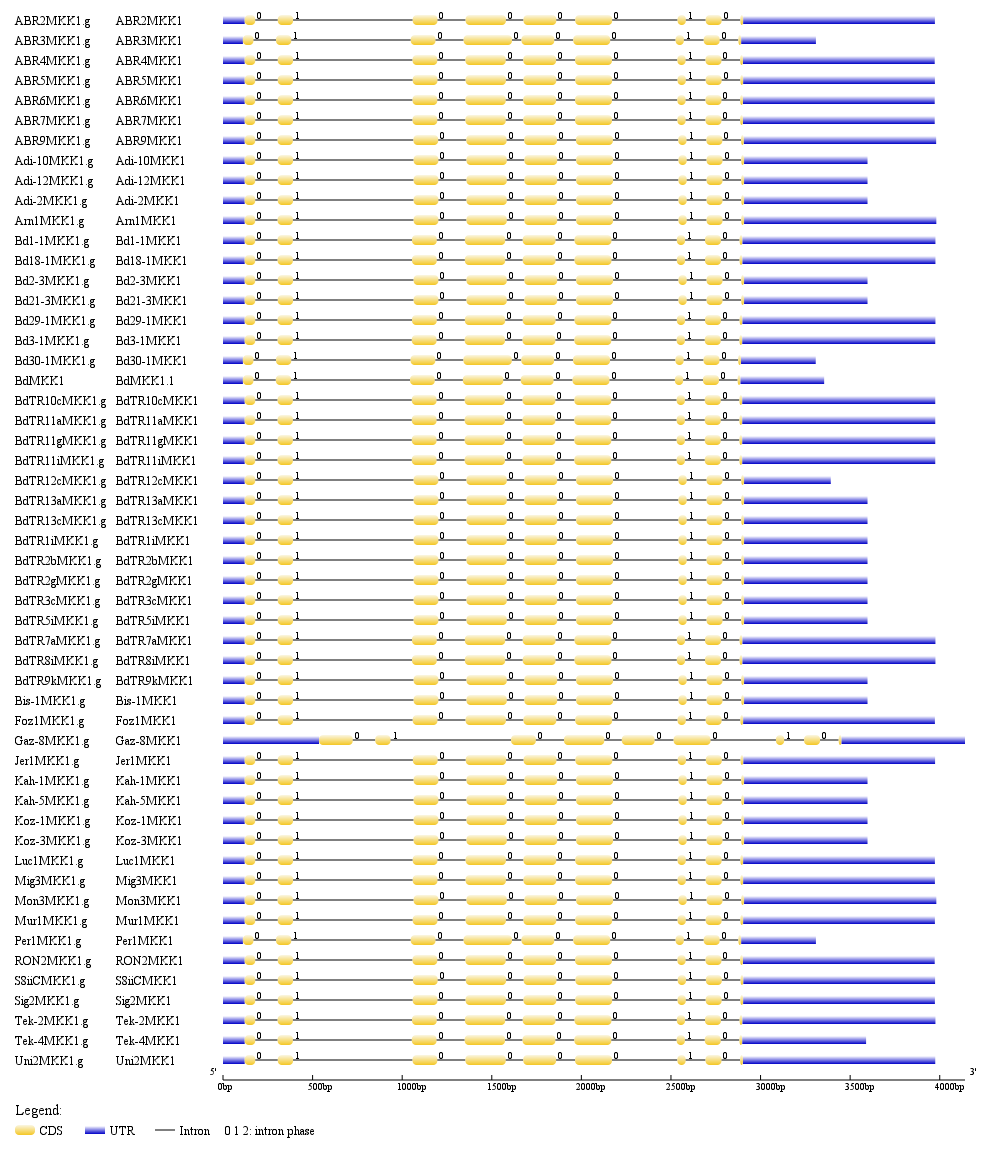


MKK3-1：


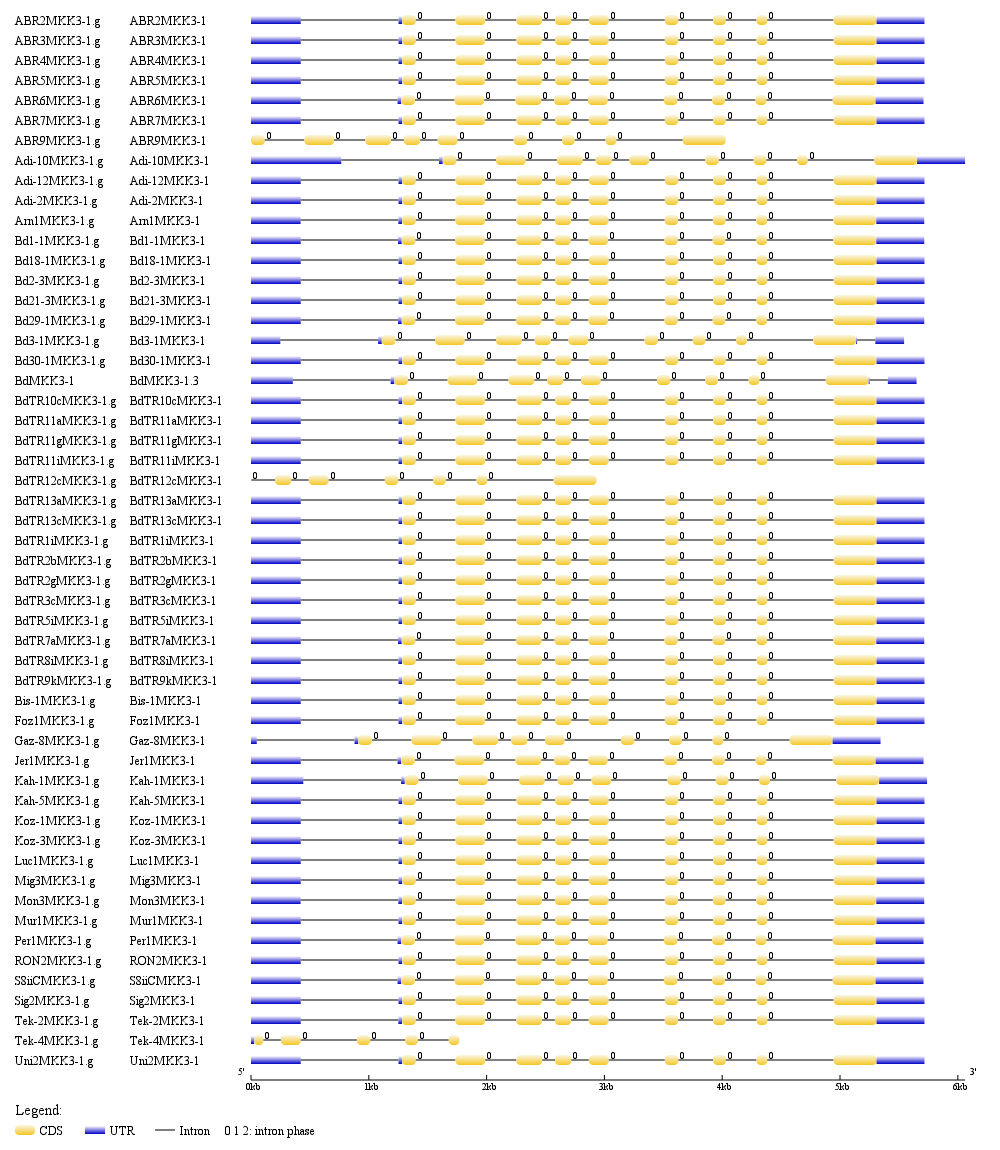


MKK3-2：


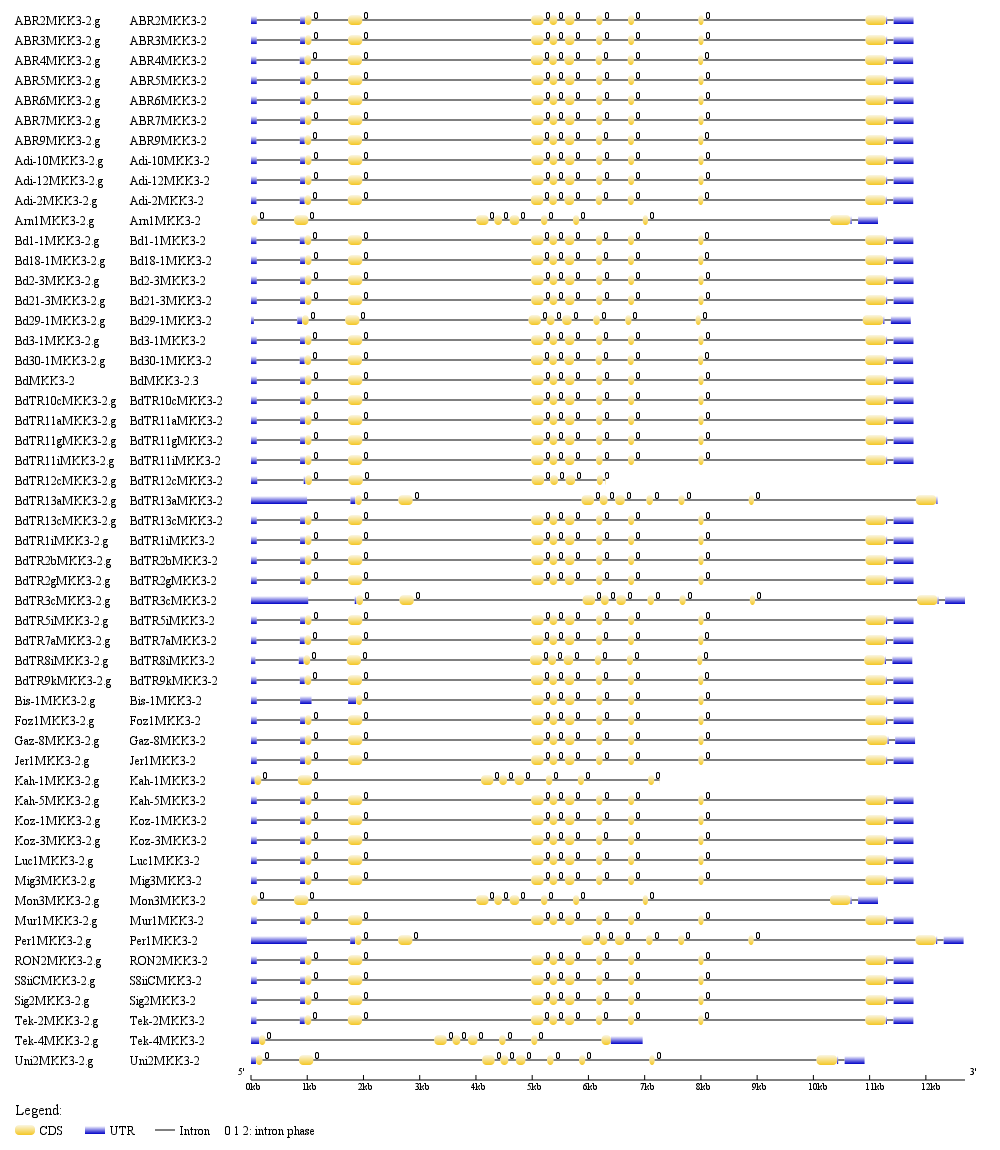


MKK3-3：


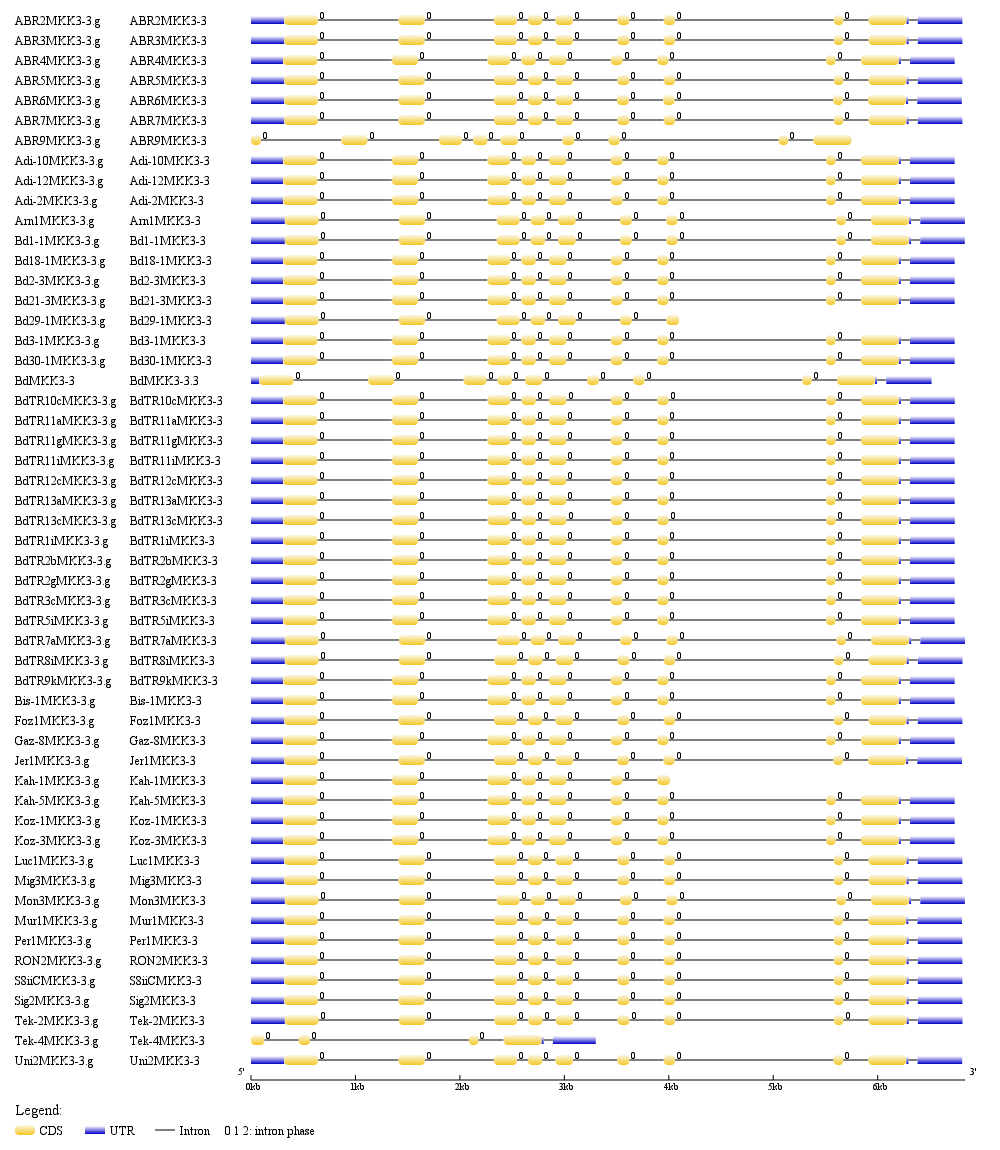


MKK4：


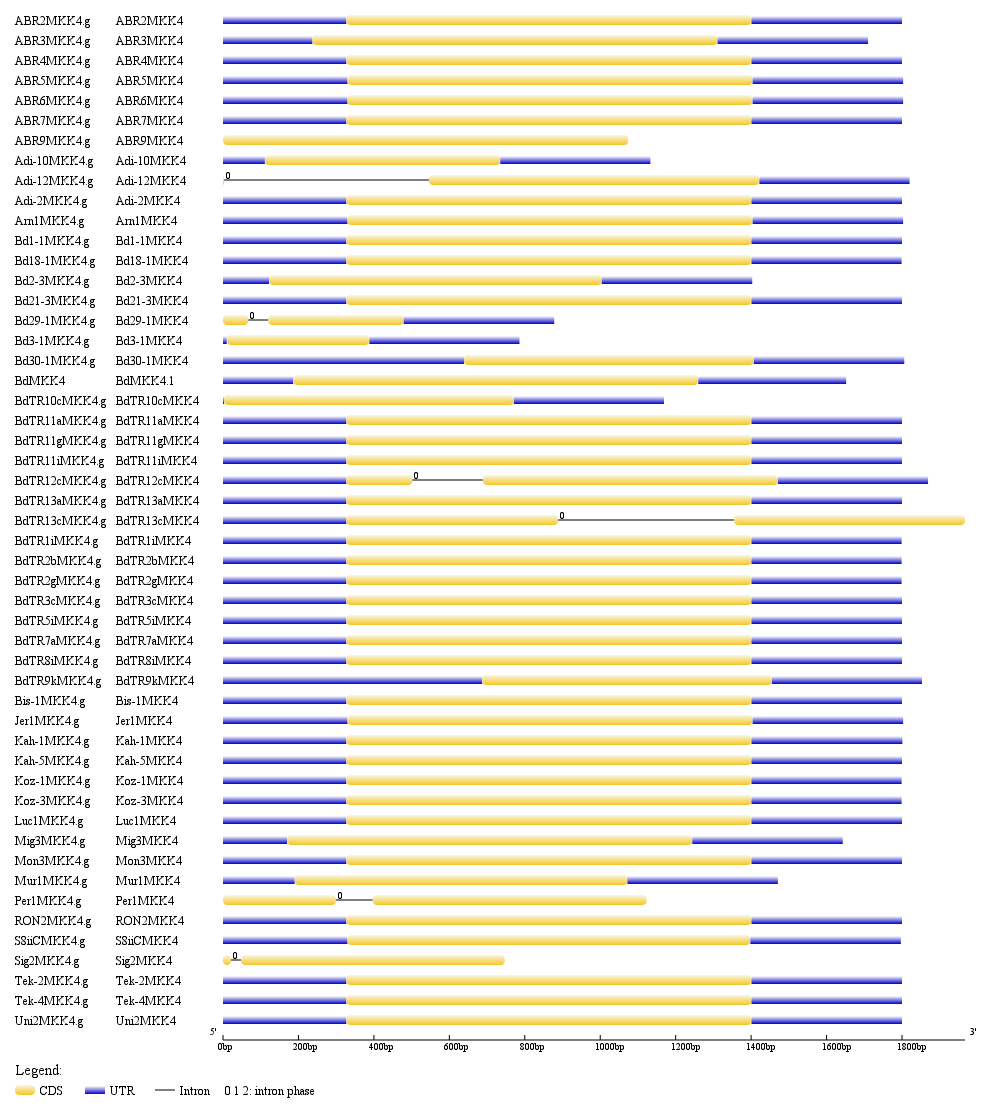


MKK5：


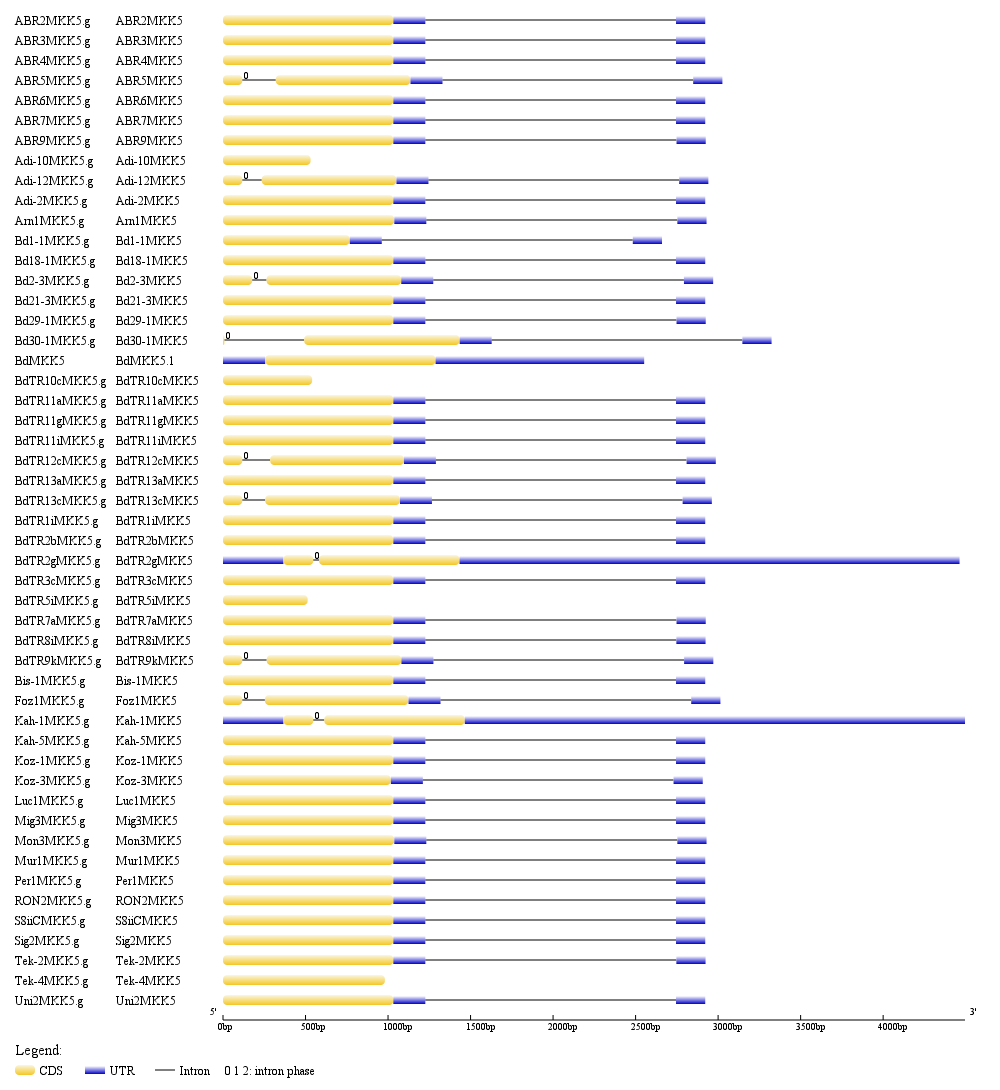


MKK6：


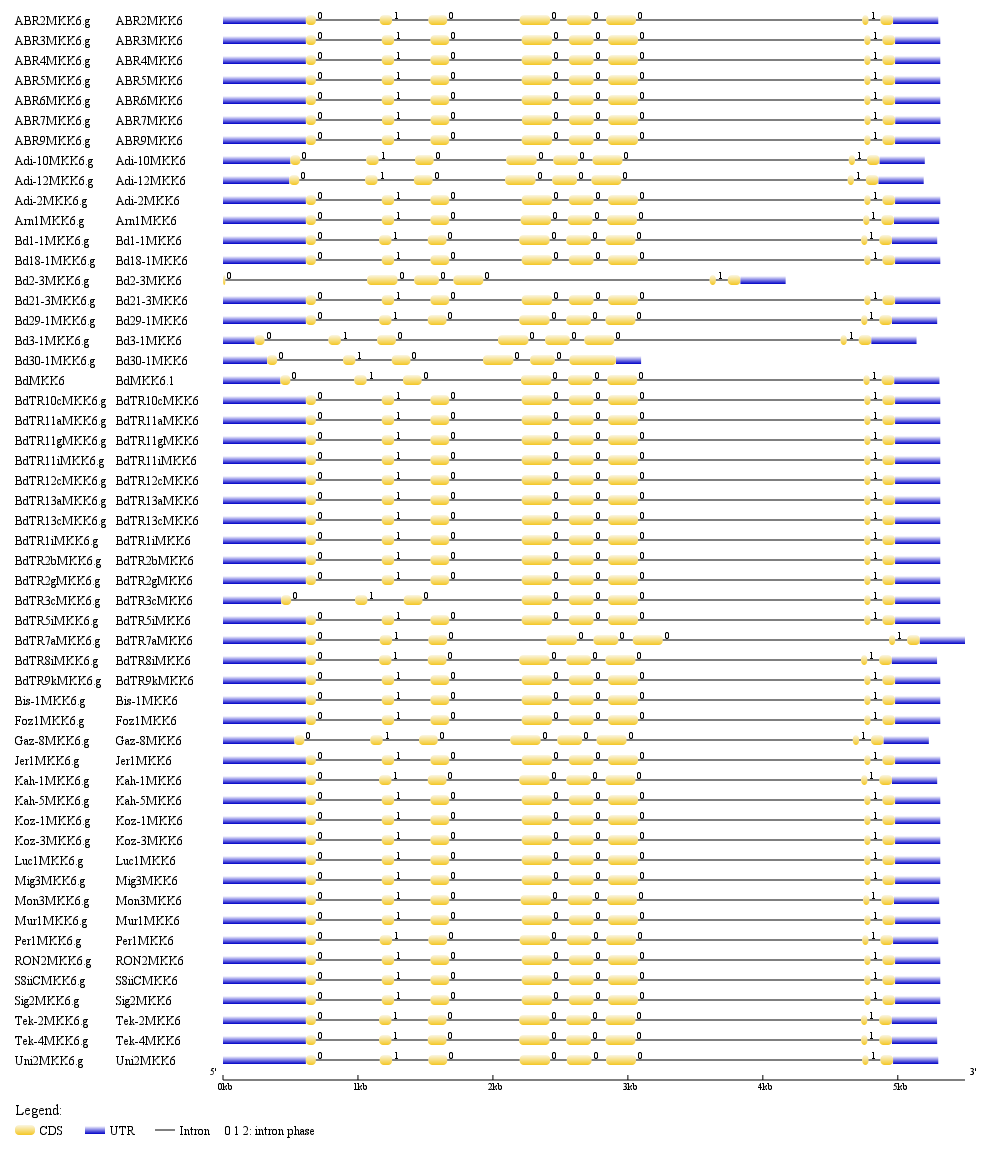


MKK10-1：


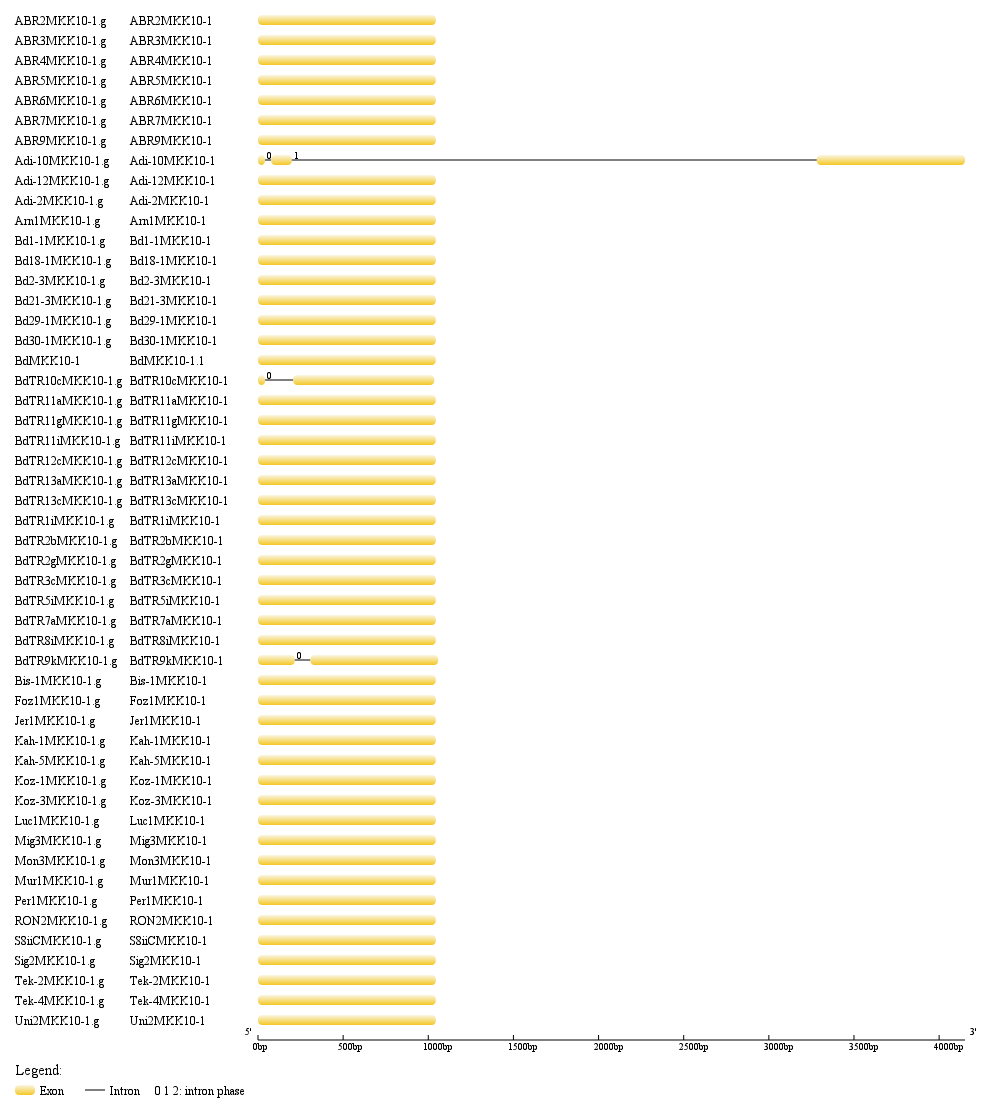


MKK10-2：


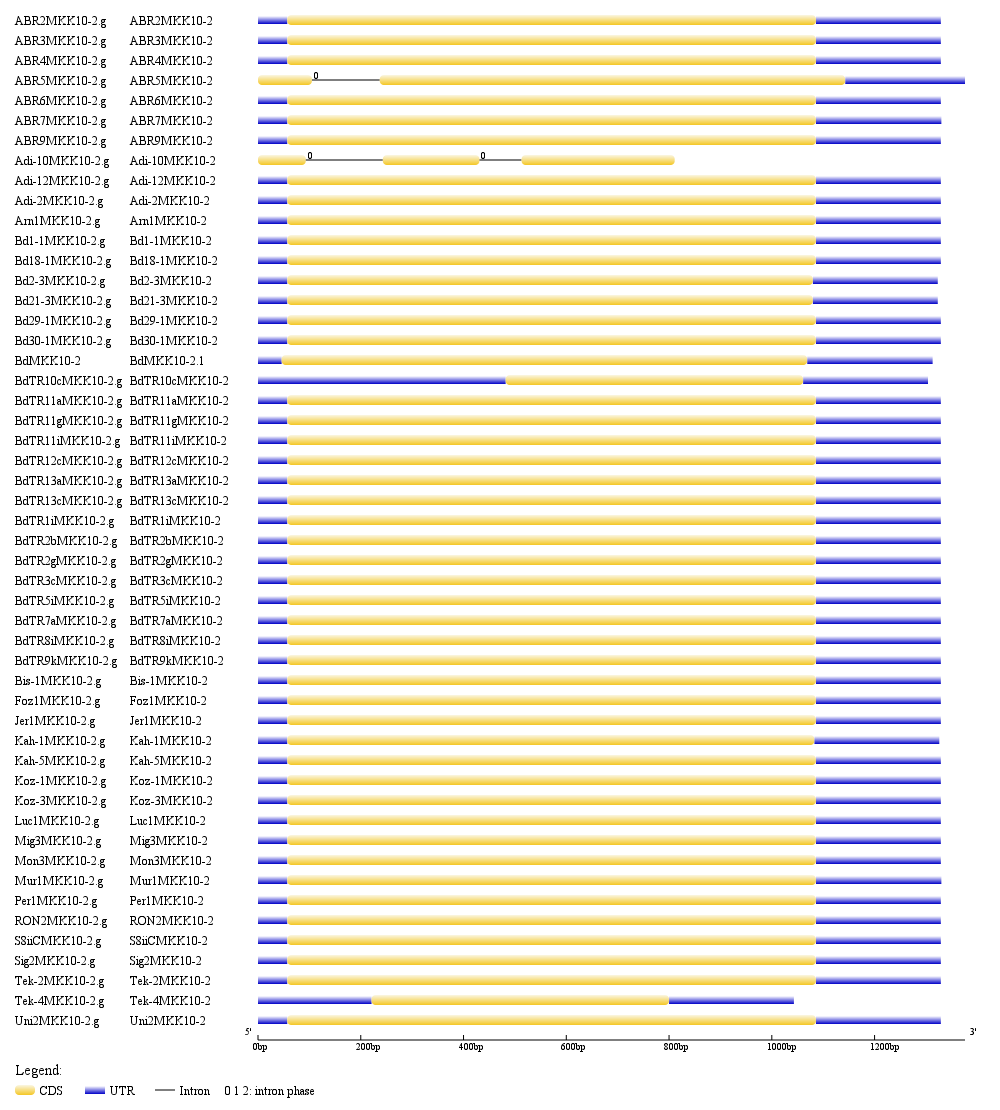


MKK10-3：


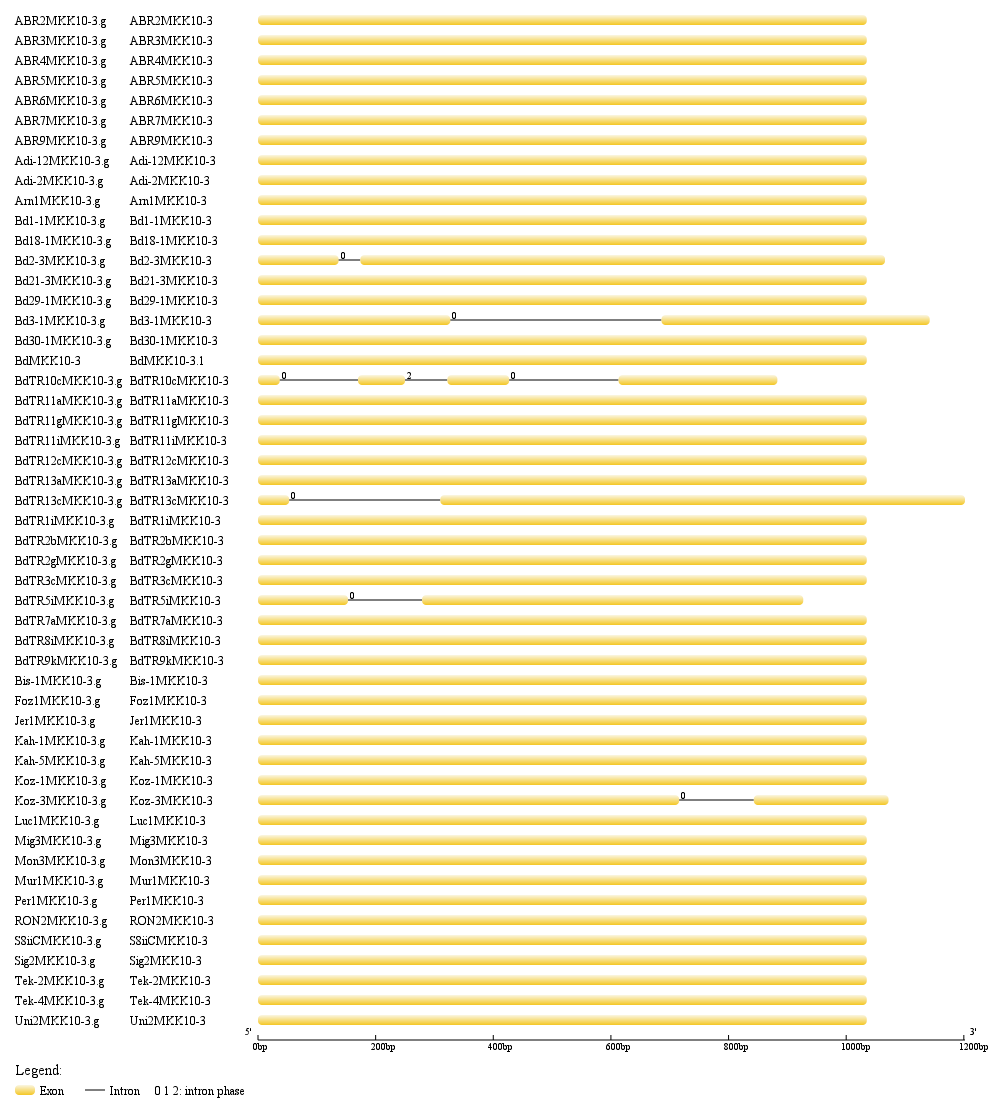


MKK10-4：


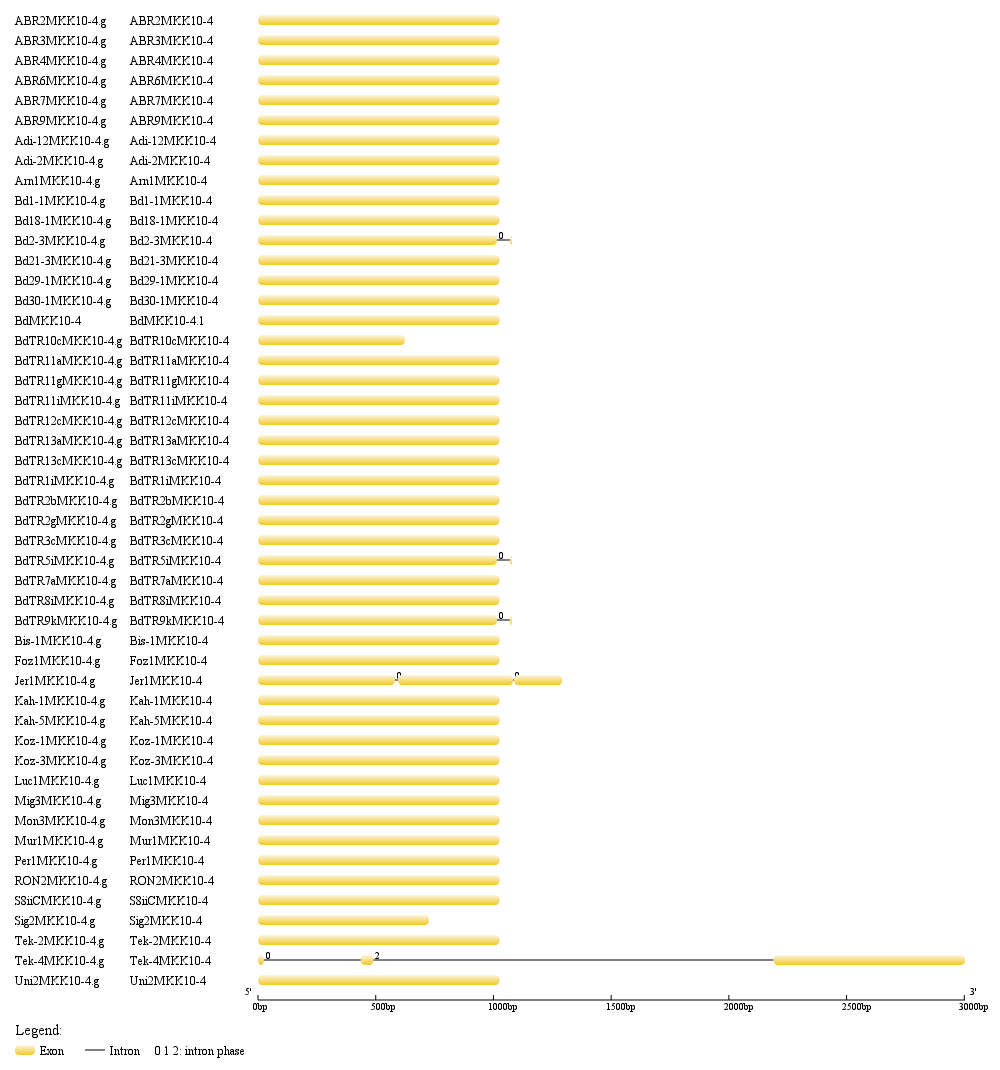


MKK10-5：


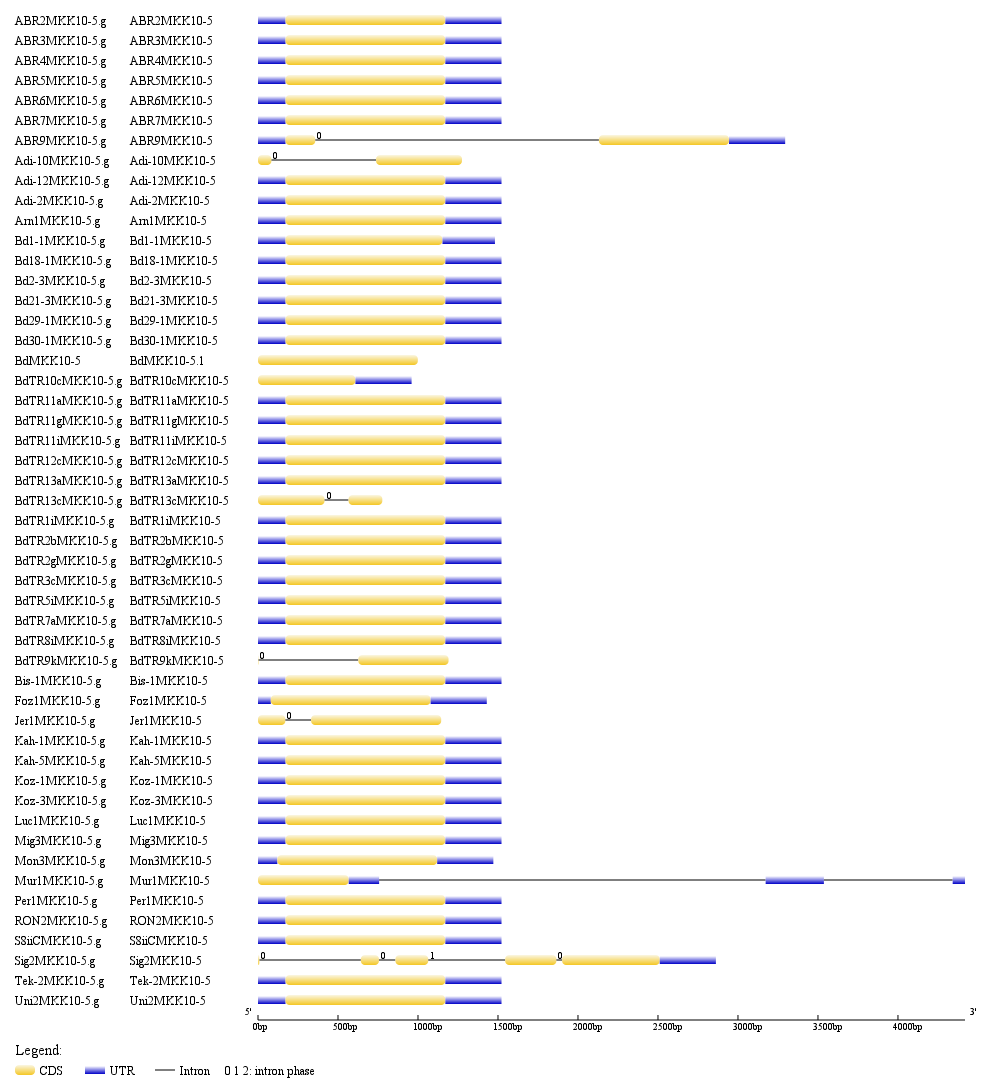

Supplement: Supplemental Information 7 [file peerj-09-11238-s007.docx]
