# Supplementary material for: Comparative analysis of MAPK and MKK gene families reveals differential evolutionary patterns in Brachypodium distachyon inbred lines
Source: PeerJ. 2021 Apr 6;9:e11238. doi: 10.7717/peerj.11238 (PMC8034371; doi:10.7717/peerj.11238)
Supplement: Supplemental Information 12 [file peerj-09-11238-s012.docx]

Table S1 Table showing nomenclatured gene name locus ID detailed genomic information *B. distachyon* inbred lines MPKs.

| Inbred Lines | Gene Name | Locus ID | Orthologous genes in Bd21 | Orientation | ORF | No. of a.a | No. of introns | 5’-3’ Coordinate |
| --- | --- | --- | --- | --- | --- | --- | --- | --- |
| EDF+ | | | | | | | | |
| Arn1 | Arn1MPK3 | Brdisv1Arn11011015m | Bradi1g65810 | + | 1110 | 369 | 4 | pseudomolecule_1:57619348-57621877 |
|  | Arn1MPK4 | Brdisv1Arn11035810m | Bradi3g32000 | + | 1134 | 377 | 7 | pseudomolecule_4:27549707-27554054 |
|  | Arn1MPK6 | Brdisv1Arn11008028m | Bradi1g49100 | + | 1176 | 391 | 5 | pseudomolecule_1: 42095232-42100896 |
|  | Arn1MPK7-1 | Brdisv1Arn11046100m | Bradi1g34030 | - | 687 | 228 | 0 | pseudomolecule_8:30677472-30678158 |
|  | Arn1MPK11 | Brdisv1Arn11033533m | Bradi3g16560 | - | 1164 | 387 | 5 | pseudomolecule_4:13060328-13063697 |
|  | Arn1MPK14 | Brdisv1Arn11031405m | Bradi3g03780 | - | 1110 | 369 | 2 | pseudomolecule_4:2370046-2374309 |
|  | Arn1MPK16 | Brdisv1Arn11018776m | Bradi2g36470 | - | 1635 | 544 | 9 | pseudomolecule_2:30043692-30050292 |
|  | Arn1MPK17 | Brdisv1Arn11005910m | Bradi1g34700 | + | 1746 | 581 | 10 | pseudomolecule_1:28582501-28587796 |
|  | Arn1MPK20-1 | Brdisv1Arn11020271m | Bradi2g44350 | - | 1848 | 615 | 9 | pseudomolecule_2:39067944-39074469 |
|  | Arn1MPK20-2 | Brdisv1Arn11015876m | Bradi2g15317 | + | 1827 | 608 | 9 | pseudomolecule_2:13071808-13077241 |
|  | Arn1MPK20-3 | Brdisv1Arn11006673m | Bradi1g41780 | + | 1266 | 421 | 8 | pseudomolecule_1:33750344-33764170 |
|  | Arn1MPK20-4 | Brdisv1Arn11020500m | Bradi2g45870 | + | 1743 | 580 | 9 | pseudomolecule_2:40481670-40487636 |
|  | Arn1MPK20-5 | Brdisv1Arn11016028m | Bradi2g16337 | + | 1812 | 603 | 9 | pseudomolecule_2:13803843-13808890 |
|  | Arn1MPK21-1 | Brdisv1Arn11015925m | Bradi2g15620 | - | 1788 | 595 | 10 | pseudomolecule_2:13288052-13293600 |
|  | Arn1MPK21-2 | Brdisv1Arn11020368m | Bradi2g45006 | - | 1233 | 410 | 7 | pseudomolecule_2:39724678-39731022 |
| Mon3 | Mon3MPK3 | Brdisv1Mon31010203m | Bradi1g65810 | + | 1572 | 524 | 4 | pseudomolecule_1:49055040..49057569 |
|  | Mon3MPK4 | Brdisv1Mon31011000m | Bradi3g32000 | - | 1134 | 377 | 7 | pseudomolecule_1:52493925..52500012 |
|  | Mon3MPK6 | Brdisv1Mon31007506m | Bradi1g49100 | + | 1152 | 383 | 5 | pseudomolecule_1:36286137..36294709 |
|  | Mon3MPK7-1 | Brdisv1Mon31044972m | Bradi1g34030 | - | 645 | 214 | 1 | pseudomolecule_8:64259660..64260799 |
|  | Mon3MPK11 | Brdisv1Mon31031562m | Bradi3g16560 | - | 1164 | 387 | 5 | pseudomolecule_4:11799544..11804261 |
|  | Mon3MPK14 | Brdisv1Mon31029522m | Bradi3g03780 | - | 1110 | 369 | 2 | pseudomolecule_4:2103863..2109832 |
|  | Mon3MPK16 | Brdisv1Mon31017564m | Bradi2g36470 | - | 1635 | 544 | 9 | pseudomolecule_2:25992974..26001477 |
|  | Mon3MPK17 | Brdisv1Mon31005542m | Bradi1g34700 | + | 1746 | 581 | 10 | pseudomolecule_1:24902370..24909739 |
|  | Mon3MPK20-1 | Brdisv1Mon31018835m | Bradi2g44350 | + | 1848 | 615 | 9 | pseudomolecule_2:32769118..32778188 |
|  | Mon3MPK20-2 | Brdisv1Mon31014790m | Bradi2g15317 | + | 1827 | 608 | 9 | pseudomolecule_2:12049323..12056901 |
|  | Mon3MPK20-3 | Brdisv1Mon31006289m | Bradi1g41780 | + | 1266 | 421 | 8 | pseudomolecule_1:29362283..29381677 |
|  | Mon3MPK20-4 | Brdisv1Mon31019053m | Bradi2g45870 | - | 1743 | 580 | 9 | pseudomolecule_2:33957396..33965248 |
|  | Mon3MPK20-5 | Brdisv1Mon31014926m | Bradi2g16337 | + | 1812 | 603 | 9 | pseudomolecule_2:12707849..12714528 |
|  | Mon3MPK21-1 | Brdisv1Mon31014824m | Bradi2g15620 | - | 1788 | 595 | 10 | pseudomolecule_2:12173898..12181666 |
|  | Mon3MPK21-2 | Brdisv1Mon31018928m | Bradi2g45006 | - | 1449 | 482 | 7 | pseudomolecule_2:33191019..33197649 |
| BdTR7a | BdTR7aMPK3 | Brdisv1BdTR7a1011021m | Bradi1g65810 | + | 1110 | 369 | 4 | pseudomolecule_1:55342868..55346409 |
|  | BdTR7aMPK4 | Brdisv1BdTR7a1035227m | Bradi3g32000 | + | 1131 | 376 | 7 | pseudomolecule_4:23774971..23781075 |
|  | BdTR7aMPK6 | Brdisv1BdTR7a1008045m | Bradi1g49100 | + | 1176 | 391 | 5 | pseudomolecule_1:40377253..40386206 |
|  | BdTR7aMPK7-1 | Brdisv1BdTR7a1005883m | Bradi1g34030 | + | 1242 | 413 | 2 | pseudomolecule_1:27537203..27541222 |
|  | BdTR7aMPK11 | Brdisv1BdTR7a1033281m | Bradi3g16560 | - | 1164 | 387 | 5 | pseudomolecule_4:12413285..12416936 |
|  | BdTR7aMPK14 | Brdisv1BdTR7a1031219m | Bradi3g03780 | - | 1110 | 369 | 2 | pseudomolecule_4:2445866..2451828 |
|  | BdTR7aMPK16 | Brdisv1BdTR7a1018519m | Bradi2g36470 | - | 1635 | 544 | 9 | pseudomolecule_2:27998541..28007040 |
|  | BdTR7aMPK17 | Brdisv1BdTR7a1006004m | Bradi1g34700 | + | 1746 | 581 | 10 | pseudomolecule_1:28146836..28154197 |
|  | BdTR7aMPK20-1 | Brdisv1BdTR7a1020019m | Bradi2g44350 | + | 1848 | 615 | 9 | pseudomolecule_2:36541419..36550489 |
|  | BdTR7aMPK20-2 | Brdisv1BdTR7a1015768m | Bradi2g15317 | + | 1827 | 608 | 9 | pseudomolecule_2:12949736..12957353 |
|  | BdTR7aMPK20-3 | Brdisv1BdTR7a1006751m | Bradi1g41780 | + | 624 | 208 | 3 | pseudomolecule_1:32680245..32687033 |
|  | BdTR7aMPK20-4 | Brdisv1BdTR7a1020237m | Bradi2g45870 | + | 1701 | 566 | 9 | pseudomolecule_2:37679189..37685383 |
|  | BdTR7aMPK20-5 | Brdisv1BdTR7a1015928m | Bradi2g16337 | + | 1812 | 603 | 9 | pseudomolecule_2:13715405..13722081 |
|  | BdTR7aMPK21-1 | Brdisv1BdTR7a1015824m | Bradi2g15620 | - | 1788 | 595 | 10 | pseudomolecule_2:13173018..13180789 |
|  | BdTR7aMPK21-2 | Brdisv1BdTR7a1020100m | Bradi2g45006 | - | 1449 | 482 | 7 | pseudomolecule_2:36957919..36964548 |
| BdTR8i | BdTR8iMPK3 | Brdisv1BdTR8i1010712m | Bradi1g65810 | + | 1110 | 369 | 4 | pseudomolecule_1:55574941..55578482 |
|  | BdTR8iMPK4 | Brdisv1BdTR8i1027174m | Bradi3g32000 | + | 1143 | 380 | 7 | pseudomolecule_3:25152497..25158619 |
|  | BdTR8iMPK6 | Brdisv1BdTR8i1007684m | Bradi1g49100 | + | 1176 | 391 | 5 | pseudomolecule_1:39869569..39878519 |
|  | BdTR8iMPK7-1 | Brdisv1BdTR8i1005547m | Bradi1g34030 | + | 1242 | 413 | 2 | pseudomolecule_1:26734910..26738935 |
|  | BdTR8iMPK11 | Brdisv1BdTR8i1025152m | Bradi3g16560 | - | 1164 | 387 | 5 | pseudomolecule_3:12622182..12626894 |
|  | BdTR8iMPK14 | Brdisv1BdTR8i1023107m | Bradi3g03780 | - | 1110 | 369 | 2 | pseudomolecule_3:2406713..2412676 |
|  | BdTR8iMPK16 | Brdisv1BdTR8i1018031m | Bradi2g36470 | - | 1635 | 544 | 9 | pseudomolecule_2:27715767..27724276 |
|  | BdTR8iMPK17 | Brdisv1BdTR8i1005684m | Bradi1g34700 | + | 1746 | 581 | 10 | pseudomolecule_1:27498121..27505482 |
|  | BdTR8iMPK20-1 | Brdisv1BdTR8i1019342m | Bradi2g44350 | - | 1848 | 615 | 9 | pseudomolecule_2:34710712..34719782 |
|  | BdTR8iMPK20-2 | Brdisv1BdTR8i1015298m | Bradi2g15317 | + | 1827 | 608 | 9 | pseudomolecule_2:12391412..12398873 |
|  | BdTR8iMPK20-3 | Brdisv1BdTR8i1006471m | Bradi1g41780 | + | 1266 | 421 | 8 | pseudomolecule_1:32589560..32604166 |
|  | BdTR8iMPK20-4 | Brdisv1BdTR8i1019564m | Bradi2g45870 | + | 1743 | 580 | 9 | pseudomolecule_2:36003514..36011876 |
|  | BdTR8iMPK20-5 | Brdisv1BdTR8i1015450m | Bradi2g16337 | + | 1812 | 603 | 9 | pseudomolecule_2:13106197..13112873 |
|  | BdTR8iMPK21-1 | Brdisv1BdTR8i1015345m | Bradi2g15620 | - | 1788 | 595 | 10 | pseudomolecule_2:12574452..12582226 |
|  | BdTR8iMPK21-2 | Brdisv1BdTR8i1019424m | Bradi2g45006 | - | 1149 | 482 | 7 | pseudomolecule_2:35161328..35167957 |
| Tek-2 | Tek-2MPK3 | Brdisv1Tek-21010027m | Bradi1g65810 | + | 1110 | 369 | 4 | pseudomolecule_1:43412456..43415997 |
|  | Tek-2MPK4 | Brdisv1Tek-21010816m | Bradi3g32000 | - | 1143 | 380 | 7 | pseudomolecule_1:46731059..46737181 |
|  | Tek-2MPK6 | Brdisv1Tek-21007328m | Bradi1g49100 | + | 1176 | 391 | 5 | pseudomolecule_1:31662592..31671539 |
|  | Tek-2MPK7-1 | Brdisv1Tek-21005308m | Bradi1g34030 | + | 1242 | 413 | 2 | pseudomolecule_1:22212657..22216682 |
|  | Tek-2MPK11 | Brdisv1Tek-21030943m | Bradi3g16560 | - | 1164 | 387 | 5 | pseudomolecule_4:11063901..11068613 |
|  | Tek-2MPK14 | Brdisv1Tek-21028844m | Bradi3g03780 | - | 1110 | 369 | 2 | pseudomolecule_4:2270600..2276563 |
|  | Tek-2MPK16 | Brdisv1Tek-21017203m | Bradi2g36470 | - | 1635 | 544 | 9 | pseudomolecule_2:21608902..21617411 |
|  | Tek-2MPK17 | Brdisv1Tek-21005426m | Bradi1g34700 | + | 1746 | 581 | 10 | pseudomolecule_1:22739780..22747200 |
|  | Tek-2MPK20-1 | Brdisv1Tek-21018480m | Bradi2g44350 | - | 1848 | 615 | 9 | pseudomolecule_2:27442614..27451684 |
|  | Tek-2MPK20-2 | Brdisv1Tek-21043384m | Bradi2g15317 | + | 1827 | 608 | 9 | pseudomolecule_7:19991157..19998618 |
|  | Tek-2MPK20-3 | Brdisv1Tek-21043278m | Bradi1g41780 | - | 651 | 216 | 3 | pseudomolecule_7:18850093..18857064 |
|  | Tek-2MPK20-4 | Brdisv1Tek-21018691m | Bradi2g45870 | + | 1743 | 580 | 9 | pseudomolecule_2:28438902..28447301 |
|  | Tek-2MPK20-5 | Brdisv1Tek-21014805m | Bradi2g16337 | + | 1812 | 603 | 9 | pseudomolecule_2:11355962..11362638 |
|  | Tek-2MPK21-1 | Brdisv1Tek-21014698m | Bradi2g15620 | - | 1788 | 595 | 10 | pseudomolecule_2:10913070..10920844 |
|  | Tek-2MPK21-2 | Brdisv1Tek-21018561m | Bradi2g45006 | - | 1149 | 482 | 7 | pseudomolecule_2:27789378..27796007 |
| Tek-4 | Tek-4MPK3 | Brdisv1Tek-41030977m | Bradi1g65810 | + | 936 | 312 | 3 | pseudomolecule_8:41813551..41815455 |
|  | Tek-4MPK4 | Brdisv1Tek-41029583m | Bradi3g32000 | + | 1143 | 380 | 5 | pseudomolecule_8:34282549..34287072 |
|  | Tek-4MPK6 | Brdisv1Tek-41035605m | Bradi1g49100 | - | 810 | 269 | 3 | pseudomolecule_8:67096582..67100480 |
|  | Tek-4MPK7-1 | Brdisv1Tek-41014125m | Bradi1g34030 | + | 1119 | 372 | 1 | pseudomolecule_3:7396306..7399021 |
|  | Tek-4MPK11 | Brdisv1Tek-41017616m | Bradi3g16560 | - | 1164 | 387 | 5 | pseudomolecule_4:5309905..5314617 |
|  | Tek-4MPK14 | Brdisv1Tek-41016381m | Bradi3g03780 | - | 1110 | 369 | 2 | pseudomolecule_4:1101634..1107597 |
|  | Tek-4MPK16 | Brdisv1Tek-41034938m | Bradi2g36470 | - | 552 | 183 | 2 | pseudomolecule_8:63284225..63286623 |
|  | Tek-4MPK17 | Brdisv1Tek-41003047m | Bradi1g34700 | + | 1746 | 581 | 11 | pseudomolecule_1:10069634..10084942 |
|  | Tek-4MPK20-1 | Brdisv1Tek-41029227m | Bradi2g44350 | + | 627 | 208 | 4 | pseudomolecule_8:32456805..32460759 |
|  | Tek-4MPK20-2 | Brdisv1Tek-41027315m | Bradi2g15317 | - | 1827 | 608 | 9 | pseudomolecule_8:22261266..22268354 |
|  | Tek-4MPK20-3 | Brdisv1Tek-41027738m | Bradi1g41780 | + | 624 | 208 | 3 | pseudomolecule_8:24493554..24500488 |
|  | Tek-4MPK20-5 | Brdisv1Tek-41008316m | Bradi2g16337 | + | 1812 | 603 | 9 | pseudomolecule_2:5517487..5524163 |
|  | Tek-4MPK21-1 | Brdisv1Tek-41008225m | Bradi2g15620 | - | 1788 | 595 | 10 | pseudomolecule_2:5255903..5263677 |
|  | Tek-4MPK21-2 | Brdisv1Tek-41031014m | Bradi2g45006 | - | 846 | 281 | 5 | pseudomolecule_8:42051868..42056440 |
| Bd29-1 | Bd29-1MPK3 | Brdisv1Bd29-11009003m | Bradi2g16337 | + | 1110 | 369 | 4 | pseudomolecule_1:38455438..38458979 |
|  | Bd29-1MPK4 | Brdisv1Bd29-11009699m | Bradi2g15620 | - | 1131 | 376 | 7 | pseudomolecule_1:41304278..41310400 |
|  | Bd29-1MPK6 | Brdisv1Bd29-11006575m | Bradi2g45006 | + | 1176 | 391 | 5 | pseudomolecule_1:28134913..28143863 |
|  | Bd29-1MPK7-1 | Brdisv1Bd29-11022801m | Bradi2g16337 | + | 1242 | 413 | 2 | pseudomolecule_3:15076390..15081067 |
|  | Bd29-1MPK11 | Brdisv1Bd29-11027994m | Bradi2g15620 | - | 1164 | 387 | 5 | pseudomolecule_4:9666102..9670818 |
|  | Bd29-1MPK14 | Brdisv1Bd29-11039982m | Bradi2g45006 | - | 1110 | 369 | 1 | pseudomolecule_8:27033227..27037211 |
|  | Bd29-1MPK16 | Brdisv1Bd29-11015609m | Bradi2g16337 | - | 1635 | 544 | 9 | pseudomolecule_2:20215341..20223843 |
|  | Bd29-1MPK17 | Brdisv1Bd29-11004857m | Bradi2g15620 | + | 1746 | 581 | 10 | pseudomolecule_1:19816626..19823987 |
|  | Bd29-1MPK20-1 | Brdisv1Bd29-11016791m | Bradi2g45006 | + | 1848 | 615 | 9 | pseudomolecule_2:25531399..25540469 |
|  | Bd29-1MPK20-2 | Brdisv1Bd29-11013150m | Bradi2g16337 | + | 1827 | 608 | 9 | pseudomolecule_2:9624587..9631754 |
|  | Bd29-1MPK20-3 | Brdisv1Bd29-11005433m | Bradi2g15620 | + | 624 | 208 | 3 | pseudomolecule_1:22601747..22608535 |
|  | Bd29-1MPK20-4 | Brdisv1Bd29-11016979m | Bradi2g45006 | + | 1743 | 580 | 9 | pseudomolecule_2:26370985..26379263 |
|  | Bd29-1MPK20-5 | Brdisv1Bd29-11013304m | Bradi2g16337 | + | 1812 | 603 | 9 | pseudomolecule_2:10234687..10241363 |
|  | Bd29-1MPK21-1 | Brdisv1Bd29-11013190m | Bradi2g15620 | - | 1788 | 595 | 10 | pseudomolecule_2:9767609..9775383 |
|  | Bd29-1MPK21-2 | Brdisv1Bd29-11016866m | Bradi2g45006 | - | 1449 | 482 | 7 | pseudomolecule_2:25810884..25817513 |
| ABR9 | ABR9MPK3 | Brdisv1ABR9_r1011010m | Bradi2g16337 | + | 1110 | 369 | 4 | pseudomolecule_1:58534758..58538299 |
|  | ABR9MPK4 | Brdisv1ABR9_r1026988m | Bradi2g15620 | + | 1131 | 376 | 5 | pseudomolecule_4:27363420..27367632 |
|  | ABR9MPK6 | Brdisv1ABR9_r1007931m | Bradi2g45006 | + | 1176 | 391 | 5 | pseudomolecule_1:42255050..42264000 |
|  | ABR9MPK7-1 | Brdisv1ABR9_r1035091m | Bradi2g16337 | - | 1119 | 372 | 1 | pseudomolecule_7:32552093..32554192 |
|  | ABR9MPK11 | Brdisv1ABR9_r1025789m | Bradi2g15620 | - | 1164 | 387 | 5 | pseudomolecule_4:13636588..13640242 |
|  | ABR9MPK14 | Brdisv1ABR9_r1024459m | Bradi2g45006 | - | 1110 | 369 | 1 | pseudomolecule_4:2553318..2556747 |
|  | ABR9MPK16 | Brdisv1ABR9_r1016603m | Bradi2g16337 | - | 1635 | 544 | 8 | pseudomolecule_2:31053209..31059131 |
|  | ABR9MPK17 | Brdisv1ABR9_r1005736m | Bradi2g15620 | + | 1746 | 581 | 10 | pseudomolecule_1:27606300..27613661 |
|  | ABR9MPK20-1 | Brdisv1ABR9_r1017513m | Bradi2g45006 | - | 1848 | 615 | 9 | pseudomolecule_2:40206601..40214393 |
|  | ABR9MPK20-2 | Brdisv1ABR9_r1014888m | Bradi2g16337 | + | 1827 | 608 | 9 | pseudomolecule_2:13963550..13969976 |
|  | ABR9MPK20-3 | Brdisv1ABR9_r1006567m | Bradi2g15620 | + | 1266 | 421 | 8 | pseudomolecule_1:33597630..33617027 |
|  | ABR9MPK20-4 | Brdisv1ABR9_r1017678m | Bradi2g45006 | + | 1743 | 580 | 9 | pseudomolecule_2:41650318..41657018 |
|  | ABR9MPK20-5 | Brdisv1ABR9_r1015005m | Bradi2g16337 | + | 1653 | 550 | 9 | pseudomolecule_2:14678875..14683321 |
|  | ABR9MPK21-1 | Brdisv1ABR9_r1014923m | Bradi2g15620 | - | 1788 | 595 | 10 | pseudomolecule_2:14147809..14154502 |
|  | ABR9MPK21-2 | Brdisv1ABR9_r1017558m | Bradi2g45006 | - | 1230 | 409 | 8 | pseudomolecule_2:40679785..40688731 |
| Bd1-1 | Bd1-1MPK3 | Brdisv1Bd1-11009760m | Bradi1g65810 | + | 1110 | 369 | 4 | pseudomolecule_1:44867005..44870546 |
|  | Bd1-1MPK4 | Brdisv1Bd1-11024723m | Bradi3g32000 | + | 1143 | 380 | 7 | pseudomolecule_3:18607349..1861347 |
|  | Bd1-1MPK6 | Brdisv1Bd1-11006994m | Bradi1g49100 | + | 1176 | 391 | 5 | pseudomolecule_1:32108331..32117281 |
|  | Bd1-1MPK11 | Brdisv1Bd1-11023075m | Bradi3g16560 | - | 1164 | 387 | 5 | pseudomolecule_3:10575922..10580634 |
|  | Bd1-1MPK14 | Brdisv1Bd1-11021222m | Bradi3g03780 | - | 1110 | 369 | 2 | pseudomolecule_3:2264970..2270932 |
|  | Bd1-1MPK16 | Brdisv1Bd1-11016431m | Bradi2g36470 | - | 1635 | 544 | 9 | pseudomolecule_2:21493583..21502080 |
|  | Bd1-1MPK17 | Brdisv1Bd1-11005322m | Bradi1g34700 | + | 1746 | 581 | 10 | pseudomolecule_1:23731715..23739076 |
|  | Bd1-1MPK20-1 | Brdisv1Bd1-11017639m | Bradi2g44350 | - | 1848 | 615 | 9 | pseudomolecule_2:27199596..27208666 |
|  | Bd1-1MPK20-2 | Brdisv1Bd1-11014003m | Bradi2g15317 | + | 1827 | 608 | 9 | pseudomolecule_2:10186990..10193644 |
|  | Bd1-1MPK20-3 | Brdisv1Bd1-11005908m | Bradi1g41780 | + | 1266 | 421 | 8 | pseudomolecule_1:26884656..26899215 |
|  | Bd1-1MPK20-4 | Brdisv1Bd1-11017837m | Bradi2g45870 | + | 1743 | 580 | 9 | pseudomolecule_2:28289286..28297659 |
|  | Bd1-1MPK20-5 | Brdisv1Bd1-11014155m | Bradi2g16337 | + | 1170 | 389 | 6 | pseudomolecule_2:10857964..10862386 |
|  | Bd1-1MPK21-1 | Brdisv1Bd1-11014051m | Bradi2g15620 | - | 1788 | 595 | 10 | pseudomolecule_2:10366073..10373844 |
|  | Bd1-1MPK21-2 | Brdisv1Bd1-11017707m | Bradi2g45006 | - | 1449 | 482 | 7 | pseudomolecule_2:27550877..27557506 |
| T+ | | | | | | | | |
| Bd21 | BdMPK3 | Bradi1g65810 | - | + | 1110 | 369 | 4 | 64739946 - 64742370 |
|  | BdMPK4 | Bradi3g32000 | - | + | 1128 | 375 | 6 | 34209148 - 34213422 |
|  | BdMPK6 | Bradi1g49100 | - | + | 1176 | 391 | 5 | 47836748 - 47843578 |
|  | BdMPK7-1 | Bradi1g34030 | - | + | 1242 | 413 | 2 | 29632402 - 29634667 |
|  | BdMPK7-2 | Bradi4g24914 | - | + | 5127 | 1708 | 11 | 29995835-30004649 |
|  | BdMPK11 | Bradi3g16560 | - | - | 1164 | 387 | 5 | 14742468 - 14745425 |
|  | BdMPK14 | Bradi3g03780 | - | - | 1110 | 369 | 2 | 2521821 - 2525826 |
|  | BdMPK16 | Bradi2g36470 | - | - | 1668 | 555 | 9 | 36786589 - 36792652 |
|  | BdMPK17 | Bradi1g34700 | - | + | 1746 | 581 | 10 | 30307750 - 30312635 |
|  | BdMPK20-1 | Bradi2g44350 | - | - | 1848 | 615 | 9 | 44852084 - 44858179 |
|  | BdMPK20-2 | Bradi2g15317 | - | + | 1794 | 597 | 9 | 13689255 - 13693771 |
|  | BdMPK20-3 | Bradi1g41780 | - | + | 1266 | 421 | 8 | 38419711 - 38433437 |
|  | BdMPK20-4 | Bradi2g45870 | - | + | 1743 | 580 | 9 | 46302880 - 46308387 |
|  | BdMPK20-5 | Bradi2g16337 | - | + | 1746 | 581 | 9 | 14412074 - 14417497 |
|  | BdMPK21-1 | Bradi2g15620 | - | - | 1788 | 595 | 10 | 13875717 - 13880863 |
|  | BdMPK21-2 | Bradi2g45006 | - | - | 1173 | 390 | 8 | 45423424..45432920 |
| Bd21Ref | Bd21RefMPK3 | Brdisv20Bd21Ref1011713m | Bradi1g65810 | + | 1110 | 369 | 4 | Bd1:65016973..65020514 |
|  | Bd21RefMPK4 | Brdisv20Bd21Ref1030338m | Bradi3g32000 | + | 1128 | 375 | 7 | Bd3:34251902..34258003 |
|  | Bd21RefMPK6 | Brdisv20Bd21Ref1008702m | Bradi1g49100 | + | 1176 | 391 | 5 | Bd1:48075752..48084720 |
|  | Bd21RefMPK7-1 | Brdisv20Bd21Ref1006178m | Bradi1g34030 | + | 1242 | 413 | 2 | Bd1:29718819..29722676 |
|  | Bd21RefMPK7-2 | Brdisv20Bd21Ref1039827m | Bradi4g24914 | + | 5127 | 1708 | 11 | Bd4:30177573..30192609 |
|  | Bd21RefMPK11 | Brdisv20Bd21Ref1027806m | Bradi3g16560 | - | 1164 | 387 | 5 | Bd3:14739869..14744585 |
|  | Bd21RefMPK14 | Brdisv20Bd21Ref1025543m | Bradi3g03780 | - | 1110 | 369 | 2 | Bd3:2519851..2525848 |
|  | Bd21RefMPK16 | Brdisv20Bd21Ref1020074m | Bradi2g36470 | - | 1635 | 544 | 9 | Bd2:36799756..36808261 |
|  | Bd21RefMPK17 | Brdisv20Bd21Ref1006298m | Bradi1g34700 | + | 1746 | 581 | 10 | Bd1:30431238..30438643 |
|  | Bd21RefMPK20-1 | Brdisv20Bd21Ref1021539m | Bradi2g44350 | - | 1848 | 615 | 9 | Bd2:44871247..44880320 |
|  | Bd21RefMPK20-2 | Brdisv20Bd21Ref1016842m | Bradi2g15317 | + | 1827 | 608 | 9 | Bd2:13676842..13683413 |
|  | Bd21RefMPK20-3 | Brdisv20Bd21Ref1007272m | Bradi1g41780 | + | 1266 | 421 | 8 | Bd1:38631007..38650409 |
|  | Bd21RefMPK20-4 | Brdisv20Bd21Ref1021778m | Bradi2g45870 | + | 1743 | 580 | 9 | Bd2:46332590..46340975 |
|  | Bd21RefMPK20-5 | Brdisv20Bd21Ref1016994m | Bradi2g16337 | + | 1812 | 603 | 9 | Bd2:14400476..14407165 |
|  | Bd21RefMPK21-1 | Brdisv20Bd21Ref1016889m | Bradi2g15620 | - | 1788 | 595 | 10 | Bd2:13863076..13870851 |
|  | Bd21RefMPK21-2 | Brdisv20Bd21Ref1021630m | Bradi2g45006 | - | 1485 | 494 | 9 | Bd2:45423411..45432971 |
| Bd21-3 | Bd21-3MPK3 | Brdisv1Bd21-3_r1010642m | Bradi1g65810 | + | 1122 | 373 | 4 | pseudomolecule_1:58804643..58807730 |
|  | Bd21-3MPK4 | Brdisv1Bd21-3_r1027304m | Bradi3g32000 | + | 1134 | 377 | 7 | pseudomolecule_3:27856142..27862230 |
|  | Bd21-3MPK6 | Brdisv1Bd21-3_r1007822m | Bradi1g49100 | + | 1176 | 391 | 5 | pseudomolecule_1:42591074..42600069 |
|  | Bd21-3MPK11 | Brdisv1Bd21-3_r1025222m | Bradi3g16560 | - | 1188 | 395 | 5 | pseudomolecule_3:13192968..13197063 |
|  | Bd21-3MPK14 | Brdisv1Bd21-3_r1023190m | Bradi3g03780 | - | 1110 | 369 | 2 | pseudomolecule_3:2475916..2481913 |
|  | Bd21-3MPK16 | Brdisv1Bd21-3_r1018023m | Bradi2g36470 | - | 1635 | 544 | 9 | pseudomolecule_2:31042699..31051218 |
|  | Bd21-3MPK17 | Brdisv1Bd21-3_r1005812m | Bradi1g34700 | + | 1746 | 581 | 10 | pseudomolecule_1:28683542..28690903 |
|  | Bd21-3MPK20-1 | Brdisv1Bd21-3_r1019381m | Bradi2g44350 | - | 1833 | 610 | 9 | pseudomolecule_2:38529828..38538883 |
|  | Bd21-3MPK20-2 | Brdisv1Bd21-3_r1015197m | Bradi2g15317 | + | 1857 | 618 | 9 | pseudomolecule_2:13204203..13211812 |
|  | Bd21-3MPK20-3 | Brdisv1Bd21-3_r1006610m | Bradi1g41780 | + | 1266 | 421 | 8 | pseudomolecule_1:34390434..34409850 |
|  | Bd21-3MPK20-4 | Brdisv1Bd21-3_r1019613m | Bradi2g45870 | + | 1743 | 580 | 9 | pseudomolecule_2:39931118..39939503 |
|  | Bd21-3MPK20-5 | Brdisv1Bd21-3_r1015339m | Bradi2g16337 | + | 1812 | 603 | 9 | pseudomolecule_2:13927333..13934022 |
|  | Bd21-3MPK21-1 | Brdisv1Bd21-3_r1015240m | Bradi2g15620 | - | 1788 | 595 | 10 | pseudomolecule_2:13391815..13399589 |
|  | Bd21-3MPK21-2 | Brdisv1Bd21-3_r1019467m | Bradi2g45006 | - | 1485 | 494 | 9 | pseudomolecule_2:39005540..39015100 |
| Bd3-1 | Bd3-1MPK3 | Brdisv1Bd3-1_r1008330m | Bradi1g65810 | + | 1110 | 369 | 4 | pseudomolecule_1:41762188..41765729 |
|  | Bd3-1MPK4 | Brdisv1Bd3-1_r1021690m | Bradi3g32000 | + | 1137 | 378 | 7 | pseudomolecule_3:17365837..17371582 |
|  | Bd3-1MPK6 | Brdisv1Bd3-1_r1006154m | Bradi1g49100 | + | 747 | 248 | 4 | pseudomolecule_1:30572679..30579705 |
|  | Bd3-1MPK11 | Brdisv1Bd3-1_r1020329m | Bradi3g16560 | - | 1164 | 387 | 5 | pseudomolecule_3:10428351..10433066 |
|  | Bd3-1MPK14 | Brdisv1Bd3-1_r1018689m | Bradi3g03780 | - | 1110 | 369 | 2 | pseudomolecule_3:2234846..2240958 |
|  | Bd3-1MPK16 | Brdisv1Bd3-1_r1014394m | Bradi2g36470 | - | 1635 | 544 | 8 | pseudomolecule_2:21777934..21784785 |
|  | Bd3-1MPK17 | Brdisv1Bd3-1_r1004808m | Bradi1g34700 | + | 1746 | 581 | 10 | pseudomolecule_1:23115283..23122647 |
|  | Bd3-1MPK20-1 | Brdisv1Bd3-1_r1015467m | Bradi2g44350 | - | 1848 | 615 | 9 | pseudomolecule_2:27424794..27433577 |
|  | Bd3-1MPK20-2 | Brdisv1Bd3-1_r1012296m | Bradi2g15317 | + | 1827 | 608 | 9 | pseudomolecule_2:10841711..10848968 |
|  | Bd3-1MPK20-3 | Brdisv1Bd3-1_r1005298m | Bradi1g41780 | + | 1221 | 408 | 7 | pseudomolecule_1:25764868..25777283 |
|  | Bd3-1MPK20-4 | Brdisv1Bd3-1_r1015662m | Bradi2g45870 | + | 1788 | 595 | 9 | pseudomolecule_2:28500781..28509235 |
|  | Bd3-1MPK20-5 | Brdisv1Bd3-1_r1012412m | Bradi2g16337 | + | 1746 | 581 | 9 | pseudomolecule_2:11428211..11434573 |
|  | Bd3-1MPK21-1 | Brdisv1Bd3-1_r1012338m | Bradi2g15620 | - | 1497 | 498 | 10 | pseudomolecule_2:11013953..11021741 |
|  | Bd3-1MPK21-2 | Brdisv1Bd3-1_r1015518m | Bradi2g45006 | - | 1173 | 390 | 8 | pseudomolecule_2:27676106..27685581 |
| Bd2-3 | Bd2-3MPK3 | Brdisv1Bd2-31009829m | Bradi1g65810 | + | 1110 | 369 | 4 | pseudomolecule_1:42505985..42509526 |
|  | Bd2-3MPK4 | Brdisv1Bd2-31032148m | Bradi3g32000 | + | 1134 | 377 | 7 | pseudomolecule_4:18793314..18799402 |
|  | Bd2-3MPK6 | Brdisv1Bd2-31007253m | Bradi1g49100 | + | 1176 | 391 | 5 | pseudomolecule_1:31294601..31303568 |
|  | Bd2-3MPK7-2 | Brdisv1Bd2-31024751m | Bradi4g24914 | + | 5127 | 1708 | 11 | pseudomolecule_3:16257630..16278452 |
|  | Bd2-3MPK11 | Brdisv1Bd2-31030457m | Bradi3g16560 | - | 1164 | 387 | 5 | pseudomolecule_4:10772699..10777414 |
|  | Bd2-3MPK14 | Brdisv1Bd2-31028513m | Bradi3g03780 | - | 1110 | 369 | 2 | pseudomolecule_4:2283022..2289019 |
|  | Bd2-3MPK16 | Brdisv1Bd2-31016926m | Bradi2g36470 | - | 1635 | 544 | 9 | pseudomolecule_2:21674364..21682882 |
|  | Bd2-3MPK17 | Brdisv1Bd2-31005374m | Bradi1g34700 | + | 1746 | 581 | 10 | pseudomolecule_1:22253276..22260645 |
|  | Bd2-3MPK20-1 | Brdisv1Bd2-31018261m | Bradi2g44350 | + | 1848 | 615 | 9 | pseudomolecule_2:27828816..27837889 |
|  | Bd2-3MPK20-2 | Brdisv1Bd2-31014393m | Bradi2g15317 | + | 1827 | 608 | 9 | pseudomolecule_2:10761850..10769452 |
|  | Bd2-3MPK20-3 | Brdisv1Bd2-31042613m | Bradi1g41780 | + | 624 | 208 | 3 | pseudomolecule_8:21064923..21071851 |
|  | Bd2-3MPK20-4 | Brdisv1Bd2-31018474m | Bradi2g45870 | + | 1743 | 580 | 9 | pseudomolecule_2:28862112..28870497 |
|  | Bd2-3MPK20-5 | Brdisv1Bd2-31014547m | Bradi2g16337 | + | 1812 | 603 | 9 | pseudomolecule_2:11355903..11362579 |
|  | Bd2-3MPK21-1 | Brdisv1Bd2-31014439m | Bradi2g15620 | - | 1788 | 595 | 10 | pseudomolecule_2:10935897..10943672 |
| Adi-10 | Adi-10MPK3 | Brdisv1Adi-101031639m | Bradi1g65810 | - | 1134 | 377 | 4 | pseudomolecule_7:6347651..6350947 |
|  | Adi-10MPK4 | Brdisv1Adi-101037043m | Bradi3g32000 | - | 957 | 318 | 5 | pseudomolecule_7:49303717..49308940 |
|  | Adi-10MPK6 | Brdisv1Adi-101005339m | Bradi1g49100 | + | 1176 | 391 | 5 | pseudomolecule_1:22660972..22670064 |
|  | Adi-10MPK7-1 | Brdisv1Adi-101018783m | Bradi1g34030 | + | 1242 | 413 | 2 | pseudomolecule_3:11981054..11985778 |
|  | Adi-10MPK11 | Brdisv1Adi-101023173m | Bradi3g16560 | - | 1164 | 387 | 5 | pseudomolecule_4:8098584..8103299 |
|  | Adi-10MPK14 | Brdisv1Adi-101031400m | Bradi3g03780 | - | 1110 | 369 | 2 | pseudomolecule_7:4530507..4536514 |
|  | Adi-10MPK16 | Brdisv1Adi-101033836m | Bradi2g36470 | + | 1635 | 544 | 9 | pseudomolecule_7:23778113..23786650 |
|  | Adi-10MPK17 | Brdisv1Adi-101004044m | Bradi1g34700 | + | 1797 | 598 | 11 | pseudomolecule_1:16456444..16463169 |
|  | Adi-10MPK20-1 | Brdisv1Adi-101013839m | Bradi2g44350 | + | 1848 | 615 | 9 | pseudomolecule_2:20267432..20276505 |
|  | Adi-10MPK20-2 | Brdisv1Adi-101033960m | Bradi2g15317 | + | 1827 | 608 | 9 | pseudomolecule_7:24700748..24708335 |
|  | Adi-10MPK20-3 | Brdisv1Adi-101034957m | Bradi1g41780 | - | 651 | 216 | 3 | pseudomolecule_7:32716951..32723959 |
|  | Adi-10MPK20-4 | Brdisv1Adi-101014002m | Bradi2g45870 | + | 1743 | 580 | 9 | pseudomolecule_2:20969757..20978008 |
|  | Adi-10MPK20-5 | Brdisv1Adi-101010961m | Bradi2g16337 | + | 1812 | 603 | 9 | pseudomolecule_2:8086940..8093619 |
|  | Adi-10MPK21-1 | Brdisv1Adi-101013900m | Bradi2g15620 | - | 1497 | 498 | 10 | pseudomolecule_2:20476315..20483658 |
|  | Adi-10MPK21-2 | Brdisv1Adi-101031490m | Bradi2g45006 | + | 1173 | 390 | 8 | pseudomolecule_7:5336808..5345501 |
| Adi-2 | Adi-2MPK3 | Brdisv1Adi-21010752m | Bradi1g65810 | + | 1110 | 369 | 4 | pseudomolecule_1:51339793..51343334 |
|  | Adi-2MPK4 | Brdisv1Adi-21035342m | Bradi3g32000 | + | 1134 | 377 | 7 | pseudomolecule_4:23946362..23952450 |
|  | Adi-2MPK6 | Brdisv1Adi-21007714m | Bradi1g49100 | + | 1176 | 391 | 5 | pseudomolecule_1:36723347..36732304 |
|  | Adi-2MPK7-1 | Brdisv1Adi-21046113m | Bradi1g34030 | - | 1119 | 372 | 1 | pseudomolecule_8:41486428..41488660 |
|  | Adi-2MPK11 | Brdisv1Adi-21033371m | Bradi3g16560 | - | 1164 | 387 | 5 | pseudomolecule_4:12807962..12812674 |
|  | Adi-2MPK14 | Brdisv1Adi-21031107m | Bradi3g03780 | - | 1110 | 369 | 2 | pseudomolecule_4:2433058..2439049 |
|  | Adi-2MPK16 | Brdisv1Adi-21018516m | Bradi2g36470 | - | 1635 | 544 | 9 | pseudomolecule_2:26607755..26616260 |
|  | Adi-2MPK17 | Brdisv1Adi-21005644m | Bradi1g34700 | + | 1746 | 581 | 10 | pseudomolecule_1:25063033..25070394 |
|  | Adi-2MPK20-1 | Brdisv1Adi-21020002m | Bradi2g44350 | - | 1848 | 615 | 9 | pseudomolecule_2:34126635..34135708 |
|  | Adi-2MPK20-2 | Brdisv1Adi-21015730m | Bradi2g15317 | + | 1917 | 638 | 9 | pseudomolecule_2:12517643..12524587 |
|  | Adi-2MPK20-3 | Brdisv1Adi-21045430m | Bradi1g41780 | + | 624 | 208 | 3 | pseudomolecule_8:21247492..21254420 |
|  | Adi-2MPK20-4 | Brdisv1Adi-21020252m | Bradi2g45870 | + | 1743 | 580 | 9 | pseudomolecule_2:35459133..35467518 |
|  | Adi-2MPK20-5 | Brdisv1Adi-21015890m | Bradi2g16337 | + | 1812 | 603 | 9 | pseudomolecule_2:13272744..13279433 |
|  | Adi-2MPK21-1 | Brdisv1Adi-21015775m | Bradi2g15620 | - | 1788 | 595 | 10 | pseudomolecule_2:12687712..12695486 |
|  | Adi-2MPK21-2 | Brdisv1Adi-21020093m | Bradi2g45006 | - | 1485 | 494 | 9 | pseudomolecule_2:34628753..34638313 |
| Adi-12 | Adi-12MPK3 | Brdisv1Adi-121041121m | Bradi1g65810 | - | 1110 | 369 | 4 | pseudomolecule_7:7473978..7477519 |
|  | Adi-12MPK4 | Brdisv1Adi-121032162m | Bradi3g32000 | + | 1077 | 358 | 6 | pseudomolecule_4:19283709..19289435 |
|  | Adi-12MPK6 | Brdisv1Adi-121007265m | Bradi1g49100 | + | 942 | 313 | 4 | pseudomolecule_1:31890613..31896022 |
|  | Adi-12MPK7-1 | Brdisv1Adi-121024789m | Bradi1g34030 | + | 1242 | 413 | 2 | pseudomolecule_3:16432718..16436141 |
|  | Adi-12MPK11 | Brdisv1Adi-121030403m | Bradi3g16560 | - | 1164 | 387 | 5 | pseudomolecule_4:10753103..10757818 |
|  | Adi-12MPK14 | Brdisv1Adi-121005230m | Bradi3g03780 | + | 1110 | 369 | 2 | pseudomolecule_1:21748963..21754869 |
|  | Adi-12MPK16 | Brdisv1Adi-121016891m | Bradi2g36470 | - | 1635 | 544 | 9 | pseudomolecule_2:22050219..22058721 |
|  | Adi-12MPK17 | Brdisv1Adi-121005354m | Bradi1g34700 | + | 1746 | 581 | 10 | pseudomolecule_1:22322524..22329888 |
|  | Adi-12MPK20-1 | Brdisv1Adi-121018246m | Bradi2g44350 | - | 1848 | 615 | 9 | pseudomolecule_2:28404394..28413467 |
|  | Adi-12MPK20-2 | Brdisv1Adi-121014318m | Bradi2g15317 | + | 1917 | 638 | 9 | pseudomolecule_2:10389830..10396774 |
|  | Adi-12MPK20-3 | Brdisv1Adi-121042759m | Bradi1g41780 | + | 624 | 208 | 3 | pseudomolecule_7:23967507..23974435 |
|  | Adi-12MPK20-4 | Brdisv1Adi-121018451m | Bradi2g45870 | + | 1743 | 580 | 9 | pseudomolecule_2:29421173..29429558 |
|  | Adi-12MPK20-5 | Brdisv1Adi-121014469m | Bradi2g16337 | + | 1812 | 603 | 9 | pseudomolecule_2:11051681..11058357 |
|  | Adi-12MPK21-1 | Brdisv1Adi-121014362m | Bradi2g15620 | - | 1788 | 595 | 10 | pseudomolecule_2:10566875..10574649 |
|  | Adi-12MPK21-2 | Brdisv1Adi-121040934m | Bradi2g45006 | - | 1713 | 570 | 9 | pseudomolecule_7:5862145..5871449 |
| BdTR9k | BdTR9kMPK3 | Brdisv1BdTR9K1009255m | Bradi1g65810 | + | 1110 | 369 | 4 | pseudomolecule_1:39370895..39374436 |
|  | BdTR9kMPK4 | Brdisv1BdTR9K1030513m | Bradi3g32000 | + | 1134 | 377 | 7 | pseudomolecule_4:17318999..17325086 |
|  | BdTR9kMPK6 | Brdisv1BdTR9K1006792m | Bradi1g49100 | + | 942 | 313 | 4 | pseudomolecule_1:28695583..28700992 |
|  | BdTR9kMPK7-1 | Brdisv1BdTR9K1043911m | Bradi1g34030 | - | 1110 | 369 | 1 | pseudomolecule_7:60053577..60055646 |
|  | BdTR9kMPK11 | Brdisv1BdTR9K1028898m | Bradi3g16560 | - | 1164 | 387 | 5 | pseudomolecule_4:9817428..9822143 |
|  | BdTR9kMPK14 | Brdisv1BdTR9K1027057m | Bradi3g03780 | - | 1110 | 369 | 2 | pseudomolecule_4:1986369..1992334 |
|  | BdTR9kMPK16 | Brdisv1BdTR9K1016140m | Bradi2g36470 | - | 1635 | 544 | 9 | pseudomolecule_2:20373969..20382471 |
|  | BdTR9kMPK17 | Brdisv1BdTR9K1005090m | Bradi1g34700 | + | 1746 | 581 | 10 | pseudomolecule_1:20559669..20567033 |
|  | BdTR9kMPK20-1 | Brdisv1BdTR9K1017427m | Bradi2g44350 | + | 1848 | 615 | 9 | pseudomolecule_2:26037303..26046376 |
|  | BdTR9kMPK20-2 | Brdisv1BdTR9K1041463m | Bradi2g15317 | + | 1827 | 608 | 9 | pseudomolecule_7:26211544..26219048 |
|  | BdTR9kMPK20-3 | Brdisv1BdTR9K1041205m | Bradi1g41780 | + | 624 | 208 | 3 | pseudomolecule_7:23889208..23896136 |
|  | BdTR9kMPK20-4 | Brdisv1BdTR9K1017617m | Bradi2g45870 | + | 1743 | 580 | 9 | pseudomolecule_2:26902618..26911003 |
|  | BdTR9kMPK20-5 | Brdisv1BdTR9K1013831m | Bradi2g16337 | + | 1812 | 603 | 9 | pseudomolecule_2:10558317..10565006 |
|  | BdTR9kMPK21-1 | Brdisv1BdTR9K1013728m | Bradi2g15620 | - | 1788 | 595 | 10 | pseudomolecule_2:10109947..10117721 |
|  | BdTR9kMPK21-2 | Brdisv1BdTR9K1038831m | Bradi2g45006 | - | 1485 | 494 | 9 | pseudomolecule_7:4362134..4371617 |
| BdTR12c | BdTR12cMPK3 | Brdisv1BdTR12c1009957m | Bradi1g65810 | + | 1110 | 369 | 4 | pseudomolecule_1:43320221..43323762 |
|  | BdTR12cMPK4 | Brdisv1BdTR12c1031941m | Bradi3g32000 | + | 1134 | 377 | 7 | pseudomolecule_4:17971730..17977818 |
|  | BdTR12cMPK6 | Brdisv1BdTR12c1007189m | Bradi1g49100 | + | 942 | 313 | 4 | pseudomolecule_1:31083070..31088475 |
|  | BdTR12cMPK7-1 | Brdisv1BdTR12c1005219m | Bradi1g34030 | + | 1242 | 413 | 2 | pseudomolecule_1:21623003..21627027 |
|  | BdTR12cMPK11 | Brdisv1BdTR12c1030387m | Bradi3g16560 | - | 1164 | 387 | 5 | pseudomolecule_4:10946879..10951594 |
|  | BdTR12cMPK14 | Brdisv1BdTR12c1028321m | Bradi3g03780 | - | 1110 | 369 | 2 | pseudomolecule_4:2097833..2103831 |
|  | BdTR12cMPK16 | Brdisv1BdTR12c1013400m | Bradi2g36470 | - | 1635 | 544 | 9 | pseudomolecule_2:6003273..6011827 |
|  | BdTR12cMPK17 | Brdisv1BdTR12c1000261m | Bradi1g34700 | + | 1746 | 581 | 10 | pseudomolecule_1:951169..958532 |
|  | BdTR12cMPK20-1 | Brdisv1BdTR12c1018145m | Bradi2g44350 | + | 1848 | 615 | 9 | pseudomolecule_2:26983119..26992192 |
|  | BdTR12cMPK20-2 | Brdisv1BdTR12c1014428m | Bradi2g15317 | + | 1827 | 608 | 9 | pseudomolecule_2:10532217..10539635 |
|  | BdTR12cMPK20-3 | Brdisv1BdTR12c1042500m | Bradi1g41780 | - | 651 | 216 | 3 | pseudomolecule_8:21452122..21459087 |
|  | BdTR12cMPK20-4 | Brdisv1BdTR12c1018156m | Bradi2g45870 | + | 1743 | 580 | 9 | pseudomolecule_2:27008710..27017095 |
|  | BdTR12cMPK20-5 | Brdisv1BdTR12c1014592m | Bradi2g16337 | + | 1812 | 603 | 9 | pseudomolecule_2:11293044..11299720 |
|  | BdTR12cMPK21-1 | Brdisv1BdTR12c1014472m | Bradi2g15620 | - | 1788 | 595 | 10 | pseudomolecule_2:10705129..10712904 |
|  | BdTR12cMPK21-2 | Brdisv1BdTR12c1018230m | Bradi2g45006 | - | 1485 | 494 | 9 | pseudomolecule_2:27329257..27338817 |
| Kah-1 | Kah-1MPK3 | Brdisv1Kah-11008192m | Bradi1g65810 | + | 1110 | 369 | 4 | pseudomolecule_1:33391424..33394965 |
|  | Kah-1MPK4 | Brdisv1Kah-11021773m | Bradi3g32000 | + | 1134 | 377 | 7 | pseudomolecule_3:15150734..15156822 |
|  | Kah-1MPK6 | Brdisv1Kah-11006028m | Bradi1g49100 | + | 822 | 273 | 3 | pseudomolecule_1:24480423..24484907 |
|  | Kah-1MPK7-1 | Brdisv1Kah-11044255m | Bradi1g34030 | + | 837 | 278 | 1 | pseudomolecule_7:89302941..89307126 |
|  | Kah-1MPK11 | Brdisv1Kah-11020359m | Bradi3g16560 | - | 1164 | 387 | 5 | pseudomolecule_3:9123269..9127984 |
|  | Kah-1MPK14 | Brdisv1Kah-11018519m | Bradi3g03780 | - | 1110 | 369 | 2 | pseudomolecule_3:1932059..1938084 |
|  | Kah-1MPK16 | Brdisv1Kah-11014205m | Bradi2g36470 | - | 1635 | 544 | 9 | pseudomolecule_2:16919471..16927976 |
|  | Kah-1MPK20-1 | Brdisv1Kah-11035683m | Bradi2g44350 | + | 1146 | 381 | 6 | pseudomolecule_7:10830364..10836109 |
|  | Kah-1MPK20-2 | Brdisv1Kah-11012143m | Bradi2g15317 | + | 1827 | 608 | 9 | pseudomolecule_2:8559863..8567357 |
|  | Kah-1MPK20-3 | Brdisv1Kah-11037354m | Bradi1g41780 | - | 651 | 216 | 3 | pseudomolecule_7:22602944..22609910 |
|  | Kah-1MPK20-4 | Brdisv1Kah-11015432m | Bradi2g45870 | + | 1011 | 336 | 7 | pseudomolecule_2:22188253..22192741 |
|  | Kah-1MPK20-5 | Brdisv1Kah-11012290m | Bradi2g16337 | + | 1812 | 603 | 9 | pseudomolecule_2:9173851..9180527 |
|  | Kah-1MPK21-1 | Brdisv1Kah-11012177m | Bradi2g15620 | - | 1788 | 595 | 10 | pseudomolecule_2:8693744..8701518 |
|  | Kah-1MPK21-2 | Brdisv1Kah-11015331m | Bradi2g45006 | - | 1485 | 494 | 9 | pseudomolecule_2:21721936..21731496 |
| Kah-5 | Kah-5MPK3 | Brdisv1Kah-51010925m | Bradi1g65810 | + | 1110 | 369 | 4 | pseudomolecule_1:51259033..51262574 |
|  | Kah-5MPK4 | Brdisv1Kah-51035392m | Bradi3g32000 | + | 1134 | 377 | 7 | pseudomolecule_4:23239965..23246053 |
|  | Kah-5MPK6 | Brdisv1Kah-51007875m | Bradi1g49100 | + | 1176 | 391 | 5 | pseudomolecule_1:36629857..36638832 |
|  | Kah-5MPK7-1 | Brdisv1Kah-51046477m | Bradi1g34030 | + | 835 | 277 | 1 | pseudomolecule_8:47747487..47749990 |
|  | Kah-5MPK11 | Brdisv1Kah-51033455m | Bradi3g16560 | - | 1164 | 387 | 5 | pseudomolecule_4:12398980..12403695 |
|  | Kah-5MPK14 | Brdisv1Kah-51031281m | Bradi3g03780 | - | 1110 | 369 | 2 | pseudomolecule_4:2371759..2377756 |
|  | Kah-5MPK16 | Brdisv1Kah-51018690m | Bradi2g36470 | - | 1635 | 544 | 9 | pseudomolecule_2:26410766..26419271 |
|  | Kah-5MPK17 | Brdisv1Kah-51005745m | Bradi1g34700 | + | 1746 | 581 | 10 | pseudomolecule_1:25178957..25186321 |
|  | Kah-5MPK20-1 | Brdisv1Kah-51020173m | Bradi2g44350 | - | 1848 | 615 | 9 | pseudomolecule_2:33905141..33914214 |
|  | Kah-5MPK20-2 | Brdisv1Kah-51015861m | Bradi2g15317 | + | 1917 | 638 | 9 | pseudomolecule_2:12397623..12404634 |
|  | Kah-5MPK20-3 | Brdisv1Kah-51045497m | Bradi1g41780 | + | 624 | 208 | 3 | pseudomolecule_8:19464794..19471722 |
|  | Kah-5MPK20-4 | Brdisv1Kah-51020414m | Bradi2g45870 | + | 1743 | 580 | 9 | pseudomolecule_2:35156808..35165193 |
|  | Kah-5MPK20-5 | Brdisv1Kah-51016005m | Bradi2g16337 | + | 1812 | 603 | 9 | pseudomolecule_2:13119110..13125799 |
|  | Kah-5MPK21-1 | Brdisv1Kah-51015904m | Bradi2g15620 | - | 1788 | 595 | 10 | pseudomolecule_2:12574512..12582286 |
|  | Kah-5MPK21-2 | Brdisv1Kah-51020257m | Bradi2g45006 | - | 1485 | 494 | 9 | pseudomolecule_2:34341474..34351034 |
| BdTR5i | BdTR5iMPK3 | Brdisv1BdTR5I1009348m | Bradi1g65810 | + | 1110 | 369 | 4 | pseudomolecule_1:40020213..40023754 |
|  | BdTR5iMPK4 | Brdisv1BdTR5I1030484m | Bradi3g32000 | + | 1134 | 377 | 7 | pseudomolecule_4:17287098..17293186 |
|  | BdTR5iMPK6 | Brdisv1BdTR5I1006823m | Bradi1g49100 | + | 1176 | 391 | 5 | pseudomolecule_1:29048133..29057121 |
|  | BdTR5iMPK7-1 | Brdisv1BdTR5I1004945m | Bradi1g34030 | + | 1242 | 413 | 2 | pseudomolecule_1:20227821..20231843 |
|  | BdTR5iMPK11 | Brdisv1BdTR5I1028828m | Bradi3g16560 | - | 1164 | 387 | 5 | pseudomolecule_4:9827604..9832319 |
|  | BdTR5iMPK14 | Brdisv1BdTR5I1026907m | Bradi3g03780 | - | 1110 | 369 | 2 | pseudomolecule_4:1941522..1947519 |
|  | BdTR5iMPK16 | Brdisv1BdTR5I1039980m | Bradi2g36470 | - | 1635 | 544 | 9 | pseudomolecule_8:14254984..14263489 |
|  | BdTR5iMPK17 | Brdisv1BdTR5I1005052m | Bradi1g34700 | + | 1746 | 581 | 10 | pseudomolecule_1:20698123..20705487 |
|  | BdTR5iMPK20-1 | Brdisv1BdTR5I1017346m | Bradi2g44350 | - | 1848 | 615 | 9 | pseudomolecule_2:25768348..25777421 |
|  | BdTR5iMPK20-2 | Brdisv1BdTR5I1040580m | Bradi2g15317 | + | 1827 | 608 | 9 | pseudomolecule_8:19629449..19636931 |
|  | BdTR5iMPK20-3 | Brdisv1BdTR5I1041219m | Bradi1g41780 | - | 651 | 216 | 3 | pseudomolecule_8:25347260..25354225 |
|  | BdTR5iMPK20-4 | Brdisv1BdTR5I1017548m | Bradi2g45870 | + | 1743 | 580 | 9 | pseudomolecule_2:26692611..26700505 |
|  | BdTR5iMPK20-5 | Brdisv1BdTR5I1013896m | Bradi2g16337 | + | 1812 | 603 | 9 | pseudomolecule_2:10757700..10764389 |
|  | BdTR5iMPK21-1 | Brdisv1BdTR5I1013788m | Bradi2g15620 | - | 1788 | 595 | 10 | pseudomolecule_2:10278503..10286313 |
|  | BdTR5iMPK21-2 | Brdisv1BdTR5I1017428m | Bradi2g45006 | - | 1485 | 494 | 9 | pseudomolecule_2:26103512..26113072 |
| BdTR10c | BdTR10cMPK3 | Brdisv1BdTR10C1009137m | Bradi1g65810 | + | 1566 | 521 | 10 | pseudomolecule_1:20429680..20437072 |
|  | BdTR10cMPK4 | Brdisv1BdTR10C1023812m | Bradi3g32000 | + | 1128 | 375 | 7 | pseudomolecule_3:17874038..17880140 |
|  | BdTR10cMPK6 | Brdisv1BdTR10C1006602m | Bradi1g49100 | + | 1143 | 380 | 5 | pseudomolecule_1:28676682..28685550 |
|  | BdTR10cMPK7-1 | Brdisv1BdTR10C1031091m | Bradi1g34030 | + | 1242 | 413 | 2 | pseudomolecule_4:15473929..15477468 |
|  | BdTR10cMPK11 | Brdisv1BdTR10C1022204m | Bradi3g16560 | - | 1164 | 387 | 5 | pseudomolecule_3:9981367..9986079 |
|  | BdTR10cMPK14 | Brdisv1BdTR10C1020364m | Bradi3g03780 | - | 1110 | 369 | 2 | pseudomolecule_3:2069122..2075037 |
|  | BdTR10cMPK16 | Brdisv1BdTR10C1015705m | Bradi2g36470 | - | 1635 | 544 | 9 | pseudomolecule_2:20271131..20279636 |
|  | BdTR10cMPK17 | Brdisv1BdTR10C1004902m | Bradi1g34700 | + | 1566 | 521 | 10 | pseudomolecule_1:20429680..20437072 |
|  | BdTR10cMPK20-1 | Brdisv1BdTR10C1016940m | Bradi2g44350 | - | 1848 | 615 | 9 | pseudomolecule_2:26155121..26164194 |
|  | BdTR10cMPK20-2 | Brdisv1BdTR10C1013382m | Bradi2g15317 | + | 1827 | 608 | 9 | pseudomolecule_2:9915281..9922840 |
|  | BdTR10cMPK20-3 | Brdisv1BdTR10C1041496m | Bradi1g41780 | - | 651 | 216 | 3 | pseudomolecule_8:37625905..37632596 |
|  | BdTR10cMPK20-4 | Brdisv1BdTR10C1017120m | Bradi2g45870 | + | 1743 | 580 | 9 | pseudomolecule_2:27077241..27085640 |
|  | BdTR10cMPK20-5 | Brdisv1BdTR10C1013523m | Bradi2g16337 | + | 1812 | 603 | 9 | pseudomolecule_2:10540978..10547667 |
|  | BdTR10cMPK21-1 | Brdisv1BdTR10C1017009m | Bradi2g15620 | - | 1497 | 498 | 10 | pseudomolecule_2:26465042..26472231 |
|  | BdTR10cMPK21-2 | Brdisv1BdTR10C1038225m | Bradi2g45006 | + | 1773 | 590 | 8 | pseudomolecule_8:5986618..5994410 |
| BdTR11a | BdTR11aMPK3 | Brdisv1BdTR11A1009650m | Bradi1g65810 | + | 1110 | 369 | 4 | pseudomolecule_1:40160100..40163641 |
|  | BdTR11aMPK4 | Brdisv1BdTR11A1031736m | Bradi3g32000 | + | 1134 | 377 | 7 | pseudomolecule_4:17796717..17802805 |
|  | BdTR11aMPK6 | Brdisv1BdTR11A1006993m | Bradi1g49100 | + | 1176 | 391 | 5 | pseudomolecule_1:29169130..29178118 |
|  | BdTR11aMPK11 | Brdisv1BdTR11A1030105m | Bradi3g16560 | - | 1164 | 387 | 5 | pseudomolecule_4:10671675..10676387 |
|  | BdTR11aMPK14 | Brdisv1BdTR11A1028042m | Bradi3g03780 | - | 1110 | 369 | 2 | pseudomolecule_4:2208183..2214145 |
|  | BdTR11aMPK16 | Brdisv1BdTR11A1016556m | Bradi2g36470 | - | 1635 | 544 | 9 | pseudomolecule_2:19887634..19896139 |
|  | BdTR11aMPK17 | Brdisv1BdTR11A1005247m | Bradi1g34700 | + | 1746 | 581 | 10 | pseudomolecule_1:21231237..21238601 |
|  | BdTR11aMPK20-1 | Brdisv1BdTR11A1017974m | Bradi2g44350 | - | 1848 | 615 | 9 | pseudomolecule_2:25859245..25868318 |
|  | BdTR11aMPK20-2 | Brdisv1BdTR11A1045880m | Bradi2g15317 | + | 1827 | 608 | 9 | pseudomolecule_7:42132782..42140325 |
|  | BdTR11aMPK20-3 | Brdisv1BdTR11A1045462m | Bradi1g41780 | - | 651 | 216 | 3 | pseudomolecule_7:38891114..38898079 |
|  | BdTR11aMPK20-4 | Brdisv1BdTR11A1018174m | Bradi2g45870 | + | 1743 | 580 | 9 | pseudomolecule_2:26742913..26751298 |
|  | BdTR11aMPK20-5 | Brdisv1BdTR11A1014255m | Bradi2g16337 | + | 1812 | 603 | 9 | pseudomolecule_2:10537076..10543765 |
|  | BdTR11aMPK21-1 | Brdisv1BdTR11A1014151m | Bradi2g15620 | - | 1788 | 595 | 10 | pseudomolecule_2:10121938..10129713 |
|  | BdTR11aMPK21-2 | Brdisv1BdTR11A1018053m | Bradi2g45006 | - | 1485 | 494 | 9 | pseudomolecule_2:26187156..26196716 |
| BdTR11i | BdTR11iMPK3 | Brdisv1BdTR11I1010553m | Bradi1g65810 | + | 1110 | 369 | 4 | pseudomolecule_1:47557340..47560881 |
|  | BdTR11iMPK4 | Brdisv1BdTR11I1034234m | Bradi3g32000 | + | 1134 | 377 | 7 | pseudomolecule_4:21083341..21089429 |
|  | BdTR11iMPK6 | Brdisv1BdTR11I1007652m | Bradi1g49100 | + | 1176 | 391 | 5 | pseudomolecule_1:34376675..34385657 |
|  | BdTR11iMPK7-1 | Brdisv1BdTR11I1045888m | Bradi1g34030 | - | 687 | 228 | 0 | pseudomolecule_7:56411564..56412524 |
|  | BdTR11iMPK11 | Brdisv1BdTR11I1032437m | Bradi3g16560 | - | 1164 | 387 | 5 | pseudomolecule_4:11986893..11991605 |
|  | BdTR11iMPK14 | Brdisv1BdTR11I1030242m | Bradi3g03780 | - | 1110 | 369 | 2 | pseudomolecule_4:2372948..2378910 |
|  | BdTR11iMPK16 | Brdisv1BdTR11I1018043m | Bradi2g36470 | - | 1635 | 544 | 9 | pseudomolecule_2:23968565..23977070 |
|  | BdTR11iMPK17 | Brdisv1BdTR11I1005619m | Bradi1g34700 | + | 1746 | 581 | 10 | pseudomolecule_1:23865130..23872494 |
|  | BdTR11iMPK20-1 | Brdisv1BdTR11I1019460m | Bradi2g44350 | - | 1848 | 615 | 9 | pseudomolecule_2:30583279..30592352 |
|  | BdTR11iMPK20-2 | Brdisv1BdTR11I1015383m | Bradi2g15317 | + | 1917 | 638 | 10 | pseudomolecule_2:11896614..11903625 |
|  | BdTR11iMPK20-3 | Brdisv1BdTR11I1044565m | Bradi1g41780 | + | 624 | 208 | 3 | pseudomolecule_7:19623328..19630256 |
|  | BdTR11iMPK20-4 | Brdisv1BdTR11I1019682m | Bradi2g45870 | + | 1743 | 580 | 9 | pseudomolecule_2:31695833..31704218 |
|  | BdTR11iMPK20-5 | Brdisv1BdTR11I1015521m | Bradi2g16337 | + | 1812 | 603 | 9 | pseudomolecule_2:12486250..12492939 |
|  | BdTR11iMPK21-1 | Brdisv1BdTR11I1015413m | Bradi2g15620 | - | 1788 | 595 | 10 | pseudomolecule_2:12011417..12019192 |
|  | BdTR11iMPK21-2 | Brdisv1BdTR11I1019550m | Bradi2g45006 | - | 1485 | 494 | 9 | pseudomolecule_2:31002471..31012031 |
| BdTR11g | BdTR11gMPK3 | Brdisv1BdTR11G1010947m | Bradi1g65810 | + | 1110 | 369 | 4 | pseudomolecule_1:52285736..52289277 |
|  | BdTR11gMPK4 | Brdisv1BdTR11G1035183m | Bradi3g32000 | + | 1134 | 377 | 7 | pseudomolecule_4:23888154..23894242 |
|  | BdTR11gMPK6 | Brdisv1BdTR11G1007922m | Bradi1g49100 | + | 1176 | 391 | 5 | pseudomolecule_1:37721315..37730316 |
|  | BdTR11gMPK7-1 | Brdisv1BdTR11G1046470m | Bradi1g34030 | - | 687 | 228 | 0 | pseudomolecule_7:46987725..46988685 |
|  | BdTR11gMPK11 | Brdisv1BdTR11G1033165m | Bradi3g16560 | - | 1164 | 387 | 5 | pseudomolecule_4:12390065..12394777 |
|  | BdTR11gMPK14 | Brdisv1BdTR11G1031078m | Bradi3g03780 | - | 1110 | 369 | 2 | pseudomolecule_4:2377692..2383654 |
|  | BdTR11gMPK16 | Brdisv1BdTR11G1018684m | Bradi2g36470 | - | 1635 | 544 | 9 | pseudomolecule_2:26890860..26899365 |
|  | BdTR11gMPK17 | Brdisv1BdTR11G1005789m | Bradi1g34700 | + | 1746 | 581 | 10 | pseudomolecule_1:25948543..25955907 |
|  | BdTR11gMPK20-1 | Brdisv1BdTR11G1020132m | Bradi2g44350 | - | 1848 | 615 | 9 | pseudomolecule_2:34335699..34344772 |
|  | BdTR11gMPK20-2 | Brdisv1BdTR11G1015901m | Bradi2g15317 | + | 1917 | 638 | 10 | pseudomolecule_2:12654976..12661987 |
|  | BdTR11gMPK20-3 | Brdisv1BdTR11G1045432m | Bradi1g41780 | + | 624 | 208 | 3 | pseudomolecule_7:18777568..18784496 |
|  | BdTR11gMPK20-4 | Brdisv1BdTR11G1024979m | Bradi2g45870 | + | 1743 | 580 | 9 | pseudomolecule_3:7570137..7578522 |
|  | BdTR11gMPK20-5 | Brdisv1BdTR11G1016043m | Bradi2g16337 | + | 1812 | 603 | 9 | pseudomolecule_2:13275024..13281713 |
|  | BdTR11gMPK21-1 | Brdisv1BdTR11G1015942m | Bradi2g15620 | - | 1788 | 595 | 10 | pseudomolecule_2:12817151..12824926 |
|  | BdTR11gMPK21-2 | Brdisv1BdTR11G1020223m | Bradi2g45006 | - | 1485 | 494 | 9 | pseudomolecule_2:34830844..34840404 |
| BdTR13a | BdTR13aMPK3 | Brdisv1BdTR13a1011439m | Bradi1g65810 | + | 1110 | 369 | 4 | pseudomolecule_1:60133106..60136647 |
|  | BdTR13aMPK4 | Brdisv1BdTR13a1037138m | Bradi3g32000 | + | 1134 | 377 | 7 | pseudomolecule_4:28686102..28692190 |
|  | BdTR13aMPK6 | Brdisv1BdTR13a1008255m | Bradi1g49100 | + | 1176 | 391 | 5 | pseudomolecule_1:43413597..43422568 |
|  | BdTR13aMPK7-1 | Brdisv1BdTR13a1047101m | Bradi1g34030 | + | 891 | 296 | 1 | pseudomolecule_8:21320615..21322547 |
|  | BdTR13aMPK11 | Brdisv1BdTR13a1035020m | Bradi3g16560 | - | 1164 | 387 | 5 | pseudomolecule_4:13866786..13871501 |
|  | BdTR13aMPK14 | Brdisv1BdTR13a1032768m | Bradi3g03780 | - | 1110 | 369 | 2 | pseudomolecule_4:2492344..2498341 |
|  | BdTR13aMPK16 | Brdisv1BdTR13a1019440m | Bradi2g36470 | - | 1635 | 544 | 9 | pseudomolecule_2:31513845..31522350 |
|  | BdTR13aMPK17 | Brdisv1BdTR13a1005975m | Bradi1g34700 | + | 1746 | 581 | 10 | pseudomolecule_1:28501788..28509149 |
|  | BdTR13aMPK20-1 | Brdisv1BdTR13a1020921m | Bradi2g44350 | - | 1848 | 615 | 9 | pseudomolecule_2:40155410..40164483 |
|  | BdTR13aMPK20-2 | Brdisv1BdTR13a1016554m | Bradi2g15317 | + | 1827 | 608 | 9 | pseudomolecule_2:14535469..14543071 |
|  | BdTR13aMPK20-3 | Brdisv1BdTR13a1006843m | Bradi1g41780 | + | 1266 | 421 | 8 | pseudomolecule_1:34672979..34692380 |
|  | BdTR13aMPK20-4 | Brdisv1BdTR13a1021151m | Bradi2g45870 | + | 1743 | 580 | 9 | pseudomolecule_2:41630330..41638715 |
|  | BdTR13aMPK20-5 | Brdisv1BdTR13a1016712m | Bradi2g16337 | + | 1812 | 603 | 9 | pseudomolecule_2:15270090..15276779 |
|  | BdTR13aMPK21-1 | Brdisv1BdTR13a1016601m | Bradi2g15620 | - | 1788 | 595 | 10 | pseudomolecule_2:14722603..14730378 |
|  | BdTR13aMPK21-2 | Brdisv1BdTR13a1021039m | Bradi2g45006 | - | 1485 | 494 | 9 | pseudomolecule_2:40933313..40942873 |
| BdTR13c | BdTR13cMPK3 | Brdisv1BdTR13C1009620m | Bradi1g65810 | + | 1110 | 369 | 4 | pseudomolecule_1:41634449..41637990 |
|  | BdTR13cMPK4 | Brdisv1BdTR13C1010368m | Bradi3g32000 | - | 1134 | 377 | 7 | pseudomolecule_1:44782860..44788948 |
|  | BdTR13cMPK6 | Brdisv1BdTR13C1007022m | Bradi1g49100 | + | 942 | 313 | 4 | pseudomolecule_1:30299025..30304434 |
|  | BdTR13cMPK7-1 | Brdisv1BdTR13C1044721m | Bradi1g34030 | - | 687 | 228 | 0 | pseudomolecule_8:70376170..70377443 |
|  | BdTR13cMPK11 | Brdisv1BdTR13C1029714m | Bradi3g16560 | - | 1164 | 387 | 5 | pseudomolecule_4:10587246..10591961 |
|  | BdTR13cMPK14 | Brdisv1BdTR13C1027735m | Bradi3g03780 | - | 1110 | 369 | 2 | pseudomolecule_4:2136160..2142157 |
|  | BdTR13cMPK16 | Brdisv1BdTR13C1016526m | Bradi2g36470 | - | 1635 | 544 | 9 | pseudomolecule_2:21105776..21114281 |
|  | BdTR13cMPK17 | Brdisv1BdTR13C1005245m | Bradi1g34700 | + | 1746 | 581 | 10 | pseudomolecule_1:21474749..21482110 |
|  | BdTR13cMPK20-1 | Brdisv1BdTR13C1017760m | Bradi2g44350 | - | 1848 | 615 | 9 | pseudomolecule_2:26695629..26704702 |
|  | BdTR13cMPK20-2 | Brdisv1BdTR13C1014043m | Bradi2g15317 | + | 1827 | 608 | 9 | pseudomolecule_2:10142501..10150103 |
|  | BdTR13cMPK20-3 | Brdisv1BdTR13C1042291m | Bradi1g41780 | + | 624 | 208 | 3 | pseudomolecule_8:25071876..25078804 |
|  | BdTR13cMPK20-4 | Brdisv1BdTR13C1017954m | Bradi2g45870 | + | 1743 | 580 | 9 | pseudomolecule_2:27593244..27601629 |
|  | BdTR13cMPK20-5 | Brdisv1BdTR13C1014187m | Bradi2g16337 | + | 1812 | 603 | 9 | pseudomolecule_2:10741081..10747770 |
|  | BdTR13cMPK21-1 | Brdisv1BdTR13C1014087m | Bradi2g15620 | - | 1788 | 595 | 10 | pseudomolecule_2:10311154..10318977 |
|  | BdTR13cMPK21-2 | Brdisv1BdTR13C1017842m | Bradi2g45006 | - | 1485 | 494 | 9 | pseudomolecule_2:27037935..27047495 |
| Bis-1 | Bis-1MPK3 | Brdisv1Bis-11010343m | Bradi1g65810 | + | 1110 | 369 | 4 | pseudomolecule_1:46105116..46108657 |
|  | Bis-1MPK4 | Brdisv1Bis-11011144m | Bradi3g32000 | - | 1194 | 397 | 7 | pseudomolecule_1:49475655..49481459 |
|  | Bis-1MPK6 | Brdisv1Bis-11007534m | Bradi1g49100 | + | 1176 | 391 | 5 | pseudomolecule_1:33626153..33635148 |
|  | Bis-1MPK7-1 | Brdisv1Bis-11045356m | Bradi1g34030 | + | 690 | 229 | 1 | pseudomolecule_8:59788887..59790539 |
|  | Bis-1MPK11 | Brdisv1Bis-11031556m | Bradi3g16560 | - | 1164 | 387 | 5 | pseudomolecule_4:11528124..11532839 |
|  | Bis-1MPK14 | Brdisv1Bis-11029431m | Bradi3g03780 | - | 1110 | 369 | 2 | pseudomolecule_4:2327120..2333117 |
|  | Bis-1MPK16 | Brdisv1Bis-11017659m | Bradi2g36470 | - | 1635 | 544 | 9 | pseudomolecule_2:23180255..23188760 |
|  | Bis-1MPK17 | Brdisv1Bis-11005576m | Bradi1g34700 | + | 1746 | 581 | 10 | pseudomolecule_1:23692879..23700240 |
|  | Bis-1MPK20-1 | Brdisv1Bis-11018992m | Bradi2g44350 | + | 1848 | 615 | 9 | pseudomolecule_2:29537073..29546146 |
|  | Bis-1MPK20-2 | Brdisv1Bis-11015089m | Bradi2g15317 | + | 1827 | 608 | 9 | pseudomolecule_2:11283418..11291025 |
|  | Bis-1MPK20-3 | Brdisv1Bis-11043552m | Bradi1g41780 | + | 624 | 208 | 3 | pseudomolecule_8:17755798..17762726 |
|  | Bis-1MPK20-4 | Brdisv1Bis-11019216m | Bradi2g45870 | + | 1743 | 580 | 9 | pseudomolecule_2:30640661..30649046 |
|  | Bis-1MPK20-5 | Brdisv1Bis-11015242m | Bradi2g16337 | + | 1812 | 603 | 9 | pseudomolecule_2:11945890..11952579 |
|  | Bis-1MPK21-1 | Brdisv1Bis-11015134m | Bradi2g15620 | - | 1788 | 595 | 10 | pseudomolecule_2:11466562..11474337 |
|  | Bis-1MPK21-2 | Brdisv1Bis-11019081m | Bradi2g45006 | - | 1485 | 494 | 9 | pseudomolecule_2:29937251..29946811 |
| Koz-1 | Koz-1MPK3 | Brdisv1Koz-11010948m | Bradi1g65810 | + | 1110 | 369 | 4 | pseudomolecule_1:52950330..52953871 |
|  | Koz-1MPK4 | Brdisv1Koz-11035762m | Bradi3g32000 | + | 1134 | 377 | 7 | pseudomolecule_4:24154892..24160980 |
|  | Koz-1MPK6 | Brdisv1Koz-11007897m | Bradi1g49100 | + | 1176 | 391 | 5 | pseudomolecule_1:37973394..37982367 |
|  | Koz-1MPK7-1 | Brdisv1Koz-11046637m | Bradi1g34030 | - | 834 | 277 | 1 | pseudomolecule_8:42698224..42700056 |
|  | Koz-1MPK11 | Brdisv1Koz-11033685m | Bradi3g16560 | - | 1164 | 387 | 5 | pseudomolecule_4:12546413..12551128 |
|  | Koz-1MPK14 | Brdisv1Koz-11031538m | Bradi3g03780 | - | 1110 | 369 | 2 | pseudomolecule_4:2401403..2407400 |
|  | Koz-1MPK16 | Brdisv1Koz-11018826m | Bradi2g36470 | - | 1635 | 544 | 9 | pseudomolecule_2:28227746..28236251 |
|  | Koz-1MPK17 | Brdisv1Koz-11005826m | Bradi1g34700 | + | 1746 | 581 | 10 | pseudomolecule_1:26063470..26070834 |
|  | Koz-1MPK20-1 | Brdisv1Koz-11020254m | Bradi2g44350 | - | 1848 | 615 | 9 | pseudomolecule_2:35657820..35666893 |
|  | Koz-1MPK20-2 | Brdisv1Koz-11015944m | Bradi2g15317 | + | 1917 | 638 | 10 | pseudomolecule_2:12923993..12931004 |
|  | Koz-1MPK20-3 | Brdisv1Koz-11045898m | Bradi1g41780 | + | 624 | 208 | 3 | pseudomolecule_8:20439353..20446281 |
|  | Koz-1MPK20-4 | Brdisv1Koz-11025196m | Bradi2g45870 | - | 1743 | 580 | 9 | pseudomolecule_3:7630781..7639166 |
|  | Koz-1MPK20-5 | Brdisv1Koz-11016096m | Bradi2g16337 | + | 1812 | 603 | 9 | pseudomolecule_2:13618829..13625505 |
|  | Koz-1MPK21-1 | Brdisv1Koz-11015994m | Bradi2g15620 | - | 1788 | 595 | 10 | pseudomolecule_2:13121935..13129709 |
|  | Koz-1MPK21-2 | Brdisv1Koz-11020344m | Bradi2g45006 | - | 1485 | 494 | 9 | pseudomolecule_2:36151306..36160866 |
| Koz-3 | Koz-3MPK3 | Brdisv1Koz-31009389m | Bradi1g65810 | + | 1110 | 369 | 4 | pseudomolecule_1:42373442..42376983 |
|  | Koz-3MPK4 | Brdisv1Koz-31024105m | Bradi3g32000 | + | 1134 | 377 | 7 | pseudomolecule_3:17839127..17845519 |
|  | Koz-3MPK6 | Brdisv1Koz-31006774m | Bradi1g49100 | + | 1176 | 391 | 5 | pseudomolecule_1:30575421..30584366 |
|  | Koz-3MPK11 | Brdisv1Koz-31022574m | Bradi3g16560 | - | 1164 | 387 | 5 | pseudomolecule_3:10438688..10443403 |
|  | Koz-3MPK14 | Brdisv1Koz-31020739m | Bradi3g03780 | - | 1110 | 369 | 2 | pseudomolecule_3:2260783..2266734 |
|  | Koz-3MPK16 | Brdisv1Koz-31016040m | Bradi2g36470 | - | 1635 | 544 | 9 | pseudomolecule_2:20949198..20957695 |
|  | Koz-3MPK17 | Brdisv1Koz-31005240m | Bradi1g34700 | + | 1746 | 581 | 10 | pseudomolecule_1:22983454..22990818 |
|  | Koz-3MPK20-1 | Brdisv1Koz-31017201m | Bradi2g44350 | - | 1848 | 615 | 9 | pseudomolecule_2:26495719..26504792 |
|  | Koz-3MPK20-2 | Brdisv1Koz-31013656m | Bradi2g15317 | + | 1827 | 608 | 9 | pseudomolecule_2:10186441..10194043 |
|  | Koz-3MPK20-3 | Brdisv1Koz-31041190m | Bradi1g41780 | + | 624 | 208 | 3 | pseudomolecule_7:22326509..22333403 |
|  | Koz-3MPK20-4 | Brdisv1Koz-31017399m | Bradi2g45870 | + | 1743 | 580 | 9 | pseudomolecule_2:27574299..27582684 |
|  | Koz-3MPK20-5 | Brdisv1Koz-31013797m | Bradi2g16337 | + | 1812 | 603 | 9 | pseudomolecule_2:10787414..10794090 |
|  | Koz-3MPK21-1 | Brdisv1Koz-31013700m | Bradi2g15620 | - | 1788 | 595 | 10 | pseudomolecule_2:10359702..10367477 |
|  | Koz-3MPK21-2 | Brdisv1Koz-31017266m | Bradi2g45006 | - | 1485 | 494 | 9 | pseudomolecule_2:26818197..26827757 |
| BdTR3c | BdTR3cMPK3 | Brdisv1BdTR3C1011415m | Bradi1g65810 | + | 1110 | 369 | 4 | pseudomolecule_1:63278125..63281666 |
|  | BdTR3cMPK4 | Brdisv1BdTR3C1004133m | Bradi3g32000 | + | 1134 | 377 | 7 | pseudomolecule_1:20116984..20123072 |
|  | BdTR3cMPK6 | Brdisv1BdTR3C1008268m | Bradi1g49100 | + | 1176 | 391 | 5 | pseudomolecule_1:45771593..45780578 |
|  | BdTR3cMPK7-1 | Brdisv1BdTR3C1006021m | Bradi1g34030 | + | 1242 | 413 | 2 | pseudomolecule_1:30032362..30036381 |
|  | BdTR3cMPK7-2 | Brdisv1BdTR3C1006046m | Bradi4g24914 | + | 5127 | 1708 | 11 | pseudomolecule_1:30139896..30154926 |
|  | BdTR3cMPK11 | Brdisv1BdTR3C1026508m | Bradi3g16560 | - | 1164 | 387 | 5 | pseudomolecule_3:15215541..15220256 |
|  | BdTR3cMPK14 | Brdisv1BdTR3C1024843m | Bradi3g03780 | - | 1110 | 369 | 2 | pseudomolecule_3:2541825..2547822 |
|  | BdTR3cMPK16 | Brdisv1BdTR3C1019706m | Bradi2g36470 | - | 1635 | 544 | 9 | pseudomolecule_2:36445059..36453564 |
|  | BdTR3cMPK17 | Brdisv1BdTR3C1006170m | Bradi1g34700 | + | 1713 | 570 | 10 | pseudomolecule_1:30918236..30924155 |
|  | BdTR3cMPK20-1 | Brdisv1BdTR3C1021125m | Bradi2g44350 | - | 1848 | 615 | 9 | pseudomolecule_2:44354962..44364035 |
|  | BdTR3cMPK20-2 | Brdisv1BdTR3C1016467m | Bradi2g15317 | + | 1827 | 608 | 9 | pseudomolecule_2:14674495..14682101 |
|  | BdTR3cMPK20-3 | Brdisv1BdTR3C1007009m | Bradi1g41780 | + | 1266 | 421 | 8 | pseudomolecule_1:37466469..37485871 |
|  | BdTR3cMPK20-4 | Brdisv1BdTR3C1021364m | Bradi2g45870 | + | 1743 | 580 | 9 | pseudomolecule_2:45818839..45827224 |
|  | BdTR3cMPK20-5 | Brdisv1BdTR3C1016625m | Bradi2g16337 | + | 1812 | 603 | 9 | pseudomolecule_2:15473125..15479801 |
|  | BdTR3cMPK21-1 | Brdisv1BdTR3C1016518m | Bradi2g15620 | - | 1788 | 595 | 10 | pseudomolecule_2:14869008..14876782 |
|  | BdTR3cMPK21-2 | Brdisv1BdTR3C1021213m | Bradi2g45006 | - | 1485 | 494 | 9 | pseudomolecule_2:44908565..44918125 |
| Gaz-8 | Gaz-8MPK3 | Brdisv1Gaz-81005117m | Bradi1g65810 | + | 873 | 290 | 4 | pseudomolecule_1:20487801..20490206 |
|  | Gaz-8MPK4 | Brdisv1Gaz-81017044m | Bradi3g32000 | + | 843 | 280 | 6 | pseudomolecule_4:9097423..9102653 |
|  | Gaz-8MPK6 | Brdisv1Gaz-81003701m | Bradi1g49100 | + | 915 | 304 | 4 | pseudomolecule_1:14783219..14789260 |
|  | Gaz-8MPK7-1 | Brdisv1Gaz-81013145m | Bradi1g34030 | + | 807 | 268 | 2 | pseudomolecule_3:8591549..8596197 |
|  | Gaz-8MPK11 | Brdisv1Gaz-81016151m | Bradi3g16560 | - | 1026 | 341 | 5 | pseudomolecule_4:5227203..5231127 |
|  | Gaz-8MPK14 | Brdisv1Gaz-81024368m | Bradi3g03780 | + | 1110 | 369 | 2 | pseudomolecule_8:21554290..21560301 |
|  | Gaz-8MPK16 | Brdisv1Gaz-81023301m | Bradi2g36470 | - | 1635 | 544 | 9 | pseudomolecule_8:14089925..14098450 |
|  | Gaz-8MPK17 | Brdisv1Gaz-81009612m | Bradi1g34700 | - | 1566 | 521 | 10 | pseudomolecule_2:13515221..13521774 |
|  | Gaz-8MPK20-1 | Brdisv1Gaz-81009550m | Bradi2g44350 | + | 1848 | 615 | 9 | pseudomolecule_2:13312651..13321724 |
|  | Gaz-8MPK20-2 | Brdisv1Gaz-81007554m | Bradi2g15317 | + | 1602 | 533 | 9 | pseudomolecule_2:5247009..5254275 |
|  | Gaz-8MPK20-3 | Brdisv1Gaz-81026459m | Bradi1g41780 | + | 624 | 208 | 3 | pseudomolecule_8:35942473..35948998 |
|  | Gaz-8MPK20-4 | Brdisv1Gaz-81009686m | Bradi2g45870 | + | 1743 | 580 | 9 | pseudomolecule_2:13771262..13778996 |
|  | Gaz-8MPK20-5 | Brdisv1Gaz-81007614m | Bradi2g16337 | + | 1812 | 603 | 9 | pseudomolecule_2:5560061..5566759 |
|  | Gaz-8MPK21-1 | Brdisv1Gaz-81009615m | Bradi2g15620 | - | 1497 | 498 | 10 | pseudomolecule_2:13526465..13533511 |
|  | Gaz-8MPK21-2 | Brdisv1Gaz-81023218m | Bradi2g45006 | + | 1533 | 510 | 9 | pseudomolecule_8:13597128..13606277 |
| BdTR1i | BdTR1iMPK3 | Brdisv1BdTR1i1011291m | Bradi1g65810 | + | 1110 | 369 | 4 | pseudomolecule_1:60417464..60421005 |
|  | BdTR1iMPK4 | Brdisv1BdTR1i1029278m | Bradi3g32000 | + | 1134 | 377 | 7 | pseudomolecule_3:29386777..29392865 |
|  | BdTR1iMPK6 | Brdisv1BdTR1i1008313m | Bradi1g49100 | + | 1176 | 391 | 5 | pseudomolecule_1:44282723..44291683 |
|  | BdTR1iMPK7-1 | Brdisv1BdTR1i1046789m | Bradi1g34030 | + | 843 | 280 | 1 | pseudomolecule_8:19687147..19690774 |
|  | BdTR1iMPK11 | Brdisv1BdTR1i1027104m | Bradi3g16560 | - | 1164 | 387 | 5 | pseudomolecule_3:14433864..14438579 |
|  | BdTR1iMPK14 | Brdisv1BdTR1i1024796m | Bradi3g03780 | - | 1110 | 369 | 2 | pseudomolecule_3:2701799..2707796 |
|  | BdTR1iMPK16 | Brdisv1BdTR1i1019190m | Bradi2g36470 | - | 1635 | 544 | 9 | pseudomolecule_2:31417569..31426074 |
|  | BdTR1iMPK17 | Brdisv1BdTR1i1006203m | Bradi1g34700 | + | 1746 | 581 | 10 | pseudomolecule_1:30087702..30095066 |
|  | BdTR1iMPK20-1 | Brdisv1BdTR1i1020772m | Bradi2g44350 | - | 1848 | 615 | 9 | pseudomolecule_2:40724872..40733945 |
|  | BdTR1iMPK20-2 | Brdisv1BdTR1i1016104m | Bradi2g15317 | + | 1827 | 608 | 9 | pseudomolecule_2:13192384..13199986 |
|  | BdTR1iMPK20-3 | Brdisv1BdTR1i1006964m | Bradi1g41780 | - | 651 | 216 | 3 | pseudomolecule_1:35790265..35797230 |
|  | BdTR1iMPK20-4 | Brdisv1BdTR1i1035579m | Bradi2g45870 | + | 1743 | 580 | 9 | pseudomolecule_4:8230487..8238872 |
|  | BdTR1iMPK20-5 | Brdisv1BdTR1i1016265m | Bradi2g16337 | + | 1812 | 603 | 9 | pseudomolecule_2:13918000..13924676 |
|  | BdTR1iMPK21-1 | Brdisv1BdTR1i1016152m | Bradi2g15620 | - | 1788 | 595 | 10 | pseudomolecule_2:13379301..13387076 |
|  | BdTR1iMPK21-2 | Brdisv1BdTR1i1020854m | Bradi2g45006 | - | 1485 | 494 | 9 | pseudomolecule_2:41207041..41216601 |
| BdTR2b | BdTR2bMPK3 | Brdisv1BdTR2B1010533m | Bradi1g65810 | + | 1110 | 369 | 4 | pseudomolecule_1:47186844..47190385 |
|  | BdTR2bMPK4 | Brdisv1BdTR2B1027249m | Bradi3g32000 | + | 1134 | 377 | 7 | pseudomolecule_3:21374558..21380646 |
|  | BdTR2bMPK6 | Brdisv1BdTR2B1007594m | Bradi1g49100 | + | 1176 | 391 | 5 | pseudomolecule_1:33843245..33852213 |
|  | BdTR2bMPK7-1 | Brdisv1BdTR2B1045837m | Bradi1g34030 | + | 687 | 228 | 0 | pseudomolecule_7:57013438..57014608 |
|  | BdTR2bMPK11 | Brdisv1BdTR2B1025399m | Bradi3g16560 | - | 1164 | 387 | 5 | pseudomolecule_3:12021079..12025794 |
|  | BdTR2bMPK14 | Brdisv1BdTR2B1023291m | Bradi3g03780 | - | 1110 | 369 | 2 | pseudomolecule_3:2414424..2420421 |
|  | BdTR2bMPK16 | Brdisv1BdTR2B1043300m | Bradi2g36470 | - | 1635 | 544 | 9 | pseudomolecule_7:7251812..7260317 |
|  | BdTR2bMPK17 | Brdisv1BdTR2B1005613m | Bradi1g34700 | + | 1746 | 581 | 10 | pseudomolecule_1:23737484..23744848 |
|  | BdTR2bMPK20-1 | Brdisv1BdTR2B1019350m | Bradi2g44350 | - | 1848 | 615 | 9 | pseudomolecule_2:30108468..30117541 |
|  | BdTR2bMPK20-2 | Brdisv1BdTR2B1015236m | Bradi2g15317 | + | 1827 | 608 | 9 | pseudomolecule_2:11355243..11362845 |
|  | BdTR2bMPK20-3 | Brdisv1BdTR2B1044368m | Bradi1g41780 | + | 624 | 208 | 3 | pseudomolecule_7:20254079..20261007 |
|  | BdTR2bMPK20-4 | Brdisv1BdTR2B1019571m | Bradi2g45870 | + | 1743 | 580 | 9 | pseudomolecule_2:31231661..31240046 |
|  | BdTR2bMPK20-5 | Brdisv1BdTR2B1015380m | Bradi2g16337 | + | 1812 | 603 | 9 | pseudomolecule_2:11984745..11991421 |
|  | BdTR2bMPK21-1 | Brdisv1BdTR2B1015282m | Bradi2g15620 | - | 1788 | 595 | 10 | pseudomolecule_2:11526861..11534636 |
|  | BdTR2bMPK21-2 | Brdisv1BdTR2B1019438m | Bradi2g45006 | - | 1485 | 494 | 9 | pseudomolecule_2:30533628..30543188 |
| BdTR2g | BdTR2gMPK3 | Brdisv1BdTR2G1009723m | Bradi1g65810 | + | 1110 | 369 | 4 | pseudomolecule_1:40617600..40621141 |
|  | BdTR2gMPK4 | Brdisv1BdTR2G1031654m | Bradi3g32000 | + | 1161 | 386 | 7 | pseudomolecule_4:17818067..17823792 |
|  | BdTR2gMPK6 | Brdisv1BdTR2G1007189m | Bradi1g49100 | + | 747 | 248 | 3 | pseudomolecule_1:29972962..29977454 |
|  | BdTR2gMPK7-1 | Brdisv1BdTR2G1044736m | Bradi1g34030 | - | 717 | 238 | 1 | pseudomolecule_8:62938893..62940637 |
|  | BdTR2gMPK11 | Brdisv1BdTR2G1029987m | Bradi3g16560 | - | 1164 | 387 | 5 | pseudomolecule_4:10440820..10445535 |
|  | BdTR2gMPK14 | Brdisv1BdTR2G1028030m | Bradi3g03780 | - | 1110 | 369 | 2 | pseudomolecule_4:2193574..2199571 |
|  | BdTR2gMPK16 | Brdisv1BdTR2G1016744m | Bradi2g36470 | - | 1635 | 544 | 9 | pseudomolecule_2:20778565..20787070 |
|  | BdTR2gMPK17 | Brdisv1BdTR2G1005338m | Bradi1g34700 | + | 1746 | 581 | 10 | pseudomolecule_1:21473422..21480786 |
|  | BdTR2gMPK20-1 | Brdisv1BdTR2G1018048m | Bradi2g44350 | - | 1848 | 615 | 9 | pseudomolecule_2:26405659..26414732 |
|  | BdTR2gMPK20-2 | Brdisv1BdTR2G1014220m | Bradi2g15317 | + | 1827 | 608 | 9 | pseudomolecule_2:10256617..10264219 |
|  | BdTR2gMPK20-3 | Brdisv1BdTR2G1042101m | Bradi1g41780 | + | 624 | 208 | 3 | pseudomolecule_8:20451444..20458372 |
|  | BdTR2gMPK20-4 | Brdisv1BdTR2G1018254m | Bradi2g45870 | + | 1743 | 580 | 9 | pseudomolecule_2:27388517..27396902 |
|  | BdTR2gMPK20-5 | Brdisv1BdTR2G1014352m | Bradi2g16337 | + | 1812 | 603 | 9 | pseudomolecule_2:10801013..10807689 |
|  | BdTR2gMPK21-1 | Brdisv1BdTR2G1014263m | Bradi2g15620 | - | 1788 | 595 | 10 | pseudomolecule_2:10419566..10427341 |
|  | BdTR2gMPK21-2 | Brdisv1BdTR2G1018131m | Bradi2g45006 | - | 1485 | 494 | 9 | pseudomolecule_2:26766379..26775939 |
| Bd18-1 | Bd18-1MPK3 | Brdisv1Bd18-11011176m | Bradi1g65810 | + | 1110 | 369 | 4 | pseudomolecule_1:61761641..61764531 |
|  | Bd18-1MPK4 | Brdisv1Bd18-11029432m | Bradi3g32000 | + | 1134 | 377 | 7 | pseudomolecule_3:34101215..34107303 |
|  | Bd18-1MPK6 | Brdisv1Bd18-11008300m | Bradi1g49100 | + | 1176 | 391 | 5 | pseudomolecule_1:45735914..45744866 |
|  | Bd18-1MPK7-1 | Brdisv1Bd18-11006146m | Bradi1g34030 | + | 1242 | 413 | 2 | pseudomolecule_1:30067400..30071258 |
|  | Bd18-1MPK7-2 | Brdisv1Bd18-11038744m | Bradi4g24914 | + | 5127 | 1708 | 11 | pseudomolecule_4:29307672..29322709 |
|  | Bd18-1MPK11 | Brdisv1Bd18-11027073m | Bradi3g16560 | - | 1164 | 387 | 5 | pseudomolecule_3:15328939..15333654 |
|  | Bd18-1MPK14 | Brdisv1Bd18-11024761m | Bradi3g03780 | - | 1110 | 369 | 2 | pseudomolecule_3:2584209..2590200 |
|  | Bd18-1MPK16 | Brdisv1Bd18-11019354m | Bradi2g36470 | - | 1635 | 544 | 8 | pseudomolecule_2:35734516..35741346 |
|  | Bd18-1MPK17 | Brdisv1Bd18-11006085m | Bradi1g34700 | - | 1746 | 581 | 10 | pseudomolecule_1:29614232..29621596 |
|  | Bd18-1MPK20-1 | Brdisv1Bd18-11020716m | Bradi2g44350 | - | 1848 | 615 | 9 | pseudomolecule_2:43608478..43617551 |
|  | Bd18-1MPK20-2 | Brdisv1Bd18-11016146m | Bradi2g15317 | + | 1827 | 608 | 9 | pseudomolecule_2:13955026..13962628 |
|  | Bd18-1MPK20-3 | Brdisv1Bd18-11006939m | Bradi1g41780 | + | 1266 | 421 | 8 | pseudomolecule_1:36580873..36600275 |
|  | Bd18-1MPK20-4 | Brdisv1Bd18-11020956m | Bradi2g45870 | + | 1743 | 580 | 9 | pseudomolecule_2:45066735..45075120 |
|  | Bd18-1MPK20-5 | Brdisv1Bd18-11016306m | Bradi2g16337 | + | 1812 | 603 | 9 | pseudomolecule_2:14746395..14753071 |
|  | Bd18-1MPK21-1 | Brdisv1Bd18-11016194m | Bradi2g15620 | - | 1788 | 595 | 10 | pseudomolecule_2:14142208..14149982 |
|  | Bd18-1MPK21-2 | Brdisv1Bd18-11020806m | Bradi2g45006 | - | 1485 | 494 | 9 | pseudomolecule_2:44157882..44167442 |
| S+ | | | | | | | | |
| Bd30-1 | Bd30-1MPK3 | Brdisv1Bd30-11010004m | Bradi1g65810 | + | 1110 | 369 | 4 | pseudomolecule_1:48125221..48128762 |
|  | Bd30-1MPK4 | Brdisv1Bd30-11025462m | Bradi3g32000 | + | 1134 | 377 | 7 | pseudomolecule_3:19241121..19247226 |
|  | Bd30-1MPK6 | Brdisv1Bd30-11007350m | Bradi1g49100 | + | 1176 | 391 | 5 | pseudomolecule_1:34917881..34926776 |
|  | Bd30-1MPK7-1 | Brdisv1Bd30-11005594m | Bradi1g34030 | + | 1242 | 413 | 2 | pseudomolecule_1:25095739..25099762 |
|  | Bd30-1MPK11 | Brdisv1Bd30-11023952m | Bradi3g16560 | - | 1164 | 387 | 5 | pseudomolecule_3:11365512..11370228 |
|  | Bd30-1MPK14 | Brdisv1Bd30-11022043m | Bradi3g03780 | - | 1110 | 369 | 2 | pseudomolecule_3:2312334..2318269 |
|  | Bd30-1MPK16 | Brdisv1Bd30-11016978m | Bradi2g36470 | - | 1635 | 544 | 9 | pseudomolecule_2:22964051..22972556 |
|  | Bd30-1MPK17 | Brdisv1Bd30-11005687m | Bradi1g34700 | + | 1746 | 581 | 10 | pseudomolecule_1:25556352..25563716 |
|  | Bd30-1MPK20-1 | Brdisv1Bd30-11018292m | Bradi2g44350 | - | 1848 | 615 | 9 | pseudomolecule_2:29643926..29652999 |
|  | Bd30-1MPK20-2 | Brdisv1Bd30-11014518m | Bradi2g15317 | + | 1827 | 608 | 9 | pseudomolecule_2:11087899..11095372 |
|  | Bd30-1MPK20-3 | Brdisv1Bd30-11006265m | Bradi1g41780 | + | 1266 | 421 | 8 | pseudomolecule_1:28925100..28945855 |
|  | Bd30-1MPK20-4 | Brdisv1Bd30-11018520m | Bradi2g45870 | + | 1701 | 566 | 9 | pseudomolecule_2:30898139..30906239 |
|  | Bd30-1MPK20-5 | Brdisv1Bd30-11008828m | Bradi2g16337 | - | 1812 | 603 | 9 | pseudomolecule_1:42153927..42160604 |
|  | Bd30-1MPK21-1 | Brdisv1Bd30-11014587m | Bradi2g15620 | - | 1785 | 594 | 10 | pseudomolecule_2:11391118..11398315 |
|  | Bd30-1MPK21-2 | Brdisv1Bd30-11018351m | Bradi2g45006 | - | 1233 | 410 | 7 | pseudomolecule_2:29971365..29980247 |
| ABR7 | ABR7MPK3 | Brdisv1ABR71010863m | Bradi1g65810 | + | 1110 | 369 | 4 | pseudomolecule_1:55177083..55180624 |
|  | ABR7MPK4 | Brdisv1ABR71035794m | Bradi3g32000 | + | 1134 | 377 | 7 | pseudomolecule_4:27095705..27101810 |
|  | ABR7MPK6 | Brdisv1ABR71007957m | Bradi1g49100 | + | 822 | 273 | 3 | pseudomolecule_1:40308930..40313415 |
|  | ABR7MPK11 | Brdisv1ABR71033630m | Bradi3g16560 | - | 1164 | 387 | 5 | pseudomolecule_4:13171974..13176690 |
|  | ABR7MPK14 | Brdisv1ABR71031394m | Bradi3g03780 | - | 1110 | 369 | 1 | pseudomolecule_4:2234150..2238131 |
|  | ABR7MPK16 | Brdisv1ABR71018675m | Bradi2g36470 | - | 1635 | 544 | 9 | pseudomolecule_2:29245022..29253527 |
|  | ABR7MPK17 | Brdisv1ABR71005821m | Bradi1g34700 | + | 1746 | 581 | 10 | pseudomolecule_1:26911075..26918439 |
|  | ABR7MPK20-1 | Brdisv1ABR71020178m | Bradi2g44350 | + | 1848 | 615 | 9 | pseudomolecule_2:37490181..37499254 |
|  | ABR7MPK20-2 | Brdisv1ABR71015792m | Bradi2g15317 | + | 1827 | 608 | 9 | pseudomolecule_2:13357908..13365537 |
|  | ABR7MPK20-3 | Brdisv1ABR71006668m | Bradi1g41780 | + | 1266 | 421 | 8 | pseudomolecule_1:32355894..32375316 |
|  | ABR7MPK20-4 | Brdisv1ABR71020433m | Bradi2g45870 | + | 1743 | 580 | 9 | pseudomolecule_2:39018443..39026828 |
|  | ABR7MPK20-5 | Brdisv1ABR71015944m | Bradi2g16337 | + | 1812 | 603 | 9 | pseudomolecule_2:14114309..14120986 |
|  | ABR7MPK21-1 | Brdisv1ABR71015840m | Bradi2g15620 | - | 1785 | 594 | 10 | pseudomolecule_2:13545264..13552461 |
|  | ABR7MPK21-2 | Brdisv1ABR71020266m | Bradi2g45006 | - | 1449 | 482 | 7 | pseudomolecule_2:38021823..38028431 |
| ABR2 | ABR2MPK3 | Brdisv1ABR21010920m | Bradi1g65810 | + | 1110 | 369 | 4 | pseudomolecule_1:54471016..54474557 |
|  | ABR2MPK4 | Brdisv1ABR21011722m | Bradi3g32000 | - | 1134 | 377 | 7 | pseudomolecule_1:57966608..57972709 |
|  | ABR2MPK6 | Brdisv1ABR21008022m | Bradi1g49100 | + | 1176 | 391 | 5 | pseudomolecule_1:39876209..39885313 |
|  | ABR2MPK11 | Brdisv1ABR21033560m | Bradi3g16560 | - | 1164 | 387 | 5 | pseudomolecule_4:12987500..12992215 |
|  | ABR2MPK14 | Brdisv1ABR21031419m | Bradi3g03780 | - | 1110 | 369 | 2 | pseudomolecule_4:2496533..2502502 |
|  | ABR2MPK16 | Brdisv1ABR21018581m | Bradi2g36470 | - | 1635 | 544 | 9 | pseudomolecule_2:28273948..28282453 |
|  | ABR2MPK17 | Brdisv1ABR21005927m | Bradi1g34700 | - | 1746 | 581 | 10 | pseudomolecule_2:37090746..37097375 |
|  | ABR2MPK20-1 | Brdisv1ABR21020048m | Bradi2g44350 | - | 1848 | 615 | 9 | pseudomolecule_2:36573348..36582421 |
|  | ABR2MPK20-2 | Brdisv1ABR21015746m | Bradi2g15317 | + | 1827 | 608 | 9 | pseudomolecule_2:12595111..12602677 |
|  | ABR2MPK20-3 | Brdisv1ABR21029756m | Bradi1g41780 | + | 1266 | 421 | 8 | pseudomolecule_3:36174122..36201001 |
|  | ABR2MPK20-4 | Brdisv1ABR21020278m | Bradi2g45870 | + | 1743 | 580 | 9 | pseudomolecule_2:37942756..37951141 |
|  | ABR2MPK20-5 | Brdisv1ABR21015895m | Bradi2g16337 | + | 1812 | 603 | 9 | pseudomolecule_2:13298612..13305289 |
|  | ABR2MPK21-1 | Brdisv1ABR21015793m | Bradi2g15620 | - | 1785 | 594 | 10 | pseudomolecule_2:12781658..12788855 |
|  | ABR2MPK21-2 | Brdisv1ABR21020133m | Bradi2g45006 | - | 1449 | 482 | 7 | pseudomolecule_2:37090746..37097375 |
| Mig3 | Mig3MPK3 | Brdisv1Mig31010710m | Bradi1g65810 | + | 1110 | 369 | 4 | pseudomolecule_1:51701490..51705031 |
|  | Mig3MPK4 | Brdisv1Mig31011481m | Bradi3g32000 | - | 1134 | 377 | 7 | pseudomolecule_1:55005471..55011576 |
|  | Mig3MPK6 | Brdisv1Mig31007919m | Bradi1g49100 | + | 942 | 313 | 4 | pseudomolecule_1:37886204..37891613 |
|  | Mig3MPK7-1 | Brdisv1Mig31046286m | Bradi1g34030 | + | 687 | 228 | 0 | pseudomolecule_8:51895752..51896894 |
|  | Mig3MPK11 | Brdisv1Mig31032921m | Bradi3g16560 | - | 1164 | 387 | 5 | pseudomolecule_4:12670496..12675212 |
|  | Mig3MPK14 | Brdisv1Mig31030748m | Bradi3g03780 | - | 1110 | 369 | 2 | pseudomolecule_4:2190788..2196750 |
|  | Mig3MPK16 | Brdisv1Mig31044690m | Bradi2g36470 | - | 1635 | 544 | 9 | pseudomolecule_8:19856539..19865043 |
|  | Mig3MPK17 | Brdisv1Mig31005860m | Bradi1g34700 | + | 1746 | 581 | 10 | pseudomolecule_1:25927686..25935050 |
|  | Mig3MPK20-1 | Brdisv1Mig31019799m | Bradi2g44350 | + | 1848 | 615 | 9 | pseudomolecule_2:34815769..34824842 |
|  | Mig3MPK20-2 | Brdisv1Mig31015536m | Bradi2g15317 | + | 1827 | 608 | 9 | pseudomolecule_2:12390640..12398200 |
|  | Mig3MPK20-3 | Brdisv1Mig31006628m | Bradi1g41780 | + | 1266 | 421 | 8 | pseudomolecule_1:30386171..30405573 |
|  | Mig3MPK20-4 | Brdisv1Mig31020024m | Bradi2g45870 | + | 1743 | 580 | 9 | pseudomolecule_2:35994866..36003246 |
|  | Mig3MPK20-5 | Brdisv1Mig31015671m | Bradi2g16337 | + | 1812 | 603 | 9 | pseudomolecule_2:12987234..12993911 |
|  | Mig3MPK21-1 | Brdisv1Mig31015571m | Bradi2g15620 | - | 1785 | 594 | 10 | pseudomolecule_2:12524221..12531418 |
|  | Mig3MPK21-2 | Brdisv1Mig31019888m | Bradi2g45006 | - | 1449 | 482 | 7 | pseudomolecule_2:35232700..35239329 |
| Uni2 | Uni2MPK3 | Brdisv1Uni21010201m | Bradi1g65810 | + | 1110 | 369 | 4 | pseudomolecule_1:46691428..46694969 |
|  | Uni2MPK4 | Brdisv1Uni21033435m | Bradi3g32000 | + | 1134 | 377 | 7 | pseudomolecule_4:20503076..20509177 |
|  | Uni2MPK6 | Brdisv1Uni21007433m | Bradi1g49100 | + | 1176 | 391 | 5 | pseudomolecule_1:34064480..34073462 |
|  | Uni2MPK7-1 | Brdisv1Uni21045875m | Bradi1g34030 | - | 687 | 228 | 0 | pseudomolecule_8:69397068..69398353 |
|  | Uni2MPK11 | Brdisv1Uni21031613m | Bradi3g16560 | - | 1164 | 387 | 5 | pseudomolecule_4:11149614..11154330 |
|  | Uni2MPK14 | Brdisv1Uni21029534m | Bradi3g03780 | - | 1110 | 369 | 2 | pseudomolecule_4:2020055..2026017 |
|  | Uni2MPK16 | Brdisv1Uni21017694m | Bradi2g36470 | - | 1635 | 544 | 9 | pseudomolecule_2:23970279..23978076 |
|  | Uni2MPK17 | Brdisv1Uni21005452m | Bradi1g34700 | + | 1746 | 581 | 10 | pseudomolecule_1:23584693..23592057 |
|  | Uni2MPK20-1 | Brdisv1Uni21019092m | Bradi2g44350 | + | 1848 | 615 | 9 | pseudomolecule_2:30537651..30546724 |
|  | Uni2MPK20-2 | Brdisv1Uni21014999m | Bradi2g15317 | + | 1917 | 638 | 10 | pseudomolecule_2:11554953..11561964 |
|  | Uni2MPK20-3 | Brdisv1Uni21044231m | Bradi1g41780 | + | 624 | 208 | 3 | pseudomolecule_8:27044155..27051083 |
|  | Uni2MPK20-4 | Brdisv1Uni21019338m | Bradi2g45870 | + | 1743 | 580 | 9 | pseudomolecule_2:31765759..31774136 |
|  | Uni2MPK20-5 | Brdisv1Uni21015139m | Bradi2g16337 | + | 1812 | 603 | 9 | pseudomolecule_2:12186293..12192957 |
|  | Uni2MPK21-1 | Brdisv1Uni21015046m | Bradi2g15620 | - | 1785 | 594 | 10 | pseudomolecule_2:11753554..11760751 |
|  | Uni2MPK21-2 | Brdisv1Uni21019188m | Bradi2g45006 | - | 1449 | 482 | 7 | pseudomolecule_2:30953071..30959700 |
| ABR4 | ABR4MPK3 | Brdisv1ABR41010880m | Bradi1g65810 | + | 1110 | 369 | 4 | pseudomolecule_1:53785501..53789042 |
|  | ABR4MPK4 | Brdisv1ABR41011661m | Bradi3g32000 | - | 1134 | 377 | 7 | pseudomolecule_1:57135240..57141355 |
|  | ABR4MPK6 | Brdisv1ABR41044656m | Bradi1g49100 | + | 942 | 313 | 4 | pseudomolecule_7:11023256..11028665 |
|  | ABR4MPK7-1 | Brdisv1ABR41046323m | Bradi1g34030 | + | 939 | 312 | 1 | pseudomolecule_7:46169826..46171569 |
|  | ABR4MPK11 | Brdisv1ABR41033293m | Bradi3g16560 | - | 1164 | 387 | 5 | pseudomolecule_4:13265150..13269866 |
|  | ABR4MPK14 | Brdisv1ABR41031114m | Bradi3g03780 | - | 1110 | 369 | 2 | pseudomolecule_4:2413497..2419459 |
|  | ABR4MPK16 | Brdisv1ABR41018512m | Bradi2g36470 | - | 1635 | 544 | 9 | pseudomolecule_2:27850925..27859430 |
|  | ABR4MPK17 | Brdisv1ABR41005759m | Bradi1g34700 | + | 1746 | 581 | 10 | pseudomolecule_1:26020825..26028189 |
|  | ABR4MPK20-1 | Brdisv1ABR41019969m | Bradi2g44350 | + | 1848 | 615 | 9 | pseudomolecule_2:36043368..36052441 |
|  | ABR4MPK20-2 | Brdisv1ABR41015659m | Bradi2g15317 | + | 1827 | 608 | 9 | pseudomolecule_2:12524016..12531568 |
|  | ABR4MPK20-3 | Brdisv1ABR41045163m | Bradi1g41780 | - | 651 | 216 | 3 | pseudomolecule_7:20106947..20113912 |
|  | ABR4MPK20-4 | Brdisv1ABR41020187m | Bradi2g45870 | + | 1743 | 580 | 9 | pseudomolecule_2:37190348..37198733 |
|  | ABR4MPK20-5 | Brdisv1ABR41015812m | Bradi2g16337 | + | 1812 | 603 | 9 | pseudomolecule_2:13274038..13280703 |
|  | ABR4MPK21-1 | Brdisv1ABR41015701m | Bradi2g15620 | - | 1785 | 594 | 10 | pseudomolecule_2:12691927..12699124 |
|  | ABR4MPK21-2 | Brdisv1ABR41020060m | Bradi2g45006 | - | 1233 | 410 | 7 | pseudomolecule_2:36463797..36472679 |
| S8iiC | S8iiCMPK3 | Brdisv1S8iiC1010629m | Bradi1g65810 | + | 1110 | 369 | 4 | pseudomolecule_1:50780061..50783602 |
|  | S8iiCMPK4 | Brdisv1S8iiC1027882m | Bradi3g32000 | + | 1134 | 377 | 7 | pseudomolecule_3:24299258..24305353 |
|  | S8iiCMPK6 | Brdisv1S8iiC1007741m | Bradi1g49100 | + | 1176 | 391 | 5 | pseudomolecule_1:36994226..37003358 |
|  | S8iiCMPK7-2 | Brdisv1S8iiC1036390m | Bradi4g24914 | + | 5127 | 1708 | 11 | pseudomolecule_4:19881352..19895967 |
|  | S8iiCMPK11 | Brdisv1S8iiC1025854m | Bradi3g16560 | - | 1164 | 387 | 5 | pseudomolecule_3:12950336..12955052 |
|  | S8iiCMPK14 | Brdisv1S8iiC1023720m | Bradi3g03780 | - | 1110 | 369 | 2 | pseudomolecule_3:2616622..2622563 |
|  | S8iiCMPK16 | Brdisv1S8iiC1018261m | Bradi2g36470 | - | 1635 | 544 | 9 | pseudomolecule_2:26396523..26405028 |
|  | S8iiCMPK17 | Brdisv1S8iiC1005709m | Bradi1g34700 | + | 1746 | 581 | 10 | pseudomolecule_1:25401221..25408585 |
|  | S8iiCMPK20-1 | Brdisv1S8iiC1019709m | Bradi2g44350 | - | 1848 | 615 | 9 | pseudomolecule_2:33761675..33770748 |
|  | S8iiCMPK20-2 | Brdisv1S8iiC1015496m | Bradi2g15317 | + | 1827 | 608 | 9 | pseudomolecule_2:12497022..12504574 |
|  | S8iiCMPK20-3 | Brdisv1S8iiC1044636m | Bradi1g41780 | - | 651 | 216 | 3 | pseudomolecule_7:17746488..17753453 |
|  | S8iiCMPK20-4 | Brdisv1S8iiC1019946m | Bradi2g45870 | + | 1743 | 580 | 9 | pseudomolecule_2:35003993..35011887 |
|  | S8iiCMPK20-5 | Brdisv1S8iiC1015656m | Bradi2g16337 | + | 1812 | 603 | 9 | pseudomolecule_2:13292933..13299598 |
|  | S8iiCMPK21-1 | Brdisv1S8iiC1015529m | Bradi2g15620 | - | 1785 | 594 | 10 | pseudomolecule_2:12624616..12631813 |
|  | S8iiCMPK21-2 | Brdisv1S8iiC1019794m | Bradi2g45006 | - | 1233 | 410 | 7 | pseudomolecule_2:34201043..34209925 |
| Jer1 | Jer1MPK3 | Brdisv1Jer11009941m | Bradi1g65810 | + | 1110 | 369 | 4 | pseudomolecule_1:44656793..44660334 |
|  | Jer1MPK4 | Brdisv1Jer11032692m | Bradi3g32000 | + | 1134 | 377 | 7 | pseudomolecule_4:20144327..20150431 |
|  | Jer1MPK6 | Brdisv1Jer11007231m | Bradi1g49100 | + | 1176 | 391 | 5 | pseudomolecule_1:32396744..32405726 |
|  | Jer1MPK11 | Brdisv1Jer11030890m | Bradi3g16560 | - | 1164 | 387 | 5 | pseudomolecule_4:10904609..10909325 |
|  | Jer1MPK14 | Brdisv1Jer11028958m | Bradi3g03780 | - | 1110 | 369 | 2 | pseudomolecule_4:2141723..2147692 |
|  | Jer1MPK16 | Brdisv1Jer11017067m | Bradi2g36470 | - | 1635 | 544 | 9 | pseudomolecule_2:23616271..23624776 |
|  | Jer1MPK17 | Brdisv1Jer11005346m | Bradi1g34700 | + | 1746 | 581 | 10 | pseudomolecule_1:22483293..22490657 |
|  | Jer1MPK20-1 | Brdisv1Jer11018464m | Bradi2g44350 | + | 1848 | 615 | 9 | pseudomolecule_2:30127273..30136346 |
|  | Jer1MPK20-2 | Brdisv1Jer11014497m | Bradi2g15317 | + | 1827 | 608 | 9 | pseudomolecule_2:11438287..11445774 |
|  | Jer1MPK20-3 | Brdisv1Jer11043553m | Bradi1g41780 | - | 651 | 216 | 3 | pseudomolecule_7:29391037..29398002 |
|  | Jer1MPK20-4 | Brdisv1Jer11018679m | Bradi2g45870 | + | 1743 | 580 | 9 | pseudomolecule_2:31179045..31186942 |
|  | Jer1MPK20-5 | Brdisv1Jer11014639m | Bradi2g16337 | + | 1812 | 603 | 9 | pseudomolecule_2:12049624..12056301 |
|  | Jer1MPK21-1 | Brdisv1Jer11014541m | Bradi2g15620 | - | 1785 | 594 | 10 | pseudomolecule_2:11609735..11616932 |
|  | Jer1MPK21-2 | Brdisv1Jer11018554m | Bradi2g45006 | - | 1449 | 482 | 7 | pseudomolecule_2:30539407..30546036 |
| Per1 | Per1MPK3 | Brdisv1Per11011066m | Bradi1g65810 | + | 1110 | 369 | 4 | pseudomolecule_1:55513485..55517026 |
|  | Per1MPK4 | Brdisv1Per11028526m | Bradi3g32000 | + | 1134 | 377 | 7 | pseudomolecule_3:26784732..26790833 |
|  | Per1MPK6 | Brdisv1Per11008140m | Bradi1g49100 | + | 1176 | 391 | 5 | pseudomolecule_1:40602052..40611023 |
|  | Per1MPK11 | Brdisv1Per11026363m | Bradi3g16560 | - | 1164 | 387 | 5 | pseudomolecule_3:13517787..13522502 |
|  | Per1MPK14 | Brdisv1Per11024087m | Bradi3g03780 | - | 1110 | 369 | 2 | pseudomolecule_3:2225283..2231337 |
|  | Per1MPK16 | Brdisv1Per11018807m | Bradi2g36470 | - | 1635 | 544 | 9 | pseudomolecule_2:28470085..28478589 |
|  | Per1MPK17 | Brdisv1Per11005829m | Bradi1g34700 | + | 1746 | 581 | 10 | pseudomolecule_1:26789611..26796980 |
|  | Per1MPK20-1 | Brdisv1Per11020227m | Bradi2g44350 | - | 1848 | 615 | 9 | pseudomolecule_2:36312103..36321176 |
|  | Per1MPK20-2 | Brdisv1Per11015923m | Bradi2g15317 | + | 1827 | 608 | 9 | pseudomolecule_2:13038775..13046312 |
|  | Per1MPK20-3 | Brdisv1Per11045486m | Bradi1g41780 | + | 624 | 208 | 3 | pseudomolecule_7:19159627..19166555 |
|  | Per1MPK20-4 | Brdisv1Per11020434m | Bradi2g45870 | + | 1743 | 580 | 9 | pseudomolecule_2:37487982..37496372 |
|  | Per1MPK20-5 | Brdisv1Per11016073m | Bradi2g16337 | + | 1812 | 603 | 9 | pseudomolecule_2:13755636..13762313 |
|  | Per1MPK21-1 | Brdisv1Per11015968m | Bradi2g15620 | - | 1785 | 594 | 10 | pseudomolecule_2:13218009..13225206 |
|  | Per1MPK21-2 | Brdisv1Per11006952m | Bradi2g45006 | + | 1449 | 482 | 7 | pseudomolecule_1:33709320..33715949 |
| ABR6 | ABR6MPK3 | Brdisv1ABR6_r1011083m | Bradi1g65810 | + | 1110 | 369 | 4 | pseudomolecule_1:60640038..60643579 |
|  | ABR6MPK4 | Brdisv1ABR6_r1036097m | Bradi3g32000 | + | 1134 | 377 | 7 | pseudomolecule_4:29339211..29345316 |
|  | ABR6MPK6 | Brdisv1ABR6_r1007995m | Bradi1g49100 | + | 1176 | 391 | 5 | pseudomolecule_1:43615240..43624222 |
|  | ABR6MPK7-1 | Brdisv1ABR6_r1045951m | Bradi1g34030 | + | 717 | 238 | 1 | pseudomolecule_8:23788625..23790933 |
|  | ABR6MPK11 | Brdisv1ABR6_r1033960m | Bradi3g16560 | - | 1164 | 387 | 5 | pseudomolecule_4:13796938..13801654 |
|  | ABR6MPK14 | Brdisv1ABR6_r1031745m | Bradi3g03780 | - | 1110 | 369 | 2 | pseudomolecule_4:2385546..2391508 |
|  | ABR6MPK16 | Brdisv1ABR6_r1018869m | Bradi2g36470 | - | 1635 | 544 | 9 | pseudomolecule_2:31353285..31361790 |
|  | ABR6MPK17 | Brdisv1ABR6_r1005816m | Bradi1g34700 | + | 1746 | 581 | 10 | pseudomolecule_1:28584304..28591717 |
|  | ABR6MPK20-1 | Brdisv1ABR6_r1020353m | Bradi2g44350 | - | 1848 | 615 | 9 | pseudomolecule_2:40513577..40522650 |
|  | ABR6MPK20-2 | Brdisv1ABR6_r1016022m | Bradi2g15317 | + | 1917 | 638 | 10 | pseudomolecule_2:13782241..13789253 |
|  | ABR6MPK20-3 | Brdisv1ABR6_r1006657m | Bradi1g41780 | + | 1266 | 421 | 8 | pseudomolecule_1:34577244..34594477 |
|  | ABR6MPK20-4 | Brdisv1ABR6_r1020616m | Bradi2g45870 | + | 1743 | 580 | 9 | pseudomolecule_2:42060966..42069353 |
|  | ABR6MPK20-5 | Brdisv1ABR6_r1016151m | Bradi2g16337 | + | 1812 | 603 | 9 | pseudomolecule_2:14449236..14455913 |
|  | ABR6MPK21-1 | Brdisv1ABR6_r1029174m | Bradi2g15620 | - | 1785 | 594 | 10 | pseudomolecule_3:36191339..36198536 |
|  | ABR6MPK21-2 | Brdisv1ABR6_r1020438m | Bradi2g45006 | - | 1233 | 410 | 7 | pseudomolecule_2:41035211..41044093 |
| Luc1 | Luc1MPK3 | Brdisv1Luc11010913m | Bradi1g65810 | + | 1110 | 369 | 4 | pseudomolecule_1:56046638..56050179 |
|  | Luc1MPK4 | Brdisv1Luc11028728m | Bradi3g32000 | + | 1134 | 377 | 7 | pseudomolecule_3:26960431..26966522 |
|  | Luc1MPK6 | Brdisv1Luc11008002m | Bradi1g49100 | + | 1176 | 391 | 5 | pseudomolecule_1:41149461..41158443 |
|  | Luc1MPK7-1 | Brdisv1Luc11046458m | Bradi1g34030 | + | 999 | 332 | 3 | pseudomolecule_8:32302505..32305309 |
|  | Luc1MPK11 | Brdisv1Luc11026612m | Bradi3g16560 | - | 1164 | 387 | 5 | pseudomolecule_3:13556498..13561214 |
|  | Luc1MPK14 | Brdisv1Luc11024410m | Bradi3g03780 | - | 1110 | 369 | 2 | pseudomolecule_3:2252421..2258397 |
|  | Luc1MPK16 | Brdisv1Luc11018823m | Bradi2g36470 | - | 1635 | 544 | 9 | pseudomolecule_2:30555358..30563863 |
|  | Luc1MPK17 | Brdisv1Luc11005901m | Bradi1g34700 | + | 1746 | 581 | 10 | pseudomolecule_1:27352459..27359828 |
|  | Luc1MPK20-1 | Brdisv1Luc11020448m | Bradi2g44350 | - | 1848 | 615 | 9 | pseudomolecule_2:39875938..39885011 |
|  | Luc1MPK20-2 | Brdisv1Luc11015867m | Bradi2g15317 | + | 1827 | 608 | 9 | pseudomolecule_2:13630400..13637868 |
|  | Luc1MPK20-3 | Brdisv1Luc11006705m | Bradi1g41780 | + | 1266 | 421 | 8 | pseudomolecule_1:33099182..33118653 |
|  | Luc1MPK20-4 | Brdisv1Luc11020695m | Bradi2g45870 | + | 1743 | 580 | 9 | pseudomolecule_2:41246781..41255166 |
|  | Luc1MPK20-5 | Brdisv1Luc11016023m | Bradi2g16337 | + | 1812 | 603 | 9 | pseudomolecule_2:14423715..14430392 |
|  | Luc1MPK21-1 | Brdisv1Luc11015913m | Bradi2g15620 | - | 1785 | 594 | 10 | pseudomolecule_2:13836429..13843626 |
|  | Luc1MPK21-2 | Brdisv1Luc11020537m | Bradi2g45006 | - | 1449 | 482 | 7 | pseudomolecule_2:40343588..40350217 |
| Mur1 | Mur1MPK3 | Brdisv1Mur11009882m | Bradi1g65810 | + | 1110 | 369 | 4 | pseudomolecule_1:43078784..43082325 |
|  | Mur1MPK4 | Brdisv1Mur11032140m | Bradi3g32000 | + | 1134 | 377 | 7 | pseudomolecule_4:18921563..18927668 |
|  | Mur1MPK7-2 | Brdisv1Mur11023342m | Bradi4g24914 | - | 5127 | 1708 | 11 | pseudomolecule_3:9181634..9196167 |
|  | Mur1MPK11 | Brdisv1Mur11030448m | Bradi3g16560 | - | 1164 | 387 | 5 | pseudomolecule_4:10587991..10592707 |
|  | Mur1MPK14 | Brdisv1Mur11028428m | Bradi3g03780 | - | 1110 | 369 | 2 | pseudomolecule_4:2042928..2048890 |
|  | Mur1MPK16 | Brdisv1Mur11016923m | Bradi2g36470 | - | 1635 | 544 | 9 | pseudomolecule_2:21760286..21768799 |
|  | Mur1MPK17 | Brdisv1Mur11005362m | Bradi1g34700 | + | 1746 | 581 | 10 | pseudomolecule_1:22018155..22025519 |
|  | Mur1MPK20-1 | Brdisv1Mur11018309m | Bradi2g44350 | - | 1848 | 615 | 9 | pseudomolecule_2:28187392..28196465 |
|  | Mur1MPK20-2 | Brdisv1Mur11014394m | Bradi2g15317 | + | 1827 | 608 | 9 | pseudomolecule_2:10584401..10591850 |
|  | Mur1MPK20-3 | Brdisv1Mur11006032m | Bradi1g41780 | + | 624 | 208 | 3 | pseudomolecule_1:25541832..25548614 |
|  | Mur1MPK20-4 | Brdisv1Mur11018511m | Bradi2g45870 | + | 1743 | 580 | 9 | pseudomolecule_2:29119879..29128264 |
|  | Mur1MPK20-5 | Brdisv1Mur11014538m | Bradi2g16337 | + | 1812 | 603 | 9 | pseudomolecule_2:11227241..11233918 |
|  | Mur1MPK21-1 | Brdisv1Mur11014435m | Bradi2g15620 | - | 1785 | 594 | 10 | pseudomolecule_2:10747037..10754234 |
|  | Mur1MPK21-2 | Brdisv1Mur11018391m | Bradi2g45006 | - | 1449 | 482 | 7 | pseudomolecule_2:28535098..28541727 |
| ABR3 | ABR3MPK3 | Brdisv1ABR31010870m | Bradi1g65810 | + | 1110 | 369 | 4 | pseudomolecule_1:54467266..54470807 |
|  | ABR3MPK4 | Brdisv1ABR31028286m | Bradi3g32000 | + | 1134 | 377 | 7 | pseudomolecule_3:25789352..25795447 |
|  | ABR3MPK6 | Brdisv1ABR31008023m | Bradi1g49100 | + | 942 | 313 | 4 | pseudomolecule_1:39815964..39821373 |
|  | ABR3MPK11 | Brdisv1ABR31026158m | Bradi3g16560 | - | 1164 | 387 | 5 | pseudomolecule_3:12627117..12631832 |
|  | ABR3MPK14 | Brdisv1ABR31024015m | Bradi3g03780 | - | 1110 | 369 | 2 | pseudomolecule_3:2191210..2197174 |
|  | ABR3MPK16 | Brdisv1ABR31018712m | Bradi2g36470 | - | 1635 | 544 | 9 | pseudomolecule_2:28935397..28943844 |
|  | ABR3MPK17 | Brdisv1ABR31005797m | Bradi1g34700 | + | 1746 | 581 | 10 | pseudomolecule_1:26409936..26417300 |
|  | ABR3MPK20-1 | Brdisv1ABR31020171m | Bradi2g44350 | - | 1848 | 615 | 9 | pseudomolecule_2:37131067..37140140 |
|  | ABR3MPK20-2 | Brdisv1ABR31015739m | Bradi2g15317 | + | 1827 | 608 | 9 | pseudomolecule_2:12975705..12983308 |
|  | ABR3MPK20-3 | Brdisv1ABR31006618m | Bradi1g41780 | + | 1266 | 421 | 8 | pseudomolecule_1:31470401..31489873 |
|  | ABR3MPK20-4 | Brdisv1ABR31020401m | Bradi2g45870 | + | 1743 | 580 | 9 | pseudomolecule_2:38421072..38429457 |
|  | ABR3MPK20-5 | Brdisv1ABR31015895m | Bradi2g16337 | + | 1812 | 603 | 9 | pseudomolecule_2:13766982..13773647 |
|  | ABR3MPK21-1 | Brdisv1ABR31015783m | Bradi2g15620 | - | 1785 | 594 | 10 | pseudomolecule_2:13162496..13169693 |
|  | ABR3MPK21-2 | Brdisv1ABR31020259m | Bradi2g45006 | - | 1449 | 482 | 7 | pseudomolecule_2:37666576..37673205 |
| ABR5 | ABR5MPK3 | Brdisv1ABR51010136m | Bradi1g65810 | + | 1110 | 369 | 4 | pseudomolecule_1:47562538..47566079 |
|  | ABR5MPK4 | Brdisv1ABR51033637m | Bradi3g32000 | + | 1134 | 377 | 7 | pseudomolecule_4:22709373..22715567 |
|  | ABR5MPK6 | Brdisv1ABR51007501m | Bradi1g49100 | + | 942 | 313 | 4 | pseudomolecule_1:34687579..34692988 |
|  | ABR5MPK7-1 | Brdisv1ABR51046822m | Bradi1g34030 | - | 672 | 223 | 1 | pseudomolecule_7:69079944..69081474 |
|  | ABR5MPK11 | Brdisv1ABR51031623m | Bradi3g16560 | - | 1164 | 387 | 5 | pseudomolecule_4:11284293..11289009 |
|  | ABR5MPK14 | Brdisv1ABR51029646m | Bradi3g03780 | - | 1110 | 369 | 2 | pseudomolecule_4:1967136..1973098 |
|  | ABR5MPK16 | Brdisv1ABR51017709m | Bradi2g36470 | - | 1635 | 544 | 9 | pseudomolecule_2:26481141..26489650 |
|  | ABR5MPK17 | Brdisv1ABR51014890m | Bradi1g34700 | - | 1746 | 581 | 10 | pseudomolecule_2:12303710..12310793 |
|  | ABR5MPK20-1 | Brdisv1ABR51019274m | Bradi2g44350 | - | 1848 | 615 | 9 | pseudomolecule_2:34173956..34183029 |
|  | ABR5MPK20-2 | Brdisv1ABR51045248m | Bradi2g15317 | - | 1827 | 608 | 9 | pseudomolecule_7:42721675..42729159 |
|  | ABR5MPK20-3 | Brdisv1ABR51006261m | Bradi1g41780 | + | 1266 | 421 | 8 | pseudomolecule_1:28029147..28048650 |
|  | ABR5MPK20-4 | Brdisv1ABR51019275m | Bradi2g45870 | + | 1743 | 580 | 9 | pseudomolecule_2:34190394..34198290 |
|  | ABR5MPK20-5 | Brdisv1ABR51014991m | Bradi2g16337 | + | 1812 | 603 | 9 | pseudomolecule_2:12822180..12828845 |
|  | ABR5MPK21-1 | Brdisv1ABR51014894m | Bradi2g15620 | - | 1785 | 594 | 10 | pseudomolecule_2:12317156..12324353 |
|  | ABR5MPK21-2 | Brdisv1ABR51019135m | Bradi2g45006 | - | 1449 | 482 | 7 | pseudomolecule_2:33484734..33491363 |
| RON2 | RON2MPK3 | Brdisv1RON21010983m | Bradi1g65810 | + | 1110 | 369 | 4 | pseudomolecule_1:59692242..59695783 |
|  | RON2MPK4 | Brdisv1RON21028798m | Bradi3g32000 | + | 1134 | 377 | 7 | pseudomolecule_3:29464591..29470692 |
|  | RON2MPK6 | Brdisv1RON21008110m | Bradi1g49100 | + | 1176 | 391 | 5 | pseudomolecule_1:43852898..43861865 |
|  | RON2MPK7-1 | Brdisv1RON21046379m | Bradi1g34030 | + | 810 | 269 | 2 | pseudomolecule_7:22308481..22309945 |
|  | RON2MPK11 | Brdisv1RON21026673m | Bradi3g16560 | - | 1164 | 387 | 5 | pseudomolecule_3:14818333..14823049 |
|  | RON2MPK14 | Brdisv1RON21024409m | Bradi3g03780 | - | 1110 | 369 | 2 | pseudomolecule_3:2393884..2399846 |
|  | RON2MPK16 | Brdisv1RON21018668m | Bradi2g36470 | - | 1635 | 544 | 9 | pseudomolecule_2:31870031..31878536 |
|  | RON2MPK17 | Brdisv1RON21005994m | Bradi1g34700 | + | 1746 | 581 | 10 | pseudomolecule_1:29638316..29645685 |
|  | RON2MPK20-1 | Brdisv1RON21020075m | Bradi2g44350 | - | 1848 | 615 | 9 | pseudomolecule_2:40449295..40458368 |
|  | RON2MPK20-2 | Brdisv1RON21015763m | Bradi2g15317 | + | 1917 | 638 | 10 | pseudomolecule_2:14236400..14243411 |
|  | RON2MPK20-3 | Brdisv1RON21006833m | Bradi1g41780 | + | 1266 | 421 | 8 | pseudomolecule_1:35622343..35641745 |
|  | RON2MPK20-4 | Brdisv1RON21020271m | Bradi2g45870 | + | 1743 | 580 | 9 | pseudomolecule_2:41674322..41682698 |
|  | RON2MPK20-5 | Brdisv1RON21015899m | Bradi2g16337 | + | 1812 | 603 | 9 | pseudomolecule_2:14946479..14953156 |
|  | RON2MPK21-1 | Brdisv1RON21015809m | Bradi2g15620 | - | 1785 | 594 | 10 | pseudomolecule_2:14423728..14430925 |
|  | RON2MPK21-2 | Brdisv1RON21020154m | Bradi2g45006 | - | 1434 | 477 | 8 | pseudomolecule_2:40913846..40922720 |
| Foz1 | Foz1MPK3 | Brdisv1Foz11010123m | Bradi1g65810 | + | 1110 | 369 | 4 | pseudomolecule_1:47207989..47211530 |
|  | Foz1MPK4 | Brdisv1Foz11026494m | Bradi3g32000 | + | 1134 | 377 | 7 | pseudomolecule_3:22410851..22416952 |
|  | Foz1MPK6 | Brdisv1Foz11007450m | Bradi1g49100 | + | 1080 | 359 | 5 | pseudomolecule_1:34599837..34607723 |
|  | Foz1MPK7-2 | Brdisv1Foz11033114m | Bradi4g24914 | + | 5127 | 1708 | 11 | pseudomolecule_4:10395134..10410161 |
|  | Foz1MPK11 | Brdisv1Foz11024517m | Bradi3g16560 | - | 1164 | 387 | 5 | pseudomolecule_3:11498688..11503403 |
|  | Foz1MPK14 | Brdisv1Foz11022432m | Bradi3g03780 | - | 1110 | 369 | 2 | pseudomolecule_3:1999590..2005568 |
|  | Foz1MPK16 | Brdisv1Foz11017343m | Bradi2g36470 | - | 1635 | 544 | 9 | pseudomolecule_2:24795209..24803714 |
|  | Foz1MPK17 | Brdisv1Foz11005555m | Bradi1g34700 | + | 1746 | 581 | 10 | pseudomolecule_1:24106465..24113829 |
|  | Foz1MPK20-1 | Brdisv1Foz11018713m | Bradi2g44350 | + | 1848 | 615 | 9 | pseudomolecule_2:31590360..31599433 |
|  | Foz1MPK20-2 | Brdisv1Foz11014653m | Bradi2g15317 | + | 1827 | 608 | 9 | pseudomolecule_2:11420004..11427493 |
|  | Foz1MPK20-3 | Brdisv1Foz11006282m | Bradi1g41780 | + | 624 | 208 | 8 | pseudomolecule_1:28170128..28176910 |
|  | Foz1MPK20-4 | Brdisv1Foz11018932m | Bradi2g45870 | + | 1743 | 580 | 9 | pseudomolecule_2:32710256..32718641 |
|  | Foz1MPK20-5 | Brdisv1Foz11014797m | Bradi2g16337 | + | 1812 | 603 | 9 | pseudomolecule_2:12072278..12078955 |
|  | Foz1MPK21-1 | Brdisv1Foz11018793m | Bradi2g15620 | - | 1785 | 594 | 10 | pseudomolecule_2:31982812..31990009 |
|  | Foz1MPK21-2 | Brdisv1Foz11018797m | Bradi2g45006 | - | 1485 | 494 | 9 | pseudomolecule_2:32006316..32015876 |
| Sig2 | Sig2MPK3 | Brdisv1Sig21010086m | Bradi1g65810 | + | 1110 | 369 | 4 | pseudomolecule_1:46751346..46754887 |
|  | Sig2MPK4 | Brdisv1Sig21032872m | Bradi3g32000 | + | 1134 | 377 | 7 | pseudomolecule_4:20797058..20803159 |
|  | Sig2MPK6 | Brdisv1Sig21007446m | Bradi1g49100 | + | 942 | 313 | 4 | pseudomolecule_1:34607047..34612456 |
|  | Sig2MPK11 | Brdisv1Sig21030965m | Bradi3g16560 | - | 1164 | 387 | 5 | pseudomolecule_4:10622236..10626951 |
|  | Sig2MPK14 | Brdisv1Sig21005335m | Bradi3g03780 | + | 1110 | 369 | 2 | pseudomolecule_1:22942380..22948057 |
|  | Sig2MPK16 | Brdisv1Sig21017372m | Bradi2g36470 | - | 1635 | 544 | 9 | pseudomolecule_2:24199266..24207771 |
|  | Sig2MPK17 | Brdisv1Sig21005462m | Bradi1g34700 | + | 1746 | 581 | 10 | pseudomolecule_1:23575590..23582954 |
|  | Sig2MPK20-1 | Brdisv1Sig21018578m | Bradi2g44350 | - | 1848 | 615 | 9 | pseudomolecule_2:30228069..30237142 |
|  | Sig2MPK20-2 | Brdisv1Sig21014736m | Bradi2g15317 | + | 1827 | 608 | 9 | pseudomolecule_2:11466276..11473764 |
|  | Sig2MPK20-3 | Brdisv1Sig21006196m | Bradi1g41780 | + | 1266 | 421 | 8 | pseudomolecule_1:27775362..27794764 |
|  | Sig2MPK20-4 | Brdisv1Sig21018795m | Bradi2g45870 | + | 1743 | 580 | 9 | pseudomolecule_2:31341817..31350202 |
|  | Sig2MPK20-5 | Brdisv1Sig21014862m | Bradi2g16337 | + | 1812 | 603 | 9 | pseudomolecule_2:12042542..12049219 |
|  | Sig2MPK21-1 | Brdisv1Sig21018673m | Bradi2g15620 | - | 1785 | 594 | 10 | pseudomolecule_2:30657662..30664859 |
|  | Sig2MPK21-2 | Brdisv1Sig21018669m | Bradi2g45006 | - | 1485 | 494 | 9 | pseudomolecule_2:30647639..30657199 |
