# Supplementary material for: Comparative analysis of MAPK and MKK gene families reveals differential evolutionary patterns in Brachypodium distachyon inbred lines
Source: PeerJ. 2021 Apr 6;9:e11238. doi: 10.7717/peerj.11238 (PMC8034371; doi:10.7717/peerj.11238)
Supplement: Supplemental Information 13 [file peerj-09-11238-s013.docx]

Table S2 Table showing nomenclatured gene name locus ID detailed genomic information *B. distachyon* inbred lines MKKs.

| Inbred Lines | Gene Name | Locus ID | Orthologous genes in Bd21 | Orientation | ORF | No. of a.a | No. of introns | | 5’-3’ Coordinate | |  |
| --- | --- | --- | --- | --- | --- | --- | --- | --- | --- | --- | --- |
| EDF+ | | | | | | | | | | |  |
| Arn1 | Arn1MKK1 | Brdisv1Arn11008382m | Bradi1g51000 | + | 1047 | 348 | 8 | | pseudomolecule_1:43639770..43645342 | |  |
|  | Arn1MKK3-1 | Brdisv1Arn11029799m | Bradi4g39490 | + | 1572 | 523 | 9 | | pseudomolecule_3:36838773..36846773 | |  |
|  | Arn1MKK3-2 | Brdisv1Arn11045189m | Bradi1g41860 | - | 1572 | 523 | 9 | | pseudomolecule_8:6389562..6405170 | |  |
|  | Arn1MKK3-3 | Brdisv1Arn11032740m | Bradi3g11260 | - | 1779 | 592 | 9 | | pseudomolecule_4:8695377..8704945 | |  |
|  | Arn1MKK4 | Brdisv1Arn11039595m | Bradi3g53650 | + | 1074 | 357 | 0 | | pseudomolecule_4:45840852..45843376 | |  |
|  | Arn1MKK5 | Brdisv1Arn11007617m | Bradi1g46880 | - | 1038 | 345 | 1 | | pseudomolecule_1:39847696..39851797 | |  |
|  | Arn1MKK6 | Brdisv1Arn11012644m | Bradi1g75150 | + | 1071 | 356 | 7 | | pseudomolecule_1:64709887..64717315 | |  |
|  | Arn1MKK10-1 | Brdisv1Arn11001761m | Bradi1g11525 | - | 1044 | 347 | 0 | | pseudomolecule_1:7832777..7834238 | |  |
|  | Arn1MKK10-2 | Brdisv1Arn11011731m | Bradi1g69400 | + | 1029 | 342 | 0 | | pseudomolecule_1:60934374..60936234 | |  |
|  | Arn1MKK10-3 | Brdisv1Arn11001664m | Bradi1g10800 | - | 1035 | 344 | 0 | | pseudomolecule_1:7200312..7201760 | |  |
|  | Arn1MKK10-4 | Brdisv1Arn11001661m | Bradi1g10770 | + | 1026 | 341 | 0 | | pseudomolecule_1:7169402..7170837 | |  |
|  | Arn1MKK10-5 | Brdisv1Arn11001663m | Bradi1g10790 | - | 999 | 332 | 0 | | pseudomolecule_1:7194540..7196669 | |  |
| Mon3 | Mon3MKK1 | Brdisv1Mon31007821m | Bradi1g51000 | + | 1047 | 348 | 8 | | pseudomolecule_1:37613592..37619164 | |  |
|  | Mon3MKK3-1 | Brdisv1Mon31028015m | Bradi4g39490 | + | 1572 | 523 | 9 | | pseudomolecule_3:31807563..31815563 | |  |
|  | Mon3MKK3-2 | Brdisv1Mon31041467m | Bradi1g41860 | - | 1572 | 523 | 9 | | pseudomolecule_8:6258504..6274112 | |  |
|  | Mon3MKK3-3 | Brdisv1Mon31030882m | Bradi3g11260 | - | 1779 | 592 | 9 | | pseudomolecule_4:8420252..8429820 | |  |
|  | Mon3MKK4 | Brdisv1Mon31036999m | Bradi3g53650 | + | 1074 | 357 | 0 | | pseudomolecule_4:39310935..39313454 | |  |
|  | Mon3MKK5 | Brdisv1Mon31007144m | Bradi1g46880 | - | 1038 | 345 | 1 | | pseudomolecule_1:34555528..34559629 | |  |
|  | Mon3MKK6 | Brdisv1Mon31011751m | Bradi1g75150 | + | 1071 | 356 | 7 | | pseudomolecule_1:55573721..55581149 | |  |
|  | Mon3MKK10-1 | Brdisv1Mon31001734m | Bradi1g11525 | - | 1044 | 347 | 0 | | pseudomolecule_1:7498008..7499469 | |  |
|  | Mon3MKK10-2 | Brdisv1Mon31010898m | Bradi1g69400 | + | 1029 | 342 | 0 | | pseudomolecule_1:52130166..52132026 | |  |
|  | Mon3MKK10-3 | Brdisv1Mon31042680m | Bradi1g10800 | - | 1035 | 344 | 0 | | pseudomolecule_8:24325893..24327341 | |  |
|  | Mon3MKK10-4 | Brdisv1Mon31001638m | Bradi1g10770 | + | 1026 | 341 | 0 | | pseudomolecule_1:6943656..6945091 | |  |
|  | Mon3MKK10-5 | Brdisv1Mon31043288m | Bradi1g10790 | + | 999 | 332 | 0 | | pseudomolecule_8:34019384..34021442 | |  |
| Bd1-1 | Bd1-1MKK1 | Brdisv1Bd1-11007340m | Bradi1g51000 | + | 1047 | 348 | 8 | | pseudomolecule_1:33438309..33443874 | |  |
|  | Bd1-1MKK3-1 | Brdisv1Bd1-11034555m | Bradi4g39490 | + | 1572 | 523 | 9 | | pseudomolecule_4:25125133..25133133 | |  |
|  | Bd1-1MKK3-2 | Brdisv1Bd1-11040480m | Bradi1g41860 | - | 1572 | 523 | 10 | | pseudomolecule_6:11267336..11283825 | |  |
|  | Bd1-1MKK3-3 | Brdisv1Bd1-11022429m | Bradi3g11260 | - | 1779 | 592 | 9 | | pseudomolecule_3:7807458..7817025 | |  |
|  | Bd1-1MKK4 | Brdisv1Bd1-11028128m | Bradi3g53650 | + | 1074 | 357 | 0 | | pseudomolecule_3:34197203..34199722 | |  |
|  | Bd1-1MKK5 | Brdisv1Bd1-11006672m | Bradi1g46880 | - | 768 | 255 | 1 | | pseudomolecule_1:30699586..30703309 | |  |
|  | Bd1-1MKK6 | Brdisv1Bd1-11011292m | Bradi1g75150 | + | 1071 | 356 | 7 | | pseudomolecule_1:51497281..51504688 | |  |
|  | Bd1-1MKK10-1 | Brdisv1Bd1-11001714m | Bradi1g11525 | + | 1044 | 347 | 0 | | pseudomolecule_1:7498905..7500366 | |  |
|  | Bd1-1MKK10-2 | Brdisv1Bd1-11010351m | Bradi1g69400 | + | 1029 | 342 | 0 | | pseudomolecule_1:47667016..47668876 | |  |
|  | Bd1-1MKK10-3 | Brdisv1Bd1-11001621m | Bradi1g10800 | + | 1035 | 344 | 0 | | pseudomolecule_1:6994373..6995821 | |  |
|  | Bd1-1MKK10-4 | Brdisv1Bd1-11001619m | Bradi1g10770 | + | 1026 | 341 | 0 | | pseudomolecule_1:6975054..6976489 | |  |
|  | Bd1-1MKK10-5 | Brdisv1Bd1-11041771m | Bradi1g10790 | - | 981 | 326 | 0 | | pseudomolecule_6:19795096..19795313 | |  |
| ABR9 | ABR9MKK1 | Brdisv1ABR9_r1008255m | Bradi1g51000 | + | 1047 | 348 | 8 | | pseudomolecule_1:43811322..43816893 | |  |
|  | ABR9MKK3-1 | Brdisv1ABR9_r1023443m | Bradi4g39490 | + | 1572 | 523 | 8 | | pseudomolecule_3:37815469..37821109 | |  |
|  | ABR9MKK3-2 | Brdisv1ABR9_r1034151m | Bradi1g41860 | - | 1572 | 523 | 10 | | pseudomolecule_7:3304600..3321089 | |  |
|  | ABR9MKK3-3 | Brdisv1ABR9_r1025291m | Bradi3g11260 | - | 1551 | 516 | 8 | | pseudomolecule_4:9449428..9457476 | |  |
|  | ABR9MKK4 | Brdisv1ABR9_r1029250m | Bradi3g53650 | + | 1074 | 357 | 0 | | pseudomolecule_4:45498080..45499583 | |  |
|  | ABR9MKK5 | Brdisv1ABR9_r1007521m | Bradi1g46880 | - | 1032 | 343 | 1 | | pseudomolecule_1:39962017..39966111 | |  |
|  | ABR9MKK6 | Brdisv1ABR9_r1012651m | Bradi1g75150 | + | 1071 | 356 | 7 | | pseudomolecule_1:65466967..65474407 | |  |
|  | ABR9MKK10-1 | Brdisv1ABR9_r1001822m | Bradi1g11525 | - | 1044 | 347 | 0 | | pseudomolecule_1:8096984..8098445 | |  |
|  | ABR9MKK10-2 | Brdisv1ABR9_r1011727m | Bradi1g69400 | + | 1029 | 342 | 0 | | pseudomolecule_1:61726238..61728098 | |  |
|  | ABR9MKK10-3 | Brdisv1ABR9_r1001726m | Bradi1g10800 | + | 1035 | 344 | 0 | | pseudomolecule_1:7511583..7513031 | |  |
|  | ABR9MKK10-4 | Brdisv1ABR9_r1001722m | Bradi1g10770 | + | 1026 | 341 | 0 | | pseudomolecule_1:7443796..7445231 | |  |
|  | ABR9MKK10-5 | Brdisv1ABR9_r1001725m | Bradi1g10790 | - | 999 | 332 | 1 | | pseudomolecule_1:7472578..7477190 | |  |
| Bd29-1 | Bd29-1MKK1 | Brdisv1Bd29-11006851m | Bradi1g51000 | + | 1047 | 348 | 8 | | pseudomolecule_1:29183009..29188574 | |  |
|  | Bd29-1MKK3-1 | Brdisv1Bd29-11024808m | Bradi4g39490 | + | 1572 | 523 | 9 | | pseudomolecule_3:23513265..23521265 | |  |
|  | Bd29-1MKK3-2 | Brdisv1Bd29-11037603m | Bradi1g41860 | - | 1572 | 523 | 10 | | pseudomolecule_8:4349276..4365699 | |  |
|  | Bd29-1MKK3-3 | Brdisv1Bd29-11005448m | Bradi3g11260 | - | 1338 | 445 | 6 | | pseudomolecule_1:22666575..22672313 | |  |
|  | Bd29-1MKK4 | Brdisv1Bd29-11032652m | Bradi3g53650 | + | 426 | 141 | 1 | | pseudomolecule_4:30470176..30471405 | |  |
|  | Bd29-1MKK5 | Brdisv1Bd29-11006252m | Bradi1g46880 | - | 1032 | 343 | 1 | | pseudomolecule_1:26702968..26707062 | |  |
|  | Bd29-1MKK6 | Brdisv1Bd29-11010393m | Bradi1g75150 | + | 1071 | 356 | 7 | | pseudomolecule_1:44073653..44081060 | |  |
|  | Bd29-1MKK10-1 | Brdisv1Bd29-11001549m | Bradi1g11525 | - | 1044 | 347 | 0 | | pseudomolecule_1:6238190..6239651 | |  |
|  | Bd29-1MKK10-2 | Brdisv1Bd29-11009613m | Bradi1g69400 | + | 1029 | 342 | 0 | | pseudomolecule_1:40999697..41001557 | |  |
|  | Bd29-1MKK10-3 | Brdisv1Bd29-11001467m | Bradi1g10800 | + | 1035 | 344 | 0 | | pseudomolecule_1:5818679..5820127 | |  |
|  | Bd29-1MKK10-4 | Brdisv1Bd29-11001465m | Bradi1g10770 | + | 1026 | 341 | 0 | | pseudomolecule_1:5798907..5800342 | |  |
|  | Bd29-1MKK10-5 | Brdisv1Bd29-11016615m | Bradi1g10790 | - | 999 | 332 | 0 | | pseudomolecule_2:24658894..24661023 | |  |
| Tek-4 | Tek-4MKK1 | Brdisv1Tek-41004208m | Bradi1g51000 | + | 1047 | 348 | 8 | | pseudomolecule_1:14196598..14201622 | |  |
|  | Tek-4MKK3-1 | Brdisv1Tek-41038463m | Bradi4g39490 | + | 561 | 186 | 4 | | pseudomolecule_8:83792394..83794870 | |  |
|  | Tek-4MKK3-2 | Brdisv1Tek-41026595m | Bradi1g41860 | + | 1023 | 340 | 6 | | pseudomolecule_8:18271693..18281442 | |  |
|  | Tek-4MKK3-3 | Brdisv1Tek-41017143m | Bradi3g11260 | - | 693 | 230 | 4 | | pseudomolecule_4:3794568..3799188 | |  |
|  | Tek-4MKK4 | Brdisv1Tek-41001069m | Bradi3g53650 | + | 1074 | 357 | 0 | | pseudomolecule_1:3292037..3294556 | |  |
|  | Tek-4MKK5 | Brdisv1Tek-41003815m | Bradi1g46880 | - | 984 | 327 | 0 | | pseudomolecule_1:12941007..12942379 | |  |
|  | Tek-4MKK6 | Brdisv1Tek-41006374m | Bradi1g75150 | + | 1071 | 356 | 7 | | pseudomolecule_1:21569749..21577156 | |  |
|  | Tek-4MKK10-1 | Brdisv1Tek-41001123m | Bradi1g11525 | - | 1044 | 347 | 0 | | pseudomolecule_1:3526986..3528447 | |  |
|  | Tek-4MKK10-2 | Brdisv1Tek-41001073m | Bradi1g69400 | + | 579 | 192 | 0 | | pseudomolecule_1:3309057..3310517 | |  |
|  | Tek-4MKK10-3 | Brdisv1Tek-41001076m | Bradi1g10800 | - | 1035 | 344 | 0 | | pseudomolecule_1:3317630..3319078 | |  |
|  | Tek-4MKK10-4 | Brdisv1Tek-41001074m | Bradi1g10770 | + | 891 | 296 | 2 | | pseudomolecule_1:3311753..3315957 | |  |
| BdTR7a | BdTR7aMKK1 | Brdisv1BdTR7a1008412m | Bradi1g51000 | + | 1047 | 348 | 8 | | pseudomolecule_1:41996239..42001804 | |  |
|  | BdTR7aMKK3-1 | Brdisv1BdTR7a1029605m | Bradi4g39490 | + | 1572 | 523 | 9 | | pseudomolecule_3:33554503..33562503 | |  |
|  | BdTR7aMKK3-2 | Brdisv1BdTR7a1006778m | Bradi1g41860 | - | 1572 | 523 | 10 | | pseudomolecule_1:32918697..32935186 | |  |
|  | BdTR7aMKK3-3 | Brdisv1BdTR7a1032485m | Bradi3g11260 | - | 1779 | 592 | 9 | | pseudomolecule_4:8437921..8447490 | |  |
|  | BdTR7aMKK4 | Brdisv1BdTR7a1006916m | Bradi3g53650 | + | 1074 | 357 | 0 | | pseudomolecule_1:33843911..33846430 | |  |
|  | BdTR7aMKK5 | Brdisv1BdTR7a1007645m | Bradi1g46880 | - | 1032 | 343 | 1 | | pseudomolecule_1:38304271..38308365 | |  |
|  | BdTR7aMKK6 | Brdisv1BdTR7a1012554m | Bradi1g75150 | + | 1071 | 356 | 7 | | pseudomolecule_1:62224402..62232099 | |  |
|  | BdTR7aMKK10-1 | Brdisv1BdTR7a1001914m | Bradi1g11525 | - | 1044 | 347 | 0 | | pseudomolecule_1:8586893..8588354 | |  |
|  | BdTR7aMKK10-2 | Brdisv1BdTR7a1011719m | Bradi1g69400 | + | 1029 | 342 | 0 | | pseudomolecule_1:58844924..58846784 | |  |
|  | BdTR7aMKK10-3 | Brdisv1BdTR7a1001789m | Bradi1g10800 | + | 1035 | 344 | 0 | | pseudomolecule_1:7925580..7927028 | |  |
|  | BdTR7aMKK10-4 | Brdisv1BdTR7a1001786m | Bradi1g10770 | + | 1026 | 341 | 0 | | pseudomolecule_1:7881168..7882603 | |  |
|  | BdTR7aMKK10-5 | Brdisv1BdTR7a1001788m | Bradi1g10790 | - | 999 | 332 | 0 | | pseudomolecule_1:7906358..7908487 | |  |
| Tek-2 | Tek-2MKK1 | Brdisv1Tek-21007638m | Bradi1g51000 | + | 1047 | 348 | 8 | | pseudomolecule_1:32859230..32864795 | |  |
|  | Tek-2MKK3-1 | Brdisv1Tek-21027306m | Bradi4g39490 | + | 1572 | 523 | 9 | | pseudomolecule_3:25474896..25482898 | |  |
|  | Tek-2MKK3-2 | Brdisv1Tek-21041684m | Bradi1g41860 | - | 1572 | 523 | 10 | | pseudomolecule_7:5736133..5752622 | |  |
|  | Tek-2MKK3-3 | Brdisv1Tek-21030208m | Bradi3g11260 | - | 1779 | 592 | 9 | | pseudomolecule_4:7950281..7959816 | |  |
|  | Tek-2MKK4 | Brdisv1Tek-21036177m | Bradi3g53650 | + | 1074 | 357 | 0 | | pseudomolecule_4:33777076..33779595 | |  |
|  | Tek-2MKK5 | Brdisv1Tek-21006949m | Bradi1g46880 | - | 1032 | 343 | | 1 | | pseudomolecule_1:30073004..30077097 | |
|  | Tek-2MKK6 | Brdisv1Tek-21011633m | Bradi1g75150 | + | 1071 | 356 | 7 | | pseudomolecule_1:49940288..49947694 | |  |
|  | Tek-2MKK10-1 | Brdisv1Tek-21001708m | Bradi1g11525 | - | 1044 | 347 | 0 | | pseudomolecule_1:7131222..7132683 | |  |
|  | Tek-2MKK10-2 | Brdisv1Tek-21010712m | Bradi1g69400 | + | 1029 | 342 | 0 | | pseudomolecule_1:46321447..46323307 | |  |
|  | Tek-2MKK10-3 | Brdisv1Tek-21001622m | Bradi1g10800 | + | 1035 | 344 | 0 | | pseudomolecule_1:6651456..6652904 | |  |
|  | Tek-2MKK10-4 | Brdisv1Tek-21001619m | Bradi1g10770 | + | 1026 | 341 | 0 | | pseudomolecule_1:6629311..6630746 | |  |
|  | Tek-2MKK10-5 | Brdisv1Tek-21001621m | Bradi1g10790 | - | 999 | 332 | 0 | | pseudomolecule_1:6640156..6642285 | |  |
| BdTR8i | BdTR8iMKK1 | Brdisv1BdTR8i1007987m | Bradi1g51000 | + | 1047 | 348 | 8 | | pseudomolecule_1:41272898..41278463 | |  |
|  | BdTR8iMKK3-1 | Brdisv1BdTR8i1037957m | Bradi4g39490 | + | 1572 | 523 | 9 | | pseudomolecule_4:33731461..33739463 | |  |
|  | BdTR8iMKK3-2 | Brdisv1BdTR8i1006485m | Bradi1g41860 | - | 1572 | 523 | 10 | | pseudomolecule_1:32702700..32719162 | |  |
|  | BdTR8iMKK3-3 | Brdisv1BdTR8i1024401m | Bradi3g11260 | - | 1779 | 592 | 9 | | pseudomolecule_3:8647449..8656984 | |  |
|  | BdTR8iMKK4 | Brdisv1BdTR8i1030790m | Bradi3g53650 | + | 1074 | 357 | 0 | | pseudomolecule_3:43200609..43203128 | |  |
|  | BdTR8iMKK5 | Brdisv1BdTR8i1007289m | Bradi1g46880 | - | 1032 | 343 | 1 | | pseudomolecule_1:37746825..37750918 | |  |
|  | BdTR8iMKK6 | Brdisv1BdTR8i1012248m | Bradi1g75150 | + | 1071 | 356 | 7 | | pseudomolecule_1:62324411..62331817 | |  |
|  | BdTR8iMKK10-1 | Brdisv1BdTR8i1001797m | Bradi1g11525 | - | 1044 | 347 | 0 | | pseudomolecule_1:7951091..7952552 | |  |
|  | BdTR8iMKK10-2 | Brdisv1BdTR8i1011347m | Bradi1g69400 | + | 1029 | 342 | 0 | | pseudomolecule_1:58581341..58583201 | |  |
|  | BdTR8iMKK10-3 | Brdisv1BdTR8i1001692m | Bradi1g10800 | + | 1035 | 344 | 0 | | pseudomolecule_1:7357413..7358861 | |  |
|  | BdTR8iMKK10-4 | Brdisv1BdTR8i1001690m | Bradi1g10770 | + | 1026 | 341 | 0 | | pseudomolecule_1:7308136..7309571 | |  |
|  | BdTR8iMKK10-5 | Brdisv1BdTR8i1044418m | Bradi1g10790 | + | 999 | 332 | 0 | | pseudomolecule_6:25945369..25947498 | |  |
| T+ | | | | | | | | | | |  |
| Bd21 | BdMKK1 | Bradi1g51000 | - | - | 1047 | 348 | 8 | | 49443017 - 49446304 | |  |
|  | BdMKK3-1 | Bradi4g39490 | - | - | 1572 | 523 | 9 | | 44107411 - 44111847 | |  |
|  | BdMKK3-2 | Bradi1g41860 | - | + | 1572 | 523 | 10 | | 38580168 - 38590500 | |  |
|  | BdMKK3-3 | Bradi3g11260 | - | + | 1704 | 568 | 9 | | 9689290 - 9697559 | |  |
|  | BdMKK4 | Bradi3g53650 | - | - | 1074 | 357 | 0 | | 54258852 - 54260320 | |  |
|  | BdMKK5 | Bradi1g46880 | - | + | 1032 | 343 | 0 | | 45433080 - 45434736 | |  |
|  | BdMKK6 | Bradi1g75150 | - | - | 1071 | 356 | 7 | | 72118481 - 72123498 | |  |
|  | BdMKK10-1 | Bradi1g11525 | - | + | 1044 | 347 | 0 | | 8573765 - 8574808 | |  |
|  | BdMKK10-2 | Bradi1g69400 | - | - | 1023 | 341 | 0 | | 67832493 - 67833815 | |  |
|  | BdMKK10-3 | Bradi1g10800 | - | - | 1035 | 344 | 0 | | 7865287 - 7866321 | |  |
|  | BdMKK10-4 | Bradi1g10770 | - | - | 1026 | 341 | 0 | | 7809375 - 7810400 | |  |
|  | BdMKK10-5 | Bradi1g10790 | - | + | 999 | 332 | 0 | | 7835644 - 7836642 | |  |
| Bd21-3 | Bd21-3MKK1 | Brdisv1Bd21-3_r1008133m | Bradi1g51000 | + | 1047 | 348 | 8 | | pseudomolecule_1:44168003..44173037 | |  |
|  | Bd21-3MKK3-1 | Brdisv1Bd21-3_r1038149m | Bradi4g39490 | + | 1572 | 523 | 9 | | pseudomolecule_4:36720925..36728927 | |  |
|  | Bd21-3MKK3-2 | Brdisv1Bd21-3_r1006624m | Bradi1g41860 | - | 1572 | 523 | 10 | | pseudomolecule_1:34533722..34550214 | |  |
|  | Bd21-3MKK3-3 | Brdisv1Bd21-3_r1024469m | Bradi3g11260 | - | 1785 | 594 | 9 | | pseudomolecule_3:9237852..9247282 | |  |
|  | Bd21-3MKK4 | Brdisv1Bd21-3_r1030936m | Bradi3g53650 | + | 1074 | 357 | 0 | | pseudomolecule_3:47163826..47166345 | |  |
|  | Bd21-3MKK5 | Brdisv1Bd21-3_r1007409m | Bradi1g46880 | - | 1032 | 343 | 1 | | pseudomolecule_1:40265870..40269959 | |  |
|  | Bd21-3MKK6 | Brdisv1Bd21-3_r1012124m | Bradi1g75150 | + | 1071 | 356 | 7 | | pseudomolecule_1:65798015..65805455 | |  |
|  | Bd21-3MKK10-1 | Brdisv1Bd21-3_r1001769m | Bradi1g11525 | - | 1044 | 347 | 0 | | pseudomolecule_1:8110055..8111516 | |  |
|  | Bd21-3MKK10-2 | Brdisv1Bd21-3_r1011270m | Bradi1g69400 | + | 1023 | 340 | 0 | | pseudomolecule_1:61915942..61917794 | |  |
|  | Bd21-3MKK10-3 | Brdisv1Bd21-3_r1001669m | Bradi1g10800 | + | 1035 | 344 | 0 | | pseudomolecule_1:7474752..7476200 | |  |
|  | Bd21-3MKK10-4 | Brdisv1Bd21-3_r1001667m | Bradi1g10770 | + | 1026 | 341 | 0 | | pseudomolecule_1:7438777..7440212 | |  |
|  | Bd21-3MKK10-5 | Brdisv1Bd21-3_r1043789m | Bradi1g10790 | - | 999 | 332 | 0 | | pseudomolecule_6:9124412..9126541 | |  |
| Bd3-1 | Bd3-1MKK1 | Brdisv1Bd3-1_r1006392m | Bradi1g51000 | + | 1047 | 348 | 8 | | pseudomolecule_1:31746617..31752181 | |  |
|  | Bd3-1MKK3-1 | Brdisv1Bd3-1_r1029979m | Bradi4g39490 | + | 1572 | 523 | 10 | | pseudomolecule_4:22985777..22993538 | |  |
|  | Bd3-1MKK3-2 | Brdisv1Bd3-1_r1035942m | Bradi1g41860 | - | 1572 | 523 | 10 | | pseudomolecule_7:15866770..15883261 | |  |
|  | Bd3-1MKK3-3 | Brdisv1Bd3-1_r1010293m | Bradi3g11260 | - | 1785 | 594 | 9 | | pseudomolecule_2:752204..761634 | |  |
|  | Bd3-1MKK4 | Brdisv1Bd3-1_r1024627m | Bradi3g53650 | + | 378 | 125 | 0 | | pseudomolecule_3:32069807..32070906 | |  |
|  | Bd3-1MKK6 | Brdisv1Bd3-1_r1009660m | Bradi1g75150 | - | 1071 | 356 | 7 | | pseudomolecule_1:48044672..48051866 | |  |
|  | Bd3-1MKK10-3 | Brdisv1Bd3-1_r1006471m | Bradi1g10800 | + | 783 | 260 | 1 | | pseudomolecule_1:32140066..32141663 | |  |
| Bd2-3 | Bd2-3MKK1 | Brdisv1Bd2-31007550m | Bradi1g51000 | + | 1047 | 348 | 8 | | pseudomolecule_1:32464939..32469973 | |  |
|  | Bd2-3MKK3-1 | Brdisv1Bd2-31026929m | Bradi4g39490 | + | 1572 | 523 | 9 | | pseudomolecule_3:25568815..25576817 | |  |
|  | Bd2-3MKK3-2 | Brdisv1Bd2-31040703m | Bradi1g41860 | - | 1572 | 523 | 10 | | pseudomolecule_8:3099796..3116288 | |  |
|  | Bd2-3MKK3-3 | Brdisv1Bd2-31029786m | Bradi3g11260 | - | 1785 | 594 | 9 | | pseudomolecule_4:7850196..7859626 | |  |
|  | Bd2-3MKK4 | Brdisv1Bd2-31001621m | Bradi3g53650 | - | 882 | 293 | 0 | | pseudomolecule_1:6369809..6371773 | |  |
|  | Bd2-3MKK5 | Brdisv1Bd2-31001622m | Bradi1g46880 | - | 993 | 330 | 2 | | pseudomolecule_1:6374512..6378669 | |  |
|  | Bd2-3MKK6 | Brdisv1Bd2-31011337m | Bradi1g75150 | + | 786 | 261 | 5 | | pseudomolecule_1:48371340..48377176 | |  |
|  | Bd2-3MKK10-1 | Brdisv1Bd2-31001731m | Bradi1g11525 | - | 1044 | 347 | 0 | | pseudomolecule_1:6908118..6909579 | |  |
|  | Bd2-3MKK10-2 | Brdisv1Bd2-31010489m | Bradi1g69400 | + | 1023 | 340 | 0 | | pseudomolecule_1:45211806..45213658 | |  |
|  | Bd2-3MKK10-3 | Brdisv1Bd2-31001625m | Bradi1g10800 | + | 1029 | 342 | 1 | | pseudomolecule_1:6387485..6388976 | |  |
|  | Bd2-3MKK10-4 | Brdisv1Bd2-31001617m | Bradi1g10770 | + | 1023 | 340 | 1 | | pseudomolecule_1:6340600..6342109 | |  |
|  | Bd2-3MKK10-5 | Brdisv1Bd2-31001619m | Bradi1g10790 | + | 999 | 332 | 0 | | pseudomolecule_1:6359003..6361132 | |  |
| Adi-10 | Adi-10MKK1 | Brdisv1Adi-101005559m | Bradi1g51000 | + | 1047 | 348 | 8 | | pseudomolecule_1:23482509..23487543 | |  |
|  | Adi-10MKK3-1 | Brdisv1Adi-101020471m | Bradi4g39490 | + | 1572 | 523 | 9 | | pseudomolecule_3:18873552..18882036 | |  |
|  | Adi-10MKK3-2 | Brdisv1Adi-101031183m | Bradi1g41860 | - | 1572 | 523 | 10 | | pseudomolecule_7:2549452..2565944 | |  |
|  | Adi-10MKK3-3 | Brdisv1Adi-101022600m | Bradi3g11260 | - | 1785 | 594 | 9 | | pseudomolecule_4:5817606..5827036 | |  |
|  | Adi-10MKK4 | Brdisv1Adi-101022194m | Bradi3g53650 | + | 624 | 207 | 0 | | pseudomolecule_4:3983117..3984703 | |  |
|  | Adi-10MKK5 | Brdisv1Adi-101005080m | Bradi1g46880 | - | 531 | 176 | 0 | | pseudomolecule_1:21596893..21597635 | |  |
|  | Adi-10MKK6 | Brdisv1Adi-101008538m | Bradi1g75150 | + | 1071 | 356 | 7 | | pseudomolecule_1:35608515..35615794 | |  |
|  | Adi-10MKK10-1 | Brdisv1Adi-101039188m | Bradi1g11525 | - | 1032 | 343 | 2 | | pseudomolecule_7:70447867..70453678 | |  |
|  | Adi-10MKK10-2 | Brdisv1Adi-101007868m | Bradi1g69400 | + | 579 | 192 | 2 | | pseudomolecule_1:33031261..33032395 | |  |
|  | Adi-10MKK10-5 | Brdisv1Adi-101038807m | Bradi1g10790 | + | 621 | 206 | 1 | | pseudomolecule_7:65830668..65832452 | |  |
| BdTR12c | BdTR12cMKK1 | Brdisv1BdTR12c1007536m | Bradi1g51000 | + | 1047 | 348 | 8 | | pseudomolecule_1:32452529..32457276 | |  |
|  | BdTR12cMKK3-1 | Brdisv1BdTR12c1026819m | Bradi4g39490 | + | 987 | 328 | 6 | | pseudomolecule_3:25198660..25202766 | |  |
|  | BdTR12cMKK3-2 | Brdisv1BdTR12c1042600m | Bradi1g41860 | + | 1008 | 335 | 7 | | pseudomolecule_8:22310596..22319428 | |  |
|  | BdTR12cMKK3-3 | Brdisv1BdTR12c1029700m | Bradi3g11260 | - | 1785 | 594 | 9 | | pseudomolecule_4:8042720..8052150 | |  |
|  | BdTR12cMKK4 | Brdisv1BdTR12c1001607m | Bradi3g53650 | + | 957 | 318 | 1 | | pseudomolecule_1:6465356..6467972 | |  |
|  | BdTR12cMKK5 | Brdisv1BdTR12c1006838m | Bradi1g46880 | - | 927 | 308 | 2 | | pseudomolecule_1:29474546..2947872 | |  |
|  | BdTR12cMKK6 | Brdisv1BdTR12c1011459m | Bradi1g75150 | + | 1071 | 356 | 7 | | pseudomolecule_1:49356125..49363565 | |  |
|  | BdTR12cMKK10-1 | Brdisv1BdTR12c1001730m | Bradi1g11525 | - | 1044 | 347 | 0 | | pseudomolecule_1:7046027..7047488 | |  |
|  | BdTR12cMKK10-2 | Brdisv1BdTR12c1010624m | Bradi1g69400 | + | 1029 | 342 | 0 | | pseudomolecule_1:46206345..46208205 | |  |
|  | BdTR12cMKK10-3 | Brdisv1BdTR12c1001612m | Bradi1g10800 | + | 1035 | 344 | 0 | | pseudomolecule_1:6498883..6500331 | |  |
|  | BdTR12cMKK10-4 | Brdisv1BdTR12c1001599m | Bradi1g10770 | + | 1026 | 341 | 0 | | pseudomolecule_1:6441347..6442782 | |  |
|  | BdTR12cMKK10-5 | Brdisv1BdTR12c1001601m | Bradi1g10790 | - | 999 | 332 | 0 | | pseudomolecule_1: 6452602-6453600 | |  |
| Adi-2 | Adi-2MKK1 | Brdisv1Adi-21008032m | Bradi1g51000 | + | 1047 | 348 | 8 | | pseudomolecule_1:38076005..38081039 | |  |
|  | Adi-2MKK3-1 | Brdisv1Adi-21029486m | Bradi4g39490 | + | 1572 | 523 | 9 | | pseudomolecule_3:32065487..32073489 | |  |
|  | Adi-2MKK3-2 | Brdisv1Adi-21044561m | Bradi1g41860 | + | 1572 | 523 | 9 | | pseudomolecule_8:4353998..4370483 | |  |
|  | Adi-2MKK3-3 | Brdisv1Adi-21032560m | Bradi3g11260 | - | 1785 | 594 | 10 | | pseudomolecule_4:9070540..9079970 | |  |
|  | Adi-2MKK4 | Brdisv1Adi-21006537m | Bradi3g53650 | + | 1074 | 357 | 0 | | pseudomolecule_1:30337692..30340211 | |  |
|  | Adi-2MKK5 | Brdisv1Adi-21007328m | Bradi1g46880 | - | 1032 | 343 | 1 | | pseudomolecule_1:34876359..34880448 | |  |
|  | Adi-2MKK6 | Brdisv1Adi-21012376m | Bradi1g75150 | + | 1071 | 356 | 7 | | pseudomolecule_1:58058495..58065935 | |  |
|  | Adi-2MKK10-1 | Brdisv1Adi-21001745m | Bradi1g11525 | - | 1044 | 347 | 0 | | pseudomolecule_1:7327728..7329189 | |  |
|  | Adi-2MKK10-2 | Brdisv1Adi-21011441m | Bradi1g69400 | + | 1029 | 342 | 0 | | pseudomolecule_1:54368687..54370547 | |  |
|  | Adi-2MKK10-3 | Brdisv1Adi-21001643m | Bradi1g10800 | + | 1035 | 344 | 0 | | pseudomolecule_1:6729429..6730877 | |  |
|  | Adi-2MKK10-4 | Brdisv1Adi-21001640m | Bradi1g10770 | + | 1026 | 341 | 0 | | pseudomolecule_1:6694320..6695755 | |  |
|  | Adi-2MKK10-5 | Brdisv1Adi-21001642m | Bradi1g10790 | + | 999 | 332 | 0 | | pseudomolecule_1:6714021..6716150 | |  |
| Adi-12 | Adi-12MKK1 | Brdisv1Adi-121007566m | Bradi1g51000 | + | 1047 | 348 | 8 | | pseudomolecule_1:33030004..33035038 | |  |
|  | Adi-12MKK3-1 | Brdisv1Adi-121026937m | Bradi4g39490 | + | 1572 | 523 | 9 | | pseudomolecule_3:25905849..25913851 | |  |
|  | Adi-12MKK3-2 | Brdisv1Adi-121040653m | Bradi1g41860 | - | 1572 | 523 | 10 | | pseudomolecule_7:3148980..3165472 | |  |
|  | Adi-12MKK3-3 | Brdisv1Adi-121029689m | Bradi3g11260 | - | 1785 | 594 | 9 | | pseudomolecule_4:7765671..7775101 | |  |
|  | Adi-12MKK4 | Brdisv1Adi-121001565m | Bradi3g53650 | + | 879 | 292 | 1 | | pseudomolecule_1:6334223..6336770 | |  |
|  | Adi-12MKK5 | Brdisv1Adi-121001563m | Bradi1g46880 | - | 933 | 310 | 2 | | pseudomolecule_1:6317299..6321415 | |  |
|  | Adi-12MKK6 | Brdisv1Adi-121011367m | Bradi1g75150 | + | 1071 | 356 | 7 | | pseudomolecule_1:49434246..49441513 | |  |
|  | Adi-12MKK10-1 | Brdisv1Adi-121001666m | Bradi1g11525 | - | 1044 | 347 | 0 | | pseudomolecule_1:6869447..6870908 | |  |
|  | Adi-12MKK10-2 | Brdisv1Adi-121010560m | Bradi1g69400 | + | 1029 | 342 | 0 | | pseudomolecule_1:46271784..46273644 | |  |
|  | Adi-12MKK10-3 | Brdisv1Adi-121001567m | Bradi1g10800 | + | 1035 | 344 | 0 | | pseudomolecule_1:6350895..6352343 | |  |
|  | Adi-12MKK10-4 | Brdisv1Adi-121001560m | Bradi1g10770 | + | 1026 | 341 | 0 | | pseudomolecule_1:6297224..6298659 | |  |
|  | Adi-12MKK10-5 | Brdisv1Adi-121001564m | Bradi1g10790 | + | 999 | 332 | 0 | | pseudomolecule_1:6330451..6332580 | |  |
| BdTR9k | BdTR9kMKK1 | Brdisv1BdTR9K1007081m | Bradi1g51000 | + | 1047 | 348 | 8 | | pseudomolecule_1:29804842..29809876 | |  |
|  | BdTR9kMKK3-1 | Brdisv1BdTR9K1025659m | Bradi4g39490 | + | 1572 | 523 | 9 | | pseudomolecule_3:23685877..23693879 | |  |
|  | BdTR9kMKK3-2 | Brdisv1BdTR9K1038970m | Bradi1g41860 | - | 1572 | 523 | 10 | | pseudomolecule_7:5561509..5577995 | |  |
|  | BdTR9kMKK3-3 | Brdisv1BdTR9K1028259m | Bradi3g11260 | - | 1785 | 594 | 9 | | pseudomolecule_4:7197557..7206987 | |  |
|  | BdTR9kMKK4 | Brdisv1BdTR9K1033576m | Bradi3g53650 | + | 768 | 255 | 0 | | pseudomolecule_4:29978013..29980607 | |  |
|  | BdTR9kMKK5 | Brdisv1BdTR9K1006454m | Bradi1g46880 | - | 933 | 310 | 2 | | pseudomolecule_1:27258179..27262337 | |  |
|  | BdTR9kMKK6 | Brdisv1BdTR9K1010742m | Bradi1g75150 | + | 1071 | 356 | 7 | | pseudomolecule_1:45281319..45288759 | |  |
|  | BdTR9kMKK10-1 | Brdisv1BdTR9K1001602m | Bradi1g11525 | - | 966 | 321 | 1 | | pseudomolecule_1:6606501..6607982 | |  |
|  | BdTR9kMKK10-2 | Brdisv1BdTR9K1009908m | Bradi1g69400 | + | 1029 | 342 | 0 | | pseudomolecule_1:42085228..42087088 | |  |
|  | BdTR9kMKK10-3 | Brdisv1BdTR9K1001500m | Bradi1g10800 | + | 1035 | 344 | 0 | | pseudomolecule_1:6155070..6156518 | |  |
|  | BdTR9kMKK10-4 | Brdisv1BdTR9K1001494m | Bradi1g10770 | + | 1023 | 340 | 1 | | pseudomolecule_1:6125484..6126993 | |  |
|  | BdTR9kMKK10-5 | Brdisv1BdTR9K1001498m | Bradi1g10790 | + | 567 | 188 | 1 | | pseudomolecule_1:6144826..6146492 | |  |
| Kah-1 | Kah-1MKK1 | Brdisv1Kah-11006282m | Bradi1g51000 | + | 1047 | 348 | 8 | | pseudomolecule_1:25403457..25408491 | |  |
|  | Kah-1MKK3-1 | Brdisv1Kah-11030282m | Bradi4g39490 | + | 1572 | 523 | 9 | | pseudomolecule_4:19776730..19784764 | |  |
|  | Kah-1MKK3-2 | Brdisv1Kah-11035802m | Bradi1g41860 | - | 1227 | 408 | 8 | | pseudomolecule_7:11786250..11796432 | |  |
|  | Kah-1MKK3-3 | Brdisv1Kah-11005087m | Bradi3g11260 | - | 1344 | 447 | 6 | | pseudomolecule_1:20316886..20322501 | |  |
|  | Kah-1MKK4 | Brdisv1Kah-11019242m | Bradi3g53650 | + | 1074 | 357 | 0 | | pseudomolecule_3:4812150..4814670 | |  |
|  | Kah-1MKK5 | Brdisv1Kah-11005707m | Bradi1g46880 | - | 1032 | 343 | 1 | | pseudomolecule_1:23195434..23201727 | |  |
|  | Kah-1MKK6 | Brdisv1Kah-11009516m | Bradi1g75150 | + | 1071 | 356 | 7 | | pseudomolecule_1:38524374..38531781 | |  |
|  | Kah-1MKK10-1 | Brdisv1Kah-11001501m | Bradi1g11525 | - | 1044 | 347 | 0 | | pseudomolecule_1:5965371..5966832 | |  |
|  | Kah-1MKK10-2 | Brdisv1Kah-11008762m | Bradi1g69400 | + | 1026 | 341 | 0 | | pseudomolecule_1:35744625..35746480 | |  |
|  | Kah-1MKK10-3 | Brdisv1Kah-11001427m | Bradi1g10800 | + | 1035 | 344 | 0 | | pseudomolecule_1:5602230..5603678 | |  |
|  | Kah-1MKK10-4 | Brdisv1Kah-11001420m | Bradi1g10770 | + | 1026 | 341 | 0 | | pseudomolecule_1:5555229..5556664 | |  |
|  | Kah-1MKK10-5 | Brdisv1Kah-11001422m | Bradi1g10790 | - | 999 | 332 | 0 | | pseudomolecule_1:5563295..5565424 | |  |
| Kah-5 | Kah-5MKK1 | Brdisv1Kah-51008203m | Bradi1g51000 | + | 1047 | 348 | 8 | | pseudomolecule_1:37916810..37921844 | |  |
|  | Kah-5MKK3-1 | Brdisv1Kah-51029647m | Bradi4g39490 | + | 1572 | 523 | 9 | | pseudomolecule_3:31499218..31507220 | |  |
|  | Kah-5MKK3-2 | Brdisv1Kah-51044528m | Bradi1g41860 | + | 1572 | 523 | 10 | | pseudomolecule_8:3547648..3564140 | |  |
|  | Kah-5MKK3-3 | Brdisv1Kah-51032682m | Bradi3g11260 | - | 1785 | 594 | 9 | | pseudomolecule_4:8840958..8850388 | |  |
|  | Kah-5MKK4 | Brdisv1Kah-51039016m | Bradi3g53650 | + | 1074 | 357 | 0 | | pseudomolecule_4:39871679..39874198 | |  |
|  | Kah-5MKK5 | Brdisv1Kah-51007471m | Bradi1g46880 | - | 1032 | 343 | 1 | | pseudomolecule_1:34748540..34752629 | |  |
|  | Kah-5MKK6 | Brdisv1Kah-51012549m | Bradi1g75150 | + | 1071 | 356 | 7 | | pseudomolecule_1:57970763..57978203 | |  |
|  | Kah-5MKK10-1 | Brdisv1Kah-51001737m | Bradi1g11525 | - | 1044 | 347 | 0 | | pseudomolecule_1:7336545..7338006 | |  |
|  | Kah-5MKK10-2 | Brdisv1Kah-51011638m | Bradi1g69400 | + | 1029 | 342 | 0 | | pseudomolecule_1:54452561..54454421 | |  |
|  | Kah-5MKK10-3 | Brdisv1Kah-51001629m | Bradi1g10800 | + | 1035 | 344 | 0 | | pseudomolecule_1:6702332..6703780 | |  |
|  | Kah-5MKK10-4 | Brdisv1Kah-51001626m | Bradi1g10770 | + | 1026 | 341 | 0 | | pseudomolecule_1:6667228..6668663 | |  |
|  | Kah-5MKK10-5 | Brdisv1Kah-51001628m | Bradi1g10790 | - | 999 | 332 | 0 | | pseudomolecule_1:6690167..6692296 | |  |
| BdTR5i | BdTR5iMKK1 | Brdisv1BdTR5I1007104m | Bradi1g51000 | + | 1047 | 348 | 8 | | pseudomolecule_1:30099930..30104964 | |  |
|  | BdTR5iMKK3-1 | Brdisv1BdTR5I1025501m | Bradi4g39490 | + | 1572 | 523 | 9 | | pseudomolecule_3:23310571..23318573 | |  |
|  | BdTR5iMKK3-2 | Brdisv1BdTR5I1038660m | Bradi1g41860 | + | 1572 | 523 | 10 | | pseudomolecule_8:2662476..2678968 | |  |
|  | BdTR5iMKK3-3 | Brdisv1BdTR5I1028137m | Bradi3g11260 | - | 1785 | 594 | 9 | | pseudomolecule_4:7054762..7064192 | |  |
|  | BdTR5iMKK4 | Brdisv1BdTR5I1027632m | Bradi3g53650 | + | 1074 | 357 | 0 | | pseudomolecule_4:4873635..4876154 | |  |
|  | BdTR5iMKK5 | Brdisv1BdTR5I1006491m | Bradi1g46880 | - | 513 | 170 | 0 | | pseudomolecule_1:27565294..27566012 | |  |
|  | BdTR5iMKK6 | Brdisv1BdTR5I1010763m | Bradi1g75150 | + | 1071 | 356 | 7 | | pseudomolecule_1:45734500..45741940 | |  |
|  | BdTR5iMKK10-1 | Brdisv1BdTR5I1001606m | Bradi1g11525 | - | 1044 | 347 | 0 | | pseudomolecule_1:6428783..6430244 | |  |
|  | BdTR5iMKK10-2 | Brdisv1BdTR5I1009931m | Bradi1g69400 | + | 1029 | 342 | 0 | | pseudomolecule_1:42549775..42551635 | |  |
|  | BdTR5iMKK10-3 | Brdisv1BdTR5I1001520m | Bradi1g10800 | + | 801 | 266 | 1 | | pseudomolecule_1:6000846..6002142 | |  |
|  | BdTR5iMKK10-4 | Brdisv1BdTR5I1001517m | Bradi1g10770 | + | 1023 | 340 | 1 | | pseudomolecule_1:5990893..5992402 | |  |
|  | BdTR5iMKK10-5 | Brdisv1BdTR5I1001515m | Bradi1g10790 | + | 999 | 332 | 0 | | pseudomolecule_1:5982275..5984404 | |  |
| BdTR10c | BdTR10cMKK1 | Brdisv1BdTR10C1006888m | Bradi1g51000 | + | 1047 | 348 | 8 | | pseudomolecule_1:29810209..29815773 | |  |
|  | BdTR10cMKK3-1 | Brdisv1BdTR10C1033120m | Bradi4g39490 | + | 1572 | 523 | 9 | | pseudomolecule_4:24226447..24234449 | |  |
|  | BdTR10cMKK3-2 | Brdisv1BdTR10C1038053m | Bradi1g41860 | + | 1572 | 523 | 10 | | pseudomolecule_8:4388550..4405042 | |  |
|  | BdTR10cMKK3-3 | Brdisv1BdTR10C1021519m | Bradi3g11260 | - | 1785 | 594 | 9 | | pseudomolecule_3:7142631..7152064 | |  |
|  | BdTR10cMKK4 | Brdisv1BdTR10C1021040m | Bradi3g53650 | + | 768 | 255 | 0 | | pseudomolecule_3:4983049..4984685 | |  |
|  | BdTR10cMKK5 | Brdisv1BdTR10C1006269m | Bradi1g46880 | - | 540 | 179 | 0 | | pseudomolecule_1:27383468..27384223 | |  |
|  | BdTR10cMKK6 | Brdisv1BdTR10C1010577m | Bradi1g75150 | + | 1071 | 356 | 7 | | pseudomolecule_1:45486319..45493759 | |  |
|  | BdTR10cMKK10-1 | Brdisv1BdTR10C1001595m | Bradi1g11525 | - | 870 | 289 | 1 | | pseudomolecule_1:6431213..6432661 | |  |
|  | BdTR10cMKK10-2 | Brdisv1BdTR10C1009777m | Bradi1g69400 | + | 579 | 192 | 0 | | pseudomolecule_1:42393752..42395577 | |  |
|  | BdTR10cMKK10-3 | Brdisv1BdTR10C1040500m | Bradi1g10800 | - | 492 | 163 | 3 | | pseudomolecule_8:27200877..27202113 | |  |
|  | BdTR10cMKK10-4 | Brdisv1BdTR10C1044301m | Bradi1g10770 | - | 624 | 207 | 0 | | pseudomolecule_8:89440489..89441362 | |  |
|  | BdTR10cMKK10-5 | Brdisv1BdTR10C1041730m | Bradi1g10790 | + | 609 | 202 | 0 | | pseudomolecule_8:40318684..40320027 | |  |
| BdTR11a | BdTR11aMKK1 | Brdisv1BdTR11A1007305m | Bradi1g51000 | + | 1047 | 348 | 8 | | pseudomolecule_1:30302767..30308331 | |  |
|  | BdTR11aMKK3-1 | Brdisv1BdTR11A1026487m | Bradi4g39490 | + | 1572 | 523 | 9 | | pseudomolecule_3:23471196..23479198 | |  |
|  | BdTR11aMKK3-2 | Brdisv1BdTR11A1042993m | Bradi1g41860 | + | 1572 | 523 | 10 | | pseudomolecule_7:22005871..22022363 | |  |
|  | BdTR11aMKK3-3 | Brdisv1BdTR11A1029299m | Bradi3g11260 | - | 1785 | 594 | 9 | | pseudomolecule_4:7340294..7349724 | |  |
|  | BdTR11aMKK4 | Brdisv1BdTR11A1034905m | Bradi3g53650 | + | 1074 | 357 | 0 | | pseudomolecule_4:30985944..30988463 | |  |
|  | BdTR11aMKK5 | Brdisv1BdTR11A1006653m | Bradi1g46880 | - | 1032 | 343 | 1 | | pseudomolecule_1:27829535..27833624 | |  |
|  | BdTR11aMKK6 | Brdisv1BdTR11A1011184m | Bradi1g75150 | + | 1071 | 356 | 7 | | pseudomolecule_1:46108780..46116220 | |  |
|  | BdTR11aMKK10-1 | Brdisv1BdTR11A1001689m | Bradi1g11525 | - | 1044 | 347 | 0 | | pseudomolecule_1:6869918..6871379 | |  |
|  | BdTR11aMKK10-2 | Brdisv1BdTR11A1010326m | Bradi1g69400 | + | 1029 | 342 | 0 | | pseudomolecule_1:42885901..42887761 | |  |
|  | BdTR11aMKK10-3 | Brdisv1BdTR11A1001594m | Bradi1g10800 | - | 1035 | 344 | 0 | | pseudomolecule_1:6387463..6388911 | |  |
|  | BdTR11aMKK10-4 | Brdisv1BdTR11A1001588m | Bradi1g10770 | + | 1026 | 341 | 0 | | pseudomolecule_1:6360902..6362337 | |  |
|  | BdTR11aMKK10-5 | Brdisv1BdTR11A1001590m | Bradi1g10790 | - | 999 | 332 | 0 | | pseudomolecule_1:6371103..6373232 | |  |
| BdTR11i | BdTR11iMKK1 | Brdisv1BdTR11I1007977m | Bradi1g51000 | + | 1047 | 348 | 8 | | pseudomolecule_1:35654944..35660508 | |  |
|  | BdTR11iMKK3-1 | Brdisv1BdTR11I1028588m | Bradi4g39490 | + | 1572 | 523 | 9 | | pseudomolecule_3:28209371..28217373 | |  |
|  | BdTR11iMKK3-2 | Brdisv1BdTR11I1043285m | Bradi1g41860 | - | 1572 | 523 | 10 | | pseudomolecule_7:3647519..3664011 | |  |
|  | BdTR11iMKK3-3 | Brdisv1BdTR11I1031672m | Bradi3g11260 | - | 1785 | 594 | 9 | | pseudomolecule_4:8707417..8716847 | |  |
|  | BdTR11iMKK4 | Brdisv1BdTR11I1037783m | Bradi3g53650 | + | 1074 | 357 | 0 | | pseudomolecule_4:36740380..36742899 | |  |
|  | BdTR11iMKK5 | Brdisv1BdTR11I1007275m | Bradi1g46880 | - | 1032 | 343 | 1 | | pseudomolecule_1:32644659..32648748 | |  |
|  | BdTR11iMKK6 | Brdisv1BdTR11I1012108m | Bradi1g75150 | + | 1071 | 356 | 7 | | pseudomolecule_1:53833599..53841039 | |  |
|  | BdTR11iMKK10-1 | Brdisv1BdTR11I1001760m | Bradi1g11525 | - | 1044 | 347 | 0 | | pseudomolecule_1:7242091..7243552 | |  |
|  | BdTR11iMKK10-2 | Brdisv1BdTR11I1011205m | Bradi1g69400 | + | 1029 | 342 | 0 | | pseudomolecule_1:50322981..50324841 | |  |
|  | BdTR11iMKK10-3 | Brdisv1BdTR11I1001659m | Bradi1g10800 | + | 1035 | 344 | 0 | | pseudomolecule_1:6680518..6681966 | |  |
|  | BdTR11iMKK10-4 | Brdisv1BdTR11I1001643m | Bradi1g10770 | + | 1026 | 341 | 0 | | pseudomolecule_1:6609761..6611196 | |  |
|  | BdTR11iMKK10-5 | Brdisv1BdTR11I1001645m | Bradi1g10790 | - | 999 | 332 | 0 | | pseudomolecule_1:6625692..6627821 | |  |
| BdTR11g | BdTR11gMKK1 | Brdisv1BdTR11G1008252m | Bradi1g51000 | + | 1047 | 348 | 8 | | pseudomolecule_1:39063017..39068581 | |  |
|  | BdTR11gMKK3-1 | Brdisv1BdTR11G1029451m | Bradi4g39490 | + | 1572 | 523 | 9 | | pseudomolecule_3:31440522..31448524 | |  |
|  | BdTR11gMKK3-2 | Brdisv1BdTR11G1044565m | Bradi1g41860 | + | 1572 | 523 | 10 | | pseudomolecule_7:4346071..4362563 | |  |
|  | BdTR11gMKK3-3 | Brdisv1BdTR11G1032411m | Bradi3g11260 | - | 1785 | 594 | 9 | | pseudomolecule_4:8673614..8683044 | |  |
|  | BdTR11gMKK4 | Brdisv1BdTR11G1038753m | Bradi3g53650 | + | 1074 | 357 | 0 | | pseudomolecule_4:40258495..40261014 | |  |
|  | BdTR11gMKK5 | Brdisv1BdTR11G1007511m | Bradi1g46880 | - | 1032 | 343 | 1 | | pseudomolecule_1:35840159..35844248 | |  |
|  | BdTR11gMKK6 | Brdisv1BdTR11G1012553m | Bradi1g75150 | + | 1071 | 356 | 7 | | pseudomolecule_1:59021519..59028959 | |  |
|  | BdTR11gMKK10-1 | Brdisv1BdTR11G1001791m | Bradi1g11525 | - | 1044 | 347 | 0 | | pseudomolecule_1:7672072..7673533 | |  |
|  | BdTR11gMKK10-2 | Brdisv1BdTR11G1011611m | Bradi1g69400 | + | 1029 | 342 | 0 | | pseudomolecule_1:55339772..55341632 | |  |
|  | BdTR11gMKK10-3 | Brdisv1BdTR11G1001687m | Bradi1g10800 | + | 1035 | 344 | 0 | | pseudomolecule_1:7014424..7015872 | |  |
|  | BdTR11gMKK10-4 | Brdisv1BdTR11G1001684m | Bradi1g10770 | + | 1026 | 341 | 0 | | pseudomolecule_1:6978480..6979915 | |  |
|  | BdTR11gMKK10-5 | Brdisv1BdTR11G1001686m | Bradi1g10790 | - | 999 | 332 | 0 | | pseudomolecule_1:7001349..7003478 | |  |
| BdTR13c | BdTR13cMKK1 | Brdisv1BdTR13C1007320m | Bradi1g51000 | + | 1047 | 348 | 8 | | pseudomolecule_1:31416425..31421459 | |  |
|  | BdTR13cMKK3-1 | Brdisv1BdTR13C1026262m | Bradi4g39490 | + | 1572 | 523 | 9 | | pseudomolecule_3:24802980..24810982 | |  |
|  | BdTR13cMKK3-2 | Brdisv1BdTR13C1039819m | Bradi1g41860 | + | 1572 | 523 | 10 | | pseudomolecule_8:2758880..2775372 | |  |
|  | BdTR13cMKK3-3 | Brdisv1BdTR13C1029001m | Bradi3g11260 | - | 1785 | 594 | 9 | | pseudomolecule_4:7598147..7607580 | |  |
|  | BdTR13cMKK4 | Brdisv1BdTR13C1043199m | Bradi3g53650 | + | 1173 | 390 | 1 | | pseudomolecule_8:35528775..35531527 | |  |
|  | BdTR13cMKK5 | Brdisv1BdTR13C1006669m | Bradi1g46880 | - | 933 | 310 | 2 | | pseudomolecule_1:28806585..28810730 | |  |
|  | BdTR13cMKK6 | Brdisv1BdTR13C1011139m | Bradi1g75150 | + | 1071 | 356 | 7 | | pseudomolecule_1:47718709..47726149 | |  |
|  | BdTR13cMKK10-1 | Brdisv1BdTR13C1001614m | Bradi1g11525 | - | 1044 | 347 | 0 | | pseudomolecule_1:6706068..6707529 | |  |
|  | BdTR13cMKK10-2 | Brdisv1BdTR13C1010293m | Bradi1g69400 | + | 1029 | 342 | 0 | | pseudomolecule_1:44462095..44463955 | |  |
|  | BdTR13cMKK10-3 | Brdisv1BdTR13C1001511m | Bradi1g10800 | + | 945 | 314 | 1 | | pseudomolecule_1:6167696..6169377 | |  |
|  | BdTR13cMKK10-4 | Brdisv1BdTR13C1001502m | Bradi1g10770 | + | 1026 | 341 | 0 | | pseudomolecule_1:6106402..6107837 | |  |
|  | BdTR13cMKK10-5 | Brdisv1BdTR13C1001510m | Bradi1g10790 | - | 627 | 208 | 1 | | pseudomolecule_1:6160854..6161940 | |  |
| BdTR13a | BdTR13aMKK1 | Brdisv1BdTR13a1008594m | Bradi1g51000 | + | 1047 | 348 | 8 | | pseudomolecule_1:44821106..44826140 | |  |
|  | BdTR13aMKK3-1 | Brdisv1BdTR13a1030929m | Bradi4g39490 | + | 1572 | 523 | 9 | | pseudomolecule_3:38696349..38704351 | |  |
|  | BdTR13aMKK3-2 | Brdisv1BdTR13a1006861m | Bradi1g41860 | - | 1572 | 523 | 10 | | pseudomolecule_1:34793204..34810949 | |  |
|  | BdTR13aMKK3-3 | Brdisv1BdTR13a1034167m | Bradi3g11260 | - | 1785 | 594 | 9 | | pseudomolecule_4:9306934..9316364 | |  |
|  | BdTR13aMKK4 | Brdisv1BdTR13a1040831m | Bradi3g53650 | + | 1074 | 357 | 0 | | pseudomolecule_4:47121600..47124119 | |  |
|  | BdTR13aMKK5 | Brdisv1BdTR13a1007813m | Bradi1g46880 | - | 1032 | 343 | 1 | | pseudomolecule_1:41105337..41109426 | |  |
|  | BdTR13aMKK6 | Brdisv1BdTR13a1013108m | Bradi1g75150 | + | 1071 | 356 | 7 | | pseudomolecule_1:67615801..67623241 | |  |
|  | BdTR13aMKK10-1 | Brdisv1BdTR13a1001801m | Bradi1g11525 | - | 1044 | 347 | 0 | | pseudomolecule_1:7678305..7679766 | |  |
|  | BdTR13aMKK10-2 | Brdisv1BdTR13a1012168m | Bradi1g69400 | + | 1029 | 342 | 0 | | pseudomolecule_1:63487320..63489180 | |  |
|  | BdTR13aMKK10-3 | Brdisv1BdTR13a1001694m | Bradi1g10800 | + | 1035 | 344 | 0 | | pseudomolecule_1:7096509..7097957 | |  |
|  | BdTR13aMKK10-4 | Brdisv1BdTR13a1001691m | Bradi1g10770 | + | 1026 | 341 | 0 | | pseudomolecule_1:7041559..7042994 | |  |
|  | BdTR13aMKK10-5 | Brdisv1BdTR13a1001693m | Bradi1g10790 | - | 999 | 332 | 0 | | pseudomolecule_1:7067390..7069519 | |  |
| Bis-1 | Bis-1MKK1 | Brdisv1Bis-11007849m | Bradi1g51000 | + | 1047 | 348 | 8 | | pseudomolecule_1:34846931..34851965 | |  |
|  | Bis-1MKK3-1 | Brdisv1Bis-11027832m | Bradi4g39490 | + | 1572 | 523 | 9 | | pseudomolecule_3:26775272..26783274 | |  |
|  | Bis-1MKK3-2 | Brdisv1Bis-11042177m | Bradi1g41860 | - | 1314 | 437 | 10 | | pseudomolecule_8:3199896..3216388 | |  |
|  | Bis-1MKK3-3 | Brdisv1Bis-11030798m | Bradi3g11260 | - | 1785 | 594 | 9 | | pseudomolecule_4:8176171..8185601 | |  |
|  | Bis-1MKK4 | Brdisv1Bis-11036660m | Bradi3g53650 | + | 1074 | 357 | 0 | | pseudomolecule_4:35132731..35135250 | |  |
|  | Bis-1MKK5 | Brdisv1Bis-11007171m | Bradi1g46880 | - | 1032 | 343 | 1 | | pseudomolecule_1:31991896..31995985 | |  |
|  | Bis-1MKK6 | Brdisv1Bis-11011940m | Bradi1g75150 | + | 1071 | 356 | 7 | | pseudomolecule_1:52652963..52660403 | |  |
|  | Bis-1MKK10-1 | Brdisv1Bis-11001784m | Bradi1g11525 | - | 1044 | 347 | 0 | | pseudomolecule_1:7484991..7486452 | |  |
|  | Bis-1MKK10-2 | Brdisv1Bis-11011036m | Bradi1g69400 | + | 1029 | 342 | 0 | | pseudomolecule_1:49070010..49071870 | |  |
|  | Bis-1MKK10-3 | Brdisv1Bis-11001672m | Bradi1g10800 | + | 1035 | 344 | 0 | | pseudomolecule_1:6824805..6826253 | |  |
|  | Bis-1MKK10-4 | Brdisv1Bis-11001669m | Bradi1g10770 | + | 1026 | 341 | 0 | | pseudomolecule_1:6800339..6801774 | |  |
|  | Bis-1MKK10-5 | Brdisv1Bis-11001671m | Bradi1g10790 | - | 999 | 332 | 0 | | pseudomolecule_1:6812249..6814378 | |  |
| Koz-3 | Koz-3MKK1 | Brdisv1Koz-31007066m | Bradi1g51000 | + | 1047 | 348 | 8 | | pseudomolecule_1:31702990..31708024 | |  |
|  | Koz-3MKK3-1 | Brdisv1Koz-31033544m | Bradi4g39490 | + | 1572 | 523 | 9 | | pseudomolecule_4:24187994..24195996 | |  |
|  | Koz-3MKK3-2 | Brdisv1Koz-31039828m | Bradi1g41860 | - | 1572 | 523 | 10 | | pseudomolecule_7:13496541..13513032 | |  |
|  | Koz-3MKK3-3 | Brdisv1Koz-31021934m | Bradi3g11260 | - | 1785 | 594 | 9 | | pseudomolecule_3:7645761..7655191 | |  |
|  | Koz-3MKK4 | Brdisv1Koz-31027327m | Bradi3g53650 | + | 1074 | 357 | 0 | | pseudomolecule_3:32319109..32321627 | |  |
|  | Koz-3MKK5 | Brdisv1Koz-31006454m | Bradi1g46880 | - | 1032 | 343 | 1 | | pseudomolecule_1:29208171..29212239 | |  |
|  | Koz-3MKK6 | Brdisv1Koz-31010882m | Bradi1g75150 | + | 1071 | 356 | 7 | | pseudomolecule_1:48681506..48688946 | |  |
|  | Koz-3MKK10-1 | Brdisv1Koz-31001651m | Bradi1g11525 | - | 1044 | 347 | 0 | | pseudomolecule_1:6991737..6993198 | |  |
|  | Koz-3MKK10-2 | Brdisv1Koz-31010012m | Bradi1g69400 | + | 1029 | 342 | 0 | | pseudomolecule_1:45193263..45195123 | |  |
|  | Koz-3MKK10-3 | Brdisv1Koz-31001571m | Bradi1g10800 | + | 945 | 314 | 1 | | pseudomolecule_1:6589778..6591277 | |  |
|  | Koz-3MKK10-4 | Brdisv1Koz-31001569m | Bradi1g10770 | + | 1026 | 341 | 0 | | pseudomolecule_1:6570992..6572427 | |  |
|  | Koz-3MKK10-5 | Brdisv1Koz-31041352m | Bradi1g10790 | - | 999 | 332 | 0 | | pseudomolecule_7:23775186..23777315 | |  |
| Koz-1 | Koz-1MKK1 | Brdisv1Koz-11008234m | Bradi1g51000 | + | 1047 | 348 | 8 | | pseudomolecule_1:39369405..39374439 | |  |
|  | Koz-1MKK3-1 | Brdisv1Koz-11029896m | Bradi4g39490 | + | 1572 | 523 | 9 | | pseudomolecule_3:32867257..32875259 | |  |
|  | Koz-1MKK3-2 | Brdisv1Koz-11045109m | Bradi1g41860 | - | 1572 | 523 | 10 | | pseudomolecule_8:4407232..4423724 | |  |
|  | Koz-1MKK3-3 | Brdisv1Koz-11032882m | Bradi3g11260 | - | 1785 | 594 | 9 | | pseudomolecule_4:8670147..8679577 | |  |
|  | Koz-1MKK4 | Brdisv1Koz-11039377m | Bradi3g53650 | + | 1074 | 357 | 0 | | pseudomolecule_4:41037845..41040363 | |  |
|  | Koz-1MKK5 | Brdisv1Koz-11007461m | Bradi1g46880 | - | 1032 | 343 | 1 | | pseudomolecule_1:35838763..35842852 | |  |
|  | Koz-1MKK6 | Brdisv1Koz-11012587m | Bradi1g75150 | + | 1071 | 356 | 7 | | pseudomolecule_1:59693605..59701045 | |  |
|  | Koz-1MKK10-1 | Brdisv1Koz-11001847m | Bradi1g11525 | - | 1044 | 347 | 0 | | pseudomolecule_1:7809668..7811129 | |  |
|  | Koz-1MKK10-2 | Brdisv1Koz-11011659m | Bradi1g69400 | + | 1029 | 342 | 0 | | pseudomolecule_1:56067413..56069273 | |  |
|  | Koz-1MKK10-3 | Brdisv1Koz-11001740m | Bradi1g10800 | + | 1035 | 344 | 0 | | pseudomolecule_1:7190652..7192100 | |  |
|  | Koz-1MKK10-4 | Brdisv1Koz-11001737m | Bradi1g10770 | + | 1026 | 341 | 0 | | pseudomolecule_1:7151188..7152623 | |  |
|  | Koz-1MKK10-5 | Brdisv1Koz-11001739m | Bradi1g10790 | - | 999 | 332 | 0 | | pseudomolecule_1:7176733..7178862 | |  |
| BdTR3c | BdTR3cMKK1 | Brdisv1BdTR3C1008613m | Bradi1g51000 | + | 1047 | 348 | 8 | | pseudomolecule_1:47366416..47371450 | |  |
|  | BdTR3cMKK3-1 | Brdisv1BdTR3C1038340m | Bradi4g39490 | + | 1572 | 523 | 9 | | pseudomolecule_4:42667612..42675614 | |  |
|  | BdTR3cMKK3-2 | Brdisv1BdTR3C1007023m | Bradi1g41860 | - | 1572 | 523 | 10 | | pseudomolecule_1:37614558..37632331 | |  |
|  | BdTR3cMKK3-3 | Brdisv1BdTR3C1025925m | Bradi3g11260 | - | 1785 | 594 | 9 | | pseudomolecule_3:10704073..10713503 | |  |
|  | BdTR3cMKK4 | Brdisv1BdTR3C1030687m | Bradi3g53650 | + | 1074 | 357 | 0 | | pseudomolecule_3:52147995..52150514 | |  |
|  | BdTR3cMKK5 | Brdisv1BdTR3C1007787m | Bradi1g46880 | - | 1032 | 343 | 1 | | pseudomolecule_1:43166828..43170917 | |  |
|  | BdTR3cMKK6 | Brdisv1BdTR3C1012997m | Bradi1g75150 | + | 1071 | 356 | 7 | | pseudomolecule_1:70201597..70209037 | |  |
|  | BdTR3cMKK10-1 | Brdisv1BdTR3C1001858m | Bradi1g11525 | - | 1044 | 347 | 0 | | pseudomolecule_1:8225400..8226861 | |  |
|  | BdTR3cMKK10-2 | Brdisv1BdTR3C1012083m | Bradi1g69400 | + | 1029 | 342 | 0 | | pseudomolecule_1:66385400..66387260 | |  |
|  | BdTR3cMKK10-3 | Brdisv1BdTR3C1001745m | Bradi1g10800 | + | 1035 | 344 | 0 | | pseudomolecule_1:7522785..7524233 | |  |
|  | BdTR3cMKK10-4 | Brdisv1BdTR3C1001741m | Bradi1g10770 | + | 1026 | 341 | 0 | | pseudomolecule_1:7465460..7466895 | |  |
|  | BdTR3cMKK10-5 | Brdisv1BdTR3C1001743m | Bradi1g10790 | - | 999 | 332 | 0 | | pseudomolecule_1:7491281..7493410 | |  |
| Gaz-8 | Gaz-8MKK1 | Brdisv1Gaz-81003865m | Bradi1g51000 | + | 1176 | 391 | 8 | | pseudomolecule_1:15343746..15349542 | |  |
|  | Gaz-8MKK3-1 | Brdisv1Gaz-81025569m | Bradi4g39490 | - | 1572 | 523 | 9 | | pseudomolecule_8:29552729..29560210 | |  |
|  | Gaz-8MKK3-2 | Brdisv1Gaz-81021746m | Bradi1g41860 | - | 1314 | 437 | 10 | | pseudomolecule_8:2428789..2445320 | |  |
|  | Gaz-8MKK3-3 | Brdisv1Gaz-81015780m | Bradi3g11260 | - | 1785 | 594 | 9 | | pseudomolecule_4:3570761..3580191 | |  |
|  | Gaz-8MKK6 | Brdisv1Gaz-81025547m | Bradi1g75150 | + | 1071 | 356 | 7 | | pseudomolecule_8:29346270..29353591 | |  |
| BdTR1i | BdTR1iMKK1 | Brdisv1BdTR1i1008615m | Bradi1g51000 | + | 1047 | 348 | 8 | | pseudomolecule_1:45790101..45795135 | |  |
|  | BdTR1iMKK3-1 | Brdisv1BdTR1i1040694m | Bradi4g39490 | + | 1572 | 523 | 9 | | pseudomolecule_4:39515438..39523440 | |  |
|  | BdTR1iMKK3-2 | Brdisv1BdTR1i1006974m | Bradi1g41860 | - | 1572 | 523 | 10 | | pseudomolecule_1:35849052..35865544 | |  |
|  | BdTR1iMKK3-3 | Brdisv1BdTR1i1026202m | Bradi3g11260 | - | 1785 | 594 | 9 | | pseudomolecule_3:9776778..9786208 | |  |
|  | BdTR1iMKK4 | Brdisv1BdTR1i1032955m | Bradi3g53650 | + | 1074 | 357 | 0 | | pseudomolecule_3:47407341..47409859 | |  |
|  | BdTR1iMKK5 | Brdisv1BdTR1i1007913m | Bradi1g46880 | - | 1032 | 343 | 1 | | pseudomolecule_1:42166992..42171081 | |  |
|  | BdTR1iMKK6 | Brdisv1BdTR1i1012919m | Bradi1g75150 | + | 1071 | 356 | 7 | | pseudomolecule_1:67407278..67414718 | |  |
|  | BdTR1iMKK10-1 | Brdisv1BdTR1i1002032m | Bradi1g11525 | - | 1044 | 347 | 0 | | pseudomolecule_1:9182033..9183494 | |  |
|  | BdTR1iMKK10-2 | Brdisv1BdTR1i1011964m | Bradi1g69400 | + | 1029 | 342 | 0 | | pseudomolecule_1:63410830..63412690 | |  |
|  | BdTR1iMKK10-3 | Brdisv1BdTR1i1001931m | Bradi1g10800 | + | 1035 | 344 | 0 | | pseudomolecule_1:8608188..8609636 | |  |
|  | BdTR1iMKK10-4 | Brdisv1BdTR1i1001928m | Bradi1g10770 | + | 1026 | 341 | 0 | | pseudomolecule_1:8567618..8569053 | |  |
|  | BdTR1iMKK10-5 | Brdisv1BdTR1i1001930m | Bradi1g10790 | - | 999 | 332 | 0 | | pseudomolecule_1:8593395..8595524 | |  |
| BdTR2b | BdTR2bMKK1 | Brdisv1BdTR2B1007913m | Bradi1g51000 | + | 1047 | 348 | 8 | | pseudomolecule_1:35143833..35148867 | |  |
|  | BdTR2bMKK3-1 | Brdisv1BdTR2B1037684m | Bradi4g39490 | + | 1572 | 523 | 9 | | pseudomolecule_4:27438342..27446344 | |  |
|  | BdTR2bMKK3-2 | Brdisv1BdTR2B1042966m | Bradi1g41860 | + | 1572 | 523 | 10 | | pseudomolecule_7:3479295..3495787 | |  |
|  | BdTR2bMKK3-3 | Brdisv1BdTR2B1024627m | Bradi3g11260 | - | 1785 | 594 | 9 | | pseudomolecule_3:8605761..8615191 | |  |
|  | BdTR2bMKK4 | Brdisv1BdTR2B1030719m | Bradi3g53650 | + | 1074 | 357 | 0 | | pseudomolecule_3:36839778..36842296 | |  |
|  | BdTR2bMKK5 | Brdisv1BdTR2B1007209m | Bradi1g46880 | - | 1032 | 343 | 1 | | pseudomolecule_1:32162148..32166237 | |  |
|  | BdTR2bMKK6 | Brdisv1BdTR2B1012093m | Bradi1g75150 | + | 1071 | 356 | 7 | | pseudomolecule_1:53371112..53378552 | |  |
|  | BdTR2bMKK10-1 | Brdisv1BdTR2B1001835m | Bradi1g11525 | - | 1044 | 347 | 0 | | pseudomolecule_1:7559749..7561210 | |  |
|  | BdTR2bMKK10-2 | Brdisv1BdTR2B1011207m | Bradi1g69400 | + | 1029 | 342 | 0 | | pseudomolecule_1:49957783..49959643 | |  |
|  | BdTR2bMKK10-3 | Brdisv1BdTR2B1001724m | Bradi1g10800 | - | 1035 | 344 | 0 | | pseudomolecule_1:6922575..6924023 | |  |
|  | BdTR2bMKK10-4 | Brdisv1BdTR2B1001716m | Bradi1g10770 | + | 1026 | 341 | 0 | | pseudomolecule_1:6876306..6877741 | |  |
|  | BdTR2bMKK10-5 | Brdisv1BdTR2B1001718m | Bradi1g10790 | - | 999 | 332 | 0 | | pseudomolecule_1:6897262..6899391 | |  |
| BdTR2g | BdTR2gMKK1 | Brdisv1BdTR2G1007476m | Bradi1g51000 | + | 1047 | 348 | 8 | | pseudomolecule_1:31070599..31075633 | |  |
|  | BdTR2gMKK3-1 | Brdisv1BdTR2G1026533m | Bradi4g39490 | + | 1572 | 523 | 9 | | pseudomolecule_3:23688975..23696977 | |  |
|  | BdTR2gMKK3-2 | Brdisv1BdTR2G1040261m | Bradi1g41860 | - | 1572 | 523 | 10 | | pseudomolecule_8:5924038..5940530 | |  |
|  | BdTR2gMKK3-3 | Brdisv1BdTR2G1029283m | Bradi3g11260 | - | 1785 | 594 | 9 | | pseudomolecule_4:7607893..7617323 | |  |
|  | BdTR2gMKK4 | Brdisv1BdTR2G1028797m | Bradi3g53650 | + | 1074 | 357 | 0 | | pseudomolecule_4:5489181..5491699 | |  |
|  | BdTR2gMKK5 | Brdisv1BdTR2G1006838m | Bradi1g46880 | - | 1032 | 343 | 1 | | pseudomolecule_1:28446531..28452779 | |  |
|  | BdTR2gMKK6 | Brdisv1BdTR2G1011225m | Bradi1g75150 | + | 1071 | 356 | 7 | | pseudomolecule_1:46614025..46621465 | |  |
|  | BdTR2gMKK10-1 | Brdisv1BdTR2G1001709m | Bradi1g11525 | - | 1044 | 347 | 0 | | pseudomolecule_1:6799077..6800538 | |  |
|  | BdTR2gMKK10-2 | Brdisv1BdTR2G1010379m | Bradi1g69400 | + | 1029 | 342 | 0 | | pseudomolecule_1:43414796..43416656 | |  |
|  | BdTR2gMKK10-3 | Brdisv1BdTR2G1001605m | Bradi1g10800 | + | 1035 | 344 | 0 | | pseudomolecule_1:6272089..6273537 | |  |
|  | BdTR2gMKK10-4 | Brdisv1BdTR2G1001602m | Bradi1g10770 | + | 1026 | 341 | 0 | | pseudomolecule_1:6255878..6257313 | |  |
|  | BdTR2gMKK10-5 | Brdisv1BdTR2G1042966m | Bradi1g10790 | - | 999 | 332 | 0 | | pseudomolecule_8:28142766..28144895 | |  |
| Bd18-1 | Bd18-1MKK1 | Brdisv1Bd18-11008720m | Bradi1g51000 | + | 1047 | 348 | 8 | | pseudomolecule_1:47700135..47705700 | |  |
|  | Bd18-1MKK3-1 | Brdisv1Bd18-11041331m | Bradi4g39490 | + | 1572 | 523 | 9 | | pseudomolecule_4:43682368..43690370 | |  |
|  | Bd18-1MKK3-2 | Brdisv1Bd18-11006956m | Bradi1g41860 | - | 1572 | 523 | 10 | | pseudomolecule_1:36742064..36758553 | |  |
|  | Bd18-1MKK3-3 | Brdisv1Bd18-11026234m | Bradi3g11260 | - | 1785 | 594 | 9 | | pseudomolecule_3:10082843..10092273 | |  |
|  | Bd18-1MKK4 | Brdisv1Bd18-11033291m | Bradi3g53650 | + | 1074 | 357 | 0 | | pseudomolecule_3:53705760..53708278 | |  |
|  | Bd18-1MKK5 | Brdisv1Bd18-11007870m | Bradi1g46880 | - | 1032 | 343 | 1 | | pseudomolecule_1:43380767..43384856 | |  |
|  | Bd18-1MKK6 | Brdisv1Bd18-11012730m | Bradi1g75150 | + | 1071 | 356 | 7 | | pseudomolecule_1:68759694..68767134 | |  |
|  | Bd18-1MKK10-1 | Brdisv1Bd18-11001858m | Bradi1g11525 | - | 1044 | 347 | 0 | | pseudomolecule_1:8295466..8296927 | |  |
|  | Bd18-1MKK10-2 | Brdisv1Bd18-11011845m | Bradi1g69400 | + | 1029 | 342 | 0 | | pseudomolecule_1:64925095..64926955 | |  |
|  | Bd18-1MKK10-3 | Brdisv1Bd18-11001742m | Bradi1g10800 | + | 1035 | 344 | 0 | | pseudomolecule_1:7588354..7589802 | |  |
|  | Bd18-1MKK10-4 | Brdisv1Bd18-11001738m | Bradi1g10770 | + | 1026 | 341 | 0 | | pseudomolecule_1:7532339..7533774 | |  |
|  | Bd18-1MKK10-5 | Brdisv1Bd18-11001740m | Bradi1g10790 | - | 999 | 332 | 0 | | pseudomolecule_1:7558156..7560285 | |  |
| S+ | | | | | | | | | | |  |
| Bd30-1 | Bd30-1MKK1 | Brdisv1Bd30-11007680m | Bradi1g51000 | + | 1092 | 363 | 8 | | pseudomolecule_1:36364865..36369496 | |  |
|  | Bd30-1MKK3-1 | Brdisv1Bd30-11035477m | Bradi4g39490 | + | 1572 | 523 | 9 | | pseudomolecule_4:26907017..26915019 | |  |
|  | Bd30-1MKK3-2 | Brdisv1Bd30-11040333m | Bradi1g41860 | - | 1572 | 523 | 10 | | pseudomolecule_6:2002634..2019118 | |  |
|  | Bd30-1MKK3-3 | Brdisv1Bd30-11023309m | Bradi3g11260 | - | 1785 | 594 | 9 | | pseudomolecule_3:8620737..8630167 | |  |
|  | Bd30-1MKK4 | Brdisv1Bd30-11029066m | Bradi3g53650 | + | 768 | 255 | 0 | | pseudomolecule_3:36265792..36268319 | |  |
|  | Bd30-1MKK5 | Brdisv1Bd30-11006970m | Bradi1g46880 | - | 951 | 316 | 2 | | pseudomolecule_1:33100913..33105566 | |  |
|  | Bd30-1MKK6 | Brdisv1Bd30-11011645m | Bradi1g75150 | + | 1056 | 351 | 5 | | pseudomolecule_1:55416943..55421280 | |  |
|  | Bd30-1MKK10-1 | Brdisv1Bd30-11001809m | Bradi1g11525 | - | 1044 | 347 | 0 | | pseudomolecule_1:8113219..8114680 | |  |
|  | Bd30-1MKK10-2 | Brdisv1Bd30-11010689m | Bradi1g69400 | + | 1029 | 342 | 0 | | pseudomolecule_1:51367620..51369480 | |  |
|  | Bd30-1MKK10-3 | Brdisv1Bd30-11001697m | Bradi1g10800 | + | 1035 | 344 | 0 | | pseudomolecule_1:7533341..7534789 | |  |
|  | Bd30-1MKK10-4 | Brdisv1Bd30-11001695m | Bradi1g10770 | + | 1026 | 341 | 0 | | pseudomolecule_1:7513865..7515300 | |  |
|  | Bd30-1MKK10-5 | Brdisv1Bd30-11041837m | Bradi1g10790 | - | 999 | 332 | 0 | | pseudomolecule_6:13791593..13793722 | |  |
| ABR7 | ABR7MKK1 | Brdisv1ABR71008279m | Bradi1g51000 | + | 1047 | 348 | 8 | | pseudomolecule_1:41714772..41720331 | |  |
|  | ABR7MKK3-1 | Brdisv1ABR71029810m | Bradi4g39490 | + | 1572 | 523 | 9 | | pseudomolecule_3:35588258..35596260 | |  |
|  | ABR7MKK3-2 | Brdisv1ABR71020756m | Bradi1g41860 | - | 1572 | 523 | 10 | | pseudomolecule_2:40513550..40530042 | |  |
|  | ABR7MKK3-3 | Brdisv1ABR71032780m | Bradi3g11260 | - | 1779 | 592 | 9 | | pseudomolecule_4:8746197..8755731 | |  |
|  | ABR7MKK4 | Brdisv1ABR71039480m | Bradi3g53650 | + | 1074 | 357 | 0 | | pseudomolecule_4:44866889..44869408 | |  |
|  | ABR7MKK5 | Brdisv1ABR71007538m | Bradi1g46880 | - | 1032 | 343 | 1 | | pseudomolecule_1:38157755..38161844 | |  |
|  | ABR7MKK6 | Brdisv1ABR71012442m | Bradi1g75150 | + | 1071 | 356 | 7 | | pseudomolecule_1:61975090..61982531 | |  |
|  | ABR7MKK10-1 | Brdisv1ABR71001829m | Bradi1g11525 | - | 1044 | 347 | 0 | | pseudomolecule_1:7775058..7776519 | |  |
|  | ABR7MKK10-2 | Brdisv1ABR71011533m | Bradi1g69400 | + | 1029 | 342 | 0 | | pseudomolecule_1:58220320..58222181 | |  |
|  | ABR7MKK10-3 | Brdisv1ABR71001729m | Bradi1g10800 | + | 1035 | 344 | 0 | | pseudomolecule_1:7208347..7209795 | |  |
|  | ABR7MKK10-4 | Brdisv1ABR71001726m | Bradi1g10770 | + | 1026 | 341 | 0 | | pseudomolecule_1:7156822..7158257 | |  |
|  | ABR7MKK10-5 | Brdisv1ABR71001728m | Bradi1g10790 | + | 999 | 332 | 0 | | pseudomolecule_1:7179366..7181495 | |  |
| ABR2 | ABR2MKK1 | Brdisv1ABR21008364m | Bradi1g51000 | + | 1047 | 348 | 8 | | pseudomolecule_1:41350180..41355742 | |  |
|  | ABR2MKK3-1 | Brdisv1ABR21029725m | Bradi4g39490 | + | 1572 | 523 | 9 | | pseudomolecule_3:36075640..36083642 | |  |
|  | ABR2MKK3-2 | Brdisv1ABR21006726m | Bradi1g41860 | - | 1572 | 523 | 10 | | pseudomolecule_1:31843837..31860329 | |  |
|  | ABR2MKK3-3 | Brdisv1ABR21032775m | Bradi3g11260 | - | 1779 | 592 | 9 | | pseudomolecule_4:9113391..9122925 | |  |
|  | ABR2MKK4 | Brdisv1ABR21039238m | Bradi3g53650 | + | 1074 | 357 | 0 | | pseudomolecule_4:42797931..42800450 | |  |
|  | ABR2MKK5 | Brdisv1ABR21007673m | Bradi1g46880 | - | 1032 | 343 | 1 | | pseudomolecule_1:38034917..38039006 | |  |
|  | ABR2MKK6 | Brdisv1ABR21012563m | Bradi1g75150 | + | 1071 | 356 | 7 | | pseudomolecule_1:61478287..61485706 | |  |
|  | ABR2MKK10-1 | Brdisv1ABR21001840m | Bradi1g11525 | - | 1044 | 347 | 0 | | pseudomolecule_1:7952147..7953608 | |  |
|  | ABR2MKK10-2 | Brdisv1ABR21011605m | Bradi1g69400 | + | 1029 | 342 | 0 | | pseudomolecule_1:57516336..57517679 | |  |
|  | ABR2MKK10-3 | Brdisv1ABR21001713m | Bradi1g10800 | + | 1035 | 344 | 0 | | pseudomolecule_1:7254568..7256016 | |  |
|  | ABR2MKK10-4 | Brdisv1ABR21001710m | Bradi1g10770 | + | 1026 | 341 | 0 | | pseudomolecule_1:7212089..7213524 | |  |
|  | ABR2MKK10-5 | Brdisv1ABR21001712m | Bradi1g10790 | + | 999 | 332 | 0 | | pseudomolecule_1:7240382..7242511 | |  |
| Mig3 | Mig3MKK1 | Brdisv1Mig31008255m | Bradi1g51000 | + | 1047 | 348 | 8 | | pseudomolecule_1:39296073..39301635 | |  |
|  | Mig3MKK3-1 | Brdisv1Mig31029163m | Bradi4g39490 | + | 1572 | 523 | 9 | | pseudomolecule_3:33118272..33126274 | |  |
|  | Mig3MKK3-2 | Brdisv1Mig31006643m | Bradi1g41860 | - | 1572 | 523 | 10 | | pseudomolecule_1:30457877..30474369 | |  |
|  | Mig3MKK3-3 | Brdisv1Mig31032176m | Bradi3g11260 | - | 1779 | 592 | 9 | | pseudomolecule_4:8847529..8857063 | |  |
|  | Mig3MKK4 | Brdisv1Mig31038354m | Bradi3g53650 | + | 1074 | 357 | 0 | | pseudomolecule_4:40747807..40750107 | |  |
|  | Mig3MKK5 | Brdisv1Mig31007509m | Bradi1g46880 | - | 1032 | 343 | 1 | | pseudomolecule_1:35874119..35878208 | |  |
|  | Mig3MKK6 | Brdisv1Mig31012305m | Bradi1g75150 | + | 1071 | 356 | 7 | | pseudomolecule_1:58441028..58448468 | |  |
|  | Mig3MKK10-1 | Brdisv1Mig31001882m | Bradi1g11525 | - | 1044 | 347 | 0 | | pseudomolecule_1:7753405..7754866 | |  |
|  | Mig3MKK10-2 | Brdisv1Mig31011374m | Bradi1g69400 | + | 1029 | 342 | 0 | | pseudomolecule_1:54643246..54645106 | |  |
|  | Mig3MKK10-3 | Brdisv1Mig31001763m | Bradi1g10800 | + | 1035 | 344 | 0 | | pseudomolecule_1:7175931..7177379 | |  |
|  | Mig3MKK10-4 | Brdisv1Mig31001757m | Bradi1g10770 | + | 1026 | 341 | 0 | | pseudomolecule_1:7098067..7099502 | |  |
|  | Mig3MKK10-5 | Brdisv1Mig31001759m | Bradi1g10790 | + | 999 | 332 | 0 | | pseudomolecule_1:7120982..7123111 | |  |
| Uni2 | Uni2MKK1 | Brdisv1Uni21007769m | Bradi1g51000 | + | 1047 | 348 | 8 | | pseudomolecule_1:35408118..35413682 | |  |
|  | Uni2MKK3-1 | Brdisv1Uni21028033m | Bradi4g39490 | + | 1572 | 523 | 9 | | pseudomolecule_3:27418356..27426358 | |  |
|  | Uni2MKK3-2 | Brdisv1Uni21042247m | Bradi1g41860 | + | 1572 | 523 | 9 | | pseudomolecule_8:2890546..2905819 | |  |
|  | Uni2MKK3-3 | Brdisv1Uni21030859m | Bradi3g11260 | - | 1779 | 592 | 9 | | pseudomolecule_4:7751034..7760566 | |  |
|  | Uni2MKK4 | Brdisv1Uni21036840m | Bradi3g53650 | + | 1074 | 357 | 0 | | pseudomolecule_4:35519457..35521976 | |  |
|  | Uni2MKK5 | Brdisv1Uni21007057m | Bradi1g46880 | - | 1032 | 343 | 1 | | pseudomolecule_1:32339602..32343691 | |  |
|  | Uni2MKK6 | Brdisv1Uni21011815m | Bradi1g75150 | + | 1071 | 356 | 7 | | pseudomolecule_1:53186831..53194250 | |  |
|  | Uni2MKK10-1 | Brdisv1Uni21001716m | Bradi1g11525 | - | 1044 | 347 | 0 | | pseudomolecule_1:7173390..7174851 | |  |
|  | Uni2MKK10-2 | Brdisv1Uni21010884m | Bradi1g69400 | + | 1029 | 342 | 0 | | pseudomolecule_1:49683566..49685426 | |  |
|  | Uni2MKK10-3 | Brdisv1Uni21001612m | Bradi1g10800 | + | 1035 | 344 | 0 | | pseudomolecule_1:6598077..6599525 | |  |
|  | Uni2MKK10-4 | Brdisv1Uni21001606m | Bradi1g10770 | + | 1026 | 341 | 0 | | pseudomolecule_1:6560030..6561465 | |  |
|  | Uni2MKK10-5 | Brdisv1Uni21001608m | Bradi1g10790 | - | 999 | 332 | 0 | | pseudomolecule_1:6578603..6580732 | |  |
| ABR4 | ABR4MKK1 | Brdisv1ABR41008171m | Bradi1g51000 | + | 1047 | 348 | 8 | | pseudomolecule_1:40207865..40213424 | |  |
|  | ABR4MKK3-1 | Brdisv1ABR41029512m | Bradi4g39490 | + | 1572 | 523 | 9 | | pseudomolecule_3:33513008..33521010 | |  |
|  | ABR4MKK3-2 | Brdisv1ABR41006591m | Bradi1g41860 | - | 1572 | 523 | 10 | | pseudomolecule_1:31265085..31281569 | |  |
|  | ABR4MKK3-3 | Brdisv1ABR41032504m | Bradi3g11260 | - | 1785 | 594 | 9 | | pseudomolecule_4:8977303..8986733 | |  |
|  | ABR4MKK4 | Brdisv1ABR41038836m | Bradi3g53650 | + | 1074 | 357 | 0 | | pseudomolecule_4:42498543..42501062 | |  |
|  | ABR4MKK5 | Brdisv1ABR41007481m | Bradi1g46880 | - | 1032 | 343 | 1 | | pseudomolecule_1:36928586..36932675 | |  |
|  | ABR4MKK6 | Brdisv1ABR41012469m | Bradi1g75150 | + | 1071 | 356 | 7 | | pseudomolecule_1:60323722..60331162 | |  |
|  | ABR4MKK10-1 | Brdisv1ABR41001806m | Bradi1g11525 | - | 1044 | 347 | 0 | | pseudomolecule_1:7638961..7640422 | |  |
|  | ABR4MKK10-2 | Brdisv1ABR41011563m | Bradi1g69400 | + | 1029 | 342 | 0 | | pseudomolecule_1:56816442..56818302 | |  |
|  | ABR4MKK10-3 | Brdisv1ABR41001706m | Bradi1g10800 | + | 1035 | 344 | 0 | | pseudomolecule_1:7118200..7119648 | |  |
|  | ABR4MKK10-4 | Brdisv1ABR41001703m | Bradi1g10770 | + | 1026 | 341 | 0 | | pseudomolecule_1:7067395..7068830 | |  |
|  | ABR4MKK10-5 | Brdisv1ABR41001705m | Bradi1g10790 | + | 999 | 332 | 0 | | pseudomolecule_1:7093264..7095393 | |  |
| S8iiC | S8iiCMKK1 | Brdisv1S8iiC1008085m | Bradi1g51000 | + | 1047 | 348 | 8 | | pseudomolecule_1:38390654..38396217 | |  |
|  | S8iiCMKK3-1 | Brdisv1S8iiC1038719m | Bradi4g39490 | + | 1572 | 523 | 9 | | pseudomolecule_4:30542695..30550687 | |  |
|  | S8iiCMKK3-2 | Brdisv1S8iiC1006489m | Bradi1g41860 | - | 1572 | 523 | 10 | | pseudomolecule_1:30076788..30093280 | |  |
|  | S8iiCMKK3-3 | Brdisv1S8iiC1025083m | Bradi3g11260 | - | 1779 | 592 | 9 | | pseudomolecule_3:8929631..8939165 | |  |
|  | S8iiCMKK4 | Brdisv1S8iiC1031440m | Bradi3g53650 | + | 1068 | 355 | 0 | | pseudomolecule_3:40822123..40824637 | |  |
|  | S8iiCMKK5 | Brdisv1S8iiC1007346m | Bradi1g46880 | - | 1032 | 343 | 1 | | pseudomolecule_1:34979967..34984056 | |  |
|  | S8iiCMKK6 | Brdisv1S8iiC1012258m | Bradi1g75150 | + | 1071 | 356 | 7 | | pseudomolecule_1:57300463..57307903 | |  |
|  | S8iiCMKK10-1 | Brdisv1S8iiC1001792m | Bradi1g11525 | - | 1044 | 347 | 0 | | pseudomolecule_1:7825255..7826716 | |  |
|  | S8iiCMKK10-2 | Brdisv1S8iiC1011300m | Bradi1g69400 | + | 1029 | 342 | 0 | | pseudomolecule_1:53646683..53648543 | |  |
|  | S8iiCMKK10-3 | Brdisv1S8iiC1001688m | Bradi1g10800 | - | 1035 | 344 | 0 | | pseudomolecule_1:7200812..7202260 | |  |
|  | S8iiCMKK10-4 | Brdisv1S8iiC1001680m | Bradi1g10770 | + | 1026 | 341 | 0 | | pseudomolecule_1:7156471..7157906 | |  |
|  | S8iiCMKK10-5 | Brdisv1S8iiC1001689m | Bradi1g10790 | - | 999 | 332 | 0 | | pseudomolecule_1:7216677..7218806 | |  |
| Jer1 | Jer1MKK1 | Brdisv1Jer11007559m | Bradi1g51000 | + | 1047 | 348 | 8 | | pseudomolecule_1:33670640..33676203 | |  |
|  | Jer1MKK3-1 | Brdisv1Jer11027394m | Bradi4g39490 | + | 1572 | 523 | 9 | | pseudomolecule_3:27815563..27823555 | |  |
|  | Jer1MKK3-2 | Brdisv1Jer11041340m | Bradi1g41860 | + | 1314 | 437 | 10 | | pseudomolecule_7:3802318..3818810 | |  |
|  | Jer1MKK3-3 | Brdisv1Jer11030183m | Bradi3g11260 | - | 1779 | 592 | 9 | | pseudomolecule_4:7704623..7714151 | |  |
|  | Jer1MKK4 | Brdisv1Jer11036023m | Bradi3g53650 | + | 1074 | 357 | 0 | | pseudomolecule_4:34628187..34630711 | |  |
|  | Jer1MKK6 | Brdisv1Jer11011416m | Bradi1g75150 | + | 1071 | 356 | 7 | | pseudomolecule_1:50497336..50504776 | |  |
|  | Jer1MKK10-1 | Brdisv1Jer11001692m | Bradi1g11525 | - | 1044 | 347 | 0 | | pseudomolecule_1:6897543..6899004 | |  |
|  | Jer1MKK10-2 | Brdisv1Jer11010524m | Bradi1g69400 | + | 1029 | 342 | 0 | | pseudomolecule_1:47132008..47133868 | |  |
|  | Jer1MKK10-3 | Brdisv1Jer11001582m | Bradi1g10800 | + | 1035 | 344 | 0 | | pseudomolecule_1:6331268..6332716 | |  |
|  | Jer1MKK10-4 | Brdisv1Jer11001579m | Bradi1g10770 | - | 1275 | 424 | 2 | | pseudomolecule_1:6297088..6298895 | |  |
|  | Jer1MKK10-5 | Brdisv1Jer11001581m | Bradi1g10790 | + | 984 | 327 | 1 | | pseudomolecule_1:6319412..6321014 | |  |
| Per1 | Per1MKK1 | Brdisv1Per11008500m | Bradi1g51000 | + | 1092 | 363 | 8 | | pseudomolecule_1:42201637..42206269 | |  |
|  | Per1MKK3-1 | Brdisv1Per11039460m | Bradi4g39490 | + | 1572 | 523 | 9 | | pseudomolecule_4:33594376..33602368 | |  |
|  | Per1MKK3-2 | Brdisv1Per11006708m | Bradi1g41860 | - | 1572 | 523 | 10 | | pseudomolecule_1:32063196..32080934 | |  |
|  | Per1MKK3-3 | Brdisv1Per11025553m | Bradi3g11260 | - | 1779 | 592 | 9 | | pseudomolecule_3:9248175..9257709 | |  |
|  | Per1MKK4 | Brdisv1Per11032157m | Bradi3g53650 | + | 1026 | 341 | 1 | | pseudomolecule_3:43806929..43808501 | |  |
|  | Per1MKK5 | Brdisv1Per11007710m | Bradi1g46880 | - | 1032 | 343 | 1 | | pseudomolecule_1:38469218..38473307 | |  |
|  | Per1MKK6 | Brdisv1Per11012631m | Bradi1g75150 | + | 1071 | 356 | 7 | | pseudomolecule_1:62051863..62059282 | |  |
|  | Per1MKK10-1 | Brdisv1Per11001811m | Bradi1g11525 | - | 1044 | 347 | 0 | | pseudomolecule_1:8182393..8183854 | |  |
|  | Per1MKK10-2 | Brdisv1Per11011705m | Bradi1g69400 | + | 1029 | 342 | 0 | | pseudomolecule_1:58376221..58378081 | |  |
|  | Per1MKK10-3 | Brdisv1Per11001709m | Bradi1g10800 | + | 1035 | 344 | 0 | | pseudomolecule_1:7631695..7633143 | |  |
|  | Per1MKK10-4 | Brdisv1Per11001706m | Bradi1g10770 | + | 1026 | 341 | 0 | | pseudomolecule_1:7591522..7592957 | |  |
|  | Per1MKK10-5 | Brdisv1Per11001708m | Bradi1g10790 | + | 999 | 332 | 0 | | pseudomolecule_1:7614575..7616704 | |  |
| ABR6 | ABR6MKK1 | Brdisv1ABR6_r1008357m | Bradi1g51000 | + | 1047 | 348 | 8 | | pseudomolecule_1:45299627..45305186 | |  |
|  | ABR6MKK3-1 | Brdisv1ABR6_r1030162m | Bradi4g39490 | + | 1572 | 523 | 9 | | pseudomolecule_3:40601765..40609757 | |  |
|  | ABR6MKK3-2 | Brdisv1ABR6_r1006674m | Bradi1g41860 | - | 1572 | 523 | 10 | | pseudomolecule_1:34730513..34747005 | |  |
|  | ABR6MKK3-3 | Brdisv1ABR6_r1033144m | Bradi3g11260 | - | 1779 | 592 | 9 | | pseudomolecule_4:9161963..9171490 | |  |
|  | ABR6MKK4 | Brdisv1ABR6_r1039790m | Bradi3g53650 | + | 1074 | 357 | 0 | | pseudomolecule_4:47945198..47947722 | |  |
|  | ABR6MKK5 | Brdisv1ABR6_r1007553m | Bradi1g46880 | - | 1032 | 343 | 1 | | pseudomolecule_1:41195522..41199611 | |  |
|  | ABR6MKK6 | Brdisv1ABR6_r1012715m | Bradi1g75150 | + | 1071 | 356 | 7 | | pseudomolecule_1:67999213..68006653 | |  |
|  | ABR6MKK10-1 | Brdisv1ABR6_r1001837m | Bradi1g11525 | - | 1044 | 347 | 0 | | pseudomolecule_1:8307546..8309007 | |  |
|  | ABR6MKK10-2 | Brdisv1ABR6_r1011767m | Bradi1g69400 | + | 1029 | 342 | 0 | | pseudomolecule_1:63859775..63861635 | |  |
|  | ABR6MKK10-3 | Brdisv1ABR6_r1001726m | Bradi1g10800 | + | 1035 | 344 | 0 | | pseudomolecule_1:7673067..7674515 | |  |
|  | ABR6MKK10-4 | Brdisv1ABR6_r1001722m | Bradi1g10770 | + | 1026 | 341 | 0 | | pseudomolecule_1:7621851..7623286 | |  |
|  | ABR6MKK10-5 | Brdisv1ABR6_r1001717m | Bradi1g10790 | - | 999 | 332 | 0 | | pseudomolecule_1:7592251..7594380 | |  |
| Luc1 | Luc1MKK1 | Brdisv1Luc11008350m | Bradi1g51000 | + | 1047 | 348 | 8 | | pseudomolecule_1:42690734..42696296 | |  |
|  | Luc1MKK3-1 | Brdisv1Luc11040018m | Bradi4g39490 | + | 1572 | 523 | 9 | | pseudomolecule_4:35407862..35415864 | |  |
|  | Luc1MKK3-2 | Brdisv1Luc11006718m | Bradi1g41860 | - | 1572 | 523 | 10 | | pseudomolecule_1:33191716..33208208 | |  |
|  | Luc1MKK3-3 | Brdisv1Luc11025812m | Bradi3g11260 | - | 1779 | 592 | 9 | | pseudomolecule_3:9027728..9037262 | |  |
|  | Luc1MKK4 | Brdisv1Luc11032413m | Bradi3g53650 | + | 1074 | 357 | 0 | | pseudomolecule_3:44640387..44642906 | |  |
|  | Luc1MKK5 | Brdisv1Luc11007623m | Bradi1g46880 | - | 1032 | 343 | 1 | | pseudomolecule_1:39066814..39070903 | |  |
|  | Luc1MKK6 | Brdisv1Luc11012565m | Bradi1g75150 | + | 1071 | 356 | 7 | | pseudomolecule_1:63081247..63088687 | |  |
|  | Luc1MKK10-1 | Brdisv1Luc11001815m | Bradi1g11525 | - | 1044 | 347 | 0 | | pseudomolecule_1:8004548..8006009 | |  |
|  | Luc1MKK10-2 | Brdisv1Luc11011611m | Bradi1g69400 | + | 1029 | 342 | 0 | | pseudomolecule_1:59257636..59259496 | |  |
|  | Luc1MKK10-3 | Brdisv1Luc11001706m | Bradi1g10800 | + | 1035 | 344 | 0 | | pseudomolecule_1:7408664..7410112 | |  |
|  | Luc1MKK10-4 | Brdisv1Luc11001703m | Bradi1g10770 | + | 1026 | 341 | 0 | | pseudomolecule_1:7367986..7369421 | |  |
|  | Luc1MKK10-5 | Brdisv1Luc11001705m | Bradi1g10790 | + | 999 | 332 | 0 | | pseudomolecule_1:7390849..7392978 | |  |
| Mur1 | Mur1MKK1 | Brdisv1Mur11007592m | Bradi1g51000 | + | 1047 | 348 | 8 | | pseudomolecule_1:32829769..32835331 | |  |
|  | Mur1MKK3-1 | Brdisv1Mur11026956m | Bradi4g39490 | + | 1572 | 523 | 9 | | pseudomolecule_3:25840911..25848913 | |  |
|  | Mur1MKK3-2 | Brdisv1Mur11040779m | Bradi1g41860 | - | 1572 | 523 | 10 | | pseudomolecule_8:2074066..2090558 | |  |
|  | Mur1MKK3-3 | Brdisv1Mur11029722m | Bradi3g11260 | - | 1779 | 592 | 9 | | pseudomolecule_4:7437065..7446596 | |  |
|  | Mur1MKK4 | Brdisv1Mur11035455m | Bradi3g53650 | + | 882 | 293 | 0 | | pseudomolecule_4:33000634..33002692 | |  |
|  | Mur1MKK5 | Brdisv1Mur11006903m | Bradi1g46880 | - | 1032 | 343 | 1 | | pseudomolecule_1:29951114..29955203 | |  |
|  | Mur1MKK6 | Brdisv1Mur11011361m | Bradi1g75150 | + | 1071 | 356 | 7 | | pseudomolecule_1:48900963..48908403 | |  |
|  | Mur1MKK10-1 | Brdisv1Mur11001679m | Bradi1g11525 | - | 1044 | 347 | 0 | | pseudomolecule_1:6720337..6721798 | |  |
|  | Mur1MKK10-2 | Brdisv1Mur11010501m | Bradi1g69400 | + | 1029 | 342 | 0 | | pseudomolecule_1:45654186..45656047 | |  |
|  | Mur1MKK10-3 | Brdisv1Mur11001569m | Bradi1g10800 | + | 1035 | 344 | 0 | | pseudomolecule_1:6176676..6178124 | |  |
|  | Mur1MKK10-4 | Brdisv1Mur11001565m | Bradi1g10770 | + | 1026 | 341 | 0 | | pseudomolecule_1:6152320..6153755 | |  |
|  | Mur1MKK10-5 | Brdisv1Mur11044815m | Bradi1g10790 | + | 567 | 188 | 2 | | pseudomolecule_8:43346431..43352617 | |  |
| ABR3 | ABR3MKK1 | Brdisv1ABR31008332m | Bradi1g51000 | + | 1092 | 363 | 8 | | pseudomolecule_1:41159097..41163729 | |  |
|  | ABR3MKK3-1 | Brdisv1ABR31039196m | Bradi4g39490 | + | 1572 | 523 | 9 | | pseudomolecule_4:35056134..35064136 | |  |
|  | ABR3MKK3-2 | Brdisv1ABR31006634m | Bradi1g41860 | - | 1572 | 523 | 10 | | pseudomolecule_1:31571071..31587563 | |  |
|  | ABR3MKK3-3 | Brdisv1ABR31025404m | Bradi3g11260 | - | 1779 | 592 | 9 | | pseudomolecule_3:8772405..8781939 | |  |
|  | ABR3MKK4 | Brdisv1ABR31037814m | Bradi3g53650 | + | 1074 | 357 | 0 | | pseudomolecule_4:28684180..28686573 | |  |
|  | ABR3MKK5 | Brdisv1ABR31007650m | Bradi1g46880 | - | 1032 | 343 | 1 | | pseudomolecule_1:37864510..37868599 | |  |
|  | ABR3MKK6 | Brdisv1ABR31012480m | Bradi1g75150 | + | 1071 | 356 | 7 | | pseudomolecule_1:61156791..61164231 | |  |
|  | ABR3MKK10-1 | Brdisv1ABR31001837m | Bradi1g11525 | - | 1044 | 347 | 0 | | pseudomolecule_1:7880450..7881911 | |  |
|  | ABR3MKK10-2 | Brdisv1ABR31011561m | Bradi1g69400 | + | 1029 | 342 | 0 | | pseudomolecule_1:57490324..57492184 | |  |
|  | ABR3MKK10-3 | Brdisv1ABR31001729m | Bradi1g10800 | + | 1035 | 344 | 0 | | pseudomolecule_1:7315002..7316450 | |  |
|  | ABR3MKK10-4 | Brdisv1ABR31001726m | Bradi1g10770 | + | 1026 | 341 | 0 | | pseudomolecule_1:7281324..7282759 | |  |
|  | ABR3MKK10-5 | Brdisv1ABR31001728m | Bradi1g10790 | + | 999 | 332 | 0 | | pseudomolecule_1:7308995..7311124 | |  |
| ABR5 | ABR5MKK1 | Brdisv1ABR51007823m | Bradi1g51000 | + | 1047 | 348 | 8 | | pseudomolecule_1:36045166..36050728 | |  |
|  | ABR5MKK3-1 | Brdisv1ABR51028122m | Bradi4g39490 | + | 1572 | 523 | 9 | | pseudomolecule_3:31279385..31287387 | |  |
|  | ABR5MKK3-2 | Brdisv1ABR51006276m | Bradi1g41860 | - | 1572 | 523 | 10 | | pseudomolecule_1:28121630..28138122 | |  |
|  | ABR5MKK3-3 | Brdisv1ABR51030947m | Bradi3g11260 | - | 1779 | 592 | 9 | | pseudomolecule_4:7988291..7997825 | |  |
|  | ABR5MKK4 | Brdisv1ABR51037002m | Bradi3g53650 | + | 1074 | 357 | 0 | | pseudomolecule_4:37570158..37572682 | |  |
|  | ABR5MKK5 | Brdisv1ABR51007117m | Bradi1g46880 | - | 933 | 310 | 1 | | pseudomolecule_1:32965648..32969883 | |  |
|  | ABR5MKK6 | Brdisv1ABR51011697m | Bradi1g75150 | + | 1071 | 356 | 7 | | pseudomolecule_1:53834226..53841666 | |  |
|  | ABR5MKK10-1 | Brdisv1ABR51001752m | Bradi1g11525 | - | 1044 | 347 | 0 | | pseudomolecule_1:7293621..7295082 | |  |
|  | ABR5MKK10-2 | Brdisv1ABR51010777m | Bradi1g69400 | + | 1011 | 336 | 1 | | pseudomolecule_1:50311409..50313334 | |  |
|  | ABR5MKK10-3 | Brdisv1ABR51001647m | Bradi1g10800 | + | 1035 | 344 | 0 | | pseudomolecule_1:6721870..6723318 | |  |
|  | ABR5MKK10-5 | Brdisv1ABR51001637m | Bradi1g10790 | + | 999 | 332 | 0 | | pseudomolecule_1:6660034..6662163 | |  |
| RON2 | RON2MKK1 | Brdisv1RON21008490m | Bradi1g51000 | + | 1047 | 348 | 8 | | pseudomolecule_1:45657368..45662927 | |  |
|  | RON2MKK3-1 | Brdisv1RON21040413m | Bradi4g39490 | + | 1572 | 523 | 9 | | pseudomolecule_4:40543599..40551601 | |  |
|  | RON2MKK3-2 | Brdisv1RON21006777m | Bradi1g41860 | + | 1572 | 523 | 10 | | pseudomolecule_1:34882433..34898925 | |  |
|  | RON2MKK3-3 | Brdisv1RON21025893m | Bradi3g11260 | - | 1779 | 592 | 9 | | pseudomolecule_3:9966668..9976202 | |  |
|  | RON2MKK4 | Brdisv1RON21032486m | Bradi3g53650 | + | 1074 | 357 | 0 | | pseudomolecule_3:48068045..48070564 | |  |
|  | RON2MKK5 | Brdisv1RON21007695m | Bradi1g46880 | - | 1032 | 343 | 1 | | pseudomolecule_1:41646928..41651017 | |  |
|  | RON2MKK6 | Brdisv1RON21012535m | Bradi1g75150 | + | 1071 | 356 | 7 | | pseudomolecule_1:66536209..66543649 | |  |
|  | RON2MKK10-1 | Brdisv1RON21001840m | Bradi1g11525 | - | 1044 | 347 | 0 | | pseudomolecule_1:8470689..8472150 | |  |
|  | RON2MKK10-2 | Brdisv1RON21011610m | Bradi1g69400 | + | 1029 | 342 | 0 | | pseudomolecule_1:62669320..62671180 | |  |
|  | RON2MKK10-3 | Brdisv1RON21001729m | Bradi1g10800 | + | 1035 | 344 | 0 | | pseudomolecule_1:7783530..7784978 | |  |
|  | RON2MKK10-4 | Brdisv1RON21001725m | Bradi1g10770 | + | 1026 | 341 | 0 | | pseudomolecule_1:7733403..7734838 | |  |
|  | RON2MKK10-5 | Brdisv1RON21001727m | Bradi1g10790 | - | 999 | 332 | 0 | | pseudomolecule_1:7763190..7765319 | |  |
| Foz1 | Foz1MKK1 | Brdisv1Foz11007784m | Bradi1g51000 | + | 1047 | 348 | 8 | | pseudomolecule_1:35880391..35885953 | |  |
|  | Foz1MKK3-1 | Brdisv1Foz11037008m | Bradi4g39490 | + | 1572 | 523 | 9 | | pseudomolecule_4:29883738..29891740 | |  |
|  | Foz1MKK3-2 | Brdisv1Foz11006306m | Bradi1g41860 | - | 1572 | 523 | 10 | | pseudomolecule_1:28346286..28362778 | |  |
|  | Foz1MKK3-3 | Brdisv1Foz11023762m | Bradi3g11260 | - | 1779 | 592 | 9 | | pseudomolecule_3:7984027..7993561 | |  |
|  | Foz1MKK5 | Brdisv1Foz11007091m | Bradi1g46880 | - | 987 | 328 | 2 | | pseudomolecule_1:32942815..32947034 | |  |
|  | Foz1MKK6 | Brdisv1Foz11011624m | Bradi1g75150 | + | 1071 | 356 | 7 | | pseudomolecule_1:53352265..53359705 | |  |
|  | Foz1MKK10-1 | Brdisv1Foz11001741m | Bradi1g11525 | - | 1044 | 347 | 0 | | pseudomolecule_1:7272765..7274226 | |  |
|  | Foz1MKK10-2 | Brdisv1Foz11010724m | Bradi1g69400 | + | 1029 | 342 | 0 | | pseudomolecule_1:49805296..49807156 | |  |
|  | Foz1MKK10-3 | Brdisv1Foz11001631m | Bradi1g10800 | + | 1035 | 344 | 0 | | pseudomolecule_1:6695916..6697364 | |  |
|  | Foz1MKK10-4 | Brdisv1Foz11001629m | Bradi1g10770 | + | 1026 | 341 | 0 | | pseudomolecule_1:6669183..6670618 | |  |
|  | Foz1MKK10-5 | Brdisv1Foz11042773m | Bradi1g10790 | + | 999 | 332 | 0 | | pseudomolecule_8:12947650..12949651 | |  |
| Sig2 | Sig2MKK1 | Brdisv1Sig21007754m | Bradi1g51000 | + | 1047 | 348 | 8 | | pseudomolecule_1:35791126..35796688 | |  |
|  | Sig2MKK3-1 | Brdisv1Sig21027513m | Bradi4g39490 | + | 1572 | 523 | 9 | | pseudomolecule_3:30069684..30077686 | |  |
|  | Sig2MKK3-2 | Brdisv1Sig21006212m | Bradi1g41860 | - | 1572 | 523 | 10 | | pseudomolecule_1:27899395..27915887 | |  |
|  | Sig2MKK3-3 | Brdisv1Sig21030257m | Bradi3g11260 | - | 1779 | 592 | 9 | | pseudomolecule_4:7592653..7602187 | |  |
|  | Sig2MKK4 | Brdisv1Sig21036157m | Bradi3g53650 | + | 720 | 239 | 1 | | pseudomolecule_4:35081644..35082688 | |  |
|  | Sig2MKK5 | Brdisv1Sig21007006m | Bradi1g46880 | - | 1032 | 343 | 1 | | pseudomolecule_1:32570411..32574500 | |  |
|  | Sig2MKK6 | Brdisv1Sig21011649m | Bradi1g75150 | + | 1071 | 356 | 7 | | pseudomolecule_1:53049823..53057263 | |  |
|  | Sig2MKK10-1 | Brdisv1Sig21001691m | Bradi1g11525 | - | 1044 | 347 | 0 | | pseudomolecule_1:6765444..6766905 | |  |
|  | Sig2MKK10-2 | Brdisv1Sig21010766m | Bradi1g69400 | + | 1029 | 342 | 0 | | pseudomolecule_1:49648154..49650014 | |  |
|  | Sig2MKK10-3 | Brdisv1Sig21001588m | Bradi1g10800 | + | 1035 | 344 | 0 | | pseudomolecule_1:6215125..6216573 | |  |
|  | Sig2MKK10-4 | Brdisv1Sig21001585m | Bradi1g10770 | + | 729 | 242 | 0 | | pseudomolecule_1:6193198..6194213 | |  |
|  | Sig2MKK10-5 | Brdisv1Sig21045238m | Bradi1g10790 | + | 1257 | 418 | 4 | | pseudomolecule_8:53142262..53146267 | |  |
