# Supplementary material for: Comparative analysis of MAPK and MKK gene families reveals differential evolutionary patterns in Brachypodium distachyon inbred lines
Source: PeerJ. 2021 Apr 6;9:e11238. doi: 10.7717/peerj.11238 (PMC8034371; doi:10.7717/peerj.11238)
Supplement: Supplemental Information 14 [file peerj-09-11238-s014.doc]

**Table S3 The list of qRT-PCR primers of MPK and MKK genes in three *B. distachyon* inbred lines selected.**

| Gene | Inbred lines | ForwardPrimer | RewardPrimer |
| --- | --- | --- | --- |
| MKK1 | Bd21,Bd30-1,TR8i | CAAATTCCTGACGCAGAGCG | CGAACCAGTTGCACGATTCC |
| MKK3-1 | Bd21,Bd30-1,TR8i | ACATTCCAAGCAAGCGGGTA | CCAATAAGGTTCCCACCATG |
| MKK3-2 | Bd21,Bd30-1,TR8i | GAGATCCATGGGGTGCTACT | AAGGTTTCCACCATGACAGC |
| MKK3-3 | Bd21,Bd30-1,TR8i | ATAGCCATCGAGGAATCGC | ATGCAATGCACTTCCTCTC |
| MKK4 | Bd21,Bd30-1,TR8i | AGGTTCGTCCCTATCGTCCA | GGTTCCCGTAGAGCACCTTG |
| MKK5 | Bd21,Bd30-1,TR8i | TGAAGCAGCTCTACGGGAAC | TTGGATTGATCCTGGGACCG |
| MKK6 | Bd21,TR8i | TGGCCGCTTCCCCTATACTC | CGCGGGATCCTTTTGTATG |
| MKK6 | Bd30-1 | TGGCCGCTTCCCCTATACTC | AGAATAATGTTTAGCATAC |
| MKK10-1 | Bd21,TR8i | GTACGCCCTCAAGGTGCAG | GGAACTGGGAGAACGCCTC |
| MKK10-1 | Bd30-1 | GTACGCGCTCAAGGTGCAG | GGAACTGGGAGAACGCCTC |
| MKK10-2 | Bd21,Bd30-1,TR8i | GGGACTTGTAGGCGAAACGA | CGCCACATGGGACAGGTTAT |
| MKK10-3 | Bd21,Bd30-1,TR8i | GTGCTGTCTGACGGTGAGG | CAACAAACGGGTGAGCCAG |
| MKK10-5 | Bd21,Bd30-1,TR8i | GGAGCTCAGGGGATTCATCG | CCATATTCCTCCCGGCGAC |
| MPK3 | Bd21,Bd30-1,TR8i | TCCCCTCCAGAGGATCACAG | TGGTCTTCCGTCAAAGGGTG |
| MPK4 | Bd21,Bd30-1,TR8i | TCAGCCATTGACCGATGACC | CAAGCCCAAAGTCCGCAATC |
| MPK6 | Bd21,Bd30-1,TR8i | TGGAGCTCAGTTTCGGCATT | TACGCAGAAGTTCGACCACC |
| MPK7-1 | Bd21,Bd30-1,TR8i | GCCTCTTCAGATCTGCTGCT | TCTTCCGATGGGCTTGATCG |
| MPK11 | Bd21,Bd30-1,TR8i | TGTGCACTCAGCGAATGTCT | CCGATACCACCGGGTAACAA |
| MPK14 | Bd21,Bd30-1,TR8i | GCCCATCGACCTCGACTTAG | ATCGCAGTAGGGCCTTTGAG |
| MPK16 | Bd21,Bd30-1,TR8i | ACTTGAGCAGTATGAGGCGG | GCGGAAGGCTCTCTATCCAC |
| MPK17 | Bd21,Bd30-1,TR8i | AGAAAGAGGTTCTCCGCTGC | TTGCGCTCTTCTGGTAGCTC |
| MPK20-1 | Bd21,Bd30-1,TR8i | CGTATTGGCCCATCAAGGGA | TACGGATGACTGGTGCCAAC |
| MPK20-2 | Bd21,Bd30-1,TR8i | GCTCTGTGGATCCTTTTTCACG | TACGGACCCGCGAAATTGTA |
| MPK20-3 | Bd21,Bd30-1,TR8i | CTGCCAAGGCAAGCCTTCAAC | CAGGCACATGTTGTGGTGGG |
| MPK20-4 | Bd21,Bd30-1,TR8i | AACTGGGAAGCCTTTGTTCC | AGGGTCTGCTTTGGGGAATC |
| MPK20-5 | Bd21,Bd30-1,TR8i | CGGTTGCATTTTTGCGGAGA | GCGCTGAAGGATCTGCATTG |
| MPK21-1 | Bd21,Bd30-1,TR8i | AAGCATGGAGATGGTGACTACA | CTCCGAAAGGGAATCCTCATC |
| MPK21-2 | Bd21,Bd30-1,TR8i | TCAGGCAAACTTTGTTTACCC | CTCATCACCGATGCCAATA |
| Actin | Bd21,Bd30-1,TR8i | CCCGATGGACAGGTTATCACTA | ATAGAGCCACCAATCCAAACAC |
