# Supplementary material for: Comparative analysis of MAPK and MKK gene families reveals differential evolutionary patterns in Brachypodium distachyon inbred lines
Source: PeerJ. 2021 Apr 6;9:e11238. doi: 10.7717/peerj.11238 (PMC8034371; doi:10.7717/peerj.11238)
Supplement: Supplemental Information 15 [file peerj-09-11238-s015.docx]

>Arn1MPK3 Brdisv1Arn11011015m.p

MDGAPVAEFRPTMTHGGRFLLYNIFGNQFEITAKYQPPIMPIGRGAYGIVCSVMNFETRE

MVAIKKIANAFDNNMDAKRTLREIKLLRHLDHENIVGLRDVIPPAIPQSFNDVYIATELM

DTDLHHIIRSNQELSEEHCQYFLYQLLRGLKYIHSANVIHRDLKPSNLLLNANCDLKICD

FGLARPSSESDMMTEYVVTRWYRAPELLLNSTDYSAAIDVWSVGCIFMELINRAPLFPGR

DHMHQMRLITEVIGTPTDDDLGFIRNEDARRYMRHLPQFPRRPFPAQFPRVQPAALDLIE

RMLAFNPLQRITVEEALEHPYLERLHDIADEPICTDPFSFDFEQHPLTEDQMKQLIFNEA

LELNPNFRY*

>Arn1MPK4 Brdisv1Arn11035810m.p

MDTSGGGGGGAGGAAQIQGMATHGGRYVLYNVYGNLFEVASKYAPPIRPIGRGAYGIVCA

AVSSDTGEEVAIKKIGNAFDNHIDAKRTLREIKLLRHMDHENIIAIKDIIRPPRRDDFKD

VYIVTELMDTDLHQIIRSNQPLTDDHCQYFLYQLLRGLKYVHSANVLHRDLKPSNLFLNA

NCDLKIADFGLARTTSETDLMTEYVVTRWYRAPELLLNCSQYTAAIDVWSVGCILGEIIT

RQPLFPGRDYIQQLKLITELIGSPDDSSLGFLRSDNARRYMKQLPQYPRQDFRLRFRNMS

DGAVDLLERMLVFDPSRRITVDEALHHPYLASLHDINEEPTCPAPFSFDFEQPSFTEEHM

KELIWRETLAFNPDPPY*

>Arn1MPK6 Brdisv1Arn11008028m.p

MDGGAQPPDAEMADAGAAVPGAGAGAAGTMENIQATLSHGGRFIQYNIFGNVFEVTVKYK

PPILPIGKGAYGIVCSALNSETGEQVAIKKIANAFDNKIDAKRTLREIKLLRHMDHENIV

AIRDIIPPAQRNSFNDVYIAYELMDTDLHQIIRSNQALSEEHCQYFLYQILRGLKYIHSA

NVLHRDLKPSNLLLNANCDLKICDFGLARTTSETDFMTEYVVTRWYRAPELLLNSSEYTA

AIDVWSVGCIFMELMDRKPLFPGRDHVHQLRLLMELIGTPNEADLDFVNENARRYIRQLP

RHARQSFSEKFPHVHPSAIDLVEKMLTFDPRQRITVEGALAHPYLASLHDISDEPVCTMP

FSFDFEQHALSEEQMKDLIHQEALAFNPDYQ*

>Arn1MPK7-1 Brdisv1Arn11046100m.p

LLRGLKYLHSAGILHRDLKPGNLLVNANCDLKICDFGLARTNNTKGQFMTEYVVTRWYRA

PELLLCCDNYGTSIDVWSVGCIFAELLGRKPIFPRTQCLDQLKLIVNVLGTMSENDLEFI

DNPKARKYIKSLPYTPGTPLTSMYPQAHPLAIDLLQKMLVFDPSKRISVTEALEHPYMSP

LYDPSANPPAQVPIDLDIDENLGVEMIREMLWQEMLQYHPEAARMVNM*

>Arn1MPK11 Brdisv1Arn11033533m.p

MRMEGGGAGPAAAAAAGGAHGLGEAQIKGTLTHGGRYVQYNVYGNLFEVSAKYVPPIRPV

GRGACGIICAAINVQTREEVAIKKIGNAFDNQIDAKRTLREVKLLRHMNHENVISIKDII

RPPRRENFNDVYIVYELMDTDLHHLLRSNQPLTDDHCQYFLYQVLRGLKYVHSANVLHRD

LRPSNLLLNAKCDLKIGDFGLARTTTETDFMMEYVVTRWYRAPELLLNCSEYTGAIDMWS

VGCILGEIATREPLFPGKDYVHQLRLITELLGSPDDTSLGFLRSDNARRYVRSLPQYPKQ

QFRSRFPNMSSGAMDLLERMLVFDPNKRITVDEALCHPYLASLHEINDEPVCPAPFSFDF

EQPSFTEEDIKELIWRESVKFNPEPIH*

>Arn1MPK14 Brdisv1Arn11031405m.p

MAMLVDPPNGMGNQGKHYYSMWQTLFEIDTKYVPIKPIGRGAYGIVCSSINRETNEKVAI

KKIHNVFDNRVDALRTLRELKLLRHLRHENVISLKDIMMPVQRRSFKDVYLVYELMDTDL

HQIIKSPQGLSNDHCQYFLFQLLRGLKYLHSAEILHRDLKPGNLLVNANCDLKICDFGLA

RTNSSKGQFMTEYVVTRWYRAPELLLCCDNYGTSIDVWSVGCIFAELLGRKPIFPGTECL

NQLKLIVNVLGTMSESDLEFIDNPKARRYIKTLPYTPGVPLASMYPHAHPLAIDLLQKML

IFDPTKRISVTEALEHPYMSPLYDPSANPPAQVPIDLDLDENISADMIREMMWQEMLHYH

PEAATAISM*

>Arn1MPK16 Brdisv1Arn11018776m.p

MDFFTEYGEGNRYKIEEVIGKGSYGVVCSALDTHTGDKVAIKKINDIFEHVSDATRILRE

IKLLRLLRHPDIVEIKHILLPPSRREFKDIYVVFELMESDLHQVIKANDDLTPEHYQFFL

YQLLRGLKYIHTANVFHRDLKPKNILANADCKLKICDFGLARVAISDTPTAIFWTDYIAT

RWYRAPELCGSFFSKYTPAIDIWSIGCIFAELLTGKPLFPGKNVVHQLDIITDLLGTPSA

ETIARIRNEKARRYLSSMRRKKTVPFTQKFPNADPLALNLLERMLAFDPKDRPSAEEALA

DPYFKNIASVDREPSAQPITKLEFEFERRRITKDDIRELIYREILEYHPNMLREFLEGAE

PTGFMYPSAVDHFKKQFTFLEEHYAKGSTAAPPERQHNSLPRPSVIYSDNRPQNAANITD

DLSRCAIRDNTQKAQKDSASVGANRIPHGAAAAAARPGKVVGSVLRYGNCSTSGTEQYEQ

RRVVRSPGIAPNGVTSGSSYPRRNNTCKSETGEAERIDTNQGGPPKPYVANKLPATVDGR

NGHW*

>Arn1MPK17 Brdisv1Arn11005910m.p

MVGGGGIVDGFRRLFHRRTPSGSGPSSNQSSAGEESSDIDAVVEDLDLVGLRAIRVPKRK

MPLPVESHKKNILEKEFFTEYGEASQYQIQEVIGKGSYGVVAAAVDTRTGERVAIKKIND

VFEHVSDATRILREVKLLRLLRHPDVVEIKHIMLPPSRREFQDIYVVFELMESDLHQVIR

ANDDLTPEHYQFFLYQLLRALKYIHAANVFHRDLKPKNILANSDCKLKICDFGLARVSFN

DAPTAIFWTDYVATRWYRAPELCGSFFSKYTPAIDIWSIGCIFAELLTGRPLFPGKNVVH

QLDIITDLLGTPSSETLSRIRNEKARRYLSCMRKKHPVPLTQKFPNVDPLALRLLGRLLA

FDPKDRPSAEEALADPYFAVLANVEREPSRNPISKLEFEFERQKVTKDDVRELIYREILE

YHPQMLQEYMQGGEQLSFLYPSGVDRFKRQFAHLEENYSKGERGSPLQRKHASLPRQRVG

ASNDNNEQHASDQERGADSIAPATGNPPGSQDAGQQHVSGGQNGVGSTNISPRSYQKSAS

ISASKCVVVNANKNPEYDDDISEEMEGAVDGLSEKVSRMHP*

>Arn1MPK20-1 Brdisv1Arn11020271m.p

MQQDQRKKSSAEAEFFTEYGDASRYKIQEIVGKGSYGVVCSAIDVHTGEKVAIKKIHDIF

EHISDAARILREIKLLRLLRHPDIVEIKHIMLPPSRRDFKDIYVVFELMESDLHQVIKAN

DDLTKEHYQFFLYQLLRALKYIHTANVYHRDLKPKNILANSNCKLKICDFGLARVAFNDT

PTTIFWTDYVATRWYRAPELCGSFFSKYTPAIDVWSIGCIFAEVLTGKPLFPGKNVVHQL

DLMTDLLGTPSMDTISRVRNEKARRYLSSMRKKEPIPFSQKFPNADPLALDLLQRLLAFD

PKDRPTAGEALSHPYFKGLAKVEREPSCQPITKMEFEFERRRATKEDIRELIFREILEYH

PQLLKDYINGTERTTFLYPSAVDQFRKQFAHLEENSESGPVVPMDRKHTSLPRSTIVHSA

PIHAKEQPRIGPSRDRPLTDESYKNPRDSEKYSGNVPRSSHHSQAPQRVPTARPGRVVGP

VLPYENVGTSHPYDPRRVAMNSGYPPQQQIPQTYGYYQTTGKSACSEPSQAERYTLHQQA

YACANSSTAPDVALDMRAPPFHQSAGPKSGSSDRLTAETNLYTRSLNGIVATTSGVAASA

HRKVSVVPYGMSQMY*

>Arn1MPK20-2 Brdisv1Arn11015876m.p

MQQPQQQGQRNKSTDVDFFSEYGDANRYRIQEVIGKGSYGVVCSAMDMQTRQKVAIKKIH

NIFDHVSDAARILREIKLLRLLRHPDVVEIKHIMLPPSRKDFKDIYVVFELMESDLHQVI

KANDDLTKEHYQFFLYQLLRALKYIHTASVYHRDLKPKNILANSNCKLKICDFGLARVAF

NDTPTTVFWTDYVATRWYRAPELCGSFFTKYTPAIDIWSIGCIFAEVLTGKPLFPGKNVV

HQLDLMTDLLGTPSMDTISRVRNEKARRYLSSMRKKDPVPFSKKFPNADPLGLKLLEKLL

AFDPKDRPTAEEALTDPYFKSLSKPDREPSCQPIRKVEFDFEHRRMSKDDIRELIFQEIL

EYHPQLLKNYIDGTEKTTFLYPSAVDQFKKQFSHLEESDGSGPVVPTERKHASLPRSTTV

HSTPIPAKEQPLVASSRGRPIANEPCKPWAPGNVPGASQTAHVAQAGRAVGVGSVPPYES

GSGKYPYDATSRPAVSSGYPPQQKIPQTYGYHHHHQTPGAGQSSQAMGGYACGYTKGTTP

PPAAAQDMRASPYHHRSAGTKNDPLNRLAAESDIYTRSLNGIVAAAASAGTGAHRKVGAV

PFGMSGMY*

>Arn1MPK20-3 Brdisv1Arn11006673m.p

MQHGDLHKKSAAEMDFFTAYDDANRYKILEVIGKGSYGLVCSANDLQTGEKVAIKKIHNI

FEHISDAARILREIKLLRLLRHPDVVEIKHILLPPSKKDFKDIYVVFELMESDLHQVIKA

NDDLTREHYQFFLYQMLRALKYMHTANVYHRDLKPKNVLANANCKLKICDFGLARVAFSD

APTTVFWTDYVATRWYRAPELCGSFYSKYTPAIDIWSIGCIFAEVLIGKPLFPGKNVVHQ

LDLITDILGTPSLDAISRVRNDKARKYLTCMRKKQPASFSQKFPKADPLALQLLRRLLAF

DPKDRPSAEEALADPYFNGLAKVEREPSCQPIPKIEFEFEGRRVTKEDIKELIFEEILEY

HPQLLKEHIIGKERPNFVHLSAVDQFKKHFTQLEENDNETGAAVSLQRKHSSLPRQAFNH

R*

>Arn1MPK20-4 Brdisv1Arn11020500m.p

MQTTEQQRKKGSSEMDFFSEYGDANRYKIQEVIGKGSYGVVCSAIDQHTGDKVAIKKIHN

IFEHLSDAARILREIKLLRLLRHPDIVEIRHIMLPPSRRDFKDIYVVFELMDTDLHQVIK

ANDDLTKEHHQFFLYQMLRALKYIHTANVYHRDLKPKNILANANCKLKICDFGLARVAFN

DTPTTVFWTDYVATRWYRAPELCGSFFTKYSRAIDIWSIGCIFAEILTGKPLFPGKNVVH

QLDLMTDLLGTPSLDTVSRIRNEKARRYLSSMRKKQSVSFSERFPKADPAALKLLQRLLA

FDPKDRPTAEEALADPYFKGLGKVEREPSCQPITKMEFEFERKNVTKADVKELIFREILE

YHPQLLKDYMNGTEKTNFLYPSAVDNFRRQFANLEENGGKGGAVVPPDRKHVSLPRNTTV

HSTPIPPKDQKYSQVPQRIPTGRPGRVVGPVIPFENSSTMDPYSQRRVARNPVLPAAATN

LSAYAYRNSDNSERELQQELEKDRMQYHPMQRFMDAKMVSPDLRSTSYYMPKGVPKADVA

ERSALQSNMMQGIAPFNGIATVGGAFNKVSAVPYGVSRMY*

>Arn1MPK20-5 Brdisv1Arn11016028m.p

MFSDIFSREGVAFSRVALRQELLSDQWFTTAGGKGQCYLVPPGTTVFLVNSEEMGFFSEY

VDASRYKILEIIGKGSYGVVCSAIDQETGDKVAIKKIQNIFEHLSDAARILREIKLLRLL

RHPDIVQIKHIMLPPSRRDFRDIYVVFELMDTDLHQVIKANDDLTKEHYQFFLYQMLRAL

KYIHTANVYHRDLKPKNILANANCKLKICDFGLARVAFNDTPTTVFWTDYVATRWYRAPE

LCGSFFTKYSPAIDTWSIGCIFAEILTGKPLFPGKNVVHQLDLMTDLLGTPSTETISRIR

NDKARKYLSSMRRKQPIPFSEKFPNADPSALKLLERLLAFDPKDRPTAEEALAHPYFKRL

ARVEREPSCQQPISKTEFEFERRKFTKEDIKELIFREILEYHPKLLKDYMNGSEKTSFLY

PSAVDNFRRQFANLEIDGGRSGAADRKHFSLPRTTTVHSAPILPTNGPTSQVPQRIPTAR

PGRVVSSAMQTDNPSVSDRHNGQRVARDPAVPPAAAYHLKSDYSDRQHQQEFEKDRVRPD

RQRQEELEKDRMQYRPGHHSMDAKVAPEISPYMRSSPYYIPPFNGIAAVASGYSKVAAVT

RMY*

>Arn1MPK21-1 Brdisv1Arn11015925m.p

MGGLIRWLRHHRSRRVSSSSSSHLPSNTTSSSSTSDLRAHSLPQHQGDHHGEVVVEWEDA

AEGPDSDPEEYIVVVLGDDEQGVVAARAPVRTKPPRVMDPGKKTSESEFFTEYGEANRYK

VSEVIGKGSYGVVAAAVDTQTGERCAIKKINDVFDHVSDATRILREIKLLRLLRHPDIVE

IKHIMLPPSRREFRDIYVVFELMESDLHQVIKANDDLTPEHHQFFLYQLLRGMKYIHTAN

VFHRDLKPKNILANADCKLKICDFGLARVSFNDGAPSAIFWTDYVATRWYRAPELCGSFF

SKYTPAIDIWSVGCIFAEMLTGKPLFPGKNVVHQLDLMTDLLGTPSAESLSKIRNEKARR

YLSNMRKKPKVPLTKKFPGIDPMALHLLERLLAFDPKDRPSADEALTDPYFTGLANSERE

PITQPISKLEFEFEKRKLAKDDVRELIYREILEYHPHMLQEYLRGGGDQMSFMFPSGVDR

FKRQFAHLEEGGAKGEKSSPQLRQNASLPRERVIGNKHGDGDYNMKLNVGEKPELASVSD

GISKPLMSARSLLKSETMSASKCIGEIKNKDEDSLSECVEGTDDDVSQKIAQLKT*

>Arn1MPK21-2 Brdisv1Arn11020368m.p

MLPPSRREFRDIYIIFELMESDLHQVIKANDDLTPEHHQFFFYQLLRGMKYIHAANVFHR

DLKPRNILANADCKLKICDFGLARVSFNDTPSAIFWTDYVATRWYRAPELCGSFFSKYTP

AIDIWSIGCIFAEMLSGRPLFPGKNVVHQLDLMTDLLGTPSAESLSRIRNEKARRYLGNM

RKKHPVPFSQKFPGVDPMALDLLERLLAFDPKDRPTAAEALADPYFTGLANSDREPTTQP

ISKLEFEFERRKLARDDVRELIYREILEYHPQMLHEYHHGGDQANFVYPSGVDRFKRQFV

HLEEGVTKGEKTSPQLRQHASLPRERIIGIGDELGRPNADYCIKLHVGEEPGHTSVTDGL

SKPLLNARNFLKSESISASQCVCHQREARKRCKCCYIKYILNQIVSKIIN*

>Mon3MPK3 Brdisv1Mon31010203m.p

MDGAPVAEFRPTMTHGGRFLLYNIFGNQFEITAKYQPPIMPIGRGAYGIVCSVMNFETRE

MVAIKKIANAFDNNMDAKRTLREIKLLRHLDHENIVGLRDVIPPAIPQSFNDVYIATELM

DTDLHHIIRSNQELSEEHCQYFLYQLLRGLKYIHSANVIHRDLKPSNLLLNANCDLKICD

FGLARPSSESDMMTEYVVTRWYRAPELLLNSTDYSAAIDVWSVGCIFMELINRAPLFPGR

DHMHQMRLITEVIGTPTDDDLGFIRNEDARRYMRHLPQFPRRPFPAQFPRVQPAALDLIE

RMLAFNPLQRITVEEALEHPYLERLHDIADEPICTDPFSFDFEQHPLTEDQMKQLIFNEA

LELNPNFRY*

>Mon3MPK4 Brdisv1Mon31011000m.p

MDTSGGGGGGAGGAAQIQGMATHGGRYVLYNVYGNLFEVASKYAPPIRPIGRGAYGIVCA

AVSSDTGEEVAIKKIGNAFDNHIDAKRTLREIKLLRHMDHENIIAIKDIIRPPRRDDFKD

VYIVTELMDTDLHQIIRSNQPLTDDHCQYFLYQLLRGLKYVHSANVLHRDLKPSNLFLNA

NCDLKIADFGLARTTSETDLMTEYVVTRWYRAPELLLNCSQYTAAIDVWSVGCILGEIIT

RQPLFPGRDYIQQLKLITELIGSPDDSSLGFLRSDNARRYMKQLPQYPRQDFRLRFRNMS

DGAVDLLERMLVFDPSRRITVDEALHHPYLASLHDINEEPTCPAPFSFDFEQPSFTEEHM

KELIWRETLAFNPDPPY*

>Mon3MPK6 Brdisv1Mon31007506m.p

MDGGAQPPDAEMADAGAAVPGAGAGAAGTMENIQATLSHGGRFIQYNIFGNVFEVTVKYK

PPILPIGKGAYGITGEQVAIKKIANAFDNKIDAKRTLREIKLLRHMDHENIVAIRDIIPP

AQRNSFNDVYIAYELMDTDLHQIIRSNQALSEEHCQYFLYQILRGLKYIHSANVLHRDLK

PSNLLLNANCDLKICDFGLARTTSETDFMTEYVVTRWYRAPELLLNSSEYTAAIDVWSVG

CIFMELMDRKPLFPGRDHVHQLRLLMELIGTPNEADLDFVNENARRYIRQLPRHARQSFS

EKFPHVHPSAIDLVEKMLTFDPRQRITVEGALAHPYLASLHDISDEPVCTMPFSFDFEQH

ALSEEQMKDLIHQEALAFNPDYQ*

>Mon3MPK7-1 Brdisv1Mon31044972m.p

LLRGLKYLHSAGILHRDLKPGNLLVNANCDLKICDFGLARTNNTKAPELLLCCDNYGTSI

DVWSVGCIFAELLGRKPIFPRTQCLDQLKLIVNVLGTMSENDLEFIDNPKARKYIKSLPY

TPGTPLTSMYPQAHPLAIDLLQKMLVFDPSKRISVTEALEHPYMSPLYDPSANPPAQVPI

DLDIDENLGVEMIREMLWQEMLQYHPEAARMVNM*

>Mon3MPK11 Brdisv1Mon31031562m.p

MRMEGGGAGPAAAAAAGGAHGLGEAQIKGTLTHGGRYVQYNVYGNLFEVSAKYVPPIRPV

GRGACGIICAAINVQTREEVAIKKIGNAFDNQIDAKRTLREVKLLRHMNHENVISIKDII

RPPRRENFNDVYIVYELMDTDLHHLLRSNQPLTDDHCQYFLYQVLRGLKYVHSANVLHRD

LRPSNLLLNAKCDLKIGDFGLARTTTETDFMMEYVVTRWYRAPELLLNCSEYTGAIDMWS

VGCILGEIATREPLFPGKDYVHQLRLITELLGSPDDTSLGFLRSDNARRYVRSLPQYPKQ

QFRSRFPNMSSGAMDLLERMLVFDPNKRITVDEALCHPYLASLHEINDEPVCPAPFSFDF

EQPSFTEEDIKELIWRESVKFNPEPIH*

>Mon3MPK14 Brdisv1Mon31029522m.p

MAMLVDPPNGMGNQGKHYYSMWQTLFEIDTKYVPIKPIGRGAYGIVCSSINRETNEKVAI

KKIHNVFDNRVDALRTLRELKLLRHLRHENVISLKDIMMPVQRRSFKDVYLVYELMDTDL

HQIIKSPQGLSNDHCQYFLFQLLRGLKYLHSAEILHRDLKPGNLLVNANCDLKICDFGLA

RTNSSKGQFMTEYVVTRWYRAPELLLCCDNYGTSIDVWSVGCIFAELLGRKPIFPGTECL

NQLKLIVNVLGTMSESDLEFIDNPKARRYIKTLPYTPGVPLASMYPHAHPLAIDLLQKML

IFDPTKRISVTEALEHPYMSPLYDPSANPPAQVPIDLDLDENISADMIREMMWQEMLHYH

PEAATAISM*

>Mon3MPK16 Brdisv1Mon31017564m.p

MDFFTEYGEGNRYKIEEVIGKGSYGVVCSALDTHTGDKVAIKKINDIFEHVSDATRILRE

IKLLRLLRHPDIVEIKHILLPPSRREFKDIYVVFELMESDLHQVIKANDDLTPEHYQFFL

YQLLRGLKYIHTANVFHRDLKPKNILANADCKLKICDFGLARVAISDTPTAIFWTDYIAT

RWYRAPELCGSFFSKYTPAIDIWSIGCIFAELLTGKPLFPGKNVVHQLDIITDLLGTPSA

ETIARIRNEKARRYLSSMRRKKTVPFTQKFPNADPLALNLLERMLAFDPKDRPSAEEALA

DPYFKNIASVDREPSAQPITKLEFEFERRRITKDDIRELIYREILEYHPNMLREFLEGAE

PTGFMYPSAVDHFKKQFTFLEEHYAKGSTAAPPERQHNSLPRPSVIYSDNRPQNAANITD

DLSRCAIRDNTQKAQKDSASVGANRIPHGAAAAAARPGKVVGSVLRYGNCSTSGTEQYEQ

RRVVRSPGIAPNGVTSGSSYPRRNNTCKSETGEAERIDTNQGGPPKPYVANKLPATVDGR

SGHW*

>Mon3MPK17 Brdisv1Mon31005542m.p

MVGGGGIVDGFRRLFHRRTPSGSGPSSNQSSAGEESSDIDAVVEDLDLVGLRAIRVPKRK

MPLPVESHKKNILEKEFFTEYGEASQYQIQEVIGKGSYGVVAAAVDTRTGERVAIKKIND

VFEHVSDATRILREVKLLRLLRHPDVVEIKHIMLPPSRREFQDIYVVFELMESDLHQVIR

ANDDLTPEHYQFFLYQLLRALKYIHAANVFHRDLKPKNILANSDCKLKICDFGLARVSFN

DAPTAIFWTDYVATRWYRAPELCGSFFSKYTPAIDIWSIGCIFAELLTGRPLFPGKNVVH

QLDIITDLLGTPSSETLSRIRNEKARRYLSCMRKKHPVPLTQKFPNVDPLALRLLGRLLA

FDPKDRPSAEEALADPYFAVLANVEREPSRNPISKLEFEFERQKVTKDDVRELIYREILE

YHPQMLQEYMQGGEQLSFLYPSGVDRFKRQFAHLEENYSKGERGSPLQRKHASLPRQRVG

ASNDNNEQHASDQERGADSIAPATGNPPGSQDAGQQHVSGGQNGVGSTNISPRSYQKSAS

ISASKCVVVNANKNPEYDDDISEEMEGAVDGLSEKVSRMHP*

>Mon3MPK20-1 Brdisv1Mon31018835m.p

MQQDQRKKSSAEAEFFTEYGDASRYKIQEIVGKGSYGVVCSAIDVHTGEKVAIKKIHDIF

EHISDAARILREIKLLRLLRHPDIVEIKHIMLPPSRRDFKDIYVVFELMESDLHQVIKAN

DDLTKEHYQFFLYQLLRALKYIHTANVYHRDLKPKNILANSNCKLKICDFGLARVAFNDT

PTTIFWTDYVATRWYRAPELCGSFFSKYTPAIDVWSIGCIFAEVLTGKPLFPGKNVVHQL

DLMTDLLGTPSMDTISRVRNEKARRYLSSMRKKEPIPFSQKFPNADPLALDLLQRLLAFD

PKDRPTAGEALSHPYFKGLAKVEREPSCQPITKMEFEFERRRATKEDIRELIFREILEYH

PQLLKDYINGTERTTFLYPSAVDQFRKQFAHLEENSESGPVVPMDRKHTSLPRSTIVHSA

PIHAKEQPRIGPSRDRPLTDESYKNPRDSEKYSGNVPRSSHHSQAPQRVPTARPGRVVGP

VLPYENVGTSHPYDPRRVAMNSGYPPQQQIPQTYGYYQTTGKSACSEPSQAERYTLHQQA

YACANSSTAPDVALDMRAPPFHQSAGPKSGSSDRLTAETNLYTRSLNGIVATTSGVAASA

HRKVSVVPYGMSQMY*

>Mon3MPK20-2 Brdisv1Mon31014790m.p

MQQPQQQGQRNKSTDVDFFSEYGDANRYRIQEVIGKGSYGVVCSAMDMQTRQKVAIKKIH

NIFDHVSDAARILREIKLLRLLRHPDVVEIKHIMLPPSRKDFKDIYVVFELMESDLHQVI

KANDDLTKEHYQFFLYQLLRALKYIHTASVYHRDLKPKNILANSNCKLKICDFGLARVAF

NDTPTTVFWTDYVATRWYRAPELCGSFFTKYTPAIDIWSIGCIFAEVLTGKPLFPGKNVV

HQLDLMTDLLGTPSMDTISRVRNEKARRYLSSMRKKDPVPFSKKFPNADPLGLKLLEKLL

AFDPKDRPTAEEALTDPYFKSLSKPDREPSCQPIRKVEFDFEHRRMSKDDIRELIFQEIL

EYHPQLLKNYIDGTEKTTFLYPSAVDQFKKQFSHLEESDGSGPVVPTERKHASLPRSTTV

HSTPIPAKEQPLVASSRGRPIANEPCKPWAPGNVPGASQTAHVAQAGRAVGVGSVPPYES

GSGKYPYDATSRPAVSSGYPPQQKIPQTYGYHHHHQTPGAGQSSQAMGGYACGYTKGTTP

PPAAAQDMRASPYHHRSAGTKNDPLNRLAAESDIYTRSLNGIVAAAASAGTGAHRKVGAV

PFGMSGMY*

>Mon3MPK20-3 Brdisv1Mon31006289m.p

MQHGDLHKKSAAEMDFFTAYDDANRYKILEVIGKGSYGLVCSANDLQTGEKVAIKKIHNI

FEHISDAARILREIKLLRLLRHPDVVEIKHILLPPSKKDFKDIYVVFELMESDLHQVIKA

NDDLTREHYQFFLYQMLRALKYMHTANVYHRDLKPKNVLANANCKLKICDFGLARVAFSD

APTTVFWTDYVATRWYRAPELCGSFYSKYTPAIDIWSIGCIFAEVLIGKPLFPGKNVVHQ

LDLITDILGTPSLDAISRVRNDKARKYLTCMRKKQPASFSQKFPKADPLALQLLRRLLAF

DPKDRPSAEEALADPYFNGLAKVEREPSCQPIPKIEFEFEGRRVTKEDIKELIFEEILEY

HPQLLKEHIIGKERPNFVHLSAVDQFKKHFTQLEENDNETGAAVSLQRKHSSLPRQAFNH

R*

>Mon3MPK20-4 Brdisv1Mon31019053m.p

MQTTEQQRKKGSSEMDFFSEYGDANRYKIQEVIGKGSYGVVCSAIDQHTGDKVAIKKIHN

IFEHLSDAARILREIKLLRLLRHPDIVEIRHIMLPPSRRDFKDIYVVFELMDTDLHQVIK

ANDDLTKEHHQFFLYQMLRALKYIHTANVYHRDLKPKNILANANCKLKICDFGLARVAFN

DTPTTVFWTDYVATRWYRAPELCGSFFTKYSRAIDIWSIGCIFAEILTGKPLFPGKNVVH

QLDLMTDLLGTPSLDTVSRIRNEKARRYLSSMRKKQSVSFSERFPKADPAALKLLQRLLA

FDPKDRPTAEEALADPYFKGLGKVEREPSCQPITKMEFEFERKNVTKADVKELIFREILE

YHPQLLKDYMNGTEKTNFLYPSAVDNFRRQFANLEENGGKGGAVVPPDRKHVSLPRNTTV

HSTPIPPKDQKYSQVPQRIPTGRPGRVVGPVIPFENSSTMDPYSQRRVARNPVLPAAATN

LSAYAYRNSDNSERELQQELEKDRMQYHPMQRFMDAKMVSPDLRSTSYYMPKGVPKADVA

ERSALQSNMMQGIAPFNGIATVGGAFNKVSAVPYGVSRMY*

>Mon3MPK20-5 Brdisv1Mon31014926m.p

MFSDIFSREGVAFSRVALRQELLSDQWFTTAGGKGQCYLVPPGTTVFLVNSEEMGFFSEY

VDASRYKILEIIGKGSYGVVCSAIDQETGDKVAIKKIQNIFEHLSDAARILREIKLLRLL

RHPDIVQIKHIMLPPSRRDFRDIYVVFELMDTDLHQVIKANDDLTKEHYQFFLYQMLRAL

KYIHTANVYHRDLKPKNILANANCKLKICDFGLARVAFNDTPTTVFWTDYVATRWYRAPE

LCGSFFTKYSPAIDTWSIGCIFAEILTGKPLFPGKNVVHQLDLMTDLLGTPSTETISRIR

NDKARKYLSSMRRKQPIPFSEKFPNADPSALKLLERLLAFDPKDRPTAEEALAHPYFKRL

ARVEREPSCQQPISKTEFEFERRKFTKEDIKELIFREILEYHPKLLKDYMNGSEKTSFLY

PSAVDNFRRQFANLEIDGGRSGAADRKHFSLPRTTTVHSAPILPTNGPTSQVPQRIPTAR

PGRVVSSAMQTDNPSVSDRHNGRRVARDPAVPPAAAYHLKSDYSDRQHQQEFEKDRVRPD

RQRQEELEKDRMQYRPGHHSMDAKVAPEISPYMRSSPYYIPPFNGIAAVASGYSKVAAVT

RMY*

>Mon3MPK21-1 Brdisv1Mon31014824m.p

MGGLIRWLRHHRSRRVSSSSSSHLPSNTTSSSSTSDLRAHSLPQHQGDHHGEVVVEWEDA

AEGPDSDPEEYIVVVLGDDEQGVVAARAPVRTKPPRVMDPGKKTSESEFFTEYGEANRYK

VSEVIGKGSYGVVAAAVDTQTGERCAIKKINDVFDHVSDATRILREIKLLRLLRHPDIVE

IKHIMLPPSRREFRDIYVVFELMESDLHQVIKANDDLTPEHHQFFLYQLLRGMKYIHTAN

VFHRDLKPKNILANADCKLKICDFGLARVSFNDGAPSAIFWTDYVATRWYRAPELCGSFF

SKYTPAIDIWSVGCIFAEMLTGKPLFPGKNVVHQLDLMTDLLGTPSAESLSKIRNEKARR

YLSNMRKKPKVPLTKKFPGIDPMALHLLERLLAFDPKDRPSADEALTDPYFTGLANSERE

PITQPISKLEFEFEKRKLAKDDVRELIYREILEYHPHMLQEYLRGGGDQMSFMFPSGVDR

FKRQFAHLEEGGAKGEKSSPQLRQNASLPRERVIGNKHGDGDYNMKLNVGEKPELASVSD

GISKPLMSARSLLKSETMSASKCIGEIKNKDEDSLSECVEGTDDDVSQKIAQLKT*

>Mon3MPK21-2 Brdisv1Mon31018928m.p

GSDEAEFFTEYGEANRYEVGEVVGKGSYGVVAAAVDTHTGERVAIKKINDVFEHVSDATR

ILREIKLLRLLRHPDIVEIKHIMLPPSRREFRDIYIIFELMESDLHQVIKANDDLTPEHH

QFFFYQLLRGMKYIHAANVFHRDLKPRNILANADCKLKICDFGLARVSFNDTPSAIFWTD

YVATRWYRAPELCGSFFSKYTPAIDIWSIGCIFAEMLSGRPLFPGKNVVHQLDLMTDLLG

TPSAESLSRIRNEKARRYLGNMRKKHPVPFSQKFPGVDPMALDLLERLLAFDPKDRPTAA

EALADPYFTGLANSDREPTTQPISKLEFEFERRKLARDDVRELIYREILEYHPQMLHEYH

HGGDQANFVYPSGVDRFKRQFVHLEEGVTKGEKTSPQLRQHASLPRERIIGIGDELGRPN

ADYCIKLHVGEEPGHTSVTDGLSKPLLNARNFLKSESISASQCVVIKEKREKDVSAVTSN

IF*

>BdTR7aMPK3 Brdisv1BdTR7a1011021m.p

MDGAPVAEFRPTMTHGGRFLLYNIFGNQFEITAKYQPPIMPIGRGAYGIVCSVMNFETRE

MVAIKKIANAFDNNMDAKRTLREIKLLRHLDHENIVGLRDVIPPAIPQSFNDVYIATELM

DTDLHHIIRSNQELSEEHCQYFLYQLLRGLKYIHSANVIHRDLKPSNLLLNANCDLKICD

FGLARPSSESDMMTEYVVTRWYRAPELLLNSPDYSAAIDVWSVGCIFMELINRAPLFPGR

DHMHQMRLITEVIGTPTDDDLGFIRNEDARRYMRPLPQFPRRPFPAQFPRVQPAALDLIE

RMLAFNPLQRITVEEALEHPYLERLHDIADEPICTDPFSFDFEQHPLTEDQMKQLIFNEA

LELNPNFRY*

>BdTR7aMPK4 Brdisv1BdTR7a1035227m.p

MDTSGGGGGAGGAAQIQGMATHGGRYVLYNVYGNLFEVASKYAPPIRPIGRGAYGIVCAA

VSSDTGEEVAIKKIGNAFDNHIDAKRTLREIKLLRHMDHENIIAIKDIIRPPRRDDFKDV

YIVTELMDTDLHQIIRSNQPLTDDHCQYFLYQLLRGLKYVHSANVLHRDLKPSNLFLNAN

CDLKIADFGLARTTSETDLMTEYVVTRWYRAPELLLNCSQYTAAIDVWSVGCILGEIITR

QPLFPGRDYIQQLKLITELIGSPDDSSLGFLRSDNARRYMKQLPQYPRQDFRLRFRNMSD

GAVDLLERMLVFDPSRRITVDEALHHPYLASLHDINEEPTCPAPFSFDFEQPSFTEEHMK

ELIWRETLAFNPDPPY*

>BdTR7aMPK6 Brdisv1BdTR7a1008045m.p

MDGGAQPPDAEMADAGAAVPGAGAGAAGTMENIQATLSHGGRFIQYNIFGNVFEVTVKYK

PPILPIGKGAYGIVCSALNSETGEQVAIKKIANAFDNKIDAKRTLREIKLLRHMDHENIV

AIRDIIPPAQRNSFNDVYIAYELMDTDLHQIIRSNQALSEEHCQYFLYQILRGLKYIHSA

NVLHRDLKPSNLLLNANCDLKICDFGLARTTSETDFMTEYVVTRWYRAPELLLNSSEYTA

AIDVWSVGCIFMELMDRKPLFPGRDHVHQLRLLMELIGTPNEADLDFVNENARRYIRQLP

RHARQSFSEKFPHVHPSAIDLVEKMLTFDPRQRITVEGALAHPYLASLHDISDEPVCTMP

FSFDFEQHALSEEQMKDLIHQEALAFNPDYQ*

>BdTR7aMPK7-1 Brdisv1BdTR7a1005883m.p

MPSRFPFLSPGCFIRLLSGSASSDLLLLLPRRGQSAPRARVLLKMAMMVDPPNGTGNHGK

HYYTMWQTMFEIDTKYVPIKPIGRGAYGIVCSSTNQENNEKVAIKKINNVFDNRVDALRT

LRELKLLRHLRHENVIALKDIMMPVHRRSFKDVYLVSELMDTDLHQIIKSSQPLSNDHCQ

YFLFQLLRGLKYLHSAGILHRDLKPGNLLVNANCDLKICDFGLARTNNTKGQFMTEYVVT

RWYRAPELLLCCDNYGTSIDVWSVGCIFAELLGRKPIFPGTECLNQLKLIVNVLGTMSEN

DLEFIDNPKARKYIKSLPYTPGTPLTSMYPQAHPLAIDLLQKMLVFDPSKRISVTEALEH

PYMSPLYDPSANPPAQVPIDLDIDENLGVEMIREMLWQEMLQYHPEAARMGNM*

>BdTR7aMPK11 Brdisv1BdTR7a1033281m.p

MRMEGGGAGPAAAAAGGGAHGLGEAQIKGTLTHGGRYVQYNVYGNLFEVSAKYVPPIRPV

GRGACGIICAAINVQTREEVAIKKIGNAFDNQIDAKRTLREVKLLRHMNHENVISIKDII

RPPRRENFNDVYIVYELMDTDLPHLLRSNQPLTDDHCQYFLYQVLRGLKYVHSANVLHRD

LRPSNLLLNAKCDLKIGDFGLARTTTETDFMMEYVVTRWYRAPELLLNCSEYTGAIDMWS

VGCILGEIATREPLFPGKDYVHQLRLITELLGSPDDTSLGFLRSDNARRYVRSLPQYPKQ

QFRSRFPNMSSGAMDLLERMLVFDPNKRITVDEALCHPYLASLHEINDEPVCPAPFSFDF

EQPSFTEEDIKELIWRESVKFNPEPIH*

>BdTR7aMPK14 Brdisv1BdTR7a1031219m.p

MAMLVDPPNGMGNQGKHYYSMWQTLFEIDTKYVPIKPIGRGAYGIVCSSINRETNEKVAI

KKIHNVFDNRVDALRTLRELKLLRHLRHENVISLKDIMMPVQRRSFKDVYLVYELMDTDL

HQIIKSPQGLSNDHCQYFLFQLLRGLKYLHSAEILHRDLKPGNLLVNANCDLKICDFGLA

RTNSSKGQFMTEYVVTRWYRAPELLLCCDNYGTSIDVWSVGCIFAELLGRKPIFPGTECL

NQLKLIVNVLGTMSESDLEFIDNPKARRYIKTLPYTPGVPLASMYPHAHPLAIDLLQKML

IFDPTKRISVTEALEHPYMSPLYDPSANPPAQVPIDLDLDENISADMIREMMWQEMLHYH

PEAATAISM*

>BdTR7aMPK16 Brdisv1BdTR7a1018519m.p

MDFFTEYGEGNRYKIEEVIGKGSYGVVCSALDTHTGDKVAIKKINDIFEHVSDATRILRE

IKLLRLLRHPDIVEIKHILLPPSRREFKDIYVVFELMESDLHQVIKANDDLTPEHYQFFL

YQLLRGLKYIHTANVFHRDLKPKNILANADCKLKICDFGLARVAISDTPTAIFWTDYIAT

RWYRAPELCGSFFSKYTPAIDIWSIGCIFAELLTGKPLFPGKNVVHQLDIITDLLGTPSA

ETIARIRNEKARRYLSSMRRKRTVPFTQKFPNADPLALNLLERMLAFDPKDRPSAEEALA

DPYFKNIASVDREPSAQPITKLEFEFERRRITKDDIRELIYREILEYHPNMLREFLEGAE

PTGFMYPSAVDHFKKQFTFLEEHYAKGSTAAPPERQHNSLPRPSVIYSDNRPQNAANITD

DLSRCAIRDNTQKAQKDSASVGANRIPHGAAAAAARPGKVVGSVLRYGNCSTSGTEQYEQ

RRVVRSPGIAPNGVTSGSSYPRRNNTCKSETGEAERIDTNQGGPPKPYVANKLPATVDGR

NGHW*

>BdTR7aMPK17 Brdisv1BdTR7a1006004m.p

MVGGGGIVDGFRRLFPRRTPSGSGPSSNQSSAGEESSDIDAVVEDLDLVGLRAIRVPKRK

MPLPVESHKKNILEKEFFTEYGEASQYQIQEVIGKGSYGVVAAAVDTRTGERVAIKKIND

VFEHVSDATRILREVKLLRLLRHPDVVEIKHIMLPPSRREFQDIYVVFELMESDLHQVIR

ANDDLTPEHYQFFLYQLLRALKYIHAANVFHRDLKPKNILANSDCKLKICDFGLARVSFN

DAPTAIFWTDYVATRWYRAPELCGSFFSKYTPAIDIWSIGCIFAELLTGRPLFPGKNVVH

QLDIITDLLGTPSSETLSRIRNEKARRYLSCMRKKHPVPLTQKFPNVDPLALRLLGRLLA

FDPKDRPSAEEALADPYFAVLANVEREPSRNPISKLEFEFERRKVTKDDVRELIYREILE

YHPQMLQEYMQGGEQLSFLYPSGVDRFKRQFAHLEENYSKGERGSPLQRKHASLPRQRVG

ASNDNNDQHASDQERGADSIAPATGNPPGSQDAGQQHVSGGQNGVGSTNISPRSYQKSAS

ISASKCVVVNANKNPEYDDDISEEMEGAVDGLSEKVSRMHP*

>BdTR7aMPK20-1 Brdisv1BdTR7a1020019m.p

MQQDQRKKSSAEAEFFTEYGDASRYKIQEIVGKGSYGVVCSAIDVHTGEKVAIKKIHDIF

EHISDAARILREIKLLRLLRHPDIVEIKHIMLPPSRRDFKDIYVVFELMESDLHQVIKAN

DDLTKEHYQFFLYQLLRALKYIHTANVYHRDLKPKNILANSNCKLKICDFGLARVAFNDT

PTTIFWTDYVATRWYRAPELCGSFFSKYTPAIDVWSIGCIFAEVLTGKPLFPGKNVVHQL

DLMTDLLGTPSMDTISRVRNEKARRYLSSMRKKEPIPFSQKFPNADPLALDLLQRLLAFD

PKDRPTAGEALSHPYFKGLAKVEREPSCQPITKMEFEFERRRATKEDIRELIFREILEYH

PQLLKDYINGTERTTFLYPSAVDQFRKQFAHLEENSESGPVVPMDRKHTSLPRSTIVHSA

PIHAKEQPRIGPSRDRPLTDESYKNPRDSEKYSGNVPRSSHHSQAPQRVPTARPGRVVGP

VLPYENVGTSHPYDPRRVAMNSGYPPQQQIPQTYGYYQTTGKSACSEPSQAERYTLHQQA

YACANSSTAPDVALDMRAPPFHQSAGPKSGSSDRLTAETNLYTRSLNGIVATTSGVAASA

HRKVSVVPYGMSQMY*

>BdTR7aMPK20-2 Brdisv1BdTR7a1015768m.p

MQQPQQQGQRNKSTDVDFFSEYGDANRYRIQEVIGKGSYGVVCSAMDMQTRQKVAIKKIH

NIFDHVSDAARILREIKLLRLLRHPDVVEIKHIMLPPSRKDFKDIYVVFELMESDLHQVI

KANDDLTKEHYQFFLYQLLRALKYIHTASVYHRDLKPKNILANSNCKLKICDFGLARVAF

NDTPTTVFWTDYVATRWYRAPELCGSFFTKYTPAIDIWSIGCIFAEVLTGKPLFPGKNVV

HQLDLMTDLLGTPSMDTISRVRNEKARRYLSSMRKKDPVPFSKKFPNADPLGLKLLEKLL

AFDPKDRPTAEEALTDPYFKSLSKPDREPSCQPIRKVEFDFEHRRMSKDDIRELIFQEIL

EYHPQLLKNYIDGTEKTTFLYPSAVDQFKKQFSHLEESDGSGPVVPTERKHASLPRSTTV

HSTPIPAKEQPLVASSRGRPIANEPCKPWAPGNVPGASQTAHVAQAGRAVGVGSVPPYES

GSGKYPYDATSRPAVSSGYPPQQKIPQTYGYHHHHQTPGAGQSSQAMGGYACGYTKGTTP

PPAAAQDMRASPYHHRSAGTKNDPLNRLAAESDIYTRSLNGIVAAAASAGTGAHRKVGAV

PFGMSGMY*

>BdTR7aMPK20-3 Brdisv1BdTR7a1006751m.p

MQHGDLHKKSAAEMDFFTAYDDANRYKILEVIGKGSYGLVCSANDLQTGEKVAIKKIHNI

FEHISDAARILREIKLLRLLRHPDVVEIKHILLPPSKKDFKDIYVVFELMESDLHQVIKA

NDDLTREHYQFFLYQMLRALKYMHTANVYHRDLKPKNVLANANCKLKICDFGLARVAFSD

APTTVFWTDYVATRWYRAPELCGSFYSK

>BdTR7aMPK20-4 Brdisv1BdTR7a1020237m.p

MDFFSEYGDANRYKIQEVIGKGSYGVVCSAIDQHTGDKVAIKKIHNIFEHLSDAARILRE

IKLLRLLRHPDIVEIRHIMLPPSRRDFKDIYVVFELMDTDLHQVIKANDDLTKEHHQFFL

YQMLRALKYIHTANVYHRDLKPKNILANANCKLKICDFGLARVAFNDTPTTVFWTDYVAT

RWYRAPELCGSFFTKYSRAIDIWSIGCIFAEILTGKPLFPGKNVVHQLDLMTDLLGTPSL

DTVSRIRNEKARRYLSSMRKKQSVSFSERFPKADPAALKLLQRLLAFDPKDRPTAEEALA

DPYFKGLGKVEREPSCQPITKMEFEFERKNVTKADIKELIFREILEYHPQLLKDYMNGTE

KTNFLYPSAVDNFRRQFANLEENGGKGGAVVPPDRKHVSLPRNTTVHSTPIPPKDQKYSQ

VPQRIPTGRPGRVVGPVIPFENSSTMDPYSQRRVARNPVLPAAATNLSAYAYRNSDNSER

ELQQELEKDRMQYHPMQRFMDAKMVSPDLRSTSYYMPKGVPKADVAERSALQSNMMQGIA

PFNGIATVGGAFNKVSAVPYGVSRMY*

>BdTR7aMPK20-5 Brdisv1BdTR7a1015928m.p

MFSDIFSREGVAFSRVALRQELLSDQWFTTAGGKGQCYLVPPGTTVFLVNSEEMGFFSEY

VDASRYKILEIIGKGSYGVVCSAIDQETGDKVAIKKIQNIFEHLSDAARILREIKLLRLL

RHPDIVQIKHIMLPPSRRDFRDIYVVFELMDTDLHQVIKANDDLTKEHYQFFLYQMLRAL

KYIHTANVYHRDLKPKNILANANCKLKICDFGLARVAFNDTPTTVFWTDYVATRWYRAPE

LCGSFFTKYSPAIDTWSIGCIFAEILTGKPLFPGKNVVHQLDLMTDLLGIPSTETISRIR

NDKARKYLSSMRRKQPIPFSEKFPNADPSALKLLERLLAFDPKDRPTAEEALAHPYFKRL

ARVEREPSCQQPISKTEFEFERRKFTKEDIKELIFREILEYHPKLLKDYMNGSEKTSFLY

PSAVDNFRRQFANLEIDGGRSGAADRKHFSLPRTTTVHSAPILPTNGPTSQVPQRIPTAR

PGRVVSSAMQTDNPSVSDRHNGRRVARDPAVPPAAAYHLKSDYSDRQHQQEFEKDRVRPD

RQRQEELEKDRMQYRPGHHSMDAKVAPEISPYMRSSPYYIPPFNGIAAVASGYSKVAAVT

RMY*

>BdTR7aMPK21-1 Brdisv1BdTR7a1015824m.p

MGGLIRWLRHPRPRRVSSSSSSHLPSNTTSSSSTSDLRAHSLPQHQGDHHGEVVVEWEDA

AEGPDSDPEEYIVVVLGDDEQGVVAARAPVRTKPPRVMDPGKKTSESEFFTEYGEANRYK

VSEVIGKGSYGVVAAAVDTQTGERCAIKKINDVFDHVSDATRILREIKLLRLLRHPDIVE

IKHIMLPPSRREFRDIYVVFELMESDLHQVIKANDDLTPEHHQFFLYQLLRGMKYIHTAS

VFHRDLKPKNILANADCKLKICDFGLARVSFNDGAPSAIFWTDYVATRWYRAPELCGSFF

SKYTPAIDIWSVGCIFAEMLTGKPLFPGKNVVHQLDLMTDLLGTPSAESLSKIRNEKARR

YLSNMRKKPKVPLTKKFPGIDPMALHLLERLLAFDPKDRPSADEALTDPYFTGLANSERE

PITQPISKLEFEFEKRKLAKDDVRELIYREILEYHPHMLQEYLRGGGDQMSFMFPSGVDR

FKRQFAHLEEGGAKGEKSSPQLRQNASLPRERVIGNKHGDGDYNMKLNVGEKPELASVSD

GISKPLMSARSLLKSETMSASKCIGEIKNKDEDSLSECVEGTDDDVSQKIAQLKT*

>BdTR7aMPK21-2 Brdisv1BdTR7a1020100m.p

GSDEAEFFTEYGEANRYEVGEVVGKGSYGVVAAAVDTHTGERVAIKKINDVFEHVSDATR

ILREIKLLRLLRHPDIVEIKHIMLPPSRREFRDIYIIFELMESDLHQVIKANDDLTPEHH

QFFFYQLLRGMKYIHAANVFHRDLKPRNILANADCKLKICDFGLARVSFNDTPSAIFWTD

YVATRWYRAPELCGSFFSKYTPAIDIWSIGCIFAEMLSGRPLFPGKNVVHQLDLMTDLLG

TPSAESLSRIRNEKARRYLGNMRKKHPVPFSQKFPGVDPMALDLLERLLAFDPKDRPTAA

EALADPYFTGLANSDREPTTQPISKLEFEFERRKLARDDVRELIYREILEYHPQMLHEYH

HGGDQANFVYPSGVDRFKRQFVHLEEGVTKGEKTSPQLRQHASLPRERIIGIGDELGRPN

ADYCIKLHVGEEPGHTSVTDGLSKPLLNARNFLKSESISASQCVVIKEKREKDVSAVTSN

IF*

>BdTR8iMPK3 Brdisv1BdTR8i1010712m.p

MDGAPVAEFRPTMTHGGRFLLYNIFGNQFEITAKYQPPIMPIGRGAYGIVCSVMNFETRE

MVAIKKIANAFDNNMDAKRTLREIKLLRHLDHENIVGLRDVIPPAIPQSFNDVYIATELM

DTDLHHIIRSNQELSEEHCQYFLYQLLRGLKYIHSANVIHRDLKPSNLLLNANCDLKICD

FGLARPSSESDMMTEYVVTRWYRAPELLLNSTDYSAAIDVWSVGCIFMELINRAPLFPGR

DHMHQMRLITEVIGTPTDDDLGFIRNEDARRYMRHLPQSPRRPFPAQFPRVQPAALDLIE

RMLAFNPLQRITVEEALEHPYLERLHDIADEPICTDPFSFDFEQHPLTEDQMKQLIFNEA

LELNPNFRY*

>BdTR8iMPK4 Brdisv1BdTR8i1027174m.p

MDTSGGGGGGGGGAGGAAQIQGMATHGGRYVLYNVYGNLFEVASKYAPPIRPIGRGAYGI

VCAAVSSDTGEEVAIKKIGNAFDNHIDAKRTLREIKLLRHMDHENIIAIKDIIRPPRRDD

FKDVYIVTELMDTDLHQIIRSNQPLTDDHCQYFLYQLLRGLKYVHSANVLHRDLKPSNLF

LNANCDLKIADFGLARTTSETDLMTEYVVTRWYRAPELLLNCSQYTAAIDVWSVGCILGE

IITRQPLFPGRDYIQQLKLITELIGSPDDSSLGFLRSDNARRYMKQLPQYPRQDFRLRFR

NMSDGAVDLLERMLVFDPSRRITVDEALHHPYLASLHDINEEPTCPAPFSFDFEQPSFTE

EHMKELIWRETLAFNPDPPY*

>BdTR8iMPK6 Brdisv1BdTR8i1007684m.p

MDGGAQPPDAEMADAGAAVPGAGAGAAGTMENIQATLSHGGRFIQYNIFGNVFEVTVKYK

PPILPIGKGAYGIVCSALNSETGEQVAIKKIANAFDNKIDAKRTLREIKLLRHMDHENIV

AIRDIIPPAQRNSFNDVYIAYELMDTDLHQIIRSNQALSEEHCQYFLYQILRGLKYIHSA

NVLHRDLKPSNLLLNANCDLKICDFGLARTTSETDFMTEYVVTRWYRAPELLLNSSEYTA

AIDVWSVGCIFMELMDRKPLFPGRDHVHQLRLLMELIGTPNEADLDFVNENARRYIRQLP

RHARQSFSEKFPHVHPSAIDLVEKMLTFDPRQRITVEGALAHPYLASLHDISDEPVCTMP

FSFDFEQHALSEEQMKDLIHQEALAFNPDYQ*

>BdTR8iMPK7-1 Brdisv1BdTR8i1005547m.p

MPSRFPFLSPGCFIRLLSGSASSDLLLLLPRRGQSAPRARVLLKMAMMVDPPNGTGNHGK

HYYTMWQTMFEIDTKYVPIKPIGRGAYGIVCSSTNQENNEKVAIKKINNVFDNRVDALRT

LRELKLLRHLRHENVIALKDIMMPVHRRSFKDVYLVSELMDTDLHQIIKSSQPLSNDHCQ

YFLFQLLRGLKYLHSAGILHRDLKPGNLLVNANCDLKICDFGLARTNNTKGQFMTEYVVT

RWYRAPELLLCCDNYGTSIDVWSVGCIFAELLGRKPIFPGTECLNQLKLIVNVLGTMSEN

DLEFIDNPKARKYIKSLPYTPGTPLTSMYPQAHPLAIDLLQKMLVFDPSKRISVTEALEH

PYMSPLYDPSANPPAQVPIDLDIDENLGVEMIREMLWQEMLQYHPEAARMVNM*

>BdTR8iMPK11 Brdisv1BdTR8i1025152m.p

MRMEGGGAGPAAAAAGGGAHGLGEAQIKGTLTHGGRYVQYNVYGNLFEVSAKYVPPIRPV

GRGACGIICAAINVQTREEVAIKKIGNAFDNQIDAKRTLREVKLLRHMNHENVISIKDII

RPPRRENFNDVYIVYELMDTDLHHLLRSNQPLTDDHCQYFLYQVLRGLKYVHSANVLHRD

LRPSNLLLNAKCDLKIGDFGLARTTTETDFMMEYVVTRWYRAPELLLNCSEYTGAIDMWS

VGCILGEIATREPLFPGKDYVHQLRLITELLGSPDDTSLGFLRSDNARRYVRSLPQYPKQ

QFRSRFPNMSSGAMDLLERMLVFDPNKRITVDEALCHPYLASLHEINDEPVCPAPFSFDF

EQPSFTEEDIKELIWRESVKFNPEPIH*

>BdTR8iMPK14 Brdisv1BdTR8i1023107m.p

MAMLVDHPNGMGNQGKHYYSMWQTLFEIDTKYVPIKPIGRGAYGIVCSSINRETNEKVAI

KKIHNVFDNRVDALRTLRELKLLRHLRHENVISLKDIMMPVQRRSFKDVYLVYELMDTDL

HQIIKSPQGLSNDHCQYFLFQLLRGLKYLHSAEILHRDLKPGNLLVNANCDLKICDFGLA

RTNSSKGQFMTEYVVTRWYRAPELLLCCDNYGTSIDVWSVGCIFAELLGRKPIFPGTECL

NQLKLIVNVLGTMSESDLEFIDNPKARRYIKTLPYTPGVPLASMYPHAHPLAIDLLQKML

IFDPTKRISVTEALEHPYMSPLYDPSANPPAQVPIDLDLDENISADMIREMMWQEMLHYH

PEAATAISM*

>BdTR8iMPK16 Brdisv1BdTR8i1018031m.p

MDFFTEYGEGNRYKIEEVIGKGSYGVVCSALDTHTGDKVAIKKINDIFEHVSDATRILRE

IKLLRLLRHPDIVEIKHILLPPSRREFKDIYVVFELMESDLHQVIKANDDLTPEHYQFFL

YQLLRGLKYIHTANVFHRDLKPKNILANADCKLKICDFGLARVAISDTPTAIFWTDYIAT

RWYRAPELCGSFFSKYTPAIDIWSIGCIFAELLTGKPLFPGKNVVHQLDIITDLLGTPSA

ETIARIRNEKARRYLSSMRRKKTVPFTQKFPNADPLALNLLERMLAFDPKDRPSAEEALA

DPYFKNIASVDREPSAQPITKLEFEFERRRITKDDIRELIYREILEYHPNMLREFLEGAE

PTGFMYPSAVDHFKKQFTFLEEHYAKGSTAAPPERQHNSLPRPSVIYSDNRPQNAANITD

DLSRCAIRDNTQKAQKDSASVGANRIPHGAAAAAARPGKVVGSVLRYGNCSTSGTEQYEQ

RRVVRSPGIAPNGVTSGSSYPRRNYTCKSETGEAERIDTNQGGPPKPYVANKLPATVDGR

NGHW*

>BdTR8iMPK17 Brdisv1BdTR8i1005684m.p

MVGGGGIVDGFRRLFHRRTPSGSGPSSNQSSAGEESSDIDAVVEDLDLVGLRAIRVPKRK

MPLPVESHKKNILEKEFFTEYGEASQYQIQEVIGKGSYGVVAAAVDTRTGERVAIKKIND

VFEHVSDATRILREVKLLRLLRHPDVVEIKHIMLPPSRREFQDIYVVFELMESDLHQVIR

ANDDLTPEHYQFFLYQLLRALKYIHAANVFHRDLKPKNILANSDCKLKICDFGLARVSFN

DAPTAIFWTDYVATRWYRAPELCGSFFSKYTPAIDIWSIGCIFAELLIGRPLFPGKNVVH

QLDIITDLLGTPSSETLSRIRNEKARRYLSCMRKKHPVPLTQKFPNVDPLALRLLGRLLA

FDPKDRPSAEEALADPYFAVLANVEREPSRNPISKLEFEFERRKVTKDDVRELIYREILE

YHPQMLQEYMQGGEQLSFLYPSGVDRFKRQFAHLEENYSKGERGSPLQRKHASLPRQRVG

ASNDNNDQHASDQERGADSIAPATGNPPGSQDAGQQHVSGGQNGVGSTNISPRSYQKSAS

ISASKCVVVNANKNPEYDDDISEEMEGAVDGLSEKVSRMHP*

>BdTR8iMPK20-1 Brdisv1BdTR8i1019342m.p

MQQDQRKKSSAEAEFFTEYGDASRYKIQEIVGKGSYGVVCSAIDVHTGEKVAIKKIHDIF

EHISDAARILREIKLLRLLRHPDIVEIKHIMLPPSRRDFKDIYVVFELMESDLHQVIKAN

DDLTKEHYQFFLYQLLRALKYIHTANVYHRDLKPKNILANSNCKLKICDFGLARVAFNDT

PTTIFWTDYVATRWYRAPELCGSFFSKYTPAIDVWSIGCIFAEVLTGKPLFPGKNVVHQL

DLMTDLLGTPSMDTISRVRNEKARRYLSSMRKKEPIPFSQKFPNADPLALDLLQRLLAFD

PKDRPTAGEALSHPYFKGLAKVEREPSCQPITKMEFEFERRRATKEDIRELIFREILEYH

PQLLKDYINGTERTTFLYPSAVDQFRKQFAHLEENSESGPVVPMDRKHTSLPRSTIVHSA

PIHAKEQPRIGPSRDRPLTDESYKNPRDSEKYSGNVPRSSHHSQAPQRVPTARPGRVVGP

VLPYENVGTSHPYDPRRVAMNSGYPPQQQIPQTYGYYQTTGKSACSEPSQAERYTLHQQA

YACANSSTAPDVALDMRAPPFHQSAGPKSGSSDRLTAETNLYTRSLNGIVATTSGVAASA

HRKVSVVPYGMSQMY*

>BdTR8iMPK20-2 Brdisv1BdTR8i1015298m.p

MQQPQQQGQRNKSTDVDFFSEYGDANRYRIQEVIGKGSYGVVCSAMDMQTRQKVAIKKIH

NIFDHVSDAARILREIKLLRLLRHPDVVEIKHIMLPPSRKDFKDIYVVFELMESDLHQVI

KANDDLTKEHYQFFLYQLLRALKYIHTASVYHRDLKPKNILANSNCKLKICDFGLARVAF

NDTPTTVFWTDYVATRWYRAPELCGSFFTKYTPAIDIWSIGCIFAEVLTGKPLFPGKNVV

HQLDLMTDLLGTPSMDTISRVRNEKARRYLSSMRKKDPVPFSKKFPNADPLGLKLLEKLL

AFDPKDRPTAEEALTDPYFKSLSKPDREPSCQPIRKVEFDFEHRRMSKDDIRELIFQEIL

EYHPQLLKNYIDGTEKTTFLYPSAVDQFKKQFSHLEESDGSGPVVPTERKHASLPRSTTV

HSTPIPAKEQPLVASSRGRPIANEPCKPWAPGNVPGASQTAHVAQAGRAVGVGSVPPYES

GSGKYPYDATSRPAVSSGYPPQQKIPQTYGYHHHHQTPGAGQSSQAMGGYACGYTKGTTP

PPAAAQDMRASPYHHRSAGTKNDPLNRLAAESDIYTRSLNGIVAAAASAGTGAHRKVGAV

PFGMSGMY*

>BdTR8iMPK20-3 Brdisv1BdTR8i1006471m.p

MQHGDLHKKSAAEMDFFTAYDDANRYKILEVIGKGSYGLVCSANDLQTGEKVAIKKIHNI

FEHISDAARILREIKLLRLLRHPDVVEIKHILLPPSKKDFKDIYVVFELMESDLHQVIKA

NDDLTREHYQFFLYQMLRALKYMHTANVYHRDLKPKNVLANANCKLKICDFGLARVAFSD

APTTVFWTDYVATRWYRAPELCGSFYSKYTPAIDIWSIGCIFAEVLIGKPLFPGKNVVHQ

LDLITDILGTPSLDAISRVRNDKARKYLTCMRKKQPASFSQKFPKADPLALQLLRRLLAF

DPKDRPSAEEALADPYFNGLAKVEREPSCQPIPKIEFEFEGRRVTKEDIKELIFEEILEY

HPQLLKEHIIGKERPNFVHLSAVDQFKKHFTQLEENDNETGAAVSLQRKHSSLPRQAFNH

R*

>BdTR8iMPK20-4 Brdisv1BdTR8i1019564m.p

MQTTEQQRKKGSSEMDFFSEYGDANRYKIQEVIGKGSYGVVCSAIDQHTGDKVAIKKIHN

IFEHLSDAARILREIKLLRLLRHPDIVEIRHIMLPPSRRDFKDIYVVFELMDTDLHQVIK

ANDDLTKEHHQFFLYQMLRALKYIHTANVYHRDLKPKNILANANCKLKICDFGLARVAFN

DTPTTVFWTDYVATRWYRAPELCGSFFTKYSRAIDIWSIGCIFAEILTGKPLFPGKNVVH

QLDLMTDLLGTPSLDTVSRIRNEKARRYLSSMRKKQSVSFSERFPKADPAALKLLQRLLA

FDPKDRPTAEEALADPYFKGLGKVEREPSCQPITKMEFEFERKNVTKADIKELIFREILE

YHPQLLKDYMNGTEKTNFLYPSAVDNFRRQFANLEENGGKGGAVVPPDRKHVSLPRNTTV

HSTPIPPKDQKYSQVPQRIPTGRPGRVVGPVIPFENSSTMDPYSQRRVARNPVLPAAATN

LSAYAYRNSDNSERELQQELEKDRMQYHPMQRFMVAKMVSPDLRSTSYYMPKGVPKADVA

ERSALQSNMMQGIAPFNGIATVGGAFNKVSAVPYGVSRMY*

>BdTR8iMPK20-5 Brdisv1BdTR8i1015450m.p

MFSDIFSREGVAFSRVALRQELLSDQWFTTAGGKGQCYLVPPGTTVFLVNSEEMGFFSEY

VDASRYKILEIIGKGSYGVVCSAIDQETGDKVAIKKIQNIFEHLSDAARILREIKLLRLL

RHPDIVQIKHIMLPPSRRDFRDIYVVFELMDTDLHQVIKANDDLTKEHYQFFLYQMLRAL

KYIHTANVYHRDLKPKNILANANCKLKICDFGLARVAFNDTPTTVFWTDYVATRWYRAPE

LCGSFFTKYSPAIDTWSIGCIFAEILTGKPLFPGKNVVHQLDLMTDLLGIPSTETISRIR

NDKARKYLSSMRRKQPIPFSEKFPNADPSALKLLERLLAFDPKDRPTAEEALAHPYFKRL

ARVEREPSCQQPISKTEFEFERRKFTKEDIKELIFREILEYHPKLLKDYMNGSEKTSFLY

PSAVDNFRRQFANLEIDGGRSGAADRKHFSLPRTTTVHSAPILPTNGPTSQVPQRIPTAR

PGRVVSSAMQTDNPSVSDRHNGRRVARDPAVPPAAAYHLKSDYSDRQHQQEFEKDRVRPD

RQRQEELEKDRMQYRPGHHSMDAKVAPEISPYMRSSPYYIPPFNGIAAVASGYSKVAAVT

RMY*

>BdTR8iMPK21-1 Brdisv1BdTR8i1015345m.p

MGGLIRWLRHHRSRRVSSSSSSHLPSNTTSSSSTSDLRAHSLPQHQGDHHGEVVVEWEDA

AEGPDSDPEEYIVVVLGDDEQGVVAARAPVRTKPPRVMDPGKKTSESEFFTEYGEANRYK

VSEVIGKGSYGVVAAAVDTQTGERCAIKKINDVFDHVSDATRILREIKLLRLLRHPDIVE

IKHIMLPPSRREFRDIYVVFELMESDLHQVIKANDDLTPEHHQFFLYQLLRGMKYIHTAS

VFHRDLKPKNILANADCKLKICDFGLARVSFNDGAPSAIFWTDYVATRWYRAPELCGSFF

SKYTPAIDIWSVGCIFAEMLTGKPLFPGKNVVHQLDLMTDLLGTPSAESLSKIRNEKARR

YLSNMRKKPKVPLTKKFPGIDPMALHLLERLLAFDPKDRPSADEALTDPYFTGLANSERE

PITQPISKLEFEFEKRKLAKDDVRELIYREILEYHPHMLQEYLRGGGDQMSFMFPSGVDR

FKRQFAHLEEGGAKGEKSSPQLRQNASLPRERVIGNKHGDGDYNMKLNVGEKPELASVSD

GISKPLMSARSLLKSETMSASKCIGEIKNKDEDSLSECVEGTDDDVSQKIAQLKT*

>BdTR8iMPK21-2 Brdisv1BdTR8i1019424m.p

GSDEAEFFTEYGEANRYEVGEVVGKGSYGVVAAAVDTHTGERVAIKKINDVFEHVSDATR

ILREIKLLRLLRHPDIVEIKHIMLPPSRREFRDIYIIFELMESDLHQVIKANDDLTPEHH

QFFFYQLLRGMKYIHAANVFHRDLKPRNILANADCKLKICDFGLARVSFNDTPSAIFWTD

YVATRWYRAPELCGSFFSKYTPAIDIWSIGCIFAEMLSGRPLFPGKNVVHQLDLMTDLLG

TPSAESLSRIRNEKARRYLGNMRKKHPVPFSQKFPGVDPMALDLLERLLAFDPKDRPTAA

EALADPYFTGLANSDREPTTQPISKLEFEFERRKLARDDVRELIYREILEYHPQMLHEYH

HGGDQANFVYPSGVDRFKRQFVHLEEGVTKGEKTSPQLRQHASLPRERIIGIGDELGRPN

ADYCIKLHVGEEPGHTSVTDGLSKPLLNARNFLKSESISASQCVVIKEKREKDVSAVTSN

IF*

>Tek-2MPK3 Brdisv1Tek-21010027m.p

MDGAPVAEFRPTMTHGGRFLLYNIFGNQFEITAKYQPPIMPIGRGAYGIVCSVMNFETRE

MVAIKKIANAFDNNMDAKRTLREIKLLRHLDHENIVGLRDVIPPAIPQSFNDVYIATELM

DTDLHHIIRSNQELSEEHCQYFLYQLLRGLKYIHSANVIHRDLKPSNLLLNANCDLKICD

FGLARPSSESDMMTEYVVTRWYRAPELLLNSTDYSAAIDVWSVGCIFMELINRAPLFPGR

DHMHQMRLITEVIGTPTDDDLGFIRNEDARRYMRHLPQFPRRPFPAQFPRVQPAALDLIE

RMLAFNPLQRITVEEALEHPYLERLHDIADEPICTDPFSFDFEQHPLTEDQMKQLIFNEA

LELNPNFRY*

>Tek-2MPK4 Brdisv1Tek-21010816m.p

MDTSGGGGGGGGGAGGAAQIQGMATHGGRYVLYNVYGNLFEVASKYAPPIRPIGRGAYGI

VCAAVSSDTGEEVAIKKIGNAFDNHIDAKRTLREIKLLRHMDHENIIAIKDIIRPPRRDD

FKDVYIVTELMDTDLHQIIRSNQPLTDDHCQYFLYQLLRGLKYVHSANVLHRDLKPSNLF

LNANCDLKIADFGLARTTSETDLMTEYVVTRWYRAPELLLNCSQYTAAIDVWSVGCILGE

IITRQPLFPGRDYIQQLKLITELIGSPDDSSLGFLRSDNARRYMKQLPQYPRQDFRLRFR

NMSDGAVDLLERMLVFDPSRRITVDEALHHPYLASLHDINEEPTCPAPFSFDFEQPSFTE

EHMKELIWRETLAFNPDPPY*

>Tek-2MPK6 Brdisv1Tek-21007328m.p

MDGGAQPPDAEMADAGAAVPGAGAGAAGTMENIQATLSHGGRFIQYNIFGNVFEVTVKYK

PPILPIGKGAYGIVCSALNSETGEQVAIKKIANAFDNKIDAKRTLREIKLLRHMDHENIV

AIRDIIPPAQRNSFNDVYIAYELMDTDLHQIIRSNQALSEEHCQYFLYQILRGLKYIHSA

NVLHRDLKPSNLLLNANCDLKICDFGLARTTSETDFMTEYVVTRWYRAPELLLNSSEYTA

AIDVWSVGCIFMELMDRKPLFPGRDHVHQLRLLMELIGTPNEADLDFVNENARRYIRQLP

RHARQSFSEKFPHVHPSAIDLVEKMLTFDPRQRITVEGALAHPYLASLHDISDEPVCTMP

FSFDFEQHALSEEQMKDLIHQEALAFNPDYQ*

>Tek-2MPK7-1 Brdisv1Tek-21005308m.p

MPSRFPFLSPGCFIRLLSGSASSDLLLLLPRRGQSAPRARVLLKMAMMVDPPNGTGNHGK

HYYTMWQTMFEIDTKYVPIKPIGRGAYGIVCSSTNQENNEKVAIKKINNVFDNRVDALRT

LRELKLLRHLRHENVIALKDIMMPVHRRSFKDVYLVSELMDTDLHQIIKSSQPLSNDHCQ

YFLFQLLRGLKYLHSAGILHRDLKPGNLLVNANCDLKICDFGLARTNNTKGQFMTEYVVT

RWYRAPELLLCCDNYGTSIDVWSVGCIFAELLGRKPIFPGTECLNQLKLIVNVLGTMSEN

DLEFIDNPKARKYIKSLPYTPGTPLTSMYPQAHPLAIDLLQKMLVFDPSKRISVTEALEH

PYMSPLYDPSANPPAQVPIDLDIDENLGVEMIREMLWQEMLQYHPEAARMVNM*

>Tek-2MPK11 Brdisv1Tek-21030943m.p

MRMEGGGAGPAAAAAGGGAHGLGEAQIKGTLTHGGRYVQYNVYGNLFEVSAKYVPPIRPV

GRGACGIICAAINVQTREEVAIKKIGNAFDNQIDAKRTLREVKLLRHMNHENVISIKDII

RPPRRENFNDVYIVYELMDTDLHHLLRSNQPLTDDHCQYFLYQVLRGLKYVHSANVLHRD

LRPSNLLLNAKCDLKIGDFGLARTTTETDFMMEYVVTRWYRAPELLLNCSEYTGAIDMWS

VGCILGEIATREPLFPGKDYVHQLRLITELLGSPDDTSLGFLRSDNARRYVRSLPQYPKQ

QFRSRFPNMSSGAMDLLERMLVFDPNKRITVDEALCHPYLASLHEINDEPVCPAPFSFDF

EQPSFTEEDIKELIWRESVKFNPEPIH*

>Tek-2MPK14 Brdisv1Tek-21028844m.p

MAMLVDPPNGMGNQGKHYYSMWQTLFEIDTKYVPIKPIGRGAYGIVCSSINRETNEKVAI

KKIHNVFDNRVDALRTLRELKLLRHLRHENVISLKDIMMPVQRRSFKDVYLVYELMDTDL

HQIIKSPQGLSNDHCQYFLFQLLRGLKYLHSAEILHRDLKPGNLLVNANCDLKICDFGLA

RTNSSKGQFMTEYVVTRWYRAPELLLCCDNYGTSIDVWSVGCIFAELLGRKPIFPGTECL

NQLKLIVNVLGTMSESDLEFIDNPKARRYIKTLPYTPGVPLASMYPHAHPLAIDLLQKML

IFDPTKRISVTEALEHPYMSPLYDPSANPPAQVPIDLDLDENISADMIREMMWQEMLHYH

PEAATAISM*

>Tek-2MPK16 Brdisv1Tek-21017203m.p

MDFFTEYGEGNRYKIEEVIGKGSYGVVCSALDTHTGDKVAIKKINDIFEHVSDATRILRE

IKLLRLLRHPDIVEIKHILLPPSRREFKDIYVVFELMESDLHQVIKANDDLTPEHYQFFL

YQLLRGLKYIHTANVFHRDLKPKNILANADCKLKICDFGLARVAISDTPTAIFWTDYIAT

RWYRAPELCGSFFSKYTPAIDIWSIGCIFAELLTGKPLFPGKNVVHQLDIITDLLGTPSA

ETIARIRNEKARRYLSSMRRKKTVPFTQKFPNADPLALNLLERMLAFDPKDRPSAEEALA

DPYFKNIASVDREPSAQPITKLEFEFERRRITKDDIRELIYREILEYHPNMLREFLEGAE

PTGFMYPSAVDHFKKQFTFLEEHYAKGSTAAPPERQHNSLPRPSVIYSDNRPQNAANITD

DLSRCAIRDNTQKAQKDSASVGANRIPHGAAAAAARPGKVVGSVLRYGNCSTSGTEQYEQ

RRVVRSPGIAPNGVTSGSSYPRRNYTCKSETGEAERIDTNQGGPPKPYVANKLPATVDGR

NGHW*

>Tek-2MPK17 Brdisv1Tek-21005426m.p

MVGGGGIVDGFRRLFHRRTPSGSGPSSNQSSAGEESSDIDAVVEDLDLVGLRAIRVPKRK

MPLPVESHKKNILEKEFFTEYGEASQYQIQEVIGKGSYGVVAAAVDTRTGERVAIKKIND

VFEHVSDATRILREVKLLRLLRHPDVVEIKHIMLPPSRREFQDIYVVFELMESDLHQVIR

ANDDLTPEHYQFFLYQLLRALKYIHAANVFHRDLKPKNILANSDCKLKICDFGLARVSFN

DAPTAIFWTDYVATRWYRAPELCGSFFSKYTPAIDIWSIGCIFAELLTGRPLFPGKNVVH

QLDIITDLLGTPSSETLSRIRNEKARRYLSCMRKKHPVPLTQKFPNVDPLALRLLGRLLA

FDPKDRPSAEEALADPYFAVLANVEREPSRNPISKLEFEFERRKVTKDDVRELIYREILE

YHPQMLQEYMQGGEQLSFLYPSGVDRFKRQFAHLEENYSKGERGSPLQRKHASLPRQRVG

ASNDNNDQHASDQERGADSIAPATGNPPGSQDAGQQHVSGGQNGVGSTNISPRSYQKSAS

ISASKCVVVNANKNPEYDDDISEEMEGAVDGLSEKVSRMHP*

>Tek-2MPK20-1 Brdisv1Tek-21018480m.p

MQQDQRKKSSAEAEFFTEYGDASRYKIQEIVGKGSYGVVCSAIDVHTGEKVAIKKIHDIF

EHISDAARILREIKLLRLLRHPDIVEIKHIMLPPSRRDFKDIYVVFELMESDLHQVIKAN

DDLTKEHYQFFLYQLLRALKYIHTANVYHRDLKPKNILANSNCKLKICDFGLARVAFNDT

PTTIFWTDYVATRWYRAPELCGSFFSKYTPAIDVWSIGCIFAEVLTGKPLFPGKNVVHQL

DLMTDLLGTPSMDTISRVRNEKARRYLSSMRKKEPIPFSQKFPNADPLALDLLQRLLAFD

PKDRPTAGEALSHPYFKGLAKVEREPSCQPITKMEFEFERRRATKEDIRELIFREILEYH

PQLLKDYINGTERTTFLYPSAVDQFRKQFAHLEENSESGPVVPMDRKHTSLPRSTIVHSA

PIHAKEQPRIGPSRDRPLTDESYKNPRDSEKYSGNVPRSSHHSQAPQRVPTARPGRVVGP

VLPYENVGTSHPYDPRRVAMNSGYPPQQQIPQTYGYYQTTGKSACSEPSQAERYTLHQQA

YACANSSTAPDVALDMRAPPFHQSAGPKSGSSDRLTAETNLYTRSLNGIVATTSGVAASA

HRKVSVVPYGMSQMY*

>Tek-2MPK20-2 Brdisv1Tek-21043384m.p

MQQPQQQGQRNKSTDVDFFSEYGDANRYRIQEVIGKGSYGVVCSAMDMQTRQKVAIKKIH

NIFDHVSDAARILREIKLLRLLRHPDVVEIKHIMLPPSRKDFKDIYVVFELMESDLHQVI

KANDDLTKEHYQFFLYQLLRALKYIHTASVYHRDLKPKNILANSNCKLKICDFGLARVAF

NDTPTTVFWTDYVATRWYRAPELCGSFFTKYTPAIDIWSIGCIFAEVLTGKPLFPGKNVV

HQLDLMTDLLGTPSMDTISRVRNEKARRYLSSMRKKDPVPFSKKFPNADPLGLKLLEKLL

AFDPKDRPTAEEALTDPYFKSLSKPDREPSCQPIRKVEFDFEHRRMSKDDIRELIFQEIL

EYHPQLLKNYIDGTEKTTFLYPSAVDQFKKQFSHLEESDGSGPVVPTERKHASLPRSTTV

HSTPIPAKEQPLVASSRGRPIANEPCKPWAPGNVPGASQTAHVAQAGRAVGVGSVPPYES

GSGKYPYDATSRPAVSSGYPPQQKIPQTYGYHHHHQTPGAGQSSQAMGGYACGYTKGTTP

PPAAAQDMRASPYHHRSAGTKNDPLNRLAAESDIYTRSLNGIVAAAASAGTGAHRKVGAV

PFGMSGMY*

>Tek-2MPK20-3 Brdisv1Tek-21043278m.p

MQHGDLHKKSAAEMDFFTAYDDANRYKILEVIGKGSYGLVCSANDLQTGEKVAIKKIHNI

FEHISDAARILREIKLLRLLRHPDVVEIKHILLPPSKKDFKDIYVVFELMESDLHQVIKA

NDDLTREHYQFFLYQMLRALKYMHTANVYHRDLKPKNVLANANCKLKICDFGLARVAFSD

APTTVFWTDYVATRWYRAPELCGSFYSKVMIVTIDH*

>Tek-2MPK20-4 Brdisv1Tek-21018691m.p

MQTTEQQRKKGSSEMDFFSEYGDANRYKIQEVIGKGSYGVVCSAIDQHTGDKVAIKKIHN

IFEHLSDAARILREIKLLRLLRHPDIVEIRHIMLPPSRRDFKDIYVVFELMDTDLHQVIK

ANDDLTKEHHQFFLYQMLRALKYIHTANVYHRDLKPKNILANANCKLKICDFGLARVAFN

DTPTTVFWTDYVATRWYRAPELCGSFFTKYSRAIDIWSIGCIFAEILTGKPLFPGKNVVH

QLDLMTDLLGTPSLDTVSRIRNEKARRYLSSMRKKQSVSFSERFPKADPAALKLLQRLLA

FDPKDRPTAEEALADPYFKGLGKVEREPSCQPITKMEFEFERKNVTKADIKELIFREILE

YHPQLLKDYMNGTEKTNFLYPSAVDNFRRQFANLEENGGKGGAVVPPDRKHVSLPRNTTV

HSTPIPPKDQKYSQVPQRIPTGRPGRVVGPVIPFENSSTMDPYSQRRVARNPVLPAAATN

LSAYAYRNSDNSERELQQELEKDRMQYHPMQRFMVAKMVSPDLRSTSYYMPKGVPKADVA

ERSALQSNMMQGIAPFNGIATVGGAFNKVSAVPYGVSRMY*

>Tek-2MPK20-5 Brdisv1Tek-21014805m.p

MFSDIFSREGVAFSRVALRQELLSDQWFTTAGGKGQCYLVPPGTTVFLVNSEEMGFFSEY

VDASRYKILEIIGKGSYGVVCSAIDQETGDKVAIKKIQNIFEHLSDAARILREIKLLRLL

RHPDIVQIKHIMLPPSRRDFRDIYVVFELMDTDLHQVIKANDDLTKEHYQFFLYQMLRAL

KYIHTANVYHRDLKPKNILANANCKLKICDFGLARVAFNDTPTTVFWTDYVATRWYRAPE

LCGSFFTKYSPAIDTWSIGCIFAEILTGKPLFPGKNVVHQLDLMTDLLGIPSTETISRIR

NDKARKYLSSMRRKQPIPFSEKFPNADPSALKLLERLLAFDPKDRPTAEEALAHPYFKRL

ARVEREPSCQQPISKTEFEFERRKFTKEDIKELIFREILEYHPKLLKDYMNGSEKTSFLY

PSAVDNFRRQFANLEIDGGRSGAADRKHFSLPRTTTVHSAPILPTNGPTSQVPQRIPTAR

PGRVVSSAMQTDNPSVSDRHNGRRVARDPAVPPAAAYHLKSDYSDRQHQQEFEKDRVRPD

RQRQEELEKDRMQYRPGHHSMDAKVAPEISPYMRSSPYYIPPFNGIAAVASGYSKVAAVT

RMY*

>Tek-2MPK21-1 Brdisv1Tek-21014698m.p

MGGLIRWLRHHRSRRVSSSSSSHLPSNTTSSSSTSDLRAHSLPQHQGDHHGEVVVEWEDA

AEGPDSDPEEYIVVVLGDDEQGVVAARAPVRTKPPRVMDPGKKTSESEFFTEYGEANRYK

VSEVIGKGSYGVVAAAVDTQTGERCAIKKINDVFDHVSDATRILREIKLLRLLRHPDIVE

IKHIMLPPSRREFRDIYVVFELMESDLHQVIKANDDLTPEHHQFFLYQLLRGMKYIHTAS

VFHRDLKPKNILANADCKLKICDFGLARVSFNDGAPSAIFWTDYVATRWYRAPELCGSFF

SKYTPAIDIWSVGCIFAEMLTGKPLFPGKNVVHQLDLMTDLLGTPSAESLSKIRNEKARR

YLSNMRKKPKVPLTKKFPGIDPMALHLLERLLAFDPKDRPSADEALTDPYFTGLANSERE

PITQPISKLEFEFEKRKLAKDDVRELIYREILEYHPHMLQEYLRGGGDQMSFMFPSGVDR

FKRQFAHLEEGGAKGEKSSPQLRQNASLPRERVIGNKHGDGDYNMKLNVGEKPELASVSD

GISKPLMSARSLLKSETMSASKCIGEIKNKDEDSLSECVEGTDDDVSQKIAQLKT*

>Tek-2MPK21-2 Brdisv1Tek-21018561m.p

GSDEAEFFTEYGEANRYEVGEVVGKGSYGVVAAAVDTHTGERVAIKKINDVFEHVSDATR

ILREIKLLRLLRHPDIVEIKHIMLPPSRREFRDIYIIFELMESDLHQVIKANDDLTPEHH

QFFFYQLLRGMKYIHAANVFHRDLKPRNILANADCKLKICDFGLARVSFNDTPSAIFWTD

YVATRWYRAPELCGSFFSKYTPAIDIWSIGCIFAEMLSGRPLFPGKNVVHQLDLMTDLLG

TPSAESLSRIRNEKARRYLGNMRKKHPVPFSQKFPGVDPMALDLLERLLAFDPKDRPTAA

EALADPYFTGLANSDREPTTQPISKLEFEFERRKLARDDVRELIYREILEYHPQMLHEYH

HGGDQANFVYPSGVDRFKRQFVHLEEGVTKGEKTSPQLRQHASLPRERIIGIGDELGRPN

ADYCIKLHVGEEPGHTSVTDGLSKPLLNARNFLKSESISASQCVVIKEKREKDVSAVTSN

IF*

>Tek-4MPK3 Brdisv1Tek-41030977m.p

MDGAPVAEFRPTMTHGGRFLLYNIFGNQFEITAKYQPPIMPIGRGAYGIVCSVMNFETRE

MVAIKKIANAFDNNMDAKRTLREIKLLRHLDHENIVGLRDVIPPAIPQSFNDVYIATELM

DTDLHHIIRSNQELSEEHCQYFLYQLLRGLKYIHSANVIHRDLKPSNLLLNANCDLKICD

FGLARPSSESDMMTEYVVTRWYRAPELLLNSTDYSAAIDVWSVGCIFMELINRAPLFPGR

DHMHQMRLITEVIGTPTDDDLGFIRNEDARRYMRPLPQFPRRPFPAQFPRVQPAALDLIE

RMLAFNPLQRIT

>Tek-4MPK4 Brdisv1Tek-41029583m.p

MDTSGGGGGGGGGAGGAAQIQGMATHGGRYVLYNVYGNLFEVASKYAPPIRPIGRGAYGI

VCAAVSSDTGEEVAIKKIGNAFDNHIDAKRTLREIKLLRHMDHENIIAIKDIIRPPRRDD

FKDVYIVTELMDTDLHQIIRSNQPLTDDHCQYFLYQLLRGLKYVHSANVLHRDLKPSNLF

LNANCDLKIADFGLARTTSETDLMTEYVVTRWYRAPELLLNCSQYTAAIDVWSVGCILGE

IITRQPLFPGRDYIQQLKLITELIGSPDDSSLGFLRSDNARRYMKQLPQYPRQDFRLRFR

NMSDGAVDLLERMLVFDPSRRITVDEALHHPYLASLHDINEEPTCPAPFSFDFEQPSFTE

EHMKELIWRETLAFNPDPPY*

>Tek-4MPK6 Brdisv1Tek-41035605m.p

VQIVAIRDIIPPAQRNSFNDVYIAYELMDTDLHQIIRSNQALSEEHCQYFLYQILRGLKY

IHSANVLHRDLKPSNLLLNANCDLKICDFGLARTTSETDFMTEYVVTRWYRAPELLLNSS

EYTAAIDVWSVGCIFMELMDRKPLFPGRDHVHQLRLLMELIGTPNEADLDFVNENARRYI

RQLPRHARQSFSEKFPHVHPSAIDLVEKMLTFDPRQRITVEGALAHPYLASLHDISDEPV

CTMPFSFDFEQHALSEEQMKDLIHQEALA*

>Tek-4MPK7-1 Brdisv1Tek-41014125m.p

MLKMAMMVDPPNGTGNHGKHYYTMWQTMFEIDTKYVPIKPIGRGAYGIVCSSTNQENNEK

VAIKKINNVFDNRVDALRTLRELKLLRHLRHENVIALKDIMMPVHRRSFKDVYLVFELMD

TDLHQIIKSSQPLSNDHCQYFLFQLLRGLKYLHSAGILHRDLKPGNLLVNANCDLKICDF

GLARTNNTKGQFMTEYVVTRWYRAPELLLCCDNYGTSIDVWSVGCIFAELLGRKPIFPGT

ECLNQLKLIVNVLGTMSENDLEFIDNPKARKYIKSLPYTPGTPLTSMYPQAHPLAIDLLQ

KMLVFDPSKRISVTEALEHPYMSPLYDPSANPPAQVPIDLDIDENLGVEMIREMLWQEML

QYHPEAARMVNM*

>Tek-4MPK11 Brdisv1Tek-41017616m.p

MRMEGGGAGPAAAAAGGGAHGLGEAQIKGTLTHGGRYVQYNVYGNLFEVSAKYVPPIRPV

GRGACGIICAAINVQTREEVAIKKIGNAFDNQIDAKRTLREVKLLRHMNHENVISIKDII

RPPRRENFNDVYIVYELMDTDLPPLLRSNQPLTDDHCQYFLYQVLRGLKYVHSANVLHRD

LRPSNLLLNAKCDLKIGDFGLARTTTETDFMMEYVVTRWYRAPELLLNCSEYTGAIDMWS

VGCILGEIATREPLFPGKDYVHQLRLITELLGSPDDTSLGFLRSDNARRYVRSLPQYPKQ

QFRSRFPNMSSGAMDLLERMLVFDPNKRITVDEALCHPYLASLHEINDEPVCPAPFSFDF

EQPSFTEEDIKELIWRESVKFNPEPIH*

>Tek-4MPK14 Brdisv1Tek-41016381m.p

MAMLVDPPNGMGNQGKHYYSMWQTLFEIDTKYVPIKPIGRGAYGIDCSSINRETNEKVAI

KKIHNVFDNRVDALRTLRELKLLRHLRHENVISLKDIMMPVQRRSFKDVYLVYELMDTDL

HQIIKSPQGLSNDHCQYFLFQLLRGLKYLHSAEILHRDLKPGNLLVNANCDLKICDFGLA

RTNSSKGQFMTEYVVTRWYRAPELLLCCDNYGTSIDVWSVGCIFAELLGRKPIFPGTECL

NQLKLIVNVLGTMSESDLEFIDNPKARRYIKTLPYTPGVPLASMYPHAHPLAIDLLQKML

IFDPTKRISVTEALEHPYMSPLYDPSANPPAQVPIDLDLDENISADMIREMMWQEMLHYH

PEAATAISM*

>Tek-4MPK16 Brdisv1Tek-41034938m.p

MDFFTEYGEGNRYKIEEVIGKGSYGVVCSALDTHTGDKVAIKKINDIFEHVSDATRILRE

IKLLRLLRHPDIVEIKHILLPPSRREFKDIYVVFELMESDLHQVIKANDDLTPEHYQFFL

YQLLRGLKYIHTANVFHRDLKPKNILANADCKLKICDFGLARDYIATRWYRAPELCGSFF

SKV*

>Tek-4MPK17 Brdisv1Tek-41003047m.p

MVGGGGIVDGFRRLFHRRTPSGSGPSSNQSSAGEESSDIDAVVEDLDLVGLRAIRVPKRK

MPLPVESHKKNILEKEFFTEYGEASQYQIQEVIGKGSYGVVAAAVDTRTGERVAIKKIND

VFEHVSDATRILREVKLLRLLRHPDVVEIKHIMLPPSRREFQDIYVVFELMESDLHQVIR

ANDDLTPEHYQFFLYQLLRALKYIHAANVFHRDLKPKNILANSDCKLKICDFGLARVSFN

DAPTAIFWTDYVATRWYRAPELCGSFFSKYTPAIDIWSIGCIFAELLTGRPLFPGKNVVH

QLDIITDLLGTPSSETLSRIRNEKARRYLSCMRKKHPVPLTQKFPNVDPLALRLLGRLLA

FDPKDRPSAEEALADPYFAVLANVEREPSRNPISKLEFEFERRKVTKDDVRELIYREILE

YHPQMLQEYMQGGEQLSFLYPSGVDRFKRQFAHLEENYSKGERGSPLQRKHASLPRQRVG

ASNDNNDQHASDQERGADSIAPATGNPPGSQDAGQQHVSGGQNGVGSTNISPRSYQKSAS

ISASKCVVVNANKNPEYDDDISEEMEGAVDGLSEKVSRMHP*

>Tek-4MPK20-1 Brdisv1Tek-41029227m.p

MQQDQRKKSSAEAEFFTEYGDASRYKIQEIVGKGSYGVVCSAIDVHTGEKVAIKKIHDIF

EHISDAARILREIKLLRLLRHPDIVEIKHIMLPPSRRDFKDIYVVFELMESDLHQVIKAN

DDLTKEHYQFFLYQLLRALKYIHTANVYHRDLKPKNILANSNCKLKICDFGLARVAFNDT

PTTIFWTDYVATRWYRAPELCGSFFSKH*

>Tek-4MPK20-2 Brdisv1Tek-41027315m.p

MQQPQQQGQRNKSTDVDFFSEYGDANRYRIQEVIGKGSYGVVCSAMDMQTRQKVAIKKIH

NIFDHVSDAARILREIKLLRLLRHPDVVEIKHIMLPPSRKDFKDIYVVFELMESDLHQVI

KANDDLTKEHYQFFLYQLLRALKYIHTASVYHRDLKPKNILANSNCKLKICDFGLARVAF

NDTPTTVFWTDYVATRWYRAPELCGSFFTKYTPAIDIWSIGCIFAEVLTGKPLFPGKNVV

HQLDLMTDLLGTPSMDTISRVRNEKARRYLSSMRKKDPVPFSKKFPNADPLGLKLLEKLL

AFDPKDRPTAEEALTDPYFKSLSKPDREPSCQPIRKVEFDFEHRRMSKDDIRELIFQEIL

EYHPQLLKNYIDGTEKTTFLYPSAVDQFKKQFSHLEESDGSGPVVPTERKHASLPRSTTV

HSTPIPAKEQPLVASSRGRPIANEPCKPWAPGNVPGASQTAHVAQAGRAVGVGSVPPYES

GSGKYPYDATSRPAVSSGYPPQQKIPQTYGYHHHHQTPGAGQSSQAMGGYACGYTKGTTP

PPAAAQDMRASPYHHRSAGTKNDPLNRLAAESDIYTRSLNGIVAAAASAGTGAHRKVGAV

PFGMSGMY*

>Tek-4MPK20-3 Brdisv1Tek-41027738m.p

MQHGDLHKKSAAEMDFFTAYDDANRYKILEVIGKGSYGLVCSANDLQTGEKVAIKKIHNI

FEHISDAARILREIKLLRLLRHPDVVEIKHILLPPSKKDFKDIYVVFELMESDLHQVIKA

NDDLTREHYQFFLYQMLRALKYMHTANVYHRDLKPKNVLANANCKLKICDFGLARVAFSD

APTTVFWTDYVATRWYRAPELCGSFYSK

>Tek-4MPK20-5 Brdisv1Tek-41008316m.p

MFSDIFSREGVAFSRVALRQELLSDQWFTTAGGKGQCYLVPPGTTVFLVNSEEMGFFSEY

VDASRYKILEIIGKGSYGVVCSAIDQETGDKVAIKKIQNIFEHLSDAARILREIKLLRLL

RHPDIVQIKHIMLPPSRRDFRDIYVVFELMDTDLHQVIKANDDLTKEHYQFFLYQMLRAL

KYIHTANVYHRDLKPKNILANANCKLKICDFGLARVAFNDTPTTVFWTDYVATRWYRAPE

LCGSFFTKYSPAIDTWSIGCIFAEILTGKPLFPGKNVVHQLDLMTDLLGIPSTETISRIR

NDKARKYLSSMRRKQPIPFSEKFPNADPSALKLLERLLAFDPKDRPTAEEALAHPYFKRL

ARVEREPSCQQPISKTEFEFERRKFTKEDIKELIFREILEYHPKLLKDYMNGSEKTSFLY

PSAVDNFRRQFANLEIDGGRSGAADRKHFSLPRTTTVHSAPILPTNGPTSQVPQRIPTAR

PGRVVSSAMQTDNPSVSDRHNGRRVARDPAVPPAAAYHLKSDYSDRQHQQEFEKDRVRPD

RQRQEELEKDRMQYRPGHHSMDAKVAPEISPYMRSSPYYIPPFNGIAAVASGYSKVAAVT

RMY*

>Tek-4MPK21-1 Brdisv1Tek-41008225m.p

MGGLIRWLRHHRSRRVSSSSSSHLPSNTTSSSSTSDLRAHSLPQHQGDHHGEVVVEWEDA

AEGPDSDPEEYIVVVLGDDEQGVVAARAPVRTKPPRVMDPGKKTSESEFFTEYGEANRYK

VSEVIGKGSYGVVAAAVDTQTGERCAIKKINDVFDHVSDATRILREIKLLRLLRHPDIVE

IKHIMLPPSRREFRDIYVVFELMESDLHQVIKANDDLTPEHHQFFLYQLLRGMKYIHTAS

VFHRDLKPKNILANADCKLKICDFGLARVSFNDGAPSAIFWTDYVATRWYRAPELCGSFF

SKYTPAIDIWSVGCIFAEMLTGKPLFPGKNVVHQLDLMTDLLGTPSAESLSKIRNEKARR

YLSNMRKKPKVPLTKKFPGIDPMALHLLERLLAFDPKDRPSADEALTDPYFTGLANSERE

PITQPISKLEFEFEKRKLAKDDVRELIYREILEYHPHMLQEYLRGGGDQMSFMFPSGVDR

FKRQFAHLEEGGAKGEKSSPQLRQNASLPRERVIGNKHGDGDYNMKLNVGEKPELASVSD

GISKPLMSARSLLKSETMSASKCIGEIKNKDEDSLSECVEGTDDDVSQKIAQLKT*

>Tek-4MPK21-2 Brdisv1Tek-41031014m.p

MAKKGSDEAEFFTEYGEANRYEVGEVVGKGSYGVVAAAVDTHTGERVAIKKINDVFEHVS

DATRILREIKLLRLLRHPDIVEIKHIMLPPSRREFRDIYIIFELMESDLHQVIKANDDLT

PEHHQFFFYQLLRGMKYIHAANVFHRDLKPRNILANADCKLKICDFGLARVSFNDTPSAI

FWTDYVATRWYRAPELCGSFFSKYTPAIDIWSIGCIFAEMLSGRPLFPGKNVVHQLDLMT

DLLGTPSAESLSRIRNEKARRYFGKHEKKASSAFFSEVSWC*

>Bd29-1MPK3 Brdisv1Bd29-11009003m.p

MDGAPVAEFRPTMTHGGRFLLYNIFGNQFEITAKYQPPIMPIGRGAYGIVCSVMNFETRE

MVAIKKIANAFDNNMDAKRTLREIKLLRHLDHENIVGLRDVIPPAIPQSFNDVYIATELM

DTDLHHIIRSNQELSEEHCQYFLYQLLRGLKYIHSANVIHRDLKPSNLLLNANCDLKICD

FGLARPSSESDMMTEYVVTRWYRAPELLLNSTDYSAAIDVWSVGCIFMELINRAPLFPGR

DHMHQMRLITEVIGTPTDDDLGFIRNEDARRYMRHLPQFPRRPFPAQFPRVQPAALDLIE

RMLAFNPLQRITVEEALEHPYLERLHDIADEPICTDPFSFDFEQHPLTEDQMKQLIFNEA

LELNPNFRY*

>Bd29-1MPK4 Brdisv1Bd29-11009699m.p

MDTSGGGGGGGGGAGGAAQIQGMATHGGRYVLYNVYGNLFEVASKYAPPIRPIGRGAYGI

VCAAVSSDTGEEVAIKKIGNAFDNHIDAKRTLREIKLLRHMDHENIIAIKDIIRPPRRDD

FKDVYIVTELMDTDLHQIIRSNQPLTDDHCQYFLYQLLRGLKYVHSANVLHRDLKPSNLF

LNANCDLKIADFGLARTTSETDLMTEYVVTRWYRAPELLLNCSQYTAAIDVWSVGCILGE

IITRQPLFPGRDYIQQLKLITELIGSPDDSSLGFLRSDNARRYMKQLPQYPRQDFRLRFR

NMSDGAVDLLERMLVFDPSRRITVDEALHHPYLASLHDINEEPTCPAPFSFDFEQPSFTE

EHMKELIWRETLAFNPDPPY*

>Bd29-1MPK6 Brdisv1Bd29-11006575m.p

MDGGAQPPDAEMADAGAAVPGAGAGAAGTMENIQATLSHGGRFIQYNIFGNVFEVTVKYK

PPILPIGKGAYGIVCSALNSETGEQVAIKKIANAFDNKIDAKRTLREIKLLRHMDHENIV

AIRDIIPPAQRNSFNDVYIAYELMDTDLHQIIRSNQALSEEHCQYFLYQILRGLKYIHSA

NVLHRDLKPSNLLLNANCDLKICDFGLARTTSETDFMTEYVVTRWYRAPELLLNSSEYTA

AIDVWSVGCIFMELMDRKPLFPGRDHVHQLRLLMELIGTPNEADLDFVNENARRYIRQLP

RHARQSFSEKFPHVHPSAIDLVEKMLTFDPRQRITVEGALAHPYLASLHDISDEPVCTMP

FSFDFEQHALSEEQMKDLIHQEALAFNPDYQ*

>Bd29-1MPK7-1 Brdisv1Bd29-11022801m.p

MPSRFPFLSPGCFIRLLSGSASSDLLLLLPRRGQSAPRARVLLKMAMMVDPPNGTGNHGK

HYYTMWQTMFEIDTKYVPIKPIGRGAYGIVCSSTNQENNEKVAIKKINNVFDNRVDALRT

LRELKLLRHLRHENVIALKDIMMPVHRRSFKDVYLVSELMDTDLHQIIKSSQPLSNDHCQ

YFLFQLLRGLKYLHSAGILHRDLKPGNLLVNANCDLKICDFGLARTNNTKGQFMTEYVVT

RWYRAPELLLCCDNYGTSIDVWSVGCIFAELLGRKPIFPGTECLNQLKLIVNVLGTMSEN

DLEFIDNPKARKYIKSLPYTPGTPLTSMYPQAHPLAIDLLQKMLVFDPSKRISVTEALEH

PYMSPLYDPSANPPAQVPIDLDIDENLGVEMIREMLWQEMLQYHPEAARMVNM*

>Bd29-1MPK11 Brdisv1Bd29-11027994m.p

MRMEGGGAGPAAAAAAGGAHGLGEAQIKGTLTHGGRYVQYNVYGNLFEVSAKYVPPIRPV

GRGACGIICAAINVQTREEVAIKKIGNAFDNQIDAKRTLREVKLLRHMNHENVISIKDII

RPPRRENFNDVYIVYELMDTDLHHLLRSNQPLTDDHCQYFLYQVLRGLKYVHSANVLHRD

LRPSNLLLNAKCDLKIGDFGLARTTTETDFMMEYVVTRWYRAPELLLNCSEYTGAIDMWS

VGCILGEIATREPLFPGKDYVHQLRLITELLGSPDDTSLGFLRSDNARRYVRSLPQYPKQ

QFRSRFPNMSSGAMDLLERMLVFDPNKRITVDEALCHPYLASLHEINDEPVCPAPFSFDF

EQPSFTEEDIKELIWRESVKFNPEPIH*

>Bd29-1MPK14 Brdisv1Bd29-11039982m.p

MAMLVDPPNGMGNQGKHYYSMWQTLFEIDTKYVPIKPIGRGAYGIVCSSINRETNEKVAI

KKIHNVFDNRVDALRTLRELKLLRHLRHENVISLKDIMMPVQRRSFKDVYLVYELMDTDL

HQIIKSPQGLSNDHCQYFLFQLLRGLKYLHSAEILHRDLKPGNLLVNANCDLKICDFGLA

RTNSSKGQFMTEYVVTRWYRAPELLLCCDNYGTSIDVWSVGCIFAELLGRKPIFPGTECL

NQLKLIVNVLGTMSESDLEFIDNPKARRYIKTLPYTPGVPLASMYPHAHPLAIDLLQKML

IFDPTKRISVTEALEHPYMSPLYDPSANPPAQVPIDLDLDENISADMIREMMWQEMLHYH

PEAATAISM*

>Bd29-1MPK16 Brdisv1Bd29-11015609m.p

MDFFTEYGEGNRYKIEEVIGKGSYGVVCSALDTHTGDKVAIKKINDIFEHVSDATRILRE

IKLLRLLRHPDIVEIKHILLPPSRREFKDIYVVFELMESDLHQVIKANDDLTPEHYQFFL

YQLLRGLKYIHTANVFHRDLKPKNILANADCKLKICDFGLARVAISDTPTAIFWTDYIAT

RWYRAPELCGSFFSKYTPAIDIWSIGCIFAELLTGKPLFPGKNVVHQLDIITDLLGTPSA

ETIARIRNEKARRYLSSMRRKKTVPFTQKFPNADPLALNLLERMLAFDPKDRPSAEEALA

DPYFKNIASVDREPSAQPITKLEFEFERRRITKDDIRELIYREILEYHPNMLREFLEGAE

PTGFMYPSAVDHFKKQFTFLEEHYAKGSTAAPPERQHNSLPRPSVIYSDNRPQNAANITD

DLSRCAIRDNTQKAQKDSASVGANRIPHGAAAAAARPGKVVGSVLRYGNCSTSGTEQYEQ

RRVVRSPGIAPNGVTSGSSYPRRNNTCKSETGEAERIDTNQGGPPKPYVANKLPATVDGR

NGHW*

>Bd29-1MPK17 Brdisv1Bd29-11004857m.p

MVGGGGIVDGFRRLFHRRTPSGSGPSSNQSSAGEESSDIDAVVEDLDLVGLRAIRVPKRK

MPLPVESHKKNILEKEFFTEYGEASQYQIQEVIGKGSYGVVAAAVDTRTGERVAIKKIND

VFEHVSDATRILREVKLLRLLRHPDVVEIKHIMLPPSRREFQDIYVVFELMESDLHQVIR

ANDDLTPEHYQFFLYQLLRALKYIHAANVFHRDLKPKNILANSDCKLKICDFGLARVSFN

DAPTAIFWTDYVATRWYRAPELCGSFFSKYTPAIDIWSIGCIFAELLTGRPLFPGKNVVH

QLDIITDLLGTPSSETLSRIRNEKARRYLSCMRKKHPVPLTQKFPNVDPLALRLLGRLLA

FDPKDRPSAEEALADPYFAVLANVEREPSRNPISKLEFEFERRKVTKDDVRELIYREILE

YHPQMLQEYMQGGEQLSFLYPSGVDRFKRQFAHLEENYSKGERGSPLQRKHASLPRQRVG

ASNDNNDQHASDQERGADSIAPATGNPPGSQDAGQQHVSGGQNGVGSTNISPRSYQKSAS

ISASKCVVVNANKNPEYDDDISEEMEGAVDGLSEKVSRMHP*

>Bd29-1MPK20-1 Brdisv1Bd29-11016791m.p

MQQDQRKKSSAEAEFFTEYGDASRYKIQEIVGKGSYGVVCSAIDVHTGEKVAIKKIHDIF

EHISDAARILREIKLLRLLRHPDIVEIKHIMLPPSRRDFKDIYVVFELMESDLHQVIKAN

DDLTKEHYQFFLYQLLRALKYIHTANVYHRDLKPKNILANSNCKLKICDFGLARVAFNDT

PTTIFWTDYVATRWYRAPELCGSFFSKYTPAIDVWSIGCIFAEVLTGKPLFPGKNVVHQL

DLMTDLLGTPSMDTISRVRNEKARRYLSSMRKKEPIPFSQKFPNADPLALDLLQRLLAFD

PKDRPTAGEALSHPYFKGLAKVEREPSCQPITKMEFEFERRRATKEDIRELIFREILEYH

PQLLKDYINGTERTTFLYPSAVDQFRKQFAHLEENSESGPVVPMDRKHTSLPRSTIVHSA

PIHAKEQPRIGPSRDRPLTDESYKNPRDSEKYSGNVPRSSHHSQAPQRVPTARPGRVVGP

VLPYENVGTSHPYDPRRVAMNSGYPPQQQIPQTYGYYQTTGKSACSEPSQAERYTLHQQA

YACANSSTAPDVALDMRAPPFHQSAGPKSGSSDRLTAETNLYTRSLNGIVATTSGVAASA

HRKVSVVPYGMSQMY*

>Bd29-1MPK20-2 Brdisv1Bd29-11013150m.p

MQQPQQQGQRNKSTDVDFFSEYGDANRYRIQEVIGKGSYGVVCSAMDMQTRQKVAIKKIH

NIFDHVSDAARILREIKLLRLLRHPDVVEIKHIMLPPSRKDFKDIYVVFELMESDLHQVI

KANDDLTKEHYQFFLYQLLRALKYIHTASVYHRDLKPKNILANSNCKLKICDFGLARVAF

NDTPTTVFWTDYVATRWYRAPELCGSFFTKYTPAIDIWSIGCIFAEVLTGKPLFPGKNVV

HQLDLMTDLLGTPSMDTISRVRNEKARRYLSSMRKKDPVPFSKKFPNADPLGLKLLEKLL

AFDPKDRPTAEEALTDPYFKSLSKPDREPSCQPIRKVEFDFEHRRMSKDDIRELIFQEIL

EYHPQLLKNYIDGTEKTTFLYPSAVDQFKKQFSHLEESDGSGPVVPTERKHASLPRSTTV

HSTPIPAKEQPLVASSRGRPIANEPCKPWAPGNVPGASQTAHVAQAGRAVGVGSVPPYES

GSGKYPYDATSRPAVSSGYPPQQKIPQTYGYHHHHQTPGAGQSSQAMGGYACGYTKGTTP

PPAAAQDMRASPYHHRSAGTKNDPLNRLAAESDIYTRSLNGIVAAAASAGTGAHRKVGAV

PFGMSGMY*

>Bd29-1MPK20-3 Brdisv1Bd29-11005433m.p

MQHGDLHKKSAAEMDFFTAYDDANRYKILEVIGKGSYGLVCSANDLQTGEKVAIKKIHNI

FEHISDAARILREIKLLRLLRHPDVVEIKHILLPPSKKDFKDIYVVFELMESDLHQVIKA

NDDLTREHYQFFLYQMLRALKYMHTANVYHRDLKPKNVLANANCKLKICDFGLARVAFSD

APTTVFWTDYVATRWYRAPELCGSFYSK

>Bd29-1MPK20-4 Brdisv1Bd29-11016979m.p

MQTTEQQRKKGSSEMDFFSEYGDANRYKIQEVIGKGSYGVVCSAIDQHTGDKVAIKKIHN

IFEHLSDAARILREIKLLRLLRHPDIVEIRHIMLPPSRRDFKDIYVVFELMDTDLHQVIK

ANDDLTKEHHQFFLYQMLRALKYIHTANVYHRDLKPKNILANANCKLKICDFGLARVAFN

DTPTTVFWTDYVATRWYRAPELCGSFFTKYSRAIDIWSIGCIFAEILTGKPLFPGKNVVH

QLDLMTDLLGTPSLDTVSRIRNEKARRYLSSMRKKQSVSFSERFPKADPAALKLLQRLLA

FDPKDRPTAEEALADPYFKGLGKVEREPSCQPITKMEFEFERKNVTKADIKELIFREILE

YHPQLLKDYMNGTEKTNFLYPSAVDNFRRQFANLEENGGKGGAVVPPDRKHVSLPRNTTV

HSTPIPPKDQKYSQVPQRIPTGRPGRVVGPVIPFENSSTMDPYSQRRVARNPVLPAAATN

LSAYAYRNSDNSERELQQELEKDRMQYHPMQRFMDAKMVSPDLRSTSYYMPKGVPKADVA

ERSALQSNMMQGIAPFNGIATVGGAFNKVSAVPYGVSRMY*

>Bd29-1MPK20-5 Brdisv1Bd29-11013304m.p

MFSDIFSREGVAFSRVALRQELLSDQWFTTAGGKGQCYLVPPGTTVFLVNSEEMGFFSEY

VDASRYKILEIIGKGSYGVVCSAIDQETGDKVAIKKIQNIFEHLSDAARILREIKLLRLL

RHPDIVQIKHIMLPPSRRDFRDIYVVFELMDTDLHQVIKANDDLTKEHYQFFLYQMLRAL

KYIHTANVYHRDLKPKNILANANCKLKICDFGLARVAFNDTPTTVFWTDYVATRWYRAPE

LCGSFFTKYSPAIDTWSIGCIFAEILTGKPLFPGKNVVHQLDLMTDLLGIPSTETISRIR

NDKARKYLSSMRRKQPIPFSEKFPNADPSALKLLERLLAFDPKDRPTAEEALAHPYFKRL

ARVEREPSCQQPISKTEFEFERRKFTKEDIKELIFREILEYHPKLLKDYMNGSEKTSFLY

PSAVDNFRRQFANLEIDGGRSGAADRKHFSLPRTTTVHSAPILPTNGPTSQVPQRIPTAR

PGRVVSSAMQTDNPSVSDRHNGRRVARDPAVPPAAAYHLKSDYSDRQHQQEFEKDRVRPD

RQRQEELEKDRMQYRPGHHSMDAKVAPEISPYMRSSPYYIPPFNGIAAVASGYSKVAAVT

RMY*

>Bd29-1MPK21-1 Brdisv1Bd29-11013190m.p

MGGLIRWLRHHRSRRVSSSSSSHLPSNTTSSSSTSDLRAHSLPQHQGDHHGEVVVEWEDA

AEGPDSDPEEYIVVVLGDDEQGVVAARAPVRTKPPRVMDPGKKTSESEFFTEYGEANRYK

VSEVIGKGSYGVVAAAVDTQTGERCAIKKINDVFDHVSDATRILREIKLLRLLRHPDIVE

IKHIMLPPSRREFRDIYVVFELMESDLHQVIKANDDLTPEHHQFFLYQLLRGMKYIHTAS

VFHRDLKPKNILANADCKLKICDFGLARVSFNDGAPSAIFWTDYVATRWYRAPELCGSFF

SKYTPAIDIWSVGCIFAEMLTGKPLFPGKNVVHQLDLMTDLLGTPSAESLSKIRNEKARR

YLSNMRKKPKVPLTKKFPGIDPMALHLLERLLAFDPKDRPSADEALTDPYFTGLANSERE

PITQPISKLEFEFEKRKLAKDDVRELIYREILEYHPHMLQEYLRGGGDQMSFMFPSGVDR

FKRQFAHLEEGGAKGEKSSPQLRQNASLPRERVIGNKHGDGDYNMKLNVGEKPELASVSD

GISKPLMSARSLLKSETMSASKCIGEIKNKDEDSLSECVEGTDDDVSQKIAQLKT*

>Bd29-1MPK21-2 Brdisv1Bd29-11016866m.p

GSDEAEFFTEYGEANRYEVGEVVGKGSYGVVAAAVDTHTGERVAIKKINDVFEHVSDATR

ILREIKLLRLLRHPDIVEIKHIMLPPSRREFRDIYIIFELMESDLHQVIKANDDLTPEHH

QFFFYQLLRGMKYIHAANVFHRDLKPRNILANADCKLKICDFGLARVSFNDTPSAIFWTD

YVATRWYRAPELCGSFFSKYTPAIDIWSIGCIFAEMLSGRPLFPGKNVVHQLDLMTDLLG

TPSAESLSRIRNEKARRYLGNMRKKHPVPFSQKFPGVDPMALDLLERLLAFDPKDRPTAA

EALADPYFTGLANSDREPTTQPISKLEFEFERRKLARDDVRELIYREILEYHPQMLHEYH

HGGDQANFVYPSGVDRFKRQFVHLEEGVTKGEKTSPQLRQHASLPRERIIGIGDELGRPN

ADYCIKLHVGEEPGHTSVTDGLSKPLLNARNFLKSESISASQCVVIKEKREKDVSAVTSN

IF*

>ABR9MPK3 Brdisv1ABR9_r1011010m.p

MDGAPVAEFRPTMTHGGRFLLYNIFGNQFEITAKYQPPIMPIGRGAYGIVCSVMNFETRE

MVAIKKIANAFDNNMDAKRTLREIKLLRHLDHENIVGLRDVIPPAIPQSFNDVYIATELM

DTDLHHIIRSNQELSEEHCQYFLYQLLRGLKYIHSANVIHRDLKPSNLLLNANCDLKICD

FGLARPSSESDMMTEYVVTRWYRAPELLLNSTDYSAAIDVWSVGCIFMELINRAPLFPGR

DHMHQMRLITEVIGTPTDDDLGFIRNEDARRYMRHLPQFPRRPFPAQFPRVQPAALDLIE

RMLAFNPLQRITVEEALEHPYLERLHDIADEPICTDPFSFDFEQHPLTEDQMKQLIFNEA

LELNPNFRY*

>ABR9MPK4 Brdisv1ABR9_r1026988m.p

MDTSGGGGGAGGAAQIQGMATHGGRYVLYNVYGNLFEVASKYAPPIRPIGRGAYGIVCAA

VSSDTGEEVAIKKIGNAFDNHIDAKRTLREIKLLRHMDHENIIAIKDIIRPPRRDDFKDV

YIVTELMDTDLHQIIRSNQPLTDDHCQYFLYQLLRGLKYVHSANVLHRDLKPSNLFLNAN

CDLKIADFGLARTTSETDLMTEYVVTRWYRAPELLLNCSQYTAAIDVWSVGCILGEIITR

QPLFPGRDYIQQLKLITELIGSPDDSSLGFLRSDNARRYMKQLPQYPRQDFRLRFRNMSD

GAVDLLERMLVFDPSRRITVDEALHHPYLASLHDINEEPTCPAPFSFDFEQPSFTEEHMK

ELIWRETLAFNPDPPY*

>ABR9MPK6 Brdisv1ABR9_r1007931m.p

MDGGAQPPDAEMADAGAAVPGAGAGAAGTMENIQATLSHGGRFIQYNIFGNVFEVTVKYK

PPILPIGKGAYGIVCSALNSETGEQVAIKKIANAFDNKIDAKRTLREIKLLRHMDHENIV

AIRDIIPPAQRNSFNDVYIAYELMDTDLHQIIRSNQALSEEHCQYFLYQILRGLKYIHSA

NVLHRDLKPSNLLLNANCDLKICDFGLARTTSETDFMTEYVVTRWYRAPELLLNSSEYTA

AIDVWSVGCIFMELMDRKPLFPGRDHVHQLRLLMELIGTPNEADLDFVNENARRYIRQLP

RHARQSFSEKFPHVHPSAIDLVEKMLTFDPRQRITVEGALAHPYLASLHDISDEPVCTMP

FSFDFEQHALSEEQMKDLIHQEALAFNPDYQ*

>ABR9MPK7-1 Brdisv1ABR9_r1035091m.p

MLKMAMMVDPPNGTGNHGKHYYTMWQTMFEINTKYMPIKPIGRGAYGIVCSSTNQENNEK

VAIKKINNVFDNRVDALRTLRELKLLRHLRHENVIALKDIMMPVHRRSFKDVYLVSELMD

TDLHQIIKSSQPLSNDHCQYFLFQLLRGLKYLHSAGILHRDLKPGNLLVNANCDLKICDF

GLARTNNTKGQFMTEYVVTRWYRAPELLLCCDNYGTSIDVWSVGCIFAELLGRKPIFPRT

QCLNQLKLIVNVLGTMSENDLEFIDNPKARKYIKSLPYTPGTPLTSMYPQAHPLAIDLLQ

KMLVFDPSKRISVTEALEHPYMSPLYDPSANPPAQVPIDLDIDENLGVEMIREMLWQEML

QYHPEAARMVNM*

>ABR9MPK11 Brdisv1ABR9_r1025789m.p

MRMEGGGAGPAAAAAGGGAHGLGEAQIKGTLTHGGRYVQYNVYGNLFEVSAKYVPPIRPV

GRGACGIICAAINVQTREEVAIKKIGNAFDNQIDAKRTLREVKLLRHMNHENVISIKDII

RPPRRENFNDVYIVYELMDTDLHHLLRSNQPLTDDHCQYFLYQVLRGLKYVHSANVLHRD

LRPSNLLLNAKCDLKIGDFGLARTTTETDFMMEYVVTRWYRAPELLLNCSEYTGAIDMWS

VGCILGEIATREPLFPGKDYVHQLRLITELLGSPDDTSLGFLRSDNARRYVRSLPQYPKQ

QFRSRFPNMSSGAMDLLERMLVFDPNKRITVDEALCHPYLASLHEINDEPVCPAPFSFDF

EQPSFTEEDIKELIWRESVKFNPEPIH*

>ABR9MPK14 Brdisv1ABR9_r1024459m.p

MAMLVDPPNGMGNQGKHYYSMWQTLFEIDTKYVPIKPIGRGAYGIVCSSINRETNEKVAI

KKIHNVFDNRVDALRTLRELKLLRHLRHENVISLKDIMMPVQRRSFKDVYLVYELMDTDL

HQIIKSPQGLSNDHCQYFLFQLLRGLKYLHSAEILHRDLKPGNLLVNANCDLKICDFGLA

RTNSSKGQFMTEYVVTRWYRAPELLLCCDNYGTSIDVWSVGCIFAELLGRKPIFPGTECL

NQLKLIVNVLGTMSESDLEFIDNPKARRYIKTLPYTPGVPLASMYPHAHPLAIDLLQKML

IFDPTKRISVTEALEHPYMSPLYDPSANPPAQVPIDLDLDENISADMIREMMWQEMLHYH

PEAATAISM*

>ABR9MPK16 Brdisv1ABR9_r1016603m.p

MDFFTEYGEGNRYKIEEVIGKGSYGVVCSALDTHTGDKVAIKKINDIFEHVSDATRILRE

IKLLRLLRHPDIVEIKHILLPPSRREFKDIYVVFELMESDLHQVIKANDDLTPEHYQFFL

YQLLRGLKYIHTANVFHRDLKPKNILANADCKLKICDFGLARVAISDTPTAIFWTDYIAT

RWYRAPELCGSFFSKYTPAIDIWSIGCIFAELLTGKPLFPGKNVVHQLDIITDLLGTPSA

ETIARIRNEKARRYLSSMRRKKTVPFTQKFPNADPLALNLLERMLAFDPKDRPSAEEALA

DPYFKNIASVDREPSAQPITKLEFEFERRRITKDDIRELIYREILEYHPNMLREFLEGAE

PTGFMYPSAVDHFKKQFTFLEEHYAKGSTAAPPERQHNSLPRPSVIYSDNRPQNAANITD

DLSRCAIRDNTQKAQKDSASVGANRIPHGAAAAAARPGKVVGSVLRYGNCSTSGTEQYEQ

RRVVRSPGIAPNGVTSGSSYPRRNNTCKSETGEAERIDTNQGGPPKPYVANKLPATVDGR

NGHW*

>ABR9MPK17 Brdisv1ABR9_r1005736m.p

MVGGGGIVDGFRRLFHRRTPSGSGPSSNQSSAGEESSDIDAVVEDLDLVGLRAIRVPKRK

MPLPVESHKKNILEKEFFTEYGEASQYQIQEVIGKGSYGVVAAAVDTRTGERVAIKKIND

VFEHVSDATRILREVKLLRLLRHPDVVEIKHIMLPPSRREFQDIYVVFELMESDLHQVIR

ANDDLTPEHYQFFLYQLLRALKYIHAANVFHRDLKPKNILANSDCKLKICDFGLARVSFN

DAPTAIFWTDYVATRWYRAPELCGSFFSKYTPAIDIWSIGCIFAELLTGRPLFPGKNVVH

QLDIITDLLGTPSSETLSRIRNEKARRYLSCMRKKHPVPLTQKFPNVDPLALRLLGRLLA

FDPKDRPSAEEALADPYFAVLANVEREPSRNPISKLEFEFERRKVTKDDVRELIYREILE

YHPQMLQEYMQGGEQLSFLYPSGVDRFKRQFAHLEENYSKGERGSPLQRKHASLPRQRVG

ASNDNNDQHASDQERGADSIAPATGNPPGSQDAGQQHVSGGQNGVGSTNISPRSYQKSAS

ISASKCVVVNANKNPEYDDDISEEMEGAVDGLSEKVSRMHP*

>ABR9MPK20-1 Brdisv1ABR9_r1017513m.p

MQQDQRKKSSAEAEFFTEYGDASRYKIQEIVGKGSYGVVCSAIDVHTGEKVAIKKIHDIF

EHISDAARILREIKLLRLLRHPDIVEIKHIMLPPSRRDFKDIYVVFELMESDLHQVIKAN

DDLTKEHYQFFLYQLLRALKYIHTANVYHRDLKPKNILANSNCKLKICDFGLARVAFNDT

PTTIFWTDYVATRWYRAPELCGSFFSKYTPAIDVWSIGCIFAEVLTGKPLFPGKNVVHQL

DLMTDLLGTPSMDTISRVRNEKARRYLSSMRKKEPIPFSQKFPNADPLALDLLQRLLAFD

PKDRPTAGEALSHPYFKGLAKVEREPSCQPITKMEFEFERRRATKEDIRELIFREILEYH

PQLLKDYINGTERTTFLYPSAVDQFRKQFAHLEENSESGPVVPMDRKHTSLPRSTIVHSA

PIHAKEQPRIGPSRDRPLTDESYKNPRDSEKYSGNVPRSSHHSQAPQRVPTARPGRVVGP

VLPYENVGTSHPYDPRRVAMNSGYPPQQQIPQTYGYYQTTGKSACSEPSQAERYTLHQQA

YACANSSTAPDVALDMRAPPFHQSAGPKSGSSDRLTAETNLYTRSLNGIVATTSGVAASA

HRKVSVVPYGMSQMY*

>ABR9MPK20-2 Brdisv1ABR9_r1014888m.p

MQQPQQQGQRNKSTDVDFFSEYGDANRYRIQEVIGKGSYGVVCSAMDMQTRQKVAIKKIH

NIFDHVSDAARILREIKLLRLLRHPDVVEIKHIMLPPSRKDFKDIYVVFELMESDLHQVI

KANDDLTKEHYQFFLYQLLRALKYIHTASVYHRDLKPKNILANSNCKLKICDFGLARVAF

NDTPTTVFWTDYVATRWYRAPELCGSFFTKYTPAIDIWSIGCIFAEVLTGKPLFPGKNVV

HQLDLMTDLLGTPSMDTISRVRNEKARRYLSSMKKKDPVPFSKKFPNADPLGLKLLEKLL

AFDPKDRPTAEEALTDPYFKSLSKPDREPSCQPIRKVEFDFEHRRMSKDDIRELIFQEIL

EYHPQLLKNYIDGTEKTTFLYPSAVDQFKKQFSHLEESDGSGPVVPTERKHASLPRSTTV

HSTPIPAKEQPLVASSRGRPIANEPCKPWAPGNVPGASQTAHVAQAGRAVGVGSVPPYES

GSGKYPYDATSRPAVSSGYPPQQKIPQTYGYHHHHQTPGAGQSSQAMGGYACGYTKGTTP

PPAAAQDMRASPYHHRSAGTKNDPLNRLAAESDIYTRSLNGIVAAAASAGTGAHRKVGAV

PFGMSGMY*

>ABR9MPK20-3 Brdisv1ABR9_r1006567m.p

MQHGDLHKKSAAEMDFFTAYDDANRYKILEVIGKGSYGLVCSANDLQTGEKVAIKKIHNI

FEHISDAARILREIKLLRLLRHPDVVEIKHILLPPSKKDFKDIYVVFELMESDLHQVIKA

NDDLTREHYQFFLYQMLRALKYMHTANVYHRDLKPKNVLANANCKLKICDFGLARVAFSD

APTTVFWTDYVATRWYRAPELCGSFYSKYTPAIDIWSIGCIFAEVLIGKPLFPGKNVVHQ

LDLITDILGTPSLDAISRVRNDKARKYLTCMRKKQPASFSQKFPKADPLALQLLRRLLAF

DPKDRPSAEEALADPYFNGLAKVEREPSCQPIPKIEFEFEGRRVTKEDIKELIFEEILEY

HPQLLKEHIIGKERPNFVHLSAVDQFKKHFTQLEENDNETGAAVSLQRKHSSLPRQAFNH

R*

>ABR9MPK20-4 Brdisv1ABR9_r1017678m.p

MQTTEQQRKKGSSEMDFFSEYGDANRYKIQEVIGKGSYGVVCSAIDQHTGDKVAIKKIHN

IFEHLSDAARILREIKLLRLLRHPDIVEIRHIMLPPSRRDFKDIYVVFELMDTDLHQVIK

ANDDLTKEHHQFFLYQMLRALKYIHTANVYHRDLKPKNILANANCKLKICDFGLARVAFN

DTPTTVFWTDYVATRWYRAPELCGSFFTKYSRAIDIWSIGCIFAEILTGKPLFPGKNVVH

QLDLMTDLLGTPSLDTVSRIRNEKARRYLSSMRKKQPVSFSERFPKADPAALKLLQRLLA

FDPKDRPTAEEALADPYFKGLGKVEREPSCQPITKMEFEFERKNVTKADIKELIFREILE

YHPQLLKDYMNGTEKTNFLYPSAVDNFRRQFANLEENGGKGGAVVPPDRKHVSLPRNTTV

HSTPIPPKDQKYSQVPQRIPTGRPGRVVGPVIPFENSSTMDPYSQRRVARNPVLPAAATN

LSAYAYRNSDNSERELQQELEKDRMQYHPMQRFMDAKMVSPDLRSTSYYMPKGVPKADVA

ERSALQSNMMQGIAPFNGIATVGGAFNKVSAVPYGVSRMY*

>ABR9MPK20-5 Brdisv1ABR9_r1015005m.p

MGFFSEYVDASRYKILEIIGKGSYGVVCSAIDQETGDKVAIKKIQNIFEHLSDAARILRE

IKLLRLLRHPDIVQIKHIMLPPSRRDFRDIYVVFELMDTDLHQVIKANDDLTKEHYQFFL

YQMLRALKYIHTANVYHRDLKPKNILANANCKLKICDFGLARVAFNDTPTTVFWTDYVAT

RWYRAPELCGSFFTKYSPAIDTWSIGCIFAEILTGKPLFPGKNVVHQLDLMTDLLGTPST

ETISRIRNDKARKYLSSMRRKQPIPFSEKFPNADPSALKLLERLLAFDPKDRPTAEEALA

HPYFKRLARVEREPSCQQPISKTEFEFERRKFTKEDVKELIFREILEYHPKLLKDYMNGS

EKTSFLYPSAVDNFRRQFANLEIDGGRSGAADRKHFSLPRTTTVHSAPILPTNGPTSQVP

QRIPTARPGRVVSSAMQTDNPSVSDRHNGRRVARDPAVPPAAAYHLKSDYSDRQHQQEFE

KDRVRPDRQRQEELEKDRMQYRPGHHSMDAKVAPEISPYMRSSPYYIPPFNGIAAVASGY

SKVAAVTRMY*

>ABR9MPK21-1 Brdisv1ABR9_r1014923m.p

MGGLIRWLRHHRSRRVSSSSSSHLPSNTTSSSSTSDLRAHSLPQHQGDHHGEVVVEWEDA

AEGPDSDPEEYIVVVLGDDEQGVVAARAPVRTKPPRVMDPGKKTSESEFFTEYGEANRYK

VSEVIGKGSYGVVAAAVDTQTGERCAIKKINDVFDHVSDATRILREIKLLRLLRHPDIVE

IKHIMLPPSRREFRDIYVVFELMESDLHQVIKANDDLTPEHHQFFLYQLLRGMKYIHTAS

VFHRDLKPKNILANADCKLKICDFGLARVSFNDGAPSAIFWTDYVATRWYRAPELCGSFF

SKYTPAIDIWSVGCIFAEMLTGKPLFPGKNVVHQLDLMTDLLGTPSAESLSKIRNEKARR

YLSNMRKKPKVPLTKKFPGIDPMALHLLERLLAFDPKDRPSADEALTDPYFTGLANSERE

PITQPISKLEFEFEKRKLAKDDVRELIYREILEYHPHMLQEYLRGGGDQMSFMFPSGVDR

FKRQFAHLEEGGAKGEKSSPQLRQNASLPRERVIGNKHGDGDYNMKLNVGEKPELASVSD

GISKPLMSARSLLKSETMSASKCIGEIKNKDEDSLSECVEGTDDDVSQKIAQLKT*

>ABR9MPK21-2 Brdisv1ABR9_r1017558m.p

MPFPHGRHAPISLTAPRAPAMIGRPPPARASFPAPLPAAIRPTARTRAQTRRRSGIPVGR

ATTRGGSDEAEFFTEYGEANRYEVGEVVGKGSYGVVAAAVDTHTGERVAIKKINDVFEHV

SDATRILREIKLLRLLRHPDIVEIKHIMLPPSRREFRDIYIIFELMESDLHQVIKANDDL

TPEHHQFFFYQLLRGMKYIHAANVFHRDLKPRNILANADCKLKICDFGLARVSFNDTPSA

IFWTDYVATRWYRAPELCGSFFSKYTPAIDIWSIGCIFAEMLSGRPLFPGKNVIRNEKAR

RYLGNMRKKHPVPFSQKFPGVDPMALDLLERLLAFDPKDRPTAAEILEYHPQMLHEYHHG

GDQANFVYPRERIIGIGDELGRPNADYCIKLHEESMSEYMHEASDATPK*

>Bd1-1MPK3 Brdisv1Bd1-11009760m.p

MDGAPVAEFRPTMTHGGRVLLYNIFGNQFEITAKYQPPIMPIGRGAYGIVCSVMNFETRE

MVAIKKIANAFDNNMDAKRTLREIKLLRHLDHENIVGLRDVIPPAIPQSFNDVYIATELM

DTDLHHIIRSNQELSEEHCQYFLYQLLRGLKYIHSANVIHRDLKPSNLLLNANCDLKICD

FGLARPSSESDMMTEYVVTRWYRAPELLLNSTDYSAAIDVWSVGCIFMELINRAPLFPGR

DHMHQMRLITEVIGTPTDDDLGFIRNEDARRYMRPLPQFPRRPFPAPFPRVPPAALDLIE

RMLAFNPLQRITVEEALEHPYLERLHDIADEPICTDPFSFDFEQHPLTEDQMKQLIFNEA

LELNPNFRY*

>Bd1-1MPK4 Brdisv1Bd1-11024723m.p

MDTSGGGGGGGGGGGGAAQIQGMATHGGRYVLYNVYGNLFEVASKYAPPIRPIGRGAYGI

VCAAVSSDTGEEVAIKKIGNAFDNHIDAKRTLREIKLLRHMDHENIIAIKDIIRPPRRDD

FKDVYIVTELMDTDLHQIIRSNQPLTDDHCQYFLYQLLRGLKYVHSANVLHRDLKPSNLF

LNANCDLKIADFGLARTTSETDLMTEYVVTRWYRAPELLLNCSQYTAAIDVWSVGCILGE

IITRQPLFPGRDYIQQLKLITELIGSPDDSSLGFLRSDNARRYMKQLPQYPRQDFRLRFR

NMSDGAVDLLERMLVFDPSRRITVDEALHHPYLASLHDINEEPTCPAPFSFDFEQPSFTE

EHMKELIWRETLAFNPDPPY*

>Bd1-1MPK6 Brdisv1Bd1-11006994m.p

MDGGAQPPDAEMADAGAAVPGAGAGAAGTMENIQATLSHGGRFIQYNIFGNVFEVTVKYK

PPILPIGKGAYGIVCSALNSETGEQVAIKKIANAFDNKIDAKRTLREIKLLRHMDHENIV

AIRDIIPPAQRNSFNDVYIAYELMDTDLHQIIRSNQALSEEHCQYFLYQILRGLKYIHSA

NVLHRDLKPSNLLLNANCDLKICDFGLARTTSETDFMTEYVVTRWYRAPELLLNSSEYTA

AIDVWSVGCIFMELMDRKPLFPGRDHVHQLRLLMELIGTPNEADLDFVNENARRYIRQLP

RHARQSFSEKFPHVHPSAIDLVEKMLTFDPRQRITVEGALAHPYLASLHDISDEPVCTMP

FSFDFEQHALSEEQMKDLIHQEALAFNPDYQ*

>Bd1-1MPK11 Brdisv1Bd1-11023075m.p

MRMEGGGAGPAAAAAGGGAHGLGEAQIKGTLTHGGRYVQYNVYGNLFEVSAKYVPPIRPV

GRGACGIICAAINVQTREEVAIKKIGNAFDNQIDAKRTLREVKLLRHMNHENVISIKDII

RPPRRENFNDVYIVYELMDTDLHHLLRSNQPLTDDHCQYFLYQVLRGLKYVHSANVLHRD

LRPSNLLLNAKCDLKIGDFGLARTTTETDFMMEYVVTRWYRAPELLLNCSEYTGAIDMWS

VGCILGEIATREPLFPGKDYVHQLRLITELLGSPDDTSLGFLRSDNARRYVRSLPQYPKQ

QFRSRFPNMSSGAMDLLERMLVFDPNKRITVDEALCHPYLASLHEINDEPVCPAPFSFDF

EQPSFTEEDIKELIWRESVKFNPEPIH*

>Bd1-1MPK14 Brdisv1Bd1-11021222m.p

MAMLVDPPNGMGNQGKHYYSMWQTLFEIDTKYVPIKPIGRGAYGIVCSSINRETNEKVAI

KKIHNVFDNRVDALRTLRELKLLRHLRHENVISLKDIMMPVQRRSFKDVYLVYELMDTDL

HQIIKSPQGLSNDHCQYFLFQLLRGLKYLHSAEILHRDLKPGNLLVNANCDLKICDFGLA

RTNSSKGQFMTEYVVTRWYRAPELLLCCDNYGTSIDVWSVGCIFAELLGRKPIFPGTECL

NQLKLIVNVLGTMSESDLEFIDNPKARRYIKTLPYTPGVPLASMYPHAHPLAIDLLQKML

IFDPTKRISVTEALEHPYMSPLYDPSANPPAQVPIDLDLDENISADMIREMMWQEMLHYH

PEAATAISM*

>Bd1-1MPK16 Brdisv1Bd1-11016431m.p

MDFFTEYGEGNRYKIEEVIGKGSYGVVCSALDTHTGDKVAIKKINDIFEHVSDATRILRE

IKLLRLLRHPDIVEIKHILLPPSRREFKDIYVVFELMESDLHQVIKANDDLTPEHYQFFL

YQLLRGLKYIHTANVFHRDLKPKNILANADCKLKICDFGLARVAISDTPTAIFWTDYIAT

RWYRAPELCGSFFSKYTPAIDIWSIGCIFAELLTGKPLFPGKNVVHQLDIITDLLGTPSA

ETIARIRNEKARRYLSSMRRKRTVPFTQKFPNADPLALNLLERMLAFDPKDRPSAEEALA

DPYFKNIASVDREPSAQPITKLEFEFERRRITKDDIRELIYREILEYHPNMLREFLEGAE

PTGFMYPSAVDHFKKQFTFLEEHYAKGSTAAPPERQHNSLPRPSVIYSDNRPQNAANITD

DLSRCAIRDNTQKAQKDSASVGANRIPHGAAAAAARPGKVVGSVLRYGNCSTSGTEQYEQ

RRVVRSPGIAPNGVTSGSSYPRRNNTCKSETGEAERIDTNQGGPPKPYVANKLPATVDGR

NGHW*

>Bd1-1MPK17 Brdisv1Bd1-11005322m.p

MVGGGGIVDGFRRLFPRRTPSGSGPSSNQSSAGEESSDIDAVVEDLDLVGLRAIRVPKRK

MPLPVESHKKNILEKEFFTEYGEASQYQIQEVIGKGSYGVVAAAVDTRTGERVAIKKIND

VFEHVSDATRILREVKLLRLLRHPDVVEIKHIMLPPSRREFQDIYVVFELMESDLHQVIR

ANDDLTPEHYQFFLYQLLRALKYIHAANVFHRDLKPKNILANSDCKLKICDFGLARVSFN

DAPTAIFWTDYVATRWYRAPELCGSFFSKYTPAIDIWSIGCIFAELLTGRPLFPGKNVVH

QLDIITDLLGTPSSETLSRIRNEKARRYLSCMRKKHPVPLTQKFPNVDPLALRLLGRLLA

FDPKDRPSAEEALADPYFAVLANVEREPSRNPISKLEFEFERRKVTKDDVRELIYREILE

YHPQMLQEYMQGGEQLSFLYPSGVDRFKRQFAHLEENYSKGERGSPLQRKHASLPRQRVG

ASNDNNDQHASDQERGADSIAPATGNPPGSQDAGQQHVSGGQNGVGSTNISPRSYQKSAS

ISASKCVVVNANKNPEYDDDISEEMEGAVDGLSEKVSRMHP*

>Bd1-1MPK20-1 Brdisv1Bd1-11017639m.p

MQQDQRKKSSAEAEFFTEYGDASRYKIQEIVGKGSYGVVCSAIDVHTGEKVAIKKIHDIF

EHISDAARILREIKLLRLLRHPDIVEIKHIMLPPSRRDFKDIYVVFELMESDLHQVIKAN

DDLTKEHYQFFLYQLLRALKYIHTANVYHRDLKPKNILANSNCKLKICDFGLARVAFNDT

PTTIFWTDYVATRWYRAPELCGSFFSKYTPAIDVWSIGCIFAEGLTGKPLFPGKNVVHQL

DLMTDLLGTPSMDTISRVRNEKARRYLSSMRKKEPIPFSQKFPNADPLALDLLQRLLAFD

PKDRPTAGEALSHPYFKGLAKVEREPSCQPITKMEFEFERRRATKEDIRELIFREILEYH

PQLLKDYINGTERTTFLYPSAVDQFRKQFAHLEENSESGPVVPMDRKHTSLPRSTIVHSA

PIHAKEQPRIGPSRDRPLTDESYKNPRDSEKYSGNVPRSSHHSQAPQRVPTARPGRVVGP

VLPYENVGTSHPYDPRRVAMNSGYPPQQQIPQTYGYYQTTGKSACSEPSQAERYTLHQQA

YACANSSTAPDVALDMRAPPFHQSAGPKSGSSDRLTAETNLYTRSLNGIVATTSGVAASA

HRKVSVVPYGMSQMY*

>Bd1-1MPK20-2 Brdisv1Bd1-11014003m.p

MQQPQQQGQRNKSTDVDFFSEYGDANRYRIQEVIGKGSYGVVCSAMDMQTRQKVAIKKIH

NIFDHVSDAARILREIKLLRLLRHPDVVEIKHIMLPPSRKDFKDIYVVFELMESDLHQVI

KANDDLTKEHYQFFLYQLLRALKYIHTASVYHRDLKPKNILANSNCKLKICDFGLARVAF

NDTPTTVFWTDYVATRWYRAPELCGSFFTKYTPAIDIWSIGCIFAEVLTGKPLFPGKNVV

HQLDLMTDLLGTPSMDTISRVRNEKARRYLSSMRKKDPVPFSKKFPNADPLGLKLLEKLL

AFDPKDRPTAEEALTDPYFKSLSKPDREPSCQPIRKVEFDFEHRRMSKDDIRELIFQEIL

EYHPQLLKNYIDGTEKTTFLYPSAVDQFKKQFSHLEESDGSGPVVPTERKHASLPRSTTV

HSTPIPAKEQPLVASSRGRPIANEPCKPWAPGNVPGASQTAHVAQAGRAVGVGSVPPYES

GSGKYPYDATSRPAVSSGYPPQQKIPQTYGYHHHHQTPGAGQSSQAMGGYACGYTKGTTP

PPAAAQDMRASPYHHRSAGTKNDPLNRLAAESDIYTRSLNGIVAAAASAGTGAHRKVGAV

PFGMSGMY*

>Bd1-1MPK20-3 Brdisv1Bd1-11005908m.p

MQHGDLHKKSAAEMDFFTAYDDANRYKILEVIGKGSYGLVCSANDLQTGEKVAIKKIHNI

FEHISDAARILREIKLLRLLRHPDVVEIKHILLPPSKKDFKDIYVVFELMESDLHQVIKA

NDDLTREHYQFFLYQMLRALKYMHTANVYHRDLKPKNVLANANCKLKICDFGLARVAFSD

APTTVFWTDYVATRWYRAPELCGSFYSKYTPAIDIWSIGCIFAEVLIGKPLFPGKNVVHQ

LDLITDILGTPSLDAISRVRNDKARKYLTCMRKKQPASFSQKFPKADPLALQLLRRLLAF

DPKDRPSAEEALADPYFNGLAKVEREPSCQPIPKIEFEFEGRRVTKEDIKELIFEEILEY

HPQLLKEHIIGKERPNFVHLSAVDQFKKHFTQLEENDNETGAAVSLQRKHSSLPRQAFNH

R*

>Bd1-1MPK20-4 Brdisv1Bd1-11017837m.p

MQTTEQQRKKGSSEMDFFSEYGDANRYKIQEVIGKGSYGVVCSAIDQHTGDKVAIKKIHN

IFEHLSDAARILREIKLLRLLRHPDIVEIRHIMLPPSRRDFKDIYVVFELMDTDLHQVIK

ANDDLTKEHHQFFLYQMLRALKYIHTANVYHRDLKPKNILANANCKLKICDFGLARVAFN

DTPTTVFWTDYVATRWYRAPELCGSFFTKYSRAIDIWSIGCIFAEILTGKPLFPGKNVVH

QLDLMTDLLGTPSLDTVSRIRNEKARRYLSSMRKKQPVSFSERFPKADPAALKLLQRLLA

FDPKDRPTAEEALADPYFKGLGKVERDPPSQPITKMEFEFERKNVTKADIKELIFREILE

YHPQLLKDYMNGTEKTNFLYPSAVDNFRRQFANLEENGGKGGAVVPPDRKHVSLPRNTTV

HSTPIPPKDQKYSQVPQRIPTGRPGRVVGPVIPFENSSTMDPYSQRRVARNPVLPAAATN

LSAYAYRNSDNSERELQQELEKDRMQYHPMQRFMDAKMVSPDLRSTSYYMPKGVPKADVA

ERSALQSNMMQGIAPFNGIATVGGAFNKVSAVPYGVSRMY*

>Bd1-1MPK20-5 Brdisv1Bd1-11014155m.p

MFSDIFSREGVAFSRVALRQELLSDQWFTTAGGKGQCYLVPPGTTVFLVNSEEMGFFSEY

VDASRYKILEIIGKGSYGVVCSAIDQETGDKVAIKKIQNIFEHLSDAARILREIKLLRLL

RHPDIVQIKHIMLPPSRRDFRDIYVVFELMDTDLHQVIKANDDLTKEHYQFFLYQMLRAL

KYIHTANVYHRDLKPKNILANANCKLKICDFGLARVAFNDTPTTVFWTDYVATRWYRAPE

LCGSFFTKYSPAIDTWSIGCIFAEILTGKPLFPGKNVVHQLDLMTDLLGTPSTETISRIR

NDKARKYLSSMRRKQPIPFSEKFPNADPSALKLLERLLAFDPKDRPTAEEALAHPYFKRL

ARGEREPSCQQPISKRSLSLSEENLPKKT*

>Bd1-1MPK21-1 Brdisv1Bd1-11014051m.p

MGGLIRWLRPPRSRRVSSSSSSHLPSNTTSSSSTSDLRAHSLPQHQGDHHGEVVVEWEDA

AEGPDSDPEEYIVVVLGDDEQGVVAARAPVRTKPPRVMDPGKKTSESEFFTEYGEANRYK

VSEVIGKGSYGVVAAAVDTQTGERCAIKKINDVFDHVSDATRILREIKLLRLLRHPDIVE

IKHIMLPPSRREFRDIYVVFELMESDLHQVIKANDDLTPEHHQFFLYQLLRGMKYIHTAS

VFHRDLKPKNILANADCKLKICDFGLARVSFNDGAPSAIFWTDYVATRWYRAPELCGSFF

SKYTPAIDIWSVGCIFAEMLTGKPLFPGKNVVHQLDLMTDLLGTPSAESLSKIRNEKARR

YLSNMRKKPKVPLTKKFPGIDPMALHLLERLLAFDPKDRPSADEALTDPYFTGLANSERE

PITQPISKLEFEFEKRKLAKDDVRELIYREILEYHPHMLQEYLRGGGDQMSFMFPSGVDR

FKRQFAHLEEGGAKGEKSSPQLRQNASLPRERVIGNKHGDGDYNMKLNVGEKPELASVSD

GISKPLMSARSLLKSETMSASKCIGEIKNKDEDSLSECVEGTDDDVSQKIAQLKT*

>Bd1-1MPK21-2 Brdisv1Bd1-11017707m.p

GSDEAEFFTEYGEANRYEVGEVVGKGSYGVVAAAVDTHTGERVAIKKINDVFEHVSDATR

ILREIKLLRLLRHPDIVEIKHIMLPPSRREFRDIYIIFELMESDLHQVIKANDDLTPEHH

QFFFYQLLRGMKYIHAANVFHRDLKPRNILANADCKLKICDFGLARVSFNDTPSAIFWTD

YVATRWYRAPELCGSFFSKYTPAIDIWSIGCIFAEMLSGRPLFPGKNVVHQLDLMTDLLG

TPSAESLSRIRNEKARRYLGNMRKKHPVPFSQKFPGVDPMALDLLERLLAFDPKDRPTAA

EALADPYFTGLANSDREPTTQPISKLEFEFERRKLARDDVRELIYREILEYHPQMLHEYH

HGGDQANFVYPSGVDRFKRQFVHLEEGVTKGEKTSPQLRQHASLPRERIIGIGDELGRPN

ADYCIKLHVGEEPGHTSVTDGLSKPLLNARNFLKSESISASQCVVIKEKREKDVSAVTSN

IF*

>BdMPK3 Bradi1g65810.1|feature=AA|genome=B. distachyon|accession=Bd21-1|len=370

MDGAPVAEFRPTMTHGGRFLLYNIFGNQFEITAKYQPPIMPIGRGAYGIVCSVMNFETREMVAIKKIANAFDNNMDAKRTLREIKLLRHLDHENIVGLRDVIPPAIPQSFNDVYIATELMDTDLHHIIRSNQELSEEHCQYFLYQLLRGLKYIHSANVIHRDLKPSNLLLNANCDLKICDFGLARPSSESDMMTEYVVTRWYRAPELLLNSTDYSAAIDVWSVGCIFMELINRAPLFPGRDHMHQMRLITEVIGTPTDDDLGFIRNEDARRYMRHLPQFPRRPFPAQFPRVQPAALDLIERMLAFNPLQRITVEEALEHPYLERLHDIADEPICTDPFSFDFEQHPLTEDQMKQLIFNEALELNPNFRY

>BdMPK4 Bradi3g32000.1|feature=AA|genome=B. distachyon|accession=Bd21-1|len=376

MDTSGGGGAGGAAQIQGMATHGGRYVLYNVYGNLFEVASKYAPPIRPIGRGAYGIVCAAVSSDTGEEVAIKKIGNAFDNHIDAKRTLREIKLLRHMDHENIIAIKDIIRPPRRDDFKDVYIVTELMDTDLHQIIRSNQPLTDDHCQYFLYQLLRGLKYVHSANVLHRDLKPSNLFLNANCDLKIADFGLARTTSETDLMTEYVVTRWYRAPELLLNCSQYTAAIDVWSVGCILGEIITRQPLFPGRDYIQQLKLITELIGSPDDSSLGFLRSDNARRYMKQLPQYPRQDFRLRFRNMSDGAVDLLERMLVFDPSRRITVDEALHHPYLASLHDINEEPTCPAPFSFDFEQPSFTEEHMKELIWRETLAFNPDPPY

>BdMPK6 Bradi1g49100.1|feature=AA|genome=B. distachyon|accession=Bd21-1|len=392

MDGGAQPPDAEMADAGAAVPGAGAGAAGTMENIQATLSHGGRFIQYNIFGNVFEVTVKYKPPILPIGKGAYGIVCSALNSETGEQVAIKKIANAFDNKIDAKRTLREIKLLRHMDHENIVAIRDIIPPAQRNSFNDVYIAYELMDTDLHQIIRSNQALSEEHCQYFLYQILRGLKYIHSANVLHRDLKPSNLLLNANCDLKICDFGLARTTSETDFMTEYVVTRWYRAPELLLNSSEYTAAIDVWSVGCIFMELMDRKPLFPGRDHVHQLRLLMELIGTPNEADLDFVNENARRYIRQLPRHARQSFSEKFPHVHPSAIDLVEKMLTFDPRQRITVEGALAHPYLASLHDISDEPVCTMPFSFDFEQHALSEEQMKDLIHQEALAFNPDYQ

>BdMPK7-1 Bradi1g34030.1|feature=AA|genome=B. distachyon|accession=Bd21-1|len=414

MPSRFPFLSPGCFIRLLSGSASSDLLLLLPRRGQSAPRARVLLKMAMMVDPPNGTGNHGKHYYTMWQTMFEIDTKYVPIKPIGRGAYGIVCSSTNQENNEKVAIKKINNVFDNRVDALRTLRELKLLRHLRHENVIALKDIMMPVHRRSFKDVYLVSELMDTDLHQIIKSSQPLSNDHCQYFLFQLLRGLKYLHSAGILHRDLKPGNLLVNANCDLKICDFGLARTNNTKGQFMTEYVVTRWYRAPELLLCCDNYGTSIDVWSVGCIFAELLGRKPIFPGTECLNQLKLIVNVLGTMSENDLEFIDNPKARKYIKSLPYTPGTPLTSMYPQAHPLAIDLLQKMLVFDPSKRISVTEALEHPYMSPLYDPSANPPAQVPIDLDIDENLGVEMIREMLWQEMLQYHPEAARMVNM

>BdMPK7-2 Bradi4g24914

MKLSPISASFGAMGSLPQKLDELLSTGHWALSGVVMDEIEELTADLHILCNLLLKLSNVH

DPPPTVRFWMKEVRELSYDVEDCADQFVIANDRAVMRRAKIHRATIRTIIITRLKISRLP

KRRKWRLWMTDKVAEFRTRVQDATQRYWRYKFDDCASNPGYASVGHEFATVYAQPGDLVG

IEGPMDELEQWLTDGEEQLKVISIVGLGGVGKTTLAQKLWATLKGQFLCRAFVRTAQKPD

MRGILRNILLQVRPHQLPNRGEMHHLIHDLREYLQDKRYFVIIDDLWAASVWDVVSRAFP

EGNCCSRIVTTTEIMEVALACCDYCPKHICKLETLSDDDSEKLLLERIVVSGNQIPQQLD

DVLPQIMRNCGGLPLAIIIVASLLVSQPEKLEQWGHTQNSFGSIFGTNPTMEGFIRQILS

ISFNSLPYYLKTCLLYLSSYPEGCLFLKDDLVKLWVAEGFIHAKEWEDMEDLAGRYFDEL

VNVGLIQVMDINYNYKLPSYSVHHMVLDFITCKSIEENFITVVDYSETTTPLTDKVRRLS

LHFGSATHATTPASARLSNVRSLFYFGLFNCMPSFMVFKLLRVLILNFWGDPGNTSFNLT

GICELVWLRYLQVSCNVTVKLPDQIESMGHLETLEIKAKVYAVPLDIVRLSSLSHLHLRG

GTIPNGIGCMRSLRTLKYFDLVNKSVDNLRGLGELANLRDLHLTYSSSLPSEHLKRNLIA

LASSLGKLCNLKSLTLASETAATVVPFDGSISMSSTPAFLERLELLPSICIFSRLPKWIF

QFQKLCILKVAVRELLANDIDSITGLPSLTVLSLCVHTAPGQRIVFNDGAFPVLKYFKFR

CGVLSMSFMAGAMPNLRRLKLGFNTHVGEKYGNMLAGIEHLLNLQDIAGKIGVATESDRK

AAESAIKGAISKHPQSPMLNVQWVDLVEEEYHASEKQHGRQEKGLSGENHAVLEKAKDTK

KHADSGVSQLPDLPSTVSSSRLKSENIAQASKHHVLESMLCDESAQPESLEVSVLEDITN

NFSDDQEISKNRFVVVYKGELQNGSVVVVKRFTIAIDYNLFLDAVDCLMSVKHNNIVRFL

GYCANSQENAAKEGSKSVSVVTQERLCCFEYLRNGNLQMHLTDESCGFDWQMWYQIIEGI

CQGVHYLHNKSITPLDLNPANIMFDDKMVPKIVHYAYTRFLGEATSPVLTNIGDSLAYMA

PEYFGFGEITSKSDIYSLGVIIMQIVTGENKKDFPSIQSVLESWRSRLESDGSQGHTSLE

TCYLQVNLCYLIGIRCIEHDPGKRPNTRDIIDSLDTLEIMECLVKHDVGNSSLEQMLVCS

KVEEPKALPAPPSVSCKLKMAMMVDPPNGTGNHGKHYYTMWQTMFEIDTKYMPIKPIGRG

AYGIVCSSTNQENNEKVAIKKINNVFDNRVDALRTLRELKLLRHLRHENVIALKDIMMPV

HRRSFKDVYLVFELMDTDLHQIIKSSQPLSNDHCQYFLFQLLRGLKYLHSAGILHRDLKP

GNLLVNANCDLKICDFGLARTNNTKGQFMTEYVVTCLYRAPELLLCCDNYGTSIDVWSVG

CIFAELLGRKPIFPRTQCLDQLKLIVNVLGTMSENDLEFIDNPKARKYIKSLPYTPGTPL

TSMYPQAHPLAIDLLQKMLVFDPSKRISVTEALEHPYMSPLYDPSANPPAQVPIDLDIDE

NLGVEMIREMLWQEMLQYHPEAARMVNM*

>BdMPK11 Bradi3g16560.1 I1I1F8|I1I1F8_BRADI MPK11 OS=Brachypodium distachyon GN=BRADI3G16560 PE=2 SV=1

MRMEGGGAGPAAAAAAGGAHGLGEAQIKGTLTHGGRYVQYNVYGNLFEVSAKYVPPIRPVGRGACGIICAAINVQTREEVAIKKIGNAFDNQIDAKRTLREVKLLRHMNHENVISIKDIIRPPRRENFNDVYIVYELMDTDLHHLLRSNQPLTDDHCQYFLYQVLRGLKYVHSANVLHRDLRPSNLLLNAKCDLKIGDFGLARTTTETDFMMEYVVTRWYRAPELLLNCSEYTGAIDMWSVGCILGEIATREPLFPGKDYVHQLRLITELLGSPDDTSLGFLRSDNARRYVRSLPQYPKQQFRSRFPNMSSGAMDLLERMLVFDPNKRITVDEALCHPYLASLHEINDEPVCPAPFSFDFEQPSFTEEDIKELIWRESVKFNPEPIH

>BdMPK14 Bradi3g03780.1|feature=AA|genome=B. distachyon|accession=Bd21-1|len=370

MAMLVDPPNGMGNQGKHYYSMWQTLFEIDTKYVPIKPIGRGAYGIVCSSINRETNEKVAIKKIHNVFDNRVDALRTLRELKLLRHLRHENVISLKDIMMPVQRRSFKDVYLVYELMDTDLHQIIKSPQGLSNDHCQYFLFQLLRGLKYLHSAEILHRDLKPGNLLVNANCDLKICDFGLARTNSSKGQFMTEYVVTRWYRAPELLLCCDNYGTSIDVWSVGCIFAELLGRKPIFPGTECLNQLKLIVNVLGTMSESDLEFIDNPKARRYIKTLPYTPGVPLASMYPHAHPLAIDLLQKMLIFDPTKRISVTEALEHPYMSPLYDPSANPPAQVPIDLDLDENISADMIREMMWQEMLHYHPEAATAISM

>BdMPK16 Bradi2g36470.1|feature=AA|genome=B. distachyon|accession=Bd21-1|len=545

MDFFTEYGEGNRYKIEEVIGKGSYGVVCSALDTHTGDKVAIKKINDIFEHVSDATRILREIKLLRLLRHPDIVEIKHILLPPSRREFKDIYVVFELMESDLHQVIKANDDLTPEHYQFFLYQLLRGLKYIHTANVFHRDLKPKNILANADCKLKICDFGLARVAISDTPTAIFWTDYIATRWYRAPELCGSFFSKYTPAIDIWSIGCIFAELLTGKPLFPGKNVVHQLDIITDLLGTPSAETIARIRNEKARRYLSSMRRKKTVPFTQKFPNADPLALNLLERMLAFDPKDRPSAEEALADPYFKNIASVDREPSAQPITKLEFEFERRRITKDDIRELIYREILEYHPNMLREFLEGAEPTGFMYPSAVDHFKKQFTFLEEHYAKGSTAAPPERQHNSLPRPSVIYSDNRPQNAANITDDLSRCAIRDNTQKAQKDSASVGANRIPHGAAAAAARPGKVVGSVLRYGNCSTSGTEQYEQRRVVRSPGIAPNGVTSGSSYPRRNNTCKSETGEAERIDTNQGGPPKPYVANKLPATVDGRNGHW

>BdMPK17 Bradi1g34700.1|feature=AA|genome=B. distachyon|accession=Bd21-1|len=582

MVGGGGIVDGFRRLFHRRTPSGSGPSSNQSSAGEESSDIDAVVEDLDLVGLRAIRVPKRKMPLPVESHKKNILEKEFFTEYGEASQYQIQEVIGKGSYGVVAAAVDTRTGERVAIKKINDVFEHVSDATRILREVKLLRLLRHPDVVEIKHIMLPPSRREFQDIYVVFELMESDLHQVIRANDDLTPEHYQFFLYQLLRALKYIHAANVFHRDLKPKNILANSDCKLKICDFGLARVSFNDAPTAIFWTDYVATRWYRAPELCGSFFSKYTPAIDIWSIGCIFAELLTGRPLFPGKNVVHQLDIITDLLGTPSSETLSRIRNEKARRYLSCMRKKHPVPLTQKFPNVDPLALRLLGRLLAFDPKDRPSAEEALADPYFAVLANVEREPSRNPISKLEFEFERRKVTKDDVRELIYREILEYHPQMLQEYMQGGEQLSFLYPSGVDRFKRQFAHLEENYSKGERGSPLQRKHASLPRQRVGASNDNNDQHASDQERGADSIAPATGNPPGSQDAGQQHVSGGQNGVGSTNISPRSYQKSASISASKCVVVNANKNPEYDDDISEEMEGAVDGLSEKVSRMHP

>BdMPK20-1 Bradi2g44350.1|feature=AA|genome=B. distachyon|accession=Bd21-1|len=616

MQQDQRKKSSAEAEFFTEYGDASRYKIQEIVGKGSYGVVCSAIDVHTGEKVAIKKIHDIFEHISDAARILREIKLLRLLRHPDIVEIKHIMLPPSRRDFKDIYVVFELMESDLHQVIKANDDLTKEHYQFFLYQLLRALKYIHTANVYHRDLKPKNILANSNCKLKICDFGLARVAFNDTPTTIFWTDYVATRWYRAPELCGSFFSKYTPAIDVWSIGCIFAEVLTGKPLFPGKNVVHQLDLMTDLLGTPSMDTISRVRNEKARRYLSSMRKKEPIPFSQKFPNADPLALDLLQRLLAFDPKDRPTAGEALSHPYFKGLAKVEREPSCQPITKMEFEFERRRATKEDIRELIFREILEYHPQLLKDYINGTERTTFLYPSAVDQFRKQFAHLEENSESGPVVPMDRKHTSLPRSTIVHSAPIHAKEQPRIGPSRDRPLTDESYKNPRDSEKYSGNVPRSSHHSQAPQRVPTARPGRVVGPVLPYENVGTSHPYDPRRVAMNSGYPPQQQIPQTYGYYQTTGKSACSEPSQAERYTLHQQAYACANSSTAPDVALDMRAPPFHQSAGPKSGSSDRLTAETNLYTRSLNGIVATTSGVAASAHRKVSVVPYGMSQMY

>BdMPK20-2 Bradi2g15317.1|feature=AA|genome=B. distachyon|accession=Bd21-1|len=598

MSTDVDFFSEYGDANRYRIQEVIGKGSYGVVCSAMDMQTRQKVAIKKIHNIFDHVSDAARILREIKLLRLLRHPDVVEIKHIMLPPSRKDFKDIYVVFELMESDLHQVIKANDDLTKEHYQFFLYQLLRALKYIHTASVYHRDLKPKNILANSNCKLKICDFGLARVAFNDTPTTVFWTDYVATRWYRAPELCGSFFTKYTPAIDIWSIGCIFAEVLTGKPLFPGKNVVHQLDLMTDLLGTPSMDTISRVRNEKARRYLSSMRKKDPVPFSKKFPNADPLGLKLLEKLLAFDPKDRPTAEEALTDPYFKSLSKPDREPSCQPIRKVEFDFEHRRMSKDDIRELIFQEILEYHPQLLKNYIDGTEKTTFLYPSAVDQFKKQFSHLEESDGSGPVVPTERKHASLPRSTTVHSTPIPAKEQPLVASSRGRPIANEPCKPWAPGNVPGASQTAHVAQAGRAVGVGSVPPYESGSGKYPYDATSRPAVSSGYPPQQKIPQTYGYHHHHQTPGAGQSSQAMGGYACGYTKGTTPPPAAAQDMRASPYHHRSAGTKNDPLNRLAAESDIYTRSLNGIVAAAASAGTGAHRKVGAVPFGMSGMY

>BdMPK20-3 Bradi1g41780.1|feature=AA|genome=B. distachyon|accession=Bd21-1|len=422

MQHGDLHKKSAAEMDFFTAYDDANRYKILEVIGKGSYGLVCSANDLQTGEKVAIKKIHNIFEHISDAARILREIKLLRLLRHPDVVEIKHILLPPSKKDFKDIYVVFELMESDLHQVIKANDDLTREHYQFFLYQMLRALKYMHTANVYHRDLKPKNVLANANCKLKICDFGLARVAFSDAPTTVFWTDYVATRWYRAPELCGSFYSKYTPAIDIWSIGCIFAEVLIGKPLFPGKNVVHQLDLITDILGTPSLDAISRVRNDKARKYLTCMRKKQPASFSQKFPKADPLALQLLRRLLAFDPKDRPSAEEALADPYFNGLAKVEREPSCQPIPKIEFEFEGRRVTKEDIKELIFEEILEYHPQLLKEHIIGKERPNFVHLSAVDQFKKHFTQLEENDNETGAAVSLQRKHSSLPRQAFNHR

>BdMPK20-4 Bradi2g45870.1|feature=AA|genome=B. distachyon|accession=Bd21-1|len=581

MQTTEQQRKKGSSEMDFFSEYGDANRYKIQEVIGKGSYGVVCSAIDQHTGDKVAIKKIHNIFEHLSDAARILREIKLLRLLRHPDIVEIRHIMLPPSRRDFKDIYVVFELMDTDLHQVIKANDDLTKEHHQFFLYQMLRALKYIHTANVYHRDLKPKNILANANCKLKICDFGLARVAFNDTPTTVFWTDYVATRWYRAPELCGSFFTKYSRAIDIWSIGCIFAEILTGKPLFPGKNVVHQLDLMTDLLGTPSLDTVSRIRNEKARRYLSSMRKKQSVSFSERFPKADPAALKLLQRLLAFDPKDRPTAEEALADPYFKGLGKVEREPSCQPITKMEFEFERKNVTKADVKELIFREILEYHPQLLKDYMNGTEKTNFLYPSAVDNFRRQFANLEENGGKGGAVVPPDRKHVSLPRNTTVHSTPIPPKDQKYSQVPQRIPTGRPGRVVGPVIPFENSSTMDPYSQRRVARNPVLPAAATNLSAYAYRNSDNSERELQQELEKDRMQYHPMQRFMDAKMVSPDLRSTSYYMPKGVPKADVAERSALQSNMMQGIAPFNGIATVGGAFNKVSAVQYGVSRMY

>BdMPK20-5 Bradi2g16337.1|feature=AA|genome=B. distachyon|accession=Bd21-1|len=582

MPEANAAARGGGGGGDARAAEQRSNNKNSEEMGFFSEYVDASRYKILEIIGKGSYGVVCSAIDQETGDKVAIKKIQNIFEHLSDAARILREIKLLRLLRHPDIVQIKHIMLPPSRRDFRDIYVVFELMDTDLHQVIKANDDLTKEHYQFFLYQMLRALKYIHTANVYHRDLKPKNILANANCKLKICDFGLARVAFNDTPTTVFWTDYVATRWYRAPELCGSFFTKYSPAIDTWSIGCIFAEILTGKPLFPGKNVVHQLDLMTDLLGTPSTETISRIRNDKARKYLSSMRRKQPIPFSEKFPNADPSALKLLERLLAFDPKDRPTAEEALAHPYFKRLARVEREPSCQQPISKTEFEFERRKFTKEDVKELIFREILEYHPKLLKDYMNGSEKTSFLYPSAVDNFRRQFANLEIDGGRSGAADRKHFSLPRTTTVHSAPILPTNGPTSQVPQRIPTARPGRVVSSAMQTDNPSVSDRHNGRRVARDPAVPPAAAYHLKSDYSDRQHQQEFEKDRVRPDRQRQEELEKDRMQYRPGHHSMDAKVAPEISPYMRSSPYYIPPFNGIAAVASGYSKVAAVTRMY

>BdMPK21-1 Bradi2g15620.1|feature=AA|genome=B. distachyon|accession=Bd21-1|len=596

MGGLIRWLRHHRSRRVSSSSSSHLPSNTTSSSSTSDLRAHSLPQHQGDHHGEVVVEWEDAAEGPDSDPEEYIVVVLGDDEQGVVAARAPVRTKPPRVMDPGKKTSESEFFTEYGEANRYKVSEVIGKGSYGVVAAAVDTQTGERCAIKKINDVFDHVSDATRILREIKLLRLLRHPDIVEIKHIMLPPSRREFRDIYVVFELMESDLHQVIKANDDLTPEHHQFFLYQLLRGMKYIHTANVFHRDLKPKNILANADCKLKICDFGLARVSFNDGAPSAIFWTDYVATRWYRAPELCGSFFSKYTPAIDIWSVGCIFAEMLTGKPLFPGKNVVHQLDLMTDLLGTPSAESLSKIRNEKARRYLSNMRKKPKVPLTKKFPGIDPMALHLLERLLAFDPKDRPSADEALTDPYFTGLANSEREPITQPISKLEFEFEKRKLAKDDVRELIYREILEYHPHMLQEYLRGGGDQMSFMFPSGVDRFKRQFAHLEEGGAKGEKSSPQLRQNASLPRERVIGNKHGDGDYNMKLNVGEKPELASVSDGISKPLMSARSLLKSETMSASKCIGEIKNKDEDSLSECVEGTDDDVSQKIAQLKT

>BdMPK21-2 Bradi2g45006

MARKGSDEAEFFTEYGEANRYEVGEVVGKGSYGVVAAAVDTHTGERVAIKKINDVFEHVSDATRILREIKLLRLLRHPDIVIKANDDLTPEHHQFFFYQL

LRGMKYIHAANVFHRDLKPRNILANADCKLKICDFGLARVSFNDTPSAIFWTDYVATRWYRAPELCGSFFSKYTPAIDIWSIGCIFAEMLSGRPLFPGKN

VVHQLDLMTDLLGTPSAESLSRIRNEKARRYLGNMRKKHPVPFSQKFPGVDPMALDLLERLLAFDPKDRPTAAEALADPYFTGLANSDREPTTQPISKLE

FEFERRKLARDDVRELIYREILEYHPQMLHEYHHGGDQANFVYPSGVDRFKRQFVHLEEGVTKGEKTSPQLRQHASLPRERIIGIGDELGRPNADYCIKL

HVGEEPGHTSVTDGLSKPLLNARNFLKSESISASQCVVIKEKREKDVSAVTSNIF*

>Bd21RefMPK3 Brdisv20Bd21Ref1011713m.p

MDGAPVAEFRPTMTHGGRFLLYNIFGNQFEITAKYQPPIMPIGRGAYGIVCSVMNFETRE

MVAIKKIANAFDNNMDAKRTLREIKLLRHLDHENIVGLRDVIPPAIPQSFNDVYIATELM

DTDLHHIIRSNQELSEEHCQYFLYQLLRGLKYIHSANVIHRDLKPSNLLLNANCDLKICD

FGLARPSSESDMMTEYVVTRWYRAPELLLNSTDYSAAIDVWSVGCIFMELINRAPLFPGR

DHMHQMRLITEVIGTPTDDDLGFIRNEDARRYMRHLPQFPRRPFPAQFPRVQPAALDLIE

RMLAFNPLQRITVEEALEHPYLERLHDIADEPICTDPFSFDFEQHPLTEDQMKQLIFNEA

LELNPNFRY*

>Bd21RefMPK4 Brdisv20Bd21Ref1030338m.p

MDTSGGGGAGGAAQIQGMATHGGRYVLYNVYGNLFEVASKYAPPIRPIGRGAYGIVCAAV

SSDTGEEVAIKKIGNAFDNHIDAKRTLREIKLLRHMDHENIIAIKDIIRPPRRDDFKDVY

IVTELMDTDLHQIIRSNQPLTDDHCQYFLYQLLRGLKYVHSANVLHRDLKPSNLFLNANC

DLKIADFGLARTTSETDLMTEYVVTRWYRAPELLLNCSQYTAAIDVWSVGCILGEIITRQ

PLFPGRDYIQQLKLITELIGSPDDSSLGFLRSDNARRYMKQLPQYPRQDFRLRFRNMSDG

AVDLLERMLVFDPSRRITVDEALHHPYLASLHDINEEPTCPAPFSFDFEQPSFTEEHMKE

LIWRETLAFNPDPPY*

>Bd21RefMPK6 Brdisv20Bd21Ref1008702m.p

MDGGAQPPDAEMADAGAAVPGAGAGAAGTMENIQATLSHGGRFIQYNIFGNVFEVTVKYK

PPILPIGKGAYGIVCSALNSETGEQVAIKKIANAFDNKIDAKRTLREIKLLRHMDHENIV

AIRDIIPPAQRNSFNDVYIAYELMDTDLHQIIRSNQALSEEHCQYFLYQILRGLKYIHSA

NVLHRDLKPSNLLLNANCDLKICDFGLARTTSETDFMTEYVVTRWYRAPELLLNSSEYTA

AIDVWSVGCIFMELMDRKPLFPGRDHVHQLRLLMELIGTPNEADLDFVNENARRYIRQLP

RHARQSFSEKFPHVHPSAIDLVEKMLTFDPRQRITVEGALAHPYLASLHDISDEPVCTMP

FSFDFEQHALSEEQMKDLIHQEALAFNPDYQ*

>Bd21RefMPK7-1 Brdisv20Bd21Ref1006178m.p

MPSRFPFLSPGCFIRLLSGSASSDLLLLLPRRGQSAPRARVLLKMAMMVDPPNGTGNHGK

HYYTMWQTMFEIDTKYVPIKPIGRGAYGIVCSSTNQENNEKVAIKKINNVFDNRVDALRT

LRELKLLRHLRHENVIALKDIMMPVHRRSFKDVYLVSELMDTDLHQIIKSSQPLSNDHCQ

YFLFQLLRGLKYLHSAGILHRDLKPGNLLVNANCDLKICDFGLARTNNTKGQFMTEYVVT

RWYRAPELLLCCDNYGTSIDVWSVGCIFAELLGRKPIFPGTECLNQLKLIVNVLGTMSEN

DLEFIDNPKARKYIKSLPYTPGTPLTSMYPQAHPLAIDLLQKMLVFDPSKRISVTEALEH

PYMSPLYDPSANPPAQVPIDLDIDENLGVEMIREMLWQEMLQYHPEAARMVNM*

>Bd21RefMPK7-2 Brdisv20Bd21Ref1039827m.p

MKLSPISASFGAMGSLPQKLDELLSTGHWALSGVVMDEIEELTADLHILCNLLLKLSNVH

DPPPTVRFWMKEVRELSYDVEDCADQFVIANDRAVMRRAKIHRATIRTIIITRLKISRLP

KRRKWRLWMTDKVAEFRTRVQDATQRYWRYKFDDCASNPGYASVGHEFATVYAQPGDLVG

IEGPMDELEQWLTDGEEQLKVISIVGLGGVGKTTLAQKLWATLKGQFLCRAFVRTAQKPD

MRGILRNILLQVRPHQLPNRGEMHHLIHDLREYLQDKRYFVIIDDLWAASVWDVVSRAFP

EGNCCSRIVTTTEIMEVALACCDYCPKHICKLETLSDDDSEKLLLERIVVSGNQIPQQLD

DVLPQIMRNCGGLPLAIIIVASLLVSQPEKLEQWGHTQNSFGSIFGTNPTMEGFIRQILS

ISFNSLPYYLKTCLLYLSSYPEGCLFLKDDLVKLWVAEGFIHAKEWEDMEDLAGRYFDEL

VNVGLIQVMDINYNYKLPSYSVHHMVLDFITCKSIEENFITVVDYSETTTPLTDKVRRLS

LHFGSATHATTPASARLSNVRSLFYFGLFNCMPSFMVFKLLRVLILNFWGDPGNTSFNLT

GICELVWLRYLQVSCNVTVKLPDQIESMGHLETLEIKAKVYAVPLDIVRLSSLSHLHLRG

GTIPNGIGCMRSLRTLKYFDLVNKSVDNLRGLGELANLRDLHLTYSSSLPSEHLKRNLIA

LASSLGKLCNLKSLTLASETAATVVPFDGSISMSSTPAFLERLELLPSICIFSRLPKWIF

QFQKLCILKVAVRELLANDIDSITGLPSLTVLSLCVHTAPGQRIVFNDGAFPVLKYFKFR

CGVLSMSFMAGAMPNLRRLKLGFNTHVGEKYGNMLAGIEHLLNLQDIAGKIGVATESDRK

AAESAIKGAISKHPQSPMLNVQWVDLVEEEYHASEKQHGRQEKGLSGENHAVLEKAKDTK

KHADSGVSQLPDLPSTVSSSRLKSENIAQASKHHVLESMLCDESAQPESLEVSVLEDITN

NFSDDQEISKNRFVVVYKGELQNGSVVVVKRFTIAIDYNLFLDAVDCLMSVKHNNIVRFL

GYCANSQENAAKEGSKSVSVVTQERLCCFEYLRNGNLQMHLTDESCGFDWQMWYQIIEGI

CQGVHYLHNKSITPLDLNPANIMFDDKMVPKIVHYAYTRFLGEATSPVLTNIGDSLAYMA

PEYFGFGEITSKSDIYSLGVIIMQIVTGENKKDFPSIQSVLESWRSRLESDGSQGHTSLE

TCYLQVNLCYLIGIRCIEHDPGKRPNTRDIIDSLDTLEIMECLVKHDVGNSSLEQMLVCS

KVEEPKALPAPPSVSCKLKMAMMVDPPNGTGNHGKHYYTMWQTMFEIDTKYMPIKPIGRG

AYGIVCSSTNQENNEKVAIKKINNVFDNRVDALRTLRELKLLRHLRHENVIALKDIMMPV

HRRSFKDVYLVFELMDTDLHQIIKSSQPLSNDHCQYFLFQLLRGLKYLHSAGILHRDLKP

GNLLVNANCDLKICDFGLARTNNTKGQFMTEYVVTCLYRAPELLLCCDNYGTSIDVWSVG

CIFAELLGRKPIFPRTQCLDQLKLIVNVLGTMSENDLEFIDNPKARKYIKSLPYTPGTPL

TSMYPQAHPLAIDLLQKMLVFDPSKRISVTEALEHPYMSPLYDPSANPPAQVPIDLDIDE

NLGVEMIREMLWQEMLQYHPEAARMVNM*

>Bd21RefMPK11 Brdisv20Bd21Ref1027806m.p

MRMEGGGAGPAAAAAAGGAHGLGEAQIKGTLTHGGRYVQYNVYGNLFEVSAKYVPPIRPV

GRGACGIICAAINVQTREEVAIKKIGNAFDNQIDAKRTLREVKLLRHMNHENVISIKDII

RPPRRENFNDVYIVYELMDTDLHHLLRSNQPLTDDHCQYFLYQVLRGLKYVHSANVLHRD

LRPSNLLLNAKCDLKIGDFGLARTTTETDFMMEYVVTRWYRAPELLLNCSEYTGAIDMWS

VGCILGEIATREPLFPGKDYVHQLRLITELLGSPDDTSLGFLRSDNARRYVRSLPQYPKQ

QFRSRFPNMSSGAMDLLERMLVFDPNKRITVDEALCHPYLASLHEINDEPVCPAPFSFDF

EQPSFTEEDIKELIWRESVKFNPEPIH*

>Bd21RefMPK14 Brdisv20Bd21Ref1025543m.p

MAMLVDPPNGMGNQGKHYYSMWQTLFEIDTKYVPIKPIGRGAYGIVCSSINRETNEKVAI

KKIHNVFDNRVDALRTLRELKLLRHLRHENVISLKDIMMPVQRRSFKDVYLVYELMDTDL

HQIIKSPQGLSNDHCQYFLFQLLRGLKYLHSAEILHRDLKPGNLLVNANCDLKICDFGLA

RTNSSKGQFMTEYVVTRWYRAPELLLCCDNYGTSIDVWSVGCIFAELLGRKPIFPGTECL

NQLKLIVNVLGTMSESDLEFIDNPKARRYIKTLPYTPGVPLASMYPHAHPLAIDLLQKML

IFDPTKRISVTEALEHPYMSPLYDPSANPPAQVPIDLDLDENISADMIREMMWQEMLHYH

PEAATAISM*

>Bd21RefMPK16 Brdisv20Bd21Ref1020074m.p

MDFFTEYGEGNRYKIEEVIGKGSYGVVCSALDTHTGDKVAIKKINDIFEHVSDATRILRE

IKLLRLLRHPDIVEIKHILLPPSRREFKDIYVVFELMESDLHQVIKANDDLTPEHYQFFL

YQLLRGLKYIHTANVFHRDLKPKNILANADCKLKICDFGLARVAISDTPTAIFWTDYIAT

RWYRAPELCGSFFSKYTPAIDIWSIGCIFAELLTGKPLFPGKNVVHQLDIITDLLGTPSA

ETIARIRNEKARRYLSSMRRKKTVPFTQKFPNADPLALNLLERMLAFDPKDRPSAEEALA

DPYFKNIASVDREPSAQPITKLEFEFERRRITKDDIRELIYREILEYHPNMLREFLEGAE

PTGFMYPSAVDHFKKQFTFLEEHYAKGSTAAPPERQHNSLPRPSVIYSDNRPQNAANITD

DLSRCAIRDNTQKAQKDSASVGANRIPHGAAAAAARPGKVVGSVLRYGNCSTSGTEQYEQ

RRVVRSPGIAPNGVTSGSSYPRRNNTCKSETGEAERIDTNQGGPPKPYVANKLPATVDGR

NGHW*

>Bd21RefMPK17 Brdisv20Bd21Ref1006298m.p

MVGGGGIVDGFRRLFHRRTPSGSGPSSNQSSAGEESSDIDAVVEDLDLVGLRAIRVPKRK

MPLPVESHKKNILEKEFFTEYGEASQYQIQEVIGKGSYGVVAAAVDTRTGERVAIKKIND

VFEHVSDATRILREVKLLRLLRHPDVVEIKHIMLPPSRREFQDIYVVFELMESDLHQVIR

ANDDLTPEHYQFFLYQLLRALKYIHAANVFHRDLKPKNILANSDCKLKICDFGLARVSFN

DAPTAIFWTDYVATRWYRAPELCGSFFSKYTPAIDIWSIGCIFAELLTGRPLFPGKNVVH

QLDIITDLLGTPSSETLSRIRNEKARRYLSCMRKKHPVPLTQKFPNVDPLALRLLGRLLA

FDPKDRPSAEEALADPYFAVLANVEREPSRNPISKLEFEFERRKVTKDDVRELIYREILE

YHPQMLQEYMQGGEQLSFLYPSGVDRFKRQFAHLEENYSKGERGSPLQRKHASLPRQRVG

ASNDNNDQHASDQERGADSIAPATGNPPGSQDAGQQHVSGGQNGVGSTNISPRSYQKSAS

ISASKCVVVNANKNPEYDDDISEEMEGAVDGLSEKVSRMHP*

>Bd21RefMPK20-1 Brdisv20Bd21Ref1021539m.p

MQQDQRKKSSAEAEFFTEYGDASRYKIQEIVGKGSYGVVCSAIDVHTGEKVAIKKIHDIF

EHISDAARILREIKLLRLLRHPDIVEIKHIMLPPSRRDFKDIYVVFELMESDLHQVIKAN

DDLTKEHYQFFLYQLLRALKYIHTANVYHRDLKPKNILANSNCKLKICDFGLARVAFNDT

PTTIFWTDYVATRWYRAPELCGSFFSKYTPAIDVWSIGCIFAEVLTGKPLFPGKNVVHQL

DLMTDLLGTPSMDTISRVRNEKARRYLSSMRKKEPIPFSQKFPNADPLALDLLQRLLAFD

PKDRPTAGEALSHPYFKGLAKVEREPSCQPITKMEFEFERRRATKEDIRELIFREILEYH

PQLLKDYINGTERTTFLYPSAVDQFRKQFAHLEENSESGPVVPMDRKHTSLPRSTIVHSA

PIHAKEQPRIGPSRDRPLTDESYKNPRDSEKYSGNVPRSSHHSQAPQRVPTARPGRVVGP

VLPYENVGTSHPYDPRRVAMNSGYPPQQQIPQTYGYYQTTGKSACSEPSQAERYTLHQQA

YACANSSTAPDVALDMRAPPFHQSAGPKSGSSDRLTAETNLYTRSLNGIVATTSGVAASA

HRKVSVVPYGMSQMY*

>Bd21RefMPK20-2 Brdisv20Bd21Ref1016842m.p

MQQPQQQGQRNKSTDVDFFSEYGDANRYRIQEVIGKGSYGVVCSAMDMQTRQKVAIKKIH

NIFDHVSDAARILREIKLLRLLRHPDVVEIKHIMLPPSRKDFKDIYVVFELMESDLHQVI

KANDDLTKEHYQFFLYQLLRALKYIHTASVYHRDLKPKNILANSNCKLKICDFGLARVAF

NDTPTTVFWTDYVATRWYRAPELCGSFFTKYTPAIDIWSIGCIFAEVLTGKPLFPGKNVV

HQLDLMTDLLGTPSMDTISRVRNEKARRYLSSMRKKDPVPFSKKFPNADPLGLKLLEKLL

AFDPKDRPTAEEALTDPYFKSLSKPDREPSCQPIRKVEFDFEHRRMSKDDIRELIFQEIL

EYHPQLLKNYIDGTEKTTFLYPSAVDQFKKQFSHLEESDGSGPVVPTERKHASLPRSTTV

HSTPIPAKEQPLVASSRGRPIANEPCKPWAPGNVPGASQTAHVAQAGRAVGVGSVPPYES

GSGKYPYDATSRPAVSSGYPPQQKIPQTYGYHHHHQTPGAGQSSQAMGGYACGYTKGTTP

PPAAAQDMRASPYHHRSAGTKNDPLNRLAAESDIYTRSLNGIVAAAASAGTGAHRKVGAV

PFGMSGMY*

>Bd21RefMPK20-3 Brdisv20Bd21Ref1007272m.p

MQHGDLHKKSAAEMDFFTAYDDANRYKILEVIGKGSYGLVCSANDLQTGEKVAIKKIHNI

FEHISDAARILREIKLLRLLRHPDVVEIKHILLPPSKKDFKDIYVVFELMESDLHQVIKA

NDDLTREHYQFFLYQMLRALKYMHTANVYHRDLKPKNVLANANCKLKICDFGLARVAFSD

APTTVFWTDYVATRWYRAPELCGSFYSKYTPAIDIWSIGCIFAEVLIGKPLFPGKNVVHQ

LDLITDILGTPSLDAISRVRNDKARKYLTCMRKKQPASFSQKFPKADPLALQLLRRLLAF

DPKDRPSAEEALADPYFNGLAKVEREPSCQPIPKIEFEFEGRRVTKEDIKELIFEEILEY

HPQLLKEHIIGKERPNFVHLSAVDQFKKHFTQLEENDNETGAAVSLQRKHSSLPRQAFNH

R*

>Bd21RefMPK20-4 Brdisv20Bd21Ref1021778m.p

MQTTEQQRKKGSSEMDFFSEYGDANRYKIQEVIGKGSYGVVCSAIDQHTGDKVAIKKIHN

IFEHLSDAARILREIKLLRLLRHPDIVEIRHIMLPPSRRDFKDIYVVFELMDTDLHQVIK

ANDDLTKEHHQFFLYQMLRALKYIHTANVYHRDLKPKNILANANCKLKICDFGLARVAFN

DTPTTVFWTDYVATRWYRAPELCGSFFTKYSRAIDIWSIGCIFAEILTGKPLFPGKNVVH

QLDLMTDLLGTPSLDTVSRIRNEKARRYLSSMRKKQSVSFSERFPKADPAALKLLQRLLA

FDPKDRPTAEEALADPYFKGLGKVEREPSCQPITKMEFEFERKNVTKADVKELIFREILE

YHPQLLKDYMNGTEKTNFLYPSAVDNFRRQFANLEENGGKGGAVVPPDRKHVSLPRNTTV

HSTPIPPKDQKYSQVPQRIPTGRPGRVVGPVIPFENSSTMDPYSQRRVARNPVLPAAATN

LSAYAYRNSDNSERELQQELEKDRMQYHPMQRFMDAKMVSPDLRSTSYYMPKGVPKADVA

ERSALQSNMMQGIAPFNGIATVGGAFNKVSAVQYGVSRMY*

>Bd21RefMPK20-5 Brdisv20Bd21Ref1016994m.p

MFSDIFSREGVAFSRVALRQELLSDQWFTTAGGKGQCYLVPPGTTVFLVNSEEMGFFSEY

VDASRYKILEIIGKGSYGVVCSAIDQETGDKVAIKKIQNIFEHLSDAARILREIKLLRLL

RHPDIVQIKHIMLPPSRRDFRDIYVVFELMDTDLHQVIKANDDLTKEHYQFFLYQMLRAL

KYIHTANVYHRDLKPKNILANANCKLKICDFGLARVAFNDTPTTVFWTDYVATRWYRAPE

LCGSFFTKYSPAIDTWSIGCIFAEILTGKPLFPGKNVVHQLDLMTDLLGTPSTETISRIR

NDKARKYLSSMRRKQPIPFSEKFPNADPSALKLLERLLAFDPKDRPTAEEALAHPYFKRL

ARVEREPSCQQPISKTEFEFERRKFTKEDVKELIFREILEYHPKLLKDYMNGSEKTSFLY

PSAVDNFRRQFANLEIDGGRSGAADRKHFSLPRTTTVHSAPILPTNGPTSQVPQRIPTAR

PGRVVSSAMQTDNPSVSDRHNGRRVARDPAVPPAAAYHLKSDYSDRQHQQEFEKDRVRPD

RQRQEELEKDRMQYRPGHHSMDAKVAPEISPYMRSSPYYIPPFNGIAAVASGYSKVAAVT

RMY*

>Bd21RefMPK21-1 Brdisv20Bd21Ref1016889m.p

MGGLIRWLRHHRSRRVSSSSSSHLPSNTTSSSSTSDLRAHSLPQHQGDHHGEVVVEWEDA

AEGPDSDPEEYIVVVLGDDEQGVVAARAPVRTKPPRVMDPGKKTSESEFFTEYGEANRYK

VSEVIGKGSYGVVAAAVDTQTGERCAIKKINDVFDHVSDATRILREIKLLRLLRHPDIVE

IKHIMLPPSRREFRDIYVVFELMESDLHQVIKANDDLTPEHHQFFLYQLLRGMKYIHTAN

VFHRDLKPKNILANADCKLKICDFGLARVSFNDGAPSAIFWTDYVATRWYRAPELCGSFF

SKYTPAIDIWSVGCIFAEMLTGKPLFPGKNVVHQLDLMTDLLGTPSAESLSKIRNEKARR

YLSNMRKKPKVPLTKKFPGIDPMALHLLERLLAFDPKDRPSADEALTDPYFTGLANSERE

PITQPISKLEFEFEKRKLAKDDVRELIYREILEYHPHMLQEYLRGGGDQMSFMFPSGVDR

FKRQFAHLEEGGAKGEKSSPQLRQNASLPRERVIGNKHGDGDYNMKLNVGEKPELASVSD

GISKPLMSARSLLKSETMSASKCIGEIKNKDEDSLSECVEGTDDDVSQKIAQLKT*

>Bd21RefMPK21-2 Brdisv20Bd21Ref1021630m.p

MARKGSDEAEFFTEYGEANRYEVGEVVGKGSYGVVAAAVDTHTGERVAIKKINDVFEHVS

DATRILREIKLLRLLRHPDIVEIKHIILPPSRREFRDIYIIFELMESDLHQVIKANDDLT

PEHHQFFFYQLLRGMKYIHAANVFHRDLKPRNILANADCKLKICDFGLARVSFNDTPSAI

FWTDYVATRWYRAPELCGSFFSKYTPAIDIWSIGCIFAEMLSGRPLFPGKNVVHQLDLMT

DLLGTPSAESLSRIRNEKARRYLGNMRKKHPVPFSQKFPGVDPMALDLLERLLAFDPKDR

PTAAEALADPYFTGLANSDREPTTQPISKLEFEFERRKLARDDVRELIYREILEYHPQML

HEYHHGGDQANFVYPSGVDRFKRQFVHLEEGVTKGEKTSPQLRQHASLPRERIIGIGDEL

GRPNADYCIKLHVGEEPGHTSVTDGLSKPLLNARNFLKSESISASQCVVIKEKREKDEES

MSEYMHEASDATPK*

>Bd21-3MPK3 Brdisv1Bd21-3_r1010642m.p

MDGARVAEFRPTMTHGGRFLLYNIFGNQFEITAKYQPPIMPIGRGAYGIVCSVMNFETRE

MVAIKKIANAFDNNMDAKRTLREIKLLRHLDHENIVGLRDVIPPAIPQSFNDVYIATELM

DTDLHHIIRSNQELSEEHCQYFLYQLLRGLKYIHSANVIHRDLKPSNLLLNANCDLKICD

FGLARPSSESDMMTEYVVTRWYRAPELLLNSTDYSAAIDVWSVGCIFMELINRAPLFPGR

DHMHQMRLITEVIGTPTDDDLGFIRNEDARRYMRHLPQFPRRPFPAQFPRVQPAALDLIE

RMLAFNPLQRITGTYILEEAREHPYLERLHDIAGEPICTDPFSFDFEQHPLTEDQMKQLI

FNEALELNPNFRY*

>Bd21-3MPK4 Brdisv1Bd21-3_r1027304m.p

MDTSGGGGGGAGGAAQIQGMATHGGRYVLYNVYGNLFEVASKYAPPIRPIGRGAYGIVCA

AVSSDTGEEVAIKKIGNAFDNHIDAKRTLREIKLLRHMDHENIIAIKDIIRPPRRDDFKD

VYIVTELMDTDLHQIIRSNQPLTDDHCQYFLYQLLRGLKYVHSANVLHRDLKPSNLFLNA

NCDLKIADFGLARTTSETDLMTEYVVTRWYRAPELLLNCSQYTAAIDVWSVGCILGEIIT

RQPLFPGRDYIQQLKLITELIGSPDDSSLGFLRSDNARRYMKQLPQYPRQDFRLRFRNMS

DGAVDLLERMLVFDPSRRITVDEALHHPYLASLHDINEEPTCPAPFSFDFEQPSFTEEHM

KELIWRETLAFNPDPPY*

>Bd21-3MPK6 Brdisv1Bd21-3_r1007822m.p

MDGGAQPPDAEMADAGAAVPGAGAGAAGTMENIQATLSHGGRFIQYNIFGNVFEVTVKYK

PPILPIGKGAYGIVCSALNSETGEQGAIKKIANAFDNKIDAKRTLREIKLLRHMDHENIV

AIRDIIPPAQRNSFNDVYIAYELMDTDLHQIIRSNQALSEEHCQYFLYQILRGLKYIHSA

NVLHRDLKPSNLLLNANCDLKICDFGLARTTSETDFMTEYVVTRWYRAPELLLNSSEYTA

AIDVWSVGCIFMELMDRKPLFPGRDHVHQLRLLMELIGTPNEADLDFVNENARRYIRQLP

RHARQSFSEKFPHVHPSAIDLVEKMLTFDPRQRITVEGALAHPYLASLHDISDEPVCTMP

FSFDFEQHALSEEQMKDLIHQEALAFNPDYQ*

>Bd21-3MPK11 Brdisv1Bd21-3_r1025222m.p

MRMEGGGAGPAAAAAAGGAHGLGEAQIKGTLTHGGRYVQYNVYGNLFEVSAKYVPPIRPV

GRGACGIICAAINVQTREEVAIKKIGNAFDNQIDAKRTLREVKLLRHMNHENVISIKDII

RPPRRENFNDVYIVYELMDTDLHHLLRSNQPLTDDHCQGHCSFHHLYFLYQVLRGLKYVH

SANVLHRDLRPSNLLLNAKCDLKIGDFGLARTTTETDFMMEYVVTRWYRAPELLLNCSEY

TGAIDMWSVGCILGEIATREPLFPGKDYVHQLRLITELLGSPDDTSLGFLRSDNARRYGR

SLPQYPKQQFRSRFPNMSSGAMDLLERMLVFDPNKRITVDEALCHPYLASLHEINDEPVC

PAPFSFDFEQPSFTEEDIKELIWRESVKFNPEPIH*

>Bd21-3MPK14 Brdisv1Bd21-3_r1023190m.p

MAMLVDPPNGMGNQGKHYYSMWQTLFEIDTKYVPIKPIGRGAYGIVCSSINRETNEKVAI

KKIHNVFDNRVDALRTLRELKLLRHLRHENVISLKDIMMPVQRRSFKDVYLVYELMDTDL

HQIIKSPQGLSNDHCQYFLFQLLRGLKYLHSAEILHRDLKPGNLLVNANCDLKICDFGLA

RTNSSKGQFMTEYVVTRWYRAPELLLCCDNYGTSIDVWSVGCIFAELLGRKPIFPGTECL

NQLKLIVNVLGTMSESDLEFIDNPKARRYIKPLPYTPGVPLASMYPHAHPLAIDLLQKML

IFDPTKRISVTEALEHPYMSPLYDPSANPPAQVPIDLDLDENISADMIREMMWQEMLHYH

PEAATAISM*

>Bd21-3MPK16 Brdisv1Bd21-3_r1018023m.p

MDFFTEYGEGNRYKIEEVIGKGSYGVVCSALDTHTGDKVAIKKINDIFEHVSDATRILRE

IKLLRLLRHPDIVEIKHILLPPSRREFKDIYVVFELMESDLHQVIKANDDLTPEHYQFFL

YQLLRGLKYIHTANVFHRDLKPKNILANADCKLKICDFGLARVAISDTPTAIFWTDYIAT

RWYRAPELCGSFFSKYTPAIDIWSIGCIFAELLTGKPLFPGKNVVHQLDIITDLLGTPSA

ETIARIRNEKARRYLSSMRRKKTVPFTQKFPNADPLALNLLERMLAFDPKDRPSAEEALA

DPYFKNIASVDREPSAQPITKLGFEFERRRITKDDIRELIYREILEYHPNMLREFLEGAE

PTGFMYPSAVDHFKKQFTFLEEHYAKGSTAAPPERQHNSLPRPSVIYSDNRPQNAANITD

DLSRCAIRDNTQKAQKDSASVGANRIPHGAAAAAARPGKVVGSVLRYGNCSTSGTEQYEQ

RRVVRSPGIAPNGVTSGSSYPRRNNTCKSETGEAERIDTNQGGPPKPYVANKLPATVDGR

NGHW*

>Bd21-3MPK17 Brdisv1Bd21-3_r1005812m.p

MVGGGGIVDGFRRLFHRRTPSGSGPSSNQSSAGEESSDIDAVVEDLDLVGLRAIRVPKRK

MPLPAESHKKNILEKEFFTEYGEKSQYQIQEVIGKGSYGVVAAAVDTRTGERGAIKKIND

VFEHVSDATRILREVKLLRLRRHPDVVEIKHIMLPPSRREFQDIYVVFELMESDLHQVIR

ANDDLTPEHYQFFLYQLLRALKYIHAANVFHRDLKPKNILANSDCKLKICDFGLARVSFN

DAPTAIFWTDYVATRWYRAPELCGSFFSKYTPAIDIWSIGCIFAELLTGRPLFPGKNVVH

QLDIITDLLGTPSSETLSRIRNEKARRYLSCMRKKHPVPLTQKFPNVDPLALRLLGRLLA

FDPKDRPSAEEALADPYFAVLANVEREPSRNPISKLEFEFERRKVTKDDVRELIYREILE

YHPQMLQEYMQGGEQLSFLYPSGVDRFKRQFAHLEENYSKGERGSPLQRKHASLPRQRVG

ASNDNNDQHASDQERGADSIAPATGNPPGSQDAGQQHVSGGQNGVGSTNISPRSYQKSAS

ISASKCVVVNANKNPEYDDDISEEMEGAVDGLSEKVSRMHP*

>Bd21-3MPK20-1 Brdisv1Bd21-3_r1019381m.p

MQQDQRKKSSAEAEFFTEYGDASRYKIQEIVGKGSYGVVCSAIDVHTGEKVAIKKIHDIF

EHISDAARILREIKLLRLLRHPDIVEIKHIMLPPSRRDFKDIYVVFELMESDLHQVIKAN

DDLTKEHYQFFLYQLLRALKYIHTANVYHRDLKPKNILANSNCKLKICDFGLARVAFNDT

PTTIFWTDYVATRWYRAPELCGSFFSKYTPAIDVWSIGCIFAGVLTGKPLFPGKNVVHQL

DLMTDLLGTPSMDTISRGWRYLSSMRKKEPIPFSQKFPNADPLALDLLQRLLAFDPKDRP

TAGEALSHPYFKGLAKVEREPSCQPITKMEFEFERRRATKEDIRELIFREILEYHPQLLK

DYINGTERTTFLYPSAVDQFRKQFAHLEENSESGPVVPMDRKHTSLPRSTIVHSAPIHAK

EQPRIGPSRDSPLTDESYKTPRDSEKYSGNVPRSSHHSQAPQRVPTARPGRVVGPVLPYE

NVGTSHPYDPRRVAMNSGYPPQQQIPQTYGYYQTTGKSACSEPSQAERYTLHQQAYACAN

SSTAPDVALDMRAPPFHQSAGPKSGSSDRLTAETNLYTRSLNGIVATTSGVAASAHRKVS

VVPYGMSQMY*

>Bd21-3MPK20-2 Brdisv1Bd21-3_r1015197m.p

MQQPQQQGQRNKSTDVDFFSEYGDANRYRIQEVIGKGSYGVVCSAMDMQTRQKVAIKKIH

NIFDHVSDAARILREIKLLRLLRHPDVVEIKHIMLPPSRKDFKDIYVVFELMESDLHQVI

KANDDLTKEHYQFFLYQLLRALKYIHTASVYHRDLKPKNILANSNCKLKICDFGLARVAF

NDTPTTVFWTDYVATRWYRAPELCGSFFTKYTPAIDIWSIGWIFAEVLTGKPLFPGKNVV

HQLDLMTDLLGTPSMDTISRVRNEKARRYLSSMRKKDPVPFSKKFPNADPLGLKLLEKLL

AFDPKDRPTAEEGWFLFLFFCPALTDPYFKSLSKPDREPSCQPIRKVEFDFEHRRMSKDD

IRELIFQEILEYHPQLLKNYIDGTEKTTFLYPSAVDQFKKQFSHLEESDGSGPVVPTERK

HASLPRSTTVHSTPIPAKEQPLVASSRGRPIANEPCKPWAPGNVPGASQTAHVAQAGRAV

GVGSVPPYESGSGKYPYDATSRPAVSSGYPPQQKIPQTYGYHHHHQTPGAGQSSQAMGGY

ACGYTKGTTPPPAAAQDMRASPYHHRSAGTKNDPLNRLAAESDIYTRSLNGIVAAAASAG

TGAHRKVGAVPFGMSGMY*

>Bd21-3MPK20-3 Brdisv1Bd21-3_r1006610m.p

MQHGDLHKKSAAEMDFFTAYDDANRYKILEVIGKGSYGLVCSANDLQTGEKVAIKKIHNI

FEHISDAARILREIKLLRLLRHPDVVEIKHILLPPSKKDFKDIYVVFELMESDLHQVIKA

NDDLTREHYQFFLYQMLRALKYMHTANVYHRDLKPKNVLANANCKLKICDFGLARVAFSD

APTTVFWTDYVATRWYRAPELCGSFYSKYTPAIDIWSIGCIFAEVLIGKPLFPGKNVVHQ

LDLITDILGTPSLDAISRVRNDKARKYLTCMRKKQPASFSQKFPKADPLALQLLRRLLAF

DPKDRPSAEEALADPYFNGLAKVEREPSCQPIPKIEFEFEGRRVTKEDIKELIFEEILEY

HPQLLKEHIIGKERPNFVHLSAVDQFKKHFTQLEENDNETGAAVSLQRKHSSLPRQAFNH

R*

>Bd21-3MPK20-4 Brdisv1Bd21-3_r1019613m.p

MQTTEQQRKKGSSEMDFFSEYGDANRYKIQEVIGKGSYGVVCSAIDQHTGDKVAIKKIHN

IFEHLSDAARILREIKLLRLLRHPDIVEIRHIMLPPSRRDFKDIYVVFELMDTDLHQVIK

ANDDLTKEHHQFFLYQMLRALKYIHTANVYHRDLKPKNILANANCKLKICDFGLARVAFN

DTPTTVFWTDYVATRWYRAPELCGSFFTKYSRAIDIWSIGCIFAEILTGKPLFPGKNVVH

QLDLMTDLLGTPSLDTVSRIRNEKARRYLSSMRKKQSVSFSERFPKADPAALKLLQRLLA

FDPKDRPTAEEALADPYFKGLGKVEREPSCQPITKMEFEFERKNVTKADVKELIFREILE

YHPQLLKDYMNGTEKTNFLYPSAVDNFRRQFANLEENGGKGGAVVPPDRKHVSLPRNTTV

HSTPIPPKDQKYSQVPQRIPTGRPGRVVGPVIPFENSSTMDPYSQRRVARNPVLPAAATN

LSAYAYRNSDNSERELQQELEKDRMQYHPMQRFMDAKMVSPDLRSTSYYMPKGVPKADVA

ERSALQSNMMQGIAPFNGIATVGGAFNKVSAVQYGVSRMY*

>Bd21-3MPK20-5 Brdisv1Bd21-3_r1015339m.p

MFSDIFSREGVAFSRVALRQELLSDQWFTTAGGKGQCYLVPPGTTVFLVNSEEMGFFSEY

VDASRYKILEIIGKGSYGVVCSAIDQETGDKVAIKKIQNIFEHLSDAARILREIKLLRLL

RHPDIVQIKHIMLPPSRRDFRDIYVVFELMDTDLHQVIKANDDLTKEHYQFFLYQMLRAL

KYIHTANVYHRDLKPKNILANANCKLKICDFGLARVAFNDTPTTVFWTDYVATRWYRAPE

LCGSFFTKYSPAIDTWSIGCIFAEILTGKPLFPGKNVVHQLDLMTDLLGTPSTETISRIR

NDKARKYLSSMRRKQPIPFSEKFPNADPSALKLLERLLAFDPKDRPTAEEALAHPYFKRL

ARVEREPSCQQPISKTEFEFERRKFTKEDVKELIFREILEYHPKLLKDYMNGSEKTSFLY

PSAVDNFRRQFANLEIDGGRSGAADRKHFSLPRTTTVHSAPILPTNGPTSQVPQRIPTAR

PGRVVSSAMQTDNPSVSDRHNGRRVARDPAVPPAAAYHLKSDYSDRQHQQEFEKDRVRPD

RQRQEELEKDRMQYRPGHHSMDAKVAPEISPYMRSSPYYIPPFNGIAAVASGYSKVAAVT

RMY*

>Bd21-3MPK21-1 Brdisv1Bd21-3_r1015240m.p

MGGLIRWLRHHRSRRVSSSSSSHLPSNTTSSSSTSDLRAHSLPQHQGDHHGEVVVEWEDA

AEGPDSDPEEYIVVVLGDDEQGVVAARAPVRTKPPRVMDPGKKTSESEFFTEYGEANRYK

VSEVIGKGSYGVVAAAVDTQTGERCAIKKINDVFDHVSDATRILREIKLLRLLRHPDIVE

IKHIMLPPSRREFRDIYVVFELMESDLHQVIKANDDLTPEHHQFFLYQLLRGMKYIHTAS

VFHRDLKPKNFLANADCKLKICDFGLARVSFNEGPPPAIFWTDYVATRWYRAPELCGSFF

SKYTPAIDIWSVGCIFAEMLTGKPLFPGKNVVHQLDLMTDLLGTPSAESLSKIRNEKARR

YLSNMRKKPKVPLTKKFPGIDPMALHLLERLLAFDPKDRPSADEALTDPYFTGLANSERE

PITQPISKLEFEFEKRKLAKDDVRELIYREILEYHPHMLQEYLRGGGDQMSFMFPSGVDR

FKRQFAHLEEGGAKGEKSSPQLRQNASLPRERVIGNKHGDGDYNMKLNVGEKPELASVSD

GISKPLMSARSLLKSETMSASKCIGEIKNKDEDSLSECVEGTDDDVSQKIAQLKT*

>Bd21-3MPK21-2 Brdisv1Bd21-3_r1019467m.p

MARKGSDEAEFFTEYGEANRYEVGEVVGKGSYGVVAAAVDTHTGERVAIKKINDVFEHVS

DATRILREIKLLRLLRHPDIVEIKHIILPPSRREFRDIYIIFELMESDLHQVIKANDDLT

PEHHQFFFYQLLRGMKYIHAANVFHRDLKPRNILANADCKLKICDFGLARVSFNDTPSAI

FWTDYVATRWYRAPELCGSFFSKYTPAIDIWSIGCIFAEMLSGRPLFPGKNVVHQLDLMT

DLLGTPSAESLSRIRNEKARRYLGNMRKKHPVPFSQKFPGVDPMALDLLERLLAFDPKDR

PTAAEALADPYFTGLANSDREPTTQPISKLEFEFERRKLARDDVRELIYREILEYHPQML

HEYHHGGDQANFVYPSGVDRFKRQFVHLEEGVTKGEKTSPQLRQHASLPRERIIGIGDEL

GRPNADYCIKLHVGEEPGHTSGTDGLRKPLLNARNFLKSESISASQCVVIKEKREKDEES

MSEYMHEASDATPK*

>Bd3-1MPK3 Brdisv1Bd3-1_r1008330m.p

MDGAPVAEFRPTMTHGGRFLLYNIFGNQFEITAKYQPPIMPIGRGAYGIVCSVMNFETRE

MVAIKKIANAFDNNMDAKRTLREIKLLRHLDHENIVGLRDVIPPAIPQSFNDVYIATELM

DTDLHHIIRSNQELSEEHCQYFLYQLLRGLKYIHSANVIHRDLKPSNLLLNANCDLKICD

FGLARPSSESDMMTEYVVTRWYRAPELLLNSTDYSAAIDVWSVGCIFMELINRAPLFPGR

DHMHQMRLITEVIGTPTDDDLGFIRNEDARRYMRHLPQFPRRPFPAQSPRGQPAALDLIE

RMLAFNPLQRITVEEALEHPYLERLHDIADEPICTDPFSFDFEQHPLTEDQMKQLIFNEA

LELNPNFRY*

>Bd3-1MPK4 Brdisv1Bd3-1_r1021690m.p

MAPHGGRYVLYNVYGNLFEVASKYAPPIRPIGRGAYGIDVPYPFRMVVFVYRLRTRVGEA

AAVSSDTGEEVAIKKIGNAFDNHIDAKRTLREIKLLRHMDHENIIAIKDIIRPPRRDDFK

DVYIVTELMDTDLHQIIRSNQPLTDDHCQYFLYQLLRGLKYVHSANVLHRDLKPSNLFLN

ANCDLKIADFGLARTTSETDLMTEYVVTRWYRAPELLLNCSQYTAAIDVWSVGCILGEII

TRQPLFPGRDYIQQLKLITELIGSPDDSSLGFLRSDNARRYMKQLPQYPRQDFRLRFRNM

SDGAVDLLERMLVFDPSRRITVDEALHHPYLASLHDINEEPTCPAPFSFDFEQPSFTEEH

MKELIWRETLAFNPDPPY*

>Bd3-1MPK6 Brdisv1Bd3-1_r1006154m.p

MDTDLHQIIRSNQALSEEHCQYFLYQILRGLKYIHSANVLHRDLKPSNLLLNANCDLKIC

DFGLARTTSETDFMTEYVVTRWYRAPELLLNSSEYTAAIDVWSVGCIFMELMDRKPLFPG

RDHVHQLRLLMELIGTPNEADLDFVNENARRYIRQLPRHARQSFSEKFPHVHPSAIDLVE

KMLTFDPRQRITVEGALAHPYLASLHDISDEPVCTMPFSFDFEQHALSEEQMKDLIHQEA

LAFNPDYQ*

>Bd3-1MPK11 Brdisv1Bd3-1_r1020329m.p

MRMEGGGAGPAAAAAAGGAHGLGEAQIKGTLTHGGRDVQYNVYGNLFEVSAKYVPPIRPA

GRGARGIICAAINVQTREEVAIKKIGNAFDNQIDAKRTLREVKLLRHMNHENVISIKDII

RPPRRENFNDVYIVYELMDTDLHHLLRSNQPLTDDHCQYFLYQVLRGLKYVHSANVLHRD

LRPSNLLLNAKCDLKIGDFGLARTTTETDFMMEYVVTRWYRAPELLLNCSEYTGAIDMWS

VGCILGEIATREPLFPGKDYVHQLRLITELLGSPDDTSLGFLRSDNARRYVRSLPQYPKQ

QFRSRFPNMSSGAMDLLERMLVFDPNKRITVDEALCHPYLASLHEINDEPVCPAPFSFDF

EQPSFTEEDIKELIWRESVKFNPEPIH*

>Bd3-1MPK14 Brdisv1Bd3-1_r1018689m.p

MAMLVDPPNGMGNQGKHYYSMWQTLFEIDTKYVPIKPIGRGAYGIVCSSINRETNEKVAI

KKIHNVFDNRVDALRTLRELKLLRHLRHENVISLKDIMMPVQRRSFKDVYLVYELMDTDL

HQIIKSPQGLSNDHCQYFLFQLLRGLKYLHSAEILHRDLKPGNLLVNANCDLKICDFGLA

RTNSSKGQFMTEYVVTRLYRAPELLLCCDNYGTSIDVWSVGCIFAELLGRKPIFPGTECL

NQLKLIVNVLGTMSESDLEFIDNPKARRYIKTLPYTPGVPLASMYPHAHPLAIDLLQKML

IFDPTKRISVTEALEHPYMSPLYDPSANPPAQVPIDLDLDENISADMIREMMWQEMLHYH

PEAATAISM*

>Bd3-1MPK16 Brdisv1Bd3-1_r1014394m.p

MDFFTEYGEGNRYKIEEVIGKGSYGVVCSALDTHTGDKVAIKKINDIFEHVSDATRILRE

IKLLRLLRHPDIVEIKHILLPPSRREFKDIYVVFELMESDLHQVIKANDDLTPEHYQFFL

YQLLRGLKYIHTANVFHRDLKPKNILANADCKLKICDFGLARVAISDTPTAIFWTDYIAT

RWYRAPELCGSFFSKYTPAIDIWSIGCIFAELLTGKPLFPGKNVVHQLDIITDLLGTPSA

ETIARIRNEKARRYLSSMRRKKTVPFTQKFPNADPLALNLLERMLAFDPKDRPSAEEALA

DPYFKNIASVDREPSAQPITKLEFEFERRRITKDDIRELIYREILEYHPNMLREFLEGAE

PTGFMYPSAVDHFKKQFTFLEEHYAKGSTAAPPERQHNSLPRPSVIYSDNRPQNAANITD

DLSRCAIRDNTQKAQKDSASVGANRIPHGAAAAAARPGKVVGSVLRYGNCSTSGTEQYEQ

RRVVRSPGIAPNGVTSGSSYPRRNNTCKSETGEAERIDTNQGGPPKPYVANKLPATVDGR

NGHW*

>Bd3-1MPK17 Brdisv1Bd3-1_r1004808m.p

MVGGGGIVDGFPRLFPRRPPSGSGPSSNQSSAGEESSDIDAVVEDLDLVGLRAIRVPQRK

MPLPVESHKKNILEKEFFTEYGEASQYQIQEVIGKGSYGVVAAAVDTRTGERVAIKKIND

VFEHVSDATRILREVKLLRLLRHPDVVEIKHIMLPPSRREFQDIYVVFELMESDLHQVIR

ANDDLTPEHYQFFLYQLLRALKYIHAANVFHRDLKPKNILANSDCKLKICDFGLARVSFN

DAPTAIFWTDYVATRWYRAPELCGSFFSKYTPAIDIWSIGCIFAELLTGRPLFPGKNVVH

QLDIITDLLGTPSSETLSRIRNEKARRYLSCMRKKHPVPLTQKFPNVDPLALRLLGRLLA

FDPKDRPSAEEALADPYFAVLANVEREPSRNPISKLEFEFERRKVTKDDVRELIYREILE

YHPQMLQEYMQGGEQLSFLYPSGVDRFKRQFAHLEENYSKGERGSPLQRKHASLPRQRVG

ASNDNNEQHASDQERGADSIAPATGNPPGSQDAGQQHVSGGQNGVGSTNISPRSYQKSAS

ISASKCVVVNANKNPEYDDDISEEMEGAVDGLSEKVSRMHP*

>Bd3-1MPK20-1 Brdisv1Bd3-1_r1015467m.p

MQQDQRKKSSAEAEFFTEYGDASRYKIQEIVGKGSYGVVCSAIDVHTGEKVAIKKIHDIF

EHISDAARILREIKLLRLLRHPDIVEIKHIMLPPSRRDFKDIYVVFELMESDLHQVIKAN

DDLTKEHYQFFLYQLLRALKYIHTANVYHRDLKPKNILANSNCKLKICDFGLARVAFNDT

PTTIFWTDYVATRWYRAPELCGSFFSKYTPAIDVWSIGCIFAEVLTGKPLFPGKNVVHQL

DLMTDLLGTPSMDTISRVRNEKARRYLSSMRKKEPIPFSQKFPNADPLALDLLQRLLAFD

PKDRPTAGEALSHPYFKGLAKVEREPSCQPITKMEFEFERRRATKEDIRELIFREILEYH

PQLLKDYINGTERTTFLYPSAVDQFRKQFAHLEENSESGPVVPMDRKHTSLPRSTIVHSA

PIHAKEQPRIGPSRDRPLTDESYKNPRDSEKYSGNVPRSSHHSQAPQRVPTARPGRVVGP

VLPYENVGTSHPYDPRRVAMNSGYPPQQQIPQTYGYYQTTGKSACSEPSQAERYTLHQQA

YACANSSTAPDVALDMRAPPFHQSAGPKSGSSDRLTAETNLYTRSLNGIVATTSGVAASA

HRKVSVVPYGMSQMY*

>Bd3-1MPK20-2 Brdisv1Bd3-1_r1012296m.p

MQQPQQQGQRNKSTDVDFFSEYGDANRYRIQEVIGKGSYGVVCSAMDMQTRQKVAIKKIH

NIFDHVSDAARILREIKLLRLLRHPDVVEIKHIMLPPSRKDFKDIYVVFELMESDLHQVI

KANDDLTKEHYQFFLYQLLRALKYIHTASVYHRDLKPKNILANSNCKLKICDFGLARVAF

NDTPTTVFWTDYVATRWYRAPELCGSFFTKYTPAIDIWSIGCIFAEVLTGKPLFPGKNVV

HQLDLMTDLLGTPSMDTISRVRNEKARRYLSSMRKKDPVPFSKKFPNADPLGLKLLEKLL

AFDPKDRPTAEEALTDPYFKSLSKPDREPSCQPIRKVEFDFEHRRMSKDDIRELIFQEIL

EYHPQLLKNYIDGTEKTTFLYPSAVDQFKKQFSHLEESDGSGPVVPTERKHASLPRSTTV

HSTPIPAKEQPLVASSRGRPIANEPCKPWAPGNVPGASQTAHVAQAGRAVGVGSVPPYES

GSGKYPYDATSRPAVSSGYPPQQKIPQTYGYHHHHQTPGAGQSSQAMGGYACGYTKGTTP

PPAAAQDMRASPYHHRSAGTKNDPLNRLAAESDIYTRSLNGIVAAAASAGTGAHRKVGAV

PFGMSGMY*

>Bd3-1MPK20-3 Brdisv1Bd3-1_r1005298m.p

MDFFTAYDDANRYKILEVIGKGSYGLVCSANDLQTGEKVAIKKIHNIFEHISDAARILRE

IKLLRLLRHPDVVEIKHILLPPSKKDFKDIYVVFELMESDLHQVIKANDDLTREHYQFFL

YQMLRALKYMHTANVYHRDLKPKNVLANANCKLKICDFGLARVAFSDAPTTVFWTDYVAT

RWYRAPELCGSFYSKYTPAIDIWSIGCIFAEVLIGKPLFPGKNVVHQLDLITDILGTPSL

DAISRVRNDKARKYLTCMRKKQPASFSQKFPKADPLALQLLRRLLAFDPKDRPSAEEALA

DPYFNGLAKVEREPSCQPIPKIEFEFEGRRVTKEDIKELIFEEILEYHPQLLKEHIIGKE

RPNFVHLSAVDQFKKHFTQLEENDNETGAAVSLQRKHSSLPRQAFNHR*

>Bd3-1MPK20-4 Brdisv1Bd3-1_r1015662m.p

MRVGSRGGGGGRGRTMQTTEQQRKKGSSEMDFFSEYGDANRYKIQEVIGKGSYGVVCSAI

DQHTGDKVAIKKIHNIFEHLSDAARILREIKLLRLLRHPDIVEIRHIMLPPSRRDFKDIY

VVFELMDTDLHQVIKANDDLTKEHHQFFLYQMLRALKYIHTANVYHRDLKPKNILANANC

KLKICDFGLARVAFNDTPTTVFWTDYVATRWYRAPELCGSFFTKYSRAIDIWSIGCIFAE

ILTGKPLFPGKNVVHQLDLMTDLLGTPSLDTVSRIRNEKARRYLSSMRKKQSVSFSERFP

KADPAALKLLQRLLAFDPKDRPTAEEALADPYFKGLGKVEREPSCQPITKMEFEFERKNV

TKADVKELIFREILEYHPQLLKDYMNGTEKTNFLYPSAVDNFRRQFANLEENGGKGGAVV

PPDRKHVSLPRNTTVHSTPIPPKDQKYSQVPQRIPTGRPGRVVGPVIPFENSSTMDPYSQ

RRGARNPVLPAAATNLSAYAYRNSDNSERELQQELEKDRMQSHPMQRFMDAKMVSPDLRS

TSYYMPKGVPKADVAERSALQSNMMQGIAPFNGIATVGGAFNKVSAVQYGVSRMY*

>Bd3-1MPK20-5 Brdisv1Bd3-1_r1012412m.p

MPEANAAARGGGGGGDARAAEQRSNNKNSEEMGFFSEYVDASRYKILEIIGKGSYGVVCS

AIDQETGDKVAIKKIQNIFEHLSDAARILREIKLLRLLRHPDIVQIKHIMLPPSRRDFRD

IYVVFELMDTDLHQVIKANDDLTKEHYQFFLYQMLRALKYIHTANVYHRDLKPKNILANA

NCKLKICDFGLARVAFNDTPTTVFWTDYVATRWYRAPELCGSFFTKYSPAIDTWSIGCIF

AEILTGKPLFPGKNVVHQLDLMTDLLGTPSTETISRIRNDKARKYLSSMRRKQPIPFSEK

FPNADPSALKLLERLLAFDPKDRPTAEEALAHPYFKRLARVEREPSCQQPISKTEFEFER

RKFTKEDIKELIFREILEYHPKLLKDYMNGSEKTSFLYPSAVDNFRRQFANLEIDGGRSG

AADRKHFSLPRTTTVHSAPILPTNGPTSQVPQRIPTARAGRVVSSAMQTDNPSVSDRHNG

RRVARDPAVPPAAAYHLKSDYSDRQHQQEFEKDRVRPDRQRQEELEKDRMQYRPGHHSMD

AKVAPEISPYMRSSPYYIPPFNGIAAVASGYSKVAAVTRMY*

>Bd3-1MPK21-1 Brdisv1Bd3-1_r1012338m.p

MDPGKKTSESEFFTEYGEANRYKVSEVIGKGSYGVVAAAVDTQTGERCAIKKINDVFDHV

SDATRILREIKLLRLLRHPDIVEIKHIMLPPSRREFRDIYVVFELMESDLHQVIKANDDL

TPEHHQFFLYQLLRGMKYIHTANVFHRDLKPKNILANADCKLKICDFGLARVSFNDGAPS

AIFWTDYVATRWYRAPELCGSFFSKYTPAIDIWSVGCIFAEMLTGKPLFPGKNVVHQLDL

MTDLLGTPSAESLSKIRNEKARRYLSNMRKKPKVPLTKKFPGIDPMALHLLERLLAFDPK

DRPSADEALTDPYFTGLANSEREPITQPISKLEFEFEKRKLAKDDVRELIYREILEYHPH

MLQEYLRGGGDQMSFMFPSGVDRFKRQFAHLEEGGAKGEKSSPQLRQNASLPRERVIGNK

HGDGDYNMKLNVGEKPELASVSDGISKPLMSARSLLKSETMSASKCIGEIKNKDEDSLSE

CVEGTDDDVSQKIAQLKT*

>Bd3-1MPK21-2 Brdisv1Bd3-1_r1015518m.p

MESDLHQVIKANDDLTPEHHQFFFYQLLRGMKYIHAANVFHRDLKPRNILANADCKLKIC

DFGLARVSFNDTPSAIFWTDYVATRWYRAPELCGSFFSKYTPAIDIWSIGCIFAEMLSGR

PLFPGKNVVHQLDLMTDLLGTPSAESLSRIRNEKARRYLGNMRKKHPVPFSQKFPGVDPM

ALDLLERLLAFDPKDRPTAAEALADPYFTGLANSDREPTTQPISKLEFEFERRKLARDDV

RELIYREILEYHPQMLHEYHHGGDQANFVYPSGVDRFKRQFVHLEEGVTKGEKTSPQLRQ

HASLPRERIIGIGDELGRPNADYCIKLHVGEEPGHTSVTDGLSKPLLNARNFLKSESISA

SQCVVIKEKREKDEESMSEYMHEASDATPK*

>Bd2-3MPK3 Brdisv1Bd2-31009829m.p

MDGAPVAEFRPTMTHGGRFLLYNIFGNQFEITAKYQPPIMPIGRGAYGIVCSVMNFETRE

MVAIKKIANAFDNNMDAKRTLREIKLLRHLDHENIVGLRDVIPPAIPQSFNDVYIATELM

DTDLHHIIRSNQELSEEHCQYFLYQLLRGLKYIHSANVIHRDLKPSNLLLNANCDLKICD

FGLARPSSESDMMTEYVVTRWYRAPELLLNSPDYSAAIDVWSVGCIFMELINRAPLFPGR

DHMHQMRLITEVIGTPTDDDLGFIRNEDARRYMRPLPQFPRRPFPAQFPRVQPAALDLIE

RMLAFNPLQRITVEEALEHPYLERLHDIADEPICTDPFSFDFEQHPLTEDQMKQLIFNEA

LELNPNFRY*

>Bd2-3MPK4 Brdisv1Bd2-31032148m.p

MDTSGGGGGGAGGAAQIQGMATHGGRYVLYNVYGNLFEVASKYAPPIRPIGRGAYGIVCA

AVSSDTGEEVAIKKIGNAFDNHIDAKRTLREIKLLRHMDHENIIAIKDIIRPPRRDDFKD

VYIVTELMDTDLHQIIRSNQPLTDDHCQYFLYQLLRGLKYVHSANVLHRDLKPSNLFLNA

NCDLKIADFGLARTTSETDLMTEYVVTRWYRAPELLLNCSQYTAAIDVWSVGCILGEIIT

RQPLFPGRDYIQQLKLITELIGSPDDSSLGFLRSDNARRYMKQLPQYPRQDFRLRFRNMS

DGAVDLLERMLVFDPSRRITVDEALHHPYLASLHDINEEPTCPAPFSFDFEQPSFTEEHM

KELIWRETLAFNPDPPY*

>Bd2-3MPK6 Brdisv1Bd2-31007253m.p

MDGGAQPPDAEMADAGAAGPGAGAGAAGTMENIQATLSHGGRFIQYNIFGNVFEVTVKYK

PPILPIGKGAYGIVCSALNSETGEQVAIKKIANAFDNKIDAKRTLREIKLLRHMDHENIV

AIRDIIPPAQRNSFNDVYIAYELMDTDLHQIIRSNQALSEEHCQYFLYQILRGLKYIHSA

NVLHRDLKPSNLLLNANCDLKICDFGLARTTSETDFMTEYVVTRWYRAPELLLNSSEYTA

AIDVWSVGCIFMELMDRKPLFPGRDHVHQLRLLMELIGTPNEADLDFVNENARRYIRQLP

RHARQSFSEKFPHVHPSAIDLVEKMLTFDPRQRITVEGALAHPYLASLHDISDEPVCTMP

FSFDFEQHALSEEQMKDLIHQEALAFNPDYQ*

>Bd2-3MPK7-2 Brdisv1Bd2-31024751m.p

MKLSPISASFGAMGSLPQKLDELLSTGHWALSGVVMDEIEELTADLHILCNLLLKLSNVH

DPPPTVRFWMKEVRELSYDVEDCADQFVIANDRAVMRRAKIHRATIRTIIITRLKISRLP

KRRKWRLWMTDKVAEFRTRVQDATQRYWRYKFDDCASNPGYASVGHEFATVYAQPGDLVG

IEGPMDELEQWLTDGEEQLKVISIVGLGGVGKTTLAQKLWATLKGQFLCRAFVRTAQKPD

MRGILRNILLQVRPHQLPNRGEMHHLIHDLREYLQDKRYFVIIDDLWAASVWDVVSRAFP

EGNCCSRIVTTTEIMEVALACCDYCPKHICKLETLSDDDSEKLLLERIVVSGNQIPQQLD

DVLPQIMRNCGGLPLAIIIVASLLVSQPEKLEQWGHTQNSFGSIFGTNPTMEGFIRQILS

ISFNSLPYYLKTCLLYLSSYPEGCLFLKDDLVKLWVAEGFIHAKEWEDMEDLAGRYFDEL

VNVGLIQVMDINYNYKLPSYSVHHMVLDFITCKSIEENFITVVDYSETTTPLTDKVRRLS

LHFGSATHATTPASARLSNVRSLFYFGLFNCMPSFMVFKLLRVLILNFWGDPGNTSFNLT

GICELVWLRYLQVSCNVTVKLPDQIESMGHLETLEIKAKVYAVPLDIVRLSSLSHLHLRG

GTIPNGIGCMRSLRTLKYFDLVNKSVDNLRGLGELANLRDLHLTYSSSLPSEHLKRNLIA

LASSLGKLCNLKSLTLASETAATVVPFDGSISMSSTPAFLERLELLPSICIFSRLPKWIF

QFQKLCILKVAVRELLANDIDSITGLPSLTVLSLCVHTAPGQRIVFNDGAFPVLKYFKFR

CGVLSMSFMAGGMPHLRRLKLGFNTHVGEKYGNMLAGIEHLLNLQDIAGKIGVATESDRK

AAESAIKGAISKHPQSPMLNVQWVDLVEEEYHASEKQHGRQEKGLSGENHAVLEKAKDTK

KHADSGVSQLPDLPSTVSSSRLKSENIAQASKHHVLESMLCDESAQPESLEVSVLEDITN

NFSDDQEISKNRFVVVYKGELQNGSVVVVKRFTIAIDYNLFLDAVDCLMSVKHNNIVRFL

GYCANSQENAAKEGSKSVSVVTQERLCCFEYLRNGNLQMHLTDESCGFDWQMWYQIIEGI

CQGVHYLHNKSITPLDLNPANIMFDDKMVPKIVHYAYTRFLGEATSPVLTNIGDSLAYMA

PEYFGFGEITSKSDIYSLGVIIMQIVTGENKKDFPSIQSVLESWRSRLESDGSQGHTSLE

TCYLQVNLCYLIGIRCIEHDPGKRPNTRDIIDSLDTLEIMECLVKHDVGNSSLEQMLVCS

KVEEPKALPAPPSVSCKLKMAMMVDPPNGTGNHGKHYYTMWQTMFEIDTKYMPIKPIGRG

AYGIVCSSTNQENNEKVAIKKINNVFDNRVDALRTLRELKLLRHLRHENVIALKDIMMPV

HRRSFKDVYLVSELMDTDLHQIIKSSQPLSNDHCQYFLFQLLRGLKYLHSAGILHRDLKP

GNLLVNANCDLKICDFGLARTNNTKGQFMTEYVVTRWYRAPELLLCCDNYGTSIDVWSVG

CIFAELLGRKPIFPGTECLNQLKLIVNVLGTMSENDLEFIDNPKARKYIKSLPYTPGTPL

TSMYPQAHPLAIDLLQKMLVFDPSKRISVTEALEHPYMSPLYDPSANPPAQVPIDLDIDE

NLGVEMIREMLWQEMLQYHPEAARMVNM*

>Bd2-3MPK11 Brdisv1Bd2-31030457m.p

MRMEGGGAGPAAAAAAGGAHGLGEAQIKGTLTHGGRYVQYNVYGNLFEVSAKYVPPIRPV

GRGACGIICAAINVQTREEVAIKKIGNAFDNQIDAKRTLREVKLLRHMNHENVISIKDII

RPPRRENFNDVYIVYELMDTDLHHLLRSNQPLTDDHCQYFLYQVLRGLKYVHSANVLHRD

LRPSNLLLNAKCDLKIGDFGLARTTTETDFMMEYVVTRWYRAPELLLNCSEYTGAIDMWS

VGCILGEIATREPLFPGKDYVHQLRLITELLGSPDDTSLGFLRSDNARRYVRSLPQYPKQ

QFRSRFPNMSSGAMDLLERMLVFDPNKRITVDEALCHPYLASLHEINDEPVCPAPFSFDF

EQPSFTEEDIKELIWRESVKFNPEPIH*

>Bd2-3MPK14 Brdisv1Bd2-31028513m.p

MAMLVDPPNGMGNQGKHYYSMWQTLFEIDTKYVPIKPIGRGAYGIVCSSINRETNEKVAI

KKIHNVFDNRVDALRTLRELKLLRHLRHENVISLKDIMMPVQRRSFKDVYLVYELMDTDL

HQIIKSPQGLSNDHCQYFLFQLLRGLKYLHSAEILHRDLKPGNLLVNANCDLKICDFGLA

RTNSSKGQFMTEYVVTRWYRAPELLLCCDNYGTSIDVWSVGCIFAELLGRKPIFPGTECL

NQLKLIVNVLGTMSESDLEFIDNPKARRYIKTLPYTPGVPLASMYPHAHPLAIDLLQKML

IFDPTKRISVTEALEHPYMSPLYDPSANPPAQVPIDLDLDENISADMIREMMWQEMLHYH

PEAATAISM*

>Bd2-3MPK16 Brdisv1Bd2-31016926m.p

MDFFTEYGEGNRYKIEEVIGKGSYGVVCSALDTHTGDKVAIKKINDIFEHVSDATRILRE

IKLLRLLRHPDIVEIKHILLPPSRREFKDIYVVFELMESDLHQVIKANDDLTPEHYQFFL

YQLLRGLKYIHTANVFHRDLKPKNILANADCKLKICDFGLARVAISDTPTAIFWTDYIAT

RWYRAPELCGSFFSKYTPAIDIWSIGWIFAELLTGKPLFPGKNVVHQLDIITDLLGTPSA

ETIARIRNEKARRYLSSMRRKKTVPFTQKFPNADPLALNLLERMLAFDPKDRPSAEEALA

DPYFKNIASVDREPSAQPITKLEFEFERRRITKDDIRELIYREILEYHPNMLREFLEGAE

PTGFMYPSAVDHFKKQFTFLEEHYAKGSTAAPPERQHNSLPRPSVIYSDNRPQNAANITD

DLSRCAIRDNTQKAQKDSASVGANRIPHGAAAAAARPGKVVGSVLRYGNCSTSGTEQYEQ

RRVVRSPGIAPNGVTSGSSYPRRNNTCKSETGEAERIDTNQGGPPKPYVANKLPATVDGR

NGHW*

>Bd2-3MPK17 Brdisv1Bd2-31005374m.p

MVGGGGIVAGFRRLFHRRTPSGSGPSSNQSSAGEESSDIDAVVEDLDLVGLRAIRVPKRK

MPLPVESHKKNILEKEFFTEYGEASQYQIQEVIGKGSYGVVAAAVDTRTGERVAIKKIND

VFEHVSDATRILREVKLLRLLRHPDVVEIKHIMLPPSRREFQDIYVVFELMESDLHQVIR

ANDDLTPEHYQFFLYQLLRALKYIHAANVFHRDLKPKNILANSDCKLKICDFGLARVSFN

DAPTAIFWTDYVATRWYRAPELCGSFFSKYTPAIDIWSIGCIFAELLTGRPLFPGKNVVH

QLDIITDLLGTPSSETLSRIRNEKARRYLSCMRKKHPVPLTQKFPNVDPLALRLLGRLLA

FDPKDRPSAEEALADPYFAVLANVEREPSRNPISKLEFEFERRKVTKDDVRELIYREILE

YHPQMLQEYMQGGEQLSFLYPSGVDRFKRQFAHLEENYSKGERGSPLQRKHASLPRQRVG

ASNDNNEQHASDQERGADSIAPATGNPPGSQDAGQQHVSGGQNGVGSTNISPRSYQKSAS

ISASKCVVVNANKNPEYDDDISEEMEGAVDGLSEKVSRMHP*

>Bd2-3MPK20-1 Brdisv1Bd2-31018261m.p

MQQDQRKKSSAEAEFFTEYGDASRYKIQEIVGKGSYGVVCSAIDVHTGEKVAIKKIHDIF

EHISDAARILREIKLLRLLRHPDIVEIKHIMLPPSRRDFKDIYVVFELMESDLHQVIKAN

DDLTKEHYQFFLYQLLRALKYIHTANVYHRDLKPKNILANSNCKLKICDFGLARVAFNDT

PTTIFWTDYVATRWYRAPELCGSFFSKYTPAIDVWSIGCIFAEVLTGKPLFPGKNVVHQL

DLMTDLLGTPSMDTISRVRNEKARRYLSSMRKKEPIPFSQKFPNADPLALDLLQRLLAFD

PKDRPTAGEALSHPYFKGLAKVEREPSCQPITKMEFEFERRRATKEDIRELIFREILEYH

PQLLKDYINGTERTTFLYPSAVDQFRKQFAHLEENSESGPVVPMDRKHTSLPRSTIVHSA

PIHAKEQPRIGPSRDRPLTDESYKNPRDSEKYSGNVPRSSHHSQAPQRVPTARPGRVVGP

VLPYENVGTSHPYDPRRVAMNSGYPPQQQIPQTYGYYQTTGKSACSEPSQAERYTLHQQA

YACANSSTAPDVALDMRAPPFHQSAGPKSGSSDRLTAETNLYTRSLNGIVATTSGVAASA

HRKGSVVPYGMSQMY*

>Bd2-3MPK20-2 Brdisv1Bd2-31014393m.p

MHQPQQQGQRNKSTDVDFFSEYGDANRYRIQEVIGKGSYGVVCSAMDMQTRQKVAIKKIH

NIFDHVSDAARILREIKLLRLLRHPDVVEIKHIMLPPSRKDFKDIYVVFELMESDLHQVI

KANDDLTKEHYQFFLYQLLRALKYIHTASVYHRDLKPKNILANSNCKLKICDFGLARVAF

NDTPTTVFWTDYVATRWYRAPELCGSFFTKYTPAIDIWSIGCIFAEVLTGKPLFPGKNVV

HQLDLMTDLLGTPSMDTISRVRNEKARRYLSSMRKKDPVPFSKKFPNADPLGLKLLEKLL

AFDPKDRPTAEEALTDPYFKSLSKPDREPSCQPIRKVEFDFEHRRMSKDDIRELIFQEIL

EYHPQLLKNYIDGTEKTTFLYPSAVDQFKKQFSHLEESDGSGPVVPTERKHASLPRSTTV

HSTPIPAKEQPLVASSRGRPIANEPCKPWAPGNVPGASQTAHVAQAGRAVGVGSVPPYES

GSGKYPYDATSRPAVSSGYPPQQKIPQTYGYHHHHQTPGAGQSSQAMGGYACGYTKGTTP

PPAAAQDMRASPYHHRSAGTKNDPLNRLAAESDIYTRSLNGIVAAAASAGTGAHRKVGAV

PFGMSGMY*

>Bd2-3MPK20-3 Brdisv1Bd2-31042613m.p

MQHGDLHKKSAAEMDFFTAYDDANRYKILEVIGKGSYGLVCSANDLQTGEKVAIKKIHNI

FEHISDAARILREIKLLRLLRHPDVVEIKHILLPPSKKDFKDIYVVFELMESDLHQVIKA

NDDLTREHYQFFLYQMLRALKYMHTANVYHRDLKPKNVLANANCKLKICDFGLARVAFSD

APTTVFWTDYVATRWYRAPELCGSFYSK

>Bd2-3MPK20-4 Brdisv1Bd2-31018474m.p

MQTTEQQRKKGSSEMDFFSEYGDANRYKIQEVIGKGSYGVVCSAIDQHTGDKVAIKKIHN

IFEHLSDAARILREIKLLRLLRHPDIVEIRHIMLPPSRRDFKDIYVVFELMDTDLHQVIK

ANDDLTKEHHQFFLYQMLRALKYIHTANVYHRDLKPKNILANANCKLKICDFGLARVAFN

DTPTTVFWTDYVATRWYRAPELCGSFFTKYSRAIDIWSIGCIFAEILTGKPLFPGKNVVH

QLDLMTDLLGTPSLDTVSRIRNEKARRYLSSMRKKQSVSFSERFPKADPAALKLLQRLLA

FDPKDRPTAEEALADPYFKGLGKVEREPSCQPITKMEFEFERKNVTKADVKELIFREILE

YHPQLLKDYMNGTEKTNFLYPSAVDNFRRQFANLEENGGKGGAVVPPDRKHVSLPRNTTV

HSTPIPPKDQKYSQVPQRIPTGRPGRVVGPVIPFENSSTMDPYSQRRVARNPVLPAAATN

LSAYAYRNSDNSERELQQELEKDRMQYHPMQRFMDAKMVSPDLRSTSYYMPKGVPKADVA

ERSALQSNMMQGIAPFNGIATVGGAFNKVSAVQYGVSRMY*

>Bd2-3MPK20-5 Brdisv1Bd2-31014547m.p

MFSDIFSREGVAFSRVALRQELLSDQWFTTAGGKGQCYLVPPGTTVFLVNSEEMGFFSEY

VDASRYKILEIIGKGSYGVVCSAIDQETGDKVAIKKIQNIFEHLSDAARILREIKLLRLL

RHPDIVQIKHIMLPPSRRDFRDIYVVFELMDTDLHQVIKANDDLTKEHYQFFLYQMLRAL

KYIHTANVYHRDLKPKNILANANCKLKICDFGLARVAFNDTPTTVFWTDYVATRWYRAPE

LCGSFFTKYSPAIDTWSIGCIFAEILTGKPLFPGKNVVHQLDLMTDLLGIPSTETISRIR

NDKARKYLSSMRRKQPIPFSEKFPNADPSALKLLERLLAFDPKDRPTAEEALAHPYFKRL

ARVEREPSCQQPISKTEFEFERRKFTKEDIKELIFREILEYHPKLLKDYMNGSEKTSFLY

PSAVDNFRRQFANLEIDGGRSGAADRKHFSLPRTTTVHSAPILPTNGPTSQVPQRIPTAR

PGRVVSSAMQTDNPSVSDRHNGRRVARDPAVPPAAAYHLKSDYSDRQHQQEFEKDRVRPD

RQRQEELEKDRMQYRPGHHSMDAKVAPEISPYMRSSPYYIPPFNGIAAVASGYSKVAAVT

RMY*

>Bd2-3MPK21-1 Brdisv1Bd2-31014439m.p

MGGLIRWLRHHRSRRVSSSSSSHLPSNTTSSSSTSDLRAHSLPQHQGDHHGEVVVEWEDA

AEGPDSDPEEYIVVVLGDDEQGVVAARAPVRTKPPRVMDPGKKTSESEFFTEYGEANRYK

VSEVIGKGSYGVVAAAVDTQTGERCAIKKINDVFDHVSDATRILREIKLLRLLRHPDIVE

IKHIMLPPSRREFRDIYVVFELMESDLHQVIKANDDLTPEHHQFFLYQLLRGMKYIHTAN

VFHRDLKPKNILANADCKLKICDFGLARVSFNDGAPSAIFWTDYVATRWYRAPELCGSFF

SKYTPAIDIWSVGCIFAEMLTGKPLFPGKNVVHQLDLMTDLLGTPSAESLSKIRNEKARR

YLSNMRKKPKVPLTKKFPGIDPMALHLLERLLAFDPKDRPSADEALTDPYFTGLANSERE

PITQPISKLEFEFEKRKLAKDDVRELIYREILEYHPHMLQEYLRGGGDQMSFMFPSGVDR

FKRQFAHLEEGGAKGEKSSPQLRQNASLPRERVIGNKHGDGDYNMKLNVGEKPELASVSD

GISKPLMSARSLLKSETMSASKCIGEIKNKDEDSLSECVEGTDDDVSQKIAQLKT*

>Adi-10MPK3 Brdisv1Adi-101031639m.p

MDGAPVAEFRPTMTHGGRFLLYNIFGNQFEITAKYQPPIMPIGRGAYGIVWDVLILARHS

VMNFETREMVAIKKIANAFDNNMDAKRTLREIKLLRHLDHENIVGLRDVIPPAIPQSFND

VYIATELMDTDLHHIIRSNQELSEEHCQYFLYQLLRGLKYIHSANVIHRDLKPSNLLLNA

NCDLKICDFGLARPSSESDMMTEYVVTRWYRAPELLLNSPDYSAAIDVWSVGCIFMELIN

RAPLFPGRDHMHQMRLITEVIGTPTDDDLGFIRNEDARRYMRHLPQFPRRPFPAQFPRVQ

PAALDLIERMLAFNPLQRITVEEALEHPYLERLHDIADEPICTDPFSFDFEQHPLTEDQM

KQLIFNEALELNPNFRY*

>Adi-10MPK4 Brdisv1Adi-101037043m.p

AAVSSDTGEEVAIKKIGNAFDNHIDAKRTLREIKLLRHMDHENIIAIKDIIRPPRRDDFK

DVYIVTELMDTDLHQIIRSNQPLTDDHCQYFLYQLLRGLKYVHSANVLHRDLKPSNLFLN

ANCDLKIADFGLARTTSETDLMTEYVVTRWYRAPELLLNCSQYTAAIDVWSVGCILGEII

TRQPLFPGRDYIQQLKLITELIGSPDDSSLGFLRSDNARRYMKQLPQYPRQDFRLRFRNM

SDGAVDLLERMLVFDPSRRITVDEALHHPYLASLHDINEEPTCPAPFSFDFEQPSFTEEH

MKELIWRETLAFNPDPPY*

>Adi-10MPK6 Brdisv1Adi-101005339m.p

MDGGAQPPDAEMADAGAAVPGAGAGAAGTMENIQATLSHGGRFIQYNIFGNVFEVTVKYK

PPILPIGKGAYGIVCSALNSETGEQVAIKKIANAFDNKIDAKRTLREIKLLRHMDHENIV

AIRDIIPPAQRNSFNDVYIAYELMDTDLHQIIRSNQALSEEHCQYFLYQILRGLKYIHSA

NVLHRDLKPSNLLLNANCDLKICDFGLARTTSETDFMTEYVVTRWYRAPELLLNSSEYTA

AIDVWSVGCIFMELMDRKPLFPGRDHVHQLRLLMELIGTPNEADLDFVNENARRYIRQLP

RHARQSFSEKFPHVHPSAIDLVEKMLTFDPRQRITVEGALAHPYLASLHDISDEPVCTMP

FSFDFEQHALSEEQMKDLIHQEALAFNPDYQ*

>Adi-10MPK7-1 Brdisv1Adi-101018783m.p

MPSRFPFLSPGCFIRLLSGSASSDLLLLLPRRGQSAPRARVLLKMAMMVDPPNGTGNHGK

HYYTMWQTMFEIDTKYVPIKPIGRGAYGIVCSSTNQENNEKVAIKKINNVFDNRVDALRT

LRELKLLRHLRHENVIALKDIMMPVHRRSFKDVYLVSELMDTDLHQIIKSSQPLSNDHCQ

YFLFQLLRGLKYLHSAGILHRDLKPGNLLVNANCDLKICDFGLARTNNTKGQFMTEYVVT

RWYRAPELLLCCDNYGTSIDVWSVGCIFAELLGRKPIFPGTECLNQLKLIVNVLGTMSEN

DLEFIDNPKARKYIKSLPYTPGTPLTSMYPQAHPLAIDLLQKMLVFDPSKRISVTEALEH

PYMSPLYDPSANPPAQVPIDLDIDENLGVEMIREMLWQGMLQYHPEAARMVNM*

>Adi-10MPK11 Brdisv1Adi-101023173m.p

MRMEGGGAGPAAAAAAGGAHGLGEAQIKGTLTHGGRYVQYNVYGNLFEVSAKYVPPIRPV

GRGACGIICAAINVQTREEVAIKKIGNAFDNQIDAKRTLREVKLLRHMNHENVISIKDII

RPPRRENFNDVYIVYELMDTDLPHLLRSNQPLTDDHCQYFLYQVLRGLKYVHSANVLHRD

LRPSNLLLNAKCDLKIGDFGLARTTTETDFMMEYVVTRWYRAPELLLNCSEYTGAIDMWS

VGCILGEIATREPLFPGKDYVHQLRLITELLGSPDDTSLGFLRSDNARRYVRSLPQYPKQ

QFRSRFPNMSSGAMDLLERMLVFDPNKRITVDEALCHPYLASLHEINDEPVCPAPFSFDF

EQPSFTEEDIKELIWRESVKFNPEPIH*

>Adi-10MPK14 Brdisv1Adi-101031400m.p

MAMLVDPPNGMGNQGKHYYSMWQTLFEIDTKYVPIKPIGRGAYGIVCSSINRETNEKVAI

KKIHNVFDNRVDALRTLRELKLLRHLRHENVISLKDIMMPVQRRSFKDVYLVYELMDTDL

HQIIKSPQGLSNDHCQYFLFQLLRGLKYLHSAEILHRDLKPGNLLVNANCDLKICDFGLA

RTNSSKGQFMTEYVVTRWYRAPELLLCCDNYGTSIDVWSVGCIFAELLGRKPIFPGTECL

NQLKLIVNVLGTMSESDLEFIDNPKARRYIKTLPYTPGVPLASMYPHAHPLAIDLLQKML

IFDPTKRISVTEALEHPYMSPLYDPSANPPAQVPIDLDLDENISADMIREMMWQEMLHYH

PEAATAISM*

>Adi-10MPK16 Brdisv1Adi-101033836m.p

MDFFTEYGEGNRYKIEEVIGKGSYGVVCSALDTHTGDKVAIKKINDIFEHVSDATRILRE

IKLLRLLRHPDIVEIKHILLPPSRREFKDIYVVFELMESDLHQVIKANDDLTPEHYQFFL

YQLLRGLKYIHTANVFHRDLKPKNILANADCKLKICDFGLARVAISDPPPAIFWTDYIAT

RWYRAPELCGSFFSKYTPAIDIWSIGWIFAELLTGKPLFPGKNVVHQLDIITDLLGTPSA

ETIARIRNEKARRYLSSMRRKKTVPFTQKFPNADPLALNLLERMLAFDPKDRPSAEEALA

DPYFKNIASVDREPSAQPITKLEFEFERRRITKDDIRELIYREILEYHPNMLREFLEGAE

PTGFMYPSAVDHFKKQFTFLEEHYAKGSTAAPPERQHNSLPRPSVIYSDNRPQNAANITD

DLSRCAIRDNTQKAQKDSASVGANRIPHGAAAAAARPGKVVGSVLRYGNCSTSGTEQYEQ

RRVVRSPGIAPNGVTSGSSYPRRNNTCKSETGEAERIDTNQGGPPKPYVANKLPATVDGR

NGHW*

>Adi-10MPK17 Brdisv1Adi-101004044m.p

MPLPSNFLTGNQVRVPPPHITGLVLPFSPALPFFFSSPGSGPSSNQSSAGEESSDIDAVV

EDLDLVGLRAIRVPKRKMPLPVESHKKNILEKEFFTEYGEESQYQIQEVIGKGSYGVVAA

AVDTRTGERVAIKKINDVFEHVSDATRILREVKLLRLLRHPDVVEIKHIMLPPSRREFQD

IYVVFELMESDLHQVIRANDDLTPEHYQFFLYQLLRALKYIHAANVFHRDLKPKNILANS

DCKLKICDFGLARVSFNDAPTAIFWTDYVATRWYRAPELCGSFFSKYTPAIDIWSIGCIF

AELLTGRPLFPGKNVVHQLDIITDLLGTPSSETLSRIRNEKARRYLSCMRKKHPVPLTQK

FPNVDPLALRLLGRLLAFDPKDRPSAEEALADPYFAVLANVEREPSRNPISKLEFEFERR

KVTKDDVRELIYREILEYHPQMLQEYMQGGEQLSFLYPSGGDRFKRQFAHLEENYSKGER

GSPLQRKHASLPRQRVGASNDNNEQHASDQERGADSIAPATGNPPGSQDAGQQHVSGGQN

GVGSTNISPRSYQKSASISASKCVVVNANKNPEYDDDISEEMEGAVDGLSEKVSRMHP*

>Adi-10MPK20-1 Brdisv1Adi-101013839m.p

MQQDQRKKSSAEAEFFTEYGDASRYKIQEIVGKGSYGVVCSAIDVHTGEKVAIKKIHDIF

EHISDAARILREIKLLRLLRHPDIVEIKHIMLPPSRRDFKDIYVVFELMESDLHQVIKAN

DDLTKEHYQFFLYQLLRALKYIHTANVYHRDLKPKNILANSNCKLKICDFGLARVAFNDT

PTTIFWTDYVATRWYRAPELCGSFFSKYTPAIDVWSIGCIFAEVLTGKPLFPGKNVVHQL

DLMTDLLGTPSMDTISRVRNEKARRYLSSMRKKEPIPFSQKFPNADPLALDLLQRLLAFD

PKDRPTAGEALSHPYFKGLAKVEREPSCQPITKMEFEFERRRATKEDIRELIFREILEYH

PQLLKDYINGTERTTFLYPSAVDQFRKQFAHLEENSESGPVVPMDRKHTSLPRSTIVHSA

PIHAKEQPRIGPSRDRPLTDESYKNPRDSEKYSGNVPRSSHHSQAPQRVPTARPGRVVGP

VLPYENVGTSHPYDPRRVAMNSGYPPQQQIPQTYGYYQTTGKSACSEPSQAERYTLHQQA

YACANSSTAPDVALDMRAPPFHQSAGPKSGSSDRLTAETNLYTRSLNGIVATTSGVAASA

HRKGSVVPYGMSQMY*

>Adi-10MPK20-2 Brdisv1Adi-101033960m.p

MQQPQQQGQRNKSTDVDFFSEYGDANRYRIQEVIGKGSYGVVCSAMDMQTRQKVAIKKIH

NIFDHVSDAARILREIKLLRLLRHPDVVEIKHIMLPPSRKDFKDIYVVFELMESDLHQVI

KANDDLTKEHYQFFLYQLLRALKYIHTASVYHRDLKPKNILANSNCKLKICDFGLARVAF

NDTPTTVFWTDYVATRWYRAPELCGSFFTKYTPAIDIWSIGCIFAEVLTGKPLFPGKNVV

HQLDLMTDLLGTPSMDTISRVRNEKARRYLSSMRKKDPVPFSKKFPNADPLGLKLLEKLL

AFDPKDRPTAEEALTDPYFKSLSKPDREPSCQPIRKVEFDFEHRRMSKDDIRELIFQEIL

EYHPQLLKNYIDGTEKTTFLYPSAVDQFKKQFSHLEESDGSGPVVPTERKHASLPRSTTV

HSTPIPAKEQPLVASSRGRPIANEPCKPWAPGNVPGASQTAHVAQAGRAVGVGSVPPYES

GSGKYPYDATSRPAVSSGYPPQQKIPQTYGYHHHHQTPGAGQSSQAMGGYACGYTKGTPP

PPAAAQDMRASPYHHRSAGTKNDPLNRLAAESDIYTRSLNGIVAAAASAGTGAHRKGGAV

PFGMSGMY*

>Adi-10MPK20-3 Brdisv1Adi-101034957m.p

MQHGDLHKKSAAEMDFFTAYDDANRYKILEVIGKGSYGLVCSANDLQTGEKVAIKKIHNI

FEHISDAARILREIKLLRLLRHPDVVEIKHILLPPSKKDFKDIYVVFELMESDLHQVIKA

NDDLTREHYQFFLYQMLRALKYMHTANVYHRDLKPKNVLANANCKLKICDFGLARVAFSD

APTTVFWTDYVATRWYRAPELCGSFYSKVMIVTIDH*

>Adi-10MPK20-4 Brdisv1Adi-101014002m.p

MQTTEQQRKKGSSEMDFFSEYGDANRYKIQEVIGKGSYGVVCSAIDQHTGDKVAIKKIHN

IFEHLSDAARILREIKLLRLLRHPDIVEIRHIMLPPSRRDFKDIYVVFELMDTDLHQVIK

ANDDLTKEHHQFFLYQMLRALKYIHTANVYHRDLKPKNILANANCKLKICDFGLARVAFN

DTPTTVFWTDYVATRWYRAPELCGSFFTKYSRAIDIWSIGCIFAEILTGKPLFPGKNVVH

QLDLMTDLLGTPSLDTVSRIRNEKARRYLSSMRKKQSVSFSERFPKADPAALKLLQRLLA

FDPKDRPTAEEALADPYFKGLGKVERDPPCQPITKMEFEFERKNVTKADVKELIFREILE

YHPQLLKDYMNGTEKTNFLYPSAVDNFRRQFANLEENGGKGGAVVPPDRKHVSLPRNTTV

HSTPIPPKDQKYSQVPQRIPTGRPGRVVGPVIPFENSSTMDPYSQRRVARNPVLPAAATN

LSAYAYRNSDNSERELQQELEKDRMQYHPMQRVMDAKMVSPDLRSTSYYMPKGVPKADVA

ERSALQSNMMQGIAPFNGIATVGGAFNKVSAVQYGVSRMY*

>Adi-10MPK20-5 Brdisv1Adi-101010961m.p

MFSDIFSREGVAFSRVALRQELLSDQWFTTAGGKGQCYLVPPGTTVFLVNSEEMGFFSEY

VDASRYKILEIIGKGSYGVVCSAIDQETGDKVAIKKIQNIFEHLSDAARILREIKLLRLL

RHPDIVQIKHIMLPPSRRDFRDIYVVFELMDTDLHQVIKANDDLTKEHYQFFLYQMLRAL

KYIHTANVYHRDLKPKNILANANCKLKICDFGLARVAFNDTPTTVFWTDYVATRWYRAPE

LCGSFFTKYSPAIDTWSIGCIFAEILTGKPLFPGKNVVHQLDLMTDLLGTPSTETISRIR

NDKARKYLSSMRRKQPIPFSEKFPNADPSALKLLERLLAFDPKDRPTAEEALAHPYFKRL

ARVEREPSCQQPISKTEFEFERRKFTKEDVKELIFREILEYHPKLLKDYMNGSEKTSFLY

PSAVDNFRRQFANLEIDGGRSGAADRKHFSLPRTTTVHSAPILPTNGPTSQVPQRIPTAR

PGRVVSSAMQTDNPSVSDRHNGRRVARDPAVPPAAAYHLKSDYSDRQHQQEFEKDRVRPD

RQRQEELEKDRMQYRPGHHSMDAKVAPEISPYMRSSPYYIPPFNGIAAVASGYSKVAAVT

RMY*

>Adi-10MPK21-1 Brdisv1Adi-101013900m.p

MDPGKKTSESEFFTEYGEANRYKVSEVIGKGSYGVVAAAVDTQTGERCAIKKINDVFDHV

SDATRILREIKLLRLLRHPDIVEIKHIMLPPSRREFRDIYVVFELMESDLHQVIKANDDL

TPEHHQFFLYQLLRGMKYIHTASVFHRDLKPKNILANADCKLKICDFGLARVSFNEGAPP

AIFWTDYVATRWYRAPELCGSFFSKYTPAIDIWSVGCIFAEMLTGKPLFPGKNVVHQLDL

MTDLLGTPSAESLSKIRNEKARRYLSNMRKKPKVPLTKKFPGIDPMALHLLERLLAFDPK

DRPSADEALTDPYFTGLANSEREPITQPISKLEFEFEKRKLAKDDVRELIYREILEYHPH

MLQEYLRGGGDQMSFMFPSGVDRFKRQFAHLEEGGAKGEKSSPQLRQNASLPRERVIGNK

HGDGDYNMKLNVGEKPELASVSDGISKPLMSARSLLKSETMSASKCIGEIKNKDEDSLSE

CVEGTDDDVSQKIAQLKT*

>Adi-10MPK21-2 Brdisv1Adi-101031490m.p

MESDLHQVIKANDDLTPEHHQFFFYQLLRGMKYIHAANVFHRDLKPRNILANADCKLKIC

DFGLARVSFNDTPSAIFWTDYVATRWYRAPELCGSFFSKYTPAIDIWSIGCIFAEMLSGR

PLFPGKNVVHQLDLMTDLLGTPSAESLSRIRNEKARRYLGNMRKKHPVPFSQKFPGVDPM

ALDLLERLLAFDPKDRPTAAEALADPYFTGLANSDREPTTQPISKLEFEFERRKLARDDV

RELIYREILEYHPQMLHEYHHGGDQANFVYPSGVDRFKRQFVHLEEGVTKGEKTSPQLRQ

HASLPRERIIGIGDELGRPNADYCIKLHVGEEPGHTSVTDGLSKPLLNARNFLKSESISA

SQCVVIKEKREKDEESMSEYMHEASDATPK*

>Adi-2MPK3 Brdisv1Adi-21010752m.p

MDGAPVAEFRPTMTHGGRFLLYNIFGNQFEITAKYQPPIMPIGRGAYGIVCSVMNFETRE

MVAIKKIANAFDNNMDAKRTLREIKLLRHLDHENIVGLRDVIPPAIPQSFNDVYIATELM

DTDLHHIIRSNQELSEEHCQYFLYQLLRGLKYIHSANVIHRDLKPSNLLLNANCDLKICD

FGLARPSSESDMMTEYVVTRWYRAPELLLNSTDYSAAIDVWSVGCIFMELINRAPLFPGR

DHMHQMRLITEVIGTPTDDDLGFIRNEDARRYMRHLPQFPRRPFPAQFPRVQPAALDLIE

RMLAFNPLQRITVEEALEHPYLERLHDIADEPICTDPFSFDFEQHPLTEDQMKQLIFNEA

LELNPNFRY*

>Adi-2MPK4 Brdisv1Adi-21035342m.p

MDTSGGGGGGAGGAAQIQGMATHGGRYVLYNVYGNLFEVASKYAPPIRPIGRGAYGIVCA

AVSSDTGEEVAIKKIGNAFDNHIDAKRTLREIKLLRHMDHENIIAIKDIIRPPRRDDFKD

VYIVTELMDTDLHQIIRSNQPLTDDHCQYFLYQLLRGLKYVHSANVLHRDLKPSNLFLNA

NCDLKIADFGLARTTSETDLMTEYVVTRWYRAPELLLNCSQYTAAIDVWSVGCILGEIIT

RQPLFPGRDYIQQLKLITELIGSPDDSSLGFLRSDNARRYMKQLPQYPRQDFRLRFRNMS

DGAVDLLERMLVFDPSRRITVDEALHHPYLASLHDINEEPTCPAPFSFDFEQPSFTEEHM

KELIWRETLAFNPDPPY*

>Adi-2MPK6 Brdisv1Adi-21007714m.p

MDGGAQPPDAEMADAGAAVPGAGAGAAGTMENIQATLSHGGRFIQYNIFGNVFEVTVKYK

PPILPIGKGAYGIVCSALNSETGEQVAIKKIANAFDNKIDAKRTLREIKLLRHMDHENIV

AIRDIIPPAQRNSFNDVYIAYELMDTDLHQIIRSNQALSEEHCQYFLYQILRGLKYIHSA

NVLHRDLKPSNLLLNANCDLKICDFGLARTTSETDFMTEYVVTRWYRAPELLLNSSEYTA

AIDVWSVGCIFMELMDRKPLFPGRDHVHQLRLLMELIGTPNEADLDFVNENARRYIRQLP

RHARQSFSEKFPHVHPSAIDLVEKMLTFDPRQRITVEGALAHPYLASLHDISDEPVCTMP

FSFDFEQHALSEEQMKDLIHQEALAFNPDYQ*

>Adi-2MPK7-1 Brdisv1Adi-21046113m.p

MLKMAMMVDPPNGTGNHGKHYYTMWQTMFEINTKYMPIKPIGRGAYGIVCSSTNQENNEK

VAIKKINNVFDNRVDALRTLRELKLLRHLRHENVIALKDIMMPVHRRSFKDVYLVSELMD

TDLHQIIKSSQPLSNDHCQYFLFQLLRGLKYLHSAGILHRDLKPGNLLVNANCDLKICDF

GLARTNNTKGQFMTEYVVTRWYRAPELLLCCDNYGTSIDVWSVGCIFAELLGRKPIFPGT

ECLNQLKLIVNVLGTMSENDLEFIDNPKARKYIKSLPYTPGTPLTSMYPQAHPLAIDLLQ

KMLVFDPSKRISVTEALEHPYMSPLYDPSANPPAQVPIDLDIDENLGVEMIREMLWQEML

QYHPEAARMVNM*

>Adi-2MPK11 Brdisv1Adi-21033371m.p

MRMEGGGAGPAAAAAGGGAHGLGEAQIKGTLTHGGRYVQYNVYGNLFEVSAKYVPPIRPV

GRGACGIICAAINVQTREEVAIKKIGNAFDNQIDAKRTLREVKLLRHMNHENVISIKDII

RPPRRENFNDVYIVYELMDTDLHHLLRSNQPLTDDHCQYFLYQVLRGLKYVHSANVLHRD

LRPSNLLLNAKCDLKIGDFGLARTTTETDFMMEYVVTRWYRAPELLLNCSEYTGAIDMWS

VGCILGEIATREPLFPGKDYVHQLRLITELLGSPDDTSLGFLRSDNARRYVRSLPQYPKQ

QFRSRFPNMSSGAMDLLERMLVFDPNKRITVDEALCHPYLASLHEINDEPVCPAPFSFDF

EQPSFTEEDIKELIWRESVKFNPEPIH*

>Adi-2MPK14 Brdisv1Adi-21031107m.p

MAMLVDPPNGMGNQGKHYYSMWQTLFEIDTKYVPIKPIGRGAYGIVCSSINRETNEKVAI

KKIHNVFDNRVDALRTLRELKLLRHLRHENVISLKDIMMPVQRRSFKDVYLVYELMDTDL

HQIIKSPQGLSNDHCQYFLFQLLRGLKYLHSAEILHRDLKPGNLLVNANCDLKICDFGLA

RTNSSKGQFMTEYVVTRWYRAPELLLCCDNYGTSIDVWSVGCIFAELLGRKPIFPGTECL

NQLKLIVNVLGTMSESDLEFIDNPKARRYIKTLPYTPGVPLASMYPHAHPLAIDLLQKML

IFDPTKRISVTEALEHPYMSPLYDPSANPPAQVPIDLDLDENISADMIREMMWQEMLHYH

PEAATAISM*

>Adi-2MPK16 Brdisv1Adi-21018516m.p

MDFFTEYGEGNRYKIEEVIGKGSYGVVCSALDTHTGDKVAIKKINDIFEHVSDATRILRE

IKLLRLLRHPDIVEIKHILLPPSRREFKDIYVVFELMESDLHQVIKANDDLTPEHYQFFL

YQLLRGLKYIHTANVFHRDLKPKNILANADCKLKICDFGLARVAISDTPTAIFWTDYIAT

RWYRAPELCGSFFSKYTPAIDIWSIGCIFAELLTGKPLFPGKNVVHQLDIITDLLGTPSA

ETIARIRNEKARRYLSSMRRKKTVPFTQKFPNADPLALNLLERMLAFDPKDRPSAEEALA

DPYFKNIASVDREPSAQPITKLEFEFERRRITKDDIRELIYREILEYHPNMLREFLEGAE

PTGFMYPSAVDHFKKQFTFLEEHYAKGSTAAPPERQHNSLPRPSVIYSDNRPQNAANITD

DLSRCAIRDNTQKAQKDSASVGANRIPHGAAAAAARPGKVVGSVLRYGNCSTSGTEQYEQ

RRVVRSPGIAPNGVTSGSSYPRRNNTCKSETGEAERIDTNQGGPPKPYVANKLPATVDGR

NGHW*

>Adi-2MPK17 Brdisv1Adi-21005644m.p

MVGGGGIVDGFRRLFHRRTPSGSGPSSNQSSAGEESSDIDAVVEDLDLVGLRAIRVPKRK

MPLPVESHKKNILEKEFFTEYGEASQYQIQEVIGKGSYGVVAAAVDTRTGERVAIKKIND

VFEHVSDATRILREVKLLRLLRHPDVVEIKHIMLPPSRREFQDIYVVFELMESDLHQVIR

ANDDLTPEHYQFFLYQLLRALKYIHAANVFHRDLKPKNILANSDCKLKICDFGLARVSFN

DAPTAIFWTDYVATRWYRAPELCGSFFSKYTPAIDIWSIGCIFAELLTGRPLFPGKNVVH

QLDIITDLLGTPSSETLSRIRNEKARRYLSCMRKKHPVPLTQKFPNVDPLALRLLGRLLA

FDPKDRPSAEEALADPYFAVLANVEREPSRNPISKLEFEFERRKVTKDDVRELIYREILE

YHPQMLQEYMQGGEQLSFLYPSGVDRFKRQFAHLEENYSKGERGSPLQRKHASLPRQRVG

ASNDNNDQHASDQERGADSIAPATGNPPGSQDAGQQHVSGGQNGVGSTNISPRSYQKSAS

ISASKCVVVNANKNPEYDDDISEEMEGAVDGLSEKVSRMHP*

>Adi-2MPK20-1 Brdisv1Adi-21020002m.p

MQQDQRKKSSAEAEFFTEYGDASRYKIQEIVGKGSYGVVCSAIDVHTGEKVAIKKIHDIF

EHISDAARILREIKLLRLLRHPDIVEIKHIMLPPSRRDFKDIYVVFELMESDLHQVIKAN

DDLTKEHYQFFLYQLLRALKYIHTANVYHRDLKPKNILANSNCKLKICDFGLARVAFNDT

PTTIFWTDYVATRWYRAPELCGSFFSKYTPAIDVWSIGCIFAEVLTGKPLFPGKNVVHQL

DLMTDLLGTPSMDTISRVRNEKARRYLSSMRKKEPIPFSQKFPNADPLALDLLQRLLAFD

PKDRPTAGEALSHPYFKGLAKVEREPSCQPITKMEFEFERRRATKEDIRELIFREILEYH

PQLLKDYINGTERTTFLYPSAVDQFRKQFAHLEENSESGPVVPMDRKHTSLPRSTIVHSA

PIHAKEQPRIGPSRDRPLTDESYKNPRDSEKYSGNVPRSSHHSQAPQRVPTARPGRVVGP

VLPYENVGTSHPYDPRRVAMNSGYPPQQQIPQTYGYYQTTGKSACSEPSQAERYTLHQQA

YACANSSTAPDVALDMRAPPFHQSAGPKSGSSDRLTAETNLYTRSLNGIVATTSGVAASA

HRKVSVVPYGMSQMY*

>Adi-2MPK20-2 Brdisv1Adi-21015730m.p

MQQPQQQGQRNKLPDGWFCCNVHILNLCRSAMIFLTPPIIMPSTDVDFFSEYGDANRYRI

QEVIGKGSYGVVCSAMDMQTRQKVAIKKIHNIFDHVSDAARILREIKLLRLLRHPDVVEI

KHIMLPPSRKDFKDIYVVFELMESDLHQVIKANDDLTKEHYQFFLYQLLRALKYIHTASV

YHRDLKPKNILANSNCKLKICDFGLARVAFNDTPTTVFWTDYVATRWYRAPELCGSFFTK

YTPAIDIWSIGCIFAEVLTGKPLFPGKNVVHQLDLMTDLLGTPSMDTISRVRNEKARRYL

SSMRKKDPVPFSKKFPNADPLGLKLLEKLLAFDPKDRPTAEEALTDPYFKSLSKPDREPS

CQPIRKVEFDFEHRRMSKDDIRELIFQEILEYHPQLLKNYIDGTEKTTFLYPSAVDQFKK

QFSHLEESDGSGPVVPTERKHASLPRSTTVHSTPIPAKEQPLVASSRGRPIANEPCKPWA

PGNVPGASQTAHVAQAGRAVGVGSVPPYESGSGKYPYDATSRPAVSSGYPPQQKIPQTYG

YHHHHQTPGAGQSSQAMGGYACGYTKGTTPPPAAAQDMRASPYHHRSAGTKNDPLNRLAA

ESDIYTRSLNGIVAAAASAGTGAHRKVGAVPFGMSGMY*

>Adi-2MPK20-3 Brdisv1Adi-21045430m.p

MQHGDLHKKSAAEMDFFTAYDDANRYKILEVIGKGSYGLVCSANDLQTGEKVAIKKIHNI

FEHISDAARILREIKLLRLLRHPDVVEIKHILLPPSKKDFKDIYVVFELMESDLHQVIKA

NDDLTREHYQFFLYQMLRALKYMHTANVYHRDLKPKNVLANANCKLKICDFGLARVAFSD

APTTVFWTDYVATRWYRAPELCGSFYSK

>Adi-2MPK20-4 Brdisv1Adi-21020252m.p

MQTTEQQRKKGSSEMDFFSEYGDANRYKIQEVIGKGSYGVVCSAIDQHTGDKVAIKKIHN

IFEHLSDAARILREIKLLRLLRHPDIVEIRHIMLPPSRRDFKDIYVVFELMDTDLHQVIK

ANDDLTKEHHQFFLYQMLRALKYIHTANVYHRDLKPKNILANANCKLKICDFGLARVAFN

DTPTTVFWTDYVATRWYRAPELCGSFFTKYSRAIDIWSIGCIFAEILTGKPLFPGKNVVH

QLDLMTDLLGTPSLDTVSRIRNEKARRYLSSMRKKQSVSFSERFPKADPAALKLLQRLLA

FDPKDRPTAEEALADPYFKGLGKVEREPSCQPITKMEFEFERKNVTKADVKELIFREILE

YHPQLLKDYMNGTEKTNFLYPSAVDNFRRQFANLEENGGKGGAVVPPDRKHVSLPRNTTV

HSTPIPPKDQKYSQVPQRIPTGRPGRVVGPVIPFENSSTMDPYSQRRVARNPVLPAAATN

LSAYAYRNSDNSERELQQELEKDRMQYHPMQRFMDAKMVSPDLRSTSYYMPKGVPKADVA

ERSALQSNMMQGIAPFNGIATVGGAFNKVSAVQYGVSRMY*

>Adi-2MPK20-5 Brdisv1Adi-21015890m.p

MFSDIFSREGVAFSRVALRQELLSDQWFTTAGGKGQCYLVPPGTTVFLVNSEEMGFFSEY

VDASRYKILEIIGKGSYGVVCSAIDQETGDKVAIKKIQNIFEHLSDAARILREIKLLRLL

RHPDIVQIKHIMLPPSRRDFRDIYVVFELMDTDLHQVIKANDDLTKEHYQFFLYQMLRAL

KYIHTANVYHRDLKPKNILANANCKLKICDFGLARVAFNDTPTTVFWTDYVATRWYRAPE

LCGSFFTKYSPAIDTWSIGCIFAEILTGKPLFPGKNVVHQLDLMTDLLGTPSTETISRIR

NDKARKYLSSMRRKQPIPFSEKFPNADPSALKLLERLLAFDPKDRPTAEEALAHPYFKRL

ARVEREPSCQQPISKTEFEFERRKFTKEDVKELIFREILEYHPKLLKDYMNGSEKTSFLY

PSAVDNFRRQFANLEIDGGRSGAADRKHFSLPRTTTVHSAPILPTNGPTSQVPQRIPTAR

PGRVVSSAMQTDNPSVSDRHNGRRVARDPAVPPAAAYHLKSDYSDRQHQQEFEKDRVRPD

RQRQEELEKDRMQYRPGHHSMDAKVAPEISPYMRSSPYYIPPFNGIAAVASGYSKVAAVT

RMY*

>Adi-2MPK21-1 Brdisv1Adi-21015775m.p

MGGLIRWLRPHRSRRVSSSSSSHLPSNTTSSSSTSDLRAHSLPQHQGDHHGEVVVEWEDA

AEGPDSDPEEYIVVVLGDDEQGVVAARAPVRTKPPRVMDPGKKTSESEFFTEYGEANRYK

VSEVIGKGSYGVVAAAVDTQTGERCAIKKINDVFDHVSDATRILREIKLLRLLRHPDIVE

IKHIMLPPSRREFRDIYVVFELMESDLHQVIKANDDLTPEHHQFFLYQLLRGMKYIHTAS

VFHRDLKPKNILANADCKLKICDFGLARVSFNDGAPSAIFWTDYVATRWYRAPELCGSFF

SKYTPAIDIWSVGCIFAEMLTGKPLFPGKNVVHQLDLMTDLLGTPSAESLSKIRNEKARR

YLSNMRKKPKVPLTKKFPGIDPMALHLLERLLAFDPKDRPSADEALTDPYFTGLANSERE

PITQPISKLEFEFEKRKLAKDDVRELIYREILEYHPHMLQEYLRGGGDQMSFMFPSGVDR

FKRQFAHLEEGGAKGEKSSPQLRQNASLPRERVIGNKHGDGDYNMKLNVGEKPELASVSD

GISKPLMSARSLLKSETMSASKCIGEIKNKDEDSLSECVEGTDDDVSQKIAQLKT*

>Adi-2MPK21-2 Brdisv1Adi-21020093m.p

MARKGSDEAEFFTEYGEANRYEVGEVVGKGSYGVVAAAVDTHTGERVAIKKINDVFEHVS

DATRILREIKLLRLLRHPDIVEIKHIILPPSRREFRDIYIIFELMESDLHQVIKANDDLT

PEHHQFFFYQLLRGMKYIHAANVFHRDLKPRNILANADCKLKICDFGLARVSFNDTPSAI

FWTDYVATRWYRAPELCGSFFSKYTPAIDIWSIGCIFAEMLSGRPLFPGKNVVHQLDLMT

DLLGTPSAESLSRIRNEKARRYLGNMRKKHPVPFSQKFPGVDPMALDLLERLLAFDPKDR

PTAAEALADPYFTGLANSDREPTTQPISKLEFEFERRKLARDDVRELIYREILEYHPQML

HEYHHGGDQANFVYPSGVDRFKRQFVHLEEGVTKGEKTSPQLRQHASLPRERIIGIGDEL

GRPNADYCIKLHVGEEPGHTSVTDGLSKPLLNARNFLKSESISASQCVVIKEKREKDEES

MSEYMHEASDATPK*

>Adi-12MPK3 Brdisv1Adi-121041121m.p

MDGAPVAEFRPTMTHGGRFLLYNIFGNQFEITAKYQPPIMPIGRGAYGIVCSVMNFETRE

MVAIKKIANAFDNNMDAKRTLREIKLLRHLDHENIVGLRDVIPPAIPQSFNDVYIATELM

DTDLHHIIRSNQELSEEHCQYFLYQLLRGLKYIHSANVIHRDLKPSNLLLNANCDLKICD

FGLARPSSESDMMTEYVVTRWYRAPELLLNSTDYSAAIDVWSVGCIFMELINRAPLFPGR

DHMHQMRLITEVIGTPTDDDLGFIRNEDARRYMRHLPQFPRRPFPAQFPRVQPAALDLIE

RMLAFNPLQRITVEEALEHPYLERLHDIADEPICTDPFSFDFEQHPLTEDQMKQLIFNEA

LELNPNFRY*

>Adi-12MPK4 Brdisv1Adi-121032162m.p

MATHGGRYVLYNVYGNLFEVASKYAPPIRPIGRGAYGIVCAAVSSDTGEEVAIKKIGNAF

DNHIDAKRTLREIKLLRHMDHENIIAIKDIIRPPRRDDFKDVYIVTELMDTDLHQIIRSN

QPLTDDHCQYFLYQLLRGLKYVHSANVLHRDLKPSNLFLNANCDLKIADFGLARTTSETD

LMTEYVVTRWYRAPELLLNCSQYTAAIDVWSVGCILGEIITRQPLFPGRDYIQQLKLITE

LIGSPDDSSLGFLRSDNARRYMKQLPQYPRQDFRLRFRNMSDGAVDLLERMLVFDPSRRI

TVDEALHHPYLASLHDINEEPTCPAPFSFDFEQPSFTEEHMKELIWRETLAFNPDPPY*

>Adi-12MPK6 Brdisv1Adi-121007265m.p

MTGDQTLASMTNYGRFSQIETAYWLVSYNKQSVSAFNVPVIVAIRDIIPPAQRNSFNDVY

IAYELMDTDLHQIIRSNQALSEEHCQYFLYQILRGLKYIHSANVLHRDLKPSNLLLNANC

DLKICDFGLARTTSETDFMTEYVVTRWYRAPELLLNSSEYTAAIDVWSVGCIFMELMDRK

PLFPGRDHVHQLRLLMELIGTPNEADLDFVNENARRYIRQLPRHARQSFSEKFPHVHPSA

IDLVEKMLTFDPRQRITVEGALAHPYLASLHDISDEPVCTMPFSFDFEQHALSEEQMKDL

IHQEALAFNPDYQ*

>Adi-12MPK7-1 Brdisv1Adi-121024789m.p

MPSRFPFLSPGCFIRLLSGSASSDLLLLLPRRGQSAPRARVLLKMAMMVDPPNGTGNHGK

HYYTMWQTMFEIDTKYVPIKPIGRGAYGIVCSSTNQENNEKVAIKKINNVFDNRVDALRT

LRELKLLRHLRHENVIALKDIMMPVHRRSFKDVYLVFELMDTDLHQIIKSSQPLSNDHCQ

YFLFQLLRGLKYLHSAGILHRDLKPGNLLVNANCDLKICDFGLARTNNTKGQFMTEYVVT

RLYRAPELLLCCDNYGTSIDVWSVGCIFAELLGRKPIFPRTQCLDQLKLIVNVLGTMSEN

DLEFIDNPKARKYIKSLPYTPGTPLTSMYPQAHPLAIDLLQKMLVFDPSKRISVTEALEH

PYMSPLYDPSANPPAQVPIDLDIDENLGVEMIREMLWQEMLQYHPEAARMVNM*

>Adi-12MPK11 Brdisv1Adi-121030403m.p

MRMEGGGAGPAAAAAAGGAHGLGEAQIKGTLTHGGRYVQYNVYGNLFEGSAKYVPPIRPV

GRGACGIICAAINVQTREEVAIKKIGNAFDNQIDAKRTLREVKLLRHMNHENVISIKDII

RPPRRENFNDVYIVYELMDTDLHHLLRSNQPLTDDHCQYFLYQVLRGLKYVHSANVLHRD

LRPSNLLLNAKCDLKIGDFGLARTTTETDFMMEYVVTRWYRAPELLLNCSEYTGAIDMWS

VGCILGEIATREPLFPGKDYVHQLRLITELLGSPDDTSLGFLRSDNARRYVRSLPQYPKQ

QFRSRFPNMSSGAMDLLERMLVFDPNKRITVDEALCHPYLASLHEINDEPVCPAPFSFDF

EQPSFTEEDIKELIWRESVKFNPEPIH*

>Adi-12MPK14 Brdisv1Adi-121005230m.p

MAMLVDPPNGMGNQGKHYYSMWQTLFEIDTKYVPIKPIGRGAYGIVCSSINRETNEKVAI

KKIHNVFDNRVDALRTLRELKLLRHLRHENVISLKDIMMPVQRRSFKDVYLVYELMDTDL

HQIIKSPQGLSNDHCQYFLFQLLRGLKYLHSAEILHRDLKPGNLLVNANCDLKICDFGLA

RTNSSKGQFMTEYVVTRWYRAPELLLCCDNYGTSIDVWSVGCIFAELLGRKPIFPGTECL

NQLKLIVNVLGTMSESDLEFIDNPKARRYIKTLPYTPGVPLASMYPHAHPLAIDLLQKML

IFDPTKRISVTEALEHPYMSPLYDPSANPPAQVPIDLDLDENISADMIREMMWQEMLHYH

PEAATAISM*

>Adi-12MPK16 Brdisv1Adi-121016891m.p

MDFFTEYGEGNRYKIEEVIGKGSYGVVCSALDTHTGDKVAIKKINDIFEHVSDATRILRE

IKLLRLLRHPDIVEIKHILLPPSRREFKDIYVVFELMESDLHQVIKANDDLTPEHYQFFL

YQLLRGLKYIHTANVFHRDLKPKNILANADCKLKICDFGLARVAISDTPTAIFWTDYIAT

RWYRAPELCGSFFSKYTPAIDIWSIGCIFAELLTGKPLFPGKNVVHQLDIITDLLGTPSA

ETIARIRNEKARRYLSSMRRKKTVPFTQKFPNADPLALNLLERMLAFDPKDRPSAEEALA

DPYFKNIASVDREPSAQPITKLEFEFERRRITKDDIRELIYREILEYHPNMLREFLEGAE

PTGFMYPSAVDHFKKQFTFLEEHYAKGSTAAPPERQHNSLPRPSVIYSDNRPQNAANITD

DLSRCAIRDNTQKAQKDSASVGANRIPHGAAAAAARPGKVVGSVLRYGNCSTSGTEQYEQ

RRVVRSPGIAPNGVTSGSSYPRRNNTCKSETGEAERIDTNQGGPPKPYVANKLPATVDGR

NGHW*

>Adi-12MPK17 Brdisv1Adi-121005354m.p

MVGGGGIVDGSRRLFHRRTPSGSGPSSNQSSAGEESSDIDAVVEDLDLVGLRAIRVPKRK

MPLPVESHKKNILEKEFFTEYGEASQYQIQEVIGKGSYGVVAAAVDTRTGERVAIKKIND

VFEHVSDATRILREVKLLRLLRHPDVVEIKHIMLPPSRREFQDIYVVFELMESDLHQVIR

ANDDLTPEHYQFFLYQLLRALKYIHAANVFHRDLKPKNILANSDCKLKICDFGLARVSFN

DAPTAIFWTDYVATRWYRAPELCGSFFSKYTPAIDIWSIGCIFAELLTGRPLFPGKNVVH

QLDIITDLLGTPSSETLSRIRNEKARRYLSCMRKKHPVPLTQKFPNVDPLALRLLGRLLA

FDPKDRPSAEEALADPYFAVLANVEREPSRNPISKLEFEFERRKVTKDDVRELIYREILE

YHPQMLQEYMQGGEQLSFLYPSGVDRFKRQFAHLEENYSKGERGSPLQRKHASLPRQRVG

ASNDNNEQHASDQERGADSIAPATGNPPGSQDAGQQHVSGGQNGVGSTNISPRSYQKSAS

ISASKCVVVNANKNPEYDDDISEEMEGAVDGLSEKVSRMHP*

>Adi-12MPK20-1 Brdisv1Adi-121018246m.p

MQQDQRKKSSAEAEFFTEYGDASRYKIQEIVGKGSYGVVCSAIDVHTGEKVAIKKIHDIF

EHISDAARILREIKLLRLLRHPDIVEIKHIMLPPSRRDFKDIYVVFELMESDLHQVIKAN

DDLTKEHYQFFLYQLLRALKYIHTANVYHRDLKPKNILANSNCKLKICDFGLARVAFNDT

PTTIFWTDYVATRWYRAPELCGSFFSKYTPAIDVWSIGCIFAEVLTGKPLFPGKNVVHQL

DLMTDLLGTPSMDTISRVRNEKARRYLSSMRKKEPIPFSQKFPNADPLALDLLQRLLAFD

PKDRPTAGEALSHPYFKGLAKVEREPSCQPITKMEFEFERRRATKEDIRELIFREILEYH

PQLLKDYINGTERTTFLYPSAVDQFRKQFAHLEENSESGPVVPMDRKHTSLPRSTIVHSA

PIHAKEQPRIGPSRDRPLTDESYKNPRDSEKYSGNVPRSSHHSQAPQRVPTARPGRVVGP

VLPYENVGTSHPYDPRRVAMNSGYPPQQQIPQTYGYYQTTGKSACSEPSQAERYTLHQQA

YACANSSTAPDVALDMRAPPFHQSAGPKSGSSDRLTAETNLYTRSLNGIVATTSGVAASA

HRKVSVVPYGMSQMY*

>Adi-12MPK20-2 Brdisv1Adi-121014318m.p

MQQPQQQGQRNKLPDGWFCCNVHILNLCRSAMIFLTPPIIMPSTDVDFFSEYGDANRYRI

QEVIGKGSYGVVCSAMDMQTRQKVAIKKIHNIFDHVSDAARILREIKLLRLLRHPDVVEI

KHIMLPPSRKDFKDIYVVFELMESDLHQVIKANDDLTKEHYQFFLYQLLRALKYIHTASV

YHRDLKPKNILANSNCKLKICDFGLARVAFNDTPTTVFWTDYVATRWYRAPELCGSFFTK

YTPAIDIWSIGCIFAEVLTGKPLFPGKNVVHQLDLMTDLLGTPSMDTISRVRNEKARRYL

SSMRKKDPVPFSKKFPNADPLGLKLLEKLLAFDPKDRPTAEEALTDPYFKSLSKPDREPS

CQPIRKVEFDFEHRRMSKDDIRELIFQEILEYHPQLLKNYIDGTEKTTFLYPSAVDQFKK

QFSHLEESDGSGPVVPTERKHASLPRSTTVHSTPIPAKEQPLVASSRGRPIANEPCKPWA

PGNVPGASQTAHVAQAGRAVGVGSVPPYESGSGKYPYDATSRPAVSSGYPPQQKIPQTYG

YHHHHQTPGAGQSSQAMGGYACGYTKGTTPPPAAAQDMRASPYHHRSAGTKNDPLNRLAA

ESDIYTRSLNGIVAAAASAGTGAHRKVGAVPFGMSGMY*

>Adi-12MPK20-3 Brdisv1Adi-121042759m.p

MQHGDLHKKSAAEMDFFTAYDDANRYKILEVIGKGSYGLVCSANDLQTGEKVAIKKIHNI

FEHISDAARILREIKLLRLLRHPDVVEIKHILLPPSKKDFKDIYVVFELMESDLHQVIKA

NDDLTREHYQFFLYQMLRALKYMHTANVYHRDLKPKNVLANANCKLKICDFGLARVAFSD

APTTVFWTDYVATRWYRAPELCGSFYSK

>Adi-12MPK20-4 Brdisv1Adi-121018451m.p

MQTTEQQRKKGSSEMDFFSEYGDANRYKIQEVIGKGSYGVVCSAIDQHTGDKVAIKKIHN

IFEHLSDAARILREIKLLRLLRHPDIVEIRHIMLPPSRRDFKDIYVVFELMDTDLHQVIK

ANDDLTKEHHQFFLYQMLRALKYIHTANVYHRDLKPKNILANANCKLKICDFGLARVAFN

DTPTTVFWTDYVATRWYRAPELCGSFFTKYSRAIDIWSIGCIFAEILTGKPLFPGKNVVH

QLDLMTDLLGTPSLDTVSRIRNEKARRYLSSMRKKQSVSFSERFPKADPAALKLLQRLLA

FDPKDRPTAEEALADPYFKGLGKVEREPSCQPITKMEFEFERKNVTKADVKELIFREILE

YHPQLLKDYMNGTEKTNFLYPSAVDNFRRQFANLEENGGKGGAVVPPDRKHVSLPRNTTV

HSTPIPPKDQKYSQVPQRIPTGRPGRVVGPVIPFENSSTMDPYSQRRVARNPVLPAAATN

LSAYAYRNSDNSERELQQELEKDRMQYHPMQRFMDAKMVSPDLRSTSYYMPKGVPKADVA

ERSALQSNMMQGIAPFNGIATVGGAFNKVSAVQYGVSRMY*

>Adi-12MPK20-5 Brdisv1Adi-121014469m.p

MFSDIFSREGVAFSRVALRQELLSDQWFTTAGGKGQCYLVPPGTTVFLVNSEEMGFFSEY

VDASRYKILEIIGKGSYGVVCSAIDQETGDKVAIKKIQNIFEHLSDAARILREIKLLRLL

RHPDIVQIKHIMLPPSRRDFRDIYVVFELMDTDLHQVIKANDDLTKEHYQFFLYQMLRAL

KYIHTANVYHRDLKPKNILANANCKLKICDFGLARVAFNDTPTTVFWTDYVATRWYRAPE

LCGSFFTKYSPAIDTWSIGCIFAEILTGKPLFPGKNVVHQLDLMTDLLGIPSTETISRIR

NDKARKYLSSMRRKQPIPFSEKFPNADPSALKLLERLLAFDPKDRPTAEEALAHPYFKRL

ARVEREPSCQQPISKTEFEFERRKFTKEDIKELIFREILEYHPKLLKDYMNGSEKTSFLY

PSAVDNFRRQFANLEIDGGRSGAADRKHFSLPRTTTVHSAPILPTNGPTSQVPQRIPTAR

PGRVVSSAMQTDNPSVSDRHNGRRVARDPAVPPAAAYHLKSDYSDRQHQQEFEKDRVRPD

RQRQEELEKDRMQYRPGHHSMDAKVAPEISPYMRSSPYYIPPFNGIAAVASGYSKVAAVT

RMY*

>Adi-12MPK21-1 Brdisv1Adi-121014362m.p

MGGLIRWLRHHRSRRVSSSSSSHLPSNTTSSSSTSDLRAHSLPQHQGDHHGEVVVEWEDA

AEGPDSDPEEYIVVVLGDDEQGVVAARAPVRTKPPRVMDPGKKTSESEFFTEYGEANRYK

VSEVIGKGSYGVVAAAVDTQTGERCAIKKINDVFDHVSDATRILREIKLLRLLRHPDIVE

IKHIMLPPSRREFRDIYVVFELMESDLHQVIKANDDLTPEHHQFFLYQLLRGMKYIHTAS

VFHRDLKPKNILANADCKLKICDFGLARVSFNDGAPSAIFWTDYVATRWYRAPELCGSFF

SKYTPAIDIWSVGCIFAEMLTGKPLFPGKNVVHQLDLMTDLLGTPSAESLSKIRNEKARR

YLSNMRKKPKVPLTKKFPGIDPMALHLLERLLAFDPKDRPSADEALTDPYFTGLANSERE

PITQPISKLEFEFEKRKLAKDDVRELIYREILEYHPHMLQEYLRGGGDQMSFMFPSGVDR

FKRQFAHLEEGGAKGEKSSPQLRQNASLPRERVIGNKHGDGDYNMKLNVGEKPELASVSD

GISKPLMSARSLLKSETMSASKCIGEIKNKDEDSLSECVEGTDDDVSQKIAQLKT*

>Adi-12MPK21-2 Brdisv1Adi-121040934m.p

MAPAPAATAHPPPPSPCPQPPTPATTSSDTAHRAHEGADAAEERDPGGEGDDPRGSGASL

RRTGRLAPRARWPGRAQMKRSSSRSTARRTGTRSAKWSARAATASWRXXXVAAAVDTHTG

ERVAIKKINDVFEHVSDATRILREIKLLRLLRHPDIVEIKHIILPPSRREFRDIYIIFEL

MESDLHQVIKANDDLTPEHHQFFFYQLLRGMKYIHAANVFHRDLKPRNILANADCKLKIC

DFGLARVSFNDTPSAIFWTDYVATRWYRAPELCGSFFSKYTPAIDIWSIGCIFAEMLSGR

PLFPGKNVVHQLDLMTDLLGTPSAESLSRIRNEKARRYLGNMRKKHPVPFSQKFPGVDPM

ALDLLERLLAFDPKDRPTAAEALADPYFTGLANSDREPTTQPISKLEFEFERRKLARDDV

RELIYREILEYHPQMLHEYHHGGDQANFVYPSGVDRFKRQFVHLEEGVTKGEKTSPQLRQ

HASLPRERIIGIGDELGRPNADYCIKLHVGEEPGHTSVTDGLSKPLLNARNFLKSESISA

SQCVVIKEKREKDEESMSEYMHEASDATPK*

>BdTR9kMPK3 Brdisv1BdTR9K1009255m.p

MDGAPVAEFRPTMTHGGRFLLYNIFGNQFEITAKYQPPIMPIGRGAYGIVCSVMNFETRE

MVAIKKIANAFDNNMDAKRTLREIKLLRHLDHENIVGLRDVIPPAIPQSFNDVYIATELM

DTDLHHIIRSNQELSEEHCQYFLYQLLRGLKYIHSANVIHRDLKPSNLLLNANCDLKICD

FGLARPSSESDMMTEYVVTRWYRAPELLLNSTDYSAAIDVWSVGCIFMELINRAPLFPGR

DHMHQMRLITEVIGTPTDDDLGFIRNEDARRYMRHLPQFPRRPFPAQFPRVQPAALDLIE

RMLAFNPLQRITVEEALEHPYLERLHDIADEPICTDPFSFDFEQHPLTEDQMKQLIFNEA

LELNPNFRY*

>BdTR9kMPK4 Brdisv1BdTR9K1030513m.p

MDTSGGGGGGAGGAAQIQGMATHGGRYVLYNVYGNLFEVASKYAPPIRPIGRGAYGIVCA

AVSSDTGEEVAIKKIGNAFDNHIDAKRTLREIKLLRHMDHENIIAIKDIIRPPRRDDFKD

VYIVTELMDTDLHQIIRSNQPLTDDHCQYFLYQLLRGLKYVHSANVLHRDLKPSNLFLNA

NCDLKIADFGLARTTSETDLMTEYVVTRWYRAPELLLNCSQYTAAIDVWSVGCILGEIIT

RQPLFPGRDYIQQLKLITELIGSPDDSSLGFLRSDNARRYMKQLPQYPRQDFRLRFRNMS

DGAVDLLERMLVFDPSRRITVDEALHHPYLASLHDINEEPTCPAPFSFDFEQPSFTEEHM

KELIWRETLAFNPDPPY*

>BdTR9kMPK6 Brdisv1BdTR9K1006792m.p

MTGDQTLASMTNYGRFSQIETAYWLVSYNKQSVSAFNVPVIVAIRDIIPPAQRNSFNDVY

IAYELMDTDLHQIIRSNQALSEEHCQYFLYQILRGLKYIHSANVLHRDLKPSNLLLNANC

DLKICDFGLARTTSETDFMTEYVVTRWYRAPELLLNSSEYTAAIDVWSVGCIFMELMDRK

PLFPGRDHVHQLRLLMELIGTPNEADLDFVNENARRYIRQLPRHARQSFSEKFPHVHPSA

IDLVEKMLTFDPRQRITVEGALAHPYLASLHDISDEPVCTMPFSFDFEQHALSEEQMKDL

IHQEALAFNPDYQ*

>BdTR9kMPK7-1 Brdisv1BdTR9K1043911m.p

MAMMVDPPNGTGNHGKHYYTMWQTMFEINTKYMPIKPIGRGAYGIVCSSTNQENNEKVAI

KKINNVFDNRVDALRTLRELKLLRHLRHENVIALKDIMMPVHRRSFKDVYLVFELMDTDL

HQIIKSSQPLSNDHCQYFLFQLLRGLKYLHSAGILHRDLKPGNLLVNANCDLKICDFGLA

RTNNTKGQFMTEYVVTRLYRAPELLLCCDNYGTSIDVWSVGCIFAELLGRKPIFPRTQCL

DQLKLIVNVLGTMSENDLEFIDNPKARKYIKSLPYTPGTPLTSMYPQAHPLAIDLLQKML

VFDPSKRISVTEALEHPYMSPLYDPSANPPAQVPIDLDIDENLGVEMIREMLWQEMLQYH

PEAARMVNM*

>BdTR9kMPK11 Brdisv1BdTR9K1028898m.p

MRMEGGGAGPAAAAAAGGAHGLGEAQIKGTLTHGGRYVQYNVYGNLFEVSAKYVPPIRPV

GRGACGIICAAINVQTREEVAIKKIGNAFDNQIDAKRTLREVKLLRHMNHENVISIKDII

RPPRRENFNDVYIVYELMDTDLPPLLRPTQPLTDDHCQYFLYQVLRGLKYVHSANVLHRD

LRPSNLLLNAKCDLKIGDFGLARTTTETDFMMEYVVTRWYRAPELLLNCSEYTGAIDMWS

VGCILGEIATREPLFPGKDYVHQLRLITELLGSPDDTSLGFLRSDNARRYVRSLPQYPKQ

QFRSRFPNMSSGAMDLLERMLVFDPNKRITVDEALCHPYLASLHEINDEPVCPAPFSFDF

EQPSFTEEDIKELIWRESVKFNPEPIH*

>BdTR9kMPK14 Brdisv1BdTR9K1027057m.p

MAMLVDPPNGMGNQGKHYYSMWQTLFEIDTKYVPIKPIGRGAYGIVCSSINRETNEKVAI

KKIHNVFDNRVDALRTLRELKLLRHLRHENVISLKDIMMPVQRRSFKDVYLVYELMDTDL

HQIIKSPQGLSNDHCQYFLFQLLRGLKYLHSAEILHRDLKPGNLLVNANCDLKICDFGLA

RTNSSKGQFMTEYVVTRWYRAPELLLCCDNYGTSIDVWSVGCIFAELLGRKPIFPGTECL

NQLKLIVNVLGTMSESDLEFIDNPKARRYIKTLPYTPGVPLASMYPHAHPLAIDLLQKML

IFDPTKRISVTEALEHPYMSPLYDPSANPPAQVPIDLDLDENISADMIREMMWQEMLHYH

PEAATAISM*

>BdTR9kMPK16 Brdisv1BdTR9K1016140m.p

MDFFTEYGEGNRYKIEEVIGKGSYGVVCSALDTHTGDKVAIKKINDIFEHVSDATRILRE

IKLLRLLRHPDIVEIKHILLPPSRREFKDIYVVFELMESDLHQVIKANDDLTPEHYQFFL

YQLLRGLKYIHTANVFHRDLKPKNILANADCKLKICDFGLARVAISDPPPAIFWTDYIAT

RWYRAPELCGSFFSKYTPAIDIWSIGCIFAELLTGKPLFPGKNVVHQLDIITDLLGTPSA

ETIARIRNEKARRYLSSMRRKKTVPFTQKFPNADPLALNLLERMLAFDPKDRPSAEEALA

DPYFKNIASVDREPSAQPITKLEFEFERRRITKDDIRELIYREILEYHPNMLREFLEGAE

PTGFMYPSAVDHFKKQFTFLEEHYAKGSTAAPPERQHNSLPRPSVIYSDNRPQNAANITD

DLSRCAIRDNTQKAQKDSASVGANRIPHGAAAAAARPGKVVGSVLRYGNCSTSGTEQYEQ

RRVVRSPGIAPNGVTSGSSYPRRNNTCKSETGEAERIDTNQGGPPKPYVANKLPATVDGR

NGHW*

>BdTR9kMPK17 Brdisv1BdTR9K1005090m.p

MVGGGGIVDGFRRLFHRRTPSGSGPSSNQSSAGEESSDIDAVVEDLDLVGLRAIRVPKRK

MPLPVESHKKNILEKEFFTEYGEESQYQIQEVIGKGSYGVVAAAVDTRTGERVAIKKIND

VFEHVSDATRILREVKLLRLLRHPDVVEIKHIMLPPSRREFQDIYVVFELMESDLHQVIR

ANDDLTPEHYQFFLYQLLRALKYIHAANVFHRDLKPKNILANSDCKLKICDFGLARVSFN

DAPTAIFWTDYVATRWYRAPELCGSFFSKYTPAIDIWSIGCIFAELLTGRPLFPGKNVVH

QLDIITDLLGTPSSETLSRIRNEKARRYLSCMRKKHPVPLTQKFPNVDPLALRLLGRLLA

FDPKDRPSAEEALADPYFAVLANVEREPSRNPISKLEFEFERRKVTKDDVRELIYREILE

YHPQMLQEYMQGGEQLSFLYPSGVDRFKRQFAHLEENYSKGERGSPLQRKHASLPRQRVG

ASNDNNEQHASDQERGADSIAPATGNPPGSQDAGQQHVSGGQNGVGSTNISPRSYQKSAS

ISASKCVVVNANKNPEYDDDISEEMEGAVDGLSEKVSRMHP*

>BdTR9kMPK20-1 Brdisv1BdTR9K1017427m.p

MQQDQRKKSSAEAEFFTEYGDASRYKIQEIVGKGSYGVVCSAIDVHTGEKVAIKKIHDIF

EHISDAARILREIKLLRLLRHPDIVEIKHIMLPPSRRDFKDIYVVFELMESDLHQVIKAN

DDLTKEHYQFFLYQLLRALKYIHTANVYHRDLKPKNILANSNCKLKICDFGLARVAFNDT

PTTIFWTDYVATRWYRAPELCGSFFSKYTPAIDVWSIGCIFAEVLTGKPLFPGKNVVHQL

DLMTDLLGTPSMDTISRVRNEKARRYLSSMRKKEPIPFSQKFPNADPLALDLLQRLLAFD

PKDRPTAGEALSHPYFKGLAKVEREPSCQPITKMEFEFERRRATKEDIRELIFREILEYH

PQLLKDYINGTERTTFLYPSAVDQFRKQFAHLEENSESGPVVPMDRKHTSLPRSTIVHSA

PIHAKEQPRIGPSRDRPLTDESYKNPRDSEKYSGNVPRSSHHSQAPQRVPTARPGRVVGP

VLPYENVGTSHPYDPRRVAMNSGYPPQQQIPQTYGYYQTTGKSACSEPSQAERYTLHQQA

YACANSSTAPDVALDMRAPPFHQSAGPKSGSSDRLTAETNLYTRSLNGIVATTSGVAASA

HRKVSVVPYGMSQMY*

>BdTR9kMPK20-2 Brdisv1BdTR9K1041463m.p

MQQPQQQGQRNKSTDVDFFSEYGDANRYRIQEVIGKGSYGVVCSAMDMQTRQKVAIKKIH

NIFDHVSDAARILREIKLLRLLRHPDVVEIKHIMLPPSRKDFKDIYVVFELMESDLHQVI

KANDDLTKEHYQFFLYQLLRALKYIHTASVYHRDLKPKNILANSNCKLKICDFGLARVAF

NDTPTTVFWTDYVATRWYRAPELCGSFFTKYTPAIDIWSIGCIFAEVLTGKPLFPGKNVV

HQLDLMTDLLGTPSMDTISRVRNEKARRYLSSMRKKDPVPFSKKFPNADPLGLKLLEKLL

AFDPKDRPTAEEALTDPYFKSLSKPDREPSCQPIRKVEFDFEHRRMSKDDIRELIFQEIL

EYHPQLLKNYIDGTEKTTFLYPSAVDQFKKQFSHLEESDGSGPVVPTERKHASLPRSTTV

HSTPIPAKEQPLVASSRGRPIANEPCKPWAPGNVPGASQTAHVAQAGRAVGVGSVPPYES

GSGKYPYDATSRPAVSSGYPPQQKIPQTYGYHHHHQTPGAGQSSQAMGGYACGYTKGTTP

PPAAAQDMRASPYHHRSAGTKNDPLNRLAAESDIYTRSLNGIVAAAASAGTGAHRKVGAV

PFGMSGMY*

>BdTR9kMPK20-3 Brdisv1BdTR9K1041205m.p

MQHGDLHKKSAAEMDFFTAYDDANRYKILEVIGKGSYGLVCSANDLQTGEKVAIKKIHNI

FEHISDAARILREIKLLRLLRHPDVVEIKHILLPPSKKDFKDIYVVFELMESDLHQVIKA

NDDLTREHYQFFLYQMLRALKYMHTANVYHRDLKPKNVLANANCKLKICDFGLARVAFSD

APTTVFWTDYVATRWYRAPELCGSFYSK

>BdTR9kMPK20-4 Brdisv1BdTR9K1017617m.p

MQTTEQQRKKGSSEMDFFSEYGDANRYKIQEVIGKGSYGVVCSAIDQHTGDKVAIKKIHN

IFEHLSDAARILREIKLLRLLRHPDIVEIRHIMLPPSRRDFKDIYVVFELMDTDLHQVIK

ANDDLTKEHHQFFLYQMLRALKYIHTANVYHRDLKPKNILANANCKLKICDFGLARVAFN

DTPTTVFWTDYVATRWYRAPELCGSFFTKYSRAIDIWSIGCIFAEILTGKPLFPGKNVVH

QLDLMTDLLGTPSLDTVSRIRNEKARRYLSSMRKKQSVSFSERFPKADPAALKLLQRLLA

FDPKDRPTAEEALADPYFKGLGKVERDPPCQPITKMEFEFERKNVTKADVKELIFREILE

YHPQLLKDYMNGTEKTNFLYPSAVDNFRRQFANLEENGGKGGAVVPPDRKHVSLPRNTTV

HSTPIPPKDQKYSQVPQRIPTGRPGRVVGPVIPFENSSTMDPYSQRRVARNPVLPAAATN

LSAYAYRNSDNSERELQQELEKDRMQYHPMQRFMDAKMVSPDLRSTSYYMPKGVPKADVA

ERSALQSNMMQGIAPFNGIATVGGAFNKVSAVQYGVSRMY*

>BdTR9kMPK20-5 Brdisv1BdTR9K1013831m.p

MFSDIFSREGVAFSRVALRQELLSDQWFTTAGGKGQCYLVPPGTTVFLVNSEEMGFFSEY

VDASRYKILEIIGKGSYGVVCSAIDQETGDKVAIKKIQNIFEHLSDAARILREIKLLRLL

RHPDIVQIKHIMLPPSRRDFRDIYVVFELMDTDLHQVIKANDDLTKEHYQFFLYQMLRAL

KYIHTANVYHRDLKPKNILANANCKLKICDFGLARVAFNDTPTTVFWTDYVATRWYRAPE

LCGSFFTKYSPAIDTWSIGCIFAEILTGKPLFPGKNVVHQLDLMTDLLGTPSTETISRIR

NDKARKYLSSMRRKQPIPFSEKFPNADPSALKLLERLLAFDPKDRPTAEEALAHPYFKRL

ARVEREPSCQQPISKTEFEFERRKFTKEDVKELIFREILEYHPKLLKDYMNGSEKTSFLY

PSAVDNFRRQFANLEIDGGRSGAADRKHFSLPRTTTVHSAPILPTNGPTSQVPQRIPTAR

PGRVVSSAMQTDNPSVSDRHNGRRVARDPAVPPAAAYHLKSDYSDRQHQQEFEKDRVRPD

RQRQEELEKDRMQYRPGHHSMDAKVAPEISPYMRSSPYYIPPFNGIAAVASGYSKVAAVT

RMY*

>BdTR9kMPK21-1 Brdisv1BdTR9K1013728m.p

MGGLIRWLRHHRSRRVSSSSSSHLPSNTTSSSSTSDLRAHSLPQHQGDPHGEVVVEWEDA

AEGPDSDPEEYIVVVLGDDEQGVVAARAPVRTKPPRVMDPGKKTSESEFFTEYGEANRYK

VSEVIGKGSYGVVAAAVDTQTGERCAIKKINDVFDHVSDATRILREIKLLRLLRHPDIVE

IKHIMLPPSRREFRDIYVVFELMESDLHQVIKANDDLTPEHHQFFLYQLLRGMKYIHTAS

VFHRDLKPKNILANADCKLKICDFGLARVSFNDGAPSAIFWTDYVATRWYRAPELCGSFF

SKYTPAIDIWSVGCIFAEMLTGKPLFPGKNVVHQLDLMTDLLGTPSAESLSKIRNEKARR

YLSNMRKKPKVPLTKKFPGIDPMALHLLERLLAFDPKDRPSADEALTDPYFTGLANSERE

PITQPISKLEFEFEKRKLAKDDVRELIYREILEYHPHMLQEYLRGGGDQMSFMFPSGVDR

FKRQFAHLEEGGAKGEKSSPQLRQNASLPRERVIGNKHGDGDYNMKLNVGEKPELASVSD

GISKPLMSARSLLKSETMSASKCIGEIKNKDEDSLSECVEGTDDDVSQKIAQLKT*

>BdTR9kMPK21-2 Brdisv1BdTR9K1038831m.p

MARKGSDEAEFFTEYGEANRYEVGEVVGKGSYGVVAAAVDTHTGERVAIKKINDVFEHVS

DATRILREIKLLRLLRHPDIVEIKHIILPPSRREFRDIYIIFELMESDLHQVIKANDDLT

PEHHQFFFYQLLRGMKYIHAANVFHRDLKPRNILANADCKLKICDFGLARVSFNDTPSAI

FWTDYVATRWYRAPELCGSFFSKYTPAIDIWSIGCIFAEMLSGRPLFPGKNVVHQLDLMT

DLLGTPSAESLSRIRNEKARRYLGNMRKKHPVPFSQKFPGVDPMALDLLERLLAFDPKDR

PTAAEALADPYFTGLANSDREPTTQPISKLEFEFERRKLARDDVRELIYREILEYHPQML

HEYHHGGDQANFVYPSGVDRFKRQFVHLEEGVTKGEKTSPQLRQHASLPRERIIGIGDEL

GRPNADYCIKLHVGEEPGHTSVTDGLRKPLLNARNFLKSESISASQCVVIKEKREKDEES

MSEYMHEASDATPK*

>BdTR12cMPK3 Brdisv1BdTR12c1009957m.p

MDGAPVAEFRPTMTHGGRFLLYNIFGNQFEITAKYQPPIMPIGRGAYGIVCSVMNFETRE

MVAIKKIANAFDNNMDAKRTLREIKLLRHLDHENIVGLRDVIPPAIPQSFNDVYIATELM

DTDLHHIIRSNQELSEEHCQYFLYQLLRGLKYIHSANVIHRDLKPSNLLLNANCDLKICD

FGLARPSSESDMMTEYVVTRWYRAPELLLNSTDYSAAIDVWSVGCIFMELINRAPLFPGR

DHMHQMRLITEVIGTPTDDDLGFIRNEDARRYMRHLPQFPRRPFPAQFPRVQPAALDLIE

RMLAFNPLQRITVEEALEHPYLERLHDIADEPICTDPFSFDFEQHPLTEDQMKQLIFNEA

LELNPNFRY*

>BdTR12cMPK4 Brdisv1BdTR12c1031941m.p

MDTSGGGGGGAGGGAQIQGMATHGGRYVLYNVYGNLFEVASKYAPPIRPIGRGAYGIVCA

AVSSDTGEEVAIKKIGNAFDNHIDAKRTLREIKLLRHMDHENIIAIKDIIRPPRRDDFKD

VYIVTELMDTDLHQIIRSNQPLTDDHCQYFLYQLLRGLKYVHSANVLHRDLKPSNLFLNA

NCDLKIADFGLARTTSETDLMTEYVVTRWYRAPELLLNCSQYTAAIDVWSVGCILGEIIT

RQPLFPGRDYIQQLKLITELIGSPDDSSLGFLRSDNARRYMKQLPQYPRQDFRLRFRNMS

DGAVDLLERMLVFDPSRRITVDEALHHPYLASLHDINEEPTCPAPFSFDFEQPSFTEEHM

KELIWRETLAFNPDPPY*

>BdTR12cMPK6 Brdisv1BdTR12c1007189m.p

MTGDQTLASMTNYGRFSQIETAYWLVSYNKQSVSAFNVPVIVAIRDIIPPAQRNSFNDVY

IAYELMDTDLHQIIRSNQALSEEHCQYFLYQILRGLKYIHSANVLHRDLKPSNLLLNANC

DLKICDFGLARTTSETDFMTEYVVTRWYRAPELLLNSSEYTAAIDVWSVGCIFMELMDRK

PLFPGRDHVHQLRLLMELIGTPNEADLDFVNENARRYIRQLPRHARQSFSEKFPHVHPSA

IDLVEKMLTFDPRQRITVEGALAHPYLASLHDISDEPVCTMPFSFDFEQHALSEEQMKDL

IHQEALAFNPDYQ*

>BdTR12cMPK7-1 Brdisv1BdTR12c1005219m.p

MPSRFPFLSPGCFIRLLSGSASSDLLLLLPRRGQSAPRARVLLKMAMMVDPPNGTGNHGK

HYYTMWQTMFEIDTKYVPIKPIGRGAYGIVCSSTNQENNEKVAIKKINNVFDNRVDALRT

LRELKLLRHLRHENVIALKDIMMPVHRRSFKDVYLVSELMDTDLHQIIKSSQPLSNDHCQ

YFLFQLLRGLKYLHSAGILHRDLKPGNLLVNANCDLKICDFGLARTNNTKGQFMTEYVVT

RLYRAPELLLCCDNYGTSIDVWSVGCIFAELLGRKPIFPRTQCLDQLKLIVNVLGTMSEN

DLEFIDNPKARKYIKSLPYTPGTPLTSMYPQAHPLAIDLLQKMLVFDPSKRISVTEALEH

PYMSPLYDPSANPPAQVPIDLDIDENLGVEMIREMLWQEMLQYHPEAARMVNM*

>BdTR12cMPK11 Brdisv1BdTR12c1030387m.p

MRMEGGGAGPAAAAAAGGAHGLGEAQIKGTLTHGGRYVQYNVYGNLFEVSAKYVPPIRPV

GRGACGIICAAINVQTREEVAIKKIGNAFDNQIDAKRTLREVKLLRHMNHENVISIKDII

RPPRRENFNDVYIVYELMDTDLHHLLRSNQPLTDDHCQYFLYQVLRGLKYVHSANVLHRD

LRPSNLLLNAKCDLKIGDFGLARTTTETDFMMEYVVTRWYRAPELLLNCSEYTGAIDMWS

VGCILGEIATREPLFPGKDYVHQLRLITELLGSPDDTSLGFLRSDNARRYVRSLPQYPKQ

QFRSRFPNMSSGAMDLLERMLVFDPNKRITVDEALCHPYLASLHEINDEPVCPAPFSFDF

EQPSFTEEDIKELIWRESVKFNPEPIH*

>BdTR12cMPK14 Brdisv1BdTR12c1028321m.p

MAMLVDPPNGMGNQGKHYYSMWQTLFEIDTKYVPIKPIGRGAYGIVCSSINRETNEKVAI

KKIHNVFDNRVDALRTLRELKLLRHLRHENVISLKDIMMPVQRRSFKDVYLVYELMDTDL

HQIIKSPQGLSNDHCQYFLFQLLRGLKYLHSAEILHRDLKPGNLLVNANCDLKICDFGLA

RTNSSKGQFMTEYVVTRWYRAPELLLCCDNYGTSIDVWSVGCIFAELLGRKPIFPGTECL

NQLKLIVNVLGTMSESDLEFIDNPKARRYIKTLPYTPGVPLASMYPHAHPLAIDLLQKML

IFDPTKRISVTEALEHPYMSPLYDPSANPPAQVPIDLDLDENISADMIREMMWQEMLHYH

PEAATAISM*

>BdTR12cMPK16 Brdisv1BdTR12c1013400m.p

MDFFTEYGEGNRYKIEEVIGKGSYGVVCSALDTHTGDKVAIKKINDIFEHVSDATRILRE

IKLLRLLRHPDIVEIKHILLPPSRREFKDIYVVFELMESDLHQVIKANDDLTPEHYQFFL

YQLLRGLKYIHTANVFHRDLKPKNILANADCKLKICDFGLARVAISDTPTAIFWTDYIAT

RWYRAPELCGSFFSKYTPAIDIWSIGCIFAELLTGKPLFPGKNVVHQLDIITDLLGTPSA

ETIARIRNEKARRYLSSMRRKKTVPFTQKFPNADPLALNLLERMLAFDPKDRPSAEEALA

DPYFKNIASVDREPSAQPITKLEFEFERRRITKDDIRELIYREILEYHPNMLREFLEGAE

PTGFMYPSAVDHFKKQFTFLEEHYAKGSTAAPPERQHNSLPRPSVIYSDNRPQNAANITD

DLSRCAIRDNTQKAQKDSASVGANRIPHGAAAAAARPGKVVGSVLRYGNCSTSGTEQYEQ

RRVVRSPGIAPNGVTSGSSYPRRNNTCKSETGEAERIDTNQGGPPKPYVANKLPATVDGR

NGHW*

>BdTR12cMPK17 Brdisv1BdTR12c1000261m.p

MVGGGGIVDGFRRLFHRRTPSGSGPSSNQSSAGEESSDIDAVVEDLDLVGLRAIRVPKRK

MPLPVESHKKNILEKEFFTEYGEASQYQIQEVIGKGSYGVVAAAVDTRTGERVAIKKIND

VFEHVSDATRILREVKLLRLLRHPDVVEIKHIMLPPSRREFQDIYVVFELMESDLHQVIR

ANDDLTPEHYQFFLYQLLRALKYIHAANVFHRDLKPKNILANSDCKLKICDFGLARVSFN

DAPTAIFWTDYVATRWYRAPELCGSFFSKYTPAIDIWSIGCIFAELLTGRPLFPGKNVVH

QLDIITDLLGTPSSETLSRIRNEKARRYLSCMRKKHPVPLTQKFPNVDPLALRLLGRLLA

FDPKDRPSAEEALADPYFAVLANVEREPSRNPISKLEFEFERRKVTKDDVRELIYREILE

YHPQMLQEYMQGGEQLSFLYPSGVDRFKRQFAHLEENYSKGERGSPLQRKHASLPRQRVG

ASNDNNEQHASDQERGADSIAPATGNPPGSQDAGQQHVSGGQNGVGSTNISPRSYQKSAS

ISASKCVVVNANKNPEYDDDISEEMEGAVDGLSEKVSRMHP*

>BdTR12cMPK20-1 Brdisv1BdTR12c1018145m.p

MQQDQRKKSSAEAEFFTEYGDASRYKIQEIVGKGSYGVVCSAIDVHTGEKVAIKKIHDIF

EHISDAARILREIKLLRLLRHPDIVEIKHIMLPPSRRDFKDIYVVFELMESDLHQVIKAN

DDLTKEHYQFFLYQLLRALKYIHTANVYHRDLKPKNILANSNCKLKICDFGLARVAFNDT

PTTIFWTDYVATRWYRAPELCGSFFSKYTPAIDVWSIGCIFAEVLTGKPLFPGKNVVHQL

DLMTDLLGTPSMDTISRVRNEKARRYLSSMRKKEPIPFSQKFPNADPLALDLLQRLLAFD

PKDRPTAGEALSHPYFKGLAKVEREPSCQPITKMEFEFERRRATKEDIRELIFREILEYH

PQLLKDYINGTERTTFLYPSAVDQFRKQFAHLEENSESGPVVPMDRKHTSLPRSTIVHSA

PIHAKEQPRIGPSRDRPLTDESYKNPRDSEKYSGNVPRSSHHSQAPQRVPTARPGRVVGP

VLPYENVGTSHPYDPRRVAMNSGYPPQQQIPQTYGYYQTTGKSACSEPSQAERYTLHQQA

YACANSSTAPDVALDMRAPPFHQSAGPKSGSSDRLTAETNLYTRSLNGIVATTSGVAASA

HRKVSVVPYGMSQMY*

>BdTR12cMPK20-2 Brdisv1BdTR12c1014428m.p

MQQPQQQGQRNKSTDVDFFSEYGDANRYRIQEVIGKGSYGVVCSAMDMQTRQKVAIKKIH

NIFDHVSDAARILREIKLLRLLRHPDVVEIKHIMLPPSRKDFKDIYVVFELMESDLHQVI

KANDDLTKEHYQFFLYQLLRALKYIHTASVYHRDLKPKNILANSNCKLKICDFGLARVAF

NDTPTTVFWTDYVATRWYRAPELCGSFFTKYTPAIDIWSIGCIFAEVLTGKPLFPGKNVV

HQLDLMTDLLGTPSMDTISRVRNEKARRYLSSMRKKDPVPFSKKFPNADPLGLKLLEKLL

AFDPKDRPTAEEALTDPYFKSLSKPDREPSCQPIRKVEFDFEHRRMSKDDIRELIFQEIL

EYHPQLLKNYIDGTEKTTFLYPSAVDQFKKQFSHLEESDGSGPVVPTERKHASLPRSTTV

HSTPIPAKEQPLVASSRGRPIANEPCKPWAPGNVPGASQTAHVAQAGRAVGVGSVPPYES

GSGKYPYDATSRPAVSSGYPPQQKIPQTYGYHHHHQTPGAGQSSQAMGGYACGYTKGTTP

PPAAAQDMRASPYHHRSAGTKNDPLNRLAAESDIYTRSLNGIVAAAASAGTGAHRKVGAV

PFGMSGMY*

>BdTR12cMPK20-3 Brdisv1BdTR12c1042500m.p

MQHGDLHKKSAAEMDFFTAYDDANRYKILEVIGKGSYGLVCSANDLQTGEKVAIKKIHNI

FEHISDAARILREIKLLRLLRHPDVVEIKHILLPPSKKDFKDIYVVFELMESDLHQVIKA

NDDLTREHYQFFLYQMLRALKYMHTANVYHRDLKPKNVLANANCKLKICDFGLARVAFSD

APTTVFWTDYVATRWYRAPELCGSFYSKVMIVTIDH*

>BdTR12cMPK20-4 Brdisv1BdTR12c1018156m.p

MQTTEQQRKKGSSEMDFFSEYGDANRYKIQEVIGKGSYGVVCSAIDQHTGDKVAIKKIHN

IFEHLSDAARILREIKLLRLLRHPDIVEIRHIMLPPSRRDFKDIYVVFELMDTDLHQVIK

ANDDLTKEHHQFFLYQMLRALKYIHTANVYHRDLKPKNILANANCKLKICDFGLARVAFN

DTPTTVFWTDYVATRWYRAPELCGSFFTKYSRAIDIWSIGCIFAEILTGKPLFPGKNVVH

QLDLMTDLLGTPSLDTVSRIRNEKARRYLSSMRKKQSVSFSERFPKADPAALKLLQRLLA

FDPKDRPTAEEALADPYFKGLGKVEREPSCQPITKMEFEFERKNVTKADVKELIFREILE

YHPQLLKDYMNGTEKTNFLYPSAVDNFRRQFANLEENGGKGGAVVPPDRKHVSLPRNTTV

HSTPIPPKDQKYSQVPQRIPTGRPGRVVGPVIPFENSSTMDPYSQRRVARNPVLPAAATN

LSAYAYRNSDNSERELQQELEKDRMQYHPMQRFMDAKMVSPDLRSTSYYMPKGVPKADVA

ERSALQSNMMQGIAPFNGIATVGGAFNKVSAVQYGVSRMY*

>BdTR12cMPK20-5 Brdisv1BdTR12c1014592m.p

MFSDIFSREGVAFSRVALRQELLSDQWFTTAGGKGQCYLVPPGTTVFLVNSEEMGFFSEY

VDASRYKILEIIGKGSYGVVCSAIDQETGDKVAIKKIQNIFEHLSDAARILREIKLLRLL

RHPDIVQIKHIMLPPSRRDFRDIYVVFELMDTDLHQVIKANDDLTKEHYQFFLYQMLRAL

KYIHTANVYHRDLKPKNILANANCKLKICDFGLARVAFNDTPTTVFWTDYVATRWYRAPE

LCGSFFTKYSPAIDTWSIGCIFAEILTGKPLFPGKNVVHQLDLMTDLLGIPSTETISRIR

NDKARKYLSSMRRKQPIPFSEKFPNADPSALKLLERLLAFDPKDRPTAEEALAHPYFKRL

ARVEREPSCQQPISKTEFEFERRKFTKEDIKELIFREILEYHPKLLKDYMNGSEKTSFLY

PSAVDNFRRQFANLEIDGGRSGAADRKHFSLPRTTTVHSAPILPTNGPTSQVPQRIPTAR

PGRVVSSAMQTDNPSVSDRHNGRRVARDPAVPPAAAYHLKSDYSDRQHQQEFEKDRVRPD

RQRQEELEKDRMQYRPGHHSMDAKVAPEISPYMRSSPYYIPPFNGIAAVASGYSKVAAVT

RMY*

>BdTR12cMPK21-1 Brdisv1BdTR12c1014472m.p

MGGLIRWLRHHRSRRVSSSSSSHLPSNTTSSSSTSDLRAHSLPQHQGDHHGEVVVEWEDA

AEGPDSDPEEYIVVVLGDDEQGVVAARAPVRTKPPRVMDPGKKTSESEFFTEYGEANRYK

VSEVIGKGSYGVVAAAVDTQTGERCAIKKINDVFDHVSDATRILREIKLLRLLRHPDIVE

IKHIMLPPSRREFRDIYVVFELMESDLHQVIKANDDLTPEHHQFFLYQLLRGMKYIHTAN

VFHRDLKPKNILANADCKLKICDFGLARVSFNDGAPSAIFWTDYVATRWYRAPELCGSFF

SKYTPAIDIWSVGCIFAEMLTGKPLFPGKNVVHQLDLMTDLLGTPSAESLSKIRNEKARR

YLSNMRKKPKVPLTKKFPGIDPMALHLLERLLAFDPKDRPSADEALTDPYFTGLANSERE

PITQPISKLEFEFEKRKLAKDDVRELIYREILEYHPHMLQEYLRGGGDQMSFMFPSGVDR

FKRQFAHLEEGGAKGEKSSPQLRQNASLPRERVIGNKHGDGDYNMKLNVGEKPELASVSD

GISKPLMSARSLLKSETMSASKCIGEIKNKDEDSLSECVEGTDDDVSQKIAQLKT*

>BdTR12cMPK21-2 Brdisv1BdTR12c1018230m.p

MARKGSDEAEFFTEYGEANRYEVGEVVGKGSYGVVAAAVDTHTGERVAIKKINDVFEHVS

DATRILREIKLLRLLRHPDIVEIKHIILPPSRREFRDIYIIFELMESDLHQVIKANDDLT

PEHHQFFFYQLLRGMKYIHAANVFHRDLKPRNILANADCKLKICDFGLARVSFNDTPSAI

FWTDYVATRWYRAPELCGSFFSKYTPAIDIWSIGCIFAEMLSGRPLFPGKNVVHQLDLMT

DLLGTPSAESLSRIRNEKARRYLGNMRKKHPVPFSQKFPGVDPMALDLLERLLAFDPKDR

PTAAEALADPYFTGLANSDREPTTQPISKLEFEFERRKLARDDVRELIYREILEYHPQML

HEYHHGGDQANFVYPSGVDRFKRQFVHLEEGVTKGEKTSPQLRQHASLPRERIIGIGDEL

GRPNADYCIKLHVGEEPGHTSVTDGLSKPLLNARNFLKSESISASQCVVIKEKREKDEES

MSEYMHEASDATPK*

>Kah-1MPK3 Brdisv1Kah-11008192m.p

MDGAPVAEFRPTMTHGGRFLLYNIFGNQFEITAKYQPPIMPIGRGAYGIVCSVMNFETRE

MVAIKKIANAFDNNMDAKRTLREIKLLRHLDHENIVGLRDVIPPAIPQSFNDVYIATELM

DTDLHHIIRSNQELSEKHCQYFLYQLLRGLKYIHSANVIHRDLKPSNLLLNANCDLKICD

FGLARPSSESDMMTEYVVTRGYRAPELLLNSTDYSAAIDVWSVGCIFMELINRAPLFPGR

DHMHQMRLITEVIGTPTDDDLGFIRNEDARRYMSPLPQFPRRPFPAQFPRVQPAALDLIE

RMLAFNPLQRITVEEALEHPYLERLHDIADEPICTDPFSFDFEQHPLTEDQMKQLIFNEA

LELNPNFRY*

>Kah-1MPK4 Brdisv1Kah-11021773m.p

MDTSGGGGGGAGGAGQIQGMATHGGRYVLYNVYGNLFEVASKYAPPIRPIGRGAYGIVCA

AVSSDTGEEVAIKKIGNAFDNHIDAKRTLREIKLLRHMDHENIIAIKDIIRPPRRDDFKD

VYIVTELMDTDLHQIIRSNQPLTDDHCQYFLYQLLRGLKYVHSANVLHRDLKPSNLFLNA

NCDLKIADFGLARTTSETDLMTEYVVTRWYRAPELLLNCSQYTAAIDVWSVGCILGEIIT

RQPLFPGRDYIQQLKLITELIGSPDDSSLGFLRSDNARRYMKQLPQYPRQDFRLRFRNMS

DGAVDLLERMLVFDPSRRITVDEALHHPYLASLHDINEEPTCPAPFSFDFEQPSFTEEHM

KELIWRETLAFNPDPPY*

>Kah-1MPK6 Brdisv1Kah-11006028m.p

IVAIRDIIPPAQRNSFNDVYIAYELMDTDLHQIIRSNQALSEEHCQYFLYQILRGLKYIH

SANVLHRDLKPSNLLLNANCDLKICDFGLARTTSETDFMTEYVVTRWYRAPELLLNSSEY

TAAIDVWSVGCIFMELMDRKPLFPGRDHFHQLRLLMELIGTPNEADLDFVNENARRYIRQ

LPRHARQSFSEKFPHVHPSAIDLVEKMLTFDPRQRITVEGALAHPYLASLHDISDEPVCT

MPFSFDFEQHALSEEQMKDLIHQEALAFNPDYQ*

>Kah-1MPK7-1 Brdisv1Kah-11044255m.p

MGQSKTDKGDQDGDLARLSGLRGEPLLHRLHRRERPRGLPRASCRSLRKELLRGLKYLHS

AGILHRDLKPGNLLVNANCDLKICDFGLARTNNTKGQFMTEYVVTRWYRAPELLLCCDNY

GTSIDVWSVGCIFAELLGRKPIFPRTQCLDQLKLIVNVLGTMSENDLEFIDNPKARKYIK

SLPYTPGTPLTSMYPQAHPLAIDLLQKMLVFDPSKRISVTEALEHPYMSPLYDPSANPPA

QVPIDLDIDENLGVEMIREMLWQEMLQYHPEAARMVNM*

>Kah-1MPK11 Brdisv1Kah-11020359m.p

MRMEGGGAGPAAAAAAGGAHGLGEAQIKGTLTHGGRYVQYNVYGNLFEVSAKYVPPIRPV

GRGACGIICAAINVQTREEVAIKKIGNAFDNQIDAKRTLREVKLLRHMNHENVISIKDII

RPPRRENFNDVYIVYELMDTDLHHLLRSTQPLTDDHCQYFLYQVLRGLKYVHSANVLHRD

LRPSNLLLNAKCDLKIGDFGLARTTTETDFMMEYVVTRWYRAPELLLNCSEYTGAIDMWS

VGCILGEIATREPLFPGKDYVHQLRLITELLGSPDDTSLGFLRSDNARRYVRSLPQYPKQ

QFRSRFPNMSSGAMDLLERMLVFDPNKRITVDEALCHPYLASLHEINDEPVCPAPFSFDF

EQPSFTEEDIKELIWRESVKFNPEPIH*

>Kah-1MPK14 Brdisv1Kah-11018519m.p

MAMLVDPPNGMGNQGKHYYSMWQTLFEIDTKYVPIKPIGRGAYGIVCSSINRETNEKVAI

KKIHNVFDNRVDALRTLRELKLLRHLRHENVISLKDIMMPVQRRSFKDVYLVYELMDTDL

HQIIKSPQGLSNDHCQYFLFQLLRGLKYLHSAEILHRDLKPGNLLVNANCDLKICDFGLA

RTNSSKGQFMTEYVVTRWYRAPELLLCCDNYGTSIDVWSVGCIFAELLGRKPIFPGTECL

NQLKLIVNVLGTMSESDLEFIDNPKARRYIKTLPYTPGVPLASMYPHAHPLAIDLLQKML

IFDPTKRISVTEALEHPYMSPLYDPSANPPAQVPIDLDLDENISADMIREMMWQEMLHYH

PEAATAISM*

>Kah-1MPK16 Brdisv1Kah-11014205m.p

MDFFTEYGEGNRYKIEEVIGKGSYGVVCSALDTHTGDKVAIKKINDIFEHVSDATRILRE

IKLLRLLRHPDIVEIKHILLPPSRREFKDIYVVFELMESDLHQVIKANDDLTPEHYQFFL

YQLLRGLKYIHTANVFHRDLKPKNILANADCKLKICDFGLARVAISDTPTAIFWTDYIAT

RWYRAPELCGSFFSKYTPAIDIWSIGCIFAELLTGKPLFPGKNVVHQLDIITDLLGTPSA

ETIARIRNEKARRYLSSMRRKKTVPFTQKFPNADPLALNLLERMLAFDPKDRPSAEEALA

DPYFKNIASVDREPSAQPITKLEFEFERRRITKDDIRELIYREILEYHPNMLREFLEGAE

PTGFMYPSAVDHFKKQFTFLEEHYAKGSTAAPPERQHNSLPRPSVIYSDNRPQNAANITD

DLSRCAIRDNTQKAQKDSASVGANRIPHGAAAAAARPGKVVGSVLRYGNCSTSGTEQYEQ

RRVVRSPGIAPNGVTSGSSYPRRNNTCKSETGEAERIDTNQGGPPKPYVANKLPATVDGR

NGHW*

>Kah-1MPK20-1 Brdisv1Kah-11035683m.p

MQQDQRKKSSAEAEFFTEYGDASRYKIQEIVGKGSYGVVCSAIDVHTGEKVAIKKIHDIF

EHISDAARILREIKLLRLLRHPDIVEIKHIMLPPSRRDFKDIYVVFELMESDLHQVIKAN

DDLTKEHYQFFLYQLLRALKYIHTANVYHRDLKPKNILANSNCKLKICDFGLARVAFNDT

PTTIFWTDYVATRWYRAPELCGSFFSKYTPAIDVWSIGCIFAEVLTGKPLFPGKNVVHQL

DLMTDLLGTPSMDTISRVRNEKARRYLSSMRKKEPIPFSQKFPNADPLALDLLQRLLAFD

PKDRPTAGEALSHPYFKGLAKVEREPSCQPITKMEFEFERRRATKEDIRELIFREILEYH

PQLLKDYINGTERTTFLYPRF*

>Kah-1MPK20-2 Brdisv1Kah-11012143m.p

MQQPQQQGQRNKSTDVDFFSEYGDANRYRIQEVIGKGSYGVVCSAMDMQTRQKVAIKKIH

NIFDHVSDAARILREIKLLRLLRHPDVVEIKHIMLPPSRKDFKDIYVVFELMESDLHQVI

KANDDLTKEHYQFFLYQLLRALKYIHTASVYHRDLKPKNILANSNCKLKICDFGLARVAF

NDTPTTVFWTDYVATRWYRAPELCGSFFTKYTPAIDIWSIGCIFAEVLTGKPLFPGKNVV

HQLDLMTDLLGTPSMDTISRVRNEKARRYLSSMRKKDPVPFSKKFPNADPLGLKLLEKLL

AFDPKDRPTAEEALTDPYFKSLSKPDREPSCQPIRKVEFDFEHRRMSKDDIRELIFQEIL

EYHPQLLKNYIDGTEKTTFLYPSAVDQFKKQFSHLEESDGSGPVVPTERKHASLPRSTTV

HSTPIPAKEQPLVASSRGRPIANEPCKPWAPGNVPGASQTAHVAQAGRAVGVGSVPPYES

GSGKYPYDATSRPAVSSGYPPQQKIPQTYGYHHHHQTPGAGQSSQAMGGYACGYTKGTTP

PPAAAQDMRASPYHHRSAGTKNDPLNRLAAESDIYTRSLNGIVAAAASAGTGAHRKVGAV

PFGMSGMY*

>Kah-1MPK20-3 Brdisv1Kah-11037354m.p

MQHGDLHKKSAAEMDFFTAYDDANRYKILEVIGKGSYGLVCSANDLQTGEKVAIKKIHNI

FEHISDAARILREIKLLRLLRHPDVVEIKHILLPPSKKDFKDIYVVFELMESDLHQVIKA

NDDLTREHYQFFLYQMLRALKYMHTANVYHRDLKPKNVLANANCKLKICDFGLARVAFSD

APTTVFWTDYVATRWYRAPELCGSFYSKVMIVTIDH*

>Kah-1MPK20-4 Brdisv1Kah-11015432m.p

MTDLLGTPSLDTVSRIRNEKARRYLSSMRKKQSVSFSERFPKADPAALKLLQRLLAFDPK

DRPTAEEALADPYFKGLGKVEREPSCQPITKMEFEFERKNVTKADVKELIFREILEYHPQ

LLKDYMNGTEKTNFLYPSAVDNFRRQFANLEENGGKGGAVVPPDRKHVSLPRNTTVHSTP

IPPKDQKYSQVPQRIPTGRPGRVVGPVIPFENSSTMDPYSQRRGARNPVLPAAATNLSAY

AYRNSDNSERELQQELEKDRMQYHPMQRFMDAKMVSPDLRATSYYMPKGVPKADVAERSA

LQSNMMQGIAPFNGIATVGGAFNKVSAVQYGVSRMY*

>Kah-1MPK20-5 Brdisv1Kah-11012290m.p

MFSDIFSREGVAFSRVALRQELLSDQWFTTAGGKGQCYLVPPGTTVFLVNSEEMGFFSEY

VDASRYKILEIIGKGSYGVVCSAIDQETGDKVAIKKIQNIFEHLSDAARILREIKLLRLL

RHPDIVQIKHIMLPPSRRDFRDIYVVFELMDTDLHQVIKANDDLTKEHYQFFLYQMLRAL

KYIHTANVYHRDLKPKNILANANCKLKICDFGLARVAFNDTPTTVFWTDYVATRWYRAPE

LCGSFFTKYSPAIDTWSIGCIFAEILTGKPLFPGKNVVHQLDLMTDLLGIPSTETISRIR

NDKARKYLSSMRRKQPIPFSEKFPNADPSALKLLERLLAFDPKDRPTAEEALAHPYFKRL

ARVEREPSCQQPISKTEFEFERRKFTKEDIKELIFREILEYHPKLLKDYMNGSEKTSFLY

PSAVDNFRRQFANLEIDGGRSGAADRKHFSLPRTTTVHSAPILPTNGPTSQVPQRIPTAR

PGRVVSSAMQTDNPSVSDRHNGRRVARDPAVPPAAAYHLKSDYSDRQHQQEFEKDRVRPD

RQRQEELEKDRMQYRPGHHSMDAKVAPEISPYMRSSPYYIPPFNGIAAVASGYSKVAAVT

RMY*

>Kah-1MPK21-1 Brdisv1Kah-11012177m.p

MGGLIRWLRPPRPRRVSSSSSSHLPSNTTSSSSTSDLRAHSLPQHQGDHHGEVVVEWEDA

AEGPDSDPEEYIVVVLGDDEQGVVAARAPVRTKPPRVMDPGKKTSESEFFTEYGEANRYK

VSEVIGKGSYGVVAAAVDTQTGERCAIKKINDVFDHVSDATRILREIKLLRLLRHPDIVE

IKHIMLPPSRREFRDIYVVFELMESDLHQVIKANDDLTPEHHQFFLYQLLRGMKYIHTAS

VFHRDLKPKNILANADCKLKICDFGLARVSFNDGAPSAIFWTDYVATRWYRAPELCGSFF

SKYTPAIDIWSVGCIFAEMLTGKPLFPGKNVVHQLDLMTDLLGTPSAESLSKIRNEKARR

YLSNMRKKPKVPLTKKFPGIDPMALHLLERLLAFDPKDRPSADEALTDPYFTGLANSERE

PITQPISKLEFEFEKRKLAKDDVRELIYREILEYHPHMLQEYLRGGGDQMSFMFPSGVDR

FKRQFAHLEEGGAKGEKSSPQLRQNASLPRERVIGNKHGDGDYNMKLNVGEKPELASVSD

GISKPLMSARSLLKSETMSASKCIGEIKNKDEDSLSECVEGTDDDVSQKIAQLKT*

>Kah-1MPK21-2 Brdisv1Kah-11015331m.p

MARKGSDEAEFFTEYGEANRYEVGEVVGKGSYGVVAAAVDTHTGERVAIKKINDVFEHVS

DATRILREIKLLRLLRHPDIVEIKHIILPPSRREFRDIYIIFELMESDLHQVIKANDDLT

PEHHQFFFYQLLRGMKYIHAANVFHRDLKPRNILANADCKLKICDFGLARVSFNDTPSAI

FWTDYVATRWYRAPELCGSFFSKYTPAIDIWSIGCIFAEMLSGRPLFPGKNVVHQLDLMT

DLLGTPSAESLSRIRNEKARRYLGNMRKKHPVPFSQKFPGVDPMALDLLERLLAFDPKDR

PTAAEALADPYFTGLANSDREPTTQPISKLEFEFERRKLARDDVRELIYREILEYHPQML

HEYHHGGDQANFVYPSGVDRFKRQFVHLEEGVTKGEKTSPQLRQHASLPRERIIGIGDEL

GRPNADYCIKLHVGEEPGHTSGTDGLRKPLLNARNFLKSESISASQCVVIKEKREKDEES

MSEYMHEASDATPK*

>Kah-5MPK3 Brdisv1Kah-51010925m.p

MDGAPVAEFRPTMTHGGRFLLYNIFGNQFEITAKYQPPIMPIGRGAYGIVCSVMNFETRE

MVAIKKIANAFDNNMDAKRTLREIKLLRHLDHENIVGLRDVIPPAIPQSFNDVYIATELM

DTDLHHIIRSNQELSEEHCQYFLYQLLRGLKYIHSANVIHRDLKPSNLLLNANCDLKICD

FGLARPSSESDMMTEYVVTRWYRAPELLLNSTDYSAAIDVWSVGCIFMELINRAPLFPGR

DHMHQMRLITEVIGTPTDDDLGFIRNEDARRYMRHLPQFPRRPFPAQFPRVQPAALDLIE

RMLAFNPLQRITVEEALEHPYLERLHDIADEPICTDPFSFDFEQHPLTEDQMKQLIFNEA

LELNPNFRY*

>Kah-5MPK4 Brdisv1Kah-51035392m.p

MDTSGGGGGGAGGAAQIQGMATHGGRYVLYNVYGNLFEVASKYAPPIRPIGRGAYGIVCA

AVSSDTGEEVAIKKIGNAFDNHIDAKRTLREIKLLRHMDHENIIAIKDIIRPPRRDDFKD

VYIVTELMDTDLHQIIRSNQPLTDDHCQYFLYQLLRGLKYVHSANVLHRDLKPSNLFLNA

NCDLKIADFGLARTTSETDLMTEYVVTRWYRAPELLLNCSQYTAAIDVWSVGCILGEIIT

RQPLFPGRDYIQQLKLITELIGSPDDSSLGFLRSDNARRYMKQLPQYPRQDFRLRFRNMS

DGAVDLLERMLVFDPSRRITVDEALHHPYLASLHDINEEPTCPAPFSFDFEQPSFTEEHM

KELIWRETLAFNPDPPY*

>Kah-5MPK6 Brdisv1Kah-51007875m.p

MDGGAQPPDAEMADAGAAVPGAGAGAAGTMENIQATLSHGGRFIQYNIFGNVFEVTVKYK

PPILPIGKGAYGIVCSALNSETGEQVAIKKIANAFDNKIDAKRTLREIKLLRHMDHENIV

AIRDIIPPAQRNSFNDVYIAYELMDTDLHQIIRSNQALSEEHCQYFLYQILRGLKYIHSA

NVLHRDLKPSNLLLNANCDLKICDFGLARTTSETDFMTEYVVTRWYRAPELLLNSSEYTA

AIDVWSVGCIFMELMDRKPLFPGRDHVHQLRLLMELIGTPNEADLDFVNENARRYIRQLP

RHARQSFSEKFPHVHPSAIDLVEKMLTFDPRQRITVEGALAHPYLASLHDISDEPVCTMP

FSFDFEQHALSEEQMKDLIHQEALAFNPDYQ*

>Kah-5MPK7-1 Brdisv1Kah-51046477m.p

MGGPLPGSAGDQSRGLSAWKSKTACRCPIAGILNTCASSTREAAEPDPQLLRGLKYLHSA

GILHRDLKPGNLLVNANCDLKICDFGLARTNNTKGQFMTEYVVTRWYRAPELLLCCDNYG

TSIDVWSVGCIFAELLGRKPIFPGTECLNQLKLIVNVLGTMSENDLEFIDNPKARKYIKS

LPYTPGTPLTSMYPQAHPLAIDLLQKMLVFDPSKRISVTEALEHPYMSPLYDPSANPPAQ

VPIDLDIDENLGVEMIREMLWQEMLQYHPEAARMVNM*

>Kah-5MPK11 Brdisv1Kah-51033455m.p

MRMEGGGAGPAAAAAAGGAHGLGEAQIKGTLTHGGRYVQYNVYGNLFEVSAKYVPPIRPV

GRGACGIICAAINVQTREEVAIKKIGNAFDNQIDAKRTLREVKLLRHMNHENVISIKDII

RPPRRENFNDVYIVYELMDTDLHHLLRSNQPLTDDHCQYFLYQVLRGLKYVHSANVLHRD

LRPSNLLLNAKCDLKIGDFGLARTTTETDFMMEYVVTRWYRAPELLLNCSEYTGAIDMWS

VGCILGEIATREPLFPGKDYVHQLRLITELLGSPDDTSLGFLRSDNARRYVRSLPQYPKQ

QFRSRFPNMSSGAMDLLERMLVFDPNKRITVDEALCHPYLASLHEINDEPVCPAPFSFDF

EQPSFTEEDIKELIWRESVKFNPEPIH*

>Kah-5MPK14 Brdisv1Kah-51031281m.p

MAMLVDPPNGMGNQGKHYYSMWQTLFEIDTKYVPIKPIGRGAYGIVCSSINRETNEKVAI

KKIHNVFDNRVDALRTLRELKLLRHLRHENVISLKDIMMPVQRRSFKDVYLVYELMDTDL

HQIIKSPQGLSNDHCQYFLFQLLRGLKYLHSAEILHRDLKPGNLLVNANCDLKICDFGLA

RTNSSKGQFMTEYVVTRWYRAPELLLCCDNYGTSIDVWSVGCIFAELLGRKPIFPGTECL

NQLKLIVNVLGTMSESDLEFIDNPKARRYIKTLPYTPGVPLASMYPHAHPLAIDLLQKML

IFDPTKRISVTEALEHPYMSPLYDPSANPPAQVPIDLDLDENISADMIREMMWQEMLHYH

PEAATAISM*

>Kah-5MPK16 Brdisv1Kah-51018690m.p

MDFFTEYGEGNRYKIEEVIGKGSYGVVCSALDTHTGDKVAIKKINDIFEHVSDATRILRE

IKLLRLLRHPDIVEIKHILLPPSRREFKDIYVVFELMESDLHQVIKANDDLTPEHYQFFL

YQLLRGLKYIHTANVFHRDLKPKNILANADCKLKICDFGLARVAISDTPTAIFWTDYIAT

RWYRAPELCGSFFSKYTPAIDIWSIGCIFAELLTGKPLFPGKNVVHQLDIITDLLGTPSA

ETIARIRNEKARRYLSSMRRKKTVPFTQKFPNADPLALNLLERMLAFDPKDRPSAEEALA

DPYFKNIASVDREPSAQPITKLEFEFERRRITKDDIRELIYREILEYHPNMLREFLEGAE

PTGFMYPSAVDHFKKQFTFLEEHYAKGSTAAPPERQHNSLPRPSVIYSDNRPQNAANITD

DLSRCAIRDNTQKAQKDSASVGANRIPHGAAAAAARPGKVVGSVLRYGNCSTSGTEQYEQ

RRVVRSPGIAPNGVTSGSSYPRRNNTCKSETGEAERIDTNQGGPPKPYVANKLPATVDGR

NGHW*

>Kah-5MPK17 Brdisv1Kah-51005745m.p

MVGGGGIVDGFRRLFHRRTPSGSGPSSNQSSAGEESSDIDAVVEDLDLVGLRAIRVPKRK

MPLPVESHKKNILEKEFFTEYGEASQYQIQEVIGKGSYGVVAAAVDTRTGERVAIKKIND

VFEHVSDATRILREVKLLRLLRHPDVVEIKHIMLPPSRREFQDIYVVFELMESDLHQVIR

ANDDLTPEHYQFFLYQLLRALKYIHAANVFHRDLKPKNILANSDCKLKICDFGLARVSFN

DAPTAIFWTDYVATRWYRAPELCGSFFSKYTPAIDIWSIGCIFAELLTGRPLFPGKNVVH

QLDIITDLLGTPSSETLSRIRNEKARRYLSCMRKKHPVPLTQKFPNVDPLALRLLGRLLA

FDPKDRPSAEEALADPYFAVLANVEREPSRNPISKLEFEFERRKVTKDDVRELIYREILE

YHPQMLQEYMQGGEQLSFLYPSGVDRFKRQFAHLEENYSKGERGSPLQRKHASLPRQRVG

ASNDNNEQHASDQERGADSIAPATGNPPGSQDAGQQHVSGGQNGVGSTNISPRSYQKSAS

ISASKCVVVNANKNPEYDDDISEEMEGAVDGLSEKVSRMHP*

>Kah-5MPK20-1 Brdisv1Kah-51020173m.p

MQQDQRKKSSAEAEFFTEYGDASRYKIQEIVGKGSYGVVCSAIDVHTGEKVAIKKIHDIF

EHISDAARILREIKLLRLLRHPDIVEIKHIMLPPSRRDFKDIYVVFELMESDLHQVIKAN

DDLTKEHYQFFLYQLLRALKYIHTANVYHRDLKPKNILANSNCKLKICDFGLARVAFNDT

PTTIFWTDYVATRWYRAPELCGSFFSKYTPAIDVWSIGCIFAEVLTGKPLFPGKNVVHQL

DLMTDLLGTPSMDTISRVRNEKARRYLSSMRKKEPIPFSQKFPNADPLALDLLQRLLAFD

PKDRPTAGEALSHPYFKGLAKVEREPSCQPITKMEFEFERRRATKEDIRELIFREILEYH

PQLLKDYINGTERTTFLYPSAVDQFRKQFAHLEENSESGPVVPMDRKHTSLPRSTIVHSA

PIHAKEQPRIGPSRDRPLTDESYKNPRDSEKYSGNVPRSSHHSQAPQRVPTARPGRVVGP

VLPYENVGTSHPYDPRRVAMNSGYPPQQQIPQTYGYYQTTGKSACSEPSQAERYTLHQQA

YACANSSTAPDVALDMRAPPFHQSAGPKSGSSDRLTAETNLYTRSLNGIVATTSGVAASA

HRKVSVVPYGMSQMY*

>Kah-5MPK20-2 Brdisv1Kah-51015861m.p

MQQPQQQGQRNKLPDGWFCCNVHILNLCRSAMIFLTPPIIMPSTDVDFFSEYGDANRYRI

QEVIGKGSYGVVCSAMDMQTRQKVAIKKIHNIFDHVSDAARILREIKLLRLLRHPDVVEI

KHIMLPPSRKDFKDIYVVFELMESDLHQVIKANDDLTKEHYQFFLYQLLRALKYIHTASV

YHRDLKPKNILANSNCKLKICDFGLARVAFNDTPTTVFWTDYVATRWYRAPELCGSFFTK

YTPAIDIWSIGCIFAEVLTGKPLFPGKNVVHQLDLMTDLLGTPSMDTISRVRNEKARRYL

SSMRKKDPVPFSKKFPNADPLGLKLLEKLLAFDPKDRPTAEEALTDPYFKSLSKPDREPS

CQPIRKVEFDFEHRRMSKDDIRELIFQEILEYHPQLLKNYIDGTEKTTFLYPSAVDQFKK

QFSHLEESDGSGPVVPTERKHASLPRSTTVHSTPIPAKEQPLVASSRGRPIANEPCKPWA

PGNVPGASQTAHVAQAGRAVGVGSVPPYESGSGKYPYDATSRPAVSSGYPPQQKIPQTYG

YHHHHQTPGAGQSSQAMGGYACGYTKGTTPPPAAAQDMRASPYHHRSAGTKNDPLNRLAA

ESDIYTRSLNGIVAAAASAGTGAHRKVGAVPFGMSGMY*

>Kah-5MPK20-3 Brdisv1Kah-51045497m.p

MQHGDLHKKSAAEMDFFTAYDDANRYKILEVIGKGSYGLVCSANDLQTGEKVAIKKIHNI

FEHISDAARILREIKLLRLLRHPDVVEIKHILLPPSKKDFKDIYVVFELMESDLHQVIKA

NDDLTREHYQFFLYQMLRALKYMHTANVYHRDLKPKNVLANANCKLKICDFGLARVAFSD

APTTVFWTDYVATRWYRAPELCGSFYSK

>Kah-5MPK20-4 Brdisv1Kah-51020414m.p

MQTTEQQRKKGSSEMDFFSEYGDANRYKIQEVIGKGSYGVVCSAIDQHTGDKVAIKKIHN

IFEHLSDAARILREIKLLRLLRHPDIVEIRHIMLPPSRRDFKDIYVVFELMDTDLHQVIK

ANDDLTKEHHQFFLYQMLRALKYIHTANVYHRDLKPKNILANANCKLKICDFGLARVAFN

DTPTTVFWTDYVATRWYRAPELCGSFFTKYSRAIDIWSIGCIFAEILTGKPLFPGKNVVH

QLDLMTDLLGTPSLDTVSRIRNEKARRYLSSMRKKQSVSFSERFPKADPAALKLLQRLLA

FDPKDRPTAEEALADPYFKGLGKVEREPSCQPITKMEFEFERKNVTKADVKELIFREILE

YHPQLLKDYMNGTEKTNFLYPSAVDNFRRQFANLEENGGKGGAVVPPDRKHVSLPRNTTV

HSTPIPPKDQKYSQVPQRIPTGRPGRVVGPVIPFENSSTMDPYSQRRVARNPVLPAAATN

LSAYAYRNSDNSERELQQELEKDRMQYHPMQRFMDAKMVSPDLRSTSYYMPKGVPKADVA

ERSALQSNMMQGIAPFNGIATVGGAFNKVSAVQYGVSRMY*

>Kah-5MPK20-5 Brdisv1Kah-51016005m.p

MFSDIFSREGVAFSRVALRQELLSDQWFTTAGGKGQCYLVPPGTTVFLVNSEEMGFFSEY

VDASRYKILEIIGKGSYGVVCSAIDQETGDKVAIKKIQNIFEHLSDAARILREIKLLRLL

RHPDIVQIKHIMLPPSRRDFRDIYVVFELMDTDLHQVIKANDDLTKEHYQFFLYQMLRAL

KYIHTANVYHRDLKPKNILANANCKLKICDFGLARVAFNDTPTTVFWTDYVATRWYRAPE

LCGSFFTKYSPAIDTWSIGCIFAEILTGKPLFPGKNVVHQLDLMTDLLGTPSTETISRIR

NDKARKYLSSMRRKQPIPFSEKFPNADPSALKLLERLLAFDPKDRPTAEEALAHPYFKRL

ARVEREPSCQQPISKTEFEFERRKFTKEDVKELIFREILEYHPKLLKDYMNGSEKTSFLY

PSAVDNFRRQFANLEIDGGRSGAADRKHFSLPRTTTVHSAPILPTNGPTSQVPQRIPTAR

PGRVVSSAMQTDNPSVSDRHNGRRVARDPAVPPAAAYHLKSDYSDRQHQQEFEKDRVRPD

RQRQEELEKDRMQYRPGHHSMDAKVAPEISPYMRSSPYYIPPFNGIAAVASGYSKVAAVT

RMY*

>Kah-5MPK21-1 Brdisv1Kah-51015904m.p

MGGLIRWLRHHRSRRVSSSSSSHLPSNTTSSSSTSDLRAHSLPQHQGDHHGEVVVEWEDA

AEGPDSDPEEYIVVVLGDDEQGVVAARAPVRTKPPRVMDPGKKTSESEFFTEYGEANRYK

VSEVIGKGSYGVVAAAVDTQTGERCAIKKINDVFDHVSDATRILREIKLLRLLRHPDIVE

IKHIMLPPSRREFRDIYVVFELMESDLHQVIKANDDLTPEHHQFFLYQLLRGMKYIHTAS

VFHRDLKPKNILANADCKLKICDFGLARVSFNDGAPSAIFWTDYVATRWYRAPELCGSFF

SKYTPAIDIWSVGCIFAEMLTGKPLFPGKNVVHQLDLMTDLLGTPSAESLSKIRNEKARR

YLSNMRKKPKVPLTKKFPGIDPMALHLLERLLAFDPKDRPSADEALTDPYFTGLANSERE

PITQPISKLEFEFEKRKLAKDDVRELIYREILEYHPHMLQEYLRGGGDQMSFMFPSGVDR

FKRQFAHLEEGGAKGEKSSPQLRQNASLPRERVIGNKHGDGDYNMKLNVGEKPELASVSD

GISKPLMSARSLLKSETMSASKCIGEIKNKDEDSLSECVEGTDDDVSQKIAQLKT*

>Kah-5MPK21-2 Brdisv1Kah-51020257m.p

MARKGSDEAEFFTEYGEANRYEVGEVVGKGSYGVVAAAVDTHTGERVAIKKINDVFEHVS

DATRILREIKLLRLLRHPDIVEIKHIILPPSRREFRDIYIIFELMESDLHQVIKANDDLT

PEHHQFFFYQLLRGMKYIHAANVFHRDLKPRNILANADCKLKICDFGLARVSFNDTPSAI

FWTDYVATRWYRAPELCGSFFSKYTPAIDIWSIGCIFAEMLSGRPLFPGKNVVHQLDLMT

DLLGTPSAESLSRIRNEKARRYLGNMRKKHPVPFSQKFPGVDPMALDLLERLLAFDPKDR

PTAAEALADPYFTGLANSDREPTTQPISKLEFEFERRKLARDDVRELIYREILEYHPQML

HEYHHGGDQANFVYPSGVDRFKRQFVHLEEGVTKGEKTSPQLRQHASLPRERIIGIGDEL

GRPNADYCIKLHVGEEPGHTSVTDGLSKPLLNARNFLKSESISASQCVVIKEKREKDEES

MSEYMHEASDATPK*

>BdTR5iMPK3 Brdisv1BdTR5I1009348m.p

MDGAPVAEFRPTMTHGGRVLLYNIFGNQFEITAKYQPPIMPIGRGAYGIVCSVMNFETRE

MVAIKKIANAFDNNMDAKRTLREIKLLRHLDHENIVGLRDVIPPAIPQSFNDVYIATELM

DTDLHHIIRSNQELSEEHCQYFLYQLLRGLKYIHSANVIHRDLKPSNLLLNANCDLKICD

FGLARPSSESDMMTEYVVTRWYRAPELLLNSTDYSAAIDVWSVGCIFMELINRAPLFPGR

DHMHQMRLITEVIGTPTDDDLGFIRNEDARRYMRHLPQFPRRPFPAQFPRVQPAALDLIE

RMLAFNPLQRITVEEAREHPYLERLHDIADEPICTDPFSFDFEQHPLTEDQMKQLIFNEA

LELNPNFRY*

>BdTR5iMPK4 Brdisv1BdTR5I1030484m.p

MDTSGGGGGGAGGGAQIQGMATHGGRYVLYNVYGNLFEVASKYAPPIRPIGRGAYGIVCA

AVSSDTGEEVAIKKIGNAFDNHIDAKRTLREIKLLRHMDHENIIAIKDIIRPPRRDDFKD

VYIVTELMDTDLHQIIRSNQPLTDDHCQYFLYQLLRGLKYVHSANVLHRDLKPSNLFLNA

NCDLKIADFGLARTTSETDLMTEYVVTRWYRAPELLLNCSQYTAAIDVWSVGCILGEIIT

RQPLFPGRDYIQQLKLITELIGSPDDSSLGFLRSDNARRYMKQLPQYPRQDFRLRFRNMS

DGAVDLLERMLVFDPSRRITVDEALHHPYLASLHDINEEPTCPAPFSFDFEQPSFTEEHM

KELIWRETLAFNPDPPY*

>BdTR5iMPK6 Brdisv1BdTR5I1006823m.p

MDGGAQPPDAEMADAGAAVPGAGAGAAGTMENIQATLSHGGRFIQYNIFGNVFEVTVKYK

PPILPIGKGAYGIVCSALNSETGEQVAIKKIANAFDNKIDAKRTLREIKLLRHMDHENIV

AIRDIIPPAQRNSFNDVYIAYELMDTDLHQIIRSNQALSEEHCQYFLYQILRGLKYIHSA

NVLHRDLKPSNLLLNANCDLKICDFGLARTTSETDFMTEYVVTRWYRAPELLLNSSEYTA

AIDVWSVGCIFMELMDRKPLFPGRDHVHQLRLLMELIGTPNEADLDFVNENARRYIRQLP

RHARQSFSEKFPHVHPSAIDLVEKMLTFDPRQRITVEGALAHPYLASLHDISDEPVCTMP

FSFDFEQHALSEEQMKDLIHQEALAFNPDYQ*

>BdTR5iMPK7-1 Brdisv1BdTR5I1004945m.p

MPSRFPFLSPGCFIRLLSGSASSDLLLLLPRRGQSAPRARVLLKMAMMVDPPNGTGNHGK

HYYTMWQTMFEIDTKYVPIKPIGRGAYGIVCSSTNQENNEKVAIKKINNVFDNRVDALRT

LRELKLLRHLRHENVIALKDIMMPVHRRSFKDVYLVSELMDTDLHQIIKSSQPLSNDHCQ

YFLFQLLRGLKYLHSAGILHRDLKPGNLLVNANCDLKICDFGLARTNNTKGQFMTEYVVT

RWYRAPELLLCCDNYGTSIDVWSVGCIFAELLGRKPIFPGTECLNQLKLIVNVLGTMSEN

DLEFIDNPKARKYIKSLPYPPGTPLTSMYPQAHPLAIDLLQKMLVFDPSKRISVTEALEH

PYMSPLYDPSANPPAQVPIDLDIDENLGVEMIREMLWQGMLQYHPEAARMVNM*

>BdTR5iMPK11 Brdisv1BdTR5I1028828m.p

MRMEGGGAGPAAAAAAGGAHGLGEAQIKGTLTHGGRYVQYNVYGNLFEVSAKYVPPIRPV

GRGACGIICAAINVQTREEVAIKKIGNAFDNQIDAKRTLREVKLLRHMNHENVISIKDII

RPPRRENFNDVYIVYELMDTDLHHLLRSNQPLTDDHCQYFLYQVLRGLKYVHSANVLHRD

LRPSNLLLNAKCDLKIGDFGLARTTTETDFMMEYVVTRWYRAPELLLNCSEYTGAIDMWS

VGCILGEIATREPLFPGKDYVHQLRLITELLGSPDDTSLGFLRSDNARRYVRALPQYPKQ

QFRSRFPNMSSGAMDLLERMLVFDPNKRITVDEALCHPYLASLHEINDEPVCPAPFSFDF

EQPSFTEEDIKELIWRESVKFNPEPIH*

>BdTR5iMPK14 Brdisv1BdTR5I1026907m.p

MAMLVDPPNGMGNQGKHYYSMWQTLFEIDTKYVPIKPIGRGAYGIVCSSINRETNEKVAI

KKIHNVFDNRVDALRTLRELKLLRHLRHENVISLKDIMMPVQRRSFKDVYLVYELMDTDL

HQIIKSPQGLSNDHCQYFLFQLLRGLKYLHSAEILHRDLKPGNLLVNANCDLKICDFGLA

RTNSSKGQFMTEYVVTRWYRAPELLLCCDNYGTSIDVWSVGCIFAELLGRKPIFPGTECL

NQLKLIVNVLGTMSESDLEFIDNPKARRYIKTLPYTPGVPLASMYPHAHPLAIDLLQKML

IFDPTKRISVTEALEHPYMSPLYDPSANPPAQVPIDLDLDENISADMIREMMWQEMLHYH

PEAATAISM*

>BdTR5iMPK16 Brdisv1BdTR5I1039980m.p

MDFFTEYGEGNRYKIEEVIGKGSYGVVCSALDTHTGDKVAIKKINDIFEHVSDATRILRE

IKLLRLLRHPDIVEIKHILLPPSRREFKDIYVVFELMESDLHQVIKANDDLTPEHYQFFL

YQLLRGLKYIHTANVFHRDLKPKNILANADCKLKICDFGLARVAISDTPPAIFWTDYIAT

RWYRAPELCGSFFSKYTPAIDIWSIGCIFAELLTGKPLFPGKNVVHQLDIITDLLGTPSA

ETIARIRNEKARRYLSSMRRKKTVPFTQKFPNADPLALNLLERMLAFDPKDRPSAEEALA

DPYFKNIASVDREPSAQPITKLEFEFERRRITKDDIRELIYREILEYHPNMLREFLEGAE

PTGFMYPSAVDHFKKQFTFLEEHYAKGSTAAPPERQHNSLPRPSVIYSDNRPQNAANITD

DLSRCAIRDNTQKAQKDSASVGANRIPHGAAAAAARPGKVVGSVLRYGNCSTSGTEQYEQ

RRVVRSPGIAPNGVTSGSSYPRRNNTCKSETGEAERIDTNQGGPPKPYVANKLPATVDGR

NGHW*

>BdTR5iMPK17 Brdisv1BdTR5I1005052m.p

MVGGGGIVDGFRRLFPRRTPSGSGPSSNQSSAGEESSDIDAVVEDLDLVGLRAIRVPKRK

MPLPVESHKKNILEKEFFTEYGEASQYQIQEVIGKGSYGVVAAAVDTRTGERVAIKKIND

VFEHVSDATRILREVKLLRLLRHPDVVEIKHIMLPPSRREFQDIYVVFELMESDLHQVIR

ANDDLTPEHYQFFLYQLLRALKYIHAANVFHRDLKPKNILANSDCKLKICDFGLARVSFN

DAPTAIFWTDYVATRWYRAPELCGSFFSKYTPAIDIWSIGCIFAELLTGRPLFPGKNVVH

QLDIITDLLGTPSSETLSRIRNEKARRYLSCMRKKHPVPLTQKFPNVDPLALRLLGRLLA

FDPKDRPSAEEALADPYFAVLANVEREPSRNPISKLEFEFERRKVTKDDVRELIYREILE

YHPQMLQEYMQGGEQLSFLYPSGVDRFKRQFAHLEENYSKGERGSPLQRKHASLPRQRVG

ASNDNNEQHASDQERGADSIAPATGNPPGSQDAGQQHVSGGQNGVGSTNISPRSYQKSAS

ISASKCVVVNANKNPEYDDDISEEMEGAVDGLSEKVSRMHP*

>BdTR5iMPK20-1 Brdisv1BdTR5I1017346m.p

MQQDQRKKSSAEAEFFTEYGDASRYKIQEIVGKGSYGVVCSAIDVHTGEKVAIKKIHDIF

EHISDAARILREIKLLRLLRHPDIVEIKHIMLPPSRRDFKDIYVVFELMESDLHQVIKAN

DDLTKEHYQFFLYQLLRALKYIHTANVYHRDLKPKNILANSNCKLKICDFGLARVAFNDT

PTTIFWTDYVATRWYRAPELCGSFFSKYTPAIDVWSIGCIFAEGLTGKPLFPGKNVVHQL

DLMTDLLGTPSMDTISRVRNEKARRYLSSMRKKEPIPFSQKFPNADPLALDLLQRLLAFD

PKDRPTAGEALSHPYFKGLAKVEREPSCQPITKMEFEFERRRATKEDIRELIFREILEYH

PQLLKDYINGTERTTFLYPSAVDQFRKQFAHLEENSESGPVVPMDRKHTSLPRSTIVHSA

PIHAKEQPRIGPSRDRPLTDESYKNPRDSEKYSGNVPRSSHHSQAPQRVPTARPGRVVGP

VLPYENVGTSHPYDPRRVAMNSGYPPQQQIPQTYGYYQTTGKSACSEPSQAERYTLHQQA

YACANSSTAPDVALDMRAPPFHQSAGPKSGSSDRLTAETNLYTRSLNGIVATTSGVAASA

HRKGSVVPYGMSQMY*

>BdTR5iMPK20-2 Brdisv1BdTR5I1040580m.p

MQQPQQQGQRNKSTDVDFFSEYGDANRYRIQEVIGKGSYGVVCSAMDMQTRQKVAIKKIH

NIFDHVSDAARILREIKLLRLLRHPDVVEIKHIMLPPSRKDFKDIYVVFELMESDLHQVI

KANDDLTKEHYQFFLYQLLRALKYIRTASVYHRDLKPKNILANSNCKLKICDFGLARVAF

NDTPTTVFWTDYVATRWYRAPELCGSFFTKYTPAIDIWSIGCIFAEVLTGKPLFPGKNVV

HQLDLMTDLLGTPSMDTISRVRNEKARRYLSSMRKKDPVPFSKKFPNADPLGLKLLEKLL

AFDPKDRPTAEEALTDPYFKSLSKPDREPSCQPIRKVEFDFEHRRMSKDDIRELIFQEIL

EYHPQLLKNYIDGTEKTTFLYPSAVDQFKKQFSHLEESDGSGPVVPTERKHASLPRSTTV

HSTPIPAKEQPLVASSRGRPIANEPCKPWAPGNVPGASQTAHVAQAGRAVGVGSVPPYES

GSGKYPYDATSRPAVSSGYPPQQKIPQTYGYHHHHQTPGAGQSSQAMGGYACGYTKGTTP

PPAAAQDMRASPYHHRSAGTKNDPLNRLAAESDIYTRSLNGIVAAAASAGTGAHRKVGAV

PFGMSGMY*

>BdTR5iMPK20-3 Brdisv1BdTR5I1041219m.p

MQHGDLHKKSAAEMDFFTAYDDANRYKILEVIGKGSYGLVCSANDLQTGEKVAIKKIHNI

FEHISDAARILREIKLLRLLRHPDVVEIKHILLPPSKKDFKDIYVVFELMESDLHQVIKA

NDDLTREHYQFFLYQMLRALKYMHTANVYHRDLKPKNVLANANCKLKICDFGLARVAFSD

APTTVFWTDYVATRWYRAPELCGSFYSKVMIVTIDH*

>BdTR5iMPK20-4 Brdisv1BdTR5I1017548m.p

MQTTEQQRKKGSSEMDFFSEYGDANRYKIQEVIGKGSYGVVCSAIDQHTGDKVAIKKIHN

IFEHLSDAARILREIKLLRLLRHPDIVEIRHIMLPPSRRDFKDIYVVFELMDTDLHQVIK

ANDDLTKEHHQFFLYQMLRALKYIHTANVYHRDLKPKNILANANCKLKICDFGLARVAFN

DTPTTVFWTDYVATRWYRAPELCGSFFTKYSRAIDIWSIGCIFAEILTGKPLFPGKNVVH

QLDLMTDLLGTPSLDTVSRIRNEKARRYLSSMRKKQSVSFSERFPKADPAALKLLQRLLA

FDPKDRPTAEEALADPYFKGLGKVERDPPCQPITKMEFEFERKNVTKADVKELIFREILE

YHPQLLKDYMNGTEKTNFLYPSAVDNFRRQFANLEENGGKGGAVVPPDRKHVSLPRNTTV

HSTPIPPKDQKYSQVPQRIPTGRPGRVVGPVIPFENSSTMDPYSQRRVARNPVLPAAATN

LSAYAYRNSDNSERELQQELEKDRMQSHPMQRFMDAKMVSPDLRSTSYYMPKGVPKADVA

ERSALQSNMMQGIAPFNGIATVGGAFNKVSAVQYGVSRMY*

>BdTR5iMPK20-5 Brdisv1BdTR5I1013896m.p

MFSDIFSREGVAFSRVALRQELLSDQWFTTAGGKGQCYLVPPGTTVFLVNSEEMGFFSEY

VDASRYKILEIIGKGSYGVVCSAIDQETGDKVAIKKIQNIFEHLSDAARILREIKLLRLL

RHPDIVQIKHIMLPPSRRDFRDIYVVFELMDTDLHQVIKANDDLTKEHYQFFLYQMLRAL

KYIHTANVYHRDLKPKNILANANCKLKICDFGLARVAFNDTPTTVFWTDYVATRWYRAPE

LCGSFFTKYSPAIDTWSIGCIFAEILTGKPLFPGKNVVHQLDLMTDLLGTPSTETISRIR

NDKARKYLSSMRRKQPIPFSEKFPNADPSALKLLERLLAFDPKDRPTAEEALAHPYFKRL

ARVEREPSCQQPISKTEFEFERRKFTKEDVKELIFREILEYHPKLLKDYMNGSEKTSFLY

PSAVDNFRRQFANLEIDGGRSGAADRKHFSLPRTTTVHSAPILPTNGPTSQVPQRIPTAR

PGRVVSSAMQTDNPSVSDRHNGRRVARDPAVPPAAAYHLKSDYSDRQHQQEFEKDRVRPD

RQRQEELEKDRMQYRPGHHSMDAKVAPEISPYMRSSPYYIPPFNGIAAVASGYSKVAAVT

RMY*

>BdTR5iMPK21-1 Brdisv1BdTR5I1013788m.p

MGGLIRWLRPHRSRRVSSSSSSHLPSNTTSSSSTSDLRAHSLPQHQGDHHGEVVVEWEDA

AEGPDSDPEEYIVVVLGDDEQGVVAARAPVRTKPPRVMDPGKKTSESEFFTEYGEANRYK

VSEVIGKGSYGVVAAAVDTQTGERCAIKKINDVFDHVSDATRILREIKLLRLLRHPDIVE

IKHIMLPPSRREFRDIYVVFELMESDLHQVIKANDDLTPEHHQFFLYQLLRGMKYIHTAS

VFHRDLKPKNILANADCKLKICDFGLARVSFNDGAPSAIFWTDYVATRWYRAPELCGSFF

SKYTPAIDIWSVGCIFAEMLTGKPLFPGKNVVHQLDLMTDLLGTPSAESLSKIRNEKARR

YLSNMRKKPKVPLTKKFPGIDPMALHLLERLLAFDPKDRPSADEALTDPYFTGLANSERE

PITQPISKLEFEFEKRKLAKDDVRELIYREILEYHPHMLQEYLRGGGDQMSFMFPSGVDR

FKRQFAHLEEGGAKGEKSSPQLRQNASLPRERVIGNKHGDGDYNMKLNVGEKPELASVSD

GISKPLMSARSLLKSETMSASKCIGEIKNKDEDSLSECVEGTDDDVSQKIAQLKT*

>BdTR5iMPK21-2 Brdisv1BdTR5I1017428m.p

MARKGSDEAEFFTEYGEANRYEAGEVVGKGSYGVVAAAVDTHTGERVAIKKINDVFEHVS

DATRILREIKLLRLLRHPDIVEIKHIILPPSRREFRDIYIIFELMESDLHQVIKANDDLT

PEHHQFFFYQLLRGMKYIHAANVFHRDLKPRNILANADCKLKICDFGLARVSFNDTPSAI

FWTDYVATRWYRAPELCGSFFSKYTPAIDIWSIGCIFAEMLSGRPLFPGKNVVHQLDLMT

DLLGTPSAESLSRIRNEKARRYLGNMRKKHPVPFSQKFPGVDPMALDLLERLLAFDPKDR

PTAAEALADPYFTGLANSDREPTTQPISKLEFEFERRKLARDDVRELIYREILEYHPQML

HEYHHGGDQANFVYPSGVDRFKRQFVHLEEGVTKGEKTSPQLRQHASLPRERIIGIGDEL

GRPNADYCIKLHVGEEPGHTSVTDGLSKPLLNARNFLKSESISASQCVVIKEKREKDEES

MSEYMHEASDATPK*

>BdTR10cMPK3 Brdisv1BdTR10C1009137m.p

MDGAPVAEFRPTMTHGGRVLLYNIFGNQFEITAKYQPPIMPIGRGAYGIVCSVMNFETRE

MVAIKKIANAFDNNMDAKRTLREIKLLRHLDHENIVGLRDVIPPAIPQSFNDVYIATELM

DTDLHHIIRSNQELSEEHCQYFLYQLLRGLKYIHSANVIHRDLKPSNLLLNANCDLKICD

FGLARPSSESDMMTEYVVTRWYRAPELLLNSTDYSAAIDVWSVGCIFMELINRAPLFPGR

DHMHQMRLITEVIGTPTDDDLGFIRNEDARRYMRHLPQFPRRPFPAQFPRVQPAALDLIE

RMLAFNPLQRITVEEAREHPYLERLHDIADEPICTDPFSFDFEQHPLTEDQMKQLIFNEA

LELNPNFRY*

>BdTR10cMPK4 Brdisv1BdTR10C1023812m.p

MDTSGGGGAGGAAQIQGMATHGGRYVLYNVYGNLFEVASKYAPPIRPIGRGAYGIVCAAV

SSDTGEEVAIKKIGNAFDNHIDAKRTLREIKLLRHMDHENIIAIKDIIRPPRRDDFKDVY

IVTELMDTDLHQIIRSNQPLTDDHCQYFLYQLLRGLKYVHSANVLHRDLKPSNLFLNANC

DLKIADFGLARTTSETDLMTEYVVTRWYRAPELLLNCSQYTAAIDVWSVGCILGEIITRQ

PLFPGRDYIQQLKLITELIGSPDDSSLGFLRSDNARRYMKQLPQYPRQDFRLRFRNMSDG

AVDLLERMLVFDPSRRITVDEALHHPYLASLHDINEEPTCPAPFSFDFEQPSFTEEHMKE

LIWRETLAFNPDPPY*

>BdTR10cMPK6 Brdisv1BdTR10C1006602m.p

MADAGAAVPGAGAGAAGTMENIQATLSHGGRFIQYNIFGNVFEVTVKYKPPILPIGKGAY

GIVCSALNSETGEQVAIKKIANAFDNKIDAKRTLREIKLLRHMDHENIVAIRDIIPPAQR

NSFNDVYIAYELMDTDLHQIIRSNQALSEEHCQYFLYQILRGLKYIHSANVLHRDLKPSN

LLLNANCDLKICDFGLARTTSETDFMTEYVVTRWYRAPELLLNSSEYTAAIDVWSVGCIF

MELMDRKPLFPGRDHVHQLRLLMELIGTPNEADLDFVNENARRYIRQLPRHARQSFSEKF

PHVHPSAIDLVEKMLTFDPRQRITVEGALAHPYLASLHDISDEPVCTMPFSFDFEQHALS

EEQMKDLIHQEALAFNPDYQ*

>BdTR10cMPK7-1 Brdisv1BdTR10C1031091m.p

MPSRFPFLSPGCFIRLLSGSASSDLLLLLPRRGQSAPRARVLLKMAMMVDPPNGTGNHGK

HYYTMWQTMFEINTKYMPIKPIGRGAYGIVCSSTNQENNEKVAIKKINNVFDNRVDALRT

LRELKLLRHLRHENVIALKDIMMPVHRRSFKDVYLVFELMDTDLHQIIKSSQPLSNDHCQ

YFLFQLLRGLKYLHSAGILHRDLKPGNLLVNANCDLKICDFGLARTNNTKGQFMTEYVVT

RWYRAPELLLCCDNYGTSIDVWSVGCIFAELLGRKPIFPGTECLNQLKLIVNVLGTMSEN

DLEFIDNPKARKYIKSLPYTPGTPLTSMYPQAHPLAIDLLQKMLVFDPSKRISVTEALEH

PYMSPLYDPSANPPAQVPIDLDIDENLGVEMIREMLWQEMLQYHPEAARMVNM*

>BdTR10cMPK11 Brdisv1BdTR10C1022204m.p

MRMEGGGAGPAAAAAGGGAHGLGEAQIKGTLTHGGRYVQYNVYGNLFEVSAKYVPPIRPV

GRGACGIICAAINVQTREEVAIKKIGNAFDNQIDAKRTLREVKLLRHMNHENVISIKDII

RPPRRENFNDVYIVYELMDTDLHHLLRSNQPLTDDHCQYFLYQVLRGLKYVHSANVLHRD

LRPSNLLLNAKCDLKIGDFGLARTTTETDFMMEYVVTRWYRAPELLLNCSEYTGAIDMWS

VGCILGEIATREPLFPGKDYVHQLRLITELLGSPDDTSLGFLRSDNARRYVRSLPQYPKQ

QFRSRFPNMSSGAMDLLERMLVFDPNKRITVDEALCHPYLASLHEINDEPVCPAPFSFDF

EQPSFTEEDIKELIWRESVKFNPEPIH*

>BdTR10cMPK14 Brdisv1BdTR10C1020364m.p

MAMLVDPPNGMGNQGKHYYSMWQTLFEIDTKYVPIKPIGRGAYGIVCSSINRETNEKVAI

KKIHNVFDNRVDALRTLRELKLLRHLRHENVISLKDIMMPVQRRSFKDVYLVYELMDTDL

HQIIKSPQGLSNDHCQYFLFQLLRGLKYLHSAEILHRDLKPGNLLVNANCDLKICDFGLA

RTNSSKGQFMTEYVVTRWYRAPELLLCCDNYGTSIDVWSVGCIFAELLGRKPIFPGTECL

NQLKLIVNVLGTMSESDLEFIDNPKARRYIKTLPYTPGVPLASMYPHAHPLAIDLLQKML

IFDPTKRISVTEALEHPYMSPLYDPSANPPAQVPIDLDLDENISADMIREMMWQEMLHYH

PEAATAISM*

>BdTR10cMPK16 Brdisv1BdTR10C1015705m.p

MDFFTEYGEGNRYKIEEVIGKGSYGVVCSALDTHTGDKVAIKKINDIFEHVSDATRILRE

IKLLRLLRHPDIVEIKHILLPPSRREFKDIYVVFELMESDLHQVIKANDDLTPEHYQFFL

YQLLRGLKYIHTANVFHRDLKPKNILANADCKLKICDFGLARVAISDTPTAIFWTDYIAT

RWYRAPELCGSFFSKYTPAIDIWSIGWIFAELLTGKPLFPGKNVVHQLDIITDLLGTPSA

ETIARIRNEKARRYLSSMRRKKTVPFTQKFPNADPLALNLLERMLAFDPKDRPSAEEALA

DPYFKNIASVDREPSAQPITKLEFEFERRRITKDDIRELIYREILEYHPNMLREFLEGAE

PTGFMYPSAVDHFKKQFTFLEEHYAKGSTAAPPERQHNSLPRPSVIYSDNRPQNAANITD

DLSRCAIRDNTQKAQKDSASVGANRIPHGAAAAAARPGKVVGSVLRYGNCSTSGTEQYEQ

RRVVRSPGIAPNGVTSGSSYPRRNNTCKSETGEAERIDTNQGGPPKPYVANKLPATVDGR

NGHW*

>BdTR10cMPK17 Brdisv1BdTR10C1004902m.p

MPLPVESHKKNILEKEFFTEYGEASQYQIQEVIGKGSYGVVAAAVDTRTGERVAIKKIND

VFEHVSDATRILREVKLLRLLRHPDVVEIKHIMLPPSRREFQDIYVVFELMESDLHQVIR

ANDDLTPEHYQFFLYQLLRALKYIHAANVFHRDLKPKNILANSDCKLKICDFGLARVSFN

DAPTAIFWTDYVATRWYRAPELCGSFFSKYTPAIDIWSIGCIFAELLTGRPLFPGKNVVH

QLDIITDLLGTPSSETLSRIRNEKARRYLSCMRKKHPVPLTQKFPNVDPLALRLLGRLLA

FDPKDRPSAEEALADPYFAVLANVEREPSRNPISKLEFEFERRKVTKDDVRELIYREILE

YHPQMLQEYMQGGEQLSFLYPSGVDRFKRQFAHLEENYSKGERGSPLQRKHASLPRQRVG

ASNDNNEQHASDQERGADSIAPATGNPPGSQDAGQQHVSGGQNGVGSTNISPRSYQKSAS

ISASKCVVVNANKNPEYDDDISEEMEGAVDGLSEKVSRMHP*

>BdTR10cMPK20-1 Brdisv1BdTR10C1016940m.p

MQQDQRKKSSAEAEFFTEYGDASRYKIQEIVGKGSYGVVCSAIDVHTGEKVAIKKIHDIF

EHISDAARILREIKLLRLLRHPDIVEIKHIMLPPSRRDFKDIYVVFELMESDLHQVIKAN

DDLTKEHYQFFLYQLLRALKYIHTANVYHRDLKPKNILANSNCKLKICDFGLARVAFNDT

PTTIFWTDYVATRWYRAPELCGSFFSKYTPAIDVWSIGCIFAEVLTGKPLFPGKNVVHQL

DLMTDLLGTPSMDTISRVRNEKARRYLSSMRKKEPIPFSQKFPNADPLALDLLQRLLAFD

PKDRPTAGEALSHPYFKGLAKVEREPSCQPITKMEFEFERRRATKEDIRELIFREILEYH

PQLLKDYINGTERTTFLYPSAVDQFRKQFAHLEENSESGPVVPMDRKHTSLPRSTIVHSA

PIHAKEQPRIGPSRDRPLTDESYKNPRDSEKYSGNVPRSSHHSQAPQRVPTARPGRVVGP

VLPYENVGTSHPYDPRRVAMNSGYPPQQQIPQTYGYYQTTGKSACSEPSQAERYTLHQQA

YACANSSTAPDVALDMRAPPFHQSAGPKSGSSDRLTAETNLYTRSLNGIVATTSGVAASA

HRKVSVVPYGMSQMY*

>BdTR10cMPK20-2 Brdisv1BdTR10C1013382m.p

MQQPQQQGQRNKSTDVDFFSEYGDANRYRIQEVIGKGSYGVVCSAMDMQTRQKVAIKKIH

NIFDHVSDAARILREIKLLRLLRHPDVVEIKHIMLPPSRKDFKDIYVVFELMESDLHQVI

KANDDLTKEHYQFFLYQLLRALKYIHTASVYHRDLKPKNILANSNCKLKICDFGLARVAF

NDTPTTVFWTDYVATRWYRAPELCGSFFTKYTPAIDIWSIGCIFAEVLTGKPLFPGKNVV

HQLDLMTDLLGTPSMDTISRVRNEKARRYLSSMRKKDPVPFSKKFPNADPLGLKLLEKLL

AFDPKDRPTAEEALTDPYFKSLSKPDREPSCQPIRKVEFDFEHRRMSKDDIRELIFQEIL

EYHPQLLKNYIDGTEKTTFLYPSAVDQFKKQFSHLEESDGSGPVVPTERKHASLPRSTTV

HSTPIPAKEQPLVASSRGRPIANEPCKPWAPGNVPGASQTAHVAQAGRAVGVGSVPPYES

GSGKYPYDATSRPAVSSGYPPQQKIPQTYGYHHHHQTPGAGQSSQAMGGYACGYTKGTTP

PPAAAQDMRASPYHHRSAGTKNDPLNRLAAESDIYTRSLNGIVAAAASAGTGAHRKVGAV

PFGMSGMY*

>BdTR10cMPK20-3 Brdisv1BdTR10C1041496m.p

MQHGDLHKKSAAEMDFFTAYDDANRYKILEVIGKGSYGLVCSANDLQTGEKVAIKKIHNI

FEHISDAARILREIKLLRLLRHPDVVEIKHILLPPSKKDFKDIYVVFELMESDLHQVIKA

NDDLTREHYQFFLYQMLRALKYMHTANVYHRDLKPKNVLANANCKLKICDFGLARVAFSD

APTTVFWTDYVATRWYRAPELCGSFYSKVMIVTIDH*

>BdTR10cMPK20-4 Brdisv1BdTR10C1017120m.p

MQTTEQQRKKGSSEMDFFSEYGDANRYKIQEVIGKGSYGVVCSAIDQHTGDKVAIKKIHN

IFEHLSDAARILREIKLLRLLRHPDIVEIRHIMLPPSRRDFKDIYVVFELMDTDLHQVIK

ANDDLTKEHHQFFLYQMLRALKYIHTANVYHRDLKPKNILANANCKLKICDFGLARVAFN

DTPTTVFWTDYVATRWYRAPELCGSFFTKYSRAIDIWSIGCIFAEILTGKPLFPGKNVVH

QLDLMTDLLGTPSLDTVSRIRNEKARRYLSSMRKKQSVSFSERFPKADPAALKLLQRLLA

FDPKDRPTAEEALADPYFKGLGKVEREPSCQPITKMEFEFERKNVTKADVKELIFREILE

YHPQLLKDYMNGTEKTNFLYPSAVDNFRRQFANLEENGGKGGAVVPPDRKHVSLPRNTTV

HSTPIPPKDQKYSQVPQRIPTGRPGRVVGPVIPFENSSTMDPYSQRRVARNPVLPAAATN

LSAYAYRNSDNSERELQQELEKDRMQYHPMQRFMDAKMVSPDLRSTSYYMPKGVPKADVA

ERSALQSNMMQGIAPFNGIATVGGAFNKVSAVQYGVSRMY*

>BdTR10cMPK20-5 Brdisv1BdTR10C1013523m.p

MFSDIFSREGVAFSRVALRQELLSDQWFTTAGGKGQCYLVPPGTTVFLVNSEEMGFFSEY

VDASRYKILEIIGKGSYGVVCSAIDQETGDKVAIKKIQNIFEHLSDAARILREIKLLRLL

RHPDIVQIKHIMLPPSRRDFRDIYVVFELMDTDLHQVIKANDDLTKEHYQFFLYQMLRAL

KYIHTANVYHRDLKPKNILANANCKLKICDFGLARVAFNDTPTTVFWTDYVATRWYRAPE

LCGSFFTKYSPAIDTWSIGCIFAEILTGKPLFPGKNVVHQLDLMTDLLGTPSTETISRIR

NDKARKYLSSMRRKQPIPFSEKFPNADPSALKLLERLLAFDPKDRPTAEEALAHPYFKRL

ARVEREPSCQQPISKTEFEFERRKFTKEDVKELIFREILEYHPKLLKDYMNGSEKTSFLY

PSAVDNFRRQFANLEIDGGRSGAADRKHFSLPRTTTVHSAPILPTNGPTSQVPQRIPTAR

PGRVVSSAMQTDNPSVSDRHNGRRVARDPAVPPAAAYHLKSDYSDRQHQQEFEKDRVRPD

RQRQEELEKDRMQYRPGHHSMDAKVAPEISPYMRSSPYYIPPFNGIAAVASGYSKVAAVT

RMY*

>BdTR10cMPK21-1 Brdisv1BdTR10C1017009m.p

MDPGKKTSESEFFTEYGEANRYKVSEVIGKGSYGVVAAAVDTQTGERCAIKKINDVFDHV

SDATRILREIKLLRLLRHPDIVEIKHIMLPPSRREFRDIYVVFELMESDLHQVIKANDDL

TPEHHQFFLYQLLRGMKYIHTANVFHRDLKPKNILANADCKLKICDFGLARVSFNDGAPS

AIFWTDYVATRWYRAPELCGSFFSKYTPAIDIWSVGCIFAEMLTGKPLFPGKNVVHQLDL

MTDLLGTPSAESLSKIRNEKARRYLSNMRKKPKVPLTKKFPGIDPMALHLLERLLAFDPK

DRPSADEALTDPYFTGLANSEREPITQPISKLEFEFEKRKLAKDDVRELIYREILEYHPH

MLQEYLRGGGDQMSFMFPSGVDRFKRQFAHLEEGGAKGEKSSPQLRQNASLPRERVIGNK

HGDGDYNMKLNVGEKPELASVSDGISKPLMSARSLLKSETMSASKCIGEIKNKDEDSLSE

CVEGTDDDVSQKIAQLKT*

>BdTR10cMPK21-2 Brdisv1BdTR10C1038225m.p

MTIASLMQQHLQSNPPSPCSPTRALSSLLGRPTGVCPPPFQLAQSVTRRQARDCTHQANQ

ALLVVSLLQRPHQIPLSGQFPPPVTKNSCQKPLPRWNSGELTQSWQRLGSDEAEFFTEYG

EANRYEVGEVVGKGSYGVVAAAVDTHTGERVAIKKINDVFEHVSDATRILREIKLLRLLR

HPDIVEIKHIMLPPSRREFRDIYIIFELMESDLHQVIKANDDLTPEHHQFFFYQLLRGMK

YIHAANVFHRDLKPRNILANADCKLKICDFGLARVSFNDTPSAIFWTDYVATRWYRAPEL

CGSFFSKYTPAIDIWSIGCIFAEMLSGRPLFPGKNVVHQLDLMTDLLGTPSAESLSRIRN

EKARRYLGNMRKKHPVPFSQKFPGVDPMALDLLERLLAFDPKDRPTAAEALADPYFTGLA

NSDREPTTQPISKLEFEFERRKLARDDVRELIYREILEYHPQMLHEYHHGGDQANFVYPS

GVDRFKRQFVHLEEGVTKGEKTSPQLRQHASLPRERIIGIGDELGRPNADYCIKLHVGEE

PGHTSVTDGLSKPLLNARNFLKSESISASQCVVIKEKREKDVSAVTSNIF*

>BdTR11aMPK3 Brdisv1BdTR11A1009650m.p

MDGAPVAEFRPTMTHGGRFLLYNIFGNQFEITAKYQPPIMPIGRGAYGIVCSVMNFETRE

MVAIKKIANAFDNNMDAKRTLREIKLLRHLDHENIVGLRDVIPPAIPQSFNDVYIATELM

DTDLHHIIRSNQELSEEHCQYFLYQLLRGLKYIHSANVIHRDLKPSNLLLNANCDLKICD

FGLARPSSESDMMTEYVVTRWYRAPELLLNSTDYSAAIDVWSVGCIFMELINRAPLFPGR

DHMHQMRLITEVIGTPTDDDLGFIRNEDARRYMRHLPQFPRRPFPAQFPRVQPAALDLIE

RMLAFNPLQRITVEEALEHPYLERLHDIADEPICTDPFSFDFEQHPLTEDQMKQLIFNEA

LELNPNFRY*

>BdTR11aMPK4 Brdisv1BdTR11A1031736m.p

MDTSGGGGGGAGGAAQIQGMATHGGRYVLYNVYGNLFEVASKYAPPIRPIGRGAYGIVCA

AVSSDTGEEVAIKKIGNAFDNHIDAKRTLREIKLLRHMDHENIIAIKDIIRPPRRDDFKD

VYIVTELMDTDLHQIIRSNQPLTDDHCQYFLYQLLRGLKYVHSANVLHRDLKPSNLFLNA

NCDLKIADFGLARTTSETDLMTEYVVTRWYRAPELLLNCSQYTAAIDVWSVGCILGEIIT

RQPLFPGRDYIQQLKLITELIGSPDDSSLGFLRSDNARRYMKQLPQYPRQDFRLRFRNMS

DGAVDLLERMLVFDPSRRITVDEALHHPYLASLHDINEEPTCPAPFSFDFEQPSFTEEHM

KELIWRETLAFNPDPPY*

>BdTR11aMPK6 Brdisv1BdTR11A1006993m.p

MDGGAQPPDAEMADAGAAVPGAGAGAAGTMENIQATLSHGGRFIQYNIFGNVFEVTVKYK

PPILPIGKGAYGIVCSALNSETGEQVAIKKIANAFDNKIDAKRTLREIKLLRHMDHENIV

AIRDIIPPAQRNSFNDVYIAYELMDTDLHQIIRSNQALSEEHCQYFLYQILRGLKYIHSA

NVLHRDLKPSNLLLNANCDLKICDFGLARTTSETDFMTEYVVTRWYRAPELLLNSSEYTA

AIDVWSVGCIFMELMDRKPLFPGRDHVHQLRLLMELIGTPNEADLDFVNENARRYIRQLP

RHARQSFSEKFPHVHPSAIDLVEKMLTFDPRQRITVEGALAHPYLASLHDISDEPVCTMP

FSFDFEQHALSEEQMKDLIHQEALAFNPDYQ*

>BdTR11aMPK11 Brdisv1BdTR11A1030105m.p

MRMEGGGAGPAAAAAGGGAHGLGEAQIKGTLTHGGRYVQYNVYGNLFEVSAKYVPPIRPV

GRGACGIICAAINVQTREEVAIKKIGNAFDNQIDAKRTLREVKLLRHMNHENVISIKDII

RPPRRENFNDVYIVYELMDTDLHHLLRSNQPLTDDHCQYFLYQVLRGLKYVHSANVLHRD

LRPSNLLLNAKCDLKIGDFGLARTTTETDFMMEYVVTRWYRAPELLLNCSEYTGAIDMWS

VGCILGEIATREPLFPGKDYVHQLRLITELLGSPDDTSLGFLRSDNARRYVRSLPQYPKQ

QFRSRFPNMSSGAMDLLERMLVFDPNKRITVDEALCHPYLASLHEINDEPVCPAPFSFDF

EQPSFTEEDIKELIWRESVKFNPEPIH*

>BdTR11aMPK14 Brdisv1BdTR11A1028042m.p

MAMLVDPPNGMGNQGKHYYSMWQTLFEIDTKYVPIKPIGRGAYGIVCSSINRETNEKVAI

KKIHNVFDNRVDALRTLRELKLLRHLRHENVISLKDIMMPVQRRSFKDVYLVYELMDTDL

HQIIKSPQGLSNDHCQYFLFQLLRGLKYLHSAEILHRDLKPGNLLVNANCDLKICDFGLA

RTNSSKGQFMTEYVVTRWYRAPELLLCCDNYGTSIDVWSVGCIFAELLGRKPIFPGTECL

NQLKLIVNVLGTMSESDLEFIDNPKARRYIKTLPYTPGVPLASMYPHAHPLAIDLLQKML

IFDPTKRISVTEALEHPYMSPLYDPSANPPAQVPIDLDLDENISADMIREMMWQEMLHYH

PEAATAISM*

>BdTR11aMPK16 Brdisv1BdTR11A1016556m.p

MDFFTEYGEGNRYKIEEVIGKGSYGVVCSALDTHTGDKVAIKKINDIFEHVSDATRILRE

IKLLRLLRHPDIVEIKHILLPPSRREFKDIYVVFELMESDLHQVIKANDDLTPEHYQFFL

YQLLRGLKYIHTANVFHRDLKPKNILANADCKLKICDFGLARVAISDTPTAIFWTDYIAT

RWYRAPELCGSFFSKYTPAIDIWSIGCIFAELLTGKPLFPGKNVVHQLDIITDLLGTPSA

ETIARIRNEKARRYLSSMRRKKTVPFTQKFPNADPLALNLLERMLAFDPKDRPSAEEALA

DPYFKNIASVDREPSAQPITKLEFEFERRRITKDDIRELIYREILEYHPNMLREFLEGAE

PTGFMYPSAVDHFKKQFTFLEEHYAKGSTAAPPERQHNSLPRPSVIYSDNRPQNAANITD

DLSRCAIRDNTQKAQKDSASVGANRIPHGAAAAAARPGKVVGSVLRYGNCSTSGTEQYEQ

RRVVRSPGIAPNGVTSGSSYPRRNNTCKSETGEAERIDTNQGGPPKPYVANKLPATVDGR

NGHW*

>BdTR11aMPK17 Brdisv1BdTR11A1005247m.p

MVGGGGIVDGFRRLFHRRTPSGSGPSSNQSSAGEESSDIDAVVEDLDLVGLRAIRVPKRK

MPLPVESHKKNILEKEFFTEYGEASQYQIQEVIGKGSYGVVAAAVDTRTGERVAIKKIND

VFEHVSDATRILREVKLLRLLRHPDVVEIKHIMLPPSRREFQDIYVVFELMESDLHQVIR

ANDDLTPEHYQFFLYQLLRALKYIHAANVFHRDLKPKNILANSDCKLKICDFGLARVSFN

DAPTAIFWTDYVATRWYRAPELCGSFFSKYTPAIDIWSIGCIFAELLTGRPLFPGKNVVH

QLDIITDLLGTPSSETLSRIRNEKARRYLSCMRKKHPVPLTQKFPNVDPLALRLLGRLLA

FDPKDRPSAEEALADPYFAVLANVEREPSRNPISKLEFEFERRKVTKDDVRELIYREILE

YHPQMLQEYMQGGEQLSFLYPSGVDRFKRQFAHLEENYSKGERGSPLQRKHASLPRQRVG

ASNDNNEQHASDQERGADSIAPATGNPPGSQDAGQQHVSGGQNGVGSTNISPRSYQKSAS

ISASKCVVVNANKNPEYDDDISEEMEGAVDGLSEKVSRMHP*

>BdTR11aMPK20-1 Brdisv1BdTR11A1017974m.p

MQQDQRKKSSAEAEFFTEYGDASRYKIQEIVGKGSYGVVCSAIDVHTGEKVAIKKIHDIF

EHISDAARILREIKLLRLLRHPDIVEIKHIMLPPSRRDFKDIYVVFELMESDLHQVIKAN

DDLTKEHYQFFLYQLLRALKYIHTANVYHRDLKPKNILANSNCKLKICDFGLARVAFNDT

PTTIFWTDYVATRWYRAPELCGSFFSKYTPAIDVWSIGCIFAEVLTGKPLFPGKNVVHQL

DLMTDLLGTPSMDTISRVRNEKARRYLSSMRKKEPIPFSQKFPNADPLALDLLQRLLAFD

PKDRPTAGEALSHPYFKGLAKVEREPSCQPITKMEFEFERRRATKEDIRELIFREILEYH

PQLLKDYINGTERTTFLYPSAVDQFRKQFAHLEENSESGPVVPMDRKHTSLPRSTIVHSA

PIHAKEQPRIGPSRDRPLTDESYKNPRDSEKYSGNVPRSSHHSQAPQRVPTARPGRVVGP

VLPYENVGTSHPYDPRRVAMNSGYPPQQQIPQTYGYYQTTGKSACSEPSQAERYTLHQQA

YACANSSTAPDVALDMRAPPFHQSAGPKSGSSDRLTAETNLYTRSLNGIVATTSGVAASA

HRKVSVVPYGMSQMY*

>BdTR11aMPK20-2 Brdisv1BdTR11A1045880m.p

MQQPQQQGQRNKSTDVDFFSEYGDANRYRIQEVIGKGSYGVVCSAMDMQTRQKVAIKKIH

NIFDHVSDAARILREIKLLRLLRHPDVVEIKHIMLPPSRKDFKDIYVVFELMESDLHQVI

KANDDLTKEHYQFFLYQLLRALKYIHTASVYHRDLKPKNILANSNCKLKICDFGLARVAF

NDTPTTVFWTDYVATRWYRAPELCGSFFTKYTPAIDIWSIGCIFAEVLTGKPLFPGKNVV

HQLDLMTDLLGTPSMDTISRVRNEKARRYLSSMRKKDPVPFSKKFPNADPLGLKLLEKLL

AFDPKDRPTAEEALTDPYFKSLSKPDREPSCQPIRKVEFDFEHRRMSKDDIRELIFQEIL

EYHPQLLKNYIDGTEKTTFLYPSAVDQFKKQFSHLEESDGSGPVVPTERKHASLPRSTTV

HSTPIPAKEQPLVASSRGRPIANEPCKPWAPGNVPGASQTAHVAQAGRAVGVGSVPPYES

GSGKYPYDATSRPAVSSGYPPQQKIPQTYGYHHHHQTPGAGQSSQAMGGYACGYTKGTTP

PPAAAQDMRASPYHHRSAGTKNDPLNRLAAESDIYTRSLNGIVAAAASAGTGAHRKVGAV

PFGMSGMY*

>BdTR11aMPK20-3 Brdisv1BdTR11A1045462m.p

MQHGDLHKKSAAEMDFFTAYDDANRYKILEVIGKGSYGLVCSANDLQTGEKVAIKKIHNI

FEHISDAARILREIKLLRLLRHPDVVEIKHILLPPSKKDFKDIYVVFELMESDLHQVIKA

NDDLTREHYQFFLYQMLRALKYMHTANVYHRDLKPKNVLANANCKLKICDFGLARVAFSD

APTTVFWTDYVATRWYRAPELCGSFYSKVMIVTIDH*

>BdTR11aMPK20-4 Brdisv1BdTR11A1018174m.p

MQTTEQQRKKGSSEMDFFSEYGDANRYKIQEVIGKGSYGVVCSAIDQHTGDKVAIKKIHN

IFEHLSDAARILREIKLLRLLRHPDIVEIRHIMLPPSRRDFKDIYVVFELMDTDLHQVIK

ANDDLTKEHHQFFLYQMLRALKYIHTANVYHRDLKPKNILANANCKLKICDFGLARVAFN

DTPTTVFWTDYVATRWYRAPELCGSFFTKYSRAIDIWSIGCIFAEILTGKPLFPGKNVVH

QLDLMTDLLGTPSLDTVSRIRNEKARRYLSSMRKKQSVSFSERFPKADPAALKLLQRLLA

FDPKDRPTAEEALADPYFKGLGKVEREPSCQPITKMEFEFERKNVTKADVKELIFREILE

YHPQLLKDYMNGTEKTNFLYPSAVDNFRRQFANLEENGGKGGAVVPPDRKHVSLPRNTTV

HSTPIPPKDQKYSQVPQRIPTGRPGRVVGPVIPFENSSTMDPYSQRRVARNPVLPAAATN

LSAYAYRNSDNSERELQQELEKDRMQYHPMQRFMDAKMVSPDLRSTSYYMPKGVPKADVA

ERSALQSNMMQGIAPFNGIATVGGAFNKVSAVQYGVSRMY*

>BdTR11aMPK20-5 Brdisv1BdTR11A1014255m.p

MFSDIFSREGVAFSRVALRQELLSDQWFTTAGGKGQCYLVPPGTTVFLVNSEEMGFFSEY

VDASRYKILEIIGKGSYGVVCSAIDQETGDKVAIKKIQNIFEHLSDAARILREIKLLRLL

RHPDIVQIKHIMLPPSRRDFRDIYVVFELMDTDLHQVIKANDDLTKEHYQFFLYQMLRAL

KYIHTANVYHRDLKPKNILANANCKLKICDFGLARVAFNDTPTTVFWTDYVATRWYRAPE

LCGSFFTKYSPAIDTWSIGCIFAEILTGKPLFPGKNVVHQLDLMTDLLGTPSTETISRIR

NDKARKYLSSMRRKQPIPFSEKFPNADPSALKLLERLLAFDPKDRPTAEEALAHPYFKRL

ARVEREPSCQQPISKTEFEFERRKFTKEDVKELIFREILEYHPKLLKDYMNGSEKTSFLY

PSAVDNFRRQFANLEIDGGRSGAADRKHFSLPRTTTVHSAPILPTNGPTSQVPQRIPTAR

PGRVVSSAMQTDNPSVSDRHNGRRVARDPAVPPAAAYHLKSDYSDRQHQQEFEKDRVRPD

RQRQEELEKDRMQYRPGHHSMDAKVAPEISPYMRSSPYYIPPFNGIAAVASGYSKVAAVT

RMY*

>BdTR11aMPK21-1 Brdisv1BdTR11A1014151m.p

MGGLIRWLRHHRSRRVSSSSSSHLPSNTTSSSSTSDLRAHSLPQHQGDHHGEVVVEWEDA

AEGPDSDPEEYIVVVLGDDEQGVVAARAPVRTKPPRVMDPGKKTSESEFFTEYGEANRYK

VSEVIGKGSYGVVAAAVDTQTGERCAIKKINDVFDHVSDATRILREIKLLRLLRHPDIVE

IKHIMLPPSRREFRDIYVVFELMESDLHQVIKANDDLTPEHHQFFLYQLLRGMKYIHTAN

VFHRDLKPKNILANADCKLKICDFGLARVSFNDGAPSAIFWTDYVATRWYRAPELCGSFF

SKYTPAIDIWSVGCIFAEMLTGKPLFPGKNVVHQLDLMTDLLGTPSAESLSKIRNEKARR

YLSNMRKKPKVPLTKKFPGIDPMALHLLERLLAFDPKDRPSADEALTDPYFTGLANSERE

PITQPISKLEFEFEKRKLAKDDVRELIYREILEYHPHMLQEYLRGGGDQMSFMFPSGVDR

FKRQFAHLEEGGAKGEKSSPQLRQNASLPRERVIGNKHGDGDYNMKLNVGEKPELASVSD

GISKPLMSARSLLKSETMSASKCIGEIKNKDEDSLSECVEGTDDDVSQKIAQLKT*

>BdTR11aMPK21-2 Brdisv1BdTR11A1018053m.p

MARKGSDEAEFFTEYGEANRYEVGEVVGKGSYGVVAAAVDTHTGERVAIKKINDVFEHVS

DATRILREIKLLRLLRHPDIVEIKHIILPPSRREFRDIYIIFELMESDLHQVIKANDDLT

PEHHQFFFYQLLRGMKYIHAANVFHRDLKPRNILANADCKLKICDFGLARVSFNDTPSAI

FWTDYVATRWYRAPELCGSFFSKYTPAIDIWSIGCIFAEMLSGRPLFPGKNVVHQLDLMT

DLLGTPSAESLSRIRNEKARRYLGNMRKKHPVPFSQKFPGVDPMALDLLERLLAFDPKDR

PTAAEALADPYFTGLANSDREPTTQPISKLEFEFERRKLARDDVRELIYREILEYHPQML

HEYHHGGDQANFVYPSGVDRFKRQFVHLEEGVTKGEKTSPQLRQHASLPRERIIGIGDEL

GRPNADYCIKLHVGEEPGHTSVTDGLSKPLLNARNFLKSESISASQCVVIKEKREKDEES

MSEYMHEASDATPK*

>BdTR11iMPK3 Brdisv1BdTR11I1010553m.p

MDGAPVAEFRPTMTHGGRFLLYNIFGNQFEITAKYQPPIMPIGRGAYGIVCSVMNFETRE

MVAIKKIANAFDNNMDAKRTLREIKLLRHLDHENIVGLRDVIPPAIPQSFNDVYIATELM

DTDLHHIIRSNQELSEEHCQYFLYQLLRGLKYIHSANVIHRDLKPSNLLLNANCDLKICD

FGLARPSSESDMMTEYVVTRWYRAPELLLNSTDYSAAIDVWSVGCIFMELINRAPLFPGR

DHMHQMRLITEVIGTPTDDDLGFIRNEDARRYMRPLPQFPRRPFPAQFPRVQPAALDLIE

RMLAFNPLQRITVEEALEHPYLERLHDIADEPICTDPFSFDFEQHPLTEDQMKQLIFNEA

LELNPNFRY*

>BdTR11iMPK4 Brdisv1BdTR11I1034234m.p

MDTSGGGGGGAGGAAQIQGMATHGGRYVLYNVYGNLFEVASKYAPPIRPIGRGAYGIVCA

AVSSDTGEEVAIKKIGNAFDNHIDAKRTLREIKLLRHMDHENIIAIKDIIRPPRRDDFKD

VYIVTELMDTDLHQIIRSNQPLTDDHCQYFLYQLLRGLKYVHSANVLHRDLKPSNLFLNA

NCDLKIADFGLARTTSETDLMTEYVVTRWYRAPELLLNCSQYTAAIDVWSVGCILGEIIT

RQPLFPGRDYIQQLKLITELIGSPDDSSLGFLRSDNARRYMKQLPQYPRQDFRLRFRNMS

DGAVDLLERMLVFDPSRRITVDEALHHPYLASLHDINEEPTCPAPFSFDFEQPSFTEEHM

KELIWRETLAFNPDPPY*

>BdTR11iMPK6 Brdisv1BdTR11I1007652m.p

MDGGAQPPDAEMADAGAAVPGAGAGAAGTMENIQATLSHGGRFIQYNIFGNVFEVTVKYK

PPILPIGKGAYGIVCSALNSETGEQVAIKKIANAFDNKIDAKRTLREIKLLRHMDHENIV

AIRDIIPPAQRNSFNDVYIAYELMDTDLHQIIRSNQALSEEHCQYFLYQILRGLKYIHSA

NVLHRDLKPSNLLLNANCDLKICDFGLARTTSETDFMTEYVVTRWYRAPELLLNSSEYTA

AIDVWSVGCIFMELMDRKPLFPGRDHVHQLRLLMELIGTPNEADLDFVNENARRYIRQLP

RHARQSFSEKFPHVHPSAIDLVEKMLTFDPRQRITVEGALAHPYLASLHDISDEPVCTMP

FSFDFEQHALSEEQMKDLIHQEALAFNPDYQ*

>BdTR11iMPK7-1 Brdisv1BdTR11I1045888m.p

LLRGLKYLHSAGILHRDLKPGNLLVNANCDLKICDFGLARTNNTKGQFMTEYVVTCLYRA

PELLLCCDNYGTSIDVWSVGCIFAELLGRKPIFPRTQCLDQLKLIVNVLGTMSENDLEFI

DNPKARKYIKSLPYTPGTPLTSMYPQAHPLAIDLLQKMLVFDPSKRISVTEALEHPYMSP

LYDPSANPPAQVPIDLDIDENLGVEMIREMLWQEMLQYHPEAARMVNM*

>BdTR11iMPK11 Brdisv1BdTR11I1032437m.p

MRMEGGGAGPAAAAAGGGAHGLGEAQIKGTLTHGGRYVQYNVYGNLFEVSAKYVPPIRPV

GRGACGIICAAINVQTREEVAIKKIGNAFDNQIDAKRTLREVKLLRHMNHENVISIKDII

RPPRRENFNDVYIVYELMDTDLHHLLRSNQPLTDDHCQYFLYQVLRGLKYVHSANVLHRD

LRPSNLLLNAKCDLKIGDFGLARTTTETDFMMEYVVTRWYRAPELLLNCSEYTGAIDMWS

VGCILGEIATREPLFPGKDYVHQLRLITELLGSPDDTSLGFLRSDNARRYVRSLPQYPKQ

QFRSRFPNMSSGAMDLLERMLVFDPNKRITVDEALCHPYLASLHEINDEPVCPAPFSFDF

EQPSFTEEDIKELIWRESVKFNPEPIH*

>BdTR11iMPK14 Brdisv1BdTR11I1030242m.p

MAMLVDPPNGMGNQGKHYYSMWQTLFEIDTKYVPIKPIGRGAYGIVCSSINRETNEKVAI

KKIHNVFDNRVDALRTLRELKLLRHLRHENVISLKDIMMPVQRRSFKDVYLVYELMDTDL

HQIIKSPQGLSNDHCQYFLFQLLRGLKYLHSAEILHRDLKPGNLLVNANCDLKICDFGLA

RTNSSKGQFMTEYVVTRWYRAPELLLCCDNYGTSIDVWSVGCIFAELLGRKPIFPGTECL

NQLKLIVNVLGTMSESDLEFIDNPKARRYIKTLPYTPGVPLASMYPHAHPLAIDLLQKML

IFDPTKRISVTEALEHPYMSPLYDPSANPPAQVPIDLDLDENISADMIREMMWQEMLHYH

PEAATAISM*

>BdTR11iMPK16 Brdisv1BdTR11I1018043m.p

MDFFTEYGEGNRYKIEEVIGKGSYGVVCSALDTHTGDKVAIKKINDIFEHVSDATRILRE

IKLLRLLRHPDIVEIKHILLPPSRREFKDIYVVFELMESDLHQVIKANDDLTPEHYQFFL

YQLLRGLKYIHTANVFHRDLKPKNILANADCKLKICDFGLARVAISDTPTAIFWTDYIAT

RWYRAPELCGSFFSKYTPAIDIWSIGCIFAELLTGKPLFPGKNVVHQLDIITDLLGTPSA

ETIARIRNEKARRYLSSMRRKKTVPFTQKFPNADPLALNLLERMLAFDPKDRPSAEEALA

DPYFKNIASVDREPSAQPITKLEFEFERRRITKDDIRELIYREILEYHPNMLREFLEGAE

PTGFMYPSAVDHFKKQFTFLEEHYAKGSTAAPPERQHNSLPRPSVIYSDNRPQNAANITD

DLSRCAIRDNTQKAQKDSASVGANRIPHGAAAAAARPGKVVGSVLRYGNCSTSGTEQYEQ

RRVVRSPGIAPNGVTSGSSYPRRNNTCKSETGEAERIDTNQGGPPKPYVANKLPATVDGR

NGHW*

>BdTR11iMPK17 Brdisv1BdTR11I1005619m.p

MVGGGGIVDGFRRLFHRRTPSGSGPSSNQSSAGEESSDIDAVVEDLDLVGLRAIRVPKRK

MPLPVESHKKNILEKEFFTEYGEASQYQIQEVIGKGSYGVVAAAVDTRTGERVAIKKIND

VFEHVSDATRILREVKLLRLLRHPDVVEIKHIMLPPSRREFQDIYVVFELMESDLHQVIR

ANDDLTPEHYQFFLYQLLRALKYIHAANVFHRDLKPKNILANSDCKLKICDFGLARVSFN

DAPTAIFWTDYVATRWYRAPELCGSFFSKYTPAIDIWSIGCIFAELLTGRPLFPGKNVVH

QLDIITDLLGTPSSETLSRIRNEKARRYLSCMRKKHPVPLTQKFPNVDPLALRLLGRLLA

FDPKDRPSAEEALADPYFAVLANVEREPSRNPISKLEFEFERRKVTKDDVRELIYREILE

YHPQMLQEYMQGGEQLSFLYPSGVDRFKRQFAHLEENYSKGERGSPLQRKHASLPRQRVG

ASNDNNEQHASDQERGADSIAPATGNPPGSQDAGQQHVSGGQNGVGSTNISPRSYQKSAS

ISASKCVVVNANKNPEYDDDISEEMEGAVDGLSEKVSRMHP*

>BdTR11iMPK20-1 Brdisv1BdTR11I1019460m.p

MQQDQRKKSSAEAEFFTEYGDASRYKIQEIVGKGSYGVVCSAIDVHTGEKVAIKKIHDIF

EHISDAARILREIKLLRLLRHPDIVEIKHIMLPPSRRDFKDIYVVFELMESDLHQVIKAN

DDLTKEHYQFFLYQLLRALKYIHTANVYHRDLKPKNILANSNCKLKICDFGLARVAFNDT

PTTIFWTDYVATRWYRAPELCGSFFSKYTPAIDVWSIGCIFAEVLTGKPLFPGKNVVHQL

DLMTDLLGTPSMDTISRVRNEKARRYLSSMRKKEPIPFSQKFPNADPLALDLLQRLLAFD

PKDRPTAGEALSHPYFKGLAKVEREPSCQPITKMEFEFERRRATKEDIRELIFREILEYH

PQLLKDYINGTERTTFLYPSAVDQFRKQFAHLEENSESGPVVPMDRKHTSLPRSTIVHSA

PIHAKEQPRIGPSRDRPLTDESYKNPRDSEKYSGNVPRSSHHSQAPQRVPTARPGRVVGP

VLPYENVGTSHPYDPRRVAMNSGYPPQQQIPQTYGYYQTTGKSACSEPSQAERYTLHQQA

YACANSSTAPDVALDMRAPPFHQSAGPKSGSSDRLTAETNLYTRSLNGIVATTSGVAASA

HRKVSVVPYGMSQMY*

>BdTR11iMPK20-2 Brdisv1BdTR11I1015383m.p

MQQPQQQGQRNKLPDGWFCCNVHILNLCRSAMIFLTPPIIMPSTDVDFFSEYGDANRYRI

QEVIGKGSYGVVCSAMDMQTRQKVAIKKIHNIFDHVSDAARILREIKLLRLLRHPDVVEI

KHIMLPPSRKDFKDIYVVFELMESDLHQVIKANDDLTKEHYQFFLYQLLRALKYIHTASV

YHRDLKPKNILANSNCKLKICDFGLARVAFNDTPTTVFWTDYVATRWYRAPELCGSFFTK

YTPAIDIWSIGCIFAEVLTGKPLFPGKNVVHQLDLMTDLLGTPSMDTISRVRNEKARRYL

SSMRKKDPVPFSKKFPNADPLGLKLLEKLLAFDPKDRPTAEEALTDPYFKSLSKPDREPS

CQPIRKVEFDFEHRRMSKDDIRELIFQEILEYHPQLLKNYIDGTEKTTFLYPSAVDQFKK

QFSHLEESDGSGPVVPTERKHASLPRSTTVHSTPIPAKEQPLVASSRGRPIANEPCKPWA

PGNVPGASQTAHVAQAGRAVGVGSVPPYESGSGKYPYDATSRPAVSSGYPPQQKIPQTYG

YHHHHQTPGAGQSSQAMGGYACGYTKGTTPPPAAAQDMRASPYHHRSAGTKNDPLNRLAA

ESDIYTRSLNGIVAAAASAGTGAHRKVGAVPFGMSGMY*

>BdTR11iMPK20-3 Brdisv1BdTR11I1044565m.p

MQHGDLHKKSAAEMDFFTAYDDANRYKILEVIGKGSYGLVCSANDLQTGEKVAIKKIHNI

FEHISDAARILREIKLLRLLRHPDVVEIKHILLPPSKKDFKDIYVVFELMESDLHQVIKA

NDDLTREHYQFFLYQMLRALKYMHTANVYHRDLKPKNVLANANCKLKICDFGLARVAFSD

APTTVFWTDYVATRWYRAPELCGSFYSK

>BdTR11iMPK20-4 Brdisv1BdTR11I1019682m.p

MQTTEQQRKKGSSEMDFFSEYGDANRYKIQEVIGKGSYGVVCSAIDQHTGDKVAIKKIHN

IFEHLSDAARILREIKLLRLLRHPDIVEIRHIMLPPSRRDFKDIYVVFELMDTDLHQVIK

ANDDLTKEHHQFFLYQMLRALKYIHTANVYHRDLKPKNILANANCKLKICDFGLARVAFN

DTPTTVFWTDYVATRWYRAPELCGSFFTKYSRAIDIWSIGCIFAEILTGKPLFPGKNVVH

QLDLMTDLLGTPSLDTVSRIRNEKARRYLSSMRKKQSVSFSERFPKADPAALKLLQRLLA

FDPKDRPTAEEALADPYFKGLGKVEREPSCQPITKMEFEFERKNVTKADVKELIFREILE

YHPQLLKDYMNGTEKTNFLYPSAVDNFRRQFANLEENGGKGGAVVPPDRKHVSLPRNTTV

HSTPIPPKDQKYSQVPQRIPTGRPGRVVGPVIPFENSSTMDPYSQRRVARNPVLPAAATN

LSAYAYRNSDNSERELQQELEKDRMQYHPMQRFMDAKMVSPDLRSTSYYMPKGVPKADVA

ERSALQSNMMQGIAPFNGIATVGGAFNKVSAVQYGVSRMY*

>BdTR11iMPK20-5 Brdisv1BdTR11I1015521m.p

MFSDIFSREGVAFSRVALRQELLSDQWFTTAGGKGQCYLVPPGTTVFLVNSEEMGFFSEY

VDASRYKILEIIGKGSYGVVCSAIDQETGDKVAIKKIQNIFEHLSDAARILREIKLLRLL

RHPDIVQIKHIMLPPSRRDFRDIYVVFELMDTDLHQVIKANDDLTKEHYQFFLYQMLRAL

KYIHTANVYHRDLKPKNILANANCKLKICDFGLARVAFNDTPTTVFWTDYVATRWYRAPE

LCGSFFTKYSPAIDTWSIGCIFAEILTGKPLFPGKNVVHQLDLMTDLLGTPSTETISRIR

NDKARKYLSSMRRKQPIPFSEKFPNADPSALKLLERLLAFDPKDRPTAEEALAHPYFKRL

ARVEREPSCQQPISKTEFEFERRKFTKEDVKELIFREILEYHPKLLKDYMNGSEKTSFLY

PSAVDNFRRQFANLEIDGGRSGAADRKHFSLPRTTTVHSAPILPTNGPTSQVPQRIPTAR

PGRVVSSAMQTDNPSVSDRHNGRRVARDPAVPPAAAYHLKSDYSDRQHQQEFEKDRVRPD

RQRQEELEKDRMQYRPGHHSMDAKVAPEISPYMRSSPYYIPPFNGIAAVASGYSKVAAVT

RMY*

>BdTR11iMPK21-1 Brdisv1BdTR11I1015413m.p

MGGLIRWLRHHRSRRVSSSSSSHLPSNTTSSSSTSDLRAHSLPQHQGDHHGEVVVEWEDA

AEGPDSDPEEYIVVVLGDDEQGVVAARAPVRTKPPRVMDPGKKTSESEFFTEYGEANRYK

VSEVIGKGSYGVVAAAVDTQTGERCAIKKINDVFDHVSDATRILREIKLLRLLRHPDIVE

IKHIMLPPSRREFRDIYVVFELMESDLHQVIKANDDLTPEHHQFFLYQLLRGMKYIHTAN

VFHRDLKPKNILANADCKLKICDFGLARVSFNDGAPSAIFWTDYVATRWYRAPELCGSFF

SKYTPAIDIWSVGCIFAEMLTGKPLFPGKNVVHQLDLMTDLLGTPSAESLSKIRNEKARR

YLSNMRKKPKVPLTKKFPGIDPMALHLLERLLAFDPKDRPSADEALTDPYFTGLANSERE

PITQPISKLEFEFEKRKLAKDDVRELIYREILEYHPHMLQEYLRGGGDQMSFMFPSGVDR

FKRQFAHLEEGGAKGEKSSPQLRQNASLPRERVIGNKHGDGDYNMKLNVGEKPELASVSD

GISKPLMSARSLLKSETMSASKCIGEIKNKDEDSLSECVEGTDDDVSQKIAQLKT*

>BdTR11iMPK21-2 Brdisv1BdTR11I1019550m.p

MARKGSDEAEFFTEYGEANRYEVGEVVGKGSYGVVAAAVDTHTGERVAIKKINDVFEHVS

DATRILREIKLLRLLRHPDIVEIKHIILPPSRREFRDIYIIFELMESDLHQVIKANDDLT

PEHHQFFFYQLLRGMKYIHAANVFHRDLKPRNILANADCKLKICDFGLARVSFNDTPSAI

FWTDYVATRWYRAPELCGSFFSKYTPAIDIWSIGCIFAEMLSGRPLFPGKNVVHQLDLMT

DLLGTPSAESLSRIRNEKARRYLGNMRKKHPVPFSQKFPGVDPMALDLLERLLAFDPKDR

PTAAEALADPYFTGLANSDREPTTQPISKLEFEFERRKLARDDVRELIYREILEYHPQML

HEYHHGGDQANFVYPSGVDRFKRQFVHLEEGVTKGEKTSPQLRQHASLPRERIIGIGDEL

GRPNADYCIKLHVGEEPGHTSVTDGLSKPLLNARNFLKSESISASQCVVIKEKREKDEES

MSEYMHEASDATPK*

>BdTR11gMPK3 Brdisv1BdTR11G1010947m.p

MDGAPVAEFRPTMTHGGRFLLYNIFGNQFEITAKYQPPIMPIGRGAYGIVCSVMNFETRE

MVAIKKIANAFDNNMDAKRTLREIKLLRHLDHENIVGLRDVIPPAIPQSFNDVYIATELM

DTDLHHIIRSNQELSEEHCQYFLYQLLRGLKYIHSANVIHRDLKPSNLLLNANCDLKICD

FGLARPSSESDMMTEYVVTRWYRAPELLLNSTDYSAAIDVWSVGCIFMELINRAPLFPGR

DHMHQMRLITEVIGTPTDDDLGFIRNEDARRYMRHLPQFPRRPFPAQFPRVQPAALDLIE

RMLAFNPLQRITVEEALEHPYLERLHDIADEPICTDPFSFDFEQHPLTEDQMKQLIFNEA

LELNPNFRY*

>BdTR11gMPK4 Brdisv1BdTR11G1035183m.p

MDTSGGGGGGAGGAAQIQGMATHGGRYVLYNVYGNLFEVASKYAPPIRPIGRGAYGIVCA

AVSSDTGEEVAIKKIGNAFDNHIDAKRTLREIKLLRHMDHENIIAIKDIIRPPRRDDFKD

VYIVTELMDTDLHQIIRSNQPLTDDHCQYFLYQLLRGLKYVHSANVLHRDLKPSNLFLNA

NCDLKIADFGLARTTSETDLMTEYVVTRWYRAPELLLNCSQYTAAIDVWSVGCILGEIIT

RQPLFPGRDYIQQLKLITELIGSPDDSSLGFLRSDNARRYMKQLPQYPRQDFRLRFRNMS

DGAVDLLERMLVFDPSRRITVDEALHHPYLASLHDINEEPTCPAPFSFDFEQPSFTEEHM

KELIWRETLAFNPDPPY*

>BdTR11gMPK6 Brdisv1BdTR11G1007922m.p

MDGGAQPPDAEMADAGAAVPGAGAGAAGTMENIQATLSHGGRFIQYNIFGNVFEVTVKYK

PPILPIGKGAYGIVCSALNSETGEQVAIKKIANAFDNKIDAKRTLREIKLLRHMDHENIV

AIRDIIPPAQRNSFNDVYIAYELMDTDLHQIIRSNQALSEEHCQYFLYQILRGLKYIHSA

NVLHRDLKPSNLLLNANCDLKICDFGLARTTSETDFMTEYVVTRWYRAPELLLNSSEYTA

AIDVWSVGCIFMELMDRKPLFPGRDHVHQLRLLMELIGTPNEADLDFVNENARRYIRQLP

RHARQSFSEKFPHVHPSAIDLVEKMLTFDPRQRITVEGALAHPYLASLHDISDEPVCTMP

FSFDFEQHALSEEQMKDLIHQEALAFNPDYQ*

>BdTR11gMPK7-1 Brdisv1BdTR11G1046470m.p

LLRGLKYLHSAGILHRDLKPGNLLVNANCDLKICDFGLARTNNTKGQFMTEYVVTCLYRA

PELLLCCDNYGTSIDVWSVGCIFAELLGRKPIFPRTQCLDQLKLIVNVLGTMSENDLEFI

DNPKARKYIKSLPYTPGTPLTSMYPQAHPLAIDLLQKMLVFDPSKRISVTEALEHPYMSP

LYDPSANPPAQVPIDLDIDENLGVEMIREMLWQEMLQYHPEAARMVNM*

>BdTR11gMPK11 Brdisv1BdTR11G1033165m.p

MRMEGGGAGPAAAAAGGGAHGLGEAQIKGTLTHGGRYVQYNVYGNLFEVSAKYVPPIRPV

GRGACGIICAAINVQTREEVAIKKIGNAFDNQIDAKRTLREVKLLRHMNHENVISIKDII

RPPRRENFNDVYIVYELMDTDLHHLLRSNQPLTDDHCQYFLYQVLRGLKYVHSANVLHRD

LRPSNLLLNAKCDLKIGDFGLARTTTETDFMMEYVVTRWYRAPELLLNCSEYTGAIDMWS

VGCILGEIATREPLFPGKDYVHQLRLITELLGSPDDTSLGFLRSDNARRYVRSLPQYPKQ

QFRSRFPNMSSGAMDLLERMLVFDPNKRITVDEALCHPYLASLHEINDEPVCPAPFSFDF

EQPSFTEEDIKELIWRESVKFNPEPIH*

>BdTR11gMPK14 Brdisv1BdTR11G1031078m.p

MAMLVDPPNGMGNQGKHYYSMWQTLFEIDTKYVPIKPIGRGAYGIVCSSINRETNEKVAI

KKIHNVFDNRVDALRTLRELKLLRHLRHENVISLKDIMMPVQRRSFKDVYLVYELMDTDL

HQIIKSPQGLSNDHCQYFLFQLLRGLKYLHSAEILHRDLKPGNLLVNANCDLKICDFGLA

RTNSSKGQFMTEYVVTRWYRAPELLLCCDNYGTSIDVWSVGCIFAELLGRKPIFPGTECL

NQLKLIVNVLGTMSESDLEFIDNPKARRYIKTLPYTPGVPLASMYPHAHPLAIDLLQKML

IFDPTKRISVTEALEHPYMSPLYDPSANPPAQVPIDLDLDENISADMIREMMWQEMLHYH

PEAATAISM*

>BdTR11gMPK16 Brdisv1BdTR11G1018684m.p

MDFFTEYGEGNRYKIEEVIGKGSYGVVCSALDTHTGDKVAIKKINDIFEHVSDATRILRE

IKLLRLLRHPDIVEIKHILLPPSRREFKDIYVVFELMESDLHQVIKANDDLTPEHYQFFL

YQLLRGLKYIHTANVFHRDLKPKNILANADCKLKICDFGLARVAISDTPTAIFWTDYIAT

RWYRAPELCGSFFSKYTPAIDIWSIGCIFAELLTGKPLFPGKNVVHQLDIITDLLGTPSA

ETIARIRNEKARRYLSSMRRKKTVPFTQKFPNADPLALNLLERMLAFDPKDRPSAEEALA

DPYFKNIASVDREPSAQPITKLEFEFERRRITKDDIRELIYREILEYHPNMLREFLEGAE

PTGFMYPSAVDHFKKQFTFLEEHYAKGSTAAPPERQHNSLPRPSVIYSDNRPQNAANITD

DLSRCAIRDNTQKAQKDSASVGANRIPHGAAAAAARPGKVVGSVLRYGNCSTSGTEQYEQ

RRVVRSPGIAPNGVTSGSSYPRRNNTCKSETGEAERIDTNQGGPPKPYVANKLPATVDGR

NGHW*

>BdTR11gMPK17 Brdisv1BdTR11G1005789m.p

MVGGGGIVDGFRRLFHRRTPSGSGPSSNQSSAGEESSDIDAVVEDLDLVGLRAIRVPKRK

MPLPVESHKKNILEKEFFTEYGEASQYQIQEVIGKGSYGVVAAAVDTRTGERVAIKKIND

VFEHVSDATRILREVKLLRLLRHPDVVEIKHIMLPPSRREFQDIYVVFELMESDLHQVIR

ANDDLTPEHYQFFLYQLLRALKYIHAANVFHRDLKPKNILANSDCKLKICDFGLARVSFN

DAPTAIFWTDYVATRWYRAPELCGSFFSKYTPAIDIWSIGCIFAELLTGRPLFPGKNVVH

QLDIITDLLGTPSSETLSRIRNEKARRYLSCMRKKHPVPLTQKFPNVDPLALRLLGRLLA

FDPKDRPSAEEALADPYFAVLANVEREPSRNPISKLEFEFERRKVTKDDVRELIYREILE

YHPQMLQEYMQGGEQLSFLYPSGVDRFKRQFAHLEENYSKGERGSPLQRKHASLPRQRVG

ASNDNNEQHASDQERGADSIAPATGNPPGSQDAGQQHVSGGQNGVGSTNISPRSYQKSAS

ISASKCVVVNANKNPEYDDDISEEMEGAVDGLSEKVSRMHP*

>BdTR11gMPK20-1 Brdisv1BdTR11G1020132m.p

MQQDQRKKSSAEAEFFTEYGDASRYKIQEIVGKGSYGVVCSAIDVHTGEKVAIKKIHDIF

EHISDAARILREIKLLRLLRHPDIVEIKHIMLPPSRRDFKDIYVVFELMESDLHQVIKAN

DDLTKEHYQFFLYQLLRALKYIHTANVYHRDLKPKNILANSNCKLKICDFGLARVAFNDT

PTTIFWTDYVATRWYRAPELCGSFFSKYTPAIDVWSIGCIFAEVLTGKPLFPGKNVVHQL

DLMTDLLGTPSMDTISRVRNEKARRYLSSMRKKEPIPFSQKFPNADPLALDLLQRLLAFD

PKDRPTAGEALSHPYFKGLAKVEREPSCQPITKMEFEFERRRATKEDIRELIFREILEYH

PQLLKDYINGTERTTFLYPSAVDQFRKQFAHLEENSESGPVVPMDRKHTSLPRSTIVHSA

PIHAKEQPRIGPSRDRPLTDESYKNPRDSEKYSGNVPRSSHHSQAPQRVPTARPGRVVGP

VLPYENVGTSHPYDPRRVAMNSGYPPQQQIPQTYGYYQTTGKSACSEPSQAERYTLHQQA

YACANSSTAPDVALDMRAPPFHQSAGPKSGSSDRLTAETNLYTRSLNGIVATTSGVAASA

HRKVSVVPYGMSQMY*

>BdTR11gMPK20-2 Brdisv1BdTR11G1015901m.p

MQQPQQQGQRNKLPDGWFCCNVHILNLCRSAMIFLTPPIIMPSTDVDFFSEYGDANRYRI

QEVIGKGSYGVVCSAMDMQTRQKVAIKKIHNIFDHVSDAARILREIKLLRLLRHPDVVEI

KHIMLPPSRKDFKDIYVVFELMESDLHQVIKANDDLTKEHYQFFLYQLLRALKYIHTASV

YHRDLKPKNILANSNCKLKICDFGLARVAFNDTPTTVFWTDYVATRWYRAPELCGSFFTK

YTPAIDIWSIGCIFAEVLTGKPLFPGKNVVHQLDLMTDLLGTPSMDTISRVRNEKARRYL

SSMRKKDPVPFSKKFPNADPLGLKLLEKLLAFDPKDRPTAEEALTDPYFKSLSKPDREPS

CQPIRKVEFDFEHRRMSKDDIRELIFQEILEYHPQLLKNYIDGTEKTTFLYPSAVDQFKK

QFSHLEESDGSGPVVPTERKHASLPRSTTVHSTPIPAKEQPLVASSRGRPIANEPCKPWA

PGNVPGASQTAHVAQAGRAVGVGSVPPYESGSGKYPYDATSRPAVSSGYPPQQKIPQTYG

YHHHHQTPGAGQSSQAMGGYACGYTKGTTPPPAAAQDMRASPYHHRSAGTKNDPLNRLAA

ESDIYTRSLNGIVAAAASAGTGAHRKVGAVPFGMSGMY*

>BdTR11gMPK20-3 Brdisv1BdTR11G1045432m.p

MQHGDLHKKSAAEMDFFTAYDDANRYKILEVIGKGSYGLVCSANDLQTGEKVAIKKIHNI

FEHISDAARILREIKLLRLLRHPDVVEIKHILLPPSKKDFKDIYVVFELMESDLHQVIKA

NDDLTREHYQFFLYQMLRALKYMHTANVYHRDLKPKNVLANANCKLKICDFGLARVAFSD

APTTVFWTDYVATRWYRAPELCGSFYSK

>BdTR11gMPK20-4 Brdisv1BdTR11G1024979m.p

MQTTEQQRKKGSSEMDFFSEYGDANRYKIQEVIGKGSYGVVCSAIDQHTGDKVAIKKIHN

IFEHLSDAARILREIKLLRLLRHPDIVEIRHIMLPPSRRDFKDIYVVFELMDTDLHQVIK

ANDDLTKEHHQFFLYQMLRALKYIHTANVYHRDLKPKNILANANCKLKICDFGLARVAFN

DTPTTVFWTDYVATRWYRAPELCGSFFTKYSRAIDIWSIGCIFAEILTGKPLFPGKNVVH

QLDLMTDLLGTPSLDTVSRIRNEKARRYLSSMRKKQSVSFSERFPKADPAALKLLQRLLA

FDPKDRPTAEEALADPYFKGLGKVEREPSCQPITKMEFEFERKNVTKADVKELIFREILE

YHPQLLKDYMNGTEKTNFLYPSAVDNFRRQFANLEENGGKGGAVVPPDRKHVSLPRNTTV

HSTPIPPKDQKYSQVPQRIPTGRPGRVVGPVIPFENSSTMDPYSQRRVARNPVLPAAATN

LSAYAYRNSDNSERELQQELEKDRMQYHPMQRFMDAKMVSPDLRSTSYYMPKGVPKADVA

ERSALQSNMMQGIAPFNGIATVGGAFNKVSAVQYGVSRMY*

>BdTR11gMPK20-5 Brdisv1BdTR11G1016043m.p

MFSDIFSREGVAFSRVALRQELLSDQWFTTAGGKGQCYLVPPGTTVFLVNSEEMGFFSEY

VDASRYKILEIIGKGSYGVVCSAIDQETGDKVAIKKIQNIFEHLSDAARILREIKLLRLL

RHPDIVQIKHIMLPPSRRDFRDIYVVFELMDTDLHQVIKANDDLTKEHYQFFLYQMLRAL

KYIHTANVYHRDLKPKNILANANCKLKICDFGLARVAFNDTPTTVFWTDYVATRWYRAPE

LCGSFFTKYSPAIDTWSIGCIFAEILTGKPLFPGKNVVHQLDLMTDLLGTPSTETISRIR

NDKARKYLSSMRRKQPIPFSEKFPNADPSALKLLERLLAFDPKDRPTAEEALAHPYFKRL

ARVEREPSCQQPISKTEFEFERRKFTKEDVKELIFREILEYHPKLLKDYMNGSEKTSFLY

PSAVDNFRRQFANLEIDGGRSGAADRKHFSLPRTTTVHSAPILPTNGPTSQVPQRIPTAR

PGRVVSSAMQTDNPSVSDRHNGRRVARDPAVPPAAAYHLKSDYSDRQHQQEFEKDRVRPD

RQRQEELEKDRMQYRPGHHSMDAKVAPEISPYMRSSPYYIPPFNGIAAVASGYSKVAAVT

RMY*

>BdTR11gMPK21-1 Brdisv1BdTR11G1015942m.p

MGGLIRWLRHPRSRRVSSSSSSHLPSNTPSSSSTSDLRAHSLPQHQGDHHGEVVVEWEDA

AEGPDSDPEEYIVVVLGDDEQGVVAARAPVRTKPPRVMDPGKKTSESEFFTEYGEANRYK

VSEVIGKGSYGVVAAAVDTQTGERCAIKKINDVFDHVSDATRILREIKLLRLLRHPDIVE

IKHIMLPPSRREFRDIYVVFELMESDLHQVIKANDDLTPEHHQFFLYQLLRGMKYIHTAN

VFHRDLKPKNILANADCKLKICDFGLARVSFNDGAPSAIFWTDYVATRWYRAPELCGSFF

SKYTPAIDIWSVGCIFAEMLTGKPLFPGKNVVHQLDLMTDLLGTPSAESLSKIRNEKARR

YLSNMRKKPKVPLTKKFPGIDPMALHLLERLLAFDPKDRPSADEALTDPYFTGLANSERE

PITQPISKLEFEFEKRKLAKDDVRELIYREILEYHPHMLQEYLRGGGDQMSFMFPSGVDR

FKRQFAHLEEGGAKGEKSSPQLRQNASLPRERVIGNKHGDGDYNMKLNVGEKPELASVSD

GISKPLMSARSLLKSETMSASKCIGEIKNKDEDSLSECVEGTDDDVSQKIAQLKT*

>BdTR11gMPK21-2 Brdisv1BdTR11G1020223m.p

MARKGSDEAEFFTEYGEANRYEVGEVVGKGSYGVVAAAVDTHTGERVAIKKINDVFEHVS

DATRILREIKLLRLLRHPDIVEIKHIILPPSRREFRDIYIIFELMESDLHQVIKANDDLT

PEHHQFFFYQLLRGMKYIHAANVFHRDLKPRNILANADCKLKICDFGLARVSFNDTPSAI

FWTDYVATRWYRAPELCGSFFSKYTPAIDIWSIGCIFAEMLSGRPLFPGKNVVHQLDLMT

DLLGTPSAESLSRIRNEKARRYLGNMRKKHPVPFSQKFPGVDPMALDLLERLLAFDPKDR

PTAAEALADPYFTGLANSDREPTTQPISKLEFEFERRKLARDDVRELIYREILEYHPQML

HEYHHGGDQANFVYPSGVDRFKRQFVHLEEGVTKGEKTSPQLRQHASLPRERIIGIGDEL

GRPNADYCIKLHVGEEPGHTSVTDGLRKPLLNARNFLKSESISASQCVVIKEKREKDEES

MSEYMHEASDATPK*

>BdTR13cMPK3 Brdisv1BdTR13C1009620m.p

MDGAPVAEFRPTMTHGGRVLLYNIFGNQFEITAKYQPPIMPIGRGAYGIVCSVMNFETRE

MVAIKKIANAFDNNMDAKRTLREIKLLRHLDHENIVGLRDVIPPAIPQSFNDVYIATELM

DTDLHHIIRSNQELSEEHCQYFLYQLLRGLKYIHSANVIHRDLKPSNLLLNANCDLKICD

FGLARPSSESDMMTEYVVTRWYRAPELLLNSTDYSAAIDVWSVGCIFMELINRAPLFPGR

DHMHQMRLITEVIGTPTDDDLGFIRNEDARRYMRPLPQFPRRPFPAQFPRVQPAALDLIE

RMLAFNPLQRITVEEALEHPYLERLHDIADEPICTDPFSFDFEQHPLTEDQMKQLIFNEA

LELNPNFRY*

>BdTR13cMPK4 Brdisv1BdTR13C1010368m.p

MDTSGGGGGGAGGGAQIQGMATHGGRYVLYNVYGNLFEVASKYAPPIRPIGRGAYGIVCA

AVSSDTGEEVAIKKIGNAFDNHIDAKRTLREIKLLRHMDHENIIAIKDIIRPPRRDDFKD

VYIVTELMDTDLHQIIRSNQPLTDDHCQYFLYQLLRGLKYVHSANVLHRDLKPSNLFLNA

NCDLKIADFGLARTTSETDLMTEYVVTRWYRAPELLLNCSQYTAAIDVWSVGCILGEIIT

RQPLFPGRDYIQQLKLITELIGSPDDSSLGFLRSDNARRYMKQLPQYPRQDFRLRFRNMS

DGAVDLLERMLVFDPSRRITVDEALHHPYLASLHDINEEPTCPAPFSFDFEQPSFTEEHM

KELIWRETLAFNPDPPY*

>BdTR13cMPK6 Brdisv1BdTR13C1007022m.p

MTGDQTLASMTNYGRFSQIETAYWLISYNKQSVSAFNVPVIVAIRDIIPPAQRNSFNDVY

IAYELMDTDLHQIIRSNQALSEEHCQYFLYQILRGLKYIHSANVLHRDLKPSNLLLNANC

DLKICDFGLARTTSETDFMTEYVVTRWYRAPELLLNSSEYTAAIDVWSVGCIFMELMDRK

PLFPGRDHVHQLRLLMELIGTPNEADLDFVNENARRYIRQLPRHARQSFSEKFPHVHPSA

IDLVEKMLTFDPRQRITVEGALAHPYLASLHDISDEPVCTMPFSFDFEQHALSEEQMKDL

IHQEALAFNPDYQ*

>BdTR13cMPK7-1 Brdisv1BdTR13C1044721m.p

LLRGLKYLHSAGILHRDLKPGNLLVNANCDLKICDFGLARTNNTKGQFMTEYVVTRWYRA

PELLLCCDNYGTSIDVWSVGCIFAELLGRKPIFPRTQCLDQLKLIVNVLGTMSENDLEFI

DNPKARKYIKSLPYTPGTPLTSMYPQAHPLAIDLLQKMLVFDPSKRISVTEALEHPYMSP

LYDPSANPPAQVPIDLDIDENLGVEMIREMLWQEMLQYHPEAARMVNM*

>BdTR13cMPK11 Brdisv1BdTR13C1029714m.p

MRMEGGGAGPAAAAAAGGAHGLGEAQIKGTLTHGGRYVQYNVYGNLFEVSAKYVPPIRPV

GRGACGIICAAINVQTREEVAIKKIGNAFDNQIDAKRTLREVKLLRHMNHENVISIKDII

RPPRRENFNDVYIVYELMDTDLHHLLRSNQPLTDDHCQYFLYQVLRGLKYVHSANVLHRD

LRPSNLLLNAKCDLKIGDFGLARTTTETDFMMEYVVTRWYRAPELLLNCSEYTGAIDMWS

VGCILGEIATREPLFPGKDYVHQLRLITELLGSPDDTSLGFLRSDNARRYVRSLPQYPKQ

QFRSRFPNMSSGAMDLLERMLVFDPNKRITVDEALCHPYLASLHEINDEPVCPAPFSFDF

EQPSFTEEDIKELIWRESVKFNPEPIH*

>BdTR13cMPK14 Brdisv1BdTR13C1027735m.p

MAMLVDPPNGMGNQGKHYYSMWQTLFEIDTKYVPIKPIGRGAYGIVCSSINRETNEKVAI

KKIHNVFDNRVDALRTLRELKLLRHLRHENVISLKDIMMPVQRRSFKDVYLVYELMDTDL

HQIIKSPQGLSNDHCQYFLFQLLRGLKYLHSAEILHRDLKPGNLLVNANCDLKICDFGLA

RTNSSKGQFMTEYVVTRWYRAPELLLCCDNYGTSIDVWSVGCIFAELLGRKPIFPGTECL

NQLKLIVNVLGTMSESDLEFIDNPKARRYIKTLPYTPGVPLASMYPHAHPLAIDLLQKML

IFDPTKRISVTEALEHPYMSPLYDPSANPPAQVPIDLDLDENISADMIREMMWQEMLHYH

PEAATAISM*

>BdTR13cMPK16 Brdisv1BdTR13C1016526m.p

MDFFTEYGEGNRYKIEEVIGKGSYGVVCSALDTHTGDKVAIKKINDIFEHVSDATRILRE

IKLLRLLRHPDIVEIKHILLPPSRREFKDIYVVFELMESDLHQVIKANDDLTPEHYQFFL

YQLLRGLKYIHTANVFHRDLKPKNILANADCKLKICDFGLARVAISDPPPAIFWTDYIAT

RWYRAPELCGSFFSKYTPAIDIWSIGCIFAELLTGKPLFPGKNVVHQLDIITDLLGTPSA

ETIARIRNEKARRYLSSMRRKKTVPFTQKFPNADPLALNLLERMLAFDPKDRPSAEEALA

DPYFKNIASVDREPSAQPITKLEFEFERRRITKDDIRELIYREILEYHPNMLREFLEGAE

PTGFMYPSAVDHFKKQFTFLEEHYAKGSTAAPPERQHNSLPRPSVIYSDNRPQNAANITD

DLSRCAIRDNTQKAQKDSASVGANRIPHGAAAAAARPGKVVGSVLRYGNCSTSGTEQYEQ

RRVVRSPGIAPNGVTSGSSYPRRNNTCKSETGEAERIDTNQGGPPKPYVANKLPATVDGR

NGHW*

>BdTR13cMPK17 Brdisv1BdTR13C1005245m.p

MVGGGGIVDGFRRLFHRRTPSGSGPSSNQSSAGEESSDIDAVVEDLDLVGLRAIRVPKRK

MPLPVESHKKNILEKEFFTEYGEESQYQIQEVIGKGSYGVVAAAVDTRTGERVAIKKIND

VFEHVSDATRILREVKLLRLLRHPDVVEIKHIMLPPSRREFQDIYVVFELMESDLHQVIR

ANDDLTPEHYQFFLYQLLRALKYIHAANVFHRDLKPKNILANSDCKLKICDFGLARVSFN

DAPTAIFWTDYVATRWYRAPELCGSFFSKYTPAIDIWSIGCIFAELLTGRPLFPGKNVVH

QLDIITDLLGTPSSETLSRIRNEKARRYLSCMRKKHPVPLTQKFPNVDPLALRLLGRLLA

FDPKDRPSAEEALADPYFAVLANVEREPSRNPISKLEFEFERRKVTKDDVRELIYREILE

YHPQMLQEYMQGGEQLSFLYPSGVDRFKRQFAHLEENYSKGERGSPLQRKHASLPRQRVG

ASNDNNDQHASDQERGADSIAPATGNPPGSQDAGQQHVSGGQNGVGSTNISPRSYQKSAS

ISASKCVVVNANKNPEYDDDISEEMEGAVDGLSEKVSRMHP*

>BdTR13cMPK20-1 Brdisv1BdTR13C1017760m.p

MQQDQRKKSSAEAEFFTEYGDASRYKIQEIVGKGSYGVVCSAIDVHTGEKVAIKKIHDIF

EHISDAARILREIKLLRLLRHPDIVEIKHIMLPPSRRDFKDIYVVFELMESDLHQVIKAN

DDLTKEHYQFFLYQLLRALKYIHTANVYHRDLKPKNILANSNCKLKICDFGLARVAFNDT

PTTIFWTDYVATRWYRAPELCGSFFSKYTPAIDVWSIGCIFAEGLTGKPLFPGKNVVHQL

DLMTDLLGTPSMDTISRVRNEKARRYLSSMRKKEPIPFSQKFPNADPLALDLLQRLLAFD

PKDRPTAGEALSHPYFKGLAKVEREPSCQPITKMEFEFERRRATKEDIRELIFREILEYH

PQLLKDYINGTERTTFLYPSAVDQFRKQFAHLEENSESGPVVPMDRKHTSLPRSTIVHSA

PIHAKEQPRIGPSRDRPLTDESYKNPRDSEKYSGNVPRSSHHSQAPQRVPTARPGRVVGP

VLPYENVGTSHPYDPRRVAMNSGYPPQQQIPQTYGYYQTTGKSACSEPSQAERYTLHQQA

YACANSSTAPDVALDMRAPPFHQSAGPKSGSSDRLTAETNLYTRSLNGIVATTSGVAASA

HRKGSVVPYGMSQMY*

>BdTR13cMPK20-2 Brdisv1BdTR13C1014043m.p

MQQPQQQGQRNKSTDVDFFSEYGDANRYRIQEVIGKGSYGVVCSAMDMQTRQKVAIKKIH

NIFDHVSDAARILREIKLLRLLRHPDVVEIKHIMLPPSRKDFKDIYVVFELMESDLHQVI

KANDDLTKEHYQFFLYQLLRALKYIHTASVYHRDLKPKNILANSNCKLKICDFGLARVAF

NDTPTTVFWTDYVATRWYRAPELCGSFFTKYTPAIDIWSIGCIFAEVLTGKPLFPGKNVV

HQLDLMTDLLGTPSMDTISRVRNEKARRYLSSMRKKDPVPFSKKFPNADPLGLKLLEKLL

AFDPKDRPTAEEALTDPYFKSLSKPDREPSCQPIRKVEFDFEHRRMSKDDIRELIFQEIL

EYHPQLLKNYIDGTEKTTFLYPSAVDQFKKQFSHLEESDGSGPVVPTERKHASLPRSTTV

HSTPIPAKEQPLVASSRGRPIANEPCKPWAPGNVPGASQTAHVAQAGRAVGVGSVPPYES

GSGKYPYDATSRPAVSSGYPPQQKIPQTYGYHHHHQTPGAGQSSQAMGGYACGYTKGTTP

PPAAAQDMRASPYHHRSAGTKNDPLNRLAAESDIYTRSLNGIVAAAASAGTGAHRKGGAV

PFGMSGMY*

>BdTR13cMPK20-3 Brdisv1BdTR13C1042291m.p

MQHGDLHKKSAAEMDFFTAYDDANRYKILEVIGKGSYGLVCSANDLQTGEKVAIKKIHNI

FEHISDAARILREIKLLRLLRHPDVVEIKHILLPPSKKDFKDIYVVFELMESDLHQVIKA

NDDLTREHYQFFLYQMLRALKYMHTANVYHRDLKPKNVLANANCKLKICDFGLARVAFSD

APTTVFWTDYVATRWYRAPELCGSFYSK

>BdTR13cMPK20-4 Brdisv1BdTR13C1017954m.p

MQTTEQQRKKGSSEMDFFSEYGDANRYKIQEVIGKGSYGVVCSAIDQHTGDKVAIKKIHN

IFEHLSDAARILREIKLLRLLRHPDIVEIRHIMLPPSRRDFKDIYVVFELMDTDLHQVIK

ANDDLTKEHHQFFLYQMLRALKYIHTANVYHRDLKPKNILANANCKLKICDFGLARVAFN

DTPTTVFWTDYVATRWYRAPELCGSFFTKYSRAIDIWSIGCIFAEILTGKPLFPGKNVVH

QLDLMTDLLGTPSLDTVSRIRNEKARRYLSSMRKKQSVSFSERFPKADPAALKLLQRLLA

FDPKDRPTAEEALADPYFKGLGKVEREPSCQPITKMEFEFERKNVTKADVKELIFREILE

YHPQLLKDYMNGTEKTNFLYPSAVDNFRRQFANLEENGGKGGAVVPPDRKHVSLPRNTTV

HSTPIPPKDQKYSQVPQRIPTGRPGRVVGPVIPFENSSTMDPYSQRRVARNPVLPAAATN

LSAYAYRNSDNSERELQQELEKDRMQYHPMQRFMDAKMVSPDLRSTSYYMPKGVPKADVA

ERSALQSNMMQGIAPFNGIATVGGAFNKVSAVQYGVSRMY*

>BdTR13cMPK20-5 Brdisv1BdTR13C1014187m.p

MFSDIFSREGVAFSRVALRQELLSDQWFTTAGGKGQCYLVPPGTTVFLVNSEEMGFFSEY

VDASRYKILEIIGKGSYGVVCSAIDQETGDKVAIKKIQNIFEHLSDAARILREIKLLRLL

RHPDIVQIKHIMLPPSRRDFRDIYVVFELMDTDLHQVIKANDDLTKEHYQFFLYQMLRAL

KYIHTANVYHRDLKPKNILANANCKLKICDFGLARVAFNDTPTTVFWTDYVATRWYRAPE

LCGSFFTKYSPAIDTWSIGCIFAEILTGKPLFPGKNVVHQLDLMTDLLGTPSTETISRIR

NDKARKYLSSMRRKQPIPFSEKFPNADPSALKLLERLLAFDPKDRPTAEEALAHPYFKRL

ARVEREPSCQQPISKTEFEFERRKFTKEDVKELIFREILEYHPKLLKDYMNGSEKTSFLY

PSAVDNFRRQFANLEIDGGRSGAADRKHFSLPRTTTVHSAPILPTNGPTSQVPQRIPTAR

PGRVVSSAMQTDNPSVSDRHNGRRVARDPAVPPAAAYHLKSDYSDRQHQQEFEKDRVRPD

RQRQEELEKDRMQYRPGHHSMDAKVAPEISPYMRSSPYYIPPFNGIAAVASGYSKVAAVT

RMY*

>BdTR13cMPK21-1 Brdisv1BdTR13C1014087m.p

MGGLIRWLRHHPSRRVSSSSSSHLPSNTTSSSSTSDLRAHSLPQHQGDHHGEVVVEWEDA

AEGPDSDPEEYIVVVLGDDEQGVVAARAPVRTKPPRVMDPGKKTSESEFFTEYGEANRYK

VSEVIGKGSYGVVAAAVDTQTGERCAIKKINDVFDHVSDATRILREIKLLRLLRHPDIVE

IKHIMLPPSRREFRDIYVVFELMESDLHQVIKANDDLTPEHHQFFLYQLLRGMKYIHTAN

VFHRDLKPKNILANADCKLKICDFGLARVSFNDGAPSAIFWTDYVATRWYRAPELCGSFF

SKYTPAIDIWSVGCIFAEMLTGKPLFPGKNVVHQLDLMTDLLGTPSAESLSKIRNEKARR

YLSNMRKKPKVPLTKKFPGIDPMALHLLERLLAFDPKDRPSADEALTDPYFTGLANSERE

PITQPISKLEFEFEKRKLAKDDVRELIYREILEYHPHMLQEYLRGGGDQMSFMFPSGVDR

FKRQFAHLEEGGAKGEKSSPQLRQNASLPRERVIGNKHGDGDYNMKLNVGEKPELASVSD

GISKPLMSARSLLKSETMSASKCIGEIKNKDEDSLSECVEGTDDDVSQKIAQLKT*

>BdTR13cMPK21-2 Brdisv1BdTR13C1017842m.p

MARKGSDEAEFFTEYGEANRYEVGEVVGKGSYGVVAAAVDTHTGERVAIKKINDVFEHVS

DATRILREIKLLRLLRHPDIVEIKHIILPPSRREFRDIYIIFELMESDLHQVIKANDDLT

PEHHQFFFYQLLRGMKYIHAANVFHRDLKPRNILANADCKLKICDFGLARVSFNDTPSAI

FWTDYVATRWYRAPELCGSFFSKYTPAIDIWSIGCIFAEMLSGRPLFPGKNVVHQLDLMT

DLLGTPSAESLSRIRNEKARRYLGNMRKKHPVPFSQKFPGVDPMALDLLERLLAFDPKDR

PTAAEALADPYFTGLANSDREPTTQPISKLEFEFERRKLARDDVRELIYREILEYHPQML

HEYHHGGDQANFVYPSGVDRFKRQFVHLEEGVTKGEKTSPQLRQHASLPRERIIGIGDEL

GRPNADYCIKLHVGEEPGHTSVTDGLSKPLLNARNFLKSESISASQCVVIKEKREKDEES

MSEYMHEASDATPK*

>BdTR13aMPK3 Brdisv1BdTR13a1011439m.p

MDGAPVAEFRPTMTHGGRFLLYNIFGNQFEITAKYQPPIMPIGRGAYGIVCSVMNFETRE

MVAIKKIANAFDNNMDAKRTLREIKLLRHLDHENIVGLRDVIPPAIPQSFNDVYIATELM

DTDLHHIIRSNQELSEEHCQYFLYQLLRGLKYIHSANVIHRDLKPSNLLLNANCDLKICD

FGLARPSSESDMMTEYVVTRWYRAPELLLNSTDYSAAIDVWSVGCIFMELINRAPLFPGR

DHMHQMRLITEVIGTPTDDDLGFIRNEDARRYMRHLPQFPRRPFPAQFPRVQPAALDLIE

RMLAFNPLQRITVEEALEHPYLERLHDIADEPICTDPFSFDFEQHPLTEDQMKQLIFNEA

LELNPNFRY*

>BdTR13aMPK4 Brdisv1BdTR13a1037138m.p

MDTSGGGGGGAGGAAQIQGMATHGGRYVLYNVYGNLFEVASKYAPPIRPIGRGAYGIVCA

AVSSDTGEEVAIKKIGNAFDNHIDAKRTLREIKLLRHMDHENIIAIKDIIRPPRRDDFKD

VYIVTELMDTDLHQIIRSNQPLTDDHCQYFLYQLLRGLKYVHSANVLHRDLKPSNLFLNA

NCDLKIADFGLARTTSETDLMTEYVVTRWYRAPELLLNCSQYTAAIDVWSVGCILGEIIT

RQPLFPGRDYIQQLKLITELIGSPDDSSLGFLRSDNARRYMKQLPQYPRQDFRLRFRNMS

DGAVDLLERMLVFDPSRRITVDEALHHPYLASLHDINEEPTCPAPFSFDFEQPSFTEEHM

KELIWRETLAFNPDPPY*

>BdTR13aMPK6 Brdisv1BdTR13a1008255m.p

MDGGAQPPDAEMADAGAAVPGAGAGAAGTMENIQATLSHGGRFIQYNIFGNVFEVTVKYK

PPILPIGKGAYGIVCSALNSETGEQVAIKKIANAFDNKIDAKRTLREIKLLRHMDHENIV

AIRDIIPPAQRNSFNDVYIAYELMDTDLHQIIRSNQALSEEHCQYFLYQILRGLKYIHSA

NVLHRDLKPSNLLLNANCDLKICDFGLARTTSETDFMTEYVVTRWYRAPELLLNSSEYTA

AIDVWSVGCIFMELMDRKPLFPGRDHVHQLRLLMELIGTPNEADLDFVNENARRYIRQLP

RHARQSFSEKFPHVHPSAIDLVEKMLTFDPRQRITVEGALAHPYLASLHDISDEPVCTMP

FSFDFEQHALSEEQMKDLIHQEALAFNPDYQ*

>BdTR13aMPK7-1 Brdisv1BdTR13a1047101m.p

MHCSFDTTTKSDWAGHFPNYLICDGILQEGTAREISKVLHREITWESDASLLEDVLIKFK

KHLKIPLLLLRGLKYLHSAGILHRDLKPGNLLVNANCDLKICDFGLARTNNTKGQFMTEY

VVTCLYRAPELLLCCDNYGTSIDVWSVGCIFAELLGRKPIFPGTECLNQLKLIVNVLGTM

SENDLEFIDNPKARKYIKSLPYTPGTPLTSMYPQAHPLAIDLLQKMLVFDPSKRISVTEA

LEHPYMSPLYDPSANPPAQVPIDLDIDENLGVEMIREMLWQEMLQYHPEAARMVNM*

>BdTR13aMPK11 Brdisv1BdTR13a1035020m.p

MRMEGGGAGPAAAAAAGGAHGLGEAQIKGTLTHGGRYVQYNVYGNLFEVSAKYVPPIRPV

GRGACGIICAAINVQTREEVAIKKIGNAFDNQIDAKRTLREVKLLRHMNHENVISIKDII

RPPRRENFNDVYIVYELMDTDLHHLLRSNQPLTDDHCQYFLYQVLRGLKYVHSANVLHRD

LRPSNLLLNAKCDLKIGDFGLARTTTETDFMMEYVVTRWYRAPELLLNCSEYTGAIDMWS

VGCILGEIATREPLFPGKDYVHQLRLITELLGSPDDTSLGFLRSDNARRYVRSLPQYPKQ

QFRSRFPNMSSGAMDLLERMLVFDPNKRITVDEALCHPYLASLHEINDEPVCPAPFSFDF

EQPSFTEEDIKELIWRESVKFNPEPIH*

>BdTR13aMPK14 Brdisv1BdTR13a1032768m.p

MAMLVDPPNGMGNQGKHYYSMWQTLFEIDTKYVPIKPIGRGAYGIVCSSINRETNEKVAI

KKIHNVFDNRVDALRTLRELKLLRHLRHENVISLKDIMMPVQRRSFKDVYLVYELMDTDL

HQIIKSPQGLSNDHCQYFLFQLLRGLKYLHSAEILHRDLKPGNLLVNANCDLKICDFGLA

RTNSSKGQFMTEYVVTRWYRAPELLLCCDNYGTSIDVWSVGCIFAELLGRKPIFPGTECL

NQLKLIVNVLGTMSESDLEFIDNPKARRYIKTLPYTPGVPLASMYPHAHPLAIDLLQKML

IFDPTKRISVTEALEHPYMSPLYDPSANPPAQVPIDLDLDENISADMIREMMWQEMLHYH

PEAATAISM*

>BdTR13aMPK16 Brdisv1BdTR13a1019440m.p

MDFFTEYGEGNRYKIEEVIGKGSYGVVCSALDTHTGDKVAIKKINDIFEHVSDATRILRE

IKLLRLLRHPDIVEIKHILLPPSRREFKDIYVVFELMESDLHQVIKANDDLTPEHYQFFL

YQLLRGLKYIHTANVFHRDLKPKNILANADCKLKICDFGLARVAISDTPTAIFWTDYIAT

RWYRAPELCGSFFSKYTPAIDIWSIGCIFAELLTGKPLFPGKNVVHQLDIITDLLGTPSA

ETIARIRNEKARRYLSSMRRKKTVPFTQKFPNADPLALNLLERMLAFDPKDRPSAEEALA

DPYFKNIASVDREPSAQPITKLEFEFERRRITKDDIRELIYREILEYHPNMLREFLEGAE

PTGFMYPSAVDHFKKQFTFLEEHYAKGSTAAPPERQHNSLPRPSVIYSDNRPQNAANITD

DLSRCAIRDNTQKAQKDSASVGANRIPHGAAAAAARPGKVVGSVLRYGNCSTSGTEQYEQ

RRVVRSPGIAPNGVTSGSSYPRRNNTCKSETGEAERIDTNQGGPPKPYVANKLPATVDGR

NGHW*

>BdTR13aMPK17 Brdisv1BdTR13a1005975m.p

MVGGGGIVDGFRRLFHRRTPSGSGPSSNQSSAGEESSDIDAVVEDLDLVGLRAIRVPKRK

MPLPVESHKKNILEKEFFTEYGEASQYQIQEVIGKGSYGVVAAAVDTRTGERVAIKKIND

VFEHVSDATRILREVKLLRLLRHPDVVEIKHIMLPPSRREFQDIYVVFELMESDLHQVIR

ANDDLTPEHYQFFLYQLLRALKYIHAANVFHRDLKPKNILANSDCKLKICDFGLARVSFN

DAPTAIFWTDYVATRWYRAPELCGSFFSKYTPAIDIWSIGCIFAELLTGRPLFPGKNVVH

QLDIITDLLGTPSSETLSRIRNEKARRYLSCMRKKHPVPLTQKFPNVDPLALRLLGRLLA

FDPKDRPSAEEALADPYFAVLANVEREPSRNPISKLEFEFERRKVTKDDVRELIYREILE

YHPQMLQEYMQGGEQLSFLYPSGVDRFKRQFAHLEENYSKGERGSPLQRKHASLPRQRVG

ASNDNNDQHASDQERGADSIAPATGNPPGSQDAGQQHVSGGQNGVGSTNISPRSYQKSAS

ISASKCVVVNANKNPEYDDDISEEMEGAVDGLSEKVSRMHP*

>BdTR13aMPK20-1 Brdisv1BdTR13a1020921m.p

MQQDQRKKSSAEAEFFTEYGDASRYKIQEIVGKGSYGVVCSAIDVHTGEKVAIKKIHDIF

EHISDAARILREIKLLRLLRHPDIVEIKHIMLPPSRRDFKDIYVVFELMESDLHQVIKAN

DDLTKEHYQFFLYQLLRALKYIHTANVYHRDLKPKNILANSNCKLKICDFGLARVAFNDT

PTTIFWTDYVATRWYRAPELCGSFFSKYTPAIDVWSIGCIFAEVLTGKPLFPGKNVVHQL

DLMTDLLGTPSMDTISRVRNEKARRYLSSMRKKEPIPFSQKFPNADPLALDLLQRLLAFD

PKDRPTAGEALSHPYFKGLAKVEREPSCQPITKMEFEFERRRATKEDIRELIFREILEYH

PQLLKDYINGTERTTFLYPSAVDQFRKQFAHLEENSESGPVVPMDRKHTSLPRSTIVHSA

PIHAKEQPRIGPSRDRPLTDESYKNPRDSEKYSGNVPRSSHHSQAPQRVPTARPGRVVGP

VLPYENVGTSHPYDPRRVAMNSGYPPQQQIPQTYGYYQTTGKSACSEPSQAERYTLHQQA

YACANSSTAPDVALDMRAPPFHQSAGPKSGSSDRLTAETNLYTRSLNGIVATTSGVAASA

HRKVSVVPYGMSQMY*

>BdTR13aMPK20-2 Brdisv1BdTR13a1016554m.p

MQQPQQQGQRNKSTDVDFFSEYGDANRYRIQEVIGKGSYGVVCSAMDMQTRQKVAIKKIH

NIFDHVSDAARILREIKLLRLLRHPDVVEIKHIMLPPSRKDFKDIYVVFELMESDLHQVI

KANDDLTKEHYQFFLYQLLRALKYIHTASVYHRDLKPKNILANSNCKLKICDFGLARVAF

NDTPTTVFWTDYVATRWYRAPELCGSFFTKYTPAIDIWSIGCIFAEVLTGKPLFPGKNVV

HQLDLMTDLLGTPSMDTISRVRNEKARRYLSSMRKKDPVPFSKKFPNADPLGLKLLEKLL

AFDPKDRPTAEEALTDPYFKSLSKPDREPSCQPIRKVEFDFEHRRMSKDDIRELIFQEIL

EYHPQLLKNYIDGTEKTTFLYPSAVDQFKKQFSHLEESDGSGPVVPTERKHASLPRSTTV

HSTPIPAKEQPLVASSRGRPIANEPCKPWAPGNVPGASQTAHVAQAGRAVGVGSVPPYES

GSGKYPYDATSRPAVSSGYPPQQKIPQTYGYHHHHQTPGAGQSSQAMGGYACGYTKGTTP

PPAAAQDMRASPYHHRSAGTKNDPLNRLAAESDIYTRSLNGIVAAAASAGTGAHRKVGAV

PFGMSGMY*

>BdTR13aMPK20-3 Brdisv1BdTR13a1006843m.p

MQHGDLHKKSAAEMDFFTAYDDANRYKILEVIGKGSYGLVCSANDLQTGEKVAIKKIHNI

FEHISDAARILREIKLLRLLRHPDVVEIKHILLPPSKKDFKDIYVVFELMESDLHQVIKA

NDDLTREHYQFFLYQMLRALKYMHTANVYHRDLKPKNVLANANCKLKICDFGLARVAFSD

APTTVFWTDYVATRWYRAPELCGSFYSKYTPAIDIWSIGCIFAEVLIGKPLFPGKNVVHQ

LDLITDILGTPSLDAISRVRNDKARKYLTCMRKKQPASFSQKFPKADPLALQLLRRLLAF

DPKDRPSAEEALADPYFNGLAKVEREPSCQPIPKIEFEFEGRRVTKEDIKELIFEEILEY

HPQLLKEHIIGKERPNFVHLSAVDQFKKHFTQLEENDNETGAAVSLQRKHSSLPRQAFNH

R*

>BdTR13aMPK20-4 Brdisv1BdTR13a1021151m.p

MQTTEQQRKKGSSEMDFFSEYGDANRYKIQEVIGKGSYGVVCSAIDQHTGDKVAIKKIHN

IFEHLSDAARILREIKLLRLLRHPDIVEIRHIMLPPSRRDFKDIYVVFELMDTDLHQVIK

ANDDLTKEHHQFFLYQMLRALKYIHTANVYHRDLKPKNILANANCKLKICDFGLARVAFN

DTPTTVFWTDYVATRWYRAPELCGSFFTKYSRAIDIWSIGCIFAEILTGKPLFPGKNVVH

QLDLMTDLLGTPSLDTVSRIRNEKARRYLSSMRKKQSVSFSERFPKADPAALKLLQRLLA

FDPKDRPTAEEALADPYFKGLGKVEREPSCQPITKMEFEFERKNVTKADVKELIFREILE

YHPQLLKDYMNGTEKTNFLYPSAVDNFRRQFANLEENGGKGGAVVPPDRKHVSLPRNTTV

HSTPIPPKDQKYSQVPQRIPTGRPGRVVGPVIPFENSSTMDPYSQRRVARNPVLPAAATN

LSAYAYRNSDNSERELQQELEKDRMQYHPMQRFMDAKMVSPDLRSTSYYMPKGVPKADVA

ERSALQSNMMQGIAPFNGIATVGGAFNKVSAVQYGVSRMY*

>BdTR13aMPK20-5 Brdisv1BdTR13a1016712m.p

MFSDIFSREGVAFSRVALRQELLSDQWFTTAGGKGQCYLVPPGTTVFLVNSEEMGFFSEY

VDASRYKILEIIGKGSYGVVCSAIDQETGDKVAIKKIQNIFEHLSDAARILREIKLLRLL

RHPDIVQIKHIMLPPSRRDFRDIYVVFELMDTDLHQVIKANDDLTKEHYQFFLYQMLRAL

KYIHTANVYHRDLKPKNILANANCKLKICDFGLARVAFNDTPTTVFWTDYVATRWYRAPE

LCGSFFTKYSPAIDTWSIGCIFAEILTGKPLFPGKNVVHQLDLMTDLLGTPSTETISRIR

NDKARKYLSSMRRKQPIPFSEKFPNADPSALKLLERLLAFDPKDRPTAEEALAHPYFKRL

ARVEREPSCQQPISKTEFEFERRKFTKEDVKELIFREILEYHPKLLKDYMNGSEKTSFLY

PSAVDNFRRQFANLEIDGGRSGAADRKHFSLPRTTTVHSAPILPTNGPTSQVPQRIPTAR

PGRVVSSAMQTDNPSVSDRHNGRRVARDPAVPPAAAYHLKSDYSDRQHQQEFEKDRVRPD

RQRQEELEKDRMQYRPGHHSMDAKVAPEISPYMRSSPYYIPPFNGIAAVASGYSKVAAVT

RMY*

>BdTR13aMPK21-1 Brdisv1BdTR13a1016601m.p

MGGLIRWLRHHRSRRVSSSSSSHLPSNTTSSSSTSDLRAHSLPQHQGDHHGEVVVEWEDA

AEGPDSDPEEYIVVVLGDDEQGVVAARAPVRTKPPRVMDPGKKTSESEFFTEYGEANRYK

VSEVIGKGSYGVVAAAVDTQTGERCAIKKINDVFDHVSDATRILREIKLLRLLRHPDIVE

IKHIMLPPSRREFRDIYVVFELMESDLHQVIKANDDLTPEHHQFFLYQLLRGMKYIHTAN

VFHRDLKPKNILANADCKLKICDFGLARVSFNDGAPSAIFWTDYVATRWYRAPELCGSFF

SKYTPAIDIWSVGCIFAEMLTGKPLFPGKNVVHQLDLMTDLLGTPSAESLSKIRNEKARR

YLSNMRKKPKVPLTKKFPGIDPMALHLLERLLAFDPKDRPSADEALTDPYFTGLANSERE

PITQPISKLEFEFEKRKLAKDDVRELIYREILEYHPHMLQEYLRGGGDQMSFMFPSGVDR

FKRQFAHLEEGGAKGEKSSPQLRQNASLPRERVIGNKHGDGDYNMKLNVGEKPELASVSD

GISKPLMSARSLLKSETMSASKCIGEIKNKDEDSLSECVEGTDDDVSQKIAQLKT*

>BdTR13aMPK21-2 Brdisv1BdTR13a1021039m.p

MARKGSDEAEFFTEYGEANRYEVGEVVGKGSYGVVAAAVDTHTGERVAIKKINDVFEHVS

DATRILREIKLLRLLRHPDIVEIKHIILPPSRREFRDIYIIFELMESDLHQVIKANDDLT

PEHHQFFFYQLLRGMKYIHAANVFHRDLKPRNILANADCKLKICDFGLARVSFNDTPSAI

FWTDYVATRWYRAPELCGSFFSKYTPAIDIWSIGCIFAEMLSGRPLFPGKNVVHQLDLMT

DLLGTPSAESLSRIRNEKARRYLGNMRKKHPVPFSQKFPGVDPMALDLLERLLAFDPKDR

PTAAEALADPYFTGLANSDREPTTQPISKLEFEFERRKLARDDVRELIYREILEYHPQML

HEYHHGGDQANFVYPSGVDRFKRQFVHLEEGVTKGEKTSPQLRQHASLPRERIIGIGDEL

GRPNADYCIKLHVGEEPGHTSVTDGLSKPLLNARNFLKSESISASQCVVIKEKREKDEES

MSEYMHEASDATPK*

>Bis-1MPK3 Brdisv1Bis-11010343m.p

MDGAPVAEFRPTMTHGGRFLLYNIFGNQFEITAKYQPPIMPIGRGAYGIVCSVMNFETRE

MVAIKKIANAFDNNMDAKRTLREIKLLRHLDHENIVGLRDVIPPAIPQSFNDVYIATELM

DTDLHHIIRSNQELSEEHCQYFLYQLLRGLKYIHSANVIHRDLKPSNLLLNANCDLKICD

FGLARPSSESDMMTEYVVTRGYRAPELLLNSTDYSAAIDVWSVGCIFMELINRAPLFPGR

DHMHQMRLITEVIGTPTDDDLGFIRNEDARRYMRHLPQFPRRPFPAQFPRVQPAALDLIE

RMLAFNPLQRITVEEALEHPYLERLHDIADEPICTDPFSFDFEQHPLTEDQMKQLIFNEA

LELNPNFRY*

>Bis-1MPK4 Brdisv1Bis-11011144m.p

MDTSGGGGGGAGGAAQIQGMATHGGRYVLYNVYGNLFEVASKYAPPIRPIGRGAYGIDVP

YPFRMVVFVYRLRTRVGEAAAVSSDTGEEVAIKKIGNAFDNHIDAKRTLREIKLLRHMDH

ENIIAIKDIIRPPRRDDFKDVYIVTELMDTDLHQIIRSNQPLTDDHCQYFLYQLLRGLKY

VHSANVLHRDLKPSNLFLNANCDLKIADFGLARTTSETDLMTEYVVTRWYRAPELLLNCS

QYTAAIDVWSVGCILGEIITRQPLFPGRDYIQQLKLITELIGSPDDSSLGFLRSDNARRY

MKQLPQYPRQDFRLRFRNMSDGAVDLLERMLVFDPSRRITVDEALHHPYLASLHDINEEP

TCPAPFSFDFEQPSFTEEHMKELIWRETLAFNPDPPY*

>Bis-1MPK6 Brdisv1Bis-11007534m.p

MDGGAQPPDAEMADAGAAVPGAGAGGAGTMENIQATLSHGGRFIQYNIFGNVFEVTVKYK

PPILPIGKGAYGIVCSALNSETGEQVAIKKIANAFDNKIDAKRTLREIKLLRHMDHENIV

AIRDIIPPAQRNSFNDVYIAYELMDTDLHQIIRSNQALSEEHCQYFLYQILRGLKYIHSA

NVLHRDLKPSNLLLNANCDLKICDFGLARTTSETDFMTEYVVTRWYRAPELLLNSSEYTA

AIDVWSVGCIFMELMDRKPLFPGRDHVHQLRLLMELIGTPNEADLDFVNENARRYIRQLP

RHARQSFSEKFPHVHPSAIDLVEKMLTFDPRQRITVEGALAHPYLASLHDISDEPVCTMP

FSFDFEQHALSEEQMKDLIHQEALAFNPDYQ*

>Bis-1MPK7-1 Brdisv1Bis-11045356m.p

MLLRGLKYLHSAGILHRDLKPGNLLVNANCDLKICDFGLARTNNTKGQFMTEYVVTRWYR

APELLLCCDNYGTSIDVWSVGCIFAELLGRKPIFPGTECLNQLKLIVNVLGTMSENDLEF

IDNPKARKYIKSLPYTPGTPLTSMYPQAHPLAIDLLQKMLVFDPSKRISVTEALEHPYMS

PLYDPSANPPAQVPIDLDIDENLGVEMIREMLWQGMLQYHPEAARMVNM*

>Bis-1MPK11 Brdisv1Bis-11031556m.p

MRMEGGGAGPAAAAAAGGAHGLGEAQIKGTLTHGGRYVQYNVYGNLFEVSAKYVPPIRPV

GRGACGIICAAINVQTREEVAIKKIGNAFDNQIDAKRTLREVKLLRHMNHENVISIKDII

RPPRRENFNDVYIVYELMDTDLHHLLRSNQPLTDDHCQYFLYQVLRGLKYVHSANVLHRD

LRPSNLLLNAKCDLKIGDFGLARTTTETDFMMEYVVTRWYRAPELLLNCSEYTGAIDMWS

VGCILGEIATREPLFPGKDYVHQLRLITELLGSPDDTSLGFLRSDNARRYVRSLPQYPKQ

QFRSRFPNMSSGAMDLLERMLVFDPNKRITVDEALCHPYLASLHEINDEPVCPAPFSFDF

EQPSFTEEDIKELIWRESVKFNPEPIH*

>Bis-1MPK14 Brdisv1Bis-11029431m.p

MAMLVDPPNGMGNQGKHYYSMWQTLFEIDTKYVPIKPIGRGAYGIVCSSINRETNEKVAI

KKIHNVFDNRVDALRTLRELKLLRHLRHENVISLKDIMMPVQRRSFKDVYLVYELMDTDL

HQIIKSPQGLSNDHCQYFLFQLLRGLKYLHSAEILHRDLKPGNLLVNANCDLKICDFGLA

RTNSSKGQFMTEYVVTRWYRAPELLLCCDNYGTSIDVWSVGCIFAELLGRKPIFPGTECL

NQLKLIVNVLGTMSESDLEFIDNPKARRYIKTLPYTPGVPLASMYPHAHPLAIDLLQKML

IFDPTKRISVTEALEHPYMSPLYDPSANPPAQVPIDLDLDENISADMIREMMWQEMLHYH

PEAATAISM*

>Bis-1MPK16 Brdisv1Bis-11017659m.p

MDFFTEYGEGNRYKIEEVIGKGSYGVVCSALDTHTGDKVAIKKINDIFEHVSDATRILRE

IKLLRLLRHPDIVEIKHILLPPSRREFKDIYVVFELMESDLHQVIKANDDLTPEHYQFFL

YQLLRGLKYIHTANVFHRDLKPKNILANADCKLKICDFGLARVAISDTPTAIFWTDYIAT

RWYRAPELCGSFFSKYTPAIDIWSIGCIFAELLTGKPLFPGKNVVHQLDIITDLLGTPSA

ETIARIRNEKARRYLSSMRRKKTVPFTQKFPNADPLALNLLERMLAFDPKDRPSAEEALA

DPYFKNIASVDREPSAQPITKLEFEFERRRITKDDIRELIYREILEYHPNMLREFLEGAE

PTGFMYPSAVDHFKKQFTFLEEHYAKGSTAAPPERQHNSLPRPSVIYSDNRPQNAANITD

DLSRCAIRDNTQKAQKDSASVGANRIPHGAAAAAARPGKVVGSVLRYGNCSTSGTEQYEQ

RRVVRSPGIAPNGVTSGSSYPRRNNTCKSETGEAERIDTNQGGPPKPYVANKLPATVDGR

NGHW*

>Bis-1MPK17 Brdisv1Bis-11005576m.p

MVGGGGIVDGFRRLFHRRTPSGSGPSSNQSSAGEESSDIDAVVEDLDLVGLRAIRVPKRK

MPLPVESHKKNILEKEFFTEYGEESQYQIQEVIGKGSYGVVAAAVDTRTGERVAIKKIND

VFEHVSDATRILREVKLLRLLRHPDVVEIKHIMLPPSRREFQDIYVVFELMESDLHQVIR

ANDDLTPEHYQFFLYQLLRALKYIHAANVFHRDLKPKNILANSDCKLKICDFGLARVSFN

DAPTAIFWTDYVATRWYRAPELCGSFFSKYTPAIDIWSIGCIFAELLTGRPLFPGKNVVH

QLDIITDLLGTPSSETLSRIRNEKARRYLSCMRKKHPVPLTQKFPNVDPLALRLLGRLLA

FDPKDRPSAEEALADPYFAVLANVEREPSRNPISKLEFEFERRKVTKDDVRELIYREILE

YHPQMLQEYMQGGEQLSFLYPSGVDRFKRQFAHLEENYSKGERGSPLQRKHASLPRQRVG

ASNDNNDQHASDQERGADSIAPATGNPPGSQDAGQQHVSGGQNGVGSTNISPRSYQKSAS

ISASKCVVVNANKNPEYDDDISEEMEGAVDGLSEKVSRMHP*

>Bis-1MPK20-1 Brdisv1Bis-11018992m.p

MQQDQRKKSSAEAEFFTEYGDASRYKIQEIVGKGSYGVVCSAIDVHTGEKVAIKKIHDIF

EHISDAARILREIKLLRLLRHPDIVEIKHIMLPPSRRDFKDIYVVFELMESDLHQVIKAN

DDLTKEHYQFFLYQLLRALKYIHTANVYHRDLKPKNILANSNCKLKICDFGLARVAFNDT

PTTIFWTDYVATRWYRAPELCGSFFSKYTPAIDVWSIGCIFAEVLTGKPLFPGKNVVHQL

DLMTDLLGTPSMDTISRVRNEKARRYLSSMRKKEPIPFSQKFPNADPLALDLLQRLLAFD

PKDRPTAGEALSHPYFKGLAKVEREPSCQPITKMEFEFERRRATKEDIRELIFREILEYH

PQLLKDYINGTERTTFLYPSAVDQFRKQFAHLEENSESGPVVPMDRKHTSLPRSTIVHSA

PIHAKEQPRIGPSRDRPLTDESYKNPRDSEKYSGNVPRSSHHSQAPQRVPTARPGRVVGP

VLPYENVGTSHPYDPRRVAMNSGYPPQQQIPQTYGYYQTTGKSACSEPSQAERYTLHQQA

YACANSSTAPDVALDMRAPPFHQSAGPKSGSSDRLTAETNLYTRSLNGIVATTSGVAASA

HRKVSVVPYGMSQMY*

>Bis-1MPK20-2 Brdisv1Bis-11015089m.p

MQQPQQQGQRNKSTDVDFFSEYGDANRYRIQEVIGKGSYGVVCSAMDMQTRQKVAIKKIH

NIFDHVSDAARILREIKLLRLLRHPDVVEIKHIMLPPSRKDFKDIYVVFELMESDLHQVI

KANDDLTKEHYQFFLYQLLRALKYIHTASVYHRDLKPKNILANSNCKLKICDFGLARVAF

NDTPTTVFWTDYVATRWYRAPELCGSFFTKYTPAIDIWSIGCIFAEVLTGKPLFPGKNVV

HQLDLMTDLLGTPSMDTISRVRNEKARRYLSSMRKKDPVPFSKKFPNADPLGLKLLEKLL

AFDPKDRPTAEEALTDPYFKSLSKPDREPSCQPIRKVEFDFEHRRMSKDDIRELIFQEIL

EYHPQLLKNYIDGTEKTTFLYPSAVDQFKKQFSHLEESDGSGPVVPTERKHASLPRSTTV

HSTPIPAKEQPLVASSRGRPIANEPCKPWAPGNVPGASQTAHVAQAGRAVGVGSVPPYES

GSGKYPYDATSRPAVSSGYPPQQKIPQTYGYHHHHQTPGAGQSSQAMGGYACGYTKGTPP

PPAAAQDMRASPYHHRSAGTKNDPLNRLAAESDIYTRSLNGIVAAAASAGTGAHRKVGAV

PFGMSGMY*

>Bis-1MPK20-3 Brdisv1Bis-11043552m.p

MQHGDLHKKSAAEMDFFTAYDDANRYKILEVIGKGSYGLVCSANDLQTGEKVAIKKIHNI

FEHISDAARILREIKLLRLLRHPDVVEIKHILLPPSKKDFKDIYVVFELMESDLHQVIKA

NDDLTREHYQFFLYQMLRALKYMHTANVYHRDLKPKNVLANANCKLKICDFGLARVAFSD

APTTVFWTDYVATRWYRAPELCGSFYSK

>Bis-1MPK20-4 Brdisv1Bis-11019216m.p

MQTTEQQRKKGSSEMDFFSEYGDANRYKIQEVIGKGSYGVVCSAIDQHTGDKVAIKKIHN

IFEHLSDAARILREIKLLRLLRHPDIVEIRHIMLPPSRRDFKDIYVVFELMDTDLHQVIK

ANDDLTKEHHQFFLYQMLRALKYIHTANVYHRDLKPKNILANANCKLKICDFGLARVAFN

DTPTTVFWTDYVATRWYRAPELCGSFFTKYSRAIDIWSIGCIFAEILTGKPLFPGKNVVH

QLDLMTDLLGTPSLDTVSRIRNEKARRYLSSMRKKQSVSFSERFPKADPAALKLLQRLLA

FDPKDRPTAEEALADPYFKGLGKVEREPSCQPITKMEFEFERKNVTKADVKELIFREILE

YHPQLLKDYMNGTEKTNFLYPSAVDNFRRQFANLEENGGKGGAVVPPDRKHVSLPRNTTV

HSTPIPPKDQKYSQVPQRIPTGRPGRVVGPVIPFENSSTMDPYSQRRVARNPVLPAAATN

LSAYAYRNSDNSERELQQELEKDRMQYHPMQRFMDAKMVSPDLRSTSYYMPKGVPKADVA

ERSALQSNMMQGIAPFNGIATVGGAFNKVSAVQYGVSRMY*

>Bis-1MPK20-5 Brdisv1Bis-11015242m.p

MFSDIFSREGVAFSRVALRQELLSDQWFTTAGGKGQCYLVPPGTTVFLVNSEEMGFFSEY

VDASRYKILEIIGKGSYGVVCSAIDQETGDKVAIKKIQNIFEHLSDAARILREIKLLRLL

RHPDIVQIKHIMLPPSRRDFRDIYVVFELMDTDLHQVIKANDDLTKEHYQFFLYQMLRAL

KYIHTANVYHRDLKPKNILANANCKLKICDFGLARVAFNDTPTTVFWTDYVATRWYRAPE

LCGSFFTKYSPAIDTWSIGCIFAEILTGKPLFPGKNVVHQLDLMTDLLGTPSTETISRIR

NDKARKYLSSMRRKQPIPFSEKFPNADPSALKLLERLLAFDPKDRPTAEEALAHPYFKRL

ARVEREPSCQQPISKTEFEFERRKFTKEDVKELIFREILEYHPKLLKDYMNGSEKTSFLY

PSAVDNFRRQFANLEIDGGRSGAADRKHFSLPRTTTVHSAPILPTNGPTSQVPQRIPTAR

PGRVVSSAMQTDNPSVSDRHNGRRVARDPAVPPAAAYHLKSDYSDRQHQQEFEKDRVRPD

RQRQEELEKDRMQYRPGHHSMDAKVAPEISPYMRSSPYYIPPFNGIAAVASGYSKVAAVT

RMY*

>Bis-1MPK21-1 Brdisv1Bis-11015134m.p

MGGLIRWLRHHRSRRVSSSSSSHLPSNTTSSSSTSDLRAHSLPQHQGDHHGEVVVEWEDA

AEGPDSDPEEYIVVVLGDDEQGVVAARAPVRTKPPRVMDPGKKTSESEFFTEYGEANRYK

VSEVIGKGSYGVVAAAVDTQTGERCAIKKINDVFDHVSDATRILREIKLLRLLRHPDIVE

IKHIMLPPSRREFRDIYVVFELMESDLHQVIKANDDLTPEHHQFFLYQLLRGMKYIHTAN

VFHRDLKPKNILANADCKLKICDFGLARVSFNDGAPSAIFWTDYVATRWYRAPELCGSFF

SKYTPAIDIWSVGCIFAEMLTGKPLFPGKNVVHQLDLMTDLLGTPSAESLSKIRNEKARR

YLSNMRKKPKVPLTKKFPGIDPMALHLLERLLAFDPKDRPSADEALTDPYFTGLANSERE

PITQPISKLEFEFEKRKLAKDDVRELIYREILEYHPHMLQEYLRGGGDQMSFMFPSGVDR

FKRQFAHLEEGGAKGEKSSPQLRQNASLPRERVIGNKHGDGDYNMKLNVGEKPELASVSD

GISKPLMSARSLLKSETMSASKCIGEIKNKDEDSLSECVEGTDDDVSQKIAQLKT*

>Bis-1MPK21-2 Brdisv1Bis-11019081m.p

MARKGSDEAEFFTEYGEANRYEVGEVVGKGSYGVVAAAVDTHTGERVAIKKINDVFEHVS

DATRILREIKLLRLLRHPDIVEIKHIILPPSRREFRDIYIIFELMESDLHQVIKANDDLT

PEHHQFFFYQLLRGMKYIHAANVFHRDLKPRNILANADCKLKICDFGLARVSFNDTPSAI

FWTDYVATRWYRAPELCGSFFSKYTPAIDIWSIGCIFAEMLSGRPLFPGKNVVHQLDLMT

DLLGTPSAESLSRIRNEKARRYLGNMRKKHPVPFSQKFPGVDPMALDLLERLLAFDPKDR

PTAAEALADPYFTGLANSDREPTTQPISKLEFEFERRKLARDDVRELIYREILEYHPQML

HEYHHGGDQANFVYPSGVDRFKRQFVHLEEGVTKGEKTSPQLRQHASLPRERIIGIGDEL

GRPNADYCIKLHVGEEPGHTSVTDGLRKPLLNARNFLKSESISASQCVVIKEKREKDEES

MSEYMHEASDATPK*

>Koz-3MPK3 Brdisv1Koz-31009389m.p

MDGAPVAEFRPTMTHGGRVLLYNIFGNQFEITAKYQPPIMPIGRGAYGIVCSVMNFETRE

MVAIKKIANAFDNNMDAKRTLREIKLLRHLDHENIVGLRDVIPPAIPQSFNDVYIATELM

DTDLHHIIRSNQELSEEHCQYFLYQLLRGLKYIHSANVIHRDLKPSNLLLNANCDLKICD

FGLARPSSESDMMTEYVVTRWYRAPELLLNSTDYSAAIDVWSVGCIFMELINRAPLFPGR

DHMHQMRLITEVIGTPTDDDLGFIRNEDARRYMRHLPQFPRRPFPAQFPRVQPAALDLIE

RMLAFNPLQRITVEEALEHPYLERLHDIADEPICTDPFSFDFEQHPLTEDQMKQLIFNEA

LELNPNFRY*

>Koz-3MPK4 Brdisv1Koz-31024105m.p

MDTSGGGGGGAGGAAQIQGMATHGGRYVLYNVYGNLFEVASKYAPPIRPIGRGAYGIVCA

AVSSDTGEEVAIKKIGNAFDNHIDAKRTLREIKLLRHMDHENIIAIKDIIRPPRRDDFKD

VYIVTELMDTDLHQIIRSNQPLTDDHCQYFLYQLLRGLKYVHSANVLHRDLKPSNLFLNA

NCDLKIADFGLARTTSETDLMTEYVVTRWYRAPELLLNCSQYTAAIDVWSVGCILGEIIT

RQPLFPGRDYIQQLKLITELIGSPDDSSLGFLRSDNARRYMKQLPQYPRQDFRLRFRNMS

DGAVDLLERMLVFDPSRRITVDEALHHPYLASLHDINEEPTCPAPFSFDFEQPSFTEEHM

KELIWRETLAFNPDPPY*

>Koz-3MPK6 Brdisv1Koz-31006774m.p

MDGGAQPPDAEMADAGAAGPGAGAGAAGAMENIQATLSHGGRFIQYNIFGNVFEVTVKYK

PPILPIGKGAYGIVCSALNSETGEQVAIKKIANAFDNKIDAKRTLREIKLLRHMDHENIV

AIRDIIPPAQRNSFNDVYIAYELMDTDLHQIIRSNQALSEEHCQYFLYQILRGLKYIHSA

NVLHRDLKPSNLLLNANCDLKICDFGLARTTSETDFMTEYVVTRWYRAPELLLNSSEYTA

AIDVWSVGCIFMELMDRKPLFPGRDHVHQLRLLMELIGTPNEADLDFVNENARRYIRQLP

RHARQSFSEKFPHVHPSAIDLVEKMLTFDPRQRITVEGALAHPYLASLHDISDEPVCTMP

FSFDFEQHALSEEQMKDLIHQEALAFNPDYQ*

>Koz-3MPK11 Brdisv1Koz-31022574m.p

MRMEGGGAGPAAAAAAGGAHGLGEAQIKGTLTHGGRYVQYNVYGNLFEVSAKYVPPIRPV

GRGACGIICAAINVQTREEVAIKKIGNAFDNQIDAKRTLREVKLLRHMNHENVISIKDII

RPPRRENFNDVYIVYELMDTDLPHLLRSNQPLTDDHCQYFLYQVLRGLKYVHSANVLHRD

LRPSNLLLNAKCDLKIGDFGLARTTTETDFMMEYVVTRWYRAPELLLNCSEYTGAIDMWS

VGCILGEIATREPLFPGKDYVHQLRLITELLGSPDDTSLGFLRSDNARRYVRSLPQYPKQ

QFRSRFPNMSSGAMDLLERMLVFDPNKRITVDEALCHPYLASLHEINDEPVCPAPFSFDF

EQPSFTEEDIKELIWRESVKFNPEPIH*

>Koz-3MPK14 Brdisv1Koz-31020739m.p

MAMLVDPPNGMGNQGKHYYSMWQTLFEIDTKYVPIKPIGRGAYGIVCSSINRETNEKVAI

KKIHNVFDNRVDALRTLRELKLLRHLRHENVISLKDIMMPVQRRSFKDVYLVYELMDTDL

HQIIKSPQGLSNDHCQYFLFQLLRGLKYLHSAEILHRDLKPGNLLVNANCDLKICDFGLA

RTNSSKGQFMTEYVVTRWYRAPELLLCCDNYGTSIDVWSVGCIFAELLGRKPIFPGTECL

NQLKLIVNVLGTMSESDLEFIDNPKARRYIKTLPYTPGVPLASMYPHAHPLAIDLLQKML

IFDPTKRISVTEALEHPYMSPLYDPSANPPAQVPIDLDLDENISADMIREMMWQEMLHYH

PETATAISM*

>Koz-3MPK16 Brdisv1Koz-31016040m.p

MDFFTEYGEGNRYKIEEVIGKGSYGVVCSALDTHTGDKVAIKKINDIFEHVSDATRILRE

IKLLRLLRHPDIVEIKHILLPPSRREFKDIYVVFELMESDLHQVIKANDDLTPEHYQFFL

YQLLRGLKYIHTANVFHRDLKPKNILANADCKLKICDFGLARVAISDTPPAIFWTDYIAT

RWYRAPELCGSFFSKYTPAIDIWSIGCIFAELLTGKPLFPGKNVVHQLDIITDLLGTPSA

ETIARIRNEKARRYLSSMRRKKTVPFTQKFPNADPLALNLLERMLAFDPKDRPSAEEALA

DPYFKNIASVDREPSAQPITKLEFEFERRRITKDDIRELIYREILEYHPNMLREFLEGAE

PTGFMYPSAVDHFKKQFTFLEEHYAKGSTAAPPERQHNSLPRPSVIYSDNRPQNAANITD

DLSRCAIRDNTQKAQKDSASVGANRIPHGAAAAAARPGKVVGSVLRYGNCSTSGTEQYEQ

RRVVRSPGIAPNGVTSGSSYPRRNNTCKSETGEAERIDTNQGGPPKPYVANKLPATVDGR

NGHW*

>Koz-3MPK17 Brdisv1Koz-31005240m.p

MVGGGGIVDGFRRLFHRRTPSGSGPSSNQSSAGEESSDIDAVVEDLDLVGLRAIRVPKRK

MPLPVESHKKNILEKEFFTEYGEASQYQIQEVIGKGSYGVVAAAVDTRTGERVAIKKIND

VFEHVSDATRILREVKLLRLLRHPDVVEIKHIMLPPSRREFQDIYVVFELMESDLHQVIR

ANDDLTPEHYQFFLYQLLRALKYIHAANVFHRDLKPKNILANSDCKLKICDFGLARVSFN

DAPTAIFWTDYVATRWYRAPELCGSFFSKYTPAIDIWSIGCIFAELLTGRPLFPGKNVVH

QLDIITDLLGTPSSETLSRIRNEKARRYLSCMRKKHPVPLTQKFPNVDPLALRLLGRLLA

FDPKDRPSAEEALADPYFAVLANVEREPSRNPISKLEFEFERRKVTKDDVRELIYREILE

YHPQMLQEYMQGGEQLSFLYPSGVDRFKRQFAHLEENYSKGERGSPLQRKHASLPRQRVG

ASNDNNEQHASDQERGADSIAPATGNPPGSQDAGQQHVSGGQNGVGSTNISPRSYQKSAS

ISASKCVVVNANKNPEYDDDISEEMEGAVDGLSEKVSRMHP*

>Koz-3MPK20-1 Brdisv1Koz-31017201m.p

MQQDQRKKSSAEAEFFTEYGDASRYKIQEIVGKGSYGVVCSAIDVHTGEKVAIKKIHDIF

EHISDAARILREIKLLRLLRHPDIVEIKHIMLPPSRRDFKDIYVVFELMESDLHQVIKAN

DDLTKEHYQFFLYQLLRALKYIHTANVYHRDLKPKNILANSNCKLKICDFGLARVAFNDT

PTTIFWTDYVATRWYRAPELCGSFFSKYTPAIDVWSIGCIFAEVLTGKPLFPGKNVVHQL

DLMTDLLGTPSMDTISRVRNEKARRYLSSMRKKEPIPFSQKFPNADPLALDLLQRLLAFD

PKDRPTAGEALSHPYFKGLAKVEREPSCQPITKMEFEFERRRATKEDIRELIFREILEYH

PQLLKDYINGTERTTFLYPSAVDQFRKQFAHLEENSESGPVVPMDRKHTSLPRSTIVHSA

PIHAKEQPRIGPSRDRPLTDESYKNPRDSEKYSGNVPRSSHHSQAPQRVPTARPGRVVGP

VLPYENVGTSHPYDPRRVAMNSGYPPQQQIPQTYGYYQTTGKSACSEPSQAERYTLHQQA

YACANSSTAPDVALDMRAPPFHQSAGPKSGSSDRLTAETNLYTRSLNGIVATTSGVAASA

HRKVSVVPYGMSQMY*

>Koz-3MPK20-2 Brdisv1Koz-31013656m.p

MQQPQQQGQRNKSTDVDFFSEYGDANRYRIQEVIGKGSYGVVCSAMDMQTRQKVAIKKIH

NIFDHVSDAARILREIKLLRLLRHPDVVEIKHIMLPPSRKDFKDIYVVFELMESDLHQVI

KANDDLTKEHYQFFLYQLLRALKYIHTASVYHRDLKPKNILANSNCKLKICDFGLARVAF

NDTPTTVFWTDYVATRWYRAPELCGSFFTKYTPAIDIWSIGCIFAEVLTGKPLFPGKNVV

HQLDLMTDLLGTPSMDTISRVRNEKARRYLSSMRKKDPVPFSKKFPNADPLGLKLLEKLL

AFDPKDRPTAEEALTDPYFKSLSKPDREPSCQPIRKVEFDFEHRRMSKDDIRELIFQEIL

EYHPQLLKNYIDGTEKTTFLYPSAVDQFKKQFSHLEESDGSGPVVPTERKHASLPRSTTV

HSTPIPAKEQPLVASSRGRPIANEPCKPWAPGNVPGASQTAHVAQAGRAVGVGSVPPYES

GSGKYPYDATSRPAVSSGYPPQQKIPQTYGYHHHHQTPGAGQSSQAMGGYACGYTKGTTP

PPAAAQDMRASPYHHRSAGTKNDPLNRLAAESDIYTRSLNGIVAAAASAGTGAHRKVGAV

PFGMSGMY*

>Koz-3MPK20-3 Brdisv1Koz-31041190m.p

MQHGDLHKKSAAEMDFFTAYDDANRYKILEVIGKGSYGLVCSANDLQTGEKVAIKKIHNI

FEHISDAARILREIKLLRLLRHPDVVEIKHILLPPSKKDFKDIYVVFELMESDLHQVIKA

NDDLTREHYQFFLYQMLRALKYMHTANVYHRDLKPKNVLANANCKLKICDFGLARVAFSD

APTTVFWTDYVATRWYRAPELCGSFYSK

>Koz-3MPK20-4 Brdisv1Koz-31017399m.p

MQTTEQQRKKGSSEMDFFSEYGDANRYKIQEVIGKGSYGVVCSAIDQHTGDKVAIKKIHN

IFEHLSDAARILREIKLLRLLRHPDIVEIRHIMLPPSRRDFKDIYVVFELMDTDLHQVIK

ANDDLTKEHHQFFLYQMLRALKYIHTANVYHRDLKPKNILANANCKLKICDFGLARVAFN

DTPTTVFWTDYVATRWYRAPELCGSFFTKYSRAIDIWSIGCIFAEILTGKPLFPGKNVVH

QLDLMTDLLGTPSLDTVSRIRNEKARRYLSSMRKKQSVSFSERFPKADPAALKLLQRLLA

FDPKDRPTAEEALADPYFKGLGKVEREPSCQPITKMEFEFERKNVTKADVKELIFREILE

YHPQLLKDYMNGTEKTNFLYPSAVDNFRRQFANLEENGGKGGAVVPPDRKHVSLPRNTTV

HSTPIPPKDQKYSQVPQRIPTGRPGRVVGPVIPFENSSTMDPYSQRRVARNPVLPAAATN

LSAYAYRNSDNSERELQQELEKDRMQYHPMQRFMDAKMVSPDLRSTSYYMPKGVPKADVA

ERSALQSNMMQGIAPFNGIATVGGAFNKVSAVQYGVSRMY*

>Koz-3MPK20-5 Brdisv1Koz-31013797m.p

MFSDIFSREGVAFSRVALRQELLSDQWFTTAGGKGQCYLVPPGTTVFLVNSEEMGFFSEY

VDASRYKILEIIGKGSYGVVCSAIDQETGDKVAIKKIQNIFEHLSDAARILREIKLLRLL

RHPDIVQIKHIMLPPSRRDFRDIYVVFELMDTDLHQVIKANDDLTKEHYQFFLYQMLRAL

KYIHTANVYHRDLKPKNILANANCKLKICDFGLARVAFNDTPTTVFWTDYVATRWYRAPE

LCGSFFTKYSPAIDTWSIGCIFAEILTGKPLFPGKNVVHQLDLMTDLLGIPSTETISRIR

NDKARKYLSSMRRKQPIPFSEKFPNADPSALKLLERLLAFDPKDRPTAEEALAHPYFKRL

ARVEREPSCQQPISKTEFEFERRKFTKEDIKELIFREILEYHPKLLKDYMNGSEKTSFLY

PSAVDNFRRQFANLEIDGGRSGAADRKHFSLPRTTTVHSAPILPTNGPTSQVPQRIPTAR

PGRVVSSAMQTDNPSVSDRHNGRRVARDPAVPPAAAYHLKSDYSDRQHQQEFEKDRVRPD

RQRQEELEKDRMQYRPGHHSMDAKVAPEISPYMRSSPYYIPPFNGIAAVASGYSKVAAVT

RMY*

>Koz-3MPK21-1 Brdisv1Koz-31013700m.p

MGGLIRWLRPHRSRRVSSSSSSHLPSNTTSSSSTSDLRAHSLPQHQGDHHGEVVVEWEDA

AEGPDSDPEEYIVVVLGDDEQGVVAARAPVRTKPPRVMDPGKKTSESEFFTEYGEANRYK

VSEVIGKGSYGVVAAAVDTQTGERCAIKKINDVFDHVSDATRILREIKLLRLLRHPDIVE

IKHIMLPPSRREFRDIYVVFELMESDLHQVIKANDDLTPEHHQFFLYQLLRGMKYIHTAN

VFHRDLKPKNILANADCKLKICDFGLARVSFNDGAPSAIFWTDYVATRWYRAPELCGSFF

SKYTPAIDIWSVGCIFAEMLTGKPLFPGKNVVHQLDLMTDLLGTPSAESLSKIRNEKARR

YLSNMRKKPKVPLTKKFPGIDPMALHLLERLLAFDPKDRPSADEALTDPYFTGLANSERE

PITQPISKLEFEFEKRKLAKDDVRELIYREILEYHPHMLQEYLRGGGDQMSFMFPSGVDR

FKRQFAHLEEGGAKGEKSSPQLRQNASLPRERVIGNKHGDGDYNMKLNVGEKPELASVSD

GISKPLMSARSLLKSETMSASKCIGEIKNKDEDSLSECVEGTDDDVSQKIAQLKT*

>Koz-3MPK21-2 Brdisv1Koz-31017266m.p

MARKGSDEAEFFTEYGEANRYEVGEVVGKGSYGVVAAAVDTHTGERVAIKKINDVFEHVS

DATRILREIKLLRLLRHPDIVEIKHIILPPSRREFRDIYIIFELMESDLHQVIKANDDLT

PEHHQFFFYQLLRGMKYIHAANVFHRDLKPRNILANADCKLKICDFGLARVSFNDTPSAI

FWTDYVATRWYRAPELCGSFFSKYTPAIDIWSIGCIFAEMLSGRPLFPGKNVVHQLDLMT

DLLGTPSAESLSRIRNEKARRYLGNMRKKHPVPFSQKFPGVDPMALDLLERLLAFDPKDR

PTAAEALADPYFTGLANSDREPTTQPISKLEFEFERRKLARDDVRELIYREILEYHPQML

HEYHHGGDQANFVYPSGVDRFKRQFVHLEEGVTKGEKTSPQLRQHASLPRERIIGIGDEL

GRPNADYCIKLHVGEEPGHTSVTDGLRKPLLNARNFLKSESISASQCVVIKEKREKDEES

MSEYMHEASDATPK*

>Koz-1MPK3 Brdisv1Koz-11010948m.p

MDGAPVAEFRPTMTHGGRFLLYNIFGNQFEITAKYQPPIMPIGRGAYGIVCSVMNFETRE

MVAIKKIANAFDNNMDAKRTLREIKLLRHLDHENIVGLRDVIPPAIPQSFNDVYIATELM

DTDLHHIIRSNQELSEEHCQYFLYQLLRGLKYIHSANVIHRDLKPSNLLLNANCDLKICD

FGLARPSSESDMMTEYVVTRWYRAPELLLNSTDYSAAIDVWSVGCIFMELINRAPLFPGR

DHMHQMRLITEVIGTPTDDDLGFIRNEDARRYMRHLPQFPRRPFPAQFPRVQPAALDLIE

RMLAFNPLQRITVEEALEHPYLERLHDIADEPICTDPFSFDFEQHPLTEDQMKQLIFNEA

LELNPNFRY*

>Koz-1MPK4 Brdisv1Koz-11035762m.p

MDTSGGGGGGAGGAAQIQGMATHGGRYVLYNVYGNLFEVASKYAPPIRPIGRGAYGIVCA

AVSSDTGEEVAIKKIGNAFDNHIDAKRTLREIKLLRHMDHENIIAIKDIIRPPRRDDFKD

VYIVTELMDTDLHQIIRSNQPLTDDHCQYFLYQLLRGLKYVHSANVLHRDLKPSNLFLNA

NCDLKIADFGLARTTSETDLMTEYVVTRWYRAPELLLNCSQYTAAIDVWSVGCILGEIIT

RQPLFPGRDYIQQLKLITELIGSPDDSSLGFLRSDNARRYMKQLPQYPRQDFRLRFRNMS

DGAVDLLERMLVFDPSRRITVDEALHHPYLASLHDINEEPTCPAPFSFDFEQPSFTEEHM

KELIWRETLAFNPDPPY*

>Koz-1MPK6 Brdisv1Koz-11007897m.p

MDGGAQPPDAEMADAGAAVPGAGAGAAGTMENIQATLSHGGRFIQYNIFGNVFEVTVKYK

PPILPIGKGAYGIVCSALNSETGEQVAIKKIANAFDNKIDAKRTLREIKLLRHMDHENIV

AIRDIIPPAQRNSFNDVYIAYELMDTDLHQIIRSNQALSEEHCQYFLYQILRGLKYIHSA

NVLHRDLKPSNLLLNANCDLKICDFGLARTTSETDFMTEYVVTRWYRAPELLLNSSEYTA

AIDVWSVGCIFMELMDRKPLFPGRDHVHQLRLLMELIGTPNEADLDFVNENARRYIRQLP

RHARQSFSEKFPHVHPSAIDLVEKMLTFDPRQRITVEGALAHPYLASLHDISDEPVCTMP

FSFDFEQHALSEEQMKDLIHQEALAFNPDYQ*

>Koz-1MPK7-1 Brdisv1Koz-11046637m.p

MVVHAIGVPRRQVHYRCAPAALPQRGGSGARVAAEALWWCIPSAFGADGLLRGLKYLHSA

GILHRDLKPGNLLVNANCDLKICDFGLARTNNTKGQFMTEYVVTCLYRAPELLLCCDNYG

TSIDVWSVGCIFAELLGRKPIFPRTQCLDQLKLIVNVLGTMSENDLEFIDNPKARKYIKS

LPYTPGTPLTSMYPQAHPLAIDLLQKMLVFDPSKRISVTEALEHPYMSPLYDPSANPPAQ

VPIDLDIDENLGVEMIREMLWQEMLQYHPEAARMVNM*

>Koz-1MPK11 Brdisv1Koz-11033685m.p

MRMEGGGAGPAAAAAAGGAHGLGEAQIKGTLTHGGRYVQYNVYGNLFEVSAKYVPPIRPV

GRGACGIICAAINVQTREEVAIKKIGNAFDNQIDAKRTLREVKLLRHMNHENVISIKDII

RPPRRENFNDVYIVYELMDTDLHHLLRSNQPLTDDHCQYFLYQVLRGLKYVHSANVLHRD

LRPSNLLLNAKCDLKIGDFGLARTTTETDFMMEYVVTRWYRAPELLLNCSEYTGAIDMWS

VGCILGEIATREPLFPGKDYVHQLRLITELLGSPDDTSLGFLRSDNARRYVRSLPQYPKQ

QFRSRFPNMSSGAMDLLERMLVFDPNKRITVDEALCHPYLASLHEINDEPVCPAPFSFDF

EQPSFTEEDIKELIWRESVKFNPEPIH*

>Koz-1MPK14 Brdisv1Koz-11031538m.p

MAMLVDPPNGMGNQGKHYYSMWQTLFEIDTKYVPIKPIGRGAYGIVCSSINRETNEKVAI

KKIHNVFDNRVDALRTLRELKLLRHLRHENVISLKDIMMPVQRRSFKDVYLVYELMDTDL

HQIIKSPQGLSNDHCQYFLFQLLRGLKYLHSAEILHRDLKPGNLLVNANCDLKICDFGLA

RTNSSKGQFMTEYVVTRWYRAPELLLCCDNYGTSIDVWSVGCIFAELLGRKPIFPGTECL

NQLKLIVNVLGTMSESDLEFIDNPKARRYIKTLPYTPGVPLASMYPHAHPLAIDLLQKML

IFDPTKRISVTEALEHPYMSPLYDPSANPPAQVPIDLDLDENISADMIREMMWQEMLHYH

PEAATAISM*

>Koz-1MPK16 Brdisv1Koz-11018826m.p

MDFFTEYGEGNRYKIEEVIGKGSYGVVCSALDTHTGDKVAIKKINDIFEHVSDATRILRE

IKLLRLLRHPDIVEIKHILLPPSRREFKDIYVVFELMESDLHQVIKANDDLTPEHYQFFL

YQLLRGLKYIHTANVFHRDLKPKNILANADCKLKICDFGLARVAISDTPTAIFWTDYIAT

RWYRAPELCGSFFSKYTPAIDIWSIGCIFAELLTGKPLFPGKNVVHQLDIITDLLGTPSA

ETIARIRNEKARRYLSSMRRKKTVPFTQKFPNADPLALNLLERMLAFDPKDRPSAEEALA

DPYFKNIASVDREPSAQPITKLEFEFERRRITKDDIRELIYREILEYHPNMLREFLEGAE

PTGFMYPSAVDHFKKQFTFLEEHYAKGSTAAPPERQHNSLPRPSVIYSDNRPQNAANITD

DLSRCAIRDNTQKAQKDSASVGANRIPHGAAAAAARPGKVVGSVLRYGNCSTSGTEQYEQ

RRVVRSPGIAPNGVTSGSSYPRRNNTCKSETGEAERIDTNQGGPPKPYVANKLPATVDGR

NGHW*

>Koz-1MPK17 Brdisv1Koz-11005826m.p

MVGGGGIVDGFRRLFHRRTPSGSGPSSNQSSAGEESSDIDAVVEDLDLVGLRAIRVPKRK

MPLPVESHKKNILEKEFFTEYGEASQYQIQEVIGKGSYGVVAAAVDTRTGERVAIKKIND

VFEHVSDATRILREVKLLRLLRHPDVVEIKHIMLPPSRREFQDIYVVFELMESDLHQVIR

ANDDLTPEHYQFFLYQLLRALKYIHAANVFHRDLKPKNILANSDCKLKICDFGLARVSFN

DAPTAIFWTDYVATRWYRAPELCGSFFSKYTPAIDIWSIGCIFAELLTGRPLFPGKNVVH

QLDIITDLLGTPSSETLSRIRNEKARRYLSCMRKKHPVPLTQKFPNVDPLALRLLGRLLA

FDPKDRPSAEEALADPYFAVLANVEREPSRNPISKLEFEFERRKVTKDDVRELIYREILE

YHPQMLQEYMQGGEQLSFLYPSGVDRFKRQFAHLEENYSKGERGSPLQRKHASLPRQRVG

ASNDNNEQHASDQERGADSIAPATGNPPGSQDAGQQHVSGGQNGVGSTNISPRSYQKSAS

ISASKCVVVNANKNPEYDDDISEEMEGAVDGLSEKVSRMHP*

>Koz-1MPK20-1 Brdisv1Koz-11020254m.p

MQQDQRKKSSAEAEFFTEYGDASRYKIQEIVGKGSYGVVCSAIDVHTGEKVAIKKIHDIF

EHISDAARILREIKLLRLLRHPDIVEIKHIMLPPSRRDFKDIYVVFELMESDLHQVIKAN

DDLTKEHYQFFLYQLLRALKYIHTANVYHRDLKPKNILANSNCKLKICDFGLARVAFNDT

PTTIFWTDYVATRWYRAPELCGSFFSKYTPAIDVWSIGCIFAEVLTGKPLFPGKNVVHQL

DLMTDLLGTPSMDTISRVRNEKARRYLSSMRKKEPIPFSQKFPNADPLALDLLQRLLAFD

PKDRPTAGEALSHPYFKGLAKVEREPSCQPITKMEFEFERRRATKEDIRELIFREILEYH

PQLLKDYINGTERTTFLYPSAVDQFRKQFAHLEENSESGPVVPMDRKHTSLPRSTIVHSA

PIHAKEQPRIGPSRDRPLTDESYKNPRDSEKYSGNVPRSSHHSQAPQRVPTARPGRVVGP

VLPYENVGTSHPYDPRRVAMNSGYPPQQQIPQTYGYYQTTGKSACSEPSQAERYTLHQQA

YACANSSTAPDVALDMRAPPFHQSAGPKSGSSDRLTAETNLYTRSLNGIVATTSGVAASA

HRKVSVVPYGMSQMY*

>Koz-1MPK20-2 Brdisv1Koz-11015944m.p

MQQPQQQGQRNKLPDGWFCCNVHILNLCRSAMIFLTPPIIMPSTDVDFFSEYGDANRYRI

QEVIGKGSYGVVCSAMDMQTRQKVAIKKIHNIFDHVSDAARILREIKLLRLLRHPDVVEI

KHIMLPPSRKDFKDIYVVFELMESDLHQVIKANDDLTKEHYQFFLYQLLRALKYIHTASV

YHRDLKPKNILANSNCKLKICDFGLARVAFNDTPTTVFWTDYVATRWYRAPELCGSFFTK

YTPAIDIWSIGCIFAEVLTGKPLFPGKNVVHQLDLMTDLLGTPSMDTISRVRNEKARRYL

SSMRKKDPVPFSKKFPNADPLGLKLLEKLLAFDPKDRPTAEEALTDPYFKSLSKPDREPS

CQPIRKVEFDFEHRRMSKDDIRELIFQEILEYHPQLLKNYIDGTEKTTFLYPSAVDQFKK

QFSHLEESDGSGPVVPTERKHASLPRSTTVHSTPIPAKEQPLVASSRGRPIANEPCKPWA

PGNVPGASQTAHVAQAGRAVGVGSVPPYESGSGKYPYDATSRPAVSSGYPPQQKIPQTYG

YHHHHQTPGAGQSSQAMGGYACGYTKGTTPPPAAAQDMRASPYHHRSAGTKNDPLNRLAA

ESDIYTRSLNGIVAAAASAGTGAHRKVGAVPFGMSGMY*

>Koz-1MPK20-3 Brdisv1Koz-11045898m.p

MQHGDLHKKSAAEMDFFTAYDDANRYKILEVIGKGSYGLVCSANDLQTGEKVAIKKIHNI

FEHISDAARILREIKLLRLLRHPDVVEIKHILLPPSKKDFKDIYVVFELMESDLHQVIKA

NDDLTREHYQFFLYQMLRALKYMHTANVYHRDLKPKNVLANANCKLKICDFGLARVAFSD

APTTVFWTDYVATRWYRAPELCGSFYSK

>Koz-1MPK20-4 Brdisv1Koz-11025196m.p

MQTTEQQRKKGSSEMDFFSEYGDANRYKIQEVIGKGSYGVVCSAIDQHTGDKVAIKKIHN

IFEHLSDAARILREIKLLRLLRHPDIVEIRHIMLPPSRRDFKDIYVVFELMDTDLHQVIK

ANDDLTKEHHQFFLYQMLRALKYIHTANVYHRDLKPKNILANANCKLKICDFGLARVAFN

DTPTTVFWTDYVATRWYRAPELCGSFFTKYSRAIDIWSIGCIFAEILTGKPLFPGKNVVH

QLDLMTDLLGTPSLDTVSRIRNEKARRYLSSMRKKQSVSFSERFPKADPAALKLLQRLLA

FDPKDRPTAEEALADPYFKGLGKVEREPSCQPITKMEFEFERKNVTKADVKELIFREILE

YHPQLLKDYMNGTEKTNFLYPSAVDNFRRQFANLEENGGKGGAVVPPDRKHVSLPRNTTV

HSTPIPPKDQKYSQVPQRIPTGRPGRVVGPVIPFENSSTMDPYSQRRVARNPVLPAAATN

LSAYAYRNSDNSERELQQELEKDRMQYHPMQRFMDAKMVSPDLRSTSYYMPKGVPKADVA

ERSALQSNMMQGIAPFNGIATVGGAFNKVSAVQYGVSRMY*

>Koz-1MPK20-5 Brdisv1Koz-11016096m.p

MFSDIFSREGVAFSRVALRQELLSDQWFTTAGGKGQCYLVPPGTTVFLVNSEEMGFFSEY

VDASRYKILEIIGKGSYGVVCSAIDQETGDKVAIKKIQNIFEHLSDAARILREIKLLRLL

RHPDIVQIKHIMLPPSRRDFRDIYVVFELMDTDLHQVIKANDDLTKEHYQFFLYQMLRAL

KYIHTANVYHRDLKPKNILANANCKLKICDFGLARVAFNDTPTTVFWTDYVATRWYRAPE

LCGSFFTKYSPAIDTWSIGCIFAEILTGKPLFPGKNVVHQLDLMTDLLGIPSTETISRIR

NDKARKYLSSMRRKQPIPFSEKFPNADPSALKLLERLLAFDPKDRPTAEEALAHPYFKRL

ARVEREPSCQQPISKTEFEFERRKFTKEDIKELIFREILEYHPKLLKDYMNGSEKTSFLY

PSAVDNFRRQFANLEIDGGRSGAADRKHFSLPRTTTVHSAPILPTNGPTSQVPQRIPTAR

PGRVVSSAMQTDNPSVSDRHNGRRVARDPAVPPAAAYHLKSDYSDRQHQQEFEKDRVRPD

RQRQEELEKDRMQYRPGHHSMDAKVAPEISPYMRSSPYYIPPFNGIAAVASGYSKVAAVT

RMY*

>Koz-1MPK21-1 Brdisv1Koz-11015994m.p

MGGLIRWLRHHRSRRVSSSSSSHLPSNTTSSSSTSDLRAHSLPQHQGDHHGEVVVEWEDA

AEGPDSDPEEYIVVVLGDDEQGVVAARAPVRTKPPRVMDPGKKTSESEFFTEYGEANRYK

VSEVIGKGSYGVVAAAVDTQTGERCAIKKINDVFDHVSDATRILREIKLLRLLRHPDIVE

IKHIMLPPSRREFRDIYVVFELMESDLHQVIKANDDLTPEHHQFFLYQLLRGMKYIHTAS

VFHRDLKPKNILANADCKLKICDFGLARVSFNDGAPSAIFWTDYVATRWYRAPELCGSFF

SKYTPAIDIWSVGCIFAEMLTGKPLFPGKNVVHQLDLMTDLLGTPSAESLSKIRNEKARR

YLSNMRKKPKVPLTKKFPGIDPMALHLLERLLAFDPKDRPSADEALTDPYFTGLANSERE

PITQPISKLEFEFEKRKLAKDDVRELIYREILEYHPHMLQEYLRGGGDQMSFMFPSGVDR

FKRQFAHLEEGGAKGEKSSPQLRQNASLPRERVIGNKHGDGDYNMKLNVGEKPELASVSD

GISKPLMSARSLLKSETMSASKCIGEIKNKDEDSLSECVEGTDDDVSQKIAQLKT*

>Koz-1MPK21-2 Brdisv1Koz-11020344m.p

MARKGSDEAEFFTEYGEANRYEVGEVVGKGSYGVVAAAVDTHTGERVAIKKINDVFEHVS

DATRILREIKLLRLLRHPDIVEIKHIILPPSRREFRDIYIIFELMESDLHQVIKANDDLT

PEHHQFFFYQLLRGMKYIHAANVFHRDLKPRNILANADCKLKICDFGLARVSFNDTPSAI

FWTDYVATRWYRAPELCGSFFSKYTPAIDIWSIGCIFAEMLSGRPLFPGKNVVHQLDLMT

DLLGTPSAESLSRIRNEKARRYLGNMRKKHPVPFSQKFPGVDPMALDLLERLLAFDPKDR

PTAAEALADPYFTGLANSDREPTTQPISKLEFEFERRKLARDDVRELIYREILEYHPQML

HEYHHGGDQANFVYPSGVDRFKRQFVHLEEGVTKGEKTSPQLRQHASLPRERIIGIGDEL

GRPNADYCIKLHVGEEPGHTSVTDGLSKPLLNARNFLKSESISASQCVVIKEKREKDEES

MSEYMHEASDATPK*

>BdTR3cMPK3 Brdisv1BdTR3C1011415m.p

MDGAPVAEFRPTMTHGGRFLLYNIFGNQFEITAKYQPPIMPIGRGAYGIVCSVMNFETRE

MVAIKKIANAFDNNMDAKRTLREIKLLRHLDHENIVGLRDVIPPAIPQSFNDVYIATELM

DTDLHHIIRSNQELSEEHCQYFLYQLLRGLKYIHSANVIHRDLKPSNLLLNANCDLKICD

FGLARPSSESDMMTEYVVTRWYRAPELLLNSTDYSAAIDVWSVGCIFMELINRAPLFPGR

DHMHQMRLITEVIGTPTDDDLGFIRNEDARRYMRHLPQFPRRPFPAQFPRVQPAALDLIE

RMLAFNPLQRITVEEALEHPYLERLHDIADEPICTDPFSFDFEQHPLTEDQMKQLIFNEA

LELNPNFRY*

>BdTR3cMPK4 Brdisv1BdTR3C1004133m.p

MDTSGGGGGGAGGAAQIQGMATHGGRYVLYNVYGNLFEVASKYAPPIRPIGRGAYGIVCA

AVSSDTGEEVAIKKIGNAFDNHIDAKRTLREIKLLRHMDHENIIAIKDIIRPPRRDDFKD

VYIVTELMDTDLHQIIRSNQPLTDDHCQYFLYQLLRGLKYVHSANVLHRDLKPSNLFLNA

NCDLKIADFGLARTTSETDLMTEYVVTRWYRAPELLLNCSQYTAAIDVWSVGCILGEIIT

RQPLFPGRDYIQQLKLITELIGSPDDSSLGFLRSDNARRYMKQLPQYPRQDFRLRFRNMS

DGAVDLLERMLVFDPSRRITVDEALHHPYLASLHDINEEPTCPAPFSFDFEQPSFTEEHM

KELIWRETLAFNPDPPY*

>BdTR3cMPK6 Brdisv1BdTR3C1008268m.p

MDGGAQPPDAEMADAGAAVPGAGAGAAGTMENIQATLSHGGRFIQYNIFGNVFEVTVKYK

PPIPPIGKGAYGIVCSALNSETGEQVAIKKIANAFDNKIDAKRTLREIKLLRHMDHENIV

AIRDIIPPAQRNSFNDVYIAYELMDTDLHQIIRSNQALSEEHCQYFLYQILRGLKYIHSA

NVLHRDLKPSNLLLNANCDLKICDFGLARTTSETDFMTEYVVTRWYRAPELLLNSSEYTA

AIDVWSVGCIFMELMDRKPLFPGRDHVHQLRLLMELIGTPNEADLDFVNENARRYIRQLP

RHARQSFSEKFPHVHPSAIDLVEKMLTFDPRQRITVEGALAHPYLASLHDISDEPVCTMP

FSFDFEQHALSEEQMKDLIHQEALAFNPDYQ*

>BdTR3cMPK7-1 Brdisv1BdTR3C1006021m.p

MPSRFPFLSPGCFIRLLSGSASSDLLLLLPRRGQSAPRARVLLKMAMMVDPPNGTGNHGK

HYYTMWQTMFEIDTKYVPIKPIGRGAYGIVCSSTNQENNEKVAIKKINNVFDNRVDALRT

LRELKLLRHLRHENVIALKDIMMPVHRRSFKDVYLVFELMDTDLHQIIKSSQPLSNDHCQ

YFLFQLLRGLKYLHSAGILHRDLKPGNLLVNANCDLKICDFGLARTNNTKGQFMTEYVVT

CLYRAPELLLCCDNYGTSIDVWSVGCIFAELLGRKPIFPGTECLNQLKLIVNVLGTMSEN

DLEFIDNPKARKYIKSLPYTPGTPLTSMYPQAHPLAIDLLQKMLVFDPSKRISVTEALEH

PYMSPLYDPSANPPAQVPIDLDIDENLGVEMIREMLWQEMLQYHPEAARMVNM*

>BdTR3cMPK7-2 Brdisv1BdTR3C1006046m.p

MKLSPISASFGAMGSLPQKLDELLSTGHWALSGVVMDEIEELTADLHILCNLLLKLSNVH

DPPPTVRFWMKEVRELSYDVEDCADQFVIANDRAVMRRAKIHRATIRTIIITRLKISRLP

KRRKWRLWMTDKVAEFRTRVQDATQRYWRYKFDDCASNPGYASVGHEFATVYAQPGDLVG

IEGPMDELEQWLTDGEEQLKVISIVGLGGVGKTTLAQKLWATLKGQFLCRAFVRTAQKPD

MRGILRNILLQVRPHQLPNRGEMHHLIHDLREYLQDKRYFVIIDDLWAASVWDVVSRAFP

EGNCCSRIVTTTEIMEVALACCDYCPKHICKLETLSDDDSEKLLLERIVVSGNQIPQQLD

DVLPQIMRNCGGLPLAIIIVASLLVSQPEKLEQWGHTQNSFGSIFGTNPTMEGFIRQILS

ISFNSLPYYLKTCLLYLSSYPEGCLFLKDDLVKLWVAEGFIHAKEWEDMEDLAGRYFDEL

VNVGLIQVMDINYNYKLPSYSVHHMVLDFITCKSIEENFITVVDYSETTTPLTDKVRRLS

LHFGSATHATTPASARLSNVRSLFYFGLFNCMPSFMVFKLLRVLILNFWGDPGNTSFNLT

GICELVWLRYLQVSCNVTVKLPDQIESMGHLETLEIKAKVYAVPLDIVRLSSLSHLHLRG

GTIPNGIGCMRSLRTLKYFDLVNKSVDNLRGLGELANLRDLHLTYSSSLPSEHLKRNLIA

LASSLGKLCNLKSLTLASETAATVVPFDGSISMSSTPAFLERLELLPSICIFSRLPKWIF

QFQKLCILKVAVRELLANDIDSITGLPSLTVLSLCVHTAPGQRIVFNDGAFPVLKYFKFR

CGVLSMSFMAGAMPNLRRLKLGFNTHVGEKYGNMLAGIEHLLNLQDIAGKIGVATESDRK

AAESAIKGAISKHPQSPMLNVQWVDLVEEEYHASEKQHGRQEKGLSGENHAVLEKAKDTK

KHADSGVSQLPDLPSTVSSSRLKSENIAQASKHHVLESMLCDESAQPESLEVSVLEDITN

NFSDDQEISKNRFVVVYKGELQNGSVVVVKRFTIAIDYNLFLDAVDCLMSVKHNNIVRFL

GYCANSQENAAKEGSKSVSVVTQERLCCFEYLRNGNLQMHLTDESCGFDWQMWYQIIEGI

CQGVHYLHNKSITPLDLNPANIMFDDKMVPKIVHYAYTRFLGEATSPVLTNIGDSLAYMA

PEYFGFGEITSKSDIYSLGVIIMQIVTGENKKDFPSIQSVLESWRSRLESDGSQGHTSLE

TCYLQVNLCYLIGIRCIEHDPGKRPNTRDIIDSLDTLEIMECLVKHDVGNSSLEQMLVCS

KVEEPKALPAPPSVSCKLKMAMMVDPPNGTGNHGKHYYTMWQTMFEIDTKYMPIKPIGRG

AYGIVCSSTNQENNEKVAIKKINNVFDNRVDALRTLRELKLLRHLRHENVIALKDIMMPV

HRRSFKDVYLVSELMDTDLHQIIKSSQPLSNDHCQYFLFQLLRGLKYLHSAGILHRDLKP

GNLLVNANCDLKICDFGLARTNNTKGQFMTEYVVTRWYRAPELLLCCDNYGTSIDVWSVG

CIFAELLGRKPIFPRTQCLDQLKLIVNVLGTMSENDLEFIDNPKARKYIKSLPYTPGTPL

TSMYPQAHPLAIDLLQKMLVFDPSKRISVTEALEHPYMSPLYDPSANPPAQVPIDLDIDE

NLGVEMIREMLWQEMLQYHPEAARMVNM*

>BdTR3cMPK11 Brdisv1BdTR3C1026508m.p

MRMEGGGAGPAAAAAAGGAHGLGEAQIKGTLTHGGRYVQYNVYGNLFEVSAKYVPPIRPV

GRGACGIICAAINVQTREEVAIKKIGNAFDNQIDAKRTLREVKLLRHMNHENVISIKDII

RPPRRENFNDVYIVYELMDTDLHHLLRSNQPLTDDHCQYFLYQVLRGLKYVHSANVLHRD

LRPSNLLLNAKCDLKIGDFGLARTTTETDFMMEYVVTRWYRAPELLLNCSEYTGAIDMWS

VGCILGEIATREPLFPGKDYVHQLRLITELLGSPDDTSLGFLRSDNARRYVRSLPQYPKQ

QFRSRFPNMSSGAMDLLERMLVFDPNKRITVDEALCHPYLASLHEINDEPVCPAPFSFDF

EQPSFTEEDIKELIWRESVKFNPEPIH*

>BdTR3cMPK14 Brdisv1BdTR3C1024843m.p

MAMLVDPPNGMGNQGKHYYSMWQTLFEIDTKYVPIKPIGRGAYGIVCSSINRETNEKVAI

KKIHNVFDNRVDALRTLRELKLLRHLRHENVISLKDIMMPVQRRSFKDVYLVYELMDTDL

HQIIKSPQGLSNDHCQYFLFQLLRGLKYLHSAEILHRDLKPGNLLVNANCDLKICDFGLA

RTNSSKGQFMTEYVVTRWYRAPELLLCCDNYGTSIDVWSVGCIFAELLGRKPIFPGTECL

NQLKLIVNVLGTMSESDLEFIDNPKARRYIKTLPYTPGVPLASMYPHAHPLAIDLLQKML

IFDPTKRISVTEALEHPYMSPLYDPSANPPAQVPIDLDLDENISADMIREMMWQEMLHYH

PEAATAISM*

>BdTR3cMPK16 Brdisv1BdTR3C1019706m.p

MDFFTEYGEGNRYKIEEVIGKGSYGVVCSALDTHTGDKVAIKKINDIFEHVSDATRILRE

IKLLRLLRHPDIVEIKHILLPPSRREFKDIYVVFELMESDLHQVIKANDDLTPEHYQFFL

YQLLRGLKYIHTANVFHRDLKPKNILANADCKLKICDFGLARVAISDTPTAIFWTDYIAT

RWYRAPELCGSFFSKYTPAIDIWSIGCIFAELLTGKPLFPGKNVVHQLDIITDLLGTPSA

ETIARIRNEKARRYLSSMRRKKTVPFTQKFPNADPLALNLLERMLAFDPKDRPSAEEALA

DPYFKNIASVDREPSAQPITKLEFEFERRRITKDDIRELIYREILEYHPNMLREFLEGAE

PTGFMYPSAVDHFKKQFTFLEEHYAKGSTAAPPERQHNSLPRPSVIYSDNRPQNAANITD

DLSRCAIRDNTQKAQKDSASVGANRIPHGAAAAAARPGKVVGSVLRYGNCSTSGTEQYEQ

RRVVRSPGIAPNGVTSGSSYPRRNNTCKSETGEAERIDTNQGGPPKPYVANKLPATVDGR

NGHW*

>BdTR3cMPK17 Brdisv1BdTR3C1006170m.p

MVGGGGIVDGFRRLFHRRTPSGSGPSSNQSSAGEESSDIDAVVEDLDLVGLRAIRVPKRK

MPLPVESHKKGEASQYQIQEVIGKGSYGVVAAAVDTRTGERVAIKKINDVFEHVSDATRI

LREVKLLRLLRHPDVVEIKHIMLPPSRREFQDIYVVFELMESDLHQVIRANDDLTPEHYQ

FFLYQLLRALKYIHAANVFHRDLKPKNILANSDCKLKICDFGLARVSFNDAPTAIFWTDY

VATRWYRAPELCGSFFSKYTPAIDIWSIGCIFAELLTGRPLFPGKNVVHQLDIITDLLGT

PSSETLSRIRNEKARRYLSCMRKKHPVPLTQKFPNVDPLALRLLGRLLAFDPKDRPSAEE

ALADPYFAVLANVEREPSRNPISKLEFEFERRKVTKDDVRELIYREILEYHPQMLQEYMQ

GGEQLSFLYPSGVDRFKRQFAHLEENYSKGERGSPLQRKHASLPRQRVGASNDNNEQHAS

DQERGADSIAPATGNPPGSQDAGQQHVSGGQNGVGSTNISPRSYQKSASISASKCVVVNA

NKNPEYDDDISEEMEGAVDGLSEKVSRMHP*

>BdTR3cMPK20-1 Brdisv1BdTR3C1021125m.p

MQQDQRKKSSAEAEFFTEYGDASRYKIQEIVGKGSYGVVCSAIDVHTGEKVAIKKIHDIF

EHISDAARILREIKLLRLLRHPDIVEIKHIMLPPSRRDFKDIYVVFELMESDLHQVIKAN

DDLTKEHYQFFLYQLLRALKYIHTANVYHRDLKPKNILANSNCKLKICDFGLARVAFNDT

PTTIFWTDYVATRWYRAPELCGSFFSKYTPAIDVWSIGCIFAEVLTGKPLFPGKNVVHQL

DLMTDLLGTPSMDTISRVRNEKARRYLSSMRKKEPIPFSQKFPNADPLALDLLQRLLAFD

PKDRPTAGEALSHPYFKGLAKVEREPSCQPITKMEFEFERRRATKEDIRELIFREILEYH

PQLLKDYINGTERTTFLYPSAVDQFRKQFAHLEENSESGPVVPMDRKHTSLPRSTIVHSA

PIHAKEQPRIGPSRDRPLTDESYKNPRDSEKYSGNVPRSSHHSQAPQRVPTARPGRVVGP

VLPYENVGTSHPYDPRRVAMNSGYPPQQQIPQTYGYYQTTGKSACSEPSQAERYTLHQQA

YACANSSTAPDVALDMRAPPFHQSAGPKSGSSDRLTAETNLYTRSLNGIVATTSGVAASA

HRKVSVVPYGMSQMY*

>BdTR3cMPK20-2 Brdisv1BdTR3C1016467m.p

MQQPQQQGQRNKSTDVDFFSEYGDANRYRIQEVIGKGSYGVVCSAMDMQTRQKVAIKKIH

NIFDHVSDAARILREIKLLRLLRHPDVVEIKHIMLPPSRKDFKDIYVVFELMESDLHQVI

KANDDLTKEHYQFFLYQLLRALKYIHTASVYHRDLKPKNILANSNCKLKICDFGLARVAF

NDTPTTVFWTDYVATRWYRAPELCGSFFTKYTPAIDIWSIGCIFAEVLTGKPLFPGKNVV

HQLDLMTDLLGTPSMDTISRVRNEKARRYLSSMRKKDPVPFSKKFPNADPLGLKLLEKLL

AFDPKDRPTAEEALTDPYFKSLSKPDREPSCQPIRKVEFDFEHRRMSKDDIRELIFQEIL

EYHPQLLKNYIDGTEKTTFLYPSAVDQFKKQFSHLEESDGSGPVVPTERKHASLPRSTTV

HSTPIPAKEQPLVASSRGRPIANEPCKPWAPGNVPGASQTAHVAQAGRAVGVGSVPPYES

GSGKYPYDATSRPAVSSGYPPQQKIPQTYGYHHHHQTPGAGQSSQAMGGYACGYTKGTTP

PPAAAQDMRASPYHHRSAGTKNDPLNRLAAESDIYTRSLNGIVAAAASAGTGAHRKVGAV

PFGMSGMY*

>BdTR3cMPK20-3 Brdisv1BdTR3C1007009m.p

MQHGDLHKKSAAEMDFFTAYDDANRYKILEVIGKGSYGLVCSANDLQTGEKVAIKKIHNI

FEHISDAARILREIKLLRLLRHPDVVEIKHILLPPSKKDFKDIYVVFELMESDLHQVIKA

NDDLTREHYQFFLYQMLRALKYMHTANVYHRDLKPKNVLANANCKLKICDFGLARVAFSD

APTTVFWTDYVATRWYRAPELCGSFYSKYTPAIDIWSIGCIFAEVLIGKPLFPGKNVVHQ

LDLITDILGTPSLDAISRVRNDKARKYLTCMRKKQPASFSQKFPKADPLALQLLRRLLAF

DPKDRPSAEEALADPYFNGLAKVEREPSCQPIPKIEFEFEGRRVTKEDIKELIFEEILEY

HPQLLKEHIIGKERPNFVHLSAVDQFKKHFTQLEENDNETGAAVSLQRKHSSLPRQAFNH

R*

>BdTR3cMPK20-4 Brdisv1BdTR3C1021364m.p

MQTTEQQRKKGSSEMDFFSEYGDANRYKIQEVIGKGSYGVVCSAIDQHTGDKVAIKKIHN

IFEHLSDAARILREIKLLRLLRHPDIVEIRHIMLPPSRRDFKDIYVVFELMDTDLHQVIK

ANDDLTKEHHQFFLYQMLRALKYIHTANVYHRDLKPKNILANANCKLKICDFGLARVAFN

DTPTTVFWTDYVATRWYRAPELCGSFFTKYSRAIDIWSIGCIFAEILTGKPLFPGKNVVH

QLDLMTDLLGTPSLDTVSRIRNEKARRYLSSMRKKQSVSFSERFPKADPAALKLLQRLLA

FDPKDRPTAEEALADPYFKGLGKVEREPSCQPITKMEFEFERKNVTKADVKELIFREILE

YHPQLLKDYMNGTEKTNFLYPSAVDNFRRQFANLEENGGKGGAVVPPDRKHVSLPRNTTV

HSTPIPPKDQKYSQVPQRIPTGRPGRVVGPVIPFENSSTMDPYSQRRGARNPVLPAAATN

LSAYAYRNSDNSERELQQELEKDRMQYHPMQRFMDAKMVSPDLRSTSYYMPKGVPKADVA

ERSALQSNMMQGIAPFNGIATVGGAFNKVSAVQYGVSRMY*

>BdTR3cMPK20-5 Brdisv1BdTR3C1016625m.p

MFSDIFSREGVAFSRVALRQELLSDQWFTTAGGKGQCYLVPPGTTVFLVNSEEMGFFSEY

VDASRYKILEIIGKGSYGVVCSAIDQETGDKVAIKKIQNIFEHLSDAARILREIKLLRLL

RHPDIVQIKHIMLPPSRRDFRDIYVVFELMDTDLHQVIKANDDLTKEHYQFFLYQMLRAL

KYIHTANVYHRDLKPKNILANANCKLKICDFGLARVAFNDTPTTVFWTDYVATRWYRAPE

LCGSFFTKYSPAIDTWSIGCIFAEILTGKPLFPGKNVVHQLDLMTDLLGIPSTETISRIR

NDKARKYLSSMRRKQPIPFSEKFPNADPSALKLLERLLAFDPKDRPTAEEALAHPYFKRL

ARVEREPSCQQPISKTEFEFERRKFTKEDIKELIFREILEYHPKLLKDYMNGSEKTSFLY

PSAVDNFRRQFANLEIDGGRSGAADRKHFSLPRTTTVHSAPILPTNGPTSQVPQRIPTAR

PGRVVSSAMQTDNPSVSDRHNGRRVARDPAVPPAAAYHLKSDYSDRQHQQEFEKDRVRPD

RQRQEELEKDRMQYRPGHHSMDAKVAPEISPYMRSSPYYIPPFNGIAAVASGYSKVAAVT

RMY*

>BdTR3cMPK21-1 Brdisv1BdTR3C1016518m.p

MGGLIRWLRHHRSRRVSSSSSSHLPSNTTSSSSTSDLRAHSLPQHQGDHHGEVVVEWEDA

AEGPDSDPEEYIVVVLGDDEQGVVAARAPVRTKPPRVMDPGKKTSESEFFTEYGEANRYK

VSEVIGKGSYGVVAAAVDTQTGERCAIKKINDVFDHVSDATRILREIKLLRLLRHPDIVE

IKHIMLPPSRREFRDIYVVFELMESDLHQVIKANDDLTPEHHQFFLYQLLRGMKYIHTAS

VFHRDLKPKNILANADCKLKICDFGLARVSFNDGAPSAIFWTDYVATRWYRAPELCGSFF

SKYTPAIDIWSVGCIFAEMLTGKPLFPGKNVVHQLDLMTDLLGTPSAESLSKIRNEKARR

YLSNMRKKPKVPLTKKFPGIDPMALHLLERLLAFDPKDRPSADEALTDPYFTGLANSERE

PITQPISKLEFEFEKRKLAKDDVRELIYREILEYHPHMLQEYLRGGGDQMSFMFPSGVDR

FKRQFAHLEEGGAKGEKSSPQLRQNASLPRERVIGNKHGDGDYNMKLNVGEKPELASVSD

GISKPLMSARSLLKSETMSASKCIGEIKNKDEDSLSECVEGTDDDVSQKIAQLKT*

>BdTR3cMPK21-2 Brdisv1BdTR3C1021213m.p

MARKGSDEAEFFTEYGEANRYEVGEVVGKGSYGVVAAAVDTHTGERVAIKKINDVFEHVS

DATRILREIKLLRLLRHPDIVEIKHIILPPSRREFRDIYIIFELMESDLHQVIKANDDLT

PEHHQFFFYQLLRGMKYIHAANVFHRDLKPRNILANADCKLKICDFGLARVSFNDTPSAI

FWTDYVATRWYRAPELCGSFFSKYTPAIDIWSIGCIFAEMLSGRPLFPGKNVVHQLDLMT

DLLGTPSAESLSRIRNEKARRYLGNMRKKHPVPFSQKFPGVDPMALDLLERLLAFDPKDR

PTAAEALADPYFTGLANSDREPTTQPISKLEFEFERRKLARDDVRELIYREILEYHPQML

HEYHHGGDQANFVYPSGVDRFKRQFVHLEEGVTKGEKTSPQLRQHASLPRERIIGIGDEL

GRPNADYCIKLHVGEEPGHTSVTDGLSKPLLNARNFLKSESISASQCVVIKEKREKDEES

MSEYMHEASDATPK*

>Gaz-8MPK3 Brdisv1Gaz-81005117m.p

MNFETREMVAIKKIANAFDNNMDAKRTLREIKLLRHLDHENIVGLRDVIPPAIPQSFNDV

YIATELMDTDLHHIIRSNQELSEEHCQYFLYQLLRGLKYIHSANVIHRDLKPSNLLLNAN

CDLKICDFGLARPSSESDMMTEYVVTRWYRAPELLLTSPDYSAAIDMRLITEVIGTPTDD

DLGFIRNEDARRYMRHLPQFPRRPFPAQFPRVQPAALDLIERMLAFNPLQRITVEEALDH

PYLERLHDIADEPICTDPFSFDFEQHPLTEDQMKQLIFNEALELTPNFRY*

>Gaz-8MPK4 Brdisv1Gaz-81017044m.p

MDHENIIAIKDIIRPPRRDDFKDVYIVTELMDTDLHQIIRSNQPLTDDHCQYFLYQLLRG

LKYVHSANVLHRDLKPSNLFLNANCDLKIADFGLARTTSETDLMTEYVVTRWYRAPELLL

NCSQYTAAIDVWSVGCILGEIITRQPLFPGRDYIQQLKLITELIGSPDDSSLGFLRSDNA

RRYMKQLPQYPRQDFRLRFRNMSDGAVDLLERMLVFDPSRRITVDEALHHPYLASLHDIN

EEPTCPAPFSFDFEQPSFTEEHMKELIWRETLAFNPDPPY*

>Gaz-8MPK6 Brdisv1Gaz-81003701m.p

MTGDQTLASMTNYGRFSQIETAYWLVSYNKQSVSAFNVPVIVAIRDIIPPAQRNSFNDVY

IAYELMDTDLHQIIRSNQALSEEHCQYFLYQILRGLKYIHSANVLHRDLKPSNLLLNANC

DLKICDFGLARTTSETDFMTEYVVTRWYRAPELLLNSSEYTAAIDVWSVGCIFMELMDRK

PLFPGRDHLIGTPNEADLDFVNENARRYIRQLPRHARQSFSEKFPHVHPSAIDLVEKMLT

FDPRQRITVEGALAHPYLASLHDISDEPVCTMPFSFDFEQHALSEEQMKDLIHQEALAFN

PDYQ*

>Gaz-8MPK7-1 Brdisv1Gaz-81013145m.p

MIHTQASDEGAPLPLAADASAGVHEGRRGSRGREGRRERQLLRGLKYLHSAGILHRDLKP

GNLLVNANCDLKICDFGLARTNNTKGQFMTEYVVTRWYRAPELLLCCDNYGTSIDVWSVG

CIFAELLGRKPIFPRTQCLDQLKLIVNVLGTMSENDLEFIDNPKARKYIKSLPYTPGTPL

TSMYPQAHPLAIDLLQKMLVFDPSKRISVTEALEHPYMSPLYDPSANPPAQVPIDLDIDE

NLGVEMIREMLWQEMLQYHPEAARMVNM*

>Gaz-8MPK11 Brdisv1Gaz-81016151m.p

XYGNPFEGSAKYVPPIRPVGRGACGIIWDTREEVAIKKIGNAFDNQIDAKRTLREVKLLR

HMNHENVISIKDIIRPPRRENFNDVYIVYELMDTDLHHLLRSNQPLTDDHCQYFLYQVLR

GLKYVHSANVLHRDLRPSNLLLNAKCDLKIGDFGLARTTTETDFMMEYVVTRWYRAPELL

LNCSEYTGAIDMWSVGCILGEIATREPLFPGKDYVHQLRLITELLGSPDDTSLGFLRSDN

ARRYVRSLPQYPKQQFRSRFPNMSSGAMDLLERMLVFDPNKRITVDEALCHPYLASLHEI

NDDPVCPAPFSFDFEQPSFTEEDIKELIWRESVKFNPEPIH*

>Gaz-8MPK14 Brdisv1Gaz-81024368m.p

MAMLVDPPNGMGNQGKHYYSMWQTLFEIDTKYVPIKPIGRGAYGIVCSSINRETNEKVAI

KKIHNVFDNRVDALRTLRELKLLRHLRHENVISLKDIMMPVQRRSFKDVYLVYELMDTDL

HQIIKSPQGLSNDHCQYFLFQLLRGLKYLHSAEILHRDLKPGNLLVNANCDLKICDFGLA

RTNSSKGQFMTEYVVPRWYRAPELLLCCDNYGTSIDVWSVGCIFAELLGRKPIFPGTECL

NQLKLIVNVLGTMSESDLEFIDNPKARRYIKTLPYTPGVPLASMYPHAHPLAIDLLQKML

IFDPTKRISVTEALEHPYMSPLYDPSANPPAQVPIDLDLDENISADMIREMMWQEMLHYH

PEAATAISM*

>Gaz-8MPK16 Brdisv1Gaz-81023301m.p

MDFFTEYGEGNRYKIEEVIGKGSYGVVCSALDTHTGDKVAIKKINDIFEHVSDATRILRE

IKLLRLLRHPDIVEIKHILLPPSRREFKDIYVVFELMESDLHQVIKANDDLTPEHYQFFL

YQLLRGLKYIHTANVFHRDLKPKNILANADCKLKICDFGLARVAISDTPTAIFWTDYIAT

RWYRAPELCGSFFSKYTPAIDIWSIGCIFAELLTGKPLFPGKNVVHQLDIITDLLGTPSA

ETIARIRNEKARRYLSSMRRKKTVPFTQKFPNADPLALNLLERMLAFDPKDRPSAEEALA

DPYFKNIASVDREPSAQPITKLEFEFERRRITKDDIRELIYREILEYHPNMLREFLEGAE

PTGFMYPSAVDHFKKQFTFLEEHYAKGSTAAPPERQHNSLPRPSVIYSDNRPQNAANITD

DLSRCAIRDNTQKAQKDSASVGANRIPHGAAAPAARPGKVVGSVLRYGNCSTSGTEQYEQ

RRVVRSPGIAPNGVTSGSSYPRRNNTCKSETGEAERIDTNQGGPPKPYVANKLPATVDGR

NGHW*

>Gaz-8MPK17 Brdisv1Gaz-81009612m.p

MPLPVESHKKNILEKEFFTEYGEASQYQIQEVIGKGSYGVVAAAVDTRTGERVAIKKIND

VFEHVSDATRILREVKLLRLVRHPDVVEIKHIMLPPSRREFQDIYVVFELMESDLHQVIR

ANDDLTPEHYQFFLYQLLRALKYIHAANVFHRDLKPKNILANSDCKLKICDFGLARVSFN

DAPTAIFWTDYVATRWYRAPELCGSFFSKYTPAIDIWSIGCIFAELLTGRPLFPGKNVVH

QLDIITDLLGTPSSETLSRIRNEKARRYLSCMRKKHPVPLTQKFPNVDPLALRLLGRLLA

FDPKDRPSAEEALADPYFAVLANVEREPSRNPISKLEFEFERRKVTKDDVRELIYREILE

YHPQMLQEYMQGGEQLSFLYPSGGDRFKRQFAHLEENYSKGERGSPLQRKHASLPRQRVG

ASNDNNEQHASDQERGADSIAPATGNPPGSQDAGQQHVSGGQNGVGSTNISPRSYQKSAS

ISASKCVVVNANKNPEYDDDISEEMEGAVDGLSEKVSRMHP*

>Gaz-8MPK20-1 Brdisv1Gaz-81009550m.p

MQQDQRKKSSAEAEVFTEYGDASRYKIQEIVGKGSYGVVCSAIDVHTGEKVAIKKIHDIF

EHISDAARILREIKLLRLLRHPDIVEIKHIMLPPSRRDFKDIYVVFELMESDLHQVIKAN

DDLTKEHYQFFLYQLLRALKYIHTANVYHRDLKPKNILANSNCKLKICDFGLARVAFNDT

PTTIFWTDYVATRWYRAPELCGSFFSKYTPAIDVWSIGCIFAEVLTGKPLFPGKNVVHQL

DLMTDLLGTPSMDTISRVRNEKARRYLSSMRKKEPIPFSQKFPNADPLALDLLQRLLAFD

PKDRPTAGEALSHPYFKGLAKVEREPSCQPITKMEFEFERRRATKEDIRELIFREILEYH

PQLLKDYINGTERTTFLYPSAVDQFRKQFAHLEENSESGPVVPMDRKHTSLPRSTIVHSA

PIHAKEQPRIGPSRDRPLTDESYKNPRDSEKYSGNVPRSSHHSQAPQRVPTARPGRVVGP

VLPYENVGTSHPYDPRRVAMNSGYPPQQQIPQTYGYYQTTGKSACSEPSQAERYTLHQQA

YACANSSTAPDVALDMRAPPFHQSAGPKSGSSDRLTAETNLYTRSLNGIVATTSGVAASA

HRKVSVVPYGMSQMY*

>Gaz-8MPK20-2 Brdisv1Gaz-81007554m.p

MQQPQQQGQRNKSTDVDFFSEYGDANRYRIQEVIGKGSYGVVCSAMDMQTRQKVAIKKIH

NIFDHVSDAARILREIKLLRLLRHPDVVEIKHIMLPPSRKDFKDIYVVFELMESDLHQVI

KANDDLTKEHYQFFLYQLLRALKYIHTASVYHRDLKPKNILANSNCKLKICDFGLARVAF

NDTPTTVFWTDYVATRWYRAPELCGSFFTKYTPAIDIWSIGCIFAEVLTGKPLFPGKNVV

HQLDLMTDLLGTPSMDTISRVRNEKARRYLSSMRKKDPVPFSKKFPNADPLGLKLLEKLL

AFDPKDRPTAEEALTDPYFKSLSKPDREPSCQPIRKVEFDFEHRRMSKDDIRELIFQEIL

EYHPQLLKNYIDGTEKTTFLYPSAVDQFKKQFSHLEESDGSGPVVPTERKHASLPRSTTV

HSTPIPAKEQPLVASSRGRPIANEPCKPWAPGNVPGASQTAHVAQAGRAVGVGSVPPYES

GSGKYPYDATSRPAVSSGYPPQQKIPQTYGYHHHHQTPGAGQSSQAMGGYACG*

>Gaz-8MPK20-3 Brdisv1Gaz-81026459m.p

MQHGDLHKKSAAEMDFFTAYDDANRYKILEVIGKGSYGLVCSANDLQTGEKVAIKKIHNI

FEHISDAARILREIKLLRLLRHPDVVEIKHILLPPSKKDFKDIYVVFELMESDLHQVIKA

NDDLTREHYQFFLYQMLRALKYMHTANVYHRDLKPKNVLANANCKLKICDFGLARVAFSD

APTTVFWTDYVATRWYRAPELCGSFYSK

>Gaz-8MPK20-4 Brdisv1Gaz-81009686m.p

MQTTEQQREKGSSEMDFFSEYGDANRYKIQEVIGKGSYGVVCSAIDQHTGDKVAIKKIHN

IFEHLSDAARILREIKLLRLLRHPDIVEIRHIMLPPSRRDFKDIYVVFELMDTDLHQVIK

ANDDLTKEHHQFFLYQMLRALKYIHTANVYHRDLKPKNILANANCKLKICDFGLARVAFN

DTPTTVFWTDYVATRWYRAPELCGSFFTKYSRAIDIWSIGCIFAEILTGKPLFPGKNVVH

QLDLMTDLLGTPSLDTVSRIRNEKARRYLSSMRKKQSVSFSERFPKADPAALKLLQRLLA

FDPKDRPTAEEALADPYFKGLGKVEREPSCQPITKMEFEFERKNVTKADVKELIFREILE

YHPQLLKDYMNGTEKTNFLYPSAVDNFRRQFANLEENGGKGGAVVPPDRKHVSLPRNTTV

HSTPIPPKDQKYSQVPQRIPTGRPGRVVGPVIPFENSSTMDPYSQRRVARNPVLPAAATN

LSAYAYRNSDNSERELQQELEKDRMQYHPMQRFMDAKMVSPDLRSTSYYMPKGVPKADVA

ERSALQSNMMQGIAPFNGIATVGGAFNKVSAVQYGVSRMY*

>Gaz-8MPK20-5 Brdisv1Gaz-81007614m.p

MFSDIFSREGVAFSRVALRQELLSDQWFTTAGGKGQCYLVPPGTTVFLVNSEEMGFFSEY

VDASRYKILEIIGKGSYGVVCSAIDQETGDKVAIKKIQNIFEHLSDAARILREIKLLRLL

RHPDIVQIKHIMLPPSRRDFRDIYVVFELMDTDLHQVIKANDDLTKEHYQFFLYQMLRAL

KYIHTANVYHRDLKPKNILANANCKLKICDFGLARVAFNDTPTTVFWTDYVATRWYRAPE

LCGSFFTKYSPAIDTWSIGCIFAEILTGKPLFPGKNVVHQLDLMTDLLGIPSTETISRIR

NDKARKYLSSMRRNQPIPFSEKFPNADPSALKLLERLLAFDPKDRPTAEEALAHPYFKRL

ARVEREPSCQQPISKTEFEFERRKFTKEDIKELIFREILEYHPKLLKDYMNGSEKTSFLY

PSAVDNFRRQFANLEIDGGRSGAADRKHFSLPRTTTVHSAPILPTNGPTSQVPQRIPTAR

PGRVVSSAMQTDNPSVSDRHNGRRVARDPAVPPAAAYHLKSDYSDRQHQQEFEKDRVRPD

RQRQEELEKDRMQYRPGHHSMDAKVAPEISPYMRSSPYYIPPFNGIAAVASGYSKVAAVT

RMY*

>Gaz-8MPK21-1 Brdisv1Gaz-81009615m.p

MDPGKKTSESEFFTEYGEANRYKVSEVIGKGSYGVVAAAVDTQTGERCAIKKINDVFDHV

SDATRILREIKLLRLLRHPDIVEIKHIMLPPSRREFRDIYVVFELMESDLHQVIKANDDL

TPEHHQFFLYQLLRGMKYIHTANVFHRDLKPKNILANADCKLKICDFGLARVSFNDGAPS

AIFWTDYVATRWYRAPELCGSFFSKYTPAIDIWSVGCIFAEMLTGKPLFPGKNVVHQLDL

MTDLLGTPSAESLSKIRNEKARRYLSNMRKKPKVPLTKKFPGIDPMALHLLERLLAFDPK

DRPSADEALTDPYFTGLANSEREPITQPISKLEFEFEKRKLAKDDVRELIYREILEYHPH

MLQEYLRGGGDQMSFMFPSGVDRFKRQFAHLEEGGAKGEKSSPQLRQNASLPRERVIGNK

HGDGDYNMKLNVGEKPELASVSDGISKPLMSARSLLKSETMSASKCIGEIKNKDEDSLSE

CVEGTDDDVSQKIAQLKT*

>Gaz-8MPK21-2 Brdisv1Gaz-81023218m.p

MARKGSDEAEFFTEYGEANRYEVGEVVGKGSYGVVAXXXXXXXXXXXXXXXXXXXXXXXX

XXVAIKKINDVFEHVSDATRILREIKLLRLLRHPDIVEIKHIILPPSRREFRDIYIIFEL

MESDLHQVIKANDDLTPEHHQFFFYQLLRGMKYIHAANVFHRDLKPRNILANADCKLKIC

DFGLARVSFNDTPSAIFWTDYVATRWYRAPELCGSFFSKYTPAIDIWSIGCIFAEMLSGR

PLFPGKNVVHQLDLMTDLLGTPSAESLSRIRNEKARRYLGNMRKKHPVPFSQKFPGVDPM

ALDLLERLLAFDPKDRPTAAEALADPYFTGLANSDREPTTQPISKLEFEFERRKLARDDV

RELIYREILEYHPQMLHEYHHGGDQANFVYPSGVDRFKRQFVHLEEGVTKGEKTSPQLRQ

HASLPRERIIGIGDELGRPNADYCIKLHVGEEPGHTSVTDGLSKPLLNARNFLKSESISA

SQCVVIKEKREKDEESMSEYMHEASDATPK*

>BdTR1iMPK3 Brdisv1BdTR1i1011291m.p

MDGAPVAEFRPTMTHGGRFLLYNIFGNQFEITAKYQPPIMPIGRGAYGIVCSVMNFETRE

MVAIKKIANAFDNNMDAKRTLREIKLLRHLDHENIVGLRDVIPPAIPQSFNDVYIATELM

DTDLHHIIRSNQELSEEHCQYFLYQLLRGLKYIHSANVIHRDLKPSNLLLNANCDLKICD

FGLARPSSESDMMTEYVVTRWYRAPELLLNSTDYSAAIDVWSVGCIFMELINRAPLFPGR

DHMHQMRLITEVIGTPTDDDLGFIRNEDARRYMRHLPQFPRRPFPAQFPRVQPAALDLIE

RMLAFNPLQRITVEEALEHPYLERLHDIADEPICTDPFSFDFEQHPLTEDQMKQLIFNEA

LELNPNFRY*

>BdTR1iMPK4 Brdisv1BdTR1i1029278m.p

MDTSGGGGGGAGGAAQIQGMATHGGRYVLYNVYGNLFEVASKYAPPIRPIGRGAYGIVCA

AVSSDTGEEVAIKKIGNAFDNHIDAKRTLREIKLLRHMDHENIIAIKDIIRPPRRDDFKD

VYIVTELMDTDLHQIIRSNQPLTDDHCQYFLYQLLRGLKYVHSANVLHRDLKPSNLFLNA

NCDLKIADFGLARTTSETDLMTEYVVTRWYRAPELLLNCSQYTAAIDVWSVGCILGEIIT

RQPLFPGRDYIQQLKLITELIGSPDDSSLGFLRSDNARRYMKQLPQYPRQDFRLRFRNMS

DGAVDLLERMLVFDPSRRITVDEALHHPYLASLHDINEEPTCPAPFSFDFEQPSFTEEHM

KELIWRETLAFNPDPPY*

>BdTR1iMPK6 Brdisv1BdTR1i1008313m.p

MDGGAQPPDAEMADAGAAVPGAGAGAAGTMENIQATLSHGGRFIQYNIFGNVFEVTVKYK

PPILPIGKGAYGIVCSALNSETGEQVAIKKIANAFDNKIDAKRTLREIKLLRHMDHENIV

AIRDIIPPAQRNSFNDVYIAYELMDTDLHQIIRSNQALSEEHCQYFLYQILRGLKYIHSA

NVLHRDLKPSNLLLNANCDLKICDFGLARTTSETDFMTEYVVTRWYRAPELLLNSSEYTA

AIDVWSVGCIFMELMDRKPLFPGRDHVHQLRLLMELIGTPNEADLDFVNENARRYIRQLP

RHARQSFSEKFPHVHPSAIDLVEKMLTFDPRQRITVEGALAHPYLASLHDISDEPVCTMP

FSFDFEQHALSEEQMKDLIHQEALAFNPDYQ*

>BdTR1iMPK7-1 Brdisv1BdTR1i1046789m.p

MPSSRTACSSSPTPRTVRPSAASAAASEVSTRTGRNSVRRTARSDRKALGVELLRGLKYL

HSAGILHRDLKPGNLLVNANCDLKICDFGLARTNNTKGQFMTEYVVTCLYRAPELLLCCD

NYGTSIDVWSVGCIFAELLGRKPIFPGTECLNQLKLIVNVLGTMSENDLEFIDNPKARKY

IKSLPYTPGTPLTSMYPQAHPLAIDLLQKMLVFDPSKRISVTEALEHPYMSPLYDPSANP

PAQVPIDLDIDENLGVEMIREMLWQEMLQYHPEAARMVNM*

>BdTR1iMPK11 Brdisv1BdTR1i1027104m.p

MRMEGGGAGPAAAAAAGGAHGLGEAQIKGTLTHGGRYVQYNVYGNLFEVSAKYVPPIRPV

GRGACGIICAAINVQTREEVAIKKIGNAFDNQIDAKRTLREVKLLRHMNHENVISIKDII

RPPRRENFNDVYIVYELMDTDLHHLLRSNQPLTDDHCQYFLYQVLRGLKYVHSANVLHRD

LRPSNLLLNAKCDLKIGDFGLARTTTETDFMMEYVVTRWYRAPELLLNCSEYTGAIDMWS

VGCILGEIATREPLFPGKDYVHQLRLITELLGSPDDTSLGFLRSDNARRYVRSLPQYPKQ

QFRSRFPNMSSGAMDLLERMLVFDPNKRITVDEALCHPYLASLHEINDEPVCPAPFSFDF

EQPSFTEEDIKELIWRESVKFNPEPIH*

>BdTR1iMPK14 Brdisv1BdTR1i1024796m.p

MAMLVDPPNGMGNQGKHYYSMWQTLFEIDTKYVPIKPIGRGAYGIVCSSINRETNEKVAI

KKIHNVFDNRVDALRTLRELKLLRHLRHENVISLKDIMMPVQRRSFKDVYLVYELMDTDL

HQIIKSPQGLSNDHCQYFLFQLLRGLKYLHSAEILHRDLKPGNLLVNANCDLKICDFGLA

RTNSSKGQFMTEYVVTRWYRAPELLLCCDNYGTSIDVWSVGCIFAELLGRKPIFPGTECL

NQLKLIVNVLGTMSESDLEFIDNPKARRYIKTLPYTPGVPLASMYPHAHPLAIDLLQKML

IFDPTKRISVTEALEHPYMSPLYDPSANPPAQVPIDLDLDENISADMIREMMWQEMLHYH

PEAATAISM*

>BdTR1iMPK16 Brdisv1BdTR1i1019190m.p

MDFFTEYGEGNRYKIEEVIGKGSYGVVCSALDTHTGDKVAIKKINDIFEHVSDATRILRE

IKLLRLLRHPDIVEIKHILLPPSRREFKDIYVVFELMESDLHQVIKANDDLTPEHYQFFL

YQLLRGLKYIHTANVFHRDLKPKNILANADCKLKICDFGLARVAISDTPTAIFWTDYIAT

RWYRAPELCGSFFSKYTPAIDIWSIGCIFAELLTGKPLFPGKNVVHQLDIITDLLGTPSA

ETIARIRNEKARRYLSSMRRKKTVPFTQKFPNADPLALNLLERMLAFDPKDRPSAEEALA

DPYFKNIASVDREPSAQPITKLEFEFERRRITKDDIRELIYREILEYHPNMLREFLEGAE

PTGFMYPSAVDHFKKQFTFLEEHYAKGSTAAPPERQHNSLPRPSVIYSDNRPQNAANITD

DLSRCAIRDNTQKAQKDSASVGANRIPHGAAAAAARPGKVVGSVLRYGNCSTSGTEQYEQ

RRVVRSPGIAPNGVTSGSSYPRRNNTCKSETGEAERIDTNQGGPPKPYVANKLPATVDGR

NGHW*

>BdTR1iMPK17 Brdisv1BdTR1i1006203m.p

MVGGGGIVDGFRRLFPRRTPSGSGPSSNQSSAGEESSDIDAVVEDLDLVGLRAIRVPKRK

MPLPVESHKKNILEKEFFTEYGEASQYQIQEVIGKGSYGVVAAAVDTRTGERVAIKKIND

VFEHVSDATRILREVKLLRLLRHPDVVEIKHIMLPPSRREFQDIYVVFELMESDLHQVIR

ANDDLTPEHYQFFLYQLLRALKYIHAANVFHRDLKPKNILANSDCKLKICDFGLARVSFN

DAPTAIFWTDYVATRWYRAPELCGSFFSKYTPAIDIWSIGCIFAELLTGRPLFPGKNVVH

QLDIITDLLGTPSSETLSRIRNEKARRYLSCMRKKHPVPLTQKFPNVDPLALRLLGRLLA

FDPKDRPSAEEALADPYFAVLANVEREPSRNPISKLEFEFERRKVTKDDVRELIYREILE

YHPQMLQEYMQGGEQLSFLYPSGVDRFKRQFAHLEENYSKGERGSPLQRKHASLPRQRVG

ASNDNNEQHASDQERGADSIAPATGNPPGSQDAGQQHVSGGQNGVGSTNISPRSYQKSAS

ISASKCVVVNANKNPEYDDDISEEMEGAVDGLSEKVSRMHP*

>BdTR1iMPK20-1 Brdisv1BdTR1i1020772m.p

MQQDQRKKSSAEAEFFTEYGDASRYKIQEIVGKGSYGVVCSAIDVHTGEKVAIKKIHDIF

EHISDAARILREIKLLRLLRHPDIVEIKHIMLPPSRRDFKDIYVVFELMESDLHQVIKAN

DDLTKEHYQFFLYQLLRALKYIHTANVYHRDLKPKNILANSNCKLKICDFGLARVAFNDT

PTTIFWTDYVATRWYRAPELCGSFFSKYTPAIDVWSIGCIFAEVLTGKPLFPGKNVVHQL

DLMTDLLGTPSMDTISRVRNEKARRYLSSMRKKEPIPFSQKFPNADPLALDLLQRLLAFD

PKDRPTAGEALSHPYFKGLAKVEREPSCQPITKMEFEFERRRATKEDIRELIFREILEYH

PQLLKDYINGTERTTFLYPSAVDQFRKQFAHLEENSESGPVVPMDRKHTSLPRSTIVHSA

PIHAKEQPRIGPSRDRPLTDESYKNPRDSEKYSGNVPRSSHHSQAPQRVPTARPGRVVGP

VLPYENVGTSHPYDPRRVAMNSGYPPQQQIPQTYGYYQTTGKSACSEPSQAERYTLHQQA

YACANSSTAPDVALDMRAPPFHQSAGPKSGSSDRLTAETNLYTRSLNGIVATTSGVAASA

HRKVSVVPYGMSQMY*

>BdTR1iMPK20-2 Brdisv1BdTR1i1016104m.p

MQQPQQQGQRNKSTDVDFFSEYGDANRYRIQEVIGKGSYGVVCSAMDMQTRQKVAIKKIH

NIFDHVSDAARILREIKLLRLLRHPDVVEIKHIMLPPSRKDFKDIYVVFELMESDLHQVI

KANDDLTKEHYQFFLYQLLRALKYIHTASVYHRDLKPKNILANSNCKLKICDFGLARVAF

NDTPTTVFWTDYVATRWYRAPELCGSFFTKYTPAIDIWSIGCIFAEVLTGKPLFPGKNVV

HQLDLMTDLLGTPSMDTISRVRNEKARRYLSSMRKKDPVPFSKKFPNADPLGLKLLEKLL

AFDPKDRPTAEEALTDPYFKSLSKPDREPSCQPIRKVEFDFEHRRMSKDDIRELIFQEIL

EYHPQLLKNYIDGTEKTTFLYPSAVDQFKKQFSHLEESDGSGPVVPTERKHASLPRSTTV

HSTPIPAKEQPLVASSRGRPIANEPCKPWAPGNVPGASQTAHVAQAGRAVGVGSVPPYES

GSGKYPYDATSRPAVSSGYPPQQKIPQTYGYHHHHQTPGAGQSSQAMGGYACGYTKGTTP

PPAAAQDMRASPYHHRSAGTKNDPLNRLAAESDIYTRSLNGIVAAAASAGTGAHRKVGAV

PFGMSGMY*

>BdTR1iMPK20-3 Brdisv1BdTR1i1006964m.p

MQHGDLHKKSAAEMDFFTAYDDANRYKILEVIGKGSYGLVCSANDLQTGEKVAIKKIHNI

FEHISDAARILREIKLLRLLRHPDVVEIKHILLPPSKKDFKDIYVVFELMESDLHQVIKA

NDDLTREHYQFFLYQMLRALKYMHTANVYHRDLKPKNVLANANCKLKICDFGLARVAFSD

APTTVFWTDYVATRWYRAPELCGSFYSKVMIVTIDH*

>BdTR1iMPK20-4 Brdisv1BdTR1i1035579m.p

MQTTEQQRKKGSSEMDFFSEYGDANRYKIQEVIGKGSYGVVCSAIDQHTGDKVAIKKIHN

IFEHLSDAARILREIKLLRLLRHPDIVEIRHIMLPPSRRDFKDIYVVFELMDTDLHQVIK

ANDDLTKEHHQFFLYQMLRALKYIHTANVYHRDLKPKNILANANCKLKICDFGLARVAFN

DTPTTVFWTDYVATRWYRAPELCGSFFTKYSRAIDIWSIGCIFAEILTGKPLFPGKNVVH

QLDLMTDLLGTPSLDTVSRIRNEKARRYLSSMRKKQSVSFSERFPKADPAALKLLQRLLA

FDPKDRPTAEEALADPYFKGLGKVEREPSCQPITKMEFEFERKNVTKADVKELIFREILE

YHPQLLKDYMNGTEKTNFLYPSAVDNFRRQFANLEENGGKGGAVVPPDRKHVSLPRNTTV

HSTPIPPKDQKYSQVPQRIPTGRPGRVVGPVIPFENSSTMDPYSQRRVARNPVLPAAATN

LSAYAYRNSDNSERELQQELEKDRMQYHPMQRFMDAKMVSPDLRSTSYYMPKGVPKADVA

ERSALQSNMMQGIAPFNGIATVGGAFNKVSAVQYGVSRMY*

>BdTR1iMPK20-5 Brdisv1BdTR1i1016265m.p

MFSDIFSREGVAFSRVALRQELLSDQWFTTAGGKGQCYLVPPGTTVFLVNSEEMGFFSEY

VDASRYKILEIIGKGSYGVVCSAIDQETGDKVAIKKIQNIFEHLSDAARILREIKLLRLL

RHPDIVQIKHIMLPPSRRDFRDIYVVFELMDTDLHQVIKANDDLTKEHYQFFLYQMLRAL

KYIHTANVYHRDLKPKNILANANCKLKICDFGLARVAFNDTPTTVFWTDYVATRWYRAPE

LCGSFFTKYSPAIDTWSIGCIFAEILTGKPLFPGKNVVHQLDLMTDLLGIPSTETISRIR

NDKARKYLSSMRRKQPIPFSEKFPNADPSALKLLERLLAFDPKDRPTAEEALAHPYFKRL

ARVEREPSCQQPISKTEFEFERRKFTKEDIKELIFREILEYHPKLLKDYMNGSEKTSFLY

PSAVDNFRRQFANLEIDGGRSGAADRKHFSLPRTTTVHSAPILPTNGPTSQVPQRIPTAR

PGRVVSSAMQTDNPSVSDRHNGRRVARDPAVPPAAAYHLKSDYSDRQHQQEFEKDRVRPD

RQRQEELEKDRMQYRPGHHSMDAKVAPEISPYMRSSPYYIPPFNGIAAVASGYSKVAAVT

RMY*

>BdTR1iMPK21-1 Brdisv1BdTR1i1016152m.p

MGGLIRWLRHPRSRRVSSSSSSHLPSNTTSSSSTSDLRAHSLPQHQGDHHGEVVVEWEDA

AEGPDSDPEEYIVVVLGDDEQGVVAARAPVRTKPPRVMDPGKKTSESEFFTEYGEANRYK

VSEVIGKGSYGVVAAAVDTQTGERCAIKKINDVFDHVSDATRILREIKLLRLLRHPDIVE

IKHIMLPPSRREFRDIYVVFELMESDLHQVIKANDDLTPEHHQFFLYQLLRGMKYIHTAN

VFHRDLKPKNILANADCKLKICDFGLARVSFNDGAPSAIFWTDYVATRWYRAPELCGSFF

SKYTPAIDIWSVGCIFAEMLTGKPLFPGKNVVHQLDLMTDLLGTPSAESLSKIRNEKARR

YLSNMRKKPKVPLTKKFPGIDPMALHLLERLLAFDPKDRPSADEALTDPYFTGLANSERE

PITQPISKLEFEFEKRKLAKDDVRELIYREILEYHPHMLQEYLRGGGDQMSFMFPSGVDR

FKRQFAHLEEGGAKGEKSSPQLRQNASLPRERVIGNKHGDGDYNMKLNVGEKPELASVSD

GISKPLMSARSLLKSETMSASKCIGEIKNKDEDSLSECVEGTDDDVSQKIAQLKT*

>BdTR1iMPK21-2 Brdisv1BdTR1i1020854m.p

MARKGSDEAEFFTEYGEANRYEVGEVVGKGSYGVVAAAVDTHTGERVAIKKINDVFEHVS

DATRILREIKLLRLLRHPDIVEIKHIILPPSRREFRDIYIIFELMESDLHQVIKANDDLT

PEHHQFFFYQLLRGMKYIHAANVFHRDLKPRNILANADCKLKICDFGLARVSFNDTPSAI

FWTDYVATRWYRAPELCGSFFSKYTPAIDIWSIGCIFAEMLSGRPLFPGKNVVHQLDLMT

DLLGTPSAESLSRIRNEKARRYLGNMRKKHPVPFSQKFPGVDPMALDLLERLLAFDPKDR

PTAAEALADPYFTGLANSDREPTTQPISKLEFEFERRKLARDDVRELIYREILEYHPQML

HEYHHGGDQANFVYPSGVDRFKRQFVHLEEGVTKGEKTSPQLRQHASLPRERIIGIGDEL

GRPNADYCIKLHVGEEPGHTSVTDGLSKPLLNARNFLKSESISASQCVVIKEKREKDEES

MSEYMHEASDATPK*

>BdTR2bMPK3 Brdisv1BdTR2B1010533m.p

MDGAPVAEFRPTMTHGGRFLLYNIFGNQFEITAKYQPPIMPIGRGAYGIVCSVMNFETRE

MVAIKKIANAFDNNMDAKRTLREIKLLRHLDHENIVGLRDVIPPAIPQSFNDVYIATELM

DTDLHHIIRSNQELSEEHCQYFLYQLLRGLKYIHSANVIHRDLKPSNLLLNANCDLKICD

FGLARPSSESDMMTEYVVTRWYRAPELLLNSTDYSAAIDVWSVGCIFMELINRAPLFPGR

DHMHQMRLITEVIGTPTDDDLGFIRNEDARRYMRHLPQFPRRPFPAQFPRVQPAALDLIE

RMLAFNPLQRITVEEALEHPYLERLHDIADEPICTDPFSFDFEQHPLTEDQMKQLIFNEA

LELNPNFRY*

>BdTR2bMPK4 Brdisv1BdTR2B1027249m.p

MDTSGGGGGGAGGAAQIQGMATHGGRYVLYNVYGNLFEVASKYAPPIRPIGRGAYGIVCA

AVSSDTGEEVAIKKIGNAFDNHIDAKRTLREIKLLRHMDHENIIAIKDIIRPPRRDDFKD

VYIVTELMDTDLHQIIRSNQPLTDDHCQYFLYQLLRGLKYVHSANVLHRDLKPSNLFLNA

NCDLKIADFGLARTTSETDLMTEYVVTRWYRAPELLLNCSQYTAAIDVWSVGCILGEIIT

RQPLFPGRDYIQQLKLITELIGSPDDSSLGFLRSDNARRYMKQLPQYPRQDFRLRFRNMS

DGAVDLLERMLVFDPSRRITVDEALHHPYLASLHDINEEPTCPAPFSFDFEQPSFTEEHM

KELIWRETLAFNPDPPY*

>BdTR2bMPK6 Brdisv1BdTR2B1007594m.p

MDGGAQPPDAEMADAGAAVPGAGAGAAGTMENIQATLSHGGRFIQYNIFGNVFEVTVKYK

PPILPIGKGAYGIVCSALNSETGEQVAIKKIANAFDNKIDAKRTLREIKLLRHMDHENIV

AIRDIIPPAQRNSFNDVYIAYELMDTDLHQIIRSNQALSEEHCQYFLYQILRGLKYIHSA

NVLHRDLKPSNLLLNANCDLKICDFGLARTTSETDFMTEYVVTRWYRAPELLLNSSEYTA

AIDVWSVGCIFMELMDRKPLFPGRDHVHQLRLLMELIGTPNEADLDFVNENARRYIRQLP

RHARQSFSEKFPHVHPSAIDLVEKMLTFDPRQRITVEGALAHPYLASLHDISDEPVCTMP

FSFDFEQHALSEEQMKDLIHQEALAFNPDYQ*

>BdTR2bMPK7-1 Brdisv1BdTR2B1045837m.p

LLRGLKYLHSAGILHRDLKPGNLLVNANCDLKICDFGLARTNNTKGQFMTEYVVTCLYRA

PELLLCCDNYGTSIDVWSVGCIFAELLGRKPIFPRTQCLDQLKLIVNVLGTMSENDLEFI

DNPKARKYIKSLPYTPGTPLTSMYPQAHPLAIDLLQKMLVFDPSKRISVTEALEHPYMSP

LYDPSANPPAQVPIDLDIDENLGVEMIREMLWQEMLQYHPEAARMVNM*

>BdTR2bMPK11 Brdisv1BdTR2B1025399m.p

MRMEGGGAGPAAAAAAGGAHGLGEAQIKGTLTHGGRYVQYNVYGNLFEVSAKYVPPIRPV

GRGACGIICAAINVQTREEVAIKKIGNAFDNQIDAKRTLREVKLLRHMNHENVISIKDII

RPPRRENFNDVYIVYELMDTDLHHLLRSNQPLTDDHCQYFLYQVLRGLKYVHSANVLHRD

LRPSNLLLNAKCDLKIGDFGLARTTTETDFMMEYVVTRWYRAPELLLNCSEYTGAIDMWS

VGCILGEIATREPLFPGKDYVHQLRLITELLGSPDDTSLGFLRSDNARRYVRSLPQYPKQ

QFRSRFPNMSSGAMDLLERMLVFDPNKRITVDEALCHPYLASLHEINDEPVCPAPFSFDF

EQPSFTEEDIKELIWRESVKFNPEPIH*

>BdTR2bMPK14 Brdisv1BdTR2B1023291m.p

MAMLVDPPNGMGNQGKHYYSMWQTLFEIDTKYVPIKPIGRGAYGIVCSSINRETNEKVAI

KKIHNVFDNRVDALRTLRELKLLRHLRHENVISLKDIMMPVQRRSFKDVYLVYELMDTDL

HQIIKSPQGLSNDHCQYFLFQLLRGLKYLHSAEILHRDLKPGNLLVNANCDLKICDFGLA

RTNSSKGQFMTEYVVTRWYRAPELLLCCDNYGTSIDVWSVGCIFAELLGRKPIFPGTECL

NQLKLIVNVLGTMSESDLEFIDNPKARRYIKTLPYTPGVPLASMYPHAHPLAIDLLQKML

IFDPTKRISVTEALEHPYMSPLYDPSANPPAQVPIDLDLDENISADMIREMMWQEMLHYH

PEAATAISM*

>BdTR2bMPK16 Brdisv1BdTR2B1043300m.p

MDFFTEYGEGNRYKIEEVIGKGSYGVVCSALDTHTGDKVAIKKINDIFEHVSDATRILRE

IKLLRLLRHPDIVEIKHILLPPSRREFKDIYVVFELMESDLHQVIKANDDLTPEHYQFFL

YQLLRGLKYIHTANVFHRDLKPKNILANADCKLKICDFGLARVAISDTPTAIFWTDYIAT

RWYRAPELCGSFFSKYTPAIDIWSIGCIFAELLTGKPLFPGKNVVHQLDIITDLLGTPSA

ETIARIRNEKARRYLSSMRRKKTVPFTQKFPNADPLALNLLERMLAFDPKDRPSAEEALA

DPYFKNIASVDREPSAQPITKLEFEFERRRITKDDIRELIYREILEYHPNMLREFLEGAE

PTGFMYPSAVDHFKKQFTFLEEHYAKGSTAAPPERQHNSLPRPSVIYSDNRPQNAANITD

DLSRCAIRDNTQKAQKDSASVGANRIPHGAAAAAARPGKVVGSVLRYGNCSTSGTEQYEQ

RRVVRSPGIAPNGVTSGSSYPRRNNTCKSETGEAERIDTNQGGPPKPYVANKLPATVDGR

NGHW*

>BdTR2bMPK17 Brdisv1BdTR2B1005613m.p

MVGGGGIVDGFRRLFPRRTPSGSGPSSNQSSAGEESSDIDAVVEDLDLVGLRAIRVPKRK

MPLPVESHKKNILEKEFFTEYGEASQYQIQEVIGKGSYGVVAAAVDTRTGERVAIKKIND

VFEHVSDATRILREVKLLRLLRHPDVVEIKHIMLPPSRREFQDIYVVFELMESDLHQVIR

ANDDLTPEHYQFFLYQLLRALKYIHAANVFHRDLKPKNILANSDCKLKICDFGLARVSFN

DAPTAIFWTDYVATRWYRAPELCGSFFSKYTPAIDIWSIGCIFAELLTGRPLFPGKNVVH

QLDIITDLLGTPSSETLSRIRNEKARRYLSCMRKKHPVPLTQKFPNVDPLALRLLGRLLA

FDPKDRPSAEEALADPYFAVLANVEREPSRNPISKLEFEFERRKVTKDDVRELIYREILE

YHPQMLQEYMQGGEQLSFLYPSGVDRFKRQFAHLEENYSKGERGSPLQRKHASLPRQRVG

ASNDNNEQHASDQERGADSIAPATGNPPGSQDAGQQHVSGGQNGVGSTNISPRSYQKSAS

ISASKCVVVNANKNPEYDDDISEEMEGAVDGLSEKVSRMHP*

>BdTR2bMPK20-1 Brdisv1BdTR2B1019350m.p

MQQDQRKKSSAEAEFFTEYGDASRYKIQEIVGKGSYGVVCSAIDVHTGEKVAIKKIHDIF

EHISDAARILREIKLLRLLRHPDIVEIKHIMLPPSRRDFKDIYVVFELMESDLHQVIKAN

DDLTKEHYQFFLYQLLRALKYIHTANVYHRDLKPKNILANSNCKLKICDFGLARVAFNDT

PTTIFWTDYVATRWYRAPELCGSFFSKYTPAIDVWSIGCIFAEVLTGKPLFPGKNVVHQL

DLMTDLLGTPSMDTISRVRNEKARRYLSSMRKKEPIPFSQKFPNADPLALDLLQRLLAFD

PKDRPTAGEALSHPYFKGLAKVEREPSCQPITKMEFEFERRRATKEDIRELIFREILEYH

PQLLKDYINGTERTTFLYPSAVDQFRKQFAHLEENSESGPVVPMDRKHTSLPRSTIVHSA

PIHAKEQPRIGPSRDRPLTDESYKNPRDSEKYSGNVPRSSHHSQAPQRVPTARPGRVVGP

VLPYENVGTSHPYDPRRVAMNSGYPPQQQIPQTYGYYQTTGKSACSEPSQAERYTLHQQA

YACANSSTAPDVALDMRAPPFHQSAGPKSGSSDRLTAETNLYTRSLNGIVATTSGVAASA

HRKVSVVPYGMSQMY*

>BdTR2bMPK20-2 Brdisv1BdTR2B1015236m.p

MQQPQQQGQRNKSTDVDFFSEYGDANRYRIQEVIGKGSYGVVCSAMDMQTRQKVAIKKIH

NIFDHVSDAARILREIKLLRLLRHPDVVEIKHIMLPPSRKDFKDIYVVFELMESDLHQVI

KANDDLTKEHYQFFLYQLLRALKYIHTASVYHRDLKPKNILANSNCKLKICDFGLARVAF

NDTPTTVFWTDYVATRWYRAPELCGSFFTKYTPAIDIWSIGCIFAEVLTGKPLFPGKNVV

HQLDLMTDLLGTPSMDTISRVRNEKARRYLSSMRKKDPVPFSKKFPNADPLGLKLLEKLL

AFDPKDRPTAEEALTDPYFKSLSKPDREPSCQPIRKVEFDFEHRRMSKDDIRELIFQEIL

EYHPQLLKNYIDGTEKTTFLYPSAVDQFKKQFSHLEESDGSGPVVPTERKHASLPRSTTV

HSTPIPAKEQPLVASSRGRPIANEPCKPWAPGNVPGASQTAHVAQAGRAVGVGSVPPYES

GSGKYPYDATSRPAVSSGYPPQQKIPQTYGYHHHHQTPGAGQSSQAMGGYACGYTKGTTP

PPAAAQDMRASPYHHRSAGTKNDPLNRLAAESDIYTRSLNGIVAAAASAGTGAHRKVGAV

PFGMSGMY*

>BdTR2bMPK20-3 Brdisv1BdTR2B1044368m.p

MQHGDLHKKSAAEMDFFTAYDDANRYKILEVIGKGSYGLVCSANDLQTGEKVAIKKIHNI

FEHISDAARILREIKLLRLLRHPDVVEIKHILLPPSKKDFKDIYVVFELMESDLHQVIKA

NDDLTREHYQFFLYQMLRALKYMHTANVYHRDLKPKNVLANANCKLKICDFGLARVAFSD

APTTVFWTDYVATRWYRAPELCGSFYSK

>BdTR2bMPK20-4 Brdisv1BdTR2B1019571m.p

MQTTEQQRKKGSSEMDFFSEYGDANRYKIQEVIGKGSYGVVCSAIDQHTGDKVAIKKIHN

IFEHLSDAARILREIKLLRLLRHPDIVEIRHIMLPPSRRDFKDIYVVFELMDTDLHQVIK

ANDDLTKEHHQFFLYQMLRALKYIHTANVYHRDLKPKNILANANCKLKICDFGLARVAFN

DTPTTVFWTDYVATRWYRAPELCGSFFTKYSRAIDIWSIGCIFAEILTGKPLFPGKNVVH

QLDLMTDLLGTPSLDTVSRIRNEKARRYLSSMRKKQSVSFSERFPKADPAALKLLQRLLA

FDPKDRPTAEEALADPYFKGLGKVEREPSCQPITKMEFEFERKNVTKADVKELIFREILE

YHPQLLKDYMNGTEKTNFLYPSAVDNFRRQFANLEENGGKGGAVVPPDRKHVSLPRNTTV

HSTPIPPKDQKYSQVPQRIPTGRPGRVVGPVIPFENSSTMDPYSQRRVARNPVLPAAATN

LSAYAYRNSDNSERELQQELEKDRMQYHPMQRFMDAKMVSPDLRSTSYYMPKGVPKADVA

ERSALQSNMMQGIAPFNGIATVGGAFNKVSAVQYGVSRMY*

>BdTR2bMPK20-5 Brdisv1BdTR2B1015380m.p

MFSDIFSREGVAFSRVALRQELLSDQWFTTAGGKGQCYLVPPGTTVFLVNSEEMGFFSEY

VDASRYKILEIIGKGSYGVVCSAIDQETGDKVAIKKIQNIFEHLSDAARILREIKLLRLL

RHPDIVQIKHIMLPPSRRDFRDIYVVFELMDTDLHQVIKANDDLTKEHYQFFLYQMLRAL

KYIHTANVYHRDLKPKNILANANCKLKICDFGLARVAFNDTPTTVFWTDYVATRWYRAPE

LCGSFFTKYSPAIDTWSIGCIFAEILTGKPLFPGKNVVHQLDLMTDLLGIPSTETISRIR

NDKARKYLSSMRRKQPIPFSEKFPNADPSALKLLERLLAFDPKDRPTAEEALAHPYFKRL

ARVEREPSCQQPISKTEFEFERRKFTKEDIKELIFREILEYHPKLLKDYMNGSEKTSFLY

PSAVDNFRRQFANLEIDGGRSGAADRKHFSLPRTTTVHSAPILPTNGPTSQVPQRIPTAR

PGRVVSSAMQTDNPSVSDRHNGRRVARDPAVPPAAAYHLKSDYSDRQHQQEFEKDRVRPD

RQRQEELEKDRMQYRPGHHSMDAKVAPEISPYMRSSPYYIPPFNGIAAVASGYSKVAAVT

RMY*

>BdTR2bMPK21-1 Brdisv1BdTR2B1015282m.p

MGGLIRWLRHPRSRRVSSSSSSHLPSNTTSSSSTSDLRAHSLPQHQGDHHGEVVVEWEDA

AEGPDSDPEEYIVVVLGDDEQGVVAARAPVRTKPPRVMDPGKKTSESEFFTEYGEANRYK

VSEVIGKGSYGVVAAAVDTQTGERCAIKKINDVFDHVSDATRILREIKLLRLLRHPDIVE

IKHIMLPPSRREFRDIYVVFELMESDLHQVIKANDDLTPEHHQFFLYQLLRGMKYIHTAN

VFHRDLKPKNILANADCKLKICDFGLARVSFNDGAPSAIFWTDYVATRWYRAPELCGSFF

SKYTPAIDIWSVGCIFAEMLTGKPLFPGKNVVHQLDLMTDLLGTPSAESLSKIRNEKARR

YLSNMRKKPKVPLTKKFPGIDPMALHLLERLLAFDPKDRPSADEALTDPYFTGLANSERE

PITQPISKLEFEFEKRKLAKDDVRELIYREILEYHPHMLQEYLRGGGDQMSFMFPSGVDR

FKRQFAHLEEGGAKGEKSSPQLRQNASLPRERVIGNKHGDGDYNMKLNVGEKPELASVSD

GISKPLMSARSLLKSETMSASKCIGEIKNKDEDSLSECVEGTDDDVSQKIAQLKT*

>BdTR2bMPK21-2 Brdisv1BdTR2B1019438m.p

MARKGSDEAEFFTEYGEANRYEVGEVVGKGSYGVVAAAVDTHTGERVAIKKINDVFEHVS

DATRILREIKLLRLLRHPDIVEIKHIILPPSRREFRDIYIIFELMESDLHQVIKANDDLT

PEHHQFFFYQLLRGMKYIHAANVFHRDLKPRNILANADCKLKICDFGLARVSFNDTPSAI

FWTDYVATRWYRAPELCGSFFSKYTPAIDIWSIGCIFAEMLSGRPLFPGKNVVHQLDLMT

DLLGTPSAESLSRIRNEKARRYLGNMRKKHPVPFSQKFPGVDPMALDLLERLLAFDPKDR

PTAAEALADPYFTGLANSDREPTTQPISKLEFEFERRKLARDDVRELIYREILEYHPQML

HEYHHGGDQANFVYPSGVDRFKRQFVHLEEGVTKGEKTSPQLRQHASLPRERIIGIGDEL

GRPNADYCIKLHVGEEPGHTSVTDGLSKPLLNARNFLKSESISASQCVVIKEKREKDEES

MSEYMHEASDATPK*

>BdTR2gMPK3 Brdisv1BdTR2G1009723m.p

MDGAPVAEFRPTMTHGGRFLLYNIFGNQFEITAKYQPPIMPIGRGAYGIVCSVMNFETRE

MVAIKKIANAFDNNMDAKRTLREIKLLRHLDHENIVGLRDVIPPAIPQSFNDVYIATELM

DTDLHHIIRSNQELSEEHCQYFLYQLLRGLKYIHSANVIHRDLKPSNLLLNANCDLKICD

FGLARPSSESDMMTEYVVTRWYRAPELLLNSTDYSAAIDVWSVGCIFMELINRAPLFPGR

DHMHQMRLITEVIGTPTDDDLGFIRNEDARRYMRHLPQFPRRPFPAQFPRVQPAALDLIE

RMLAFNPLQRITVEEALEHPYLERLHDIADEPICTDPFSFDFEQHPLTEDQMKQLIFNEA

LELNPNFRY*

>BdTR2gMPK4 Brdisv1BdTR2G1031654m.p

MATHGGRYVLYNVYGNLFEVASKYAPPIRPIGRGAYGIIIYAPRAVNRKSELLSMPCVRM

FRIHSVWCAAVSSDTGEEVAIKKIGNAFDNHIDAKRTLREIKLLRHMDHENIIAIKDIIR

PPRRDDFKDVYIVTELMDTDLHQIIRSNQPLTDDHCQYFLYQLLRGLKYVHSANVLHRDL

KPSNLFLNANCDLKIADFGLARTTSETDLMTEYVVTRWYRAPELLLNCSQYTAAIDVWSV

GCILGEIITRQPLFPGRDYIQQLKLITELIGSPDDSSLGFLRSDNARRYMKQLPQYPRQD

FRLRFRNMSDGAVDLLERMLVFDPSRRITVDEALHHPYLASLHDINEEPTCPAPFSFDFE

QPSFTEEHMKELIWRETLAFNPDPPY*

>BdTR2gMPK6 Brdisv1BdTR2G1007189m.p

MDTDLHQIIRSNQALSEEHCQYFLYQILRGLKYIHSANVLHRDLKPSNLLLNANCDLKIC

DFGLARTTSETDFMTEYVVTRWYRAPELLLNSSEYTAAIDVWSVGCIFMELMDRKPLFPG

RDHVHQLRLLMELIGTPNEADLDFVNENARRYIRQLPRHARQSFSEKFPHVHPSAIDLVE

KMLTFDPRQRITVEGALAHPYLASLHDISDEPVCTMPFSFDFEQHALSEEQMKDLIHQEA

LAFNPDYQ*

>BdTR2gMPK7-1 Brdisv1BdTR2G1044736m.p

MHTVASGISQLLRGLKYLHSAGILHRDLKPGNLLVNANCDLKICDFGLARTNNTKGQFMT

EYVVTRWYRAPELLLCCDNYGTSIDVWSVGCIFAELLGRKPIFPRTQCLDQLKLIVNVLG

TMSENDLEFIDNPKARKYIKSLPYTPGTPLTSMYPQAHPLAIDLLQKMLVFDPSKRISVT

EALEHPYMSPLYDPSANPPAQVPIDLDIDENLGVEMIREMLWQEMLQYHPEAARMVNM*

>BdTR2gMPK11 Brdisv1BdTR2G1029987m.p

MRMEGGGAGPAAAAAAGGAHGLGEAQIKGTLTHGGRYVQYNVYGNLFEVSAKYVPPIRPV

GRGACGIICAAINVQTREEVAIKKIGNAFDNQIDAKRTLREVKLLRHMNHENVISIKDII

RPPRRENFNDVYIVYELMDTDLPHLLRSNQPLTDDHCQYFLYQVLRGLKYVHSANVLHRD

LRPSNLLLNAKCDLKIGDFGLARTTTETDFMMEYVVTRWYRAPELLLNCSEYTGAIDMWS

VGCILGEIATREPLFPGKDYVHQLRLITELLGSPDDTSLGFLRSDNARRYVRSLPQYPKQ

QFRSRFPNMSSGAMDLLERMLVFDPNKRITVDEALCHPYLASLHEINDEPVCPAPFSFDF

EQPSFTEEDIKELIWRESVKFNPEPIH*

>BdTR2gMPK14 Brdisv1BdTR2G1028030m.p

MAMLVDPPNGMGNQGKHYYSMWQTLFEIDTKYVPIKPIGRGAYGIVCSSINRETNEKVAI

KKIHNVFDNRVDALRTLRELKLLRHLRHENVISLKDIMMPVQRRSFKDVYLVYELMDTDL

HQIIKSPQGLSNDHCQYFLFQLLRGLKYLHSAEILHRDLKPGNLLVNANCDLKICDFGLA

RTNSSKGQFMTEYVVTRWYRAPELLLCCDNYGTSIDVWSVGCIFAELLGRKPIFPGTECL

NQLKLIVNVLGTMSESDLEFIDNPKARRYIKTLPYTPGVPLASMYPHAHPLAIDLLQKML

IFDPTKRISVTEALEHPYMSPLYDPSANPPAQVPIDLDLDENISADMIREMMWQEMLHYH

PEAATAISM*

>BdTR2gMPK16 Brdisv1BdTR2G1016744m.p

MDFFTEYGEGNRYKIEEVIGKGSYGVVCSALDTHTGDKVAIKKINDIFEHVSDATRILRE

IKLLRLLRHPDIVEIKHILLPPSRREFKDIYVVFELMESDLHQVIKANDDLTPEHYQFFL

YQLLRGLKYIHTANVFHRDLKPKNILANADCKLKICDFGLARVAISDTPTAIFWTDYIAT

RWYRAPELCGSFFSKYTPAIDIWSIGCIFAELLTGKPLFPGKNVVHQLDIITDLLGTPSA

ETIARIRNEKARRYLSSMRRKKTVPFTQKFPNADPLALNLLERMLAFDPKDRPSAEEALA

DPYFKNIASVDREPSAQPITKLEFEFERRRITKDDIRELIYREILEYHPNMLREFLEGAE

PTGFMYPSAVDHFKKQFTFLEEHYAKGSTAAPPERQHNSLPRPSVIYSDNRPQNAANITD

DLSRCAIRDNTQKAQKDSASVGANRIPHGAAAAAARPGKVVGSVLRYGNCSTSGTEQYEQ

RRVVRSPGIAPNGVTSGSSYPRRNNTCKSETGEAERIDTNQGGPPKPYVANKLPATVDGR

NGHW*

>BdTR2gMPK17 Brdisv1BdTR2G1005338m.p

MVGGGGIVDGFRRLFHRRTPSGSGPSSNQSSAGEESSDIDAVVEDLDLVGLRAIRVPKRK

MPLPVESHKKNILEKEFFTEYGEASQYQIQEVIGKGSYGVVAAAVDTRTGERVAIKKIND

VFEHVSDATRILREVKLLRLLRHPDVVEIKHIMLPPSRREFQDIYVVFELMESDLHQVIR

ANDDLTPEHYQFFLYQLLRALKYIHAANVFHRDLKPKNILANSDCKLKICDFGLARVSFN

DAPTAIFWTDYVATRWYRAPELCGSFFSKYTPAIDIWSIGCIFAELLTGRPLFPGKNVVH

QLDIITDLLGTPSSETLSRIRNEKARRYLSCMRKKHPVPLTQKFPNVDPLALRLLGRLLA

FDPKDRPSAEEALADPYFAVLANVEREPSRNPISKLEFEFERRKVTKDDVRELIYREILE

YHPQMLQEYMQGGEQLSFLYPSGVDRFKRQFAHLEENYSKGERGSPLQRKHASLPRQRVG

ASNDNNEQHASDQERGADSIAPATGNPPGSQDAGQQHVSGGQNGVGSTNISPRSYQKSAS

ISASKCVVVNANKNPEYDDDISEEMEGAVDGLSEKVSRMHP*

>BdTR2gMPK20-1 Brdisv1BdTR2G1018048m.p

MQQDQRKKSSAEAEFFTEYGDASRYKIQEIVGKGSYGVVCSAIDVHTGEKVAIKKIHDIF

EHISDAARILREIKLLRLLRHPDIVEIKHIMLPPSRRDFKDIYVVFELMESDLHQVIKAN

DDLTKEHYQFFLYQLLRALKYIHTANVYHRDLKPKNILANSNCKLKICDFGLARVAFNDT

PTTIFWTDYVATRWYRAPELCGSFFSKYTPAIDVWSIGCIFAEVLTGKPLFPGKNVVHQL

DLMTDLLGTPSMDTISRVRNEKARRYLSSMRKKEPIPFSQKFPNADPLALDLLQRLLAFD

PKDRPTAGEALSHPYFKGLAKVEREPSCQPITKMEFEFERRRATKEDIRELIFREILEYH

PQLLKDYINGTERTTFLYPSAVDQFRKQFAHLEENSESGPVVPMDRKHTSLPRSTIVHSA

PIHAKEQPRIGPSRDRPLTDESYKNPRDSEKYSGNVPRSSHHSQAPQRVPTARPGRVVGP

VLPYENVGTSHPYDPRRVAMNSGYPPQQQIPQTYGYYQTTGKSACSEPSQAERYTLHQQA

YACANSSTAPDVALDMRAPPFHQSAGPKSGSSDRLTAETNLYTRSLNGIVATTSGVAASA

HRKGSVVPYGMSQMY*

>BdTR2gMPK20-2 Brdisv1BdTR2G1014220m.p

MQQPQQQGQRNKSTDVDFFSEYGDANRYRIQEVIGKGSYGVVCSAMDMQTRQKVAIKKIH

NIFDHVSDAARILREIKLLRLLRHPDVVEIKHIMLPPSRKDFKDIYVVFELMESDLHQVI

KANDDLTKEHYQFFLYQLLRALKYIHTASVYHRDLKPKNILANSNCKLKICDFGLARVAF

NDTPTTVFWTDYVATRWYRAPELCGSFFTKYTPAIDIWSIGCIFAEVLTGKPLFPGKNVV

HQLDLMTDLLGTPSMDTISRVRNEKARRYLSSMRKKDPVPFSKKFPNADPLGLKLLEKLL

AFDPKDRPTAEEALTDPYFKSLSKPDREPSCQPIRKVEFDFEHRRMSKDDIRELIFQEIL

EYHPQLLKNYIDGTEKTTFLYPSAVDQFKKQFSHLEESDGSGPVVPTERKHASLPRSTTV

HSTPIPAKEQPLVASSRGRPIANEPCKPWAPGNVPGASQTAHVAQAGRAVGVGSVPPYES

GSGKYPYDATSRPAVSSGYPPQQKIPQTYGYHHHHQTPGAGQSSQAMGGYACGYTKGTTP

PPAAAQDMRASPYHHRSAGTKNDPLNRLAAESDIYTRSLNGIVAAAASAGTGAHRKVGAV

PFGMSGMY*

>BdTR2gMPK20-3 Brdisv1BdTR2G1042101m.p

MQHGDLHKKSAAEMDFFTAYDDANRYKILDVIGKGSYGLVCSANDLQTGEKVAIKKIHNI

FEHISDAARILREIKLLRLLRHPDVVEIKHILLPPSKKDFKDIYVVFELMESDLHQVIKA

NDDLTREHYQFFLYQMLRALKYMHTANVYHRDLKPKNVLANANCKLKICDFGLARVAFSD

APTTVFWTDYVATRWYRAPELCGSFYSK

>BdTR2gMPK20-4 Brdisv1BdTR2G1018254m.p

MQTTEQQRKKGSSEMDFFSEYGDANRYKIQEVIGKGSYGVVCSAIDQHTGDKVAIKKIHN

IFEHLSDAARILREIKLLRLLRHPDIVEIRHIMLPPSRRDFKDIYVVFELMDTDLHQVIK

ANDDLTKEHHQFFLYQMLRALKYIHTANVYHRDLKPKNILANANCKLKICDFGLARVAFN

DTPTTVFWTDYVATRWYRAPELCGSFFTKYSRAIDIWSIGCIFAEILTGKPLFPGKNVVH

QLDLMTDLLGTPSLDTVSRIRNEKARRYLSSMRKKQSVSFSERFPKADPAALKLLQRLLA

FDPKDRPTAEEALADPYFKGLGKVERDPPCQPITKMEFEFERKNVTKADVKELIFREILE

YHPQLLKDYMNGTEKTNFLYPSAVDNFRRQFANLEENGGKGGAVVPPDRKHVSLPRNTTV

HSTPIPPKDQKYSQVPQRIPTGRPGRVVGPVIPFENSSTMDPYSQRRVARNPVLPAAATN

LSAYAYRNSDNSERELQQELEKDRMQYHPMQRFMDAKMVSPDLRSTSYYMPKGVPKADVA

ERSALQSNMMQGIAPFNGIATVGGAFNKVSAVQYGVSRMY*

>BdTR2gMPK20-5 Brdisv1BdTR2G1014352m.p

MFSDIFSREGVAFSRVALRQELLSDQWFTTAGGKGQCYLVPPGTTVFLVNSEEMGFFSEY

VDASRYKILEIIGKGSYGVVCSAIDQETGDKVAIKKIQNIFEHLSDAARILREIKLLRLL

RHPDIVQIKHIMLPPSRRDFRDIYVVFELMDTDLHQVIKANDDLTKEHYQFFLYQMLRAL

KYIHTANVYHRDLKPKNILANANCKLKICDFGLARVAFNDTPTTVFWTDYVATRWYRAPE

LCGSFFTKYSPAIDTWSIGCIFAEILTGKPLFPGKNVVHQLDLMTDLLGIPSTETISRIR

NDKARKYLSSMRRKQPIPFSEKFPNADPSALKLLERLLAFDPKDRPTAEEALAHPYFKRL

ARVEREPSCQQPISKTEFEFERRKFTKEDIKELIFREILEYHPKLLKDYMNGSEKTSFLY

PSAVDNFRRQFANLEIDGGRSGAADRKHFSLPRTTTVHSAPILPTNGPTSQVPQRIPTAR

PGRVVSSAMQTDNPSVSDRHNGRRVARDPAVPPAAAYHLKSDYSDRQHQQEFEKDRVRPD

RQRQEELEKDRMQYRPGHHSMDAKVAPEISPYMRSSPYYIPPFNGIAAVASGYSKVAAVT

RMY*

>BdTR2gMPK21-1 Brdisv1BdTR2G1014263m.p

MGGLIRWLRPHRSRRVSSSSSSHLPSNTTSSSSTSDLRAHSLPQHQGDHHGEVVVEWEDA

AEGPDSDPEEYIVVVLGDDEQGVVAARAPVRTKPPRVMDPGKKTSESEFFTEYGEANRYK

VSEVIGKGSYGVVAAAVDTQTGERCAIKKINDVFDHVSDATRILREIKLLRLLRHPDIVE

IKHIMLPPSRREFRDIYVVFELMESDLHQVIKANDDLTPEHHQFFLYQLLRGMKYIHTAN

VFHRDLKPKNILANADCKLKICDFGLARVSFNDGAPSAIFWTDYVATRWYRAPELCGSFF

SKYTPAIDIWSVGCIFAEMLTGKPLFPGKNVVHQLDLMTDLLGTPSAESLSKIRNEKARR

YLSNMRKKPKVPLTKKFPGIDPMALHLLERLLAFDPKDRPSADEALTDPYFTGLANSERE

PITQPISKLEFEFEKRKLAKDDVRELIYREILEYHPHMLQEYLRGGGDQMSFMFPSGVDR

FKRQFAHLEEGGAKGEKSSPQLRQNASLPRERVIGNKHGDGDYNMKLNVGEKPELASVSD

GISKPLMSARSLLKSETMSASKCIGEIKNKDEDSLSECVEGTDDDVSQKIAQLKT*

>BdTR2gMPK21-2 Brdisv1BdTR2G1018131m.p

MARKGSDEAEFFTEYGEANRYEVGEVVGRGSYGVVAAAVDTHTGERVAIKKINDVFEHVS

DATRILREIKLLRLLRHPDIVEIKHIILPPSRREFRDIYIIFELMESDLHQVIKANDDLT

PEHHQFFFYQLLRGMKYIHAANVFHRDLKPRNILANADCKLKICDFGLARVSFNDTPSAI

FWTDYVATRWYRAPELCGSFFSKYTPAIDIWSIGCIFAEMLSGRPLFPGKNVVHQLDLMT

DLLGTPSAESLSRIRNEKARRYLGNMRKKHPVPFSQKFPGVDPMALDLLERLLAFDPKDR

PTAAEALADPYFTGLANSDREPTTQPISKLEFEFERRKLARDDVRELIYREILEYHPQML

HEYHHGGDQANFVYPSGVDRFKRQFVHLEEGVTKGEKTSPQLRQHASLPRERIIGIGDEL

GRPNADYCIKLHVGEEPGHTSVTDGLSKPLLNARNFLKSESISASQCVVIKEKREKDEES

MSEYMHEASDATPK*

>Bd18-1MPK3 Brdisv1Bd18-11011176m.p

MDGAPVAEFRPTMTHGGRFLLYNIFGNQFEITAKYQPPIMPIGRGAYGIVCSVMNFETRE

MVAIKKIANAFDNNMDAKRTLREIKLLRHLDHENIVGLRDVIPPAIPQSFNDVYIATELM

DTDLHHIIRSNQELSEEHCQYFLYQLLRGLKYIHSANVIHRDLKPSNLLLNANCDLKICD

FGLARPSSESDMMTEYVVTRWYRAPELLLNSTDYSAAIDVWSVGCIFMELINRAPLFPGR

DHMHQMRLITEVIGTPTDDDLGFIRNEDARRYMRHLPQFPRRPFPAQFPRVQPAALDLIE

RMLAFNPLQRITVEEALEHPYLERLHDIADEPICTDPFSFDFEQHPLTEDQMKQLIFNEA

LELNPNFRY*

>Bd18-1MPK4 Brdisv1Bd18-11029432m.p

MDTSGGGGGGAGGAAQIQGMATHGGRYVLYNVYGNLFEVASKYAPPIRPIGRGAYGIVCA

AVSSDTGEEVAIKKIGNAFDNHIDAKRTLREIKLLRHMDHENIIAIKDIIRPPRRDDFKD

VYIVTELMDTDLHQIIRSNQPLTDDHCQYFLYQLLRGLKYVHSANVLHRDLKPSNLFLNA

NCDLKIADFGLARTTSETDLMTEYVVTRWYRAPELLLNCSQYTAAIDVWSVGCILGEIIT

RQPLFPGRDYIQQLKLITELIGSPDDSSLGFLRSDNARRYMKQLPQYPRQDFRLRFRNMS

DGAVDLLERMLVFDPSRRITVDEALHHPYLASLHDINEEPTCPAPFSFDFEQPSFTEEHM

KELIWRETLAFNPDPPY*

>Bd18-1MPK6 Brdisv1Bd18-11008300m.p

MDGGAQPPDAEMADAGAAVPGAGAGAAGTMENIQATLSHGGRFIQYNIFGNVFEVTVKYK

PPILPIGKGAYGIVCSALNSETGEQVAIKKIANAFDNKIDAKRTLREIKLLRHMDHENIV

AIRDIIPPAQRNSFNDVYIAYELMDTDLHQIIRSNQALSEEHCQYFLYQILRGLKYIHSA

NVLHRDLKPSNLLLNANCDLKICDFGLARTTSETDFMTEYVVTRWYRAPELLLNSSEYTA

AIDVWSVGCIFMELMDRKPLFPGRDHVHQLRLLMELIGTPNEADLDFVNENARRYIRQLP

RHARQSFSEKFPHVHPSAIDLVEKMLTFDPRQRITVEGALAHPYLASLHDISDEPVCTMP

FSFDFEQHALSEEQMKDLIHQEALAFNPDYQ*

>Bd18-1MPK7-1 Brdisv1Bd18-11006146m.p

MPSRFPFLSPGCFIRLLSGSASSDLLLLLPRRGQSAPRARVLLKMAMMVDPPNGTGNHGK

HYYTMWQTMFEIDTKYMPIKPIGRGAYGIVCSSTNQENNEKVAIKKINNVFDNRVDALRT

LRELKLLRHLRHENVIALKDIMMPVHRRSFKDVYLVSELMDTDLHQIIKSSQPLSNDHCQ

YFLFQLLRGLKYLHSAGILHRDLKPGNLLVNANCDLKICDFGLARTNNTKGQFMTEYVVT

RWYRAPELLLCCDNYGTSIDVWSVGCIFAELLGRKPIFPGTECLNQLKLIVNVLGTMSEN

DLEFIDNPKARKYIKSLPYTPGTPLTSMYPQAHPLAIDLLQKMLVFDPSKRISVTEALEH

PYMSPLYDPSANPPAQVPIDLDIDENLGVEMIREMLWQEMLQYHPEAARMVNM*

>Bd18-1MPK7-2 Brdisv1Bd18-11038744m.p

MKLSPISASFGAMGSLPQKLDELLSTGHWALSGVVMDEIEELTADLHILCNLLLKLSNVH

DPPPTVRFWMKEVRELSYDVEDCADQFVIANDRAVMRRAKIHRATIRTIIITRLKISRLP

KRRKWRLWMTDKVAEFRTRVQDATQRYWRYKFDDCASNPGYASVGHEFATVYAQPGDLVG

IEGPMDELEQWLTDGEEQLKVISIVGLGGVGKTTLAQKLWATLKGQFLCRAFVRTAQKPD

MRGILRNILLQVRPHQLPNRGEMHHLIHDLREYLQDKRYFVIIDDLWAASVWDVVSRAFP

EGNCCSRIVTTTEIMEVALACCDYCPKHICKLETLSDDDSEKLLLERIVVSGNQIPQQLD

DVLPQIMRNCGGLPLAIIIVASLLVSQPEKLEQWGHTQNSFGSIFGTNPTMEGFIRQILS

ISFNSLPYYLKTCLLYLSSYPEGCLFLKDDLVKLWVAEGFIHAKEWEDMEDLAGRYFDEL

VNVGLIQVMDINYNYKLPSYSVHHMVLDFITCKSIEENFITVVDYSETTTPLTDKVRRLS

LHFGSATHATTPASARLSNVRSLFYFGLFNCMPSFMVFKLLRVLILNFWGDPGNTSFNLT

GICELVWLRYLQVSCNVTVKLPDQIESMGHLETLEIKAKVYAVPLDIVRLSSLSHLHLRG

GTIPNGIGCMRSLRTLKYFDLVNKSVDNLRGLGELANLRDLHLTYSSSLPSEHLKRNLIA

LASSLGKLCNLKSLTLASETAATVVPFDGSISMSSTPAFLERLELLPSICIFSRLPKWIF

QFQKLCILKVAVRELLANDIDSITGLPSLTVLSLCVHTAPGQRIVFNDGAFPVLKYFKFR

CGVLSMSFMAGAMPNLRRLKLGFNTHVGEKYGNMLAGIEHLLNLQDIAGKIGVATESDRK

AAESAIKGAISKHPQSPMLNVQWVDLVEEEYHASEKQHGRQEKGLSGENHAVLEKAKDTK

KHADSGVSQLPDLPSTVSSSRLKSENIAQASKHHVLESMLCDESAQPESLEVSVLEDITN

NFSDDQEISKNRFVVVYKGELQNGSVVVVKRFTIAIDYNLFLDAVDCLMSVKHNNIVRFL

GYCANSQENAAKEGSKSVSVVTQERLCCFEYLRNGNLQMHLTDESCGFDWQMWYQIIEGI

CQGVHYLHNKSITPLDLNPANIMFDDKMVPKIVHYAYTRFLGEATSPVLTNIGDSLAYMA

PEYFGFGEITSKSDIYSLGVIIMQIVTGENKKDFPSIQSVLESWRSRLESDGSQGHTSLE

TCYLQVNLCYLIGIRCIEHDPGKRPNTRDIIDSLDTLEIMECLVKHDVGNSSLEQMLVCS

KVEEPKALPAPPSVSCKLKMAMMVDPPNGTGNHGKHYYTMWQTMFEIDTKYMPIKPIGRG

AYGIVCSSTNQENNEKVAIKKINNVFDNRVDALRTLRELKLLRHLRHENVIALKDIMMPV

HRRSFKDVYLVFELMDTDLHQIIKSSQPLSNDHCQYFLFQLLRGLKYLHSAGILHRDLKP

GNLLVNANCDLKICDFGLARTNNTKGQFMTEYVVTCLYRAPELLLCCDNYGTSIDVWSVG

CIFAELLGRKPIFPRTQCLDQLKLIVNVLGTMSENDLEFIDNPKARKYIKSLPYTPGTPL

TSMYPQAHPLAIDLLQKMLVFDPSKRISVTEALEHPYMSPLYDPSANPPAQVPIDLDIDE

NLGVEMIREMLWQEMLQYHPEAARMVNM*

>Bd18-1MPK11 Brdisv1Bd18-11027073m.p

MRMEGGGAGPAAAAAAGGAHGLGEAQIKGTLTHGGRYVQYNVYGNLFEVSAKYVPPIRPV

GRGACGIICAAINVQTREEVAIKKIGNAFDNQIDAKRTLREVKLLRHMNHENVISIKDII

RPPRRENFNDVYIVYELMDTDLHHLLRSNQPLTDDHCQYFLYQVLRGLKYVHSANVLHRD

LRPSNLLLNAKCDLKIGDFGLARTTTETDFMMEYVVTRWYRAPELLLNCSEYTGAIDMWS

VGCILGEIATREPLFPGKDYVHQLRLITELLGSPDDTSLGFLRSDNARRYVRSLPQYPKQ

QFRSRFPNMSSGAMDLLERMLVFDPNKRITVDEALCHPYLASLHEINDEPVCPAPFSFDF

EQPSFTEEDIKELIWRESVKFNPEPIH*

>Bd18-1MPK14 Brdisv1Bd18-11024761m.p

MAMLVDPPNGMGNQGKHYYSMWQTLFEIDTKYVPIKPIGRGAYGIVCSSINRETNEKVAI

KKIHNVFDNRVDALRTLRELKLLRHLRHENVISLKDIMMPVQRRSFKDVYLVYELMDTDL

HQIIKSPQGLSNDHCQYFLFQLLRGLKYLHSAEILHRDLKPGNLLVNANCDLKICDFGLA

RTNSSKGQFMTEYVVTRWYRAPELLLCCDNYGTSIDVWSVGCIFAELLGRKPIFPGTECL

NQLKLIVNVLGTMSESDLEFIDNPKARRYIKTLPYTPGVPLASMYPHAHPLAIDLLQKML

IFDPTKRISVTEALEHPYMSPLYDPSANPPAQVPIDLDLDENISADMIREMMWQEMLHYH

PEAATAISM*

>Bd18-1MPK16 Brdisv1Bd18-11019354m.p

MDFFTEYGEGNRYKIEEVIGKGSYGVVCSALDTHTGDKVAIKKINDIFEHVSDATRILRE

IKLLRLLRHPDIVEIKHILLPPSRREFKDIYVVFELMESDLHQVIKANDDLTPEHYQFFL

YQLLRGLKYIHTANVFHRDLKPKNILANADCKLKICDFGLARVAISDTPTAIFWTDYIAT

RWYRAPELCGSFFSKYTPAIDIWSIGCIFAELLTGKPLFPGKNVVHQLDIITDLLGTPSA

ETIARIRNEKARRYLSSMRRKKTVPFTQKFPNADPLALNLLERMLAFDPKDRPSAEEALA

DPYFKNIASVDREPSAQPITKLEFEFERRRITKDDIRELIYREILEYHPNMLREFLEGAE

PTGFMYPSAVDHFKKQFTFLEEHYAKGSTAAPPERQHNSLPRPSVIYSDNRPQNAANITD

DLSRCAIRDNTQKAQKDSASVGANRIPHGAAAAAARPGKVVGSVLRYGNCSTSGTEQYEQ

RRVVRSPGIAPNGVTSGSSYPRRNNTCKSETGEAERIDTNQGGPPKPYVANKLPATVDGR

NGHW*

>Bd18-1MPK17 Brdisv1Bd18-11006085m.p

MVGGGGIVDGFRRLFHRRTPSGSGPSSNQSSAGEESSDIDAVVEDLDLVGLRAIRVPKRK

MPLPVESHKKNILEKEFFTEYGEASQYQIQEVIGKGSYGVVAAAVDTRTGERVAIKKIND

VFEHVSDATRILREVKLLRLLRHPDVVEIKHIMLPPSRREFQDIYVVFELMESDLHQVIR

ANDDLTPEHYQFFLYQLLRALKYIHAANVFHRDLKPKNILANSDCKLKICDFGLARVSFN

DAPTAIFWTDYVATRWYRAPELCGSFFSKYTPAIDIWSIGCIFAELLTGRPLFPGKNVVH

QLDIITDLLGTPSSETLSRIRNEKARRYLSCMRKKHPVPLTQKFPNVDPLALRLLGRLLA

FDPKDRPSAEEALADPYFAVLANVEREPSRNPISKLEFEFERRKVTKDDVRELIYREILE

YHPQMLQEYMQGGEQLSFLYPSGVDRFKRQFAHLEENYSKGERGSPLQRKHASLPRQRVG

ASNDNNEQHASDQERGADSIAPATGNPPGSQDAGQQHVSGGQNGVGSTNISPRSYQKSAS

ISASKCVVVNANKNPEYDDDISEEMEGAVDGLSEKVSRMHP*

>Bd18-1MPK20-1 Brdisv1Bd18-11020716m.p

MQQDQRKKSSAEAEFFTEYGDASRYKIQEIVGKGSYGVVCSAIDVHTGEKVAIKKIHDIF

EHISDAARILREIKLLRLLRHPDIVEIKHIMLPPSRRDFKDIYVVFELMESDLHQVIKAN

DDLTKEHYQFFLYQLLRALKYIHTANVYHRDLKPKNILANSNCKLKICDFGLARVAFNDT

PTTIFWTDYVATRWYRAPELCGSFFSKYTPAIDVWSIGCIFAEVLTGKPLFPGKNVVHQL

DLMTDLLGTPSMDTISRVRNEKARRYLSSMRKKEPIPFSQKFPNADPLALDLLQRLLAFD

PKDRPTAGEALSHPYFKGLAKVEREPSCQPITKMEFEFERRRATKEDIRELIFREILEYH

PQLLKDYINGTERTTFLYPSAVDQFRKQFAHLEENSESGPVVPMDRKHTSLPRSTIVHSA

PIHAKEQPRIGPSRDRPLTDESYKNPRDSEKYSGNVPRSSHHSQAPQRVPTARPGRVVGP

VLPYENVGTSHPYDPRRVAMNSGYPPQQQIPQTYGYYQTTGKSACSEPSQAERYTLHQQA

YACANSSTAPDVALDMRAPPFHQSAGPKSGSSDRLTAETNLYTRSLNGIVATTSGVAASA

HRKVSVVPYGMSQMY*

>Bd18-1MPK20-2 Brdisv1Bd18-11016146m.p

MQQPQQQGQRNKSTDVDFFSEYGDANRYRIQEVIGKGSYGVVCSAMDMQTRQKVAIKKIH

NIFDHVSDAARILREIKLLRLLRHPDVVEIKHIMLPPSRKDFKDIYVVFELMESDLHQVI

KANDDLTKEHYQFFLYQLLRALKYIHTASVYHRDLKPKNILANSNCKLKICDFGLARVAF

NDTPTTVFWTDYVATRWYRAPELCGSFFTKYTPAIDIWSIGCIFAEVLTGKPLFPGKNVV

HQLDLMTDLLGTPSMDTISRVRNEKARRYLSSMRKKDPVPFSKKFPNADPLGLKLLEKLL

AFDPKDRPTAEEALTDPYFKSLSKPDREPSCQPIRKVEFDFEHRRMSKDDIRELIFQEIL

EYHPQLLKNYIDGTEKTTFLYPSAVDQFKKQFSHLEESDGSGPVVPTERKHASLPRSTTV

HSTPIPAKEQPLVASSRGRPIANEPCKPWAPGNVPGASQTAHVAQAGRAVGVGSVPPYES

GSGKYPYDATSRPAVSSGYPPQQKIPQTYGYHHHHQTPGAGQSSQAMGGYACGYTKGTTP

PPAAAQDMRASPYHHRSAGTKNDPLNRLAAESDIYTRSLNGIVAAAASAGTGAHRKVGAV

PFGMSGMY*

>Bd18-1MPK20-3 Brdisv1Bd18-11006939m.p

MQHGDLHKKSAAEMDFFTAYDDANRYKILEVIGKGSYGLVCSANDLQTGEKVAIKKIHNI

FEHISDAARILREIKLLRLLRHPDVVEIKHILLPPSKKDFKDIYVVFELMESDLHQVIKA

NDDLTREHYQFFLYQMLRALKYMHTANVYHRDLKPKNVLANANCKLKICDFGLARVAFSD

APTTVFWTDYVATRWYRAPELCGSFYSKYTPAIDIWSIGCIFAEVLIGKPLFPGKNVVHQ

LDLITDILGTPSLDAISRVRNDKARKYLTCMRKKQPASFSQKFPKADPLALQLLRRLLAF

DPKDRPSAEEALADPYFNGLAKVEREPSCQPIPKIEFEFEGRRVTKEDIKELIFEEILEY

HPQLLKEHIIGKERPNFVHLSAVDQFKKHFTQLEENDNETGAAVSLQRKHSSLPRQAFNH

R*

>Bd18-1MPK20-4 Brdisv1Bd18-11020956m.p

MQTTEQQRKKGSSEMDFFSEYGDANRYKIQEVIGKGSYGVVCSAIDQHTGDKVAIKKIHN

IFEHLSDAARILREIKLLRLLRHPDIVEIRHIMLPPSRRDFKDIYVVFELMDTDLHQVIK

ANDDLTKEHHQFFLYQMLRALKYIHTANVYHRDLKPKNILANANCKLKICDFGLARVAFN

DTPTTVFWTDYVATRWYRAPELCGSFFTKYSRAIDIWSIGCIFAEILTGKPLFPGKNVVH

QLDLMTDLLGTPSLDTVSRIRNEKARRYLSSMRKKQSVSFSERFPKADPAALKLLQRLLA

FDPKDRPTAEEALADPYFKGLGKVEREPSCQPITKMEFEFERKNVTKADVKELIFREILE

YHPQLLKDYMNGTEKTNFLYPSAVDNFRRQFANLEENGGKGGAVVPPDRKHVSLPRNTTV

HSTPIPPKDQKYSQVPQRIPTGRPGRVVGPVIPFENSSTMDPYSQRRVARNPVLPAAATN

LSAYAYRNSDNSERELQQELEKDRMQYHPMQRFMDAKMVSPDLRSTSYYMPKGVPKADVA

ERSALQSNMMQGIAPFNGIATVGGAFNKVSAVQYGVSRMY*

>Bd18-1MPK20-5 Brdisv1Bd18-11016306m.p

MFSDIFSREGVAFSRVALRQELLSDQWFTTAGGKGQCYLVPPGTTVFLVNSEEMGFFSEY

VDASRYKILEIIGKGSYGVVCSAIDQETGDKVAIKKIQNIFEHLSDAARILREIKLLRLL

RHPDIVQIKHIMLPPSRRDFRDIYVVFELMDTDLHQVIKANDDLTKEHYQFFLYQMLRAL

KYIHTANVYHRDLKPKNILANANCKLKICDFGLARVAFNDTPTTVFWTDYVATRWYRAPE

LCGSFFTKYSPAIDTWSIGCIFAEILTGKPLFPGKNVVHQLDLMTDLLGIPSTETISRIR

NDKARKYLSSMRRKQPIPFSEKFPNADPSALKLLERLLAFDPKDRPTAEEALAHPYFKRL

ARVEREPSCQQPISKTEFEFERRKFTKEDIKELIFREILEYHPKLLKDYMNGSEKTSFLY

PSAVDNFRRQFANLEIDGGRSGAADRKHFSLPRTTTVHSAPILPTNGPTSQVPQRIPTAR

PGRVVSSAMQTDNPSVSDRHNGRRVARDPAVPPAAAYHLKSDYSDRQHQQEFEKDRVRPD

RQRQEELEKDRMQYRPGHHSMDAKVAPEISPYMRSSPYYIPPFNGIAAVASGYSKVAAVT

RMY*

>Bd18-1MPK21-1 Brdisv1Bd18-11016194m.p

MGGLIRWLRHHRSRRVSSSSSSHLPSNTTSSSSTSDLRAHSLPQHQGDHHGEVVVEWEDA

AEGPDSDPEEYIVVVLGDDEQGVVAARAPVRTKPPRVMDPGKKTSESEFFTEYGEANRYK

VSEVIGKGSYGVVAAAVDTQTGERCAIKKINDVFDHVSDATRILREIKLLRLLRHPDIVE

IKHIMLPPSRREFRDIYVVFELMESDLHQVIKANDDLTPEHHQFFLYQLLRGMKYIHTAS

VFHRDLKPKNILANADCKLKICDFGLARVSFNDGAPSAIFWTDYVATRWYRAPELCGSFF

SKYTPAIDIWSVGCIFAEMLTGKPLFPGKNVVHQLDLMTDLLGTPSAESLSKIRNEKARR

YLSNMRKKPKVPLTKKFPGIDPMALHLLERLLAFDPKDRPSADEALTDPYFTGLANSERE

PITQPISKLEFEFEKRKLAKDDVRELIYREILEYHPHMLQEYLRGGGDQMSFMFPSGVDR

FKRQFAHLEEGGAKGEKSSPQLRQNASLPRERVIGNKHGDGDYNMKLNVGEKPELASVSD

GISKPLMSARSLLKSETMSASKCIGEIKNKDEDSLSECVEGTDDDVSQKIAQLKT*

>Bd18-1MPK21-2 Brdisv1Bd18-11020806m.p

MARKGSDEAEFFTEYGEANRYEVGEVVGKGSYGVVAAAVDTHTGERVAIKKINDVFEHVS

DATRILREIKLLRLLRHPDIVEIKHIILPPSRREFRDIYIIFELMESDLHQVIKANDDLT

PEHHQFFFYQLLRGMKYIHAANVFHRDLKPRNILANADCKLKICDFGLARVSFNDTPSAI

FWTDYVATRWYRAPELCGSFFSKYTPAIDIWSIGCIFAEMLSGRPLFPGKNVVHQLDLMT

DLLGTPSAESLSRIRNEKARRYLGNMRKKHPVPFSQKFPGVDPMALDLLERLLAFDPKDR

PTAAEALADPYFTGLANSDREPTTQPISKLEFEFERRKLARDDVRELIYREILEYHPQML

HEYHHGGDQANFVYPSGVDRFKRQFVHLEEGVTKGEKTSPQLRQHASLPRERIIGIGDEL

GRPNADYCIKLHVGEEPGHTSVTDGLSKPLLNARNFLKSESISASQCVVIKEKREKDEES

MSEYMHEASDATPK*

>Bd30-1MPK3 Brdisv1Bd30-11010004m.p

MDGAPVAEFRPTMTHGGRFLLYNIFGNQFEITAKYQPPIMPIGRGAYGIVCSVMNFETRE

MVAIKKIANAFDNNMDAKRTLREIKLLRHLDHENIVGLRDVIPPAIPQSFNDVYIATELM

DTDLHHIIRSNQELSEEHCQYFLYQLLRGLKYIHSANVIHRDLKPSNLLLNANCDLKICD

FGLARPSSESDMMTEYVVTRWYRAPELLLNSPDYPAAIDVWSVGCIFMELINRAPLFPGR

DHMHQMRLITEVIGTPTDDDLGFIRNEDARRYMRPLPQFPRRPFPAQFPRVQPAALDLIE

RMLAFNPLQRITVEEALEHPYLERLHDIADEPICTDPFSFDFEQHPLTEDQMKQLIFNEA

LELNPNFRY*

>Bd30-1MPK4 Brdisv1Bd30-11025462m.p

MDTSGGGGGGAGGGAQIQGMAAHGGRYVLYNVYGNLFEVASKYAPPIRPIGRGAYGIVCA

AVSSDTGEEVAIKKIGNAFDNHIDAKRTLREIKLLRHMDHENIIAIKDIIRPPRRDDFKD

VYIVTELMDTDLHQIIRSNQPLTDDHCQYFLYQLLRGLKYVHSANVLHRDLKPSNLFLNA

NCDLKIADFGLARTTSETDLMTEYVVTRWYRAPELLLNCSQYTAAIDVWSVGCILGEIIT

RQPLFPGRDYIQQLKLITELIGSPDDSSLGFLRSDNARRYMKQLPQYPRQDFRLRFRNMS

DGAVDLLERMLVFDPSRRITVDEALHHPYLASLHDINEEPTCPAPFSFDFEQPSFTEEHM

KELIWRETLAFNPDPPY*

>Bd30-1MPK6 Brdisv1Bd30-11007350m.p

MDGGAQPPDAEMADAGAAGPGAGGGGAGTMENIQATLSHGGRFIQYNIFGNVFEVTVKYK

PPILPIGKGAYGIVCSALNSETGEQVAIKKIANAFDNKIDAKRTLREIKLLRHMDHENIV

AIRDIIPPAQRNSFNDVYIAYELMDTDLHQIIRSNQALSEEHCQYFLYQILRGLKYIHSA

NVLHRDLKPSNLLLNANCDLKICDFGLARTTSETDFMTEYVVTRWYRAPELLLNSSEYTA

AIDVWSVGCIFMELMDRKPLFPGRDHVHQLRLLMELIGTPNEADLDFVNENARRYIRQLP

RHARQSFSEKFPHVHPSAIDLVEKMLTFDPRQRITVEGALAHPYLASLHDISDEPVCTMP

FSFDFEQHALSEEQMKDLIHQEALAFNPDYQ*

>Bd30-1MPK7-1 Brdisv1Bd30-11005594m.p

MPSRFPFLSPGCFIRLLSGSASSDLLLLLPRRGQSAPRARVLLKMAMMVDPPNGTGNHGK

HYYTMWQTMFEIDTKYVPIKPIGRGAYGIVCSSTNQENNEKVAIKKINNVFDNRVDALRT

LRELKLLRHLRHENVIALKDIMMPVHRRSFKDVYLVSELMDTDLHQIIKSSQPLSNDHCQ

YFLFQLLRGLKYLHSAGILHRDLKPGNLLVNANCDLKICDFGLARTNNTKGQFMTEYVVT

RWYRAPELLLCCDNYGTSIDVWSVGCIFAELLGRKPIFPGTECLNQLKLIVNVLGTMSEN

DLEFIDNPKARKYIKSLPYTPGTPLTSMYPQAHPLAIDLLQKMLVFDPSKRISVTEALEH

PYMSPLYDPSANPPAQVPIDLDIDENLGVEMIREMLWQEMLQYHPEAARMGNM*

>Bd30-1MPK11 Brdisv1Bd30-11023952m.p

MRMEGGGAGPAAAAAAGGAHGLGEAQIKGTLTHGGRDVQYNVYGNLFEVSAKYVPPIRPV

GRGACGIICAAINVQTREEVAIKKIGNAFDNQIDAKRTLREVKLLRHMNHENVISIKDII

RPPRRENFNDVYIVYELMDTDLHHLLRSNQPLTDDHCQYFLYQVLRGLKYVHSANVLHRD

LRPSNLLLNAKCDLKIGDFGLARTTTETDFMMEYVVTRWYRAPELLLNCSEYTGAIDMWS

VGCILGEIATREPLFPGKDYVHQLRLITELLGSPDDTSLGFLRSDNARRYVRSLPQYPKQ

QFRSRFPNMSSGAMDLLERMLVFDPNKRITVDEALCHPYLASLHEINDEPVCPAPFSFDF

EQPSFTEEDIKELIWRESVKFNPEPIH*

>Bd30-1MPK14 Brdisv1Bd30-11022043m.p

MAMLVDPPNGMGNQGKHYYSMWQTLFEIDTKYVPIKPIGRGAYGIVCSSINRETNEKVAI

KKIHNVFDNRVDALRTLRELKLLRHLRHENVISLKDIMMPVQRRSFKDVYLVYELMDTDL

HQIIKSPQGLSNDHCQYFLFQLLRGLKYLHSAEILHRDLKPGNLLVNANCDLKICDFGLA

RTNSSKGQFMTEYVVTRWYRAPELLLCCDNYGTSIDVWSVGCIFAELLGRKPIFPGTECL

NQLKLIVNVLGTMSESDLEFIDNPKARRYIKTLPYTPGVPLASMYPHAHPLAIDLLQKML

IFDPTKRISVTEALEHPYMSPLYDPSANPPAQVPIDLDLDENISADMIREMMWQEMLHYH

PEAATAISM*

>Bd30-1MPK16 Brdisv1Bd30-11016978m.p

MDFFTEYGEGNRYKIEEVIGKGSYGVVCSALDTHTGDKVAIKKINDIFEHVSDATRILRE

IKLLRLLRHPDIVEIKHILLPPSRREFKDIYVVFELMESDLHQVIKANDDLTPEHYQFFL

YQLLRGLKYIHTANVFHRDLKPKNILANADCKLKICDFGLARVAISDTPTAIFWTDYIAT

RWYRAPELCGSFFSKYTPAIDIWSIGWIFAELLTGKPLFPGKNVVHQLDIITDLLGTPSA

ETIARIRNEKARRYLSSMRRKKTVPFTQKFPNADPLALNLLERMLAFDPKDRPSAEEALA

DPYFKNIASVDREPSAQPITKLEFEFERRRITKDDIRELIYREILEYHPNMLREFLEGAE

PTGFMYPSAVDHFKKQFTFLEEHYAKGSTAAPPERQHNSLPRPSVIYSDNRPQNAANITD

DLSRCAIRDNTQKAQKDSASVGANRIPHGAAAAAARPGKVVGSVLRYGNCSTSGTEQYEQ

RRVVRSPGIAPNGVTSGSSYPRRNNTCKSETGEAERIDTNQGGPPKPYVANKLPATVDGR

NGHW*

>Bd30-1MPK17 Brdisv1Bd30-11005687m.p

MVGGGGIVDGFPPLFPRRTPSGSGPSSNQSSAGEESSDIDAVVEDLDLVGLRAIRVPKRK

MPLPVESHKKNILEKEFFTEYGEASQYQIQEVIGKGSYGVVAAAVDTRTGERVAIKKIND

VFEHVSDATRILREVKLLRLLRHPDVVEIKHIMLPPSRREFQDIYVVFELMESDLHQVIR

ANDDLTPEHYQFFLYQLLRALKYIHAANVFHRDLKPKNILANSDCKLKICDFGLARVSFN

DAPTAIFWTDYVATRWYRAPELCGSFFSKYTPAIDIWSIGCIFAELLTGRPLFPGKNVVH

QLDIITDLLGTPSSETLSRIRNEKARRYLSCMRKKHPVPLTQKFPNVDPLALRLLGRLLA

FDPKDRPSAEEALADPYFAVLANVEREPSRNPISKLEFEFERRKVTKDDVRELIYREILE

YHPQMLQEYMQGGEQLSFLYPSGVDRFKRQFAHLEENYSKGERGSPLQRKHASLPRQRVG

ASNDNNEQHASDQERGADSIAPATGNPPGSQDAGQQHVSGGQNGVGSTNISPRSYQKSAS

ISASKCVVVNANKNPEYDDDISEEMEGAVDGLSEKVSRMHP*

>Bd30-1MPK20-1 Brdisv1Bd30-11018292m.p

MQQDQRKKSSAEAEFFTEYGDASRYKIQEIVGKGSYGVVCSAIDVHTGEKVAIKKIHDIF

EHISDAARILREIKLLRLLRHPDIVEIKHIMLPPSRRDFKDIYVVFELMESDLHQVIKAN

DDLTKEHYQFFLYQLLRALKYIHTANVYHRDLKPKNILANSNCKLKICDFGLARVAFNDT

PTTIFWTDYVATRWYRAPELCGSFFSKYTPAIDVWSIGCIFAEVLTGKPLFPGKNVVHQL

DLMTDLLGTPSMDTISRVRNEKARRYLSSMRKKEPIPFSQKFPNADPLALDLLQRLLAFD

PKDRPTAGEALSHPYFKGLAKVEREPSCQPITKMEFEFERRRATKEDIRELIFREILEYH

PQLLKDYINGTERTTFLYPSAVDQFRKQFAHLEENSESGPVVPMDRKHTSLPRSTIVHSA

PIHAKEQPRIGPSRDRPLTDESYKNPRDSEKYSGNVPRSSHHSQAPQRVPTARPGRVVGP

VLPYENVGTSHPYDPRRVAMNSGYPPQQQIPQTYGYYQTTGKSACSEPSQAERYTLHQQA

YACANSSTAPDVALDMRAPPFHQSAGPKSGSSDRLTAETNLYTRSLNGIVATTSGVAGSA

HRKVSVVPYGMSQMY*

>Bd30-1MPK20-2 Brdisv1Bd30-11014518m.p

MQQPQQQGQRNKSTDVDFFSEYGDANRYRIQEVIGKGSYGVVCSAMDMQTRQKVAIKKIH

NIFDHVSDAARILREIKLLRLLRHPDVVEIKHIMLPPSRKDFKDIYVVFELMESDLHQVI

KANDDLTKEHYQFFLYQLLRALKYIHTASVYHRDLKPKNILANSNCKLKICDFGLARVAF

NDTPTTVFWTDYVATRWYRAPELCGSFFTKYTPAIDIWSIGCIFAEVLTGKPLFPGKNVV

HQLDLMTDLLGTPSMDTISRVRNEKARRYLSSMRKKDPVPFSKKFPNADPLGLKLLEKLL

AFDPKDRPTAEEALTDPYFKSLSKPDREPSCQPIRKVEFDFEHRRMSKDDIRELIFQEIL

EYHPQLLKNYIDGTEKTTFLYPSAVDQFKKQFSHLEESDGSGPVVQTERKHASLPRSTTV

HSTPIPAKEQPLVASSRGRPIANEPCKPWAPGNVPGASQTAHVAQAGRAVGVGSVPPYES

GSGKYPYDATSRPAVSSGYPPQQKIPQTYGYHHHHQTPGAGQSSQAMGGYACGYTKGTTP

PPAAAQDMRASPYHHRSAGTKNDPLNRLAAESDIYTRSLNGIVAAAASAGTGAHRKVGAV

PFGMSGMY*

>Bd30-1MPK20-3 Brdisv1Bd30-11006265m.p

MQHGDLHKKSAAEMDFFTAYDDANRYKILEVIGKGSYGLVCSANDLQTGEKVAIKKIHNI

FEHISDAARILREIKLLRLLRHPDVVEIKHILLPPSKKDFKDIYVVFELMESDLHQVIKA

NDDLTREHYQFFLYQMLRALKYMHTANVYHRDLKPKNVLANANCKLKICDFGLARVAFSD

APTTVFWTDYVATRWYRAPELCGSFYSKYTPAIDIWSIGCIFAEVLIGKPLFPGKNVVHQ

LDLITDILGTPSLDAISRVRNDKARKYLTCMRKKQPASFSQKFPKADPLALQLLRRLLAF

DPKDRPSAEEALADPYFNGLAKVEREPSCQPIPKIEFEFEGRRVTKEDIKELIFEEILEY

HPQLLKEHIIGKERPNFVHLSAVDQFKKHFTQLEENDNETGAAVSLQRKHSSLPRQAFNH

R*

>Bd30-1MPK20-4 Brdisv1Bd30-11018520m.p

MDFFSEYGDANRYKIQEVIGKGSYGVVCSAIDQHTGDKVAIKKIHNIFEHLSDAARILRE

IKLLRLLRHPDIVEIRHIMLPPSRRDFKDIYVVFELMDTDLHQVIKANDDLTKEHHQFFL

YQMLRALKYIHTANVYHRDLKPKNILANANCKLKICDFGLARVAFNDTPTTVFWTDYVAT

RWYRAPELCGSFFTKYSRAIDIWSIGCIFAEILTGKPLFPGKNVVHQLDLMTDLLGTPSL

DTVSRIRNEKARRYLSSMRKKQSVSFSERFPKADPAALKLLQRLLAFDPKDRPTAEEALA

DPYFKGLGKVEREPPCQPITKMEFEFERKNVTKADVKELIFREILEYHPQLLKDYMNGTE

KTNFLYPSAVDNFRRQFANLEENGGKGGAVVPPDRKHVSLPRNTTVHSTPIPPKDQKYSQ

VPQRIPTGRPGRVVGPVIPFENSSTMDPYSQRRGARNPVLPAAATNLSAYAYRNSDNSER

ELQQELEKDRMQYHPMQRFMDAKMVSPDLRSTSYYMPKGVPKADVAERSALQSNMMQGIA

PFNGIATVGGAFNKVSAVQYGVSRMY*

>Bd30-1MPK20-5 Brdisv1Bd30-11008828m.p

MFSDIFSREGVAFSRVALRQELLSDQWFTTAGGKGQCYLVPPGTTVFLVNSEEMGFFSEY

VDASRYKILEIIGKGSYGVVCSAIDQETGDKVAIKKIQNIFEHLSDAARILREIKLLRLL

RHPDIVQIKHIMLPPSRRDFRDIYVVFELMDTDLHQVIKANDDLTKEHYQFFLYQMLRAL

KYIHTANVYHRDLKPKNILANANCKLKICDFGLARVAFNDTPTTVFWTDYVATRWYRAPE

LCGSFFTKYSPAIDTWSIGCIFAEILTGKPLFPGKNVVHQLDLMTDLLGTPSTETISRIR

NDKARKYLSSMRRKQPIPFSEKFPNADPSALKLLERLLAFDPKDRPTAEEALAHPYFKRL

ARVEREPSCQQPISKTEFEFERRKFTKEDIKELIFREILEYHPKLLKDYMNGSEKTSFLY

PSAVDNFRRQFANLEIDGGRSGAADRKHFSLPRTTTVHSAPILPTNGPTSQVPQRIPTAR

PGRVVSSAMQTDNPSVSDRHNGRRVARDPAVPPAAAYHLKSDYSDRQHQQEFEKDRVRPD

RQRQEELEKDRMQYRPGHHSMDAKVAPEISPYMRSSPYYIPPFNGIAAVASGYSKVAAVT

RMY*

>Bd30-1MPK21-1 Brdisv1Bd30-11014587m.p

MGGLIRWLRPPRPRRVSSSSSSHLPSNTTSSSTSDLRAHSLPQHQGDHHGEGVVEWEDAA

EGPDSDPEEYIVVVLGDDEQGVVAARAPGRTKPPRVMDPGKKTSESEFFTEYGEANRYKV

SEVIGKGSYGVVAAAVDTQTGERCAIKKINDVFDHVSDATRILREIKLLRLLRHPDIVEI

KHIMLPPSRREFRDIYVVFELMESDLHQVIKANDDLTPEHHQFFLYQLLRGMKYIHTANV

FHRDLKPKNILANADCKLKICDFGLARVSFNDGAPSAIFWTDYVATRWYRAPELCGSFFS

KYTPAIDIWSVGCIFAEMLTGKPLFPGKNVVHQLDLMTDLLGTPSAESLSKIRNEKARRY

LSNMRKKPKVPLTKKFPGIDPMALHLLERLLAFDPKDRPSADEALTDPYFTGLANSEREP

ITQPISKLEFEFEKRKLAKDDVRELIYREILEYHPHMLQEYLRGGGDQMSFMFPSGVDRF

KRQFAHLEEGGAKGEKSSPQLRQNASLPRERVIGNKHGDGDYNMKLNVGEKPELASVSDG

ISKPLMSARSLLKSETMSASKCIGEIKNKDEDSLSECVEGTDDDVSQKIAQLKT*

>Bd30-1MPK21-2 Brdisv1Bd30-11018351m.p

MLPPSRREFRDIYIIFELMESDLHQVIKANDDLTPEHHQFFFYQLLRGMKYIHAANVFHR

DLKPRNILANADCKLKICDFGLARVSFNDTPSAIFWTDYVATRWYRAPELCGSFFSKYTP

AIDIWSIGCIFAEMLSGRPLFPGKNVVHQLDLMTDLLGTPSAESLSRIRNEKARRYLGNM

RKKHPVPFSQKFPGVDPMALDLLERLLAFDPKDRPTAAEALADPYFTGLANSDREPTTQP

ISKLEFEFERRKLARDDVRELIYREILEYHPQMLHEYHHGGDQANFVYPSGVDRFKRQFV

HLEEGVTKGEKTSPQLRQHASLPRERIIGIGDELGRPNADYCIKLHVGEEPGHTSVTDGL

RKPLLNARNFLKSESISASQCVCHQREARKRCKCCYIKYILNQIVSKIIN*

>ABR7MPK3 Brdisv1ABR71010863m.p

MDGAPVAEFRPTMTHGGRFLLYNIFGNQFEITAKYQPPIMPIGRGAYGIVCSVMNFETRE

MVAIKKIANAFDNNMDAKRTLREIKLLRHLDHENIVGLRDVIPPAIPQSFNDVYIATELM

DTDLHHIIRSNQELSEEHCQYFLYQLLRGLKYIHSANVIHRDLKPSNLLLNANCDLKICD

FGLARPSSESDMMTEYVVTRWYRAPELLLNSTDYSAAIDVWSVGCIFMELINRAPLFPGR

DHMHQMRLITEVIGTPTDDDLGFIRNEDARRYMRHLPQFPRRPFPAQFPRVQPAALDLIE

RMLAFNPLQRITVEEALEHPYLERLHDIADEPICTDPFSFDFEQHPLTEDQMKQLIFNEA

LELNPNFRY*

>ABR7MPK4 Brdisv1ABR71035794m.p

MDTSGGGGGGAGGAAQIQGMATHGGRYVLYNVYGNLFEVASKYAPPIRPIGRGAYGIVCA

AVSSDTGEEVAIKKIGNAFDNHIDAKRTLREIKLLRHMDHENIIAIKDIIRPPRRDDFKD

VYIVTELMDTDLHQIIRSNQPLTDDHCQYFLYQLLRGLKYVHSANVLHRDLKPSNLFLNA

NCDLKIADFGLARTTSETDLMTEYVVTRWYRAPELLLNCSQYTAAIDVWSVGCILGEIIT

RQPLFPGRDYIQQLKLITELIGSPDDSSLGFLRSDNARRYMKQLPQYPRQDFRLRFRNMS

DGAVDLLERMLVFDPSRRITVDEALHHPYLASLHDINEEPTCPAPFSFDFEQPSFTEEHM

KELIWRETLAFNPDPPY*

>ABR7MPK6 Brdisv1ABR71007957m.p

IVAIRDIIPPAQRNSFNDVYIAYELMDTDLHQIIRSNQALSEEHCQYFLYQILRGLKYIH

SANVLHRDLKPSNLLLNANCDLKICDFGLARTTSETDFMTEYVVTRWYRAPELLLNSSEY

TAAIDVWSVGCIFMELMDRKPLFPGRDHVHQLRLLMELIGTPNEADLDFVNENARRYIRQ

LPRHARQSFSEKFPHVHPSAIDLVEKMLTFDPRQRITVEGALAHPYLASLHDISDEPVCT

MPFSFDFEQHALSEEQMKDLIHQEALAFNPDYQ*

>ABR7MPK11 Brdisv1ABR71033630m.p

MRMEGGGAGPAAAAAAGGAHGLGEAQIKGTLTHGGRYVQYNVYGNLFEVSAKYVPPIRPV

GRGACGIICAAINVQTREEVAIKKIGNAFDNQIDAKRTLREVKLLRHMNHENVISIKDII

RPPRRENFNDVYIVYELMDTDLHHLLRSNQPLTDDHCQYFLYQVLRGLKYVHSANVLHRD

LRPSNLLLNAKCDLKIGDFGLARTTTETDFMMEYVVTRWYRAPELLLNCSEYTGAIDMWS

VGCILGEIATREPLFPGKDYVHQLRLITELLGSPDDTSLGFLRSDNARRYVRSLPQYPKQ

QFRSRFPNMSSGAMDLLERMLVFDPNKRITVDEALCHPYLASLHEINDEPVCPAPFSFDF

EQPSFTEEDIKELIWRESVKFNPEPIH*

>ABR7MPK14 Brdisv1ABR71031394m.p

MAMLVDPPNGMGNQGKHYYSMWQTLFEIDTKYVPIKPIGRGAYGIVCSSINRETNEKVAI

KKIHNVFDNRVDALRTLRELKLLRHLRHENVISLKDIMMPVQRRSFKDVYLVYELMDTDL

HQIIKSPQGLSNDHCQYFLFQLLRGLKYLHSAEILHRDLKPGNLLVNANCDLKICDFGLA

RTNSSKGQFMTEYVVTRWYRAPELLLCCDNYGTSIDVWSVGCIFAELLGRKPIFPGTECL

NQLKLIVNVLGTMSESDLEFIDNPKARRYIKTLPYTPGVPLASMYPHAHPLAIDLLQKML

IFDPTKRISVTEALEHPYMSPLYDPSANPPAQVPIDLDLDENISADMIREMMWQEMLHYH

PEAATAISM*

>ABR7MPK16 Brdisv1ABR71018675m.p

MDFFTEYGEGNRYKIEEVIGKGSYGVVCSALDTHTGDKVAIKKINDIFEHVSDATRILRE

IKLLRLLRHPDIVEIKHILLPPSRREFKDIYVVFELMESDLHQVIKANDDLTPEHYQFFL

YQLLRGLKYIHTANVFHRDLKPKNILANADCKLKICDFGLARVAISDTPTAIFWTDYIAT

RWYRAPELCGSFFSKYTPAIDIWSIGCIFAELLTGKPLFPGKNVVHQLDIITDLLGTPSA

ETIARIRNEKARRYLSSMRRKKTVPFTQKFPNADPLALNLLERMLAFDPKDRPSAEEALA

DPYFKNIASVDREPSAQPITKLEFEFERRRITKDDIRELIYREILEYHPNMLREFLEGAE

PTGFMYPSAVDHFKKQFTFLEEHYAKGSTAAPPERQHNSLPRPSVIYSDNRPQNAANITD

DLSRCAIRDNTQKAQKDSASVGANRIPHGAAAAAARPGKVVGSVLRYGNCSTSGTEQYEQ

RRVVRSPGIAPNGVTSGSSYPRRNNTCKSETGEAERIDTNQGGPPKPYVANKLPATVDGR

NGHW*

>ABR7MPK17 Brdisv1ABR71005821m.p

MVGGGGIVDGFRRLFHRRTPSGSGPSSNQSSAGEESSDIDAVVEDLDLVGLRAIRVPKRK

MPLPVESHKKNILEKEFFTEYGEASQYQIQEVIGKGSYGVVAAAVDTRTGERVAIKKIND

VFEHVSDATRILREVKLLRLLRHPDVVEIKHIMLPPSRREFQDIYVVFELMESDLHQVIR

ANDDLTPEHYQFFLYQLLRALKYIHAANVFHRDLKPKNILANSDCKLKICDFGLARVSFN

DAPTAIFWTDYVATRWYRAPELCGSFFSKYTPAIDIWSIGCIFAELLTGRPLFPGKNVVH

QLDIITDLLGTPSSETLSRIRNEKARRYLSCMRKKHPVPLTQKFPNVDPLALRLLGRLLA

FDPKDRPSAEEALADPYFAVLANVEREPSRNPISKLEFEFERRKVTKDDVRELIYREILE

YHPQMLQEYMQGGEQLSFLYPSGVDRFKRQFAHLEENYSKGERGSPLQRKHASLPRQRVG

ASNDNNEQHASDQERGADSIAPATGNPPGSQDAGQQHVSGGQNGVGSTNISPRSYQKSAS

ISASKCVVVNANKNPEYDDDISEEMEGAVDGLSEKVSRMHP*

>ABR7MPK20-1 Brdisv1ABR71020178m.p

MQQDQRKKSSAEAEFFTEYGDASRYKIQEIVGKGSYGVVCSAIDVHTGEKVAIKKIHDIF

EHISDAARILREIKLLRLLRHPDIVEIKHIMLPPSRRDFKDIYVVFELMESDLHQVIKAN

DDLTKEHYQFFLYQLLRALKYIHTANVYHRDLKPKNILANSNCKLKICDFGLARVAFNDT

PTTIFWTDYVATRWYRAPELCGSFFSKYTPAIDVWSIGCIFAEVLTGKPLFPGKNVVHQL

DLMTDLLGTPSMDTISRVRNEKARRYLSSMRKKEPIPFSQKFPNADPLALDLLQRLLAFD

PKDRPTAGEALSHPYFKGLAKVEREPSCQPITKMEFEFERRRATKEDIRELIFREILEYH

PQLLKDYINGTERTTFLYPSAVDQFRKQFAHLEENSESGPVVPMDRKHTSLPRSTIVHSA

PIHAKEQPRIGPSRDRPLTDESYKNPRDSEKYSGNVPRSSHHSQAPQRVPTARPGRVVGP

VLPYENVGTSHPYDPRRVAMNSGYPPQQQIPQTYGYYQTTGKSACSEPSQAERYTLHQQA

YACANSSTAPDVALDMRAPPFHQSAGPKSGSSDRLTAETNLYTRSLNGIVATTSGVAASA

HRKVSVVPYGMSQMY*

>ABR7MPK20-2 Brdisv1ABR71015792m.p

MQQPQQQGQRNKSTDVDFFSEYGDANRYRIQEVIGKGSYGVVCSAMDMQTRQKVAIKKIH

NIFDHVSDAARILREIKLLRLLRHPDVVEIKHIMLPPSRKDFKDIYVVFELMESDLHQVI

KANDDLTKEHYQFFLYQLLRALKYIHTASVYHRDLKPKNILANSNCKLKICDFGLARVAF

NDTPTTVFWTDYVATRWYRAPELCGSFFTKYTPAIDIWSIGCIFAEVLTGKPLFPGKNVV

HQLDLMTDLLGTPSMDTISRVRNEKARRYLSSMRKKDPVPFSKKFPNADPLGLKLLEKLL

AFDPKDRPTAEEALTDPYFKSLSKPDREPSCQPIRKVEFDFEHRRMSKDDIRELIFQEIL

EYHPQLLKNYIDGTEKTTFLYPSAVDQFKKQFSHLEESDGSGPVVPTERKHASLPRSTTV

HSTPIPAKEQPLVASSRGRPIANEPCKPWAPGNVPGASQTAHVAQAGRAVGVGSVPPYES

GSGKYPYDATSRPAVSSGYPPQQKIPQTYGYHHHHQTPGAGQSSQAMGGYACGYTKGTTP

PPAAAQDMRASPYHHRSAGTKNDPLNRLAAESDIYTRSLNGIVAAAASAGTGAHRKVGAV

PFGMSGMY*

>ABR7MPK20-3 Brdisv1ABR71006668m.p

MQHGDLHKKSAAEMDFFTAYDDANRYKILEVIGKGSYGLVCSANDLQTGEKVAIKKIHNI

FEHISDAARILREIKLLRLLRHPDVVEIKHILLPPSKKDFKDIYVVFELMESDLHQVIKA

NDDLTREHYQFFLYQMLRALKYMHTANVYHRDLKPKNVLANANCKLKICDFGLARVAFSD

APTTVFWTDYVATRWYRAPELCGSFYSKYTPAIDIWSIGCIFAEVLIGKPLFPGKNVVHQ

LDLITDILGTPSLDAISRVRNDKARKYLTCMRKKQPASFSQKFPKADPLALQLLRRLLAF

DPKDRPSAEEALADPYFNGLAKVEREPSCQPIPKIEFEFEGRRVTKEDIKELIFEEILEY

HPQLLKEHIIGKERPNFVHLSAVDQFKKHFTQLEENDNETGAAVSLQRKHSSLPRQAFNH

R*

>ABR7MPK20-4 Brdisv1ABR71020433m.p

MQTTEQQRKKGSSEMDFFSEYGDANRYKIQEVIGKGSYGVVCSAIDQHTGDKVAIKKIHN

IFEHLSDAARILREIKLLRLLRHPDIVEIRHIMLPPSRRDFKDIYVVFELMDTDLHQVIK

ANDDLTKEHHQFFLYQMLRALKYIHTANVYHRDLKPKNILANANCKLKICDFGLARVAFN

DTPTTVFWTDYVATRWYRAPELCGSFFTKYSRAIDIWSIGCIFAEILTGKPLFPGKNVVH

QLDLMTDLLGTPSLDTVSRIRNEKARRYLSSMRKKQSVSFSERFPKADPAALKLLQRLLA

FDPKDRPTAEEALADPYFKGLGKVEREPSCQPITKMEFEFERKNVTKADVKELIFREILE

YHPQLLKDYMNGTEKTNFLYPSAVDNFRRQFANLEENGGKGGAVVPPDRKHVSLPRNTTV

HSTPIPPKDQKYSQVPQRIPTGRPGRVVGPVIPFENSSTMDPYSQRRVARNPVLPAAATN

LSAYAYRNSDNSERELQQELEKDRMQYHPMQRFMDAKMVSPDLRSTSYYMPKGVPKADVA

ERSALQSNMMQGIAPFNGIATVGGAFNKVSAVQYGVSRMY*

>ABR7MPK20-5 Brdisv1ABR71015944m.p

MFSDIFSREGVAFSRVALRQELLSDQWFTTAGGKGQCYLVPPGTTVFLVNSEEMGFFSEY

VDASRYKILEIIGKGSYGVVCSAIDQETGDKVAIKKIQNIFEHLSDAARILREIKLLRLL

RHPDIVQIKHIMLPPSRRDFRDIYVVFELMDTDLHQVIKANDDLTKEHYQFFLYQMLRAL

KYIHTANVYHRDLKPKNILANANCKLKICDFGLARVAFNDTPTTVFWTDYVATRWYRAPE

LCGSFFTKYSPAIDTWSIGCIFAEILTGKPLFPGKNVVHQLDLMTDLLGTPSTETISRIR

NDKARKYLSSMRRKQPIPFSEKFPNADPSALKLLERLLAFDPKDRPTAEEALAHPYFKRL

ARVEREPSCQQPISKTEFEFERRKFTKEDIKELIFREILEYHPKLLKDYMNGSEKTSFLY

PSAVDNFRRQFANLEIDGGRSGAADRKHFSLPRTTTVHSAPILPTNGPTSQVPQRIPTAR

PGRVVSSAMQTDNPSVSDRHNGRRVARDPAVPPAAAYHLKSDYSDRPHQQEFEKDRVRPD

RQRQEELEKDRMQYRPGHHSMDAKVAPEISPYMRSSPYYIPPFNGIAAVASGYSKVAAVT

RMY*

>ABR7MPK21-1 Brdisv1ABR71015840m.p

MGGLIRWLRHHRSRRVSSSSSSHLPSNTTSSSTSDLRAHSLPQHQGDHHGEVVVEWEDAA

EGPDSDPEEYIVVVLGDDEQGVVAARAPVRTKPPRVMDPGKKTSESEFFTEYGEANRYKV

SEVIGKGSYGVVAAAVDTQTGERCAIKKINDVFDHVSDATRILREIKLLRLLRHPDIVEI

KHIMLPPSRREFRDIYVVFELMESDLHQVIKANDDLTPEHHQFFLYQLLRGMKYIHTANV

FHRDLKPKNILANADCKLKICDFGLARVSFNDGAPSAIFWTDYVATRWYRAPELCGSFFS

KYTPAIDIWSVGCIFAEMLTGKPLFPGKNVVHQLDLMTDLLGTPSAESLSKIRNEKARRY

LSNMRKKPKVPLTKKFPGIDPMALHLLERLLAFDPKDRPSADEALTDPYFTGLANSEREP

ITQPISKLEFEFEKRKLAKDDVRELIYREILEYHPHMLQEYLRGGGDQMSFMFPSGVDRF

KRQFAHLEEGGAKGEKSSPQLRQNASLPRERVIGNKHGDGDYNMKLNVGEKPELASVSDG

ISKPLMSARSLLKSETMSASKCIGEIKNKDEDSLSECVEGTDDDVSQKIAQLKT*

>ABR7MPK21-2 Brdisv1ABR71020266m.p

GSDEAEFFTEYGEANRYEVGEVVGKGSYGVVAAAVDTHTGERVAIKKINDVFEHVSDATR

ILREIKLLRLLRHPDIVEIKHIMLPPSRREFRDIYIIFELMESDLHQVIKANDDLTPEHH

QFFFYQLLRGMKYIHAANVFHRDLKPRNILANADCKLKICDFGLARVSFNDTPSAIFWTD

YVATRWYRAPELCGSFFSKYTPAIDIWSIGCIFAEMLSGRPLFPGKNVVHQLDLMTDLLG

TPSAESLSRIRNEKARRYLGNMRKKHPVPFSQKFPGVDPMALDLLERLLAFDPKDRPTAA

EALADPYFTGLANSDREPTTQPISKLEFEFERRKLARDDVRELIYREILEYHPQMLHEYH

HGGDQANFVYPSGVDRFKRQFVHLEEGVTKGEKTSPQLRQHASLPRERIIGIGDELGRPN

ADYCIKLHVGEEPGHTSVTDGLSKPLLNARNFLKSESISASQCVVIKEKREKDVSAVTSN

IF*

>ABR2MPK3 Brdisv1ABR21010920m.p

MDGAPVAEFRPTMTHGGRFLLYNIFGNQFEITAKYQPPIMPIGRGAYGIVCSVMNFETRE

MVAIKKIANAFDNNMDAKRTLREIKLLRHLDHENIVGLRDVIPPAIPQSFNDVYIATELM

DTDLHHIIRSNQELSEEHCQYFLYQLLRGLKYIHSANVIHRDLKPSNLLLNANCDLKICD

FGLARPSSESDMMTEYVVTRWYRAPELLLNSTDYSAAIDVWSVGCIFMELINRAPLFPGR

DHMHQMRLITEVIGTPTDDDLGFIRNEDARRYMRHLPQFPRRPFPAQFPRVQPAALDLIE

RMLAFNPLQRITVEEALEHPYLERLHDIADEPICTDPFSFDFEQHPLTEDQMKQLIFNEA

LELNPNFRY*

>ABR2MPK4 Brdisv1ABR21011722m.p

MDTSGGGGGGAGGAAQIQGMATHGGRYVLYNVYGNLFEVASKYAPPIRPIGRGAYGIVCA

AVSSDTGEEVAIKKIGNAFDNHIDAKRTLREIKLLRHMDHENIIAIKDIIRPPRRDDFKD

VYIVTELMDTDLHQIIRSNQPLTDDHCQYFLYQLLRGLKYVHSANVLHRDLKPSNLFLNA

NCDLKIADFGLARTTSETDLMTEYVVTRWYRAPELLLNCSQYTAAIDVWSVGCILGEIIT

RQPLFPGRDYIQQLKLITELIGSPDDSSLGFLRSDNARRYMKQLPQYPRQDFRLRFRNMS

DGAVDLLERMLVFDPSRRITVDEALHHPYLASLHDINEEPTCPAPFSFDFEQPSFTEEHM

KELIWRETLAFNPDPPY*

>ABR2MPK6 Brdisv1ABR21008022m.p

MDGGAQPPDAEMADAGAAVPGAGAGAAGTMENIQATLSHGGRFIQYNIFGNVFEVTVKYK

PPILPIGKGAYGIVCSALNSETGEQVAIKKIANAFDNKIDAKRTLREIKLLRHMDHENIV

AIRDIIPPAQRNSFNDVYIAYELMDTDLHQIIRSNQALSEEHCQYFLYQILRGLKYIHSA

NVLHRDLKPSNLLLNANCDLKICDFGLARTTSETDFMTEYVVTRWYRAPELLLNSSEYTA

AIDVWSVGCIFMELMDRKPLFPGRDHVHQLRLLMELIGTPNEADLDFVNENARRYIRQLP

RHARQSFSEKFPHVHPSAIDLVEKMLTFDPRQRITVEGALAHPYLASLHDISDEPVCTMP

FSFDFEQHALSEEQMKDLIHQEALAFNPDYQ*

>ABR2MPK11 Brdisv1ABR21033560m.p

MRMEGGGAGPAAAAAAGGAHGLGEAQIKGTLTHGGRYVQYNVYGNLFEVSAKYVPPIRPV

GRGACGIICAAINVQTREEVAIKKIGNAFDNQIDAKRTLREVKLLRHMNHENVISIKDII

RPPRRENFNDVYIVYELMDTDLHHLLRSNQPLTDDHCQYFLYQVLRGLKYVHSANVLHRD

LRPSNLLLNAKCDLKIGDFGLARTTTETDFMMEYVVTRWYRAPELLLNCSEYTGAIDMWS

VGCILGEIATREPLFPGKDYVHQLRLITELLGSPDDTSLGFLRSDNARRYVRSLPQYPKQ

QFRSRFPNMSSGAMDLLERMLVFDPNKRITVDEALCHPYLASLHEINDEPVCPAPFSFDF

EQPSFTEEDIKELIWRESVKFNPEPIH*

>ABR2MPK14 Brdisv1ABR21031419m.p

MAMLVDPPNGMGNQGKHYYSMWQTLFEIDTKYVPIKPIGRGAYGIVCSSINRETNEKVAI

KKIHNVFDNRVDALRTLRELKLLRHLRHENVISLKDIMMPVQRRSFKDVYLVYELMDTDL

HQIIKSPQGLSNDHCQYFLFQLLRGLKYLHSAEILHRDLKPGNLLVNANCDLKICDFGLA

RTNSSKGQFMTEYVVTRWYRAPELLLCCDNYGTSIDVWSVGCIFAELLGRKPIFPGTECL

NQLKLIVNVLGTMSESDLEFIDNPKARRYIKTLPYTPGVPLASMYPHAHPLAIDLLQKML

IFDPTKRISVTEALEHPYMSPLYDPSANPPAQVPIDLDLDENISADMIREMMWQEMLHYH

PEAATAISM*

>ABR2MPK16 Brdisv1ABR21018581m.p

MDFFTEYGEGNRYKIEEVIGKGSYGVVCSALDTHTGDKVAIKKINDIFEHVSDATRILRE

IKLLRLLRHPDIVEIKHILLPPSRREFKDIYVVFELMESDLHQVIKANDDLTPEHYQFFL

YQLLRGLKYIHTANVFHRDLKPKNILANADCKLKICDFGLARVAISDTPTAIFWTDYIAT

RWYRAPELCGSFFSKYTPAIDIWSIGCIFAELLTGKPLFPGKNVVHQLDIITDLLGTPSA

ETIARIRNEKARRYLSSMRRKKTVPFTQKFPNADPLALNLLERMLAFDPKDRPSAEEALA

DPYFKNIASVDREPSAQPITKLEFEFERRRITKDDIRELIYREILEYHPNMLREFLEGAE

PTGFMYPSAVDHFKKQFTFLEEHYAKGSTAAPPERQHNSLPRPSVIYSDNRPQNAANITD

DLSRCAIRDNTQKAQKDSASVGANRIPHGAAAAAARPGKVVGSVLRYGNCSTSGTEQYEQ

RRVVRSPGIAPNGVTSGSSYPRRNNTCKSETGEAERIDTNQGGPPKPYVANKLPATVDGR

NGHW*

>ABR2MPK17 Brdisv1ABR21005927m.p

MVGGGGIVDGFRRLFHRRTPSGSGPSSNQSSAGEESSDIDAVVEDLDLVGLRAIRVPKRK

MPLPVESHKKNILEKEFFTEYGEASQYQIQEVIGKGSYGVVAAAVDTRTGERVAIKKIND

VFEHVSDATRILREVKLLRLLRHPDVVEIKHIMLPPSRREFQDIYVVFELMESDLHQVIR

ANDDLTPEHYQFFLYQLLRALKYIHAANVFHRDLKPKNILANSDCKLKICDFGLARVSFN

DAPTAIFWTDYVATRWYRAPELCGSFFSKYTPAIDIWSIGCIFAELLTGRPLFPGKNVVH

QLDIITDLLGTPSSETLSRIRNEKARRYLSCMRKKHPVPLTQKFPNVDPLALRLLGRLLA

FDPKDRPSAEEALADPYFAVLANVEREPSRNPISKLEFEFERRKVTKDDVRELIYREILE

YHPQMLQEYMQGGEQLSFLYPSGVDRFKRQFAHLEENYSKGERGSPLQRKHASLPRQRVG

ASNDNNEQHASDQERGADSIAPATGNPPGSQDAGQQHVSGGQNGVGSTNISPRSYQKSAS

ISASKCVVVNANKNPEYDDDISEEMEGAVDGLSEKVSRMHP*

>ABR2MPK20-1 Brdisv1ABR21020048m.p

MQQDQRKKSSAEAEFFTEYGDASRYKIQEIVGKGSYGVVCSAIDVHTGEKVAIKKIHDIF

EHISDAARILREIKLLRLLRHPDIVEIKHIMLPPSRRDFKDIYVVFELMESDLHQVIKAN

DDLTKEHYQFFLYQLLRALKYIHTANVYHRDLKPKNILANSNCKLKICDFGLARVAFNDT

PTTIFWTDYVATRWYRAPELCGSFFSKYTPAIDVWSIGCIFAEVLTGKPLFPGKNVVHQL

DLMTDLLGTPSMDTISRVRNEKARRYLSSMRKKEPIPFSQKFPNADPLALDLLQRLLAFD

PKDRPTAGEALSHPYFKGLAKVEREPSCQPITKMEFEFERRRATKEDIRELIFREILEYH

PQLLKDYINGTERTTFLYPSAVDQFRKQFAHLEENSESGPVVPMDRKHTSLPRSTIVHSA

PIHAKEQPRIGPSRDRPLTDESYKNPRDSEKYSGNVPRSSHHSQAPQRVPTARPGRVVGP

VLPYENVGTSHPYDPRRVAMNSGYPPQQQIPQTYGYYQTTGKSACSEPSQAERYTLHQQA

YACANSSTAPDVALDMRAPPFHQSAGPKSGSSDRLTAETNLYTRSLNGIVATTSGVAASA

HRKVSVVPYGMSQMY*

>ABR2MPK20-2 Brdisv1ABR21015746m.p

MQQPQQQGQRNKSTDVDFFSEYGDANRYRIQEVIGKGSYGVVCSAMDMQTRQKVAIKKIH

NIFDHVSDAARILREIKLLRLLRHPDVVEIKHIMLPPSRKDFKDIYVVFELMESDLHQVI

KANDDLTKEHYQFFLYQLLRALKYIHTASVYHRDLKPKNILANSNCKLKICDFGLARVAF

NDTPTTVFWTDYVATRWYRAPELCGSFFTKYTPAIDIWSIGCIFAEVLTGKPLFPGKNVV

HQLDLMTDLLGTPSMDTISRVRNEKARRYLSSMRKKDPVPFSKKFPNADPLGLKLLEKLL

AFDPKDRPTAEEALTDPYFKSLSKPDREPSCQPIRKVEFDFEHRRMSKDDIRELIFQEIL

EYHPQLLKNYIDGTEKTTFLYPSAVDQFKKQFSHLEESDGSGPVVQTERKHASLPRSTTV

HSTPIPAKEQPLVASSRGRPIANEPCKPWAPGNVPGASQTAHVAQAGRAVGVGSVPPYES

GSGKYPYDATSRPAVSSGYPPQQKIPQTYGYHHHHQTPGAGQSSQAMGGYACGYTKGTTP

PPAAAQDMRASPYHHRSAGTKNDPLNRLAAESDIYTRSLNGIVAAAASAGTGAHRKVGAV

PFGMSGMY*

>ABR2MPK20-3 Brdisv1ABR21029756m.p

MQHGDLHKKSAAEMDFFTAYDDANRYKILEVIGKGSYGLVCSANDLQTGEKVAIKKIHNI

FEHISDAARILREIKLLRLLRHPDVVEIKHILLPPSKKDFKDIYVVFELMESDLHQVIKA

NDDLTREHYQFFLYQMLRALKYMHTANVYHRDLKPKNVLANANCKLKICDFGLARVAFSD

APTTVFWTDYVATRWYRAPELCGSFYSKYTPAIDIWSIGCIFAEVLIGKPLFPGKNVVHQ

LDLITDILGTPSLDAISRVRNDKARKYLTCMRKKQPASFSQKFPKADPLALQLLRRLLAF

DPKDRPSAEEALADPYFNGLAKVEREPSCQPIPKIEFEFEGRRVTKEDIKELIFEEILEY

HPQLLKEHIIGKERPNFVHLSAVDQFKKHFTQLEENDNETGAAVSLQRKHSSLPRQAFNH

R*

>ABR2MPK20-4 Brdisv1ABR21020278m.p

MQTTEQQRKKGSSEMDFFSEYGDANRYKIQEVIGKGSYGVVCSAIDQHTGDKVAIKKIHN

IFEHLSDAARILREIKLLRLLRHPDIVEIRHIMLPPSRRDFKDIYVVFELMDTDLHQVIK

ANDDLTKEHHQFFLYQMLRALKYIHTANVYHRDLKPKNILANANCKLKICDFGLARVAFN

DTPTTVFWTDYVATRWYRAPELCGSFFTKYSRAIDIWSIGCIFAEILTGKPLFPGKNVVH

QLDLMTDLLGTPSLDTVSRIRNEKARRYLSSMRKKQSVSFSERFPKADPAALKLLQRLLA

FDPKDRPTAEEALADPYFKGLGKVEREPSCQPITKMEFEFERKNVTKADVKELIFREILE

YHPQLLKDYMNGTEKTNFLYPSAVDNFRRQFANLEENGGKGGAVVPPDRKHVSLPRNTTV

HSTPIPPKDQKYSQVPQRIPTGRPGRVVGPVIPFENSSTMDPYSQRRVARNPVLPAAATN

LSAYAYRNSDNSERELQQELEKDRMQYHPMQRFMDAKMVSPDLRSTSYYMPKGVPKADVA

ERSALQSNMMQGIAPFNGIATVGGAFNKVSAVQYGVSRMY*

>ABR2MPK20-5 Brdisv1ABR21015895m.p

MFSDIFSREGVAFSRVALRQELLSDQWFTTAGGKGQCYLVPPGTTVFLVNSEEMGFFSEY

VDASRYKILEIIGKGSYGVVCSAIDQETGDKVAIKKIQNIFEHLSDAARILREIKLLRLL

RHPDIVQIKHIMLPPSRRDFRDIYVVFELMDTDLHQVIKANDDLTKEHYQFFLYQMLRAL

KYIHTANVYHRDLKPKNILANANCKLKICDFGLARVAFNDTPTTVFWTDYVATRWYRAPE

LCGSFFTKYSPAIDTWSIGCIFAEILTGKPLFPGKNVVHQLDLMTDLLGTPSTETISRIR

NDKARKYLSSMRRKQPIPFSEKFPNADPSALKLLERLLAFDPKDRPTAEEALAHPYFKRL

ARVEREPSCQQPISKTEFEFERRKFTKEDIKELIFREILEYHPKLLKDYMNGSEKTSFLY

PSAVDNFRRQFANLEIDGGRSGAADRKHFSLPRTTTVHSAPILPTNGPTSQVPQRIPTAR

PGRVVSSAMQTDNPSVSDRHNGRRVARDPAVPPAAAYHLKSDYSDRQHQQEFEKDRVRPD

RQRQEELEKDRMQYRPGHHSMDAKVAPEISPYMRSSPYYIPPFNGIAAVASGYSKVAAVT

RMY*

>ABR2MPK21-1 Brdisv1ABR21015793m.p

MGGLIRWLRHHRSRRVSSSSSSHLPSNTTSSSTSDLRAHSLPQHQGDHHGEVVVEWEDAA

EGPDSDPEEYIVVVLGDDEQGVVAARAPVRTKPPRVMDPGKKTSESEFFTEYGEANRYKV

SEVIGKGSYGVVAAAVDTQTGERCAIKKINDVFDHVSDATRILREIKLLRLLRHPDIVEI

KHIMLPPSRREFRDIYVVFELMESDLHQVIKANDDLTPEHHQFFLYQLLRGMKYIHTANV

FHRDLKPKNILANADCKLKICDFGLARVSFNDGAPSAIFWTDYVATRWYRAPELCGSFFS

KYTPAIDIWSVGCIFAEMLTGKPLFPGKNVVHQLDLMTDLLGTPSAESLSKIRNEKARRY

LSNMRKKPKVPLTKKFPGIDPMALHLLERLLAFDPKDRPSADEALTDPYFTGLANSEREP

ITQPISKLEFEFEKRKLAKDDVRELIYREILEYHPHMLQEYLRGGGDQMSFMFPSGVDRF

KRQFAHLEEGGAKGEKSSPQLRQNASLPRERVIGNKHGDGDYNMKLNVGEKPELASVSDG

ISKPLMSARSLLKSETMSASKCIGEIKNKDEDSLSECVEGTDDDVSQKIAQLKT*

>ABR2MPK21-2 Brdisv1ABR21020133m.p

GSDEAEFFTEYGEANRYEVGEVVGKGSYGVVAAAVDTHTGERVAIKKINDVFEHVSDATR

ILREIKLLRLLRHPDIVEIKHIMLPPSRREFRDIYIIFELMESDLHQVIKANDDLTPEHH

QFFFYQLLRGMKYIHAANVFHRDLKPRNILANADCKLKICDFGLARVSFNDTPSAIFWTD

YVATRWYRAPELCGSFFSKYTPAIDIWSIGCIFAEMLSGRPLFPGKNVVHQLDLMTDLLG

TPSAESLSRIRNEKARRYLGNMRKKHPVPFSQKFPGVDPMALDLLERLLAFDPKDRPTAA

EALADPYFTGLANSDREPTTQPISKLEFEFERRKLARDDVRELIYREILEYHPQMLHEYH

HGGDQANFVYPSGVDRFKRQFVHLEEGVTKGEKTSPQLRQHASLPRERIIGIGDELGRPN

ADYCIKLHVGEEPGHTSVTDGLSKPLLNARNFLKSESISASQCVVIKEKREKDVSAVTSN

IF*

>Mig3MPK3 Brdisv1Mig31010710m.p

MDGAPVAEFRPTMTHGGRFLLYNIFGNQFEITAKYQPPIMPIGRGAYGIVCSVMNFETRE

MVAIKKIANAFDNNMDAKRTLREIKLLRHLDHENIVGLRDVIPPAIPQSFNDVYIATELM

DTDLHHIIRSNQELSEEHCQYFLYQLLRGLKYIHSANVIHRDLKPSNLLLNANCDLKICD

FGLARPSSESDMMTEYVVTRWYRAPELLLNSTDYSAAIDVWSVGCIFMELINRAPLFPGR

DHMHQMRLITEVIGTPTDDDLGFIRNEDARRYMRHLPQFPRRPFPAQFPRVQPAALDLIE

RMLAFNPLQRITVEEALEHPYLERLHDIADEPICTDPFSFDFEQHPLTEDQMKQLIFNEA

LELNPNFRY*

>Mig3MPK4 Brdisv1Mig31011481m.p

MDTSGGGGGGAGGAAQIQGMATHGGRYVLYNVYGNLFEVASKYAPPIRPIGRGAYGIVCA

AVSSDTGEEVAIKKIGNAFDNHIDAKRTLREIKLLRHMDHENIIAIKDIIRPPRRDDFKD

VYIVTELMDTDLHQIIRSNQPLTDDHCQYFLYQLLRGLKYVHSANVLHRDLKPSNLFLNA

NCDLKIADFGLARTTSETDLMTEYVVTRWYRAPELLLNCSQYTAAIDVWSVGCILGEIIT

RQPLFPGRDYIQQLKLITELIGSPDDSSLGFLRSDNARRYMKQLPQYPRQDFRLRFRNMS

DGAVDLLERMLVFDPSRRITVDEALHHPYLASLHDINEEPTCPAPFSFDFEQPSFTEEHM

KELIWRETLAFNPDPPY*

>Mig3MPK6 Brdisv1Mig31007919m.p

MTGDQTLASMTNYGRFSQIETAYWLVSYNKQSVSAFNVPVIVAIRDIIPPAQRNSFNDVY

IAYELMDTDLHQIIRSNQALSEEHCQYFLYQILRGLKYIHSANVLHRDLKPSNLLLNANC

DLKICDFGLARTTSETDFMTEYVVTRWYRAPELLLNSSEYTAAIDVWSVGCIFMELMDRK

PLFPGRDHVHQLRLLMELIGTPNEADLDFVNENARRYIRQLPRHARQSFSEKFPHVHPSA

IDLVEKMLTFDPRQRITVEGALAHPYLASLHDISDEPVCTMPFSFDFEQHALSEEQMKDL

IHQEALAFNPDYQ*

>Mig3MPK7-1 Brdisv1Mig31046286m.p

LLRGLKYLHSAGILHRDLKPGNLLVNANCDLKICDFGLARTNNTKGQFMTEYVVTRWYRA

PELLLCCDNYGTSIDVWSVGCIFAELLGRKPIFPRTQCLDQLKLIVNVLGTMSENDLEFI

DNPKARKYIKSLPYTPGTPLTSMYPQAHPLAIDLLQKMLVFDPSKRISVTEALEHPYMSP

LYDPSANPPAQVPIDLDIDENLGVEMIREMLWQEMLQYHPEAARMVNM*

>Mig3MPK11 Brdisv1Mig31032921m.p

MRMEGGGAGPAAAAAAGGAHGLGEAQIKGTLTHGGRYVQYNVYGNLFEVSAKYVPPIRPV

GRGACGIICAAINVQTREEVAIKKIGNAFDNQIDAKRTLREVKLLRHMNHENVISIKDII

RPPRRENFNDVYIVYELMDTDLHHLLRSNQPLTDDHCQYFLYQVLRGLKYVHSANVLHRD

LRPSNLLLNAKCDLKIGDFGLARTTTETDFMMEYVVTRWYRAPELLLNCSEYTGAIDMWS

VGCILGEIATREPLFPGKDYVHQLRLITELLGSPDDTSLGFLRSDNARRYVRSLPQYPKQ

QFRSRFPNMSSGAMDLLERMLVFDPNKRITVDEALCHPYLASLHEINDEPVCPAPFSFDF

EQPSFTEEDIKELIWRESVKFNPEPIH*

>Mig3MPK14 Brdisv1Mig31030748m.p

MAMLVDPPNGMGNQGKHYYSMWQTLFEIDTKYVPIKPIGRGAYGIVCSSINRETNEKVAI

KKIHNVFDNCVDALRTLRELKLLRHLRHENVISLKDIMMPVQRRSFKDVYLVYELMDTDL

HQIIKSPQGLSNDHCQYFLFQLLRGLKYLHSAEILHRDLKPGNLLVNANCDLKICDFGLA

RTNSSKGQFMTEYVVTRWYRAPELLLCCDNYGTSIDVWSVGCIFAELLGRKPIFPGTECL

NQLKLIVNVLGTMSESDLEFIDNPKARRYIKTLPYTPGVPLASMYPHAHPLAIDLLQKML

IFDPTKRISVTEALEHPYMSPLYDPSANPPAQVPIDLDLDENISADMIREMMWQEMLHYH

PEAATAISM*

>Mig3MPK16 Brdisv1Mig31044690m.p

MDFFTEYGEGNRYKIEEVIGKGSYGVVCSALDTHTGDKVAIKKINDIFEHVSDATRILRE

IKLLRLLRHPDIVEIKHILLPPSRREFKDIYVVFELMESDLHQVIKANDDLTPEHYQFFL

YQLLRGLKYIHTANVFHRDLKPKNILANADCKLKICDFGLARVAISDTPTAIFWTDYIAT

RWYRAPELCGSFFSKYTPAIDIWSIGCIFAELLTGKPLFPGKNVVHQLDIITDLLGTPSA

ETIARIRNEKARRYLSSMRRKKTVPFTQKFPNADPLALNLLERMLAFDPKDRPSAEEALA

DPYFKNIASVDREPSAQPITKLEFEFERRRITKDDIRELIYREILEYHPNMLREFLEGAE

PTGFMYPSAVDHFKKQFTFLEEHYAKGSTAAPPERQHNSLPRPSVIYSDNRPQNAANITD

DLSRCAIRDNTQKAQKDSASVGANRIPHGAAAAATRPGKVVGSVLRYGNCSTSGTEQYEQ

RRVVRSPGIAPNGVTSGSSYPRRNNTCKSETGEAERIDTNQGGPPKPYVANKLPATVDGR

NGHW*

>Mig3MPK17 Brdisv1Mig31005860m.p

MVGGGGIVDGFRRLFPRRTPSGSGPSSNQSSAGEESSDIDAVVEDLDLVGLRAIRVPKRK

MPLPVESHKKNILEKEFFTEYGEASQYQIQEVIGKGSYGVVAAAVDTRTGERVAIKKIND

VFEHVSDATRILREVKLLRLLRHPDVVEIKHIMLPPSRREFQDIYVVFELMESDLHQVIR

ANDDLTPEHYQFFLYQLLRALKYIHAANVFHRDLKPKNILANSDCKLKICDFGLARVSFN

DAPTAIFWTDYVATRWYRAPELCGSFFSKYTPAIDIWSIGCIFAELLTGRPLFPGKNVVH

QLDIITDLLGTPSSETLSRIRNEKARRYLSCMRKKHPVPLTQKFPNVDPLALRLLGRLLA

FDPKDRPSAEEALADPYFAVLANVEREPSRNPISKLEFEFERRKVTKDDVRELIYREILE

YHPQMLQEYMQGGEQLSFLYPSGVDRFKRQFAHLEENYSKGERGSPLQRKHASLPRQRVG

ASNDNNEQHASDQERGADSIAPATGNPPGSQDAGQQHVSGGQNGVGSTNISPRSYQKSAS

ISASKCVVVNANKNPEYDDDISEEMEGAVDGLSEKVSRMHP*

>Mig3MPK20-1 Brdisv1Mig31019799m.p

MQQDQRKKSSAEAEFFTEYGDASRYKIQEIVGKGSYGVVCSAIDVHTGEKVAIKKIHDIF

EHISDAARILREIKLLRLLRHPDIVEIKHIMLPPSRRDFKDIYVVFELMESDLHQVIKAN

DDLTKEHYQFFLYQLLRALKYIHTANVYHRDLKPKNILANSNCKLKICDFGLARVAFNDT

PTTIFWTDYVATRWYRAPELCGSFFSKYTPAIDVWSIGCIFAEVLTGKPLFPGKNVVHQL

DLMTDLLGTPSMDTISRVRNEKARRYLSSMRKKEPIPFSQKFPNADPLALDLLQRLLAFD

PKDRPTAGEALSHPYFKGLAKVEREPSCQPITKMEFEFERRRATKEDIRELIFREILEYH

PQLLKDYINGTERTTFLYPSAVDQFRKQFAHLEENSESGPVVPMDRKHTSLPRSTIVHSA

PIHAKEQPRIGPSRDRPLTDESYKNPRDSEKYSGNVPRSSHHSQAPQRVPTARPGRVVGP

VLPYENVGTSHPYDPRRVAMNSGYPPQQQIPQTYGYYQTTGKSACSEPSQAERYTLHQQA

YACANSSTAPDVALDMRAPPFHQSAGPKSGSSDRLTAETNLYTRSLNGIVATTSGVAASA

HRKVSVVPYGMSQMY*

>Mig3MPK20-2 Brdisv1Mig31015536m.p

MQQPQQQGQRNKSTDVDFFSEYGDANRYRIQEVIGKGSYGVVCSAMDMQTRQKVAIKKIH

NIFDHVSDAARILREIKLLRLLRHPDVVEIKHIMLPPSRKDFKDIYVVFELMESDLHQVI

KANDDLTKEHYQFFLYQLLRALKYIHTASVYHRDLKPKNILANSNCKLKICDFGLARVAF

NDTPTTVFWTDYVATRWYRAPELCGSFFTKYTPAIDIWSIGCIFAEVLTGKPLFPGKNVV

HQLDLMTDLLGTPSMDTISRVRNEKARRYLSSMRKKDPVPFSKKFPNADPLGLKLLEKLL

AFDPKDRPTAEEALTDPYFKSLSKPDREPSCQPIRKVEFDFEHRRMSKDDIRELIFQEIL

EYHPQLLKNYIDGTEKTTFLYPSAVDQFKKQFSHLEESDGSGPVVQTERKHASLPRSTTV

HSTPIPAKEQPLVASSRGRPIANEPCKPWAPGNVPGASQTAHVAQAGRAVGVGSVPPYES

GSGKYPYDATSRPAVSSGYPPQQKIPQTYGYHHHHQTPGAGQSSQAMGGYACGYTKGTTP

PPAAAQDMRASPYHHRSAGTKNDPLNRLAAESDIYTRSLNGIVAAAASAGTGAHRKVGAV

PFGMSGMY*

>Mig3MPK20-3 Brdisv1Mig31006628m.p

MQHGDLHKKSAAEMDFFTAYDDANRYKILEVIGKGSYGLVCSANDLQTGEKVAIKKIHNI

FEHISDAARILREIKLLRLLRHPDVVEIKHILLPPSKKDFKDIYVVFELMESDLHQVIKA

NDDLTREHYQFFLYQMLRALKYMHTANVYHRDLKPKNVLANANCKLKICDFGLARVAFSD

APTTVFWTDYVATRWYRAPELCGSFYSKYTPAIDIWSIGCIFAEVLIGKPLFPGKNVVHQ

LDLITDILGTPSLDAISRVRNDKARKYLTCMRKKQPASFSQKFPKADPLALQLLRRLLAF

DPKDRPSAEEALADPYFNGLAKVEREPSCQPIPKIEFEFEGRRVTKEDIKELIFEEILEY

HPQLLKEHIIGKERPNFVHLSAVDQFKKHFTQLEENDNETGAAVSLQRKHSSLPRQAFNH

R*

>Mig3MPK20-4 Brdisv1Mig31020024m.p

MQTTEQQRKKGSSEMDFFSEYGDANRYKIQEVIGKGSYGVVCSAIDQHTGDKVAIKKIHN

IFEHLSDAARILREIKLLRLLRHPDIVEIRHIMLPPSRRDFKDIYVVFELMDTDLHQVIK

ANDDLTKEHHQFFLYQMLRALKYIHTANVYHRDLKPKNILANANCKLKICDFGLARVAFN

DTPTTVFWTDYVATRWYRAPELCGSFFTKYSRAIDIWSIGCIFAEILTGKPLFPGKNVVH

QLDLMTDLLGTPSLDTVSRIRNEKARRYLSSMRKKQSVSFSERFPKADPAALKLLQRLLA

FDPKDRPTAEEALADPYFKGLGKVEREPSCQPITKMEFEFERKNVTKADVKELIFREILE

YHPQLLKDYMNGTEKTNFLYPSAVDNFRRQFANLEENGGKGGAVVPPDRKHVSLPRNTTV

HSTPIPPKDQKYSQVPQRIPTGRPGRVVGPVIPFENSSTMDPYSQRRVARNPVLPAAATN

LSAYAYRNSDNSERELQQELEKDRMQYHPMQRFMDAKMVSPDLRSTSYYMPKGVPKADVA

ERSALQSNMMQGIAPFNGIATVGGAFNKVSAVQYGVSRMY*

>Mig3MPK20-5 Brdisv1Mig31015671m.p

MFSDIFSREGVAFSRVALRQELLSDQWFTTAGGKGQCYLVPPGTTVFLVNSEEMGFFSEY

VDASRYKILEIIGKGSYGVVCSAIDQETGDKVAIKKIQNIFEHLSDAARILREIKLLRLL

RHPDIVQIKHIMLPPSRRDFRDIYVVFELMDTDLHQVIKANDDLTKEHYQFFLYQMLRAL

KYIHTANVYHRDLKPKNILANANCKLKICDFGLARVAFNDTPTTVFWTDYVATRWYRAPE

LCGSFFTKYSPAIDTWSIGCIFAEILTGKPLFPGKNVVHQLDLMTDLLGTPSTETISRIR

NDKARKYLSSMRRKQPIPFSEKFPNADPSALKLLERLLAFDPKDRPTAEEALAHPYFKRL

ARVEREPSCQQPISKTEFEFERRKFTKEDIKELIFREILEYHPKLLKDYMNGSEKTSFLY

PSAVDNFRRQFANLEIDGGRSGAADRKHFSLPRTTTVHSAPILPTNGPTSQVPQRIPTAR

PGRVVSSAMQTDNPSVSDRHNGRRVARDPAVPPAAAYHLKSDYSDRQHQQEFEKDRVRPD

RQRQEELEKDRMQYRPGHHSMDAKVAPEISPYMRSSPYYIPPFNGIAAVASGYSKVAAVT

RMY*

>Mig3MPK21-1 Brdisv1Mig31015571m.p

MGGLIRWLRHHRSRRVSSSSSSHLPSNTTSSSTSDLRAHSLPQHQGDHHGEVVVEWEDAA

EGPDSDPEEYIVVVLGDDEQGVVAARAPVRTKPPRVMDPGKKTSESEFFTEYGEANRYKV

SEVIGKGSYGVVAAAVDTQTGERCAIKKINDVFDHVSDATRILREIKLLRLLRHPDIVEI

KHIMLPPSRREFRDIYVVFELMESDLHQVIKANDDLTPEHHQFFLYQLLRGMKYIHTANV

FHRDLKPKNILANADCKLKICDFGLARVSFNDGAPSAIFWTDYVATRWYRAPELCGSFFS

KYTPAIDIWSVGCIFAEMLTGKPLFPGKNVVHQLDLMTDLLGTPSAESLSKIRNEKARRY

LSNMRKKPKVPLTKKFPGIDPMALHLLERLLAFDPKDRPSADEALTDPYFTGLANSEREP

ITQPISKLEFEFEKRKLAKDDVRELIYREILEYHPHMLQEYLRGGGDQMSFMFPSGVDRF

KRQFAHLEEGGAKGEKSSPQLRQNASLPRERVIGNKHGDGDYNMKLNVGEKPELASVSDG

ISKPLMSARSLLKSETMSASKCIGEIKNKDEDSLSECVEGTDDDVSQKIAQLKT*

>Mig3MPK21-2 Brdisv1Mig31019888m.p

GSDEAEFFTEYGEANRYEVGEVVGKGSYGVVAAAVDTHTGERVAIKKINDVFEHVSDATR

ILREIKLLRLLRHPDIVEIKHIMLPPSRREFRDIYIIFELMESDLHQVIKANDDLTPEHH

QFFFYQLLRGMKYIHAANVFHRDLKPRNILANADCKLKICDFGLARVSFNDTPSAIFWTD

YVATRWYRAPELCGSFFSKYTPAIDIWSIGCIFAEMLSGRPLFPGKNVVHQLDLMTDLLG

TPSAESLSRIRNEKARRYLGNMRKKHPVPFSQKFPGVDPMALDLLERLLAFDPKDRPTAA

EALADPYFTGLANSDREPTTQPISKLEFEFERRKLARDDVRELIYREILEYHPQMLHEYH

HGGDQANFVYPSGVDRFKRQFVHLEEGVTKGEKTSPQLRQHASLPRERIIGIGDELGRPN

ADYCIKLHVGEEPGHTSVTDGLSKPLLNARNFLKSESISASQCVVIKEKREKDVSAVTSN

IF*

>Uni2MPK3 Brdisv1Uni21010201m.p

MDGAPVAEFRPTMTHGGRFLLYNIFGNQFEITAKYQPPIMPIGRGAYGIVCSVMNFETRE

MVAIKKIANAFDNNMDAKRTLREIKLLRHLDHENIVGLRDVIPPAIPQSFNDVYIATELM

DTDLHHIIRSNQELSEEHCQYFLYQLLRGLKYIHSANVIHRDLKPSNLLLNANCDLKICD

FGLARPSSESDMMTEYVVTRWYRAPELLLNSTDYSAAIDVWSVGCIFMELINRAPLFPGR

DHMHQMRLITEVIGTPTDDDLGFIRNEDARRYMRHLPQFPRRPFPAQFPRVQPAALDLIE

RMLAFNPLQRITVEEALEHPYLERLHDIADEPICTDPFSFDFEQHPLTEDQMKQLIFNEA

LELNPNFRY*

>Uni2MPK4 Brdisv1Uni21033435m.p

MDTSGGGGGGAGGAAQIQGMATHGGRYVLYNVYGNLFEVASKYAPPIRPIGRGAYGIVCA

AVSSDTGEEVAIKKIGNAFDNHIDAKRTLREIKLLRHMDHENIIAIKDIIRPPRRDDFKD

VYIVTELMDTDLHQIIRSNQPLTDDHCQYFLYQLLRGLKYVHSANVLHRDLKPSNLFLNA

NCDLKIADFGLARTTSETDLMTEYVVTRWYRAPELLLNCSQYTAAIDVWSVGCILGEIIT

RQPLFPGRDYIQQLKLITELIGSPDDSSLGFLRSDNARRYMKQLPQYPRQDFRLRFRNMS

DGAVDLLERMLVFDPSRRITVDEALHHPYLASLHDINEEPTCPAPFSFDFEQPSFTEEHM

KELIWRETLAFNPDPPY*

>Uni2MPK6 Brdisv1Uni21007433m.p

MDGGAQPPDAEMADAGAAVPGAGAGAAGTMENIQATLSHGGRFIQYNIFGNVFEVTVKYK

PPILPIGKGAYGIVCSALNSETGEQVAIKKIANAFDNKIDAKRTLREIKLLRHMDHENIV

AIRDIIPPAQRNSFNDVYIAYELMDTDLHQIIRSNQALSEEHCQYFLYQILRGLKYIHSA

NVLHRDLKPSNLLLNANCDLKICDFGLARTTSETDFMTEYVVTRWYRAPELLLNSSEYTA

AIDVWSVGCIFMELMDRKPLFPGRDHVHQLRLLMELIGTPNEADLDFVNENARRYIRQLP

RHARQSFSEKFPHVHPSAIDLVEKMLTFDPRQRITVEGALAHPYLASLHDISDEPVCTMP

FSFDFEQHALSEEQMKDLIHQEALAFNPDYQ*

>Uni2MPK7-1 Brdisv1Uni21045875m.p

LLRGLKYLHSAGILHRDLKPGNLLVNANCDLKICDFGLARTNNTKGQFMTEYVVTRWYRA

PELLLCCDNYGTSIDVWSVGCIFAELLGRKPIFPRTQCLDQLKLIVNVLGTMSENDLEFI

DNPKARKYIKSLPYTPGTPLTSMYPQAHPLAIDLLQKMLVFDPSKRISVTEALEHPYMSP

LYDPSANPPAQVPIDLDIDENLGVEMIREMLWQEMLQYHPEAARMVNM*

>Uni2MPK11 Brdisv1Uni21031613m.p

MRMEGGGAGPAAAAAAGGAHGLGEAQIKGTLTHGGRYVQYNVYGNLFEVSAKYVPPIRPV

GRGACGIICAAINVQTREEVAIKKIGNAFDNQIDAKRTLREVKLLRHMNHENVISIKDII

RPPRRENFNDVYIVYELMDTDLHHLLRSNQPLTDDHCQYFLYQVLRGLKYVHSANVLHRD

LRPSNLLLNAKCDLKIGDFGLARTTTETDFMMEYVVTRWYRAPELLLNCSEYTGAIDMWS

VGCILGEIATREPLFPGKDYVHQLRLITELLGSPDDTSLGFLRSDNARRYVRSLPQYPKQ

QFRSRFPNMSSGAMDLLERMLVFDPNKRITVDEALCHPYLASLHEINDEPVCPAPFSFDF

EQPSFTEEDIKELIWRESVKFNPEPIH*

>Uni2MPK14 Brdisv1Uni21029534m.p

MAMLVDPPNGMGNQGKHYYSMWQTLFEIDTKYVPIKPIGRGAYGIVCSSINRETNEKVAI

KKIHNVFDNRVDALRTLRELKLLRHLRHENVISLKDIMMPVQRRSFKDVYLVYELMDTDL

HQIIKSPQGLSNDHCQYFLFQLLRGLKYLHSAEILHRDLKPGNLLVNANCDLKICDFGLA

RTNSSKGQFMTEYVVTRWYRAPELLLCCDNYGTSIDVWSVGCIFAELLGRKPIFPGTECL

NQLKLIVNVLGTMSESDLEFIDNPKARRYIKTLPYTPGVPLASMYPHAHPLAIDLLQKML

IFDPTKRISVTEALEHPYMSPLYDPSANPPAQVPIDLDLDENISADMIREMMWQEMLHYH

PEAATAISM*

>Uni2MPK16 Brdisv1Uni21017694m.p

MDFFTEYGEGNRYKIEEVIGKGSYGVVCSALDTHTGDKVAIKKINDIFEHVSDATRILRE

IKLLRLLRHPDIVEIKHILLPPSRREFKDIYVVFELMESDLHQVIKANDDLTPEHYQFFL

YQLLRGLKYIHTANVFHRDLKPKNILANADCKLKICDFGLARVAISDTPTAIFWTDYIAT

RWYRAPELCGSFFSKYTPAIDIWSIGCIFAELLTGKPLFPGKNVVHQLDIITDLLGTPSA

ETIARIRNEKARRYLSSMRRKKTVPFTQKFPNADPLALNLLERMLAFDPKDRPSAEEALA

DPYFKNIASVDREPSAQPITKLEFEFERRRITKDDIRELIYREILEYHPNMLREFLEGAE

PTGFMYPSAVDHFKKQFTFLEEHYAKGSTAAPPERQHNSLPRPSVIYSDNRPQNAANITD

DLSRCAIRDNTQKAQKDSASVGANRIPHGAAAAAARPGKVVGSVLRYGNCSTSGTEQYEQ

RRVVRSPGIAPNGVTSGSSYPRRNNTCKSETGEAERIDTNQGGPPKPYVANKLPATVDGR

NGHW*

>Uni2MPK17 Brdisv1Uni21005452m.p

MVGGGGIVDGFRRLFHRRTPSGSGPSSNQSSAGEESSDIDAVVEDLDLVGLRAIRVPKRK

MPLPVESHKKNILEKEFFTEYGEASQYQIQEVIGKGSYGVVAAAVDTRTGERVAIKKIND

VFEHVSDATRILREVKLLRLLRHPDVVEIKHIMLPPSRREFQDIYVVFELMESDLHQVIR

ANDDLTPEHYQFFLYQLLRALKYIHAANVFHRDLKPKNILANSDCKLKICDFGLARVSFN

DAPTAIFWTDYVATRWYRAPELCGSFFSKYTPAIDIWSIGCIFAELLTGRPLFPGKNVVH

QLDIITDLLGTPSSETLSRIRNEKARRYLSCMRKKHPVPLTQKFPNVDPLALRLLGRLLA

FDPKDRPSAEEALADPYFAVLANVEREPSRNPISKLEFEFERRKVTKDDVRELIYREILE

YHPQMLQEYMQGGEQLSFLYPSGVDRFKRQFAHLEENYSKGERGSPLQRKHASLPRQRVG

ASNDNNEQHASDQERGADSIAPATGNPPGSQDAGQQHVSGGQNGVGSTNISPRSYQKSAS

ISASKCVVVNANKNPEYDDDISEEMEGAVDGLSEKVSRMHP*

>Uni2MPK20-1 Brdisv1Uni21019092m.p

MQQDQRKKSSAEAEFFTEYGDASRYKIQEIVGKGSYGVVCSAIDVHTGEKVAIKKIHDIF

EHISDAARILREIKLLRLLRHPDIVEIKHIMLPPSRRDFKDIYVVFELMESDLHQVIKAN

DDLTKEHYQFFLYQLLRALKYIHTANVYHRDLKPKNILANSNCKLKICDFGLARVAFNDT

PTTIFWTDYVATRWYRAPELCGSFFSKYTPAIDVWSIGCIFAEVLTGKPLFPGKNVVHQL

DLMTDLLGTPSMDTISRVRNEKARRYLSSMRKKEPIPFSQKFPNADPLALDLLQRLLAFD

PKDRPTAGEALSHPYFKGLAKVEREPSCQPITKMEFEFERRRATKEDIRELIFREILEYH

PQLLKDYINGTERTTFLYPSAVDQFRKQFAHLEENSESGPVVPMDRKHTSLPRSTIVHSA

PIHAKEQPRIGPSRDRPLTDESYKNPRDSEKYSGNVPRSSHHSQAPQRVPTARPGRVVGP

VLPYENVGTSHPYDPRRVAMNSGYPPQQQIPQTYGYYQTTGKSACSEPSQAERYTLHQQA

YACANSSTAPDVALDMRAPPFHQSAGPKSGSSDRLTAETNLYTRSLNGIVATTSGVAASA

HRKVSVVPYGMSQMY*

>Uni2MPK20-2 Brdisv1Uni21014999m.p

MQQPQQQGQRNKLPDGWFCCNVHILNLCRSAMIFLTPPIIMPSTDVDFFSEYGDANRYRI

QEVIGKGSYGVVCSAMDMQTRQKVAIKKIHNIFDHVSDAARILREIKLLRLLRHPDVVEI

KHIMLPPSRKDFKDIYVVFELMESDLHQVIKANDDLTKEHYQFFLYQLLRALKYIHTASV

YHRDLKPKNILANSNCKLKICDFGLARVAFNDTPTTVFWTDYVATRWYRAPELCGSFFTK

YTPAIDIWSIGCIFAEVLTGKPLFPGKNVVHQLDLMTDLLGTPSMDTISRVRNEKARRYL

SSMRKKDPVPFSKKFPNADPLGLKLLEKLLAFDPKDRPTAEEALTDPYFKSLSKPDREPS

CQPIRKVEFDFEHRRMSKDDIRELIFQEILEYHPQLLKNYIDGTEKTTFLYPSAVDQFKK

QFSHLEESDGSGPVVQTERKHASLPRSTTVHSTPIPAKEQPLVASSRGRPIANEPCKPWA

PGNVPGASQTAHVAQAGRAVGVGSVPPYESGSGKYPYDATSRPAVSSGYPPQQKIPQTYG

YHHHHQTPGAGQSSQAMGGYACGYTKGTTPPPAAAQDMRASPYHHRSAGTKNDPLNRLAA

ESDIYTRSLNGIVAAAASAGTGAHRKVGAVPFGMSGMY*

>Uni2MPK20-3 Brdisv1Uni21044231m.p

MQHGDLHKKSAAEMDFFTAYDDANRYKILEVIGKGSYGLVCSANDLQTGEKVAIKKIHNI

FEHISDAARILREIKLLRLLRHPDVVEIKHILLPPSKKDFKDIYVVFELMESDLHQVIKA

NDDLTREHYQFFLYQMLRALKYMHTANVYHRDLKPKNVLANANCKLKICDFGLARVAFSD

APTTVFWTDYVATRWYRAPELCGSFYSK

>Uni2MPK20-4 Brdisv1Uni21019338m.p

MQTTEQQRKKGSSEMDFFSEYGDANRYKIQEVIGKGSYGVVCSAIDQHTGDKVAIKKIHN

IFEHLSDAARILREIKLLRLLRHPDIVEIRHIMLPPSRRDFKDIYVVFELMDTDLHQVIK

ANDDLTKEHHQFFLYQMLRALKYIHTANVYHRDLKPKNILANANCKLKICDFGLARVAFN

DTPTTVFWTDYVATRWYRAPELCGSFFTKYSRAIDIWSIGCIFAEILTGKPLFPGKNVVH

QLDLMTDLLGTPSLDTVSRIRNEKARRYLSSMRKKQSVSFSERFPKADPAALKLLQRLLA

FDPKDRPTAEEALADPYFKGLGKVEREPSCQPITKMEFEFERKNVTKADVKELIFREILE

YHPQLLKDYMNGTEKTNFLYPSAVDNFRRQFANLEENGGKGGAVVPPDRKHVSLPRNTTV

HSTPIPPKDQKYSQVPQRIPTGRPGRVVGPVIPFENSSTMDPYSQRRVARNPVLPAAATN

LSAYAYRNSDNSEQELQQELEKDRMQYHPMQRFMDAKMVSPDLRSTSYYMPKGVPKADVA

ERSALQSNMMQGIAPFNGIATVGGAFNKVSAVQYGVSRMY*

>Uni2MPK20-5 Brdisv1Uni21015139m.p

MFSDIFSREGVAFSRVALRQELLSDQWFTTAGGKGQCYLVPPGTTVFLVNSEEMGFFSEY

VDASRYKILEIIGKGSYGVVCSAIDQETGDKVAIKKIQNIFEHLSDAARILREIKLLRLL

RHPDIVQIKHIMLPPSRRDFRDIYVVFELMDTDLHQVIKANDDLTKEHYQFFLYQMLRAL

KYIHTANVYHRDLKPKNILANANCKLKICDFGLARVAFNDTPTTVFWTDYVATRWYRAPE

LCGSFFTKYSPAIDTWSIGCIFAEILTGKPLFPGKNVVHQLDLMTDLLGTPSTETISRIR

NDKARKYLSSMRRKQPIPFSEKFPNADPSALKLLERLLAFDPKDRPTAEEALAHPYFKRL

ARVEREPSCQQPISKTEFEFERRKFTKEDIKELIFREILEYHPKLLKDYMNGSEKTSFLY

PSAVDNFRRQFANLEIDGGRSGAADRKHFSLPRTTTVHSAPILPTNGPTSQVPQRIPTAR

PGRVVSSAMQTDNPSVSDRHNGRRVARDPAVPPAAAYHLKSDYSDRQHQQEFEKDRVRPD

RQRQEELEKDRMQYRPGHHSMDAKVAPEISPYMRSSPYYIPPFNGIAAVASGYSKVAAVT

RMY*

>Uni2MPK21-1 Brdisv1Uni21015046m.p

MGGLIRWLRHHRSRRVSSSSSSHLPSNTTSSSTSDLRAPSLPQHQGDHHGEVVVEWEDAA

EGPDSDPEEYIVVVLGDDEQGVVAARAPVRTKPPRVMDPGKKTSESEFFTEYGEANRYKV

SEVIGKGSYGVVAAAVDTQTGERCAIKKINDVFDHVSDATRILREIKLLRLLRHPDIVEI

KHIMLPPSRREFRDIYVVFELMESDLHQVIKANDDLTPEHHQFFLYQLLRGMKYIHTANV

FHRDLKPKNILANADCKLKICDFGLARVSFNDGAPSAIFWTDYVATRWYRAPELCGSFFS

KYTPAIDIWSVGCIFAEMLTGKPLFPGKNVVHQLDLMTDLLGTPSAESLSKIRNEKARRY

LSNMRKKPKVPLTKKFPGIDPMALHLLERLLAFDPKDRPSADEALTDPYFTGLANSEREP

ITQPISKLEFEFEKRKLAKDDVRELIYREILEYHPHMLQEYLRGGGDQMSFMFPSGVDRF

KRQFAHLEEGGAKGEKSSPQLRQNASLPRERVIGNKHGDGDYNMKLNVGEKPELASVSDG

ISKPLMSARSLLKSETMSASKCIGEIKNKDEDSLSECVEGTDDDVSQKIAQLKT*

>Uni2MPK21-2 Brdisv1Uni21019188m.p

GSDEAEFFTEYGEANRYEVGEVVGKGSYGVVAAAVDTHTGERVAIKKINDVFEHVSDATR

ILREIKLLRLLRHPDIVEIKHIMLPPSRREFRDIYIIFELMESDLHQVIKANDDLTPEHH

QFFFYQLLRGMKYIHAANVFHRDLKPRNILANADCKLKICDFGLARVSFNDTPSAIFWTD

YVATRWYRAPELCGSFFSKYTPAIDIWSIGCIFAEMLSGRPLFPGKNVVHQLDLMTDLLG

TPSAESLSRIRNEKARRYLGNMRKKHPVPFSQKFPGVDPMALDLLERLLAFDPKDRPTAA

EALADPYFTGLANSDREPTTQPISKLEFEFERRKLARDDVRELIYREILEYHPQMLHEYH

HGGDQANFVYPSGVDRFKRQFVHLEEGVTKGEKTSPQLRQHASLPRERIIGIGDELGRPN

ADYCIKLHVGEEPGHTSVTDGLSKPLLNARNFLKSESISASQCVVIKEKREKDVSAVTSN

IF*

>ABR4MPK3 Brdisv1ABR41010880m.p

MDGAPVAEFRPTMTHGGRFLLYNIFGNQFEITAKYQPPIMPIGRGAYGIVCSVMNFETRE

MVAIKKIANAFDNNMDAKRTLREIKLLRHLDHENIVGLRDVIPPAIPQSFNDVYIATELM

DTDLHHIIRSNQELSEEHCQYFLYQLLRGLKYIHSANVIHRDLKPSNLLLNANCDLKICD

FGLARPSSESDMMTEYVVTRWYRAPELLLNSTDYSAAIDVWSVGCIFMELINRAPLFPGR

DHMHQMRLITEVIGTPTDDDLGFIRNEDARRYMRHLPQFPRRPFPAQFPRVQPAALDLIE

RMLAFNPLQRITVEEALEHPYLERLHDIADEPICTDPFSFDFEQHPLTEDQMKQLIFNEA

LELNPNFRY*

>ABR4MPK4 Brdisv1ABR41011661m.p

MDTSGGGGGGAGGAAQIQGMATHGGRYVLYNVYGNLFEVASKYAPPIRPIGRGAYGIVCA

AVSSDTGEEVAIKKIGNAFDNHIDAKRTLREIKLLRHMDHENIIAIKDIIRPPRRDDFKD

VYIVTELMDTDLHQIIRSNQPLTDDHCQYFLYQLLRGLKYVHSANVLHRDLKPSNLFLNA

NCDLKIADFGLARTTSETDLMTEYVVTRWYRAPELLLNCSQYTAAIDVWSVGCILGEIIT

RQPLFPGRDYIQQLKLITELIGSPDDSSLGFLRSDNARRYMKQLPQYPRQDFRLRFRNMS

DGAVDLLERMLVFDPSRRITVDEALHHPYLASLHDINEEPTCPAPFSFDFEQPSFTEEHM

KELIWRETLAFNPDPPY*

>ABR4MPK6 Brdisv1ABR41044656m.p

MTGDQTLASMTNYGRFSQIETAYWLVSYNKQSVSAFNVPVIVAIRDIIPPAQRNSFNDVY

IAYELMDTDLHQIIRSNQALSEEHCQYFLYQILRGLKYIHSANVLHRDLKPSNLLLNANC

DLKICDFGLARTTSETDFMTEYVVTRWYRAPELLLNSSEYTAAIDVWSVGCIFMELMDRK

PLFPGRDHVHQLRLLMELIGTPNEADLDFVNENARRYIRQLPRHARQSFSEKFPHVHPSA

IDLVEKMLTFDPRQRITVEGALAHPYLASLHDISDEPVCTMPFSFDFEQHALSEEQMKDL

IHQEALAFNPDYQ*

>ABR4MPK7-1 Brdisv1ABR41046323m.p

MCTRNLPFFKVAVDPKMKLPHDNHKTIRHEVVNLLHDKCARKLPFFKVVVHRKLKIPHGK

HKTTKHEAEKLPHDKCTNKLHFSKLLRGLKYLHSAGILHRDLKPGNLLVNANCDLKICDF

GLARTNNTKGQFMTEYVVTRWYRAPELLLCCDNYGTSIDVWSVGCIFAELLGRKPIFPRT

QCLDQLKLIVNVLGTMSENDLEFIDNPKARKYIKSLPYTPGTPLTSMYPQAHPLAIDLLQ

KMLVFDPSKRISVTEALEHPYMSPLYDPSANPPAQVPIDLDIDENLGVEMIREMLWQEML

QYHPEAARMVNM*

>ABR4MPK11 Brdisv1ABR41033293m.p

MRMEGGGAGPAAAAAAGGAHGLGEAQIKGTLTHGGRYVQYNVYGNLFEVSAKYVPPIRPV

GRGACGIICAAINVQTREEVAIKKIGNAFDNQIDAKRTLREVKLLRHMNHENVISIKDII

RPPRRENFNDVYIVYELMDTDLHHLLRSNQPLTDDHCQYFLYQVLRGLKYVHSANVLHRD

LRPSNLLLNAKCDLKIGDFGLARTTTETDFMMEYVVTRWYRAPELLLNCSEYTGAIDMWS

VGCILGEIATREPLFPGKDYVHQLRLITELLGSPDDTSLGFLRSDNARRYVRSLPQYPKQ

QFRSRFPNMSSGAMDLLERMLVFDPNKRITVDEALCHPYLASLHEINDEPVCPAPFSFDF

EQPSFTEEDIKELIWRESVKFNPEPIH*

>ABR4MPK14 Brdisv1ABR41031114m.p

MAMLVDPPNGMGNQGKHYYSMWQTLFEIDTKYVPIKPIGRGAYGIVCSSINRETNEKVAI

KKIHNVFDNRVDALRTLRELKLLRHLRHENVISLKDIMMPVQRRSFKDVYLVYELMDTDL

HQIIKSPQGLSNDHCQYFLFQLLRGLKYLHSAEILHRDLKPGNLLVNANCDLKICDFGLA

RTNSSKGQFMTEYVVTRWYRAPELLLCCDNYGTSIDVWSVGCIFAELLGRKPIFPGTECL

NQLKLIVNVLGTMSESDLEFIDNPKARRYIKTLPYTPGVPLASMYPHAHPLAIDLLQKML

IFDPTKRISVTEALEHPYMSPLYDPSANPPAQVPIDLDLDENISADMIREMMWQEMLHYH

PEAATAISM*

>ABR4MPK16 Brdisv1ABR41018512m.p

MDFFTEYGEGNRYKIEEVIGKGSYGVVCSALDTHTGDKVAIKKINDIFEHVSDATRILRE

IKLLRLLRHPDIVEIKHILLPPSRREFKDIYVVFELMESDLHQVIKANDDLTPEHYQFFL

YQLLRGLKYIHTANVFHRDLKPKNILANADCKLKICDFGLARVAISDTPTAIFWTDYIAT

RWYRAPELCGSFFSKYTPAIDIWSIGCIFAELLTGKPLFPGKNVVHQLDIITDLLGTPSA

ETIARIRNEKARRYLSSMRRKKTVPFTQKFPNADPLALNLLERMLAFDPKDRPSAEEALA

DPYFKNIASVDREPSAQPITKLEFEFERRRITKDDIRELIYREILEYHPNMLREFLEGAE

PTGFMYPSAVDHFKKQFTFLEEHYAKGSTAAPPERQHNSLPRPSVIYSDNRPQNAANITD

DLSRCAIRDNTQKAQKDSASVGANRIPHGAAAAAARPGKVVGSVLRYGNCSTSGTEQYEQ

RRVVRSPGIAPNGVTSGSSYPRRNNTCKSETGEAERIDTNQGGPPKPYVANKLPATVDGR

NGHW*

>ABR4MPK17 Brdisv1ABR41005759m.p

MVGGGGIVDGFRRLFHRRTPSGSGPSSNQSSAGEESSDIDAVVEDLDLVGLRAIRVPKRK

MPLPVESHKKNILEKEFFTEYGEASQYQIQEVIGKGSYGVVAAAVDTRTGERVAIKKIND

VFEHVSDATRILREVKLLRLLRHPDVVEIKHIMLPPSRREFQDIYVVFELMESDLHQVIR

ANDDLTPEHYQFFLYQLLRALKYIHAANVFHRDLKPKNILANSDCKLKICDFGLARVSFN

DAPTAIFWTDYVATRWYRAPELCGSFFSKYTPAIDIWSIGCIFAELLTGRPLFPGKNVVH

QLDIITDLLGTPSSETLSRIRNEKARRYLSCMRKKHPVPLTQKFPNVDPLALRLLGRLLA

FDPKDRPSAEEALADPYFAVLANVEREPSRNPISKLEFEFERRKVTKDDVRELIYREILE

YHPQMLQEYMQGGEQLSFLYPSGVDRFKRQFAHLEENYSKGERGSPLQRKHASLPRQRVG

ASNDNNEQHASDQERGADSIAPATGNPPGSQDAGQQHVSGGQNGVGSTNISPRSYQKSAS

ISASKCVVVNANKNPEYDDDISEEMEGAVDGLSEKVSRMHP*

>ABR4MPK20-1 Brdisv1ABR41019969m.p

MQQDQRKKSSAEAEFFTEYGDASRYKIQEIVGKGSYGVVCSAIDVHTGEKVAIKKIHDIF

EHISDAARILREIKLLRLLRHPDIVEIKHIMLPPSRRDFKDIYVVFELMESDLHQVIKAN

DDLTKEHYQFFLYQLLRALKYIHTANVYHRDLKPKNILANSNCKLKICDFGLARVAFNDT

PTTIFWTDYVATRWYRAPELCGSFFSKYTPAIDVWSIGCIFAEVLTGKPLFPGKNVVHQL

DLMTDLLGTPSMDTISRVRNEKARRYLSSMRKKEPIPFSQKFPNADPLALDLLQRLLAFD

PKDRPTAGEALSHPYFKGLAKVEREPSCQPITKMEFEFERRRATKEDIRELIFREILEYH

PQLLKDYINGTERTTFLYPSAVDQFRKQFAHLEENSESGPVVPMDRKHTSLPRSTIVHSA

PIHAKEQPRIGPSRDRPLTDESYKNPRDSEKYSGNVPRSSHHSQAPQRVPTARPGRVVGP

VLPYENVGTSHPYDPRRVAMNSGYPPQQQIPQTYGYYQTTGKSACSEPSQAERYTLHQQA

YACANSSTAPDVALDMRAPPFHQSAGPKSGSSDRLTAETNLYTRSLNGIVATTSGVAASA

HRKVSVVPYGMSQMY*

>ABR4MPK20-2 Brdisv1ABR41015659m.p

MQQPQQQGQRNKSTDVDFFSEYGDANRYRIQEVIGKGSYGVVCSAMDMQTRQKVAIKKIH

NIFDHVSDAARILREIKLLRLLRHPDVVEIKHIMLPPSRKDFKDIYVVFELMESDLHQVI

KANDDLTKEHYQFFLYQLLRALKYIHTASVYHRDLKPKNILANSNCKLKICDFGLARVAF

NDTPTTVFWTDYVATRWYRAPELCGSFFTKYTPAIDIWSIGCIFAEVLTGKPLFPGKNVV

HQLDLMTDLLGTPSMDTISRVRNEKARRYLSSMRKKDPVPFSKKFPNADPLGLKLLEKLL

AFDPKDRPTAEEALTDPYFKSLSKPDREPSCQPIRKVEFDFEHRRMSKDDIRELIFQEIL

EYHPQLLKNYIDGTEKTTFLYPSAVDQFKKQFSHLEESDGSGPVVQTERKHASLPRSTTV

HSTPIPAKEQPLVASSRGRPIANEPCKPWAPGNVPGASQTAHVAQAGRAVGVGSVPPYES

GSGKYPYDATSRPAVSSGYPPQQKIPQTYGYHHHHQTPGAGQSSQAMGGYACGYTKGTTP

PPAAAQDMRASPYHHRSAGTKNDPLNRLAAESDIYTRSLNGIVAAAASAGTGAHRKVGAV

PFGMSGMY*

>ABR4MPK20-3 Brdisv1ABR41045163m.p

MQHGDLHKKSAAEMDFFTAYDDANRYKILEVIGKGSYGLVCSANDLQTGEKVAIKKIHNI

FEHISDAARILREIKLLRLLRHPDVVEIKHILLPPSKKDFKDIYVVFELMESDLHQVIKA

NDDLTREHYQFFLYQMLRALKYMHTANVYHRDLKPKNVLANANCKLKICDFGLARVAFSD

APTTVFWTDYVATRWYRAPELCGSFYSKVMIVTIDH*

>ABR4MPK20-4 Brdisv1ABR41020187m.p

MQTTEQQRKKGSSEMDFFSEYGDANRYKIQEVIGKGSYGVVCSAIDQHTGDKVAIKKIHN

IFEHLSDAARILREIKLLRLLRHPDIVEIRHIMLPPSRRDFKDIYVVFELMDTDLHQVIK

ANDDLTKEHHQFFLYQMLRALKYIHTANVYHRDLKPKNILANANCKLKICDFGLARVAFN

DTPTTVFWTDYVATRWYRAPELCGSFFTKYSRAIDIWSIGCIFAEILTGKPLFPGKNVVH

QLDLMTDLLGTPSLDTVSRIRNEKARRYLSSMRKKQSVSFSERFPKADPAALKLLQRLLA

FDPKDRPTAEEALADPYFKGLGKVEREPSCQPITKMEFEFERKNVTKADVKELIFREILE

YHPQLLKDYMNGTEKTNFLYPSAVDNFRRQFANLEENGGKGGAVVPPDRKHVSLPRNTTV

HSTPIPPKDQKYSQVPQRIPTGRPGRVVGPVIPFENSSTMDPYSQRRVARNPVLPAAATN

LSAYAYRNSDNSERELQQELEKDRMQYHPMQRFMDAKMVSPDLRSTSYYMPKGVPKADVA

ERSALQSNMMQGIAPFNGIATVGGAFNKVSAVQYGVSRMY*

>ABR4MPK20-5 Brdisv1ABR41015812m.p

MFSDIFSREGVAFSRVALRQELLSDQWFTTAGGKGQCYLVPPGTTVFLVNSEEMGFFSEY

VDASRYKILEIIGKGSYGVVCSAIDQETGDKVAIKKIQNIFEHLSDAARILREIKLLRLL

RHPDIVQIKHIMLPPSRRDFRDIYVVFELMDTDLHQVIKANDDLTKEHYQFFLYQMLRAL

KYIHTANVYHRDLKPKNILANANCKLKICDFGLARVAFNDTPTTVFWTDYVATRWYRAPE

LCGSFFTKYSPAIDTWSIGCIFAEILTGKPLFPGKNVVHQLDLMTDLLGTPSTETISRIR

NDKARKYLSSMRRKQPIPFSEKFPNADPSALKLLERLLAFDPKDRPTAEEALAHPYFKRL

ARVEREPSCQQPISKTEFEFERRKFTKEDIKELIFREILEYHPKLLKDYMNGSEKTSFLY

PSAVDNFRRQFANLEIDGGRSGAADRKHFSLPRTTTVHSAPILPTNGPTSQVPQRIPTAR

PGRVVSSAMQTDNPSVSDRHNGRRVARDPAVPPAAAYHLKSDYSDRQHQQEFEKDRVRPD

RQRQEELEKDRMQYRPGHHSMDAKVAPEISPYMRSSPYYIPPFNGIAAVASGYSKVAAVT

RMY*

>ABR4MPK21-1 Brdisv1ABR41015701m.p

MGGLIRWLRHHRSRRVSSSSSSHLPSNTTSSSTSDLRAHSLPQHQGDHHGEVVVEWEDAA

EGPDSDPEEYIVVVLGDDEQGVVAARAPVRTKPPRVMDPGKKTSESEFFTEYGEANRYKV

SEVIGKGSYGVVAAAVDTQTGERCAIKKINDVFDHVSDATRILREIKLLRLLRHPDIVEI

KHIMLPPSRREFRDIYVVFELMESDLHQVIKANDDLTPEHHQFFLYQLLRGMKYIHTANV

FHRDLKPKNILANADCKLKICDFGLARVSFNDGAPSAIFWTDYVATRWYRAPELCGSFFS

KYTPAIDIWSVGCIFAEMLTGKPLFPGKNVVHQLDLMTDLLGTPSAESLSKIRNEKARRY

LSNMRKKPKVPLTKKFPGIDPMALHLLERLLAFDPKDRPSADEALTDPYFTGLANSEREP

ITQPISKLEFEFEKRKLAKDDVRELIYREILEYHPHMLQEYLRGGGDQMSFMFPSGVDRF

KRQFAHLEEGGAKGEKSSPQLRQNASLPRERVIGNKHGDGDYNMKLNVGEKPELASVSDG

ISKPLMSARSLLKSETMSASKCIGEIKNKDEDSLSECVEGTDDDVSQKIAQLKT*

>ABR4MPK21-2 Brdisv1ABR41020060m.p

MLPPSRREFRDIYIIFELMESDLHQVIKANDDLTPEHHQFFFYQLLRGMKYIHAANVFHR

DLKPRNILANADCKLKICDFGLARVSFNDTPSAIFWTDYVATRWYRAPELCGSFFSKYTP

AIDIWSIGCIFAEMLSGRPLFPGKNVVHQLDLMTDLLGTPSAESLSRIRNEKARRYLGNM

RKKHPVPFSQKFPGVDPMALDLLERLLAFDPKDRPTAAEALADPYFTGLANSDREPTTQP

ISKLEFEFERRKLARDDVRELIYREILEYHPQMLHEYHHGGDQANFVYPSGVDRFKRQFV

HLEEGVTKGEKTSPQLRQHASLPRERIIGIGDELGRPNADYCIKLHVGEEPGHTSVTDGL

SKPLLNARNFLKSESISASQCVCHQREARKRCKCCYIKYILNQIVSKIIN*

>S8iiCMPK3 Brdisv1S8iiC1010629m.p

MDGAPVAEFRPTMTHGGRFLLYNIFGNQFEITAKYQPPIMPIGRGAYGIVCSVMNFETRE

MVAIKKIANAFDNNMDAKRTLREIKLLRHLDHENIVGLRDVIPPAIPQSFNDVYIATELM

DTDLHHIIRSNQELSEEHCQYFLYQLLRGLKYIHSANVIHRDLKPSNLLLNANCDLKICD

FGLARPSSESDMMTEYVVTRWYRAPELLLNSTDYSAAIDVWSVGCIFMELINRAPLFPGR

DHMHQMRLITEVIGTPTDDDLGFIRNEDARRYMRHLPQFPRRPFPAQFPRVQPAALDLIE

RMLAFNPLQRITVEEALEHPYLERLHDIADEPICTDPFSFDFEQHPLTEDQMKQLIFNEA

LELNPNFRY*

>S8iiCMPK4 Brdisv1S8iiC1027882m.p

MDTSGGGGGGAGGAAQIQGMATHGGRYVLYNVYGNLFEVASKYAPPIRPIGRGAYGIVCA

AVSSDTGEEVAIKKIGNAFDNHIDAKRTLREIKLLRHMDHENIIAIKDIIRPPRRDDFKD

VYIVTELMDTDLHQIIRSNQPLTDDHCQYFLYQLLRGLKYVHSANVLHRDLKPSNLFLNA

NCDLKIADFGLARTTSETDLMTEYVVTRWYRAPELLLNCSQYTAAIDVWSVGCILGEIIT

RQPLFPGRDYIQQLKLITELIGSPDDSSLGFLRSDNARRYMKQLPQYPRQDFRLRFRNMS

DGAVDLLERMLVFDPSRRITVDEALHHPYLASLHDINEEPTCPAPFSFDFEQPSFTEEHM

KELIWRETLAFNPDPPY*

>S8iiCMPK6 Brdisv1S8iiC1007741m.p

MDGGAQPPDAEMADAGAAVPGAGAGAAGTMENIQATLSHGGRFIQYNIFGNVFEVTVKYK

PPILPIGKGAYGIVCSALNSETGEQVAIKKIANAFDNKIDAKRTLREIKLLRHMDHENIV

AIRDIIPPAQRNSFNDVYIAYELMDTDLHQIIRSNQALSEEHCQYFLYQILRGLKYIHSA

NVLHRDLKPSNLLLNANCDLKICDFGLARTTSETDFMTEYVVTRWYRAPELLLNSSEYTA

AIDVWSVGCIFMELMDRKPLFPGRDHVHQLRLLMELIGTPNEADLDFVNENARRYIRQLP

RHARQSFSEKFPHVHPSAIDLVEKMLTFDPRQRITVEGALAHPYLASLHDISDEPVCTMP

FSFDFEQHALSEEQMKDLIHQEALAFNPDYQ*

>S8iiCMPK7-2 Brdisv1S8iiC1036390m.p

MKLSPISASFGAMGSLPQKLDELLSTGHWALSGVVMDEIEELTADLHILCNLLLKLSNVH

DPPPTVRFWMKEVRELSYDVEDCADQFVIANDRAVMRRAKIHRATIRTIIITRLKISRLP

KRRKWRLWMTDKVAEFRTRVQDATQRYWRYKFDDCASNPGYASVGHEFATVYAQPGDLVG

IEGPMDELEQWLTDGEEQLKVISIVGLGGVGKTTLAQKLWATLKGQFLCRAFVRTAQKPD

MRGILRNILLQVRPHQLPNRGEMHHLIHDLREYLQDKRYFVIIDDLWAASVWDVVSRAFP

EGNCCSRIVTTTEIMEVALACCDYCPKHICKLETLSDDDSEKLLLERIVVSGNQIPQQLD

DVLPQIMRNCGGLPLAIIIVASLLVSQPEKLEQWGHTQNSFGSIFGTNPTMEGFIRQILS

ISFNSLPYYLKTCLLYLSSYPEGCLFLKDDLVKLWVAEGFIHAKEWEDMEDLAGRYFDEL

VNVGLIQVMDINYNYKLPSYSVHHMVLDFITCKSIEENFITVVDYSETTTPLTDKVRRLS

LHFGSATHATTPASARLSNVRSLFYFGLFNCMPSFMVFKLLRVLILNFWGDPGNTSFNLT

GICELVWLRYLQVSCNVTVKLPDQIESMGHLETLEIKAKVYAVPLDIVRLSSLSHLHLRG

GTIPNGIGCMRSLRTLKYFDLVNKSVDNLRGLGELANLRDLHLTYSSSLPSEHLKRNLIA

LASSLGKLCNLKSLTLASETAATVVPFDGSISMSSTPAFLERLELLPSICIFSRLPKWIF

QFQKLCILKVAVRELLANDIDSITGLPSLTVLSLCVHTAPGQRIVFNDGAFPVLKYFKFR

CGVLSMSFMAGAMPNLRRLKLGFNTHVGEKYGNMLAGIEHLLNLQDIAGKIGVATESDRK

AAESAIKGAISKHPQSPMLNVQWVDLVEEEYHASEKQHGRQEKGLSGENHAVLEKAKDTK

KHADSGVSQLPDLPSTVSSSRLKSENIAQASKHHVLESMLCDESAQPESLEVSVLEDITN

NFSDDQEISKNRFVVVYKGELQNGSVVVVKRFTIAIDYNLFLDAVDCLMSVKHNNIVRFL

GYCANSQENAAKEGSKSVSVVTQERLCCFEYLRNGNLQMHLTDESCGFDWQMWYQIIEGI

CQGVHYLHNKSITPLDLNPANIMFDDKMVPKIVHYAYTRFLGAATSPVLTNIGDSLAYMA

PEYFGFGEITSKSDIYSLGVIIMQIVTGENKKDFPSIQSVLESWRSRLESDGSQGHTSLE

TCYLQVNLCYLIGIRCIEHDPGKRPNTRDIIDSLDTLEIMECLVKHDVGNSSLEQMLVCS

KVEEPKALPAPPSVSCKLKMAMMVDPPNGTGNHGKHYYTMWQTMFEIDTKYVPIKPIGRG

AYGIVFSSTNQENNEKVAIKKINNVFDNRVDALRTLRELKLLRHLRHENVIALKDIMMPV

HRRSFKDVYLVFELMDTDLHQIIKSSQPLSNDHCQYFLFQLLRGLKYLHSAGILHRDLKP

GNLLVNANCDLKICDFGLARTNNTKGQFMTEYVVTRLYRAPELLLCCDNYGTSIDVWSVG

CIFAELLGRKPIFPGTECLNQLKLIVNVLGTMSENDLEFIDNPKARKYIKSLPYTPGTPL

TSMYPQAHPLAIDLLQKMLVFDPSKRISVTEALEHPYMSPLYDPSANPPAQVPIDLDIDE

NLGVEMIREMLWQEMLQYHPEAARMVNM*

>S8iiCMPK11 Brdisv1S8iiC1025854m.p

MRMEGGGAGPAAAAAAGGAHGLGEAQIKGTLTHGGRYVQYNVYGNLFEVSAKYVPPIRPV

GRGACGIICAAINVQTREEVAIKKIGNAFDNQIDAKRTLREVKLLRHMNHENVISIKDII

RPPRRENFNDVYIVYELMDTDLHHLLRSNQPLTDDHCQYFLYQVLRGLKYVHSANVLHRD

LRPSNLLLNAKCDLKIGDFGLARTTTETDFMMEYVVTRWYRAPELLLNCSEYTGAIDMWS

VGCILGEIATREPLFPGKDYVHQLRLITELLGSPDDTSLGFLRSDNARRYVRSLPQYPKQ

QFRSRFPNMSSGAMDLLERMLVFDPNKRITVDEALCHPYLASLHEINDEPVCPAPFSFDF

EQPSFTEEDIKELIWRESVKFNPEPIH*

>S8iiCMPK14 Brdisv1S8iiC1023720m.p

MAMLVDPPNGMGNQGKHYYSMWQTLFEIDTKYVPIKPIGRGAYGIVCSSINRETNEKVAI

KKIHNVFDNRVDALRTLRELKLLRHLRHENVISLKDIMMPVQRRSFKDVYLVYELMDTDL

HQIIKSPQGLSNDHCQYFLFQLLRGLKYLHSAEILHRDLKPGNLLVNANCDLKICDFGLA

RTNSSKGQFMTEYVVTRWYRAPELLLCCDNYGTSIDVWSVGCIFAELLGRKPIFPGTECL

NQLKLIVNVLGTMSESDLEFIDNPKARRYIKTLPYTPGVPLASMYPHAHPLAIDLLQKML

IFDPTKRISVTEALEHPYMSPLYDPSANPPAQVPIDLDLDENISADMIREMMWQEMLHYH

PEAATAISM*

>S8iiCMPK16 Brdisv1S8iiC1018261m.p

MDFFTEYGEGNRYKIEEVIGKGSYGVVCSALDTHTGDKVAIKKINDIFEHVSDATRILRE

IKLLRLLRHPDIVEIKHILLPPSRREFKDIYVVFELMESDLHQVIKANDDLTPEHYQFFL

YQLLRGLKYIHTANVFHRDLKPKNILANADCKLKICDFGLARVAISDTPTAIFWTDYIAT

RWYRAPELCGSFFSKYTPAIDIWSIGCIFAELLTGKPLFPGKNVVHQLDIITDLLGTPSA

ETIARIRNEKARRYLSSMRRKKTVPFTQKFPNADPLALNLLERMLAFDPKDRPSAEEALA

DPYFKNIASVDREPSAQPITKLEFEFERRRITKDDIRELIYREILEYHPNMLREFLEGAE

PTGFMYPSAVDHFKKQFTFLEEHYAKGSTAAPPERQHNSLPRPSVIYSDNRPQNAANITD

DLSRCAIRDNTQKAQKDSASVGANRIPHGAAAAAARPGKVVGSVLRYGNCSTSGTEQYEQ

RRVVRSPGIAPNGVTSGSSYPRRNNTCKSETGEAERIDTNQGGPPKPYVANKLPATVDGR

NGHW*

>S8iiCMPK17 Brdisv1S8iiC1005709m.p

MVGGGGIVDGFRRLFHRRTPSGSGPSSNQSSAGEESSDIDAVVEDLDLVGLRAIRVPKRK

MPLPVESHKKNILEKEFFTEYGEASQYQIQEVIGKGSYGVVAAAVDTRTGERVAIKKIND

VFEHVSDATRILREVKLLRLLRHPDVVEIKHIMLPPSRREFQDIYVVFELMESDLHQVIR

ANDDLTPEHYQFFLYQLLRALKYIHAANVFHRDLKPKNILANSDCKLKICDFGLARVSFN

DAPTAIFWTDYVATRWYRAPELCGSFFSKYTPAIDIWSIGCIFAELLTGRPLFPGKNVVH

QLDIITDLLGTPSSETLSRIRNEKARRYLSCMRKKHPVPLTQKFPNVDPLALRLLGRLLA

FDPKDRPSAEEALADPYFAVLANVEREPSRNPISKLEFEFERRKVTKDDVRELIYREILE

YHPQMLQEYMQGGEQLSFLYPSGVDRFKRQFAHLEENYSKGERGSPLQRKHASLPRQRVG

ASNDNNEQHASDQERGADSIAPATGNPPGSQDAGQQHVSGGQNGVGSTNISPRSYQKSAS

ISASKCVVVNANKNPEYDDDISEEMEGAVDGLSEKVSRMHP*

>S8iiCMPK20-1 Brdisv1S8iiC1019709m.p

MQQDQRKKSSAEAEFFTEYGDASRYKIQEIVGKGSYGVVCSAIDVHTGEKVAIKKIHDIF

EHISDAARILREIKLLRLLRHPDIVEIKHIMLPPSRRDFKDIYVVFELMESDLHQVIKAN

DDLTKEHYQFFLYQLLRALKYIHTANVYHRDLKPKNILANSNCKLKICDFGLARVAFNDT

PTTIFWTDYVATRWYRAPELCGSFFSKYTPAIDVWSIGCIFAEVLTGKPLFPGKNVVHQL

DLMTDLLGTPSMDTISRVRNEKARRYLSSMRKKEPIPFSQKFPNADPLALDLLQRLLAFD

PKDRPTAGEALSHPYFKGLAKVEREPSCQPITKMEFEFERRRATKEDIRELIFREILEYH

PQLLKDYINGTERTTFLYPSAVDQFRKQFAHLEENSESGPVVPMDRKHTSLPRSTIVHSA

PIHAKEQPRIGPSRDRPLTDESYKNPRDSEKYSGNVPRSSHHSQAPQRVPTARPGRVVGP

VLPYENVGTSHPYDPRRVAMNSGYPPQQQIPQTYGYYQTTGKSACSEPSQAERYTLHQQA

YACANSSTAPDVALDMRAPPFHQSAGPKSGSSDRLTAETNLYTRSLNGIVATTSGVAASA

HRKVSVVPYGMSQMY*

>S8iiCMPK20-2 Brdisv1S8iiC1015496m.p

MQQPQQQGQRNKSTDVDFFSEYGDANRYRIQEVIGKGSYGVVCSAMDMQTRQKVAIKKIH

NIFDHVSDAARILREIKLLRLLRHPDVVEIKHIMLPPSRKDFKDIYVVFELMESDLHQVI

KANDDLTKEHYQFFLYQLLRALKYIHTASVYHRDLKPKNILANSNCKLKICDFGLARVAF

NDTPTTVFWTDYVATRWYRAPELCGSFFTKYTPAIDIWSIGCIFAEVLTGKPLFPGKNVV

HQLDLMTDLLGTPSMDTISRVRNEKARRYLSSMRKKDPVPFSKKFPNADPLGLKLLEKLL

AFDPKDRPTAEEALTDPYFKSLSKPDREPSCQPIRKVEFDFEHRRMSKDDIRELIFQEIL

EYHPQLLKNYIDGTEKTTFLYPSAVDQFKKQFSHLEESDGSGPVVQTERKHASLPRSTTV

HSTPIPAKEQPLVASSRGRPIANEPCKPWAPGNVPGASQTAHVAQAGRAVGVGSVPPYES

GSGKYPYDATSRPAVSSGYPPQQKIPQTYGYHHHHQTPGAGQSSQAMGGYACGYTKGTTP

PPAAAQDMRASPYHHRSAGTKNDPLNRLAAESDIYTRSLNGIVAAAASAGTGAHRKVGAV

PFGMSGMY*

>S8iiCMPK20-4 Brdisv1S8iiC1019946m.p

MQTTEQQRKKGSSEMDFFSEYGDANRYKIQEVIGKGSYGVVCSAIDQHTGDKVAIKKIHN

IFEHLSDAARILREIKLLRLLRHPDIVEIRHIMLPPSRRDFKDIYVVFELMDTDLHQVIK

ANDDLTKEHHQFFLYQMLRALKYIHTANVYHRDLKPKNILANANCKLKICDFGLARVAFN

DTPTTVFWTDYVATRWYRAPELCGSFFTKYSRAIDIWSIGCIFAEILTGKPLFPGKNVVH

QLDLMTDLLGTPSLDTVSRIRNEKARRYLSSMRKKQSVSFSERFPKADPAALKLLQRLLA

FDPKDRPTAEEALADPYFKGLGKVEREPSCQPITKMEFEFERKNVTKADVKELIFREILE

YHPQLLKDYMNGTEKTNFLYPSAVDNFQRQFANLEENGGKGGAVVPPDRKHVSLPRNTTV

HSTPIPPKDQKYSQVPQRIPTGRPGRVVGPVIPFENSSTMDPYSQRRVARNPVLPAAATN

LSAYAYRNSDNSERELQQELEKDRMQYHPMQRFMDAKMVSPDLRSTSYYMPKGVPKADVA

ERSALQSNMMQGIAPFNGIATVGGAFNKVSAVQYGVSRMY*

>S8iiCMPK20-5 Brdisv1S8iiC1015656m.p

MFSDIFSREGVAFSRVALRQELLSDQWFTTAGGKGQCYLVPPGTTVFLVNSEEMGFFSEY

VDASRYKILEIIGKGSYGVVCSAIDQETGDKVAIKKIQNIFEHLSDAARILREIKLLRLL

RHPDIVQIKHIMLPPSRRDFRDIYVVFELMDTDLHQVIKANDDLTKEHYQFFLYQMLRAL

KYIHTANVYHRDLKPKNILANANCKLKICDFGLARVAFNDTPTTVFWTDYVATRWYRAPE

LCGSFFTKYSPAIDTWSIGCIFAEILTGKPLFPGKNVVHQLDLMTDLLGTPSTETISRIR

NDKARKYLSSMRRKQPIPFSEKFPNADPSALKLLERLLAFDPKDRPTAEEALAHPYFKRL

ARVEREPSCQQPISKTEFEFERRKFTKEDIKELIFREILEYHPKLLKDYMNGSEKTSFLY

PSAVDNFRRQFANLEIDGGRSGAADRKHFSLPRTTTVHSAPILPTNGPTSQVPQRIPTAR

PGRVVSSAMQTDNPSVSDRHNGRRVARDPAVPPAAAYHLKSDYSDRQHQQEFEKDRVRPD

RQRQEELEKDRMQYRPGHHSMDAKVAPEISPYMRSSPYYIPPFNGIAAVASGYSKVAAVT

RMY*

>S8iiCMPK21-1 Brdisv1S8iiC1015529m.p

MGGLIRWLRHHRSRRVSSSSSSHLPSNTTSSSTSDLRAHSLPQHQGDHHGEVVVEWEDAA

EGPDSDPEEYIVVVLGDDEQGVVAARAPVRTKPPRVMDPGKKTSESEFFTEYGEANRYKV

SEVIGKGSYGVVAAAVDTQTGERCAIKKINDVFDHVSDATRILREIKLLRLLRHPDIVEI

KHIMLPPSRREFRDIYVVFELMESDLHQVIKANDDLTPEHHQFFLYQLLRGMKYIHTANV

FHRDLKPKNILANADCKLKICDFGLARVSFNDGAPSAIFWTDYVATRWYRAPELCGSFFS

KYTPAIDIWSVGCIFAEMLTGKPLFPGKNVVHQLDLMTDLLGTPSAESLSKIRNEKARRY

LSNMRKKPKVPLTKKFPGIDPMALHLLERLLAFDPKDRPSADEALTDPYFTGLANSEREP

ITQPISKLEFEFEKRKLAKDDVRELIYREILEYHPHMLQEYLRGGGDQMSFMFPSGVDRF

KRQFAHLEEGGAKGEKSSPQLRQNASLPRERVIGNKHGDGDYNMKLNVGEKPELASVSDG

ISKPLMSARSLLKSETMSASKCIGEIKNKDEDSLSECVEGTDDDVSQKIAQLKT*

>S8iiCMPK21-2 Brdisv1S8iiC1019794m.p

MLPPSRREFRDIYIIFELMESDLHQVIKANDDLTPEHHQFFFYQLLRGMKYIHAANVFHR

DLKPRNILANADCKLKICDFGLARVSFNDTPSAIFWTDYVATRWYRAPELCGSFFSKYTP

AIDIWSIGCIFAEMLSGRPLFPGKNVVHQLDLMTDLLGTPSAESLSRIRNEKARRYLGNM

RKKHPVPFSQKFPGVDPMALDLLERLLAFDPKDRPTAAEALADPYFTGLANSDREPTTQP

ISKLEFEFERRKLARDDVRELIYREILEYHPQMLHEYHHGGDQANFVYPSGVDRFKRQFV

HLEEGVTKGEKTSPQLRQHASLPRERIIGIGDELGRPNADYCIKLHVGEEPGHTSVTDGL

SKPLLNARNFLKSESISASQCVCHQREARKRCKCCYIKYILNQIVSKIIN*

>S8iiCMPK20-3 Brdisv1S8iiC1044636m.p

MQHGDLHKKSAAEMDFFTAYDDANRYKILEVIGKGSYGLVCSANDLQTGEKVAIKKIHNI

FEHISDAARILREIKLLRLLRHPDVVEIKHILLPPSKKDFKDIYVVFELMESDLHQVIKA

NDDLTREHYQFFLYQMLRALKYMHTANVYHRDLKPKNVLANANCKLKICDFGLARVAFSD

APTTVFWTDYVATRWYRAPELCGSFYSKVMIVTIDH*

>Jer1MPK3 Brdisv1Jer11009941m.p

MDGAPVAEFRPTMTHGGRFLLYNIFGNQFEITAKYQPPIMPIGRGAYGIVCSVMNFETRE

MVAIKKIANAFDNNMDAKRTLREIKLLRHLDHENIVGLRDVIPPAIPQSFNDVYIATELM

DTDLHHIIRSNQELSEEHCQYFLYQLLRGLKYIHSANVIHRDLKPSNLLLNANCDLKICD

FGLARPSSESDMMTEYVVTRWYRAPELLLNSTDYSAAIDVWSVGCIFMELINRAPLFPGR

DHMHQMRLITEVIGTPTDDDLGFIRNEDARRYMRHLPQFPRRPFPAQFPRVQPAALDLIE

RMLAFNPLQRITVEEALEHPYLERLHDIADEPICTDPFSFDFEQHPLTEDQMKQLIFNEA

LELNPNFRY*

>Jer1MPK4 Brdisv1Jer11032692m.p

MDTSGGGGGGAGGAAQIQGMATHGGRYVLYNVYGNLFEVASKYAPPIRPIGRGAYGIVCA

AVSSDTGEEVAIKKIGNAFDNHIDAKRTLREIKLLRHMDHENIIAIKDIIRPPRRDDFKD

VYIVTELMDTDLHQIIRSNQPLTDDHCQYFLYQLLRGLKYVHSANVLHRDLKPSNLFLNA

NCDLKIADFGLARTTSETDLMTEYVVTRWYRAPELLLNCSQYTAAIDVWSVGCILGEIIT

RQPLFPGRDYIQQLKLITELIGSPDDSSLGFLRSDNARRYMKQLPQYPRQDFRLRFRNMS

DGAVDLLERMLVFDPSRRITVDEALHHPYLASLHDINEEPTCPAPFSFDFEQPSFTEEHM

KELIWRETLAFNPDPPY*

>Jer1MPK6 Brdisv1Jer11007231m.p

MDGGAQPPDAEMADAGAAVPGAGAGAAGTMENIQATLSHGGRFIQYNIFGNVFEVTVKYK

PPILPIGKGAYGIVCSALNSETGEQVAIKKIANAFDNKIDAKRTLREIKLLRHMDHENIV

AIRDIIPPAQRNSFNDVYIAYELMDTDLHQIIRSNQALSEEHCQYFLYQILRGLKYIHSA

NVLHRDLKPSNLLLNANCDLKICDFGLARTTSETDFMTEYVVTRWYRAPELLLNSSEYTA

AIDVWSVGCIFMELMDRKPLFPGRDHVHQLRLLMELIGTPNEADLDFVNENARRYIRQLP

RHARQSFSEKFPHVHPSAIDLVEKMLTFDPRQRITVEGALAHPYLASLHDISDEPVCTMP

FSFDFEQHALSEEQMKDLIHQEALAFNPDYQ*

>Jer1MPK11 Brdisv1Jer11030890m.p

MRMEGGGAGPAAAAAAGGAHGLGEAQIKGTLTHGGRYVQYNVYGNLFEVSAKYVPPIRPV

GRGACGIICAAINVQTREEVAIKKIGNAFDNQIDAKRTLREVKLLRHMNHENVISIKDII

RPPRRENFNDVYIVYELMDTDLHHLLRSNQPLTDDHCQYFLYQVLRGLKYVHSANVLHRD

LRPSNLLLNAKCDLKIGDFGLARTTTETDFMMEYVVTRWYRAPELLLNCSEYTGAIDMWS

VGCILGEIATREPLFPGKDYVHQLRLITELLGSPDDTSLGFLRSDNARRYVRSLPQYPKQ

QFRSRFPNMSSGAMDLLERMLVFDPNKRITVDEALCHPYLASLHEINDEPVCPAPFSFDF

EQPSFTEEDIKELIWRESVKFNPEPIH*

>Jer1MPK14 Brdisv1Jer11028958m.p

MAMLVDPPNGMGNQGKHYYSMWQTLFEIDTKYVPIKPIGRGAYGIVCSSINRETNEKVAI

KKIHNVFDNRVDALRTLRELKLLRHLRHENVISLKDIMMPVQRRSFKDVYLVYELMDTDL

HQIIKSPQGLSNDHCQYFLFQLLRGLKYLHSAEILHRDLKPGNLLVNANCDLKICDFGLA

RTNSSKGQFMTEYVVTRWYRAPELLLCCDNYGTSIDVWSVGCIFAELLGRKPIFPGTECL

NQLKLIVNVLGTMSESDLEFIDNPKARRYIKTLPYTPGVPLASMYPHAHPLAIDLLQKML

IFDPTKRISVTEALEHPYMSPLYDPSANPPAQVPIDLDLDENISADMIREMMWQEMLHYH

PEAATAISM*

>Jer1MPK16 Brdisv1Jer11017067m.p

MDFFTEYGEGNRYKIEEVIGKGSYGVVCSALDTHTGDKVAIKKINDIFEHVSDATRILRE

IKLLRLLRHPDIVEIKHILLPPSRREFKDIYVVFELMESDLHQVIKANDDLTPEHYQFFL

YQLLRGLKYIHTANVFHRDLKPKNILANADCKLKICDFGLARVAISDTPTAIFWTDYIAT

RWYRAPELCGSFFSKYTPAIDIWSIGCIFAELLTGKPLFPGKNVVHQLDIITDLLGTPSA

ETIARIRNEKARRYLSSMRRKKTVPFTQKFPNADPLALNLLERMLAFDPKDRPSAEEALA

DPYFKNIASVDREPSAQPITKLEFEFERRRITKDDIRELIYREILEYHPNMLREFLEGAE

PTGFMYPSAVDHFKKQFTFLEEHYAKGSTAAPPERQHNSLPRPSVIYSDNRPQNAANITD

DLSRCAIRDNTQKAQKDSASVGANRIPHGAAAAAARPGKVVGSVLRYGNCSTSGTEQYEQ

RRVVRSPGIAPNGVTSGSSYPRRNNTCKSETGEAERIDTNQGGPPKPYVANKLPATVDGR

NGHW*

>Jer1MPK17 Brdisv1Jer11005346m.p

MVGGGGIVDGFRRLFHRRTPSGSGPSSNQSSAGEESSDIDAVVEDLDLVGLRAIRVPKRK

MPLPVESHKKNILEKEFFTEYGEASQYQIQEVIGKGSYGVVAAAVDTRTGERVAIKKIND

VFEHVSDATRILREVKLLRLLRHPDVVEIKHIMLPPSRREFQDIYVVFELMESDLHQVIR

ANDDLTPEHYQFFLYQLLRALKYIHAANVFHRDLKPKNILANSDCKLKICDFGLARVSFN

DAPTAIFWTDYVATRWYRAPELCGSFFSKYTPAIDIWSIGCIFAELLTGRPLFPGKNVVH

QLDIITDLLGTPSSETLSRIRNEKARRYLSCMRKKHPVPLTQKFPNVDPLALRLLGRLLA

FDPKDRPSAEEALADPYFAVLANVEREPSRNPISKLEFEFERRKVTKDDVRELIYREILE

YHPQMLQEYMQGGEQLSFLYPSGVDRFKRQFAHLEENYSKGERGSPLQRKHASLPRQRVG

ASNDNNEQHASDQERGADSIAPATGNPPGSQDAGQQHVSGGQNGVGSTNISPRSYQKSAS

ISASKCVVVNANKNPEYDDDISEEMEGAVDGLSEKVSRMHP*

>Jer1MPK20-1 Brdisv1Jer11018464m.p

MQQDQRKKSSAEAEFFTEYGDASRYKIQEIVGKGSYGVVCSAIDVHTGEKVAIKKIHDIF

EHISDAARILREIKLLRLLRHPDIVEIKHIMLPPSRRDFKDIYVVFELMESDLHQVIKAN

DDLTKEHYQFFLYQLLRALKYIHTANVYHRDLKPKNILANSNCKLKICDFGLARVAFNDT

PTTIFWTDYVATRWYRAPELCGSFFSKYTPAIDVWSIGCIFAEVLTGKPLFPGKNVVHQL

DLMTDLLGTPSMDTISRVRNEKARRYLSSMRKKEPIPFSQKFPNADPLALDLLQRLLAFD

PKDRPTAGEALSHPYFKGLAKVEREPSCQPITKMEFEFERRRATKEDIRELIFREILEYH

PQLLKDYINGTERTTFLYPSAVDQFRKQFAHLEENSESGPVVPMDRKHTSLPRSTIVHSA

PIHAKEQPRIGPSRDRPLTDESYKNPRDSEKYSGNVPRSSHHSQAPQRVPTARPGRVVGP

VLPYENVGTSHPYDPRRVAMNSGYPPQQQIPQTYGYYQTTGKSACSEPSQAERYTLHQQA

YACANSSTAPDVALDMRAPPFHQSAGPKSGSSDRLTAETNLYTRSLNGIVATTSGVAASA

HRKVSVVPYGMSQMY*

>Jer1MPK20-2 Brdisv1Jer11014497m.p

MQQPQQQGQRNKSTDVDFFSEYGDANRYRIQEVIGKGSYGVVCSAMDMQTRQKVAIKKIH

NIFDHVSDAARILREIKLLRLLRHPDVVEIKHIMLPPSRKDFKDIYVVFELMESDLHQVI

KANDDLTKEHYQFFLYQLLRALKYIHTASVYHRDLKPKNILANSNCKLKICDFGLARVAF

NDTPTTVFWTDYVATRWYRAPELCGSFFTKYTPAIDIWSIGCIFAEVLTGKPLFPGKNVV

HQLDLMTDLLGTPSMDTISRVRNEKARRYLSSMRKKDPVPFSKKFPNADPLGLKLLEKLL

AFDPKDRPTAEEALTDPYFKSLSKPDREPSCQPIRKVEFDFEHRRMSKDDIRELIFQEIL

EYHPQLLKNYIDGTEKTTFLYPSAVDQFKKQFSHLEESDGSGPVVPTERKHASLPRSTTV

HSTPIPAKEQPLVASSRGRPIANEPCKPWAPGNVPGASQTAHVAQAGRAVGVGSVPPYES

GSGKYPYDATSRPAVSSGYPPQQKIPQTYGYHHHHQTPGAGQSSQAMGGYACGYTKGTTP

PPAAAQDMRASPYHHRSAGTKNDPLNRLAAESDIYTRSLNGIVAAAASAGTGAHRKVGAV

PFGMSGMY*

>Jer1MPK20-3 Brdisv1Jer11043553m.p

MQHGDLHKKSAAEMDFFTAYDDANRYKILEVIGKGSYGLVCSANDLQTGEKVAIKKIHNI

FEHISDAARILREIKLLRLLRHPDVVEIKHILLPPSKKDFKDIYVVFELMESDLHQVIKA

NDDLTREHYQFFLYQMLRALKYMHTANVYHRDLKPKNVLANANCKLKICDFGLARVAFSD

APTTVFWTDYVATRWYRAPELCGSFYSKVMIVTIDH*

>Jer1MPK20-4 Brdisv1Jer11018679m.p

MQTTEQQRKKGSSEMDFFSEYGDANRYKIQEVIGKGSYGVVCSAIDQHTGDKVAIKKIHN

IFEHLSDAARILREIKLLRLLRHPDIVEIRHIMLPPSRRDFKDIYVVFELMDTDLHQVIK

ANDDLTKEHHQFFLYQMLRALKYIHTANVYHRDLKPKNILANANCKLKICDFGLARVAFN

DTPTTVFWTDYVATRWYRAPELCGSFFTKYSRAIDIWSIGCIFAEILTGKPLFPGKNVVH

QLDLMTDLLGTPSLDTVSRIRNEKARRYLSSMRKKQSVSFSERFPKADPAALKLLQRLLA

FDPKDRPTAEEALADPYFKGLGKVEREPSCQPITKMEFEFERKNVTKADVKELIFREILE

YHPQLLKDYMNGTEKTNFLYPSAVDNFRRQFANLEENGGKGGAVVPPDRKHVSLPRNTTV

HSTPIPPKDQKYSQVPQRIPTGRPGRVVGPVIPFENSSTMDPYSQRRVARNPVLPAAATN

LSAYAYRNSDNSERELQQELEKDRMQYHPMQRFMDAKMVSPDLRSTSYYMPKGVPKADVA

ERSALQSNMMQGIAPFNGIATVGGAFNKVSAVQYGVSRMY*

>Jer1MPK20-5 Brdisv1Jer11014639m.p

MFSDIFSREGVAFSRVALRQELLSDQWFTTAGGKGQCYLVPPGTTVFLVNSEEMGFFSEY

VDASRYKILEIIGKGSYGVVCSAIDQETGDKVAIKKIQNIFEHLSDAARILREIKLLRLL

RHPDIVQIKHIMLPPSRRDFRDIYVVFELMDTDLHQVIKANDDLTKEHYQFFLYQMLRAL

KYIHTANVYHRDLKPKNILANANCKLKICDFGLARVAFNDTPTTVFWTDYVATRWYRAPE

LCGSFFTKYSPAIDTWSIGCIFAEILTGKPLFPGKNVVHQLDLMTDLLGTPSTETISRIR

NDKARKYLSSMRRKQPIPFSEKFPNADPSALKLLERLLAFDPKDRPTAEEALAHPYFKRL

ARVEREPSCQQPISKTEFEFERRKFTKEDIKELIFREILEYHPKLLKDYMNGSEKTSFLY

PSAVDNFRRQFANLEIDGGRSGAADRKHFSLPRTTTVHSAPILPTNGPTSQVPQRIPTAR

PGRVVSSAMQTDNPSVSDRHNGRRVARDPAVPPAAAYHLKSDYSDRQHQQEFEKDRVRPD

RQRQEELEKDRMQYRPGHHSMDAKVAPEISPYMRSSPYYIPPFNGIAAVASGYSKVAAVT

RMY*

>Jer1MPK21-1 Brdisv1Jer11014541m.p

MGGLIRWLRHHRSRRVSSSSSSHLPSNTTSSSTSDLRAHSLPQHQGDHHGEVVVEWEDAA

EGPDSDPEEYIVVVLGDDEQGVVAARAPVRTKPPRVMDPGKKTSESEFFTEYGEANRYKV

SEVIGKGSYGVVAAAVDTQTGERCAIKKINDVFDHVSDATRILREIKLLRLLRHPDIVEI

KHIMLPPSRREFRDIYVVFELMESDLHQVIKANDDLTPEHHQFFLYQLLRGMKYIHTANV

FHRDLKPKNILANADCKLKICDFGLARVSFNDGAPSAIFWTDYVATRWYRAPELCGSFFS

KYTPAIDIWSVGCIFAEMLTGKPLFPGKNVVHQLDLMTDLLGTPSAESLSKIRNEKARRY

LSNMRKKPKVPLTKKFPGIDPMALHLLERLLAFDPKDRPSADEALTDPYFTGLANSEREP

ITQPISKLEFEFEKRKLAKDDVRELIYREILEYHPHMLQEYLRGGGDQMSFMFPSGVDRF

KRQFAHLEEGGAKGEKSSPQLRQNASLPRERVIGNKHGDGDYNMKLNVGEKPELASVSDG

ISKPLMSARSLLKSETMSASKCIGEIKNKDEDSLSECVEGTDDDVSQKIAQLKT*

>Jer1MPK21-2 Brdisv1Jer11018554m.p

GSDEAEFFTEYGEANRYEVGEVVGKGSYGVVAAAVDTHTGERVAIKKINDVFEHVSDATR

ILREIKLLRLLRHPDIVEIKHIMLPPSRREFRDIYIIFELMESDLHQVIKANDDLTPEHH

QFFFYQLLRGMKYIHAANVFHRDLKPRNILANADCKLKICDFGLARVSFNDTPSAIFWTD

YVATRWYRAPELCGSFFSKYTPAIDIWSIGCIFAEMLSGRPLFPGKNVVHQLDLMTDLLG

TPSAESLSRIRNEKARRYLGNMRKKHPVPFSQKFPGVDPMALDLLERLLAFDPKDRPTAA

EALADPYFTGLANSDREPTTQPISKLEFEFERRKLARDDVRELIYREILEYHPQMLHEYH

HGGDQANFVYPSGVDRFKRQFVHLEEGVTKGEKTSPQLRQHASLPRERIIGIGDELGRPN

ADYCIKLHVGEEPGHTSVTDGLSKPLLNARNFLKSESISASQCVVIKEKREKDVSAVTSN

IF*

>Per1MPK3 Brdisv1Per11011066m.p

MDGAPVAEFRPTMTHGGRFLLYNIFGNQFEITAKYQPPIMPIGRGAYGIVCSVMNFETRE

MVAIKKIANAFDNNMDAKRTLREIKLLRHLDHENIVGLRDVIPPAIPQSFNDVYIATELM

DTDLHHIIRSNQELSEEHCQYFLYQLLRGLKYIHSANVIHRDLKPSNLLLNANCDLKICD

FGLARPSSESDMMTEYVVTRWYRAPELLLNSTDYSAAIDVWSVGCIFMELINRAPLFPGR

DHMHQMRLITEVIGTPTDDDLGFIRNEDARRYMRHLPQFPRRPFPAQFPRVQPAALDLIE

RMLAFNPLQRITVEEALEHPYLERLHDIADEPICTDPFSFDFEQHPLTEDQMKQLIFNEA

LELNPNFRY*

>Per1MPK4 Brdisv1Per11028526m.p

MDTSGGGGGGAGGAAQIQGMATHGGRYVLYNVYGNLFEVASKYAPPIRPIGRGAYGIVCA

AVSSDTGEEVAIKKIGNAFDNHIDAKRTLREIKLLRHMDHENIIAIKDIIRPPRRDDFKD

VYIVTELMDTDLHQIIRSNQPLTDDHCQYFLYQLLRGLKYVHSANVLHRDLKPSNLFLNA

NCDLKIADFGLARTTSETDLMTEYVVTRWYRAPELLLNCSQYTAAIDVWSVGCILGEIIT

RQPLFPGRDYIQQLKLITELIGSPDDSSLGFLRSDNARRYMKQLPQYPRQDFRLRFRNMS

DGAVDLLERMLVFDPSRRITVDEALHHPYLASLHDINEEPTCPAPFSFDFEQPSFTEEHM

KELIWRETLAFNPDPPY*

>Per1MPK6 Brdisv1Per11008140m.p

MDGGAQPPDAEMADAGAAVPGAGAGAAGTMENIQATLSHGGRFIQYNIFGNVFEVTVKYK

PPILPIGKGAYGIVCSALNSETGEQVAIKKIANAFDNKIDAKRTLREIKLLRHMDHENIV

AIRDIIPPAQRNSFNDVYIAYELMDTDLHQIIRSNQALSEEHCQYFLYQILRGLKYIHSA

NVLHRDLKPSNLLLNANCDLKICDFGLARTTSETDFMTEYVVTRWYRAPELLLNSSEYTA

AIDVWSVGCIFMELMDRKPLFPGRDHVHQLRLLMELIGTPNEADLDFVNENARRYIRQLP

RHARQSFSEKFPHVHPSAIDLVEKMLTFDPRQRITVEGALAHPYLASLHDISDEPVCTMP

FSFDFEQHALSEEQMKDLIHQEALAFNPDYQ*

>Per1MPK11 Brdisv1Per11026363m.p

MRMEGGGAGPAAAAAAGGAHGLGEAQIKGTLTHGGRYVQYNVYGNLFEVSAKYVPPIRPV

GRGACGIICAAINVQTREEVAIKKIGNAFDNQIDAKRTLREVKLLRHMNHENVISIKDII

RPPRRENFNDVYIVYELMDTDLHHLLRSNQPLTDDHCQYFLYQVLRGLKYVHSANVLHRD

LRPSNLLLNAKCDLKIGDFGLARTTTETDFMMEYVVTRWYRAPELLLNCSEYTGAIDMWS

VGCILGEIATREPLFPGKDYVHQLRLITELLGSPDDTSLGFLRSDNARRYVRSLPQYPKQ

QFRSRFPNMSSGAMDLLERMLVFDPNKRITVDEALCHPYLASLHEINDEPVCPAPFSFDF

EQPSFTEEDIKELIWRESVKFNPEPIH*

>Per1MPK14 Brdisv1Per11024087m.p

MAMLVDPPNGMGNQGKHYYSMWQTLFEIDTKYVPIKPIGRGAYGIVCSSINRETNEKVAI

KKIHNVFDNRVDALRTLRELKLLRHLRHENVISLKDIMMPVQRRSFKDVYLVYELMDTDL

HQIIKSPQGLSNDHCQYFLFQLLRGLKYLHSAEILHRDLKPGNLLVNANCDLKICDFGLA

RTNSSKGQFMTEYVVTRWYRAPELLLCCDNYGTSIDVWSVGCIFAELLGRKPIFPGTECL

NQLKLIVNVLGTMSESDLEFIDNPKARRYIKTLPYTPGVPLASMYPHAHPLAIDLLQKML

IFDPTKRISVTEALEHPYMSPLYDPSANPPAQVPIDLDLDENISADMIREMMWQEMLHYH

PEAATAISM*

>Per1MPK16 Brdisv1Per11018807m.p

MDFFTEYGEGNRYKIEEVIGKGSYGVVCSALDTHTGDKVAIKKINDIFEHVSDATRILRE

IKLLRLLRHPDIVEIKHILLPPSRREFKDIYVVFELMESDLHQVIKANDDLTPEHYQFFL

YQLLRGLKYIHTANVFHRDLKPKNILANADCKLKICDFGLARVAISDTPTAIFWTDYIAT

RWYRAPELCGSFFSKYTPAIDIWSIGCIFAELLTGKPLFPGKNVVHQLDIITDLLGTPSA

ETIARIRNEKARRYLSSMRRKKTVPFTQKFPNADPLALNLLERMLAFDPKDRPSAEEALA

DPYFKNIASVDREPSAQPITKLEFEFERRRITKDDIRELIYREILEYHPNMLREFLEGAE

PTGFMYPSAVDHFKKQFTFLEEHYAKGSTAAPPERQHNSLPRPSVIYSDNRPQNAANITD

DLSRCAIRDNTQKAQKDSASVGANRIPHGAAAAAARPGKVVGSVLRYGNCSTSGTEQYEQ

RRVVRSPGIAPNGVTSGSSYPRRNNTCKSETGEAERIDTNQGGPPKPYVANKLPATVDGR

NGHW*

>Per1MPK17 Brdisv1Per11005829m.p

MVGGGGIVDGFRRLFHRRTPSGSGPSSNQSSAGEESSDIDAVVEDLDLVGLRAIRVPKRK

MPLPVESHKKNILEKEFFTEYGEASQYQIQEVIGKGSYGVVAAAVDTRTGERVAIKKIND

VFEHVSDATRILREVKLLRLLRHPDVVEIKHIMLPPSRREFQDIYVVFELMESDLHQVIR

ANDDLTPEHYQFFLYQLLRALKYIHAANVFHRDLKPKNILANSDCKLKICDFGLARVSFN

DAPTAIFWTDYVATRWYRAPELCGSFFSKYTPAIDIWSIGCIFAELLTGRPLFPGKNVVH

QLDIITDLLGTPSSETLSRIRNEKARRYLSCMRKKHPVPLTQKFPNVDPLALRLLGRLLA

FDPKDRPSAEEALADPYFAVLANVEREPSRNPISKLEFEFERQKVTKDDVRELIYREILE

YHPQMLQEYMQGGEQLSFLYPSGVDRFKRQFAHLEENYSKGERGSPLQRKHASLPRQRVG

ASNDNNEQHASDQERGADSIAPATGNPPGSQDAGQQHVSGGQNGVGSTNISPRSYQKSAS

ISASKCVVVNANKNPEYDDDISEEMEGAVDGLSEKVSRMHP*

>Per1MPK20-1 Brdisv1Per11020227m.p

MQQDQRKKSSAEAEFFTEYGDASRYKIQEIVGKGSYGVVCSAIDVHTGEKVAIKKIHDIF

EHISDAARILREIKLLRLLRHPDIVEIKHIMLPPSRRDFKDIYVVFELMESDLHQVIKAN

DDLTKEHYQFFLYQLLRALKYIHTANVYHRDLKPKNILANSNCKLKICDFGLARVAFNDT

PTTIFWTDYVATRWYRAPELCGSFFSKYTPAIDVWSIGCIFAEVLTGKPLFPGKNVVHQL

DLMTDLLGTPSMDTISRVRNEKARRYLSSMRKKEPIPFSQKFPNADPLALDLLQRLLAFD

PKDRPTAGEALSHPYFKGLAKVEREPSCQPITKMEFEFERRRATKEDIRELIFREILEYH

PQLLKDYINGTERTTFLYPSAVDQFRKQFAHLEENSESGPVVPMDRKHTSLPRSTIVHSA

PIHAKEQPRIGPSRDRPLTDESYKNPRDSEKYSGNVPRSSHHSQAPQRVPTARPGRVVGP

VLPYENVGTSHPYDPRRVAMNSGYPPQQQIPQTYGYYQTTGKSACSEPSQAERYTLHQQA

YACANSSTAPDVALDMRAPPFHQSAGPKSGSSDRLTAETNLYTRSLNGIVATTSGVAASA

HRKVSVVPYGMSQMY*

>Per1MPK20-2 Brdisv1Per11015923m.p

MQQPQQQGQRNKSTDVDFFSEYGDANRYRIQEVIGKGSYGVVCSAMDMQTRQKVAIKKIH

NIFDHVSDAARILREIKLLRLLRHPDVVEIKHIMLPPSRKDFKDIYVVFELMESDLHQVI

KANDDLTKEHYQFFLYQLLRALKYIHTASVYHRDLKPKNILANSNCKLKICDFGLARVAF

NDTPTTVFWTDYVATRWYRAPELCGSFFTKYTPAIDIWSIGCIFAEVLTGKPLFPGKNVV

HQLDLMTDLLGTPSMDTISRVRNEKARRYLSSMRKKDPVPFSKKFPNADPLGLKLLEKLL

AFDPKDRPTAEEALTDPYFKSLSKPDREPSCQPIRKVEFDFEHRRMSKDDIRELIFQEIL

EYHPQLLKNYIDGTEKTTFLYPSAVDQFKKQFSHLEESDGSGPVVQTERKHASLPRSTTV

HSTPIPAKEQPLVASSRGRPIANEPCKPWAPGNVPGASQTAHVAQAGRAVGVGSVPPYES

GSGKYPYDATSRPAVSSGYPPQQKIPQTYGYHHHHQTPGAGQSSQAMGGYACGYTKGTTP

PPAAAQDMRASPYHHRSAGTKNDPLNRLAAESDIYTRSLNGIVAAAASAGTGAHRKVGAV

PFGMSGMY*

>Per1MPK20-3 Brdisv1Per11045486m.p

MQHGDLHKKSAAEMDFFTAYDDANRYKILEVIGKGSYGLVCSANDLQTGEKVAIKKIHNI

FEHISDAARILREIKLLRLLRHPDVVEIKHILLPPSKKDFKDIYVVFELMESDLHQVIKA

NDDLTREHYQFFLYQMLRALKYMHTANVYHRDLKPKNVLANANCKLKICDFGLARVAFSD

APTTVFWTDYVATRWYRAPELCGSFYSK

>Per1MPK20-4 Brdisv1Per11020434m.p

MQTTEQQRKKGSSEMDFFSEYGDANRYKIQEVIGKGSYGVVCSAIDQHTGDKVAIKKIHN

IFEHLSDAARILREIKLLRLLRHPDIVEIRHIMLPPSRRDFKDIYVVFELMDTDLHQVIK

ANDDLTKEHHQFFLYQMLRALKYIHTANVYHRDLKPKNILANANCKLKICDFGLARVAFN

DTPTTVFWTDYVATRWYRAPELCGSFFTKYSRAIDIWSIGCIFAEILTGKPLFPGKNVVH

QLDLMTDLLGTPSLDTVSRIRNEKARRYLSSMRKKQSVSFSERFPKADPAALKLLQRLLA

FDPKDRPTAEEALADPYFKGLGKVEREPSCQPITKMEFEFERKNVTKADVKELIFREILE

YHPQLLKDYMNGTEKTNFLYPSAVDNFRRQFANLEENGGKGGAVVPPDRKHVSLPRNTTV

HSTPIPPKDQKYSQVPQRIPTGRPGRVVGPVIPFENSSTMDPYSQRRVARNPVLPAAATN

LSAYAYRNSDNSERELQQELEKDRMQYHPMQRFMDAKMVSPDLRSTSYYMPKGVPKADVA

ERSALQSNMMQGIAPFNGIATVGGAFNKVSAVQYGVSRMY*

>Per1MPK20-5 Brdisv1Per11016073m.p

MFSDIFSREGVAFSRVALRQELLSDQWFTTAGGKGQCYLVPPGTTVFLVNSEEMGFFSEY

VDASRYKILEIIGKGSYGVVCSAIDQETGDKVAIKKIQNIFEHLSDAARILREIKLLRLL

RHPDIVQIKHIMLPPSRRDFRDIYVVFELMDTDLHQVIKANDDLTKEHYQFFLYQMLRAL

KYIHTANVYHRDLKPKNILANANCKLKICDFGLARVAFNDTPTTVFWTDYVATRWYRAPE

LCGSFFTKYSPAIDTWSIGCIFAEILTGKPLFPGKNVVHQLDLMTDLLGTPSTETISRIR

NDKARKYLSSMRRKQPIPFSEKFPNADPSALKLLERLLAFDPKDRPTAEEALAHPYFKRL

ARVEREPSCQQPISKTEFEFERRKFTKEDIKELIFREILEYHPKLLKDYMNGSEKTSFLY

PSAVDNFRRQFANLEIDGGRSGAADRKHFSLPRTTTVHSAPILPTNGPTSQVPQRIPTAR

PGRVVSSAMQTDNPSVSDRHNGRRVARDPAVPPAAAYHLKSDYSDRQHQQEFEKDRVRPD

RQRQEELEKDRMQYRPGHHSMDAKVAPEISPYMRSSPYYIPPFNGIAAVASGYSKVAAVT

RMY*

>Per1MPK21-1 Brdisv1Per11015968m.p

MGGLIRWLRHHRSRRVSSSSSSHLPSNTTSSSTSDLRAHSLPQHQGDHHGEVVVEWEDAA

EGPDSDPEEYIVVVLGDDEQGVVAARAPVRTKPPRVMDPGKKTSESEFFTEYGEANRYKV

SEVIGKGSYGVVAAAVDTQTGERCAIKKINDVFDHVSDATRILREIKLLRLLRHPDIVEI

KHIMLPPSRREFRDIYVVFELMESDLHQVIKANDDLTPEHHQFFLYQLLRGMKYIHTANV

FHRDLKPKNILANADCKLKICDFGLARVSFNDGAPSAIFWTDYVATRWYRAPELCGSFFS

KYTPAIDIWSVGCIFAEMLTGKPLFPGKNVVHQLDLMTDLLGTPSAESLSKIRNEKARRY

LSNMRKKPKVPLTKKFPGIDPMALHLLERLLAFDPKDRPSADEALTDPYFTGLANSEREP

ITQPISKLEFEFEKRKLAKDDVRELIYREILEYHPHMLQEYLRGGGDQMSFMFPSGVDRF

KRQFAHLEEGGAKGEKSSPQLRQNASLPRERVIGNKHGDGDYNMKLNVGEKPELASVSDG

ISKPLMSARSLLKSETMSASKCIGEIKNKDEDSLSECVEGTDDDVSQKIAQLKT*

>Per1MPK21-2 Brdisv1Per11006952m.p

GSDEAEFFTEYGEANRYEVGEVVGKGSYGVVAAAVDTHTGERVAIKKINDVFEHVSDATR

ILREIKLLRLLRHPDIVEIKHIMLPPSRREFRDIYIIFELMESDLHQVIKANDDLTPEHH

QFFFYQLLRGMKYIHAANVFHRDLKPRNILANADCKLKICDFGLARVSFNDTPSAIFWTD

YVATRWYRAPELCGSFFSKYTPAIDIWSIGCIFAEMLSGRPLFPGKNVVHQLDLMTDLLG

TPSAESLSRIRNEKARRYLGNMRKKHPVPFSQKFPGVDPMALDLLERLLAFDPKDRPTAA

EALADPYFTGLANSDREPTTQPISKLEFEFERRKLARDDVRELIYREILEYHPQMLHEYH

HGGDQANFVYPSGVDRFKRQFVHLEEGVTKGEKTSPQLRQHASLPRERIIGIGDELGRPN

ADYCIKLHVGEEPGHTSVTDGLSKPLLNARNFLKSESISASQCVVIKEKREKDVSAVTSN

IF*

>ABR6MPK3 Brdisv1ABR6_r1011083m.p

MDGAPVAEFRPTMTHGGRFLLYNIFGNQFEITAKYQPPIMPIGRGAYGIVCSVMNFETRE

MVAIKKIANAFDNNMDAKRTLREIKLLRHLDHENIVGLRDVIPPAIPQSFNDVYIATELM

DTDLHHIIRSNQELSEEHCQYFLYQLLRGLKYIHSANVIHRDLKPSNLLLNANCDLKICD

FGLARPSSESDMMTEYVVTRWYRAPELLLNSTDYSAAIDVWSVGCIFMELINRAPLFPGR

DHMHQMRLITEVIGTPTDDDLGFIRNEDARRYMRHLPQFPRRPFPAQFPRVQPAALDLIE

RMLAFNPLQRITVEEALEHPYLERLHDIADEPICTDPFSFDFEQHPLTEDQMKQLIFNEA

LELNPNFRY*

>ABR6MPK4 Brdisv1ABR6_r1036097m.p

MDTSGGGGGGAGGAAQIQGMATHGGRYVLYNVYGNLFEVASKYAPPIRPIGRGAYGIVCA

AVSSDTGEEVAIKKIGNAFDNHIDAKRTLREIKLLRHMDHENIIAIKDIIRPPRRDDFKD

VYIVTELMDTDLHQIIRSNQPLTDDHCQYFLYQLLRGLKYVHSANVLHRDLKPSNLFLNA

NCDLKIADFGLARTTSETDLMTEYVVTRWYRAPELLLNCSQYTAAIDVWSVGCILGEIIT

RQPLFPGRDYIQQLKLITELIGSPDDSSLGFLRSDNARRYMKQLPQYPRQDFRLRFRNMS

DGAVDLLERMLVFDPSRRITVDEALHHPYLASLHDINEEPTCPAPFSFDFEQPSFTEEHM

KELIWRETLAFNPDPPY*

>ABR6MPK6 Brdisv1ABR6_r1007995m.p

MDGGAQPPDAEMADAGAAVPGAGAGAAGTMENIQATLSHGGRFIQYNIFGNVFEVTVKYK

PPILPIGKGAYGIVCSALNSETGEQVAIKKIANAFDNKIDAKRTLREIKLLRHMDHENIV

AIRDIIPPAQRNSFNDVYIAYELMDTDLHQIIRSNQALSEEHCQYFLYQILRGLKYIHSA

NVLHRDLKPSNLLLNANCDLKICDFGLARTTSETDFMTEYVVTRWYRAPELLLNSSEYTA

AIDVWSVGCIFMELMDRKPLFPGRDHVHQLRLLMELIGTPNEADLDFVNENARRYIRQLP

RHARQSFSEKFPHVHPSAIDLVEKMLTFDPRQRITVEGALAHPYLASLHDISDEPVCTMP

FSFDFEQHALSEEQMKDLIHQEALAFNPDYQ*

>ABR6MPK7-1 Brdisv1ABR6_r1045951m.p

MLAGKANKKELLRGLKYLHSAGILHRDLNPGNLLVNANCDLKICDFGLARTNNTKGQFMT

EYVVTRLYRAPELLLCCDNYGTSIDVWSVGCIFAELLGRKPIFPGTECLNQLKLIVNVLG

TMSENDLEFIDNPKARKYIKSLPYTPGTPLTSMYPQAHPLAIDLLQKMLVFDPSKRISVT

EALEHPYMSPLYDPSANPPAQVPIDLDIDENLGVEMIREMLWQEMLQYHPEAARMVNM*

>ABR6MPK11 Brdisv1ABR6_r1033960m.p

MRMEGGGAGPAAAAAAGGAHGLGEAQIKGTLTHGGRYVQYNVYGNLFEVSAKYVPPIRPV

GRGACGIICAAINVQTREEVAIKKIGNAFDNQIDAKRTLREVKLLRHMNHENVISIKDII

RPPRRENFNDVYIVYELMDTDLHHLLRSNQPLTDDHCQYFLYQVLRGLKYVHSANVLHRD

LRPSNLLLNAKCDLKIGDFGLARTTTETDFMMEYVVTRWYRAPELLLNCSEYTGAIDMWS

VGCILGEIATREPLFPGKDYVHQLRLITELLGSPDDTSLGFLRSDNARRYVRSLPQYPKQ

QFRSRFPNMSSGAMDLLERMLVFDPNKRITVDEALCHPYLASLHEINDEPVCPAPFSFDF

EQPSFTEEDIKELIWRESVKFNPEPIH*

>ABR6MPK14 Brdisv1ABR6_r1031745m.p

MAMLVDPPNGMGNQGKHYYSMWQTLFEIDTKYVPIKPIGRGAYGIVCSSINRETNEKVAI

KKIHNVFDNRVDALRTLRELKLLRHLRHENVISLKDIMMPVQRRSFKDVYLVYELMDTDL

HQIIKSPQGLSNDHCQYFLFQLLRGLKYLHSAEILHRDLKPGNLLVNANCDLKICDFGLA

RTNSSKGQFMTEYVVTRWYRAPELLLCCDNYGTSIDVWSVGCIFAELLGRKPIFPGTECL

NQLKLIVNVLGTMSESDLEFIDNPKARRYIKTLPYTPGVPLASMYPHAHPLAIDLLQKML

IFDPTKRISVTEALEHPYMSPLYDPSANPPAQVPIDLDLDENISADMIREMMWQEMLHYH

PEAATAISM*

>ABR6MPK16 Brdisv1ABR6_r1018869m.p

MDFFTEYGEGNRYKIEEVIGKGSYGVVCSALDTHTGDKVAIKKINDIFEHVSDATRILRE

IKLLRLLRHPDIVEIKHILLPPSRREFKDIYVVFELMESDLHQVIKANDDLTPEHYQFFL

YQLLRGLKYIHTANVFHRDLKPKNILANADCKLKICDFGLARVAISDTPTAIFWTDYIAT

RWYRAPELCGSFFSKYTPAIDIWSIGCIFAELLTGKPLFPGKNVVHQLDIITDLLGTPSA

ETIARIRNEKARRYLSSMRRKKTVPFTQKFPNADPLALNLLERMLAFDPKDRPSAEEALA

DPYFKNIASVDREPSAQPITKLEFEFERRRITKDDIRELIYREILEYHPNMLREFLEGAE

PTGFMYPSAVDHFKKQFTFLEEHYAKGSTAAPPERQHNSLPRPSVIYSDNRPQNAANITD

DLSRCAIRDNTQKAQKDSASVGANRIPHGAAAAAARPGKVVGSVLRYGNCSTSGTEQYEQ

RRVVRSPGIAPNGVTSGSSYPRRNNTCKSETGEAERIDTNQGGPPKPYVANKLPATVDGR

NGHW*

>ABR6MPK17 Brdisv1ABR6_r1005816m.p

MVGGGGIVDGFRRLFHRRTPSGSGPSSNQSSAGEESSDIDAVVEDLDLVGLRAIRVPKRK

MPLPVESHKKNILEKEFFTEYGEASQYQIQEVIGKGSYGVVAAAVDTRTGERVAIKKIND

VFEHVSDATRILREVKLLRLLRHPDVVEIKHIMLPPSRREFQDIYVVFELMESDLHQVIR

ANDDLTPEHYQFFLYQLLRALKYIHAANVFHRDLKPKNILANSDCKLKICDFGLARVSFN

DAPTAIFWTDYVATRWYRAPELCGSFFSKYTPAIDIWSIGCIFAELLTGRPLFPGKNVVH

QLDIITDLLGTPSSETLSRIRNEKARRYLSCMRKKHPVPLTQKFPNVDPLALRLLGRLLA

FDPKDRPSAEEALADPYFAVLANVEREPSRNPISKLEFEFERQKVTKDDVRELIYREILE

YHPQMLQEYMQGGEQLSFLYPSGVDRFKRQFAHLEENYSKGERGSPLQRKHASLPRQRVG

ASNDNNEQHASDQERGADSIAPATGNPPGSQDAGQQHVSGGQNGVGSTNISPRSYQKSAS

ISASKCVVVNANKNPEYDDDISEEMEGAVDGLSEKVSRMHP*

>ABR6MPK20-1 Brdisv1ABR6_r1020353m.p

MQQDQRKKSSAEAEFFTEYGDASRYKIQEIVGKGSYGVVCSAIDVHTGEKVAIKKIHDIF

EHISDAARILREIKLLRLLRHPDIVEIKHIMLPPSRRDFKDIYVVFELMESDLHQVIKAN

DDLTKEHYQFFLYQLLRALKYIHTANVYHRDLKPKNILANSNCKLKICDFGLARVAFNDT

PTTIFWTDYVATRWYRAPELCGSFFSKYTPAIDVWSIGCIFAEVLTGKPLFPGKNVVHQL

DLMTDLLGTPSMDTISRVRNEKARRYLSSMRKKEPIPFSQKFPNADPLALDLLQRLLAFD

PKDRPTAGEALSHPYFKGLAKVEREPSCQPITKMEFEFERRRATKEDIRELIFREILEYH

PQLLKDYINGTERTTFLYPSAVDQFRKQFAHLEENSESGPVVPMDRKHTSLPRSTIVHSA

PIHAKEQPRIGPSRDRPLTDESYKNPRDSEKYSGNVPRSSHHSQAPQRVPTARPGRVVGP

VLPYENVGTSHPYDPRRVAMNSGYPPQQQIPQTYGYYQTTGKSACSEPSQAERYTLHQQA

YACANSSTAPDVALDMRAPPFHQSAGPKSGSSDRLTAETNLYTRSLNGIVATTSGVAASA

HRKVSVVPYGMSQMY*

>ABR6MPK20-2 Brdisv1ABR6_r1016022m.p

MQQPQQQGQRNKLPDGWFCCNVHILNLCRSAMIFLTPPIIMPSTDVDFFSEYGDANRYRI

QEVIGKGSYGVVCSAMDMQTRQKVAIKKIHNIFDHVSDAARILREIKLLRLLRHPDVVEI

KHIMLPPSRKDFKDIYVVFELMESDLHQVIKANDDLTKEHYQFFLYQLLRALKYIHTASV

YHRDLKPKNILANSNCKLKICDFGLARVAFNDTPTTVFWTDYVATRWYRAPELCGSFFTK

YTPAIDIWSIGCIFAEVLTGKPLFPGKNVVHQLDLMTDLLGTPSMDTISRVRNEKARRYL

SSMRKKDPVPFSKKFPNADPLGLKLLEKLLAFDPKDRPTAEEALTDPYFKSLSKPDREPS

CQPIRKVEFDFEHRRMSKDDIRELIFQEILEYHPQLLKNYIDGTEKTTFLYPSAVDQFKK

QFSHLEESDGSGPVVQTERKHASLPRSTTVHSTPIPAKEQPLVASSRGRPIANEPCKPWA

PGNVPGASQTAHVAQAGRAVGVGSVPPYESGSGKYPYDATSRPAVSSGYPPQQKIPQTYG

YHHHHQTPGAGQSSQAMGGYACGYTKGTTPPPAAAQDMRASPYHHRSAGTKNDPLNRLAA

ESDIYTRSLNGIVAAAASAGTGAHRKVGAVPFGMSGMY*

>ABR6MPK20-3 Brdisv1ABR6_r1006657m.p

MQHGDLHKKSAAEMDFFTAYDDANRYKILEVIGKGSYGLVCSANDLQTGEKVAIKKIHNI

FEHISDAARILREIKLLRLLRHPDVVEIKHILLPPSKKDFKDIYVVFELMESDLHQVIKA

NDDLTREHYQFFLYQMLRALKYMHTANVYHRDLKPKNVLANANCKLKICDFGLARVAFSD

APTTVFWTDYVATRWYRAPELCGSFYSKYTPAIDIWSIGCIFAEVLIGKPLFPGKNVVHQ

LDLITDILGTPSLDAISRVRNDKARKYLTCMRKKQPASFSQKFPKADPLALQLLRRLLAF

DPKDRPSAEEALADPYFNGLAKVEREPSCQPIPKIEFEFEGRRVTKEDIKELIFEEILEY

HPQLLKEHIIGKERPNFVHLSAVDQFKKHFTQLEENDNETGAAVSLQRKHSSLPRQAFNH

R*

>ABR6MPK20-4 Brdisv1ABR6_r1020616m.p

MQTTEQQRKKGSSEMDFFSEYGDANRYKIQEVIGKGSYGVVCSAIDQHTGDKVAIKKIHN

IFEHLSDAARILREIKLLRLLRHPDIVEIRHIMLPPSRRDFKDIYVVFELMDTDLHQVIK

ANDDLTKEHHQFFLYQMLRALKYIHTANVYHRDLKPKNILANANCKLKICDFGLARVAFN

DTPTTVFWTDYVATRWYRAPELCGSFFTKYSRAIDIWSIGCIFAEILTGKPLFPGKNVVH

QLDLMTDLLGTPSLDTVSRIRNEKARRYLSSMRKKQSVSFSERFPKADPAALKLLQRLLA

FDPKDRPTAEEALADPYFKGLGKVEREPSCQPITKMEFEFERKNVTKADVKELIFREILE

YHPQLLKDYMNGTEKTNFLYPSAVDNFRRQFANLEENGGKGGAVVPPDRKHVSLPRNTTV

HSTPIPPKDQKYSQVPQRIPTGRPGRVVGPVIPFENSSTMDPYSQRRVARNPVLPAAATN

LSAYAYRNSDNSERELQQELEKDRMQYHPMQRFMDAKMVSPDLRSTSYYMPKGVPKADVA

ERSALQSNMMQGIAPFNGIATVGGAFNKVSAVQYGVSRMY*

>ABR6MPK20-5 Brdisv1ABR6_r1016151m.p

MFSDIFSREGVAFSRVALRQELLSDQWFTTAGGKGQCYLVPPGTTVFLVNSEEMGFFSEY

VDASRYKILEIIGKGSYGVVCSAIDQETGDKVAIKKIQNIFEHLSDAARILREIKLLRLL

RHPDIVQIKHIMLPPSRRDFRDIYVVFELMDTDLHQVIKANDDLTKEHYQFFLYQMLRAL

KYIHTANVYHRDLKPKNILANANCKLKICDFGLARVAFNDTPTTVFWTDYVATRWYRAPE

LCGSFFTKYSPAIDTWSIGCIFAEILTGKPLFPGKNVVHQLDLMTDLLGTPSTETISRIR

NDKARKYLSSMRRKQPIPFSEKFPNADPSALKLLERLLAFDPKDRPTAEEALAHPYFKRL

ARVEREPSCQQPISKTEFEFERRKFTKEDIKELIFREILEYHPKLLKDYMNGSEKTSFLY

PSAVDNFRRQFANLEIDGGRSGAADRKHFSLPRTTTVHSAPILPTNGPTSQVPQRIPTAR

PGRVVSSAMQTDNPSVSDRHNGRRVARDPAVPPAAAYHLKSDYSDRQHQQEFEKDRVRPD

RQRQEELEKDRMQYRPGHHSMDAKVAPEISPYMRSSPYYIPPFNGIAAVASGYSKVAAVT

RMY*

>ABR6MPK21-1 Brdisv1ABR6_r1029174m.p

MGGLIRWLRHHRSRRVSSSSSSHLPSNTTSSSTSDLRAHSLPQHQGDHHGEVVVEWEDAA

EGPDSDPEEYIVVVLGDDEQGVVAARAPVRTKPPRVMDPGKKTSESEFFTEYGEANRYKV

SEVIGKGSYGVVAAAVDTQTGERCAIKKINDVFDHVSDATRILREIKLLRLLRHPDIVEI

KHIMLPPSRREFRDIYVVFELMESDLHQVIKANDDLTPEHHQFFLYQLLRGMKYIHTANV

FHRDLKPKNILANADCKLKICDFGLARVSFNDGAPSAIFWTDYVATRWYRAPELCGSFFS

KYTPAIDIWSVGCIFAEMLTGKPLFPGKNVVHQLDLMTDLLGTPSAESLSKIRNEKARRY

LSNMRKKPKVPLTKKFPGIDPMALHLLERLLAFDPKDRPSADEALTDPYFTGLANSEREP

ITQPISKLEFEFEKRKLAKDDVRELIYREILEYHPHMLQEYLRGGGDQMSFMFPSGVDRF

KRQFAHLEEGGAKGEKSSPQLRQNASLPRERVIGNKHGDGDYNMKLNVGEKPELASVSDG

ISKPLMSARSLLKSETMSASKCIGEIKNKDEDSLSECVEGTDDDVSQKIAQLKT*

>ABR6MPK21-2 Brdisv1ABR6_r1020438m.p

MLPPSRREFRDIYIIFELMESDLHQVIKANDDLTPEHHQFFFYQLLRGMKYIHAANVFHR

DLKPRNILANADCKLKICDFGLARVSFNDTPSAIFWTDYVATRWYRAPELCGSFFSKYTP

AIDIWSIGCIFAEMLSGRPLFPGKNVVHQLDLMTDLLGTPSAESLSRIRNEKARRYLGNM

RKKHPVPFSQKFPGVDPMALDLLERLLAFDPKDRPTAAEALADPYFTGLANSDREPTTQP

ISKLEFEFERRKLARDDVRELIYREILEYHPQMLHEYHHGGDQANFVYPSGVDRFKRQFV

HLEEGVTKGEKTSPQLRQHASLPRERIIGIGDELGRPNADYCIKLHVGEEPGHTSVTDGL

SKPLLNARNFLKSESISASQCVCHQREARKRCKCCYIKYILNQIVSKIIN*

>Luc1MPK3 Brdisv1Luc11010913m.p

MDGAPVAEFRPTMTHGGRFLLYNIFGNQFEITAKYQPPIMPIGRGAYGIVCSVMNFETRE

MVAIKKIANAFDNNMDAKRTLREIKLLRHLDHENIVGLRDVIPPAIPQSFNDVYIATELM

DTDLHHIIRSNQELSEEHCQYFLYQLLRGLKYIHSANVIHRDLKPSNLLLNANCDLKICD

FGLARPSSESDMMTEYVVTRWYRAPELLLNSTDYSAAIDVWSVGCIFMELINRAPLFPGR

DHMHQMRLITEVIGTPTDDDLGFIRNEDARRYMRHLPQFPRRPFPAQFPRVQPAALDLIE

RMLAFNPLQRITVEEALEHPYLERLHDIADEPICTDPFSFDFEQHPLTEDQMKQLIFNEA

LELNPNFRY*

>Luc1MPK4 Brdisv1Luc11028728m.p

MDTSGGGGGGAGGAAQIQGMATHGGRYVLYNVYGNLFEVASKYAPPIRPIGRGAYGIVCA

AVSSDTGEEVAIKKIGNAFDNHIDAKRTLREIKLLRHMDHENIIAIKDIIRPPRRDDFKD

VYIVTELMDTDLHQIIRSNQPLTDDHCQYFLYQLLRGLKYVHSANVLHRDLKPSNLFLNA

NCDLKIADFGLARTTSETDLMTEYVVTRWYRAPELLLNCSQYTAAIDVWSVGCILGEIIT

RQPLFPGRDYIQQLKLITELIGSPDDSSLGFLRSDNARRYMKQLPQYPRQDFRLRFRNMS

DGAVDLLERMLVFDPSRRITVDEALHHPYLASLHDINEEPTCPAPFSFDFEQPSFTEEHM

KELIWRETLAFNPDPPY*

>Luc1MPK6 Brdisv1Luc11008002m.p

MDGGAQPPDAEMADAGAAVPGAGAGAAGTMENIQATLSHGGRFIQYNIFGNVFEVTVKYK

PPILPIGKGAYGIVCSALNSETGEQVAIKKIANAFDNKIDAKRTLREIKLLRHMDHENIV

AIRDIIPPAQRNSFNDVYIAYELMDTDLHQIIRSNQALSEEHCQYFLYQILRGLKYIHSA

NVLHRDLKPSNLLLNANCDLKICDFGLARTTSETDFMTEYVVTRWYRAPELLLNSSEYTA

AIDVWSVGCIFMELMDRKPLFPGRDHVHQLRLLMELIGTPNEADLDFVNENARRYIRQLP

RHARQSFSEKFPHVHPSAIDLVEKMLTFDPRQRITVEGALAHPYLASLHDISDEPVCTMP

FSFDFEQHALSEEQMKDLIHQEALAFNPDYQ*

>Luc1MPK7-1 Brdisv1Luc11046458m.p

MDTSDLTDSXRRDRARRRPSPHGGRAGNRGRQFSRGKPLGGSRRRRRGRRGSPRNPTAAQ

GTSAPAPGTGATVVDLDPVVEVAGAEVEPQPALTSSPAEIETVAAVDTAAAGTAPGILHR

DLNPGNLLVNANCDLKICDFGLARTNNTKGQFMTEYVVTRWYRAPELLLCCDNYGTSIDV

WSVGCIFAELLGRKPIFPGTECLNQLKLIVNVLGTMSENDLEFIDNPKARKYIKSLPYTP

GTPLTSMYPQAHPLAIDLLQKMLVFDPSKRISVTEALEHPYMSPLYDPSANPPAQVPIDL

DIDENLGVEMIREMLWQEMLQYHPEAARMVNM*

>Luc1MPK11 Brdisv1Luc11026612m.p

MRMEGGGAGPAAAAAAGGAHGLGEAQIKGTLTHGGRYVQYNVYGNLFEVSAKYVPPIRPV

GRGACGIICAAINVQTREEVAIKKIGNAFDNQIDAKRTLREVKLLRHMNHENVISIKDII

RPPRRENFNDVYIVYELMDTDLHHLLRSNQPLTDDHCQYFLYQVLRGLKYVHSANVLHRD

LRPSNLLLNAKCDLKIGDFGLARTTTETDFMMEYVVTRWYRAPELLLNCSEYTGAIDMWS

VGCILGEIATREPLFPGKDYVHQLRLITELLGSPDDTSLGFLRSDNARRYVRSLPQYPKQ

QFRSRFPNMSSGAMDLLERMLVFDPNKRITVDEALCHPYLASLHEINDEPVCPAPFSFDF

EQPSFTEEDIKELIWRESVKFNPEPIH*

>Luc1MPK14 Brdisv1Luc11024410m.p

MAMLVDPPNGMGNQGKHYYSMWQTLFEIDTKYVPIKPIGRGAYGIVCSSINRETNEKVAI

KKIHNVFDNRVDALRTLRELKLLRHLRHENVISLKDIMMPVQRRSFKDVYLVYELMDTDL

HQIIKSPQGLSNDHCQYFLFQLLRGLKYLHSAEILHRDLKPGNLLVNANCDLKICDFGLA

RTNSSKGQFMTEYVVTRWYRAPELLLCCDNYGTSIDVWSVGCIFAELLGRKPIFPGTECL

NQLKLIVNVLGTMSESDLEFIDNPKARRYIKTLPYTPGVPLASMYPHAHPLAIDLLQKML

IFDPTKRISVTEALEHPYMSPLYDPSANPPAQVPIDLDLDENISADMIREMMWQEMLHYH

PEAATAISM*

>Luc1MPK16 Brdisv1Luc11018823m.p

MDFFTEYGEGNRYKIEEVIGKGSYGVVCSALDTHTGDKVAIKKINDIFEHVSDATRILRE

IKLLRLLRHPDIVEIKHILLPPSRREFKDIYVVFELMESDLHQVIKANDDLTPEHYQFFL

YQLLRGLKYIHTANVFHRDLKPKNILANADCKLKICDFGLARVAISDTPTAIFWTDYIAT

RWYRAPELCGSFFSKYTPAIDIWSIGCIFAELLTGKPLFPGKNVVHQLDIITDLLGTPSA

ETIARIRNEKARRYLSSMRRKKTVPFTQKFPNADPLALNLLERMLAFDPKDRPSAEEALA

DPYFKNIASVDREPSAQPITKLEFEFERRRITKDDIRELIYREILEYHPNMLREFLEGAE

PTGFMYPSAVDHFKKQFTFLEEHYAKGSTAAPPERQHNSLPRPSVIYSDNRPQNAANITD

DLSRCAIRDNTQKAQKDSASVGANRIPHGAAAAAARPGKVVGSVLRYGNCSTSGTEQYEQ

RRVVRSPGIAPNGVTSGSSYPRRNNTCKSETGEAERIDTNQGGPPKPYVANKLPATVDGR

NGHW*

>Luc1MPK17 Brdisv1Luc11005901m.p

MVGGGGIVDGFRRLFHRRTPSGSGPSSNQSSAGEESSDIDAVVEDLDLVGLRAIRVPKRK

MPLPVESHKKNILEKEFFTEYGEASQYQIQEVIGKGSYGVVAAAVDTRTGERVAIKKIND

VFEHVSDATRILREVKLLRLLRHPDVVEIKHIMLPPSRREFQDIYVVFELMESDLHQVIR

ANDDLTPEHYQFFLYQLLRALKYIHAANVFHRDLKPKNILANSDCKLKICDFGLARVSFN

DAPTAIFWTDYVATRWYRAPELCGSFFSKYTPAIDIWSIGCIFAELLTGRPLFPGKNVVH

QLDIITDLLGTPSSETLSRIRNEKARRYLSCMRKKHPVPLTQKFPNVDPLALRLLGRLLA

FDPKDRPSAEEALADPYFAVLANVEREPSRNPISKLEFEFERQKVTKDDVRELIYREILE

YHPQMLQEYMQGGEQLSFLYPSGVDRFKRQFAHLEENYSKGERGSPLQRKHASLPRQRVG

ASNDNNEQHASDQERGADSIAPATGNPPGSQDAGQQHVSGGQNGVGSTNISPRSYQKSAS

ISASKCVVVNANKNPEYDDDISEEMEGAVDGLSEKVSRMHP*

>Luc1MPK20-1 Brdisv1Luc11020448m.p

MQQDQRKKSSAEAEFFTEYGDASRYKIQEIVGKGSYGVVCSAIDVHTGEKVAIKKIHDIF

EHISDAARILREIKLLRLLRHPDIVEIKHIMLPPSRRDFKDIYVVFELMESDLHQVIKAN

DDLTKEHYQFFLYQLLRALKYIHTANVYHRDLKPKNILANSNCKLKICDFGLARVAFNDT

PTTIFWTDYVATRWYRAPELCGSFFSKYTPAIDVWSIGCIFAEVLTGKPLFPGKNVVHQL

DLMTDLLGTPSMDTISRVRNEKARRYLSSMRKKEPIPFSQKFPNADPLALDLLQRLLAFD

PKDRPTAGEALSHPYFKGLAKVEREPSCQPITKMEFEFERRRATKEDIRELIFREILEYH

PQLLKDYINGTERTTFLYPSAVDQFRKQFAHLEENSESGPVVPMDRKHTSLPRSTIVHSA

PIHAKEQPRIGPSRDRPLTDESYKNPRDSEKYSGNVPRSSHHSQAPQRVPTARPGRVVGP

VLPYENVGTSHPYDPRRVAMNSGYPPQQQIPQTYGYYQTTGKSACSEPSQAERYTLHQQA

YACANSSTAPDVALDMRAPPFHQSAGPKSGSSDRLTAETNLYTRSLNGIVATTSGVAASA

HRKVSVVPYGMSQMY*

>Luc1MPK20-2 Brdisv1Luc11015867m.p

MQQPQQQGQRNKSTDVDFFSEYGDANRYRIQEVIGKGSYGVVCSAMDMQTRQKVAIKKIH

NIFDHVSDAARILREIKLLRLLRHPDVVEIKHIMLPPSRKDFKDIYVVFELMESDLHQVI

KANDDLTKEHYQFFLYQLLRALKYIHTASVYHRDLKPKNILANSNCKLKICDFGLARVAF

NDTPTTVFWTDYVATRWYRAPELCGSFFTKYTPAIDIWSIGCIFAEVLTGKPLFPGKNVV

HQLDLMTDLLGTPSMDTISRVRNEKARRYLSSMRKKDPVPFSKKFPNADPLGLKLLEKLL

AFDPKDRPTAEEALTDPYFKSLSKPDREPSCQPIRKVEFDFEHRRMSKDDIRELIFQEIL

EYHPQLLKNYIDGTEKTTFLYPSAVDQFKKQFSHLEESDGSGPVVQTERKHASLPRSTTV

HSTPIPAKEQPLVASSRGRPIANEPCKPWAPGNVPGASQTAHVAQAGRAVGVGSVPPYES

GSGKYPYDATSRPAVSSGYPPQQKIPQTYGYHHHHQTPGAGQSSQAMGGYACGYTKGTTP

PPAAAQDMRASPYHHRSAGTKNDPLNRLAAESDIYTRSLNGIVAAAASAGTGAHRKVGAV

PFGMSGMY*

>Luc1MPK20-3 Brdisv1Luc11006705m.p

MQHGDLHKKSAAEMDFFTAYDDANRYKILEVIGKGSYGLVCSANDLQTGEKVAIKKIHNI

FEHISDAARILREIKLLRLLRHPDVVEIKHILLPPSKKDFKDIYVVFELMESDLHQVIKA

NDDLTREHYQFFLYQMLRALKYMHTANVYHRDLKPKNVLANANCKLKICDFGLARVAFSD

APTTVFWTDYVATRWYRAPELCGSFYSKYTPAIDIWSIGCIFAEVLIGKPLFPGKNVVHQ

LDLITDILGTPSLDAISRVRNDKARKYLTCMRKKQPASFSQKFPKADPLALQLLRRLLAF

DPKDRPSAEEALADPYFNGLAKVEREPSCQPIPKIEFEFEGRRVTKEDIKELIFEEILEY

HPQLLKEHIIGKERPNFVHLSAVDQFKKHFTQLEENDNETGAAVSLQRKHSSLPRQAFNH

R*

>Luc1MPK20-4 Brdisv1Luc11020695m.p

MQTTEQQRKKGSSEMDFFSEYGDANRYKIQEVIGKGSYGVVCSAIDQHTGDKVAIKKIHN

IFEHLSDAARILREIKLLRLLRHPDIVEIRHIMLPPSRRDFKDIYVVFELMDTDLHQVIK

ANDDLTKEHHQFFLYQMLRALKYIHTANVYHRDLKPKNILANANCKLKICDFGLARVAFN

DTPTTVFWTDYVATRWYRAPELCGSFFTKYSRAIDIWSIGCIFAEILTGKPLFPGKNVVH

QLDLMTDLLGTPSLDTVSRIRNEKARRYLSSMRKKQSVSFSERFPKADPAALKLLQRLLA

FDPKDRPTAEEALADPYFKGLGKVEREPSCQPITKMEFEFERKNVTKADVKELIFREILE

YHPQLLKDYMNGTEKTNFLYPSAVDNFRRQFANLEENGGKGGAVVPPDRKHVSLPRNTTV

HSTPIPPKDQKYSQVPQRIPTGRPGRVVGPVIPFENSSTMDPYSQRRVARNPVLPAAATN

LSAYAYRNSDNSERELQQELEKDRMQYHPMQRFMDAKMVSPDLRSTSYYMPKGVPKADVA

ERSALQSNMMQGIAPFNGIATVGGAFNKVSAVQYGVSRMY*

>Luc1MPK20-5 Brdisv1Luc11016023m.p

MFSDIFSREGVAFSRVALRQELLSDQWFTTAGGKGQCYLVPPGTTVFLVNSEEMGFFSEY

VDASRYKILEIIGKGSYGVVCSAIDQETGDKVAIKKIQNIFEHLSDAARILREIKLLRLL

RHPDIVQIKHIMLPPSRRDFRDIYVVFELMDTDLHQVIKANDDLTKEHYQFFLYQMLRAL

KYIHTANVYHRDLKPKNILANANCKLKICDFGLARVAFNDTPTTVFWTDYVATRWYRAPE

LCGSFFTKYSPAIDTWSIGCIFAEILTGKPLFPGKNVVHQLDLMTDLLGTPSTETISRIR

NDKARKYLSSMRRKQPIPFSEKFPNADPSALKLLERLLAFDPKDRPTAEEALAHPYFKRL

ARVEREPSCQQPISKTEFEFERRKFTKEDIKELIFREILEYHPKLLKDYMNGSEKTSFLY

PSAVDNFRRQFANLEIDGGRSGAADRKHFSLPRTTTVHSAPILPTNGPTSQVPQRIPTAR

PGRVVSSAMQTDNPSVSDRHNGRRVARDPAVPPAAAYHLKSDYSDRQHQQEFEKDRVRPD

RQRQEELEKDRMQYRPGHHSMDAKVAPEISPYMRSSPYYIPPFNGIAAVASGYSKVAAVT

RMY*

>Luc1MPK21-1 Brdisv1Luc11015913m.p

MGGLIRWLRHHRSRRVSSSSSSHLPSNTTSSSTSDLRAHSLPQHQGDHHGEVVVEWEDAA

EGPDSDPEEYIVVVLGDDEQGVVAARAPVRTKPPRVMDPGKKTSESEFFTEYGEANRYKV

SEVIGKGSYGVVAAAVDTQTGERCAIKKINDVFDHVSDATRILREIKLLRLLRHPDIVEI

KHIMLPPSRREFRDIYVVFELMESDLHQVIKANDDLTPEHHQFFLYQLLRGMKYIHTANV

FHRDLKPKNILANADCKLKICDFGLARVSFNDGAPSAIFWTDYVATRWYRAPELCGSFFS

KYTPAIDIWSVGCIFAEMLTGKPLFPGKNVVHQLDLMTDLLGTPSAESLSKIRNEKARRY

LSNMRKKPKVPLTKKFPGIDPMALHLLERLLAFDPKDRPSADEALTDPYFTGLANSEREP

ITQPISKLEFEFEKRKLAKDDVRELIYREILEYHPHMLQEYLRGGGDQMSFMFPSGVDRF

KRQFAHLEEGGAKGEKSSPQLRQNASLPRERVIGNKHGDGDYNMKLNVGEKPELASVSDG

ISKPLMSARSLLKSETMSASKCIGEIKNKDEDSLSECVEGTDDDVSQKIAQLKT*

>Luc1MPK21-2 Brdisv1Luc11020537m.p

GSDEAEFFTEYGEANRYEVGEVVGKGSYGVVAAAVDTHTGERVAIKKINDVFEHVSDATR

ILREIKLLRLLRHPDIVEIKHIMLPPSRREFRDIYIIFELMESDLHQVIKANDDLTPEHH

QFFFYQLLRGMKYIHAANVFHRDLKPRNILANADCKLKICDFGLARVSFNDTPSAIFWTD

YVATRWYRAPELCGSFFSKYTPAIDIWSIGCIFAEMLSGRPLFPGKNVVHQLDLMTDLLG

TPSAESLSRIRNEKARRYLGNMRKKHPVPFSQKFPGVDPMALDLLERLLAFDPKDRPTAA

EALADPYFTGLANSDREPTTQPISKLEFEFERRKLARDDVRELIYREILEYHPQMLHEYH

HGGDQANFVYPSGVDRFKRQFVHLEEGVTKGEKTSPQLRQHASLPRERIIGIGDELGRPN

ADYCIKLHVGEEPGHTSVTDGLSKPLLNARNFLKSESISASQCVVIKEKREKDVSAVTSN

IF*

>Mur1MPK3 Brdisv1Mur11009882m.p

MDGAPVAEFRPTMTHGGRFLLYNIFGNQFEITAKYQPPIMPIGRGAYGIVCSVMNFETRE

MVAIKKIANAFDNNMDAKRTLREIKLLRHLDHENIVGLRDVIPPAIPQSFNDVYIATELM

DTDLHHIIRSNQELSEEHCQYFLYQLLRGLKYIHSANVIHRDLKPSNLLLNANCDLKICD

FGLARPSSESDMMTEYVVTRWYRAPELLLNSTDYSAAIDVWSVGCIFMELINRAPLFPGR

DHMHQMRLITEVIGTPTDDDLGFIRNEDARRNMRHLPQFPRRPFPAQFPRVQPAALDLIE

RMLAFNPLQRITVEEALEHPYLERLHDIADEPICTDPFSFDFEQHPLTEDQMKQLIFNEA

LELNPNFRY*

>Mur1MPK4 Brdisv1Mur11032140m.p

MDTSGGGGGGAGGAAQIQGMATHGGRYVLYNVYGNLFEVASKYAPPIRPIGRGAYGIVCA

AVSSDTGEEVAIKKIGNAFDNHIDAKRTLREIKLLRHMDHENIIAIKDIIRPPRRDDFKD

VYIVTELMDTDLHQIIRSNQPLTDDHCQYFLYQLLRGLKYVHSANVLHRDLKPSNLFLNA

NCDLKIADFGLARTTSETDLMTEYVVTRWYRAPELLLNCSQYTAAIDVWSVGCILGEIIT

RQPLFPGRDYIQQLKLITELIGSPDDSSLGFLRSDNARRYMKQLPQYPRQDFRLRFRNMS

DGAVDLLERMLVFDPSRRITVDEALHHPYLASLHDINEEPTCPAPFSFDFEQPSFTEEHM

KELIWRETLAFNPDPPY*

>Mur1MPK7-2 Brdisv1Mur11023342m.p

MKLSPISASFGAMGSLPQKLDELLSTGHWALSGVVMDEIEELTADLHILCNLLLKLSNVH

DPPPTVRFWMKEVRELSYDVEDCADQFVIANDRAVMRRAKIHRATIRTIIITRLKISRLP

KRRKWRLWMTDKVAEFRTRVQDATQRYWRYKFDDCASNPGYASVGHEFATVYAQPGDLVG

IEGPMDELEQWLTDGEEQLKVISIVGLGGVGKTTLAQKLWATLKGQFLCRAFVRTAQKPD

MRGILRNILLQVRPHQLPNRGEMHHLIHDLREYLQDKRYFVIIDDLWAASVWDVVSRAFP

EGNCCSRIVTTTEIMEVALACCDYCPKHICKLETLSDDDSEKLLLERIVVSGNQIPQQLD

DVLPQIMRNCGGLPLAIIIVASLLVSQPEKLEQWGHTQNSFGSIFGTNPTMEGFIRQILS

ISFNSLPYYLKTCLLYLSSYPEGCLFLKDDLVKLWVAEGFIHAKEWEDMEDLAGRYFDEL

VNVGLIQVMDINYNYKLPSYSVHHMVLDFITCKSIEENFITVVDYSETTTPLTDKVRRLS

LHFGSATHATTPASARLSNVRSLFYFGLFNCMPSFMVFKLLRVLILNFWGDPGNTSFNLT

GICELVWLRYLQVSCNVTVKLPDQIESMGHLETLEIKAKVYAVPLDIVRLSSLSHLHLRG

GTIPNGIGCMRSLRTLKYFDLVNKSVDNLRGLGELANLRDLHLTYSSSLPSEHLKRNLIA

LASSLGKLCNLKSLTLASETAATVVPFDGSISMSSTPAFLERLELLPSICIFSRLPKWIF

QFQKLCILKVAVRELLANDIDSITGLPSLTVLSLCVHTAPGQRIVFNDGAFPVLKYFKFR

CGVLSMSFMAGAMPNLRRLKLGFNTHVGEKYGNMLAGIEHLLNLQDIAGKIGVATESDRK

AAESAIKGAISKHPQSPMLNVQWVDLVEEEYHASEKQHGRQEKGLSGENHAVLEKAKDTK

KHADSGVSQLPDLPSTVSSSRLKSENIAQASKHHVLESMLCDESAQPESLEVSVLEDITN

NFSDDQEISKNRFVVVYKGELQNGSVVVVKRFTIAIDYNLFLDAVDCLMSVKHNNIVRFL

GYCANSQENAAKEGSKSVSVVTQERLCCFEYLRNGNLQMHLTDESCGFDWQMWYQIIEGI

CQGVHYLHNKSITPLDLNPANIMFDDKMVPKIVHYAYTRFLGAATSPVLTNIGDSLAYMA

PEYFGFGEITSKSDIYSLGVIIMQIVTGENKKDFPSIQSVLESWRSRLESDGSQGHTSLE

TCYLQVNLCYLIGIRCIEHDPGKRPNTRDIIDSLDTLEIMECLVKHDVGNSSLEQMLVCS

KVEEPKALPAPPSVSCKLKMAMMVDPPNGTGNHGKHYYTMWQTMFEIDTKYVPIKPIGRG

AYGIVFSSTNQENNEKVAIKKINNVFDNRVDALRTLRELKLLRHLRHENVIALKDIMMPV

HRRSFKDVYLVSELMDTDLHQIIKSSQPLSNDHCQYFLFQLLRGLKYLHSAGILHRDLNP

GNLLVNANCDLKICDFGLARTNNTKGQFMTEYVVTRWYRAPELLLCCDNYGTSIDVWSVG

CIFAELLGRKPIFPGTECLNQLKLIVNVLGTMSENDLEFIDNPKARKYIKSLPYTPGTPL

TSMYPQAHPLAIDLLQKMLVFDPSKRISVTEALEHPYMSPLYDPSANPPAQVPIDLDIDE

NLGVEMIREMLWQEMLQYHPEAARMVNM*

>Mur1MPK11 Brdisv1Mur11030448m.p

MRMEGGGAGPAAAAAAGGAHGLGEAQIKGTLTHGGRYVQYNVYGNLFEVSAKYVPPIRPV

GRGACGIICAAINVQTREEVAIKKIGNAFDNQIDAKRTLREVKLLRHMNHENVISIKDII

RPPRRENFNDVYIVYELMDTDLHHLLRSNQPLTDDHCQYFLYQVLRGLKYVHSANVLHRD

LRPSNLLLNAKCDLKIGDFGLARTTTETDFMMEYVVTRWYRAPELLLNCSEYTGAIDMWS

VGCILGEIATREPLFPGKDYVHQLRLITELLGSPDDTSLGFLRSDNARRYVRSLPQYPKQ

QFRSRFPNMSSGAMDLLERMLVFDPNKRITVDEALCHPYLASLHEINDEPVCPAPFSFDF

EQPSFTEEDIKELIWRESVKFNPEPIH*

>Mur1MPK14 Brdisv1Mur11028428m.p

MAMLVDPPNGMGNQGKHYYSMWQTLFEIDTKYVPIKPIGRGAYGIVCSSINRETNEKVAI

KKIHNVFDNRVDALRTLRELKLLRHLRHENVISLKDIMMPVQRRSFKDVYLVYELMDTDL

HQIIKSPQGLSNDHCQYFLFQLLRGLKYLHSAEILHRDLKPGNLLVNANCDLKICDFGLA

RTNSSKGQFMTEYVVTRWYRAPELLLCCDNYGTSIDVWSVGCIFAELLGRKPIFPGTECL

NQLKLIVNVLGTMSESDLEFIDNPKARRYIKTLPYTPGVPLASMYPHAHPLAIDLLQKML

IFDPTKRISVTEALEHPYMSPLYDPSANPPAQVPIDLDLDENISADMIREMMWQEMLHYH

PEAATAISM*

>Mur1MPK16 Brdisv1Mur11016923m.p

MDFFTEYGEGNRYKIEEVIGKGSYGVVCSALDTHTGDKVAIKKINDIFEHVSDATRILRE

IKLLRLLRHPDIVEIKHILLPPSRREFKDIYVVFELMESDLHQVIKANDDLTPEHYQFFL

YQLLRGLKYIHTANVFHRDLKPKNILANADCKLKICDFGLARVAISDTPTAIFWTDYIAT

RWYRAPELCGSFFSKYTPAIDIWSIGCIFAELLTGKPLFPGKNVVHQLDIITDLLGTPSA

ETIARIRNEKARRYLSSMRRKKTVPFTQKFPNADPLALNLLERMLAFDPKDRPSAEEALA

DPYFKNIASVDREPSAQPITKLEFEFERRRITKDDIRELIYREILEYHPNMLREFLEGAE

PTGFMYPSAVDHFKKQFTFLEEHYAKGSTAAPPERQHNSLPRPSVIYSDNRPQNAANITD

DLSRCAIRDNTQKAQKDSASVGANRIPHGAAAAAARPGKVVGSVLRYGNCSTSGTEQYEQ

RRVVRSPGIAPNGVTSGSSYPRRNNTCKSETGEAERIDTNQGGPPKPYVANKLPATVDGR

SGHW*

>Mur1MPK17 Brdisv1Mur11005362m.p

MVGGGGIVDGFRRLFHRRTPSGSGPSSNQSSAGEESSDIDAVVEDLDLVGLRAIRVPKRK

MPLPVESHKKNILEKEFFTEYGEASQYQIQEVIGKGSYGVVAAAVDTRTGERVAIKKIND

VFEHVSDATRILREVKLLRLLRHPDVVEIKHIMLPPSRREFQDIYVVFELMESDLHQVIR

ANDDLTPEHYQFFLYQLLRALKYIHAANVFHRDLKPKNILANSDCKLKICDFGLARVSFN

DAPTAIFWTDYVATRWYRAPELCGSFFSKYTPAIDIWSIGCIFAELLTGRPLFPGKNVVH

QLDIITDLLGTPSSETLSRIRNEKARRYLSCMRKKHPVPLTQKFPNVDPLALRLLGRLLA

FDPKDRPSAEEALADPYFAVLANVEREPSRNPISKLEFEFERRKVTKDDVRELIYREILE

YHPQMLQEYMQGGEQLSFLYPSGVDRFKRQFAHLEENYSKGERGSPLQRKHASLPRQRVG

ASNDNNEQHASDQERGADSIAPATGNPPGSQDAGQQHVSGGQNGVGSTNISPRSYQKSAS

ISASKCVVVNANKNPEYDDDISEEMEGAVDGLSEKVSRMHP*

>Mur1MPK20-1 Brdisv1Mur11018309m.p

MQQDQRKKSSAEAEFFTEYGDASRYKIQEIVGKGSYGVVCSAIDVHTGEKVAIKKIHDIF

EHISDAARILREIKLLRLLRHPDIVEIKHIMLPPSRRDFKDIYVVFELMESDLHQVIKAN

DDLTKEHYQFFLYQLLRALKYIHTANVYHRDLKPKNILANSNCKLKICDFGLARVAFNDT

PTTIFWTDYVATRWYRAPELCGSFFSKYTPAIDVWSIGCIFAEVLTGKPLFPGKNVVHQL

DLMTDLLGTPSMDTISRVRNEKARRYLSSMRKKEPIPFSQKFPNADPLALDLLQRLLAFD

PKDRPTAGEALSHPYFKGLAKVEREPSCQPITKMEFEFERRRATKEDIRELIFREILEYH

PQLLKDYINGTERTTFLYPSAVDQFRKQFAHLEENSESGPVVPMDRKHTSLPRSTIVHSA

PIHAKEQPRIGPSRDRPLTDESYKNPRDSEKYSGNVPRSSHHSQAPQRVPTARPGRVVGP

VLPYENVGTSHPYDPRRVAMNSGYPPQQQIPQTYGYYQTTGKSACSEPSQAERYTLHQQA

YACANSSTAPDVALDMRAPPFHQSAGPKSGSSDRLTAETNLYTRSLNGIVATTSGVAASA

HRKVSVVPYGMSQMY*

>Mur1MPK20-2 Brdisv1Mur11014394m.p

MQQPQQQGQRNKSTDVDFFSEYGDANRYRIQEVIGKGSYGVVCSAMDMQTRQKVAIKKIH

NIFDHVSDAARILREIKLLRLLRHPDVVEIKHIMLPPSRKDFKDIYVVFELMESDLHQVI

KANDDLTKEHYQFFLYQLLRALKYIHTASVYHRDLKPKNILANSNCKLKICDFGLARVAF

NDTPTTVFWTDYVATRWYRAPELCGSFFTKYTPAIDIWSIGCIFAEVLTGKPLFPGKNVV

HQLDLMTDLLGTPSMDTISRVRNEKARRYLSSMRKKDPVPFSKKFPNADPLGLKLLEKLL

AFDPKDRPTAEEALTDPYFKSLSKPDREPSCQPIRKVEFDFEHRRMSKDDIRELIFQEIL

EYHPQLLKNYIDGTEKTTFLYPSAVDQFKKQFSHLEESDGSGPVVQTERKHASLPRSTTV

HSTPIPAKEQPLVASSRGRPIANEPCKPWAPGNVPGASQTAHVAQAGRAVGVGSVPPYES

GSGKYPYDATSRPAVSSGYPPQQKIPQTYGYHHHHQTPGAGQSSQAMGGYACGYTKGTTP

PPAAAQDMRASPYHHRSAGTKNDPLNRLAAESDIYTRSLNGIVAAAASAGTGAHRKVGAV

PFGMSGMY*

>Mur1MPK20-3 Brdisv1Mur11006032m.p

MQHGDLHKKSAAEMDFFTAYDDANRYKILEVIGKGSYGLVCSANDLQTGEKVAIKKIHNI

FEHISDAARILREIKLLRLLRHPDVVEIKHILLPPSKKDFKDIYVVFELMESDLHQVIKA

NDDLTREHYQFFLYQMLRALKYMHTANVYHRDLKPKNVLANANCKLKICDFGLARVAFSD

APTTVFWTDYVATRWYRAPELCGSFYSK

>Mur1MPK20-4 Brdisv1Mur11018511m.p

MQTTEQQRKKGSSEMDFFSEYGDANRYKIQEVIGKGSYGVVCSAIDQHTGDKVAIKKIHN

IFEHLSDAARILREIKLLRLLRHPDIVEIRHIMLPPSRRDFKDIYVVFELMDTDLHQVIK

ANDDLTKEHHQFFLYQMLRALKYIHTANVYHRDLKPKNILANANCKLKICDFGLARVAFN

DTPTTVFWTDYVATRWYRAPELCGSFFTKYSRAIDIWSIGCIFAEILTGKPLFPGKNVVH

QLDLMTDLLGTPSLDTVSRIRNEKARRYLSSMRKKQSVSFSERFPKADPAALKLLQRLLA

FDPKDRPTAEEALADPYFKGLGKVEREPSCQPITKMEFEFERKNVTKADVKELIFREILE

YHPQLLKDYMNGTEKTNFLYPSAVDNFRRQFANLEENGGKGGAVVPPDRKHVSLPRNTTV

HSTPIPPKDQKYSQVPQRIPTGRPGRVVGPVIPFENSSTMDPYSQRRVARNPVLPAAATN

LSAYAYRNSDNSERELQQELEKDRMQYHPMQRFMDAKMVSPDLRSTSYYMPKGVPKADVA

ERSALQSNMMQGIAPFNGIATVGGAFNKVSAVQYGVSRMY*

>Mur1MPK20-5 Brdisv1Mur11014538m.p

MFSDIFSREGVAFSRVALRQELLSDQWFTTAGGKGQCYLVPPGTTVFLVNSEEMGFFSEY

VDASRYKILEIIGKGSYGVVCSAIDQETGDKVAIKKIQNIFEHLSDAARILREIKLLRLL

RHPDIVQIKHIMLPPSRRDFRDIYVVFELMDTDLHQVIKANDDLTKEHYQFFLYQMLRAL

KYIHTANVYHRDLKPKNILANANCKLKICDFGLARVAFNDTPTTVFWTDYVATRWYRAPE

LCGSFFTKYSPAIDTWSIGCIFAEILTGKPLFPGKNVVHQLDLMTDLLGTPSTETISRIR

NDKARKYLSSMRRKQPIPFSEKFPNADPSALKLLERLLAFDPKDRPTAEEALAHPYFKRL

ARVEREPSCQQPISKTEFEFERRKFTKEDIKELIFREILEYHPKLLKDYMNGSEKTSFLY

PSAVDNFRRQFANLEIDGGRSGAADRKHFSLPRTTTVHSAPILPTNGPTSQVPQRIPTAR

PGRVVSSAMQTDNPSVSDRHNGRRVARDPAVPPAAAYHLKSDYSDRQHQQEFEKDRVRPD

RQRQEELEKDRMQYRPGHHSMDAKVAPEISPYMRSSPYYIPPFNGIAAVASGYSKVAAVT

RMY*

>Mur1MPK21-1 Brdisv1Mur11014435m.p

MGGLIRWLRHPRSRRVSSSSSSHLPSNTTSSSTSDLRAPSLPQHQGDHHGEVVVEWEDAA

EGPDSDPEEYIVVVLGDDEQGVVAARAPVRTKPPRVMDPGKKTSESEFFTEYGEANRYKV

SEVIGKGSYGVVAAAVDTQTGERCAIKKINDVFDHVSDATRILREIKLLRLLRHPDIVEI

KHIMLPPSRREFRDIYVVFELMESDLHQVIKANDDLTPEHHQFFLYQLLRGMKYIHTANV

FHRDLKPKNILANADCKLKICDFGLARVSFNDGAPSAIFWTDYVATRWYRAPELCGSFFS

KYTPAIDIWSVGCIFAEMLTGKPLFPGKNVVHQLDLMTDLLGTPSAESLSKIRNEKARRY

LSNMRKKPKVPLTKKFPGIDPMALHLLERLLAFDPKDRPSADEALTDPYFTGLANSEREP

ITQPISKLEFEFEKRKLAKDDVRELIYREILEYHPHMLQEYLRGGGDQMSFMFPSGVDRF

KRQFAHLEEGGAKGEKSSPQLRQNASLPRERVIGNKHGDGDYNMKLNVGEKPELASVSDG

ISKPLMSARSLLKSETMSASKCIGEIKNKDEDSLSECVEGTDDDVSQKIAQLKT*

>Mur1MPK21-2 Brdisv1Mur11018391m.p

GSDEAEFFTEYGEANRYEVGEVVGKGSYGVVAAAVDTHTGERVAIKKINDVFEHVSDATR

ILREIKLLRLLRHPDIVEIKHIMLPPSRREFRDIYIIFELMESDLHQVIKANDDLTPEHH

QFFFYQLLRGMKYIHAANVFHRDLKPRNILANADCKLKICDFGLARVSFNDTPSAIFWTD

YVATRWYRAPELCGSFFSKYTPAIDIWSIGCIFAEMLSGRPLFPGKNVVHQLDLMTDLLG

TPSAESLSRIRNEKARRYLGNMRKKHPVPFSQKFPGVDPMALDLLERLLAFDPKDRPTAA

EALADPYFTGLANSDREPTTQPISKLEFEFERRKLARDDVRELIYREILEYHPQMLHEYH

HGGDQANFVYPSGVDRFKRQFVHLEEGVTKGEKTSPQLRQHASLPRERIIGIGDELGRPN

ADYCIKLHVGEEPGHTSVTDGLSKPLLNARNFLKSESISASQCVVIKEKREKDVSAVTSN

IF*

>ABR3MPK3 Brdisv1ABR31010870m.p

MDGAPVAEFRPTMTHGGRFLLYNIFGNQFEITAKYQPPIMPIGRGAYGIVCSVMNFETRE

MVAIKKIANAFDNNMDAKRTLREIKLLRHLDHENIVGLRDVIPPAIPQSFNDVYIATELM

DTDLHHIIRSNQELSEEHCQYFLYQLLRGLKYIHSANVIHRDLKPSNLLLNANCDLKICD

FGLARPSSESDMMTEYVVTRWYRAPELLLNSTDYSAAIDVWSVGCIFMELINRAPLFPGR

DHMHQMRLITEVIGTPTDDDLGFIRNEDARRYMRHLPQFPRRPFPAQFPRVQPAALDLIE

RMLAFNPLQRITVEEALEHPYLERLHDIADEPICTDPFSFDFEQHPLTEDQMKQLIFNEA

LELNPNFRY*

>ABR3MPK4 Brdisv1ABR31028286m.p

MDTSGGGGGGAGGAAQIQGMATHGGRYVLYNVYGNLFEVASKYAPPIRPIGRGAYGIVCA

AVSSDTGEEVAIKKIGNAFDNHIDAKRTLREIKLLRHMDHENIIAIKDIIRPPRRDDFKD

VYIVTELMDTDLHQIIRSNQPLTDDHCQYFLYQLLRGLKYVHSANVLHRDLKPSNLFLNA

NCDLKIADFGLARTTSETDLMTEYVVTRWYRAPELLLNCSQYTAAIDVWSVGCILGEIIT

RQPLFPGRDYIQQLKLITELIGSPDDSSLGFLRSDNARRYMKQLPQYPRQDFRLRFRNMS

DGAVDLLERMLVFDPSRRITVDEALHHPYLASLHDINEEPTCPAPFSFDFEQPSFTEEHM

KELIWRETLAFNPDPPY*

>ABR3MPK6 Brdisv1ABR31008023m.p

MTGDQTLASMTNYGRFSQIETAYWLVSYNKQSVSAFNVPVIVAIRDIIPPAQRNSFNDVY

IAYELMDTDLHQIIRSNQALSEEHCQYFLYQILRGLKYIHSANVLHRDLKPSNLLLNANC

DLKICDFGLARTTSETDFMTEYVVTRWYRAPELLLNSSEYTAAIDVWSVGCIFMELMDRK

PLFPGRDHVHQLRLLMELIGTPNEADLDFVNENARRYIRQLPRHARQSFSEKFPHVHPSA

IDLVEKMLTFDPRQRITVEGALAHPYLASLHDISDEPVCTMPFSFDFEQHALSEEQMKDL

IHQEALAFNPDYQ*

>ABR3MPK11 Brdisv1ABR31026158m.p

MRMEGGGAGPAAAAAAGGAHGLGEAQIKGTLTHGGRYVQYNVYGNLFEVSAKYVPPIRPV

GRGACGIICAAINVQTREEVAIKKIGNAFDNQIDAKRTLREVKLLRHMNHENVISIKDII

RPPRRENFNDVYIVYELMDTDLHHLLRSNQPLTDDHCQYFLYQVLRGLKYVHSANVLHRD

LRPSNLLLNAKCDLKIGDFGLARTTTETDFMMEYVVTRWYRAPELLLNCSEYTGAIDMWS

VGCILGEIATREPLFPGKDYVHQLRLITELLGSPDDTSLGFLRSDNARRYVRSLPQYPKQ

QFRSRFPNMSSGAMDLLERMLVFDPNKRITVDEALCHPYLASLHEINDEPVCPAPFSFDF

EQPSFTEEDIKELIWRESVKFNPEPIH*

>ABR3MPK14 Brdisv1ABR31024015m.p

MAMLVDPPNGMGNQGKHYYSMWQTLFEIDTKYVPIKPIGRGAYGIVCSSINRETNEKVAI

KKIHNVFDNRVDALRTLRELKLLRHLRHENVISLKDIMMPVQRRSFKDVYLVYELMDTDL

HQIIKSPQGLSNDHCQYFLFQLLRGLKYLHSAEILHRDLKPGNLLVNANCDLKICDFGLA

RTNSSKGQFMTEYVVTRWYRAPELLLCCDNYGTSIDVWSVGCIFAELLGRKPIFPGTECL

NQLKLIVNVLGTMSESDLEFIDNPKARRYIKTLPYTPGVPLASMYPHAHPLAIDLLQKML

IFDPTKRISVTEALEHPYMSPLYDPSANPPAQVPIDLDLDENISADMIREMMWQEMLHYH

PEAATAISM*

>ABR3MPK16 Brdisv1ABR31018712m.p

MDFFTEYGEGNRYKIEEVIGKGSYGVVCSALDTHTGDKVAIKKINDIFEHVSDATRILRE

IKLLRLLRHPDIVEIKHILLPPSRREFKDIYVVFELMESDLHQVIKANDDLTPEHYQFFL

YQLLRGLKYIHTANVFHRDLKPKNILANADCKLKICDFGLARVAISDTPTAIFWTDYIAT

RWYRAPELCGSFFSKYTPAIDIWSIGCIFAELLTGKPLFPGKNVVHQLDIITDLLGTPSA

ETIARIRNEKARRYLSSMRRKKTVPFTQKFPNADPLALNLLERMLAFDPKDRPSAEEALA

DPYFKNIASVDREPSAQPITKLEFEFERRRITKDDIRELIYREILEYHPNMLREFLEGAE

PTGFMYPSAVDHFKKQFTFLEEHYAKGSTAAPPERQHNSLPRPSVIYSDNRPQNAANITD

DLSRCAIRDNTQKAQKDSASVGANRIPHGAAAAAARPGKVVGSVLRYGNCSTSGTEQYEQ

RRVVRSPGIAPNGVTSGSSYPRRNNTCKSETGEAERIDTNQGGPPKPYVANKLPATVDGR

NGHW*

>ABR3MPK17 Brdisv1ABR31005797m.p

MVGGGGIVDGFRRLFHRRTPSGSGPSSNQSSAGEESSDIDAVVEDLDLVGLRAIRVPKRK

MPLPVESHKKNILEKEFFTEYGEASQYQIQEVIGKGSYGVVAAAVDTRTGERVAIKKIND

VFEHVSDATRILREVKLLRLLRHPDVVEIKHIMLPPSRREFQDIYVVFELMESDLHQVIR

ANDDLTPEHYQFFLYQLLRALKYIHAANVFHRDLKPKNILANSDCKLKICDFGLARVSFN

DAPTAIFWTDYVATRWYRAPELCGSFFSKYTPAIDIWSIGCIFAELLTGRPLFPGKNVVH

QLDIITDLLGTPSSETLSRIRNEKARRYLSCMRKKHPVPLTQKFPNVDPLALRLLGRLLA

FDPKDRPSAEEALADPYFAVLANVEREPSRNPISKLEFEFERRKVTKDDVRELIYREILE

YHPQMLQEYMQGGEQLSFLYPSGVDRFKRQFAHLEENYSKGERGSPLQRKHASLPRQRVG

ASNDNNEQHASDQERGADSIAPATGNPPGSQDAGQQHVSGGQNGVGSTNISPRSYQKSAS

ISASKCVVVNANKNPEYDDDISEEMEGAVDGLSEKVSRMHP*

>ABR3MPK20-1 Brdisv1ABR31020171m.p

MQQDQRKKSSAEAEFFTEYGDASRYKIQEIVGKGSYGVVCSAIDVHTGEKVAIKKIHDIF

EHISDAARILREIKLLRLLRHPDIVEIKHIMLPPSRRDFKDIYVVFELMESDLHQVIKAN

DDLTKEHYQFFLYQLLRALKYIHTANVYHRDLKPKNILANSNCKLKICDFGLARVAFNDT

PTTIFWTDYVATRWYRAPELCGSFFSKYTPAIDVWSIGCIFAEVLTGKPLFPGKNVVHQL

DLMTDLLGTPSMDTISRVRNEKARRYLSSMRKKEPIPFSQKFPNADPLALDLLQRLLAFD

PKDRPTAGEALSHPYFKGLAKVEREPSCQPITKMEFEFERRRATKEDIRELIFREILEYH

PQLLKDYINGTERTTFLYPSAVDQFRKQFAHLEENSESGPVVPMDRKHTSLPRSTIVHSA

PIHAKEQPRIGPSRDRPLTDESYKNPRDSEKYSGNVPRSSHHSQAPQRVPTARPGRVVGP

VLPYENVGTSHPYDPRRVAMNSGYPPQQQIPQTYGYYQTTGKSACSEPSQAERYTLHQQA

YACANSSTAPDVALDMRAPPFHQSAGPKSGSSDRLTAETNLYTRSLNGIVATTSGVAASA

HRKVSVVPYGMSQMY*

>ABR3MPK20-2 Brdisv1ABR31015739m.p

MQQPQQQGQRNKSTDVDFFSEYGDANRYRIQEVIGKGSYGVVCSAMDMQTRQKVAIKKIH

NIFDHVSDAARILREIKLLRLLRHPDVVEIKHIMLPPSRKDFKDIYVVFELMESDLHQVI

KANDDLTKEHYQFFLYQLLRALKYIHTASVYHRDLKPKNILANSNCKLKICDFGLARVAF

NDTPTTVFWTDYVATRWYRAPELCGSFFTKYTPAIDIWSIGCIFAEVLTGKPLFPGKNVV

HQLDLMTDLLGTPSMDTISRVRNEKARRYLSSMRKKDPVPFSKKFPNADPLGLKLLEKLL

AFDPKDRPTAEEALTDPYFKSLSKPDREPSCQPIRKVEFDFEHRRMSKDDIRELIFQEIL

EYHPQLLKNYIDGTEKTTFLYPSAVDQFKKQFSHLEESDGSGPVVPTERKHASLPRSTTV

HSTPIPAKEQPLVASSRGRPIANEPCKPWAPGNVPGASQTAHVAQAGRAVGVGSVPPYES

GSGKYPYDATSRPAVSSGYPPQQKIPQTYGYHHHHQTPGAGQSSQAMGGYACGYTKGTTP

PPAAAQDMRASPYHHRSAGTKNDPLNRLAAESDIYTRSLNGIVAAAASAGTGAHRKVGAV

PFGMSGMY*

>ABR3MPK20-3 Brdisv1ABR31006618m.p

MQHGDLHKKSAAEMDFFTAYDDANRYKILEVIGKGSYGLVCSANDLQTGEKVAIKKIHNI

FEHISDAARILREIKLLRLLRHPDVVEIKHILLPPSKKDFKDIYVVFELMESDLHQVIKA

NDDLTREHYQFFLYQMLRALKYMHTANVYHRDLKPKNVLANANCKLKICDFGLARVAFSD

APTTVFWTDYVATRWYRAPELCGSFYSKYTPAIDIWSIGCIFAEVLIGKPLFPGKNVVHQ

LDLITDILGTPSLDAISRVRNDKARKYLTCMRKKQPASFSQKFPKADPLALQLLRRLLAF

DPKDRPSAEEALADPYFNGLAKVEREPSCQPIPKIEFEFEGRRVTKEDIKELIFEEILEY

HPQLLKEHIIGKERPNFVHLSAVDQFKKHFTQLEENDNETGAAVSLQRKHSSLPRQAFNH

R*

>ABR3MPK20-4 Brdisv1ABR31020401m.p

MQTTEQQRKKGSSEMDFFSEYGDANRYKIQEVIGKGSYGVVCSAIDQHTGDKVAIKKIHN

IFEHLSDAARILREIKLLRLLRHPDIVEIRHIMLPPSRRDFKDIYVVFELMDTDLHQVIK

ANDDLTKEHHQFFLYQMLRALKYIHTANVYHRDLKPKNILANANCKLKICDFGLARVAFN

DTPTTVFWTDYVATRWYRAPELCGSFFTKYSRAIDIWSIGCIFAEILTGKPLFPGKNVVH

QLDLMTDLLGTPSLDTVSRIRNEKARRYLSSMRKKQSVSFSERFPKADPAALKLLQRLLA

FDPKDRPTAEEALADPYFKGLGKVEREPSCQPITKMEFEFERKNVTKADVKELIFREILE

YHPQLLKDYMNGTEKTNFLYPSAVDNFRRQFANLEENGGKGGAVVPPDRKHVSLPRNTTV

HSTPIPPKDQKYSQVPQRIPTGRPGRVVGPVIPFENSSTMDPYSQRRVARNPVLPAAATN

LSAYAYRNSDNSERELQQELEKDRMQYHPMQRFMDAKMVSPDLRSTSYYMPKGVPKADVA

ERSALQSNMMQGIAPFNGIATVGGAFNKVSAVQYGVSRMY*

>ABR3MPK20-5 Brdisv1ABR31015895m.p

MFSDIFSREGVAFSRVALRQELLSDQWFTTAGGKGQCYLVPPGTTVFLVNSEEMGFFSEY

VDASRYKILEIIGKGSYGVVCSAIDQETGDKVAIKKIQNIFEHLSDAARILREIKLLRLL

RHPDIVQIKHIMLPPSRRDFRDIYVVFELMDTDLHQVIKANDDLTKEHYQFFLYQMLRAL

KYIHTANVYHRDLKPKNILANANCKLKICDFGLARVAFNDTPTTVFWTDYVATRWYRAPE

LCGSFFTKYSPAIDTWSIGCIFAEILTGKPLFPGKNVVHQLDLMTDLLGTPSTETISRIR

NDKARKYLSSMRRKQPIPFSEKFPNADPSALKLLERLLAFDPKDRPTAEEALAHPYFKRL

ARVEREPSCQQPISKTEFEFERRKFTKEDIKELIFREILEYHPKLLKDYMNGSEKTSFLY

PSAVDNFRRQFANLEIDGGRSGAADRKHFSLPRTTTVHSAPILPTNGPTSQVPQRIPTAR

PGRVVSSAMQTDNPSVSDRHNGRRVARDPAVPPAAAYHLKSDYSDRQHQQEFEKDRVRPD

RQRQEELEKDRMQYRPGHHSMDAKVAPEISPYMRSSPYYIPPFNGIAAVASGYSKVAAVT

RMY*

>ABR3MPK21-1 Brdisv1ABR31015783m.p

MGGLIRWLRHHRSRRVSSSSSSHLPSNTTSSSTSDLRAHSLPQHQGDHHGEVVVEWEDAA

EGPDSDPEEYIVVVLGDDEQGVVAARAPVRTKPPRVMDPGKKTSESEFFTEYGEANRYKV

SEVIGKGSYGVVAAAVDTQTGERCAIKKINDVFDHVSDATRILREIKLLRLLRHPDIVEI

KHIMLPPSRREFRDIYVVFELMESDLHQVIKANDDLTPEHHQFFLYQLLRGMKYIHTANV

FHRDLKPKNILANADCKLKICDFGLARVSFNDGAPSAIFWTDYVATRWYRAPELCGSFFS

KYTPAIDIWSVGCIFAEMLTGKPLFPGKNVVHQLDLMTDLLGTPSAESLSKIRNEKARRY

LSNMRKKPKVPLTKKFPGIDPMALHLLERLLAFDPKDRPSADEALTDPYFTGLANSEREP

ITQPISKLEFEFEKRKLAKDDVRELIYREILEYHPHMLQEYLRGGGDQMSFMFPSGVDRF

KRQFAHLEEGGAKGEKSSPQLRQNASLPRERVIGNKHGDGDYNMKLNVGEKPELASVSDG

ISKPLMSARSLLKSETMSASKCIGEIKNKDEDSLSECVEGTDDDVSQKIAQLKT*

>ABR3MPK21-2 Brdisv1ABR31020259m.p

GSDEAEFFTEYGEANRYEVGEVVGKGSYGVVAAAVDTHTGERVAIKKINDVFEHVSDATR

ILREIKLLRLLRHPDIVEIKHIMLPPSRREFRDIYIIFELMESDLHQVIKANDDLTPEHH

QFFFYQLLRGMKYIHAANVFHRDLKPRNILANADCKLKICDFGLARVSFNDTPSAIFWTD

YVATRWYRAPELCGSFFSKYTPAIDIWSIGCIFAEMLSGRPLFPGKNVVHQLDLMTDLLG

TPSAESLSRIRNEKARRYLGNMRKKHPVPFSQKFPGVDPMALDLLERLLAFDPKDRPTAA

EALADPYFTGLANSDREPTTQPISKLEFEFERRKLARDDVRELIYREILEYHPQMLHEYH

HGGDQANFVYPSGVDRFKRQFVHLEEGVTKGEKTSPQLRQHASLPRERIIGIGDELGRPN

ADYCIKLHVGEEPGHTSVTDGLSKPLLNARNFLKSESISASQCVVIKEKREKDVSAVTSN

IF*

>ABR5MPK3 Brdisv1ABR51010136m.p

MDGAPVAEFRPTMTHGGRFLLYNIFGNQFEITAKYQPPIMPIGRGAYGIVCSVMNFETRE

MVAIKKIANAFDNNMDAKRTLREIKLLRHLDHENIVGLRDVIPPAIPQSFNDVYIATELM

DTDLHHIIRSNQELSEEHCQYFLYQLLRGLKYIHSANVIHRDLKPSNLLLNANCDLKICD

FGLARPSSESDMMTEYVVTRWYRAPELLLNSTDYSAAIDVWSVGCIFMELINRAPLFPGR

DHMHQMRLITEVIGTPTDDDLGFIRNEDARRYMRHLPQFPRRPFPAQFPRVQPAALDLIE

RMLAFNPLQRITVEEALEHPYLERLHDIADEPICTDPFSFDFEQHPLTEDQMKQLIFNEA

LELNPNFRY*

>ABR5MPK4 Brdisv1ABR51033637m.p
[truncated: 32,252 more chars]
