# Supplementary material for: Comparative analysis of MAPK and MKK gene families reveals differential evolutionary patterns in Brachypodium distachyon inbred lines
Source: PeerJ. 2021 Apr 6;9:e11238. doi: 10.7717/peerj.11238 (PMC8034371; doi:10.7717/peerj.11238)
Supplement: Supplemental Information 16 [file peerj-09-11238-s016.docx]

>ABR9MKK1 Brdisv1ABR9_r1008255m.p

MRKPGKLALPSHESTIGKFLTQSGTFKDGDLLVNKDGLRIVHNSEEGEAPPIEPLDDHQL

SLDDLDAIKVIGKGSSGIVQLVRHKWTDQFFALKVIQLNIQESIRKQIAQELKISLSTQC

QYVVTCYQCFYVNGVISIVLEYMDGGSLADFLKTVRTIPEAYLAGICKQVLQGLMYLHHE

KRVIHRDLKPSNILINHRGEVKISDFGVSAIIASSSAQRDTFTGTFNYMAPERICGQKHG

YMSDIWSLGLVMLECATGNFPYPSPDSFYELLEAVVDQPPPSAPTDQFSPEFCSFISACI

QKEATDRSSAQVLSDHPFLSMYDDLNIDLADYFTTTGSPLATFKQIVL*

>ABR9MKK3-1 Brdisv1ABR9_r1023443m.p

MAGLEELKKKLQPLLFDDPDKDSISTRVPFPEDNCDSYVVSDGGTINLLSRSFGEYNINE

HGFHKRSTGADESDFGEKAYRCASHDMHIFGPIGNGASSVVQRAIFIPVHRILALKKINI

FEKEKRQQILNEMRTLCEASCYPGLVEFQGAFYMPDSGQISIALEYMDGGSLADVIKVKK

SIPEQVLAHMLQKVLLGLRYLHEVRHLVHRDIKPANMLVNLKGEAKITDFGVSAGLDNTM

AMCATFVGTVTYMSPERIRNENYSYAADIWSLGLTILECATGKFPYNVNEGPANLMLQIL

DDPSPTPPADAYSAEFCSFVNDCLQKDPDARPTCEQLFGHPFIKRYENAGVDLIAYVKGV

VDPTERLKEIAEMLAVHYYLLFNGSDGLWHHMKTFYMEESTFSFSGNVYVGRNDIFDTLS

SIRKKLKGDRPREKIVHVVEKLHCRANGETGIAIRVSGSLIVGNQFLVCGEGLQAEGMPS

VEELSIDIPSKRVGQFREQFMMLPGISMGSFHISRQDLYIIQA*

>ABR9MKK3-2 Brdisv1ABR9_r1034151m.p

MAGLEELKKKLQPLMFNDPDKDGVSTRVPFPEDTCDSYVVSDGGTINLLSRSFGEYNINE

HGFHKRSAGADESDFGEKAYRCASQDMHIFGPIGNGASSVVQRAIFIPVHRILALKKINI

FEKEKRQQILNEMRTLCEACCYPGLVEFQGAFYMPDSGQISIALEYMDGGSLADVIKVKK

SIPEPVLAHMLQKVLLGLRYLHEVRHLVHRDIKPANMLVNLKGEAKITDFGVSAGLDNTM

AMCATFVGTVTYMSPERIRNENYSYAADIWSLGLTILECATGKFPYNVNEGPANLMLQIL

DDPSPAPPENAFSSEFCSFVNDCLQKDADARPTCEQLLSHPFIKRYENAGVDLAAYVKGV

VNPEERLKQIAEMLAVHYYLLFNGSDGLWHHMKTFYMEDSTFSFSGNLYVGQSDIFDTLS

NIRTKLKGDRPREKIVHVVEKLHCRANEETGIAIRVSGSFIVSNQFLICGEGLQAEGMPS

LEELSIDIPSKRVGQFREQFIMHPGRSMGCYYISRQDLYIIQA*

>ABR9MKK3-3 Brdisv1ABR9_r1025291m.p

MAAGLEDLRRRVQPIFFDADGNVMPAPDDDSEVLDGGTINLLSRSSDEYNINERGFHKRT

IRSDDEYSSEKAFRCSCHDMHIFDSVGNGASSVVHRAIYVPVHRVLALKKINIFEKERRQ

QILNEIITLSEACCYPGLVEFHGVFYTPDSGEIYFALEYMDGGSLADIIRVKKFISEPVL

SHMLQKVLLALRYLHEVRHLVHRDIKPANLLLNLKGDTKITDFGVTSGLHDSIDMCATFL

GSVTYMSPERIRNESYSYSADIWSLGLTALECATGRYPYDVNGGEADLMLQILEDPSPTP

PHDIYSEEFCSFINACLQKDADARPTCDQLLSHSFIKRYEGPGVDLSEYNKSVHDPSERL

SQIAHMLAVHYYLIFDGGDDQWCHMKTFYQQDSIFSFSGETHVGKSEIFETLSRIRKMLK

GNSPCEKIAHVMEKVYCRSHGEEGMRVRVSGSFIVGNEFVVCADGVRAEGMLSIDELSPD

ILSKQAGHFQEDFFMEPGTALGCYVISKQELHIADT*

>ABR9MKK4 Brdisv1ABR9_r1029250m.p

MRPGGPPNARPQQPGTPGRARRRPDLTLPLPQRDLTSLAVPLPLPPPPSSAPSSASSSGS

SLSSMGAPTPPNSAGSAPPPPPPLAELERVRRIGSGAGGTVWMVRHRPTGRPYALKVLYG

NHDDAVRRQITREIAILRTAEHPAIVRCHGMYEQAGELQILLEFMDGGSLEGRRIASEAF

LADVARQVLSGIAYLPRRHIVHRDIKPSNLLIDSGRRVKIADFGVGRILNQTMDPCNSSV

GTIAYMSPERINTDLNDGAYDGYAGDIWSFGLSILEFYLGRFPLGENLGKQGDWAALMCA

ICYSDSPAPPPIASPEFKSFISCCLQKNPARRPSAAQLLQHRFIAGPQPQVLAAPPS*

>ABR9MKK5 Brdisv1ABR9_r1007521m.p

MRPAGSLPSPQPGTPGRPRRRPDLTLPMPQRPDVSSSLAVPLPLPPPSSLGLAQPPAAAA

ATAAPPPPPLGELERVRRVGSGAGGTVWMVRHRPTGRCYALKQLYGNHDDAVRRQIAREI

AILRTAEHPAVVRCHGMYERGGELQILLEYMDGGSLDGRRIAAEGFLADVARQVLSGIAY

LHRRHIVHRDIKPSNLLIDSARRVKIADFGVGRILNQTMDPCNSSVGTIAYMSPERINTD

LNDGAYDGYAGDIWSFGLSILEFYLGRFPFGENLGKQGDWAALMVAICYNDPPEPSAAAS

PEFRGFISCCLQKNPAKRLSAAQLLQHPFVAGPQPLPLAAPPS*

>ABR9MKK6 Brdisv1ABR9_r1012651m.p

MRGKKPLKELKLSVPAQETSVDKFLTASGTFKDGELRLNQRGLRLISEEENGDEHQSTNM

KVEDVQLSMDDLEMIQVIGKGSGGVVQLVQHKWVGTFYALKGIQMNIQEAVRKQIVQELK

INQATQSPHIVSCHQSFYHNGVIYLVLEYMDRGSLADIIKQVKTILEPYLAVLCKQVLEG

LLYLHHERHVIHRDIKPSNLLVNHKGEVKITDFGVSAVLASSIGQRDTFVGTYNYMAPER

ISGSSYDYKSDVWSLGLVILECAIGRFPYTPSEGEGWLSFYELLEAIVDQPPPGAPADQF

SPEFCSFISACIQKDPAERMSASELLNHAFIKKFEGKDLDLRILVESLEPPMNVPE*

>ABR9MKK10-1 Brdisv1ABR9_r1001822m.p

MALLREKRLQLSLHVPTRAADAQEAGLHRRPNPAAALPLAATTPAARSSQFRVADFEKLA

VLGRGNGGTVYKVRHRETCELYALKVQHCNGDATAEAEVLSRTASPFVVRCHSVLPAAAS

GDVAMLLELVDGGSLDSIVKSRSRGQAEAFSQFPEEALAEVAAQALSGLAYLHARRIVHL

DVKPGNLLVSTGGEVKIADFGIARVLPRAGGNDVRCTAYAGTAAYMSPERFDPEAHGGHY

DPYAADVWGLGVTVLELLMGRYPLLPAGQRPSWAALMCAICFGETPALSDGEASAELRGF

VAACLHKDYRRRASVAELLVHPFVAGRDVAASKCALRKLVTEASMSP*

>ABR9MKK10-2 Brdisv1ABR9_r1011727m.p

MALVRQRRQLPHLTLPLDHFALRPPPVPAPAPTVAASTSSEAAGLRLSDFERISLLGQGN

GGTVYKARHRRAAAQPPVALKLFVAGDPSAAREAEILRLAADAPHVVRLHAVVPSSSPAA

GAEQPPPAALALELLPGGSLAGLLRRLGRSMGERPIAAVARQALLGLDALHALRVVHRDL

KPSNLLLGSHGEVKIADFGAGKVLRRRLDPCASYVGTAAYMSPERFDPEAYSGDYDPYAA

DVWSLGLAILELYLGHFPLLPAGQRPDWAALMCAICFGDAPEAPAAASEEFRDFVARCLE

KKAGQRASVAELLEHPFIAERDAEEAKRALAALVAEAELGDL*

>ABR9MKK10-3 Brdisv1ABR9_r1001726m.p

MALLREKRLQLSLHVPTRAAEALDAAHRRPNPVAATLAASTPAAARSSQFRLADFDKLTV

LGRGNGGTVYKVRHRETCELYALKVQHCNGDPTAAAEAEVLSRTASPFIVRCHSVLPGAA

SGDVAMLLELVDGGSLDSIVKSRRAHAFPFPEEALAEVAAQALSGLAYLHARRIVHLDIK

PGNLLVSTGGEVKVADFGIAKVLPRAGADAARCTSYAGTAAYMSPERFDPEAHGGHYDAY

AADVWGLGVTVLELLMGRYPLLPAGQRPSWPALMCAICFGETPVLSDGEASAELRGFVAA

CLRKDHTKRASVAELLAHPFVAGRDVATSKCALRKLVTEASTSP*

>ABR9MKK10-4 Brdisv1ABR9_r1001722m.p

MASAKERRLPQLHLKLDVPTCAFRCAAPAPAPATAATPATSASRPPHGEFRLNDFDRLSV

LGRGNGGSVYKVSHRRTSALYALKIIHGAHARPGAADEEADIVRRVVDSPNVVRCHSVLP

TASGDAAALLLELVDGGTLDSLVGGGGFLPEAAVADVAAQALSGLAPLRARRVAHRDIKP

ANLLLSAAGEVKIADFGIAKVVVSGAGGRARALAYEGTVAYMSPERFDSERHADADPYAA

DVWGLGVTLLELLMGRYPLLPAGQKPTWAALMCAICFGELPALPEGAASLEFRGFVAACL

RKDHRKRASVVELLAHPFVAGRDVAASRRALREAIERRCSC*

>ABR9MKK10-5 Brdisv1ABR9_r1001725m.p

MALTVRQRRLPQLHISLDLPSCSFRCPNPPVAATASTSGEFRASDFERLAVLGRGNGGTV

YKVAHRRTSAQYALKVLHGGGDPGAAAAEADVLRRAADSPYVVRCHSVFPAASGSGETAL

LLELVDGGSLDSVRRGVGVSVFFPEAALAEVAAQALAGLAHLHARRVVHRDIKPANLLVS

GAGGVKVADFGIAMVLPSRAGGERCAAAYEGTVAYMSPERFDSEGRADADPRGADVWGLG

VTVLELLMGRYPLLPAGQKPTWAALMCAICFGELPALPEGAASTELRGFIAACLRKDHTK

RASVAELIKHPFVAGRNMAASRLALRRLVAGA*

>Arn1MKK1 Brdisv1Arn11008382m.p

MRKPGKLALPSHESTIGKFLTQSGTFKDGDLLVNKDGLRIVHNSEEGEAPPIEPLDDHQL

SLDDLDAIKVIGKGSSGIVQLVRHKWTDQFFALKVIQLNIQESIRKQIAQELKISLSTQC

QYVVTCYQCFYVNGVISIVLEYMDSGSLADFLKTVRTIPEAYLAGICKQVLQGLMYLHHE

KRVIHRDLKPSNILINHRGEVKISDFGVSAIIASSSAQRDTFTGTFNYMAPERISGQKHG

YMSDIWSLGLVMLECATGNFPYPSPDSFYELLEAVVDQPPPSAPTDQFSPEFCSFISACI

QKEATDRSSAQVLSDHPFLSMYDDLNIDLADYFTTTGSPLATFKQIVL*

>Arn1MKK3-1 Brdisv1Arn11029799m.p

MAGLEELKKKLQPLLFDDPDKDGISTRVPFPEDNCDSYVVSDGGTINLLSRSFGEYNINE

HGFHKRSTGADESDFGEKAYRCASHDMHIFGPIGNGASSVVQRAIFIPVHRILALKKINI

FEKEKRQQILNEMRTLCEASCYPGLVEFQGAFYMPDSGQISIALEYMDGGSLADVIKVKK

SIPEQVLAHMLQKVLLGLRYLHEVRHLVHRDIKPANMLVNLKGEAKITDFGVSAGLDNTM

AMCATFVGTVTYMSPERIRNENYSYAADIWSLGLTILECATGKFPYNVNEGPANLMLQIL

DDPSPTPPADAYSPEFCSFVNDCLQKDPDARPTCEQLFGHPFIKRYENAGVDLIAYVKGV

VDPTERLKEIAEMLAVHYYLLFNGSDGLWHHMKTFYMEESTFSFSGNVYVGRNDIFDTLS

SIRKKLKGDRPREKIVHVVEKLHCRANGETGIAIRVSGSLIVGNQFLVCGEGLQAEGMPS

VEELSIDIPSKRVGQFREQFMMLPGISMGSFHISRQDLYIIQA*

>Arn1MKK3-2 Brdisv1Arn11045189m.p

MAGLEELKKKLQPLMFNDPDKDGVSTRVPFPEDTCDSYVVSDGGTINLLSRSFGEYNINE

HGFHKRSAGADESDFGEKAYRCASQDMHIFGPIGNGASSVVQRAIFIPVHRILALKKINI

FEKEKRQQILNEMRTLCEACCYPGLVEFQGAFYMPDSGQISIALEYMDGGSLADVIKVKK

SIPEPVLAHMLQKVLVGLRYLHEVRHLVHRDIKPANMLVNLKGEAKITDFGVSAGLDNTM

AMCATFVGTVTYMSPERIRNENYSYAADIWSLGLTILECATGKFPYNVNEGPANLMLQIL

DDPSPAPPENAFSSEFCSFVNDCLQKDADARPTCEQLLSHPFIKRYENAGVDLAAYVKGV

VNPEERLKQIAEMLAVHYYLLFNGSDGLWHHMKTFYMEDSTFSFSGNLYVGQSDIFDTLS

NIRTKLKGDRPREKIVHVVEKLHCRANEETGIAIRVSGSFIVSNQFLICGEGLQAEGMPS

LEELSIDIPSKRVGQFREQFIMHPGRSMGCYYISRQDLYIIQA*

>Arn1MKK3-3 Brdisv1Arn11032740m.p

MGWDGMGQRKRRHRAGIPIPTYPGLAASLEFCFVTAGACHTAPGPGSKIAASLFAALLFQ

RPPASTSRAIFFCFVIMAAGLEDLRRRVQPIFFDADGNVMPAPDDDSEVLDGGTINLLSR

SSDEYNINERGFHKRTIRSDDEYSSEKAFRCSCHDMHIFDSVGNGASSVVHRAIYVPVHR

VLALKKINIFEKERRQQILNEIITLSEACCYPGLVEFHGVFYTPDSGEIYFALEYMDGGS

LADIIRVKKFISEPVLSHMLQKVLLALRYLHEVRHLVHRDIKPANLLLNLKGDTKITDFG

VTSGLHDSIDMCATFLGSVTYMSPERIRNESYSYSADIWSLGLTALECATGRYPYDVNGG

EADLMLQILEDPSPTPPHDIYSEEFCSFINACLQKDADARPTCDQLLSHSFIKRYEGPGV

DLSEYNKSVHDPSERLSQIAHMLAVHYYLIFDGGDDQWCHMKTFYQQDSIFSFSGETHVG

KSEIFETLSRIRKMLKGNSPCEKIAHVMEKVYCRSHGEEGMRVRVSGSFIVGNEFVVCAD

GVRAEGMLSIDELSPDILSKQAGHFQEDFFMEPGTALGCYVISKQELHIADT*

>Arn1MKK4 Brdisv1Arn11039595m.p

MRPGGPPNARPQQPGTPGRARRRPDLTLPLPQRDLTSLAVPLPLPPPPSSAPSSASSSGS

SLSSMGAPTPPNSAGSAPPPPPPLAELERVRRIGSGAGGTVWMVRHRPTGRPYALKVLYG

NHDDAVRRQITREIAILRTAEHPAIVRCHGMYEQAGELQILLEFMDGGSLEGRRIASEAF

LADVARQVLSGIAYLHRRHIVHRDIKPSNLLIDSGRRVKIADFGVGRILNQTMDPCNSSV

GTIAYMSPERINTDLNDGAYDGYAGDIWSFGLSILEFYLGRFPLGENLGKQGDWAALMCA

ICYSDSPAPPPIASPEFKSFISCCLQKNPARRPSAAQLLQHRFIAGPQPQVLAAPPS*

>Arn1MKK5 Brdisv1Arn11007617m.p

MRPAGSLPSPQPGTPGRPRRRPDLTLPMPQRPDVSSSLAVPLPLPPPSSLGLAQPPAAAA

AAAAAAPPPPPLGELERVRRVGSGAGGTVWMVRHRPTGRCYALKQLYGNHDDAVRRQIAR

EIAILRTAEHPAVVRCHGMYERGGELQILLEYMDGGSLDGRRIAAEGFLADVARQVLSGI

AYLHRRHIVHRDIKPSNLLIDSARRVKIADFGVGRILNQTMDPCNSSVGTIAYMSPERIN

TDLNDGAYDGYAGDIWSFGLSILEFYLGRFPFGENLGKQGDWAALMVAICYNDPPEPSAA

ASPEFRGFISCCLQKNPAKRLSAAQLLQHPFVARPQPLPLAAPPS*

>Arn1MKK6 Brdisv1Arn11012644m.p

MRGKKPLKELKLSVPAQETSVDKFLTASGTFKDGELRLNQRGLRLISEEENGDEHQSTNM

KVEDVQLSMDDLEMIQVIGKGSGGVVQLVQHKWVGTFYALKGIQMNIQEAVRKQIVQELK

INQATQSPHIVSCHQSFYHNGVIYLVLEYMDRGSLADIIKQVKTILEPYLAVLCKQVLEG

LLYLHHERHVIHRDIKPSNLLVNHKGEVKITDFGVSAVLASSIGQRDTFVGTYNYMAPER

ISGSSYDYKSDVWSLGLVILECAIGRFPYTPSEGEGWLSFYELLEAIVDQPPPGAPADQF

SPEFCSFISACIQKDPAERMSASELLNHAFIKKFEGKDLDLRILVESLEPPMNVPE*

>Arn1MKK10-1 Brdisv1Arn11001761m.p

MALLREKRLQLSLHVPTRAADAQEAGLHRRPNPAAALPLAAPTPAARSSQFRVADFEKLA

VLGRGNGGTVYKVRHRETCELYALKVQHCNGDATAEAEVLSRTASPFVVRCHSVLPAAAS

GDVAMLLELVDGGSLDSIVKSRSRGQAEAFSQFPEEALAEVAAQALSGLAYLHARRIVHL

DVKPGNLLVSTGGEVKIADFGIARVLPRAGGDDVRCTAYAGTAAYMSPERFDPEAHGGHY

DPYAADVWGLGVTVLELLMGRYPLLPAGQRPSWAALMCAICFGETPALSDGEASAELRGF

VAACLHKDYRRRASVAELLAHPFVAGRDVAASKCALRKLVTEASMSP*

>Arn1MKK10-2 Brdisv1Arn11011731m.p

MALVRQRRQLPHLTLPLDHFALRPPPVPAPAPTVAASTSSEAAGLRLSDFERISLLGQGN

GGTVYKARHRRAAAQPPVALKLFVAGDPSAAREAEILRLAADAPHVVRLHAVVPSSSPAA

GAEQPPPAALALELLPGGSLAGLLRRLGRSMGERPIAAVARQALLGLDALHALRVVHRDL

KPSNLLLGSHGEVKIADFGAGKVLRRRLDPCASYVGTAAYMSPERFDPEAYSGDYDPYAA

DVWSLGLAILELYLGHFPLLPAGQRPDWAALMCAICFGDAPEAPAAASEEFRDFVARCLE

KKAGQRASVAELLEHPFIAERDAEEAKRALAALVAEAELGDL*

>Arn1MKK10-3 Brdisv1Arn11001664m.p

MALLREKRLQLSLHVPTRAAEALDAAHRRPNPVAATLAASTPAAARSSQFRLADFDKLTV

LGRGNGGTVYKVRHRETCELYALKVQHCNGDPTAAAEAEVLSRTASPFIVRCHSVLPGAA

SGDVAMLLELVDGGSLDSIVKSRRAHAFPFPEEALAEVAAQALSGLAYLHARRIVHLDIK

PGNLLVSTSGEVKVADFGIAKVLPRAGADDARCTSYAGTAAYMSPERFDPEAHGGHYDAY

AADVWGLGVTVLELLMGRYPLLPAGQRPSWPALMCAICFGETPVLSDGEASAELRGFVAA

CLRKDHTKRASVAELLAHPFVAGRDVATSKCALRKLVTEASTSP*

>Arn1MKK10-4 Brdisv1Arn11001661m.p

MASAKERRLPQLHLKLDVPTCAFRCAAPAPAPATAATPATSASRPPHGEFRLNDFDRLSV

LGRGNGGSVYKVSHRRTSALYALKIIHGAHARPGAADEEADIVRRVVDSPNVVRCHSVLP

TASGDAAALLLELVDGGSLDSLVGGGGFLPEAAVADVAAQALSGLAHLRARRVAHRDIKP

ANLLLSAAGEVKIADFGIAKVVVSGAGGRARALAYEGTVAYMSPERFDSERHADADPYAA

DVWGLGVTLLELLMGRYPLLPAGQKPTWAALMCAICFGELPALPEGAASLEFRGFVAACL

RKDHRKRASVVELLAHPFVAGRDVAASRRALREAIERRCSC*

>Arn1MKK10-5 Brdisv1Arn11001663m.p

MALTVRQRRLPQLHISLDLPSCSFRCPNPPVAATASTSGEFRASDFERLAVLGRGNGGTV

YKVAHRRTSAQYALKVLHGGGDPGAAAAEADVLRRAADSPYVVRCHSVFPAASGSGETAL

LLELVDGGSLDSVRRGVGVSVFFPEAALAEVAAQALAGLAHLHARRVVHRDIKPANLLVS

GAGGVKVADFGIAMVLPSRAGGERCAAAYEGTVAYMSPERFDSEGRADADPRGADVWGLG

VTVLELLMGRYPLLPAGQKPTWAALMCAICFGELPALPEGAASTELRGFIAACLRKDHTK

RASVAELIKHPFVAGRNMAASRLALRRLVAGA*

>Bd1-1MKK1 Brdisv1Bd1-11007340m.p

MRKPGKLALPSHESTIGKFLTQSGTFKDGDLLVNKDGLRIVHNSEEGEAPPIEPLDDHQL

SLDDLDAIKVIGKGSSGIVQLVRHKWTDQFFALKVIQLNIQESIRKQIAQELKISLSTQC

QYVVTCYQCFYVNGVISIVLEYMDGGSLADFLKTVRTIPEAYLAAICKQVLQGLMYLHHE

KRVIHRDLKPSNILINHRGEVKISDFGVSAIIASSSAQRDTFTGTFNYMAPERISGQKHG

YMSDIWSLGLVMLECATGNFPYPSPDSFYELLEAVVDQPPPSAPTDQFSPEFCSFISACI

QKEATDRSSAQVLSDHPFLSMYDDLNIDLADYFTTAGSPLATFKQIVL*

>Bd1-1MKK3-1 Brdisv1Bd1-11034555m.p

MAGLEELKKKLQPLLFDDPDKDGISTRVPFPEDNCDSYVVSDGGTINLLSRSFGEYNINE

HGFHKRSTGADESDFGEKAYRCASHDMHIFGPIGNGASSVVQRAIFIPVHRILALKKINI

FEKEKRQQILNEMRTLCEASCYPGLVEFQGAFYMPDSGQISIALEYMDGGSLADVIKVKK

SIPEQVLAHMLQKVLLGLRYLHEVRHLVHRDIKPANMLVNLKGEAKITDFGVSAGLDNTM

AMCATFVGTVTYMSPERIRNENYSYAADIWSLGLTILECATGKFPYNVNEGPANLMLQIL

DDPSPTPPADAYSAEFCSFVNDCLQKDPDARPTCEQLFGHPFIKRYENAGVDLIAYVKGV

VDPTERLKEIAEMLAVHYYLLFNGSDGLWHHMKTFYMEESTFSFSGNVYVGRNDIFDTLS

SIRKKLKGDRPREKIVHVVEKLHCRANGETGIAIRVSGSLIVGNQFLVCGEGLQAEGMPS

VEELSIDIPSKRVGQFREQFMMLPGISMGSFHISRQDLYIIQA*

>Bd1-1MKK3-2 Brdisv1Bd1-11040480m.p

MAGLEELKKKVQPLMFNDPDKDGVSTRVPFPEDTCDSYVVSDGGTINLLSRSFGEYNINE

HGFHKRSAGADESDFGEKAYRCASQDMHIFGPIGNGASSVVQRAIFIPVHRILALKKINI

FEKEKRQQILNEMRTLCEACCYPGLVEFQGAFYMPDSGQISIALEYMDGGSLADVIKVKK

SIPEPVLAHMLQKVLLGLRYLHEVRHLVHRDIKPANMLVNLKGEAKITDFGVSAGLDNTM

AMCATFVGTVTYMSPERIRNENYSYAADIWSLGLTILECATGKFPYNVNEGPANLMLQIL

DDPSPAPPENAFSSEFCSFVNDCLQKDADARPTCEQLLSHPFIKRYENAGVDLAAYVKGV

VNPEERLKQIAEMLAVHYYLLFNGSDGLWHHMKTFYMEDSTFSFSGNLYVGQSDIFDTLS

NIRTKLKGDRPREKIVHVVEKLHCRANEETGIAIRVSGSFIVSNQFLICGEGLQAEGMPS

LEELSIDIPSKRVGQFREQFIMHPGRSMGCYYISRQDLYIIQA*

>Bd1-1MKK3-3 Brdisv1Bd1-11022429m.p

MGWDGMGQRKRRHRAGIPIPTYPGLAASLEFCFVTAGACHTAPGPGSKIAASLFAALLFQ

RPPASTSRAIFFCFVIMAAGLEDLRRRGQPIFFDADGNVMPAPDDDSEVLDGGTINLLSR

SSDEYNINERGFHKRTIRSDDEYSSEKAFRCSCHDMHIFDSVGNGASSVVHRAIYVPVHR

VLALKKINIFEKERRQQILNEIITLSEACCYPGLVEFHGVFYTPDSGEIYFALEYMDGGS

LADIIRVKKFISEPVLSHMLQKVLLALRYLHEVRHLVHRDIKPANLLLNLKGDTKITDFG

VTSGLHDSIDMCATFLGSVTYMSPERIRNESYSYSADIWSLGLTALECATGRYPYDVNGG

EADLMLQILEDPSPTPPHDIYSEEFCSFINACLQKDADARPTCDQLLSHSFIKRYEGPGV

DLSEYNKSVHDPSERLSQIAHMLAVHYYLIFDGGDDQWCHMKTFYQQDSIFSFSGETHVG

KSEIFETLSRIRKMLKGNSPCEKIAHVMEKVYCRSHGEEGMRVRVSGSFIVGNEFVVCAD

GVRAEGMLSIDELSPDILSKQAGHFQEDFFMEPGTALGCYVISKQELHIADT*

>Bd1-1MKK4 Brdisv1Bd1-11028128m.p

MRPGGPPNARPQQPGPPGRARRRPDLTLPLPQRDLTSLAVPLPLPPPPSSAPSSASSSGS

SLSSMGAPPPPTPAGSAPPPPPPLAELERVRRIGSGAGGTGWMVRHRPTGRPYALKVLYG

NHDDAVRRQITREIAILRTAEHPAIVRCHGMYEQAGELQILLEFMDGGSLEGRRIASEAF

LADVARQVLSGIASLHRRHIVHRDIKPSTLLIASGRRVKIADFGVGRILNQTMDPCNSSV

GTIAYMSPERINTDLNDGAYDGYAGDIWSFGLSILEFYLGRFPLGENLGKQGDWAALMCA

ICYSDSPAPPPIASPEFKSFISCCLQKNPARRPSAAQLLQPRFIAGPQPQVLAAPPS*

>Bd1-1MKK5 Brdisv1Bd1-11006672m.p

MGRHRPTGRCYALKQLYGNHDDAVRRQIAREIAILRTAEPPAVVRCHGMYERGGELQILL

EYMDGGSLDGRRIAAEGFLADVARQVLSGIAYLHRRHIVHRDIKPSNLLIDSARRVKIAD

FGVGRILNQTMDPCNSSVGTIAYMSPERINTDLNDGAYDGYAGDIWSFGLSILEFYLGRF

PFGENLGKQGDWAALMVAICYNDPPEPSAAASPEFRGFISCCLQKNPAKRLSAAQLLQPP

FVAGPQPLPLAAPPS*

>Bd1-1MKK6 Brdisv1Bd1-11011292m.p

MRGKKPLKELKLSVPAQETSVDKFLTASGTFKDGELRLNQRGLRLISEEDNGDEHQSTNM

KVEDVQLSMDDLEMIQVIGKGSGGVVQLVQHKWVGTFYALKGIQMNIQEAVRKQIVQELK

INQATQSPHIVSCHQSFYHNGVIYLVLEYMDRGSLADIIKQVKTILEPYLAVLCKQVLEG

LLYLHHERHVIHRDIKPSNLLVNHKGEVKITDFGVSAVLASSIGQRDTFVGTYNYMAPER

ISGSSYDYKSDVWSLGLVILECAIGRFPYTPSEGEGWLSFYELLEAIVDQPPPGAPADQF

SPEFCSFISACIQKDPAERMSASELLNHAFIKKFEGKDLDLRILVESLEPPMNVPE*

>Bd1-1MKK10-1 Brdisv1Bd1-11001714m.p

MALLREKRLQLSLHVPTRAADAQEAGLPRRPTPAAALPLAAPPPAARASQFRVADFEKLA

VLGRGNGGTVYKVRHRETCELYALKVQHCNGDATAEAEVLSRTASPFVVRCPSALPAAAS

GDVAMLLELVDGGSLDSIVKSRSRGQAEAFSQFPEEALAEVAAQALSGLAYLHARRIVHL

DVKPGNLLVSTGGEVKIADFGIARVLPRAGGDDVRCTAYAGTAAYMSPERFDPEAHGGHY

DPYAADVWGLGVTVLELLMGRYPLLPAGQRPSWAALMCAICFGETPALSDGEASAELRGF

VAACLHKNYRRRASVAELLAHPFVAGRDVAASKCALRKLVTEASMSP*

>Bd1-1MKK10-2 Brdisv1Bd1-11010351m.p

MALVRQRRQLPHLPLPLAPFALRPPPVPAPAPPVAASPSSEAAGLRLSDFERISLLGQGN

GGTVYKARPRRAAAQPPVALKLFVAGDPSAAREAEILRLAADAPHVVRLHAVVPSSSPAA

GAEQPPPAALALELLPGGSLAGLLRRLGRAMGERPIAAVARPALPGLDALHALRVVHRDL

KPSNLLLGSHGEVKIADFGAGKVLRRRLDPCASYVGTAAYMSPERFDPEAYSGDYDPYAA

DVWSLGLAILELYLGHFPLLPAGQRPDWAALMCAICFGAAPEAPAAASEEFRDFVARCLE

KKAGQRASVAELLEHPFIAERDAEEAKRALAALVAEAELGDL*

>Bd1-1MKK10-3 Brdisv1Bd1-11001621m.p

MALLREKRLQLSLHVPTRAAEALDAAHRRPNPVAAPLAASTPAAAGASQFRLADFDKLTV

LGRGNGGTVYKVRHRETCELYALKVQHCNGDPTAAAEAEVLSRPASPFIVRCHSVLPGAA

SGDVAMLLELVDGGSLDSIVKSRRAHAFPFPEEALAEVAAQALSGLAYLHARRIVHLDIK

PGNLLVSTGGEVKVADFGIAKVLPRAGADDARCTSYAGTAAYMSPERFDPEAHGGHYDAY

AADVWGLGVTVLELLMGRYPLLPAGQRPSWPALMCAICFGETPVLSDGEASAELRGFVAA

CLRKDHTKRASVAELLAHPFVAGRDVATSKCALRKLVTEASTSP*

>Bd1-1MKK10-4 Brdisv1Bd1-11001619m.p

MASAKERRLPQLHLKLDVPTCAFRCAAPAPAPATAATPATSASRPPHGEFRLNDFDRLSV

LGRGNGGSVYKVSHRRTSALYALKIIHGGPARPGAADEEADIVRRVVDSPNVVRCYSVLP

PASGAAPALLLELVDGGSLDSLVGGGGFLPEAAVADVAAQALSGLAHLRARRVAHRDIKP

ANLLLSAAGEVKIADFGIAKVVVSGAGARARALAYEGTVAYMSPERFDSERHADADPSAA

DVWGLGVTLLELLMGRYPLLPAGQKPTWAALMCAICFGELPALPEGAASLEFRGFVAACL

RKDHRKRASVVELLAHPFVAGRDGAAARRALREAIERRCSC*

>Bd1-1MKK10-5 Brdisv1Bd1-11041771m.p

MALTVRQRRLPQLHISLDLPSCSFRCPNPPVAATASTSGEFRASDFERLAVPGRGNGGTV

YKVAPRRTSAQYALKVLHGGGDPGAAAAEADGLRRAAGSPYVVRCHSVFPAASGSGETAL

LLELVDGGSLDSVRRGVGVSVFFPEAALAEVAAQALAGLAPLPARRVVPRDIKPANLLVS

GAGGVKVADFGIAMVLPSRAGGERCAAAYEGTVAYMSPERFDSEGRAGADPRGADVWGLG

VPVLELLMGRAHVRHLLRGAAGSARRRGVHGAQGIHRRVPAEGSHEASVRRGAYQAPVRR

REEYGGVQARAPAAGGRSLNWCWHVN*

>Bd29-1MKK1 Brdisv1Bd29-11006851m.p

MRKPGKLALPSHESTIGKFLTQSGTFKDGDLLVNKDGLRIVHNSEEGEAPPIEPLDDHQL

SLDDLDAIKVIGKGSSGIVQLVRHKWTDQFFALKVIQLNIQESIRKQIAQELKISLSTQC

QYVVTCYQCFYVNGVISIVLEYMDGGSLADFLKTVRTIPEAYLAAICKQVLQGLMYLHHE

KRVIHRDLKPSNILINHRGEVKISDFGVSAIIASSSAQRDTFTGTFNYMAPERISGQKHG

YMSDIWSLGLVMLECATGNFPYPSPDSFYELLEAVVDQPPPSAPTDQFSPEFCSFISACI

QKEATDRSSAQVLSDHPFLSMYDDLNIDLADYFTTAGSPLATFKQIVL*

>Bd29-1MKK3-1 Brdisv1Bd29-11024808m.p

MAGLEELKKKLQPLLFDDPDKDGISTRVPFPEDNCDSYVVSDGGTINLLSRSFGEYNINE

HGFHKRSTGADESDFGEKAYRCASHDMHIFGPIGNGASSVVQRAIFIPVHRILALKKINI

FEKEKRQQILNEMRTLCEASCYPGLVEFQGAFYMPDSGQISIALEYMDGGSLADVIKVKK

SIPEQVLAHMLQKVLLGLRYLHEVRHLVHRDIKPANMLVNLKGEAKITDFGVSAGLDNTM

AMCATFVGTVTYMSPERIRNENYSYAADIWSLGLTILECATGKFPYNVNEGPANLMLQIL

DDPSPTPPADAYSAEFCSFVNDCLQKDPDARPTCEQLFGHPFIKRYENAGVDLIAYVKGV

VDPTERLKEIAEMLAVHYYLLFNGSDGLWHHMKTFYMEESTFSFSGNVYVGRNDIFDTLS

SIRKKLKGDRPREKIVHVVEKLHCRANGETGIAIRVSGSLIVGNQFLVCGEGLQAEGMPS

VEELSIDIPSKRVGQFREQFMMLPGISMGSFHISRQDLYIIQA*

>Bd29-1MKK3-2 Brdisv1Bd29-11037603m.p

MAGLEELKKKLQPLMFNDPDKDGVSTRVPFPEDTCDSYVVSDGGTINLLSRSFGEYNINE

HGFHKRSAGADESDFGEKAYRCASQDMHIFGPIGNGASSVVQRAIFIPVHRILALKKINI

FEKEKRQQILNEMRTLCEACCYPGLVEFQGAFYMPDSGQISIALEYMDGGSLADVIKVKK

SIPEPVLAHMLQKVLLGLRYLHEVRHLVHRDIKPANMLVNLKGEAKITDFGVSAGLDNTM

AMCATFVGTVTYMSPERIRNENYSYAADIWSLGLTILECATGKFPYNVNEGPANLMLQIL

DDPSPAPPENAFSSEFCSFVNDCLQKDADARPTCEQLLSHPFIKRYENAGVDLAAYVKGV

VNPEERLKQIAEMLAVHYYLLFNGSDGLWHHMKTFYMEDSTFSFSGNLYVGQSDIFDTLS

NIRTKLKGDRPREKIVHVVEKLHCRANEETGIAIRVSGSFIVSNQFLICGEGLQAEGMPS

LEELSIDIPSKRVGQFREQFIMHPGRSMGCYYISRQDLYIIQA*

>Bd29-1MKK3-3 Brdisv1Bd29-11005448m.p

MGWDGMGQRKRRHRAGIPIPTYPGLAASLEFCFVTAGACHTAPGPGSKIAASLFAALLFQ

RPPASTSRAIFFCFVIMAAGLEDLRRRVQPIFFDADGNVMPAPDDDSEVLDGGTINLLSR

SSDEYNINERGFHKRTIRSDDEYSSEKAFRCSCHDMHIFDSVGNGASSVVHRAIYVPVHR

VLALKKINIFEKERRQQILNEIITLSEACCYPGLVEFHGVFYTPDSGEIYFALEYMDGGS

LADIIRVKKFISEPVLSHMLQKVLLALRYLHEVRHLVHRDIKPANLLLNLKGDTKITDFG

VTSGLHDSIDMCATFLGSVTYMSPERIRNESYSYSADIWSLGLTALECATGRYPYDVNGG

EADLMLQILEDPSPTPPHDIYSEEFCSFINACLQKDADARPTCDQLLSHSFIKRYEGPGV

DLSEYNKSVHDPSERLSQIAHVSSL*

>Bd29-1MKK4 Brdisv1Bd29-11032652m.p

MELTCGPTPGMTGGAVVGADVGAXGTIAYMSPERINTDLNDGAYDGYAGDIWSFGLSILE

FYLGRFPLGENLGKQGDWAALMCAICYSDSPAPPPIASPEFKSFISCCLQKNPARRPSAA

QLLQHRFIAGPQPQVLAAPPS*

>Bd29-1MKK5 Brdisv1Bd29-11006252m.p

MRPAGSLPSPQPGTPGRPRRRPDLTLPMPQRPDVSSSLAVPLPLPPPSSLGLAQPPAAAA

AAAAPPPPPLGELERVRRVGSGAGGTVWMVRHRPTGRCYALKQLYGNHDDAVRRQIAREI

AILRTAEHPAVVRCHGMYERGGELQILLEYMDGGSLDGRRIAAEGFLADVARQVLSGIAY

LHRRHIVHRDIKPSNLLIDSARRVKIADFGVGRILNQTMDPCNSSVGTIAYMSPERINTD

LNDGAYDGYAGDIWSFGLSILEFYLGRFPFGENLGKQGDWAALMVAICYNDPPEPSAAAS

PEFRGFISCCLQKNPAKRLSAAQLLQHPFVAGPQPLPLAAPPS*

>Bd29-1MKK6 Brdisv1Bd29-11010393m.p

MRGKKPLKELKLSVPAQETSVDKFLTASGTFKDGELRLNQRGLRLISEEDNGDEHQSTNM

KVEDVQLSMDDLEMIQVIGKGSGGVVQLVQHKWVGTFYALKGIQMNIQEAVRKQIVQELK

INQATQSPHIVSCHQSFYHNGVIYLVLEYMDRGSLADIIKQVKTILEPYLAVLCKQVLEG

LLYLHHERHVIHRDIKPSNLLVNHKGEVKITDFGVSAVLASSIGQRDTFVGTYNYMAPER

ISGSSYDYKSDVWSLGLVILECAIGRFPYTPSEGEGWLSFYELLEAIVDQPPPGAPADQF

SPEFCSFISACIQKDPAERMSASELLNHAFIKKFEGKDLDLRILVESLEPPMNVPE*

>Bd29-1MKK10-1 Brdisv1Bd29-11001549m.p

MALLREKRLQLSLHVPTRAADAQEAGLHRRPNPAAALPLAATTPAARSSQFRVADFEKLA

VLGRGNGGTVYKVRHRETCELYALKVQHCNGDATAEAEVLSRTASPFVVRCHSVLPAAAS

GDVAMLLELVDGGSLDSIVKSRSRGQAEAFSQFPEEALAEVAAQALSGLAYLHARRIVHL

DVKPGNLLVSTGGEVKIADFGIARVLPRAGGDDVRCTAYAGTAAYMSPERFDPEAHGGHY

DPYAADVWGLGVTVLELLMGRYPLLPAGQXPSWAALMCAICFGETPALSDGEASAELRGF

VAACLHKNYRRRASVAELLAHPFVAGRDVAASKCALRKLVTEASMSP*

>Bd29-1MKK10-2 Brdisv1Bd29-11009613m.p

MALVRQRRQLPHLTLPLDHFALRPPPVPAPAPTVAASTSSEAAGLRLSDFERISLLGQGN

GGTVYKARHRRAAAQPPVALKLFVAGDPSAAREAEILRLAADAPHVVRLHAVVPSSSPAA

GAEQPPPAALALELLPGGSLAGLLRRLGRSMGERPIAAVARQALLGLDALHALRVVHRDL

KPSNLLLGSHGEVKIADFGAGKVLRRRLDPCASYVGTAAYMSPERFDPEAYSGDYDPYAA

DVWSLGLAILELYLGHFPLLPAGQRPDWAALMCAICFGDAPEAPAAASEEFRDFVARCLE

KKAGQRASVAELLEHPFIAERDAEEAKRALAALVAEAELGDL*

>Bd29-1MKK10-3 Brdisv1Bd29-11001467m.p

MALLREKRLQLSLHVPTRAAEALDAAHRRPNPVAATLAASTPAAARSSQFRLADFDKLTV

LGRGNGGTVYKVRHRETCELYALKVQHCNGDPTAAAEAEVLSRTASPFIVRCHSVLPGAA

SGDVAMLLELVDGGSLDSIVKSRRAHAFPFPEEALAEVAAQALSGLAYLHARRIVHLDIK

PGNLLVSTGGEVKVADFGIAKVLPRAGADDARCTSYAGTAAYMSPERFDPEAHGGHYDAY

AADVWGLGVTVLELLMGRYPLLPAGQRPSWPALMCAICFGETPVLSDGEASAELRGFVAA

CLRKDHTKRASVAELLAHPFVAGRDVATSKCALRKLVTEASTSP*

>Bd29-1MKK10-4 Brdisv1Bd29-11001465m.p

MASAKERRLPQLHLKLDVPTCAFRCAAPAPAPATAATPATSASRPPHGEFRLNDFDRLSV

LGRGNGGSVYKVSHRRTSALYALKIIHGGHARPGAADEEADIVRRVVDSPNVVRCYSVLP

TASGDAAALLLELVDGGSLDSLVGGGGFLPEAAVADVAAQALSGLAHLRARRVAHRDIKP

ANLLLSAAGEVKIADFGIAKVVVSGAGARARALAYEGTVAYMSPERFDSERHADADPYAA

DVWGLGVTLLELLMGRYPLLPAGQKPTWAALMCAICFGELPALPEGAASLEFRGFVAACL

RKDHRKRASVVELLAHPFVAGRDVAASRRALREAIERRCSC*

>Bd29-1MKK10-5 Brdisv1Bd29-11016615m.p

MALTVRQRRLPQLHISLDLPSCSFRCPNPPVAATASTSGEFRASDFERLAVLGRGNGGTV

YKVAHRRTSAQYALKVLHGGGDPGAAAAEADVLRRAADSPYVVRCHSVFPAASGSGETAL

LLELVDGGSLDSVRRGVGVSVFFPEAALAEVAAQALAGLAHLHARRVVHRDIKPANLLVS

GAGGVKVADFGIAMVLPSRAGGERCAAAYEGTVAYMSPERFDSEGRADADPRGADVWGLG

VTVLELLMGRYPLLPAGQKPTWAALMCAICFGELPALPEGAASTELRGFIAACLRKDHTK

RASVAELIKHPFVAGRNMAASRLALRRLVAGA*

>BdTR7aMKK1 Brdisv1BdTR7a1008412m.p

MRKPGKLALPSHESTIGKFLTQSGTFKDGDLLVNKDGLRIVHNSEEGEAPPIEPLDDHQL

SLDDLDAIKVIGKGSSGIVQLVRHKWTDQFFALKVIQLNIQESIRKQIAQELKISLSTQC

QYVVTCYQCFYVNGVISIVLEYMDGGSLADFLKTVRTIPEAYLAAICKQVLQGLMYLHHE

KRVIHRDLKPSNILINHRGEVKISDFGVSAIIASSSAQRDTFTGTFNYMAPERISGQKHG

YMSDIWSLGLVMLECATGNFPYPSPDSFYELLEAVVDQPPPSAPTDQFSPEFCSFISACI

QKEATDRSSAQVLSDHPFLSMYDDLNIDLADYFTTAGSPLATFKQIVL*

>BdTR7aMKK3-1 Brdisv1BdTR7a1029605m.p

MAGLEELKKKLQPLLFDDPDKDGISTRVPFPEDNCDSYVVSDGGTINLLSRSFGEYNINE

HGFHKRSTGADESDFGEKAYRCASHDMHIFGPIGNGASSVVQRAIFIPVHRILALKKINI

FEKEKRQQILNEMRTLCEASCYPGLVEFQGAFYMPDSGQISIALEYMDGGSLADVIKVKK

SIPEQVLAHMLQKVLLGLRYLHEVRHLVHRDIKPANMLVNLKGEAKITDFGVSAGLDNTM

AMCATFVGTVTYMSPERIRNENYSYAADIWSLGLTILECATGKFPYNVNEGPANLMLQIL

DDPSPTPPADAYSAEFCSFVNDCLQKDPDARPTCEQLFGHPFIKRYENAGVDLIAYVKGV

VDPTERLKEIAEMLAVHYYLLFNGSDGLWHHMKTFYMEESTFSFSGNVYVGRNDIFDTLS

SIRKKLKGDRPREKIVHVVEKLHCRANGETGIAIRVSGSLIVGNQFLVCGEGLQAEGMPS

VEELSIDIPSKRVGQFREQFMMLPGISMGSFHISRQDLYIIQA*

>BdTR7aMKK3-2 Brdisv1BdTR7a1006778m.p

MAGLEELKKKLQPLMFNDPDKDGVSTRVPFPEDTCDSYVVSDGGTINLLSRSFGEYNINE

HGFHKRSAGADESDFGEKAYRCASQDMHIFGPIGNGASSVVQRAIFIPVHRILALKKINI

FEKEKRQQILNEMRTLCEACCYPGLVEFQGAFYMPDSGQISIALEYMDGGSLADVIKVKK

SIPEPVLAHMLQKVLLGLRYLHEVRHLVHRDIKPANMLVNLKGEAKITDFGVSAGLDNTM

AMCATFVGTVTYMSPERIRNENYSYAADIWSLGLTILECATGKFPYNVNEGPANLMLQIL

DDPSPAPPENAFSSEFCSFVNDCLQKDADARPTCEQLLSHPFIKRYENAGVDLAAYVKGV

VNPEERLKQIAEMLAVHYYLLFNGSDGLWHHMKTFYMEDSTFSFSGNLYVGQSDIFDTLS

NIRTKLKGDRPREKIVHVVEKLHCRANEETGIAIRVSGSFIVSNQFLICGEGLQAEGMPS

LEELSIDIPSKRVGQFREQFIMHPGRSMGCYYISRQDLYIIQA*

>BdTR7aMKK3-3 Brdisv1BdTR7a1032485m.p

MGWDGMGQRKRRHRAGIPIPTYPGLAASLEFCFVTAGACHTAPGPGSKIAASLFAALLFQ

RPPASTSRAIFFCFVIMAAGLEDLRRRGQPIFFDADGNVMPAPDDDSEVLDGGTINLLSR

SSDEYNINERGFHKRTIRSDDEYSSEKAFRCSCHDMHIFDSVGNGASSVVHRAIYVPVHR

VLALKKINIFEKERRQQILNEIITLSEACCYPGLVEFHGVFYTPDSGEIYFALEYMDGGS

LADIIRVKKFISEPVLSHMLQKVLLALRYLHEVRHLVHRDIKPANLLLNLKGDTKITDFG

VTSGLHDSIDMCATFLGSVTYMSPERIRNESYSYSADIWSLGLTALECATGRYPYDVNGG

EADLMLQILEDPSPTPPHDIYSEEFCSFINACLQKDADARPTCDQLLSHSFIKRYEGPGV

DLSEYNKSVHDPSERLSQIAHMLAVHYYLIFDGGDDQWCHMKTFYQQDSIFSFSGETHVG

KSEIFETLSRIRKMLKGNSPCEKIAHVMEKVYCRSHGEEGMRVRVSGSFIVGNEFVVCAD

GVRAEGMLSIDELSPDILSKQAGHFQEDFFMEPGTALGCYVISKQELHIADT*

>BdTR7aMKK4 Brdisv1BdTR7a1006916m.p

MRPGGPPNARPQQPGPPGRARRRPDLPLPLPQRDLTSLAVPLPLPPPPSSAPSSASSSGS

SLSSMGAPTPPNSAGSAPPPPPPLAELERVRRIGSGAGGTVWMVRHRPTGRPYALKVLYG

NHDDAVRRQITREIAILRTAEHPAIVRCHGMYEQAGELQILLEFMDGGSLEGRRIASEAF

LADVARQVLSGIAYLHRRHIVHRDIKPSNLLIDSGRRVKIADFGVGRILNQTMDPCNSSV

GTIAYMSPERINTDLNDGAYDGYAGDIWSFGLSILEFYLGRFPLGENLGKQGDWAALMCA

ICYSDSPAPPPIASPEFKSFISCCLQKNPARRPSAAQLLQHRFIAGPQPQVLAAPPS*

>BdTR7aMKK5 Brdisv1BdTR7a1007645m.p

MRPAGSLPSPQPGTPGRPRRRPDLTLPMPQRPDVSSSLAVPLPLPPPSSLGLAQPPAAAA

AAAAPPPPPLGELERVRRVGSGAGGTGWMVRHRPTGRCYALKQLYGNHDDAVRRQIAREI

AILRTAEHPAVVRCHGMYERGGGLQILLEYMDGGSLDGRRIAAEGFLADVARQVLSGIAY

LHRRHIVHRDIKPSNLLIDSARRVKIADFGVGRILNQTMDPCNSSVGTIAYMSPERINTD

LNDGAYDGYAGDIWSFGLSILEFYLGRFPFGENLGKQGDWAALMVAICYNDPPEPSAAAS

PEFRGFISCCLQKNPAKRLSAAQLLQHPFVAGPQPLPLAAPPS*

>BdTR7aMKK6 Brdisv1BdTR7a1012554m.p

MRGKKPLKELKLSVPAQETSVDKFLTASGTFKDGELRLNQRGLRLISEEENGDEHQSTNM

KVEDVQLSMDDLEMIQVIGKGSGGVVQLVQHKWVGTFYALKGIQMNIQEAVRKQIVQELK

INQATQSPHIVSCHQSFYHNGVIYLVLEYMDRGSLADIIKQVKTILEPYLAVLCKQVLEG

LLYLHHERHVIHRDIKPSNLLVNHKGEVKITDFGVSAVLASSIGQRDTFVGTYNYMAPER

ISGSSYDYKSDVWSLGLVILECAIGRFPYTPSEGEGWLSFYELLEAIVDQPPPGAPADQF

SPEFCSFISACIQKDPAERMSASELLNHAFIKKFEGKDLDLRILVESLEPPMNVPE*

>BdTR7aMKK10-1 Brdisv1BdTR7a1001914m.p

MALLREKRLQLSLHVPTRAADAQEAGLHRRPNPAAALPLAAPTPAARSSQFRVADFEKLA

VLGRGNGGTVYKVRHRETCELYALKVQHCNGDATAEAEVLSRTASPFVVRCPSVLPAAAS

GDVAMLLELVDGGSLDSIVKSRSRGQAEAFSQFPEEALAEVAAQALSGLAYLHARRIVHL

DVKPGNLLVSTGGEVKIADFGIARVLPRAGGDDVRCTAYAGTAAYMSPERFDPEAHGGHY

DPYAADVWGLGVTVLELLMGRYPLLPAGQRPSWAALMCAICFGETPALSDGEASAELRGF

VAACLHKNYRRRASVAELLAHPFVAGRDVAASKCALRKLVTEASMSP*

>BdTR7aMKK10-2 Brdisv1BdTR7a1011719m.p

MALVRQRRQLPHLTLPLDHFALRPPPVPAPAPTVAASPSSEAAGLRLSDFERISLLGQGN

GGTVYKARHRRAAAQPPVALKLFVAGDPSAAREAEILRLAADAPHVVRLHAVVPSSSPAA

GAEQPPPAALALELLPGGSLAGLLRRLGRSMGERPIAAVARQALLGLDALHALRVVHRDL

KPSNLLLGSHGEVKIADFGAGKVLRRRLDPCASYVGTAAYMSPERFDPEAYSGDYDPYAA

DVWSLGLAILELYLGHFPLLPAGQRPDWAALMCAICFGDAPEAPAAASEEFRDFVARCLE

KKAGQRASVAELLEHPFIAERDAEEAKRALAALVAEAELGDL*

>BdTR7aMKK10-3 Brdisv1BdTR7a1001789m.p

MALLREKRLQLSLHVPTRAAEALDAAHRRPNPVAATLAASTPAAAGASQFRLADFDKLTV

LGRGNGGTVYKVRHRETCELYALKVQHCNGDPTAAAEAEVLSRTASPFIVRCHSVLPGAA

SGDVAMLLELVDGGSLDSIVKSRRAHAFPFPEEALAEVAAQALSGLAYLHARRIVHLDIK

PGNLLVSTGGEVKVADFGIAKVLPRAGADDARCTSYAGTAAYMSPERFDPEAHGGHYDAY

AADVWGLGVTVLELLMGRYPLLPAGQRPSWPALMCAICFGETPVLSDGEASAELRGFVAA

CLRKDHTKRASVAELLAHPFVAGRDVATSKCALRKLVTEASTSP*

>BdTR7aMKK10-4 Brdisv1BdTR7a1001786m.p

MASAKERRLPQLHLKLDVPTCAFRCAAPAPAPATAATPATSASRPPHGEFRLNDFDRLSV

LGRGNGGSVYKVSHRRTSALYALKIIHGAHARPGAADEEADIVRRVVDSPNVVRCHSVLP

TASGDAAALLLELVDGGSLDSLVGGGGFLPEAAVADVAAQALSGLAPPRARRVAHRDIKP

ANLLLSAAGEVKIADFGIAKVVVSGAGGRARALAYEGTVAYMSPERFDSERHADADPYAA

DVWGLGVTLLELLMGRYPLLPAGQKPTWAALMCAICFGELPAMPEGAASLEFRGFVAACL

RKDHRKRASVVELLAHPFVAGRDVAASRRALREAIERRCSC*

>BdTR7aMKK10-5 Brdisv1BdTR7a1001788m.p

MALTVRQRRLPQLHISLALPSCSFRCPNPPVAATASTSGEFRASDFERLAVLGRGNGGTV

YKVAHRRTSAQYALKVLHGGGDPGAAAAEADVLRRAADSPYVVRCHSVFPAASGSGETAL

LLELVDGGSLDSVRRGVGVSVFFPEAALAEVAAQALAGLAPLHARRVVHRDIKPANLLVS

GAGGVKVADFGIAMVLPSRAGGERCAAAYEGTVAYMSPERFDSEGRADADPRGADVWGLG

VTVLELLMGRYPLLPAGQKPTWAALMCAICFGELPALPEGAASTELRGFIAACLRKDHTK

RASVAELIKHPFVAGRNMAASRLALRRLVAGA*

>BdTR8iMKK1 Brdisv1BdTR8i1007987m.p

MRKPGKLALPSHESTIGKFLTQSGTFKDGDLLVNKDGLRIVHNSEEGEAPPIEPLDDHQL

SLDDLDAIKVIGKGSSGIVQLVRHKWTDQFFALKVIQLNIQESIRKQIAQELKISLSTQC

QYVVTCYQCFYVNGVISIVLEYMDGGSLADFLKTVRTTPEAYLAAICKQVLQGLMYLHHE

KRVIHRDLKPSNILINHRGEVKISDFGVSAIIASSSAQRDTFTGTFNYMAPERISGQKHG

YMSDIWSLGLVMLECATGNFPYPSPDSFYELLEAVVDQPPPSAPTDQFSPEFCSFISACI

QKEATDRSSAQVLSDHPFLSMYDDLNIDLADYFTTAGSPLATFKQIVL*

>BdTR8iMKK3-1 Brdisv1BdTR8i1037957m.p

MAGLEELKKKLQPLLFDDPDKDGISTRVPFPEDNCDSYVVSDGGTINLLSRSFGEYNINE

HGFHKRSTGADESDFGEKAYRCASHDMHIFGPIGNGASSVVQRAIFIPVHRILALKKINI

FEKEKRQQILNEMRTLCEASCYPGLVEFQGAFYMPDSGQISIALEYMDGGSLADVIKVKK

SIPEQVLAHMLQKVLLGLRYLHEVRHLVHRDIKPANMLVNLKGEAKITDFGVSAGLDNTM

AMCATFVGTVTYMSPERIRNENYSYAADIWSLGLTILECATGKFPYNVNEGPANLMLQIL

DDPSPTPPADAYSPEFCSFVNDCLQKDPDARPTCEQLFGHPFIKRYENAGVDLIAYVKGV

VDPTERLKEIAEMLAVHYYLLFNGSDGLWHHMKTFYMEESTFSFSGNVYVGRNDIFDTLS

SIRKKLKGDRPREKIVHVVEKLHCRANGETGIAIRVSGSLIVGNQFLVCGEGLQAEGMPS

VEELSIDIPSKRVGQFREQFMMLPGISMGSFHISRQDLYITQA*

>BdTR8iMKK3-2 Brdisv1BdTR8i1006485m.p

MAGLGELKKKLQPLMFNDPDKDGVSTRVPFPEDTCDSYVVSDGGTINLLSRSFGEYNINE

HGFHKRSAGADESDFGEKAYRCASQDMHIFGPIGNGASSVVQRAIFIPVHRILALKKINI

FEKEKRQQILNEMRTLCEACCYPGLVEFQGAFYMPDSGQISIALEYMDGGSLADVIKVKK

SIPEPVLAHMLQKVLLGLRYLHEVRHLVHRDIKPANMLVNLKGEAKITDFGVSAGLDNTM

AMCATFVGTVTYMSPERIRNENYSYAADIWSLGLTILECATGKFPYNVNEGPANLMLQIL

DDPSPAPPENAFSSEFCSFVNDCLQKDADARPTCEQLLSHPFIKRYENAGVDLAAYVKGV

VNPEERLKQIAEMLAVHYYLLFNGSDGLWHHMKTFYMEDSTFSFSGNLYVGQSDIFDTLS

NIRTKLKGDRPREKIVHVVEKLHCRANEETGIAIRVSGSFIVSNQFLICGEGLQAEGMPS

LEELSIDIPSKRVGQFREQFIMHPGRSMGCYYISRQDLYIIQA*

>BdTR8iMKK3-3 Brdisv1BdTR8i1024401m.p

MGWDGMGQRKRRHRAGIPIPTYPGLAASLEFCFVTAGACHTAPGPGSKIAASLFAALLFQ

RPPASTSRAIFFCFVIMAAGLEDLRRRGQPIFFDADGNVMPAPDDDSEVLDGGTINLLSR

SSDEYNINERGFHKRTIRSDDEYSSEKAFRCSCHDMHIFDSVGNGASSVVHRAIYVPVHR

VLALKKINIFEKERRQQILNEIITLSEACCYPGLVEFHGVFYTPDSGEIYFALEYMDGGS

LADIIRVKKFISEPVLSHMLQKVLLALRYLHEVRHLVHRDIKPANLLLNLKGDTKITDFG

VTSGLHDSIDMCATFLGSVTYMSPERIRNESYSYSADIWSLGLTALECATGRYPYDVNGG

EADLMLQILEDPSPTPPHDIYSEEFCSFINACLQKDADARPTCDQLLSHSFIKRYEGPGV

DLSEYNKSVHDPSERLSQIAHMLAVHYYLIFDGGDDQWCHMKTFYQQDSIFSFSGETHVG

KSEIFETLSRIRKMLKGNSPCEKIAHVMEKVYCRSHGEEGMRVRVSGSFIVGNEFVVCAD

GVRAEGMLSIDELSPDILSKQAGHFQEDFFMEPGTALGCYVISKQELHIADT*

>BdTR8iMKK4 Brdisv1BdTR8i1030790m.p

MRPGGPPNARPQQPGTPGRARRRPDLTLPLPQRDLTSLAVPLPLPPPPSSAPSSASSSGS

SLSSMGAPTPPNSAGSAPPPPPPLAELERVRRIGSGAGGTVWMVRHRPTGRPYALKVLYG

NHDDAVRRQITREIAILRTAEHPAIVRCHGMYEQAGELQILLEFMDGGSLEGRRIASEAF

LADVARQVLSGIAYLHRRHIVHRDIKPSNLLIDSGRRVKIADFGVGRILNQTMDPCNSSV

GTIAYMSPERINTDLNDGAYDGYAGDIWSFGLSILEFYLGRFPLGENLGKQGDWAALMCA

ICYSDSPAPPPIASPEFKSFISCCLQKNPARRPSAAQLLQHRFIAGPQPQVLAAPPS*

>BdTR8iMKK5 Brdisv1BdTR8i1007289m.p

MRPAGSLPSPQPGTPGRPRRRPDLTLPMPQRPDVSSSLAVPLPLPPPSSLGLAQPPAAAA

AAAAPPPPPLGELGRVRRVGSGAGGTVWMVRHRPTGRCYALKQLYGNHDDAVRRQIAREI

AILRTAEHPAVVRCHGMYERGGELQILLEYMDGGSLDGRRIAAEGFLADVARQVLSGIAY

LPRRHIVHRDIKPSNLLIDSARRVKIADFGVGRILNQTMDPCNSSVGTIAYMSPERINTD

LNDGAYDGYAGDIWSFGLSILEFYLGRFPFGENLGKQGDWAALMVAICYNDPPEPSAAAS

PEFRGFISCCLQKNPAKRLSAAQLLQHPFVAGPQPLPLAAPPS*

>BdTR8iMKK6 Brdisv1BdTR8i1012248m.p

MRGKKPLKELKLSVPAQETSVDKFLTASGTFKDGELRLNQRGLRLISEEDNGDEHQSTNM

KVEDVQLSMDDLEMIQVIGKGSGGVVQLVQHKWVGTFYALKGIQMNIQEAVRKQIVQELK

INQATQSPHIVSCHQSFYHNGVIYLVLEYMDRGSLADIIKQVKTILEPYLAVLCKQVLEG

LLYLHHERHVIHRDIKPSNLLVNHKGEVKITDFGVSAVLASSIGQRDTFVGTYNYMAPER

ISGSSYDYKSDVWSLGLVILECAIGRFPYTPSEGEGWLSFYELLEAIVDQPPPGAPADQF

SPEFCSFISACIQKDPAERMSASELLNHAFIKKFEGKDLDLRILVESLEPPMNVPE*

>BdTR8iMKK10-1 Brdisv1BdTR8i1001797m.p

MALLREKRLQLSLHVPPRAADAQEAGLHRRPNPAAALPLAATTPAARSSQFRVADFEKLA

VLGRGNGGTVYKVRHRETCELYALKVQHCNGDATAEAEVLSRTASPFVVRCHSVLPAAAS

GDVAMLLELVDGGSLDSIVKSRSRGQAEAFSQFPEEALAEVAAQALSGLAYLHARRIVHL

DVKPGNLLVSTGGEVKIADFGIARVLPRAGGDDVRCTAYAGTAAYMSPERFDPEAHGGHY

DPYAADVWGLGVTVLELLMGRYPLLPAGQRPSWAALMCAICFGETPALSDGEASAELRGF

VAACLHKDYRRRASVAELLAHPFVAGRDVAASKCALRKLVTEASMSP*

>BdTR8iMKK10-2 Brdisv1BdTR8i1011347m.p

MALVRQRRQLPHLTLPLDHFALRPPPVPAPAPTVAASTSSEAAGLRLSDFERISLLGQGN

GGTVYKARHRRAAAQPPVALKLFVAGDPSAAREAEILRLAADAPHVVRLHAVVPSSSPAA

GAEQPPPAALALELLPGGSLAGLLRRLGRSMGERPIAAVARQALLGLDALHALRVVHRDL

KPSNLLLGSHGEVKIADFGAGKVLRRRLDPCASYVGTAAYMSPERFDPEAYSGDYDPYAA

DVWSLGLAILELYLGHFPLLPAGQRPDWAALMCAICFGDAPEAPAAASEEFRDFVARCLE

KKAGQRASVAELLEHPFIAERDAEEAKRALAALVAEAELGDL*

>BdTR8iMKK10-3 Brdisv1BdTR8i1001692m.p

MALLREKRLQLSLHVPTRAAEALDAAHRRPNPVAATLAASTPAAARSSQFRLADFDKLTV

LGRGNGGTVYKVRHRETCELYALKVQHCNGDPTAAAEAEVLSRTASPFIVRCHSVLPGAA

SGDVAMLLELVDGGSLDSIVKSRRAHAFPFPEEALAEVAAQALSGLAYLHARRIVHLDIK

PGNLLVSTGGEVKVADFGIAKVLPRAGADDARCTSYAGTAAYMSPERFDPEAHGGHYDAY

AADVWGLGVTVLELLMGRYPLLPAGQRPSWPALMCAICFGETPVLSDGEASAELRGFVAA

CLRKDHTKRASVAELLAHPFVAGRDVATSKCALRKLVTEASTSP*

>BdTR8iMKK10-4 Brdisv1BdTR8i1001690m.p

MASAKERRLPQLPLKLDVPPCAFRCAAPAPAPATAATPATSASRPPHGEFRLNDFDRLSV

LGRGNGGSVYKVSHRRTSALYALKIIHGGHARPGAADEEADIVRRVVDSPNVVRCYSVLP

TASGDAAALLLELVDGGSLDSLVGGGGFLPEAAVADVAGQALSGLAHLRARRVAHRDIKP

ANLLLSAAGEVKIADFGIAKVVVSGAGARARALAYEGTVAYMSPERFDSERHADADPYAA

DVWGLGVTLLELLMGRYPLLPAGQKPTWAALMCAICFGELPALPEGAASLEFRGFVAACL

RKDHRKRASVVELLAPPFVAGRDVAASRRALREAIERRCSC*

>BdTR8iMKK10-5 Brdisv1BdTR8i1044418m.p

MALTVRQRRLPQLHISLDLPSCSFRCPNPPVAATASTSGEFRASDFERLAVLGRGNGGTV

YKVAHRRTSAQYALKVLHGGGDPGAAAAEADVLRRAADSPYVVRCHSVFPAASGSGETAL

LLELVDGGSLDSVRRGVGVSVFFPEAALAEVAAQALAGLAHLHARRVVHRDIKPANLLVS

GAGGVKVADFGIAMVLPSRAGGERCAAAYEGTVAYMSPERFDSEGRADADPRGADVWGLG

VTVLELLMGRYPLLPAGQKPTWAALMCAICFGELPALPEGAASTELRGFIAACLRKDHTK

RASVAELIKHPFVAGRNMAASRLALRRLVAGA*

>Mon3MKK1 Brdisv1Mon31007821m.p

MRKPGKLALPSHESTIGKFLTQSGTFKDGDLLVNKDGLRIVHNSEEGEAPPIEPLDDHQL

SLDDLDAIKVIGKGSSGIVQLVRHKWTDQFFALKVIQLNIQESIRKQIAQELKISLSTQC

QYVVTCYQCFYVNGVISIVLEYMDSGSLADFLKTVRTIPEAYLAGICKQVLQGLMYLHHE

KRVIHRDLKPSNILINHRGEVKISDFGVSAIIASSSAQRDTFTGTFNYMAPERISGQKHG

YMSDIWSLGLVMLECATGNFPYPSPDSFYELLEAVVDQPPPSAPTDQFSPEFCSFISACI

QKEATDRSSAQVLSDHPFLSMYDDLNIDLADYFTTTGSPLATFKQIVL*

>Mon3MKK3-1 Brdisv1Mon31028015m.p

MAGLEELKKKLQPLLFDDPDKDGISTRVPFPEDNCDSYVVSDGGTINLLSRSFGEYNINE

HGFHKRSTGADESDFGEKAYRCASHDMHIFGPIGNGASSVVQRAIFIPVHRILALKKINI

FEKEKRQQILNEMRTLCEASCYPGLVEFQGAFYMPDSGQISIALEYMDGGSLADVIKVKK

SIPEQVLAHMLQKVLLGLRYLHEVRHLVHRDIKPANMLVNLKGEAKITDFGVSAGLDNTM

AMCATFVGTVTYMSPERIRNENYSYAADIWSLGLTILECATGKFPYNVNEGPANLMLQIL

DDPSPTPPADAYSPEFCSFVNDCLQKDPDARPTCEQLFGHPFIKRYENAGVDLIAYVKGV

VDPTERLKEIAEMLAVHYYLLFNGSDGLWHHMKTFYMEESTFSFSGNVYVGRNDIFDTLS

SIRKKLKGDRPREKIVHVVEKLHCRANGETGIAIRVSGSLIVGNQFLVCGEGLQAEGMPS

VEELSIDIPSKRVGQFREQFIMLPGISMGSFHISRQDLYIIQA*

>Mon3MKK3-2 Brdisv1Mon31041467m.p

MAGLEELKKKLQPLMFNDPDKDGVSTRVPFPEDTCDSYVVSDGGTINLLSRSFGEYNINE

HGFHKRSAGADESDFGEKAYRCASQDMHIFGPIGNGASSVVQRAIFIPVHRILALKKINI

FEKEKRQQILNEMRTLCEACCYPGLVEFQGAFYMPDSGQISIALEYMDGGSLADVIKVKK

SIPEPVLAHMLQKVLVGLRYLHEVRHLVHRDIKPANMLVNLKGEAKITDFGVSAGLDNTM

AMCATFVGTVTYMSPERIRNENYSYAADIWSLGLTILECATGKFPYNVNEGPANLMLQIL

DDPSPAPPENAFSSEFCSFVNDCLQKDADARPTCEQLLSHPFIKRYENAGVDLAAYVKGV

VNPEERLKQIAEMLAVHYYLLFNGSDGLWHHMKTFYMEDSTFSFSGNLYVGQSDIFDTLS

NIRTKLKGDRPREKIVHVVEKLHCRANEETGIAIRVSGSFIVSNQFLICGEGLQAEGMPS

LEELSIDIPSKRVGQFREQFIMHPGRSMGCYYISRQDLYIIQA*

>Mon3MKK3-3 Brdisv1Mon31030882m.p

MGWDGMGQRKRRHRAGIPIPTYPGLAASLEFCFVTAGACHTAPGPGSKIAASLFAALLFQ

RPPASTSRAIFFCFVIMAAGLEDLRRRVQPIFFDADGNVMPAPDDDSEVLDGGTINLLSR

SSDEYNINERGFHKRTIRSDDEYSSEKAFRCSCHDMHIFDSVGNGASSVVHRAIYVPVHR

VLALKKINIFEKERRQQILNEIITLSEACCYPGLVEFHGVFYTPDSGEIYFALEYMDGGS

LADIIRVKKFISEPVLSHMLQKVLLALRYLHEVRHLVHRDIKPANLLLNLKGDTKITDFG

VTSGLHDSIDMCATFLGSVTYMSPERIRNESYSYSADIWSLGLTALECATGRYPYDVNGG

EADLMLQILEDPSPTPPHDIYSEEFCSFINACLQKDADARPTCDQLLSHSFIKRYEGPGV

DLSEYNKSVHDPSERLSQIAHMLAVHYYLIFDGGDDQWCHMKTFYQQDSIFSFSGETHVG

KSEIFETLSRIRKMLKGNSPCEKIAHVMEKVYCRSHGEEGMRVRVSGSFIVGNEFVVCAD

GVRAEGMLSIDELSPDILSKQAGHFQEDFFMEPGTALGCYVISKQELHIADT*

>Mon3MKK4 Brdisv1Mon31036999m.p

MRPGGPPNARPQQPGTPGRARRRPDLTLPLPQRDLTSLAVPLPLPPPPSSAPSSASSSGS

SLSSMGAPTPPNSAGSAPPPPPPLAELERVRRIGSGAGGTVWMVRHRPTGRPYALKVLYG

NHDDAVRRQITREIAILRTAEHPAIVRCHGMYEQAGELQILLEFMDGGSLEGRRIASEAF

LADVARQVLSGIAYLHRRHIVHRDIKPSNLLIDSGRRVKIADFGVGRILNQTMDPCNSSV

GTIAYMSPERINTDLNDGAYDGYAGDIWSFGLSILEFYLGRFPLGENLGKQGDWAALMCA

ICYSDSPAPPPIASPEFKSFISCCLQKNPARRPSAAQLLQHRFIAGPQPQVLAAPPS*

>Mon3MKK5 Brdisv1Mon31007144m.p

MRPAGSLPSPQPGTPGRPRRRPDLTLPMPQRPDVSSSLAVPLPLPPPSSLGLAQPPAAAA

AAAAAAPPPPPLGELERVRRVGSGAGGTVWMVRHRPTGRCYALKQLYGNHDDAVRRQIAR

EIAILRTAEHPAVVRCHGMYERGGELQILLEYMDGGSLDGRRIAAEGFLADVARQVLSGI

AYLHRRHIVHRDIKPSNLLIDSARRVKIADFGVGRILNQTMDPCNSSVGTIAYMSPERIN

TDLNDGAYDGYAGDIWSFGLSILEFYLGRFPFGENLGKQGDWAALMVAICYNDPPEPSAA

ASPEFRGFISCCLQKNPAKRLSAAQLLQHPFVARPQPLPLAAPPS*

>Mon3MKK6 Brdisv1Mon31011751m.p

MRGKKPLKELKLSVPAQETSVDKFLTASGTFKDGELRLNQRGLRLISEEENGDEHQSTNM

KVEDVQLSMDDLEMIQVIGKGSGGVVQLVQHKWVGTFYALKGIQMNIQEAVRKQIVQELK

INQATQSPHIVSCHQSFYHNGVIYLVLEYMDRGSLADIIKQVKTILEPYLAVLCKQVLEG

LLYLHHERHVIHRDIKPSNLLVNHKGEVKITDFGVSAVLASSIGQRDTFVGTYNYMAPER

ISGSSYDYKSDVWSLGLVILECAIGRFPYTPSEGEGWLSFYELLEAIVDQPPPGAPADQF

SPEFCSFISACIQKDPAERMSASELLNHAFIKKFEGKDLDLRILVESLEPPMNVPE*

>Mon3MKK10-1 Brdisv1Mon31001734m.p

MALLREKRLQLSLHVPTRAADAQEAGLHRRPNPAAALPLAATTPAARSSQFRVADFEKLA

VLGRGNGGTVYKVRHRETCELYALKVQHCNGDATAEAEVLSRTASPFVVRCHSVLPAAAS

GDVAMLLELVDGGSLDSIVKSRSRGQAEAFSQFPEEALAEVAAQALSGLAYLHARRIVHL

DVKPGNLLVSTGGEVKIADFGIARVLPRAGGDDVRCTAYAGTAAYMSPERFDPEAHGGHY

DPYAADVWGLGVTVLELLMGRYPLLPAGQRPSWAALMCAICFGETPALSDGEASAELRGF

VAACLHKDYRRRASVAELLAHPFVAGRDVAASKCALRKLVTEASMSP*

>Mon3MKK10-2 Brdisv1Mon31010898m.p

MALVRQRRQLPHLTLPLDHFALRPPPVPAPAPTVAASTSSEAAGLRLSDFERISLLGQGN

GGTVYKARHRRAAAQPPVALKLFVAGDPSAAREAEILRLAADAPHVVRLHAVVPSSSPAA

GAEQPPPAALALELLPGGSLAGLLRRLGRSMGERPIAAVARQALLGLDALHALRVVHRDL

KPSNLLLGSHGEVKIADFGAGKVLRRRLDPCASYVGTAAYMSPERFDPEAYSGDYDPYAA

DVWSLGLAILELYLGHFPLLPAGQRPDWAALMCAICFGDAPEAPAAASEEFRDFVARCLE

KKAGQRASVAELLEHPFIAERDAEEAKRALAALVAEAELGDL*

>Mon3MKK10-3 Brdisv1Mon31042680m.p

MALLREKRLQLSLHVPTRAAEALDAAHRRPNPVAATLAASTPAAARSSQFRLADFDKLTV

LGRGNGGTVYKVRHRETCELYALKVQHCNGDPTAAAEAEVLSRTASPFIVRCHSVLPGAA

SGDVAMLLELVDGGSLDSIVKSRRAHAFPFPEEALAEVAAQALSGLAYLHARRIVHLDIK

PGNLLVSTSGEVKVADFGIAKVLPRAGADDARCTSYAGTAAYMSPERFDPEAHGGHYDAY

AADVWGLGVTVLELLMGRYPLLPAGQRPSWPALMCAICFGETPVLSDGEASAELRGFVAA

CLRKDHTKRASVAELLAHPFVAGRDVATSKCALRKLVTEASTSP*

>Mon3MKK10-4 Brdisv1Mon31001638m.p

MASAKERRLPQLHLKLDVPTCAFRCAAPAPAPATAATPATSASRPPHGEFRLNDFDRLSV

LGRGNGGSVYKVSHRRTSALYALKIIHGAHARPGAADEEADIVRRVVDSPNVVRCHSVLP

TASGDAAALLLELVDGGSLDSLVGGGGFLPEAAVADVAAQALSGLAHLRARRVAHRDIKP

ANLLLSAAGEVKIADFGIAKVVVSGAGGRARALAYEGTVAYMSPERFDSERHADADPYAA

DVWGLGVTLLELLMGRYPLLPAGQKPTWAALMCAICFGELPALPEGAASLEFRGFVAACL

RKDHRKRASVVELLAHPFVAGRDVAASRRALREAIERRCSC*

>Mon3MKK10-5 Brdisv1Mon31043288m.p

MALTVRQRRLPQLHISLDLPSCSFRCPNPPVAATASTSGEFRASDFERLAVLGRGNGGTV

YKVAHRRTSAQYALKVLHGGGDPGAAAAEADVLRRAADSPYVVRCHSVFPAASGSGETAL

LLELVDGGSLDSVRRGVGVSVFFPEAALAEVAAQALAGLAHLHARRVVHRDIKPANLLVS

GAGGVKVADFGIAMVLPSRAGGERCAAAYEGTVAYMSPERFDSEGRADADPRGADVWGLG

VTVLELLMGRYPLLPAGQKPTWAALMCAICFGELPALPEGAASTELRGFIAACLRKDHTK

RASVAELIKHPFVAGRNMAASRLALRRLVAGA*

>Tek-2MKK1 Brdisv1Tek-21007638m.p

MRKPGKLALPSHESTIGKFLTQSGTFKDGDLLVNKDGLRIVHNSEEGEAPPIEPLDDHQL

SLDDLDAIKVIGKGSSGIVQLVRHKWTDQFFALKVIQLNIQESIRKQIAQELKISLSTQC

QYVVTCYQCFYVNGVISIVLEYMDGGSLADFLKTVRTIPEAYLAAICKQVLQGLMYLHHE

KRVIHRDLKPSNILINHRGEVKISDFGVSAIIASSSAQRDTFTGTFNYMAPERISGQKHG

YMSDIWSLGLVMLECATGNFPYPSPDSFYELLEAVVDQPPPSAPTDQFSPEFCSFISACI

QKEATDRSSAQVLSDHPFLSMYDDLNIDLADYFTTAGSPLATFKQIVL*

>Tek-2MKK3-1 Brdisv1Tek-21027306m.p

MAGLEELKKKLQPLLFDDPDKDGISTRVPFPEDNCDSYVVSDGGTINLLSRSFGEYNINE

HGFHKRSTGADESDFGEKAYRCASHDMHIFGPIGNGASSVVQRAIFIPVHRILALKKINI

FEKEKRQQILNEMRTLCEASCYPGLVEFQGAFYMPDSGQISIALEYMDGGSLADVIKVKK

SIPEQVLAHMLQKVLLGLRYLHEVRHLVHRDIKPANMLVNLKGEAKITDFGVSAGLDNTM

AMCATFVGTVTYMSPERIRNENYSYAADIWSLGLTILECATGKFPYNVNEGPANLMLQIL

DDPSPTPPADAYSPEFCSFVNDCLQKDPDARPTCEQLFGHPFIKRYENAGVDLIAYVKGV

VDPTERLKEIAEMLAVHYYLLFNGSDGLWHHMKTFYMEESTFSFSGNVYVGRNDIFDTLS

SIRKKLKGDRPREKIVHVVEKLHCRANGETGIAIRVSGSLIVGNQFLVCGEGLQAEGMPS

VEELSIDIPSKRVGQFREQFMMLPGISMGSFHISRQDLYIIQA*

>Tek-2MKK3-2 Brdisv1Tek-21041684m.p

MAGLEELKKKLQPLMFNDPDKDGVSTRVPFPEDTCDSYVVSDGGTINLLSRSFGEYNINE

HGFHKRSAGADESDFGEKAYRCASQDMHIFGPIGNGASSVVQRAIFIPVHRILALKKINI

FEKEKRQQILNEMRTLCEACCYPGLVEFQGAFYMPDSGQISIALEYMDGGSLADVIKVKK

SIPEPVLAHMLQKVLLGLRYLHEVRHLVHRDIKPANMLVNLKGEAKITDFGVSAGLDNTM

AMCATFVGTVTYMSPERIRNENYSYAADIWSLGLTILECATGKFPYNVNEGPANLMLQIL

DDPSPAPPENAFSSEFCSFVNDCLQKDADARPTCEQLLSHPFIKRYENAGVDLAAYVKGV

VNPEERLKQIAEMLAVHYYLLFNGSDGLWHHMKTFYMEDSTFSFSGNLYVGQSDIFDTLS

NIRTKLKGDRPREKIVHVVEKLHCRANEETGIAIRVSGSFIVSNQFLICGEGLQAEGMPS

LEELSIDIPSKRVGQFREQFIMHPGRSMGCYYISRQDLYIIQA*

>Tek-2MKK3-3 Brdisv1Tek-21030208m.p

MGWDGMGQRKRRHRAGIPIPTYPGLAASLEFCFVTAGACHTAPGPGSKIAASLFAALLFQ

RPPASTSRAIFFCFVIMAAGLEDLRRRVQPIFFDADGNVMPAPDDDSEVLDGGTINLLSR

SSDEYNINERGFHKRTIRSDDEYSSEKAFRCSCHDMHIFDSVGNGASSVVHRAIYVPVHR

VLALKKINIFEKERRQQILNEIITLSEACCYPGLVEFHGVFYTPDSGEIYFALEYMDGGS

LADIIRVKKFISEPVLSHMLQKVLLALRYLHEVRHLVHRDIKPANLLLNLKGDTKITDFG

VTSGLHDSIDMCATFLGSVTYMSPERIRNESYSYSADIWSLGLTALECATGRYPYDVNGG

EADLMLQILEDPSPTPPHDIYSEEFCSFINACLQKDADARPTCDQLLSHSFIKRYEGPGV

DLSEYNKSVHDPSERLSQIAHMLAVHYYLIFDGGDDQWCHMKTFYQQDSIFSFSGETHVG

KSEIFETLSRIRKMLKGNSPCEKIAHVMEKVYCRSHGEEGMRVRVSGSFIVGNEFVVCAD

GVRAEGMLSIDELSPDILSKQAGHFQEDFFMEPGTALGCYVISKQELHIADT*

>Tek-2MKK4 Brdisv1Tek-21036177m.p

MRPGGPPNARPQQPGTPGRARRRPDLTLPLPQRDLTSLAVPLPLPPPPSSAPSSASSSGS

SLSSMGAPTPPNSAGSAPPPPPPLAELERVRRIGSGAGGTVWMVRHRPTGRPYALKVLYG

NHDDAVRRQITREIAILRTAEHPAIVRCHGMYEQAGELQILLEFMDGGSLEGRRIASEAF

LADVARQVLSGIAYLHRRHIVHRDIKPSNLLIDSGRRVKIADFGVGRILNQTMDPCNSSV

GTIAYMSPERINTDLNDGAYDGYAGDIWSFGLSILEFYLGRFPLGENLGKQGDWAALMCA

ICYSDSPAPPPIASPEFKSFISCCLQKNPARRPSAAQLLQHRFIAGPQPQVLAAPPS*

>Tek-2MKK5 Brdisv1Tek-21006949m.p

MRPAGSLPSPQPGTPGRPRRRPDLTLPMPQRPDVSSSLAVPLPLPPPSSLGLAQPPAAAA

AAAAPPPPPLGELERVRRVGSGAGGTVWMVRHRPTGRCYALKQLYGNHDDAVRRQIAREI

AILRTAEHPAVVRCHGMYERGGELQILLEYMDGGSLDGRRIAAEGFLADVARQVLSGIAY

LHRRHIVHRDIKPSNLLIDSARRVKIADFGVGRILNQTMDPCNSSVGTIAYMSPERINTD

LNDGAYDGYAGDIWSFGLSILEFYLGRFPFGENLGKQGDWAALMVAICYNDPPEPSAAAS

PEFRGFISCCLQKNPAKRLSAAQLLQHPFVAGPQPLPLAAPPS*

>Tek-2MKK6 Brdisv1Tek-21011633m.p

MRGKKPLKELKLSVPAQETSVDKFLTASGTFKDGELRLNQRGLRLISEEDNGDEHQSTNM

KVEDVQLSMDDLEMIQVIGKGSGGVVQLVQHKWVGTFYALKGIQMNIQEAVRKQIVQELK

INQATQSPHIVSCHQSFYHNGVIYLVLEYMDRGSLADIIKQVKTILEPYLAVLCKQVLEG

LLYLHHERHVIHRDIKPSNLLVNHKGEVKITDFGVSAVLASSIGQRDTFVGTYNYMAPER

ISGSSYDYKSDVWSLGLVILECAIGRFPYTPSEGEGWLSFYELLEAIVDQPPPGAPADQF

SPEFCSFISACIQKDPAERMSASELLNHAFIKKFEGKDLDLRILVESLEPPMNVPE*

>Tek-2MKK10-1 Brdisv1Tek-21001708m.p

MALLREKRLQLSLHVPTRAADAQEAGLHRRPNPAAALPLAATTPAARSSQFRVADFEKLA

VLGRGNGGTVYKVRHRETCELYALKVQHCNGDATAEAEVLSRTASPFVVRCHSVLPAAAS

GDVAMLLELVDGGSLDSIVKSRSRGQAEAFSQFPEEALAEVAAQALSGLAYLHARRIVHL

DVKPGNLLVSTGGEVKIADFGIARVLPRAGGDDVRCTAYAGTAAYMSPERFDPEAHGGHY

DPYAADVWGLGVTVLELLMGRYPLLPAGQRPSWAALMCAICFGETPALSDGEASAELRGF

VAACLHKDYRRRASVAELLAHPFVAGRDVAASKCALRKLVTEASMSP*

>Tek-2MKK10-2 Brdisv1Tek-21010712m.p

MALVRQRRQLPHLTLPLDHFALRPPPVPAPAPTVAASTSSEAAGLRLSDFERISLLGQGN

GGTVYKARHRRAAAQPPVALKLFVAGDPSAAREAEILRLAADAPHVVRLHAVVPSSSPAA

GAEQPPPAALALELLPGGSLAGLLRRLGRSMGERPIAAVARQALLGLDALHALRVVHRDL

KPSNLLLGSHGEVKIADFGAGKVLRRRLDPCASYVGTAAYMSPERFDPEAYSGDYDPYAA

DVWSLGLAILELYLGHFPLLPAGQRPDWAALMCAICFGDAPEAPAAASEEFRDFVARCLE

KKAGQRASVAELLEHPFIAERDAEEAKRALAALVAEAELGDL*

>Tek-2MKK10-3 Brdisv1Tek-21001622m.p

MALLREKRLQLSLHVPTRAAEALDAAHRRPNPVAATLAASTPAAARSSQFRLADFDKLTV

LGRGNGGTVYKVRHRETCELYALKVQHCNGDPTAAAEAEVLSRTASPFIVRCHSVLPGAA

SGDVAMLLELVDGGSLDSIVKSRRAHAFPFPEEALAEVAAQALSGLAYLHARRIVHLDIK

PGNLLVSTGGEVKVADFGIAKVLPRAGADDARCTSYAGTAAYMSPERFDPEAHGGHYDAY

AADVWGLGVTVLELLMGRYPLLPAGQRPSWPALMCAICFGETPVLSDGEASAELRGFVAA

CLRKDHTKRASVAELLAHPFVAGRDVATSKCALRKLVTEASTSP*

>Tek-2MKK10-4 Brdisv1Tek-21001619m.p

MASAKERRLPQLHLKLDVPTCAFRCAAPAPAPATAATPATSASRPPHGEFRLNDFDRLSV

LGRGNGGSVYKVSHRRTSALYALKIIHGGHARPGAADEEADIVRRVVDSPNVVRCYSVLP

TASGDAAALLLELVDGGSLDSLVGGGGFLPEAAVADVAAQALSGLAHLRARRVAHRDIKP

ANLLLSAAGEVKIADFGIAKVVVSGAGARARALAYEGTVAYMSPERFDSERHADADPYAA

DVWGLGVTLLELLMGRYPLLPAGQKPTWAALMCAICFGELPALPEGAASLEFRGFVAACL

RKDHRKRASVVELLAHPFVAGRDVAASRRALREAIERRCSC*

>Tek-2MKK10-5 Brdisv1Tek-21001621m.p

MALTVRQRRLPQLHISLDLPSCSFRCPNPPVAATASTSGEFRASDFERLAVLGRGNGGTV

YKVAHRRTSAQYALKVLHGGGDPGAAAAEADVLRRAADSPYVVRCHSVFPAASGSGETAL

LLELVDGGSLDSVRRGVGVSVFFPEAALAEVAAQALAGLAHLHARRVVHRDIKPANLLVS

GAGGVKVADFGIAMVLPSRAGGERCAAAYEGTVAYMSPERFDSEGRADADPRGADVWGLG

VTVLELLMGRYPLLPAGQKPTWAALMCAICFGELPALPEGAASTELRGFIAACLRKDHTK

RASVAELIKHPFVAGRNMAASRLALRRLVAGA*

>Tek-4MKK1 Brdisv1Tek-41004208m.p

MRKPGKLALPSHESTIGKFLTQSGTFKDGDLLVNKDGLRIVHNSEEGEAPPIVPLDDHQL

SLDDLDAIKVIGKGSSGIVQLVRHKWTDQFFALKVIQLNIQESIRKQIAQELKISLSTQC

QYVVTCYQCFYVNGVISIVLEYMDGGSLADFLKTVRTIPEAYLAAICKQVLQGLMYLHHE

KRVIHRDLKPSNILINHRGEVKISDFGVSAIIASSSAQRDTFTGTFNYMAPERISGQKHG

YMSDIWSLGLVMVECATGNFPYPSPDSFYELLEAVVDQPPPSAPTDQFSPEFCSFISACI

QKEATDRSSAQVLSDHPFLSMYDDLNIDLADYFTTAGSPLATFKQIVL*

>Tek-4MKK3-1 Brdisv1Tek-41038463m.p

MLVNLKGEAKITDFGVSAGLDNTMAMCATFVGTVTYMSPERIRNENYSYAADIWSLGLTI

LECATGKFPYNVNEGPANLMLQILDDPSPTPPADAYSAEFCSFVNDCLQKDPDARPTCEQ

LFGHSFIKRYENAGVDLIAYVKGVVDPTERLKEIAEMLAVHYYLLFNGSDGLWHHMKTFY

MEESTF

>Tek-4MKK3-2 Brdisv1Tek-41026595m.p

MHIFGPIGNGASSVVQRAIFIPVHRILALKKINIFEKEKRQQILNEMRTLCEACCYPGLV

EFQGAFYMPDSGQISIALEYMDGGSLADVIKVKKSIPEPVLAHMLQKVLLGLRYLHEVRH

LVHRDIKPANMLVNLKGEAKITDFGVSAGLDNTMAMCATFVGTVTYMSPERIRNENYSYA

ADIWSLGLTILECATGKFPYNVNEGPANLMLQILDDPSPAPPENAFSSEFCSFVNDCLQK

DADARPTCEQLLSHPFIKRYENAGVDLAAYVKGVVNPEERLKQIAEMLAVHYYLLFNGSD

GLWHHMKTFYXXXXXXXXXXXXHLLASAVGLKKENTVQSA*

>Tek-4MKK3-3 Brdisv1Tek-41017143m.p

VAVLQILEDPSPTPPHDIYSEEFCSFINACLQKDADARPTCDQLLSHSFIKRYEGPGVDL

SEYNKSVHDPSERLSQIAHMLAVHYYLIFDGGDDQWCHMKTFYQQDSIFSFSGETHVGKS

EIFETLSRIRKMLKGNSPCEKIAHVMEKVYCRSHGEEGMRVRVSGSFIVGNEFVVCADGV

RAEGMLSIDELSPDILSKQAGHFQEDFFMEPGTALGCYVISKQELHIADT*

>Tek-4MKK4 Brdisv1Tek-41001069m.p

MRPGGPPNARPQQPGTPGRARRRPDLTLPLPQRDLTSLAVPLPLPPPPSSAPSSASSSGS

SLSSMGAPTPPNSAGSAPPPPPPLAELERVRRIGSGAGGTVWMVRHRPTGRPYALKVLYG

NHDDAVRRQITREIAILRTAEHPAIVRCHGMYEQAGELQILLEFMDGGSLEGRRIASEAF

LADVARQVLSGIASLHRRHIVHRDIKPSNLLIDSGRRVKIADFGVGRILNQTMDPCNSSV

GTIAYMSPERINTDLNDGAYDGYAGDIWSFGLSILEFYLGRFPLGENLGKQGDWAALMCA

ICYSDSPAPPPIASPEFKSFISCCLQKNPARRPSAAQLLQHRFIAGPQPQVLAAPPS*

>Tek-4MKK5 Brdisv1Tek-41003815m.p

MRPAGSLPSPQPGTPGRPRRRPDLTLPMPQRPDVSSSLAVPLPLPPPSSLGLAQPPAAAA

AAAAPPPPPLGELERVRRVGSGAGGTVWMVRHRPTGRCYALKQLYGNHDDAVRRQIAREI

AILRTAEHPAVVRCHGMYERGGELQILLEYMDGGSLDGRRIAAEGFLADVARQVLSGIAY

LHRRHIVHRDIKPSNLLIDSARRVKIADFGVGRILNQTMDPCNSSVGTIAYMSPERINTD

LNDGAYDGYAGDIWSFGLSILEFYLGRFPFGENLGKQGDWAALMVAICYNDPPEPSAAAS

PEFRGFISCCLQKNPAKRLSAAQLLQH

>Tek-4MKK6 Brdisv1Tek-41006374m.p

MRGKKPLKELKLSVPAQETSVDKFLTASGTFKDGELRLNQRGLRLISEEDNGDEHQSTNM

KVEDVQLSMDDLEMIQVIGKGSGGVVQLVQHKWVGTFYALKGIQMNIQEAVRKQIVQELK

INQATQSPHIVSCHQSFYHNGVIYLVLEYMDRGSLADIIKQVKTILEPYLAVLCKQVLEG

LLYLHHERHVIHRDIKPSNLLVNHKGEVKITDFGVSAVLASSIGQRDTFVGTYNYMAPER

ISGSSYDYKSDVWSLGLVILECAIGRFPYTPSEGEGWLSFYELLEAIVDQPPPGAPADQF

SPEFCSFISACIQKDPAERMSASELLNHAFIKKFEGKDLDLRILVESLEPPMNVPE*

>Tek-4MKK10-1 Brdisv1Tek-41001123m.p

MALLREKRLQLSLHVPTRAADAQEAGLHRRPNPAAALPRAATPPAARSSQFRVADFEKLA

VLGRGNGGTVYKVRHRETCELYALKVQHCNGDATAEAEVLSRTASPFVVRCHSVLPAAAS

GDVAMLLELVDGGSLDSIVKSRSRGQAEAFSQFPEEALAEVAAQALSGLAYLHARRIVHL

DVKPGNLLVSTGGEVKIADFGIARVLPRAGGDDVRCTAYAGTAAYMSPERFDPEAHGGHY

DPYAADVWGLGVTVLELLMGRYPLLPAGQRPSWAALMCAICFGETPALSDGEASAELRGF

VAACLHKNYRRRASVAELLAHPFVAGRDVAASKCALRKLVTEASMSP*

>Tek-4MKK10-2 Brdisv1Tek-41001073m.p

MGERPIAAVARQALLGLDALHALRVVHRDLKPSNLLLGSHGEVKIADFGAGKVLRRRLDP

CASYVGTAAYMSPERFDPEAYSGDYDPYAADVWSLGLAILELYLGHFPLLPAGQRPDWAA

LMCAICFGDAPEAPAAASEEFRDFVARCLEKKAGQRASVAELLEHPFIAERDAEEAKRAL

AALVAEAELGDL*

>Tek-4MKK10-3 Brdisv1Tek-41001076m.p

MALLREKRLQLSLHVPTRAAEALDAAHRRPTPVAATLAASTPAAARSSQFRLADFDKLTV

LGRGNGGTVYKVRHRETCELYALKVQHCNGDPTAAAEAEVLSRTASPFIVRCHSVLPGAA

SGDVAMLLELVDGGSLDSIVKSRRAHAFPFPEEALAEVAAQALSGLAYLHARRIVHLDIK

PGNLLVSTGGEVKVADFGIAKVLPRAGADDARCTSYAGTAAYMSPERFDPEAHGGHYDAY

AADVWGLGVTVLELLMGRYPLLPAGQRPSWPALMCAICFGETPVLSDGEASAELRGFVAA

CLRKDHTKRASVAELLAHPFVAGRDVATSKCALRKLVTEASTSP*

>Tek-4MKK10-4 Brdisv1Tek-41001074m.p

MVRKRRARDLVAQLAPRDHGPDRAAHVSHRRTSALYALKIIHGGHARPGAADEEADIVRR

VVDSPNVVRCYSVLPTASGDAAALLLELVDGGSLDSLVGGGGFLPEAAVADVAAQGPSGL

APLRARRVAHRDIKPANLLLSAAGEVKIADFGIAKVVVSGAGGRARALAYEGTVAYMSPE

RFDSERHADADPYAADVWGLGVTLLELLMGRYPLLPAGQKPTWAALMCAICFGELPALPE

GAASLEFRGFVAACLRKDHRKRASVVELLAHPFVAGRDVAASRRALREAIERRCSC*

>BdMKK1 Bradi1g51000

MRKPGKLALPSHESTIGKFLTQSGTFKDGDLLVNKDGLRIVHNSEEGEAPPIVPLDDHQLSLDDLDAIKVIGKGSSGIVQLVRHKWTDQFFALKVIQLNIQESIRKQIAQELKISLSTQCQYVVTCYQCFYVNGVISIVLEYMDGGSLADFLKTVRTIPEAYLAAICKQVLQGLMYLHHEKRVIHRDLKPSNILINHRGEVKISDFGVSAIIASSSAQRDTFTGTFNYMAPERISGQKHGYMSDIWSLGLVMLECATGNFPYPSPDSFYELLEAVVDQPPPSAPTDQFSPEFCSFISACIQKEATDRSSAQVLSDHPFLSMYDDLNIDLADYFTTAGSPLATFKQIVL*

>BdMKK3-1 Bradi4g39490

MAGLEELKKKLQPLLFDDPDKDGISTRVPFLEDNCDSYVVSDGGTINLLSRSFGEYNINEHGFHKRSTGADESDFGEKAYRCASHDMHIFGPIGNGASSVVQRAIFIPVHRILALKKINIFEKEKRQQILNEMRTLCEASCYPGLVEFQGAFYMPDSGQISIALEYMDGGSLADVIKVKKSIPEQVLAHMLQKVLLGLRYLHEVRHLVHRDIKPANMLVNLKGEAKITDFGVSAGLDNTMAMCATFVGTVTYMSPERIRNENYSYAADIWSLGLTILECATGKFPYNVNEGPANLMLQILDDPSPTPPADAYSPEFCSFVNDCLQKDPDARPTCEQLFGHPFIKRYENAGVDLIAYVKGVVDPTERLKEIAEMLAVHYYLLFNGSDGLWHHMKTFYMEESTFSFSGNVYVGRNDIFDTLSSIRKKLKGDRPREKIVHVVEKLHCRANGETGIAIRVSGSLIVGNQFLVCGEGLQAEGMPSVEELSIDIPSKRVGQFREQFMMLPGISMGSFHISRQDLYIIQA*

>BdMKK3-2 Bradi1g41860

MAGLEELKKKLQPLMFNDPDKDGFSTRVPFPEDTCDSYVVSDGGTINLLSRSFGEYNINEHGFHKRSAGADESDFGEKAYRCASQDMHIFGPIGNGASSVVQRAIFIPVHRILALKKINIFEKEKRQQILNEMRTLCEACCYPGLVEFQGAFYMPDSGQISIALEYMDGGSLADVIKVKKSIPEPVLAHMLQKVLLGLRYLHEVRHLVHRDIKPANMLVNLKGEAKITDFGVSAGLDNTMAMCATFVGTVTYMSPERIRNENYSYAADIWSLGLTILECATGKFPYNVNEGPANLMLQILDDPSPAPPENAFSSEFCSFVNDCLQKDADARPTCEQLLSHPFIKRYENAGVDLAAYVKGVVNPEERLKQIAEMLAVHYYLLFNGSDGLWHHMKTFYMEDSTFSFSGNLYVGQSDIFDTLSNIRTKLKGDRPREKIVHVVEKLHCRANEETGIAIRVSGSFIVSNQFLICGEGLQAEGMPSLEELSIDIPSKRVGQFREQFIMHPGRSMGCYYISRQDLYIIQA*

>BdMKK3-3 Bradi3g11260

MDGGWDGMGQRKRRHRAGIPIPTYPGLAASLEFCFVTAGACHTAPGPGSKIAASLFAALLFQRPPASTSRAIFFCFVIMAAGLEDLRRRVQPIFFDADGNVMPAPDDDSEVLDGGTINLLSRSSDEYNINERGFHKRTIRSDDEYSSEKAFRCSCHDMHIFDSVGNGASSVVHRAIYVPVHRVLALKKINIFEKERRQQILNEIITLSEACCYPGLVEFHGVFYTPDSGEIYFALEYMDGGSLADIIRVKKFISEPVLSHMLQKVLLALRYLHEVRHLVHRDIKPANLLLNLKGDTKITDFGVTSGLHDSIDMCATFLGSVTYMSPERIRNESYSYSADIWSLGLTALECATGRYPYDVNGGEADLMLQKDADARPTCDQLLSHSFIKRYEGPGVDLSEYNKSVHDPSERLSQIAHMLAVHYYLIFDGGDDQWCHMKTFYQQDSIFSFSGETHVGKSEIFETLSRIRKMLKGNSPCEKIAHVMEKVYCRSHGEEGMRVRVSGSFIVGNEFVVCADGVRAEGMLSIDELSPDILSKQAGHFQEDFFMEPGTALGCYVISKQELHIADT*

>BdMKK4 Bradi3g53650

MRPGGPPNARPQQPGTPGRARRRPDLTLPLPQRDLTSLAVPLPLPPPPSSAPSSASSSGSSLSSMGAPTPPNSAGSAPPPPPPLAELERVRRIGSGAGGTVWMVRHRPTGRPYALKVLYGNHDDAVRRQITREIAILRTAEHPAIVRCHGMYEQAGELQILLEFMDGGSLEGRRIASEAFLADVARQVLSGIAYLHRRHIVHRDIKPSNLLIDSGRRVKIADFGVGRILNQTMDPCNSSVGTIAYMSPERINTDLNDGAYDGYAGDIWSFGLSILEFYLGRFPLGENLGKQGDWAALMCAICYSDSPAPPPIASPEFKSFISCCLQKNPARRPSAAQLLQHRFIAGPQPQVLAAPPS*

>BdMKK5 Bradi1g46880

MRPAGSLPSPQPGTPGRPRRRPDLTLPMPQRPDVSSSLAVPLPLPPPSSLGLAQPPAAAAAAAAPPPPPLGELERVRRVGSGAGGTVWMVRHRPTGRCYALKQLYGNHDDAVRRQIAREIAILRTAEHPAVVRCHGMYERGGELQILLEYMDGGSLDGRRIAAEGFLADVARQVLSGIAYLHRRHIVHRDIKPSNLLIDSARRVKIADFGVGRILNQTMDPCNSSVGTIAYMSPERINTDLNDGAYDGYAGDIWSFGLSILEFYLGRFPFGENLGKQGDWAALMVAICYNDPPEPSAAASPEFRGFISCCLQKNPAKRLSAAQLLQHPFVAGPQPLPLAAPPS*

>BdMKK6 Bradi1g75150

MRGKKPLKELKLSVPAQETSVDKFLTASGTFKDGELRLNQRGLRLISEEENGDEHQSTNMKVEDVQLSMDDLEMIQVIGKGSGGVVQLVQHKWVGTFYALKGIQMNIQEAVRKQIVQELKINQATQSPHIVSCHQSFYHNGVIYLVLEYMDRGSLADIIKQVKTILEPYLAVLCKQVLEGLLYLHHERHVIHRDIKPSNLLVNHKGEVKITDFGVSAVLASSIGQRDTFVGTYNYMAPERISGSSYDYKSDVWSLGLVILECAIGRFPYTPSEGEGWLSFYELLEAIVDQPPPGAPADQFSPEFCSFISACIQKDPAERMSASELLNHAFIKKFEGKDLDLRILVESLEPPMNVPE*

>BdMKK10-1 Bradi1g11525

MALLREKRLQLSLHVPTRAADAQEAGLHRRPNPAAALPLAATTPAARSSQFRVADFEKLAVLGRGNGGTVYKVRHRETCELYALKVQHCNGDATAEAEVLSRTASPFVVRCHSVLPAAASGDVAMLLELVDGGSLDSIVKSRSRGQAEAFSQFPEEALAEVAAQALSGLAYLHARRIVHLDVKPGNLLVSTGGEVKIADFGIARVLPRAGGDDVRCTAYAGTAAYMSPERFDPEAHGGHYDPYAADVWGLGVTVLELLMGRYPLLPAGQRPSWAALMCAICFGETPALSDGEASAELRGFVAACLHKDYRRRASVAELLAHPFVAGRDVAASKCALRKLVTEASMSP*

>BdMKK10-2 Bradi1g69400

MALVRQRRQLPHLTLPLDHFALRPPPAPAPTVAASTSSEAAGLRLSDFERISLLGQGNGGTVYKARHRRAAAQPPVALKLFVAGDPSAAREAEILRLAADAPHVVRLHAVVPSSSPAAGAEQPPPAALALELLPGGSLAGLLRRLGRSMGERPIAAVARQALLGLDALHALRVVHRDLKPSNLLLGSHGEVKIADFGAGKVLRRRLDPCASYVGTAAYMSPERFDPEAYSGDYDPYAADVWSLGLAILELYLGHFPLLPAGQRPDWAALMCAICFGDAPEAPAAASEEFRDFVARCLEKKAGQRASVAELLEHPFIAERDAEEAKRALAALVAEAELGDL*

>BdMKK10-3 Bradi1g10800

MALLREKRLQLSLHVPTRAAEALDAVHRRPNPVAATLAASTPAAARSSQFRLADFDKLTVLGRGNGGTVYKVRHRETCELYALKVQHCNGDPTAAAEAEVLSRTASPFIVRCHSVLPGAASGDVAMLLELVDGGSLDSIVKSRRAHAFPFPEEALAEVAAQALSGLAYLHARRIVHLDIKPGNLLVSTGGEVKVADFGIAKVLPRAGADDARCKSYAGTAAYMSPERFDPEAHGGHYDAYAADVWGLGVTVLELLMGRYPLLPAGQRPSWPALMCAICFGETPVLSDGEASAELRGFVAACLRKDHTKRASVAELLAHPFVAGRDVATSKCALRKLVTEASTSP*

>BdMKK10-4 Bradi1g10770

MASAKERRLPQLHLKLDVPTCAFRCAAPAPAPATAATPATSASRPPHGEFRLNDFDRLSVLGRGNGGSVYKVSHRRTSALYALKIIHGAHARPGAADEEADIVRRVVDSPNVVRCHSVLPTASGDAAALLLELVDGGSLDSLVGGGGFLPEAAVADVAAQALSGLAHLRARRVAHRDIKPANLLLSAAGEVKIADFGIAKVVVSGAGGRARALAYEGTVAYMSPERFDSERHADADPYAADVWGLGVTLLELLMGRYPLLPAGQKPTWAALMCAICFGELPALPEGAASLEFRGFVAACLRKDHRKRASVVELLAHPFVAGRDVAASRRALREAIERRCSC*

>BdMKK10-5 Bradi1g10790

MALTVRQRRLPQLHISLDLPSCSFRCPNPPVAATASTSGEFRASDFERLAVLGRGNGGTVYKVAHRRTSAQYALKVLHGGGDPGAAAAEADVLRRAADSPYVVRCHSVFPAASGSGETALLLELVDGGSLDSVRRGVGVSVFFPEAALAEVAAQALAGLAHLHARRVVHRDIKPANLLVSGAGGVKVADFGIAMVLPSRAGGERCAAAYEGTVAYMSPERFDSEGRADADPRGADVWGLGVTVLELLMGRYPLLPAGQKPTWAALMCAICFGELPALPEGAASTELRGFIAACLRKDHTKRASVAELIKHPFVAGRNMAASRLALRRLVAGA*

>Adi-10MKK1 Brdisv1Adi-101005559m.p

MRKPGKLALPSHESTIGKFLTQSGTFKDGDLLVNKDGLRIVHNSEEGEAPPIEPLDDHQL

SLDDLDAIKVIGKGSSGIVQLVRHKWTDQFFALKVIQLNIQESIRKQIAQELKISLSTQC

QYVVTCYQCFYVNGVISIVLEYMDGGSLADFLKTVRTIPEAYLAAIFKQVLQGLMYLHHE

KRVIHRDLKPSNILINHRGEVKISDFGVSAIIASSSAQRDTFTGTFNYMAPERISGQKHG

YMSDIWSLGLVMLECATGNFPYPSPDSFYELLEAVVDQPPPSAPTDQFSPEFCSFISACI

QKEATDRSSAQVLSDHPFLSMYDDLNIDLADYFTTAGSPLATFKQIVL*

>Adi-10MKK3-1 Brdisv1Adi-101020471m.p

MAGLEELKKKLQPLLFDDPDKDGISTRVPFLEDNCDSYVVSDGGTINLLSRSFGEYNINE

HGFHKRSTGADESDFGEKAYRCASHDMHIFGPIGNGASSVVQRAIFIPVHRILALKKINI

FEKEKRQQILNEMRTLCEASCYPGLVEFQGAFYMPDSGQISIALEYMDGGSLADVIKVKK

SIPEQVLAHMLQKVLLGLRYLHEVRHLVHRDIKPANMLVNLKGEAKITDFGVSAGLDNTM

AMCATFVGTVTYMSPERIRNENYSYAADIWSLGLTILECATGKFPYNVNEGPANLMLQIL

DDPSPTPPADAYSPEFCSFVNDCLQKDPDARPTCEQLFGHPFIKRYENAGVDLIAYVKGV

VDPTERLKEIAEMLAVHYYLLFNGSDGLWHHMKTFYMEESTFSFSGNVYVGRNDIFDTLS

SIRKKLKGDRPREKIVHVVEKLHCRANGETGIAIRVSGSLIVGNQFLVCGEGLQAEGMPS

VEELSIDIPSKRVGQFREQFMMLPGISMGSFHISRQDLYIIQA*

>Adi-10MKK3-2 Brdisv1Adi-101031183m.p

MAGLGELKKKLQPLMFNDPDKDGFSTRVPFPEDTCDSYVVSDGGTINLLSRSFGEYNINE

HGFHKRSAGADESDFGEKAYRCASQDMHIFGPIGNGASSVVQRAIFIPVHRILALKKINI

FEKEKRQQILNEMRTLCEACCYPGLVEFQGAFYMPDSGQISIALEYMDGGSLADVIKVKK

SIPEPVLAHMLQKVLLGLRYLHEVRHLVHRDIKPANMLVNLKGEAKITDFGVSAGLDNTM

AMCATFVGTVTYMSPERIRNENYSYAADIWSLGLTILECATGKFPYNVNEGPANLMLQIL

DDPSPAPPENAFSSEFCSFVNDCLQKDADARPTCEQLLSHPFIKRYENAGVDLAAYVKGV

VNPEERLKQIAEMLAVHYYLLFNGSDGLWHHMKTFYMEDSTFSFSGNLYVGQSDIFDTLS

NIRTKLKGDRPREKIVHVVEKLHCRANEETGIAIRVSGSFIVSNQFLICGEGLQAEGMPS

LEELSIDIPSKRVGQFREQFIMHPGRSMGCYYISRQDLYIIQA*

>Adi-10MKK3-3 Brdisv1Adi-101022600m.p

MDGGWDGMGQRKRRHRAGIPIPPYPGLAASLEFCFVTAGACHTAPGPGSKIAASLFAALL

FQRPPASTSRAIFFCFVIMAAGLEGLRRRGQPIFFDADGNVMPAPDDDSEVLDGGTINLL

SRSSDEYNINERGFHKRTIRSDDEYSSEKAFRCSCHDMHIFDSVGNGASSVVHRAIYVPV

HRVLALKKINIFEKERRQQILNEIITLSEACCYPGLVEFHGVFYTPDSGEIYFALEYMDG

GSLADIIRVKKFISEPVLSHMLQKVLLALRYLHEVRHLVHRDIKPANLLLNLKGDTKITD

FGVTSGLHDSIDMCATFLGSVTYMSPERIRNESYSYSADIWSLGLTALECATGRYPYDVN

GGEADLMLQILEDPSPTPPHDIYSEEFCSFINACLQKDADARPTCDQLLSHSFIKRYEGP

GVDLSEYNKSVHDPSERLSQIAHMLAVHYYLIFDGGDDQWCHMKTFYQQDSIFSFSGETH

VGKSEIFETLSRIRKMLKGNSPCEKIAHVMEKVYCRSHGEEGMRVRVSGSFIVGNEFVVC

ADGVRAEGMLSIDELSPDILSKQAGHFQEDFFMEPGTALGCYVISKQELHIADT*

>Adi-10MKK4 Brdisv1Adi-101022194m.p

MYEQAGELQILLEFMDGGSLEGRRIASEAFLADVARQVLSGIAYLPRRHIVHRDIKPSTL

LIDSGRRVKIADFGVGRILNQTMDPCNSSVGTIAYMSPERINTDLNDGAYDGYAGDIWSF

GLSILEFYLGRFPLGENLGKQGDWAALMCAICYSDSPAPPPIASPEFKSFISCCLQKNPA

RRPSAAQLLQHRFIAGPQPQVLAAPPP*

>Adi-10MKK5 Brdisv1Adi-101005080m.p

GFLADVARQVLSGIAYLHRRHIVHRDIKPSNLLIDSARRVKIADFGVGRILNQTMDPCNS

SVGTIAYMSPERINTDLNDGAYDGYAGDIWSFGLSILEFYLGRFPFGENLGKQGDWAALM

VAICYNDPPEPSAAASPEFRGFISCCLQKNPAKRLSAAQLLQHPFVAGPQPLPPPS*

>Adi-10MKK6 Brdisv1Adi-101008538m.p

MRGKKPLKELKLSVPAQETSVDKFLTASGTFKDGELRLNQRGLRLISEEENGDEHQSTNM

KVEDVQLSMDDLEMIQVIGKGSGGVVQLVQHKWVGTFYALKGIQMNIQEAVRKQIVQELK

INQATQSPHIVSCHQSFYHNGVIYLVLEYMDRGSLADIIKQVKTIREPYLAVLCKQVLEG

LLYLHHERHVIHRDIKPSNLLVNHKGEVKITDFGVSAVLASSIGQRDTFVGTYNYMAPER

ISGSSYDYKSDVWSLGLVILECAIGRFPYTPSEGEGWLSFYELLEAIVDQPPPGAPADQF

SPEFCSFISACIQKDPAERMSASELLNHAFIKKFEGKDLDLRILVESLEPPMNVPE*

>Adi-10MKK10-1 Brdisv1Adi-101039188m.p

MDPSASPPSVFSRRRRGSVGRRKKGRREEEVAAPVGELMEEEGVAASEVEPMEELAVLGR

GNGGTVYKVRHRETCELYALKVQHCNGDATAEAEVLSRTASPFVVRCHSVLPAAASGDVA

MLLELVDGGSLDSIVKSRSRGQAEAFSQFPEEALAEVAAQALSGLAYLHARRIVHLDVKP

GNLLVSTGGEVKIADFGIARVLPRAGGDDVRCTAYAGTAAYMSPERFDPEAHGGHYDPYA

ADVWGLGVTVLELLMGRYPLLPAGQRPSWAALMCAICFGETPALSDGEASAELRGFVAAC

LHKDYRRRASVAELLAHPFVAGRDVAASKCALRKLVTEASMSP*

>Adi-10MKK10-2 Brdisv1Adi-101007868m.p

MALVRQRRQLPHLTLPLDHFALRPPPAPAPPALLGLDALHALRVVHRDLKPSNLLLGSHG

EVKIADFGAGKVLRRRLDPCASYVGTAAYMSPERLGLAILELYLGHFPLLPAGQRPDWAA

LMCAICFGDAPEAPAAASEEFRDFVARCLEKKAGQRASVAELLEHPFIAERDAEEAKRAL

AALVAEAELGDL*

>Adi-10MKK10-5 Brdisv1Adi-101038807m.p

MGVLPVKSLVERSESNTGSAAVASKLRPALAGLAHLHARRVVHRDIKPANLLVSGAGGVK

VADFGIAMVLPSRAGGERCAAAYEGTVAYMSPERFDSEGRADADPRGADVWGLGVTVLEL

LMGRYPLLPAGQKPTWAALMCAICFGELPALPEGAASTELRGFIAACLRKDHTKRASVAE

LIKHPFVAGRNMAASRLALRRLVAGA*

>Adi-12MKK1 Brdisv1Adi-121007566m.p

MRKPGKLALPSHESTIGKFLTQSGTFKDGDLLVNKDGLRIVHNSEEGEAPPIEPLDDHQL

SLDDLDAIKVIGKGSSGIVQLVRHKWTDQFFALKVIQLNIQESIRKQIAQELKISLSTQC

QYVVTCYQCFYVNGVISIVLEYMDGGSLADFLKTVRTIPEAYLAAIFKQVLQGLMYLHHE

KRVIHRDLKPSNILINHRGEVKISDFGVSAIIASSSAQRDTFTGTFNYMAPERISGQKHG

YMSDIWSLGLVMLECATGNFPYPSPDSFYELLEAVVDQPPPSAPTDQFSPEFCSFISACI

QKEATDRSSAQVLSDHPFLSMYDDLNIDLADYFTTAGSPLATFKQIVL*

>Adi-12MKK3-1 Brdisv1Adi-121026937m.p

MAGLEELKKKLQPLLFDDPDKDGISTRVPFLEDNCDSYVVSDGGTINLLSRSFGEYNINE

HGFHKRSTGADESDFGEKAYRCASHDMHIFGPIGNGASSVVQRAIFIPVHRILALKKINI

FEKEKRQQILNEMRTLCEASCYPGLVEFQGAFYMPDSGQISIALEYMDGGSLADVIKVKK

SIPEQVLAHMLQKVLLGLRYLHEVRHLVHRDIKPANMLVNLKGEAKITDFGVSAGLDNTM

AMCATFVGTVTYMSPERIRNENYSYAADIWSLGLTILECATGKFPYNVNEGPANLMLQIL

DDPSPTPPADAYSPEFCSFVNDCLQKDPDARPTCEQLFGHPFIKRYENAGVDLIAYVKGV

VDPTERLKEIAEMLAVHYYLLFNGSDGLWHHMKTFYMEESTFSFSGNVYVGRNDIFDTLS

SIRKKLKGDRPREKIVHVVEKLHCRANGETGIAIRVSGSLIVGNQFLVCGEGLQAEGMPS

VEELSIDIPSKRVGQFREQFMMLPGISMGSFHISRQDLYIIQA*

>Adi-12MKK3-2 Brdisv1Adi-121040653m.p

MAGLEELKKKLQPLMFNDPDKDGFSTRVPFPEDTCDSYVVSDGGTINLLSRSFGEYNINE

HGFHKRSAGADESDFGEKAYRCASQDMHIFGPIGNGASSVVQRAIFIPVHRILALKKINI

FEKEKRQQILNEMRTLCEACCYPGLVEFQGAFYMPDSGQISIALEYMDGGSLADVIKVKK

SIPEPVLAHMLQKVLLGLRYLHEVRHLVHRDIKPANMLVNLKGEAKITDFGVSAGLDNTM

AMCATFVGTVTYMSPERIRNENYSYAADIWSLGLTILECATGKFPYNVNEGPANLMLQIL

DDPSPAPPENAFSSEFCSFVNDCLQKDADARPTCEQLLSHPFIKRYENAGVDLAAYVKGV

VNPEERLKQIAEMLAVHYYLLFNGSDGLWHHMKTFYMEDSTFSFSGNLYVGQSDIFDTLS

NIRTKLKGDRPREKIVHVVEKLHCRANEETGIAIRVSGSFIVSNQFLICGEGLQAEGMPS

LEELSIDIPSKRVGQFREQFIMHPGRSMGCYYISRQDLYIIQA*

>Adi-12MKK3-3 Brdisv1Adi-121029689m.p

MDGGWDGMGQRKRRHRAGIPIPTYPGLAASLEFCFVTAGACHTAPGPGSKIAASLFAALL

FQRPPASTSRAIFFCFVIMAAGLEDLRRRGQPIFFDADGNVMPAPDDDSEVLDGGTINLL

SRSSDEYNINERGFHKRTIRSDDEYSSEKAFRCSCHDMHIFDSVGNGASSVVHRAIYVPV

HRVLALKKINIFEKERRQQILNEIITLSEACCYPGLVEFHGVFYTPDSGEIYFALEYMDG

GSLADIIRVKKFISEPVLSHMLQKVLLALRYLHEVRHLVHRDIKPANLLLNLKGDTKITD

FGVTSGLHDSIDMCATFLGSVTYMSPERIRNESYSYSADIWSLGLTALECATGRYPYDVN

GGEADLMLQILEDPSPTPPHDIYSEEFCSFINACLQKDADARPTCDQLLSHSFIKRYEGP

GVDLSEYNKSVHDPSERLSQIAHMLAVHYYLIFDGGDDQWCHMKTFYQQDSIFSFSGETH

VGKSEIFETLSRIRKMLKGNSPCEKIAHVMEKVYCRSHGEEGMRVRVSGSFIVGNEFVVC

ADGVRAEGMLSIDELSPDILSKQAGHFQEDFFMEPGTALGCYVISKQELHIADT*

>Adi-12MKK4 Brdisv1Adi-121001565m.p

MLYISGGRXGSAPPPPPPLAELERVRRIGSGAGGTVWMVRHRPTGRPYALKVLYGNHDDA

VRRQITREIAILRTAEHPAIVRCHGMYEQAGELQILLEFMDGGSLGARRIASEAFLADVA

RQVLSGIASPPRRHIVHRDIKPSNLLIDSGRRVKIADFGVGRILNQTMDPCNSSVGTIAY

MSPERINTDLNDGAYDGYAGDIWSFGLSILEFYLGRFPLGENLGKQGDWAALMCAICYSD

SPAPPPIASPEFKSFISCCLQKNPARRPSAAQLLQHRFIAGPQPQVLAAPPS*

>Adi-12MKK5 Brdisv1Adi-121001563m.p

MRPAGSLPSPQPGTPGRPRRRPDLTLPMPQRPDVSSSLALERVRRVGSGAGGTVWMVRHR

PTGRCYALKQLYGNHDDAVRRQIAREIAILRTAEHPAVVRCHGMYERGGELQILLEYMDG

GSLGGRRIAAEGFLADVARQVLSGIAYLHRRHIVHRDIKPSNLLIDSARRVKIADFGVGR

ILNQTMDPCNSSVGTIAYMSPERINTDLNDGAYDGYAGDIWSFGLSILEFYLGRFPFGEN

LGKQGDWAALMVAICYNDPPEPPAAASPEFRGFISCCLQKNPAKRLSAAQLLQHPFVAGP

QPLPLAAPPS*

>Adi-12MKK6 Brdisv1Adi-121011367m.p

MRGKKPLKELKLSVPAQETSVDKFLTASGTFKDGELRLNQRGLRLISEEENGDEHQSTNM

KVEDVQLSMDDLEMIQVIGKGSGGVVQLVQHKWVGTFYALKGIQMNIQEAVRKQIVQELK

INQATQSPHIVSCHQSFYHNGVIYLVLEYMDRGSLADIIKQVKTILEPYLAVLCKQVLEG

LLYLHHERHVIHRDIKPSNLLVNHKGEVKITDFGVSAVLASSIGQRDTFVGTYNYMAPER

ISGSSYDYKSDVWSLGLVILECAIGRFPYTPSEGEGWLSFYELLEAIVDQPPPGAPADQF

SPEFCSFISACIQKDPAERMSASELLNHAFIKKFEGKDLDLRILVESLEPPMNVPE*

>Adi-12MKK10-1 Brdisv1Adi-121001666m.p

MALLREKRLQLSLHVPTRAADAQEAGLHRRPNPAAALPLAATTPAARWSQFRVADFEKLA

VLGRGNGGTVYKVRHRETCELYALKVQHCNGDATAEAEVLSRTASPFVVRCHSVLPAAAS

GDVAMLLELVDGGSLDSIVKSRSRGQAEAFSQFPEEALAEVAAQALSGLAYLHARRIVHL

DVKPGNLLVSTGGEVKIADFGIARVLPRAGGDDVRCTAYAGTAAYMSPERFDPEAHGGHY

DPYAADVWGLGVTVLELLMGRYPLLPAGQRPSWAALMCAICFGETPALSDGEASAELRGF

VAACLHKDYRRRASVAELLAHPFVAGRDVAASKCALRKLVTEASMSP*

>Adi-12MKK10-2 Brdisv1Adi-121010560m.p

MALVRQRRQLPHLTLPLDHFALRPPPVPAPAPTVAASTSSEAAGLRLSDFERISLLGQGN

GGTVYKARHRRAAAQPPVALKLFVAGDPSAAREAEILRLAADAPHVVRLHAVVPSSSPAA

GAEQPPPAALALELLPGGSLAGLLRRLGRSMGERPIAAVARQALLGLDALHALRVVHRDL

KPSNLLLGSHGEVKIADFGAGKVLRRRLDPCASYVGTAAYMSPERFDPEAYSGDYDPYAA

DVWSLGLAILELYLGHFPLLPAGQRPDWAALMCAICFGDAPEAPAAASEEFRDFVARCLE

KKAGQRASVAELLEHPFIAERDAEEAKRALAALVAEAELGDL*

>Adi-12MKK10-3 Brdisv1Adi-121001567m.p

MALLREKRLQLSLHVPTRAAEALDAVHRRPNPVAATLAASTPAAARSSQFRLADFDKLTV

LGRGNGGTVYKVRHRETCELYALKVQHCNGDPTAAAEAEVLSRTASPFIARCHSVLPGAA

SGDVAMLLELVDGGSLDSIVKSRRAHAFPFPEEALAEVAAQALSGLAYLHARRIVHLDIK

PGNLLVSTGGEVKVADFGIAKVLPRAGADDARCKSYAGTAAYMSPERFDPEAHGGHYDAY

AADVWGLGVTVLELLMGRYPLLPAGQRPSWPALMCAICFGETPVLSDGEASAELRGFVAA

CLRKDHTKRASVAELLAHPFVAGRDVATSKCALRKLVTEASTSP*

>Adi-12MKK10-4 Brdisv1Adi-121001560m.p

MASAKERRLPQLHLKLDVPPCAFRCAAPAPAPATAAPPATSASRPPHGEFRLNDFDRLSV

LGRGNGGSVYKVSHRRTSALYALKIIHGAHARPGAADEEADIVRRVVDSPNVVRCHSVLP

TASGDAAALLLELVDGGSLDSLVGGGGFLPEAAVADVAAPALSGLAHLRARRVAHRDIKP

ANLLLSAAGEVKIADFGIAKVVVSGAGGRARALAYEGTVAYMSPERFDSERHADADPYAA

DVWGLGVTLLELLMGRYPLLPAGQKPTWAALMCAICFGELPALPEGAASLEFRGFVAACL

RKDHRKRASVVELLAHPFVAGRDVAASRRALREAIERRCSC*

>Adi-12MKK10-5 Brdisv1Adi-121001564m.p

MALTVRQRRLPQLHISLDLPSCSFRCPTPPVAATASTSGEFRASDFERLPVLGRGNGGTV

YKVAHRRTSAQYALKVLHGGGDPGAAAAEADVLRRAADSPYVVRCHSVFPAASGSGETAL

LLELVDGGSLDSVRRGVGVSVFFPEAALAEVAAQALAGLAHLHARRVVHRDIKPANLLVS

GAGGVKVADFGIAMVLPSRAGGERCAAAYEGTVAYMSPERFDSEGRAGADPRGADVWGLG

VTVLELLMGRYPLLPAGQKPTWAALMCAICFGELPALPEGAASTELRGFIAACLRKDHTK

RASVAELIKHPFVAGRNMAASRLALRRLVAGA*

>Adi-2MKK1 Brdisv1Adi-21008032m.p

MRKPGKLALPSHESTIGKFLTQSGTFKDGDLLVNKDGLRIVHNSEEGEAPPIEPLDDHQL

SLDDLDAIKVIGKGSSGIVQLVRHKWTDQFFALKVIQLNIQESIRKQIAQELKISLSTQC

QYVVTCYQCFYVNGVISIVLEYMDGGSLADFLKTVRTIPEAYLAAIFKQVLQGLMYLHHE

KRVIHRDLKPSNILINHRGEVKISDFGVSAIIASSSAQRDTFTGTFNYMAPERISGQKHG

YMSDIWSLGLVMLECATGNFPYPSPDSFYELLEAVVDQPPPSAPTDQFSPEFCSFISACI

QKEATDRSSAQVLSDHPFLSMYDDLNIDLADYFTTAGSPLATFKQIVL*

>Adi-2MKK3-1 Brdisv1Adi-21029486m.p

MAGLEELKKKLQPLLFDDPDKDGISTRVPFLEDNCDSYVVSDGGTINLLSRSFGEYNINE

HGFHKRSTGADESDFGEKAYRCASHDMHIFGPIGNGASSVVQRAIFIPVHRILALKKINI

FEKEKRQQILNEMRTLCEASCYPGLVEFQGAFYMPDSGQISIALEYMDGGSLADVIKVKK

SIPEQVLAHMLQKVLLGLRYLHEVRHLVHRDIKPANMLVNLKGEAKITDFGVSAGLDNTM

AMCATFVGTVTYMSPERIRNENYSYAADIWSLGLTILECATGKFPYNVNEGPANLMLQIL

DDPSPTPPADAYSPEFCSFVNDCLQKDPDARPTCEQLFGHPFIKRYENAGVDLIAYVKGV

VDPTERLKEIAEMLAVHYYLLFNGSDGLWHHMKTFYMEESTFSFSGNVYVGRNDIFDTLS

SIRKKLKGDRPREKIVHVVEKLHCRANGETGIAIRVSGSLIVGNQFLVCGEGLQAEGMPS

VEELSIDIPSKRVGQFREQFMMLPGISMGSFHISRQDLYIIQA*

>Adi-2MKK3-2 Brdisv1Adi-21044561m.p

MAGLEELKKKLQPLMFNDPDKDGFSTRVPFPEDTCDSYVVSDGGTINLLSRSFGEYNINE

HGFHKRSAGADESDFGEKAYRCASQDMHIFGPIGNGASSVVQRAIFIPVHRILALKKINI

FEKEKRQQILNEMRTLCEACCYPGLVEFQGAFYMPDSGQISIALEYMDGGSLADVIKVKK

SIPEPVLAHMLQKVLLGLRYLHEVRHLVHRDIKPANMLVNLKGEAKITDFGVSAGLDNTM

AMCATFVGTVTYMSPERIRNENYSYAADIWSLGLTILECATGKFPYNVNEGPANLMLQIL

DDPSPAPPENAFSSEFCSFVNDCLQKDADARPTCEQLLSHPFIKRYENAGVDLAAYVKGV

VNPEERLKQIAEMLAVHYYLLFNGSDGLWHHMKTFYMEDSTFSFSGNLYVGQSDIFDTLS

NIRTKLKGDRPREKIVHVVEKLHCRANEETGIAIRVSGSFIVSNQFLICGEGLQAEGMPS

LEELSIDIPSKRVGQFREQFIMHPGRSMGCYYISRQDLYIIQA*

>Adi-2MKK3-3 Brdisv1Adi-21032560m.p

MDGGWDGMGQRKRRHRAGIPIPTYPGLAASLEFCFVTAGACHTAPGPGSKIAASLFAALL

FQRPPASTSRAIFFCFVIMAAGLEDLRRRGQPIFFDADGNVMPAPDDDSEVLDGGTINLL

SRSSDEYNINERGFHKRTIRSDDEYSSEKAFRCSCHDMHIFDSVGNGASSVVHRAIYVPV

HRVLALKKINIFEKERRQQILNEIITLSEACCYPGLVEFHGVFYTPDSGEIYFALEYMDG

GSLADIIRVKKFISEPVLSHMLQKVLLALRYLHEVRHLVHRDIKPANLLLNLKGDTKITD

FGVTSGLHDSIDMCATFLGSVTYMSPERIRNESYSYSADIWSLGLTALECATGRYPYDVN

GGEADLMLQILEDPSPTPPHDIYSEEFCSFINACLQKDADARPTCDQLLSHSFIKRYEGP

GVDLSEYNKSVHDPSERLSQIAHMLAVHYYLIFDGGDDQWCHMKTFYQQDSIFSFSGETH

VGKSEIFETLSRIRKMLKGNSPCEKIAHVMEKVYCRSHGEEGMRVRVSGSFIVGNEFVVC

ADGVRAEGMLSIDELSPDILSKQAGHFQEDFFMEPGTALGCYVISKQELHIADT*

>Adi-2MKK4 Brdisv1Adi-21006537m.p

MRPGGPPNARPQQPGTPGRARRRPDLTLPLPQRDLTSLAVPLPLPPPPSSAPSSASSSGS

SLSSMGAPTPPNSAGSAPPPPPPLAELERVRRIGSGAGGTVWMVRHRPTGRPYALKVLYG

NHDDAVRRQITREIAILRTAEHPAIVRCHGMYEQAGELQILLEFMDGGSLEGRRIASEAF

LADVARQVLSGIAYLHRRHIVHRDIKPSNLLIDSGRRVKIADFGVGRILNQTMDPCNSSV

GTIAYMSPERINTDLNDGAYDGYAGDIWSFGLSILEFYLGRFPLGENLGKQGDWAALMCA

ICYSDSPAPPPIASPEFKSFISCCLQKNPARRPSAAQLLQHRFIAGPQPQVLAAPPS*

>Adi-2MKK5 Brdisv1Adi-21007328m.p

MRPAGSLPSPQPGTPGRPRRRPDLTLPMPQRPDVSSSLAVPLPLPPPSSLGLAQPPAAAA

AAAAPPPPPLGELERVRRVGSGAGGTVWMVRHRPTGRCYALKQLYGNHDDAVRRQIAREI

AILRTAEHPAVVRCHGMYERGGELQILLEYMDGGSLDGRRIAAEGFLADVARQVLSGIAY

LHRRHIVHRDIKPSNLLIDSARRVKIADFGVGRILNQTMDPCNSSVGTIAYMSPERINTD

LNDGAYDGYAGDIWSFGLSILEFYLGRFPFGENLGKQGDWAALMVAICYNDPPEPSAAAS

PEFRGFISCCLQKNPAKRLSAAQLLQHPFVAGPQPLPLAAPPS*

>Adi-2MKK6 Brdisv1Adi-21012376m.p

MRGKKPLKELKLSVPAQETSVDKFLTASGTFKDGELRLNQRGLRLISEEENGDEHQSTNM

KVEDVQLSMDDLEMIQVIGKGSGGVVQLVQHKWVGTFYALKGIQMNIQEAVRKQIVQELK

INQATQSPHIVSCHQSFYHNGVIYLVLEYMDRGSLADIIKQVKTILEPYLAVLCKQVLEG

LLYLHHERHVIHRDIKPSNLLVNHKGEVKITDFGVSAVLASSIGQRDTFVGTYNYMAPER

ISGSSYDYKSDVWSLGLVILECAIGRFPYTPSEGEGWLSFYELLEAIVDQPPPGAPADQF

SPEFCSFISACIQKDPAERMSASELLNHAFIKKFEGKDLDLRILVESLEPPMNVPE*

>Adi-2MKK10-1 Brdisv1Adi-21001745m.p

MALLREKRLQLSLHVPTRAADAQEAGLHRRPNPAAALPLAATPPAARSSQFRVADFEKLA

VLGRGNGGTVYKVRHRETCELYALKVQHCNGDATAEAEVLSRPASPFVVRCHSVLPAAAS

GDVAMLLELVDGGSLDSIVKSRSRGQAEAFSQFPEEALAEVAAQALSGLAYLHARRIVHL

DVKPGNLLVSTGGEVKIADFGIARVLPRAGGDDVRCTAYAGTAAYMSPERFDPEAHGGHY

DPYAADVWGLGVTVLELLMGRYPLLPAGQRPSWAALMCAICFGETPALSDGEASAELRGF

VAACLHKDYRRRASVAELLAHPFVAGRDVAASKCALRKLVTEASMSP*

>Adi-2MKK10-2 Brdisv1Adi-21011441m.p

MALVRQRRQLPHLTLPLDHFALRPPPVPAPAPTVAASTSSEAAGLRLSDFERISLLGQGN

GGTVYKARHRRAAAQPPVALKLFVAGDPSAAREAEILRLAADAPHVVRLHAVVPSSSPAA

GAEQPPPAALALELLPGGSLAGLLRRLGRSMGERPIAAVARQALLGLDALHALRVVHRDL

KPSNLLLGSHGEVKIADFGAGKVLRRRLDPCASYVGTAAYMSPERFDPEAYSGDYDPYAA

DVWSLGLAILELYLGHFPLLPAGQRPDWAALMCAICFGDAPEAPAAASEEFRDFVARCLE

KKAGQRASVAELLEHPFIAERDAEEAKRALAALVAEAELGDL*

>Adi-2MKK10-3 Brdisv1Adi-21001643m.p

MALLREKRLQLSLHVPTRAAEALDAVHRRPTPVAATLAASTPAAARSSQFRLADFDKLTV

LGRGNGGTVYKVRHRETCELYALKVQHCNGDPTAAAEAEVLSRTASPFIVRCHSVLPGAA

SGDVAMLLELVDGGSLDSIVKSRRAHAFPFPEEALAEVAAQALSGLAYLHARRIVHLDIK

PGNLLVSTGGEVKVADFGIAKVLPRAGADDARCKSYAGTAAYMSPERFDPEAHGGHYDAY

AADVWGLGVTVLELLMGRYPLLPAGQRPSWPALMCAICFGETPVLSDGEASAELRGFVAA

CLRKDHTKRASVAELLAHPFVAGRDVATSKCALRKLVTEASTSP*

>Adi-2MKK10-4 Brdisv1Adi-21001640m.p

MASAKERRLPQLHLKLDVPPCAFRCAAPAPAPATAATPATSASRPPHGEFRLNDFDRLSV

LGRGNGGSVYKVSHRRTSALYALKIIHGAHARPGAADEEADIVRRVVDSPNVVRCHSVLP

TASGDAAALLLELVDGGSLDSLVGGGGFLPEAAVADVAAQALSGLAHLRARRVAHRDIKP

ANLLLSAAGEVKIADFGIAKVVVSGAGGRARALAYEGTVAYMSPERFDSERHADADPYAA

DVWGLGVTLLELLMGRYPLLPAGQKPTWAALMCAICFGELPALPEGAASLEFRGFVAACL

RKDHRKRASVVELLAHPFVAGRDVAASRRALREAIERRCSC*

>Adi-2MKK10-5 Brdisv1Adi-21001642m.p

MALTVRQRRLPQLHISLDLPSCSFRCPNPPVAATASTSGEFRASDFERLAVLGRGNGGTV

YKVAHRRTSAQYALKVLHGGGDPGAAAAEADVLRRAADSPYVVRCHSVFPAASGSGETAL

LLELVDGGSLDSVRRGVGGSVFFPEAALAEVAAQALAGLAHLHARRVVHRDIKPANLLVS

GAGGVKVADFGIAMVLPSRAGGERCAAAYEGTVAYMSPERFDSEGRADADPRGADVWGLG

VTVLELLMGRYPLLPAGQKPTWAALMCAICFGELPALPEGAASTELRGFIAACLRKDHTK

RASVAELIKHPFVAGRNMAASRLALRRLVAGA*

>Bd18-1MKK1 Brdisv1Bd18-11008720m.p

MRKPGKLALPSHESTIGKFLTQSGTFKDGDLLVNKDGLRIVHNSEEGEAPPIEPLDDHQL

SLDDLDAIKVIGKGSSGIVQLVRHKWTDQFFALKVIQLNIQESIRKQIAQELKISLSTQC

QYVVTCYQCFYVNGVISIVLEYMDGGSLADFLKTVRTIPEAYLAAICKQVLQGLMYLHHE

KRVIHRDLKPSNILINHRGEVKISDFGVSAIIASSSAQRDTFTGTFNYMAPERISGQKHG

YMSDIWSLGLVMLECATGNFPYPSPDSFYELLEAVVDQPPPSAPTDQFSPEFCSFISACI

QKEATDRSSAQVLSDHPFLSMYDDLNIDLADYFTTAGSPLATFKQIVL*

>Bd18-1MKK3-1 Brdisv1Bd18-11041331m.p

MAGLEELKKKLQPLLFDDPDKDGISTRVPFLEDNCDSYVVSDGGTINLLSRSFGEYNINE

HGFHKRSTGADESDFGEKAYRCASHDMHIFGPIGNGASSVVQRAIFIPVHRILALKKINI

FEKEKRQQILNEMRTLCEASCYPGLVEFQGAFYMPDSGQISIALEYMDGGSLADVIKVKK

SIPEQVLAHMLQKVLLGLRYLHEVRHLVHRDIKPANMLVNLKGEAKITDFGVSAGLDNTM

AMCATFVGTVTYMSPERIRNENYSYAADIWSLGLTILECATGKFPYNVNEGPANLMLQIL

DDPSPTPPADAYSPEFCSFVNDCLQKDPDARPTCEQLFGHPFIKRYENAGVDLIAYVKGV

VDPTERLKEIAEMLAVHYYLLFNGSDGLWHHMKTFYMEESTFSFSGNVYVGRNDIFDTLS

SIRKKLKGDRPREKIVHVVEKLHCRANGETGIAIRVSGSLIVGNQFLVCGEGLQAEGMPS

VEELSIDIPSKRVGQFREQFMMLPGISMGSFHISRQDLYIIQA*

>Bd18-1MKK3-2 Brdisv1Bd18-11006956m.p

MAGLEELKKKLQPLMFNDPDKDGFSTRVPFPEDTCDSYVVSDGGTINLLSRSFGEYNINE

HGFHKRSAGADESDFGEKAYRCASQDMHIFGPIGNGASSVVQRAIFIPVHRILALKKINI

FEKEKRQQILNEMRTLCEACCYPGLVEFQGAFYMPDSGQISIALEYMDGGSLADVIKVKK

SIPEPVLAHMLQKVLLGLRYLHEVRHLVHRDIKPANMLVNLKGEAKITDFGVSAGLDNTM

AMCATFVGTVTYMSPERIRNENYSYAADIWSLGLTILECATGKFPYNVNEGPANLMLQIL

DDPSPAPPENAFSSEFCSFVNDCLQKDADARPTCEQLLSHPFIKRYENAGVDLAAYVKGV

VNPEERLKQIAEMLAVHYYLLFNGSDGLWHHMKTFYMEDSTFSFSGNLYVGQSDIFDTLS

NIRTKLKGDRPREKIVHVVEKLHCRANEETGIAIRVSGSFIVSNQFLICGEGLQAEGMPS

LEELSIDIPSKRVGQFREQFIMHPGRSMGCYYISRQDLYIIQA*

>Bd18-1MKK3-3 Brdisv1Bd18-11026234m.p

MDGGWDGMGQRKRRHRAGIPIPTYPGLAASLEFCFVTAGACHTAPGPGSKIAASLFAALL

FQRPPASTSRAIFFCFVIMAAGLEDLRRRVQPIFFDADGNVMPAPDDDSEVLDGGTINLL

SRSSDEYNINERGFHKRTIRSDDEYSSEKAFRCSCHDMHIFDSVGNGASSVVHRAIYVPV

HRVLALKKINIFEKERRQQILNEIITLSEACCYPGLVEFHGVFYTPDSGEIYFALEYMDG

GSLADIIRVKKFISEPVLSHMLQKVLLALRYLHEVRHLVHRDIKPANLLLNLKGDTKITD

FGVTSGLHDSIDMCATFLGSVTYMSPERIRNESYSYSADIWSLGLTALECATGRYPYDVN

GGEADLMLQILEDPSPTPPHDIYSEEFCSFINACLQKDADARPTCDQLLSHSFIKRYEGP

GVDLSEYNKSVHDPSERLSQIAHMLAVHYYLIFDGGDDQWCHMKTFYQQDSIFSFSGETH

VGKSEIFETLSRIRKMLKGNSPCEKIAHVMEKVYCRSHGEEGMRVRVSGSFIVGNEFVVC

ADGVRAEGMLSIDELSPDILSKQAGHFQEDFFMEPGTALGCYVISKQELHIADT*

>Bd18-1MKK4 Brdisv1Bd18-11033291m.p

MRPGGPPNARPQQPGTPGRARRRPDLTLPLPQRDLTSLAVPLPLPPPPSSAPSSASSSGS

SLSSMGAPTPPNSAGSAPPPPPPLAELERVRRIGSGAGGTVWMVRHRPTGRPYALKVLYG

NHDDAVRRQITREIAILRTAEHPAIVRCHGMYEQAGELQILLEFMDGGSLEGRRIASEAF

LADVARQVLSGIAYLHRRHIVHRDIKPSNLLIDSGRRVKIADFGVGRILNQTMDPCNSSV

GTIAYMSPERINTDLNDGAYDGYAGDIWSFGLSILEFYLGRFPLGENLGKQGDWAALMCA

ICYSDSPAPPPIASPEFKSFISCCLQKNPARRPSAAQLLQHRFIAGPQPQVLAAPPS*

>Bd18-1MKK5 Brdisv1Bd18-11007870m.p

MRPAGSLPSPQPGTPGRPRRRPDLTLPMPQRPDVSSSLAVPLPLPPPSSLGLAQPPAAAA

AAAAPPPPPLGELERVRRVGSGAGGTVWMVRHRPTGRCYALKQLYGNHDDAVRRQIAREI

AILRTAEHPAVVRCHGMYERGGELQILLEYMDGGSLDGRRIAAEGFLADVARQVLSGIAY

LHRRHIVHRDIKPSNLLIDSARRVKIADFGVGRILNQTMDPCNSSVGTIAYMSPERINTD

LNDGAYDGYAGDIWSFGLSILEFYLGRFPFGENLGKQGDWAALMVAICYNDPPEPSAAAS

PEFRGFISCCLQKNPAKRLSAAQLLQHPFVAGPQPLPLAAPPS*

>Bd18-1MKK6 Brdisv1Bd18-11012730m.p

MRGKKPLKELKLSVPAQETSVDKFLTASGTFKDGELRLNQRGLRLISEEENGDEHQSTNM

KVEDVQLSMDDLEMIQVIGKGSGGVVQLVQHKWVGTFYALKGIQMNIQEAVRKQIVQELK

INQATQSPHIVSCHQSFYHNGVIYLVLEYMDRGSLADIIKQVKTILEPYLAVLCKQVLEG

LLYLHHERHVIHRDIKPSNLLVNHKGEVKITDFGVSAVLASSIGQRDTFVGTYNYMAPER

ISGSSYDYKSDVWSLGLVILECAIGRFPYTPSEGEGWLSFYELLEAIVDQPPPGAPADQF

SPEFCSFISACIQKDPAERMSASELLNHAFIKKFEGKDLDLRILVESLEPPMNVPE*

>Bd18-1MKK10-1 Brdisv1Bd18-11001858m.p

MALLREKRLQLSLHVPTRAADAQEAGLHRRPNPAAALPLAATTPAARSSQFRVADFEKLA

VLGRGNGGTVYKVRHRETCELYALKVQHCNGDATAEAEVLSRTASPFVVRCHSVLPAAAS

GDVAMLLELVDGGSLDSIVKSRSRGQAEAFSQFPEEALAEVAAQALSGLAYLHARRIVHL

DVKPGNLLVSTGGEVKIADFGIARVLPRAGGDDVRCTAYAGTAAYMSPERFDPEAHGGHY

DPYAADVWGLGVTVLELLMGRYPLLPAGQRPSWAALMCAICFGETPALSDGEASAELRGF

VAACLHKDYRRRASVAELLAHPFVAGRDVAASKCALRKLVTEASMSP*

>Bd18-1MKK10-2 Brdisv1Bd18-11011845m.p

MALVRQRRQLPHLTLPLDHFALRPPPVPAPAPTVAASTSSEAAGLRLSDFERISLLGQGN

GGTVYKARHRRAAAQPPVALKLFVAGDPSAAREAEILRLAADAPHVVRLHAVVPSSSPAA

GAEQPPPAALALELLPGGSLAGLLRRLGRSMGERPIAAVARQALLGLDALHALRVVHRDL

KPSNLLLGSHGEVKIADFGAGKVLRRRLDPCASYVGTAAYMSPERFDPEAYSGDYDPYAA

DVWSLGLAILELYLGHFPLLPAGQRPDWAALMCAICFGDAPEAPAAASEEFRDFVARCLE

KKAGQRASVAELLEHPFIAERDAEEAKRALAALVAEAELGDL*

>Bd18-1MKK10-3 Brdisv1Bd18-11001742m.p

MALLREKRLQLSLHVPTRAAEALDAVHRRPNPVAATLAASTPAAARSSQFRLADFDKLTV

LGRGNGGTVYKVRHRETCELYALKVQHCNGDPTAAAEAEVLSRTASPFIVRCHSVLPGAA

SGDVAMLLELVDGGSLDSIVKSRRAHAFPFPEEALAEVAAQALSGLAYLHARRIVHLDIK

PGNLLVSTGGEVKVADFGIAKVLPRAGADDARCKSYAGTAAYMSPERFDPEAHGGHYDAY

AADVWGLGVTVLELLMGRYPLLPAGQRPSWPALMCAICFGETPVLSDGEASAELRGFVAA

CLRKDHTKRASVAELLAHPFVAGRDVATSKCALRKLVTEASTSP*

>Bd18-1MKK10-4 Brdisv1Bd18-11001738m.p

MASAKERRLPQLHLKLDVPPCAFRCAAPAPAPATAATPATSASRPPHGEFRLNDFDRLSV

LGRGNGGSVYKVSHRRTSALYALKIIHGAHARPGAADEEADIVRRVVDSPNVVRCHSVLP

TASGDAAALLLELVDGGSLDSLVGGGGFLPEAAVADVAAQALSGLAHLRARRVAHRDIKP

ANLLLSAAGEVKIADFGIAKVVVSGAGGRARALAYEGTVAYMSPERFDSERHADADPYAA

DVWGLGVTLLELLMGRYPLLPAGQKPTWAALMCAICFGELPALPEGAASLEFRGFVAACL

RKDHRKRASVVELLAHPFVAGRDVAASRRALREAIERRCSC*

>Bd18-1MKK10-5 Brdisv1Bd18-11001740m.p

MALTVRQRRLPQLHISLDLPSCSFRCPNPPVAATASTSGEFRASDFERLAVLGRGDGGTV

YKGAHRRTSAQYALKVLHGGGDPGAAAAEADVLRRAADSPYVVRCHSVFPAASGSGETAL

LLELVDGGSLDSVRRGVGVSVFFPEAALAEVAAQALAGLAHLHARRVVHRDIKPANLLVS

GAGGVKVADFGIAMVLPSRAGGERCAAAYEGTVAYMSPERFDSEGRADADPRGADVWGLG

VTVLELLMGRYPLLPAGQKPTWAALMCAICFGELPALPEGAASTELRGFIAACLRKDHTK

RASVAELIKHPFVAGRNMAASRLALRRLVAGA*

>Bd2-3MKK1 Brdisv1Bd2-31007550m.p

MRKPGKLALPSHESTIGKFLTQSGTFKDGDLLVNKDGLRIVHNSEEGEAPPIEPLDDHQL

SLDDLDAIKVIGKGSSGIVQLVRHKWTDQFFALKVIQLNIQESIRKQIAQELKISLSTQC

QYVVTCYQCFYVNGVISIVLEYMDGGSLADFLKTVRTIPEAYLAAIFKQVLQGLMYLHHE

KRVIHRDLKPSNILINHRGEVKISDFGVSAIIASSSAQRDTFTGTFNYMAPERISGQKHG

YMSDIWSLGLVMLECATGNFPYPSPDSFYELLEAVVDQPPPSAPTDQFSPEFCSFISACI

QKEATDRSSAQVLSDHPFLSMYDDLNIDLADYFTTAGSPLATFKQIVL*

>Bd2-3MKK3-1 Brdisv1Bd2-31026929m.p

MAGLEELKKKLQPLLFDDPDKDGISTRVPFLEDNCDSYVVSDGGTINLLSRSFGEYNINE

HGFHKRSTGADESDFGEKAYRCASHDMHIFGPIGNGASSVVQRAIFIPVHRILALKKINI

FEKEKRQQILNEMRTLCEASCYPGLVEFQGAFYMPDSGQISIALEYMDGGSLADVIKVKK

SIPEQVLAHMLQKVLLGLRYLHEVRHLVHRDIKPANMLVNLKGEAKITDFGVSAGLDNTM

AMCATFVGTVTYMSPERIRNENYSYAADIWSLGLTILECATGKFPYNVNEGPANLMLQIL

DDPSPTPPADAYSPEFCSFVNDCLQKDPDARPTCEQLFGHPFIKRYENAGVDLIAYVKGV

VDPTERLKEIAEMLAVHYYLLFNGSDGLWHHMKTFYMEESTFSFSGNVYVGRNDIFDTLS

SIRKKLKGDRPREKIVHVVEKLHCRANGETGIAIRVSGSLIVGNQFLVCGEGLQAEGMPS

VEELSIDIPSKRVGQFREQFMMLPGISMGSFHISRQDLYIIQA*

>Bd2-3MKK3-2 Brdisv1Bd2-31040703m.p

MAGLEELKKKLQPLMFNDPDKDGFSTRVPFPEDTCDSYVVSDGGTINLLSRSFGEYNINE

HGFHKRSAGADESDFGEKAYRCASQDMHIFGPIGNGASSVVQRAIFIPVHRILALKKINI

FEKEKRQQILNEMRTLCEACCYPGLVEFQGAFYMPDSGQISIALEYMDGGSLADVIKVKK

SIPEPVLAHMLQKVLLGLRYLHEVRHLVHRDIKPANMLVNLKGEAKITDFGVSAGLDNTM

AMCATFVGTVTYMSPERIRNENYSYAADIWSLGLTILECATGKFPYNVNEGPANLMLQIL

DDPSPAPPENAFSSEFCSFVNDCLQKDADARPTCEQLLSHPFIKRYENAGVDLAAYVKGV

VNPEERLKQIAEMLAVHYYLLFNGSDGLWHHMKTFYMEDSTFSFSGNLYVGQSDIFDTLS

NIRTKLKGDRPREKIVHVVEKLHCRANEETGIAIRVSGSFIVSNQFLICGEGLQAEGMPS

LEELSIDIPSKRVGQFREQFIMHPGRSMGCYYISRQDLYIIQA*

>Bd2-3MKK3-3 Brdisv1Bd2-31029786m.p

MDGGWDGMGQRKRRHRAGIPIPTYPGLAASLEFCFVTAGACHTAPGPGSKIAASLFAALL

FQRPPASTSRAIFFCFVIMAAGLEDLRRRGQPIFFDADGNVMPAPDDDSEVLDGGTINLL

SRSSDEYNINERGFHKRTIRSDDEYSSEKAFRCSCHDMHIFDSVGNGASSVVHRAIYVPV

HRVLALKKINIFEKERRQQILNEIITLSEACCYPGLVEFHGVFYTPDSGEIYFALEYMDG

GSLADIIRVKKFISEPVLSHMLQKVLLALRYLHEVRHLVHRDIKPANLLLNLKGDTKITD

FGVTSGLHDSIDMCATFLGSVTYMSPERIRNESYSYSADIWSLGLTALECATGRYPYDVN

GGEADLMLQILEDPSPTPPHDIYSEEFCSFINACLQKDADARPTCDQLLSHSFIKRYEGP

GVDLSEYNKSVHDPSERLSQIAHMLAVHYYLIFDGGDDQWCHMKTFYQQDSIFSFSGETH

VGKSEIFETLSRIRKMLKGNSPCEKIAHVMEKVYCRSHGEEGMRVRVSGSFIVGNEFVVC

ADGVRAEGMLSIDELSPDILSKQAGHFQEDFFMEPGTALGCYVISKQELHIADT*

>Bd2-3MKK4 Brdisv1Bd2-31001621m.p

MGAPTPPNSAGSAPPPPPPLAELERVRRIGSGAGGTVWMGRHRPTGRPYALKVLYGNHDD

AVRRQITREIAILRTAEPPAIVRCHGMYEQAGELQILLEFMDGGSLEGRRIASEAFLADV

ARQVLSGIAYLHRRHIVHRDIKPSNLLIDSGRRVKIADFGVGRILNQTMDPCNSSVGTIA

YMSPERINTDLNDGAYDGYAGDIWSFGLSILEFYLGRFPLGENLGKQGDWAALMCAICYS

DSPAPPPIASPEFKSFISCCLQKNPARRPSAAQLLQPRFIAGPQPQVLAAPPS*

>Bd2-3MKK5 Brdisv1Bd2-31001622m.p

MRPAGSLPSPQPGTPGRPRRRPDLPLPMPQRPDVSSSLAVPLPLPPPSSLGLAQPPAAAL

ERVRRVGSGAGGTVWMVRHRPTGRCYALKQLYGNHDDAVRRQIAREIAILRTAEHPAVVR

CHGMYERGGELQILLEYMDGGPPAGRRIAAEGFLADVARQVLSGIASPPRRHIVHRDIKP

SNLLIDSARRVKIADFGVGRILNQTMDPCNSSVGTIAYMSPERINTDLNDGAYDGYAGDI

WSFGLSILEFYLGRFPFGENLGKQGDWAALMVPICYNDPPEPSAAASPEFRGFISCCLQK

NPAKRLSAAQLLQHPFVAGPQPLPLAAPPS*

>Bd2-3MKK6 Brdisv1Bd2-31011337m.p

MGWPVLGIQMNIQEAVRKQIVQELKINQATQSPHIVSCHQSFYHNGVIYLVLEYMDRGSL

ADIIKQVKTILEPYLAVLCKQVLEGLLYLHHERHVIHRDIKPSNLLVNHKGEVKITDFGV

SAVLASSIGQRDTFVGTYNYMAPERISGSSYDYKSDVWSLGLVILECAIGRFPYTPSEGE

GWLSFYELLEAIVDQPPPGAPADQFSPEFCSFISACIQKDPAERMSASELLNHAFIKKFE

GKDLDLRILVESLEPPMNVPE*

>Bd2-3MKK10-1 Brdisv1Bd2-31001731m.p

MALLREKRLQLSLHVPTRAADAQEAGLHRRPNPAAALPLAATTPAARWSQFRVADFEKLA

VLGRGNGGTVYKVRHRETCELYALKVQHCNGDATAEAEVLSRTASPFVARCHSVLPAAAS

GDVAMLLELVDGGALDSIVKSRSRGQAEAFSQFPEEALAEVAAQALSGLAYLHARRIVHL

DVKPGNLLVSTGGEVKIADFGIARVLPRAGGDDVRCTAYAGTAAYMSPERFDPEAHGGHY

DPYAADVWGLGVTVLELLMGRYPLLPAGQRPSWAALMCAICFGETPALSDGEASAELRGF

VAACLHKDYRRRASVAELLAHPFVAGRDVAASKCALRKLVTEASMSP*

>Bd2-3MKK10-2 Brdisv1Bd2-31010489m.p

MALVRQRRQLPHLPLPLDHFALRPPPAPAPTVAASTSSEAAGLRLSDFERISLLGQGNGG

TVYKGRHRRAAAQPPVALKLFVAGDPSAAREAEILRLAADAPHVVRLHAVVPSSSPAAGA

EQPPPAALALELLPGGSLAGLLRRLGRSMGGRPIAAVARQALLGLDALHALRVVHRDLKP

SNLLLGSHGEVKIADFGAGKVLRRRLDPCASYVGTAAYMSPERFDPEAYSGDYDPYAADV

WSLGLAILELYLGHFPLLPAGQRPDWAALMCAICFGDAPEAPAAASEEFRDFVARCLEKK

AGQRASVAELLEHPFIAERDAEEAKRALAALVAEAELGDL*

>Bd2-3MKK10-3 Brdisv1Bd2-31001625m.p

MALLREKRLQLSLHVPTRAAEALDAVHRRPNPVAATLAASTPAAARQFRLADFDKLTVLG

RGNGGTVYKVRHRETCELYALKVQHCNGDPTAAAEAEVLSRPASPFIARCHSVLPGAASG

DVAMLLELVDGGSLDSIVKSRRAHAFPFPEEALAEVAAQALSGLAYLHARRIVHLDIKPG

NLLVSTGGEVKVADFGIAKVLPRAGADDARCKSYAGTAAYMSPERFDPEAHGGHYDAYAA

DVWGLGVTVLELLMGRYPLLPAGQRPSWPALMCAICFGETPVLSDGEASAELRGFVAACL

RKDHTKRASVAELLAHPFVAGRDVATSKCALRKLVTEASTSP*

>Bd2-3MKK10-4 Brdisv1Bd2-31001617m.p

MASAKERRLPQLHLKLDVPTCAFRCAAPAPAPATAATPATSASRPPHGEFRLNDFDRLSV

LGRGNGGSVYKVSHRRTSALYALKIIHGAHARPGAADEEADIVRRVVDSPNVVPCHSVLP

TASGDAAALLLELVDGGSLDSLVGGGGFLPEAAGADVAAQALSGLAPLRARRVAHRDIKP

ANLLLSAAGEVKIADFGIAKVVVSGAGGRARALAYEGTVAYMSPERFDSERHADADPYAA

DVWGLGVTLLELLMGRYPLLPAGQKPTWAALMCAICFGELPALPEGAASLEFRGFVAACL

RKDHRKRASVVELLAHPFVAGRDVAASRRALREAIERRGT*

>Bd2-3MKK10-5 Brdisv1Bd2-31001619m.p

MALTVRQRRLPQLHISLAPPSCSFGCPTPPVAATASTSGEFRASDFERLAVLGRGNGGTV

YKVAHRRTSAQYALKVLHGGGDPGAAAAEAGGLRRAADSPYVVRCHSVFPAASGSGETAL

LLELVDGGSPDSVRRGVGGSVFFPEAALAEVAAQALAGLAHLHARRVVHRDIKPANLLVS

GAGGVKVADFGIAMVLPSRAGGERCAAAYEGTVAYMSPERFDSEGRADADPRGADVWGLG

VTVLELLMGRYPLLPAGQKPTWAALMCAICFGELPALPEGAASTELRGFIAACLRKDHTK

RASVAELIKHPFVAGRNMAASRLALRRLVAGA*

>Bd21-3MKK1 Brdisv1Bd21-3_r1008133m.p

MRKPGKLALPSHESTIGKFLTQSGTFKDGDLLVNKDGLRIVHNSEEGEAPPIEPLDDHQL

SLDDLDAIKVIGKGSSGIVQLVRHKWTDQFFALKVIQLNIQESIRKQIAQELKISLSTQC

QYVVTCYQCFYVNGVISIVLEYMDGGSLADFLKTVRTIPEAYLAAIFKQVLQGLMYLHHE

KRVIHRDLKPSNILINHRGEVKISDFGVSAIIASSSAQRDTFTGTFNYMAPERISGQKHG

YMSDIWSLGLVMLECATGNFPYPSPDSFYELLEAVVDQPPPSAPTDQFSPEFCSFISACI

QKEATDRSSAQVLSDHPFLSMYDDLNIDLADYFTTAGSPLATFKQIVL*

>Bd21-3MKK3-1 Brdisv1Bd21-3_r1038149m.p

MAGLEGVKKKLQPLLFDDPDKDGISTRVPFLEDNCDSYVVSDGGTINLLSRSFGEYNINE

HGFHKRSTGADESDFGEKAYRCASHDMHIFGPIGNGASSVVQRAIFIPVHRILALKKINI

FEKEKRQQILNEMRTLCEASCYPGLVEFQGAFYMPDSGQISIALEYMDGGSLADVIKVKK

SIPEQVLAHMLQKVLLGLRYLHEVRHLVHRDIKPANMLVNLKGEAKITDFGVSAGLDNTM

AMCATFVGTVTYMSPERIRNENYSYAADIWSLGLTILECATGKFPYNVNEGPANLMLQIL

DDPSPTPPADAYSPEFCSFVNDCLQKDPDARPTCEQLFGHPFIKRYENAGVDLIAYVKGV

VDPTERLKEIAEMLAVHYYLLFNGSDGLWHHMKTFYMEESTFSFSGNVYVGRNDIFDTLS

SIRKKLKGDRPREKIVHVVEKLHCRANGETGIAIRVSGSLIVGNQFLVCGEGLQAEGMPS

VEELSIDIPSKRVGQFREQFMMLPGISMGSFHISRQDLYIIQA*

>Bd21-3MKK3-2 Brdisv1Bd21-3_r1006624m.p

MAGLEELKKKLQPLMFNDPDKDGFSTRVPFPEDTCDSYVVSDGGTINLLSRSFGEYNINE

HGFHKRSAGADESDFGEKAYRCASQDMHIFGPIGNGASSVVQRAIFIPVHRILALKKINI

FEKEKRQQILNEMRTLCEACCYPGLVEFQGAFYMPDSGQISIALEYMDGGSLADVIKGKK

SIPEPVLAHMLQKVLLGLRYLHEVRHLVHRDIKPANMLVNLKGEAKITDFGVSAGLDNTM

AMCATFVGTVTYMSPERIRNENYSYAADIWSLGLTILECATGKFPYNENEGPANLMLQIL

DDPSPAPPENAFSSEFCSFVNDCLQKDADARPTCEQLLSHPFIKRYENAGVDLAAYVKGV

VNPEERLKQIAEMLAVHYYLLFNGSDGLWHHMKTFYMEDSTFSFSGNLYVGQSDIFDTLS

NIRTKLKGDRPREKIVHVVEKLHCRANEETGIAIRVSGSFIVSNQFLICGEGLQAEGMPS

LEELSIDIPSKRVGQFREQFIMHPGRSMGCYYISRQDLYIIQA*

>Bd21-3MKK3-3 Brdisv1Bd21-3_r1024469m.p

MDGGWDGMGQRKRRHRAGIPIPTYPGLAASLEFCFVTAGACHTAPGPGSKIAASLFAALL

FQRPPASTSRAIFFCFVIMAAGLEDLRRRVQPIFFDADGNVMPAPDDDSEVLDGGTINLL

SRSSDEYNINERGFHKRTIRSDDEYSSEKAFRCSCHDMHIFDSVGNGASSVVHRAIYVPV

HRVLALKKINIFEKERRQQILNEIITLSEACCYPGLVEFHGVFYTPDSGEIYFALEYMDG

GSLADIIRVKKFISEPVLSHMLQKVLLALRYLHEVRHLVHRDIKPANLLLNLKGDTKITD

FGVTSGLHDSIDMCATFLGSVTYMSPERIRNESYSYSADIWSLGLTALECATGRYPYDVN

GGEADLMLQILEDPSPTPPHDIYSEEFCSFINACLQKDADARPTCDQLLSHSFIKRYEGP

GVDLSEYNKSVHDPSERLSQIAHMLAVHYYLIFDGGDDQWCHMKTFYQQDSIFSFSGETH

VGKSEIFETLSRIRKMLKGNSPCEKIAHVMEKVYCRSHGEEGMRVRVSGSFIVGNEFVVC

ADGVRAEGMLSIDELSPDILSKQAGHFQEDFFMEPGTALGCYVISKQELHIADT*

>Bd21-3MKK4 Brdisv1Bd21-3_r1030936m.p

MRPGGPPNARPQQPGTPGRARRRPDLTLPLPQRDLTSLAVPLPLPPPPSSAPSSASSSGS

SLSSMGAPTPPNSAGSAPPPPPPLAELERVRRIGSGAGGTVWMVRHRPTGRPYALKVLYG

NHDDAVRRQITREIAILRTAEHPAIVRCHGMYEQAGELQILLEFMDGGSLEGRRIASEAF

LADVARQVLSGIAYPHRRHIVHRDIKPSNLLIDSGRRVKIADFGVGRILNQTMDPCNSSV

GTIAYMSPERINTDLNDGAYDGYAGDIWSFGLSILEFYLGRFPLGENLGKQGDWAALMCA

ICSSDSPAPPPIASPEFKSFISCCLQKNPARRPSAAQLLQHRFIAGPQPQVLAAPPS*

>Bd21-3MKK5 Brdisv1Bd21-3_r1007409m.p

MRPAGSLPSPQPGTPGRPRRRPDLTLPMPQRPDVSSSLAVPLPLPPPSSLGLAQPPAAAA

AAAAPPPPPLGELERVRRVGSGAGGTVWMVRHRPTGRCYALKQLYGNHDDAVRRQIAREI

AILRTAEHPAVVRCHGMYERGGELQILLEYMDGGSLDGRRIAAEGFLADVARQVLSGIAY

LHRRHIVHRDIKPSNLLIDSARRVKIADFGVGRILNQTMDPCNSSVGTIAYMSPERINTD

LNDGAYDGYAGDIWSFGLSILEFYLGRFPFGENLGKQGDWAALMVAICYNDPPEPSAAAS

PEFRGFISCCLQKNPAKRLSSAPLLPHPFVAGPQPLPLAAPPS*

>Bd21-3MKK6 Brdisv1Bd21-3_r1012124m.p

MRGKKPLKELKLSVPAQETSVDKFLTASGTFKDGELRLNQRGLRLISEEENGDEHQSTNM

KVEDVQLSMDDLEMIQVIGKGSGGVVQLVQHKWVGTFYALKGIQMNIQEAVRKQIVQELK

INQATQSPHIVSCHQSFYHNGVIYLVLEYMDRGSLADIIKQVKTIREPYLAVLCKQVLEG

LLYLHHERHVIHRDIKPSNLLVNHKGEVKITDFGVSAVLASSIGQRDTFVGTYNYMAPER

ISGSSYDYKSDVWSLGLVILECAIGRFPYTPSEGEGWLSFYELLEAIVDQPPPGAPADQF

SPEFCSFISACIQKDPAERMSASELLNHAFIKKFEGKDLALRILVESLEPPMNVPE*

>Bd21-3MKK10-1 Brdisv1Bd21-3_r1001769m.p

MALLREKRLQLSLHAPTRAADAQEAGLHRRPNPAAALPLAATTPAARSSQFRVADFEKLA

VLGRGNGGTVYKVRHRETCELYALKVQHCNGDATAEAEVLSRTASPFVVRCHSVLPAAAS

GDVAMLLELVDGGSLDSIVKSRSRGQAEAFSQFPEEALAEVAAQALSGLAYLHARRIVHL

DVKPGNLLVSTGGEVKIADFGIARVLPRAGGDDVRCTAYAGTAAYMSPERFDPEAHGGHY

DPYAADVWGLGVTVLELLMGRYPLLPAGQRPSWAALMCAICFGETPALSDGEASAELRGF

VAACLHKDYRRRASVAELLAHPFVAGRDVAASKCALRKRVTEASMSP*

>Bd21-3MKK10-2 Brdisv1Bd21-3_r1011270m.p

MALVRQRRQLPHLTLPLDHFALRPPPAPAPTVAASTSSEAAGLRLSDFERISLLGQGNGG

TVYKARHRRAAAQPPVALKLFVAGDPSAAREAEILRLAADAPHVVRLHAVVPSSSPAAGA

EQPPPAALALELLPGGSLAGLLRRLGRSMGERPIAAVARQALLGLDALHALRVVHRDLKP

SNLLLGSHGEVKIADFGAGKVLRRRLDPCASYVGTAAYMSPERFDPEAYSGDYDPYAADV

WSLGLAILELYLGHFPLLPAGQRPDWAALMCAICFGDAPEAPAAASEEFRDFVARCLEKK

AGQRASVAELLEHPFIAERDAEEAKRALAALVAEAELGDL*

>Bd21-3MKK10-3 Brdisv1Bd21-3_r1001669m.p

MALLREKRLQLSLHVPTRAAEALDAVHRRPNPVAATLAASTPAAARWSQFRLADFDKLTV

LGRGNGGTVYKVRHRETCELYALKVQHCNGDPTAAAEAEVLSRTASPFIVRCHSVLPGAA

SGDVAMLLELVDGGSLDSIVKSRRAHAFPFPEEALAEVAAQALSGLAYLHARRIVHLDIK

PGNLLVSTGGEVKVADFGIAKVLPRAGADDARCKSYAGTAAYMSPERFDPEAHGGHYDAY

AADVWGLGVTVLELLMGRYPLLPAGQRPSWPALMCAICFGETPVLSDGEASAELRGFVAA

CLRKDHTKRASVAELLAHPFVAGRDVATSKCALRKLVTEASTSP*

>Bd21-3MKK10-4 Brdisv1Bd21-3_r1001667m.p

MASAKERRLPQLHLKLDVPPGAFRCAAPAPAPATAATPATSASRPPHGEFRLNDFDRLSV

LGRGNGGSVYKVSHRRTSALYALKIIHGAHARPGAADEEADIVRRVVDSPNVVRCHSVLP

TASGDAPALLLELVDGGSLDSLVGGGGFLPEAAVADVAAQALSGLAHLRARRVAHRDIKP

ANLLLSAAGEVKIADFGIAKVVVSGAGGRARALAYEGTVAYMSPERFDSERHADADPYAA

DVWGLGVTLLELLMGRYPLLPAGQKPTWAALMCAICFGELPALPEGAASLEFRGFVAACL

RKDHRKRASVVELLAHPFVAGRDVAASRRALREAIERRCSC*

>Bd21-3MKK10-5 Brdisv1Bd21-3_r1043789m.p

MALTVRQRRLPQLHISLDLPSCSFRCPNPPVAATASTSGEFRASDFERLAVLGRGNGGTV

YKVAHRRTSAQYALKVLHGGGDPGAAAAEADVLRRAADSPYVVRCHSVFPAASGSGETAL

LLELVDGGSLDSVRRGVGVSVFFPEAALAEVAAQALAGLAHLHARRVVHRDIKPANLLVS

GAGGVKVADFGIAMVLPSRAGGERCAAAYEGTVAYMSPERFDSEGRADADPRGADVWGLG

VTVLELLMGRYPLLPAGQKPTWAALMCAICFGELPALPEGAASTELRGFIAACLRKDHTK

RASVAELIKHPFVAGRNMAASRLALRRLVAGA*

>Bd3-1MKK1 Brdisv1Bd3-1_r1006392m.p

MRKPGKLALPSHESTIGKFLTQSGTFKDGDLLVNKDGLRIVHNSEEGEAPPIVPLDDHQL

SLDDLDAIKVIGKGSSGIVQLVRHKWTDQFFALKVIQLNIQESIRKQIAQELKISLSTQC

QYVVTCYQCFYVNGVISIVLEYMDGGSLADFLKTVRTIPEAYLAAICKQVLQGLMYLHHE

KRVIHRDLKPSNILINHRGEVKISDFGVSAIIASSSAQRDTFTGTFNYMAPERISGQKHG

YMSDIWSLGLVMLECATGNFPYPSPDSFYELLEAVVDQPPPSAPTDQFSPEFCSFISACI

QKEATDRSSAQVLSDHPFLSMYDDLNIDLADYFTTAGSPLATFKQIVL*

>Bd3-1MKK3-1 Brdisv1Bd3-1_r1029979m.p

MAGLEELKKKLQPLLFDDPDKDGISTRVPFLEDNCDSYVVSDGGTINLLSRSFGEYNINE

HGFHKRSTGADESDFGEKAYRCASHDMHIFGPIGNGASSVVQRAIFIPVHRILALKKINI

FEKEKRQQILNEMRTLCEASCYPGLVEFQGAFYMPDSGQISIALEYMDGGSLADVIKVKK

SIPEQVLAHMLQKVLLGLRYLHEVRHLVHRDIKPANMLVNLKGEAKITDFGVSAGLDNTM

AMCATFVGTVTYMSPERIRNENYSYAADIWSLGLTILECATGKFPYNVNEGPANLMLQIL

DDPSPTPPADAYSPEFCSFVNDCLQKDPDARPTCEQLFGHPFIKRYENAGVDLIAYVKGV

VDPTERLKEIAEMLAVHYYLLFNGSDGLWHHMKTFYMEESTFSFSGNVYVGRNDIFDTLS

SIRKKLKGDRPREKIVHVVEKLHCRANGETGIAIRVSGSLIVGNQFLVCGEGLQAEGMPS

VEELSIDIPSKRVGQFREQFMMLPGISMGSFHISRQDLYIIQA*

>Bd3-1MKK3-2 Brdisv1Bd3-1_r1035942m.p

MAGLEELKKKLQPLMFNDPDKDGFSTRVPFPEDTCDSYVVSDGGTINLLSRSFGEYNINE

HGFHKRSAGADESDFGEKAYRCASQDMHIFGPIGNGASSVVQRAIFIPVHRILALKKINI

FEKEKRQQILNEMRTLCEACCYPGLVEFQGAFYMPDSGQISIALEYMDGGSLADVIKVKK

SIPEPVLAHMLQKVLLGLRYLHEVRHLVHRDIKPANMLVNLKGEAKITDFGVSAGLDNTM

AMCATFVGTVTYMSPERIRNENYSYAADIWSLGLTILECATGKFPYNVNEGPANLMLQIL

DDPSPAPPENAFSSEFCSFVNDCLQKDADARPTCEQLLSHPFIKRYENAGVDLAAYVKGV

VNPEERLKQIAEMLAVHYYLLFNGSDGLWHHMKTFYMEDSTFSFSGNLYVGQSDIFDTLS

NIRTKLKGDRPREKIVHVVEKLHCRANEETGIAIRVSGSFIVSNQFLICGEGLQAEGMPS

LEELSIDIPSKRVGQFREQFIMHPGRSMGCYYISRQDLYIIQA*

>Bd3-1MKK3-3 Brdisv1Bd3-1_r1010293m.p

MDGGWDGMGQRKRRHRAGIPIPTYPGLAASLEFCFVTAGACHTAPGPGSKIAASLFAALL

FQRPPASTSRAIFFCFVIMAAGLEDLRRRVQPIFFDADGNVMPAPDDDSEVLDGGTINLL

SRSSDEYNINERGFHKRTIRSDDEYSSEKAFRCSCHDMHIFDSVGNGASSVVHRAIYVPV

HRVLALKKINIFEKERRQQILNEIITLSEACCYPGLVEFHGVFYTPDSGEIYFALEYMDG

GSLADIIRVKKFISEPVLSHMLQKVLLALRYLHEVRHLVHRDIKPANLLLNLKGDTKITD

FGVTSGLHDSIDMCATFLGSVTYMSPERIRNESYSYSADIWSLGLTALECATGRYPYDVN

GGEADLMLQILEDPSPTPPHDIYSEEFCSFINACLQKDADARPTCDQLLSHSFIKRYEGP

GVDLSEYNKSVHDPSERLSQIAHMLAVHYYLIFDGGDDQWCHMKTFYQQDSIFSFSGETH

VGKSEIFETLSRIRKMLKGNSPCEKIAHVMEKVYCRSHGEEGMRVRVSGSFIVGNEFVVC

ADGVRAEGMLSIDELSPDILSKQAGHFQEDFFMEPGTALGCYVISKQELHIADT*

>Bd3-1MKK4 Brdisv1Bd3-1_r1024627m.p

MDPCNSSVGTIAYMSPERINTDLNDGAYDGDAGDIWSFGLSILEFYLGRFPLGENLGKQG

DWAALMCAICYSDSPAPPPIASPEFKSFISCCLQKNPARRPSAAQLLQHRFIAGPQPQVL

AAPPS*

>Bd3-1MKK6 Brdisv1Bd3-1_r1009660m.p

MRGKKPLKELKLSVPAQETSVDKFLTASGTFKDGELRLNQRGLRLISEEENGDEHQSTNM

KVEDVQLSMDDLEMIQVIGKGSGGVVQLVQHKWVGTFYALKGIQMNIQEAVRKQIVQELK

INQATQSPHIVSCHQSFYHNGVIYLVLEYMDRGSLADIIKQVKTILEPYLAVLCKQVLEG

LLYLHHERHVIHRDIKPSNLLVNHKGEVKITDFGVSAVLASSIGQRDTFVGTYNYMAPER

ISGSSYDYKSDVWSLGLVILECAIGRFPYTPSEGEGWLSFYELLEAIVDQPPPGAPADQF

SPEFCSFISACIQKDPAERMSASELLNHAFIKKFEGKDLDLRILVESLEPPMNVPE*

>Bd3-1MKK10-3 Brdisv1Bd3-1_r1006471m.p

MALLREKRLQLSLHVPTRAAEALDAAPRRPNPVAATLAASTPAAAGGSQFRLADFDKLTV

LGRGNGGTVYKVRHRETCELYALKVQHCNGDPTAAAEAEVLSRTASPFVVADFGIAKVLP

RAGGDDARCKSYAGTAAYMSPERFDPEAHGGHYDAYAADVWGLGGTVLELLMGRYPLPPP

GQRPSWPALMCAICFGETPVLSDGEASAELRGFVAACLRKDHTKRASVAELLAHPFVAGR

GVAPPKCALRKLVTEASTSP*

>BdTR10cMKK1 Brdisv1BdTR10c1006888m.p

MRKPGKLALPSHESTIGKFLTQSGTFKDGDLLVNKDGLRIVHNSEEGEAPPIVPLDDHQL

SLDDLDAIKVIGKGSSGIVQLVRHKWTDQFFALKVIQLNIQESIRKQIAQELKISLSTQC

QYVVTCYQCFYVNGVISIVLEYMDGGSLADFLKTVRTIPEAYLAAICKQVLQGLMYLHHE

KRVIHRDLKPSNILINHRGEVKISDFGVSAIIASSSAQRDTFTGTFNYMAPERISGQKHG

YMSDIWSLGLVMLECATGNFPYPSPDSFYELLEAVVDQPPPSAPTDQFSPEFCSFISACI

QKEATDRSSAQVLSDHPFLSMYDDLNIDLADYFTTAGSPLATFKQIVL*

>BdTR10cMKK3-1 Brdisv1BdTR10c1033120m.p

MAGLEELKKKLQPLLFDDPDKDGISTRVPFLEDNCDSYVVSDGGTINLLSRSFGEYNINE

HGFHKRSTGADESDFGEKAYRCASHDMHIFGPIGNGASSVVQRAIFIPVHRILALKKINI

FEKEKRQQILNEMRTLCEASCYPGLVEFQGAFYMPDSGQISIALEYMDGGSLADVIKVKK

SIPEQVLAHMLQKVLLGLRYLHEVRHLVHRDIKPANMLVNLKGEAKITDFGVSAGLDNTM

AMCATFVGTVTYMSPERIRNENYSYAADIWSLGLTILECATGKFPYNVNEGPANLMLQIL

DDPSPTPPADAYSPEFCSFVNDCLQKDPDARPTCEQLFGHPFIKRYENAGVDLIAYVKGV

VDPTERLKEIAEMLAVHYYLLFNGSDGLWHHMKTFYMEESTFSFSGNVYVGRNDIFDTLS

SIRKKLKGDRPREKIVHVVEKLHCRANGETGIAIRVSGSLIVGNQFLVCGEGLQAEGMPS

VEELSIDIPSKRVGQFREQFMMLPGISMGSFHISRQDLYIIQA*

>BdTR10cMKK3-2 Brdisv1BdTR10c1038053m.p

MAGLEELKKKLQPLMFNDPDKDGFSTRVPFPEDTCDSYVVSDGGTINLLSRSFGEYNINE

HGFHKRSAGADESDFGEKAYRCASQDMHIFGPIGNGASSVVQRAIFIPVHRILALKKINI

FEKEKRQQILNEMRTLCEACCYPGLVEFQGAFYMPDSGQISIALEYMDGGSLADVIKVKK

SIPEPVLAHMLQKVLLGLRYLHEVRHLVHRDIKPANMLVNLKGEAKITDFGVSAGLDNTM

AMCATFVGTVTYMSPERIRNENYSYAADIWSLGLTILECATGKFPYNVNEGPANLMLQIL

DDPSPAPPENAFSSEFCSFVNDCLQKDADARPTCEQLLSHPFIKRYENAGVDLAAYVKGV

VNPEERLKQIAEMLAVHYYLLFNGSDGLWHHMKTFYMEDSTFSFSGNLYVGQSDIFDTLS

NIRTKLKGDRPREKIVHVVEKLHCRANEETGIAIRVSGSFIVSNQFLICGEGLQAEGMPS

LEELSIDIPSKRVGQFREQFIMHPGRSMGCYYISRQDLYIIQA*

>BdTR10cMKK3-3 Brdisv1BdTR10c1021519m.p

MDGGWDGMGQRKRRHRAGIPIPTYPGLAASLEFCFVTAGACHTAPGPGSKIAASLFAALL

FQRPPASTSRAIFFCFVIMAGGLEGLRRRVQPIFFDADGNVMPAPDDDSEVLDGGTINLL

SRSSDEYNINERGFHKRTIRSDDEYSSEKAFRCSCHDMHIFDSVGNGASSVVHRAIYVPV

HRVLALKKINIFEKERRQQILNEIITLSEACCYPGLVEFHGVFYTPDSGEIYFALEYMDG

GSLADIIRVKKFISEPVLSHMLQKVLLALRYLHEVRHLVHRDIKPANLLLNLKGDTKITD

FGVTSGLHDSIDMCATFLGSVTYMSPERIRNESYSYSADIWSLGLTALECATGRYPYDVN

GGEADLMLQILEDPSPTPPHDIYSEEFCSFINACLQKDADARPTCDQLLSHSFIKRYEGP

GVDLSEYNKSVHDPSERLSQIAHMLAVHYYLIFDGGDDQWCHMKTFYQQDSIFSFSGETH

VGKSEIFETLSRIRKMLKGNSPCEKIAHVMEKVYCRSHGEEGMRVRVSGSFIVGNEFVVC

ADGVRAEGMLSIDELSPDILSKQAGHFQEDFFMEPGTALGCYVISKQELHIADT*

>BdTR10cMKK4 Brdisv1BdTR10c1021040m.p

MVRHRPTGRPYALKVLYGNHDDAVRRQITREIAILRPAEHPAIVRCHGMYEQAGELQILL

EFMDGGSLEGRRIASEAFLADVARQVLSGIAYLPRRHIVHRDIKPSNLLIDSGRRVKIAD

FGVGRILNQTMDPCNSSVGTIAYMSPERINTDLNDGAYDGYAGDIWSFGLSILEFYLGRF

PLGENLGKQGDWAALMCAICYSDSPAPPPIASPEFKSFISCCLQKNPARRPSAAQLLQPR

FIAGPQPQVLAAPPS*

>BdTR10cMKK5 Brdisv1BdTR10c1006269m.p

GFLADVARQVLSGIAYLHRRHIVHRDIKPSNLLIDSARRVKIADFGVGRILNQTMDPCNS

SVGTIAYMSPERINTDLNDGAYDGYAGDIWSFGLSILEFYLGRFPFGENLGKQGDWAALM

VAICYNDPPEPSAAASPEFRGFISCCLQKNPAKRLSAAQLLQHPFVAGPQPLPLAPPPS*

>BdTR10cMKK6 Brdisv1BdTR10c1010577m.p

MRGKKPLKELKLSVPAQETSVDKFLTASGTFKDGELRLNQRGLRLISEEENGDEHQSTNM

KVEDVQLSMDDLEMIQVIGKGSGGVVQLVQHKWVGTFYALKGIQMNIQEAVRKQIVQELK

INQATQSPHIVSCHQSFYHNGVIYLVLEYMDRGSLADIIKQVKTILEPYLAVLCKQVLEG

LLYLHHERHVIHRDIKPSNLLVNHKGEVKITDFGVSAVLASSIGQRDTFVGTYNYMAPER

ISGSSYDYKSDVWSLGLVILECAIGRFPYTPSEGEGWLSFYELLEAIVDQPPPGAPADQF

SPEFCSFISACIQKDPAERMSASELLNHAFIKKFEGKDLDLRILVESLEPPMNVPE*

>BdTR10cMKK10-1 Brdisv1BdTR10c1001595m.p

MALLREKRLQLSLHVRHRETCELYALKVQHCNGDATAEAEVLSRTASPFVAPCHSVLPAA

ASGDVAMLLELVDGGSLDSIVKSRSRGQAEAFSQFPEEALAEVAAQALSGLAYLHARRIV

HLDVKPGNLLVSTGGEVKIADFGIARVLPRAGGDDVRCTAYAGTAAYMSPERFDPEAHGG

HYDPYAADVWGLGVTVLELLMGRYPLLPAGQRPSWAALMCAICFGETPALSDGEASAELR

GVVAACLHKDYRRRASVAELLAHPFVAGRDVAASKCALRKLVTEASMSP*

>BdTR10cMKK10-2 Brdisv1BdTR10c1009777m.p

MGERPIAAVARQALLGLDALHALRVVHRDLKPSNLLLGSHGEVKIADFGAGKVLRRRLDP

CASYVGTAAYMSPERFDPEAYSGDYDPYAADVWSLGLAILELYLGHFPLLPAGQRPDWAA

LMCAICFGDAPEAPAAASEEFRDFVARCLEKKAGQRASVAELLEHPFIAERDAEEAKRAL

AALVAEAELGDL*

>BdTR10cMKK10-3 Brdisv1BdTR10c1040500m.p

MTLVKATPTEIRQKRLQLSLHVPTRAAEALDAVHRRPNPFRLADFDKLTVLGRGNGGTVY

KVRHRETCELYALKLVDGGSLDSIVKSRRAHAFPFPEEALAEVAAQALSGLAYLHARRIV

HLDIKPGNLLVSTGGEVKVADFGIAKVLPRAGADDARVLFSGG*

>BdTR10cMKK10-4 Brdisv1BdTR10c1044301m.p

SLVDSPNVVRCHSVLPTASGDAAALLLELVDGGSLDSLVGGGGFLPEAAVADVAAQALSG

LAHLRARRVAHRDIKPANLLLSAAGEVKIADFGIAKVVVSGAGGRARALAYEGTVAYMSP

ERFDSERHADADPYAADVWGLGVTLLELLMGRYPLLPAGQKPTWAALMCAICFGELPALP

EGAASLEFRGFVAACLRKDHRTACWGC*

>BdTR10cMKK10-5 Brdisv1BdTR10c1041730m.p

MXVRRGVGVSVFFPEAALAEVAAQALAGLAHLHARRVVHRDIKPANLLVSGAGGVKVADF

GIAMVLPSRAGGERCAAAYEGTVAYMSPERFDSEGRADADPRGADVWGLGVTVLELLMGR

YPLLPAGQKPTWAALMCAICFGELPALPEGAASTELRGFIAACLRKDHTKRASVAELIKH

PFVAGRNMAASRLALRRLVAGA*

>BdTR11aMKK1 Brdisv1BdTR11A1007305m.p

MRKPGKLALPSHESTIGKFLTQSGTFKDGDLLVNKDGLRIVHNSEEGEAPPIVPLDDHQL

SLDDLDAIKVIGKGSSGIVQLVRHKWTDQFFALKVIQLNIQESIRKQIAQELKISLSTQC

QYVVTCYQCFYVNGVISIVLEYMDGGSLADFLKTVRTIPEAYLAAICKQVLQGLMYLHHE

KRVIHRDLKPSNILINHRGEVKISDFGVSAIIASSSAQRDTFTGTFNYMAPERISGQKHG

YMSDIWSLGLVMLECATGNFPYPSPDSFYELLEAVVDQPPPSAPTDQFSPEFCSFISACI

QKEATDRSSAQVLSDHPFLSMYDDLNIDLADYFTTAGSPLATFKQIVL*

>BdTR11aMKK3-1 Brdisv1BdTR11A1026487m.p

MAGLEELKKKLQPLLFDDPDKDGISTRVPFLEDNCDSYVVSDGGTINLLSRSFGEYNINE

HGFHKRSTGADESDFGEKAYRCASHDMHIFGPIGNGASSVVQRAIFIPVHRILALKKINI

FEKEKRQQILNEMRTLCEASCYPGLVEFQGAFYMPDSGQISIALEYMDGGSLADVIKVKK

SIPEQVLAHMLQKVLLGLRYLHEVRHLVHRDIKPANMLVNLKGEAKITDFGVSAGLDNTM

AMCATFVGTVTYMSPERIRNENYSYAADIWSLGLTILECATGKFPYNVNEGPANLMLQIL

DDPSPTPPADAYSPEFCSFVNDCLQKDPDARPTCEQLFGHPFIKRYENAGVDLIAYVKGV

VDPTERLKEIAEMLAVHYYLLFNGSDGLWHHMKTFYMEESTFSFSGNVYVGRNDIFDTLS

SIRKKLKGDRPREKIVHVVEKLHCRANGETGIAIRVSGSLIVGNQFLVCGEGLQAEGMPS

VEELSIDIPSKRVGQFREQFMMLPGISMGSFHISRQDLYIIQA*

>BdTR11aMKK3-2 Brdisv1BdTR11A1042993m.p

MAGLEELKKKLQPLMFNDPDKDGFSTRVPFPEDTCDSYVVSDGGTINLLSRSFGEYNINE

HGFHKRSAGADESDFGEKAYRCASQDMHIFGPIGNGASSVVQRAIFIPVHRILALKKINI

FEKEKRQQILNEMRTLCEACCYPGLVEFQGAFYMPDSGQISIALEYMDGGSLADVIKVKK

SIPEPVLAHMLQKVLLGLRYLHEVRHLVHRDIKPANMLVNLKGEAKITDFGVSAGLDNTM

AMCATFVGTVTYMSPERIRNENYSYAADIWSLGLTILECATGKFPYNVNEGPANLMLQIL

DDPSPAPPENAFSSEFCSFVNDCLQKDADARPTCEQLLSHPFIKRYENAGVDLAAYVKGV

VNPEERLKQIAEMLAVHYYLLFNGSDGLWHHMKTFYMEDSTFSFSGNLYVGQSDIFDTLS

NIRTKLKGDRPREKIVHVVEKLHCRANEETGIAIRVSGSFIVSNQFLICGEGLQAEGMPS

LEELSIDIPSKRVGQFREQFIMHPGRSMGCYYISRQDLYIIQA*

>BdTR11aMKK3-3 Brdisv1BdTR11A1029299m.p

MDGGWDGMGQRKRRHRAGIPIPTYPGLAASLEFCFVTAGACHTAPGPGSKIAASLFAALL

FQRPPASTSRAIFFCFVIMAAGLEDLRRRVQPIFFDADGNVMPAPDDDSEVLDGGTINLL

SRSSDEYNINERGFHKRTIRSDDEYSSEKAFRCSCHDMHIFDSVGNGASSVVHRAIYVPV

HRVLALKKINIFEKERRQQILNEIITLSEACCYPGLVEFHGVFYTPDSGEIYFALEYMDG

GSLADIIRVKKFISEPVLSHMLQKVLLALRYLHEVRHLVHRDIKPANLLLNLKGDTKITD

FGVTSGLHDSIDMCATFLGSVTYMSPERIRNESYSYSADIWSLGLTALECATGRYPYDVN

GGEADLMLQILEDPSPTPPHDIYSEEFCSFINACLQKDADARPTCDQLLSHSFIKRYEGP

GVDLSEYNKSVHDPSERLSQIAHMLAVHYYLIFDGGDDQWCHMKTFYQQDSIFSFSGETH

VGKSEIFETLSRIRKMLKGNSPCEKIAHVMEKVYCRSHGEEGMRVRVSGSFIVGNEFVVC

ADGVRAEGMLSIDELSPDILSKQAGHFQEDFFMEPGTALGCYVISKQELHIADT*

>BdTR11aMKK4 Brdisv1BdTR11A1034905m.p

MRPGGPPNARPQQPGTPGRARRRPDLTLPLPQRDLTSLAVPLPLPPPPSSAPSSASSSGS

SLSSMGAPPPPNSAGSAPPPPPPLAELERVRRIGSGAGGTVWMVRHRPTGRPYALKVLYG

NHDDAVRRQITREIAILRTAEHPAIVRCHGMYEQAGELQILLEFMDGGSLEGRRIASEAF

LADVARQVLSGIASLHRRHIVHRDIKPSNLLIDSGRRVKIADFGVGRILNQTMDPCNSSV

GTIAYMSPERINTDLNDGAYDGYAGDIWSFGLSILEFYLGRFPLGENLGKQGDWAALMCA

ICYSDSPAPPPIASPEFKSFISCCLQKNPARRPSAAQLLQHRFIAGPQPQVLAAPPS*

>BdTR11aMKK5 Brdisv1BdTR11A1006653m.p

MRPAGSLPSPQPGTPGRPRRRPDLTLPMPQRPDVSSSLAVPLPLPPPSSLGLAQPPAAAA

AAAAPPPPPLGELERVRRVGSGAGGTVWMVRHRPTGRCYALKQLYGNHDDAVRRQIAREI

AILRTAEHPAVVRCHGMYERGGELQILLEYMDGGSLDGRRIAAEGFLADVARQVLSGIAY

LPRRHIVHRDIKPSNLLIDSARRVKIADFGVGRILNQTMDPCNSSVGTIAYMSPERINTD

LNDGAYDGYAGDIWSFGLSILEFYLGRFPFGENLGKQGDWAALMVAICYNDPPEPSAAAS

PEFRGFISCCLQKNPAKRLSAAQLLQHPFVAGPQPLPLAAPPS*

>BdTR11aMKK6 Brdisv1BdTR11A1011184m.p

MRGKKPLKELKLSVPAQETSVDKFLTASGTFKDGELRLNQRGLRLISEEENGDEHQSTNM

KVEDVQLSMDDLEMIQVIGKGSGGVVQLVQHKWVGTFYALKGIQMNIQEAVRKQIVQELK

INQATQSPHIVSCHQSFYHNGVIYLVLEYMDRGSLADIIKQVKTILEPYLAVLCKQVLEG

LLYLHHERHVIHRDIKPSNLLVNHKGEVKITDFGVSAVLASSIGQRDTFVGTYNYMAPER

ISGSSYDYKSDVWSLGLVILECAIGRFPYTPSEGEGWLSFYELLEAIVDQPPPGAPADQF

SPEFCSFISACIQKDPAERMSASELLNHAFIKKFEGKDLDLRILVESLEPPMNVPE*

>BdTR11aMKK10-1 Brdisv1BdTR11A1001689m.p

MALLREKRLQLSLHVPTRAADAQEAGLHRRPNPAAALPLAATPPAARSSQFRVADFEKLA

VLGRGNGGTVYKVRHRETCELYALKVQHCNGDATAEAEVLSRTASPFVVRCHSVLPAAAS

GDVAMLLELVDGGSLDSIVKSRSRGQAEAFSQFPEEALAEVAAQALSGLAYLHARRIVHL

DVKPGNLLVSTGGEVKIADFGIARVLPRAGGDDVRCTAYAGTAAYMSPERFDPEAHGGHY

DPYAADVWGLGVTVLELLMGRYPLLPAGQRPSWAALMCAICFGETPALSDGEASAELRGF

VAACLHKDYRRRASVAELLAHPFVAGRDVAASKCALRKLVTEASMSP*

>BdTR11aMKK10-2 Brdisv1BdTR11A1010326m.p

MALVRQRRQLPHLTLPLDHFALRPPPVPAPAPTVAASTSSEAAGLRLSDFERISLLGQGN

GGTVYKARHRRAAAQPPVALKLFVAGDPSAAREAEILRLAADAPHVVRLHAVVPSSSPAA

GAEQPPPAALALELLPGGSLAGLLRRLGRSMGERPIAAVARQALLGLDALHALRVVHRDL

KPSNLLLGSHGEVKIADFGAGKVLRRRLDPCASYVGTAAYMSPERFDPEAYSGDYDPYAA

DVWSLGLAILELYLGHFPLLPAGQRPDWAALMCAICFGDAPEAPAAASEEFRDFVARCLE

KKAGQRASVAELLEHPFIAERDAEEAKRALAALVAEAELGDL*

>BdTR11aMKK10-3 Brdisv1BdTR11A1001594m.p

MALLREKRLQLSLHVPTRAAEALDAVHRRPNPVAATLAASTPAAARSSQFRLADFDKLTV

LGRGNGGTVYKVRHRETCELYALKVQHCNGDPTAAAEAEVLSRTASPFIVRCHSVLPGAA

SGDVAMLLELVDGGSLDSIVKSRRAHAFPFPEEALAEVAAQALSGLAYLHARRIVHLDIK

PGNLLVSTGGEVKVADFGIAKVLPRAGADDARCKSYAGTAAYMSPERFDPEAHGGHYDAY

AADVWGLGVTVLELLMGRYPLLPAGQRPSWPALMCAICFGETPVLSDGEASAELRGFVAA

CLRKDHTKRASVAELLAHPFVAGRDVATSKCALRKLVTEASTSP*

>BdTR11aMKK10-4 Brdisv1BdTR11A1001588m.p

MASAKERRLPQLHLKLDVPTCAFRCAAPAPAPATAATPATSASRPPHGEFRLNDFDRLSV

LGRGNGGSVYKVSHRRTSALYALKIIHGAHARPGAADEEADIVRRVVDSPNVVRCHSVLP

TASGDAAALLLELVDGGSLDSLVGGGGFLPEAAVADVAAQALSGLAHLRARRVAHRDIKP

ANLLLSAAGEVKIADFGIAKVVVSGAGGRARALAYEGTVAYMSPERFDSERHADADPYAA

DVWGLGVTLLELLMGRYPLLPAGQKPTWAALMCAICFGELPALPEGAASLEFRGFVAACL

RKDHRKRASVVELLAHPFVAGRDVAASRRALREAIERRCSC*

>BdTR11aMKK10-5 Brdisv1BdTR11A1001590m.p

MALTVRQRRLPQLHISLDLPSCSFRCPTPPVAATASTSGEFRASDFERLAGLGRGNGGTV

YKVAHRRTSAQYALKVLHGGGDPGAAAAEADVLRRAADSPYVVRCHSVFPAASGSGETAL

LLELVDGGSLDSVRRGVGVSVFFPEAALAEVAAQALAGLAHLHARRVVHRDIKPANLLVS

GAGGVKVADFGIAMVLPSRAGGERCAAAYEGTVAYMSPERFDSEGRADADPRGADVWGLG

VTVLELLMGRYPLLPAGQKPTWAALMCAICFGELPALPEGAASTELRGFIAACLRKDHTK

RASVAELIKHPFVAGRNMAASRLALRRLVAGA*

>BdTR11gMKK1 Brdisv1BdTR11G1008252m.p

MRKPGKLALPSHESTIGKFLTQSGTFKDGDLLVNKDGLRIVHNSEEGEAPPIVPLDDHQL

SLDDLDAIKVIGKGSSGIVQLVRHKWTDQFFALKVIQLNIQESIRKQIAQELKISLSTQC

QYVVTCYQCFYVNGVISIVLEYMDGGSLADFLKTVRTIPEAYLAAICKQVLQGLMYLHHE

KRVIHRDLKPSNILINHRGEVKISDFGVSAIIASSSAQRDTFTGTFNYMAPERISGQKHG

YMSDIWSLGLVMLECATGNFPYPSPDSFYELLEAVVDQPPPSAPTDQFSPEFCSFISACI

QKEATDRSSAQVLSDHPFLSMYDDLNIDLADYFTTAGSPLATFKQIVL*

>BdTR11gMKK3-1 Brdisv1BdTR11G1029451m.p

MAGLEELKKKLQPLLFDDPDKDGISTRVPFLEDNCDSYVVSDGGTINLLSRSFGEYNINE

HGFHKRSTGADESDFGEKAYRCASHDMHIFGPIGNGASSVVQRAIFIPVHRILALKKINI

FEKEKRQQILNEMRTLCEASCYPGLVEFQGAFYMPDSGQISIALEYMDGGSLADVIKVKK

SIPEQVLAHMLQKVLLGLRYLHEVRHLVHRDIKPANMLVNLKGEAKITDFGVSAGLDNTM

AMCATFVGTVTYMSPERIRNENYSYAADIWSLGLTILECATGKFPYNVNEGPANLMLQIL

DDPSPTPPADAYSPEFCSFVNDCLQKDPDARPTCEQLFGHPFIKRYENAGVDLIAYVKGV

VDPTERLKEIAEMLAVHYYLLFNGSDGLWHHMKTFYMEESTFSFSGNVYVGRNDIFDTLS

SIRKKLKGDRPREKIVHVVEKLHCRANGETGIAIRVSGSLIVGNQFLVCGEGLQAEGMPS

VEELSIDIPSKRVGQFREQFMMLPGISMGSFHISRQDLYIIQA*

>BdTR11gMKK3-2 Brdisv1BdTR11G1044565m.p

MAGLEELKKKLQPLMFNDPDKDGFSTRVPFPEDTCDSYVVSDGGTINLLSRSFGEYNINE

HGFHKRSAGADESDFGEKAYRCASQDMHIFGPIGNGASSVVQRAIFIPVHRILALKKINI

FEKEKRQQILNEMRTLCEACCYPGLVEFQGAFYMPDSGQISIALEYMDGGSLADVIKVKK

SIPEPVLAHMLQKVLLGLRYLHEVRHLVHRDIKPANMLVNLKGEAKITDFGVSAGLDNTM

AMCATFVGTVTYMSPERIRNENYSYAADIWSLGLTILECATGKFPYNVNEGPANLMLQIL

DDPSPAPPENAFSSEFCSFVNDCLQKDADARPTCEQLLSHPFIKRYENAGVDLAAYVKGV

VNPEERLKQIAEMLAVHYYLLFNGSDGLWHHMKTFYMEDSTFSFSGNLYVGQSDIFDTLS

NIRTKLKGDRPREKIVHVVEKLHCRANEETGIAIRVSGSFIVSNQFLICGEGLQAEGMPS

LEELSIDIPSKRVGQFREQFIMHPGRSMGCYYISRQDLYIIQA*

>BdTR11gMKK3-3 Brdisv1BdTR11G1032411m.p

MDGGWDGMGQRKRRHRAGIPIPTYPGLAASLEFCFVTAGACHTAPGPGSKIAASLFAALL

FQRPPASTSRAIFFCFVIMAAGLEDLRRRVQPIFFDADGNVMPAPDDDSEVLDGGTINLL

SRSSDEYNINERGFHKRTIRSDDEYSSEKAFRCSCHDMHIFDSVGNGASSVVHRAIYVPV

HRVLALKKINIFEKERRQQILNEIITLSEACCYPGLVEFHGVFYTPDSGEIYFALEYMDG

GSLADIIRVKKFISEPVLSHMLQKVLLALRYLHEVRHLVHRDIKPANLLLNLKGDTKITD

FGVTSGLHDSIDMCATFLGSVTYMSPERIRNESYSYSADIWSLGLTALECATGRYPYDVN

GGEADLMLQILEDPSPTPPHDIYSEEFCSFINACLQKDADARPTCDQLLSHSFIKRYEGP

GVDLSEYNKSVHDPSERLSQIAHMLAVHYYLIFDGGDDQWCHMKTFYQQDSIFSFSGETH

VGKSEIFETLSRIRKMLKGNSPCEKIAHVMEKVYCRSHGEEGMRVRVSGSFIVGNEFVVC

ADGVRAEGMLSIDELSPDILSKQAGHFQEDFFMEPGTALGCYVISKQELHIADT*

>BdTR11gMKK4 Brdisv1BdTR11G1038753m.p

MRPGGPPNARPQQPGTPGRARRRPDLTLPLPQRDLTSLAVPLPLPPPPSSAPSSASSSGS

SLSSMGAPPPPNSAGSAPPPPPPLAELERVRRIGSGAGGTVWMVRHRPTGRPYALKVLYG

NHDDAVRRQITREIAILRTAEHPAIVRCHGMYEQAGELQILLEFMDGGSLEGRRIASEAF

LADVARQVLSGIAYLHRRHIVHRDIKPSNLLIDSGRRVKIADFGVGRILNQTMDPCNSSV

GTIAYMSPERINTDLNDGAYDGYAGDIWSFGLSILEFYLGRFPLGENLGKQGDWAALMCA

ICYSDSPAPPPIASPEFKSFISCCLQKNPARRPSAAQLLQHRFIAGPQPQVLAAPPS*

>BdTR11gMKK5 Brdisv1BdTR11G1007511m.p

MRPAGSLPSPQPGTPGRPRRRPDLTLPMPQRPDVSSSLAVPLPLPPPSSLGLAQPPAAAA

AAAAPPPPPLGELERVRRVGSGAGGTVWMVRHRPTGRCYALKQLYGNHDDAVRRQIAREI

AILRTAEHPAVVRCHGMYERGGELQILLEYMDGGSLDGRRIAAEGFLADVARQVLSGIAY

LHRRHIVHRDIKPSNLLIDSARRVKIADFGVGRILNQTMDPCNSSVGTIAYMSPERINTD

LNDGAYDGYAGDIWSFGLSILEFYLGRFPFGENLGKQGDWAALMVAICYNDPPEPSAAAS

PEFRGFISCCLQKNPAKRLSAAQLLQHPFVAGPQPLPLAAPPS*

>BdTR11gMKK6 Brdisv1BdTR11G1012553m.p

MRGKKPLKELKLSVPAQETSVDKFLTASGTFKDGELRLNQRGLRLISEEENGDEHQSTNM

KVEDVQLSMDDLEMIQVIGKGSGGVVQLVQHKWVGTFYALKGIQMNIQEAVRKQIVQELK

INQATQSPHIVSCHQSFYHNGVIYLVLEYMDRGSLADIIKQVKTILEPYLAVLCKQVLEG

LLYLHHERHVIHRDIKPSNLLVNHKGEVKITDFGVSAVLASSIGQRDTFVGTYNYMAPER

ISGSSYDYKSDVWSLGLVILECAIGRFPYTPSEGEGWLSFYELLEAIVDQPPPGAPADQF

SPEFCSFISACIQKDPAERMSASELLNHAFIKKFEGKDLDLRILVESLEPPMNVPE*

>BdTR11gMKK10-1 Brdisv1BdTR11G1001791m.p

MALLREKRLQLSLHVPTRAADAQEAGLHRRPNPAAALPLAATPPAARSSQFRVADFEKLA

VLGRGNGGTVYKVRHRETCELYALKVQHCNGDATAEAEVLSRTASPFVVRCHSVLPAAAS

GDVAMLLELVDGGSLDSIVKSRSRGQAEAFSQFPEEALAEVAAQALSGLAYLHARRIVHL

DVKPGNLLVSTGGEVKIADFGIARVLPRAGGDDVRCTAYAGTAAYMSPERFDPEAHGGHY

DPYAADVWGLGVTVLELLMGRYPLLPAGQRPSWAALMCAICFGETPALSDGEASAELRGF

VAACLHKDYRRRASVAELLAHPFVAGRDVAASKCALRKLVTEASMSP*

>BdTR11gMKK10-2 Brdisv1BdTR11G1011611m.p

MALVRQRRQLPHLPLPLDHFALRPPPVPAPAPTVAASTSSEAAGLRLSDFERISLLGQGN

GGTVYKARHRRAAAQPPVALKLFVAGDPSAAREAEILRLAADAPHVVRLHAVAPSSPPAA

GAEQPPPAALALELLPGGSLAGLLRRLGRSMGERPIAAVARQALLGLDALHALRVVHRDL

KPSNLLLGSHGEVKIADFGAGKVLRRRLDPCASYVGTAAYMSPERFDPEAYSGDYDPYAA

DVWSLGLAILELYLGHFPLLPAGQRPDWAALMCAICFGDAPEAPAAASEEFRDFVARCLE

KKAGQRASVAELLEHPFIAERDAEEAKRALAALVAEAELGDL*

>BdTR11gMKK10-3 Brdisv1BdTR11G1001687m.p

MALLREKRLQLSLHVPTRAAEALDAVHRRPNPVAATLAASTPAAARSSQFRLADFDKLTV

LGRGNGGTVYKVRHRETCELYALKVQHCNGDPTAAAEAEVLSRTASPFIVRCHSVLPGAA

SGDVAMLLELVDGGSLDSIVKSRRAHAFPFPEEALAEVAAQALSGLAYLHARRIVHLDIK

PGNLLVSTGGEVKVADFGIAKVLPRAGADDARCKSYAGTAAYMSPERFDPEAHGGHYDAY

AADVWGLGVTVLELLMGRYPLLPAGQRPSWPALMCAICFGETPVLSDGEASAELRGFVAA

CLRKDHTKRASVAELLAHPFVAGRDVATSKCALRKLVTEASTSP*

>BdTR11gMKK10-4 Brdisv1BdTR11G1001684m.p

MASAKERRLPQLHLKLDVPTCAFRCAAPAPAPATAATPATSASRPPHGEFRLNDFDRLSV

LGRGNGGSVYKVSHRRTSALYALKIIHGAHARPGAADEEADIVRRVVDSPNVVRCHSVLP

TASGDAAALLLELVDGGSLDSLVGGGGFLPEAAVADVAAQALSGLAHLRARRVAHRDIKP

ANLLLSAAGEVKIADFGIAKVVVSGAGGRARALAYEGTVAYMSPERFDSERHADADPYAA

DVWGLGVTLLELLMGRYPLLPAGQKPTWAALMCAICFGELPALPEGAASLEFRGFVAACL

RKDHRKRASVVELLAHPFVAGRDVAASRRALREAIERRCSC*

>BdTR11gMKK10-5 Brdisv1BdTR11G1001686m.p

MALTVRQRRLPQLHISLDLPSCSFRCPTPPVAATASTSGEFRASDFERLAVLGRGNGGTV

YKGAHRRTSAQYALKVLHGGGDPGAAAAEADVLRRAADSPYVVRCHSVFPAASGSGETAL

LLELVDGGSLDSVRRGVGGSVFFPEAALAEVAAQALAGLAHLHARRVVHRDIKPANLLVS

GAGGVKVADFGIAMVLPSRAGGERCAAAYEGTVAYMSPERFDSEGRADADPRGADVWGLG

VTVLELLMGRYPLLPAGQKPTWAALMCAICFGELPALPEGAASTELRGFIAACLRKDHTK

RASVAELIKHPFVAGRNMAASRLALRRLVAGA*

>BdTR11iMKK1 Brdisv1BdTR11I1007977m.p

MRKPGKLALPSHESTIGKFLTQSGTFKDGDLLVNKDGLRIVHNSEEGEAPPIVPLDDHQL

SLDDLDAIKVIGKGSSGIVQLVRHKWTDQFFALKVIQLNIQESIRKQIAQELKISLSTQC

QYVVTCYQCFYVNGVISIVLEYMDGGSLADFLKTVRTIPEAYLAAICKQVLQGLMYLHHE

KRVIHRDLKPSNILINHRGEVKISDFGVSAIIASSSAQRDTFTGTFNYMAPERISGQKHG

YMSDIWSLGLVMLECATGNFPYPSPDSFYELLEAVVDQPPPSAPTDQFSPEFCSFISACI

QKEATDRSSAQVLSDHPFLSMYDDLNIDLADYFTTAGSPLATFKQIVL*

>BdTR11iMKK3-1 Brdisv1BdTR11I1028588m.p

MAGLEELKKKLQPLLFDDPDKDGISTRVPFLEDNCDSYVVSDGGTINLLSRSFGEYNINE

HGFHKRSTGADESDFGEKAYRCASHDMHIFGPIGNGASSVVQRAIFIPVHRILALKKINI

FEKEKRQQILNEMRTLCEASCYPGLVEFQGAFYMPDSGQISIALEYMDGGSLADVIKVKK

SIPEQVLAHMLQKVLLGLRYLHEVRHLVHRDIKPANMLVNLKGEAKITDFGVSAGLDNTM

AMCATFVGTVTYMSPERIRNENYSYAADIWSLGLTILECATGKFPYNVNEGPANLMLQIL

DDPSPTPPADAYSPEFCSFVNDCLQKDPDARPTCEQLFGHPFIKRYENAGVDLIAYVKGV

VDPTERLKEIAEMLAVHYYLLFNGSDGLWHHMKTFYMEESTFSFSGNVYVGRNDIFDTLS

SIRKKLKGDRPREKIVHVVEKLHCRANGETGIAIRVSGSLIVGNQFLVCGEGLQAEGMPS

VEELSIDIPSKRVGQFREQFMMLPGISMGSFHISRQDLYIIQA*

>BdTR11iMKK3-2 Brdisv1BdTR11I1043285m.p

MAGLEELKKKLQPLMFNDPDKDGFSTRVPFPEDTCDSYVVSDGGTINLLSRSFGEYNINE

HGFHKRSAGADESDFGEKAYRCASQDMHIFGPIGNGASSVVQRAIFIPVHRILALKKINI

FEKEKRQQILNEMRTLCEACCYPGLVEFQGAFYMPDSGQISIALEYMDGGSLADVIKVKK

SIPEPVLAHMLQKVLLGLRYLHEVRHLVHRDIKPANMLVNLKGEAKITDFGVSAGLDNTM

AMCATFVGTVTYMSPERIRNENYSYAADIWSLGLTILECATGKFPYNVNEGPANLMLQIL

DDPSPAPPENAFSSEFCSFVNDCLQKDADARPTCEQLLSHPFIKRYENAGVDLAAYVKGV

VNPEERLKQIAEMLAVHYYLLFNGSDGLWHHMKTFYMEDSTFSFSGNLYVGQSDIFDTLS

NIRTKLKGDRPREKIVHVVEKLHCRANEETGIAIRVSGSFIVSNQFLICGEGLQAEGMPS

LEELSIDIPSKRVGQFREQFIMHPGRSMGCYYISRQDLYIIQA*

>BdTR11iMKK3-3 Brdisv1BdTR11I1031672m.p

MDGGWDGMGQRKRRHRAGIPIPTYPGLAASLEFCFVTAGACHTAPGPGSKIAASLFAALL

FQRPPASTSRAIFFCFVIMAAGLEDLRRRVQPIFFDADGNVMPAPDDDSEVLDGGTINLL

SRSSDEYNINERGFHKRTIRSDDEYSSEKAFRCSCHDMHIFDSVGNGASSVVHRAIYVPV

HRVLALKKINIFEKERRQQILNEIITLSEACCYPGLVEFHGVFYTPDSGEIYFALEYMDG

GSLADIIRVKKFISEPVLSHMLQKVLLALRYLHEVRHLVHRDIKPANLLLNLKGDTKITD

FGVTSGLHDSIDMCATFLGSVTYMSPERIRNESYSYSADIWSLGLTALECATGRYPYDVN

GGEADLMLQILEDPSPTPPHDIYSEEFCSFINACLQKDADARPTCDQLLSHSFIKRYEGP

GVDLSEYNKSVHDPSERLSQIAHMLAVHYYLIFDGGDDQWCHMKTFYQQDSIFSFSGETH

VGKSEIFETLSRIRKMLKGNSPCEKIAHVMEKVYCRSHGEEGMRVRVSGSFIVGNEFVVC

ADGVRAEGMLSIDELSPDILSKQAGHFQEDFFMEPGTALGCYVISKQELHIADT*

>BdTR11iMKK4 Brdisv1BdTR11I1037783m.p

MRPGGPPNARPQQPGTPGRARRRPDLTLPLPQRDLTSLAVPLPLPPPPSSAPSSASSSGS

SLSSMGAPTPPNSAGSAPPPPPPLAELERVRRIGSGAGGTVWMVRHRPTGRPYALKVLYG

NHDDAVRRQITREIAILRTAEHPAIVRCHGMYEQAGELQILLEFMDGGSLEGRRIASEAF

LADVARQVLSGIAYLHRRHIVHRDIKPSNLLIDSGRRVKIADFGVGRILNQTMDPCNSSV

GTIAYMSPERINTDLNDGAYDGYAGDIWSFGLSILEFYLGRFPLGENLGKQGDWAALMCA

ICYSDSPAPPPIASPEFKSFISCCLQKNPARRPSAAQLLQHRFIAGPQPQVLAAPPS*

>BdTR11iMKK5 Brdisv1BdTR11I1007275m.p

MRPAGSLPSPQPGTPGRPRRRPDLTLPMPQRPDVSSSLAVPLPLPPPSSLGLAQPPAAAA

AAAAPPPPPLGELERVRRVGSGAGGTVWMVRHRPTGRCYALKQLYGNHDDAVRRQIAREI

AILRTAEHPAVVRCHGMYERGGELQILLEYMDGGSLDGRRIAAEGFLADVARQVLSGIAY

LHRRHIVHRDIKPSNLLIDSARRVKIADFGVGRILNQTMDPCNSSVGTIAYMSPERINTD

LNDGAYDGYAGDIWSFGLSILEFYLGRFPFGENLGKQGDWAALMVAICYNDPPEPSAAAS

PEFRGFISCCLQKNPAKRLSAAQLLQHPFVAGPQPLPLAAPPS*

>BdTR11iMKK6 Brdisv1BdTR11I1012108m.p

MRGKKPLKELKLSVPAQETSVDKFLTASGTFKDGELRLNQRGLRLISEEENGDEHQSTNM

KVEDVQLSMDDLEMIQVIGKGSGGVVQLVQHKWVGTFYALKGIQMNIQEAVRKQIVQELK

INQATQSPHIVSCHQSFYHNGVIYLVLEYMDRGSLADIIKQVKTILEPYLAVLCKQVLEG

LLYLHHERHVIHRDIKPSNLLVNHKGEVKITDFGVSAVLASSIGQRDTFVGTYNYMAPER

ISGSSYDYKSDVWSLGLVILECAIGRFPYTPSEGEGWLSFYELLEAIVDQPPPGAPADQF

SPEFCSFISACIQKDPAERMSASELLNHAFIKKFEGKDLDLRILVESLEPPMNVPE*

>BdTR11iMKK10-1 Brdisv1BdTR11I1001760m.p

MALLREKRLQLSLHVPTRAADAQEAGLHRRPNPAAALPLAATTPAARSSQFRVADFEKLA

VLGRGNGGTVYKVRHRETCELYALKVQHCNGDATAEAEVLSRTASPFVVRCHSVLPAAAS

GDVAMLLELVDGGSLDSIVKSRSRGQAEAFSQFPEEALAEVAAQALSGLAYLHARRIVHL

DVKPGNLLVSTGGEVKIADFGIARVLPRAGGDDVRCTAYAGTAAYMSPERFDPEAHGGHY

DPYAADVWGLGVTVLELLMGRYPLLPAGQRPSWAALMCAICFGETPALSDGEASAELRGF

VAACLHKDYRRRASVAELLAHPFVAGRDVAASKCALRKLVTEASMSP*

>BdTR11iMKK10-2 Brdisv1BdTR11I1011205m.p

MALVRQRRQLPHLTLPLDHFALRPPPVPAPAPTVAASTSSEAAGLRLSDFERISLLGQGN

GGTVYKARHRRAAAQPPVALKLFVAGDPSAAREAEILRLAADAPHVVRLHAVVPSSPPAA

GAEQPPPAALALELLPGGSLAGLLRRLGRSMGERPIAAVARQALLGLDALHALRVVHRDL

KPSNLLLGSHGEVKIADFGAGKVLRRRLDPCASYVGTAAYMSPERFDPEAYSGDYDPYAA

DVWSLGLAILELYLGHFPLLPAGQRPDWAALMCAICFGDAPEAPAAASEEFRDFVARCLE

KKAGQRASVAELLEHPFIAERDAEEAKRALAALVAEAELGDL*

>BdTR11iMKK10-3 Brdisv1BdTR11I1001659m.p

MALLREKRLQLSLHVPTRAAEALDAVHRRPNPVAATLAASTPAAARSSQFRLADFDKLTV

LGRGNGGTVYKVRHRETCELYALKVQHCNGDPTAAAEAEVLSRTASPFIVRCHSVLPGAA

SGDVAMLLELVDGGSLDSIVKSRRAHAFPFPEEALAEVAAQALSGLAYLHARRIVHLDIK

PGNLLVSTGGEVKVADFGIAKVLPRAGADDARCKSYAGTAAYMSPERFDPEAHGGHYDAY

AADVWGLGVTVLELLMGRYPLLPAGQRPSWPALMCAICFGETPVLSDGEASAELRGFVAA

CLRKDHTKRASVAELLAHPFVAGRDVATSKCALRKLVTEASTSP*

>BdTR11iMKK10-4 Brdisv1BdTR11I1001643m.p

MASAKERRLPQLHLKLDVPTCAFRCAAPAPAPATAATPATSASRPPHGEFRLNDFDRLSV

LGRGNGGSVYKVSHRRTSALYALKIIHGAHARPGAADEEADIVRRVVDSPNVVRCHSVLP

TASGDAAALLLELVDGGSLDSLVGGGGFLPEAAVADVAAQALSGLAHLRARRVAHRDIKP

ANLLLSAAGEVKIADFGIAKVVVSGAGGRARALAYEGTVAYMSPERFDSERHADADPYAA

DVWGLGVTLLELLMGRYPLLPAGQKPTWAALMCAICFGELPALPEGAASLEFRGFVAACL

RKDHRKRASVVELLAHPFVAGRDVAASRRALREAIERRCSC*

>BdTR11iMKK10-5 Brdisv1BdTR11I1001645m.p

MALTVRQRRLPQLHISLDLPSCSFRCPNPPVAATASTSGEFRASDFERLAVLGRGNGGTV

YKVAHRRTSAQYALKVLHGGGDPGAAAAEADVLRRAADSPYVVRCHSVFPAASGSGETAL

LLELVDGGSLDSVRRGVGVSVFFPEAALAEVAAQALAGLAHLHARRVVHRDIKPANLLVS

GAGGVKVADFGIAMVLPSRAGGERCAAAYEGTVAYMSPERFDSEGRADADPRGADVWGLG

VTVLELLMGRYPLLPAGQKPTWAALMCAICFGELPALPEGAASTELRGFIAACLRKDHTK

RASVAELIKHPFVAGRNMAASRLALRRLVAGA*

>BdTR12cMKK1 Brdisv1BdTR12c1007536m.p

MRKPGKLALPSHESTIGKFLTQSGTFKDGDLLVNKDGLRIVHNSEEGEAPPIEPLDDHQL

SLDDLDAIKVIGKGSSGIVQLVRHKWTDQFFALKVIQLNIQESIRKQIAQELKISLSTQC

QYVVTCYQCFYVNGVISIVLEYMDGGSLADFLKTVRTIPEAYLAAIFKQVLQGLMYLHHE

KRVIHRDLKPSNILINHRGEVKISDFGVSAIIASSSAQRDTFTGTFNYMAPERISGQKHG

YMSDIWSLGLVMLECATGNFPYPSPDSFYELLEAVVDQPPPSAPTDQFSPEFCSFISACI

QKEATDRSSAQVLSDHPFLSMYDDLNIDLADYFTTAGSPLATFKQIVL*

>BdTR12cMKK3-1 Brdisv1BdTR12c1026819m.p

MGLRYLHEVRHLVHRDIKPANMLVNLKGEAKITDFGVSAGLDNTMAMCATFVGTVTYMSP

ERIRNENYSYAADIWSLGLTILECATGKFPYNVNEGPANLMLQILDDPSPTPPADAYSPE

FCSFVNDCLQKDPDARPTCEQLFGHPFIKRYENAGVDLIAYVKGVVDPTERLKEIAEMLA

VHYYLLFNGSDGLWHHMKTFYMEESTFSFSGNVYVGRNDIFDTLSSIRKKLKGDRPREKI

VHVVEKLHCRANGETGIAIRVSGSLIVGNQFLVCGEGLQAEGMPSVEELSIDIPSKRVGQ

FREQFMMLPGISMGSFHISRQDLYIIQA*

>BdTR12cMKK3-2 Brdisv1BdTR12c1042600m.p

MAGLEELKKKLQPLMFNDPDKDGFSTRVPFPEDTCDSYVVSDGGTINLLSRSFGEYNINE

HGFHKRSAGADESDFGEKAYRCASQDMHIFGPIGNGASSVVQRAIFIPVHRILALKKINI

FEKEKRQQILNEMRTLCEACCYPGLVEFQGAFYMPDSGQISIALEYMDGGSLADVIKVKK

SIPEQVLAHMLQKVLLGLRYLHEVRHLVHRDIKPANMLVNLKGEAKITDFGVSAGLDNTM

AMCATFVGTVTYMSPERIRNENYSYAADIWSLGLTILECATGKFPYNVNEGPANLMLQIL

DDPSPAPPENAFSSEFCSFVNDCLQKDADARPTLL*

>BdTR12cMKK3-3 Brdisv1BdTR12c1029700m.p

MDGGWDGMGQRKRRHRAGIPIPTYPGLAASLEFCFVTAGACHTAPGPGSKIAASLFAALL

FQRPPASTSRAIFFCFVIMAAGLEDLRRRGQPIFFDADGNVMPAPDDDSEVLDGGTINLL

SRSSDEYNINERGFHKRTIRSDDEYSSEKAFRCSCHDMHIFDSVGNGASSVVHRAIYVPV

HRVLALKKINIFEKERRQQILNEIITLSEACCYPGLVEFHGVFYTPDSGEIYFALEYMDG

GSLADIIRVKKFISEPVLSHMLQKVLLALRYLHEVRHLVHRDIKPANLLLNLKGDTKITD

FGVTSGLHDSIDMCATFLGSVTYMSPERIRNESYSYSADIWSLGLTALECATGRYPYDVN

GGEADLMLQILEDPSPTPPHDIYSEEFCSFINACLQKDADARPTCDQLLSHSFIKRYEGP

GVDLSEYNKSVHDPSERLSQIAHMLAVHYYLIFDGGDDQWCHMKTFYQQDSIFSFSGETH

VGKSEIFETLSRIRKMLKGNSPCEKIAHVMEKVYCRSHGEEGMRVRVSGSFIVGNEFVVC

ADGVRAEGMLSIDELSPDILSKQAGHFQEDFFMEPGTALGCYVISKQELHIADT*

>BdTR12cMKK4 Brdisv1BdTR12c1001607m.p

MRPGGPPNARPQQPGTPGRARRRPDLTLPLPQRDLTSLAVPLPLPPPPSSAPSSASSSGG

TVWMVRHRPTGRPYALKVLYGNHDDAVRRQITREIAILRTAEHPAIVRCHGMYEQAGELQ

ILLEFMDGGSLEGRRIASEAFLADVARQVLSGIASLHRRHIVHRDIKPSNLLIDSGRRVK

IADFGVGRILNQTMDPCNSSVGTIAYMSPERINTDLNDGAYDGYAGDIWSFGLSILEFYL

GRFPLGENLGKQGDWAALMCAICYSDSPAPPPIASPEFKSFISCCLQKNPARRPSAAQLL

QHRFIAGPQPQVLAAPPS*

>BdTR12cMKK5 Brdisv1BdTR12c1006838m.p

MRPAGSLPSPQPGTPGRPRRRPDLTLPMPQRPDVSSSLARVRRVGSGAGGTVWMGRHRPT

GRCYALKQLYGNHDDAVRRQIAREIAILRPAEPPAVVRCHGMYERGGELQILLEYMDGGP

PDGRRIAAEGFLADVARQVLSGIAYLHRRHIVHRDIKPSNLLIDSARRVKIADFGVGRIL

NQTMDPCNSSVGTIAYMSPERINTDLNDGAYDGYAGDIWSFGLSILEFYLGRFPFGENLG

KQGDWAALMVAICYNDPPEPPAAASPEFRGFISCCLQKNPAKRLSAAQLLQHPFVAGPQP

LPLAAPPS*

>BdTR12cMKK6 Brdisv1BdTR12c1011459m.p

MRGKKPLKELKLSVPAQETSVDKFLTASGTFKDGELRLNQRGLRLISEEENGDEHQSTNM

KVEDVQLSMDDLEMIQVIGKGSGGVVQLVQHKWVGTFYALKGIQMNIQEAVRKQIVQELK

INQATQSPHIVSCHQSFYHNGVIYLVLEYMDRGSLADIIKQVKTILEPYLAVLCKQVLEG

LLYLHHERHVIHRDIKPSNLLVNHKGEVKITDFGVSAVLASSIGQRDTFVGTYNYMAPER

ISGSSYDYKSDVWSLGLVILECAIGRFPYTPSEGEGWLSFYELLEAIVDQPPPGAPADQF

SPEFCSFISACIQKDPAERMSASELLNHAFIKKFEGKDLDLRILVESLEPPMNVPE*

>BdTR12cMKK10-1 Brdisv1BdTR12c1001730m.p

MALLREKRLQLSLHVPTRAADAQEAGLHRRPNPAAALPLAAPPPAARSSQFRVADFEKLA

VLGRGNGGTVYKVRHRETCELYALKVQHCNGAAPAEAEVLSRTASPFVVRCHSVLPAAAS

GDVAMLLELVDGGALDSIVKSRSRGQAEAFSQFPEEALAEVAAQALSGLAYLHARRIVHL

DVKPGNLLVSTGGEVKIADFGIARVLPRAGGDDVRCTAYAGTAAYMSPERFDPEAHGGHY

DPYAADVWGLGVTVLELLMGRYPLLPAGQRPSWAALMCAICFGETPALSDGEASAELRGF

VAACLHKDYRRRASVAELLAHPFVAGRDVAASKCALRKLVTEASMSP*

>BdTR12cMKK10-2 Brdisv1BdTR12c1010624m.p

MALVRQRRQLPHLTPPLDHFALRPPPVPAPAPPVAASTSSEAAGLRLSDFERISLLGQGN

GGTVYKARHRRAAAQPPVALKLFVAGDPSAAREAEILRLAADAPHVVRLHAVVPSSSPAA

GAGRPPPAALALELLPGGSLAGLLRRLGRSMGERPIAAVARQALPGPDALHALRVVHRDL

KPSNLLLGSHGEVKIADFGAGKVLRRRLDPCASYVGTAAYMSPERFDPEAYSGDYDPYAA

DVWSLGLAILELYLGHFPLLPAGQRPDWAALMCAICFGDAPGAPAAASEEFRDFVARCLE

KKAGQRASVAELLEHPFIAERDAEEAKRALAALVAEAELGDL*

>BdTR12cMKK10-3 Brdisv1BdTR12c1001612m.p

MALLREKRLQLSLHVPTRAAEALDAVHRRPNPVAATLAASTPAAARGSQFRLADFDKLTV

LGRGNGGTVYKVRHRETCELYALKVQHCNGDPTAAAEAEVLSRTASPFIARCHSVLPGAA

SGDVAMLLELVDGGSLDSIVKSRRAHAFPFPEEALAEVAAQALSGLAYLHARRIVHLDIK

PGNLLVSTGGEVKVADFGIAKVLPRAGADDARCKSYAGTAAYMSPERFDPEAHGGHYDAY

AADVWGLGVTVLELLMGRYPLLPAGQRPSWPALMCAICFGETPVLSDGEASAELRGFVAA

CLRKDHTKRASVAELLAHPFVAGRDVATSKCALRKLVTEASTSP*

>BdTR12cMKK10-4 Brdisv1BdTR12c1001599m.p

MASAKERRLPQLHLKLDVPTCAFRCAAPAPAPATAATPATSASRPPHGEFRLNDFDRLSV

LGRGNGGSVYKVSHRRTSALYALKIIHGAHARPGAADEEADIVRRVVDSPNVVRCHSVPP

TASGDAAALLLELVDGGSLDSLVGGGGFLPEAAGADVAAQALSGLAHLRARRVAHRDIKP

ANLLLSAAGEVKIADFGIAKVVVSGAGGRARALAYEGTVAYMSPERFDSERHADAAPYAA

DVWGLGVTLLELLMGRYPLLPAGQKPTWAALMCAICFGELPALPEGAASLEFRGFVAACL

RKDHRKRASVVELLAHPFVAGRDVAASRRALREAIERRCSC*

>BdTR12cMKK10-5 Brdisv1BdTR12c1001601m.p

MALTVRQRRLPQLHISLDLPSCSFRCPNPPVAATASTSGEFRASDFERLAVLGRGNGGTV

YKVAHRRTSAQYALKVLHGGGDPGAAAAEADVLRRAADSPYVVRCHSVFPAASGSGETAL

LLELVDGGSLASVRRGGGGSVFFPEAALAEVAAQALAGLAHLHARRVVHRDIKPANLLVS

GAGGVKVADFGIAMVLPSRAGGERCAAAYEGTVAYMSPERFDSEGRAGADPRGADVWGLG

VTVLELLMGRYPLLPAGQKPTWAALMCAICFGELPALPEGAASTELRGFIAACLRKDHTK

RASVAELIKHPFVAGRNMAASRLALRRLVAGA*

>BdTR13aMKK1 Brdisv1BdTR13a1008594m.p

MRKPGKLALPSHESTIGKFLTQSGTFKDGDLLVNKDGLRIVHNSEEGEAPPIEPLDDHQL

SLDDLDAIKVIGKGSSGIVQLVRHKWTDQFFALKVIQLNIQESIRKQIAQELKISLSTQC

QYVVTCYQCFYVNGVISIVLEYMDGGSLADFLKTVRTIPEAYLAAICKQVLQGLMYLHHE

KRVIHRDLKPSNILINHRGEVKISDFGVSAIIASSSAQRDTFTGTFNYMAPERISGQKHG

YMSDIWSLGLVMLECATGNFPYPSPDSFYELLEAVVDQPPPSAPTDQFSPEFCSFISACI

QKEATDRSSAQVLSDHPFLSMYDDLNIDLADYFTTAGSPLATFKQIVL*

>BdTR13aMKK3-1 Brdisv1BdTR13a1030929m.p

MAGLEELKKKLQPLLFDDPDKDGISTRVPFLEDNCDSYVVSDGGTINLLSRSFGEYNINE

HGFHKRSTGADESDFGEKAYRCASHDMHIFGPIGNGASSVVQRAIFIPVHRILALKKINI

FEKEKRQQILNEMRTLCEASCYPGLVEFQGAFYMPDSGQISIALEYMDGGSLADVIKVKK

SIPEQVLAHMLQKVLLGLRYLHEVRHLVHRDIKPANMLVNLKGEAKITDFGVSAGLDNTM

AMCATFVGTVTYMSPERIRNENYSYAADIWSLGLTILECATGKFPYNVNEGPANLMLQIL

DDPSPTPPADAYSPEFCSFVNDCLQKDPDARPTCEQLFGHPFIKRYENAGVDLIAYVKGV

VDPTERLKEIAEMLAVHYYLLFNGSDGLWHHMKTFYMEESTFSFSGNVYVGRNDIFDTLS

SIRKKLKGDRPREKIVHVVEKLHCRANGETGIAIRVSGSLIVGNQFLVCGEGLQAEGMPS

VEELSIDIPSKRVGQFREQFMMLPGISMGSFHISRQDLYIIQA*

>BdTR13aMKK3-2 Brdisv1BdTR13a1006861m.p

MAGLEELKKKLQPLMFNDPDKDGFSTRVPFPEDTCDSYVVSDGGTINLLSRSFGEYNINE

HGFHKRSAGADESDFGEKAYRCASQDMHIFGPIGNGASSVVQRAIFIPVHRILALKKINI

FEKEKRQQILNEMRTLCEACCYPGLVEFQGAFYMPDSGQISIALEYMDGGSLADVIKVKK

SIPEPVLAHMLQKVLLGLRYLHEVRHLVHRDIKPANMLVNLKGEAKITDFGVSAGLDNTM

AMCATFVGTVTYMSPERIRNENYSYAADIWSLGLTILECATGKFPYNVNEGPANLMLQIL

DDPSPAPPENAFSSEFCSFVNDCLQKDADARPTCEQLLSHPFIKRYENAGVDLAAYVKGV

VNPEERLKQIAEMLAVHYYLLFNGSDGLWHHMKTFYMEDSTFSFSGNLYVGQSDIFDTLS

NIRTKLKGDRPREKIVHVVEKLHCRANEETGIAIRVSGSFIVSNQFLICGEGLQAEGMPS

LEELSIDIPSKRVGQFREQFIMHPGRSMGCYYISRQDLYIIQA*

>BdTR13aMKK3-3 Brdisv1BdTR13a1034167m.p

MDGGWDGMGQRKRRHRAGIPIPTYPGLAASLEFCFVTAGACHTAPGPGSKIAASLFAALL

FQRPPASTSRAIFFCFVIMAAGLEDLRRRVQPIFFDADGNVMPAPDDDSEVLDGGTINLL

SRSSDEYNINERGFHKRTIRSDDEYSSEKAFRCSCHDMHIFDSVGNGASSVVHRAIYVPV

HRVLALKKINIFEKERRQQILNEIITLSEACCYPGLVEFHGVFYTPDSGEIYFALEYMDG

GSLADIIRVKKFISEPVLSHMLQKVLLALRYLHEVRHLVHRDIKPANLLLNLKGDTKITD

FGVTSGLHDSIDMCATFLGSVTYMSPERIRNESYSYSADIWSLGLTALECATGRYPYDVN

GGEADLMLQILEDPSPTPPHDIYSEEFCSFINACLQKDADARPTCDQLLSHSFIKRYEGP

GVDLSEYNKSVHDPSERLSQIAHMLAVHYYLIFDGGDDQWCHMKTFYQQDSIFSFSGETH

VGKSEIFETLSRIRKMLKGNSPCEKIAHVMEKVYCRSHGEEGMRVRVSGSFIVGNEFVVC

ADGVRAEGMLSIDELSPDILSKQAGHFQEDFFMEPGTALGCYVISKQELHIADT*

>BdTR13aMKK4 Brdisv1BdTR13a1040831m.p

MRPGGPPNARPQQPGTPGRARRRPDLTLPLPQRDLTSLAVPLPLPPPPSSAPSSASSSGS

SLSSMGAPTPPNSAGSAPPPPPPLAELERVRRIGSGAGGTVWMVRHRPTGRPYALKVLYG

NHDDAVRRQITREIAILRTAEHPAIVRCHGMYEQAGELQILLEFMDGGSLEGRRIASEAF

LADVARQVLSGIAYLHRRHIVHRDIKPSNLLIDSGRRVKIADFGVGRILNQTMDPCNSSV

GTIAYMSPERINTDLNDGAYDGYAGDIWSFGLSILEFYLGRFPLGENLGKQGDWAALMCA

ICYSDSPAPPPIASPEFKSFISCCLQKNPARRPSAAQLLQHRFIAGPQPQVLAAPPS*

>BdTR13aMKK5 Brdisv1BdTR13a1007813m.p

MRPAGSLPSPQPGTPGRPRRRPDLTLPMPQRPDVSSSLAVPLPLPPPSSLGLAQPPAAAA

AAAAPPPPPLGELERVRRVGSGAGGTVWMVRHRPTGRCYALKQLYGNHDDAVRRQIAREI

AILRTAEHPAVVRCHGMYERGGELQILLEYMDGGSLDGRRIAAEGFLADVARQVLSGIAY

LHRRHIVHRDIKPSNLLIDSARRVKIADFGVGRILNQTMDPCNSSVGTIAYMSPERINTD

LNDGAYDGYAGDIWSFGLSILEFYLGRFPFGENLGKQGDWAALMVAICYNDPPEPSAAAS

PEFRGFISCCLQKNPAKRLSAAQLLQHPFVAGPQPLPLAAPPS*

>BdTR13aMKK6 Brdisv1BdTR13a1013108m.p

MRGKKPLKELKLSVPAQETSVDKFLTASGTFKDGELRLNQRGLRLISEEENGDEHQSTNM

KVEDVQLSMDDLEMIQVIGKGSGGVVQLVQHKWVGTFYALKGIQMNIQEAVRKQIVQELK

INQATQSPHIVSCHQSFYHNGVIYLVLEYMDRGSLADIIKQVKTILEPYLAVLCKQVLEG

LLYLHHERHVIHRDIKPSNLLVNHKGEVKITDFGVSAVLASSIGQRDTFVGTYNYMAPER

ISGSSYDYKSDVWSLGLVILECAIGRFPYTPSEGEGWLSFYELLEAIVDQPPPGAPADQF

SPEFCSFISACIQKDPAERMSASELLNHAFIKKFEGKDLDLRILVESLEPPMNVPE*

>BdTR13aMKK10-1 Brdisv1BdTR13a1001801m.p

MALLREKRLQLSLHVPTRAADAQEAGLHRRPNPAAALPLAATTPAARSSQFRVADFEKLA

VLGRGNGGTVYKVRHRETCELYALKVQHCNGDATAEAEVLSRTASPFVVRCHSVLPAAAS

GDVAMLLELVDGGSLDSIVKSRSRGQAEAFSQFPEEALAEVAAQALSGLAYLHARRIVHL

DVKPGNLLVSTGGEVKIADFGIARVLPRAGGDDVRCTAYAGTAAYMSPERFDPEAHGGHY

DPYAADVWGLGVTVLELLMGRYPLLPAGQRPSWAALMCAICFGETPALSDGEASAELRGF

VAACLHKDYRRRASVAELLAHPFVAGRDVAASKCALRKLVTEASMSP*

>BdTR13aMKK10-2 Brdisv1BdTR13a1012168m.p

MALVRQRRQLPHLTLPLDHFALRPPPVPAPAPTVAASTSSEAAGLRLSDFERISLLGQGN

GGTVYKARHRRAAAQPPVALKLFVAGDPSAAREAEILRLAADAPHVVRLHAVVPSSSPAA

GAEQPPPAALALELLPGGSLAGLLRRLGRSMGERPIAAVARQALLGLDALHALRVVHRDL

KPSNLLLGSHGEVKIADFGAGKVLRRRLDPCASYVGTAAYMSPERFDPEAYSGDYDPYAA

DVWSLGLAILELYLGHFPLLPAGQRPDWAALMCAICFGDAPEAPAAASEEFRDFVARCLE

KKAGQRASVAELLEHPFIAERDAEEAKRALAALVAEAELGDL*

>BdTR13aMKK10-3 Brdisv1BdTR13a1001694m.p

MALLREKRLQLSLHVPTRAAEALDAVHRRPNPVAATLAASTPAAARSSQFRLADFDKLTV

LGRGNGGTVYKVRHRETCELYALKVQHCNGDPTAAAEAEVLSRTASPFIVRCHSVLPGAA

SGDVAMLLELVDGGSLDSIVKSRRAHAFPFPEEALAEVAAQALSGLAYLHARRIVHLDIK

PGNLLVSTGGEVKVADFGIAKVLPRAGADDARCKSYAGTAAYMSPERFDPEAHGGHYDAY

AADVWGLGVTVLELLMGRYPLLPAGQRPSWPALMCAICFGETPVLSDGEASAELRGFVAA

CLRKDHTKRASVAELLAHPFVAGRDVATSKCALRKLVTEASTSP*

>BdTR13aMKK10-4 Brdisv1BdTR13a1001691m.p

MASAKERRLPQLHLKLDVPTCAFRCAAPAPAPATAATPATSASRPPHGEFRLNDFDRLSV

LGRGNGGSVYKVSHRRTSALYALKIIHGAHARPGAADEEADIVRRVVDSPNVVRCHSVLP

TASGDAAALLLELVDGGSLDSLVGGGGFLPEAAVADVAAQALSGLAHLRARRVAHRDIKP

ANLLLSAAGEVKIADFGIAKVVVSGAGGRARALAYEGTVAYMSPERFDSERHADADPYAA

DVWGLGVTLLELLMGRYPLLPAGQKPTWAALMCAICFGELPALPEGAASLEFRGFVAACL

RKDHRKRASVVELLAHPFVAGRDVAASRRALREAIERRCSC*

>BdTR13aMKK10-5 Brdisv1BdTR13a1001693m.p

MALTVRQRRLPQLHISLDLPSCSFRCPNPPVAATASTSGEFRASDFERLAVLGRGNGGTV

YKVAHRRTSAQYALKVLHGGGDPGAAAAEADVLRRAADSPYVVRCHSVFPAASGSGETAL

LLELVDGGSLDSVRRGVGVSVFFPEAALAEVAAQALAGLAHLHARRVVHRDIKPANLLVS

GAGGVKVADFGIAMVLPSRAGGERCAAAYEGTVAYMSPERFDSEGRADADPRGADVWGLG

VTVLELLMGRYPLLPAGQKPTWAALMCAICFGELPALPEGAASTELRGFIAACLRKDHTK

RASVAELIKHPFVAGRNMAASRLALRRLVAGA*

>BdTR13cMKK1 Brdisv1BdTR13C1007320m.p

MRKPGKLALPSHESTIGKFLTQSGTFKDGDLLVNKDGLRIVHNSEEGEAPPIEPLDDHQL

SLDDLDAIKVIGKGSSGIVQLVRHKWTDQFFALKVIQLNIQESIRKQIAQELKISLSTQC

QYVVTCYQCFYVNGVISIVLEYMDGGSLADFLKTVRTIPEAYLAAICKQVLQGLMYLHHE

KRVIHRDLKPSNILINHRGEVKISDFGVSAIIASSSAQRDTFTGTFNYMAPERISGQKHG

YMSDIWSLGLVMLECATGNFPYPSPDSFYELLEAVVDQPPPSAPTDQFSPEFCSFISACI

QKEATDRSSAQVLSDHPFLSMYDDLNIDLADYFTTAGSPLATFKQIVL*

>BdTR13cMKK3-1 Brdisv1BdTR13C1026262m.p

MAGLEELKKKLQPLLFDDPDKDGISTRVPFLEDNCDSYVVSDGGTINLLSRSFGEYNINE

HGFHKRSTGADESDFGEKAYRCASHDMHIFGPIGNGASSVVQRAIFIPVHRILALKKINI

FEKEKRQQILNEMRTLCEASCYPGLVEFQGAFYMPDSGQISIALEYMDGGSLADVIKVKK

SIPEQVLAHMLQKVLLGLRYLHEVRHLVHRDIKPANMLVNLKGEAKITDFGVSAGLDNTM

AMCATFVGTVTYMSPERIRNENYSYAADIWSLGLTILECATGKFPYNVNEGPANLMLQIL

DDPSPTPPADAYSPEFCSFVNDCLQKDPDARPTCEQLFGHPFIKRYENAGVDLIAYVKGV

VDPTERLKEIAEMLAVHYYLLFNGSDGLWHHMKTFYMEESTFSFSGNVYVGRNDIFDTLS

SIRKKLKGDRPREKIVHVVEKLHCRANGETGIAIRVSGSLIVGNQFLVCGEGLQAEGMPS

VEELSIDIPSKRVGQFREQFMMLPGISMGSFHISRQDLYIIQA*

>BdTR13cMKK3-2 Brdisv1BdTR13C1039819m.p

MAGLEELKKKLQPLMFNDPDKDGFSTRVPFPEDTCDSYVVSDGGTINLLSRSFGEYNINE

HGFHKRSAGADESDFGEKAYRCASQDMHIFGPIGNGASSVVQRAIFIPVHRILALKKINI

FEKEKRQQILNEMRTLCEACCYPGLVEFQGAFYMPDSGQISIALEYMDGGSLADVIKVKK

SIPEPVLAHMLQKVLLGLRYLHEVRHLVHRDIKPANMLVNLKGEAKITDFGVSAGLDNTM

AMCATFVGTVTYMSPERIRNENYSYAADIWSLGLTILECATGKFPYNVNEGPANLMLQIL

DDPSPAPPENAFSSEFCSFVNDCLQKDADARPTCEQLLSHPFIKRYENAGVDLAAYVKGV

VNPEERLKQIAEMLAVHYYLLFNGSDGLWHHMKTFYMEDSTFSFSGNLYVGQSDIFDTLS

NIRTKLKGDRPREKIVHVVEKLHCRANEETGIAIRVSGSFIVSNQFLICGEGLQAEGMPS

LEELSIDIPSKRVGQFREQFIMHPGRSMGCYYISRQDLYIIQA*

>BdTR13cMKK3-3 Brdisv1BdTR13C1029001m.p

MDGGWDGMGQRKRRHRAGIPIPTYPGLAASLEFCFVTAGACHTAPGPGSKIAASLFAALL

FQRPPASTSRAIFFCFVIMAAGLEDLRRRGQPIFFDADGNVMPAPDDDSEVLDGGTINLL

SRSSDEYNINERGFHKRTIRSDDEYSSEKAFRCSCHDMHIFDSVGNGASSVVHRAIYVPV

HRVLALKKINIFEKERRQQILNEIITLSEACCYPGLVEFHGVFYTPDSGEIYFALEYMDG

GSLADIIRVKKFISEPVLSHMLQKVLLALRYLHEVRHLVHRDIKPANLLLNLKGDTKITD

FGVTSGLHDSIDMCATFLGSVTYMSPERIRNESYSYSADIWSLGLTALECATGRYPYDVN

GGEADLMLQILEDPSPTPPHDIYSEEFCSFINACLQKDADARPTCDQLLSHSFIKRYEGP

GVDLSEYNKSVHDPSERLSQIAHMLAVHYYLIFDGGDDQWCHMKTFYQQDSIFSFSGETH

VGKSEIFETLSRIRKMLKGNSPCEKIAHVMEKVYCRSHGEEGMRVRVSGSFIVGNEFVVC

ADGVRAEGMLSIDELSPDILSKQAGHFQEDFFMEPGTALGCYVISKQELHIADT*

>BdTR13cMKK4 Brdisv1BdTR13C1043199m.p

MRPGGPPNARPQQPGTPGRARRRPDLTLPLPQRDLTSLAVPLPLPPPPSSAPSSASSSGS

SLSSMGAPTPPNSAGSAPPPPPPLAELERARRIGSGAGGTVWMVRHRPTGRPYALKVLYG

NHDDAVRRQITREIAILRTAEHPAIVRCHGMYEQAGELQILLEFMDGGSLEGRRIASEAF

LADVARQLLLEFVQTWSMLQHADILLLPSVPDTIRWTITADGCYSARPAYRLHFEGHIRS

NHERNVWRVWAPLKLKFFAWLLLHDRLWCADRLQRRGWPNDYFCALCRRNLETSHHLFVE

SPGARQIWCEVALWPNCFGITTAICHVSVSIDSFHERMISATQSKHRQGIKSMFILVYWA

IWRESNSGVFNDKEIPFCQICYFIKDEARE*

>BdTR13cMKK5 Brdisv1BdTR13C1006669m.p

MRPAGSLPSPQPGTPGRPRRRPDLTLPMPQRPDVSSSLALERVRRVGSGAGGTVWMGRHR

PTGRCYALKQLYGNHDDAVRRQIAREIAILRTAEHPAVVRCHGMYERGGELQILLEYMDG

GPPDARRIAAEGFLADVARQVLSGIASPPRRHIVHRDIKPSNLLIDSARRVKIADFGVGR

ILNQTMDPCNSSVGTIAYMSPERINTDLNDGAYDGYAGDIWSFGLSILEFYLGRFPFGEN

LGKQGDWAALMVAICYNDPPEPSAAASPEFRGFISCCLQKNPAKRLSAAQLLQHPFVAGP

QPLPLAAPPS*

>BdTR13cMKK6 Brdisv1BdTR13C1011139m.p

MRGKKPLKELKLSVPAQETSVDKFLTASGTFKDGELRLNQRGLRLISEEENGDEHQSTNM

KVEDVQLSMDDLEMIQVIGKGSGGVVQLVQHKWVGTFYALKGIQMNIQEAVRKQIVQELK

INQATQSPHIVSCHQSFYHNGVIYLVLEYMDRGSLADIIKQVKTILEPYLAVLCKQVLEG

LLYLHHERHVIHRDIKPSNLLVNHKGEVKITDFGVSAVLASSIGQRDTFVGTYNYMAPER

ISGSSYDYKSDVWSLGLVILECAIGRFPYTPSEGEGWLSFYELLEAIVDQPPPGAPADQF

SPEFCSFISACIQKDPAERMSASELLNHAFIKKFEGKDLDLRILVESLEPPMNVPE*

>BdTR13cMKK10-1 Brdisv1BdTR13C1001614m.p

MALLREKRLQLSLHVPTRAADAQEAGLHRRPNPAAALPLAAPPPAARWSQFRVADFEKLA

VLGRGNGGTVYKVRHRETCELYALKVQHCNGDATAEAEVLSRTASPFVVRCHSVLPAAAS

GDVAMLLELVDGGSLDSIVKSRSRGQAEAFSQFPEEALAEVAAQALSGLAYLHARRIVHL

DVKPGNLLVSTGGEVKIADFGIARVLPRAGGDDVRCTAYAGTAAYMSPERFDPEAHGGHY

DPYAADVWGLGVTVLELLMGRYPLLPAGQRPSWAALMCAICFGETPAPSDGEASAELRGF

VAACLHKDYRRRASVAELLAHPFVAGRDVAASKCALRKLVTEASMSP*

>BdTR13cMKK10-2 Brdisv1BdTR13C1010293m.p

MALVRQRRQLPHLTLPLDHFALRPPPVPAPAPTVAASTSSEAAGLRLSDFERISLLGQGN

GGTVYKARHRRAAAQPPVALKLFVAGDPSAAREAEILRLAADAPHVVRLHAVVPSSSPAA

GAEQPPPAALALELLPGGSLAGLLRRLGRSMGERPIAAVARQALLGLDALHALRVVHRDL

KPSNLLLGSHGEVKIADFGAGKVLRRRLDPCASYVGTAAYMSPERFAPEAYSGDYDPYAA

DVWSLGLAILELYLGHFPLLPAGQRPDWAALMCAICFGDAPEAPAAASEEFRDFVARCLE

KKAGQRASVAELLEHPFIAERDAEEAKRALAALVAEAELGDL*

>BdTR13cMKK10-3 Brdisv1BdTR13C1001511m.p

MAMPKSATLTPPAPLTRSQFRLADFDKLTVLGRGNGGTVYKVRHRETCELYALKVQHCNG

DPTAAAEAEVLSRTASPFIVRCHSVLPGAASGDVAMLLELVDGGSLDSIVKSRRAHAFPF

PEEALAEVAAQALSGLAYLHARRIVHLDIKPGNLLVSTGGEVKVADFGIAKVLPRAGADD

ARCKSYAGTAAYMSPERFDPEAHGGHYDAYAADVWGLGVTVLELLMGRYPLLPAGQRPSW

PALMCAICFGETPVLSDGEASAELRGFVAACLRKDHTKRASVAELLAHPFVAGRDVATSK

CALRKLVTEASTSP*

>BdTR13cMKK10-4 Brdisv1BdTR13C1001502m.p

MASAKERRLPQLHLKLDVPPCAFRCAAPAPAPATAATPATSASRPPHGEFRLNDFDRLSV

LGRGNGGSVYKVSHRRTSALYALKIIHGAHARPGAADEEADIVRRVVDSPNVVRCHSVLP

TASGDAAALLLELVDGGSLDSLVGGGGFLPEAAVGDVAGQALSGLAHLRARRVAHRDIKP

ANLLLSAAGEVKIADFGIAKVVVSGAGGRARALAYEGTVAYMSPERFDSERHADADPYAA

DVWGLGVTLLELLMGRYPLLPAGQKPTWAALMCAICFGELPALPEGAASLEFRGFVAACL

RKDHRKRASVVELLAHPFVAGRDVAASRRALREAIERRCSC*

>BdTR13cMKK10-5 Brdisv1BdTR13C1001510m.p

MALTVRQRRLPQLHISLDLPSCSFRCPTPPVAATASTSGEFRASDFERLAVLGRGNGGTV

YKVAHRRTSAQYALKVLHGGGDPGAAAAEADVLRRAAASPYVVRCHSVFPAASGSGETAL

LLELVDGGSLDSARRGVGVKHLVTKFLHIYTSEICTFAHLKHLHIHTSTFAHLTILALAQ

DKRKSGGERQASHGARGLCLPWQRRSRG*

>BdTR2bMKK1 Brdisv1BdTR2B1007913m.p

MRKPGKLALPSHESTIGKFLTQSGTFKDGDLLVNKDGLRIVHNSEEGEAPPIEPLDDHQL

SLDDLDAIKVIGKGSSGIVQLVRHKWTDQFFALKVIQLNIQESIRKQIAQELKISLSTQC

QYVVTCYQCFYVNGVISIVLEYMDGGSLADFLKTVRTIPEAYLAAIFKQVLQGLMYLHHE

KRVIHRDLKPSNILINHRGEVKISDFGVSAIIASSSAQRDTFTGTFNYMAPERISGQKHG

YMSDIWSLGLVMLECATGNFPYPSPDSFYELLEAVVDQPPPSAPTDQFSPEFCSFISACI

QKEATDRSSAQVLSDHPFLSMYDDLNIDLADYFTTAGSPLATFKQIVL*

>BdTR2bMKK3-1 Brdisv1BdTR2B1037684m.p

MAGLEELKKKLQPLLFDDPDKDGISTRVPFLEDNCDSYVVSDGGTINLLSRSFGEYNINE

HGFHKRSTGADESDFGEKAYRCASHDMHIFGPIGNGASSVVQRAIFIPVHRILALKKINI

FEKEKRQQILNEMRTLCEASCYPGLVEFQGAFYMPDSGQISIALEYMDGGSLADVIKVKK

SIPEQVLAHMLQKVLLGLRYLHEVRHLVHRDIKPANMLVNLKGEAKITDFGVSAGLDNTM

AMCATFVGTVTYMSPERIRNENYSYAADIWSLGLTILECATGKFPYNVNEGPANLMLQIL

DDPSPTPPADAYSPEFCSFVNDCLQKDPDARPTCEQLFGHPFIKRYENAGVDLIAYVKGV

VDPTERLKEIAEMLAVHYYLLFNGSDGLWHHMKTFYMEESTFSFSGNVYVGRNDIFDTLS

SIRKKLKGDRPREKIVHVVEKLHCRANGETGIAIRVSGSLIVGNQFLVCGEGLQAEGMPS

VEELSIDIPSKRVGQFREQFMMLPGISMGSFHISRQDLYIIQA*

>BdTR2bMKK3-2 Brdisv1BdTR2B1042966m.p

MAGLEELKKKLQPLMFNDPDKDGFSTRVPFPEDTCDSYVVSDGGTINLLSRSFGEYNINE

HGFHKRSAGADESDFGEKAYRCASQDMHIFGPIGNGASSVVQRAIFIPVHRILALKKINI

FEKEKRQQILNEMRTLCEACCYPGLVEFQGAFYMPDSGQISIALEYMDGGSLADVIKVKK

SIPEPVLAHMLQKVLLGLRYLHEVRHLVHRDIKPANMLVNLKGEAKITDFGVSAGLDNTM

AMCATFVGTVTYMSPERIRNENYSYAADIWSLGLTILECATGKFPYNVNEGPANLMLQIL

DDPSPAPPENAFSSEFCSFVNDCLQKDADARPTCEQLLSHPFIKRYENAGVDLAAYVKGV

VNPEERLKQIAEMLAVHYYLLFNGSDGLWHHMKTFYMEDSTFSFSGNLYVGQSDIFDTLS

NIRTKLKGDRPREKIVHVVEKLHCRANEETGIAIRVSGSFIVSNQFLICGEGLQAEGMPS

LEELSIDIPSKRVGQFREQFIMHPGRSMGCYYISRQDLYIIQA*

>BdTR2bMKK3-3 Brdisv1BdTR2B1024627m.p

MDGGWDGMGQRKRRHRAGIPIPTYPGLAASLEFCFVTAGACHTAPGPGSKIAASLFAALL

FQRPPASTSRAIFFCFVIMAAGLEDLRRRGQPIFFDADGNVMPAPDDDSEVLDGGTINLL

SRSSDEYNINERGFHKRTIRSDDEYSSEKAFRCSCHDMHIFDSVGNGASSVVHRAIYVPV

HRVLALKKINIFEKERRQQILNEIITLSEACCYPGLVEFHGVFYTPDSGEIYFALEYMDG

GSLADIIRVKKFISEPVLSHMLQKVLLALRYLHEVRHLVHRDIKPANLLLNLKGDTKITD

FGVTSGLHDSIDMCATFLGSVTYMSPERIRNESYSYSADIWSLGLTALECATGRYPYDVN

GGEADLMLQILEDPSPTPPHDIYSEEFCSFINACLQKDADARPTCDQLLSHSFIKRYEGP

GVDLSEYNKSVHDPSERLSQIAHMLAVHYYLIFDGGDDQWCHMKTFYQQDSIFSFSGETH

VGKSEIFETLSRIRKMLKGNSPCEKIAHVMEKVYCRSHGEEGMRVRVSGSFIVGNEFVVC

ADGVRAEGMLSIDELSPDILSKQAGHFQEDFFMEPGTALGCYVISKQELHIADT*

>BdTR2bMKK4 Brdisv1BdTR2B1030719m.p

MRPGGPPNARPQQPGTPGRARRRPDLPLPLPQRDLTSLAVPLPLPPPPSSAPSSASSSGS

SLSSMGAPTPPNSAGSAPPPPPPLAELERVRRIGSGAGGTVWMVRHRPTGRPYALKVLYG

NHDDAVRRQITREIAILRTAEHPAIVRCHGMYEQAGELQILLEFMDGGSLEGRRIASEAF

LADVARQVLSGIAYLPRRHIVHRDIKPSNLLIDSGRRVKIADFGVGRILNQTMDPCNSSV

GTIAYMSPERINTDLNDGAYDGYAGDIWSFGLSILEFYLGRFPLGENLGKQGDWAALMCA

ICYSDSPAPPPIASPEFKSFISCCLQKNPARRPSAAQLLQHRFIAGPQPQVLAAPPS*

>BdTR2bMKK5 Brdisv1BdTR2B1007209m.p

MRPAGSLPSPQPGTPGRPRRRPDLTLPMPQRPDVSSSLAVPLPLPPPSPLGLAQPPAAAA

AAAAPPPPPLGELERVRRVGSGAGGTVWMVRHRPTGRCYALKQLYGNHDDAVRRQIAREI

AILRTAEHPAVVRCHGMYERGGELQILLEYMDGGSLDGRRIAAEGFLADVARQVLSGIAY

LHRRHIVHRDIKPSNLLIDSARRVKIADFGVGRILNQTMDPCNSSVGTIAYMSPERINTD

LNDGAYDGYAGDIWSFGLSILEFYLGRFPFGENLGKQGDWAALMVAICYNDPPEPSAAAS

PEFRGFISCCLQKNPAKRLSAAQLLQHPFVAGPQPLPLAAPPS*

>BdTR2bMKK6 Brdisv1BdTR2B1012093m.p

MRGKKPLKELKLSVPAQETSVDKFLTASGTFKDGELRLNQRGLRLISEEENGDEHQSTNM

KVEDVQLSMDDLEMIQVIGKGSGGVVQLVQHKWVGTFYALKGIQMNIQEAVRKQIVQELK

INQATQSPHIVSCHQSFYHNGVIYLVLEYMDRGSLADIIKQVKTILEPYLAVLCKQVLEG

LLYLHHERHVIHRDIKPSNLLVNHKGEVKITDFGVSAVLASSIGQRDTFVGTYNYMAPER

ISGSSYDYKSDVWSLGLVILECAIGRFPYTPSEGEGWLSFYELLEAIVDQPPPGAPADQF

SPEFCSFISACIQKDPAERMSASELLNHAFIKKFEGKDLDLRILVESLEPPMNVPE*

>BdTR2bMKK10-1 Brdisv1BdTR2B1001835m.p

MALLREKRLQLSLHVPTRAADAQEAGLHRRPNPAAALPLAAPPPAARSSQFRVADFEKLA

VLGRGNGGTVYKVRHRETCELYALKVQHCNGDATAEAEVLSRTASPFVVRCHSVLPAAAS

GDVAMLLELVDGGSLDSIVKSRSRGQAEAFSQFPEEALAEVAAQALSGLAYLHARRIVHL

DVKPGNLLVSTGGEVKIADFGIARVLPRAGGDDVRCTAYAGTAAYMSPERFDPEAHGGHY

DPYAADVWGLGVTVLELLMGRYPLLPAGQRPSWAALMCAICFGETPALSDGEASAELRGF

VAACLHKDYRRRASVAELLAHPFVAGRDVAASKCALRKLVTEASMSP*

>BdTR2bMKK10-2 Brdisv1BdTR2B1011207m.p

MALVRQRRQLPHLTLPLDHFALRPPPVPAPAPTVAASPSSEAAGLRLSDFERISLLGQGN

GGTVYKARHRRAAAQPPVALKLFVAGAPSAAREAEILRLAADAPHVVRLHAVVPSSSPAA

GAEQPPPAALALELLPGGSLAGLLRRLGRSMGERPIAAVARQALLGLDALHALRVVHRDL

KPSNLLLGSHGEVKIADFGAGKVLRRRLDPCASYVGTAAYMSPERFDPEAYSGDYDPYAA

DVWSLGLAILELYLGHFPLLPAGQRPDWAALMCAICFGDAPEAPAAASEEFRDFVARCLE

KKAGQRASVAELLEHPFIAERDAEEAKRALAALVAEAELGDL*

>BdTR2bMKK10-3 Brdisv1BdTR2B1001724m.p

MALLREKRLQLSLHVPTRAAEALDAVHRRPNPVAATLAASTPAAARSSQFRLADFDKLTV

LGRGNGGTVYKVRHRETCELYALKVQHCNGDPTAAAEAEVLSRTASPFIVRCHSVLPGAA

SGDVAMLLELVDGGSLDSIVKSRRAHAFPFPEEALAEVAAQALSGLAYLHARRIVHLDIK

PGNLLVSTGGEVKVADFGIAKVLPRAGADDARCKSYAGTAAYMSPERFDPEAHGGHYDAY

AADVWGLGVTVLELLMGRYPLLPAGQRPSWPALMCAICFGETPVLSDGEASAELRGFVAA

CLRKDHTKRASVAELLAHPFVAGRDVATSKCALRKLVTEASTSP*

>BdTR2bMKK10-4 Brdisv1BdTR2B1001716m.p

MASAKERRLPQLHLKLDVPTCAFRCAAPAPAPATAATPATSASRPPHGEFRLNDFDRLSV

LGRGNGGSVYKVSHRRTSALYALKIIHGAHARPGAADEEADIVRRVVDSPNVVRCHSVLP

TASGDAAALLLELVDGGSLDSLVGGGGFLPEAAVADVAAQALSGLAPLRARRVAHRDIKP

ANLLLSAAGEVKIADFGIAKVVVSGAGGRARALAYEGTVAYMSPERFDSERHADADPYAA

DVWGLGVTLLELLMGRYPLLPAGQKPTWAALMCAICFGELPALPEGAASLEFRGFVAACL

RKDHRKRASVVELLAHPFVAGRDVAASRRALREAIERRCSC*

>BdTR2bMKK10-5 Brdisv1BdTR2B1001718m.p

MALTVRQRRLPQLHISLDLPSCSFRCPNPPVAATASTSGEFRASDFERLAVLGRGNGGTV

YKVAHRRTSAQYALKVLHGGGDPGAAAAEADVLRRAADSPYVVRCHSVFPAASGSGETAL

LLELVDGGSLDSVRRGVGGSVFFPEAALAEVAAQALAGLAHLHARRVVHRDIKPANLLVS

GAGGGKVADFGIAMVLPSRAGGERCAAAYEGTVAYMSPERFDSEGRAGAAPRGADVWGLG

VTVLELLMGRYPLLPAGQKPTWAALMCAICFGELPALPEGAASTELRGFIAACLRKDHTK

RASVAELIKHPFVAGRNMAASRLALRRLVAGA*

>BdTR1iMKK1 Brdisv1BdTR1i1008615m.p

MRKPGKLALPSHESTIGKFLTQSGTFKDGDLLVNKDGLRIVHNSEEGEAPPIEPLDDHQL

SLDDLDAIKVIGKGSSGIVQLVRHKWTDQFFALKVIQLNIQESIRKQIAQELKISLSTQC

QYVVTCYQCFYVNGVISIVLEYMDGGSLADFLKTVRTIPEAYLAAIFKQVLQGLMYLHHE

KRVIHRDLKPSNILINHRGEVKISDFGVSAIIASSSAQRDTFTGTFNYMAPERISGQKHG

YMSDIWSLGLVMLECATGNFPYPSPDSFYELLEAVVDQPPPSAPTDQFSPEFCSFISACI

QKEATDRSSAQVLSDHPFLSMYDDLNIDLADYFTTAGSPLATFKQIVL*

>BdTR1iMKK3-1 Brdisv1BdTR1i1040694m.p

MAGLEELKKKLQPLLFDDPDKDGISTRVPFLEDNCDSYVVSDGGTINLLSRSFGEYNINE

HGFHKRSTGADESDFGEKAYRCASHDMHIFGPIGNGASSVVQRAIFIPVHRILALKKINI

FEKEKRQQILNEMRTLCEASCYPGLVEFQGAFYMPDSGQISIALEYMDGGSLADVIKVKK

SIPEQVLAHMLQKVLLGLRYLHEVRHLVHRDIKPANMLVNLKGEAKITDFGVSAGLDNTM

AMCATFVGTVTYMSPERIRNENYSYAADIWSLGLTILECATGKFPYNVNEGPANLMLQIL

DDPSPTPPADAYSPEFCSFVNDCLQKDPDARPTCEQLFGHPFIKRYENAGVDLIAYVKGV

VDPTERLKEIAEMLAVHYYLLFNGSDGLWHHMKTFYMEESTFSFSGNVYVGRNDIFDTLS

SIRKKLKGDRPREKIVHVVEKLHCRANGETGIAIRVSGSLIVGNQFLVCGEGLQAEGMPS

VEELSIDIPSKRVGQFREQFMMLPGISMGSFHISRQDLYIIQA*

>BdTR1iMKK3-2 Brdisv1BdTR1i1006974m.p

MAGLEELKKKLQPLMFNDPDKDGFSTRVPFPEDTCDSYVVSDGGTINLLSRSFGEYNINE

HGFHKRSAGADESDFGEKAYRCASQDMHIFGPIGNGASSVVQRAIFIPVHRILALKKINI

FEKEKRQQILNEMRTLCEACCYPGLVEFQGAFYMPDSGQISIALEYMDGGSLADVIKVKK

SIPEPVLAHMLQKVLLGLRYLHEVRHLVHRDIKPANMLVNLKGEAKITDFGVSAGLDNTM

AMCATFVGTVTYMSPERIRNENYSYAADIWSLGLTILECATGKFPYNVNEGPANLMLQIL

DDPSPAPPENAFSSEFCSFVNDCLQKDADARPTCEQLLSHPFIKRYENAGVDLAAYVKGV

VNPEERLKQIAEMLAVHYYLLFNGSDGLWHHMKTFYMEDSTFSFSGNLYVGQSDIFDTLS

NIRTKLKGDRPREKIVHVVEKLHCRANEETGIAIRVSGSFIVSNQFLICGEGLQAEGMPS

LEELSIDIPSKRVGQFREQFIMHPGRSMGCYYISRQDLYIIQA*

>BdTR1iMKK3-3 Brdisv1BdTR1i1026202m.p

MDGGWDGMGQRKRRHRAGIPIPTYPGLAASLEFCFVTAGACHTAPGPGSKIAASLFAALL

FQRPPASTSRAIFFCFVIMAAGLEDLRRRVQPIFFDADGNVMPAPDDDSEVLDGGTINLL

SRSSDEYNINERGFHKRTIRSDDEYSSEKAFRCSCHDMHIFDSVGNGASSVVHRAIYVPV

HRVLALKKINIFEKERRQQILNEIITLSEACCYPGLVEFHGVFYTPDSGEIYFALEYMDG

GSLADIIRVKKFISEPVLSHMLQKVLLALRYLHEVRHLVHRDIKPANLLLNLKGDTKITD

FGVTSGLHDSIDMCATFLGSVTYMSPERIRNESYSYSADIWSLGLTALECATGRYPYDVN

GGEADLMLQILEDPSPTPPHDIYSEEFCSFINACLQKDADARPTCDQLLSHSFIKRYEGP

GVDLSEYNKSVHDPSERLSQIAHMLAVHYYLIFDGGDDQWCHMKTFYQQDSIFSFSGETH

VGKSEIFETLSRIRKMLKGNSPCEKIAHVMEKVYCRSHGEEGMRVRVSGSFIVGNEFVVC

ADGVRAEGMLSIDELSPDILSKQAGHFQEDFFMEPGTALGCYVISKQELHIADT*

>BdTR1iMKK4 Brdisv1BdTR1i1032955m.p

MRPGGPPNARPQQPGTPGRARRRPDLTLPLPQRDLTSLAVPLPLPPPPSSAPSSASSSGS

SLSSMGAPTPPNSAGSAPPPPPPLAELERVRRIGSGAGGTVWMVRHRPTGRPYALKVLYG

NHDDAVRRQITREIAILRTAEHPAIVRCHGMYEQAGELQILLEFMDGGSLEGRRIASEAF

LADVARQVLSGIAYLHRRHIVHRDIKPSNLLIDPGRRVKIADFGVGRILNQTMDPCNSSV

GTIAYMSPERINTDLNDGAYDGYAGDIWSFGLSILEFYLGRFPLGENLGKQGDWAALMCA

ICYSDSPAPPPIASPEFKSFISCCLQKNPARRPSAAQLLQHRFIAGPQPQVLAAPPS*

>BdTR1iMKK5 Brdisv1BdTR1i1007913m.p

MRPAGSLPSPQPGTPGRPRRRPDLTLPMPQRPDVSSSLAVPLPLPPPSSLGLAQPPAAAA

AAAAPPPPPLGELERVRRVGSGAGGTVWMVRHRPTGRCYALKQLYGNHDDAVRRQIAREI

AILRTAEHPAVVRCHGMYERGGELQILLEYMDGGSLDGRRIAAEGFLADVARQVLSGIAY

LHRRHIVHRDIKPSNLLIDSARRVKIADFGVGRILNQTMDPCNSSVGTIAYMSPERINTD

LNDGAYDGYAGDIWSFGLSILEFYLGRFPFGENLGKQGDWAALMVAICYNDPPEPSAAAS

PEFRGFISCCLQKNPAKRLSAAQLLQHPFVAGPQPLPLAAPPS*

>BdTR1iMKK6 Brdisv1BdTR1i1012919m.p

MRGKKPLKELKLSVPAQETSVDKFLTASGTFKDGELRLNQRGLRLISEEENGDEHQSTNM

KVEDVQLSMDDLEMIQVIGKGSGGVVQLVQHKWVGTFYALKGIQMNIQEAVRKQIVQELK

INQATQSPHIVSCHQSFYHNGVIYLVLEYMDRGSLADIIKQVKTILEPYLAVLCKQVLEG

LLYLHHERHVIHRDIKPSNLLVNHKGEVKITDFGVSAVLASSIGQRDTFVGTYNYMAPER

ISGSSYDYKSDVWSLGLVILECAIGRFPYTPSEGEGWLSFYELLEAIVDQPPPGAPADQF

SPEFCSFISACIQKDPAERMSASELLNHAFIKKFEGKDLDLRILVESLEPPMNVPE*

>BdTR1iMKK10-1 Brdisv1BdTR1i1002032m.p

MALLREKRLQLSLHVPTRAADAQEAGLHRRPNPAAALPLAATTPAARSSQFRVADFEKLA

VLGRGNGGTVYKVRHRETCELYALKVQHCNGDATAEAEVLSRTASPFVVRCHSVLPAAAS

GDVAMLLELVDGGSLDSIVKSRSRGQAEAFSQFPEEALAEVAAQALSGLAYLHARRIVHL

DVKPGNLLVSTGGEVKIADFGIARVLPRAGGDDVRCTAYAGTAAYMSPERFDPEAHGGHY

DPYAADVWGLGVTVLELLMGRYPLLPAGQRPSWAALMCAICFGETPALSDGEASAELRGF

VAACLHKDYRRRASVAELLAHPFVAGRDVAASKCALRKLVTEASMSP*

>BdTR1iMKK10-2 Brdisv1BdTR1i1011964m.p

MALVRQRRQLPHLTLPLDHFALRPPPVPAPAPTVAASTSSEAAGLRLSDFERISLLGQGN

GGTVYKARHRRAAAQPPVALKLFVAGDPSAAREAEILRLAADAPHVVRLHAVVPSSSPAA

GAEQPPPAALALELLPGGSLAGLLRRLGRSMGERPIAAVARQALLGLDALHALRVVHRDL

KPSNLLLGSHGEVKIADFGAGKVLRRRLDPCASYVGTAAYMSPERFDPEAYSGDYDPYAA

DVWSLGLAILELYLGHFPLLPAGQRPDWAALMCAICFGDAPEAPAAASEEFRDFVARCLE

KKAGQRASVAELLEHPFIAERDAEEAKRALAALVAEAELGDL*

>BdTR1iMKK10-3 Brdisv1BdTR1i1001931m.p

MALLREKRLQLSLHVPTRAAEALDAVHRRPNPVAATLAASTPAAARASQFRLADFDKLTV

LGRGNGGTVYKVRHRETCELYALKVQHCNGDPTAAAEAEVLSRTASPFIVRCHSVLPGAA

SGDVAMLLELVDGGSLDSIVKSRRAHAFPFPEEALAEVAAQALSGLAYLHARRIVHLDIK

PGNLLVSTGGEVKVADFGIAKVLPRAGADDARCKSYAGTAAYMSPERFDPEAHGGHYDAY

AADVWGLGVTVLELLMGRYPLLPAGQRPSWPALMCAICFGETPVLSDGEASAELRGFVAA

CLRKDHTKRASVAELLAHPFVAGRDVATSKCALRKLVTEASTSP*

>BdTR1iMKK10-4 Brdisv1BdTR1i1001928m.p

MASAKERRLPQLHLKLDVPTCAFRCAAPAPAPATAATPATSASRPPHGEFRLNDFDRLSV

LGRGNGGSVYKVSHRRTSALYALKIIHGAHARPGAADEEADIVRRVVDSPNVVRCHSVLP

TASGDAAALLLELVDGGSLDSLVGGGGFLPEAAVADVAAQALSGLAPLRARRVAHRDIKP

ANLLLSAAGEVKIADFGIAKVVVSGAGGRARALAYEGTVAYMSPERFDSERHADADPYAA

DVWGLGVTLLELLMGRYPLLPAGQKPTWAALMCAICFGELPALPEGAASLEFRGFVAACL

RKDHRKRASVVELLAHPFVAGRDVAASRRALREAIERRCSC*

>BdTR1iMKK10-5 Brdisv1BdTR1i1001930m.p

MALTVRQRRLPQLHISLDLPSCSFRCPNPPVAATASTSGEFRASDFERLAVLGRGNGGTV

YKVAHRRPSAQYALKVLHGGGDPGAAAAEADVLRRAADSPYVVRCHSVFPAASGSGETAL

LLELVDGGSLDSVRRGVGVSVFFPEAALAEVAAQALAGLAHLHARRVVHRDIKPANLLVS

GAGGVKVADFGIAMVLPSRAGGERCAAAYEGTVAYMSPERFDSEGRADADPRGADVWGLG

VTVLELLMGRYPLLPAGQKPTWAALMCAICFGELPALPEGAASTELRGFIAACLRKDHTK

RASVAELIKHPFVAGRNMAASRLALRRLVAGA*

>BdTR2gMKK1 Brdisv1BdTR2G1007476m.p

MRKPGKLALPSHESTIGKFLTQSGTFKDGDLLVNKDGLRIVHNSEEGEAPPIEPLDDHQL

SLDDLDAIKVIGKGSSGIVQLVRHKWTDQFFALKVIQLNIQESIRKQIAQELKISLSTQC

QYVVTCYQCFYVNGVISIVLEYMDGGSLADFLKTVRTIPEAYLAAIFKQVLQGLMYLHHE

KRVIHRDLKPSNILINHRGEVKISDFGVSAIIASSSAQRDTFTGTFNYMAPERISGQKHG

YMSDIWSLGLVMLECATGNFPYPSPDSFYELLEAVVDQPPPSAPTDQFSPEFCSFISACI

QKEATDRSSAQVLSDHPFLSMYDDLNIDLADYFTTAGSPLATFKQIVL*

>BdTR2gMKK3-1 Brdisv1BdTR2G1026533m.p

MAGLEELKKKLQPLLFDDPDKDGISTRVPFLEDNCDSYVVSDGGTINLLSRSFGEYNINE

HGFHKRSTGADESDFGEKAYRCASHDMHIFGPIGNGASSVVQRAIFIPVHRILALKKINI

FEKEKRQQILNEMRTLCEASCYPGLVEFQGAFYMPDSGQISIALEYMDGGSLADVIKVKK

SIPEQVLAHMLQKVLLGLRYLHEVRHLVHRDIKPANMLVNLKGEAKITDFGVSAGLDNTM

AMCATFVGTVTYMSPERIRNENYSYAADIWSLGLTILECATGKFPYNVNEGPANLMLQIL

DDPSPTPPADAYSPEFCSFVNDCLQKDPDARPTCEQLFGHPFIKRYENAGVDLIAYVKGV

VDPTERLKEIAEMLAVHYYLLFNGSDGLWHHMKTFYMEESTFSFSGNVYVGRNDIFDTLS

SIRKKLKGDRPREKIVHVVEKLHCRANGETGIAIRVSGSLIVGNQFLVCGEGLQAEGMPS

VEELSIDIPSKRVGQFREQFMMLPGISMGSFHISRQDLYIIQA*

>BdTR2gMKK3-2 Brdisv1BdTR2G1040261m.p

MAGLEELKKKLQPLMFNDPDKDGFSTRVPFPEDTCDSYVVSDGGTINLLSRSFGEYNINE

HGFHKRSAGADESDFGEKAYRCASQDMHIFGPIGNGASSVVQRAIFIPVHRILALKKINI

FEKEKRQQILNEMRTLCEACCYPGLVEFQGAFYMPDSGQISIALEYMDGGSLADVIKVKK

SIPEPVLAHMLQKVLLGLRYLHEVRHLVHRDIKPANMLVNLKGEAKITDFGVSAGLDNTM

AMCATFVGTVTYMSPERIRNENYSYAADIWSLGLTILECATGKFPYNVNEGPANLMLQIL

DDPSPAPPENAFSSEFCSFVNDCLQKDADARPTCEQLLSHPFIKRYENAGVDLAAYVKGV

VNPEERLKQIAEMLAVHYYLLFNGSDGLWHHMKTFYMEDSTFSFSGNLYVGQSDIFDTLS

NIRTKLKGDRPREKIVHVVEKLHCRANEETGIAIRVSGSFIVSNQFLICGEGLQAEGMPS

LEELSIDIPSKRVGQFREQFIMHPGRSMGCYYISRQDLYIIQA*

>BdTR2gMKK3-3 Brdisv1BdTR2G1029283m.p

MDGGWDGMGQRKRRHRAGIPIPTYPGLAASLEFCFVTAGACHTAPGPGSKIAASLFAALL

FQRPPASTSRAIFFCFVIMAAGLEDLRRRGQPIFFDADGNVMPAPDDDSEVLDGGTINLL

SRSSDEYNINERGFHKRTIRSDDEYSSEKAFRCSCHDMHIFDSVGNGASSVVHRAIYVPV

HRVLALKKINIFEKERRQQILNEIITLSEACCYPGLVEFHGVFYTPDSGEIYFALEYMDG

GSLADIIRVKKFISEPVLSHMLQKVLLALRYLHEVRHLVHRDIKPANLLLNLKGDTKITD

FGVTSGLHDSIDMCATFLGSVTYMSPERIRNESYSYSADIWSLGLTALECATGRYPYDVN

GGEADLMLQILEDPSPTPPHDIYSEEFCSFINACLQKDADARPTCDQLLSHSFIKRYEGP

GVDLSEYNKSVHDPSERLSQIAHMLAVHYYLIFDGGDDQWCHMKTFYQQDSIFSFSGETH

VGKSEIFETLSRIRKMLKGNSPCEKIAHVMEKVYCRSHGEEGMRVRVSGSFIVGNEFVVC

ADGVRAEGMLSIDELSPDILSKQAGHFQEDFFMEPGTALGCYVISKQELHIADT*

>BdTR2gMKK4 Brdisv1BdTR2G1028797m.p

MRPGGPPNARPQQPGPPGRARRRPDLPLPLPQRALTSLAVPLPLPPPPSSAPSSASSSGS

SLSSMGAPTPPNSAGSAPPPPPPLAGLGRVRRIGSGAGGTVWMVRHRPTGRPYALKVLYG

NHDDAVRRQITREIAILRTAEHPAIVRCHGMYEQAGELQILLEFMDGGSLEGRRIASEAF

LADVARQVLSGIAYPHRRHIVHRDIKPSNLLIDSGRRVKIADFGVGRILNQTMDPCNSSV

GTIAYMSPERINTDLNDGAYDGYAGDIWSFGLSILEFYLGRFPLGENLGKQGDWAALMCA

ICYSDSPAPPPIASPEFKSFISCCLQKNPARRPSAAQLLQHRFIAGPQPQVLAAPPS*

>BdTR2gMKK5 Brdisv1BdTR2G1006838m.p

MRPAGSLPSPQPGTPGRPRRRPDLTLPMPQRPDVSSSLAVPLPLPPPSSLGLAQPPAAAA

AAAAPPPPPLGELERVRRVGSGAGGTVWMGRHRPTGRCYALKQLYGNHDDAVRRQIAREI

AILRTAEPPAVVRCHGMYERGGELQILLEYMDGGPPAARRIAAEGFLADVARQVLSGIAY

LHRRHIVHRDIKPSTLLIDSARRVKIADFGVGRILNQTMDPCNSSVGTIAYMSPERINTD

LNDGAYDGYAGDIWSFGLSILEFYLGRFPFGENLGKQGDWAALMVAICYNDPPEPSAAAS

PEFRGFISCCLQKNPAKRLSAAQLLQHPFVAGPQPLPLAAPPS*

>BdTR2gMKK6 Brdisv1BdTR2G1011225m.p

MRGKKPLKELKLSVPAQETSVDKFLTASGTFKDGELRLNQRGLRLISEEENGDEHQSTNM

KVEDVQLSMDDLEMIQVIGKGSGGVVQLVQHKWVGTFYALKGIQMNIQEAVRKQIVQELK

INQATQSPHIVSCHQSFYHNGVIYLVLEYMDRGSLADIIKQVKTILEPYLAVLCKQVLEG

LLYLHHERHVIHRDIKPSNLLVNHKGEVKITDFGVSAVLASSIGQRDTFVGTYNYMAPER

ISGSSYDYKSDVWSLGLVILECAIGRFPYTPSEGEGWLSFYELLEAIVDQPPPGAPADQF

SPEFCSFISACIQKDPAERMSASELLNHAFIKKFEGKDLDLRILVESLEPPMNVPE*

>BdTR2gMKK10-1 Brdisv1BdTR2G1001709m.p

MALLREKRLQLSLHVPTRAADAQEAGLHRRPNPAAALPLAATPPAGRSSQFRVADFEKLA

GLGRGNGGTVYKVRHRETCELYALKVQHCNGDATAEAEVLSRTASPFVVRCHSVLPAAAS

GDVAMLLELVDGGSLDSIVKSRSRGQAEAFSQFPEEALAEVAAQALSGLAYLHARRIVHL

DVKPGNLLVSTGGEVKIADFGIARVLPRAGGDDVRCTAYAGTAAYMSPERFDPEAHGGHY

DPYAADVWGLGVPVLELLMGRYPLLPAGQRPSWAALMCAICFGETPALSDGEASAELRGF

VAACLHKDYRRRASVAELLAHPFVAGRDVAASKCALRKLVTEASMSP*

>BdTR2gMKK10-2 Brdisv1BdTR2G1010379m.p

MALVRQRRQLPHLTLPLDHFALRPPPVPAPAPTVAASTSSEAAGLRLSDFERISLLGQGN

GGTVYKARHRRAAAQPPVALKLFVAGDPSAAREAEILRLAADAPHVVRLHAVVPSSSPAA

GAGQPPPAALALELLPGGSLAGLLRRLGRSMGERPIAAVARQALLGLDALHALRVVHRDL

KPSNLLLGSHGEVKIADFGAGKVLRRRLDPCASYVGTAAYMSPERFDPEAYSGDYDPYAA

DVWSLGLAILELYLGHFPLLPAGQRPDWAALMCAICFGDAPEAPAAASEEFRDFVARCLE

KKAGQRASVAELLEHPFIAERDAEEAKRALAALVAEAELGDL*

>BdTR2gMKK10-3 Brdisv1BdTR2G1001605m.p

MALLREKRLQLSLHVPTRAAEALDAVHRRPNPVAATLAASTPAAAGWSQFRLADFDKLTV

LGRGNGGTVYKVRHRETCELYALKVQHCNGDPTAAAEAEVLSRPASPFIVRCHSVLPGAA

SGDVAMLLELVDGGSLDSIVKSRRAHAFPFPEEALAEVAAQALSGLAYLHARRIVHLDIK

PGNLLVSTGGEVKVADFGIAKVLPRAGADDARCKSYAGTAAYMSPERFDPEAHGGHYDAY

AADVWGLGVTVLELLMGRYPLLPAGQRPSWPALMCAICFGETPVLSDGEASAELRGFVAA

CLRKDHTKRASVAELLAHPFVAGRDVATSKCALRKLVTEASTSP*

>BdTR2gMKK10-4 Brdisv1BdTR2G1001602m.p

MASAKERRLPQLHLKLDVPTCAFRCAAPAPAPATAATPATSASRPPHGEFRLNDFDRLSV

LGRGNGGSVYKVSHRRTSALYALKIIHGAHARPGAADEEADIVRRVVDSPNVVRCHSVLP

TASGDAAALLLELVDGGSLDSLVGGGGFLPEAAGADVAAQALSGLAPLRARRVAHRDIKP

ANLLLSAAGEVKIADFGIAKVVVSGAGGRARALAYEGTVAYMSPERFDSERHADADPYAA

DVWGLGVTLLELLMGRYPLLPAGQKPTWAALMCAICFGELPALPEGAASLEFRGFVAACL

RKDHRKRASVVELLAHPFVAGRDVAASRRALREAIERRCSC*

>BdTR2gMKK10-5 Brdisv1BdTR2G1042966m.p

MALTVRQRRLPQLHISLDLPSCSFRCPNPPVAATASTSGEFRASDFERLAVPGRGNGGTV

YKVAHRRTSAQYALKVLHGGGDPGAAAAEADVLRRAADSPYVVRCHSVFPAASGSGETAL

LLELVDGGSLDSVRRGGGGSVFFPEAALAEVAAQALAGLAHLHARRVVHRDIKPANLLVS

GAGGVKVADFGIAMVLPSRAGGERCAAAYEGTVAYMSPERFDSEGRAGADPRGADVWGLG

VTVLELLMGRYPLLPAGQKPTWAALMCAICFGELPALPEGAASTELRGFIAACLRKDHTK

RASVAELIKHPFVAGRNMAASRLALRRLVAGA*

>BdTR3cMKK1 Brdisv1BdTR3C1008613m.p

MRKPGKLALPSHESTIGKFLTQSGTFKDGDLLVNKDGLRIVHNSEEGEAPPIEPLDDHQL

SLDDLDAIKVIGKGSSGIVQLVRHKWTDQFFALKVIQLNIQESIRKQIAQELKISLSTQC

QYVVTCYQCFYVNGVISIVLEYMDGGSLADFLKTVRTIPEAYLAAICKQVLQGLMYLHHE

KRVIHRDLKPSNILINHRGEVKISDFGVSAIIASSSAQRDTFTGTFNYMAPERISGQKHG

YMSDIWSLGLVMLECATGNFPYPSPDSFYELLEAVVDQPPPSAPTDQFSPEFCSFISACI

QKEATDRSSAQVLSDHPFLSMYDDLNIDLADYFTTAGSPLATFKQIVL*

>BdTR3cMKK3-1 Brdisv1BdTR3C1038340m.p

MAGLEELKKKLQPLLFDDPDKDGISTRVPFLEDNCDSYVVSDGGTINLLSRSFGEYNINE

HGFHKRSTGADESDFGEKAYRCASHDMHIFGPIGNGASSVVQRAIFIPVHRILALKKINI

FEKEKRQQILNEMRTLCEASCYPGLVEFQGAFYMPDSGQISIALEYMDGGSLADVIKVKK

SIPEQVLAHMLQKVLLGLRYLHEVRHLVHRDIKPANMLVNLKGEAKITDFGVSAGLDNTM

AMCATFVGTVTYMSPERIRNENYSYAADIWSLGLTILECATGKFPYNVNEGPANLMLQIL

DDPSPTPPADAYSPEFCSFVNDCLQKDPDARPTCEQLFGHPFIKRYENAGVDLIAYVKGV

VDPTERLKEIAEMLAVHYYLLFNGSDGLWHHMKTFYMEESTFSFSGNVYVGRNDIFDTLS

SIRKKLKGDRPREKIVHVVEKLHCRANGETGIAIRVSGSLIVGNQFLVCGEGLQAEGMPS

VEELSIDIPSKRVGQFREQFMMLPGISMGSFHISRQDLYIIQA*

>BdTR3cMKK3-2 Brdisv1BdTR3C1007023m.p

MAGLEELKKKLQPLMFNDPDKDGFSTRVPFPEDTCDSYVVSDGGTINLLSRSFGEYNINE

HGFHKRSAGADESDFGEKAYRCASQDMHIFGPIGNGASSVVQRAIFIPVHRILALKKINI

FEKEKRQQILNEMRTLCEACCYPGLVEFQGAFYMPDSGQISIALEYMDGGSLADVIKVKK

SIPEPVLAHMLQKVLLGLRYLHEVRHLVHRDIKPANMLVNLKGEAKITDFGVSAGLDNTM

AMCATFVGTVTYMSPERIRNENYSYAADIWSLGLTILECATGKFPYNVNEGPANLMLQIL

DDPSPAPPENAFSSEFCSFVNDCLQKDADARPTCEQLLSHPFIKRYENAGVDLAAYVKGV

VNPEERLKQIAEMLAVHYYLLFNGSDGLWHHMKTFYMEDSTFSFSGNLYVGQSDIFDTLS

NIRTKLKGDRPREKIVHVVEKLHCRANEETGIAIRVSGSFIVSNQFLICGEGLQAEGMPS

LEELSIDIPSKRVGQFREQFIMHPGRSMGCYYISRQDLYIIQA*

>BdTR3cMKK3-3 Brdisv1BdTR3C1025925m.p

MDGGWDGMGQRKRRHRAGIPIPTYPGLAASLEFCFVTAGACHTAPGPGSKIAASLFAALL

FQRPPASTSRAIFFCFVIMAAGLEDLRRRGQPIFFDADGNVMPAPDDDSEVLDGGTINLL

SRSSDEYNINERGFHKRTIRSDDEYSSEKAFRCSCHDMHIFDSVGNGASSVVHRAIYVPV

HRVLALKKINIFEKERRQQILNEIITLSEACCYPGLVEFHGVFYTPDSGEIYFALEYMDG

GSLADIIRVKKFISEPVLSHMLQKVLLALRYLHEVRHLVHRDIKPANLLLNLKGDTKITD

FGVTSGLHDSIDMCATFLGSVTYMSPERIRNESYSYSADIWSLGLTALECATGRYPYDVN

GGEADLMLQILEDPSPTPPHDIYSEEFCSFINACLQKDADARPTCDQLLSHSFIKRYEGP

GVDLSEYNKSVHDPSERLSQIAHMLAVHYYLIFDGGDDQWCHMKTFYQQDSIFSFSGETH

VGKSEIFETLSRIRKMLKGNSPCEKIAHVMEKVYCRSHGEEGMRVRVSGSFIVGNEFVVC

ADGVRAEGMLSIDELSPDILSKQAGHFQEDFFMEPGTALGCYVISKQELHIADT*

>BdTR3cMKK4 Brdisv1BdTR3C1030687m.p

MRPGGPPNARPQQPGTPGRARRRPDLTLPLPQRDLTSLAVPLPLPPPPSSAPSSASSSGS

SLSSMGAPTPPNSAGSAPPPPPPLAERERVRRIGSGAGGTVWMVRHRPTGRPYALKVLYG

NHDDAVRRQITREIAILRPAEHPAIVRCHGMYEQAGELQILLEFMDGGSLEGRRIASEAF

LADVARQVLSGIASPHRRHIVHRDIKPSNLLIDSGRRVKIADFGVGRILNQTMDPCNSSV

GTIAYMSPERINTDLNDGAYDGYAGDIWSFGLSILEFYLGRFPLGENLGKQGDWAALMCA

ICYSDSPAPPPIASPEFKSFISCCLQKNPARRPSAAQLLQHRFIAGPQPQGLAPPPS*

>BdTR3cMKK5 Brdisv1BdTR3C1007787m.p

MRPAGSLPSPQPGTPGRPRRRPDLTLPMPQRPDVSSSLAVPLPLPPPSSLGLAQPPAAAP

AAAAPPPPPLGELERVRRVGSGAGGTVWMVRHRPPGRCYALKQLYGNHDDAVRRQIAREI

AILRTAEHPAVVRCHGMYERGGELQILLEYMDGGSLDGRRIAAEGFLADVARQVLSGIAY

LHRRHIVHRDIKPSNLLIDSARRVKIADFGVGRILNQTMDPCNSSVGTIAYMSPERINTD

LNDGAYDGYAGDIWSFGLSILEFYLGRFPFGENLGKQGDWAALMVAICYNDPPEPPAAAS

PEFRGFISCCLQKNPAKRLSAAQLLQHPFVAGPQPLPLAAPPS*

>BdTR3cMKK6 Brdisv1BdTR3C1012997m.p

MRGKKPLKELKLSVPAQETSVDKFLTASGTFKDGELRLNQRGLRLISEEENGDEHQSTNM

KVEDVQLSMDDLEMIQVIGKGSGGVVQLVQHKWVGTFYALKGIQMNIQEAVRKQIVQELK

INQATQSPHIVSCHQSFYHNGVIYLVLEYMDRGSLADIIKQVKTILEPYLAVLCKQVLEG

LLYLHHERHVIHRDIKPSNLLVNHKGEVKITDFGVSAVLASSIGQRDTFVGTYNYMAPER

ISGSSYDYKSDVWSLGLVILECAIGRFPYTPSEGEGWLSFYELLEAIVDQPPPGAPADQF

SPEFCSFISACIQKDPAERMSASELLNHAFIKKFEGKDLDLRILVESLEPPMNVPE*

>BdTR3cMKK10-1 Brdisv1BdTR3C1001858m.p

MALLREKRLQLSLHVPTRAADAQEAGLHRRPNPAAALPLAATPPAARSSQFRVADFEKLA

VLGRGNGGTVYKVRHRETCELYALKVQHCNGDATAEAEVLSRTASPFVVRCHSVLPAAAS

GDVAMLLELVDGGSLDSIVKSRSRGQAEAFSQFPEEALAEVAAQALSGLAYLHARRIVHL

DVKPGNLLVSTGGEVKIADFGIARVLPRAGGDDVRCTAYAGTAAYMSPERFDPEAHGGHY

DPYAADVWGLGVTVLELLMGRYPLLPAGQRPSWAALMCAICFGETPALSDGEASAELRGF

VAACLHKDYRRRASVAELLAHPFVAGRDVAASKCALRKLVTEASMSP*

>BdTR3cMKK10-2 Brdisv1BdTR3C1012083m.p

MALVRQRRQLPHLTLPLDHFALRPPPVPAPAPTVAASTSSEAAGLRLSDFERISLLGQGN

GGTVYKARHRRAAAQPPVALKLFVAGDPSAAREAEILRLAADAPHVVRLHAVVPSSSPAA

GAEQPPPAALALELLPGGSLAGLLRRLGRSMGERPIAAVARQALLGLDALHALRVVHRDL

KPSNLLLGSHGEVKIADFGAGKVLRRRLDPCASYVGTAAYMSPERFDPEAYSGDYDPYAA

DVWSLGLAILELYLGHFPLLPAGQRPDWAALMCAICFGDAPEAPAAASEEFRDFVARCLE

KKAGQRASVAELLEHPFIAERDAEEAKRALAALVAEAELGDL*

>BdTR3cMKK10-3 Brdisv1BdTR3C1001745m.p

MALLREKRLQLSLHVPTRAAEALDAVHRRPNPVAATLAAATPAAARWSQFRLADFDKLTV

LGRGNGGTVYKVRHRETCELYALKVQHCNGDPTAAAEAEVLSRTASPFIVRCHSVLPGAA

SGDVAMLLELVDGGSLDSIVKSRRAHAFPFPEEALAEVAAQALSGLAYLHARRIVHLDIK

PGNLLVSTGGEVKVADFGIAKVLPRAGADDARCKSYAGTAAYMSPERFDPEAPGGHYDAY

AADVWGLGVTVLELLMGRYPLLPAGQRPSWPALMCAICFGETPVLSDGEASAELRGFVAA

CLRKDHTKRASVAELLAHPFVAGRDVATSKCALRKLVTEASTSP*

>BdTR3cMKK10-4 Brdisv1BdTR3C1001741m.p

MASAKERRLPQLHLKLDVPTCAFRCAAPAPAPATAAPPATSASRPPHGEFRLNDFDRLSV

LGRGNGGSVYKVSHRRTSALYALKIIHGAHARPGAADEEADIVRRVVDSPTVVRCHSGLP

TASGDAAALLLELVDGGSLDSLVGGGGFLPEAAVADVAAQALSGLAHLRARRVAPRDIKP

ANLLLSAAGEVKIADFGIAKVVVSGAGGRARALAYEGTVAYMSPERFDSERHADADPYAA

DVWGLGVTLLELLMGRYPLLPAGQKPTWAALMCAICFGELPALPEGAASLEFRGFVAACL

RKDHRKRASVVELLAHPFVAGRDVAASRRALREAIERRCSC*

>BdTR3cMKK10-5 Brdisv1BdTR3C1001743m.p

MALTVRQRRLPQLHISLDLPSCSFRCPNPPVAATASTSGEFRASDFERLAALGRGNGGTV

YKVAHRRTSAQYALKVLHGGGDPGAAAAEADVLRRAADSPYVVRCHSVFPAASGSGETAL

LLELVDGGSLDSARRGVGVSVFFPEAALAEVAAQALAGLAHLHARRVVPRDIKPANLLVS

GAGGVKVADFGIAMVLPSRAGGERCAAAYEGTVAYMSPERFDSEGRADAAPRGADVWGLG

VTVLELLMGRYPLLPAGQKPTWAALMCAICFGELPALPEGAASTELRGFIAACLRKDHTK

RASVAELIKHPFVAGRNMAASRLALRRLVAGA*

>BdTR5iMKK1 Brdisv1BdTR5I1007104m.p

MRKPGKLALPSHESTIGKFLTQSGTFKDGDLLVNKDGLRIVHNSEEGEAPPIEPLDDHQL

SLDDLDAIKVIGKGSSGIVQLVRHKWTDQFFALKVIQLNIQESIRKQIAQELKISLSTQC

QYVVTCYQCFYVNGVISIVLEYMDGGSLADFLKTVRTIPEAYLAAICKQVLQGLMYLHHE

KRVIHRDLKPSNILINHRGEVKISDFGVSAIIASSSAQRDTFTGTFNYMAPERISGQKHG

YMSDIWSLGLVMLECATGNFPYPSPDSFYELLEAVVDQPPPSAPTDQFSPEFCSFISACI

QKEATDRSSAQVLSDHPFLSMYDDLNIDLADYFTTAGSPLATFKQIVL*

>BdTR5iMKK3-1 Brdisv1BdTR5I1025501m.p

MAGLEELKKKLQPLLFDDPDKDGISTRVPFLEDNCDSYVVSDGGTINLLSRSFGEYNINE

HGFHKRSTGADESDFGEKAYRCASHDMHIFGPIGNGASSVVQRAIFIPVHRILALKKINI

FEKEKRQQILNEMRTLCEASCYPGLVEFQGAFYMPDSGQISIALEYMDGGSLADVIKVKK

SIPEQVLAHMLQKVLLGLRYLHEVRHLVHRDIKPANMLVNLKGEAKITDFGVSAGLDNTM

AMCATFVGTVTYMSPERIRNENYSYAADIWSLGLTILECATGKFPYNVNEGPANLMLQIL

DDPSPTPPADAYSPEFCSFVNDCLQKDPDARPTCEQLFGHPFIKRYENAGVDLIAYVKGV

VDPTERLKEIAEMLAVHYYLLFNGSDGLWHHMKTFYMEESTFSFSGNVYVGRNDIFDTLS

SIRKKLKGDRPREKIVHVVEKLHCRANGETGIAIRVSGSLIVGNQFLVCGEGLQAEGMPS

VEELSIDIPSKRVGQFREQFMMLPGISMGSFHISRQDLYIIQA*

>BdTR5iMKK3-2 Brdisv1BdTR5I1038660m.p

MAGLEELKKKLQPLMFNDPDKDGFSTRVPFPEDTCDSYVVSDGGTINLLSRSFGEYNINE

HGFHKRSAGADESDFGEKAYRCASQDMHIFGPIGNGASSVVQRAIFIPVHRILALKKINI

FEKEKRQQILNEMRTLCEACCYPGLVEFQGAFYMPDSGQISIALEYMDGGSLADVIKVKK

SIPEPVLAHMLQKVLLGLRYLHEVRHLVHRDIKPANMLVNLKGEAKITDFGVSAGLDNTM

AMCATFVGTVTYMSPERIRNENYSYAADIWSLGLTILECATGKFPYNVNEGPANLMLQIL

DDPSPAPPENAFSSEFCSFVNDCLQKDADARPTCEQLLSHPFIKRYENAGVDLAAYVKGV

VNPEERLKQIAEMLAVHYYLLFNGSDGLWHHMKTFYMEDSTFSFSGNLYVGQSDIFDTLS

NIRTKLKGDRPREKIVHVVEKLHCRANEETGIAIRVSGSFIVSNQFLICGEGLQAEGMPS

LEELSIDIPSKRVGQFREQFIMHPGRSMGCYYISRQDLYIIQA*

>BdTR5iMKK3-3 Brdisv1BdTR5I1028137m.p

MDGGWDGMGQRKRRDRAGIPIPTYPGLAASLEFCFVTAGACHTAPGPGSKIAASLFAALL

FQRPPASTSRAIFFCFVIMAAGLEDLRRRGQPIFFDADGNVMPAPDDDSEVLDGGTINLL

SRSSDEYNINERGFHKRTIRSDDEYSSEKAFRCSCHDMHIFDSVGNGASSVVHRAIYVPV

HRVLALKKINIFEKERRQQILNEIITLSEACCYPGLVEFHGVFYTPDSGEIYFALEYMDG

GSLADIIRVKKFISEPVLSHMLQKVLLALRYLHEVRHLVHRDIKPANLLLNLKGDTKITD

FGVTSGLHDSIDMCATFLGSVTYMSPERIRNESYSYSADIWSLGLTALECATGRYPYDVN

GGEADLMLQILEDPSPTPPHDIYSEEFCSFINACLQKDADARPTCDQLLSHSFIKRYEGP

GVDLSEYNKSVHDPSERLSQIAHMLAVHYYLIFDGGDDQWCHMKTFYQQDSIFSFSGETH

VGKSEIFETLSRIRKMLKGNSPCEKIAHVMEKVYCRSHGEEGMRVRVSGSFIVGNEFVVC

ADGVRAEGMLSIDELSPDILSKQAGHFQEDFFMEPGTALGCYVISKQELHIADT*

>BdTR5iMKK4 Brdisv1BdTR5I1027632m.p

MRPGGPPNARPQQPGTPGRARRRPDLTLPLPQRDLTSLAVPLPLPPPPSSAPSSASSSGS

SLSSMGAPTPPNSAGSAPPPPPPPAELERVRRIGSGAGGTVWMVRHRPTGRPYALKVLYG

NHDDAVRRQITREIAILRTAEHPAIVRCHGMYEQAGELQILLEFMDGGSLEGRRIASEAF

LADVARQVLSGIAYLHRRHIVHRDIKPSNLLIDSGRRVKIADFGVGRILNQTMDPCNSSV

GTIAYMSPERINTDLNDGAYDGYAGDIWSFGLSILEFYLGRFPLGENLGKQGDWAALMCA

ICYSDSPAPPPIASPEFKSFISCCLQKNPARRPPAAQLLQPRFIAGPQPQVLAAPPS*

>BdTR5iMKK5 Brdisv1BdTR5I1006491m.p

VLSGIAYLHRRHIVHRDIKPSNLLIDSARRVKIADFGVGRILNQTMDPCNSSVGTIAYMS

PERINTDLNDGAYDGYAGDIWSFGLSILEFYLGRFPFGENLGKQGDWAALMVAICYNDPP

EPSAAASPEFRGFISCCLQKNPAKRLSAAQLLQHPFVAGPQPLPLAAPPS*

>BdTR5iMKK6 Brdisv1BdTR5I1010763m.p

MRGKKPLKELKLSVPAQETSVDKFLTASGTFKDGELRLNQRGLRLISEEENGDEHQSTNM

KVEDVQLSMDDLEMIQVIGKGSGGVVQLVQHKWVGTFYALKGIQMNIQEAVRKQIVQELK

INQATQSPHIVSCHQSFYHNGVIYLVLEYMDRGSLADIIKQVKTILEPYLAVLCKQVLEG

LLYLHHERHVIHRDIKPSNLLVNHKGEVKITDFGVSAVLASSIGQRDTFVGTYNYMAPER

ISGSSYDYKSDVWSLGLVILECAIGRFPYTPSEGEGWLSFYELLEAIVDQPPPGAPADQF

SPEFCSFISACIQKDPAERMSASELLNHAFIKKFEGKDLDLRILVESLEPPMNVPE*

>BdTR5iMKK10-1 Brdisv1BdTR5I1001606m.p

MALLREKRLQLSLHVPTRAADAQEAGLHRRPNPAAALPLAAPPPAARSSQFRVADFEKLA

VLGRGNGGTVYKVRHRETCELYALKVQHCNGDATAEAEVLSRTASPFVVRCHSVLPAAAS

GDVAMLLELVDGGSLDSIVKSRSRGQAEAFSQFPEEALAEVAAQALSGLAYLHARRIVHL

DVKPGNLLVSTGGEVKIADFGIARVLPRAGGDDVRCTAYAGTAAYMSPERFDPEAHGGHY

DPYAADVWGLGVTVLELLMGRSPLLPAGQRPSWAALMCAICFGETPALSDGEASAELRGF

VAACLHKDYRRRASVAELLAHPFVAGRDVAASKCALRKLVTEASMSP*

>BdTR5iMKK10-2 Brdisv1BdTR5I1009931m.p

MALVRQRRQLPHLTLPLDHFALRPPPVPAPAPTVAASPSSEAAGLRLSDFERISLLGQGN

GGTVYKARHRRAAAQPPVALKLFVAGDPSAAREAEILRLAADAPHVVRLHAVVPSSSPAA

GAEQPPPAALALELLPGGSLAGLLRRLGRSMGERPIAAVARQALLGLDALHALRVVHRDL

KPSNLLLGSHGEVKIADFGAGKVLRRRLDPCASYVGTAAYMSPERFDPEAYSGDYAPYAA

DVWSLGLAILELYLGHFPLLPAGQRPDWAALMCAICFGDAPEAPAAASEEFRDFVARCLE

KKAGQRASVAELLEHPFIAERDAEEAKRALAALVAEAELGDL*

>BdTR5iMKK10-3 Brdisv1BdTR5I1001520m.p

QFRLADFDKLTVLGRGNGGTVYKVRHRETCELYALKVQHCNGAPTAAAEAELVDGGSLDS

IVKSRRAHAFPFPEEALAEVAAQALSGLAYLHARRIVHLDIKPGNLLVSTGGEVKVADFG

IAKVLPRAGADDARCKSYAGTAAYMSPERFDPEAHGGHYDAYAADVWGLGVTVLELLMGR

YPLLPAGQRPSWPALMCAICFGETPVLSDGEASAELRGFVAACLRKDHTKRASVAELLAH

PFVAGRDVATSKCALRKLVTEASTSP*

>BdTR5iMKK10-4 Brdisv1BdTR5I1001517m.p

MASAKERRLPQLHLKLDVPTCAFRCAAPAPAPATAATPATSASRPPHGEFRLNDFDRLSV

LGRGNGGSVYKVSHRRTSALYALKIIHGAHARPGAADEEADIVRRVVDSPNVARCHSVLP

TASGDAAALLLELVDGGSLDSLVGGGGFLPEAAVADVAAQALSGLAHLRARRVAHRDIKP

ANLLLSAAGEVKIADFGIAKVVVSGAGGRARALAYEGTVAYMSPERFDSERHADADPYAA

DVWGLGVTLLELLMGRYPLLPAGQKPTWAALMCAICFGELPALPEGAASLEFRGFVAACL

RKDHRKRASVVELLAPPFVAGRDVAASRRALREAIERRGT*

>BdTR5iMKK10-5 Brdisv1BdTR5I1001515m.p

MALTVRQRRLPQLHISLDLPSCSFRCPNPPVAATASTSGEVRASDFERLAVLGRGNGGTV

YKVAHRRTSAQYALKVLHGGGDPGAAAAEADVLRRAADSPYVVRCHSVFPAASGSGETAL

LLELVDGGSLDSVRRGGGGSVFFPEAALAEVAAQALAGLAHLHARRVVHRDIKPANLLVS

GAGGVKVADFGIAMVLPSRAGGERCAAAYEGTVAYMSPERFDSEGRADADPRGADVWGLG

VTVLELLMGRYPLLPAGQKPTWAALMCAICFGELPALPEGAASTELRGFIAACLRKDHTK

RASVAELIKHPFVAGRNMAASRLALRRLVAGA*

>BdTR9kMKK1 Brdisv1BdTR9K1007081m.p

MRKPGKLALPSHESTIGKFLTQSGTFKDGDLLVNKDGLRIVHNSEEGEAPPIEPLDDHQL

SLDDLDAIKVIGKGSSGIVQLVRHKWTDQFFALKVIQLNIQESIRKQIAQELKISLSTQC

QYVVTCYQCFYVNGVISIVLEYMDGGSLADFLKTVRTIPEAYLAAIFKQVLQGLMYLHHE

KRVIHRDLKPSNILINHRGEVKISDFGVSAIIASSSAQRDTFTGTFNYMAPERISGQKHG

YMSDIWSLGLVMLECATGNFPYPSPDSFYELLEAVVDQPPPSAPTDQFSPEFCSFISACI

QKEATDRSSAQVLSDHPFLSMYDDLNIDLADYFTTAGSPLATFKQIVL*

>BdTR9kMKK3-1 Brdisv1BdTR9K1025659m.p

MAGLEELKKKLQPLLFDDPDKDGISTRVPFLEDNCDSYVVSDGGTINLLSRSFGEYNINE

HGFHKRSTGADESDFGEKAYRCASHDMHIFGPIGNGASSVVQRAIFIPVHRILALKKINI

FEKEKRQQILNEMRTLCEASCYPGLVEFQGAFYMPDSGQISIALEYMDGGSLADVIKVKK

SIPEQVLAHMLQKVLLGLRYLHEVRHLVHRDIKPANMLVNLKGEAKITDFGVSAGLDNTM

AMCATFVGTVTYMSPERIRNENYSYAADIWSLGLTILECATGKFPYNVNEGPANLMLQIL

DDPSPTPPADAYSPEFCSFVNDCLQKDPDARPTCEQLFGHPFIKRYENAGVDLIAYVKGV

VDPTERLKEIAEMLAVHYYLLFNGSDGLWHHMKTFYMEESTFSFSGNVYVGRNDIFDTLS

SIRKKLKGDRPREKIVHVVEKLHCRANGETGIAIRVSGSLIVGNQFLVCGEGLQAEGMPS

VEELSIDIPSKRVGQFREQFMMLPGISMGSFHISRQDLYIIQA*

>BdTR9kMKK3-2 Brdisv1BdTR9K1038970m.p

MAGLEELKKKLQPLMFNDPDKDGFSTRVPFPEDTCDSYVVSDGGTINLLSRSFGEYNINE

HGFHKRSAGADESDFGEKAYRCASQDMHIFGPIGNGASSVVQRAIFIPVHRILALKKINI

FEKEKRQQILNEMRTLCEACCYPGLVEFQGAFYMPDSGQISIALEYMDGGSLADVIKVKK

SIPEPVLAHMLQKVLLGLRYLHEVRHLVHRDIKPANMLVNLKGEAKITDFGVSAGLDNTM

AMCATFVGTVTYMSPERIRNENYSYAADIWSLGLTILECATGKFPYNVNEGPANLMLQIL

DDPSPAPPENAFSSEFCSFVNDCLQKDADARPTCEQLLSHPFIKRYENAGVDLAAYVKGV

VNPEERLKQIAEMLAVHYYLLFNGSDGLWHHMKTFYMEDSTFSFSGNLYVGQSDIFDTLS

NIRTKLKGDRPREKIVHVVEKLHCRANEETGIAIRVSGSFIVSNQFLICGEGLQAEGMPS

LEELSIDIPSKRVGQFREQFIMHPGRSMGCYYISRQDLYIIQA*

>BdTR9kMKK3-3 Brdisv1BdTR9K1028259m.p

MDGGWDGMGQRKRRHRAGIPIPTYPGLAASLEFCFVTAGACHTAPGPGSKIAASLFAALL

FQRPPASTSRAIFFCFVIMAAGLEDLRRRGQPIFFDADGNVMPAPDDDSEVLDGGTINLL

SRSSDEYNINERGFHKRTIRSDDEYSSEKAFRCSCHDMHIFDSVGNGASSVVHRAIYVPV

HRVLALKKINIFEKERRQQILNEIITLSEACCYPGLVEFHGVFYTPDSGEIYFALEYMDG

GSLADIIRVKKFISEPVLSHMLQKVLLALRYLHEVRHLVHRDIKPANLLLNLKGDTKITD

FGVTSGLHDSIDMCATFLGSVTYMSPERIRNESYSYSADIWSLGLTALECATGRYPYDVN

GGEADLMLQILEDPSPTPPHDIYSEEFCSFINACLQKDADARPTCDQLLSHSFIKRYEGP

GVDLSEYNKSVHDPSERLSQIAHMLAVHYYLIFDGGDDQWCHMKTFYQQDSIFSFSGETH

VGKSEIFETLSRIRKMLKGNSPCEKIAHVMEKVYCRSHGEEGMRVRVSGSFIVGNEFVVC

ADGVRAEGMLSIDELSPDILSKQAGHFQEDFFMEPGTALGCYVISKQELHIADT*

>BdTR9kMKK4 Brdisv1BdTR9K1033576m.p

MVRHRPTGRPYALKVLYGNHDDAVRRQITREIAILRPAEHPAIVRCHGMYEQAGELQILL

EFMDGGSLGGRRIASEAFLADVARQVLSGIAYPPRRHIVHRDIKPSNLLIAPGRRVKIAD

FGVGRILNQTMDPCNSSVGTIAYMSPERINTDLNDGAYDGYAGDIWSFGLSILEFYLGRF

PLGENLGKQGDWAALMCAICYSDSPAPPPIASPEFKSFISCCLQKNPARRPSAAQLLQHR

FIAGPQPQVLAAPPS*

>BdTR9kMKK5 Brdisv1BdTR9K1006454m.p

MRPAGSLPSPQPGTPGRPRRRPDLTLPMPQRPDVSSSLALERVRRVGSGAGGTVWMGRHR

PTGRCYALKQLYGNHDDAVRRQIAREIAILRTAEHPAVVRCHGMYERGGELQILLEYMDG

GPLAARRIAAEGFLADVARQVLSGIAYPHRRHIVHRDIKPSNLLIDSARRVKIADFGVGR

ILNQTMDPCNSSVGTIAYMSPERINTDLNDGAYDGYAGDIWSFGLSILEFYLGRFPFGEN

LGKQGDWAALMVAICYNDPPEPSAAASPEFRGFISCCLQKNPAKRLSAAQLLQPPFVAGP

QPLPLAAPPS*

>BdTR9kMKK6 Brdisv1BdTR9K1010742m.p

MRGKKPLKELKLSVPAQETSVDKFLTASGTFKDGELRLNQRGLRLISEEENGDEHQSTNM

KVEDVQLSMDDLEMIQVIGKGSGGVVQLVQHKWVGTFYALKGIQMNIQEAVRKQIVQELK

INQATQSPHIVSCHQSFYHNGVIYLVLEYMDRGSLADIIKQVKTILEPYLAVLCKQVLEG

LLYLHHERHVIHRDIKPSNLLVNHKGEVKINDFGVSAVLASSIGQRDTFVGTYNYMAPER

ISGSSYDYKSDVWSLGLVILECAIGRFPYTPSEGEGWLSFYELLEAIVDQPPPGAPADQF

SPEFCSFISACIQKDPAERMSASELLNHAFIKKFEGKDLDLRILVESLEPPMNVPE*

>BdTR9kMKK10-1 Brdisv1BdTR9K1001602m.p

MALLREKRLQLSLHVPTRAADAQEAGLHRRPNPAAALPLAATPPAARWSQFRVADFEKLA

VLGRGNGGTVYKVLSRTASPFVVRCHSVLPAAASGDVAMLLELVDGGSLDSIVKSRSRGQ

AEAFSQFPEEALAEVAAQALSGLAYLPARRIVHLDVKPGNLLVSTGGEVKIADFGIARVL

PRAGGDDVRCTAYAGTAAYMSPERFDPEAHGGHYDPYAADVWGLGVTVLELLMGRYPLLP

AGQRPSWAALMCAICFGETPALSDGEASAELRGFVAACLHKDYRRRASVAELLAHPFVAG

RDVAASKCALRKLVTEASMSP*

>BdTR9kMKK10-2 Brdisv1BdTR9K1009908m.p

MALVRQRRQLPHLPLPLDHFALRPPPVPAPAPTVAASTSSEAAGLRLSDFERISLLGQGN

GGTVYKARHRRAAAQPPVALKLFVAGDPSAAREAEILRLAADAPHVVRLHAVVPSSPPAA

GAEQPPPAALALELLPGGSLAGLLRRLGRSMGERPIAAVARQALLGLDALHALRVVHRDL

KPSNLLLGSHGEVKIADFGAGKVLRRRLDPCASSVGTAAYMSPERFDPEAYSGDYDPYAA

DVWSLGLAILELYLGHFPLLPAGQRPDWAALMCAICFGDAPEAPAAASEEFRDFVARCLE

KKAGQRASVAELLEHPFIAERDAEEAKRALAALVAEAELGDL*

>BdTR9kMKK10-3 Brdisv1BdTR9K1001500m.p

MALLREKRLQLSLHVPTRAAEALDAVHRRPNPVAATLAASTPAGARASQFRPADFDKLTV

LGRGNGGTVYKVRHRETCELYALKVQHCNGDPTAAAEAEVLSRPASPFIVRCHSVLPGAA

SGDVAMLLELVDGGSLDSIVKSRRAHAFPFPEEALAEVAAQALSGLAYLHARRIVHLDIK

PGNLLVSTGGEVKVADFGIAKVLPRAGADDARCKSYAGTAAYMSPERFDPEAHGGHYDAY

AADVWGLGVTVLELLMGRYPLLPAGQRPSWPALMCAICFGETPVLSDGEASAELRGFVAA

CLRKDHTKRASVAELLAHPFVAGRDVATSKCALRKLVTEASTSP*

>BdTR9kMKK10-4 Brdisv1BdTR9K1001494m.p

MASAKERRLPQLHLKLDVPTCAFRCAAPAPAPATAATPATSASRPPHGEFRLNDFDRLSV

LGRGNGGSVYKVSHRRTSALYALKIIHGAHARPGAADEEADIVRRVVDSPNVVRCHSVLP

TASGDAAALLLELVDGGSLDSLVGGGGFLPEAAVADVAAQALSGLAHLRARRVAHRDIKP

ANLLLSAAGEVKIADFGIAKVVVSGAGGRARALAYEGTVAYMSPERFDSERHADADPYAA

DVWGLGVTLLELLMGRSPLLPAGQKPTWAALMCAICFGELPALPEGAASLEFRGFVAACL

RKDHRKRASVVELLAHPFVAGRDVAASRRALREAIERRGT*

>BdTR9kMKK10-5 Brdisv1BdTR9K1001498m.p

MAALAEVAAQALAGLAHLHARRVVHRDIKPANLLVSGAGGVKVADFGIAMVLPSRAGGER

CAAAYEGTVAYMSPERFDSEGRADADPRGADVWGLGVTVLELLMGRYPLPPAGQKPTWAA

LMCAICFGELPALPEGAASTELRGFIAACLRKDHTKRASVAELIKHPFVAGRNMAASRLA

LRRLVAGA*

>Bis-1MKK1 Brdisv1Bis-11007849m.p

MRKPGKLALPSHESTIGKFLTQSGTFKDGDLLVNKDGLRIVHNSEEGEAPPIEPLDDHQL

SLDDLDAIKVIGKGSSGIVQLVRHKWTDQFFALKVIQLNIQESIRKQIAQELKISLSTQC

QYVVTCYQCFYVNGVISIVLEYMDGGSLADFLKTVRTIPEAYLAAICKQVLQGLMYLHHE

KRVIHRDLKPSNILINHRGEVKISDFGVSAIIASSSAQRDTFTGTFNYMAPERISGQKHG

YMSDIWSLGLVMLECATGNFPYPSPDSFYELLEAVVDQPPPSAPTDQFSPEFCSFISACI

QKEATDRSSAQVLSDHPFLSMYDDLNIDLADYFTTAGSPLATFKQIVL*

>Bis-1MKK3-1 Brdisv1Bis-11027832m.p

MAGLEELKKKLQPLLFDDPDKDGISTRVPFLEDNCDSYVVSDGGTINLLSRSFGEYNINE

HGFHKRSTGADESDFGEKAYRCASHDMHIFGPIGNGASSVVQRAIFIPVHRILALKKINI

FEKEKRQQILNEMRTLCEASCYPGLVEFQGAFYMPDSGQISIALEYMDGGSLADVIKVKK

SIPEQVLAHMLQKVLLGLRYLHEVRHLVHRDIKPANMLVNLKGEAKITDFGVSAGLDNTM

AMCATFVGTVTYMSPERIRNENYSYAADIWSLGLTILECATGKFPYNVNEGPANLMLQIL

DDPSPTPPADAYSPEFCSFVNDCLQKDPDARPTCEQLFGHPFIKRYENAGVDLIAYVKGV

VDPTERLKEIAEMLAVHYYLLFNGSDGLWHHMKTFYMEESTFSFSGNVYVGRNDIFDTLS

SIRKKLKGDRPREKIVHVVEKLHCRANGETGIAIRVSGSLIVGNQFLVCGEGLQAEGMPS

VEELSIDIPSKRVGQFREQFMMLPGISMGSFHISRQDLYIIQA*

>Bis-1MKK3-2 Brdisv1Bis-11042177m.p

MHIFGPIGNGASSVVQRAIFIPVHRILALKKINIFEKEKRQQILNEMRTLCEACCYPGLV

EFQGAFYMPDSGQISIALEYMDGGSLADVIKVKKSIPEPVLAHMLQKVLLGLRYLHEVRH

LVHRDIKPANMLVNLKGEAKITDFGVSAGLDNTMAMCATFVGTVTYMSPERIRNENYSYA

ADIWSLGLTILECATGKFPYNVNEGPANLMLQILDDPSPAPPENAFSSEFCSFVNDCLQK

DADARPTCEQLLSHPFIKRYENAGVDLAAYVKGVVNPEERLKQIAEMLAVHYYLLFNGSD

GLWHHMKTFYMEDSTFSFSGNLYVGQSDIFDTLSNIRTKLKGDRPREKIVHVVEKLHCRA

NEETGIAIRVSGSFIVSNQFLICGEGLQAEGMPSLEELSIDIPSKRVGQFREQFIMHPGR

SMGCYYISRQDLYIIQA*

>Bis-1MKK3-3 Brdisv1Bis-11030798m.p

MDGGWDGMGQRKRRHRAGIPIPTYPGLAASLEFCFVTAGACHTAPGPGSKIAASLFAALL

FQRPPASTSRAIFFCFVIMAAGLEDLRRRGQPIFFDADGNVMPAPDDDSEVLDGGTINLL

SRSSDEYNINERGFHKRTIRSDDEYSSEKAFRCSCHDMHIFDSVGNGASSVVHRAIYVPV

HRVLALKKINIFEKERRQQILNEIITLSEACCYPGLVEFHGVFYTPDSGEIYFALEYMDG

GSLADIIRVKKFISEPVLSHMLQKVLLALRYLHEERHLVHRDIKPANLLLNLKGDTKITD

FGVTSGLHDSIDMCATFLGSVTYMSPERIRNESYSYSADIWSLGLTALECATGRYPYDVN

GGEADLMLQILEDPSPTPPHDIYSEEFCSFINACLQKDADARPTCDQLLSHSFIKRYEGP

GVDLSEYNKSVHDPSERLSQIAHMLAVHYYLIFDGGDDQWCHMKTFYQQDSIFSFSGETH

VGKSEIFETLSRIRKMLKGNSPCEKIAHVMEKVYCRSHGEEGMRVRVSGSFIVGNEFVVC

ADGVRAEGMLSIDELSPDILSKQAGHFQEDFFMEPGTALGCYVISKQELHIADT*

>Bis-1MKK4 Brdisv1Bis-11036660m.p

MRPGGPPNARPQQPGTPGRARRRPDLPLPLPQRDLTSLAVPLPLPPPPSSAPSSASSSGS

SLSSMGAPTPPNSAGPAPPPPPPLAELERVRRIGSGAGGTVWMVRHRPTGRPYALKVLYG

NHDDAVRRQITREIAILRTAEHPAIVRCHGMYEQAGELQILLEFMAGGSLEGRRIASEAF

LADVARQVLSGIAYLHRRHIVHRDIKPSNLLIDSGRRVKIADFGVGRILNQTMDPGNSSV

GTIAYMSPERINTDLNDGAYDGYAGDIWSFGLSILEFYLGRFPLGENLGKQGDWAALMCA

ICYSDSPAPPPIASPEFKSFISCCLQKNPARRPSAAQLLQHRFIAGPQPQVLAAPPS*

>Bis-1MKK5 Brdisv1Bis-11007171m.p

MRPAGSLPSPQPGTPGRPRRRPDLTLPMPQRPDVSSSLAVPLPLPPPSSLGLAQPPAAAA

AAAAPPPPPLGELERVRRVGSGAGGTVWMVRHRPTGRCYALKQLYGNHDDAGRRQIAREI

AILRTAEPPAVVRCHGMYERGGELQILLEYMDGGSLDGRRIAAEGFLADVARQVLSGIAS

LHRRHIVHRDIKPSNLLIDSARRVKIADFGVGRILNQTMDPCNSSVGTIAYMSPERINTD

LNDGAYDGYAGDIWSFGLSILEFYLGRFPFGENLGKQGDWAALMVAICYNDPPEPSAAAS

PEFRGFISCCLQKNPAKRLSAAQLLQHPFVAGPQPLPLAAPPS*

>Bis-1MKK6 Brdisv1Bis-11011940m.p

MRGKKPLKELKLSVPAQETSVDKFLTASGTFKDGELRLNQRGLRLISEEENGDEHQSTNM

KVEDVQLSMDDLEMIQVIGKGRGGVVQLVQHKWVGTFYALKGIQMNIQEAVRKQIVQELK

INQATQSPHIVSCHQSFYHNGVIYLVLEYMDRGSLADIIKQVKTILEPYLAVLCKQVLEG

LLYLHHERHVIHRDIKPSNLLVNHKGEVKITDFGVSAVLASSIGQRDTFVGTYNYMAPER

ISGSSYDYKSDVWSLGLVILECAIGRFPYTPSEGEGWLSFYELLEAIVDQPPPGAPADQF

SPEFCSFISACIQKDPAERMSASELLNHAFIKKFEGKDLDLRILVESLEPPMNVPE*

>Bis-1MKK10-1 Brdisv1Bis-11001784m.p

MALLREKRLQLSLHVPTRAADAQEAGLHRRPNPAAALPLAATPPAARSSQFRVADFEKLA

VLGRGNGGTVYKVRHRETCELYALKVQHCNGAAPAEAEVLSRTASPFVVRCHSVLPAAAS

GDVAMLLELVDGGSLDSIVKSRSRGQAEAFSQFPEEALAEVAAQALSGLAYLHARRIVHL

DVKPGNLLVSTGGEVKIADFGIARVLPRAGGDDVRCTAYAGTAAYMSPERFDPEAHGGHY

DPYAADVWGLGVTVLELLMGRYPLLPAGQRPSWAALMCAICFGETPALSDGEASAELRGF

VAACLHKDYRRRASVAELLAHPFVAGRDVAASKCALRKLVTEASMSP*

>Bis-1MKK10-2 Brdisv1Bis-11011036m.p

MALVRQRRQLPHLTLPLDHFALRPPPVPAPAPTVAASPSSEAAGLRLSDFERISLLGQGN

GGTVYKARHRRAAAQPPVALKLFVAGDPSAAREAEILRLAADAPHVVRLHAGAPSSPPAA

GAEQPPPAALALELLPGGSLAGLLRRLGRSMGERPIAAVARQALLGLDALHALRVVHRDL

KPSNLLLGSHGEVKIADFGAGKVLRRRLDPCASYVGTAAYMSPERFDPEAYSGDYDPYAA

DVWSLGLAILELYLGHFPLLPAGQRPDWAALMCAICFGDAPEAPAAASEEFRDFVARCLE

KKAGQRASVAELLEHPFIAERDAEEAKRALAALVAEAELGDL*

>Bis-1MKK10-3 Brdisv1Bis-11001672m.p

MALLREKRLQLSLHVPTRAAEALDAVHRRPNPVAATLAASTPAAARSSQFRLADFDKLTV

LGRGNGGTVYKVRHRETCELYALKVQHCNGAPTAAAEAEVLSRTASPFIVRCHSVLPGAA

SGDVAMLLELVDGGSLDSIVKSRRAHAFPFPEEALAEVAAQALSGLAYLHARRIVHLDIK

PGNLLVSTGGEVKVADFGIAKVLPRAGADDARCKSYAGTAAYMSPERFDPEAHGGHYDAY

AADVWGLGVTVLELLMGRYPLLPAGQRPSWPALMCAICFGETPVLSDGEASAELRGFVAA

CLRKDHTKRASVAELLAHPFVAGRDVATSKCALRKLVTEASTSP*

>Bis-1MKK10-4 Brdisv1Bis-11001669m.p

MASAKERRLPQLHLKLDVPTCAFRCAAPAPAPATAATPATSASRPPHGEFRLNDFDRLSV

LGRGNGGSVYKVSHRRTSALYALKIIPGAHARPGAADEEADIVRRVVDSPNVVRCHSVLP

TASGDAAALLLELVDGGSLDSLVGGGGFLPEAAVADVAAQALSGLAHLRARRVAHRDIKP

ANLLLSAAGEVKIADFGIAKVVVSGAGGRARALAYEGTVAYMSPERFDSERHADADPYAA

DVWGLGVTLLELLMGRYPLLPAGQKPTWAALMCAICFGELPALPEGAASLEFRGFVAACL

RKDHRKRASVVELLAHPFVAGRDVAASRRALREAIERRCSC*

>Bis-1MKK10-5 Brdisv1Bis-11001671m.p

MALTVRQRRLPQLHISLDLPSCSFRCPNPPVAATASTSGEFRASDFERLAVPGRGNGGTV

YKVAHRRTSAQYALKVLHGGGDPGAAAAEADVLRRAADSPYVVRCHSVFPAASGSGETAL

LLELVDGGSLDSVRRGVGVSVFFPEAALAEVAAQALAGLAHLHARRVVHRDIKPANLLVS

GAGGVKVADFGIAMVLPSRAGGERCAAAYEGTVAYMSPERFDSEGRADADPRGADVWGLG

VTVLELLMGRYPLLPAGQKPTWAALMCAICFGELPALPEGAASTELRGFIAACLRKDHTK

RASVAELIKHPFVAGRNMAASRLALRRLVAGA*

>Gaz-8MKK1 Brdisv1Gaz-81003865m.p

MSTSLQTSTTSFFVMNMAGXFSPPAASLSLRPSTQTSARRPPPMRKPGKLALPSHESTIG

KFLTQSGTFKDGDLLVNKDGLRIVHNSEEGEAPPIEPLDDHQLSLDDLDAIKVIGKGSSG

IVQLVRHKWTDQFFALKVIQLNIQESIRKQIAQELKISLSTQCQYVVTCYQCFYVNGVIS

IVLEYMDGGSLADFLKTVRTIPEAYLAAIFKQVLQGLMYLHHEKRVIHRDLKPSNILINH

RGEVKISDFGVSAIIASSSAQRDTFTGTFNYMAPERISGQKHGYMSDIWSLGLVMLECAT

GNFPYPSPDSFYELLEAVVDQPPPSAPTDQFSPEFCSFISACIQKEATDRSSAQVLSDHP

FLSMYDDLNIDLADYFTTAGSPLATFKQIVL*

>Gaz-8MKK3-1 Brdisv1Gaz-81025569m.p

MAGLEEVKKKVQPLLFDDPDKDGISTRVPFLEDNCDSYVVSDGGTINLLSRSFGEYNINE

HGFHKRSTGADESDFGEKAYRCASHDMHIFGPIGNGASSVVQRAIFIPVHRILALKKINI

FEKEKRQQILNEMRTLCEASCYPGLVEFQGAFYMPDSGQISIALEYMDGGSLADVIKVKK

SIPEQVLAHMLQKVLLGLRYLHEVRHLVHRDIKPANMLVNLKGEAKITDFGVSAGLDNTM

AMCATFVGTVTYMSPERIRNENYSYAADIWSLGLTILECATGKFPYNVNEGPANLMLQIL

DDPSPTPPADAYSPEFCSFVNDCLQKDPDARPTCEQLFGHPFIKRYENAGVDLIAYVKGV

VDPTERLKEIAEMLAVHYYLLFNGSDGLWHHMKTFYMEESTFSFSGNVYVGRNDIFDTLS

SIRKKLKGDRPREKIVHVVEKLHCRANGETGIAIRVSGSLIVGNQFLVCGEGLQAEGMPS

VEELSIDIPSKRVGQFREQFMMLPGISMGSFHISRQDLYIIQA*

>Gaz-8MKK3-2 Brdisv1Gaz-81021746m.p

MAGLEELKKKLQPLMFNDPDKDGFSTRVPFPEDTCDSYVVSDGGTINLLSRSFGEYNINE

HGFHKRSAGADESDFGEKAYRCASQDMHIFGPIGNGASSVVQRAIFIPVHRILALKKINI

FEKEKRQQILNEMRTLCEACCYPGLVEFQGAFYMPDSGQISIALEYMDGGSLADVIKVKK

SIPEPVLAHMLQKVLLGLRYLHEVRHLVHRDIKPANMLVNLKGEAKITDFGVSAGLDNTM

AMCATFVGTVTYMSPERIRNENYSYAADIWSLGLTILECATGKFPYNVNEGPANLMLQIL

DDPSPAPPENAFSSEFCSFVNDCLQKDADARPTCEQLLSHPFIKRYENAGVDLAAYVKGV

VNPEERLKQIAEMLAVHYYLLFNGSDGLWHHMKTFYMEDSTFSFSGNLYVGQSDIFDTLS

NIRTKLKGDRPREKIVHVVEKLHCRANEETGIAIRVSGSFIVSNQFLICGEGLQAEGMPS

LEELSIDIPSKRVGQFREQFIMHPGRSMGCYYISRQDLYIIQA*

>Gaz-8MKK3-3 Brdisv1Gaz-81015780m.p

MDGGWDGMGQRKRRHRAGIPIPTYPGLAASLEFCFVTAGAGHTAPGPGSKIAASLFAALL

FQRPPASTSRAIFFCFVIMAGGVGGLRRGVQPIFFDADGNVMPAPDDDSEVLDGGTINLL

SRSSDEYNINERGFHKRTIRSDDEYSSEKAFRCSCHDMHIFDSVGNGASSVVHRAIYVPV

HRVLALKKINIFEKERRQQILNEIITLSEACCYPGLVEFHGVFYTPDSGEIYFALEYMDG

GSLADIIRVKKFISEPVLSHMLQKVLLALRYLHEVRHLVHRDIKPANLLLNLKGDTKITD

FGVTSGLHDSIDMCATFLGSVTYMSPERIRNESYSYSADIWSLGLTALECATGRYPYDVN

GGEADLMLQILEDPSPTPPHDIYSEEFCSFINACLQKDADARPTCDQLLSHSFIKRYEGP

GVDLSEYNKSVHDPSERLSQIAHMLAVHYYLIFDGGDDQWCHMKTFYQQDSIFSFSGETH

VGKSEIFETLSRIRKMLKGNSPCEKIAHVMEKVYCRSHGEEGMRVRVSGSFIVGNEFVVC

ADGVRAEGMLSIDDLSPDILSKQAGHFQEDFFMEPGTALGCYVISKQELHIADT*

>Gaz-8MKK6 Brdisv1Gaz-81025547m.p

MRGKKPLKELKLSVPAQETSVDKFLTASGTFKDGELRLNQRGLRLISEEENGDEHQSTNM

KVEDVQLSMDDLEMIQVIGKGSGGVVQLVQHKWVGTFYALKGIQMNIQEAVRKQIVQELK

INQATQSPHIVSCHQSFYHNGVIYLVLEYMDRGSLADIIKQVKTILEPYLAVLCKQVLEG

LLYLHHERHVIHRDIKPSNLLVNHKGEVKITDFGVSAVLASSIGQRDTFVGTYNYMAPER

ISGSSYDYKSDVWSLGLVILECAIGRFPYTPSEGEGWLSFYELLEAIVDQPPPGAPADQF

SPEFCSFISACIQKDPAERMSASELLNHAFIKKFEGKDLDLRILVESLEPPMNVPE*

>Kah-1MKK1 Brdisv1Kah-11006282m.p

MRKPGKLALPSHESTIGKFLTQSGTFKDGDLLVNKDGLRIVHNSEEGEAPPIEPLDDHQL

SLDDLDAIKVIGKGSSGIVQLVRHKWTDQFFALKVIQLNIQESIRKQIAQELKISLSTQC

QYVVTCYQCFYVNGVISIVLEYMDGGSLADFLKTVRTIPEAYLAAICKQVLQGLMYLHHE

KRVIHRDLKPSNILINHRGEVKISDFGVSAIIASSSAQRDTFTGTFNYMAPERISGQKHG

YMSDIWSLGLVMLECATGNFPYPSPDSFYELLEAVVDQPPPSAPTDQFSPEFCSFISACI

QKEATDRSSAQVLSDHPFLSMYDDLNIDLADYFTTAGSPLATFKQIVL*

>Kah-1MKK3-1 Brdisv1Kah-11030282m.p

MAGLEELKKKLQPLLFDDPDKDGISTRVPFLEDNCDSYVVSDGGTINLLSRSFGEYNINE

HGFHKRSTGADESDFGEKAYRCASHDMHIFGPIGNGASSVVQRAIFIPVHRILALKKINI

FEKEKRQQILNEMRTLCEASCYPGLVEFQGAFYMPDSGQISIALEYMDGGSLADVIKVKK

SIPEQVLAHMLQKVLLGLRYLHEVRHLVHRDIKPANMLVNLKGEAKITDFGVSAGLDNTM

AMCATFVGTVTYMSPERIRNENYSYAADIWSLGLTILECATGKFPYNVNEGPANLMLQIL

DDPSPTPPADAYSPEFCSFVNDCLQKDPDARPTCEQLFGHPFIKRYENAGVDLIAYVKGV

VDPTERLKEIAEMLAVHYYLLFNGSDGLWHHMKTFYMEESTFSFSGNVYVGRNDIFDTLS

SIRKKLKGDRPREKIVHVVEKLHCRANGETGIAIRVSGSLIVGNQFLVCGEGLQAEGMPS

VEELSIDIPSKRVGQFREQFMMLPGISMGSFHISRQDLYIIQA*

>Kah-1MKK3-2 Brdisv1Kah-11035802m.p

MAGLGGLKKKLQPLMFNDPDKDGFSTRVPFPEDTCDSYVVSDGGTINLLSRSFGEYNINE

HGFHKRSAGADESDFGEKAYRCASQDMHIFGPIGNGASSVVQRAIFIPVHRILALKKINI

FEKEKRQQILNEMRTLCEACCYPGLVEFQGAFYMPDSGQISIALEYMDGGSLADVIKVKK

SIPEPVLAHMLQKVLLGLRYLHEVRHLVHRDIKPANMLVNLKGEAKITDFGVSAGLDNTM

AMCATFVGTVTYMSPERIRNENYSYAADIWSLGLTILECATGKFPYNVNEGPANLMLQIL

DDPSPAPPENAFSSEFCSFVNDCLQKDADARPTCEQLLSHPFIKRYENAGVDLAAYVKGV

VNPEERLKQIAEMLAVHYYLLFNGSDGLWHHMKTFYMEDSTFRYLIQC*

>Kah-1MKK3-3 Brdisv1Kah-11005087m.p

MDGGWDGMGQRKRRHRAGIPIPTYPGLAASLEFCFVTAGACHTAPGPGSKIAASLFAALL

FQRPPASTSRAIFFCFVIMAAGLEDLRRRGQPIFFDADGNVMPAPDDDSEVLDGGTINLL

SRSSDEYNINERGFHKRTIRSDDEYSSEKAFRCSCHDMHIFDSVGNGASSVVHRAIYVPV

HRVLALKKINIFEKERRQQILNEIITLSEACCYPGLVEFHGVFYTPDSGEIYFALEYMDG

GSLADIIRVKKFISEPVLSHMLQKVLLALRYLHEVRHLVHRDIKPANLLLNLKGDTKITD

FGVTSGLHDSIDMCATFLGSVTYMSPERIRNESYSYSADIWSLGLTALECATGRYPYDVN

GGEADLMLQILEDPSPTPPHDIYSEEFCSFINACLQKDADARPTCDQLLSHSFIKRYEGP

GVDLSEYNKSVHDPSERLSQIAHVSSL*

>Kah-1MKK4 Brdisv1Kah-11019242m.p

MRPGGPPNARPQQPGTPGRARRRPDLTLPLPQRALTSLAVPLPLPPPPSSAPSSASSSGS

SLSSMGAPTPPNSAGSAPPPPPPLAELERARRIGSGAGGTVWMVRHRPTGRPYALKVLYG

NHDDAVRRQITREIAILRTAEHPAIVRCHGMYEQAGELQILLEFMDGGSLEGRRIASEAF

LADVARQVLSGIASPHRRHIVHRDIKPSNLLIASGRRVKIADFGVGRILNQTMDPCNSSV

GTIAYMSPERINTDLNDGAYDGYAGDIWSFGLSILEFYLGRFPLGENLGKQGDWAALMCA

ICYSDSPAPPPIASPEFKSFISCCLQKNPARRPSAAQLLQHRFIAGPQPQVLAAPPS*

>Kah-1MKK5 Brdisv1Kah-11005707m.p

MRPAGSLPSPQPGTPGRPRRRPDLPLPMPQRPDVSSSLAVPLPLPPPSSLGLAQPPAAAA

AAAAPPPPPLGELERVRRVGSGAGGTVWMVRHRPTGRCYALKQLYGNHDDAVRRQIAREI

AILRPAEPPAVVRCHGMYERGGELQILLEYMDGGPLDGRRIAAEGFLADVARQVLSGIAY

PHRRHIVHRDIKPSNLLIDSARRVKIADFGVGRILNQTMDPCNSSVGTIAYMSPERINTD

LNDGAYDGYAGDIWSFGLSILEFYLGRFPFGENLGKQGDWAALMVAICYNDPPEPSAAAS

PEFRGFISCCLQKNPAKRLSAAQLLQPPFVAGPQPLPLAAPPS*

>Kah-1MKK6 Brdisv1Kah-11009516m.p

MRGKKPLKELKLSVPAQETSVDKFLTASGTFKDGELRLNQRGLRLISEEDNGDEHQSTNM

KVEDVQLSMDDLEMIQVIGKGSGGVVQLVQHKWVGTFYALKGIQMNIQEAVRKQIVQELK

INQATQSPHIVSCHQSFYHNGVIYLVLEYMDRGSLADIIKQVKTILEPYLAVLCKQVLEG

LLYLHHERHVIHRDIKPSNLLVNHKGEVKITDFGVSAVLASSIGQRDTFVGTYNYMAPER

ISGSSYDYKSDVWSLGLVILECAIGRFPYTPSEGEGWLSFYELLEAIVDQPPPGAPADQF

SPEFCSFISACIQKDPAERMSASELLNHAFIKKFEGKDLDLRILVESLEPPMNVPE*

>Kah-1MKK10-1 Brdisv1Kah-11001501m.p

MALLREKRLQLSLHVPTRAADAQEAGLHRRPNPAAALPLAATTPAARSSQFRVADFEKLA

VLGRGNGGTGYKVRHRETCELYALKVQHCNGDATAEAEVLSRTASPFVARCHPVPPAAAS

GDVAMLLELVDGGSLDSIVKSRSRGQAEAFSQFPEEALAEVAAQALSGLAYLHARRIVHL

DVKPGNLLVSTGGEVKIADFGIARVLPRAGGDDVRCTAYAGTAAYMSPERFDPEAHGGHY

DPYAADVWGLGVTVLELLMGRYPLLPAGQRPSWAALMCAICFGETPALSDGEASAELRGF

VAACLHKDYRRRASVAELLAHPFVAGRDVAASKCALRKLVTEASMSP*

>Kah-1MKK10-2 Brdisv1Kah-11008762m.p

MALVRQRRQLPHLTLPLDHFALRPPPAPAPTVAAPPPPEAAGPRLSDFERISLLGQGNGG

TVYKARHRRAAAQPPVALKLFVAGDPSAAREAEILRLAADAPHVVRLHAGAPSSPPAAGA

EQPPPPAALALELLPGGSLAGLLRRLGRSMGERPIAAVARQALLGLDALHALRVVHRDLK

PSNLLLGSHGEVKIADFGAGKVLRRRLDPCASYVGTAAYMSPERFDPEAYSGDYAPYAAD

VWSLGLAILELYLGHFPLLPAGQRPDWAALMCAICFGDAPEAPAAASEEFRDFVARCLEK

KAGQRASVAELLEHPFIAERDAEEAKRALAALVAEAELGDL*

>Kah-1MKK10-3 Brdisv1Kah-11001427m.p

MALLREKRLQLSLHVPTRAAEALDAVHRRPNPVAAPLAASTPAAARGSQFRLADFDKLTV

LGRGNGGTVYKVRHRETCELYALKVQHCNGDPTAAAEAEVLSRTASPFIVRCHSVLPGAA

SGDVAMLLELVDGGSLDSIVKSRRAHAFPFPEEALAEVAAQALSGLAYLHARRIVHLDIK

PGNLLVSTGGEVKVADFGIAKVLPRAGADDARCKSYAGTAAYMSPERFDPEAHGGHYDAY

AADVWGLGVTVLELLMGRYPLLPAGQRPSWPALMCAICFGETPVLSDGEASAELRGFVAA

CLRKDHTKRASVAELLAHPFVAGRDVATSKCALRKLVTEASTSP*

>Kah-1MKK10-4 Brdisv1Kah-11001420m.p

MASAKERRLPQLPLKLDVPPCAFRCAAPAPAPATAATPATSASRPPHGEFRLNDFDRLSV

LGRGNGGSVYKVSHRRTSALYALKIIHGAHARPGAADEEADIVRRVVDSPNVVRCHSVLP

TASGDAAALLLELVDGGSLDSLVGGGGFLPEAAVADVAGQAPSGLAPPRARRVAHRDIKP

ANLLLSAAGEVKIADFGIAKVVVSGAGGRARALAYEGTVAYMSPERFDSERHAAAAPSAA

DVWGLGVTLLELLMGRYPLLPAGQKPTWAALMCAICFGELPALPEGAASLEFRGFVAACL

RKDHRKRASVVELLAHPFVAGRDVAASRRALREAIERRCSC*

>Kah-1MKK10-5 Brdisv1Kah-11001422m.p

MALTVRQRRLPQLHISLDLPSCSFRCPNPPVAATASTSGEFRASDFERLAGLGRGNGGTV

YKVAHRRTSAQYALKVLHGGGDPGAAAAEADVLRRAADSPYVVRCHSVFPAASGSGETAL

LLELVDGGSLAPARRGVGVSVFFPEAALAGVAAQALAGLAHLHARRVVHRDIKPANLLVS

GAGGVKVADFGIAMVLPSRAGGERCAAAYEGTVAYMSPERFDSEGRADADPRGADVWGLG

VTVLELLMGRYPLLPAGQKPTWAALMCAICFGELPALPEGAASTELRGFIAACLRKDHTK

RASVAELIKHPFVAGRNMAASRLALRRLGAGA*

>Kah-5MKK1 Brdisv1Kah-51008203m.p

MRKPGKLALPSHESTIGKFLTQSGTFKDGDLLVNKDGLRIVHNSEEGEAPPIEPLDDHQL

SLDDLDAIKVIGKGSSGIVQLVRHKWTDQFFALKVIQLNIQESIRKQIAQELKISLSTQC

QYVVTCYQCFYVNGVISIVLEYMDGGSLADFLKTVRTIPEAYLAAICKQVLQGLMYLHHE

KRVIHRDLKPSNILINHRGEVKISDFGVSAIIASSSAQRDTFTGTFNYMAPERISGQKHG

YMSDIWSLGLVMLECATGNFPYPSPDSFYELLEAVVDQPPPSAPTDQFSPEFCSFISACI

QKEATDRSSAQVLSDHPFLSMYDDLNIDLADYFTTAGSPLATFKQIVL*

>Kah-5MKK3-1 Brdisv1Kah-51029647m.p

MAGLEELKKKLQPLLFDDPDKDGISTRVPFLEDNCDSYVVSDGGTINLLSRSFGEYNINE

HGFHKRSTGADESDFGEKAYRCASHDMHIFGPIGNGASSVVQRAIFIPVHRILALKKINI

FEKEKRQQILNEMRTLCEASCYPGLVEFQGAFYMPDSGQISIALEYMDGGSLADVIKVKK

SIPEQVLAHMLQKVLLGLRYLHEVRHLVHRDIKPANMLVNLKGEAKITDFGVSAGLDNTM

AMCATFVGTVTYMSPERIRNENYSYAADIWSLGLTILECATGKFPYNVNEGPANLMLQIL

DDPSPTPPADAYSPEFCSFVNDCLQKDPDARPTCEQLFGHPFIKRYENAGVDLIAYVKGV

VDPTERLKEIAEMLAVHYYLLFNGSDGLWHHMKTFYMEESTFSFSGNVYVGRNDIFDTLS

SIRKKLKGDRPREKIVHVVEKLHCRANGETGIAIRVSGSLIVGNQFLVCGEGLQAEGMPS

VEELSIDIPSKRVGQFREQFMMLPGISMGSFHISRQDLYIIQA*

>Kah-5MKK3-2 Brdisv1Kah-51044528m.p

MAGLEELKKKLQPLMFNDPDKDGFSTRVPFPEDTCDSYVVSDGGTINLLSRSFGEYNINE

HGFHKRSAGADESDFGEKAYRCASQDMHIFGPIGNGASSVVQRAIFIPVHRILALKKINI

FEKEKRQQILNEMRTLCEACCYPGLVEFQGAFYMPDSGQISIALEYMDGGSLADVIKVKK

SIPEPVLAHMLQKVLLGLRYLHEVRHLVHRDIKPANMLVNLKGEAKITDFGVSAGLDNTM

AMCATFVGTVTYMSPERIRNENYSYAADIWSLGLTILECATGKFPYNVNEGPANLMLQIL

DDPSPAPPENAFSSEFCSFVNDCLQKDADARPTCEQLLSHPFIKRYENAGVDLAAYVKGV

VNPEERLKQIAEMLAVHYYLLFNGSDGLWHHMKTFYMEDSTFSFSGNLYVGQSDIFDTLS

NIRTKLKGDRPREKIVHVVEKLHCRANEETGIAIRVSGSFIVSNQFLICGEGLQAEGMPS

LEELSIDIPSKRVGQFREQFIMHPGRSMGCYYISRQDLYIIQA*

>Kah-5MKK3-3 Brdisv1Kah-51032682m.p

MDGGWDGMGQRKRRHRAGIPIPTYPGLAASLEFCFVTAGACHTAPGPGSKIAASLFAALL

FQRPPASTSRAIFFCFVIMAAGLEDLRRRVQPIFFDADGNVMPAPDDDSEVLDGGTINLL

SRSSDEYNINERGFHKRTIRSDDEYSSEKAFRCSCHDMHIFDSVGNGASSVVHRAIYVPV

HRVLALKKINIFEKERRQQILNEIITLSEACCYPGLVEFHGVFYTPDSGEIYFALEYMDG

GSLADIIRVKKFISEPVLSHMLQKVLLALRYLHEVRHLVHRDIKPANLLLNLKGDTKITD

FGVTSGLHDSIDMCATFLGSVTYMSPERIRNESYSYSADIWSLGLTALECATGRYPYDVN

GGEADLMLQILEDPSPTPPHDIYSEEFCSFINACLQKDADARPTCDQLLSHSFIKRYEGP

GVDLSEYNKSVHDPSERLSQIAHMLAVHYYLIFDGGDDQWCHMKTFYQQDSIFSFSGETH

VGKSEIFETLSRIRKMLKGNSPCEKIAHVMEKVYCRSHGEEGMRVRVSGSFIVGNEFVVC

ADGVRAEGMLSIDELSPDILSKQAGHFQEDFFMEPGTALGCYVISKQELHIADT*

>Kah-5MKK4 Brdisv1Kah-51039016m.p

MRPGGPPNARPQQPGTPGRARRRPDLTLPLPQRDLTSLAVPLPLPPPPSSAPSSASSSGS

SLSSMGAPTPPNSAGSAPPPPPPLAELERVRRIGSGAGGTVWMVRHRPTGRPYALKVLYG

NHDDAVRRQITREIAILRTAEHPAIVRCHGMYEQAGELQILLEFMDGGSLEGRRIASEAF

LADVARQVLSGIAYLHRRHIVHRDIKPSNLLIDSGRRVKIADFGVGRILNQTMDPCNSSV

GTIAYMSPERINTDLNDGAYDGYAGDIWSFGLSILEFYLGRFPLGENLGKQGDWAALMCA

ICYSDSPAPPPIASPEFKSFISCCLQKNPARRPSAAQLLQHRFIAGPQPQVLAAPPS*

>Kah-5MKK5 Brdisv1Kah-51007471m.p

MRPAGSLPSPQPGTPGRPRRRPDLTLPMPQRPDVSSSLAVPLPLPPPSSLGLAQPPAAAA

AAAAPPPPPLGELERVRRVGSGAGGTVWMVRHRPTGRCYALKQLYGNHDDAVRRQIAREI

AILRTAEHPAVVRCHGMYERGGELQILLEYMDGGSLDGRRIAAEGFLADVARQVLSGIAY

LHRRHIVHRDIKPSNLLIDSARRVKIADFGVGRILNQTMDPCNSSVGTIAYMSPERINTD

LNDGAYDGYAGDIWSFGLSILEFYLGRFPFGENLGKQGDWAALMVAICYNDPPEPSAAAS

PEFRGFISCCLQKNPAKRLSAAQLLQHPFVAGPQPLPLAAPPS*

>Kah-5MKK6 Brdisv1Kah-51012549m.p

MRGKKPLKELKLSVPAQETSVDKFLTASGTFKDGELRLNQRGLRLISEEENGDEHQSTNM

KVEDVQLSMDDLEMIQVIGKGSGGVVQLVQHKWVGTFYALKGIQMNIQEAVRKQIVQELK

INQATQSPHIVSCHQSFYHNGVIYLVLEYMDRGSLADIIKQVKTILEPYLAVLCKQVLEG

LLYLHHERHVIHRDIKPSNLLVNHKGEVKITDFGVSAVLASSIGQRDTFVGTYNYMAPER

ISGSSYDYKSDVWSLGLVILECAIGRFPYTPSEGEGWLSFYELLEAIVDQPPPGAPADQF

SPEFCSFISACIQKDPAERMSASELLNHAFIKKFEGKDLDLRILVESLEPPMNVPE*

>Kah-5MKK10-1 Brdisv1Kah-51001737m.p

MALLREKRLQLSLHVPTRAADAQEAGLHRRPNPAAALPLAATTPAARSSQFRVADFEKLA

VLGRGNGGTVYKVRHRETCELYALKVQHCNGDATAEAEVLSRTASPFVVRCHSVLPAAAS

GDVAMLLELVDGGSLDSIVKSRSRGQAEAFSQFPEEALAEVAAQALSGLAYLHARRIVHL

DVKPGNLLVSTGGEVKIADFGIARVLPRAGGDDVRCTAYAGTAAYMSPERFDPEAHGGHY

DPYAADVWGLGVTVLELLMGRYPLLPAGQRPSWAALMCAICFGETPALSDGEASAELRGF

VAACLHKDYRRRASVAELLAHPFVAGRDVAASKCALRKLVTEASMSP*

>Kah-5MKK10-2 Brdisv1Kah-51011638m.p

MALVRQRRQLPHLTLPLDHFALRPPPVPAPAPTVAASTSSEAAGLRLSDFERISLLGQGN

GGTVYKARHRRAAAQPPVALKLFVAGDPSAAREAEILRLAADAPHVVRLHAVVPSSSPAA

GAEQPPPAALALELLPGGSLAGLLRRLGRSMGERPIAAVARQALLGLDALHALRVVHRDL

KPSNLLLGSHGEVKIADFGAGKVLRRRLDPCASYVGTAAYMSPERFDPEAYSGDYDPYAA

DVWSLGLAILELYLGHFPLLPAGQRPDWAALMCAICFGDAPEAPAAASEEFRDFVARCLE

KKAGQRASVAELLEHPFIAERDAEEAKRALAALVAEAELGDL*

>Kah-5MKK10-3 Brdisv1Kah-51001629m.p

MALLREKRLQLSLHVPTRAAEALDAVHRRPNPVAATLAASTPAAARSSQFRLADFDKLTV

LGRGNGGTVYKVRHRETCELYALKVQHCNGDPTAAAEAEVLSRTASPFIVRCHSVLPGAA

SGDVAMLLELVDGGSLDSIVKSRRAHAFPFPEEALAEVAAQALSGLAYLHARRIVHLDIK

PGNLLVSTGGEVKVADFGIAKVLPRAGADDARCKSYAGTAAYMSPERFDPEAHGGHYDAY

AADVWGLGVTVLELLMGRYPLLPAGQRPSWPALMCAICFGETPVLSDGEASAELRGFVAA

CLRKDHTKRASVAELLAHPFVAGRDVATSKCALRKLVTEASTSP*

>Kah-5MKK10-4 Brdisv1Kah-51001626m.p

MASAKERRLPQLHLKLDVPTCAFRCAAPAPAPATAATPATSASRPPHGEFRLNDFDRLSV

LGRGNGGSVYKVSHRRTSALYALKIIHGAHARPGAADEEADIVRRVVDSPNVVRCHSVLP

TASGDAAALLLELVDGGSLDSLVGGGGFLPEAAVADVAAQALSGLAHLRARRVAHRDIKP

ANLLLSAAGEVKIADFGIAKVVVSGAGGRARALAYEGTVAYMSPERFDSERHADADPYAA

DVWGLGVTLLELLMGRYPLLPAGQKPTWAALMCAICFGELPALPEGAASLEFRGFVAACL

RKDHRKRASVVELLAHPFVAGRDVAASRRALREAIERRCSC*

>Kah-5MKK10-5 Brdisv1Kah-51001628m.p

MALTVRQRRLPQLHISLDLPSCSFRCPNPPVAATASTSGEFRASDFERLAVLGRGNGGTV

YKVAHRRTSAQYALKVLHGGGDPGAAAAEADVLRRAADSPYVVRCHSVFPAASGSGETAL

LLELVDGGSLDSVRRGVGVSVFFPEAALAEVAAQALAGLAHLHARRVVHRDIKPANLLVS

GAGGVKVADFGIAMVLPSRAGGERCAAAYEGTVAYMSPERFDSEGRADADPRGADVWGLG

VTVLELLMGRYPLLPAGQKPTWAALMCAICFGELPALPEGAASTELRGFIAACLRKDHTK

RASVAELIKHPFVAGRNMAASRLALRRLVAGA*

>Koz-1MKK1 Brdisv1Koz-11008234m.p

MRKPGKLALPSHESTIGKFLTQSGTFKDGDLLVNKDGLRIVHNSEEGEAPPIEPLDDHQL

SLDDLDAIKVIGKGSSGIVQLVRHKWTDQFFALKVIQLNIQESIRKQIAQELKISLSTQC

QYVVTCYQCFYVNGVISIVLEYMDGGSLADFLKTVRTIPEAYLAAICKQVLQGLMYLHHE

KRVIHRDLKPSNILINHRGEVKISDFGVSAIIASSSAQRDTFTGTFNYMAPERISGQKHG

YMSDIWSLGLVMLECATGNFPYPSPDSFYELLEAVVDQPPPSAPTDQFSPEFCSFISACI

QKEATDRSSAQVLSDHPFLSMYDDLNIDLADYFTTAGSPLATFKQIVL*

>Koz-1MKK3-1 Brdisv1Koz-11029896m.p

MAGLEELKKKLQPLLFDDPDKDGISTRVPFLEDNCDSYVVSDGGTINLLSRSFGEYNINE

HGFHKRSTGADESDFGEKAYRCASHDMHIFGPIGNGASSVVQRAIFIPVHRILALKKINI

FEKEKRQQILNEMRTLCEASCYPGLVEFQGAFYMPDSGQISIALEYMDGGSLADVIKVKK

SIPEQVLAHMLQKVLLGLRYLHEVRHLVHRDIKPANMLVNLKGEAKITDFGVSAGLDNTM

AMCATFVGTVTYMSPERIRNENYSYAADIWSLGLTILECATGKFPYNVNEGPANLMLQIL

DDPSPTPPADAYSPEFCSFVNDCLQKDPDARPTCEQLFGHPFIKRYENAGVDLIAYVKGV

VDPTERLKEIAEMLAVHYYLLFNGSDGLWHHMKTFYMEESTFSFSGNVYVGRNDIFDTLS

SIRKKLKGDRPREKIVHVVEKLHCRANGETGIAIRVSGSLIVGNQFLVCGEGLQAEGMPS

VEELSIDIPSKRVGQFREQFMMLPGISMGSFHISRQDLYIIQA*

>Koz-1MKK3-2 Brdisv1Koz-11045109m.p

MAGLEELKKKLQPLMFNDPDKDGFSTRVPFPEDTCDSYVVSDGGTINLLSRSFGEYNINE

HGFHKRSAGADESDFGEKAYRCASQDMHIFGPIGNGASSVVQRAIFIPVHRILALKKINI

FEKEKRQQILNEMRTLCEACCYPGLVEFQGAFYMPDSGQISIALEYMDGGSLADVIKVKK

SIPEPVLAHMLQKVLLGLRYLHEVRHLVHRDIKPANMLVNLKGEAKITDFGVSAGLDNTM

AMCATFVGTVTYMSPERIRNENYSYAADIWSLGLTILECATGKFPYNVNEGPANLMLQIL

DDPSPAPPENAFSSEFCSFVNDCLQKDADARPTCEQLLSHPFIKRYENAGVDLAAYVKGV

VNPEERLKQIAEMLAVHYYLLFNGSDGLWHHMKTFYMEDSTFSFSGNLYVGQSDIFDTLS

NIRTKLKGDRPREKIVHVVEKLHCRANEETGIAIRVSGSFIVSNQFLICGEGLQAEGMPS

LEELSIDIPSKRVGQFREQFIMHPGRSMGCYYISRQDLYIIQA*

>Koz-1MKK3-3 Brdisv1Koz-11032882m.p

MDGGWDGMGQRKRRHRAGIPIPTYPGLAASLEFCFVTAGACHTAPGPGSKIAASLFAALL

FQRPPASTSRAIFFCFVIMAAGLEDLRRRVQPIFFDADGNVMPAPDDDSEVLDGGTINLL

SRSSDEYNINERGFHKRTIRSDDEYSSEKAFRCSCHDMHIFDSVGNGASSVVHRAIYVPV

HRVLALKKINIFEKERRQQILNEIITLSEACCYPGLVEFHGVFYTPDSGEIYFALEYMDG

GSLADIIRVKKFISEPVLSHMLQKVLLALRYLHEVRHLVHRDIKPANLLLNLKGDTKITD

FGVTSGLHDSIDMCATFLGSVTYMSPERIRNESYSYSADIWSLGLTALECATGRYPYDVN

GGEADLMLQILEDPSPTPPHDIYSEEFCSFINACLQKDADARPTCDQLLSHSFIKRYEGP

GVDLSEYNKSVHDPSERLSQIAHMLAVHYYLIFDGGDDQWCHMKTFYQQDSIFSFSGETH

VGKSEIFETLSRIRKMLKGNSPCEKIAHVMEKVYCRSHGEEGMRVRVSGSFIVGNEFVVC

ADGVRAEGMLSIDELSPDILSKQAGHFQEDFFMEPGTALGCYVISKQELHIADT*

>Koz-1MKK4 Brdisv1Koz-11039377m.p

MRPGGPPNARPQQPGTPGRARRRPDLTLPLPQRDLTSLAVPLPLPPPPSSAPSSASSSGS

SLSSMGAPTPPNSAGSAPPPPPPLAELERVRRIGSGAGGTVWMVRHRPTGRPYALKVLYG

NHDDAVRRQITREIAILRTAEHPAIVRCHGMYEQAGELQILLEFMDGGSLEGRRIASEAF

LADVARQVLSGIAYLHRRHIVHRDIKPSNLLIDSGRRVKIADFGVGRILNQTMDPCNSSV

GTIAYMSPERINTDLNDGAYDGYAGDIWSFGLSILEFYLGRFPLGENLGKQGDWAALMCA

ICYSDSPAPPPIASPEFKSFISCCLQKNPARRPSAAQLLQHRFIAGPQPQVLAAPPS*

>Koz-1MKK5 Brdisv1Koz-11007461m.p

MRPAGSLPSPQPGTPGRPRRRPDLTLPMPQRPDVSSSLAVPLPLPPPSSLGLAQPPAAAA

AAAAPPPPPLGELERVRRVGSGAGGTVWMVRHRPTGRCYALKQLYGNHDDAVRRQIAREI

AILRTAEHPAVVRCHGMYERGGELQILLEYMDGGSLDGRRIAAEGFLADVARQVLSGIAY

LHRRHIVHRDIKPSNLLIDSARRVKIADFGVGRILNQTMDPCNSSVGTIAYMSPERINTD

LNDGAYDGYAGDIWSFGLSILEFYLGRFPFGENLGKQGDWAALMVAICYNDPPEPSAAAS

PEFRGFISCCLQKNPAKRLSAAQLLQHPFVAGPQPLPLAAPPS*

>Koz-1MKK6 Brdisv1Koz-11012587m.p

MRGKKPLKELKLSVPAQETSVDKFLTASGTFKDGELRLNQRGLRLISEEENGDEHQSTNM

KVEDVQLSMDDLEMIQVIGKGSGGVVQLVQHKWVGTFYALKGIQMNIQEAVRKQIVQELK

INQATQSPHIVSCHQSFYHNGVIYLVLEYMDRGSLADIIKQVKTILEPYLAVLCKQVLEG

LLYLHHERHVIHRDIKPSNLLVNHKGEVKITDFGVSAVLASSIGQRDTFVGTYNYMAPER

ISGSSYDYKSDVWSLGLVILECAIGRFPYTPSEGEGWLSFYELLEAIVDQPPPGAPADQF

SPEFCSFISACIQKDPAERMSASELLNHAFIKKFEGKDLDLRILVESLEPPMNVPE*

>Koz-1MKK10-1 Brdisv1Koz-11001847m.p

MALLREKRLQLSLHVPTRAADAQEAGLHRRPNPAAALPLAATTPAARSSQFRVADFEKLA

VLGRGNGGTVYKVRHRETCELYALKVQHCNGDATAEAEVLSRTASPFVVRCHSVLPAAAS

GDVAMLLELVDGGSLDSIVKSRSRGQAEAFSQFPEEALAEVAAQALSGLAYLHARRIVHL

DVKPGNLLVSTGGEVKIADFGIARVLPRAGGDDVRCTAYAGTAAYMSPERFDPEAHGGHY

DPYAADVWGLGVTVLELLMGRYPLLPAGQRPSWAALMCAICFGETPALSDGEASAELRGF

VAACLHKDYRRRASVAELLAHPFVAGRDVAASKCALRKLVTEASMSP*

>Koz-1MKK10-2 Brdisv1Koz-11011659m.p

MALVRQRRQLPHLTLPLDHFALRPPPVPAPAPTVAASTSSEAAGLRLSDFERISLLGQGN

GGTVYKARHRRAAAQPPVALKLFVAGDPSAAREAEILRLAADAPHVVRLHAVVPSSSPAA

GAEQPPPAALALELLPGGSLAGLLRRLGRSMGERPIAAVARQALLGLDALHALRVVHRDL

KPSNLLLGSHGEVKIADFGAGKVLRRRLDPCASYVGTAAYMSPERFDPEAYSGDYDPYAA

DVWSLGLAILELYLGHFPLLPAGQRPDWAALMCAICFGDAPEAPAAASEEFRDFVARCLE

KKAGQRASVAELLEHPFIAERDAEEAKRALAALVAEAELGDL*

>Koz-1MKK10-3 Brdisv1Koz-11001740m.p

MALLREKRLQLSLHVPTRAAEALDAVHRRPNPVAATLAASTPAAARSSQFRLADFDKLTV

LGRGNGGTVYKVRHRETCELYALKVQHCNGDPTAAAEAEVLSRTASPFIVRCHSVLPGAA

SGDVAMLLELVDGGSLDSIVKSRRAHAFPFPEEALAEVAAQALSGLAYLHARRIVHLDIK

PGNLLVSTGGEVKVADFGIAKVLPRAGADDARCKSYAGTAAYMSPERFDPEAHGGHYDAY

AADVWGLGVTVLELLMGRYPLLPAGQRPSWPALMCAICFGETPVLSDGEASAELRGFVAA

CLRKDHTKRASVAELLAHPFVAGRDVATSKCALRKLVTEASTSP*

>Koz-1MKK10-4 Brdisv1Koz-11001737m.p

MASAKERRLPQLHLKLDVPTCAFRCAAPAPAPATAATPATSASRPPHGEFRLNDFDRLSV

LGRGNGGSVYKVSHRRTSALYALKIIHGAHARPGAADEEADIVRRVVDSPNVVRCHSVLP

TASGDAAALLLELVDGGSLDSLVGGGGFLPEAAVADVAAQALSGLAHLRARRVAHRDIKP

ANLLLSAAGEVKIADFGIAKVVVSGAGGRARALAYEGTVAYMSPERFDSERHADADPYAA

DVWGLGVTLLELLMGRYPLLPAGQKPTWAALMCAICFGELPALPEGAASLEFRGFVAACL

RKDHRKRASVVELLAHPFVAGRDVAASRRALREAIERRCSC*

>Koz-1MKK10-5 Brdisv1Koz-11001739m.p

MALTVRQRRLPQLHISLDLPSCSFRCPNPPVAATASTSGEFRASDFERLAVLGRGNGGTV

YKVAHRRTSAQYALKVLHGGGDPGAAAAEADVLRRAADSPYVVRCHSVFPAASGSGETAL

LLELVDGGSLDSVRRGVGVSVFFPEAALAEVAAQALAGLAHLHARRVVHRDIKPANLLVS

GAGGVKVADFGIAMVLPSRAGGERCAAAYEGTVAYMSPERFDSEGRADADPRGADVWGLG

VTVLELLMGRYPLLPAGQKPTWAALMCAICFGELPALPEGAASTELRGFIAACLRKDHTK

RASVAELIKHPFVAGRNMAASRLALRRLVAGA*

>Koz-3MKK1 Brdisv1Koz-31007066m.p

MRKPGKLALPSHESTIGKFLTQSGTFKDGDLLVNKDGLRIVHNSEEGEAPPIEPLDDHQL

SLDDLDAIKVIGKGSSGIVQLVRHKWTDQFFALKVIQLNIQESIRKQIAQELKISLSTQC

QYVVTCYQCFYVNGVISIVLEYMDGGSLADFLKTVRTIPEAYLAAIFKQVLQGLMYLHHE

KRVIHRDLKPSNILINHRGEVKISDFGVSAIIASSSAQRDTFTGTFNYMAPERISGQKHG

YMSDIWSLGLVMLECATGNFPYPSPDSFYELLEAVVDQPPPSAPTDQFSPEFCSFISACI

QKEATDRSSAQVLSDHPFLSMYDDLNIDLADYFTTAGSPLATFKQIVL*

>Koz-3MKK3-1 Brdisv1Koz-31033544m.p

MAGLEELKKKLQPLLFDDPDKDGISTRVPFLEDNCDSYVVSDGGTINLLSRSFGEYNINE

HGFHKRSTGADESDFGEKAYRCASHDMHIFGPIGNGASSVVQRAIFIPVHRILALKKINI

FEKEKRQQILNEMRTLCEASCYPGLVEFQGAFYMPDSGQISIALEYMDGGSLADVIKVKK

SIPEQVLAHMLQKVLLGLRYLHEVRHLVHRDIKPANMLVNLKGEAKITDFGVSAGLDNTM

AMCATFVGTVTYMSPERIRNENYSYAADIWSLGLTILECATGKFPYNVNEGPANLMLQIL

DDPSPTPPADAYSPEFCSFVNDCLQKDPDARPTCEQLFGHPFIKRYENAGVDLIAYVKGV

VDPTERLKEIAEMLAVHYYLLFNGSDGLWHHMKTFYMEESTFSFSGNVYVGRNDIFDTLS

SIRKKLKGDRPREKIVHVVEKLHCRANGETGIAIRVSGSLIVGNQFLVCGEGLQAEGMPS

VEELSIDIPSKRVGQFREQFMMLPGISMGSFHISRQDLYIIQA*

>Koz-3MKK3-2 Brdisv1Koz-31039828m.p

MAGLGGLKKKLQPLMFNDPDKDGFSTRVPFPEDTCDSYVVSDGGTINLLSRSFGEYNINE

HGFHKRSAGADESDFGEKAYRCASQDMHIFGPIGNGASSVVQRAIFIPVHRILALKKINI

FEKEKRQQILNEMRTLCEACCYPGLVEFQGAFYMPDSGQISIALEYMDGGSLADVIKVKK

SIPEPVLAHMLQKVLLGLRYLHEVRHLVHRDIKPANMLVNLKGEAKITDFGVSAGLDNTM

AMCATFVGTVTYMSPERIRNENYSYAADIWSLGLTILECATGKFPYNVNEGPANLMLQIL

DDPSPAPPENAFSSEFCSFVNDCLQKDADARPTCEQLLSHPFIKRYENAGVDLAAYVKGV

VNPEERLKQIAEMLAVHYYLLFNGSDGLWHHMKTFYMEDSTFSFSGNLYVGQSDIFDTLS

NIRTKLKGDRPREKIVHVVEKLHCRANEETGIAIRVSGSFIVSNQFLICGEGLQAEGMPS

LEELSIDIPSKRVGQFREQFIMHPGRSMGCYYISRQDLYIIQA*

>Koz-3MKK3-3 Brdisv1Koz-31021934m.p

MDGGWDGMGQRKRRHRAGIPIPTYPGLAASLEFCFVTAGACHTAPGPGSKIAASLFAALL

FQRPPASTSRAIFFCFVIMAAGLEDLRRRGQPIFFDADGNVMPAPDDDSEVLDGGTINLL

SRSSDEYNINERGFHKRTIRSDDEYSSEKAFRCSCHDMHIFDSVGNGASSVVHRAIYVPV

HRVLALKKINIFEKERRQQILNEIITLSEACCYPGLVEFHGVFYTPDSGEIYFALEYMDG

GSLADIIRVKKFISEPVLSHMLQKVLLALRYLHEVRHLVHRDIKPANLLLNLKGDTKITD

FGVTSGLHDSIDMCATFLGSVTYMSPERIRNESYSYSADIWSLGLTALECATGRYPYDVN

GGEADLMLQILEDPSPTPPHDIYSEEFCSFINACLQKDADARPTCDQLLSHSFIKRYEGP

GVDLSEYNKSVHDPSERLSQIAHMLAVHYYLIFDGGDDQWCHMKTFYQQDSIFSFSGETH

VGKSEIFETLSRIRKMLKGNSPCEKIAHVMEKVYCRSHGEEGMRVRVSGSFIVGNEFVVC

ADGVRAEGMLSIDELSPDILSKQAGHFQEDFFMEPGTALGCYVISKQELHIADT*

>Koz-3MKK4 Brdisv1Koz-31027327m.p

MRPGGPPNARPQQPGTPGRARRRPDLPLPLPQRDLTSLAVPLPLPPPPSSAPSSASSSGS

SLSSMGAPTPPNSAGSAPPPPPPPAELERVRRIGSGAGGTVWMVRHRPTGRPYALKVLYG

NHDDAVRRQITREIAILRTAEHPAIVRCHGMYEQAGELQILLEFMDGGSLEGRRIASEAF

LADVARQVLSGIAYLHRRHIVHRDIKPSTLLIDPGRRVKIADFGVGRILNQTMDPCNSSV

GTIAYMSPERINTDLNDGAYDGYAGDIWSFGLSILEFYLGRFPLGENLGKQGDWAALMCA

ICYSDSPAPPPIASPEFKSFISCCLQKNPARRPSAAQLLQPRFIAGPQPQVLAAPPS*

>Koz-3MKK5 Brdisv1Koz-31006454m.p

MRPAGSLPSPQPGTPGRPRRRPDLTLPMPQRPDVSSSLAVPLPLPPPSSLGLAQPPAAAP

PPPPLGELERVRRVGSGAGGTVWMGRHRPTGRCYALKQLYGNHDDAVRRQIAREIAILRT

AEHPAVVRCHGMYERGGELQILLEYMDGGPPAARRIAAEGFLADVARQVLSGIAYLHRRH

IVHRDIKPSNLLIDSARRVKIADFGVGRILNQTMDPCNSSVGTIAYMSPERINTDLNDGA

YDGYAGDIWSFGLSILEFYLGRFPFGENLGKQGDWAALMVAICYNDPPEPSAAASPEFRG

FISCCLQKNPAKRLSAAQLLQHPFVAGPQPLPLAAPPS*

>Koz-3MKK6 Brdisv1Koz-31010882m.p

MRGKKPLKELKLSVPAQETSVDKFLTASGTFKDGELRLNQRGLRLISEEENGDEHQSTNM

KVEDVQLSMDDLEMIQVIGKGSGGVVQLVQHKWVGTFYALKGIQMNIQEAVRKQIVQELK

INQATQSPHIVSCHQSFYHNGVIYLVLEYMDRGSLADIIKQVKTIREPYLAVLCKQVLEG

LLYLHHERHVIHRDIKPSNLLVNHKGEVKITDFGVSAVLASSIGQRDTFVGTYNYMAPER

ISGSSYDYKSDVWSLGLVILECAIGRFPYTPSEGEGWLSFYELLEAIVDQPPPGAPADQF

SPEFCSFISACIQKDPAERMSASELLNHAFIKKFEGKDLDLRILVESLEPPMNVPE*

>Koz-3MKK10-1 Brdisv1Koz-31001651m.p

MALLREKRLQLSLHVPTRAADAQEAGLHRRPNPAAALPLAATTPAARSSQFRVADFEKLA

VLGRGNGGTVYKVRHRETCELYALKVQHCNGDATAEAEVLSRTASPFVARCHSVPPAAAS

GDVAMLLELVDGGSLDSIVKSRSSGQAEAFSQFPEEALAEVAAQALSGLAYLHARRIVHL

DVKPGNLLVSTGGEVKIADFGIARVLPRAGGDDVRCTAYAGTAAYMSPERFDPEAHGGHY

DPYAADVWGLGVTVLELLMGRYPLLPAGQRPSWAALMCAICFGETPALSDGEASAELRGF

VAACLHKDYRRRASVAELLAHPFVAGRDVAASKCALRKLVTEASMSP*

>Koz-3MKK10-2 Brdisv1Koz-31010012m.p

MALVRQRRQLPHLPPPLDHFALRPPPVPAPAPPVAASTSSEAAGLRLSDFERISLLGQGN

GGTVYKARHRRAAAQPPVALKLFVAGDPSAAREAEILRLAADAPHVVRLHAVVPSSSPAA

GAGQPPPAALALELLPGGSLAGLLRRLGRSMGERPIAAVARQALLGLDALHALRVVHRDL

KPSNLLLGSHGEVKIADFGAGKVLRRRLDPCASYVGTAAYMSPERFDPEAYSGDYDPYAA

DVWSLGLAILELYLGHFPLLPAGQRPDWAALMCAICFGDAPEAPAAASEEFRDFVARCLE

KKAGQRASVAELLEHPFIAERDAEEAKRALAALVAEAELGDL*

>Koz-3MKK10-3 Brdisv1Koz-31001571m.p

MALLREKRLQLSLHVPTRAAEALDAVHRRPNPVAATLAASTPAAARASQFRLADFDKLTV

LGRGNGGTVYKVRHRETCELYALKVQHCNGDPTAAAEAEVLSRTASPFIVRCHSVLPGAA

SGDVAMLLELVDGGSLDSIVKSRRAHAFPFPEEALAEVAAQALSGLAYLHARRIVHLDIK

PGNLLVSTGGEVKVADFGIAKVLPRAGADDARCKSYAGTAAYMSPERFDPEAHGGHYDAW

PALMCAICFGETPVLSDGEASAELRGFVAACLRKDHTKRASVAELLAHPFVAGRDVATSK

CALRKLVTEASTSP*

>Koz-3MKK10-4 Brdisv1Koz-31001569m.p

MASAKERRLPQLHLKLDVPTCAFRCAAPAPAPATAAPPATSASRPPHGEFRLNDFDRLSV

LGRGNGGSVYKVSHRRTSALYALKIIHGAPARPGAADEEADIARRVVDSPNVVRCHSVPP

TASGDAAALLLELVDGGSLDSLVGGGGFLPEAAVGGVAAQALSGLAHLRARRVAHRDIKP

ANLLLSAAGEVKIADFGIAKVVVSGAGGRARALAYEGTVAYMSPERFDSERHADADPYAA

DVWGLGVTLLELLMGRYPLLPAGQKPTWAALMCAICFGELPALPEGAASLEFRGFVAACL

RKDHRKRASVVELLAHPFVAGRDVAASRRALREAIERRCSC*

>Koz-3MKK10-5 Brdisv1Koz-31041352m.p

MALTVRQRRLPQLHISLDLPSCSFRCPNPPVAATASTSGEFRASDFERLAVLGRGNGGTV

YKVAHRRTSAQYALKVRHGGGDPGAAAAEADVLRRAADSPYVVRCHSVFPAASGSGETAL

LLELVDGGSLAPARRGVGVSVFFPEAALAEVAAQALAGLAHLHARRVVHRDIKPANLLVS

GAGGVKVADFGIAMVLPSRAGGERCAAAYEGTVAYMSPERFDSEGRAGAAPRGADVWGLG

VTVLELLMGRYPLLPAGQKPTWAALMCAICFGELPALPEGAASTELRGFIAACLRKDHTK

RASVAELIKHPFVAGRNMAASRLALRRLVAGA*

>Sig2MKK1 Brdisv1Sig21007754m.p

MRKPGKLALPSHESTIGKFLTQSGTFKDGDLLVNKDGLRIVHNSEEGEAPPIEPLDDHQL

SLDDLDAIKVIGKGSSGIVQLVRHKWTDQFFALKVIQLNIQESIRKQIAQELKISLSTQC

QYVVTCYQCFYVNGVISIVLEYMDGGSLADFLKTVRTIPEAYLAAICKQVLQGLMYLHHE

KRVIHRDLKPSNILINHRGEVKISDFGVSAIIASSSAQRDTFTGTFNYMAPERISGQKHG

YMSDIWSLGLVMLECATGNFPYPSPDSFYELLEAVVDQPPPSAPTDQFSPEFCSFISACI

QKEATDRSSAQVLSDHPFLSMYDDLNIDLADYFTTAGSPLATFKQIVL*

>Sig2MKK3-1 Brdisv1Sig21027513m.p

MAGLEELKKKLQPLLFDDPDKDGISTRVPFLEDNCDSYVVSDGGTINLLSRSFGEYNINE

HGFHKRSTGADESDFGEKAYRCASHDMHIFGPIGNGASSVVQRAIFIPVHRILALKKINI

FEKEKRQQILNEMRTLCEASCYPGLVEFQGAFYMPDSGQISIALEYMDGGSLADVIKVKK

SIPEQVLAHMLQKVLLGLRYLHEVRHLVHRDIKPANMLVNLKGEAKITDFGVSAGLDNTM

AMCATFVGTVTYMSPERIRNENYSYAADIWSLGLTILECATGKFPYNVNEGPANLMLQIL

DDPSPTPPADAYSPEFCSFVNDCLQKDPDARPTCEQLFGHPFIKRYENAGVDLIAYVKGV

VDPTERLKEIAEMLAVHYYLLFNGSDGLWHHMKTFYMEESTFSFSGNVYVGRNDIFDTLS

SIRKKLKGDRPREKIVHVVEKLHCRANGETGIAIRVSGSLIVGNQFLVCGEGLQAEGMPS

VEELSIDIPSKRVGQFREQFMMLPGISMGSFHISRQDLYIIQA*

>Sig2MKK3-2 Brdisv1Sig21006212m.p

MAGLEELKKKLQPLMFNDPDKDGFSTRVPFPEDTCDSYVVSDGGTINLLSRSFGEYNINE

HGFHKRSAGADESDFGEKAYRCASQDMHIFGPIGNGASSVVQRAIFIPVHRILALKKINI

FEKEKRQQILNEMRTLCEACCYPGLVEFQGAFYMPDSGQISIALEYMDGGSLADVIKVKK

SIPEPVLAHMLQKVLLGLRYLHEVRHLVHRDIKPANMLVNLKGEAKITDFGVSAGLDNTM

AMCATFVGTVTYMSPERIRNENYSYAADIWSLGLTILECATGKFPYNVNEGPANLMLQIL

DDPSPAPPENAFSSEFCSFVNDCLQKDADARPTCEQLLSHPFIKRYENAGVDLAAYVKGV

VNPEERLKQIAEMLAVHYYLLFNGSDGLWHHMKTFYMEDSTFSFSGNLYVGQSDIFDTLS

NIRTKLKGDRPREKIVHVVEKLHCRANEETGIAIRVSGSFIVSNQFLICGEGLQAEGMPS

LEELSIDIPSKRVGQFREQFIMHPGRSMGCYYISRQDLYIIQA*

>Sig2MKK3-3 Brdisv1Sig21030257m.p

MGWDGMGQRKRRHRAGIPIPTYPGLAASLEFCFVTAGACHTAPGPGSKIAASLFAALLFQ

RPPASTSRAIFFCFVIMAAGLEDLRRRVQPIFFDADGNVMPAPDDDSEVLDGGTINLLSR

SSDEYNINERGFHKRTIRSDDEYSSEKAFRCSCHDMHIFDSVGNGASSVVHRAIYVPVHR

VLALKKINIFEKERRQQILNEIITLSEACCYPGLVEFHGVFYTPDSGEIYFALEYMDGGS

LADIIRVKKFISEPVLSHMLQKVLLALRYLHEVRHLVHRDIKPANLLLNLKGDTKITDFG

VTSGLHDSIDMCATFLGSVTYMSPERIRNESYSYSADIWSLGLTALECATGRYPYDVNGG

EADLMLQILEDPSPTPPHDIYSEEFCSFINACLQKDADARPTCDQLLSHSFIKRYEGPGV

DLSEYNKSVHDPSERLSQIAHMLAVHYYLIFDGGDDQWCHMKTFYQQDSIFSFSGETHVG

KSEIFETLSRIRKMLKGNSPCEKIAHVMEKVYCRSHGEEGMRVRVSGSFIVGNEFVVCAD

GVRAEGMLSIDELSPDILSKQAGHFQEDFFMEPGTALGCYVISKQELHIADT*

>Sig2MKK4 Brdisv1Sig21036157m.p

MAHPTALCPQAPRAPRQVLERASYMAFSGTTHAATTCSGLHPPGPPLPRNRFCAYGPPCE

CTSAHPARQVLSGIASLEGRRIVHRDIKPSNLLIDSGRRVKIADFGVGRILNQTMDPCNS

SVGTIAYMSPERINTDLNDGAYDGYAGDIWSFGLSILEFYLGRFPLGENLGKQGDWAALM

CAICYSDSPAPPPIASPEFKSFISCCLQKNPARRPSAAQLLQHRFIAGPQPQVLAAPPS*

>Sig2MKK5 Brdisv1Sig21007006m.p

MRPAGSLPSPQPGTPGRPRRRPDLTLPMPQRPDVSSSLAVPLPLPPPSSLGLAQPPAAAA

AAAAPPPPPLGELERVRRVGSGAGGTVWMVRHRPTGRCYALKQLYGNHDDAVRRQIAREI

AILRTAEHPAVVRCHGMYERGGELQILLEYMDGGSLDGRRIAAEGFLADVARQVLSGIAY

LHRRHIVHRDIKPSNLLIDSARRVKIADFGVGRILNQTMDPCNSSVGTIAYMSPERINTD

LNDGAYDGYAGDIWSFGLSILEFYLGRFPFGENLGKQGDWAALMVAICYNDPPEPSAAAS

PEFRGFISCCLQKNPAKRLSAAQLLQHPFVAGPQPLPLAAPPS*

>Sig2MKK6 Brdisv1Sig21011649m.p

MRGKKPLKELKLSVPAQETSVDKFLTASGTFKDGELRLNQRGLRLISEEENGDEHQSTNM

KVEDVQLSMDDLEMIQVIGKGSGGVVQLVQHKWVGTFYALKGIQMNIQEAVRKQIVQELK

INQATQSPHIVSCHQSFYHNGVIYLVLEYMDRGSLADIIKQVKTILEPYLAVLCKQVLEG

LLYLHHERHVIHRDIKPSNLLVNHKGEVKITDFGVSAVLASSIGQRDTFVGTYNYMAPER

ISGSSYDYKSDVWSLGLVILECAIGRFPYTPSEGEGWLSFYELLEAIVDQPPPGAPADQF

SPEFCSFISACIQKDPAERMSASELLNHAFIKKFEGKDLDLRILVESLEPPMNVPE*

>Sig2MKK10-1 Brdisv1Sig21001691m.p

MALLREKRLQLSLHVPTRAADAQEAGLHRRPNPAAALPLAATTPAARSSQFRVADFEKLA

VLGRGNGGTVYKVRHRETCELYALKVQHCNGDATAEAEVLSRTASPFVVRCHSVLPAAAS

GDVAMLLELVDGGSLDSIVKSRSRGQAEAFSQFPEEALAEVAAQALSGLAYLHARRIVHL

DVKPGNLLVSTGGEVKIADFGIARVLPRAGGDDVRCTAYAGTAAYMSPERFDPEAHGGHY

DPYAADVWGLGVTVLELLMGRYPLLPAGQRPSWAALMCAICFGETPALSDGEASAELRGF

VAACLHKDYRRRASVAELLAHPFVAGRDVAASKCALRKLVTEASMSP*

>Sig2MKK10-2 Brdisv1Sig21010766m.p

MALVRQRRQLPHLTLPLDHFALRPPPVPAPAPTVAASTSSEAAGLRLSDFERISLLGQGN

GGTVYKARHRRAAAQPPVALKLFVAGDPSAAREAEILRLAADAPHVVRLHAVVPSSSPAA

GAEQPPPAALALELLPGGSLAGLLRRLGRSMGERPIAAVARQALLGLDALHALRVVHRDL

KPSNLLLGSHGEVKIADFGAGKVLRRRLDPCASYVGTAAYMSPERFDPEAYSGDYDPYAA

DVWSLGLAILELYLGHFPLLPAGQRPDWAALMCAICFGDAPEAPAAASEEFRDFVARCLE

KKAGQRASVAELLEHPFIAERDAEEAKRALAALVAEAELGDL*

>Sig2MKK10-3 Brdisv1Sig21001588m.p

MALLREKRLQLSLHVPTRAAEALDAVHRRPNPVAATLAASTPAAARSSQFRLADFDKLTV

LGRGNGGTVYKVRHRETCELYALKVQHCNGDPTAAAEAEVLSRTASPFIVRCHSVLPGAA

SGDVAMLLELVDGGSLDSIVKSRRAHAFPFPEEALAEVAAQALSGLAYLHARRIVHLDIK

PGNLLVSTGGEVKVADFGIAKVLPRAGADDARCKSYAGTAAYMSPERFDPEAHGGHYDAY

AADVWGLGVTVLELLMGRYPLLPAGQRPSWPALMCAICFGETPVLSDGEASAELRGFVAA

CLRKDHTKRASVAELLAHPFVAGRDVATSKCALRKLVTEASTSP*

>Sig2MKK10-4 Brdisv1Sig21001585m.p

MASAKERRLPQLHLKLDVPTCAFRCAAPAPAPATAATPATSASRPPHGEFRLNDFDRLSV

LGRGNGGSVYKVSHRRTSALYALKIIHGAHARPGAADEEADIVRRVVDSPNVVRCHSVLP

TASGDAAALLLELVDGGSLDSLVGGGGFLPEAAVADVAAQALSGLAHLRARRVAHRDIKP

ANLLLSAAGEVKIADFGIAKVVVSGAGGRARALAYEGTVAYMSPERFDSERHADADPXGR

KT

>Sig2MKK10-5 Brdisv1Sig21045238m.p

MTQAGKGGFLAGTGAPDLGVSLTTGGSFHGRPSARTTMRSRCASVHESMVTERTKLMCAG

SSVVAGGGGDLRGVVCGGRQRRLWWGSTTSLFAGRKGHLCRHRQRCVPVQSNSHSRVLLP

FPSGRSRRLHSAALLCRASPPRLLAPAIRATRHHHLPRLAAVGHTTSVGGAAASRARRPP

CFPVGGAAASRARRNRRPAPLLQRPPPTSASAATDSPRQQTPPSPPPAHDCPDAESSRPV

AAPFLRRPHFPXVVHRDIKPANLLVSGAGGVKVADFGIAMVLPSRAGGERCAAAYEGTVA

YMSPERFDSEGRADADPRGADVWGLGVTVLELLMGRYPLLPAGQKPTWAALMCAICFGEL

PALPEGAASTELRGFIAACLRKDHTKRASVAELIKHPFVAGRNMAASRLALRRLVAGA*

>ABR2MKK1 Brdisv1ABR21008364m.p

MRKPGKLALPSHESTIGKFLTQSGTFKDGDLLVNKDGLRIVHNSEEGEAPPIEPLDDHQL

SLDDLDAIKVIGKGSSGIVQLVRHKWTDQFFALKVIQLNIQESIRKQIAQELKISLSTQC

QYVVTCYQCFYVNGVISIVLEYMDGGSLADFLKTVRTIPEAYLAAICKQVLQGLMYLHHE

KRVIHRDLKPSNILINHRGEVKISDFGVSAIIASSSAQRDTFTGTFNYMAPERISGQKHG

YMSDIWSLGLVMLECATGNFPYPSPDSFYELLEAVVDQPPPSAPTDQFSPEFCSFISACI

QKEATDRSSAQVLSDHPFLSMYDDLNIDLADYFTTAGSPLATFKQIVL*

>ABR2MKK3-1 Brdisv1ABR21029725m.p

MAGLEELKKKLQPLLFDDPDKDGISTRVPFLEDNCDSYVVSDGGTINLLSRSFGEYNINE

HGFHKRSTGADESDFGEKAYRCASHDMHIFGPIGNGASSVVQRAIFIPVHRILALKKINI

FEKEKRQQILNEMRTLCEASCYPGLVEFQGAFYMPDSGQISIALEYMDGGSLADVIKVKK

SIPEQVLAHMLQKVLLGLRYLHEVRHLVHRDIKPANMLVNLKGEAKITDFGVSAGLDNTM

AMCATFVGTVTYMSPERIRNENYSYAADIWSLGLTILECATGKFPYNVNEGPANLMLQIL

DDPSPTPPADAYSPEFCSFVNDCLQKDPDARPTCEQLFGHPFIKRYENAGVDLIAYVKGV

VDPTERLKEIAEMLAVHYYLLFNGSDGLWHHMKTFYMEESTFSFSGNVYVGRNDIFDTLS

SIRKKLKGDRPREKIVHVVEKLHCRANGETGIAIRVSGSLIVGNQFLVCGEGLQAEGMPS

VEELSIDIPSKRVGQFREQFMMLPGISMGSFHISRQDLYIIQA*

>ABR2MKK3-2 Brdisv1ABR21006726m.p

MAGLEELKKKLQPLMFNDPDKDGFSTRVPFPEDTCDSYVVSDGGTINLLSRSFGEYNINE

HGFHKRSAGADESDFGEKAYRCASQDMHIFGPIGNGASSVVQRAIFIPVHRILALKKINI

FEKEKRQQILNEMRTLCEACCYPGLVEFQGAFYMPDSGQISIALEYMDGGSLADVIKVKK

SIPEPVLAHMLQKVLLGLRYLHEVRHLVHRDIKPANMLVNLKGEAKITDFGVSAGLDNTM

AMCATFVGTVTYMSPERIRNENYSYAADIWSLGLTILECATGKFPYNVNEGPANLMLQIL

DDPSPAPPENAFSSEFCSFVNDCLQKDADARPTCEQLLSHPFIKRYENAGVDLAAYVKGV

VNPEERLKQIAEMLAVHYYLLFNGSDGLWHHMKTFYMEDSTFSFSGNLYVGQSDIFDTLS

NIRTKLKGDRPREKIVHVVEKLHCRANEETGIAIRVSGSFIVSNQFLICGEGLQAEGMPS

LEELSIDIPSKRVGQFREQFIMHPGRSMGCYYISRQDLYIIQA*

>ABR2MKK3-3 Brdisv1ABR21032775m.p

MGWDGMGQRKRRHRAGIPIPTYPGLAASLEFCFVTAGACHTAPGPGSKIAASLFAALLFQ

RPPASTSRAIFFCFVIMAAGLEDLRRRVQPIFFDADGNVMPAPDDDSEVLDGGTINLLSR

SSDEYNINERGFHKRTIRSDDEYSSEKAFRCSCHDMHIFDSVGNGASSVVHRAIYVPVHR

VLALKKINIFEKERRQQILNEIITLSEACCYPGLVEFHGVFYTPDSGEIYFALEYMDGGS

LADIIRVKKFISEPVLSHMLQKVLLALRYLHEVRHLVHRDIKPANLLLNLKGDTKITDFG

VTSGLHDSIDMCATFLGSVTYMSPERIRNESYSYSADIWSLGLTALECATGRYPYDVNGG

EADLMLQILEDPSPTPPHDIYSEEFCSFINACLQKDADARPTCDQLLSHSFIKRYEGPGV

DLSEYNKSVHDPSERLSQIAHMLAVHYYLIFDGGDDQWCHMKTFYQQDSIFSFSGETHVG

KSEIFETLSRIRKMLKGNSPCEKIAHVMEKVYCRSHGEEGMRVRVSGSFIVGNEFVVCAD

GVRAEGMLSIDELSPDILSKQAGHFQEDFFMEPGTALGCYVISKQELHIADT*

>ABR2MKK4 Brdisv1ABR21039238m.p

MRPGGPPNARPQQPGTPGRARRRPDLTLPLPQRDLTSLAVPLPLPPPPSSAPSSASSSGS

SLSSMGAPTPPNSAGSAPPPPPPLAELERVRRIGSGAGGTVWMVRHRPTGRPYALKVLYG

NHDDAVRRQITREIAILRTAEHPAIVRCHGMYEQAGELQILLEFMDGGSLEGRRIASEAF

LADVARQVLSGIAYLHRRHIVHRDIKPSNLLIDSGRRVKIADFGVGRILNQTMDPCNSSV

GTIAYMSPERINTDLNDGAYDGYAGDIWSFGLSILEFYLGRFPLGENLGKQGDWAALMCA

ICYSDSPAPPPIASPEFKSFISCCLQKNPARRPSAAQLLQHRFIAGPQPQVLAAPPS*

>ABR2MKK5 Brdisv1ABR21007673m.p

MRPAGSLPSPQPGTPGRPRRRPDLTLPMPQRPDVSSSLAVPLPLPPPSSLGLAQPPAAAA

AAAAPPPPPLGELERVRRVGSGAGGTVWMVRHRPTGRCYALKQLYGNHDDAVRRQIAREI

AILRTAEHPAVVRCHGMYERGGELQILLEYMDGGSLDGRRIAAEGFLADVARQVLSGIAY

LHRRHIVHRDIKPSNLLIDSARRVKIADFGVGRILNQTMDPCNSSVGTIAYMSPERINTD

LNDGAYDGYAGDIWSFGLSILEFYLGRFPFGENLGKQGDWAALMVAICYNDPPEPSAAAS

PEFRGFISCCLQKNPAKRLSAAQLLQHPFVAGPQPLPLAAPPS*

>ABR2MKK6 Brdisv1ABR21012563m.p

MRGKKPLKELKLSVPAQETSVDKFLTASGTFKDGELRLNQRGLRLISEEENGDEHQSTNM

KVEDVQLSMDDLEMIQVIGKGSGGVVQLVQHKWVGTFYALKGIQMNIQEAVRKQIVQELK

INQATQSPHIVSCHQSFYHNGVIYLVLEYMDRGSLADIIKQVKTILEPYLAVLCKQVLEG

LLYLHHERHVIHRDIKPSNLLVNHKGEVKITDFGVSAVLASSIGQRDTFVGTYNYMAPER

ISGSSYDYKSDVWSLGLVILECAIGRFPYTPSEGEGWLSFYELLEAIVDQPPPGAPEDQF

SPEFCSFISACIQKDPAERMSASELLNHAFIKKFEGKDLDLRILVESLEPPMNVPE*

>ABR2MKK10-1 Brdisv1ABR21001840m.p

MALLREKRLQLSLHVPTRAADAQEAGLHRRPNPAAALPLAATTPAARSSQFRVADFEKLA

VLGRGNGGTVYKVRHRETCELYALKVQHCNGDATAEAEVLSRTASPFVVRCHSVLPAAAS

GDVAMLLELVDGGSLDSIVKSRSRGQAEAFSQFPEEALAEVAAQALSGLAYLHARRIVHL

DVKPGNLLVSTGGEVKIADFGIARVLPRAGGDDVRCTAYAGTAAYMSPERFDPEAHGGHY

DPYAADVWGLGVTVLELLMGRYPLLPAGQRPSWAALMCAICFGETPALSDGEASAELRGF

VAACLHKDYRRRASVAELLAHPFVAGRDVAASKCALRKLVTEASMSP*

>ABR2MKK10-2 Brdisv1ABR21011605m.p

MALVRQRRQLPHLTLPLDHFALRPPPVPAPAPTVAASTSSEAAGLRLSDFERISLLGQGN

GGTVYKARHRRAAAQPPVALKLFVAGDPSAAREAEILRLAADAPHVVRLHAVVPSSSPAA

GAEQPPPAALALELLPGGSLAGLLRRLGRSMGERPIAAVARQALLGLDALHALRVVHRDL

KPSNLLLGSHGEVKIADFGAGKVLRRRLDPCASYVGTAAYMSPERFDPEAYSGDYDPYAA

DVWSLGLAILELYLGHFPLLPAGQRPDWAALMCAICFGDAPEAPAAASEEFRDFVARCLE

KKAGQRASVAELLEHPFIAERDAEEAKRALAALVAEAELGDL*

>ABR2MKK10-3 Brdisv1ABR21001713m.p

MALLREKRLQLSLHVPTRAAEALDAVHRRPNPVAATLAASTPAAARSSQFRLADFDKLTV

LGRGNGGTVYKVRHRETCELYALKVQHCNGDPTAAAEAEVLSRTASPFIVRCHSVLPGAA

SGDVAMLLELVDGGSLDSIVKSRRAHAFPFPEEALAEVAAQALSGLAYLHARRIVHLDIK

PGNLLVSTGGEVKVADFGIAKVLPRAGADDARCKSYAGTAAYMSPERFDPEAHGGHYDAY

AADVWGLGVTVLELLMGRYPLLPAGQRPSWPALMCAICFGETPVLSDGEASAELRGFVAA

CLRKDHTKRASVAELLAHPFVAGRDVATSKCALRKLVTEASTSP*

>ABR2MKK10-4 Brdisv1ABR21001710m.p

MASAKERRLPQLHLKLDVPTCAFRCAAPAPAPATAATPATSASRPPHGEFRLNDFDRLSV

LGRGNGGSVYKVSHRRTSALYALKIIHGAHARPGAADEEADIVRRVVDSPNVVRCHSVLP

TASGDAAALLLELVDGGSLDSLVGGGGFLPEAAVADVAAQALSGLAHLRARRVAHRDIKP

ANLLLSAAGEVKIADFGIAKVVVSGAGGRARALAYEGTVAYMSPERFDSERHADADPYAA

DVWGLGVTLLELLMGRYPLLPAGQKPTWAALMCAICFGELPALPEGAASLEFRGFVAACL

RKDHRKRASVVELLAHPFVAGRDVAASRRALREAIERRCSC*

>ABR2MKK10-5 Brdisv1ABR21001712m.p

MALTVRQRRLPQLHISLDLPSCSFRCPNPPVAATASTSGEFRASDFEPLAVLGRGNGGTV

YKVAHRRTSAQYALKVLHGGGDPGAAAAEADVLRRAADSPYVVRCHSVFPAASGSGETAL

LLELVDGGSLDSVRRGVGVSVFFPEAALAEVAAQALAGLAHLHARRVVHRDIKPANLLVS

GAGGVKVADFGIAMVLPSRAGGERCAAAYEGTVAYMSPERFDSEGRADADPRGADVWGLG

VTVLELLMGRYPLLPAGQKPTWAALMCAICFGELPALPEGAASTELRGFIAACLRKDHTK

RASVAELIKHPFVAGRNMAASRLALRRLVAGA*

>ABR3MKK1 Brdisv1ABR31008332m.p

MRKPGKLALPSHESTIGKFLTQSGTFKDGDLLVNKDGLRIVHNSEEGEAPPIEPLDDHQL

SLDDLDAIKVIGKGSSGIVQLVRHKWTDQFFALKVIQLNIQESIRKQIAQELKISLSTQC

QYVVTCYQCFYVNGVISIVLEYMDGGSLADFLKTVRTIPEAYLAAICKQASETCHTRNQF

CYWNVLQGLMYLHHEKRVIHRDLKPSNILINHRGEVKISDFGVSAIIASSSAQRDTFTGT

FNYMAPERISGQKHGYMSDIWSLGLVMLECATGNFPYPSPDSFYELLEAVVDQPPPSAPT

DQFSPEFCSFISACIQKEATDRSSAQVLSDHPFLSMYDDLNIDLADYFTTAGSPLATFKQ

IVL*

>ABR3MKK3-1 Brdisv1ABR31039196m.p

MAGLEELKKKLQPLLFDDPDKDGISTRVPFLEDNCDSYVVSDGGTINLLSRSFGEYNINE

HGFHKRSTGADESDFGEKAYRCASHDMHIFGPIGNGASSVVQRAIFIPVHRILALKKINI

FEKEKRQQILNEMRTLCEASCYPGLVEFQGAFYMPDSGQISIALEYMDGGSLADVIKVKK

SIPEQVLAHMLQKVLLGLRYLHEVRHLVHRDIKPANMLVNLKGEAKITDFGVSAGLDNTM

AMCATFVGTVTYMSPERIRNENYSYAADIWSLGLTILECATGKFPYNVNEGPANLMLQIL

DDPSPTPPADAYSPEFCSFVNDCLQKDPDARPTCEQLFGHPFIKRYENAGVDLIAYVKGV

VDPTERLKEIAEMLAVHYYLLFNGSDGLWHHMKTFYMEESTFSFSGNVYVGRNDIFDTLS

SIRKKLKGDRPREKIVHVVEKLHCRANGETGIAIRVSGSLIVGNQFLVCGEGLQAEGMPS

VEELSIDIPSKRVGQFREQFMMLPGISMGSFHISRQDLYIIQA*

>ABR3MKK3-2 Brdisv1ABR31006634m.p

MAGLEELKKKLQPLMFNDPDKDGFSTRVPFPEDTCDSYVVSDGGTINLLSRSFGEYNINE

HGFHKRSAGADESDFGEKAYRCASQDMHIFGPIGNGASSVVQRAIFIPVHRILALKKINI

FEKEKRQQILNEMRTLCEACCYPGLVEFQGAFYMPDSGQISIALEYMDGGSLADVIKVKK

SIPEPVLAHMLQKVLLGLRYLHEVRHLVHRDIKPANMLVNLKGEAKITDFGVSAGLDNTM

AMCATFVGTVTYMSPERIRNENYSYAADIWSLGLTILECATGKFPYNVNEGPANLMLQIL

DDPSPAPPENAFSSEFCSFVNDCLQKDADARPTCEQLLSHPFIKRYENAGVDLAAYVKGV

VNPEERLKQIAEMLAVHYYLLFNGSDGLWHHMKTFYMEDSTFSFSGNLYVGQSDIFDTLS

NIRTKLKGDRPREKIVHVVEKLHCRANEETGIAIRVSGSFIVSNQFLICGEGLQAEGMPS

LEELSIDIPSKRVGQFREQFIMHPGRSMGCYYISRQDLYIIQA*

>ABR3MKK3-3 Brdisv1ABR31025404m.p

MGWDGMGQRKRRHRAGIPIPTYPGLAASLEFCFVTAGACHTAPGPGSKIAASLFAALLFQ

RPPASTSRAIFFCFVIMAAGLEDLRRRVQPIFFDADGNVMPAPDDDSEVLDGGTINLLSR

SSDEYNINERGFHKRTIRSDDEYSSEKAFRCSCHDMHIFDSVGNGASSVVHRAIYVPVHR

VLALKKINIFEKERRQQILNEIITLSEACCYPGLVEFHGVFYTPDSGEIYFALEYMDGGS

LADIIRVKKFISEPVLSHMLQKVLLALRYLHEVRHLVHRDIKPANLLLNLKGDTKITDFG

VTSGLHDSIDMCATFLGSVTYMSPERIRNESYSYSADIWSLGLTALECATGRYPYDVNGG

EADLMLQILEDPSPTPPHDIYSEEFCSFINACLQKDADARPTCDQLLSHSFIKRYEGPGV

DLSEYNKSVHDPSERLSQIAHMLAVHYYLIFDGGDDQWCHMKTFYQQDSIFSFSGETHVG

KSEIFETLSRIRKMLKGNSPCEKIAHVMEKVYCRSHGEEGMRVRVSGSFIVGNEFVVCAD

GVRAEGMLSIDELSPDILSKQAGHFQEDFFMEPGTALGCYVISKQELHIADT*

>ABR3MKK4 Brdisv1ABR31037814m.p

MRPGGPPNARPQQPGTPGRARRRPDLTLPLPQRDLTSLAVPLPLPPPPSSAPSSASSSGS

SLSSMGAPTPPNSAGSAPPPPPPLAELERVRRIGSGAGGTVWMVRHRPTGRPYALKVLYG

NHDDAVRRQITREIAILRTAEHPAIVRCHGMYEQAGELQILLEFMDGGSLEGRRIASEAF

LADVARQVLSGIAYLHRRHIVHRDIKPSNLLIDSGRRVKIADFGVGRILNQTMDPCNSSV

GTIAYMSPERINTDLNDGAYDGYAGDIWSFGLSILEFYLGRFPLGENLGKQGDWAALMCA

ICYSDSPAPPPIASPEFKSFISCCLQKNPARRPSAAQLLQHRFIAGPQPQVLAAPPS*

>ABR3MKK5 Brdisv1ABR31007650m.p

MRPAGSLPSPQPGTPGRPRRRPDLTLPMPQRPDVSSSLAVPLPLPPPSSLGLAQPPAAAA

AAAAPPPPPLGELERVRRVGSGAGGTVWMVRHRPTGRCYALKQLYGNHDDAVRRQIAREI

AILRTAEHPAVVRCHGMYERGGELQILLEYMDGGSLDGRRIAAEGFLADVARQVLSGIAY

LHRRHIVHRDIKPSNLLIDSARRVKIADFGVGRILNQTMDPCNSSVGTIAYMSPERINTD

LNDGAYDGYAGDIWSFGLSILEFYLGRFPFGENLGKQGDWAALMVAICYNDPPEPSAAAS

PEFRGFISCCLQKNPAKRLSAAQLLQHPFVAGPQPLPLAAPPS*

>ABR3MKK6 Brdisv1ABR31012480m.p

MRGKKPLKELKLSVPAQETSVDKFLTASGTFKDGELRLNQRGLRLISEEENGDEHQSTNM

KVEDVQLSMDDLEMIQVIGKGSGGVVQLVQHKWVGTFYALKGIQMNIQEAVRKQIVQELK

INQATQSPHIVSCHQSFYHNGVIYLVLEYMDRGSLADIIKQVKTILEPYLAVLCKQVLEG

LLYLHHERHVIHRDIKPSNLLVNHKGEVKITDFGVSAVLASSIGQRDTFVGTYNYMAPER

ISGSSYDYKSDVWSLGLVILECAIGRFPYTPSEGEGWLSFYELLEAIVDQPPPGAPADQF

SPEFCSFISACIQKDPAERMSASELLNHAFIKKFEGKDLDLRILVESLEPPMNVPE*

>ABR3MKK10-1 Brdisv1ABR31001837m.p

MALLREKRLQLSLHVPTRAADAQEAGLHRRPNPAAALPLAATTPAARSSQFRVADFEKLA

VLGRGNGGTVYKVRHRETCELYALKVQHCNGDATAEAEVLSRTASPFVVRCHSVLPAAAS

GDVAMLLELVDGGSLDSIVKSRSRGQAEAFSQFPEEALAEVAAQALSGLAYLHARRIVHL

DVKPGNLLVSTGGEVKIADFGIARVLPRAGGDDVRCTAYAGTAAYMSPERFDPEAHGGHY

DPYAADVWGLGVTVLELLMGRYPLLPAGQRPSWAALMCAICFGETPALSDGEASAELRGF

VAACLHKDYRRRASVAELLAHPFVAGRDVAASKCALRKLVTEASMSP*

>ABR3MKK10-2 Brdisv1ABR31011561m.p

MALVRQRRQLPHLTLPLDHFALRPPPVPAPAPTVAASTSSEAAGLRLSDFERISLLGQGN

GGTVYKARHRRAAAQPPVALKLFVAGDPSAAREAEILRLAADAPHVVRLHAVVPSSSPAA

GAEQPPPAALALELLPGGSLAGLLRRLGRSMGERPIAAVARQALLGLDALHALRVVHRDL

KPSNLLLGSHGEVKIADFGAGKVLRRRLDPCASYVGTAAYMSPERFDPEAYSGDYDPYAA

DVWSLGLAILELYLGHFPLLPAGQRPDWAALMCAICFGDAPEAPAAASEEFRDFVARCLE

KKAGQRASVAELLEHPFIAERDAEEAKRALAALVAEAELGDL*

>ABR3MKK10-3 Brdisv1ABR31001729m.p

MALLREKRLQLSLHVPTRAAEALDAVHRRPNPVAATLAASTPAAARSSQFRLADFDKLTV

LGRGNGGTVYKVRHRETCELYALKVQHCNGDPTAAAEAEVLSRTASPFIVRCHSVLPGAA

SGDVAMLLELVDGGSLDSIVKSRRAHAFPFPEEALAEVAAQALSGLAYLHARRIVHLDIK

PGNLLVSTGGEVKVADFGIAKVLPRAGADDARCKSYAGTAAYMSPERFDPEAHGGHYDAY

AADVWGLGVTVLELLMGRYPLLPAGQRPSWPALMCAICFGETPVLSDGEASAELRGFVAA

CLRKDHTKRASVAELLAHPFVAGRDVATSKCALRKLVTEASTSP*

>ABR3MKK10-4 Brdisv1ABR31001726m.p

MASAKERRLPQLHLKLDVPTCAFRCAAPAPAPATAATPATSASRPPHGEFRLNDFDRLSV

LGRGNGGSVYKVSHRRTSALYALKIIHGAHARPGAADEEADIVRRVVDSPNVVRCHSVLP

TASGDAAALLLELVDGGSLDSLVGGGGFLPEAAVADVAAQALSGLAHLRARRVAHRDIKP

ANLLLSAAGEVKIADFGIAKVVVSGAGGRARALAYEGTVAYMSPERFDSERHADADPYAA

DVWGLGVTLLELLMGRYPLLPAGQKPTWAALMCAICFGELPALPEGAASLEFRGFVAACL

RKDHRKRASVVELLAHPFVAGRDVAASRRALREAIERRCSC*

>ABR3MKK10-5 Brdisv1ABR31001728m.p

MALTVRQRRLPQLHISLDLPSCSFRCPNPPVAATASTSGEFRASDFEPLAVLGRGNGGTV

YKVAHRRTSAQYALKVLHGGGDPGAAAAEADVLRRAADSPYVVRCHSVFPAASGSGETAL

LLELVDGGSLDSVRRGVGVSVFFPEAALAEVAAQALAGLAHLHARRVVHRDIKPANLLVS

GAGGVKVADFGIAMVLPSRAGGERCAAAYEGTVAYMSPERFDSEGRADADPRGADVWGLG

VTVLELLMGRYPLLPAGQKPTWAALMCAICFGELPALPEGAASTELRGFIAACLRKDHTK

RASVAELIKHPFVAGRNMAASRLALRRLVAGA*

>ABR4MKK1 Brdisv1ABR41008171m.p

MRKPGKLALPSHESTIGKFLTQSGTFKDGDLLVNKDGLRIVHNSEEGEAPPIEPLDDHQL

SLDDLDAIKVIGKGSSGIVQLVRHKWTDQFFALKVIQLNIQESIRKQIAQELKISLSTQC

QYVVTCYQCFYVNGVISIVLEYMDGGSLADFLKTVRTIPEAYLAAICKQVLQGLMYLHHE

KRVIHRDLKPSNILINHRGEVKISDFGVSAIIASSSAQRDTFTGTFNYMAPERISGQKHG

YMSDIWSLGLVMLECATGNFPYPSPDSFYELLEAVVDQPPPSAPTDQFSPEFCSFISACI

QKEATDRSSAQVLSDHPFLSMYDDLNIDLADYFTTAGSPLATFKQIVL*

>ABR4MKK3-1 Brdisv1ABR41029512m.p

MAGLEELKKKLQPLLFDDPDKDGISTRVPFLEDNCDSYVVSDGGTINLLSRSFGEYNINE

HGFHKRSTGADESDFGEKAYRCASHDMHIFGPIGNGASSVVQRAIFIPVHRILALKKINI

FEKEKRQQILNEMRTLCEASCYPGLVEFQGAFYMPDSGQISIALEYMDGGSLADVIKVKK

SIPEQVLAHMLQKVLLGLRYLHEVRHLVHRDIKPANMLVNLKGEAKITDFGVSAGLDNTM

AMCATFVGTVTYMSPERIRNENYSYAADIWSLGLTILECATGKFPYNVNEGPANLMLQIL

DDPSPTPPADAYSPEFCSFVNDCLQKDPDARPTCEQLFGHPFIKRYENAGVDLIAYVKGV

VDPTERLKEIAEMLAVHYYLLFNGSDGLWHHMKTFYMEESTFSFSGNVYVGRNDIFDTLS

SIRKKLKGDRPREKIVHVVEKLHCRANGETGIAIRVSGSLIVGNQFLVCGEGLQAEGMPS

VEELSIDIPSKRVGQFREQFMMLPGISMGSFHISRQDLYIIQA*

>ABR4MKK3-2 Brdisv1ABR41006591m.p

MAGLEELKKKLQPLMFNDPDKDGFSTRVPFPEDTCDSYVVSDGGTINLLSRSFGEYNINE

HGFHKRSAGADESDFGEKAYRCASQDMHIFGPIGNGASSVVQRAIFIPVHRILALKKINI

FEKEKRQQILNEMRTLCEACCYPGLVEFQGAFYMPDSGQISIALEYMDGGSLADVIKVKK

SIPEPVLAHMLQKVLLGLRYLHEVRHLVHRDIKPANMLVNLKGEAKITDFGVSAGLDNTM

AMCATFVGTVTYMSPERIRNENYSYAADIWSLGLTILECATGKFPYNVNEGPANLMLQIL

DDPSPAPPENAFSSEFCSFVNDCLQKDADARPTCEQLLSHPFIKRYENAGVDLAAYVKGV

VNPEERLKQIAEMLAVHYYLLFNGSDGLWHHMKTFYMEDSTFSFSGNLYVGQSDIFDTLS

NIRTKLKGDRPREKIVHVVEKLHCRANEETGIAIRVSGSFIVSNQFLICGEGLQAEGMPS

LEELSIDIPSKRVGQFREQFIMHPGRSMGCYYISRQDLYIIQA*

>ABR4MKK3-3 Brdisv1ABR41032504m.p

MDGGWDGMGQRKRRHRAGIPIPTYPGLAASLEFCFVTAGACHTAPGPGSKIAASLFAALL

FQRPPASTSRAIFFCFVIMAAGLEDLRRRVQPIFFDADGNVMPAPDDDSEVLDGGTINLL

SRSSDEYNINERGFHKRTIRSDDEYSSEKAFRCSCHDMHIFDSVGNGASSVVHRAIYVPV

HRVLALKKINIFEKERRQQILNEIITLSEACCYPGLVEFHGVFYTPDSGEIYFALEYMDG

GSLADIIRVKKFISEPVLSHMLQKVLLALRYLHEVRHLVHRDIKPANLLLNLKGDTKITD

FGVTSGLHDSIDMCATFLGSVTYMSPERIRNESYSYSADIWSLGLTALECATGRYPYDVN

GGEADLMLQILEDPSPTPPHDIYSEEFCSFINACLQKDADARPTCDQLLSHSFIKRYEGP

GVDLSEYNKSVHDPSERLSQIAHMLAVHYYLIFDGGDDQWCHMKTFYQQDSIFSFSGETH

VGKSEIFETLSRIRKMLKGNSPCEKIAHVMEKVYCRSHGEEGMRVRVSGSFIVGNEFVVC

ADGVRAEGMLSIDELSPDILSKQAGHFQEDFFMEPGTALGCYVISKQELHIADT*

>ABR4MKK4 Brdisv1ABR41038836m.p

MRPGGPPNARPQQPGTPGRARRRPDLTLPLPQRDLTSLAVPLPLPPPPSSAPSSASSSGS

SLSSMGAPTPPNSAGSAPPPPPPLAELERVRRIGSGAGGTVWMVRHRPTGRPYALKVLYG

NHDDAVRRQITREIAILRTAEHPAIVRCHGMYEQAGELQILLEFMDGGSLEGRRIASEAF

LADVARQVLSGIAYLHRRHIVHRDIKPSNLLIDSGRRVKIADFGVGRILNQTMDPCNSSV

GTIAYMSPERINTDLNDGAYDGYAGDIWSFGLSILEFYLGRFPLGENLGKQGDWAALMCA

ICYSDSPAPPPIASPEFKSFISCCLQKNPARRPSAAQLLQHRFIAGPQPQVLAAPPS*

>ABR4MKK5 Brdisv1ABR41007481m.p

MRPAGSLPSPQPGTPGRPRRRPDLTLPMPQRPDVSSSLAVPLPLPPPSSLGLAQPPAAAA

AAAAPPPPPLGELERVRRVGSGAGGTVWMVRHRPTGRCYALKQLYGNHDDAVRRQIAREI

AILRTAEHPAVVRCHGMYERGGELQILLEYMDGGSLDGRRIAAEGFLADVARQVLSGIAY

LHRRHIVHRDIKPSNLLIDSARRVKIADFGVGRILNQTMDPCNSSVGTIAYMSPERINTD

LNDGAYDGYAGDIWSFGLSILEFYLGRFPFGENLGKQGDWAALMVAICYNDPPEPSAAAS

PEFRGFISCCLQKNPAKRLSAAQLLQHPFVAGPQPLPLAAPPS*

>ABR4MKK6 Brdisv1ABR41012469m.p

MRGKKPLKELKLSVPAQETSVDKFLTASGTFKDGELRLNQRGLRLISEEENGDEHQSTNM

KVEDVQLSMDDLEMIQVIGKGSGGVVQLVQHKWVGTFYALKGIQMNIQEAVRKQIVQELK

INQATQSPHIVSCHQSFYHNGVIYLVLEYMDRGSLADIIKQVKTILEPYLAVLCKQVLEG

LLYLHHERHVIHRDIKPSNLLVNHKGEVKITDFGVSAVLASSIGQRDTFVGTYNYMAPER

ISGSSYDYKSDVWSLGLVILECAIGRFPYTPSEGEGWLSFYELLEAIVDQPPPGAPADQF

SPEFCSFISACIQKDPAERMSASELLNHAFIKKFEGKDLDLRILVESLEPPMNVPE*

>ABR4MKK10-1 Brdisv1ABR41001806m.p

MALLREKRLQLSLHVPTRAADAQEAGLHRRPNPAAALPLAATTPAARSSQFRVADFEKLA

VLGRGNGGTVYKVRHRETCELYALKVQHCNGDATAEAEVLSRTASPFVVRCHSVLPAAAS

GDVAMLLELVDGGSLDSIVKSRSRGQAEAFSQFPEEALAEVAAQALSGLAYLHARRIVHL

DVKPGNLLVSTGGEVKIADFGIARVLPRAGGDDVRCTAYAGTAAYMSPERFDPEAHGGHY

DPYAADVWGLGVTVLELLMGRYPLLPAGQRPSWAALMCAICFGETPALSDGEASAELRGF

VAACLHKDYRRRASVAELLAHPFVAGRDVAASKCALRKLVTEASMSP*

>ABR4MKK10-2 Brdisv1ABR41011563m.p

MALVRQRRQLPHLTLPLDHFALRPPPVPAPAPTVAASTSSEAAGLRLSDFERISLLGQGN

GGTVYKARHRRAAAQPPVALKLFVAGDPSAAREAEILRLAADAPHVVRLHAVVPSSSPAA

GAEQPPPAALALELLPGGSLAGLLRRLGRSMGERPIAAVARQALLGLDALHALRVVHRDL

KPSNLLLGSHGEVKIADFGAGKVLRRRLDPCASYVGTAAYMSPERFDPEAYSGDYDPYAA

DVWSLGLAILELYLGHFPLLPAGQRPDWAALMCAICFGDAPEAPAAASEEFRDFVARCLE

KKAGQRASVAELLEHPFIAERDAEEAKRALAALVAEAELGDL*

>ABR4MKK10-3 Brdisv1ABR41001706m.p

MALLREKRLQLSLHVPTRAAEALDAVHRRPNPVAATLAASTPAAARSSQFRLADFDKLTV

LGRGNGGTVYKVRHRETCELYALKVQHCNGDPTAAAEAEVLSRTASPFIVRCHSVLPGAA

SGDVAMLLELVDGGSLDSIVKSRRAHAFPFPEEALAEVAAQALSGLAYLHARRIVHLDIK

PGNLLVSTGGEVKVADFGIAKVLPRAGADDARCKSYAGTAAYMSPERFDPEAHGGHYDAY

AADVWGLGVTVLELLMGRYPLLPAGQRPSWPALMCAICFGETPVLSDGEASAELRGFVAA

CLRKDHTKRASVAELLAHPFVAGRDVATSKCALRKLVTEASTSP*

>ABR4MKK10-4 Brdisv1ABR41001703m.p

MASAKERRLPQLHLKLDVPTCAFRCAAPAPAPATAATPATSASRPPHGEFRLNDFDRLSV

LGRGNGGSVYKVSHRRTSALYALKIIHGAHARPGAADEEADIVRRVVDSPNVVRCHSVLP

TASGDAAALLLELVDGGSLDSLVGGGGFLPEAAVADVAAQALSGLAHLRARRVAHRDIKP

ANLLLSAAGEVKIADFGIAKVVVSGAGGRARALAYEGTVAYMSPERFDSERHADADPYAA

DVWGLGVTLLELLMGRYPLLPAGQKPTWAALMCAICFGELPALPEGAASLEFRGFVAACL

RKDHRKRASVVELLAHPFVAGRDVAASRRALREAIERRCSC*

>ABR4MKK10-5 Brdisv1ABR41001705m.p

MALTVRQRRLPQLHISLDLPSCSFRCPNPPVAATASTSGEFRASDFEPLAVLGRGNGGTV

YKVAHRRTSAQYALKVLHGGGDPGAAAAEADVLRRAADSPYVVRCHSVFPAASGSGETAL

LLELVDGGSLDSVRRGVGVSVFFPEAALAEVAAQALAGLAHLHARRVVHRDIKPANLLVS

GAGGVKVADFGIAMVLPSRAGGERCAAAYEGTVAYMSPERFDSEGRADADPRGADVWGLG

VTVLELLMGRYPLLPAGQKPTWAALMCAICFGELPALPEGAASTELRGFIAACLRKDHTK

RASVAELIKHPFVAGRNMAASRLALRRLVAGA*

>ABR5MKK1 Brdisv1ABR51007823m.p

MRKPGKLALPSHESTIGKFLTQSGTFKDGDLLVNKDGLRIVHNSEEGEAPPIEPLDDHQL

SLDDLDAIKVIGKGSSGIVQLVRHKWTDQFFALKVIQLNIQESIRKQIAQELKISLSTQC

QYVVTCYQCFYVNGVISIVLEYMDGGSLADFLKTVRTIPEAYLAAICKQVLQGLMYLHHE

KRVIHRDLKPSNILINHRGEVKISDFGVSAIIASSSAQRDTFTGTFNYMAPERISGQKHG

YMSDIWSLGLVMLECATGNFPYPSPDSFYELLEAVVDQPPPSAPTDQFSPEFCSFISACI

QKEATDRSSAQVLSDHPFLSMYDDLNIDLADYFTTAGSPLATFKQIVL*

>ABR5MKK3-1 Brdisv1ABR51028122m.p

MAGLEELKKKLQPLLFDDPDKDGISTRVPFLEDNCDSYVVSDGGTINLLSRSFGEYNINE

HGFHKRSTGADESDFGEKAYRCASHDMHIFGPIGNGASSVVQRAIFIPVHRILALKKINI

FEKEKRQQILNEMRTLCEASCYPGLVEFQGAFYMPDSGQISIALEYMDGGSLADVIKVKK

SIPEQVLAHMLQKVLLGLRYLHEVRHLVHRDIKPANMLVNLKGEAKITDFGVSAGLDNTM

AMCATFVGTVTYMSPERIRNENYSYAADIWSLGLTILECATGKFPYNVNEGPANLMLQIL

DDPSPTPPADAYSPEFCSFVNDCLQKDPDARPTCEQLFGHPFIKRYENAGVDLIAYVKGV

VDPTERLKEIAEMLAVHYYLLFNGSDGLWHHMKTFYMEESTFSFSGNVYVGRNDIFDTLS

SIRKKLKGDRPREKIVHVVEKLHCRANGETGIAIRVSGSLIVGNQFLVCGEGLQAEGMPS

VEELSIDIPSKRVGQFREQFMMLPGISMGSFHISRQDLYIIQA*

>ABR5MKK3-2 Brdisv1ABR51006276m.p

MAGLEELKKKLQPLMFNDPDKDGFSTRVPFPEDTCDSYVVSDGGTINLLSRSFGEYNINE

HGFHKRSAGADESDFGEKAYRCASQDMHIFGPIGNGASSVVQRAIFIPVHRILALKKINI

FEKEKRQQILNEMRTLCEACCYPGLVEFQGAFYMPDSGQISIALEYMDGGSLADVIKVKK

SIPEPVLAHMLQKVLLGLRYLHEVRHLVHRDIKPANMLVNLKGEAKITDFGVSAGLDNTM

AMCATFVGTVTYMSPERIRNENYSYAADIWSLGLTILECATGKFPYNVNEGPANLMLQIL

DDPSPAPPENAFSSEFCSFVNDCLQKDADARPTCEQLLSHPFIKRYENAGVDLAAYVKGV

VNPEERLKQIAEMLAVHYYLLFNGSDGLWHHMKTFYMEDSTFSFSGNLYVGQSDIFDTLS

NIRTKLKGDRPREKIVHVVEKLHCRANEETGIAIRVSGSFIVSNQFLICGEGLQAEGMPS

LEELSIDIPSKRVGQFREQFIMHPGRSMGCYYISRQDLYIIQA*

>ABR5MKK3-3 Brdisv1ABR51030947m.p

MGWDGMGQRKRRHRAGIPIPTYPGLAASLEFCFVTAGACHTAPGPGSKIAASLFAALLFQ

RPPASTSRAIFFCFVIMAAGLEDLRRRVQPIFFDADGNVMPAPDDDSEVLDGGTINLLSR

SSDEYNINERGFHKRTIRSDDEYSSEKAFRCSCHDMHIFDSVGNGASSVVHRAIYVPVHR

VLALKKINIFEKERRQQILNEIITLSEACCYPGLVEFHGVFYTPDSGEIYFALEYMDGGS

LADIIRVKKFISEPVLSHMLQKVLLALRYLHEVRHLVHRDIKPANLLLNLKGDTKITDFG

VTSGLHDSIDMCATFLGSVTYMSPERIRNESYSYSADIWSLGLTALECATGRYPYDVNGG

EADLMLQILEDPSPTPPHDIYSEEFCSFINACLQKDADARPTCDQLLSHSFIKRYEGPGV

DLSEYNKSVHDPSERLSQIAHMLAVHYYLIFDGGDDQWCHMKTFYQQDSIFSFSGETHVG

KSEIFETLSRIRKMLKGNSPCEKIAHVMEKVYCRSHGEEGMRVRVSGSFIVGNEFVVCAD

GVRAEGMLSIDELSPDILSKQAGHFQEDFFMEPGTALGCYVISKQELHIADT*

>ABR5MKK4 Brdisv1ABR51037002m.p

MRPGGPPNARPQQPGTPGRARRRPDLTLPLPQRDLTSLAVPLPLPPPPSSAPSSASSSGS

SLSSMGAPTPPNSAGSAPPPPPPLAELERVRRIGSGAGGTVWMVRHRPTGRPYALKVLYG

NHDDAVRRQITREIAILRTAEHPAIVRCHGMYEQAGELQILLEFMDGGSLEGRRIASEAF

LADVARQVLSGIAYLHRRHIVHRDIKPSNLLIDSGRRVKIADFGVGRILNQTMDPCNSSV

GTIAYMSPERINTDLNDGAYDGYAGDIWSFGLSILEFYLGRFPLGENLGKQGDWAALMCA

ICYSDSPAPPPIASPEFKSFISCCLQKNPARRPSAAQLLQHRFIAGPQPQVLAAPPS*

>ABR5MKK5 Brdisv1ABR51007117m.p

MRPAGSLPSPQPGTPGRPRRRPDLTLPMPQRPDVSSSLALERVRRVGSGAGGTVWMVRHR

PTGRCYALKQLYGNHDDAVRRQIAREIAILRTAEHPAVVRCHGMYERGGELQILLEYMDG

GSLDGRRIAAEGFLADVARQVLSGIAYLHRRHIVHRDIKPSNLLIDSARRVKIADFGVGR

ILNQTMDPCNSSVGTIAYMSPERINTDLNDGAYDGYAGDIWSFGLSILEFYLGRFPFGEN

LGKQGDWAALMVAICYNDPPEPSAAASPEFRGFISCCLQKNPAKRLSAAQLLQHPFVAGP

QPLPLAAPPS*

>ABR5MKK6 Brdisv1ABR51011697m.p

MRGKKPLKELKLSVPAQETSVDKFLTASGTFKDGELRLNQRGLRLISEEENGDEHQSTNM

KVEDVQLSMDDLEMIQVIGKGSGGVVQLVQHKWVGTFYALKGIQMNIQEAVRKQIVQELK

INQATQSPHIVSCHQSFYHNGVIYLVLEYMDRGSLADIIKQVKTILEPYLAVLCKQVLEG

LLYLHHERHVIHRDIKPSNLLVNHKGEVKITDFGVSAVLASSIGQRDTFVGTYNYMAPER

ISGSSYDYKSDVWSLGLVILECAIGRFPYTPSEGEGWLSFYELLEAIVDQPPPGAPADQF

SPEFCSFISACIQKDPAERMSASELLNHAFIKKFEGKDLDLRILVESLEPPMNVPE*

>ABR5MKK10-1 Brdisv1ABR51001752m.p

MALLREKRLQLSLHVPTRAADAQEAGLHRRPNPAAALPLAATTPAARSSQFRVADFEKLA

VLGRGNGGTVYKVRHRETCELYALKVQHCNGDATAEAEVLSRTASPFVVRCHSVLPAAAS

GDVAMLLELVDGGSLDSIVKSRSRGQAEAFSQFPEEALAEVAAQALSGLAYLHARRIVHL

DVKPGNLLVSTGGEVKIADFGIARVLPRAGGDDVRCTAYAGTAAYMSPERFDPEAHGGHY

DPYAADVWGLGVTVLELLMGRYPLLPAGQRPSWAALMCAICFGETPALSDGEASAELRGF

VAACLHKDYRRRASVAELLAHPFVAGRDVAASKCALRKLVTEASMSP*

>ABR5MKK10-2 Brdisv1ABR51010777m.p

MALVRQRRQLPHLTLPLDHFALRPPPVPAPAPTVAAAGLRLSDFERISLLGQGNGGTVYK

ARHRRAAAQPPVALKLFVAGDPSAAREAEILRLAADAPHVVRLHAVVPSSSPAAGAEQPP

PAALALELLPGGSLAGLLRRLGRSMGERPIAAVARQALLGLDALHALRVVHRDLKPSNLL

LGSHGEVKIADFGAGKVLRRRLDPCASYVGTAAYMSPERFDPEAYSGDYDPYAADVWSLG

LAILELYLGHFPLLPAGQRPDWAALMCAICFGDAPEAPAAASEEFRDFVARCLEKKAGQR

ASVAELLEHPFIAERDAEEAKRALAALVAEAELGDL*

>ABR5MKK10-3 Brdisv1ABR51001647m.p

MALLREKRLQLSLHVPTRAAEALDAVHRRPNPVAATLAASTPAAARSSQFRLADFDKLTV

LGRGNGGTVYKVRHRETCELYALKVQHCNGDPTAAAEAEVLSRTASPFIVRCHSVLPGAA

SGDVAMLLELVDGGSLDSIVKSRRAHAFPFPEEALAEVAAQALSGLAYLHARRIVHLDIK

PGNLLVSTGGEVKVADFGIAKVLPRAGADDARCKSYAGTAAYMSPERFDPEAHGGHYDAY

AADVWGLGVTVLELLMGRYPLLPAGQRPSWPALMCAICFGETPVLSDGEASAELRGFVAA

CLRKDHTKRASVAELLAHPFVAGRDVATSKCALRKLVTEASTSP*

>ABR5MKK10-5 Brdisv1ABR51001637m.p

MALTVRQRRLPQLHISLDLPSCSFRCPNPPVAATASTSGEFRASDFEPLAVLGRGNGGTV

YKVAHRRTSAQYALKVLHGGGDPGAAAAEADVLRRAADSPYVVRCHSVFPAASGSGETAL

LLELVDGGSLDSVRRGVGVSVFFPEAALAEVAAQALAGLAHLHARRVVHRDIKPANLLVS

GAGGVKVADFGIAMVLPSRAGGERCAAAYEGTVAYMSPERFDSEGRADADPRGADVWGLG

VTVLELLMGRYPLLPAGQKPTWAALMCAICFGELPALPEGAASTELRGFIAACLRKDHTK

RASVAELIKHPFVAGRNMAASRLALRRLVAGA*

>ABR6MKK1 Brdisv1ABR6_r1008357m.p

MRKPGKLALPSHESTIGKFLTQSGTFKDGDLLVNKDGLRIVHNSEEGEAPPIEPLDDHQL

SLDDLDAIKVIGKGSSGIVQLVRHKWTDQFFALKVIQLNIQESIRKQIAQELKISLSTQC

QYVVTCYQCFYVNGVISIVLEYMDGGSLADFLKTVRTIPEAYLAAICKQVLQGLMYLHHE

KRVIHRDLKPSNILINHRGEVKISDFGVSAIIASSSAQRDTFTGTFNYMAPERISGQKHG

YMSDIWSLGLVMLECATGNFPYPSPDSFYELLEAVVDQPPPSAPTDQFSPEFCSFISACI

QKEATDRSSAQVLSDHPFLSMYDDLNIDLADYFTTAGSPLATFKQIVL*

>ABR6MKK3-1 Brdisv1ABR6_r1030162m.p

MAGLEELKKKLQPLLFDDPDKDGISTRVPFPEDNCDSYVVSDGGTINLLSRSFGEYNINE

HGFHKRSTGADESDFGEKAYRCASHDMHIFGPIGNGASSVVQRAIFIPVHRILALKKINI

FEKEKRQQILNEMRTLCEASCYPGLVEFQGAFYMPDSGQISIALEYMDGGSLADVIKVKK

SIPEQVLAHMLQKVLLGLRYLHEVRHLVHRDIKPANMLVNLKGEAKITDFGVSAGLDNTM

AMCATFVGTVTYMSPERIRNENYSYAADIWSLGLTILECATGKFPYNVNEGPANLMLQIL

DDPSPTPPADAYSPEFCSFVNDCLQKDPDARPTCEQLFGHPFIKRYENAGVDLIAYVKGV

VDPTERLKEIAEMLAVHYYLLFNGSDGLWHHMKTFYMEESTFSFSGNVYVGRNDIFDTLS

SIRKKLKGDRPREKIVHVVEKLHCRANGETGIAIRVSGSLIVGNQFLVCGEGLQAEGMPS

VEELSIDIPSKRVGQFREQFMMLPGISMGSFHISRQDLYIIQA*

>ABR6MKK3-2 Brdisv1ABR6_r1006674m.p

MAGLEELKKKLQPLMFNDPDKDGFSTRVPFPEDTCDSYVVSDGGTINLLSRSFGEYNINE

HGFHKRSAGADESDFGEKAYRCASQDMHIFGPIGNGASSVVQRAIFIPVHRILALKKINI

FEKEKRQQILNEMRTLCEACCYPGLVEFQGAFYMPDSGQISIALEYMDGGSLADVIKVKK

SIPEPVLAHMLQKVLLGLRYLHEVRHLVHRDIKPANMLVNLKGEAKITDFGVSAGLDNTM

AMCATFVGTVTYMSPERIRNENYSYAADIWSLGLTILECATGKFPYNVNEGPANLMLQIL

DDPSPAPPENAFSSEFCSFVNDCLQKDADARPTCEQLLSHPFIKRYENAGVDLAAYVKGV

VNPEERLKQIAEMLAVHYYLLFNGSDGLWHHMKTFYMEDSTFSFSGNLYVGQSDIFDTLS

NIRTKLKGDRPREKIVHVVEKLHCRANEETGIAIRVSGSFIVSNQFLICGEGLQAEGMPS

LEELSIDIPSKRVGQFREQFIMHPGRSMGCYYISRQDLYIIQA*

>ABR6MKK3-3 Brdisv1ABR6_r1033144m.p

MGWDGMGQRKRRHRAGIPIPTYPGLAASLEFCFLTAGACHTAPGPGSKIAASLFAALLFQ

RPPASTSRAIFFCFGIMAAGLEDLRRRVQPIFFDADGNVMPAPDDDSEVLDGGTINLLSR

SSDEYNINERGFHKRTIRSDDEYSSEKAFRCSCHDMHIFDSVGNGASSVVHRAIYVPVHR

VLALKKINIFEKERRQQILNEIITLSEACCYPGLVEFHGVFYTPDSGEIYFALEYMDGGS

LADIIRVKKFISEPVLSHMLQKVLLALRYLHEVRHLVHRDIKPANLLLNLKGDTKITDFG

VTSGLHDSIDMCATFLGSVTYMSPERIRNESYSYSADIWSLGLTALECATGRYPYDVNGG

EADLMLQILEDPSPTPPHDIYSEEFCSFINACLQKDADARPTCDQLLSHSFIKRYEGPGV

DLSEYNKSVHDPSERLSQIAHMLAVHYYLIFDGGDDQWCHMKTFYQQDSIFSFSGETHVG

KSEIFETLSRIRKMLKGNSPCEKIAHVMEKVYCRSHGEEGMRVRVSGSFIVGNEFVVCAD

GVRAEGMLSIDELSPDILSKQAGHFQEDFFMEPGTALGCYVISKQELHIADT*

>ABR6MKK4 Brdisv1ABR6_r1039790m.p

MRPGGPPNARPQQPGTPGRARRRPDLTLPLPQRDLTSLAVPLPLPPPPSSAPSSASSSGS

SLSSMGAPTPPNSAGSAPPPPPPLAELERVRRIGSGAGGTVWMVRHRPTGRPYALKVLYG

NHDDAVRRQITREIAILRTAEHPAIVRCHGMYEQAGELQILLEFMDGGSLEGRRIASEAF

LADVARQVLSGIAYLHRRHIVHRDIKPSNLLIDSGRRVKIADFGVGRILNQTMDPCNSSV

GTIAYMSPERINTDLNDGAYDGYAGDIWSFGLSILEFYLGRFPLGENLGKQGDWAALMCA

ICYSDSPAPPPIASPEFKSFISCCLQKNPARRPSAAQLLQHRFIAGPQPQVLAAPPS*

>ABR6MKK5 Brdisv1ABR6_r1007553m.p

MRPAGSLPSPQPGTPGRPRRRPDLTLPMPQRPDVSSSLAVPLPLPPPSSLGLAQPPAAAA

AAAAPPPPPLGELERVRRVGSGAGGTVWMVRHRPTGRCYALKQLYGNHDDAVRRQIAREI

AILRTAEHPAVVRCHGMYERGGELQILLEYMDGGSLDGRRIAAEGFLADVARQVLSGIAY

LHRRHIVHRDIKPSNLLIDSARRVKIADFGVGRILNQTMDPCNSSVGTIAYMSPERINTD

LNDGAYDGYAGDIWSFGLSILEFYLGRFPFGENLGKQGDWAALMVAICYNDPPEPSAAAS

PEFRGFISCCLQKNPAKRLSAAQLLQHPFVAGPQPLPLAAPPS*

>ABR6MKK6 Brdisv1ABR6_r1012715m.p

MRGKKPLKELKLSVPAQETSVDKFLTASGTFKDGELRLNQRGLRLISEEENGDEHQSTNM

KVEDVQLSMDDLEMIQVIGKGSGGVVQLVQHKWVGTFYALKGIQMNIQEAVRKQIVQELK

INQATQSPHIVSCHQSFYHNGVIYLVLEYMDRGSLADIIKQVKTILEPYLAVLCKQVLEG

LLYLHHERHVIHRDIKPSNLLVNHKGEVKITDFGVSAVLASSIGQRDTFVGTYNYMAPER

ISGSSYDYKSDVWSLGLVILECAIGRFPYTPSEGEGWLSFYELLEAIVDQPPPGAPADQF

SPEFCSFISACIQKDPAERMSASELLNHAFIKKFEGKDLDLRILVESLEPPMNVPE*

>ABR6MKK10-1 Brdisv1ABR6_r1001837m.p

MALLREKRLQLSLHVPTRAADAQEAGLHRRPNPAAALPLAATTPAARSSQFRVADFEKLA

VLGRGNGGTVYKVRHRETCELYALKVQHCNGDATAEAEVLSRTASPFVVRCHSVLPAAAS

GDVAMLLELVDGGSLDSIVKSRSRGQAEAFSQFPEEALAEVAAQALSGLAYLHARRIVHL

DVKPGNLLVSTGGEVKIADFGIARVLPRAGGDDVRCTAYAGTAAYMSPERFDPEAHGGHY

DPYAADVWGLGVTVLELLMGRYPLLPAGQRPSWAALMCAICFGETPALSDGEASAELRGF

VAACLHKDYRRRASVAELLAHPFVAGRDVAASKCALRKLVTEASMSP*

>ABR6MKK10-2 Brdisv1ABR6_r1011767m.p

MALVRQRRQLPHLTLPLDHFALRPPPVPAPAPTVAASTSSEAAGLRLSDFERISLLGQGN

GGTVYKARHRRAAAQPPVALKLFVAGDPSAAREAEILRLAADAPHVVRLHAVVPSSSPAA

GAEQPPPAALALELLPGGSLAGLLRRLGRSMGERPIAAVARQALLGLDALHALRVVHRDL

KPSNLLLGSHGEVKIADFGAGKVLRRRLDPCASYVGTAAYMSPERFDPEAYSGDYDPYAA

DVWSLGLAILELYLGHFPLLPAGQRPDWAALMCAICFGDAPEAPAAASEEFRDFVARCLE

KKAGQRASVAELLEHPFIAERDAEEAKRALAALVAEAELGDL*

>ABR6MKK10-3 Brdisv1ABR6_r1001726m.p

MALLREKRLQLSLHVPTRAAEALDAVHRRPNPVAATLAASTPAAARSSQFRLADFDKLTV

LGRGNGGTVYKVRHRETCELYALKVQHCNGDPTAAAEAEVLSRTASPFIVRCHSVLPGAA

SGDVAMLLELVDGGSLDSIVKSRRAHAFPFPEEALAEVAAQALSGLAYLHARRIVHLDIK

PGNLLVSTGGEVKVADFGIAKVLPRAGADDARCKSYAGTAAYMSPERFDPEAHGGHYDAY

AADVWGLGVTVLELLMGRYPLLPAGQRPSWPALMCAICFGETPVLSDGEASAELRGFVAA

CLRKDHTKRASVAELLAHPFVAGRDVATSKCALRKLVTEASTSP*

>ABR6MKK10-4 Brdisv1ABR6_r1001722m.p

MASAKERRLPQLHLKLDVPTCAFRCAAPAPAPATAATPATSASRPPHGEFRLNDFDRLSV

LGRGNGGSVYKVSHRRTSALYALKIIHGAHARPGAADEEADIVRRVVDSPNVVRCHSVLP

TASGDAAALLLELVDGGSLDSLVGGGGFLPEAAVADVAAQALSGLAHLRARRVAHRDIKP

ANLLLSAAGEVKIADFGIAKVVVSGAGGRARALAYEGTVAYMSPERFDSERHADADPYAA

DVWGLGVTLLELLMGRYPLLPAGQKPTWAALMCAICFGELPALPEGAASLEFRGFVAACL

RKDHRKRASVVELLAHPFVAGRDVAASRRALREAIERRCSC*

>ABR6MKK10-5 Brdisv1ABR6_r1001717m.p

MALTVRQRRLPQLHISLDLPSCSFRCPNPPVAATASTSGEFRASDFEPLAVLGRGNGGTV

YKVAHRRTSAQYALKVLHGGGDPGAAAAEADVLRRAADSPYVVRCHSVFPAASGSGETAL

LLELVDGGSLDSVRRGVGVSVFFPEAALAEVAAQALAGLAHLHARRVVHRDIKPANLLVS

GAGGVKVADFGIAMVLPSRAGGERCAAAYEGTVAYMSPERFDSEGRADADPRGADVWGLG

VTVLELLMGRYPLLPAGQKPTWAALMCAICFGELPALPEGAASTELRGFIAACLRKDHTK

RASVAELIKHPFVAGRNMAASRLALRRLVAGA*

>ABR7MKK1 Brdisv1ABR71008279m.p

MRKPGKLALPSHESTIGKFLTQSGTFKDGDLLVNKDGLRIVHNSEEGEAPPIEPLDDHQL

SLDDLDAIKVIGKGSSGIVQLVRHKWTDQFFALKVIQLNIQESIRKQIAQELKISLSTQC

QYVVTCYQCFYVNGVISIVLEYMDGGSLADFLKTVRTIPEAYLAAICKQVLQGLMYLHHE

KRVIHRDLKPSNILINHRGEVKISDFGVSAIIASSSAQRDTFTGTFNYMAPERISGQKHG

YMSDIWSLGLVMLECATGNFPYPSPDSFYELLEAVVDQPPPSAPTDQFSPEFCSFISACI

QKEATDRSSAQVLSDHPFLSMYDDLNIDLADYFTTAGSPLATFKQIVL*

>ABR7MKK3-1 Brdisv1ABR71029810m.p

MAGLEELKKKLQPLLFDDPDKDGISTRVPFLEDNCDSYVVSDGGTINLLSRSFGEYNINE

HGFHKRSTGADESDFGEKAYRCASHDMHIFGPIGNGASSVVQRAIFIPVHRILALKKINI

FEKEKRQQILNEMRTLCEASCYPGLVEFQGAFYMPDSGQISIALEYMDGGSLADVIKVKK

SIPEQVLAHMLQKVLLGLRYLHEVRHLVHRDIKPANMLVNLKGEAKITDFGVSAGLDNTM

AMCATFVGTVTYMSPERIRNENYSYAADIWSLGLTILECATGKFPYNVNEGPANLMLQIL

DDPSPTPPADAYSPEFCSFVNDCLQKDPDARPTCEQLFGHPFIKRYENAGVDLIAYVKGV

VDPTERLKEIAEMLAVHYYLLFNGSDGLWHHMKTFYMEESTFSFSGNVYVGRNDIFDTLS

SIRKKLKGDRPREKIVHVVEKLHCRANGETGIAIRVSGSLIVGNQFLVCGEGLQAEGMPS

VEELSIDIPSKRVGQFREQFMMLPGISMGSFHISRQDLYIIQA*

>ABR7MKK3-2 Brdisv1ABR71020756m.p

MAGLEELKKKLQPLMFNDPDKDGFSTRVPFPEDTCDSYVVSDGGTINLLSRSFGEYNINE

HGFHKRSAGADESDFGEKAYRCASQDMHIFGPIGNGASSVVQRAIFIPVHRILALKKINI

FEKEKRQQILNEMRTLCEACCYPGLVEFQGAFYMPDSGQISIALEYMDGGSLADVIKVKK

SIPEPVLAHMLQKVLLGLRYLHEVRHLVHRDIKPANMLVNLKGEAKITDFGVSAGLDNTM

AMCATFVGTVTYMSPERIRNENYSYAADIWSLGLTILECATGKFPYNVNEGPANLMLQIL

DDPSPAPPENAFSSEFCSFVNDCLQKDADARPTCEQLLSHPFIKRYENAGVDLAAYVKGV

VNPEERLKQIAEMLAVHYYLLFNGSDGLWHHMKTFYMEDSTFSFSGNLYVGQSDIFDTLS

NIRTKLKGDRPREKIVHVVEKLHCRANEETGIAIRVSGSFIVSNQFLICGEGLQAEGMPS

LEELSIDIPSKRVGQFREQFIMHPGRSMGCYYISRQDLYIIQA*

>ABR7MKK3-3 Brdisv1ABR71032780m.p

MGWDGMGQRKRRHRAGIPIPTYPGLAASLEFCFVTAGACHTAPGPGSKIAASLFAALLFQ

RPPASTSRAIFFCFVIMAAGLEDLRRRVQPIFFDADGNVMPAPDDDSEVLDGGTINLLSR

SSDEYNINERGFHKRTIRSDDEYSSEKAFRCSCHDMHIFDSVGNGASSVVHRAIYVPVHR

VLALKKINIFEKERRQQILNEIITLSEACCYPGLVEFHGVFYTPDSGEIYFALEYMDGGS

LADIIRVKKFISEPVLSHMLQKVLLALRYLHEVRHLVHRDIKPANLLLNLKGDTKITDFG

VTSGLHDSIDMCATFLGSVTYMSPERIRNESYSYSADIWSLGLTALECATGRYPYDVNGG

EADLMLQILEDPSPTPPHDIYSEEFCSFINACLQKDADARPTCDQLLSHSFIKRYEGPGV

DLSEYNKSVHDPSERLSQIAHMLAVHYYLIFDGGDDQWCHMKTFYQQDSIFSFSGETHVG

KSEIFETLSRIRKMLKGNSPCEKIAHVMEKVYCRSHGEEGMRVRVSGSFIVGNEFVVCAD

GVRAEGMLSIDELSPDILSKQAGHFQEDFFMEPGTALGCYVISKQELHIADT*

>ABR7MKK4 Brdisv1ABR71039480m.p

MRPGGPPNARPQQPGTPGRARRRPDLTLPLPQRDLTSLAVPLPLPPPPSSAPSSASSSGS

SLSSMGAPTPPNSAGSAPPPPPPLAELERVRRIGSGAGGTVWMVRHRPTGRPYALKVLYG

NHDDAVRRQITREIAILRTAEHPAIVRCHGMYEQAGELQILLEFMDGGSLEGRRIASEAF

LADVARQVLSGIAYLHRRHIVHRDIKPSNLLIDSGRRVKIADFGVGRILNQTMDPCNSSV

GTIAYMSPERINTDLNDGAYDGYAGDIWSFGLSILEFYLGRFPLGENLGKQGDWAALMCA

ICYSDSPAPPPIASPEFKSFISCCLQKNPARRPSAAQLLQHRFIAGPQPQVLAAPPS*

>ABR7MKK5 Brdisv1ABR71007538m.p

MRPAGSLPSPQPGTPGRPRRRPDLTLPMPQRPDVSSSLAVPLPLPPPSSLGLAQPPAAAA

AAAAPPPPPLGELERVRRVGSGAGGTVWMVRHRPTGRCYALKQLYGNHDDAVRRQIAREI

AILRTAEHPAVVRCHGMYERGGELQILLEYMDGGSLDGRRIAAEGFLADVARQVLSGIAY

LHRRHIVHRDIKPSNLLIDSARRVKIADFGVGRILNQTMDPCNSSVGTIAYMSPERINTD

LNDGAYDGYAGDIWSFGLSILEFYLGRFPFGENLGKQGDWAALMVAICYNDPPEPSAAAS

PEFRGFISCCLQKNPAKRLSAAQLLQHPFVAGPQPLPLAAPPS*

>ABR7MKK6 Brdisv1ABR71012442m.p

MRGKKPLKELKLSVPAQETSVDKFLTASGTFKDGELRLNQRGLRLISEEENGDEHQSTNM

KVEDVQLSMDDLEMIQVIGKGSGGVVQLVQHKWVGTFYALKGIQMNIQEAVRKQIVQELK

INQATQSPHIVSCHQSFYHNGVIYLVLEYMDRGSLADIIKQVKTILEPYLAVLCKQVLEG

LLYLHHERHVIHRDIKPSNLLVNHKGEVKITDFGVSAVLASSIGQRDTFVGTYNYMAPER

ISGSSYDYKSDVWSLGLVILECAIGRFPYTPSEGEGWLSFYELLEAIVDQPPPGAPADQF

SPEFCSFISACIQKDPAERMSASELLNHAFIKKFEGKDLDLRILVESLEPPMNVPE*

>ABR7MKK10-1 Brdisv1ABR71001829m.p

MALLREKRLQLSLHVPTRAADAQEAGLHRRPNPAAALPLAATTPAARSSQFRVADFEKLA

VLGRGNGGTVYKVRHRETCELYALKVQHCNGDATAEAEVLSRTASPFVVRCHSVLPAAAS

GDVAMLLELVDGGSLDSIVKSRSRGQAEAFSQFPEEALAEVAAQALSGLAYLHARRIVHL

DVKPGNLLVSTGGEVKIADFGIARVLPRAGGDDVRCTAYAGTAAYMSPERFDPEAHGGHY

DPYAADVWGLGVTVLELLMGRYPLLPAGQRPSWAALMCAICFGETPALSDGEASAELRGF

VAACLHKDYRRRASVAELLAHPFVAGRDVAASKCALRKLVTEASMSP*

>ABR7MKK10-2 Brdisv1ABR71011533m.p

MALVRQRRQLPHLTLPLDHFALRPPPVPASAPTVAASTSSEAAGLRLSDFERISLLGQGN

GGTVYKARHRRAAAQPPVALKLFVAGDPSAAREAEILRLAADAPHVVRLHAVVPSSSPAA

GAEQPPPAALALELLPGGSLAGLLRRLGRSMGERPIAAVARQALLGLDALHALRVVHRDL

KPSNLLLGSHGEVKIADFGAGKVLRRRLDPCASYVGTAAYMSPERFDPEAYSGDYDPYAA

DVWSLGLAILELYLGHFPLLPAGQRPDWAALMCAICFGDAPEAPAAASEEFRDFVARCLE

KKAGQRASVAELLEHPFIAERDAEEAKRALAALVAEAELGDL*

>ABR7MKK10-3 Brdisv1ABR71001729m.p

MALLREKRLQLSLHVPTRAAEALDAVHRRPNPVAATLAASTPAAARSSQFRLADFDKLTV

LGRGNGGTVYKVRHRETCELYALKVQHCNGDPTAAAEAEVLSRTASPFIVRCHSVLPGAA

SGDVAMLLELVDGGSLDSIVKSRRAHAFPFPEEALAEVAAQALSGLAYLHARRIVHLDIK

PGNLLVSTGGEVKVADFGIAKVLPRAGADDARCKSYAGTAAYMSPERFDPEAHGGHYDAY

AADVWGLGVTVLELLMGRYPLLPAGQRPSWPALMCAICFGETPVLSDGEASAELRGFVAA

CLRKDHTKRASVAELLAHPFVAGRDVATSKCALRKLVTEASTSP*

>ABR7MKK10-4 Brdisv1ABR71001726m.p

MASAKERRLPQLHLKLDVPTCAFRCAAPAPAPATAATPATSASRPPHGEFRLNDFDRLSV

LGRGNGGSVYKVSHRRTSALYALKIIHGAHARPGAADEEADIVRRVVDSPNVVRCHSVLP

TASGDAAALLLELVDGGSLDSLVGGGGFLPEAAVADVAAQALSGLAHLRARRVAHRDIKP

ANLLLSAAGEVKIADFGIAKVVVSGAGGRARALAYEGTVAYMSPERFDSERHADADPYAA

DVWGLGVTLLELLMGRYPLLPAGQKPTWAALMCAICFGELPALPEGAASLEFRGFVAACL

RKDHRKRASVVELLAHPFVAGRDVAASRRALREAIERRCSC*

>ABR7MKK10-5 Brdisv1ABR71001728m.p

MALTVRQRRLPQLHISLDLPSCSFRCPNPPVAATASTSGEFRASDFEPLAVLGRGNGGTV

YKVAHRRTSAQYALKVLHGGGDPGAAAAEADVLRRAADSPYVVRCHSVFPAASGSGETAL

LLELVDGGSLDSVRRGVGVSVFFPEAALAEVAAQALAGLAHLHARRVVHRDIKPANLLVS

GAGGVKVADFGIAMVLPSRAGGERCAAAYEGTVAYMSPERFDSEGRADADPRGADVWGLG

VTVLELLMGRYPLLPAGQKPTWAALMCAICFGELPALPEGAASTELRGFIAACLRKDHTK

RASVAELIKHPFVAGRNMAASRLALRRLVAGA*

>Bd30-1MKK1 Brdisv1Bd30-11007680m.p

MRKPGKLALPSHESTIGKFLTQSGTFKDGDLLVNKDGLRIVHNSEEGEAPPIEPLDDHQL

SLDDLDAIKVIGKGSSGIVQLVRHKWTDQFFALKVIQLNIQESIRKQIAQELKISLSTQC

QYVVTCYQCFYVNGVISIVLEYMDGGSLADFLKTVRTIPEAYLAAICKQASETCHTRNQF

CYWNVLQGLMYLHHEKRVIHRDLKPSNILINHRGEVKISDFGVSAIIASSSAQRDTFTGT

FNYMAPERISGQKHGYMSDIWSLGLVMLECATGNFPYPSPDSFYELLEAVVDQPPPSAPT

DQFSPEFCSFISACIQKEATDRSSAQALSDHPFLSMYDDLNIDLADYFTTAGSPLATFKQ

IVL*

>Bd30-1MKK3-1 Brdisv1Bd30-11035477m.p

MAGLEELKKKLQPLLFDDPDKDGISTRVPFPEDNCDSYVVSDGGTINLLSRSFGEYNINE

HGFHKRSTGADESDFGEKAYRCASHDMHIFGPIGNGASSVVQRAIFIPVHRILALKKINI

FEKEKRQQILNEMRTLCEASCYPGLVEFQGAFYMPDSGQISIALEYMDGGSLADVIKVKK

SIPEQVLAHMLQKVLLGLRYLHEVRHLVHRDIKPANMLVNLKGEAKITDFGVSAGLDNTM

AMCATFVGTVTYMSPERIRNENYSYAADIWSLGLTILECATGKFPYNVNEGPANLMLQIL

DDPSPTPPADAYSPEFCSFVNDCLQKDPDARPTCEQLFGHPFIKRYENAGVDLIAYVKGV

VDPTERLKEIAEMLAVHYYLLFNGSDGLWHHMKTFYMEESTFSFSGNVYVGRNDIFDTLS

SIRKKLKGDRPREKIVHVVEKLHCRANGETGIAIRVSGSLIVGNQFLVCGEGLQAEGMPS

VEELSIDIPSKRVGQFREQFMMLPGISMGSFHISRQDLYIIQA*

>Bd30-1MKK3-2 Brdisv1Bd30-11040333m.p

MAGLGEVKKKLQPLMFNDPDKDGVSTRVPFPEDTCDSYVVSDGGTINLLSRSFGEYNINE

HGFHKRSAGADESDFGEKAYRCASQDMHIFGPIGNGASSVVQRAIFIPVHRILALKKINI

FEKEKRQQILNEMRTLCEACCYPGLVEFQGAFYMPDSGQISIALEYMDGGSLADVIKVKK

SIPEPVLAHMLQKVLLGLRYLHEVRHLVHRDIKPANMLVNLKGEAKITDFGVSAGLDNTM

AMCATFVGTVTYMSPERIRNENYSYAADIWSLGLTILECATGKFPYNVNEGPANLMLQIL

DDPSPAPPENAFSSEFCSFVNDCLQKDADARPTCEQLLSHPFIKRYENAGVDLAAYVKGV

VNPEERLKQIAEMLAVHYYLLFNGSDGLWHHMKTFYMEDSTFSFSGNLYVGQSDIFDTLS

NIRTKLKGDRPREKIVHVVEKLHCRANEETGIAIRVSGSFIVSNQFLICGEGLQAEGMPS

LEELSIDIPSKRVGQFREQFIMHPGRSMGCYYISRQDLYIIQA*

>Bd30-1MKK3-3 Brdisv1Bd30-11023309m.p

MDGGWDGMGQRKRRHRAGIPIPTYPGLAASLEFCFVTAGACHTAPGPGSKIAASLFAALL

FQRPPASTSRAIFFCFVIMAGGLGGLRRGGQPIFFDADGNVMPAPDDDSEVLDGGTINLL

SRSSDEYNINERGFHKRTIRSDDEYSSEKAFRCSCHDMHIFDSVGNGASSVVHRAIYVPV

HRVLALKKINIFEKERRQQILNEIITLSEACCYPGLVEFHGVFYTPDSGEIYFALEYMDG

GSLADIIRVKKFISEPVLSHMLQKVLLALRYLHEVRHLVHRDIKPANLLLNLKGDTKITD

FGVTSGLHDSIDMCATFLGSVTYMSPERIRNESYSYSADIWSLGLTALECATGRYPYDVN

GGEADLMLQILEDPSPTPPHDIYSEEFCSFINACLQKDADARPTCDQLLSHSFIKRYEGP

GVDLSEYNKSVHDPSERLSQIAHMLAVHYYLIFDGGDDQWCHMKTFYQQDSIFSFSGETH

VGKSEIFETLSRIRKMLKGNSPCEKIAHVMEKVYCRSHGEEGMRVRVSGSFIVGNEFVVC

ADGVRAEGMLSIDELSPDILSKQAGHFQEDFFMEPGTALGCYVISKQELHIADT*

>Bd30-1MKK4 Brdisv1Bd30-11029066m.p

MGRHRPTGRPYALKVLYGNHDDAVRRQITREIAILRPAEHPAIVRCHGMYEQAGELQILL

EFMDGGSLEGRRIASEAFLADVARQVLSGIAYLHRRHIVHRDIKPSNLLIDSGRRVKIAD

FGVGRILNQTMDPCNSSVGTIAYMSPERINTDLNDGAYDGYAGDIWSFGLSILEFYLGRF

PLGENLGKQGDWAALMCAICYSDSPAPPPIAPPEFKSFISCCLQKNPARRPPAAQLLQPR

FIAGPQPQVLAAPPS*

>Bd30-1MKK5 Brdisv1Bd30-11006970m.p

MGMRPDVSSSLAVPLPLPPPSSLGLAQPPAAAPAAAAPPPPPLGELERVRRVGSGAGGTV

WMGGHRPTGRCYALKQLYGNHDDAVRRQIAREIAILRTAAPPAVVRCHGMYERGGGLQIL

LEYMGGGPPAGRRIAAEGFLADVARQVLSGIAYLHRRHIVHRDIKPSNLLIDSARRVKIA

DFGVGRILNQTMDPCNSSVGTIAYMSPERINTDLNDGAYDGYAGDIWSFGLSILEFYLGR

FPFGENLGKQGDWAALMVAICYNDPPEPSAAASPEFRGFISCCLQKNPAKRLSAAQLLQH

PFVAGPQPLPLAAPPS*

>Bd30-1MKK6 Brdisv1Bd30-11011645m.p

MRGKKPLKELKLSVPAQETSVDKFLTASGTFKDGELRLNQRGLRLISEEENGDEHQSTNM

KVEDVQLSMDDLEMIQVIGKGSGGVVQLVQHKWVGTFYALKGIQMNIQEAVRKQIVQELK

INQATQSPHIVSCHQSFYHNGVIYLVLEYMDRGSLADIIKQVKTILEPYLAVLCKQVLEG

LLYLHHERHVIHRDIKPSNLLVNHKGEVKITDFGVSAVLASSIGQRDTFVGTYNYMAPER

ISGSSYDYKSDVWSLGLVILECAIGRFPYTPSEGEGWLSFYELLEAIVDQPPPGAPADQF

SPEFCSFISAWYAKHYSPKLHTKLAHLLHSCLFSCLVNLNEPLALFACPCW*

>Bd30-1MKK10-1 Brdisv1Bd30-11001809m.p

MALLREKRLQLSLHVPPRAADAQEAGLHRRPNPAAALPRAAPPPAARSSQFRVADFEKLA

VLGRGNGGTVYKVRHRETCELYALKVQHCNGDATAEAEVLSRTASPFVVRCPSVPPAAAS

GDVAMLLELVDGGALDSIVKSRSRGQAEAFSQFPEEALAEVAAQALSGLAYLHARRIVHL

DVKPGNLLVSTGGEVKIADFGIARVLPRAGGDDVRCTAYAGTAAYMSPERFDPEAHGGHY

DPYAADVWGLGVTVLELLMGRYPLPPAGQRPSWAALMCAICFGETPALSDGEASAELRGF

VAACLHKDYRRRASVAELLAHPFVAGRDVAASKCALRKLVTEASMSP*

>Bd30-1MKK10-2 Brdisv1Bd30-11010689m.p

MALVRQRRQLPHLTLPLDPSALRPPPVPAPAPPVAASPSSEAAGPRLSDFERISLLGQGN

GGTVYKARHRRAAAQPPVALKLFVAGAPSAAREAEILRLAADAPHVVRLHAVVPSSSPAA

GAEQPPPAALALELLPGGSLAGLLRRLGRAMGGRPIAAVARQALPGLDALHALRVVHRDL

KPSNLLLGSHGEVKIADFGAGKVLRRRLDPCASYVGTAAYMSPERFDPEAYSGDYDPYAA

DVWSLGLAILELYLGHFPLLPAGQRPDWAALMCAICFGDAPEAPAAASEEFRDFVARCLE

KKAGQRASVAELLEHPFIAERDAEEAKRALAALVAEAELGDL*

>Bd30-1MKK10-3 Brdisv1Bd30-11001697m.p

MALLREKRLQLSLHVPTRAAEALDAVHRRPNPVAATLAAPTPAGARSSQFRLADFDKLTV

LGRGNGGTVYKVRHRETCELYALKVQHCNGDPTAAAEAEGLSRPASPFIAPCHSVLPGAA

SGDVAMLLELVDGGSLDSIVKSRRAHAFPFPEEALAEVAAQALSGLAYLHARRIVHLDIK

PGNLLVSTGGEVKVADFGIAKVLPRAGADDARCKSYAGTAAYMSPERFDPEAHGGHYDAY

AADVWGLGVTVLELLMGRYPLPPAGQRPSWPALMCAICFGETPVLSDGEASAELRGFVAA

CLRKDHTKRASVAELLAHPFVAGRDVATSKCALRKLVTEASTSP*

>Bd30-1MKK10-4 Brdisv1Bd30-11001695m.p

MASAKERRLPQLHLKLDVPPCAFRCAAPAPAPATAATPATSASRPPHGEFRLNDFDRLSV

LGRGNGGSVYKVSHRRTSALYALKIIHGAHARPGAADEEADIVRRVVDSPNVVRCHSVLP

TASGDAAALPPELVAGGSLDSLVGGGGFLPEAAVADVAAQALSGLAPLRARRVAHRDIKP

ANLLLSAAGEVKIADFGIAKVVVSGAGGRARALAYEGTVAYMSPERFDSGRHAAAAPYAA

DVWGLGVTLLELLMGRYPLLPAGQKPTWAALMCAICFGELPALPEGAASLEFRGFVAACL

RKDHRKRASVVELLAHPFVAGRDVAASRRALREAIERRCSC*

>Bd30-1MKK10-5 Brdisv1Bd30-11041837m.p

MALTVRQRRLPQLHISLDPPSCSFGCPNPPGAATASTSGEFRASAFEPLAVLGRGNGGTV

YKVAHRRTSAQYALKVLHGGGDPGAAAAEADVLRRAADSPYVVRCHSVFPAASGSGETAL

LLELVDGGSLPPVRRGGGVSVFFPEAALAEVAAQALAGLAHLHARRVVHRDIKPANLLVN

GAGGVKVADFGIAMVLPSRAGGERCAAAYEGTVAYMSPERFDSEGRAGAHPRGADVWGLG

VTVLELLMGRYPLLPAGQKPTWAALMCAICFGELPALPEGAASTELRGFIAACLRKDHTK

RASVAELIKHPFVAGRNMAASRLALRRLVAGA*

>Foz1MKK1 Brdisv1Foz11007784m.p

MRKPGKLALPSHESTIGKFLTQSGTFKDGDLLVNKDGLRIVHNSEEGEAPPIEPLDDHQL

SLDDLDAIKVIGKGSSGIVQLVRHKWTDQFFALKVIQLNIQESIRKQIAQELKISLSTQC

QYVVTCYQCFYVNGVISIVLEYMDGGSLADFLKTVRTIPEAYLAAICKQVLQGLMYLHHE

KRVIHRDLKPSNILINHRGEVKISDFGVSAIIASSSAQRDTFTGTFNYMAPERISGQKHG

YMSDIWSLGLVMLECATGNFPYPSPDSFYELLEAVVDQPPPSAPTDQFSPEFCSFISACI

QKEATDRSSAQVLSDHPFLSMYDDLNIDLADYFTTAGSPLATFKQIVL*

>Foz1MKK3-1 Brdisv1Foz11037008m.p

MAGLEELKKKLQPLLFDDPDKDGISTRVPFLEDNCDSYVVSDGGTINLLSRSFGEYNINE

HGFHKRSTGADESDFGEKAYRCASHDMHIFGPIGNGASSVVQRAIFIPVHRILALKKINI

FEKEKRQQILNEMRTLCEASCYPGLVEFQGAFYMPDSGQISIALEYMDGGSLADVIKVKK

SIPEQVLAHMLQKVLLGLRYLHEVRHLVHRDIKPANMLVNLKGEAKITDFGVSAGLDNTM

AMCATFVGTVTYMSPERIRNENYSYAADIWSLGLTILECATGKFPYNVNEGPANLMLQIL

DDPSPTPPADAYSPEFCSFVNDCLQKDPDARPTCEQLFGHPFIKRYENAGVDLIAYVKGV

VDPTERLKEIAEMLAVHYYLLFNGSDGLWHHMKTFYMEESTFSFSGNVYVGRNDIFDTLS

SIRKKLKGDRPREKIVHVVEKLHCRANGETGIAIRVSGSLIVGNQFLVCGEGLQAEGMPS

VEELSIDIPSKRVGQFREQFMMLPGISMGSFHISRQDLYIIQA*

>Foz1MKK3-2 Brdisv1Foz11006306m.p

MAGLEELKKKLQPLMFNDPDKDGFSTRVPFPEDTCDSYVVSDGGTINLLSRSFGEYNINE

HGFHKRSAGADESDFGEKAYRCASQDMHIFGPIGNGASSVVQRAIFIPVHRILALKKINI

FEKEKRQQILNEMRTLCEACCYPGLVEFQGAFYMPDSGQISIALEYMDGGSLADVIKVKK

SIPEPVLAHMLQKVLLGLRYLHEVRHLVHRDIKPANMLVNLKGEAKITDFGVSAGLDNTM

AMCATFVGTVTYMSPERIRNENYSYAADIWSLGLTILECATGKFPYNVNEGPANLMLQIL

DDPSPAPPENAFSSEFCSFVNDCLQKDADARPTCEQLLSHPFIKRYENAGVDLAAYVKGV

VNPEERLKQIAEMLAVHYYLLFNGSDGLWHHMKTFYMEDSTFSFSGNLYVGQSDIFDTLS

NIRTKLKGDRPREKIVHVVEKLHCRANEETGIAIRVSGSFIVSNQFLICGEGLQAEGMPS

LEELSIDIPSKRVGQFREQFIMHPGRSMGCYYISRQDLYIIQA*

>Foz1MKK3-3 Brdisv1Foz11023762m.p

MGWDGMGQRKRRHRAGIPIPTYPGLAASLEFCFVTAGACHTAPGPGSKIAASLFAALLFQ

RPPASTSRAIFFCFVIMAAGLEDLRRRGQPIFFDADGNVMPAPDDDSEVLDGGTINLLSR

SSDEYNINERGFHKRTIRSDDEYSSEKAFRCSCHDMHIFDSVGNGASSVVHRAIYVPVHR

VLALKKINIFEKERRQQILNEIITLSEACCYPGLVEFHGVFYTPDSGEIYFALEYMDGGS

LADIIRVKKFISEPVLSHMLQKVLLALRYLHEVRHLVHRDIKPANLLLNLKGDTKITDFG

VTSGLHDSIDMCATFLGSVTYMSPERIRNESYSYSADIWSLGLTALECATGRYPYDVNGG

EADLMLQILEDPSPTPPHDIYSEEFCSFINACLQKDADARPTCDQLLSHSFIKRYEGPGV

DLSEYNKSVHDPSERLSQIAHMLAVHYYLIFDGGDDQWCHMKTFYQQDSIFSFSGETHVG

KSEIFETLSRIRKMLKGNSPCEKIAHVMEKVYCRSHGEEGMRVRVSGSFIVGNEFVVCAD

GVRAEGMLSIDELSPDILSKQAGHFQEDFFMEPGTALGCYVISKQELHIADT*

>Foz1MKK5 Brdisv1Foz11007091m.p

MRPAGSLPSPQPGTPGRPRRRPDLTLPMPQRPDVSSSLAPPAAAAAAAAPPPPPLGELER

VRRVGSGAGGTVWMVRHRPTGRCYALKQLYGNHDDAVRRQIAREIAILRTAEHPAVVRCH

GMYERGGELQILLEYMDGGSLDGRRIAAEGFLADVARQVLSGIAYLHRRHIVHRDIKPSN

LLIDSARRVKIADFGVGRILNQTMDPCNSSVGTIAYMSPERINTDLNDGAYDGYAGDIWS

FGLSILEFYLGRFPFGENLGKQGDWAALMVAICYNDPPEPSAAASPEFRGFISCCLQKNP

AKRLSAAQLLQHPFVAGPQPLPLAAPPS*

>Foz1MKK6 Brdisv1Foz11011624m.p

MRGKKPLKELKLSVPAQETSVDKFLTASGTFKDGELRLNQRGLRLISEEENGDEHQSTNM

KVEDVQLSMDDLEMIQVIGKGSGGVVQLVQHKWVGTFYALKGIQMNIQEAVRKQIVQELK

INQATQSPHIVSCHQSFYHNGVIYLVLEYMDRGSLADIIKQVKTILEPYLAVLCKQVLEG

LLYLHHERHVIHRDIKPSNLLVNHKGEVKITDFGVSAVLASSIGQRDTFVGTYNYMAPER

ISGSSYDYKSDVWSLGLVILECAIGRFPYTPSEGEGWLSFYELLEAIVDQPPPGAPADQF

SPEFCSFISACIQKDPAERMSASELLNHAFIKKFEGKDLDLRILVESLEPPMNVPE*

>Foz1MKK10-1 Brdisv1Foz11001741m.p

MALLREKRLQLSLHVPTRAADAQEAGLHRRPNPAAALPLAATTPAARSSQFRVADFEKLA

VLGRGNGGTVYKVRHRETCELYALKVQHCNGDATAEAEVLSRTASPFVVRCHSVLPAAAS

GDVAMLLELVDGGSLDSIVKSRSRGQAEAFSQFPEEALAEVAAQALSGLAYLHARRIVHL

DVKPGNLLVSTGGEVKIADFGIARVLPRAGGDDVRCTAYAGTAAYMSPERFDPEAHGGHY

DPYAADVWGLGVTVLELLMGRYPLLPAGQRPSWAALMCAICFGETPALSDGEASAELRGF

VAACLHKDYRRRASVAELLAHPFVAGRDVAASKCALRKLVTEASMSP*

>Foz1MKK10-2 Brdisv1Foz11010724m.p

MALVRQRRQLPHLPLPLDHFALRPPPVPAPAPTVAASTSSEAAGLRLSDFERISLLGQGN

GGTVYKARHRRAAAQPPVALKLFVAGDPSAAREAEILRLAADAPHVVRLHAVVPSSSPAA

GAEQPPPAALALELLPGGSLAGLLRRLGRSMGERPIAAVARQALLGLDALHALRVVHRDL

KPSNLLLGSHGEVKIADFGAGKVLRRRLDPCASYVGTAAYMSPERFDPEAYSGDYDPYAA

DVWSLGLAILELYLGHFPLLPAGQRPDWAALMCAICFGDAPEAPAAASEEFRDFVARCLE

KKAGQRASVAELLEHPFIAERDAEEAKRALAALVAEAELGDL*

>Foz1MKK10-3 Brdisv1Foz11001631m.p

MALLREKRLQLSLHVPTRAAEALDAVHRRPNPVAATLAASTPAAARSSQFRLADFDKLTV

LGRGNGGTVYKVRHRETCELYALKVQHCNGDPTAAAEAEVLSRTASPFIVRCHSVLPGAA

SGDVAMLLELVDGGSLDSIVKSRRAHAFPFPEEALAEVAAQALSGLAYLHARRIVHLDIK

PGNLLVSTGGEVKVADFGIAKVLPRAGADDARCKSYAGTAAYMSPERFDPEAHGGHYDAY

AADVWGLGVTVLELLMGRYPLLPAGQRPSWPALMCAICFGETPVLSDGEASAELRGFVAA

CLRKDHTKRASVAELLAHPFVAGRDVATSKCALRKLVTEASTSP*

>Foz1MKK10-4 Brdisv1Foz11001629m.p

MASAKERRLPQLHLKLDVPTCAFRCAAPAPAPATAATPATSASRPPHGEFRLNDFDRLSV

LGRGNGGSVYKVSHRRTSALYALKIIHGAHARPGAADEEADIVRRVVDSPNVVRCHSVLP

TASGDAAALLLELVDGGSLDSLVGGGGFLPEAAVADVAAQALSGLAHLRARRVAHRDIKP

ANLLLSAAGEVKIADFGIAKVVVSGAGGRARALAYEGTVAYMSPERFDSERHADADPYAA

DVWGLGVTLLELLMGRYPLLPAGQKPTWAALMCAICFGELPALPEGAASLEFRGFVAACL

RKDHRKRASVVELLAHPFVAGRDVAASRRALREAIERRCSC*

>Foz1MKK10-5 Brdisv1Foz11042773m.p

MALTVRQRRLPQLHISLDLPSCSFRCPNPPVAATASTSGEFRASDFEPLAVLGRGNGGTV

YKVAHRRTSAQYALKVLHGGGDPGAAAAEADVLRRAADSPYVVRCHSVFPAASGSGETAL

LLELVDGGSLDSVRRGVGVSVFFPEAALAEVAAQALAGLAHLHARRVVHRDIKPANLLVS

GAGGVKVADFGIAMVLPSRAGGERCAAAYEGTVAYMSPERFDSEGRADADPRGADVWGLG

VTVLELLMGRYPLLPAGQKPTWAALMCAICFGELPALPEGAASTELRGFIAACLRKDHTK

RASVAELIKHPFVAGRNMAASRLALRRLVAGA*

>Jer1MKK1 Brdisv1Jer11007559m.p

MRKPGKLALPSHESTIGKFLTQSGTFKDGDLLVNKDGLRIVHNSEEGEAPPIEPLDDHQL

SLDDLDAIKVIGKGSSGIVQLVRHKWTDQFFALKVIQLNIQESIRKQIAQELKISLSTQC

QYVVTCYQCFYVNGVISIVLEYMDGGSLADFLKTVRTIPEAYLAAICKQVLQGLMYLHHE

KRVIHRDLKPSNILINHRGEVKISDFGVSAIIASSSAQRDTFTGTFNYMAPERISGQKHG

YMSDIWSLGLVMLECATGNFPYPSPDSFYELLEAVVDQPPPSAPTDQFSPEFCSFISACI

QKEATDRSSAQVLSDHPFLSMYDDLNIDLADYFTTAGSPLATFKQIVL*

>Jer1MKK3-1 Brdisv1Jer11027394m.p

MAGLEELKKKLQPLLFDDPDKDGISTRVPFPEDNCDSYVVSDGGTINLLSRSFGEYNINE

HGFHKRSTGADESDFGEKAYRCASHDMHIFGPIGNGASSVVQRAIFIPVHRILALKKINI

FEKEKRQQILNEMRTLCEASCYPGLVEFQGAFYMPDSGQISIALEYMDGGSLADVIKVKK

SIPEQVLAHMLQKVLLGLRYLHEVRHLVHRDIKPANMLVNLKGEAKITDFGVSAGLDNTM

AMCATFVGTVTYMSPERIRNENYSYAADIWSLGLTILECATGKFPYNVNEGPANLMLQIL

DDPSPTPPADAYSPEFCSFVNDCLQKDPDARPTCEQLFGHPFIKRYENAGVDLIAYVKGV

VDPTERLKEIAEMLAVHYYLLFNGSDGLWHHMKTFYMEESTFSFSGNVYVGRNDIFDTLS

SIRKKLKGDRPREKIVHVVEKLHCRANGETGIAIRVSGSLIVGNQFLVCGEGLQAEGMPS

VEELSIDIPSKRVGQFREQFMMLPGISMGSFHISRQDLYIIQA*

>Jer1MKK3-2 Brdisv1Jer11041340m.p

MAGLEELKKKLQPLMFNDPDKDGFSTRVPFPEDTCDSYVVSDGGTINLLSRSFGEYNINE

HGFHKRSAGADESDFGEKAYRCASQDMHIFGPIGNGASSVVQRAIFIPVHRILALKKINI

FEKEKRQQILNEMRTLCEACCYPGLVEFQGAFYMPDSGQISIALEYMDGGSLADVIKVKK

SIPEPVLAHMLQKVLLGLRYLHEVRHLVHRDIKPANMLVNLKGEAKITDFGVSAGLDNTM

GMCATFVGTVTYMSPERIRNENYSYAADIWSLGLTILECATGKFPYNVNEGPANLMLQIL

DDPSPAPPENAFSSEFCSFVNDCLQKDADARPTCEQLLSHPFIKRYENAGVDLAAYVKGV

VNPEERLKQIAEMLAVHYYLLFNGSDGLWHHMKTFYMEDSTFSFSGNLYVGQSDIFDTLS

NIRTKLKGDRPREKIVHVVEKLHCRANEETGIAIRVSGSFIVSNQFLICGEGLQAEGMPS

LEELSIDIPSKRVGQFREQFIMHPGRSMGCYYISRQDLYIIQA*

>Jer1MKK3-3 Brdisv1Jer11030183m.p

MGWDGMGQRKRRHRAGIPIPTYPGLAASLEFCFLTAGACHTAPGPGSKIAASLFAALLFQ

RPPASTSRAIFFCFGIMAAGLEDLRRRVQPIFFDADGNVMPAPDDDSEVLDGGTINLLSR

SSDEYNINERGFHKRTIRSDDEYSSEKAFRCSCHDMHIFDSVGNGASSVVHRAIYVPVHR

VLALKKINIFEKERRQQILNEIITLSEACCYPGLVEFHGVFYTPDSGEIYFALEYMDGGS

LADIIRVKKFISEPVLSHMLQKVLLALRYLHEVRHLVHRDIKPANLLLNLKGDTKITDFG

VTSGLHDSIDMCATFLGSVTYMSPERIRNESYSYSADIWSLGLTALECATGRYPYDVNGG

EADLMLQILEDPSPTPPHDIYSEEFCSFINACLQKDADARPTCDQLLSHSFIKRYEGPGV

DLSEYNKSVHDPSERLSQIAHMLAVHYYLIFDGGDDQWCHMKTFYQQDSIFSFSGETHVG

KSEIFETLSRIRKMLKGNSPCEKIAHVMEKVYCRSHGEEGMRVRVSGSFIVGNEFVVCAD

GVRAEGMLSIDELSPDILSKQAGHFQEDFFMEPGTALGCYVISKQELHIADT*

>Jer1MKK4 Brdisv1Jer11036023m.p

MRPGGPPNARPQQPGTPGRARRRPDLTLPLPQRDLTSLAVPLPLPPPPSSAPSSASSSGS

SLSSMGAPTPPNSAGSAPPPPPPLAELERVRRIGSGAGGTVWMVRHRPTGRPYALKVLYG

NHDDAVRRQITREIAILRTAEHPAIVRCHGMYEQAGELQILLEFMDGGSLEGRRIASEAF

LADVARQVLSGIAYLHRRHIVHRDIKPSNLLIDSGRRVKIADFGVGRILNQTMDPCNSSV

GTIAYMSPERINTDLNDGAYDGYAGDIWSFGLSILEFYLGRFPLGENLGKQGDWAALMCA

ICYSDSPAPPPIASPEFKSFISCCLQKNPARRPSAAQLLQHRFIAGPQPQVLAAPPS*

>Jer1MKK6 Brdisv1Jer11011416m.p

MRGKKPLKELKLSVPAQETSVDKFLTASGTFKDGELRLNQRGLRLISEEENGDEHQSTNM

KVEDVQLSMDDLEMIQVIGKGSGGVVQLVQHKWVGTFYALKGIQMNIQEAVRKQIVQELK

INQATQSPHIVSCHQSFYHNGVIYLVLEYMDRGSLADIIKQVKTILEPYLAVLCKQVLEG

LLYLHHERHVIHRDIKPSNLLVNHKGEVKITDFGVSAVLASSIGQRDTFVGTYNYMAPER

ISGSSYDYKSDVWSLGLVILECAIGRFPYTPSEGEGWLSFYELLEAIVDQPPPGAPADQF

SPEFCSFISACIQKDPAERMSASELLNHAFIKKFEGKDLDLRILVESLEPPMNVPE*

>Jer1MKK10-1 Brdisv1Jer11001692m.p

MALLREKRLQLSLHVPTRAADAQEAGLHRRPNPAAALPLAATTPAARSSQFRVADFEKLA

VLGRGNGGTVYKVRHRETCELYALKVQHCNGDATAEAEVLSRTASPFVVRCHSVLPAAAS

GDVAMLLELVDGGSLDSIVKSRSRGQAEAFSQFPEEALAEVAAQALSGLAYLHARRIVHL

DVKPGNLLVSTGGEVKIADFGIARVLPRAGGDDVRCTAYAGTAAYMSPERFDPEAHGGHY

DPYAADVWGLGVTVLELLMGRYPLLPAGQRPSWAALMCAICFGETPALSDGEASAELRGF

VAACLHKDYRRRASVAELLAHPFVAGRDVAASKCALRKLVTEASMSP*

>Jer1MKK10-2 Brdisv1Jer11010524m.p

MALVRQRRQLPHLTLPLDHFALRPPPVPAPAPTVAASTSSEAAGLRLSDFERISLLGQGN

GGTVYKARHRRAAAQPPVALKLFVAGDPSAAREAEILRLAADAPHVVRLHAVVPSSSPAA

GAEQPPPAALALELLPGGSLAGLLRRLGRSMGERPIAAVARQALLGLDALHALRVVHRDL

KPSNLLLGSHGEVKIADFGAGKVLRRRLDPCASYVGTAAYMSPERFDPEAYSGDYDPYAA

DVWSLGLAILELYLGHFPLLPAGQRPDWAALMCAICFGDAPEAPAAASEEFRDFVARCLE

KKAGQRASVAELLEHPFIAERDAEEAKRALAALVAEAELGDL*

>Jer1MKK10-3 Brdisv1Jer11001582m.p

MALLREKRLQLSLHVPTRAAEALDAVHRRPNPVAATLAASTPAAARSSQFRLADFDKLTV

LGRGNGGTVYKVRHRETCELYALKVQHCNGDPTAAAEAEVLSRTASPFIVRCHSVLPGAA

SGDVAMLLELVDGGSLDSIVKSRRAHAFPFPEEALAEVAAQALSGLAYLHARRIVHLDIK

PGNLLVSTGGEVKVADFGIAKVLPRAGADDARCKSYAGTAAYMSPERFDPEAHGGHYDAY

AADVWGLGVTVLELLMGRYPLLPAGQRPSWPALMCAICFGETPVLSDGEASAELRGFVAA

CLRKDHTKRASVAELLAHPFVAGRDVATSKCALRKLVTEASTSP*

>Jer1MKK10-4 Brdisv1Jer11001579m.p

MALTVRQRRLPQLHISLDLPSCSFRCPNPPVAATASTSGEFRASDFEPLAVLGRGNGGTV

YKVAHRRTSAQYALKVLHGGGDPGAAAAEADVLRRAADSPYVVRCHSVFPAASGSGETAL

LLELVDGGSLDSVRRGVGVSVFFPEAALAEVAAQALAGLAHLHARRVVHRDIKPANLLVS

GAGGVKVADFGIAMAPHVGGVGVGVGVPLRVEPLGAHVGHGALVGQGAGAAAGAGDDDLG

DAEVGDLDLAGGAQEEIRRLDVAVRYATGAEVRQAGERLRRHIRHRRFREESAAADERIE

RAAVDELEEQGGGVARRRGEDRVAADDVRRVDDAADDVRFLVGGARAGVGAVDDLQRSAD

VRRCETLYTEPPLPRPRTDSLSKSFSRNSPCGGRDADVAGVAAVAGAGAGAAQRKAQVGT

SSFR*

>Jer1MKK10-5 Brdisv1Jer11001581m.p

MSRCATRRARRCARPESACAATSATAASGRNPPPPTSESSEPPSTSSRSKAAASPDAVAH

RRTSAQYALKVLHGGGDPGAAAAEADVLRRAADSPYVVRCHSVFPAASGSGETALLLELV

DGGSLDSVRRGVGVSVFFPEAALAEVAAQALAGLAHLHARRVVHRDIKPANLLVSGAGGV

KVADFGIAMVLPSRAGGERCAAAYEGTVAYMSPERFDSEGRADADPRGADVWGLGVTVLE

LLMGRYPLLPAGQKPTWAALMCAICFGELPALPEGAASTELRGFIAACLRKDHTKRASVA

ELIKHPFVAGRNMAASRLALRRLVAGA*

>Luc1MKK1 Brdisv1Luc11008350m.p

MRKPGKLALPSHESTIGKFLTQSGTFKDGDLLVNKDGLRIVHNSEEGEAPPIEPLDDHQL

SLDDLDAIKVIGKGSSGIVQLVRHKWTDQFFALKVIQLNIQESIRKQIAQELKISLSTQC

QYVVTCYQCFYVNGVISIVLEYMDGGSLADFLKTVRTIPEAYLAAICKQVLQGLMYLHHE

KRVIHRDLKPSNILINHRGEVKISDFGVSAIIASSSAQRDTFTGTFNYMAPERISGQKHG

YMSDIWSLGLVMLECATGNFPYPSPDSFYELLEAVVDQPPPSAPTDQFSPEFCSFISACI

QKEATDRSSAQVLSDHPFLSMYDDLNIDLADYFTTAGSPLATFKQIVL*

>Luc1MKK3-1 Brdisv1Luc11040018m.p

MAGLEELKKKLQPLLFDDPDKDGISTRVPFLEDNCDSYVVSDGGTINLLSRSFGEYNINE

HGFHKRSTGADESDFGEKAYRCASHDMHIFGPIGNGASSVVQRAIFIPVHRILALKKINI

FEKEKRQQILNEMRTLCEASCYPGLVEFQGAFYMPDSGQISIALEYMDGGSLADVIKVKK

SIPEQVLAHMLQKVLLGLRYLHEVRHLVHRDIKPANMLVNLKGEAKITDFGVSAGLDNTM

AMCATFVGTVTYMSPERIRNENYSYAADIWSLGLTILECATGKFPYNVNEGPANLMLQIL

DDPSPTPPADAYSPEFCSFVNDCLQKDPDARPTCEQLFGHPFIKRYENAGVDLIAYVKGV

VDPTERLKEIAEMLAVHYYLLFNGSDGLWHHMKTFYMEESTFSFSGNVYVGRNDIFDTLS

SIRKKLKGDRPREKIVHVVEKLHCRANGETGIAIRVSGSLIVGNQFLVCGEGLQAEGMPS

VEELSIDIPSKRVGQFREQFMMLPGISMGSFHISRQDLYIIQA*

>Luc1MKK3-2 Brdisv1Luc11006718m.p

MAGLEELKKKLQPLMFNDPDKDGFSTRVPFPEDTCDSYVVSDGGTINLLSRSFGEYNINE

HGFHKRSAGADESDFGEKAYRCASQDMHIFGPIGNGASSVVQRAIFIPVHRILALKKINI

FEKEKRQQILNEMRTLCEACCYPGLVEFQGAFYMPDSGQISIALEYMDGGSLADVIKVKK

SIPEPVLAHMLQKVLLGLRYLHEVRHLVHRDIKPANMLVNLKGEAKITDFGVSAGLDNTM

AMCATFVGTVTYMSPERIRNENYSYAADIWSLGLTILECATGKFPYNVNEGPANLMLQIL

DDPSPAPPENAFSSEFCSFVNDCLQKDADARPTCEQLLSHPFIKRYENAGVDLAAYVKGV

VNPEERLKQIAEMLAVHYYLLFNGSDGLWHHMKTFYMEDSTFSFSGNLYVGQSDIFDTLS

NIRTKLKGDRPREKIVHVVEKLHCRANEETGIAIRVSGSFIVSNQFLICGEGLQAEGMPS

LEELSIDIPSKRVGQFREQFIMHPGRSMGCYYISRQDLYIIQA*

>Luc1MKK3-3 Brdisv1Luc11025812m.p

MGWDGMGQRKRRHRAGIPIPTYPGLAASLEFCFVTAGACHTAPGPGSKIAASLFAALLFQ

RPPASTSRAIFFCFVIMAAGLEDLRRRVQPIFFDADGNVMPAPDDDSEVLDGGTINLLSR

SSDEYNINERGFHKRTIRSDDEYSSEKAFRCSCHDMHIFDSVGNGASSVVHRAIYVPVHR

VLALKKINIFEKERRQQILNEIITLSEACCYPGLVEFHGVFYTPDSGEIYFALEYMDGGS

LADIIRVKKFISEPVLSHMLQKVLLALRYLHEVRHLVHRDIKPANLLLNLKGDTKITDFG

VTSGLHDSIDMCATFLGSVTYMSPERIRNESYSYSADIWSLGLTALECATGRYPYDVNGG

EADLMLQILEDPSPTPPHDIYSEEFCSFINACLQKDADARPTCDQLLSHSFIKRYEGPGV

DLSEYNKSVHDPSERLSQIAHMLAVHYYLIFDGGDDQWCHMKTFYQQDSIFSFSGETHVG

KSEIFETLSRIRKMLKGNSPCEKIAHVMEKVYCRSHGEEGMRVRVSGSFIVGNEFVVCAD

GVRAEGMLSIDELSPDILSKQAGHFQEDFFMEPGTALGCYVISKQELHIADT*

>Luc1MKK4 Brdisv1Luc11032413m.p

MRPGGPPNARPQQPGTPGRARRRPDLTLPLPQRDLTSLAVPLPLPPPPSSAPSSASSSGS

SLSSMGAPTPPNSAGSAPPPPPPLAELERVRRIGSGAGGTVWMVRHRPTGRPYALKVLYG

NHDDAVRRQITREIAILRTAEHPAIVRCHGMYEQAGELQILLEFMDGGSLEGRRIASEAF

LADVARQVLSGIAYLHRRHIVHRDIKPSNLLIDSGRRVKIADFGVGRILNQTMDPCNSSV

GTIAYMSPERINTDLNDGAYDGYAGDIWSFGLSILEFYLGRFPLGENLGKQGDWAALMCA

ICYSDSPAPPPIASPEFKSFISCCLQKNPARRPSAAQLLQHRFIAGPQPQVLAAPPS*

>Luc1MKK5 Brdisv1Luc11007623m.p

MRPAGSLPSPQPGTPGRPRRRPDLTLPMPQRPDVSSSLAVPLPLPPPSSLGLAQPPAAAA

AAAAPPPPPLGELERVRRVGSGAGGTVWMVRHRPTGRCYALKQLYGNHDDAVRRQIAREI

AILRTAEHPAVVRCHGMYERGGELQILLEYMDGGSLDGRRIAAEGFLADVARQVLSGIAY

LHRRHIVHRDIKPSNLLIDSARRVKIADFGVGRILNQTMDPCNSSVGTIAYMSPERINTD

LNDGAYDGYAGDIWSFGLSILEFYLGRFPFGENLGKQGDWAALMVAICYNDPPEPSAAAS

PEFRGFISCCLQKNPAKRLSAAQLLQHPFVAGPQPLPLAAPPS*

>Luc1MKK6 Brdisv1Luc11012565m.p

MRGKKPLKELKLSVPAQETSVDKFLTASGTFKDGELRLNQRGLRLISEEENGDEHQSTNM

KVEDVQLSMDDLEMIQVIGKGSGGVVQLVQHKWVGTFYALKGIQMNIQEAVRKQIVQELK

INQATQSPHIVSCHQSFYHNGVIYLVLEYMDRGSLADIIKQVKTILEPYLAVLCKQVLEG

LLYLHHERHVIHRDIKPSNLLVNHKGEVKITDFGVSAVLASSIGQRDTFVGTYNYMAPER

ISGSSYDYKSDVWSLGLVILECAIGRFPYTPSEGEGWLSFYELLEAIVDQPPPGAPADQF

SPEFCSFISACIQKDPAERMSASELLNHAFIKKFEGKDLDLRILVESLEPPMNVPE*

>Luc1MKK10-1 Brdisv1Luc11001815m.p

MALLREKRLQLSLHVPTRAADAQEAGLHRRPNPAAALPLAATTPAARSSQFRVADFEKLA

VLGRGNGGTVYKVRHRETCELYALKVQHCNGDATAEAEVLSRTASPFVVRCHSVLPAAAS

GDVAMLLELVDGGSLDSIVKSRSRGQAEAFSQFPEEALAEVAAQALSGLAYLHARRIVHL

DVKPGNLLVSTGGEVKIADFGIARVLPRAGGDDVRCTAYAGTAAYMSPERFDPEAHGGHY

DPYAADVWGLGVTVLELLMGRYPLLPAGQRPSWAALMCAICFGETPALSDGEASAELRGF

VAACLHKDYRRRASVAELLAHPFVAGRDVAASKCALRKLVTEASMSP*

>Luc1MKK10-2 Brdisv1Luc11011611m.p

MALVRQRRQLPHLTLPLDHFALRPPPVPAPAPTVAASTSSEAAGLRLSDFERISLLGQGN

GGTVYKARHRRAAAQPPVALKLFVAGDPSAAREAEILRLAADAPHVVRLHAVVPSSSPAA

GAEQPPPAALALELLPGGSLAGLLRRLGRSMGERPIAAVARQALLGLDALHALRVVHRDL

KPSNLLLGSHGEVKIADFGAGKVLRRRLDPCASYVGTAAYMSPERFDPEAYSGDYDPYAA

DVWSLGLAILELYLGHFPLLPAGQRPDWAALMCAICFGDAPEAPAAASEEFRDFVARCLE

KKAGQRASVAELLEHPFIAERDAEEAKRALAALVAEAELGDL*

>Luc1MKK10-3 Brdisv1Luc11001706m.p

MALLREKRLQLSLHVPTRAAEALDAVHRRPNPVAATLAASTPAAARSSQFRLADFDKLTV

LGRGNGGTVYKVRHRETCELYALKVQHCNGDPTAAAEAEVLSRTASPFIVRCHSVLPGAA

SGDVAMLLELVDGGSLDSIVKSRRAHAFPFPEEALAEVAAQALSGLAYLHARRIVHLDIK

PGNLLVSTGGEVKVADFGIAKVLPRAGADDARCKSYAGTAAYMSPERFDPEAHGGHYDAY

AADVWGLGVTVLELLMGRYPLLPAGQRPSWPALMCAICFGETPVLSDGEASAELRGFVAA

CLRKDHTKRASVAELLAHPFVAGRDVATSKCALRKLVTEASTSP*

>Luc1MKK10-4 Brdisv1Luc11001703m.p

MASAKERRLPQLHLKLDVPTCAFRCAAPAPAPATAATPATSASRPPHGEFRLNDFDRLSV

LGRGNGGSVYKVSHRRTSALYALKIIHGAHARPGAADEEADIVRRVVDSPNVVRCHSVLP

TASGDAAALLLELVDGGSLDSLVGGGGFLPEAAVADVAAQALSGLAHLRARRVAHRDIKP

ANLLLSAAGEVKIADFGIAKVVVSGAGGRARALAYEGTVAYMSPERFDSERHADADPYAA

DVWGLGVTLLELLMGRYPLLPAGQKPTWAALMCAICFGELPALPEGAASLEFRGFVAACL

RKDHRKRASVVELLAHPFVAGRDVAASRRALREAIERRCSC*

>Luc1MKK10-5 Brdisv1Luc11001705m.p

MALTVRQRRLPQLHISLDLPSCSFRCPNPPVAATASTSGEFRASDFEPLAVLGRGNGGTV

YKVAHRRTSAQYALKVLHGGGDPGAAAAEADVLRRAADSPYVVRCHSVFPAASGSGETAL

LLELVDGGSLDSVRRGVGVSVFFPEAALAEVAAQALAGLAHLHARRVVHRDIKPANLLVS

GAGGVKVADFGIAMVLPSRAGGERCAAAYEGTVAYMSPERFDSEGRADADPRGADVWGLG

VTVLELLMGRYPLLPAGQKPTWAALMCAICFGELPALPEGAASTELRGFIAACLRKDHTK

RASVAELIKHPFVAGRNMAASRLALRRLVAGA*

>Mig3MKK1 Brdisv1Mig31008255m.p

MRKPGKLALPSHESTIGKFLTQSGTFKDGDLLVNKDGLRIVHNSEEGEAPPIEPLDDHQL

SLDDLDAIKVIGKGSSGIVQLVRHKWTDQFFALKVIQLNIQESIRKQIAQELKISLSTQC

QYVVTCYQCFYVNGVISIVLEYMDGGSLADFLKTVRTIPEAYLAAICKQVLQGLMYLHHE

KRVIHRDLKPSNILINHRGEVKISDFGVSAIIASSSAQRDTFTGTFNYMAPERISGQKHG

YMSDIWSLGLVMLECATGNFPYPSPDSFYELLEAVVDQPPPSAPTDQFSPEFCSFISACI

QKEATDRSSAQVLSDHPFLSMYDDLNIDLADYFTTAGSPLATFKQIVL*

>Mig3MKK3-1 Brdisv1Mig31029163m.p

MAGLEELKKKLQPLLFDDPDKDGISTRVPFLEDNCDSYVVSDGGTINLLSRSFGEYNINE

HGFHKRSTGADESDFGEKAYRCASHDMHIFGPIGNGASSVVQRAIFIPVHRILALKKINI

FEKEKRQQILNEMRTLCEASCYPGLVEFQGAFYMPDSGQISIALEYMDGGSLADVIKVKK

SIPEQVLAHMLQKVLLGLRYLHEVRHLVHRDIKPANMLVNLKGEAKITDFGVSAGLDNTM

AMCATFVGTVTYMSPERIRNENYSYAADIWSLGLTILECATGKFPYNVNEGPANLMLQIL

DDPSPTPPADAYSPEFCSFVNDCLQKDPDARPTCEQLFGHPFIKRYENAGVDLIAYVKGV

VDPTERLKEIAEMLAVHYYLLFNGSDGLWHHMKTFYMEESTFSFSGNVYVGRNDIFDTLS

SIRKKLKGDRPREKIVHVVEKLHCRANGETGIAIRVSGSLIVGNQFLVCGEGLQAEGMPS

VEELSIDIPSKRVGQFREQFMMLPGISMGSFHISRQDLYIIQA*

>Mig3MKK3-2 Brdisv1Mig31006643m.p

MAGLEELKKKLQPLMFNDPDKDGFSTRVPFPEDTCDSYVVSDGGTINLLSRSFGEYNINE

HGFHKRSAGADESDFGEKAYRCASQDMHIFGPIGNGASSVVQRAIFIPVHRILALKKINI

FEKEKRQQILNEMRTLCEACCYPGLVEFQGAFYMPDSGQISIALEYMDGGSLADVIKVKK

SIPEPVLAHMLQKVLLGLRYLHEVRHLVHRDIKPANMLVNLKGEAKITDFGVSAGLDNTM

GMCATFVGTVTYMSPERIRNENYSYAADIWSLGLTILECATGKFPYNVNEGPANLMLQIL

DDPSPAPPENAFSSEFCSFVNDCLQKDADARPTCEQLLSHPFIKRYENAGVDLAAYVKGV

VNPEERLKQIAEMLAVHYYLLFNGSDGLWHHMKTFYMEDSTFSFSGNLYVGQSDIFDTLS

NIRTKLKGDRPREKIVHVVEKLHCRANEETGIAIRVSGSFIVSNQFLICGEGLQAEGMPS

LEELSIDIPSKRVGQFREQFIMHPGRSMGCYYISRQDLYIIQA*

>Mig3MKK3-3 Brdisv1Mig31032176m.p

MGWDGMGQRKRRHRAGIPIPTYPGLAASLEFCFVTAGACHTAPGPGSKIAASLFAALLFQ

RPPASTSRAIFFCFVIMAAGLEDLRRRVQPIFFDADGNVMPAPDDDSEVLDGGTINLLSR

SSDEYNINERGFHKRTIRSDDEYSSEKAFRCSCHDMHIFDSVGNGASSVVHRAIYVPVHR

VLALKKINIFEKERRQQILNEIITLSEACCYPGLVEFHGVFYTPDSGEIYFALEYMDGGS

LADIIRVKKFISEPVLSHMLQKVLLALRYLHEVRHLVHRDIKPANLLLNLKGDTKITDFG

VTSGLHDSIDMCATFLGSVTYMSPERIRNESYSYSADIWSLGLTALECATGRYPYDVNGG

EADLMLQILEDPSPTPPHDIYSEEFCSFINACLQKDADARPTCDQLLSHSFIKRYEGPGV

DLSEYNKSVHDPSERLSQIAHMLAVHYYLIFDGGDDQWCHMKTFYQQDSIFSFSGETHVG

KSEIFETLSRIRKMLKGNSPCEKIAHVMEKVYCRSHGEEGMRVRVSGSFIVGNEFVVCAD

GVRAEGMLSIDELSPDILSKQAGHFQEDFFMEPGTALGCYVISKQELHIADT*

>Mig3MKK4 Brdisv1Mig31038354m.p

MRPGGPPNARPQQPGTPGRARRRPDLTLPLPQRDLTSLAVPLPLPPPPSSAPSSASSSGS

SLSSMGAPTPPNSAGSAPPPPPPLAELERVRRIGSGAGGTVWMVRHRPTGRPYALKVLYG

NHDDAVRRQITREIAILRTAEHPAIVRCHGMYEQAGELQILLEFMDGGSLEGRRIASEAF

LADVARQVLSGIAYLHRRHIVHRDIKPSNLLIDSGRRVKIADFGVGRILNQTMDPCNSSV

GTIAYMSPERINTDLNDGAYDGYAGDIWSFGLSILEFYLGRFPLGENLGKQGDWAALMCA

ICYSDSPAPPPIASPEFKSFISCCLQKNPARRPSAAQLLQHRFIAGPQPQVLAAPPS*

>Mig3MKK5 Brdisv1Mig31007509m.p

MRPAGSLPSPQPGTPGRPRRRPDLTLPMPQRPDVSSSLAVPLPLPPPSSLGLAQPPAAAA

AAAAPPPPPLGELERVRRVGSGAGGTVWMVRHRPTGRCYALKQLYGNHDDAVRRQIAREI

AILRTAEHPAVVRCHGMYERGGELQILLEYMDGGSLDGRRIAAEGFLADVARQVLSGIAY

LHRRHIVHRDIKPSNLLIDSARRVKIADFGVGRILNQTMDPCNSSVGTIAYMSPERINTD

LNDGAYDGYAGDIWSFGLSILEFYLGRFPFGENLGKQGDWAALMVAICYNDPPEPSAAAS

PEFRGFISCCLQKNPAKRLSAAQLLQHPFVAGPQPLPLAAPPS*

>Mig3MKK6 Brdisv1Mig31012305m.p

MRGKKPLKELKLSVPAQETSVDKFLTASGTFKDGELRLNQRGLRLISEEENGDEHQSTNM

KVEDVQLSMDDLEMIQVIGKGSGGVVQLVQHKWVGTFYALKGIQMNIQEAVRKQIVQELK

INQATQSPHIVSCHQSFYHNGVIYLVLEYMDRGSLADIIKQVKTILEPYLAVLCKQVLEG

LLYLHHERHVIHRDIKPSNLLVNHKGEVKITDFGVSAVLASSIGQRDTFVGTYNYMAPER

ISGSSYDYKSDVWSLGLVILECAIGRFPYTPSEGEGWLSFYELLEAIVDQPPPGAPEDQF

SPEFCSFISACIQKDPAERMSASELLNHAFIKKFEGKDLDLRILVESLEPPMNVPE*

>Mig3MKK10-1 Brdisv1Mig31001882m.p

MALLREKRLQLSLHVPTRAADAQEAGLHRRPNPAAALPLAAPTPAARSSQFRVADFEKLA

VLGRGNGGTVYKVRHRETCELYALKVQHCNGDATAEAEVLSRTASPFVVRCHSVLPAAAS

GDVAMLLELVDGGSLDSIVKSRSRGQAEAFSQFPEEALAEVAAQALSGLAYLHARRIVHL

DVKPGNLLVSTGGEVKIADFGIARVLPRAGGDDVRCTAYAGTAAYMSPERFDPEAHGGHY

DPYAADVWGLGVTVLELLMGRYPLLPAGQRPSWAALMCAICFGETPALSDGEASAELRGF

VAACLHKDYRRRASVAELLAHPFVAGRDVAASKCALRKLVTEASMSP*

>Mig3MKK10-2 Brdisv1Mig31011374m.p

MALVRQRRQLPHLTLPLDHFALRPPPVPAPAPTVAASTSSEAAGLRLSDFERISLLGQGN

GGTVYKARHRRAAAQPPVALKLFVAGDPSAAREAEILRLAADAPHVVRLHAVVPSSSPAA

GAEQPPPAALALELLPGGSLAGLLRRLGRSMGERPIAAVARQALLGLDALHALRVVHRDL

KPSNLLLGSHGEVKIADFGAGKVLRRRLDPCASYVGTAAYMSPERFDPEAYSGDYDPYAA

DVWSLGLAILELYLGHFPLLPAGQRPDWAALMCAICFGDAPEAPAAASEEFRDFVARCLE

KKAGQRASVAELLEHPFIAERDAEEAKRALAALVAEAELGDL*

>Mig3MKK10-3 Brdisv1Mig31001763m.p

MALLREKRLQLSLHVPTRAAEALDAVHRRPNPVAATLAASTPAAARASQFRLADFDKLTV

LGRGNGGTVYKVRHRETCELYALKVQHCNGDPTAAAEAEVLSRTASPFIVRCHSVLPGAA

SGDVAMLLELVDGGSLDSIVKSRRAHAFPFPEEALAEVAAQALSGLAYLHARRIVHLDIK

PGNLLVSTGGEVKVADFGIAKVLPRAGADDARCKSYAGTAAYMSPERFDPEAHGGHYDAY

AADVWGLGVTVLELLMGRYPLLPAGQRPSWPALMCAICFGETPVLSDGEASAELRGFVAA

CLRKDHTKRASVAELLAHPFVAGRDVATSKCALRKLVTEASTSP*

>Mig3MKK10-4 Brdisv1Mig31001757m.p

MASAKERRLPQLHLKLDVPTCAFRCAAPAPAPATAATPATSASRPPHGEFRLNDFDRLSV

LGRGNGGSVYKVSHRRTSALYALKIIHGAHARPGAADEEADIVRRVVDSPNVVRCHSVLP

TASGDAAALLLELVDGGSLDSLVGGGGFLPEAAVADVAAQALSGLAPLRARRVAHRDIKP

ANLLLSAAGEVKIADFGIAKVVVSGAGGRARALAYEGTVAYMSPERFDSERHADADPYAA

DVWGLGVTLLELLMGRYPLLPAGQKPTWAALMCAICFGELPALPEGAASLEFRGFVAACL

RKDHRKRASVVELLAHPFVAGRDVAASRRALREAIERRCSC*

>Mig3MKK10-5 Brdisv1Mig31001759m.p

MALTVRQRRLPQLHISLDLPSCSFRCPNPPVAATASTSGEFRASDFEPLAVLGRGNGGTV

YKVAHRRTSAQYALKVLHGGGDPGAAAAEADVLRRAADSPYVVRCHSVFPAASGSGETAL

LLELVDGGSLASVRRGVGVSVFFPEAALAEVAAQALAGLAHLHARRVVHRDIKPANLLVS

GAGGVKVADFGIAMVLPSRAGGERCAAAYEGTVAYMSPERFDSEGRADADPRGADVWGLG

VTVLELLMGRYPLLPAGQKPTWAALMCAICFGELPALPEGAASTELRGFIAACLRKDHTK

RASVAELIKHPFVAGRNMAASRLALRRLVAGA*

>Mur1MKK1 Brdisv1Mur11007592m.p

MRKPGKLALPSHESTIGKFLTQSGTFKDGDLLVNKDGLRIVHNSEEGEAPPIEPLDDHQL

SLDDLDAIKVIGKGSSGIVQLVRHKWTDQFFALKVIQLNIQESIRKQIAQELKISLSTQC

QYVVTCYQCFYVNGVISIVLEYMDGGSLADFLKTVRTIPEAYLAAICKQVLQGLMYLHHE

KRVIHRDLKPSNILINHRGEVKISDFGVSAIIASSSAQRDTFTGTFNYMAPERISGQKHG

YMSDIWSLGLVMLECATGNFPYPSPDSFYELLEAVVDQPPPSAPTDQFSPEFCSFISACI

QKEATDRSSAQVLSDHPFLSMYDDLNIDLADYFTTAGSPLATFKQIVL*

>Mur1MKK3-1 Brdisv1Mur11026956m.p

MAGLEELKKKLQPLLFDDPDKDGISTRVPFLEDNCDSYVVSDGGTINLLSRSFGEYNINE

HGFHKRSTGADESDFGEKAYRCASHDMHIFGPIGNGASSVVQRAIFIPVHRILALKKINI

FEKEKRQQILNEMRTLCEASCYPGLVEFQGAFYMPDSGQISIALEYMDGGSLADVIKVKK

SIPEQVLAHMLQKVLLGLRYLHEVRHLVHRDIKPANMLVNLKGEAKITDFGVSAGLDNTM

AMCATFVGTVTYMSPERIRNENYSYAADIWSLGLTILECATGKFPYNVNEGPANLMLQIL

DDPSPTPPADAYSPEFCSFVNDCLQKDPDARPTCEQLFGHPFIKRYENAGVDLIAYVKGV

VDPTERLKEIAEMLAVHYYLLFNGSDGLWHHMKTFYMEESTFSFSGNVYVGRNDIFDTLS

SIRKKLKGDRPREKIVHVVEKLHCRANGETGIAIRVSGSLIVGNQFLVCGEGLQAEGMPS

VEELSIDIPSKRVGQFREQFMMLPGISMGSFHISRQDLYIIQA*

>Mur1MKK3-2 Brdisv1Mur11040779m.p

MAGLEELKKKLQPLMFNDPDKDGFSTRVPFPEDTCDSYVVSDGGTINLLSRSFGEYNINE

HGFHKRSAGADESDFGEKAYRCASQDMHIFGPIGNGASSVVQRAIFIPVHRILALKKINI

FEKEKRQQILNEMRTLCEACCYPGLVEFQGAFYMPDSGQISIALEYMDGGSLADVIKVKK

SIPEPVLAHMLQKVLLGLRYLHEVRHLVHRDIKPANMLVNLKGEAKITDFGVSAGLDNTM

AMCATFVGTVTYMSPERIRNENYSYAADIWSLGLTILECATGKFPYNVNEGPANLMLQIL

DDPSPAPPENAFSSEFCSFVNDCLQKDADARPTCEQLLSHPFIKRYENAGVDLAAYVKGV

VNPEERLKQIAEMLAVHYYLLFNGSDGLWHHMKTFYMEDSTFSFSGNLYVGQSDIFDTLS

NIRTKLKGDRPREKIVHVVEKLHCRANEETGIAIRVSGSFIVSNQFLICGEGLQAEGMPS

LEELSIDIPSKRVGQFREQFIMHPGRSMGCYYISRQDLYIIQA*

>Mur1MKK3-3 Brdisv1Mur11029722m.p

MGWDGMGQRKRRHRAGIPIPTYPGLAASLEFCFLTAGACHTAPGPGSKIAASLFAALLFQ

RPPASTSRAIFFCFGIMAAGLEDLRRRVQPIFFDADGNVMPAPDDDSEVLDGGTINLLSR

SSDEYNINERGFHKRTIRSDDEYSSEKAFRCSCHDMHIFDSVGNGASSVVHRAIYVPVHR

VLALKKINIFEKERRQQILNEIITLSEACCYPGLVEFHGVFYTPDSGEIYFALEYMDGGS

LADIIRVKKFISEPVLSHMLQKVLLALRYLHEVRHLVHRDIKPANLLLNLKGDTKITDFG

VTSGLHDSIDMCATFLGSVTYMSPERIRNESYSYSADIWSLGLTALECATGRYPYDVNGG

EADLMLQILEDPSPTPPHDIYSEEFCSFINACLQKDADARPTCDQLLSHSFIKRYEGPGV

DLSEYNKSVHDPSERLSQIAHMLAVHYYLIFDGGDDQWCHMKTFYQQDSIFSFSGETHVG

KSEIFETLSRIRKMLKGNSPCEKIAHVMEKVYCRSHGEEGMRVRVSGSFIVGNEFVVCAD

GVRAEGMLSIDELSPDILSKQAGHFQEDFFMEPGTALGCYVISKQELHIADT*

>Mur1MKK4 Brdisv1Mur11035455m.p

MGAPTPPNSAGSAPPPPPPLAELERVRRIGSGAGGTVWMVRHRPTGRPYALKVLYGNHDD

AVRRQITREIAILRTAEHPAIVRCHGMYEQAGELQILLEFMDGGSLEGRRIASEAFLADV

ARQVLSGIAYLHRRHIVHRDIKPSNLLIDSGRRVKIADFGVGRILNQTMDPCNSSVGTIA

YMSPERINTDLNDGAYDGYAGDIWSFGLSILEFYLGRFPLGENLGKQGDWAALMCAICYS

DSPAPPPIASPEFKSFISCCLQKNPARRPSAAQLLQPRFIAGPQPQVLAAPPS*

>Mur1MKK5 Brdisv1Mur11006903m.p

MRPAGSLPSPQPGTPGRPRRRPDLTLPMPQRPDVSSSLAVPLPLPPPSSLGLAQPPAAAA

AAAAPPPPPLGELERVRRVGSGAGGTVWMVRHRPTGRCYALKQLYGNHDDAVRRQIAREI

AILRTAEHPAVVRCHGMYERGGELQILLEYMDGGSLDGRRIAAEGFLADVARQVLSGIAY

LHRRHIVHRDIKPSNLLIDSARRVKIADFGVGRILNQTMDPCNSSVGTIAYMSPERINTD

LNDGAYDGYAGDIWSFGLSILEFYLGRFPFGENLGKQGDWAALMVAICYNDPPEPSAAAS

PEFRGFISCCLQKNPAKRLSAAQLLQHPFVAGPQPLPLAAPPS*

>Mur1MKK6 Brdisv1Mur11011361m.p

MRGKKPLKELKLSVPAQETSVDKFLTASGTFKDGELRLNQRGLRLISEEENGDEHQSTNM

KVEDVQLSMDDLEMIQVIGKGSGGVVQLVQHKWVGTFYALKGIQMNIQEAVRKQIVQELK

INQATQSPHIVSCHQSFYHNGVIYLVLEYMDRGSLADIIKQVKTILEPYLAVLCKQVLEG

LLYLHHERHVIHRDIKPSNLLVNHKGEVKITDFGVSAVLASSIGQRDTFVGTYNYMAPER

ISGSSYDYKSDVWSLGLVILECAIGRFPYTPSEGEGWLSFYELLEAIVDQPPPGAPADQF

SPEFCSFISACIQKDPAERMSASELLNHAFIKKFEGKDLDLRILVESLEPPMNVPE*

>Mur1MKK10-1 Brdisv1Mur11001679m.p

MALLREKRLQLSLHVPTRAADAQEAGLHRRPNPAAALPLAATTPAARSSQFRVADFEKLA

VLGRGNGGTVYKVRHRETCELYALKVQHCNGDATAEAEVLSRTASPFVVRCHSVLPAAAS

GDVAMLLELVDGGSLDSIVKSRSRGQAEAFSQFPEEALAEVAAQALSGLAYLHARRIVHL

DVKPGNLLVSTGGEVKIADFGIARVLPRAGGDDVRCTAYAGTAAYMSPERFDPEAHGGHY

DPYAADVWGLGVTVLELLMGRYPLLPAGQRPSWAALMCAICFGETPALSDGEASAELRGF

VAACLHKDYRRRASVAELLAHPFVAGRDVAASKCALRKLVTEASMSP*

>Mur1MKK10-2 Brdisv1Mur11010501m.p

MALVRQRRQLPHLTLPLDHFALRPPPVPAPAPTVAASTSSEAAGLRLSDFERISLLGQGN

GGTVYKARHRRAAAQPPVALKLFVAGDPSAAREAEILRLAADAPHVVRLHAVVPSSSPAA

GAEQPPPAALALELLPGGSLAGLLRRLGRSMGERPIAAVARQALLGLDALHALRVVHRDL

KPSNLLLGSHGEVKIADFGAGKVLRRRLDPCASYVGTAAYMSPERFDPEAYSGDYDPYAA

DVWSLGLAILELYLGHFPLLPAGQRPDWAALMCAICFGDAPEAPAAASEEFRDFVARCLE

KKAGQRASVAELLEHPFIAERDAEEAKRALAALVAEAELGDL*

>Mur1MKK10-3 Brdisv1Mur11001569m.p

MALLREKRLQLSLHVPTRAAEALDAVHRRPNPVAATLAASTPAAARASQFRLADFDKLTV

LGRGNGGTVYKVRHRETCELYALKVQHCNGDPTAAAEAEVLSRTASPFIVRCHSVLPGAA

SGDVAMLLELVDGGSLDSIVKSRRAHAFPFPEEALAEVAAQALSGLAYLHARRIVHLDIK

PGNLLVSTGGEVKVADFGIAKVLPRAGADDARCKSYAGTAAYMSPERFDPEAHGGHYDAY

AADVWGLGVTVLELLMGRYPLLPAGQRPSWPALMCAICFGETPVLSDGEASAELRGFVAA

CLRKDHTKRASVAELLAHPFVAGRDVATSKCALRKLVTEASTSP*

>Mur1MKK10-4 Brdisv1Mur11001565m.p

MASAKERRLPQLHLKLDVPTCAFRCAAPAPAPATAATPATSASRPPHGEFRLNDFDRLSV

LGRGNGGSVYKVSHRRTSALYALKIIHGAHARPGAADEEADIVRRVVDSPNVVRCHSVLP

TASGDAAALLLELVDGGSLDSLVGGGGFLPEAAVADVAAQALSGLAHLRARRVAHRDIKP

ANLLLSAAGEVKIADFGIAKVVVSGAGGRARALAYEGTVAYMSPERFDSERHADADPYAA

DVWGLGVTLLELLMGRYPLLPAGQKPTWAALMCAICFGELPALPEGAASLEFRGFVAACL

RKDHRKRASVVELLAHPFVAGRDVAASRRALREAIERRCSC*

>Mur1MKK10-5 Brdisv1Mur11044815m.p

MALTVRQRRLPQLHISLDLPSCSFRCPNPPVAATASTSGEFRASDFEPLAVLGRGNGGTV

YKVAHRRTSAQYALKVLHGGGDPGAAAAEADVLRRAADSPYVVRCPSVFPAASGSGETAL

LLELVDGGSLDSVRRGVGVSVFFPEAALAEVAAQALAGLAHLHARRVVHRDIKPXRLLAV

ALPLHGKL*

>Per1MKK1 Brdisv1Per11008500m.p

MRKPGKLALPSHESTIGKFLTQSGTFKDGDLLVNKDGLRIVHNSEEGEAPPIEPLDDHQL

SLDDLDAIKVIGKGSSGIVQLVRHKWTDQFFALKVIQLNIQESIRKQIAQELKISLSTQC

QYVVTCYQCFYVNGVISIVLEYMDGGSLADFLKTVRTIPEAYLAAICKQASETCHTRNQF

CYWNVLQGLMYLHHEKRVIHRDLKPSNILINHRGEVKISDFGVSAIIASSSAQRDTFTGT

FNYMAPERISGQKHGYMSDIWSLGLVMLECATGNFPYPSPDSFYELLEAVVDQPPPSAPT

DQFSPEFCSFISACIQKEATDRSSAQVLSDHPFLSMYDDLNIDLADYFTTAGSPLATFKQ

IVL*

>Per1MKK3-1 Brdisv1Per11039460m.p

MAGLEELKKKLQPLLFDDPDKDGISTRVPFPEDNCDSYVVSDGGTINLLSRSFGEYNINE

HGFHKRSTGADESDFGEKAYRCASHDMHIFGPIGNGASSVVQRAIFIPVHRILALKKINI

FEKEKRQQILNEMRTLCEASCYPGLVEFQGAFYMPDSGQISIALEYMDGGSLADVIKVKK

SIPEQVLAHMLQKVLLGLRYLHEVRHLVHRDIKPANMLVNLKGEAKITDFGVSAGLDNTM

AMCATFVGTVTYMSPERIRNENYSYAADIWSLGLTILECATGKFPYNVNEGPANLMLQIL

DDPSPTPPADAYSPEFCSFVNDCLQKDPDARPTCEQLFGHPFIKRYENAGVDLIAYVKGV

VDPTERLKEIAEMLAVHYYLLFNGSDGLWHHMKTFYMEESTFSFSGNVYVGRNDIFDTLS

SIRKKLKGDRPREKIVHVVEKLHCRANGETGIAIRVSGSLIVGNQFLVCGEGLQAEGMPS

VEELSIDIPSKRVGQFREQFMMLPGISMGSFHISRQDLYIIQA*

>Per1MKK3-2 Brdisv1Per11006708m.p

MAGLEELKKKLQPLMFNDPDKDGFSTRVPFPEDTCDSYVVSDGGTINLLSRSFGEYNINE

HGFHKRSAGADESDFGEKAYRCASQDMHIFGPIGNGASSVVQRAIFIPVHRILALKKINI

FEKEKRQQILNEMRTLCEACCYPGLVEFQGAFYMPDSGQISIALEYMDGGSLADVIKVKK

SIPEPVLAHMLQKVLLGLRYLHEVRHLVHRDIKPANMLVNLKGEAKITDFGVSAGLDNTM

AMCATFVGTVTYMSPERIRNENYSYAADIWSLGLTILECATGKFPYNVNEGPANLMLQIL

DDPSPAPPENAFSSEFCSFVNDCLQKDADARPTCEQLLSHPFIKRYENAGVDLAAYVKGV

VNPEERLKQIAEMLAVHYYLLFNGSDGLWHHMKTFYMEDSTFSFSGNLYVGQSDIFDTLS

NIRTKLKGDRPREKIVHVVEKLHCRANEETGIAIRVSGSFIVSNQFLICGEGLQAEGMPS

LEELSIDIPSKRVGQFREQFIMHPGRSMGCYYISRQDLYIIQA*

>Per1MKK3-3 Brdisv1Per11025553m.p

MGWDGMGQRKRRHRAGIPIPTYPGLAASLEFCFVTAGACHTAPGPGSKIAASLFAALLFQ

RPPASTSRAIFFCFVIMAAGLEDLRRRVQPIFFDADGNVMPAPDDDSEVLDGGTINLLSR

SSDEYNINERGFHKRTIRSDDEYSSEKAFRCSCHDMHIFDSVGNGASSVVHRAIYVPVHR

VLALKKINIFEKERRQQILNEIITLSEACCYPGLVEFHGVFYTPDSGEIYFALEYMDGGS

LADIIRVKKFISEPVLSHMLQKVLLALRYLHEVRHLVHRDIKPANLLLNLKGDTKITDFG

VTSGLHDSIDMCATFLGSVTYMSPERIRNESYSYSADIWSLGLTALECATGRYPYDVNGG

EADLMLQILEDPSPTPPHDIYSEEFCSFINACLQKDADARPTCDQLLSHSFIKRYEGPGV

DLSEYNKSVHDPSERLSQIAHMLAVHYYLIFDGGDDQWCHMKTFYQQDSIFSFSGETHVG

KSEIFETLSRIRKMLKGNSPCEKIAHVMEKVYCRSHGEEGMRVRVSGSFIVGNEFVVCAD

GVRAEGMLSIDELSPDILSKQAGHFQEDFFMEPGTALGCYVISKQELHIADT*

>Per1MKK4 Brdisv1Per11032157m.p

MRPGGPPNARPQQPGTPGRARRRPDLTLPLPQRDLTSLAVPLPLPPPPSSAPSSASSSGS

SLSSMGAPTPPNSAGSAPPPPPPLAELERVRRIGSGAGGTVLYGNHDDAVRRQITREIAI

LRTAEHPAIVRCHGMYEQAGELQILLEFMDGGSLEGRRIASEAFLADVARQVLSGIAYLH

RRHIVHRDIKPSNLLIDSGRRVKIADFGVGRILNQTMDPCNSSVGTIAYMSPERINTDLN

DGAYDGYAGDIWSFGLSILEFYLGRFPLGENLGKQGDWAALMCAICYSDSPAPPPIASPE

FKSFISCCLQKNPARRPSAAQLLQHRFIAGPQPQVLAAPPS*

>Per1MKK5 Brdisv1Per11007710m.p

MRPAGSLPSPQPGTPGRPRRRPDLTLPMPQRPDVSSSLAVPLPLPPPSSLGLAQPPAAAA

AAAAPPPPPLGELERVRRVGSGAGGTVWMVRHRPTGRCYALKQLYGNHDDAVRRQIAREI

AILRTAEHPAVVRCHGMYERGGELQILLEYMDGGSLDGRRIAAEGFLADVARQVLSGIAY

LHRRHIVHRDIKPSNLLIDSARRVKIADFGVGRILNQTMDPCNSSVGTIAYMSPERINTD

LNDGAYDGYAGDIWSFGLSILEFYLGRFPFGENLGKQGDWAALMVAICYNDPPEPSAAAS

PEFRGFISCCLQKNPAKRLSAAQLLQHPFVAGPQPLPLAAPPS*

>Per1MKK6 Brdisv1Per11012631m.p

MRGKKPLKELKLSVPAQETSVDKFLTASGTFKDGELRLNQRGLRLISEEENGDEHQSTNM

KVEDVQLSMDDLEMIQVIGKGSGGVVQLVQHKWVGTFYALKGIQMNIQEAVRKQIVQELK

INQATQSPHIVSCHQSFYHNGVIYLVLEYMDRGSLADIIKQVKTILEPYLAVLCKQVLEG

LLYLHHERHVIHRDIKPSNLLVNHKGEVKITDFGVSAVLASSIGQRDTFVGTYNYMAPER

ISGSSYDYKSDVWSLGLVILECAIGRFPYTPSEGEGWLSFYELLEAIVDQPPPGAPEDQF

SPEFCSFISACIQKDPAERMSASELLNHAFIKKFEGKDLDLRILVESLEPPMNVPE*

>Per1MKK10-1 Brdisv1Per11001811m.p

MALLREKRLQLSLHVPTRAADAQEAGLHRRPNPAAALPLAATTPAARSSQFRVADFEKLA

VLGRGNGGTVYKVRHRETCELYALKVQHCNGDATAEAEVLSRTASPFVVRCHSVLPAAAS

GDVAMLLELVDGGSLDSIVKSRSRGQAEAFSQFPEEALAEVAAQALSGLAYLHARRIVHL

DVKPGNLLVSTGGEVKIADFGIARVLPRAGGDDVRCTAYAGTAAYMSPERFDPEAHGGHY

DPYAADVWGLGVTVLELLMGRYPLLPAGQRPSWAALMCAICFGETPALSDGEASAELRGF

VAACLHKDYRRRASVAELLAHPFVAGRDVAASKCALRKLVTEASMSP*

>Per1MKK10-2 Brdisv1Per11011705m.p

MALVRQRRQLPHLTLPLDHFALRPPPVPAPAPTVAASTSSEAAGLRLSDFERISLLGQGN

GGTVYKARHRRAAAQPPVALKLFVAGDPSAAREAEILRLAADAPHVVRLHAVVPSSSPAA

GAEQPPPAALALELLPGGSLAGLLRRLGRSMGERPIAAVARQALLGLDALHALRVVHRDL

KPSNLLLGSHGEVKIADFGAGKVLRRRLDPCASYVGTAAYMSPERFDPEAYSGDYDPYAA

DVWSLGLAILELYLGHFPLLPAGQRPDWAALMCAICFGDAPEAPAAASEEFRDFVARCLE

KKAGQRASVAELLEHPFIAERDAEEAKRALAALVAEAELGDL*

>Per1MKK10-3 Brdisv1Per11001709m.p

MALLREKRLQLSLHVPTRAAEALDAVHRRPNPVAATLAASTPAAARSSQFRLADFDKLTV

LGRGNGGTVYKVRHRETCELYALKVQHCNGDPTAAAEAEVLSRTASPFIVRCHSVLPGAA

SGDVAMLLELVDGGSLDSIVKSRRAHAFPFPEEALAEVAAQALSGLAYLHARRIVHLDIK

PGNLLVSTGGEVKVADFGIAKVLPRAGADDARCKSYAGTAAYMSPERFDPEAHGGHYDAY

AADVWGLGVTVLELLMGRYPLLPAGQRPSWPALMCAICFGETPVLSDGEASAELRGFVAA

CLRKDHTKRASVAELLAHPFVAGRDVATSKCALRKLVTEASTSP*

>Per1MKK10-4 Brdisv1Per11001706m.p

MASAKERRLPQLHLKLDVPTCAFRCAAPAPAPATAATPATSASRPPHGEFRLNDFDRLSV

LGRGNGGSVYKVSHRRTSALYALKIIHGAHARPGAADEEADIVRRVVDSPNVVRCHSVLP

TASGDAAALLLELVDGGSLDSLVGGGGFLPEAAVADVAAQALSGLAHLRARRVAHRDIKP

ANLLLSAAGEVKIADFGIAKVVVSGAGGRARALAYEGTVAYMSPERFDSERHADADPYAA

DVWGLGVTLLELLMGRYPLLPAGQKPTWAALMCAICFGELPALPEGAASLEFRGFVAACL

RKDHRKRASVVELLAHPFVAGRDVAASRRALREAIERRCSC*

>Per1MKK10-5 Brdisv1Per11001708m.p

MALTVRQRRLPQLHISLDLPSCSFRCPNPPVAATASTSGEFRASDFEPLAVLGRGNGGTV

YKVAHRRTSAQYALKVLHGGGDPGAAAAEADVLRRAADSPYVVRCHSVFPAASGSGETAL

LLELVDGGSLDSVRRGVGVSVFFPEAALAEVAAQALAGLAHLHARRVVHRDIKPANLLVS

GAGGVKVADFGIAMVLPSRAGGERCAAAYEGTVAYMSPERFDSEGRADADPRGADVWGLG

VTVLELLMGRYPLLPAGQKPTWAALMCAICFGELPALPEGAASTELRGFIAACLRKDHTK

RASVAELIKHPFVAGRNMAASRLALRRLVAGA*

>RON2MKK1 Brdisv1RON21008490m.p

MRKPGKLALPSHESTIGKFLTQSGTFKDGDLLVNKDGLRIVHNSEEGEAPPIEPLDDHQL

SLDDLDAIKVIGKGSSGIVQLVRHKWTDQFFALKVIQLNIQESIRKQIAQELKISLSTQC

QYVVTCYQCFYVNGVISIVLEYMDGGSLADFLKTVRTIPEAYLAAICKQVLQGLMYLHHE

KRVIHRDLKPSNILINHRGEVKISDFGVSAIIASSSAQRDTFTGTFNYMAPERISGQKHG

YMSDIWSLGLVMLECATGNFPYPSPDSFYELLEAVVDQPPPSAPTDQFSPEFCSFISACI

QKEATDRSSAQVLSDHPFLSMYDDLNIDLADYFTTAGSPLATFKQIVL*

>RON2MKK3-1 Brdisv1RON21040413m.p

MAGLEELKKKLQPLLFDDPDKDGISTRVPFLEDNCDSYVVSDGGTINLLSRSFGEYNINE

HGFHKRSTGADESDFGEKAYRCASHDMHIFGPIGNGASSVVQRAIFIPVHRILALKKINI

FEKEKRQQILNEMRTLCEASCYPGLVEFQGAFYMPDSGQISIALEYMDGGSLADVIKVKK

SIPEQVLAHMLQKVLLGLRYLHEVRHLVHRDIKPANMLVNLKGEAKITDFGVSAGLDNTM

AMCATFVGTVTYMSPERIRNENYSYAADIWSLGLTILECATGKFPYNVNEGPANLMLQIL

DDPSPTPPADAYSPEFCSFVNDCLQKDPDARPTCEQLFGHPFIKRYENAGVDLIAYVKGV

VDPTERLKEIAEMLAVHYYLLFNGSDGLWHHMKTFYMEESTFSFSGNVYVGRNDIFDTLS

SIRKKLKGDRPREKIVHVVEKLHCRANGETGIAIRVSGSLIVGNQFLVCGEGLQAEGMPS

VEELSIDIPSKRVGQFREQFMMLPGISMGSFHISRQDLYIIQA*

>RON2MKK3-2 Brdisv1RON21006777m.p

MAGLEELKKKLQPLMFNDPDKDGFSTRVPFPEDTCDSYVVSDGGTINLLSRSFGEYNINE

HGFHKRSAGADESDFGEKAYRCASQDMHIFGPIGNGASSVVQRAIFIPVHRILALKKINI

FEKEKRQQILNEMRTLCEACCYPGLVEFQGAFYMPDSGQISIALEYMDGGSLADVIKVKK

SIPEPVLAHMLQKVLLGLRYLHEVRHLVHRDIKPANMLVNLKGEAKITDFGVSAGLDNTM

AMCATFVGTVTYMSPERIRNENYSYAADIWSLGLTILECATGKFPYNVNEGPANLMLQIL

DDPSPAPPENAFSSEFCSFVNDCLQKDADARPTCEQLLSHPFIKRYENAGVDLAAYVKGV

VNPEERLKQIAEMLAVHYYLLFNGSDGLWHHMKTFYMEDSTFSFSGNLYVGQSDIFDTLS

NIRTKLKGDRPREKIVHVVEKLHCRANEETGIAIRVSGSFIVSNQFLICGEGLQAEGMPS

LEELSIDIPSKRVGQFREQFIMHPGRSMGCYYISRQDLYIIQA*

>RON2MKK3-3 Brdisv1RON21025893m.p

MGWDGMGQRKRRHRAGIPIPTYPGLAASLEFCFVTAGACHTAPGPGSKIAASLFAALLFQ

RPPASTSRAIFFCFVIMAAGLEDLRRRVQPIFFDADGNVMPAPDDDSEVLDGGTINLLSR

SSDEYNINERGFHKRTIRSDDEYSSEKAFRCSCHDMHIFDSVGNGASSVVHRAIYVPVHR

VLALKKINIFEKERRQQILNEIITLSEACCYPGLVEFHGVFYTPDSGEIYFALEYMDGGS

LADIIRVKKFISEPVLSHMLQKVLLALRYLHEVRHLVHRDIKPANLLLNLKGDTKITDFG

VTSGLHDSIDMCATFLGSVTYMSPERIRNESYSYSADIWSLGLTALECATGRYPYDVNGG

EADLMLQILEDPSPTPPHDIYSEEFCSFINACLQKDADARPTCDQLLSHSFIKRYEGPGV

DLSEYNKSVHDPSERLSQIAHMLAVHYYLIFDGGDDQWCHMKTFYQQDSIFSFSGETHVG

KSEIFETLSRIRKMLKGNSPCEKIAHVMEKVYCRSHGEEGMRVRVSGSFIVGNEFVVCAD

GVRAEGMLSIDELSPDILSKQAGHFQEDFFMEPGTALGCYVISKQELHIADT*

>RON2MKK4 Brdisv1RON21032486m.p

MRPGGPPNARPQQPGTPGRARRRPDLTLPLPQRDLTSLAVPLPLPPPPSSAPSSASSSGS

SLSSMGAPTPPNSAGSAPPPPPPLAELERVRRIGSGAGGTVWMVRHRPTGRPYALKVLYG

NHDDAVRRQITREIAILRTAEHPAIVRCHGMYEQAGELQILLEFMDGGSLEGRRIASEAF

LADVARQVLSGIAYLHRRHIVHRDIKPSNLLIDSGRRVKIADFGVGRILNQTMDPCNSSV

GTIAYMSPERINTDLNDGAYDGYAGDIWSFGLSILEFYLGRFPLGENLGKQGDWAALMCA

ICYSDSPAPPPIASPEFKSFISCCLQKNPARRPSAAQLLQHRFIAGPQPQVLAAPPS*

>RON2MKK5 Brdisv1RON21007695m.p

MRPAGSLPSPQPGTPGRPRRRPDLTLPMPQRPDVSSSLAVPLPLPPPSSLGLAQPPAAAA

AAAAPPPPPLGELERVRRVGSGAGGTVWMVRHRPTGRCYALKQLYGNHDDAVRRQIAREI

AILRTAEHPAVVRCHGMYERGGELQILLEYMDGGSLDGRRIAAEGFLADVARQVLSGIAY

LHRRHIVHRDIKPSNLLIDSARRVKIADFGVGRILNQTMDPCNSSVGTIAYMSPERINTD

LNDGAYDGYAGDIWSFGLSILEFYLGRFPFGENLGKQGDWAALMVAICYNDPPEPSAAAS

PEFRGFISCCLQKNPAKRLSAAQLLQHPFVAGPQPLPLAAPPS*

>RON2MKK6 Brdisv1RON21012535m.p

MRGKKPLKELKLSVPAQETSVDKFLTASGTFKDGELRLNQRGLRLISEEENGDEHQSTNM

KVEDVQLSMDDLEMIQVIGKGSGGVVQLVQHKWVGTFYALKGIQMNIQEAVRKQIVQELK

INQATQSPHIVSCHQSFYHNGVIYLVLEYMDRGSLADIIKQVKTILEPYLAVLCKQVLEG

LLYLHHERHVIHRDIKPSNLLVNHKGEVKITDFGVSAVLASSIGQRDTFVGTYNYMAPER

ISGSSYDYKSDVWSLGLVILECAIGRFPYTPSEGEGWLSFYELLEAIVDQPPPGAPADQF

SPEFCSFISACIQKDPAERMSASELLNHAFIKKFEGKDLDLRILVESLEPPMNVPE*

>RON2MKK10-1 Brdisv1RON21001840m.p

MALLREKRLQLSLHVPTRAADAQEAGLHRRPNPAAALPLAATTPAARSSQFRVADFEKLA

VLGRGNGGTVYKVRHRETCELYALKVQHCNGDATAEAEVLSRTASPFVVRCHSVLPAAAS

GDVAMLLELVDGGSLDSIVKSRSRGQAEAFSQFPEEALAEVAAQALSGLAYLHARRIVHL

DVKPGNLLVSTGGEVKIADFGIARVLPRAGGDDVRCTAYAGTAAYMSPERFDPEAHGGHY

DPYAADVWGLGVTVLELLMGRYPLLPAGQRPSWAALMCAICFGETPALSDGEASAELRGF

VAACLHKDYRRRASVAELLAHPFVAGRDVAASKCALRKLVTEASMSP*

>RON2MKK10-2 Brdisv1RON21011610m.p

MALVRQRRQLPHLTLPLDHFALRPPPVPAPAPTVAASTSSEAAGLRLSDFERISLLGQGN

GGTVYKARHRRAAAQPPVALKLFVAGDPSAAREAEILRLAADAPHVVRLHAVVPSSSPAA

GAEQPPPAALALELLPGGSLAGLLRRLGRSMGERPIAAVARQALLGLDALHALRVVHRDL

KPSNLLLGSHGEVKIADFGAGKVLRRRLDPCASYVGTAAYMSPERFDPEAYSGDYDPYAA

DVWSLGLAILELYLGHFPLLPAGQRPDWAALMCAICFGDAPEAPAAASEEFRDFVARCLE

KKAGQRASVAELLEHPFIAERDAEEAKRALAALVAEAELGDL*

>RON2MKK10-3 Brdisv1RON21001729m.p

MALLREKRLQLSLHVPTRAAEALDAVHRRPNPVAATLAASTPAAARSSQFRLADFDKLTV

LGRGNGGTVYKVRHRETCELYALKVQHCNGDPTAAAEAEVLSRTASPFIVRCHSVLPGAA

SGDVAMLLELVDGGSLDSIVKSRRAHAFPFPEEALAEVAAQALSGLAYLHARRIVHLDIK

PGNLLVSTGGEVKVADFGIAKVLPRAGADDARCKSYAGTAAYMSPERFDPEAHGGHYDAY

AADVWGLGVTVLELLMGRYPLLPAGQRPSWPALMCAICFGETPVLSDGEASAELRGFVAA

CLRKDHTKRASVAELLAHPFVAGRDVATSKCALRKLVTEASTSP*

>RON2MKK10-4 Brdisv1RON21001725m.p

MASAKERRLPQLHLKLDVPTCAFRCAAPAPAPATAATPATSASRPPHGEFRLNDFDRLSV

LGRGNGGSVYKVSHRRTSALYALKIIHGAHARPGAADEEADIVRRVVDSPNVVRCHSVLP

TASGDAAALLLELVDGGSLDSLVGGGGFLPEAAVADVAAQALSGLAHLRARRVAHRDIKP

ANLLLSAAGEVKIADFGIAKVVVSGAGGRARALAYEGTVAYMSPERFDSERHADADPYAA

DVWGLGVTLLELLMGRYPLLPAGQKPTWAALMCAICFGELPALPEGAASLEFRGFVAACL

RKDHRKRASVVELLAHPFVAGRDVAASRRALREAIERRCSC*

>RON2MKK10-5 Brdisv1RON21001727m.p

MALTVRQRRLPQLHISLDLPSCSFRCPNPPVAATASTSGEFRASDFEPLAVLGRGNGGTV

YKVAHRRTSAQYALKVLHGGGDPGAAAAEADVLRRAADSPYVVRCHSVFPAASGSGETAL

LLELVDGGSLDSVRRGVGVSVFFPEAALAEVAAQALAGLAHLHARRVVHRDIKPANLLVS

GAGGVKVADFGIAMVLPSRAGGERCAAAYEGTVAYMSPERFDSEGRADADPRGADVWGLG

VTVLELLMGRYPLLPAGQKPTWAALMCAICFGELPALPEGAASTELRGFIAACLRKDHTK

RASVAELIKHPFVAGRNMAASRLALRRLVAGA*

>S8iiCMKK1 Brdisv1S8iiC1008085m.p

MRKPGKLALPSHESTIGKFLTQSGTFKDGDLLVNKDGLRIVHNSEEGEAPPIEPLDDHQL

SLDDLDAIKVIGKGSSGIVQLVRHKWTDQFFALKVIQLNIQESIRKQIAQELKISLSTQC

QYVVTCYQCFYVNGVISIVLEYMDGGSLADFLKTVRTIPEAYLAAICKQVLQGLMYLHHE

KRVIHRDLKPSNILINHRGEVKISDFGVSAIIASSSAQRDTFTGTFNYMAPERISGQKHG

YMSDIWSLGLVMLECATGNFPYPSPDSFYELLEAVVDQPPPSAPTDQFSPEFCSFISACI

QKEATDRSSAQVLSDHPFLSMYDDLNIDLADYFTTAGSPLATFKQIVL*

>S8iiCMKK3-1 Brdisv1S8iiC1038719m.p

MAGLEELKKKLQPLLFDDPDKDGISTRVPFPEDNCDSYVVSDGGTINLLSRSFGEYNINE

HGFHKRSTGADESDFGEKAYRCASHDMHIFGPIGNGASSVVQRAIFIPVHRILALKKINI

FEKEKRQQILNEMRTLCEASCYPGLVEFQGAFYMPDSGQISIALEYMDGGSLADVIKVKK

SIPEQVLAHMLQKVLLGLRYLHEVRHLVHRDIKPANMLVNLKGEAKITDFGVSAGLDNTM

AMCATFVGTVTYMSPERIRNENYSYAADIWSLGLTILECATGKFPYNVNEGPANLMLQIL

DDPSPTPPADAYSPEFCSFVNDCLQKDPDARPTCEQLFGHPFIKRYENAGVDLIAYVKGV

VDPTERLKEIAEMLAVHYYLLFNGSDGLWHHMKTFYMEESTFSFSGNVYVGRNDIFDTLS

SIRKKLKGDRPREKIVHVVEKLHCRANGETGIAIRVSGSLIVGNQFLVCGEGLQAEGMPS

VEELSIDIPSKRVGQFREQFMMLPGISMGSFHISRQDLYIIQA*

>S8iiCMKK3-2 Brdisv1S8iiC1006489m.p

MAGLEELKKKLQPLMFNDPDKDGFSTRVPFPEDTCDSYVVSDGGTINLLSRSFGEYNINE

HGFHKRSAGADESDFGEKAYRCASQDMHIFGPIGNGASSVVQRAIFIPVHRILALKKINI

FEKEKRQQILNEMRTLCEACCYPGLVEFQGAFYMPDSGQISIALEYMDGGSLADVIKVKK

SIPEPVLAHMLQKVLLGLRYLHEVRHLVHRDIKPANMLVNLKGEAKITDFGVSAGLDNTM

GMCATFVGTVTYMSPERIRNENYSYAADIWSLGLTILECATGKFPYNVNEGPANLMLQIL

DDPSPAPPENAFSSEFCSFVNDCLQKDADARPTCEQLLSHPFIKRYENAGVDLAAYVKGV

VNPEERLKQIAEMLAVHYYLLFNGSDGLWHHMKTFYMEDSTFSFSGNLYVGQSDIFDTLS

NIRTKLKGDRPREKIVHVVEKLHCRANEETGIAIRVSGSFIVSNQFLICGEGLQAEGMPS

LEELSIDIPSKRVGQFREQFIMHPGRSMGCYYISRQDLYIIQA*

>S8iiCMKK3-3 Brdisv1S8iiC1025083m.p

MGWDGMGQRKRRHRAGIPIPTYPGLAASLEFCFVTAGACHTAPGPGSKIAASLFAALLFQ

RPPASTSRAIFFCFVIMAAGLEDLRRRVQPIFFDADGNVMPAPDDDSEVLDGGTINLLSR

SSDEYNINERGFHKRTIRSDDEYSSEKAFRCSCHDMHIFDSVGNGASSVVHRAIYVPVHR

VLALKKINIFEKERRQQILNEIITLSEACCYPGLVEFHGVFYTPDSGEIYFALEYMDGGS

LADIIRVKKFISEPVLSHMLQKVLLALRYLHEVRHLVHRDIKPANLLLNLKGDTKITDFG

VTSGLHDSIDMCATFLGSVTYMSPERIRNESYSYSADIWSLGLTALECAAGRYPYDVNGG

EADLMLQILEDPSPTPPHDIYSEEFCSFINACLQKDADARPTCDQLLSHSFIKRYEGPGV

DLSEYNKSVHDPSERLSQIAHMLAVHYYLIFDGGDDQWCHMKTFYQQDSIFSFSGETHVG

KSEIFETLSRIRKMLKGNSPCEKIAHVMEKVYCRSHGEEGMRVRVSGSFIVGNEFVVCAD

GVRAEGMLSIDELSPDILSKQAGHFQEDFFMEPGTALGCYVISKQELHIADT*

>S8iiCMKK4 Brdisv1S8iiC1031440m.p

MRPGGPPNARPQQPGTPGXXXXXXXXXXPQRDLTSLAVPLPLPPPPSSAPSSASSSGSSL

SSMGAPTPPNSAGSAPPPPPPLAELERVRRIGSGAGGTVWMVRHRPTGRPYALKVLYGNH

DDAVRRQITREIAILRTAEHPAIVRCHGMYEQAGELQILLEFMDGGSLEGRRIASEAFLA

DVARQVLSGIAYLHRRHIVHRDIKPSNLLIDSGRRVKIADFGVGRILNQTMDPCNSSVGT

IAYMSPERINTDLNDGAYDGYAGDIWSFGLSILEFYLGRFPLGENLGKQGDWAALMCAIC

YSDSPAPPPIASPEFKSFISCCLQKNPARRPSAAQLLQHRFIAGPQPQVLAAPPS*

>S8iiCMKK5 Brdisv1S8iiC1007346m.p

MRPAGSLPSPQPGTPGRPRRRPDLTLPMPQRPDVSSSLAVPLPLPPPSSLGLAQPPAAAA

AAAAPPPPPLGELERVRRVGSGAGGTVWMVRHRPTGRCYALKQLYGNHDDAVRRQIAREI

AILRTAEHPAVVRCHGMYERGGELQILLEYMDGGSLDGRRIAAEGFLADVARQVLSGIAY

LHRRHIVHRDIKPSNLLIDSARRVKIADFGVGRILNQTMDPCNSSVGTIAYMSPERINTD

LNDGAYDGYAGDIWSFGLSILEFYLGRFPFGENLGKQGDWAALMVAICYNDPPEPSAAAS

PEFRGFISCCLQKNPAKRLSAAQLLQHPFVAGPQPLPLAAPPS*

>S8iiCMKK6 Brdisv1S8iiC1012258m.p

MRGKKPLKELKLSVPAQETSVDKFLTASGTFKDGELRLNQRGLRLISEEENGDEHQSTNM

KVEDVQLSMDDLEMIQVIGKGSGGVVQLVQHKWVGTFYALKGIQMNIQEAVRKQIVQELK

INQATQSPHIVSCHQSFYHNGVIYLVLEYMDRGSLADIIKQVKTILEPYLAVLCKQVLEG

LLYLHHERHVIHRDIKPSNLLVNHKGEVKITDFGVSAVLASSIGQRDTFVGTYNYMAPER

ISGSSYDYKSDVWSLGLVILECAIGRFPYTPSEGEGWLSFYELLEAIVDQPPPGAPADQF

SPEFCSFISACIQKDPAERMSASELLNHAFIKKFEGKDLDLRILVESLEPPMNVPE*

>S8iiCMKK10-1 Brdisv1S8iiC1001792m.p

MALLREKRLQLSLHVPTRAADAQEAGLHRRPNPAAALPLAATTPAARSSQFRVADFEKLA

VLGRGNGGTVYKVRHRETCELYALKVQHCNGDATAEAEVLSRTASPFVVRCHSVLPAAAS

GDVAMLLELVDGGSLDSIVKSRSRGQAEAFSQFPEEALAEVAAQALSGLAYLHARRIVHL

DVKPGNLLVSTGGEVKIADFGIARVLPRAGGDDVRCTAYAGTAAYMSPERFDPEAHGGHY

DPYAADVWGLGVTVLELLMGRYPLLPAGQRPSWAALMCAICFGETPALSDGEASAELRGF

VAACLHKDYRRRASVAELLAHPFVAGRDVAASKCALRKLVTEASMSP*

>S8iiCMKK10-2 Brdisv1S8iiC1011300m.p

MALVRQRRQLPHLTLPLDHFALRPPPVPAPAPTVAASTSSEAAGLRLSDFERISLLGQGN

GGTVYKARHRRAAAQPPVALKLFVAGDPSAAREAEILRLAADAPHVVRLHAVVPSSSPAA

GAEQPPPAALALELLPGGSLAGLLRRLGRSMGERPIAAVARQALLGLDALHALRVVHRDL

KPSNLLLGSHGEVKIADFGAGKVLRRRLDPCASYVGTAAYMSPERFDPEAYSGDYDPYAA

DVWSLGLAILELYLGHFPLLPAGQRPDWAALMCAICFGDAPEAPAAASEEFRDFVARCLE

KKAGQRASVAELLEHPFIAERDAEEAKRALAALVAEAELGDL*

>S8iiCMKK10-3 Brdisv1S8iiC1001688m.p

MALLREKRLQLSLHVPTRAAEALDAVHRRPNPVAATLAASTPAAARSSQFRLADFDKLTV

LGRGNGGTVYKVRHRETCELYALKVQHCNGDPTAAAEAEVLSRTASPFIVRCHSVLPGAA

SGDVAMLLELVDGGSLDSIVKSRRAHAFPFPEEALAEVAAQALSGLAYLHARRIVHLDIK

PGNLLVSTGGEVKVADFGIAKVLPRAGADDARCKSYAGTAAYMSPERFDPEAHGGHYDAY

AADVWGLGVTVLELLMGRYPLLPAGQRPSWPALMCAICFGETPVLSDGEASAELRGFVAA

CLRKDHTKRASVAELLAHPFVAGRDVATSKCALRKLVTEASTSP*

>S8iiCMKK10-4 Brdisv1S8iiC1001680m.p

MASAKERRLPQLHLKLDVPTCAFRCAAPAPAPATAATPATSASRPPHGEFRLNDFDRLSV

LGRGNGGSVYKVSHRRTSALYALKIIHGAHARPGAADEEADIVRRVVDSPNVVRCHSVLP

TASGDAAALLLELVDGGSLDSLVGGGGFLPEAAVADVAAQALSGLAHLRARRVAHRDIKP

ANLLLSAAGEVKIADFGIAKVVVSGAGGRARALAYEGTVAYMSPERFDSERHADADPYAA

DVWGLGVTLLELLMGRYPLLPAGQKPTWAALMCAICFGELPALPEGAASLEFRGFVAACL

RKDHRKRASVVELLAHPFVAGRDVAASRRALREAIERRCSC*

>S8iiCMKK10-5 Brdisv1S8iiC1001689m.p

MALTVRQRRLPQLHISLDLPSCSFRCPNPPVAATASTSGEFRASDFEPLAVLGRGNGGTV

YKVAHRRTSAQYALKVLHGGGDPGAAAAEADVLRRAADSPYVVRCHSVFPAASGSGETAL

LLELVDGGSLDSVRRGVGVSVFFPEAALAEVAAQALAGLAHLHARRVVHRDIKPANLLVS

GAGGVKVADFGIAMVLPSRAGGERCAAAYEGTVAYMSPERFDSEGRADADPRGADVWGLG

VTVLELLMGRYPLLPAGQKPTWAALMCAICFGELPALPEGAASTELRGFIAACLRKDHTK

RASVAELIKHPFVAGRNMAASRLALRRLVAGA*

>Uni2MKK1 Brdisv1Uni21007769m.p

MRKPGKLALPSHESTIGKFLTQSGTFKDGDLLVNKDGLRIVHNSEEGEAPPIEPLDDHQL

SLDDLDAIKVIGKGSSGIVQLVRHKWTDQFFALKVIQLNIQESIRKQIAQELKISLSTQC

QYVVTCYQCFYVNGVISIVLEYMDGGSLADFLKTVRTIPEAYLAAICKQVLQGLMYLHHE

KRVIHRDLKPSNILINHRGEVKISDFGVSAIIASSSAQRDTFTGTFNYMAPERISGQKHG

YMSDIWSLGLVMLECATGNFPYPSPDSFYELLEAVVDQPPPSAPTDQFSPEFCSFISACI

QKEATDRSSAQVLSDHPFLSMYDDLNIDLADYFTTAGSPLATFKQIVL*

>Uni2MKK3-1 Brdisv1Uni21028033m.p

MAGLEELKKKLQPLLFDDPDKDGISTRVPFLEDNCDSYVVSDGGTINLLSRSFGEYNINE

HGFHKRSTGADESDFGEKAYRCASHDMHIFGPIGNGASSVVQRAIFIPVHRILALKKINI

FEKEKRQQILNEMRTLCEASCYPGLVEFQGAFYMPDSGQISIALEYMDGGSLADVIKVKK

SIPEQVLAHMLQKVLLGLRYLHEVRHLVHRDIKPANMLVNLKGEAKITDFGVSAGLDNTM

AMCATFVGTVTYMSPERIRNENYSYAADIWSLGLTILECATGKFPYNVNEGPANLMLQIL

DDPSPTPPADAYSPEFCSFVNDCLQKDPDARPTCEQLFGHPFIKRYENAGVDLIAYVKGV

VDPTERLKEIAEMLAVHYYLLFNGSDGLWHHMKTFYMEESTFSFSGNVYVGRNDIFDTLS

SIRKKLKGDRPREKIVHVVEKLHCRANGETGIAIRVSGSLIVGNQFLVCGEGLQAEGMPS

VEELSIDIPSKRVGQFREQFMMLPGISMGSFHISRQDLYIIQA*

>Uni2MKK3-2 Brdisv1Uni21042247m.p

MAGLEELKKKLQPLMFNDPDKDGFSTRVPFPEDTCDSYVVSDGGTINLLSRSFGEYNINE

HGFHKRSAGADESDFGEKAYRCASQDMHIFGPIGNGASSVVQRAIFIPVHRILALKKINI

FEKEKRQQILNEMRTLCEACCYPGLVEFQGAFYMPDSGQISIALEYMDGGSLADVIKVKK

SIPEPVLAHMLQKVLLGLRYLHEVRHLVHRDIKPANMLVNLKGEAKITDFGVSAGLDNTM

AMCATFVGTVTYMSPERIRNENYSYAADIWSLGLTILECATGKFPYNVNEGPANLMLQIL

DDPSPAPPENAFSSEFCSFVNDCLQKDADARPTCEQLLSHPFIKRYENAGVDLAAYVKGV

VNPEERLKQIAEMLAVHYYLLFNGSDGLWHHMKTFYMEDSTFSFSGNLYVGQSDIFDTLS

NIRTKLKGDRPREKIVHVVEKLHCRANEETGIAIRVSGSFIVSNQFLICGEGLQAEGMPS

LEELSIDIPSKRVGQFREQFIMHPGRSMGCYYISRQDLYIIQA*

>Uni2MKK3-3 Brdisv1Uni21030859m.p

MGWDGMGQRKRRHRAGIPIPTYPGLAASLEFCFVTAGACHTAPGPGSKIAASLFAALLFQ

RPPASTSRAIFFCFVIMAAGLEDLRRRVQPIFFDADGNVMPAPDDDSEVLDGGTINLLSR

SSDEYNINERGFHKRTIRSDDEYSSEKAFRCSCHDMHIFDSVGNGASSVVHRAIYVPVHR

VLALKKINIFEKERRQQILNEIITLSEACCYPGLVEFHGVFYTPDSGEIYFALEYMDGGS

LADIIRVKKFISEPVLSHMLQKVLLALRYLHEVRHLVHRDIKPANLLLNLKGDTKITDFG

VTSGLHDSIDMCATFLGSVTYMSPERIRNESYSYSADIWSLGLTALECATGRYPYDVNGG

EADLMLQILEDPSPTPPHDIYSEEFCSFINACLQKDADARPTCDQLLSHSFIKRYEGPGV

DLSEYNKSVHDPSERLSQIAHMLAVHYYLIFDGGDDQWCHMKTFYQQDSIFSFSGETHVG

KSEIFETLSRIRKMLKGNSPCEKIAHVMEKVYCRSHGEEGMRVRVSGSFIVGNEFVVCAD

GVRAEGMLSIDELSPDILSKQAGHFQEDFFMEPGTALGCYVISKQELHIADT*

>Uni2MKK4 Brdisv1Uni21036840m.p

MRPGGPPNARPQQPGTPGRARRRPDLTLPLPQRDLTSLAVPLPLPPPPSSAPSSASSSGS

SLSSMGAPTPPNSAGSAPPPPPPLAELERVRRIGSGAGGTVWMVRHRPTGRPYALKVLYG

NHDDAVRRQITREIAILRTAEHPAIVRCHGMYEQAGELQILLEFMDGGSLEGRRIASEAF

LADVARQVLSGIAYLHRRHIVHRDIKPSNLLIDSGRRVKIADFGVGRILNQTMDPCNSSV

GTIAYMSPERINTDLNDGAYDGYAGDIWSFGLSILEFYLGRFPLGENLGKQGDWAALMCA

ICYSDSPAPPPIASPEFKSFISCCLQKNPARRPSAAQLLQHRFIAGPQPQVLAAPPS*

>Uni2MKK5 Brdisv1Uni21007057m.p

MRPAGSLPSPQPGTPGRPRRRPDLTLPMPQRPDVSSSLAVPLPLPPPSSLGLAQPPAAAA

AAAAPPPPPLGELERVRRVGSGAGGTVWMVRHRPTGRCYALKQLYGNHDDAVRRQIAREI

AILRTAEHPAVVRCHGMYERGGELQILLEYMDGGSLDGRRIAAEGFLADVARQVLSGIAY

LHRRHIVHRDIKPSNLLIDSARRVKIADFGVGRILNQTMDPCNSSVGTIAYMSPERINTD

LNDGAYDGYAGDIWSFGLSILEFYLGRFPFGENLGKQGDWAALMVAICYNDPPEPSAAAS

PEFRGFISCCLQKNPAKRLSAAQLLQHPFVAGPQPLPLAAPPS*

>Uni2MKK6 Brdisv1Uni21011815m.p

MRGKKPLKELKLSVPAQETSVDKFLTASGTFKDGELRLNQRGLRLISEEENGDEHQSTNM

KVEDVQLSMDDLEMIQVIGKGSGGVVQLVQHKWVGTFYALKGIQMNIQEAVRKQIVQELK

INQATQSPHIVSCHQSFYHNGVIYLVLEYMDRGSLADIIKQVKTILEPYLAVLCKQVLEG

LLYLHHERHVIHRDIKPSNLLVNHKGEVKITDFGVSAVLASSIGQRDTFVGTYNYMAPER

ISGSSYDYKSDVWSLGLVILECAIGRFPYTPSEGEGWLSFYELLEAIVDQPPPGAPEDQF

SPEFCSFISACIQKDPAERMSASELLNHAFIKKFEGKDLDLRILVESLEPPMNVPE*

>Uni2MKK10-1 Brdisv1Uni21001716m.p

MALLREKRLQLSLHVPTRAADAQEAGLHRRPNPAAALPLAATTPAARSSQFRVADFEKLA

VLGRGNGGTVYKVRHRETCELYALKVQHCNGDATAEAEVLSRTASPFVVRCHSVLPAAAS

GDVAMLLELVDGGSLDSIVKSRSRGQAEAFSQFPEEALAEVAAQALSGLAYLHARRIVHL

DVKPGNLLVSTGGEVKIADFGIARVLPRAGGDDVRCTAYAGTAAYMSPERFDPEAHGGHY

DPYAADVWGLGVTVLELLMGRYPLLPAGQRPSWAALMCAICFGETPALSDGEASAELRGF

VAACLHKDYRRRASVAELLAHPFVAGRDVAASKCALRKLVTEASMSP*

>Uni2MKK10-2 Brdisv1Uni21010884m.p

MALVRQRRQLPHLTLPLDHFALRPPPVPAPAPTVAASTSSEAAGLRLSDFERISLLGQGN

GGTVYKARHRRAAAQPPVALKLFVAGDPSAAREAEILRLAADAPHVVRLHAVVPSSSPAA

GAEQPPPAALALELLPGGSLAGLLRRLGRSMGERPIAAVARQALLGLDALHALRVVHRDL

KPSNLLLGSHGEVKIADFGAGKVLRRRLDPCASYVGTAAYMSPERFDPEAYSGDYDPYAA

DVWSLGLAILELYLGHFPLLPAGQRPDWAALMCAICFGDAPEAPAAASEEFRDFVARCLE

KKAGQRASVAELLEHPFIAERDAEEAKRALAALVAEAELGDL*

>Uni2MKK10-3 Brdisv1Uni21001612m.p

MALLREKRLQLSLHVPTRAAEALDAVHRRPNPVAATLAASTPAAARSSQFRLADFDKLTV

LGRGNGGTVYKVRHRETCELYALKVQHCNGDPTAAAEAEVLSRTASPFIVRCHSVLPGAA

SGDVAMLLELVDGGSLDSIVKSRRAHAFPFPEEALAEVAAQALSGLAYLHARRIVHLDIK

PGNLLVSTGGEVKVADFGIAKVLPRAGADDARCKSYAGTAAYMSPERFDPEAHGGHYDAY

AADVWGLGVTVLELLMGRYPLLPAGQRPSWPALMCAICFGETPVLSDGEASAELRGFVAA

CLRKDHTKRASVAELLAHPFVAGRDVATSKCALRKLVTEASTSP*

>Uni2MKK10-4 Brdisv1Uni21001606m.p

MASAKERRLPQLHLKLDVPTCAFRCAAPAPAPATAATPATSASRPPHGEFRLNDFDRLSV

LGRGNGGSVYKVSHRRTSALYALKIIHGAHARPGAADEEADIVRRVVDSPNVVRCHSVLP

TASGDAAALLLELVDGGSLDSLVGGGGFLPEAAVADVAAQALSGLAHLRARRVAHRDIKP

ANLLLSAAGEVKIADFGIAKVVVSGAGGRARALAYEGTVAYMSPERFDSERHADADPYAA

DVWGLGVTLLELLMGRYPLLPAGQKPTWAALMCAICFGELPALPEGAASLEFRGFVAACL

RKDHRKRASVVELLAHPFVAGRDVAASRRALREAIERRCSC*

>Uni2MKK10-5 Brdisv1Uni21001608m.p

MALTVRQRRLPQLHISLDLPSCSFRCPNPPVAATASTSGEFRASDFEPLAVLGRGNGGTV

YKVAHRRTSAQYALKVLHGGGDPGAAAAEADVLRRAADSPYVVRCHSVFPAASGSGETAL

LLELVDGGSLDSVRRGVGVSVFFPEAALAEVAAQALAGLAHLHARRVVHRDIKPANLLVS

GAGGVKVADFGIAMVLPSRAGGERCAAAYEGTVAYMSPERFDSEGRADADPRGADVWGLG

VTVLELLMGRYPLLPAGQKPTWAALMCAICFGELPALPEGAASTELRGFIAACLRKDHTK

RASVAELIKHPFVAGRNMAASRLALRRLVAGA*
