# Supplementary material for: Comparative analysis of MAPK and MKK gene families reveals differential evolutionary patterns in Brachypodium distachyon inbred lines
Source: PeerJ. 2021 Apr 6;9:e11238. doi: 10.7717/peerj.11238 (PMC8034371; doi:10.7717/peerj.11238)
Supplement: Supplemental Information 17 [file peerj-09-11238-s017.docx]

Table.S6. The distribution of MPK21-2 of *B. distachyon* inbred lines.

| Type | Inbred line |
| --- | --- |
| I | Mon3, ABR9, Tek-4, BdTR7a, BdTR8i, Bd1-1, Bd29-1, Tek-2, BdTR10c, ABR2, ABR3, ABR5, ABR7, Mig3, Uni2, Mur1, Jer1, Per1, Luc1, RON2 |
| II | Arn1, Bd21, Bd21-3, Bd3-1, Bd2-3,Adi-10, BdTR12c, Adi-2, Adi-12, BdTR9k, Kah-1, Kah-5, BdTR5i, BdTR11a, BdTR11i, BdTR11g, BdTR13c, BdTR13a, Bis-1, Koz-3, Koz-1, BdTR3c, Gaz-8, BdTR1i, BdTR2b, BdTR2g, Bd18-1, Bd30-1, Foz1, ABR4, ABR6, S8iiC, Sig2 |
