# Supplementary material for: Comparative analysis of MAPK and MKK gene families reveals differential evolutionary patterns in Brachypodium distachyon inbred lines
Source: PeerJ. 2021 Apr 6;9:e11238. doi: 10.7717/peerj.11238 (PMC8034371; doi:10.7717/peerj.11238)
Supplement: Supplemental Information 18 [file peerj-09-11238-s018.docx]

**Table S7** Table showing average amino acid composition (AAAC) of *B. distachyon* MAPKs and MKKs.

|  | AAAC of MPK gene | | AAAC of MKK gene | |
| --- | --- | --- | --- | --- |
| Amino Acid | Brachypodium inbreds | Bd21 | Brachypodium inbreds | Bd21 |
| Ala | 7.23 | 7.01 | 10.89 | 11.06 |
| Cys | 1.49 | 1.67 | 2.12 | 2.08 |
| Asp | 6.33 | 6.20 | 5.44 | 5.42 |
| Glu | 6.21 | 6.15 | 5.9 | 5.83 |
| Phe | 4.48 | 4.36 | 3.92 | 3.86 |
| Gly | 5.32 | 5.34 | 7.97 | 8.01 |
| His | 3.33 | 3.30 | 3.05 | 3.05 |
| Ile | 5.99 | 6.01 | 4.96 | 4.85 |
| Lys | 5.80 | 5.82 | 3.93 | 3.9 |
| Leu | 9.27 | 9.75 | 10.29 | 10.38 |
| Met | 2.51 | 2.60 | 2.34 | 2.33 |
| Asn | 3.90 | 4.15 | 2.52 | 2.48 |
| Pro | 6.38 | 6.13 | 6.35 | 6.31 |
| Gln | 3.63 | 3.53 | 3.14 | 3.11 |
| Arg | 6.95 | 6.54 | 6.56 | 6.69 |
| Ser | 6.27 | 6.48 | 7.2 | 7.2 |
| Thr | 4.67 | 4.62 | 3.32 | 3.32 |
| Val | 5.62 | 5.79 | 6.52 | 6.59 |
| Trp | 0.71 | 0.74 | 0.6 | 0.59 |
| Tyr | 3.91 | 3.81 | 2.98 | 2.94 |
